# Revised estimates of types and duration of long COVID symptoms based on claims records from 245 million US patients

**DOI:** 10.1073/pnas.2604414123

**Published:** 2026-10-27

**Authors:** Hamed Nilforoshan, Julia Reisler, Erfan Jahanparast, Michael Moor, Steven N. Goodman, Stefan Wager, Jure Leskovec

**Affiliations:** ^a^ Department of Computer Science, Stanford University, Stanford, CA 94305; ^b^ Department of Computer Science, University of California, Berkeley, CA 94720; ^c^ Department of Biosystems Science and Engineering, ETH Zurich, Basel 4056, Switzerland; ^d^ Department of Epidemiology and Population Health, Stanford University, Stanford, CA 94305; ^e^ Stanford Graduate School of Business, Stanford University, Stanford, CA 94305

**Keywords:** long COVID, causal inference, epidemiology

## Abstract

COVID-19 has been reported to cause multisystem effects lasting years [Z. Al-Aly, E. Topol, Science 383, 830–832 (2024)]. These findings are primarily based on cohort studies comparing test-positive COVID-19 patients to controls without known COVID-19 infection, tested or untested [M. Cai, Y. Xie, E. J. Topol, Z. Al-Aly, Nat. Med. 30, 1564–1573 (2024)]. However, such comparisons can be biased by the reasons people were tested. Using 14.4 billion insurance claims from 244.7 million US patients, we find that most long-term effects attributed to COVID-19 by unrestricted control designs likely reflect bias due to selective testing of individuals with non-COVID conditions, causing long-term effects of those conditions to be misattributed to COVID-19. We removed this bias by analyzing only tested individuals, comparing test-positive to test-negative patients. Across 614 clinical outcomes followed over 2 y, the test-based design resulted in 83.5% fewer symptoms being attributable to COVID-19 between 30 to 119 d after infection (43 attributable symptoms vs. 262 for the unrestricted control design), a 98.2% reduction at 120 to 359 d (5 vs. 286), and a 100% reduction at 360 to 719 d (0 vs. 369). The unrestricted control design also attributed unrelated outcomes (e.g., firearm injuries) to COVID-19 at a high rate, whereas the testing-based design did not. The test-based control design may modestly underestimate some effects. This study does not indicate that symptoms beyond one year are not genuine but that their attributability to COVID-19 is uncertain.

## Significance

Long COVID has been linked to many long-term health effects. Some commonly used study designs can misattribute symptoms to long COVID when they are actually due to non-COVID conditions that prompted care-seeking and testing. Drawing from a nationwide database of insurance claims, we mitigate this selective-testing bias using a study design that restricts analyses to individuals who sought PCR testing. With this design, known acute COVID symptoms are detected, but the number and duration of long-term outcomes attributable to COVID-19 is substantially smaller and shorter than in high profile prior reports. This design also markedly reduces false attribution of negative control outcomes to COVID. This work shows how study design can affect causal attribution in large-scale observational analyses.

Long COVID (postacute sequelae of COVID-19, also known as PASC) describes a varied constellation of long-term adverse health effects after a severe acute respiratory syndrome coronavirus 2 (SARS-CoV-2) infection (1–4). Since the start of the global COVID-19 pandemic in early 2020, an extensive body of evidence has characterized long COVID as a multisystem disease with over 200 symptoms (5–19). Long COVID manifests as both persistent symptoms after initial infection (13, 20) and new-onset symptoms (6, 21), affecting almost all organ systems. New-onset long COVID symptoms include neurocognitive (15, 22, 23), reproductive (12), cardiovascular (7, 24–26), and gastrointestinal effects (27).

The evidence about which symptoms are attributable to long COVID comes from observational analyses. Most studies have used electronic health records or claims data to compare individuals that were diagnosed with COVID-19 via testing to similar individuals without a confirmed COVID-19 diagnosis (6, 11, 13, 21, 28–31). This approach can be biased if there are differences between people with and without a COVID-19 diagnosis that are not recorded in the data (32). For instance, individuals who receive a COVID-19 diagnosis may have actually been tested while seeking care for a non-COVID condition (e.g., diabetes). If non-COVID conditions are not well recorded in the patient’s medical history, symptoms resulting from these conditions could be falsely attributed to COVID-19.

Another source of bias is care-seeking behavior: Individuals who test for COVID-19 may also be more likely to seek medical care for non-COVID conditions. Overall, these non-COVID factors could cause COVID-19 patients to appear less healthy over time, independent of the effects of COVID-19 itself. Additionally, prior studies characterizing long COVID symptoms have reported mixed findings (4–19, 21, 28–31, 33–35), partly due to methodological limitations such as small sample sizes, geographic variation, and differing study populations. Therefore, research with large, nationally representative samples is needed, as is thorough investigation into potential sources of bias inherent in unrestricted control studies.

**Fig. 1.**
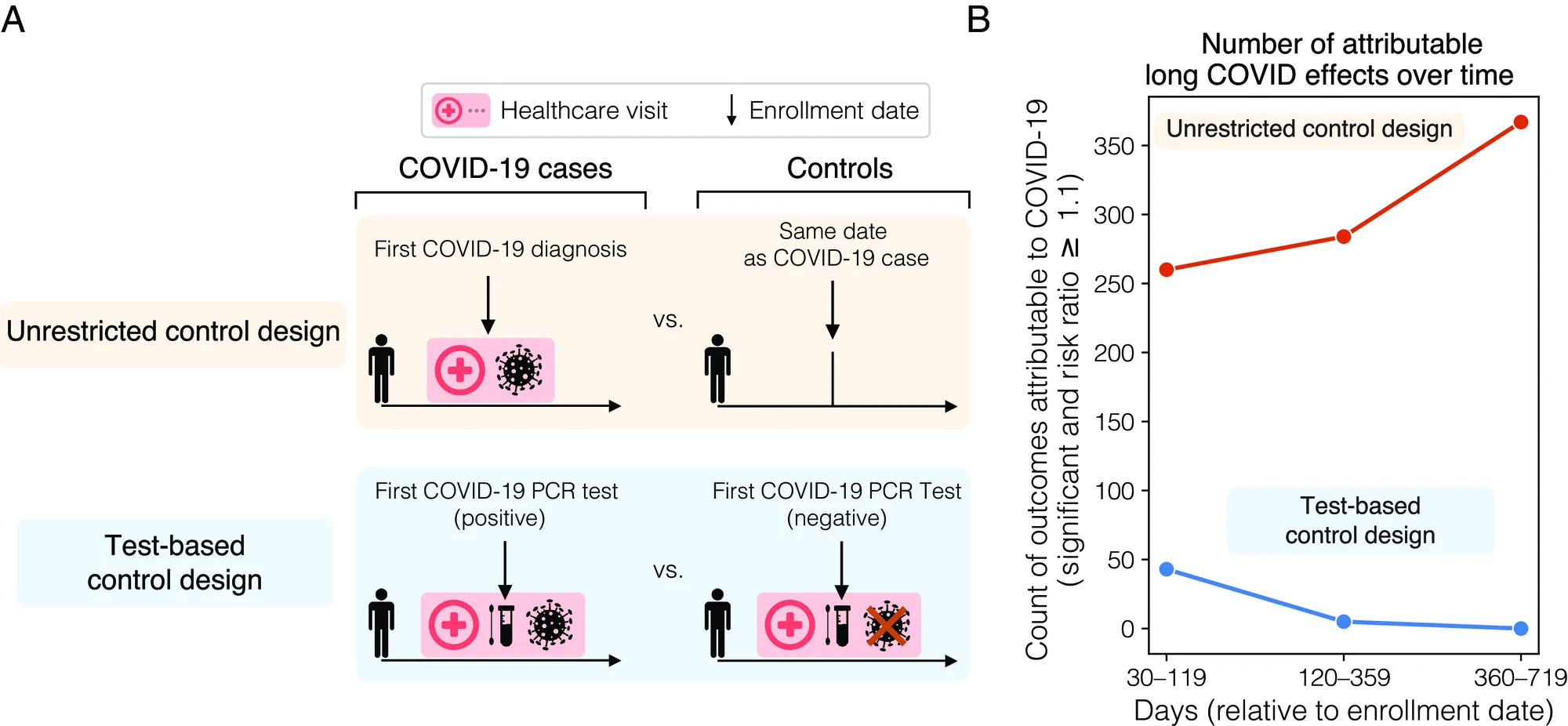
Test-based control design reduces the number of attributable long COVID effects by an order of magnitude. (*A*, *Top*) Unrestricted control designs compare test-positive COVID-19 cases to matched individuals (i.e., individuals with similar medical histories and demographics) who are not known to have COVID-19 (controls). This approach does not account for why individuals were having a healthcare visit or taking a test. In such a design, COVID-19 cases are typically enrolled during a healthcare visit, whereas the majority of controls will not have taken a COVID-19 test or visited a healthcare center at the time they were enrolled in the study. As such, any long-term consequences of unrelated health conditions and non-COVID factors that prompted the visit and COVID-19 testing may be misattributed to the COVID-19 infection (32, 36–39). (*A*, *Bottom*) The test-based control design mitigates this bias by enrolling only individuals who had a healthcare visit and received their first COVID-19 PCR test. This design compares test-positive individuals (cases) to test-negative individuals who also took a COVID-19 PCR test but received a negative result (controls), after matching on medical history and demographics. The control group is much more similar to the COVID-19 cases, thus reducing confounding bias. (*B*) The test-based control design (blue) detects fewer long COVID symptoms (risk ratio ≥ 1.1 and *P* < 0.05 with Bonferroni correction for multiple comparisons; *Methods M3*) compared to an unrestricted control analysis of the same data (orange), especially at longer timescales. Out of a set of 614 considered outcomes, the test-based control analysis attributes 83.5% fewer outcomes to COVID-19 at 30 to 119 d than the unrestricted control analysis, and 98.2% fewer at 120 to 359 d postinfection. At 360 to 719 d, the test-based control analysis finds no attributable outcomes, while the unrestricted control analysis reported 369.

In this paper, we attempt to disentangle genuine long COVID symptoms from those that might arise from factors leading to testing. To achieve this, we consider a study design that studies individuals starting at the time of their first recorded COVID-19 PCR test, comparing the test-positive individuals (cases) with the test-negative individuals (controls). We call this a “test-based control” design, in contrast to “unrestricted control” designs where cases are based on test results but controls are a mix of test-negative and untested individuals (Fig. 1).

We applied the test-based control design to evaluate 614 health outcomes over a 2-y follow-up period. Compared to an unrestricted control analysis using the same data, the test-based control design identifies substantially fewer and shorter-lasting long COVID effects. Moreover, we show that the unrestricted control design can overestimate the effects of long COVID and identify implausible associations, e.g. with firearm injuries and adult congenital abnormalities, associations that are detectable due to the large scale and granularity of our dataset. Finally, we demonstrate how selective COVID-19 testing, where individuals with non-COVID health conditions and related factors (e.g., a higher tendency to seek medical care) are more likely to be tested, explaining why unrestricted control designs are prone to detecting spurious associations.

## Results

To identify symptoms attributable to long COVID, we designed a study that corrects two key biases common in prior research. Many studies have compared COVID-19 cases—all tested and enrolled during a healthcare visit—with an undiagnosed group that included untested controls who had not visited a healthcare center at the time. We call this an unrestricted control approach (10, 12, 14, 40, 41). This approach leads to the attribution of future health problems to COVID-19 infection when they might instead be due to non-COVID factors or conditions that prompted the healthcare visit and testing. Furthermore, some controls in these studies were selected among those who were never diagnosed with COVID-19, which allows future information about who remained uninfected to influence eligibility, incurring immortal time bias (42).

We address both sources of bias using a test-based control design, where test-positive individuals (cases) are compared to test-negative individuals (controls), i.e., the controls are restricted to those who were tested, making them more comparable to the COVID-19 cases on factors that influenced testing. Enrolling all participants at the time of their first PCR test also ensures that the design is not biased by future testing information or by patient outcomes. This approach has different limitations, possibly conservative because controls may have illnesses with long-term consequences, resulting in collider bias (*Discussion*).

We compared the results of the two designs on the same dataset. For both analyses, we reweighted the control group so that its medical history and demographics matched those of the COVID-19 cases (*Methods M2*), thereby reducing confounding (43, 44) and collider bias (36). Specifically, we used a synthetic control approach (45, 46) that allows us to match medical histories over time (*Methods M3*; *SI Appendix*, Fig. S1). We observed that the test-positive and test-negative individuals have similar medical histories even before reweighting (*SI Appendix*, Tables S6 and S7), in contrast to unrestricted controls which have fewer recorded health claims (*SI Appendix*, Tables S4 and S5). Moreover, we found that the test-positive and test-negative groups are well balanced by enrollment place and time (*SI Appendix*, Figs. S10 and S11).

### Number and Duration of Long COVID Signs and Symptoms.

We highlight effects that cleared both a minimum effect size threshold (risk ratio ≥ 1.1) and a statistical significance threshold (*P* < 0.05 with Bonferroni correction for multiple comparisons; *Methods M3*). This risk ratio threshold was used to identify symptoms that can most confidently be attributed to long COVID, since the large sample size enabled finding statistical significance for minuscule effects. That said, small effects can still be meaningful from clinical or public health perspectives for common or severe outcomes.

Of the 614 health outcomes analyzed over 2 y, the test-based control analysis found significant and harmful long-term effects of COVID-19 for 43 outcomes on days 30 to 119 after infection, five outcomes on days 120 to 359 after infection, and no outcomes 360 to 719 d after infection (Fig. 2 and *SI Appendix*, Fig. S5). In contrast, the unrestricted control approach found significant and harmful effects for 262, 286, and 369 outcomes on days 30 to 119, 120 to 359, and 360 to 719 respectively. The test-based control design thus detected significantly fewer long COVID symptoms than a comparable unrestricted control approach and the symptoms generally persisted a shorter amount of time: The reduction in the number of attributed symptoms was 83.5% at 30 to 119 d after infection, 98.2% at 120 to 359 d, and 100% at 360 to 719 d (*SI Appendix*, Fig. S5 for overall results, *SI Appendix*, Figs. S15–S56 and S60–S80 for individual outcomes, for temporal trends, *SI Appendix*, Figs. S81–S105 (test-based), *SI Appendix*, Figs. S131–S155 (unrestricted control).

**Fig. 2.**
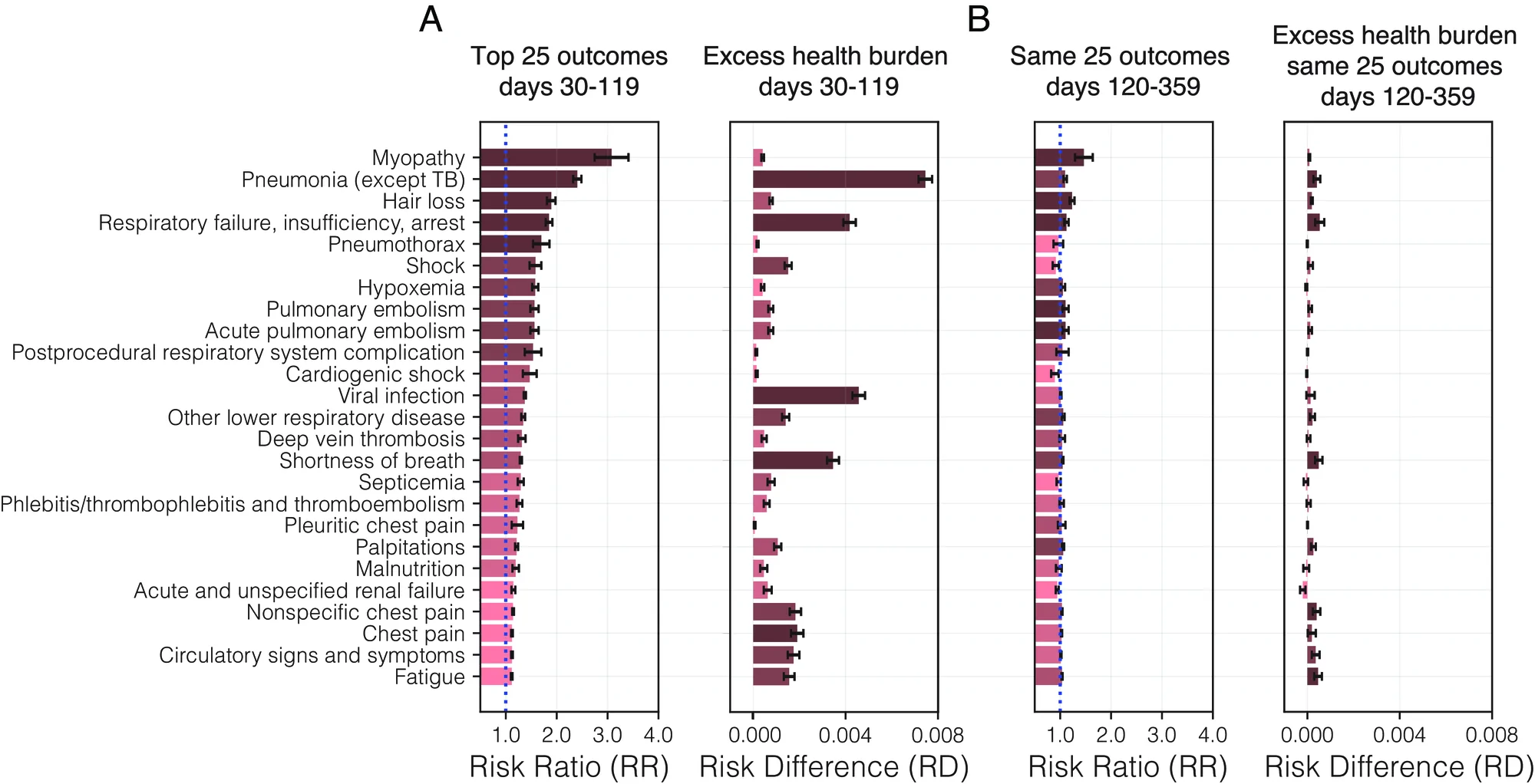
Long-term COVID-19 outcomes identified by the test-based control design. (*A*, *Left*) Top 25 outcomes most attributable to long COVID, ranked by relative risk (RR) at 30 to 119 d after infection. We detect diverse, multisystem impacts of long COVID that extend beyond acute respiratory effects. For instance, myopathy (RR 3.1), hair loss (RR 1.89), palpitations (RR 1.21), malnutrition (RR 1.20), and fatigue (RR 1.12) are more likely after COVID-19 infection. All outcomes shown have RR ≥ 1.1, with Bonferroni-corrected 95% two-sided bootstrap CI also ≥1.1, indicating high-confidence attributable effects. (*Right*) Corresponding risk differences (RDs; claims per month; *Methods M3*) quantify the absolute excess risk attributable to long COVID symptoms. Respiratory (pneumonia, respiratory failure, shortness of breath), immune (viral infection, septicemia), and circulatory issues (chest pain, circulatory symptoms) pose the highest absolute burden, while conditions like myopathy, despite higher relative risks, have a smaller impact due to lower baseline prevalence. Overall, our findings support prior evidence that long COVID is a multisystem disease, but indicate that the largest population-level impacts are concentrated in a smaller subset of symptoms than previously reported. (*B*) The same outcomes as in Fig. 2*A* for 120 to 359 d postinfection, revealing a narrowed set of symptoms that persist up to 1 y after infection. (*Left*) Risks of myopathy, hair loss, respiratory failure/insufficiency/arrest, and pulmonary embolism remain elevated (RR > 1.1), indicating continued multisystem impacts. (*Right*) Across outcomes, risk differences (RDs) fall to near zero, indicating low absolute excess risk. However, pneumonia, shortness of breath, chest pain, and fatigue continue to contribute a small but statistically significant excess risk (*P* < 0.05 with Bonferroni correction) due to high baseline prevalence.

These findings demonstrate that analyses of long COVID are highly sensitive to study design, with the test-based control design yielding far fewer symptoms of long COVID than reported in unrestricted control studies (2, 10). Moreover, unrestricted control designs (40, 41), suggest that there is a persistent burden of long COVID symptoms lasting up to 3 y postinfection, whereas our findings indicate that the frequency of signs and symptoms in the test positive group decreased to that of the test-negative group within approximately 1 y.

### Comparing False Attribution Rates of Test-Based vs. Unrestricted Control Designs.

We also evaluated how often methods used in existing studies of long COVID detected spurious symptoms and whether our study design addresses this issue. We did so by using “negative control outcomes” that are highly unlikely to have been caused by COVID-19, such as congenital disorders (e.g., cerebral palsy in adults), or external events (e.g., firearm injuries). Any relationship between COVID-19 and these outcomes is presumably due to chance or design bias, and the magnitude of these estimated relationships allows us to compare the biases of different study designs. Our large data scale allowed us to analyze 49 negative control outcomes, many of which are too rare to be studied with smaller datasets.

**Fig. 3.**
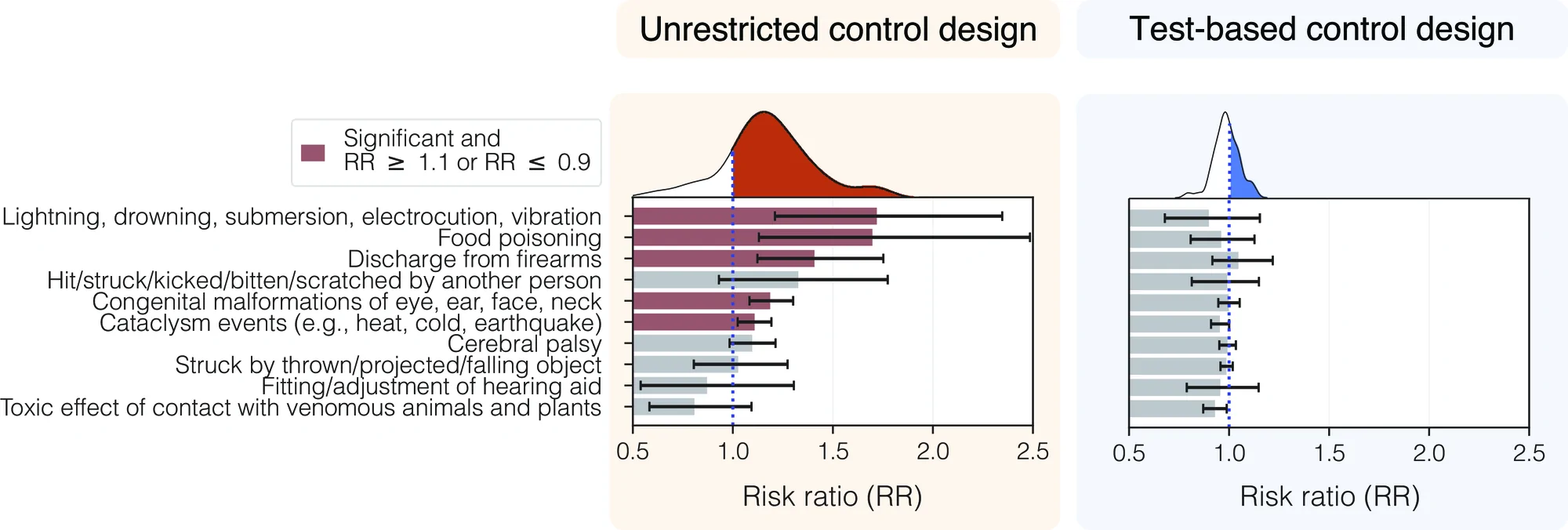
Test-based control design corrects for overestimation of effects found in unrestricted control studies. We investigate outcomes not expected to be caused by COVID-19 infection (e.g., firearm injuries, congenital conditions in adults). An unrestricted control design, where COVID-19 cases are compared to untested and test-negative individuals, suggests that COVID-19 has implausible long-term effects (*Left* panel). For instance, the unrestricted control analysis shows that COVID-19 infection increases the likelihood of injury due to lightning, drowning, submersion, electrocution, or vibration by 76% on days 30 to 119 postinfection (*P* < 0.05). The test-based control design corrects for this bias and does not find such an effect (*Right* panel). This is one of 49 “negative control” outcomes that we examined—events that COVID-19 could not plausibly cause (10 shown here; full set in *SI Appendix*, Figs. S57–S59). These serve as a benchmark to measure bias. Overall, the unrestricted control analysis shows strong positive bias: 53.1% of these implausible outcomes are significant with risk ratios ≥1.1 (*P* < 0.05). By contrast, only 4.1% of these outcomes are significant in the test-based control analysis, with risk ratios ≤0.9 (*P* < 0.05), suggesting only mild negative bias (*Discussion*). Unrestricted control designs thus falsely attribute many effects to COVID-19, whereas the test-based control design yields more reliable estimates. Red indicates statistically significant results (*P* < 0.05; two-sided, unadjusted for multiple comparisons) with risk ratios that are ≥1.1 or ≤0.9. Error bars show 95% bootstrap CI.

We found that the unrestricted control design, comparing COVID-19 cases to never-diagnosed controls, falsely detected associations for 53.1% of negative control outcomes, meaning the unrestricted control analysis attributed sizable and significant effects to COVID-19 for more than half of these negative control outcomes (RR ≥ 1.1 and *P* < 0.05; two-sided bootstrap, unadjusted for multiple comparisons). The test-based control design lowered the false association rate to 4.1%. For instance, the unrestricted control analysis of these data showed that COVID-19 infection increased the likelihood of future injury due to lightning, drowning, submersion, electrocution, or vibration by 76% and increased the likelihood of congenital malformations of the eye, ear, face, or neck in adults by 18.6%. The test-based control analysis detected no effect of COVID-19 on either, with nonsignificant RRs below or near 1.0. (Fig. 3) Thus, the test-based control design offered a more reliable way to attribute signs or symptoms to preceding COVID infection. (Fig. 3 and *SI Appendix*, Figs. S57–S59)

### Characterizing Long COVID Symptoms and Recovery Trajectory.

We investigated the long-term effect of COVID on all 614 health outcomes across three postacute time periods: Early (30 to 119 d after infection, or 1 to 4 mo), middle (120 to 359 d after infection, 5 to 12 mo), and late (360 to 719 d after infection, 13 to 24 mo). (Figs. 2 and 4 and *SI Appendix*, Figs. S81–S105) In the early postacute period, we find that although the number of attributable effects is smaller than reported in many previous studies, COVID-19 nevertheless impacts multiple organ systems: respiratory, cardiovascular, renal, immune, musculoskeletal, gastrointestinal, neurologic, and dermatological (hair loss). These findings confirm prior work showing that long COVID is a multisystem illness (1, 2, 47).

**Fig. 4.**
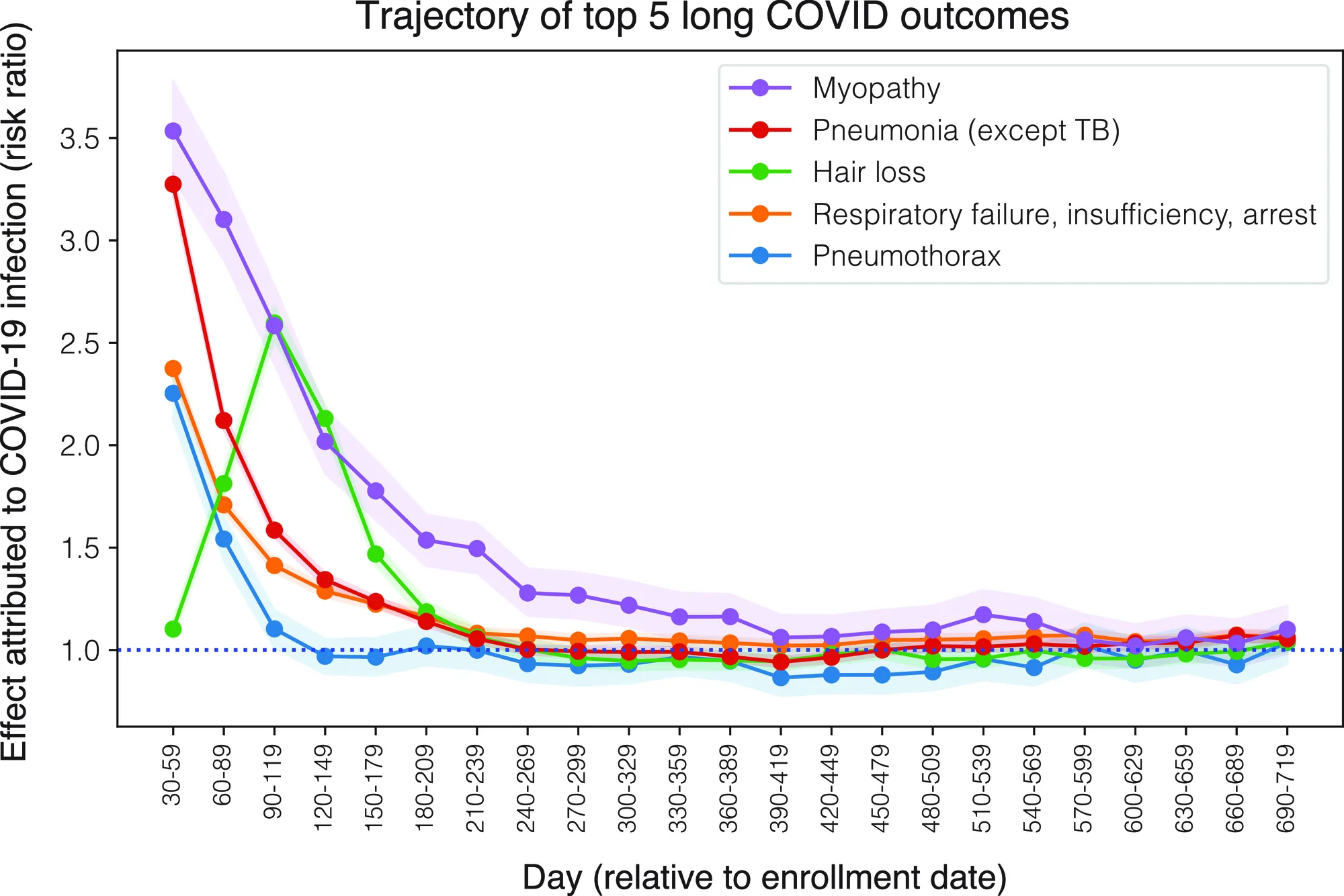
Trajectories of RR for the top five long COVID symptoms shown in Fig. 2*A*, as estimated by the test-based control design. We find that the average health of COVID-19 cases returns to baseline within 1 y after infection. Shading indicates Bonferroni-corrected 95% CI (error bars in Fig. 2 *A* and *B* and *Methods M3*).

After the first 120 d, we find reduced but still clinically meaningful impacts of long COVID. In the middle postacute period (days 120 to 359), the only symptoms with risk ratios greater than 1.1 are myopathy, hair loss, pulmonary embolism, acute pulmonary embolism, and respiratory failure, insufficiency, and respiratory arrest (two-sided bootstrap with Bonferroni correction). However, several outcomes—including pneumonia, shortness of breath, chest pain, and fatigue—continue to pose a significant public health burden (risk difference; *Methods M3*) despite exhibiting only modest risk ratios (between 1.0 and 1.1), owing to their high baseline prevalence in the general population. This suggests that while the intensity of risk fades, high-prevalence conditions remain burdensome at a population level up to a year after COVID-19 infection.

By 1 y postinfection, the measurable impact of long COVID appears to diminish significantly, with potential symptoms no longer clearly attributable to COVID-19. On days 360 to 719, there are no symptoms with significant risk ratios greater than 1.1 (two-sided bootstrap with Bonferroni correction). To further illustrate the trajectory of long COVID symptoms, we show the risk ratio of the top five early postacute period symptoms over the entire 2-y follow-up period (Fig. 4).

Downward trends indicate population-level recovery from COVID-19 by the end of the first year after infection. These findings differ from prior work showing significant harmful impacts of long COVID up to 2 (40) or 3 y (41) after infection. This is not to diminish the reality of such symptoms; rather, it suggests that they are unlikely to be specific to COVID-19. These symptoms may be due to a non-COVID condition that led to the initial hospital visit or COVID-19 testing, or some other factor. Ultimately, their cause is not discernible with the methods used here.

### Biases Due to Selective COVID-19 Testing.

We show that selection bias in who receives COVID-19 testing explains why our study design produces very different results compared to an unrestricted control design using the same data. We assessed these biases by rerunning an unrestricted control study, but replacing the COVID-19 cases with “placebo cases”: Individuals who were never diagnosed with COVID-19 and also received a negative (rather than positive) COVID-19 PCR test. We then compared these test-negative placebo cases to untested controls with the same medical history (*SI Appendix*, Figs. S2A and S106–S130). Receiving a negative COVID-19 test cannot plausibly cause harmful health effects. Thus, differences in future outcomes between the placebo cases and controls must be caused by non-COVID factors associated with testing (*SI Appendix*, Fig. S2 *B*, x-axis). This allows us to measure the long-term effects of these non-COVID factors among tested individuals, an approach known in epidemiology as a negative control exposure (48, 49).

We found that individuals who tested negative for COVID-19 experienced more long-term health problems compared to untested individuals with similar medical histories. Notably, 82.5% of outcomes show elevated risk (*RR* ≥ 1.1) on days 30 to 119 after a negative COVID-19 test (*SI Appendix*, Fig. S5). Furthermore, these elevated risks among test-negative individuals are positively correlated with those attributed to COVID-19 infection by the unrestricted control analysis (Spearman Corr. 0.48; *P* < 10^−4^; *SI Appendix*, Fig. S2 *B*, *Left*). This demonstrates that tested individuals shared non-COVID risk factors that are associated with long-term outcomes. This selection bias—where some individuals are more likely to be tested due to non-COVID factors—affects unrestricted control designs and explains in part their false attribution of long-term effects to COVID-19 infection. The test-based control design significantly reduced the correlation between outcomes associated with negative testing and COVID-19 infection (*P* < 10^−4^; Steiger’s Z-test; *SI Appendix*, Fig. S2 *B*, *Right*).

### Study Design Validation: Detecting Acute COVID-19 Symptoms.

We validated the ability of the test-based control design to identify from insurance claims the known clinical effects of COVID-19 by examining the well-established acute COVID-19 symptoms presenting within the first 30 d of infection (*SI Appendix*, Fig. S6). Our analysis detected known acute symptoms of mild and severe COVID-19 (50, 51) across multiple systems. We observed only one symptom, anorexia, not known to be caused by COVID-19, which may be coded due to appetite decrease caused by COVID-19 (52). These findings demonstrate that when properly analyzed using a test-based control design, insurance claims data can identify clinical outcomes known to occur acutely. This supports the utility of insurance claims data for studying longer-term effects of COVID-19.

## Discussion

As we continue to deal with the long-term impacts of the COVID-19 pandemic, it is important to identify genuine long COVID effects. We show that many previous studies were susceptible to selection bias, particularly due to differences in the health status of tested COVID-19 patients compared to untested controls. By using a study design in which patients were enrolled at the time of their first PCR test, we mitigated many of these biases and more accurately attributed health outcomes to COVID-19. This study design reveals a smaller, yet still clinically significant array of long COVID symptoms affecting multiple organ systems. This analysis suggests that while long COVID remains a serious concern, it may have fewer symptoms—and ones of shorter duration and lower overall severity—than previously thought.

Recent studies have addressed related but distinct questions using different outcome definitions and comparison groups. Meta-analyses and prevalence studies, including Lopez-Leon et al. (5) and the GBD analysis (20), synthesized symptoms among infected populations without individual-level uninfecteds and therefore estimate symptom burden rather than risk attributable to infection. Large EHR studies, including Al-Aly et al. (10), Subramanian et al. (12), the RECOVER analysis of Zang et al. (14), and the 2- and 3-y VA studies of Bowe et al. (40) and Cai et al. (41), used the unrestricted control designs and reported multiple system sequelae that attenuated over time but remained detectable for at least 3 y. We designed our test positive and unrestricted control cohorts to closely follow Al-Aly et al. (10), although our demographic matching is less fine-grained in that we used ZIP-3 rather than residential address.

The prospective RECOVER cohort (4) compared infected (N = 7,992) and uninfected (N = 1,347) participants, both test-based, but testing in the symptom-free volunteer control group was per protocol, i.e., not due to test seeking (and patients were matched on other factors), thereby less subject to test-seeking selection issues posed by our design. Test-negative patients who later developed COVID were crossed over to the infected group, further improving attribution. However, this design was used only to derive a 12-symptom PASC score at 6 mo. This provided a clinically based syndrome definition rather than an assessment of all outcome-specific attributable risks, was orders of magnitude smaller than our study, and included only cancer as a negative control outcome. Finally, our requirement that relative risks exceed 1.1 raised the threshold for what is considered a meaningful attributable effect, but the RECOVER initiative identified 37 significant long COVID symptoms at 6 mo using a higher RR threshold (RR > 1.5) from a preselected set of 44 self-reported symptoms. We analyzed 614 outcomes and showed that correcting for testing bias and comparing to a test-negative control group substantially reduces the number and duration of attributable effects. The RECOVER cohort reported a 15 mo follow-up of 4,323 test-positive patients, but without comparison to their uninfected control group (53).

Our study combined a large population database, matched test-negative controls, comparison to an unrestricted control analysis of the same data, long follow-up of both groups, 49 negative control outcomes, and a negative-test exposure, allowing us to directly quantify selective-testing bias. Across 614 outcomes, this design identified 43 COVID attributable outcomes at 30 to 119 d (similar to RECOVER’s identification of 37 symptoms at 6 mo), five at 120 to 359 d, and none at 360 to 719 d, indicating a narrower range of effects and shorter duration than prior studies without test-negative controls.

Our approach, while a substantial improvement over unrestricted control studies, has limitations. First, we relied on insurance claims data. While such insurance claims offer extensive coverage and longitudinal tracking, they are primarily designed for billing purposes rather than clinical research and may be lacking in clinical accuracy and granularity. It is thus reassuring that our study design is able to detect established acute COVID-19 symptoms (*SI Appendix*, Fig. S6). Our study could not include individuals who took at-home COVID-19 tests unless they presented to the health system. Moreover, by restricting the analysis to individuals that received PCR testing, the test-based control design narrows the target population, i.e., it is only able to estimate long COVID symptoms for the subgroup of individuals that sought healthcare. Our analyses show that our sample was geographically well-balanced (*SI Appendix*, Fig. S9) and well-balanced along the dimensions of age, race, income, and education (*SI Appendix*, Tables S1–S3), consistent with prior work on Komodo data (54). Patients in this study were followed longitudinally, linked by a hash of their first name, last name, gender, and date of birth (*Methods M1*), which has the risk of misidentification and improper linkage. However, we show that our study results are robust to efforts to filter out these overlapping patients (*SI Appendix*, Fig. S8).

While the test-based control design study mitigates the strong positive bias induced by previous unrestricted control designs, the negative control outcome results raise the possibility of a mild negative bias. Specifically, two of 49 negative control outcomes were significantly reduced in the test-positive individuals. This is likely due to conditioning on testing, which may lead to collider bias (36). For instance, patients who tested negative in their first PCR test may be arriving at the hospital seeking treatment for other ailments (e.g., cancer) or for a non-COVID respiratory illness that could produce a nonspecific prolonged viral syndrome (36). We reduced this bias by reweighting test-negative controls to match COVID-19 cases on medical history (55). While we were not able to adjust for unmeasured differences between test-positive and test-negative individuals, under relevant ignorability assumptions, such weighting enables us to target the average long-term effect of COVID-19 among COVID-19 cases who took a PCR test (56).

Several other factors could affect comparability of outcomes between the test-negative and test-positive groups. Tested individuals were required to survive 30 d after testing. 1.2% of test positives died in this period vs. 0.62% of test negatives. This survivor selection potentially enriches the surviving COVID-19 group with healthier individuals and can lead to a downward bias, but in this cohort the effect was very small. Furthermore, individuals in the test-negative control group could later test positive for COVID-19, again resulting in conservative effect estimates (*SI Appendix*, Fig. S7). We observed that between 0.6 to 1.1% of controls tested positive in any given 30-d period. In total, 4.2% tested positive within 119 d of enrollment (affecting outcome estimates for days 30 to 119), 10.1% tested positive within 359 d (affecting outcome estimates for days 120 to 359), and 20.2% tested positive within 2 y (affecting outcome estimates for days 360 to 719) (*SI Appendix*, Fig. S7).

Relative risks near 1.0 indicate long-term symptoms occurring at a similar rate in COVID-19 cases and test-negative controls. These data should not be interpreted as indicating that symptoms lasting more than a year in COVID-19 patients aren’t “real,” only that their etiology is uncertain. Finally, our requirement that relative risks exceed 1.1 set a threshold magnitude for what is considered a meaningful attributable effect. While the positive effects we detect are credible, there may be undetected small effects. Our study complements the RECOVER initiative (4), which focused on defining a long COVID phenotype using a much smaller prospective clinical cohort. They used a higher RR threshold of 1.5 to identify 37 significant long COVID symptoms at 6 mo from a set of 44 self-reported symptoms, similar to the 43 outcomes we identified 30 to 119 d after infection out of 614 candidates. However, they have not reported a longer-term contrast between their cases and controls.

Overall, our findings contribute substantively to the understanding of long COVID, reinforcing the importance of rigorous epidemiologic designs on large cohorts. We investigated in depth and demonstrated the degree to which large-scale studies using unrestricted control designs have likely substantially overestimated the number and duration of long COVID symptoms. Future long COVID studies should continue to leverage large, diverse datasets and use study designs that minimize the biases noted here. The approach we have taken can be applied to datasets from previous studies to develop a more complete understanding of the extent to which previous findings were due to bias or genuine long COVID effects. Finally, because of the limitations of observational population-based studies, a deeper understanding of the biological mechanisms underlying long COVID together with the development of new biomarkers will be essential for most reliably identifying and treating COVID’s long-term effects.

## Materials and Methods

### M1. Data Sources and Study Population.

We analyzed deidentified US health insurance claims from the Komodo Health database (57). The Komodo dataset aggregates insurance claims from multiple sources, including claims clearinghouses, pharmacy transaction networks, insurers, and government programs such as Medicare and Medicaid (58, 59). Komodo is estimated to cover over 95% of individuals in the United States, although this does not imply complete or continuous observation of each individual. The availability of claims varies with insurance coverage, healthcare utilization, and participation of the contributing sources; care received outside these sources, or care that does not generate insurance claims, is not observed. For cohort construction, Komodo provided two complementary extracts, comprising 14.4 billion claims from 244.7 million patients: a *Pandemic* extract containing patients with COVID-related activity (e.g., testing, vaccination, diagnosis) and a *Control* extract comprising a random sample of patients with no recorded COVID-19 diagnosis (the Control extract may include individuals who received COVID-19 testing and/or vaccination). The observation period ended on April 4, 2023. Following prior work, we restricted analyses to individuals with at least one claim in 2019 to ensure measurable medical history before enrollment. Additional details on the Komodo dataset and representativeness checks are provided in *SI Appendix*, Figs. S8 and S9 and *Extended Methods* S1.

We used the Area Deprivation Index (ADI) as a measure of socioeconomic disadvantage (national percentile 1 to 100; higher indicates greater deprivation). Because Komodo provides only the first three digits of ZIP code (ZIP-3), we mapped five-digit ZIP-level ADI values to ZIP-3 using population-weighted aggregation; individuals with multiple ZIP-3 values were assigned the mean of their linked ADI values. We also linked ZIP-3 to area-level demographic and socioeconomic measures from the 2016–2020 American Community Survey and the 2020 Decennial Census (mean family income; percent non-white or Hispanic; percent with less than a Bachelor’s degree), aggregated to ZIP-3 by population-weighted averaging (*SI Appendix*, *Extended Methods* S1.1).

### M2. Data Processing and Study Design.

#### COVID-related events.

We identified COVID-19 diagnoses and PCR tests using ICD-10, CPT, and HCPCS codes (full code lists in *SI Appendix*, *Extended Methods* S2). Briefly, confirmed COVID-19 diagnosis was defined using ICD-10 codes consistent with clinical coding guidance, and PCR testing was defined using standard PCR CPT/HCPCS codes.

#### Observational cohort construction.

We constructed three observational cohort studies. In all designs, follow-up for postacute outcomes began 30 d after enrollment, and individuals were required to be alive at 30 d postenrollment.

##### Unrestricted control design.

We implemented an unrestricted control design as used in prior long COVID studies, in which COVID-19 cases are matched to controls with no recorded COVID-19 diagnosis. Cases were individuals with a confirmed COVID-19 diagnosis between March 1, 2020, and March 1, 2021, excluding individuals with evidence of COVID-19 prior to their first confirmed diagnosis. Controls were sampled from the Control extract (no recorded COVID-19 diagnosis). To reduce seasonal differences in enrollment, controls were assigned enrollment dates drawn from the distribution of case enrollment dates.

##### Test-based control design (primary design).

In our primary design, patients were enrolled at their first PCR COVID-19 test between March 1, 2020, and March 1, 2021, excluding individuals with any evidence of COVID-19 prior to that test. Cases were test-positive individuals with no prior evidence of COVID-19 and a positive PCR result within 7 d of their first recorded PCR test. Controls were test-negative individuals whose first PCR test was negative and who had no evidence of COVID-19 through the following 7 d. To align enrollment timing and reduce seasonal differences, we down-sampled test-negative controls within each enrollment date to match the enrollment-date distribution of cases (*SI Appendix*, *Extended Methods* S2).

##### Placebo-case unrestricted control design (negative control exposure).

To quantify spurious associations induced by unrestricted control enrollment, we constructed an analogous unrestricted control study in which “cases” were defined as patients receiving a negative PCR test with no evidence of COVID-19 before or after the test (a negative control exposure). Because a negative PCR test cannot plausibly cause adverse health outcomes, nonzero effects in this design indicate residual bias. Controls were sampled from the Control extract with enrollment dates drawn from the placebo-case enrollment-date distribution (*SI Appendix*, *Extended Methods* S2).

#### Clinical outcomes.

We evaluated 614 clinical outcomes spanning multiple organ systems, including a set of negative control outcomes not plausibly caused by COVID-19. Outcomes were derived primarily from the Clinical Classifications Software Refined (CCSR), augmented with additional outcomes reported in prior long COVID studies and a curated set of negative controls (*SI Appendix*, Table S9 and *Extended Methods* S1.2). To ensure stable estimates, we excluded extremely rare outcomes. Outcomes were analyzed over four postenrollment windows: first 30 d (acute), 30 to 119 d (early postacute), 120 to 359 d (mid postacute), and 360 to 719 d (late postacute).

#### Covariates.

Across all designs, we balanced cases and controls on demographics and detailed pre-enrollment health history. Static covariates included sex/gender (indicator variables), age at enrollment, ADI, and ZIP-3 census-linked measures (mean family income; percent non-white or Hispanic; percent with less than a Bachelor’s degree). Pre-enrollment health status was measured over the 4 y prior to enrollment using the outcome set and measures of healthcare use (e.g., inpatient/outpatient/emergency visits and pharmacy use). We aggregated outcomes and utilization to monthly granularity and used a monthly indicator of whether each outcome occurred to reduce sensitivity to claim frequency. Missing age and ADI values were imputed using the mean. Additional covariate engineering details are provided in the *SI Appendix*.

### M3. Analysis.

#### Balancing pre-enrollment histories via synthetic-control weights.

We re-weighted controls using a synthetic-control approach to achieve a close balance of pre-enrollment medical histories and demographics between cases and controls. Let $X_{\text{control}}\in R^{n\times p}$ denote the control covariate matrix and ${\bar{X}}_{\text{cases}}\in R^{p}$ the vector of covariate means among cases. We derived nonnegative weights $W\in R^{n}$ by solving:

$$\begin{array}{c} \text{minimize}\;∥X_{\text{control}}^{⊤}W-{\bar{X}}_{\text{cases}}∥\\ \text{subject to}\;W\geq 0,\sum_{i\;=\;1}^{n}w_{i}=1. \end{array}$$

The covariates in X included pre-enrollment trends across the outcome set and summary measures of healthcare use, along with demographics (sex/gender, age, ADI, and ZIP-3 census-linked measures). This reweighting targets balance over the full 4-y pre-enrollment period, as illustrated in *SI Appendix*, Fig. S1 (implementation details in *SI Appendix*, *Extended Methods* S3).

#### Risk ratio and risk difference.

For each outcome and time window, we computed a risk ratio (RR) and risk difference (RD) comparing cases to the weighted controls. Let $Y_{i}$ denote the number of claims for a given outcome within a specified postenrollment time window for individual i. The RR was defined as

RR=∑i∈CasesYinCases∑j∈ControlswjYj∑j∈Controlswj,

and the RD (claims per month) as

$$RD=\frac{\sum_{i\in \text{Cases}}\;Y_{i}}{n_{\text{Cases}}\;m}-\frac{\sum_{j\in \text{Controls}}\;w_{j}Y_{j}}{\left(\sum_{j\in \text{Controls}}w_{j}\right)m},$$

where m is the number of months in the follow-up window. RR captures proportional changes in outcome incidence, while RD captures absolute excess burden (in claims per month), which is particularly relevant for population-level impact.

#### Uncertainty and multiple comparisons.

We estimated CI and *P*-values using a bootstrap with 1,000 resamples of patient records. For each bootstrap replicate, we sampled individuals with replacement and recomputed the statistic of interest; SE were estimated as the SD across bootstrap replicates. Statistical significance was assessed using Gaussian 95% CI (estimate ±1.96 SE). We controlled for multiple comparisons using a Bonferroni correction to maintain an overall α = 0.05 across outcomes within each analysis window (details in *SI Appendix*, *Extended Methods* S3).

## Supplementary Material

Appendix 01 (PDF)

## Data Availability

The insurance claims data used in this study were obtained from the Komodo Health database under a data use agreement with Stanford University. Due to data use agreement restrictions and patient privacy requirements, the raw claims data cannot be shared publicly. Researchers interested in accessing similar data may contact Komodo Health (https://www.komodohealth.com) to inquire about data access for academic research (57). The analysis code used in this study is available at https://github.com/snap-stanford/longcovid. Supplementary tables and aggregate summary statistics that support the findings are provided in the *SI Appendix*.

## References

[1] Z. Al-Aly, E. Topol, Solving the puzzle of long Covid. Science **383**, 830–832 (2024). 10.1126/science.adl0867 38386747

[2] H. E. Davis, L. McCorkell, J. M. Vogel, E. J. Topol, Long COVID: Major findings, mechanisms and recommendations. Nat. Rev. Microbiol. **21**, 133–146 (2023). 10.1038/s41579-022-00846-2 PMC9839201 36639608

[3] A. Nalbandian , Post-acute COVID-19 syndrome. Nat. Med. **27**, 601–615 (2021). 10.1038/s41591-021-01283-z PMC8893149 33753937

[4] T. Thaweethai , Development of a definition of postacute sequelae of SARS-CoV-2 infection. JAMA **329**, 1934–1946 (2023). 10.1001/jama.2023.8823 PMC10214179 37278994

[5] S. Lopez-Leon , More than 50 long-term effects of COVID-19: A systematic review and meta-analysis. Sci. Rep. **11**, 16144 (2021). 10.1038/s41598-021-95565-8 PMC8352980 34373540

[6] Y. Xie, B. Bowe, Z. Al-Aly, Burdens of post-acute sequelae of COVID-19 by severity of acute infection, demographics and health status. Nat. Commun. **12**, 6571 (2021). 10.1038/s41467-021-26513-3 PMC8589966 34772922

[7] Y. Xie, E. Xu, B. Bowe, Z. Al-Aly, Long-term cardiovascular outcomes of COVID-19. Nat. Med. **28**, 583–590 (2022). 10.1038/s41591-022-01689-3 PMC8938267 35132265

[8] S. Spudich, A. Nath, Nervous system consequences of COVID-19. Science **375**, 267–269 (2022). 10.1126/science.abm2052 35050660

[9] H. E. Davis , Characterizing long COVID in an international cohort: 7 months of symptoms and their impact. EClinicalMedicine **38**, 101019 (2021). 10.1016/j.eclinm.2021.101019 PMC8280690 34308300

[10] Z. Al-Aly, Y. Xie, B. Bowe, High-dimensional characterization of post-acute sequelae of COVID-19. Nature **594**, 259–264 (2021). 10.1038/s41586-021-03553-9 33887749

[11] Z. Al-Aly, B. Bowe, Y. Xie, Long COVID after breakthrough SARS-CoV-2 infection. Nat. Med. **28**, 1461–1467 (2022). 10.1038/s41591-022-01840-0 PMC9307472 35614233

[12] A. Subramanian , Symptoms and risk factors for long COVID in non-hospitalized adults. Nat. Med. **28**, 1706–1714 (2022). 10.1038/s41591-022-01909-w PMC9388369 35879616

[13] A. V. Ballering, S. K. R. van Zon, T. C. Olde Hartman, J. G. M. Rosmalen, Persistence of somatic symptoms after COVID-19 in the Netherlands: An observational cohort study. Lancet **400**, 452–461 (2022). 10.1016/S0140-6736(22)01214-4 PMC9352274 35934007

[14] C. Zang , Data-driven analysis to understand long COVID using electronic health records from the RECOVER initiative. Nat. Commun. **14**, 1948 (2023). 10.1038/s41467-023-37653-z PMC10080528 37029117

[15] M. Taquet , Neurological and psychiatric risk trajectories after SARS-CoV-2 infection: An analysis of 2-year retrospective cohort studies including 1 284 437 patients. Lancet Psychiatry **9**, 815–827 (2022). 10.1016/S2215-0366(22)00260-7 PMC9385200 35987197

[16] I. Katsoularis , Risks of deep vein thrombosis, pulmonary embolism, and bleeding after covid-19: Nationwide self-controlled cases series and matched cohort study. BMJ **377**, e069590 (2022). 10.1136/bmj-2021-069590 PMC8984137 35387772

[17] H. Moldofsky, J. Patcai, Chronic widespread musculoskeletal pain, fatigue, depression and disordered sleep in chronic post-SARS syndrome; A case-controlled study. BMC Neurol. **11**, 37 (2011). 10.1186/1471-2377-11-37 PMC3071317 21435231

[18] A. Carfì , Persistent symptoms in patients after acute COVID-19. JAMA **324**, 603–605 (2020). 10.1001/jama.2020.12603 PMC7349096 32644129

[19] C. H. Sudre , Attributes and predictors of long COVID. Nat. Med. **27**, 626–631 (2021). 10.1038/s41591-021-01292-y PMC7611399 33692530

[20] S. W. Hanson , Estimated global proportions of individuals with persistent fatigue, cognitive, and respiratory symptom clusters following symptomatic COVID-19 in 2020 and 2021. JAMA **328**, 1604–1615 (2022). 10.1001/jama.2022.18931 PMC9552043 36215063

[21] I. C. H. Lam , Long-term post-acute sequelae of COVID-19 infection: A retrospective, multi-database cohort study in Hong Kong and the UK. EClinicalMedicine **60**, 102000 (2023). 10.1016/j.eclinm.2023.102000 PMC10173760 37197226

[22] R. A. Evans , Physical, cognitive, and mental health impacts of COVID-19 after hospitalisation (PHOSP-COVID): a UK multicentre, prospective cohort study. Lancet Respir. Med. **9**, 1275–1287 (2021). 10.1016/S2213-2600(21)00383-0 PMC8497028 34627560

[23] G. Douaud , SARS-CoV-2 is associated with changes in brain structure in UK Biobank. Nature **604**, 697–707 (2022). 10.1038/s41586-022-04569-5 PMC9046077 35255491

[24] V. O. Puntmann , Long-term cardiac pathology in individuals with mild initial COVID-19 illness. Nat. Med. **28**, 2117–2123 (2022). 10.1038/s41591-022-02000-0 PMC9556300 36064600

[25] N. L. DePace, J. Colombo, Long-COVID syndrome and the cardiovascular system: A review of neurocardiologic effects on multiple systems. Curr. Cardiol. Rep. **24**, 1711–1726 (2022). 10.1007/s11886-022-01786-2 PMC9524329 36178611

[26] B. Raman, D. A. Bluemke, T. F. Lüscher, S. Neubauer, Long COVID: Post-acute sequelae of COVID-19 with a cardiovascular focus. Eur. Heart J. **43**, 1157–1172 (2022). 10.1093/eurheartj/ehac031 PMC8903393 35176758

[27] E. Xu, Y. Xie, Z. Al-Aly, Long-term gastrointestinal outcomes of COVID-19. Nat. Commun. **14**, 983 (2023). 10.1038/s41467-023-36223-7 PMC9992516 36882400

[28] E. J. Thompson , Long COVID burden and risk factors in 10 UK longitudinal studies and electronic health records. Nat. Commun. **13**, 3528 (2022). 10.1038/s41467-022-30836-0 PMC9240035 35764621

[29] M. Roessler , Post-COVID-19-associated morbidity in children, adolescents, and adults: A matched cohort study including more than 157,000 individuals with COVID-19 in Germany. PLoS Med. **19**, e1004122 (2022). 10.1371/journal.pmed.1004122 PMC9648706 36355754

[30] M. Taquet , Incidence, co-occurrence, and evolution of long-COVID features: A 6-month retrospective cohort study of 273,618 survivors of COVID-19. PLoS Med. **18**, e1003773 (2021). 10.1371/journal.pmed.1003773 PMC8478214 34582441

[31] E. Xu, Y. Xie, Z. Al-Aly, Long-term neurologic outcomes of COVID-19. Nat. Med. **28**, 2406–2415 (2022). 10.1038/s41591-022-02001-z PMC9671811 36138154

[32] T. B. Høeg, S. Ladhani, V. Prasad, How methodological pitfalls have created widespread misunderstanding about long COVID. BMJ Evid. Based Med. **29**, 142–146 (2024). 10.1136/bmjebm-2023-112338 PMC11137465 37748921

[33] D. Munblit , A core outcome set for post-COVID-19 condition in adults for use in clinical practice and research: An international Delphi consensus study. Lancet Respir. Med. **10**, 715–724 (2022). 10.1016/S2213-2600(22)00169-2 PMC9197249 35714658

[34] D. M. Altmann, E. M. Whettlock, S. Liu, D. J. Arachchillage, R. J. Boyton, The immunology of long COVID. Nat. Rev. Immunol. **23**, 618–634 (2023). 10.1038/s41577-023-00904-7 37433988

[35] F. M. Iqbal , Characteristics and predictors of acute and chronic post-COVID syndrome: A systematic review and meta-analysis. EClinicalMedicine **36**, 100899 (2021). 10.1016/j.eclinm.2021.100899 PMC8141371 34036253

[36] G. J. Griffith , Collider bias undermines our understanding of COVID-19 disease risk and severity. Nat. Commun. **11**, 5749 (2020). 10.1038/s41467-020-19478-2 PMC7665028 33184277

[37] Z. Amin-Chowdhury, S. N. Ladhani, Causation or confounding: Why controls are critical for characterizing long COVID. Nat. Med. **27**, 1129–1130 (2021). 10.1038/s41591-021-01402-w 34140704

[38] J. Ž Nikolich, C. J. Rosen, Toward comprehensive care for long Covid. N. Engl. J. Med. **388**, 2113–2115 (2023). 10.1056/NEJMp2304550 37158440

[39] J. Szanyi, S. Howe, T. Blakely, The importance of reporting accurate estimates of long COVID prevalence. Lancet **403**, 1136–1137 (2024). 10.1016/S0140-6736(23)01120-0 38521555

[40] B. Bowe, Y. Xie, Z. Al-Aly, Postacute sequelae of COVID-19 at 2 years. Nat. Med. **29**, 2347–2357 (2023). 10.1038/s41591-023-02521-2 PMC10504070 37605079

[41] M. Cai, Y. Xie, E. J. Topol, Z. Al-Aly, Three-year outcomes of post-acute sequelae of COVID-19. Nat. Med. **30**, 1564–1573 (2024). 10.1038/s41591-024-02987-8 PMC11186764 38816608

[42] S. Suissa, Immortal time bias in pharmacoepidemiology. Am. J. Epidemiol. **167**, 492–499 (2008). 10.1093/aje/kwm324 18056625

[43] M. A. Hernán, J. M. Robins, Causal Inference: What If (Chapman & Hall/CRC, 2020).

[44] E. A. Stuart, Matching methods for causal inference: A review and a look forward. Stat. Sci. **25**, 1–21 (2010). 10.1214/09-STS313 PMC2943670 20871802

[45] A. Abadie, Using synthetic controls: Feasibility, data requirements, and methodological aspects. J. Econ. Lit. **59**, 391–425 (2021).

[46] X. Wu, E. Sverdrup, M. D. Mastrandrea, M. W. Wara, S. Wager, Low-intensity fires mitigate the risk of high-intensity wildfires in California’s forests. Sci. Adv. **9**, eadi4123 (2023). 10.1126/sciadv.adi4123 PMC10637742 37948522

[47] H. Crook, S. Raza, J. Nowell, M. Young, P. Edison, Long covid—mechanisms, risk factors, and management. BMJ **374**, n1648 (2021). 10.1136/bmj.n1648 34312178

[48] M. Lipsitch, E. Tchetgen Tchetgen, T. Cohen, Negative controls: A tool for detecting confounding and bias in observational studies. Epidemiology **21**, 383–388 (2010). 10.1097/EDE.0b013e3181d61eeb PMC3053408 20335814

[49] E. Sanderson, C. Macdonald-Wallis, G. Davey Smith, Negative control exposure studies in the presence of measurement error: Implications for attempted effect estimate calibration. Int. J. Epidemiol. **47**, 587–596 (2018). 10.1093/ije/dyx213 PMC5913619 29088358

[50] C. Huang , Clinical features of patients infected with 2019 novel coronavirus in Wuhan, China. Lancet **395**, 497–506 (2020). 10.1016/S0140-6736(20)30183-5 PMC7159299 31986264

[51] W.-J. Guan , Clinical characteristics of coronavirus disease 2019 in China. N. Engl. J. Med. **382**, 1708–1720 (2020). 10.1056/NEJMoa2002032 PMC7092819 32109013

[52] A. T. Z. B. Høier, N. Chaaban, B. V. Andersen, Possibilities for maintaining appetite in recovering COVID-19 patients. Foods **10**, 464 (2021). 10.3390/foods10020464 PMC7923765 33672528

[53] T. Thaweethai , Long COVID trajectories in the prospectively followed RECOVER-adult US cohort. Nat. Commun. **16**, 9557 (2025). 10.1038/s41467-025-65239-4 PMC12623977 41249167

[54] D. H. Jaffe, L. Lee, S. Huynh, T. P. Haskell, Health inequalities in the use of telehealth in the United States in the lens of COVID-19. Popul. Health Manag. **23**, 368–377 (2020). 10.1089/pop.2020.0186 32816644

[55] M. A. Hernán, S. Hernández-Díaz, J. M. Robins, A structural approach to selection bias. Epidemiology **15**, 615–625 (2004). 10.1097/01.ede.0000135174.63482.43 15308962

[56] J. J. Heckman, H. Ichimura, P. Todd, Matching as an econometric evaluation estimator. Rev. Econ. Stud. **65**, 261–294 (1998).

[57] Komodo Health, US health insurance claims. Komodo Health database. https://www.komodohealth.com/. Accessed 9 June 2023.

[58] Komodo Health, Are “counts” your best bet? Three examples show why commercial teams should dig deeper. (2026). https://www.komodohealth.com/perspectives/are-counts-your-best-bet-3-examples-show-why-commercial-teams-should-dig-deeper/. Accessed 10 December 2026.

[59] Komodo Health, Healthcare Map: The data behind every defensible answer. (2026). https://www.komodohealth.com/product/healthcare-map/. Accessed 10 December 2026.

