# Supplementary material for: Revised estimates of types and duration of long COVID symptoms based on claims records from 245 million US patients
Source: Proc Natl Acad Sci U S A. 2026 Oct 27;123(44):e2604414123. doi: 10.1073/pnas.2604414123 (PMC13732191; doi:10.1073/pnas.2604414123)
Supplement: Supplementary file 1 [file pnas.2604414123.sapp.pdf]

# Supporting Information for

## Revised estimates of types and duration of long COVID symptoms based on claims records from 245 million US patients

**Hamed Nilforoshan, Julia Reisler, Erfan Jahanparast, Michael Moor, Steven N. Goodman, Stefan Wager, and Jure Leskovec**

**Steven N. Goodman**
**E-mail: steve.goodman@stanford.edu**

**This PDF file includes:**

Supporting Methods
Figs. S1 to S155
Tables S1 to S9
SI References

## Supporting Information Guide

**Extended methods.** Extended Methods S1–S3 provide full methodological details complementing the main text (data sources and governance, code lists, cohort construction/attrition, outcome construction, covariates, and statistical implementation). Related methodological/design figures: Figs. S1–S4.

**Robustness checks.**

- *Patient identification. Fig. S8* shows that results are robust to filtering out individuals when there is a likely collision between two patients with the same name, gender, and date of birth.
- *Enrollment period.* Fig. S12 confirms that risk ratios estimated separately in two consecutive 6-month enrollment intervals are consistent.
- *Matching on age category (18 and under, 18-65, and 65+) in the unrestricted control design.* Fig. S13 shows that unrestricted control analysis risk ratios are robust to matching on age category (rather than average age); Table S8 reports post-weighting demographic balance for the age-distribution-matched design.
- *High vs. low incidence rate settings.* Fig. S14 confirms that risk ratios estimated separately in high vs. low incidence rate ZIP-3s are consistent.

**Population representations across ZIP codes.** Fig. S9 demonstrates that the Komodo dataset is geographically well-balanced, using the U.S. Census population distribution by ZIP code as a reference.

**Comparability of COVID-19 cases and unweighted controls across designs.** Tables S4–S7 compare the pre-weighting incidence of claims between COVID-19 cases and unweighted controls. For the unrestricted control design, standardized mean differences are large, (Tables S4 and S5), reflecting baseline differences between COVID-19-positive cases and unrestricted controls. For the test-based control design, the same comparison shows that test-positive and test-negative cohorts already have comparable medical histories prior to weighting (Tables S6 and S7).

**Risk ratios for all 614 outcomes and study designs.** Figs. S15–S80 show the full list of 614 outcomes and the estimated effect across all three study designs (unrestricted control, test-based control, and unrestricted control with placebo cases).

**Geographic and temporal balance between the test-positive and test-negative cohorts.** Figs. S10 and S11 show that the test-positive and test-negative cohorts of the test-based control design have highly aligned geographic distributions across each of the four enrollment quarters, at both the ZIP-3 level and the state level.

**Medical histories across all outcomes.** Figs. S81–S105, S106–S130, and S131–S155 show the fraction of patients with relevant claims over time for all diagnostic categories within each experiment:

- Test-based control design: Figs. S81–S105.
- Unrestricted control design with placebo cases (negative COVID-19 test recipients): Figs. S106–S130.
- Unrestricted control design: Figs. S131–S155.

**Diagnosis groups.** Table S9 contains a comprehensive mapping of diagnosis groups to their corresponding ICD-10 and ICD-9 codes.

## Extended Methods

Additional methodological details complementing the main text, including data sources and governance, code lists, cohort construction and attrition, outcome construction, covariate engineering, and statistical implementation.

### Extended Methods S1. Data sources and study population.

***Komodo Health.*** We use health insurance claims data from a sample of individuals in the United States, provided by the company Komodo Health (Komodo). The data are de-identified insurance claims which are collected and transmitted to Komodo by participating healthcare providers and payers (1). Komodo data is de-identified per the standard specified in Section §164.514(a) of the Health Insurance Portability and Accountability Act of 1996 (HIPAA) Privacy Rule. Data de-identification is attested to through a formal determination by a qualified expert per Section §164.514(b)(1) of the HIPAA Privacy Rule. All data provided by Komodo is stored on a secure server behind a firewall. Data handling and analysis was conducted in accordance with Komodo policies and in accordance with the guidelines of the Stanford University Institutional Review Board.

Komodo traces the longitudinal patient journey of over 330 million unique U.S. patient lives, across multiple healthcare payers and providers. Komodo links patients across multiple data sources using a hash of the first name and last name coded by soundex(2), date of birth, and gender of the patient. The Komodo dataset contains date-stamped hospital visits (inpatient, outpatient, emergency room, etc.), diagnosis codes (ICD-9, ICD-10), procedure codes (CPT, HCPCS, and ICD-10-PCS), drug prescription events (NDC codes). While the sample is not random sample, we find—consistent with prior work(3)—Komodo data is geographically representative (i.e. an approximately unbiased sample of different ZIP codes within each State), and representative along the dimensions of age, race, income, and education (Fig. S9 and Tables S1–S3). Furthermore, Komodo data is a widely used standard in large-scale studies of health outcomes across many different areas including COVID-19 treatments (4–6), telehealth (3, 7), drug safety (8), mental health (9), time-to-treatment (10), cancer incident (11), and health resource utilization (12).

**Komodo extracts used for cohort construction.** The raw data provided by Komodo consist of two datasets. The first dataset, the *Pandemic* dataset, consists of 48,812,613,382 claims from 228,010,550 patients (mean 214.0 claims per patient). The Pandemic dataset consists of all patients with any COVID-related claim including COVID-19 testing, vaccination, diagnosis, etc. The second dataset, the *Control* dataset, consists of 5,759,362,767 claims from 33,749,570 patients (mean 170.6 claims per patient). The Control dataset is a random sample of all patients in the Komodo database that had no evidence of COVID-19 diagnosis, but includes individuals who received COVID-19 testing and vaccination. The observation period of both datasets ended on April 4th, 2023.

Following prior work (13), we filter these datasets to only include individuals who had at least one claim in the year 2019 (*Pandemic* dataset: $n = 158{,}213{,}552$; *Control* dataset: $n = 18{,}084{,}992$).

### Extended Methods S1.1 Area-level socioeconomic and demographic linkage.

***Area Deprivation Index.*** We use the Area Deprivation Index (ADI) as a measure of socioeconomic disadvantage based on patient residential location (14). Patients in the cohort were linked via ZIP code to their residential ADI, a national percentile ranking from 1 to 100, where a higher ADI score indicates a higher level of socioeconomic disadvantage. Komodo data only includes the first three digits of the patient ZIP code to ensure anonymity. Thus, we aggregate neighborhood ADI measures for each five-digit ZIP code to the first three digits using a population weighted average. Individuals with multiple addresses with different ZIP codes were assigned the average of all their assigned ADIs.

***U.S. Census.*** We obtained data on demographic and socioeconomic features from the 2016–2020 American Community Survey (ACS) census estimates (15) and 2020 Decennial Census (16). Specifically, we retrieved mean family income, percentage of population with less than a Bachelor's degree, and percentage of population that is non-white or Hispanic for each ZIP code. To ensure patient anonymity, Komodo data only includes the first three digits of the patient ZIP code. Thus, we aggregate five-digit ZIP codes to the first three digits using a population weighted average. While only ZIP code level information was available to describe patient socioeconomic and racial demographics, area-level measures have been demonstrated by prior work to meaningfully explain health outcomes (17, 18).

**Extended Methods S1.2 High-dimensional clinical outcomes.** We consider 614 health outcomes which were aggregated from multiple sources, consisting of 565 outcomes which could plausibly be caused by COVID-19, and 49 outcomes which are negative control outcomes (outcomes which are not plausibly caused by COVID-19 and which are used to analyze the extent of bias in our estimates).

We obtained an initial set of 695 outcomes by combining (a) an outcomes list generated from the Clinical Classification Software Refined (CCSR) (19), consistent with a previous high-dimensional study of long-term COVID-19 effects (13) (b) additional outcomes plausibly caused by COVID-19 from previous studies (20–22) and (c) manually generating a list of outcomes unlikely to be caused by COVID-19. The CCSR categorizes ICD-10-CM codes into clinically meaningful groups, and was developed as part of the Healthcare Cost and Utilization Project sponsored by the Agency for Healthcare Research and Quality. We categorized these 695 outcomes into 611 outcomes plausibly caused by COVID-19, and 84 outcomes which are negative control outcomes (23). Our initial list of outcomes consisted of 695 outcomes, which we filtered down (including 49 negative controls) by excluding outcomes which were extremely sparse (i.e., those that occurred in less than 0.0001% of patients in the pre-enrollment period across all cohorts).

### Extended Methods S2. Data processing and cohort construction.

***COVID-related events.*** We used the following ICD-10, CPT, and HCPCS codes to identify COVID-19 related diagnoses and tests:

- **Confirmed COVID-19 positive diagnosis**: ICD-10 code B97.29 before April 1, 2020, or ICD-10 Code U07.1 at any time.
- **Evidence of COVID-19**: confirmed COVID-19 diagnosis, or suspected COVID-19 infection, or history of COVID-19 from ICD-10 codes U07.2, U08.9, U09.9, or Z86.16.
- **COVID-19 PCR test**: CPT codes 87635, 87636, 87637, 0202U, 0223U, 0225U, 0240U, 0241U; HCPCS codes U0001, U0003, or U0005.

***Observational study cohort construction (full definitions and attrition).*** We construct three observational cohort studies. In all designs, post-acute follow-up begins 30 days after enrollment, and individuals are required to be alive 30 days after enrollment.

**Unrestricted control design.** We first use an unrestricted control design based on prior high-dimensional studies of long COVID(13, 20–22), in which COVID-positive patients (COVID-19 cases) are matched with patients for which there is no evidence of COVID-19 diagnosis (control). To construct the COVID-19 cases group, we sample all individuals who had a confirmed positive COVID-19 diagnosis between March 1st, 2020 and March 1st, 2021 ($n = 10{,}445{,}894$), filtering for only those who had no evidence of COVID-19 (e.g., suspected COVID-19) prior to their first confirmed COVID-19 positive diagnosis date ($n = 10{,}431{,}860$). To examine post-acute outcomes, we then selected those that were alive 30 days after positive COVID-19 diagnosis ($n = 10{,}118{,}877$).

Following prior work (13, 22), we generate a "control" group (no COVID-19 evidence) by selecting all individuals in our Control dataset who had no record of any COVID-19 diagnosis ($n = 18{,}084{,}711$). To eliminate any effect of seasonality in cohort enrollment, we sampled cohort enrollment dates for each individual in the control group from the distribution of cohort enrollment dates in the COVID-19 cases (i.e. the day in which the COVID-19 case tested positive). For the control group as well, we further filter for individuals that were alive on their enrollment date ($n = 17{,}728{,}714$), and finally for those who were alive 30 days after enrollment into the cohort ($n = 17{,}717{,}308$).

**Test-based control design (primary design).** In the proposed study design, patients are enrolled into the cohort the first time they take a PCR COVID-19 test between March 1st, 2020 and March 1st, 2021 ($n = 39{,}967{,}054$), if they had no prior evidence of COVID-19 diagnosis ($n = 39{,}199{,}683$). COVID-19 cases are defined as those who received a positive test result subsequent to testing, that is, they were diagnosed with COVID-19 within 7 days of testing ($n = 2{,}866{,}888$). We filter the COVID-19 cases for only those that remained alive 30 days after their enrollment date ($n = 2{,}832{,}575$). The control group is defined as those who received a negative test result, that is, they had no evidence of COVID-19 within 7 days of testing ($n = 36{,}320{,}411$). We further filter the control group for only individuals who were alive 30 days after the enrollment date ($n = 36{,}095{,}303$).

To mitigate seasonality, we down-sampled the control group to ensure alignment between its enrollment date distributions and the treatment group enrollment dates ($n = 14{,}162{,}875$). To do so, for each day, we calculate the minimum ratio between control and COVID-19 cases enrolled for each day, rounded down to an integer value (ratio = 5:1). We down-sample the control units for each enrollment day so that the ratio of control to COVID-19 cases is 5:1 for each of the enrollment dates.

**Unrestricted control design with placebo cases (negative COVID-19 PCR test recipients).** To validate the prior study design, we conduct an analogous cohort study in which we replace the COVID-19 cases with a group of patients that received a negative COVID-19 PCR test, which cannot plausibly have severe adverse health effects. We refer to these as "placebo" cases. This approach is also known as a negative control exposure in the epidemiology literature(24, 25). Here, we use negative COVID-19 tests, by sampling all individuals who received their first PCR COVID-19 test between March 1st, 2020 and March 1st, 2021 ($n = 39{,}967{,}054$), and only including those for whom there was no evidence of a COVID-19 diagnosis before or after their test date ($n = 27{,}439{,}975$). As before, we further select for those that were alive 30 days after enrollment into the cohort, that is, those who were alive 30 days after receiving their first COVID-19 test ($n = 27{,}263{,}919$).

We generate a "control" group analogous the to the prior study, which consists of all individuals in the Control dataset ($n = 18{,}084{,}711$). We use an enrollment date randomly sampled from the distribution of enrollment dates in the null treatment group, i.e. COVID-19 test dates. We further filter for those who were alive on their enrollment date ($n = 17{,}737{,}596$) and were alive for 30 days after enrollment into the study ($n = 17{,}726{,}124$).

***Covariates.*** Across all experiments, we match on several static covariates used in prior work (13): gender (one-hot encoded as male, female, and unknown/other), age at time of enrollment (years), and area deprivation index (ADI), which are taken or calculated from the patient data. We additionally match on U.S. Census demographic features described in Supporting Methods S1.1 (mean family income, non-white or Hispanic percentage, and no-bachelor degree percentage) by first aggregating these features at the ZIP-3 level and then mapping them to respective patients residing in each ZIP-3 area (Tables S1–S3).

We also measure pre-enrollment health status from the four years prior to enrollment date. We follow prior work (13) and use a high-dimensional set of health outcomes from before treatment. We also use several high-level proxies of healthcare usage: the number of inpatient visits, outpatient visits, home visits, emergency room visits, ambulance visits, pharmacy visits, and non-hospital institution visits. Lastly, we include a marker of data quality, the number of months prior to the testing date in which insurance data was payer-complete.

We aggregate each of the health status outcomes to the monthly granularity, creating 48 features for all of the 614 outcomes and proxies of healthcare usage for each of the four years (48 months) prior to study enrollment. To reduce noise from claim frequency variations, we binarize each outcome at the monthly level, indicating whether any claim occurred in that month. Before balancing covariates, we normalize the covariate matrix $X$ to the unit norm. For missing age and ADI values, we impute the feature using the mean.

## Extended Methods S3. Analysis details.

***Matching pre-enrollment medical histories and demographics.*** We re-weight the control sample using a synthetic-control-type approach (26–29) to eliminate biases that can be explained away using observed pre-exposure covariates (30–32). Our re-weighting is designed to achieve fine-grained balance of medical histories and patient demographics between COVID-19 (or placebo) cases and controls over the four-year period before enrollment, as illustrated in Fig. S1. We derive weights by solving the following optimization problem using the `cvxpy` package in `Python` with the `MOSEK` solver,

$$\begin{aligned} \text{minimize} \quad & \|X_{control}^T W - \bar{X}_{cases}\| \\ \text{subject to} \quad & W \geq 0 \\ & \sum_{i=1}^{n} w_i = 1, \end{aligned}$$

where:

- $X_{control}^T$ is the control matrix of covariates, transposed,
- $\bar{X}_{cases}$ is the vector of treatment covariate means,
- $W$ is the weight vector consisting of $n$ weights, one for each control unit.

We balance covariates ($X$) that capture individuals' medical history prior to enrollment. These include health trends across all 614 outcomes and summary measures of healthcare utilization. Consistent with prior work (13), we also include additional static demographic covariates in $X$, such as gender, age at enrollment, and the area deprivation index. A full description of the covariates used for balancing is provided in Supporting Methods S2 (Covariates).

***Risk ratio and risk difference.*** We assess the effect of COVID-19 on each of the 614 outcomes by computing a relative risk ratio (RR) and risk difference (RD) for the occurrence of the outcome over several time windows in the subsequent two years. To calculate the RR and RD for each outcome, we compare the incidence of the outcome in the cases group to the incidence in the weighted control group. The risk ratio (RR) is defined as the ratio of the probability of the outcome occurring in the cases group to the probability of the outcome occurring in the weighted control group,

$$RR = \frac{\sum_{i \in \text{Cases}} Y_i}{n_{\text{Cases}}} \Bigg/ \frac{\sum_{j \in \text{Control}} w_j Y_j}{\sum_{j \in \text{Control}} w_j},$$

where $Y_i$ represents the number of claims for the outcome per time period (e.g., days 30–120) for individual $i$, $n_{\text{Cases}}$ is the number of individuals in the cases group, $w_j$ is the weight assigned to individual $j$ in the control group, derived from the synthetic controls, and $Y_j$ is the number of claims per time period for individual $j$ in the control group.

The risk difference (RD, in claims per month) is defined as the difference in the average number of outcome occurrences per month, between the cases group and the weighted control group,

$$RD = \frac{\sum_{i \in \text{Cases}} Y_i}{n_{\text{Cases}} \times m} - \frac{\sum_{j \in \text{Control}} w_j Y_j}{\sum_{j \in \text{Control}} w_j \times m},$$

where $m$ is the number of months in the follow-up period, that is, 3 months for days 30–120, 8 months for days 120–360, and 12 months for days 360–720.

Relative Risk (RR) and Risk Difference (RD) offer complementary insights into the impact of COVID-19 on health outcomes. RR indicates the proportional change in risk due to COVID-19 infection, making it valuable for comparing effects across varying baseline risks. However, its interpretation depends on baseline incidence: a large RR for rare outcomes might represent only a small absolute risk increase, while a moderate RR for common outcomes can imply substantial absolute differences. Conversely, RD measures the absolute change in risk directly, highlighting the actual disease burden. This makes RD especially relevant for public health decisions and resource allocation. However—unlike the RR which is unit free—the definition of the RD depends closely on the mechanics of how claims are recorded; for example, if a healthcare system doubles the number of claims recorded per health event then the RD would double.

RRs and RDs are computed for each outcome across the following four time periods:

- **Acute period**: 1–30 days after enrollment (index event).
- **Early post-acute period**: 30–120 days after enrollment.
- **Middle post-acute period**: 120–360 days after enrollment.
- **Late post-acute period**: 360–720 days after enrollment.

***Statistical analysis.*** Confidence intervals and p-values were estimated using a bootstrap with 1,000 resamples. For each bootstrap replicate, patient records were sampled with replacement and the statistic of interest was computed; the standard error was then computed as the standard deviation of the bootstrap replicates (33). Statistical significance was assessed using Gaussian 95% confidence intervals obtained using the bootstrap standard error (i.e., estimate $\pm$ 1.96 standard errors). Multiple comparisons were controlled using a Bonferroni correction to maintain an overall significance level of $\alpha = 0.05$. Steiger's $Z$-test was used to compare pairs of Spearman correlations, and hypothesis tests for single Spearman correlation coefficients were computed using two-sided Student's $t$-tests (34–36).

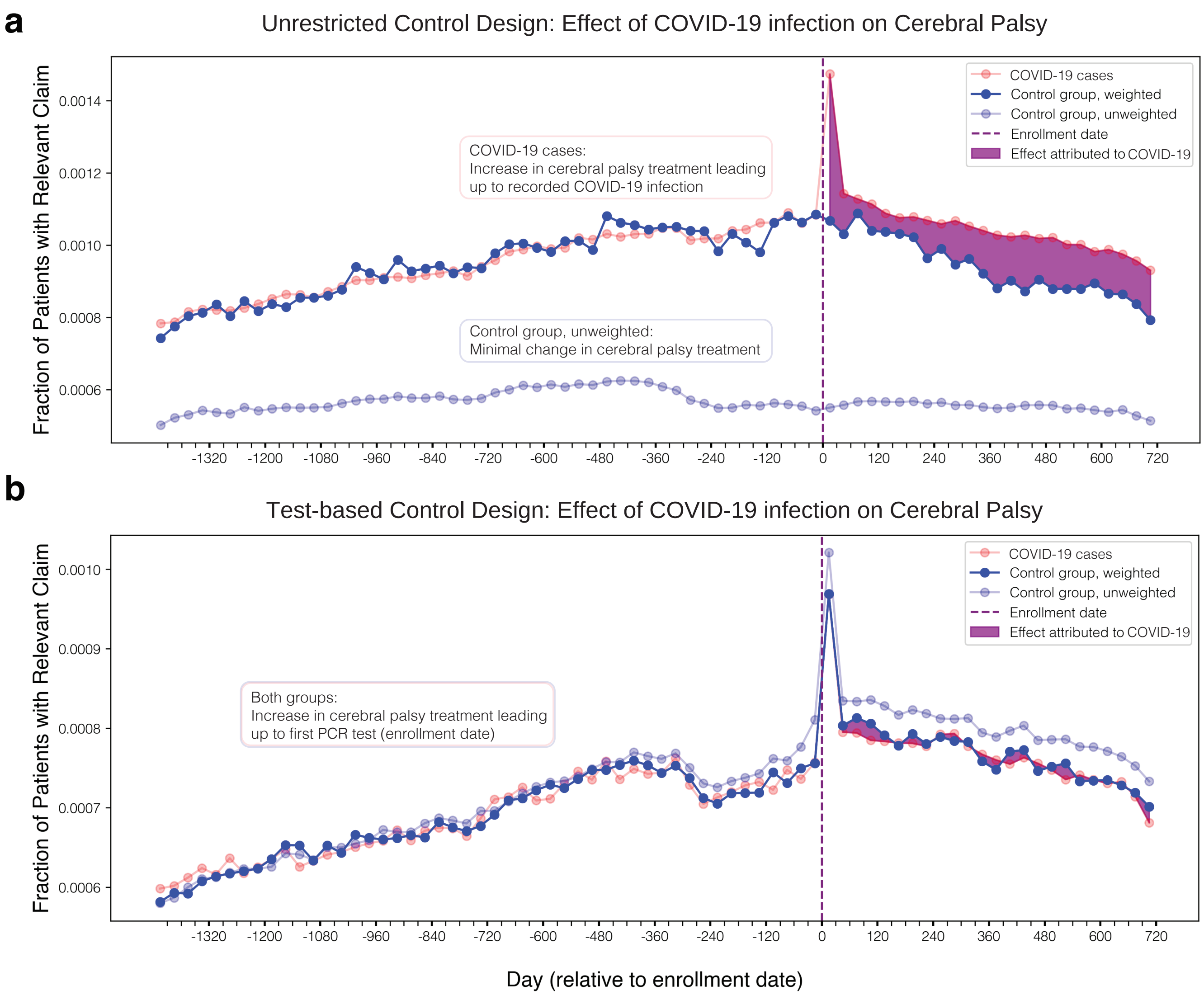


**Fig. S1. Comparison of an unrestricted control design and the test-based control design, examining cerebral palsy in adults, an outcome that cannot plausibly be caused by COVID-19. (a)** Unrestricted control design. Spurious effects (purple) persist for up to two years post-COVID. The control group differs substantially from COVID-19 cases. On one hand, COVID-19 cases show rising cerebral palsy diagnoses prior to infection, likely reflecting individuals already seeking care for cerebral palsy who incidentally tested positive for COVID-19. On the other hand, cerebral palsy diagnoses remain flat among unweighted controls—revealing a mismatch between groups. **(b)** Proposed design. No spurious effects observed. Both COVID-19 cases and test-negative controls (unweighted) exhibit similar pre-test increases in cerebral palsy diagnoses, suggesting comparable care-seeking behavior and populations with similar baseline health status.

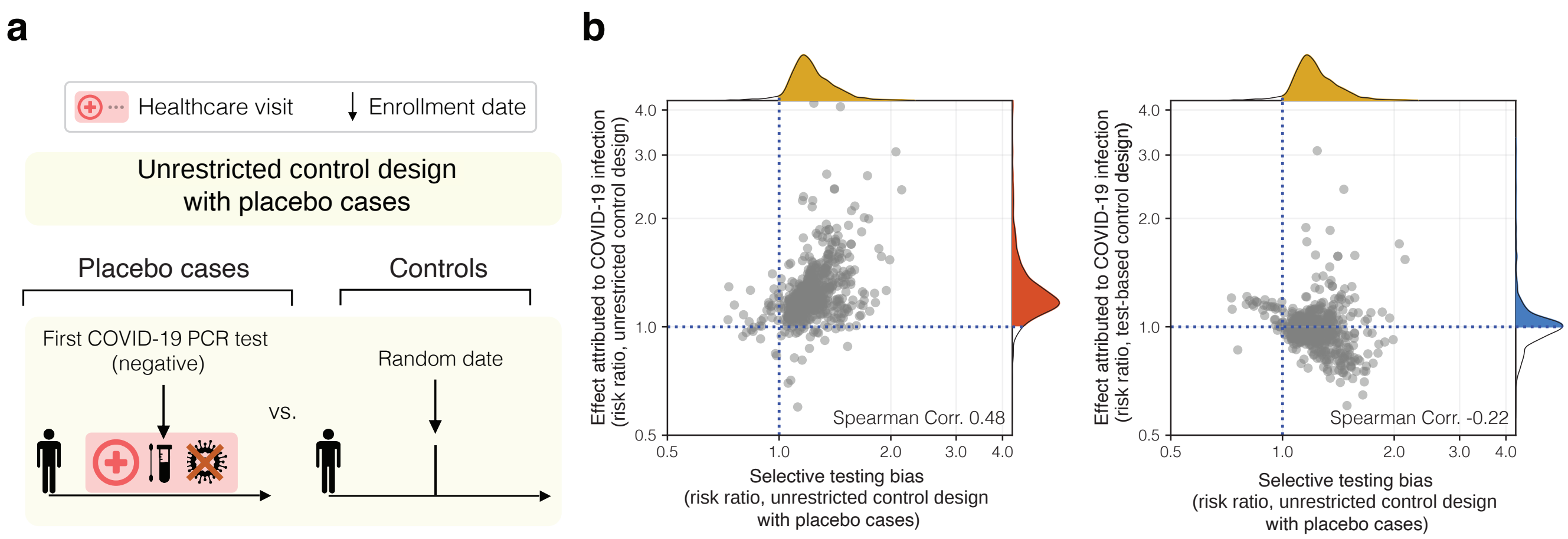


**Fig. S2. Selective testing bias explains results from unrestricted control analyses (a)** We measure bias introduced by selective testing of patients with non-COVID health problems. To do this, we replicate an unrestricted control design but replace COVID-19 cases with individuals who tested negative for COVID-19. These "placebo cases" had a healthcare visit and COVID-19 PCR test but were never diagnosed with COVID-19. We compare placebo cases to controls—individuals with similar histories, no COVID-19 diagnosis, and typically no visit or testing at enrollment. Because a negative test result cannot plausibly cause future health effects, any later differences between placebo cases and controls must be due to non-COVID factors among tested individuals. **(b)** The test-based control design appears less susceptible to selective testing bias compared to an unrestricted control analysis. Each of the $N = 614$ points represents one outcome (e.g., headache, nausea, etc.) on days 30-120 post-infection. *Left:* Selective COVID-19 testing bias explains the effects attributed to COVID-19 by an unrestricted control analysis (Spearman Corr 0.48). *Right:* In the test-based control design comparing COVID-19 cases to test-negative controls, this correlation is significantly weaker (Spearman Corr -0.22), indicating only mild bias(30) (Discussion). Our findings show that accurate attribution of long-term health outcomes to COVID-19 requires accounting for testing behavior, not just medical history.

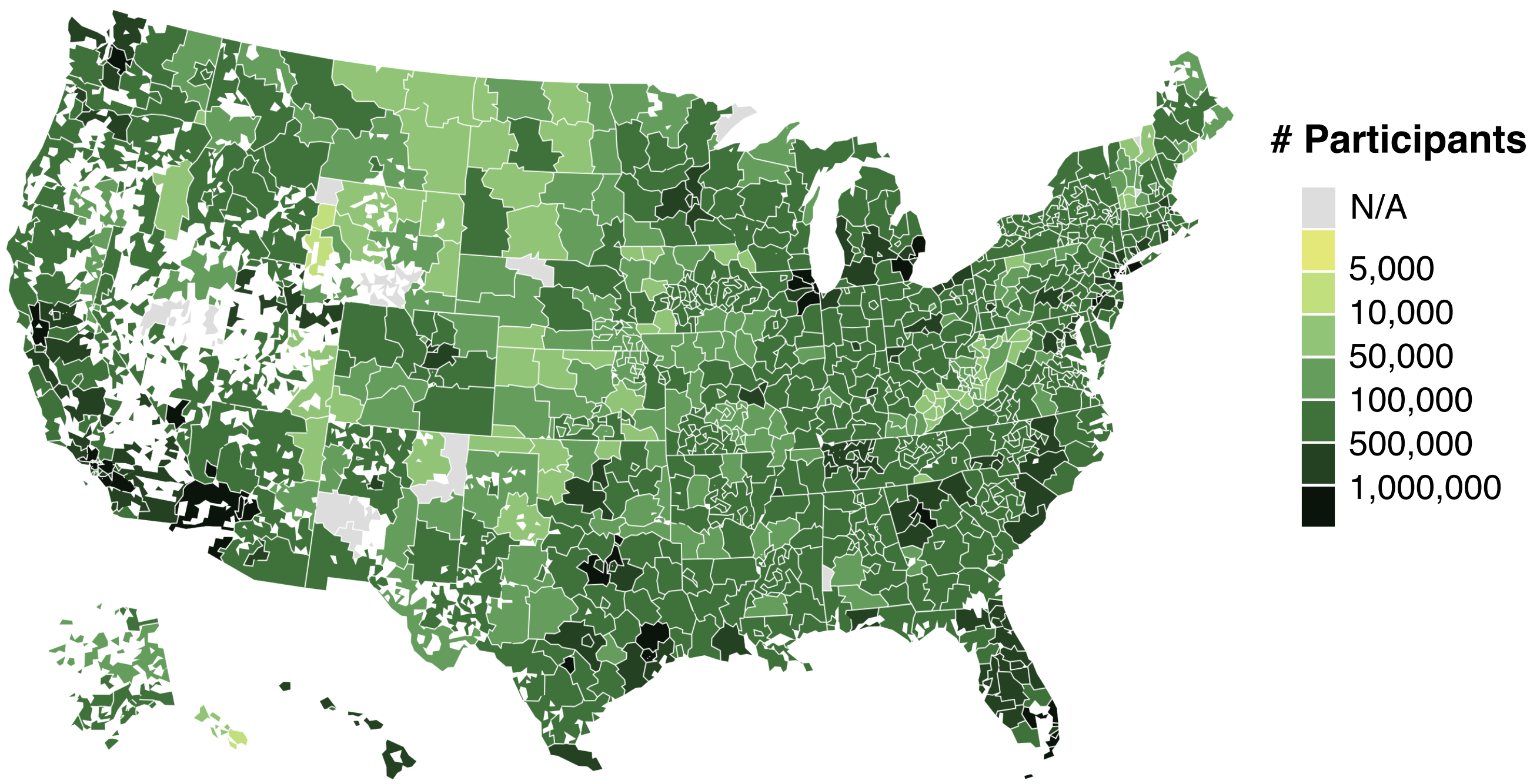


**Fig. S3. Number of participants across U.S. zipcodes.** This nationwide study leverages a healthcare map (Komodo) which aggregates 14.4 billion insurance claims across 244.7 million patients. Zipcodes are truncated to three digits to ensure patient anonymity.

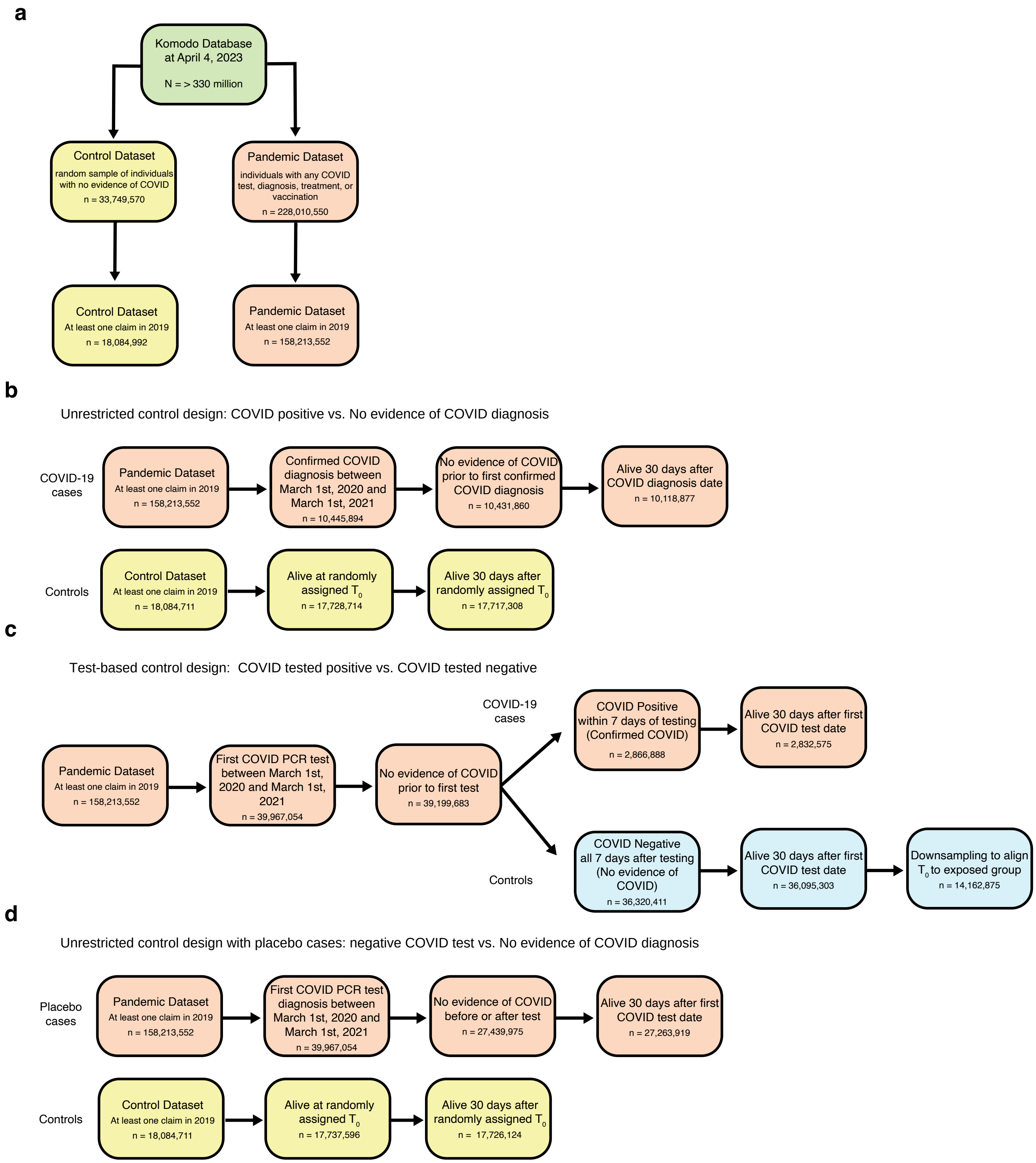


**Fig. S4. Large-scale health claims dataset enables varied observational studies designs to measure the long-term health effects of COVID (a)** Overall dataset. **(b)** The construction of the cohort for an unrestricted control design **(c)**. The construction of the cohort for the test-based control design. **(d)** The construction of the cohort for an unrestricted control design with placebo cases: an event that cannot plausibly have adverse health effects (negative COVID-19 PCR test).

**Table S1. Unrestricted control design; summary statistics of patient demographics and healthcare system interactions, stratified by total, COVID-19 cases, and control groups.**

| | Total | COVID-19 cases | Controls unweighted | Controls weighted‡ | SMD§ | Reference† |
|---|---|---|---|---|---|---|
| | (n=27,833,849) | (n=10,116,899) | (n=17,716,950) | (n=17,716,950) | | (n=326,569,308) |
| **Demographics** | | | | | | |
| Male | 12,280,227 (44%) | 4,271,857 (42%) | 8,008,370 (45%) | 7,468,685 (42%) | 0.00 | 160,818,530 (49%) |
| Age, years (mean [SD]) | 43 (23.6) | 46 (21.7) | 42 (24.5) | 45 (23.9) | 0.05 | |
| Age, years | | | | | | |
| Under 18 | 5,120,207 (18%) | 1,068,146 (11%) | 4,052,061 (23%) | 3,071,318 (17%) | -0.19 | 73,296,738 (22%) |
| 18-64 | 16,552,829 (59%) | 6,788,089 (67%) | 9,764,740 (55%) | 10,242,120 (58%) | 0.19 | 200,909,753 (62%) |
| 65+ | 6,160,813 (22%) | 2,260,664 (22%) | 3,900,149 (22%) | 4,403,512 (25%) | -0.06 | 52,362,817 (16%) |
| Non-White or Hispanic | 43% | 44% | 42% | 43% | -0.04 | 43% |
| Less than Bachelor Degree | 67% | 68% | 67% | 67% | 0.09 | 67% |
| Mean Income* | 91,634 | 91,091 | 91,934 | 90,744 | 0.01 | 90,981 |
| **Visit history** (mean / patient) | | | | | | |
| Emergency Room | 1.08 | 1.56 | 0.8 | 1.56 | 0.00 | |
| Inpatient | 0.83 | 1.24 | 0.6 | 1.24 | 0.00 | |
| Outpatient | 27.59 | 35.7 | 22.96 | 35.7 | 0.00 | |
| Total | 39.71 | 54.0 | 31.56 | 54.0 | 0.00 | |

‡ We weight controls to have covariate distributions matching COVID-19 cases, including pre-enrollment medical history across all 614 outcomes, healthcare utilization summary measures, and static demographics. See Methods M2: Covariates for details.
§ Standardized mean difference computed between COVID-19 cases and control weighted groups.
† Calculated on 2020 census US population.
* Mean Income values are adjusted to 2020 inflation dollars.

**Table S2. Test-based control design; summary statistics of patient demographics and healthcare system interactions, stratified by total, COVID-19 cases, and control groups.**

| | Total (n=16,994,746) | COVID-19 cases (n=2,832,376) | Controls unweighted (n=14,162,370) | Controls weighted‡ (n=14,162,370) | SMD§ | Reference† (n=326,569,308) |
|---|---|---|---|---|---|---|
| **Demographics** | | | | | | |
| Male | 7,063,814 (42%) | 1,219,533 (43%) | 5,844,281 (41%) | 6,065,745 (43%) | 0.00 | 160,818,530 (49%) |
| Age, years (mean [SD]) | 41 (21.4) | 42 (21.0) | 41 (21.5) | 42 (21.5) | 0.02 | |
| Age, years | | | | | | |
| Under 18 | 2,740,157 (16%) | 411,173 (15%) | 2,328,984 (16%) | 2,208,927 (16%) | -0.03 | 73,296,738 (22%) |
| 18-64 | 11,666,190 (69%) | 1,991,693 (70%) | 9,674,497 (68%) | 9,704,918 (69%) | 0.04 | 200,909,753 (62%) |
| 65+ | 2,588,399 (15%) | 429,510 (15%) | 2,158,889 (15%) | 2,248,525 (16%) | -0.02 | 52,362,817 (16%) |
| Non-White or Hispanic | 43% | 44% | 42% | 45% | 0.04 | 43% |
| Less than Bachelor Degree | 67% | 68% | 67% | 65% | 0.27 | 67% |
| Mean Income* | 93,225 | 90,539 | 93,761 | 88,819 | 0.07 | 90,981 |
| **Visit history** (mean / patient) | | | | | | |
| Emergency Room | 1.23 | 1.25 | 1.23 | 1.25 | 0.00 | |
| Inpatient | 0.88 | 0.86 | 0.89 | 0.86 | 0.00 | |
| Outpatient | 34.62 | 32.86 | 34.98 | 32.88 | 0.00 | |
| Total | 47.69 | 46.53 | 47.92 | 46.55 | 0.00 | |

‡ We weight controls to have covariate distributions matching COVID-19 cases, including pre-enrollment medical history across all 614 outcomes, healthcare utilization summary measures, and static demographics. See Methods M2: Covariates for details.
§ Standardized mean difference computed between COVID-19 cases and control weighted groups.
† Calculated on 2020 census US population.
* Mean Income values are adjusted to 2020 inflation dollars.

**Table S3. Unrestricted control design with placebo cases (individuals that tested negative for COVID-19); summary statistics of patient demographics and healthcare system interactions, stratified by total, placebo cases, and control groups.**

| | Total (n=44,965,453) | Placebo cases (n=27,263,919) | Controls unweighted (n=17,701,534) | Controls weighted‡ (n=17,701,534) | SMD§ | Reference† (n=326,569,308) |
|---|---|---|---|---|---|---|
| **Demographics** | | | | | | |
| Male | 19,517,875 (43%) | 11,517,280 (42%) | 8,000,595 (45%) | 7,498,223 (42%) | -0.00 | 160,818,530 (49%) |
| Age, years (mean [SD]) | 41 (22.9) | 41 (21.8) | 41 (24.4) | 41 (23.7) | -0.01 | |
| Age, years | | | | | | |
| Under 18 | 8,708,798 (19%) | 4,669,746 (17%) | 4,039,052 (23%) | 3,923,967 (22%) | -0.13 | 73,296,738 (22%) |
| 18-64 | 27,926,674 (62%) | 18,195,826 (67%) | 9,730,848 (55%) | 10,289,702 (58%) | 0.18 | 200,909,753 (62%) |
| 65+ | 8,186,881 (18%) | 4,311,375 (16%) | 3,875,506 (22%) | 3,430,882 (19%) | -0.09 | 52,362,817 (16%) |
| Non-White or Hispanic | 42% | 42% | 42% | 42% | 0.00 | 43% |
| Less than Bachelor Degree | 67% | 66% | 67% | 66% | 0.00 | 67% |
| Mean Income* | 93,162 | 93,976 | 91,939 | 92,333 | 0.00 | 90,981 |
| **Visit history** (mean / patient) | | | | | | |
| Emergency Room | 0.97 | 1.09 | 0.79 | 1.09 | 0.00 | |
| Inpatient | 0.74 | 0.83 | 0.6 | 0.83 | 0.00 | |
| Outpatient | 29.13 | 33.14 | 22.95 | 33.14 | 0.00 | |
| Total | 39.74 | 45.07 | 31.53 | 45.08 | 0.00 | |

‡ We weight controls to have covariate distributions matching placebo cases, including pre-enrollment medical history across all 614 outcomes, healthcare utilization summary measures, and static demographics. See Methods M2: Covariates for details.
§ Standardized mean difference computed between placebo cases and weighted control groups.
† Calculated on 2020 census US population.
* Mean Income values are adjusted to 2020 inflation dollars.

**Table S4. Unrestricted control design; summary statistics of pre-enrollment diagnosis incidence across clinical categories, comparing COVID-19 cases and unweighted controls. Values report the mean number of recorded diagnoses per individual across the outcomes within each category.**

| Category | Total | COVID-19 cases | Controls unweighted | SMD§ | Number of outcomes† |
|---|---|---|---|---|---|
| Endocrine, Nutritional and Metabolic Diseases | 0.501 | 0.763 | 0.351 | 0.441 | 18 |
| Cognitive health | 0.041 | 0.085 | 0.015 | 0.212 | 9 |
| Diseases of the Nervous System | 0.139 | 0.215 | 0.096 | 0.228 | 30 |
| Diseases of the Circulatory System | 0.124 | 0.190 | 0.086 | 0.205 | 62 |
| Substance use | 0.046 | 0.057 | 0.039 | 0.075 | 21 |
| Mental health | 0.238 | 0.324 | 0.189 | 0.189 | 20 |
| Factors Influencing Health Status and Contact with Health Services | 0.428 | 0.565 | 0.349 | 0.236 | 20 |
| Diseases of the Genitourinary System | 0.127 | 0.194 | 0.088 | 0.234 | 24 |
| Diseases of the Blood and Blood Forming Organs and Certain Disorders Involving the Immune Mechanism | 0.186 | 0.273 | 0.136 | 0.252 | 10 |
| Muscles and joints | 0.147 | 0.207 | 0.113 | 0.170 | 63 |
| Neoplasms | 0.025 | 0.031 | 0.021 | 0.038 | 70 |
| Certain Infectious and Parasitic Diseases | 0.152 | 0.220 | 0.113 | 0.223 | 8 |
| Stomach and digestion | 0.156 | 0.232 | 0.113 | 0.223 | 34 |
| Ear, nose and throat | 0.114 | 0.149 | 0.094 | 0.134 | 24 |
| Misc | 0.134 | 0.199 | 0.097 | 0.179 | 58 |
| Hair, skin and nails | 0.144 | 0.196 | 0.115 | 0.183 | 16 |
| Breathing | 0.196 | 0.283 | 0.147 | 0.250 | 24 |
| Movement | 0.031 | 0.051 | 0.019 | 0.137 | 7 |
| Diseases of the Eye and Adnexa | 0.155 | 0.197 | 0.131 | 0.142 | 12 |
| Reproductive health | 0.042 | 0.057 | 0.033 | 0.088 | 39 |
| Pain | 0.141 | 0.216 | 0.099 | 0.258 | 6 |
| Sleep | 0.101 | 0.151 | 0.072 | 0.195 | 3 |
| Eyes | 0.044 | 0.057 | 0.037 | 0.077 | 9 |
| Fatigue | 0.181 | 0.268 | 0.132 | 0.221 | 2 |

§ Standardized mean difference computed between COVID-19 cases and unweighted controls using mean diagnosis incidence within each clinical category.
† Number of individual outcomes included in each clinical category.

**Table S5. Unrestricted control design; summary statistics of pre-enrollment diagnosis prevalence across clinical categories, comparing COVID-19 cases and unweighted controls. Values report the percentage of individuals with at least one recorded diagnosis among the outcomes within each category.**

| Category | Total | COVID-19 cases | Controls unweighted | SMD[§] | Number of outcomes[†] |
|---|---|---|---|---|---|
| Endocrine, Nutritional and Metabolic Diseases | 9.70% | 13.26% | 7.67% | 0.168 | 18 |
| Cognitive health | 0.68% | 1.22% | 0.37% | 0.078 | 9 |
| Diseases of the Nervous System | 3.70% | 5.18% | 2.86% | 0.096 | 30 |
| Diseases of the Circulatory System | 3.03% | 4.26% | 2.33% | 0.096 | 62 |
| Substance use | 1.53% | 1.88% | 1.33% | 0.042 | 21 |
| Mental health | 4.39% | 5.60% | 3.70% | 0.072 | 20 |
| Factors Influencing Health Status and Contact with Health Services | 13.60% | 16.38% | 12.00% | 0.115 | 20 |
| Diseases of the Genitourinary System | 4.59% | 6.39% | 3.56% | 0.119 | 24 |
| Diseases of the Blood and Blood Forming Organs and Certain Disorders Involving the Immune Mechanism | 6.34% | 8.43% | 5.15% | 0.121 | 10 |
| Muscles and joints | 4.59% | 6.06% | 3.75% | 0.090 | 63 |
| Neoplasms | 0.95% | 1.14% | 0.84% | 0.018 | 70 |
| Certain Infectious and Parasitic Diseases | 7.41% | 9.82% | 6.04% | 0.127 | 8 |
| Stomach and digestion | 6.40% | 8.56% | 5.17% | 0.124 | 34 |
| Ear, nose and throat | 6.34% | 7.95% | 5.42% | 0.093 | 24 |
| Misc | 5.62% | 7.53% | 4.54% | 0.104 | 58 |
| Hair, skin and nails | 6.90% | 8.65% | 5.90% | 0.105 | 16 |
| Breathing | 7.93% | 10.67% | 6.37% | 0.142 | 24 |
| Movement | 1.12% | 1.65% | 0.81% | 0.065 | 7 |
| Diseases of the Eye and Adnexa | 6.76% | 8.25% | 5.91% | 0.084 | 12 |
| Reproductive health | 1.85% | 2.42% | 1.52% | 0.055 | 39 |
| Pain | 7.13% | 10.27% | 5.34% | 0.174 | 6 |
| Sleep | 3.57% | 5.00% | 2.76% | 0.108 | 3 |
| Eyes | 2.61% | 3.28% | 2.24% | 0.056 | 9 |
| Fatigue | 8.87% | 12.44% | 6.83% | 0.149 | 2 |

[§] Standardized mean difference computed between COVID-19 cases and unweighted controls using patient prevalence within each clinical category.
[†] Number of individual outcomes included in each clinical category.

**Table S6. Test-based control design; summary statistics of pre-enrollment diagnosis incidence across clinical categories, comparing test-positive COVID-19 cases and unweighted test-negative controls. Values report the mean number of recorded diagnoses per individual across the outcomes within each category.**

| Category | Total | COVID-19 cases | Controls unweighted | SMD[§] | Number of outcomes[†] |
|---|---|---|---|---|---|
| Endocrine, Nutritional and Metabolic Diseases | 0.520 | 0.586 | 0.507 | 0.073 | 18 |
| Cognitive health | 0.025 | 0.038 | 0.022 | 0.055 | 9 |
| Diseases of the Nervous System | 0.153 | 0.154 | 0.153 | 0.008 | 30 |
| Diseases of the Circulatory System | 0.121 | 0.129 | 0.120 | 0.011 | 62 |
| Substance use | 0.060 | 0.047 | 0.062 | -0.045 | 21 |
| Mental health | 0.302 | 0.266 | 0.310 | -0.060 | 20 |
| Factors Influencing Health Status and Contact with Health Services | 0.498 | 0.491 | 0.500 | -0.009 | 20 |
| Diseases of the Genitourinary System | 0.138 | 0.145 | 0.137 | 0.012 | 24 |
| Diseases of the Blood and Blood Forming Organs and Certain Disorders Involving the Immune Mechanism | 0.209 | 0.214 | 0.209 | 0.002 | 10 |
| Muscles and joints | 0.176 | 0.173 | 0.176 | -0.007 | 63 |
| Neoplasms | 0.030 | 0.026 | 0.031 | -0.022 | 70 |
| Certain Infectious and Parasitic Diseases | 0.173 | 0.178 | 0.172 | 0.013 | 8 |
| Stomach and digestion | 0.186 | 0.183 | 0.187 | -0.015 | 34 |
| Ear, nose and throat | 0.137 | 0.134 | 0.138 | -0.009 | 24 |
| Misc | 0.152 | 0.153 | 0.151 | 0.002 | 58 |
| Hair, skin and nails | 0.171 | 0.168 | 0.171 | -0.002 | 16 |
| Breathing | 0.228 | 0.225 | 0.228 | -0.006 | 24 |
| Movement | 0.029 | 0.030 | 0.029 | 0.003 | 7 |
| Diseases of the Eye and Adnexa | 0.165 | 0.167 | 0.165 | 0.001 | 12 |
| Reproductive health | 0.058 | 0.060 | 0.058 | 0.006 | 39 |
| Pain | 0.172 | 0.173 | 0.172 | 0.001 | 6 |
| Sleep | 0.118 | 0.114 | 0.119 | -0.011 | 3 |
| Eyes | 0.051 | 0.050 | 0.052 | -0.006 | 9 |
| Fatigue | 0.224 | 0.227 | 0.223 | 0.006 | 2 |

[§] Standardized mean difference computed between test-positive COVID-19 cases and unweighted test-negative controls using mean diagnosis incidence within each clinical category.
[†] Number of individual outcomes included in each clinical category.

**Table S7. Test-based control design; summary statistics of pre-enrollment diagnosis prevalence across clinical categories, comparing test-positive COVID-19 cases and unweighted test-negative controls. Values report the percentage of individuals with at least one recorded diagnosis among the outcomes within each category.**

| Category | Total | COVID-19 cases | Controls unweighted | SMD[§] | Number of outcomes[†] |
|---|---|---|---|---|---|
| Endocrine, Nutritional and Metabolic Diseases | 10.24% | 11.10% | 10.06% | 0.028 | 18 |
| Cognitive health | 0.49% | 0.61% | 0.47% | 0.014 | 9 |
| Diseases of the Nervous System | 4.20% | 4.17% | 4.21% | -0.001 | 30 |
| Diseases of the Circulatory System | 3.10% | 3.15% | 3.09% | 0.001 | 62 |
| Substance use | 1.91% | 1.62% | 1.96% | -0.021 | 21 |
| Mental health | 5.46% | 4.94% | 5.56% | -0.023 | 20 |
| Factors Influencing Health Status and Contact with Health Services | 15.55% | 15.36% | 15.59% | -0.008 | 20 |
| Diseases of the Genitourinary System | 5.37% | 5.42% | 5.36% | 0.002 | 24 |
| Diseases of the Blood and Blood Forming Organs and Certain Disorders Involving the Immune Mechanism | 7.20% | 7.20% | 7.20% | -0.004 | 10 |
| Muscles and joints | 5.39% | 5.35% | 5.39% | -0.004 | 63 |
| Neoplasms | 1.12% | 1.02% | 1.14% | -0.009 | 70 |
| Certain Infectious and Parasitic Diseases | 8.61% | 8.73% | 8.59% | 0.005 | 8 |
| Stomach and digestion | 7.55% | 7.41% | 7.58% | -0.008 | 34 |
| Ear, nose and throat | 7.55% | 7.44% | 7.57% | -0.005 | 24 |
| Misc | 6.47% | 6.51% | 6.47% | -0.000 | 58 |
| Hair, skin and nails | 8.23% | 8.02% | 8.27% | -0.006 | 16 |
| Breathing | 9.22% | 9.25% | 9.21% | -0.002 | 24 |
| Movement | 1.19% | 1.17% | 1.20% | -0.003 | 7 |
| Diseases of the Eye and Adnexa | 7.46% | 7.49% | 7.46% | -0.000 | 12 |
| Reproductive health | 2.52% | 2.57% | 2.51% | 0.003 | 39 |
| Pain | 8.57% | 8.77% | 8.53% | 0.006 | 6 |
| Sleep | 4.37% | 4.16% | 4.41% | -0.009 | 3 |
| Eyes | 3.10% | 3.06% | 3.11% | -0.004 | 9 |
| Fatigue | 10.81% | 10.92% | 10.79% | 0.004 | 2 |

[§] Standardized mean difference computed between test-positive COVID-19 cases and unweighted test-negative controls using patient prevalence within each clinical category.
[†] Number of individual outcomes included in each clinical category.

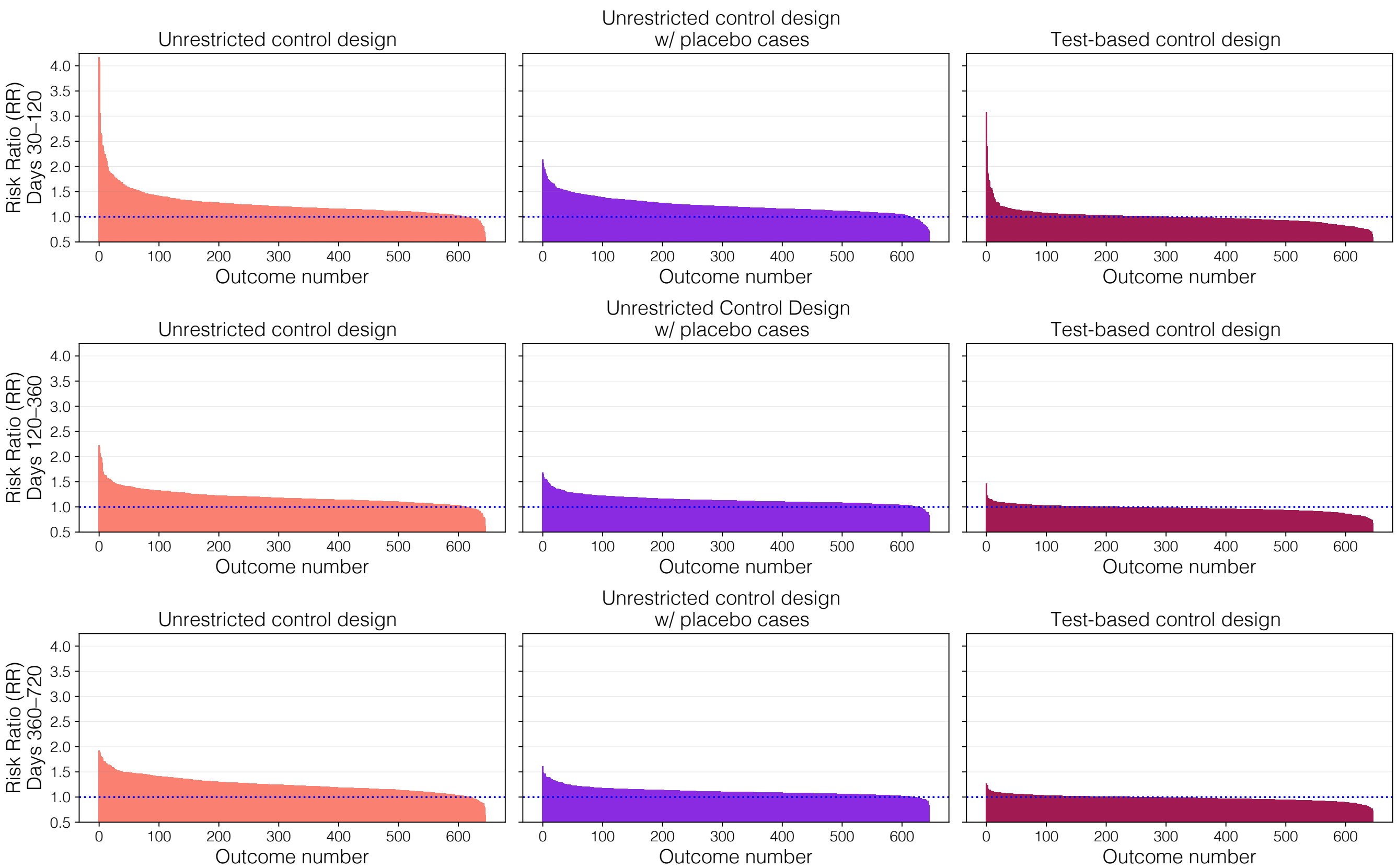


**Fig. S5. COVID symptoms and sequelae detected by an unrestricted control design vs. the test-based control design: all 614 outcomes**. Risk ratios (RRs; Extended Methods S3) for the effect of COVID-19 on days 30-120 (top row), days 120-360 (middle row), and days 360-720 (bottom row) after infection. Outcomes are sorted in descending order by RR. The unrestricted control design observes positive effects (RR $\geq$ 1.1) for the vast majority of outcomes: 82.7% on days 30-120, 77.5% on days 120-360, and 85.0% on days 360-720. However, an unrestricted control design with placebo cases, a similar proportion of outcomes have positive effects: 82.5% on days 30-120, 66.6% on days 120-360, and 54.8% on days 360-720. This suggests that many effects detected by the unrestricted control design may be spurious and an artifact of selective testing bias. By contrast, the test-based control design only detects positive effects for a minority of outcomes: 8.8% on days 30-120, 2.3% on days 120-360, and 1.6% on days 360-720, indicating that many previously reported effects may be overstated. These findings underscore the importance of adjusting for selective testing bias when assessing the long-term health consequences of COVID-19.

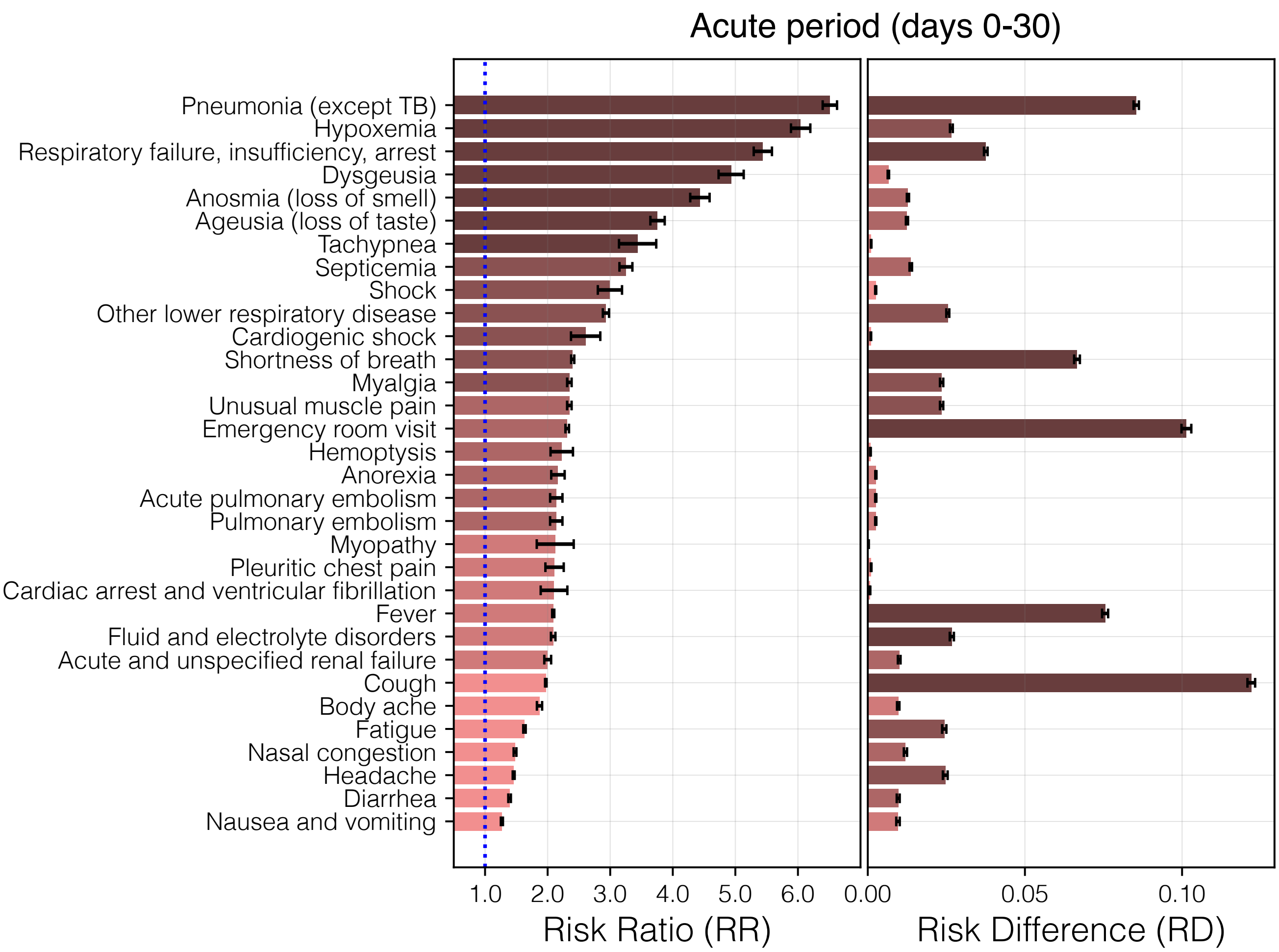


**Fig. S6. Acute COVID-19 symptoms and sequelae detected by the test-based control design**. *Left:* relative risk ratios (RRs; Extended Methods S3: Analysis details). *Right:* risk differences (RDs; Extended Methods S3: Analysis details). Common COVID-19 symptoms (37) and the top 25 most attributable (highest RR) adverse effects of COVID-19 are shown, during the acute period (days 0-30). Bars are colored by RR quintile; small effects ($RR < 1.1$) are not displayed. The test-based control design recovers known acute COVID-19 symptoms from the clinical literature, validating that the test-based control design can be used to analyze insurance claims in order to infer population-level disease effects.

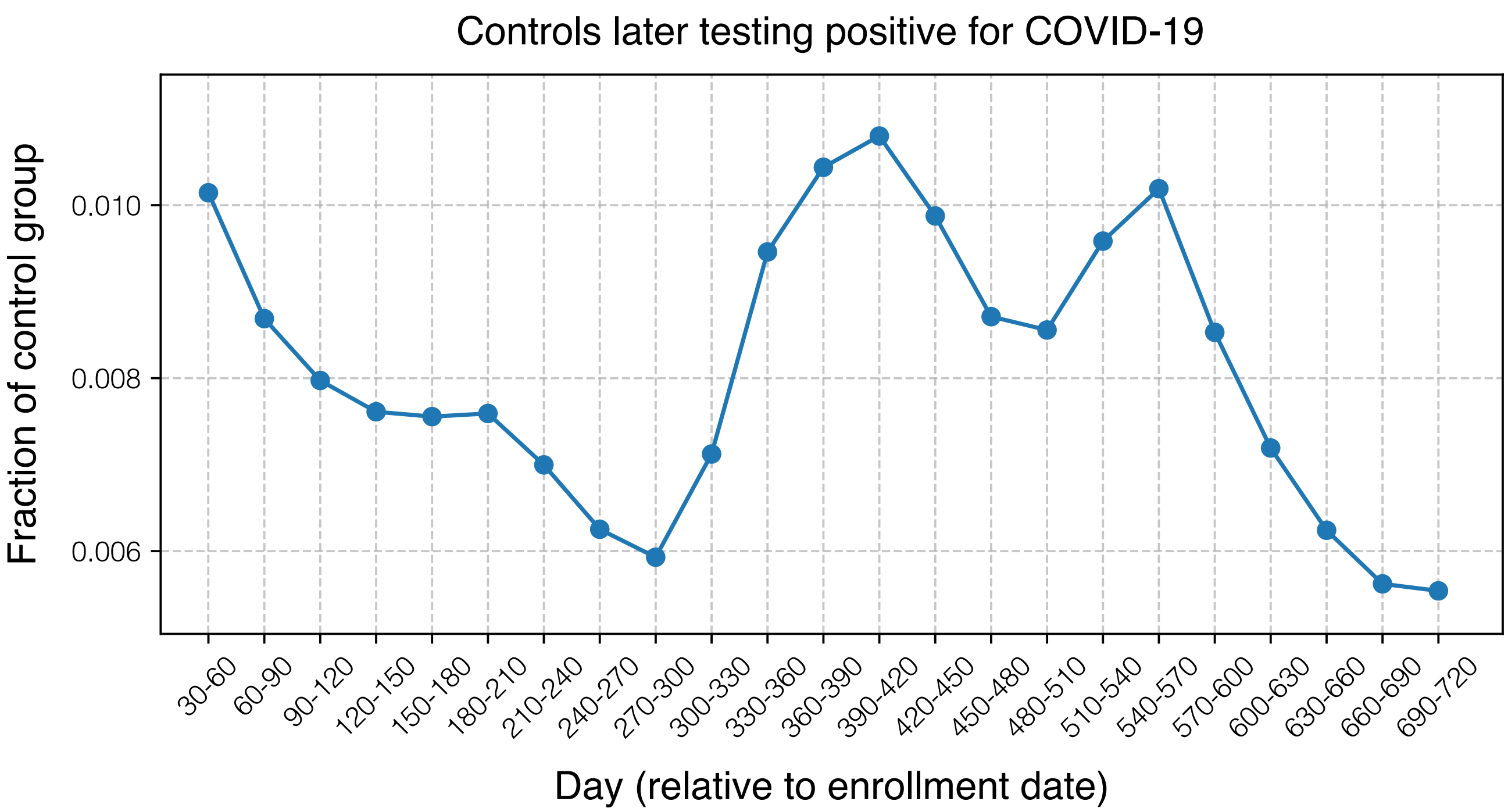


**Fig. S7. Proportion of controls in the test-based control design who later test positive for COVID-19.** In the test-based control design, individuals are enrolled into the control group at the time of their first negative PCR test. Some controls subsequently test positive for COVID-19. We retain these individuals to avoid selection bias that could arise from using future information to exclude participants, thereby preserving the integrity of a true prospective study design. While this approach prevents bias, it may dilute effect size estimates, making them more conservative. To quantify the potential impact, we plot the proportion of controls who later test positive over time. We observe that between 0.6%–1.1% of controls test positive in any given month. In total, 4.2% tested positive 120 days after enrollment (affecting outcome estimates on days 30-120), 10.1% tested positive 360 days after enrollment (affecting outcome estimates on days 120-360), and 20.2% have tested positive after 2 years of enrollment (affecting outcome estimates on days 360-720).

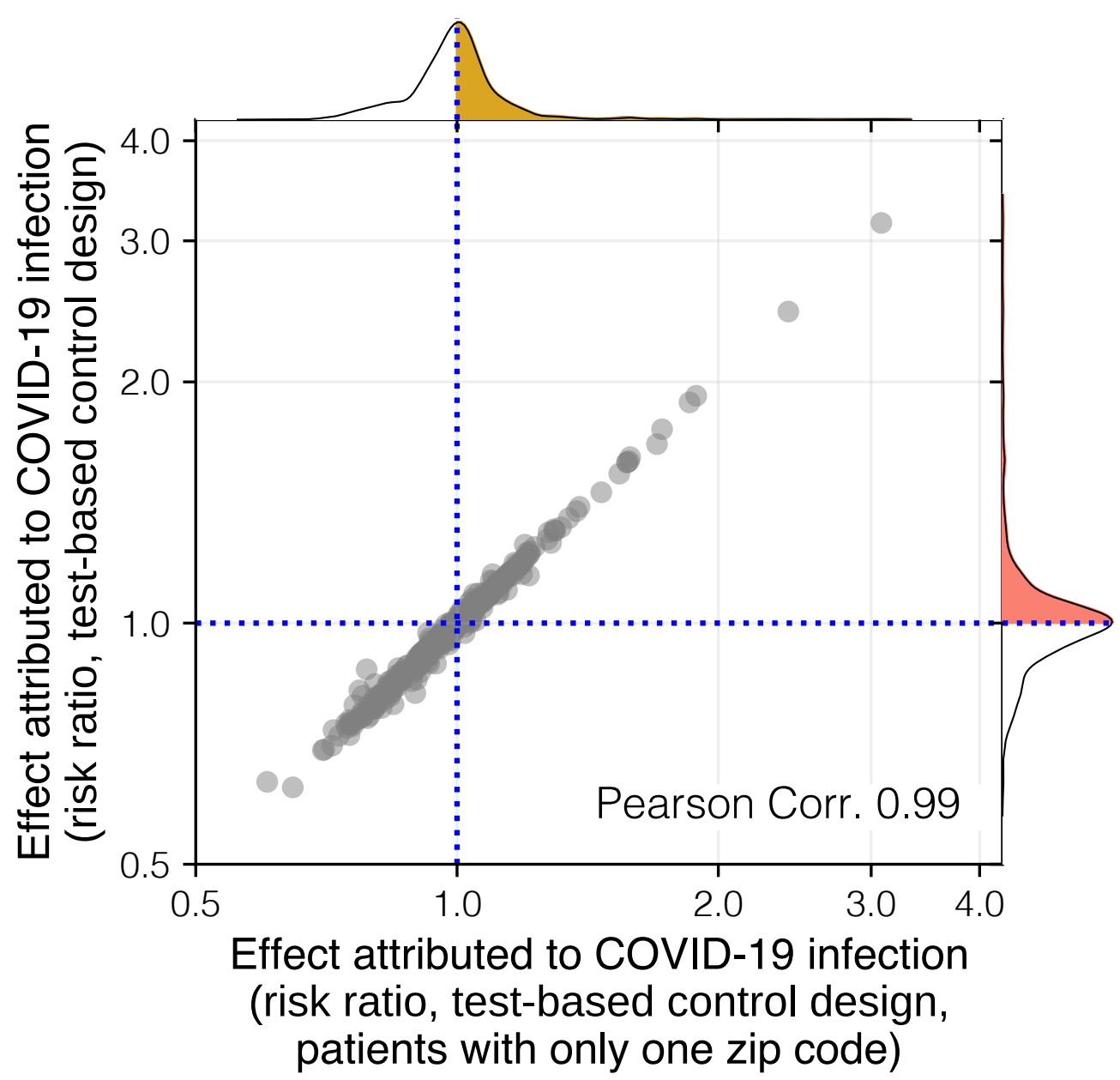


**Fig. S8. Robustness to patient identity collisions.** To assess whether our findings are affected by potential collisions between different patients sharing the same name, gender, and date of birth, we re-ran our analysis after excluding individuals with more than one zipcode (ZIP-3) in their medical history (a proxy for possible misidentification). The results remained nearly unchanged (Pearson correlation = 0.99), demonstrating that our findings are robust to patient identity resolution in the Komodo dataset.

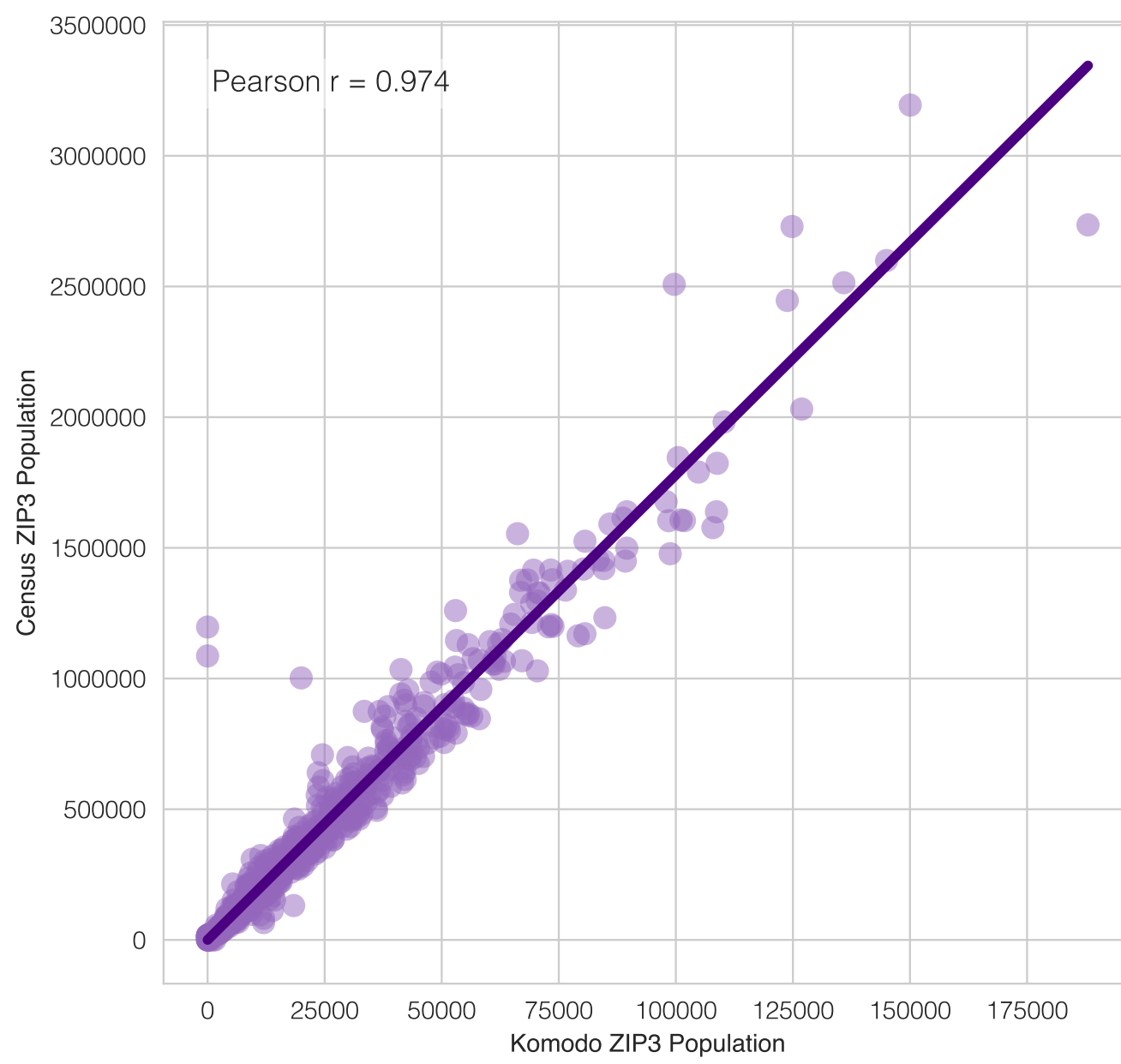


**Fig. S9. Komodo claims data is geographically well-balanced.** We compared how people are distributed across ZIP-3 regions in our dataset to how they are distributed in the U.S. Census. The two distributions are highly aligned (Pearson R = 0.97), demonstrating that the Komodo dataset closely reflects the geographic makeup of the U.S. population and is not heavily biased toward certain regions.

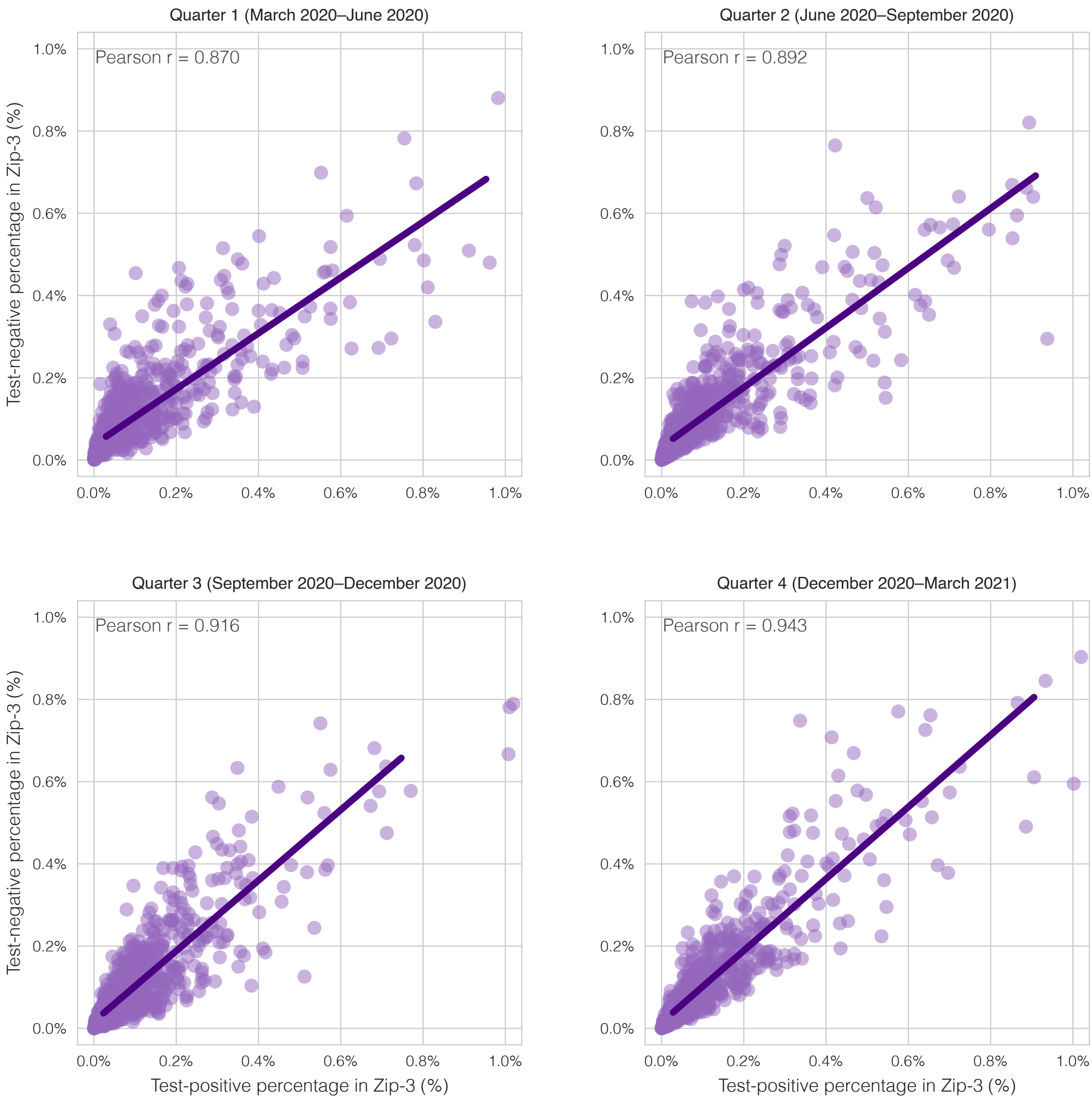


**Fig. S10. Quarterly geographic balance of test-positive and test-negative cohorts across ZIP-3 areas.** We compare the ZIP-3 concentration of the test-positive and test-negative cohorts in each of the four enrollment quarters (Q1: March–June 2020; Q2: June–September 2020; Q3: September–December 2020; Q4: December 2020–March 2021). Each point corresponds to one ZIP-3; axes show the percentage of the respective cohort residing in that ZIP-3. Geographic distributions of the two cohorts remain highly aligned across the enrollment period: Pearson correlations range from 0.87–0.94.

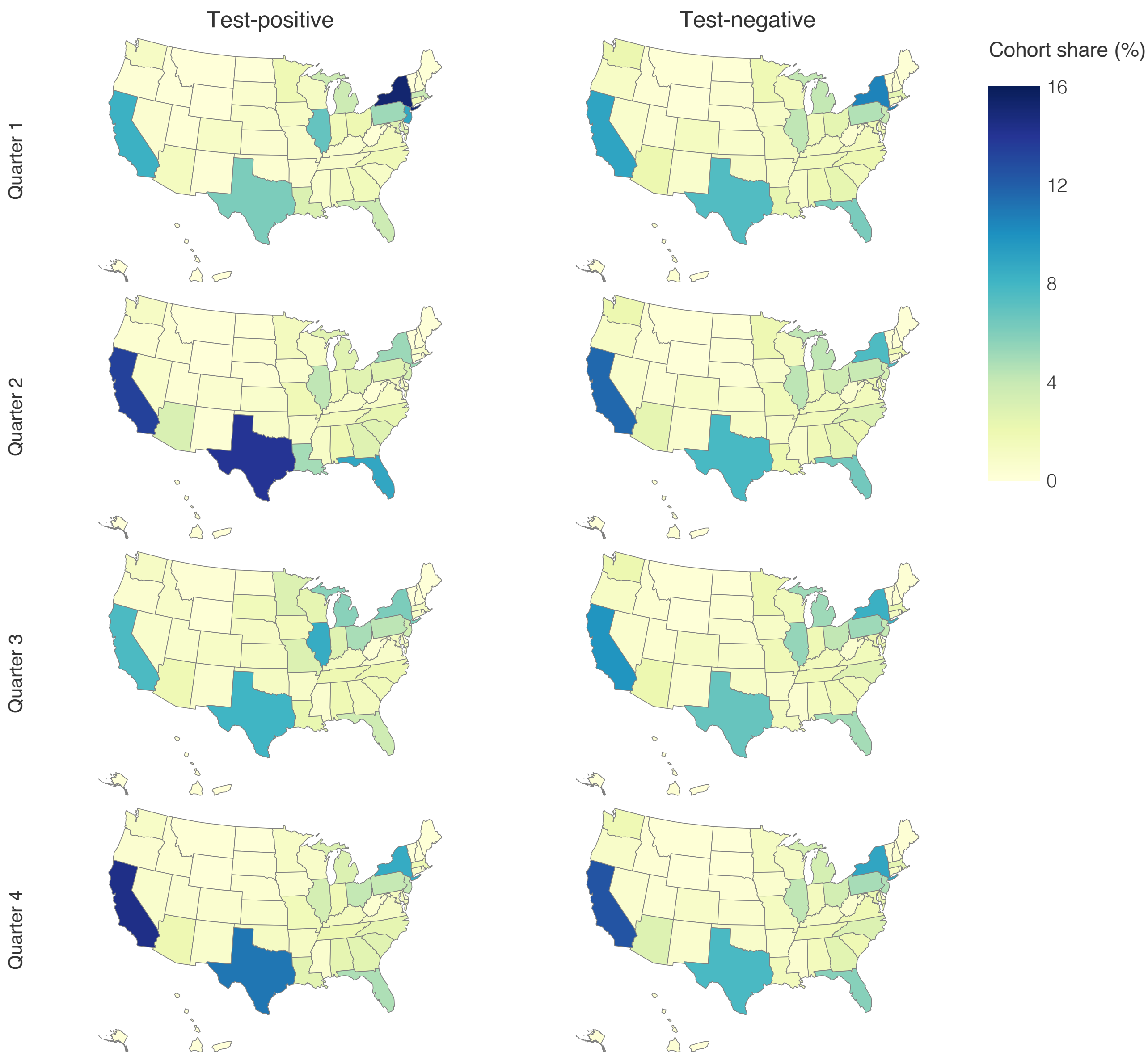


**Fig. S11. Quarterly state-level distribution of test-positive and test-negative cohorts.** State-level heatmaps of cohort share (color scale, 0–16%) for the test-positive (left) and test-negative (right) cohorts, one row per enrollment quarter (Q1–Q4 of the March 2020–March 2021 enrollment window). The spatial distributions of the two cohorts closely track each other in every quarter, corroborating the quantitative ZIP-3 balance shown in Fig. S10.

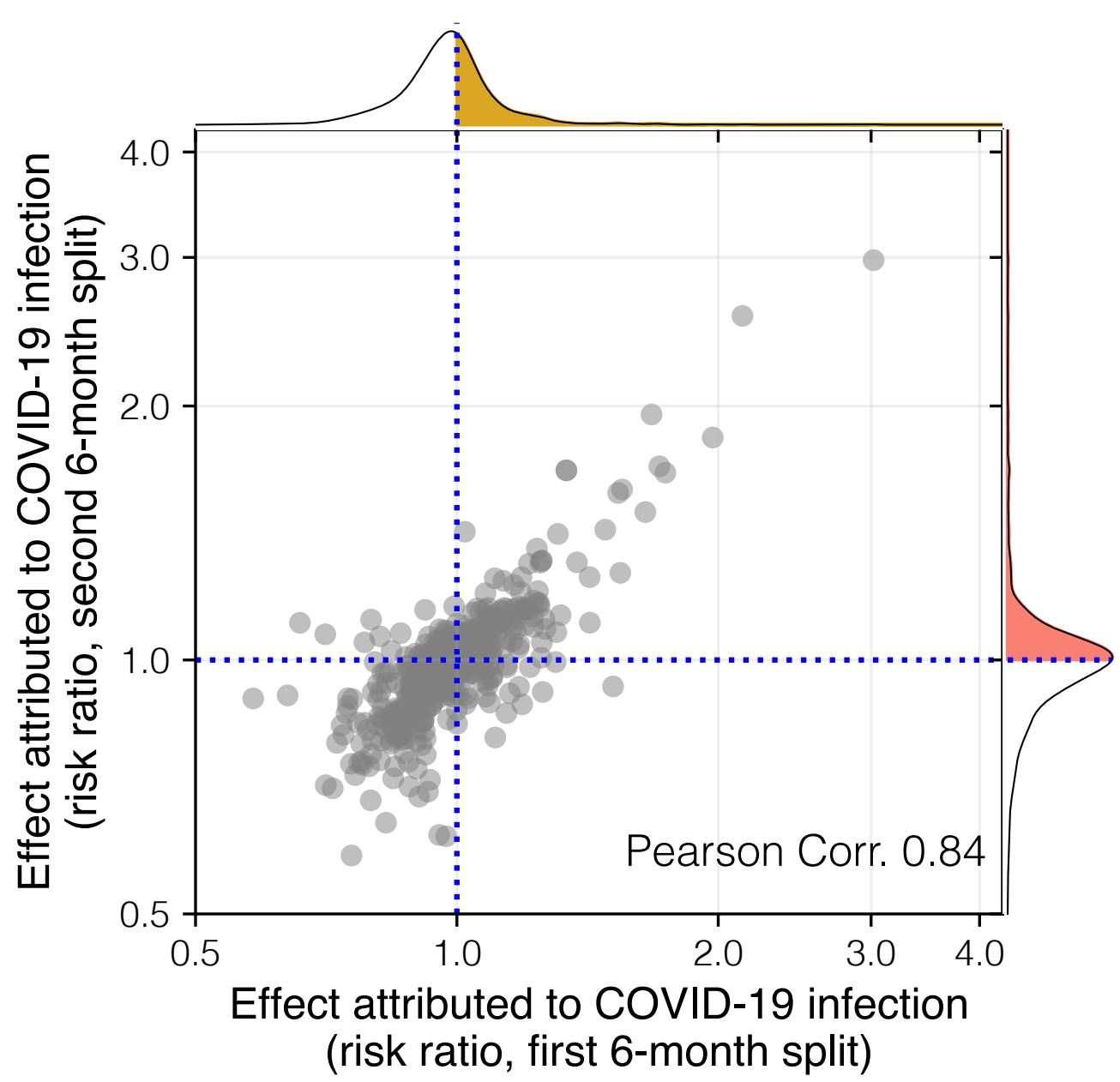


**Fig. S12. Robustness to splitting the enrollment period into first and second halves.** We split the March 2020–March 2021 enrollment window into two consecutive 6-month intervals. Notably, the incidence rate of COVID-19 was 285% higher (1.82% vs. 7.01%) in the second sixth months of the pandemic (38). We then re-estimate risk ratios for each interval separately using the test-based control design. Each point corresponds to one of 614 outcomes. Risk ratios are consistent between the two halves (Pearson correlation 0.84), indicating that the estimated effects attributed to COVID-19 are stable over the enrollment period and are not driven by particular sub-intervals.

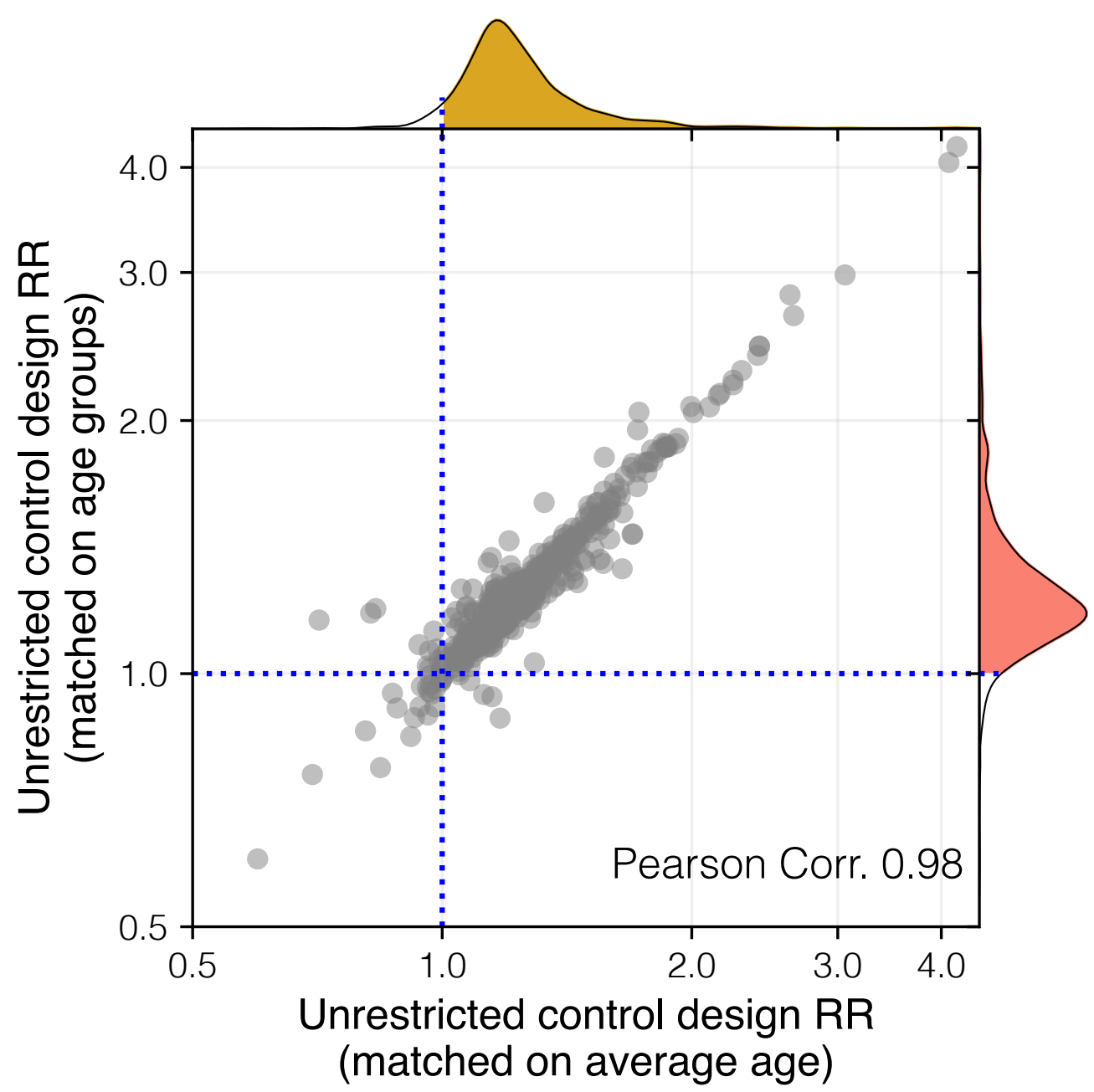


**Fig. S13. Unrestricted control analysis is robust to matching on age category (18 and under, 18-65, and 65+) rather than average age.** Each point corresponds to one of 614 outcomes. Risk ratios estimated after matching on age category are nearly identical to those estimated after matching on average age (Pearson correlation 0.98), demonstrating that the main-text results reported for the unrestricted control design are not sensitive to the choice of age-matching scheme.

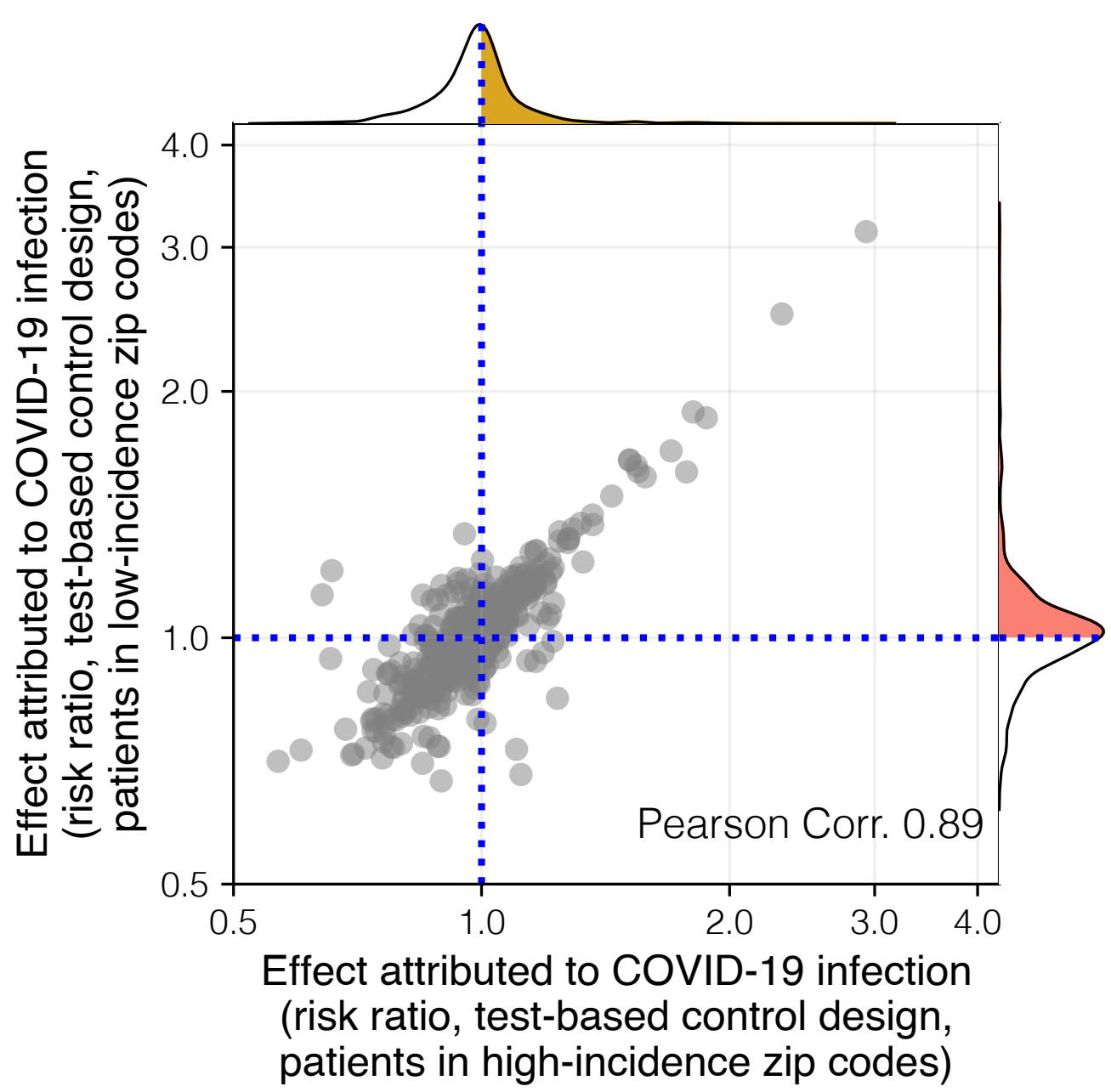


**Fig. S14. Unrestricted control analysis is robust to splitting by high vs. low incidence rate locations.** Each point corresponds to one of 614 outcomes. Risk ratios estimated in ZIP-3s with below-median incidence rates of COVID-19 (mean incidence rate 11.2% among patients receiving a test in our claims data) are nearly identical to those estimated in ZIP-3s with above-median incidence rates of COVID-19 (mean incidence rate 22.1% among patients receiving a test in our claims data), demonstrating that the main-text results reported for the unrestricted control design are not sensitive to local testing patterns.

**Table S8. Unrestricted control design with age-group matching; summary statistics of patient demographics and healthcare system interactions, stratified by total, COVID-19 cases, and control groups.**

| | Total (n=27,833,849) | COVID-19 cases (n=10,116,899) | Controls unweighted (n=17,716,950) | Controls weighted‡ (n=17,716,950) | SMD§ | Reference† (n=326,569,308) |
|---|---|---|---|---|---|---|
| **Demographics** | | | | | | |
| Male | 12,280,227 (44%) | 4,271,857 (42%) | 8,008,370 (45%) | 7,469,482 (42%) | 0.00 | 160,818,530 (49%) |
| Age, years (mean [SD]) | 43 (23.6) | 46 (21.7) | 42 (24.5) | 46 (22.1) | 0.01 | |
| Age, years | | | | | | |
| Under 18 | 5,120,207 (18%) | 1,068,146 (11%) | 4,052,061 (23%) | 1,870,592 (11%) | 0.00 | 73,296,738 (22%) |
| 18-64 | 16,552,829 (59%) | 6,788,089 (67%) | 9,764,740 (55%) | 11,887,433 (67%) | 0.00 | 200,909,753 (62%) |
| 65+ | 6,160,813 (22%) | 2,260,664 (22%) | 3,900,149 (22%) | 3,958,925 (22%) | 0.00 | 52,362,817 (16%) |
| Non-White or Hispanic | 43% | 44% | 42% | 43% | -0.04 | 43% |
| Less than Bachelor Degree | 67% | 68% | 67% | 67% | 0.09 | 67% |
| Mean Income* | 91,634 | 91,091 | 91,934 | 90,802 | 0.01 | 90,981 |
| **Visit history** (mean / patient) | | | | | | |
| Emergency Room | 1.08 | 1.56 | 0.8 | 1.56 | 0.00 | |
| Inpatient | 0.83 | 1.24 | 0.6 | 1.24 | 0.00 | |
| Outpatient | 27.59 | 35.7 | 22.96 | 35.7 | 0.00 | |
| Total | 39.71 | 54.0 | 31.56 | 54.0 | 0.00 | |

‡ Controls are re-weighted to match COVID-19 cases on age group (rather than average age) in addition to all other pre-enrollment medical history covariates, healthcare utilization summary measures, and static demographics. See Methods M2: Covariates for details.
§ Standardized mean difference computed between COVID-19 cases and control weighted groups.
† Calculated on 2020 census US population.
* Mean Income values are adjusted to 2020 inflation dollars.

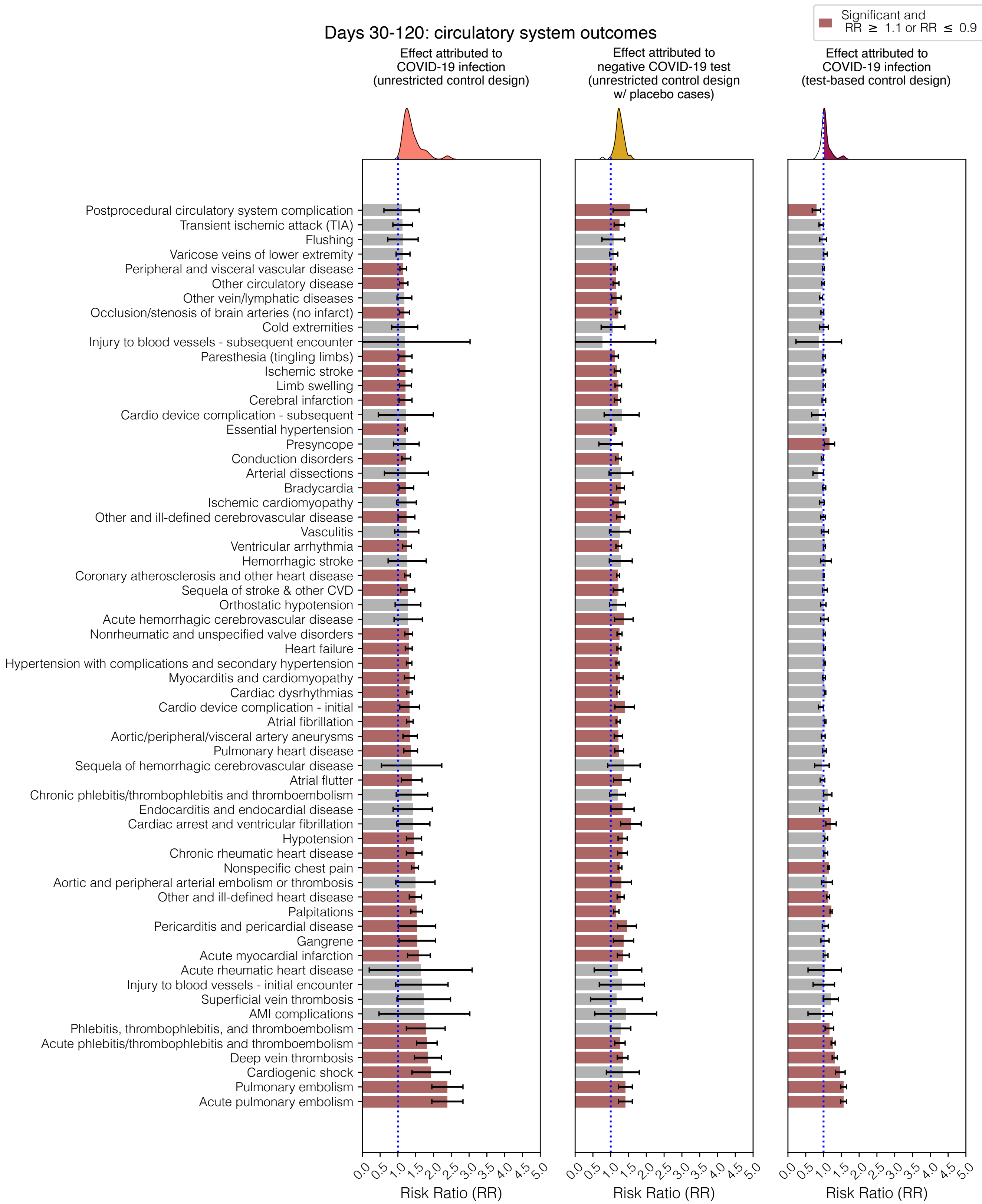


**Fig. S15.** Risk ratios for all outcomes related to the circulatory system on days 30–120 post-enrollment. Effects are shown for an unrestricted control design (left), the test-based control design (right), and an unrestricted control design with placebo cases (middle). Red bars indicate significant effects ($p<0.05$ with Bonferroni correction for multiple comparisons; two-sided bootstrap) with $RR \geq 1.1$ or $RR \leq 0.9$.

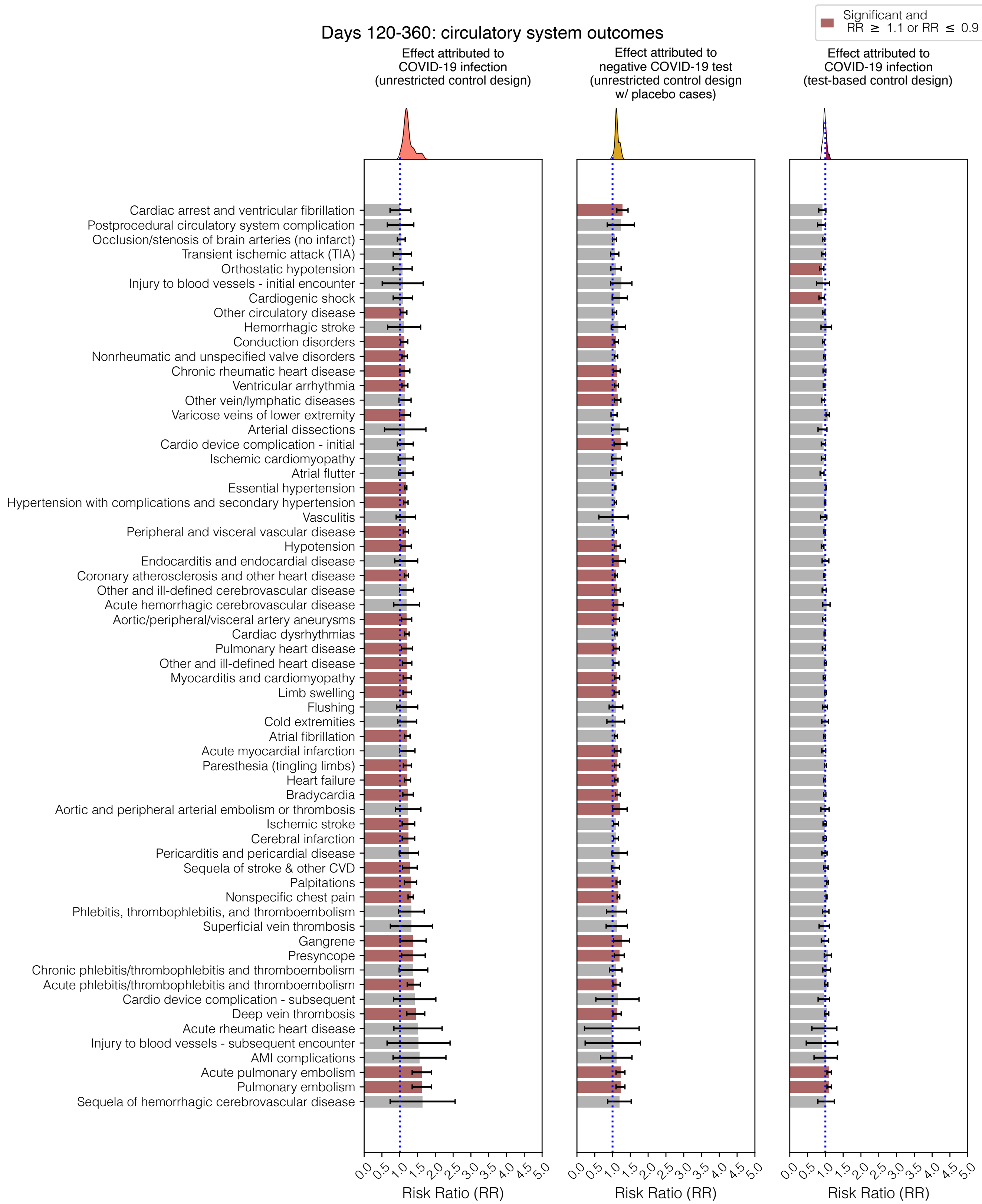


**Fig. S16.** Risk ratios for all outcomes related to the circulatory system on days 120–360 post-enrollment. Effects are shown for an unrestricted control design (left), the test-based control design (right), and an unrestricted control design with placebo cases (middle). Red bars indicate significant effects ($p<0.05$ with Bonferroni correction for multiple comparisons; two-sided bootstrap) with $RR \geq 1.1$ or $RR \leq 0.9$.

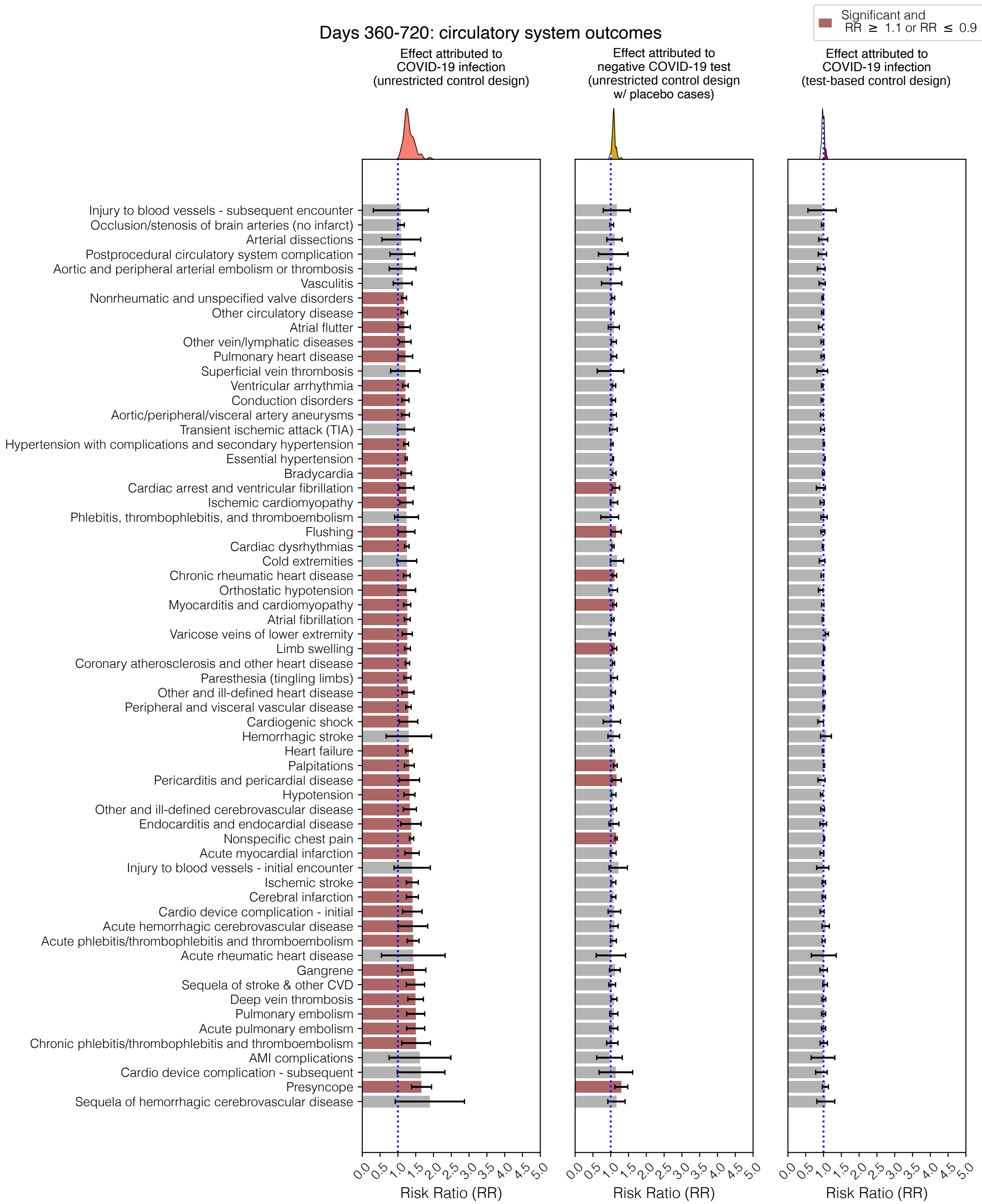


**Fig. S17.** Risk ratios for all outcomes related to the circulatory system on days 360–720 post-enrollment. Effects are shown for an unrestricted control design (left), the test-based control design (right), and an unrestricted control design with placebo cases (middle). Red bars indicate significant effects (p<0.05 with Bonferroni correction for multiple comparisons; two-sided bootstrap) with RR≥1.1 or RR≤0.9.

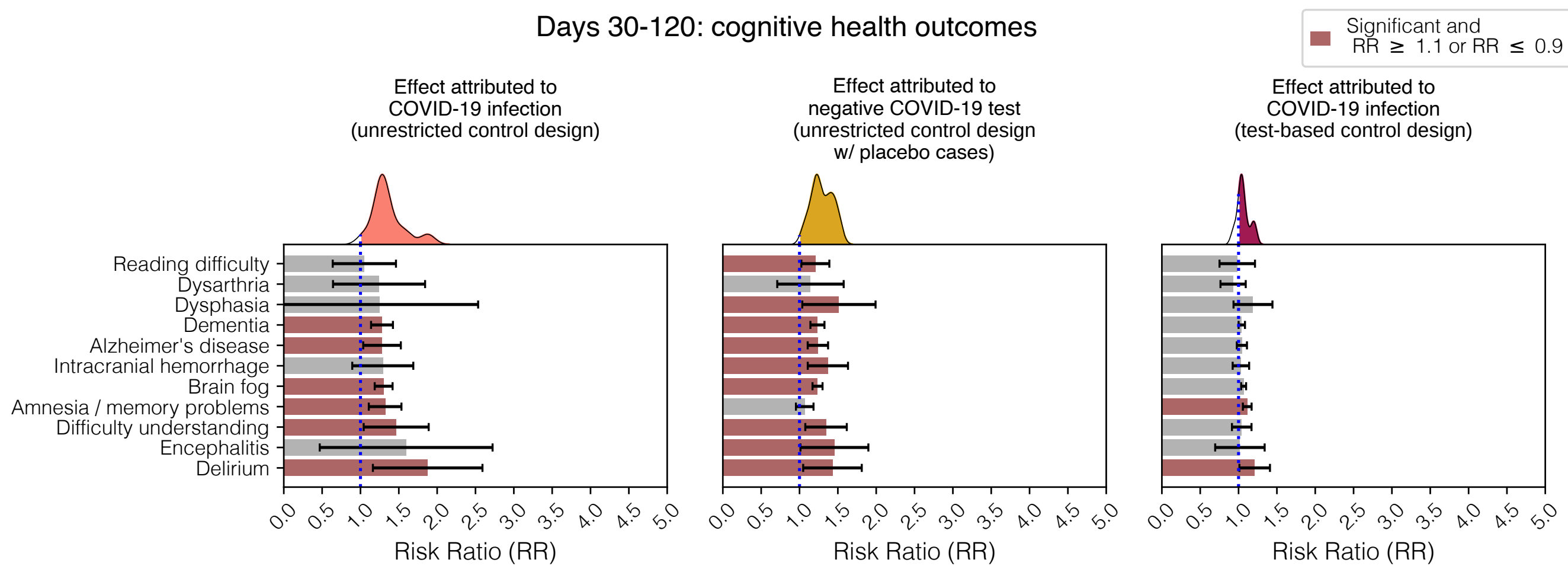


**Fig. S18.** Risk ratios for all outcomes related to cognitive health on days 30–120 post-enrollment. Effects are shown for an unrestricted control design (left), the test-based control design (right), and an unrestricted control design with placebo cases (middle). Red bars indicate significant effects ($p<0.05$ with Bonferroni correction for multiple comparisons; two-sided bootstrap) with $RR \geq 1.1$ or $RR \leq 0.9$.

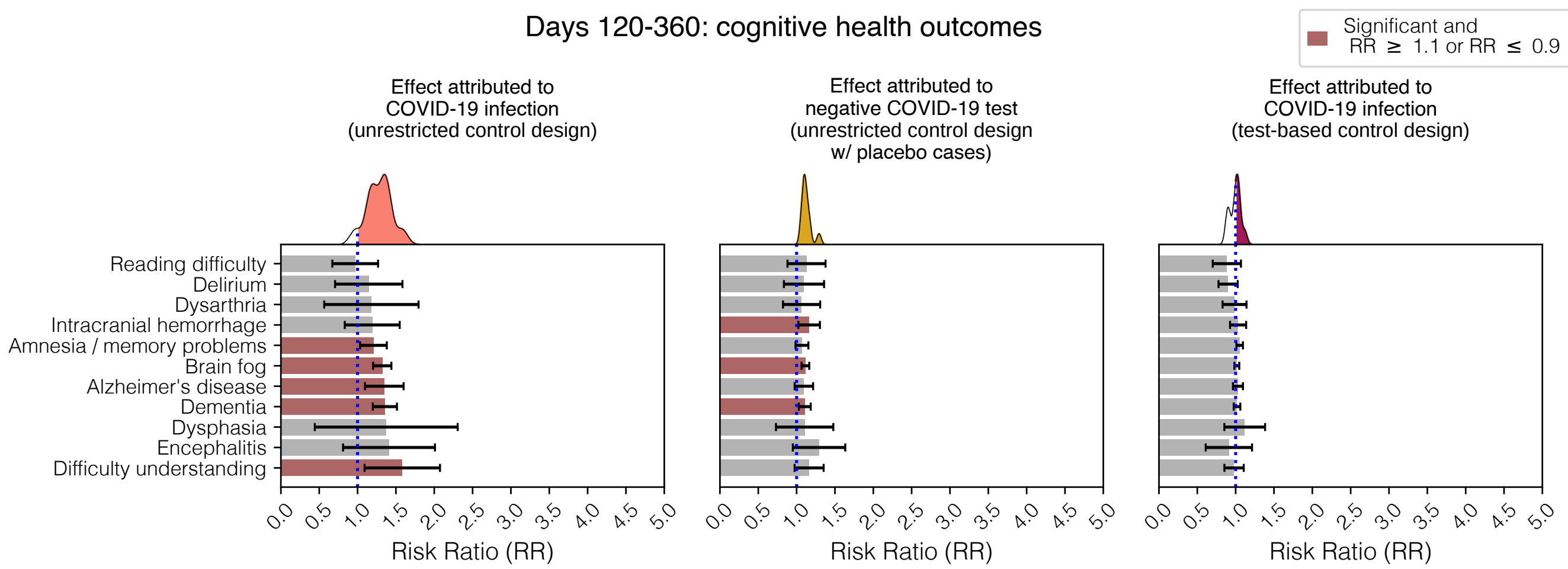


**Fig. S19.** Risk ratios for all outcomes related to cognitive health on days 120–360 post-enrollment. Effects are shown for an unrestricted control design (left), the test-based control design (right), and an unrestricted control design with placebo cases (middle). Red bars indicate significant effects ($p < 0.05$ with Bonferroni correction for multiple comparisons; two-sided bootstrap) with $RR \geq 1.1$ or $RR \leq 0.9$.

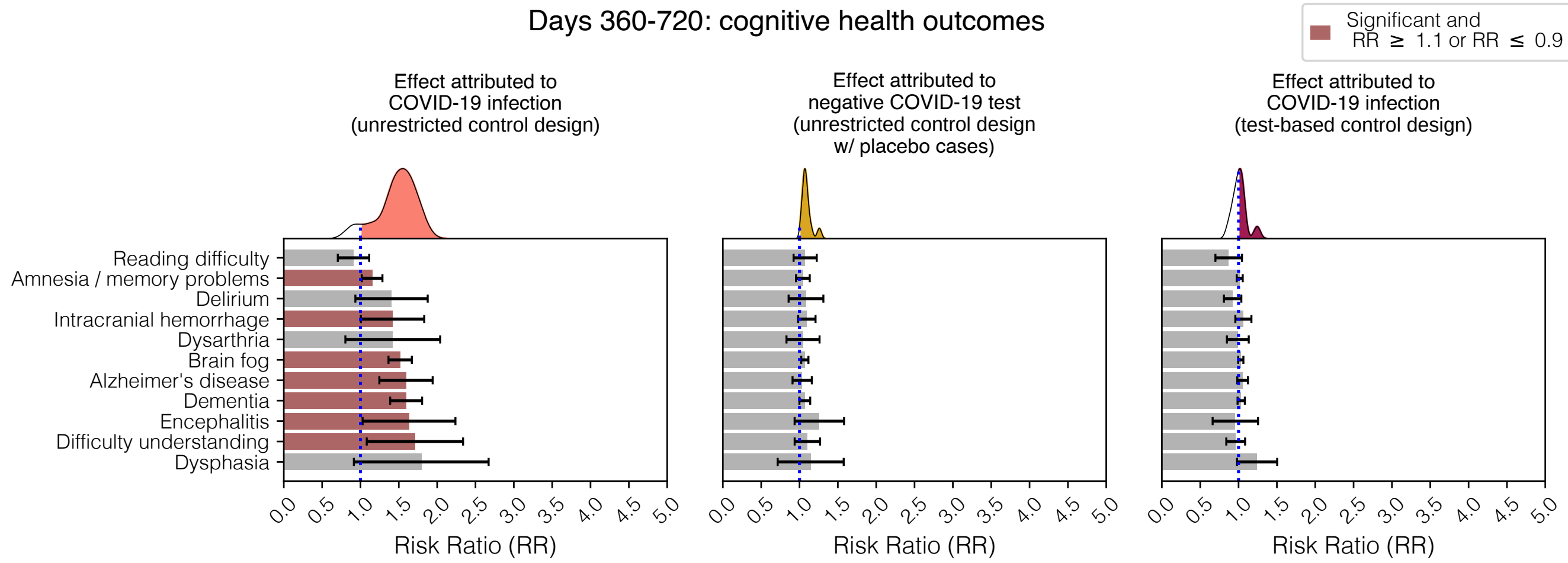


**Fig. S20.** Risk ratios for all outcomes related to cognitive health on days 360–720 post-enrollment. Effects are shown for an unrestricted control design (left), the test-based control design (right), and an unrestricted control design with placebo cases (middle). Red bars indicate significant effects ($p<0.05$ with Bonferroni correction for multiple comparisons; two-sided bootstrap) with $RR \geq 1.1$ or $RR \leq 0.9$.

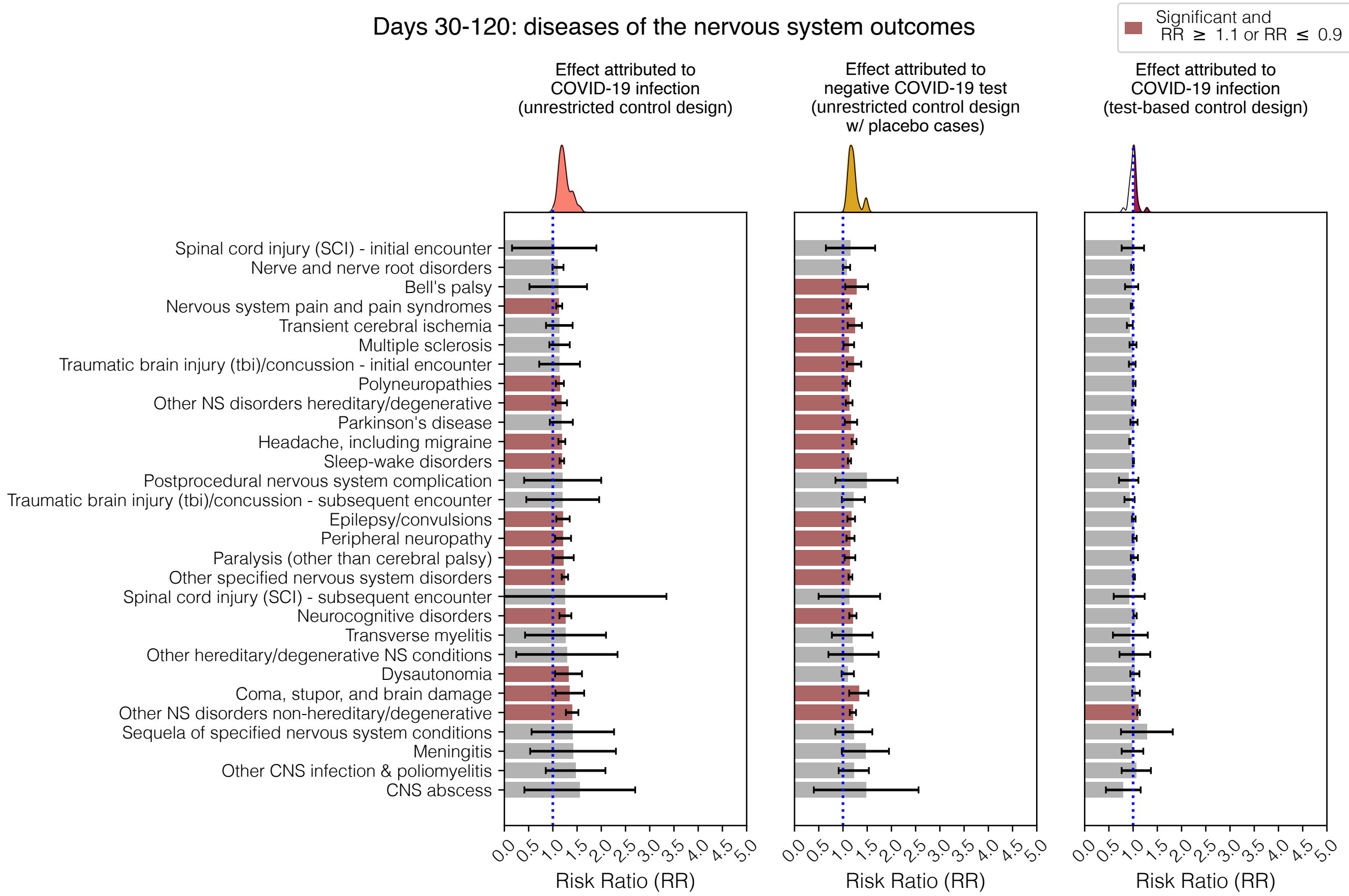


**Fig. S21.** Risk ratios for all outcomes related to diseases of the nervous system on days 30–120 post-enrollment. Effects are shown for an unrestricted control design (left), the test-based control design (right), and an unrestricted control design with placebo cases (middle). Red bars indicate significant effects ($p<0.05$ with Bonferroni correction for multiple comparisons; two-sided bootstrap) with $RR \geq 1.1$ or $RR \leq 0.9$.

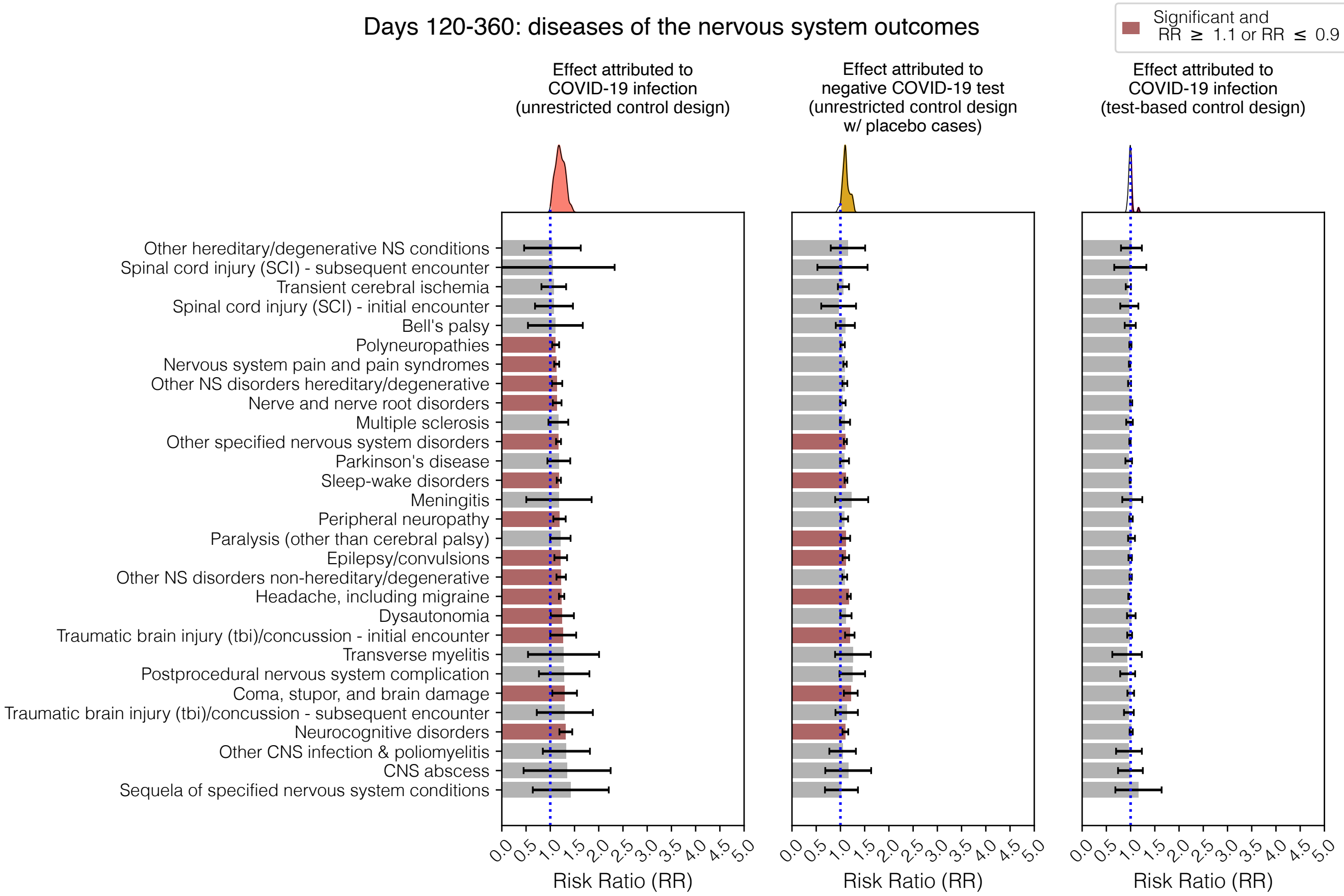


**Fig. S22.** Risk ratios for all outcomes related to diseases of the nervous system on days 120–360 post-enrollment. Effects are shown for an unrestricted control design (left), the test-based control design (right), and an unrestricted control design with placebo cases (middle). Red bars indicate significant effects ($p<0.05$ with Bonferroni correction for multiple comparisons; two-sided bootstrap) with $RR \geq 1.1$ or $RR \leq 0.9$.

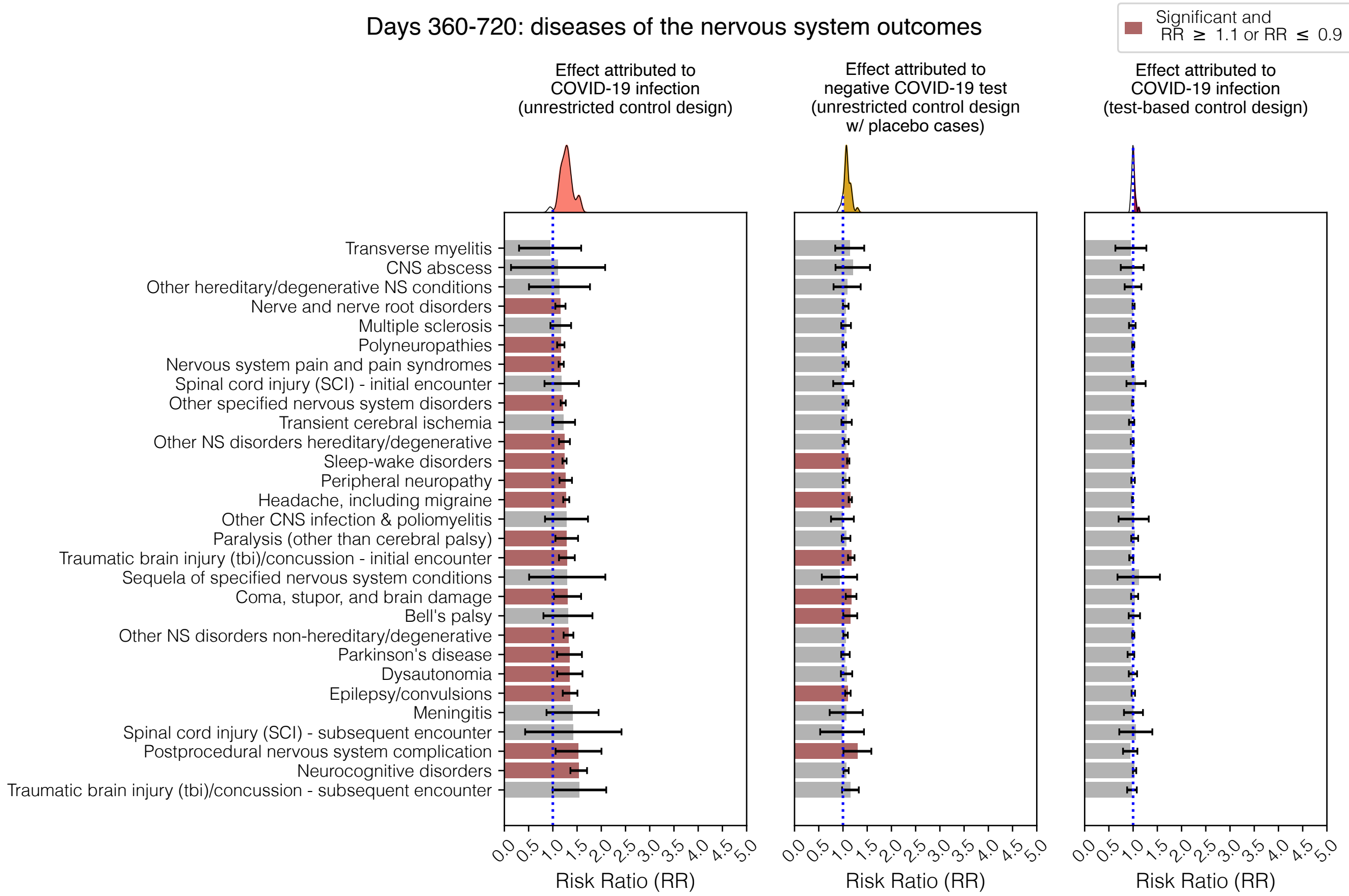


**Fig. S23.** Risk ratios for all outcomes related to diseases of the nervous system on days 360–720 post-enrollment. Effects are shown for an unrestricted control design (left), the test-based control design (right), and an unrestricted control design with placebo cases (middle). Red bars indicate significant effects ($p<0.05$ with Bonferroni correction for multiple comparisons; two-sided bootstrap) with RR$\geq$1.1 or RR$\leq$0.9.

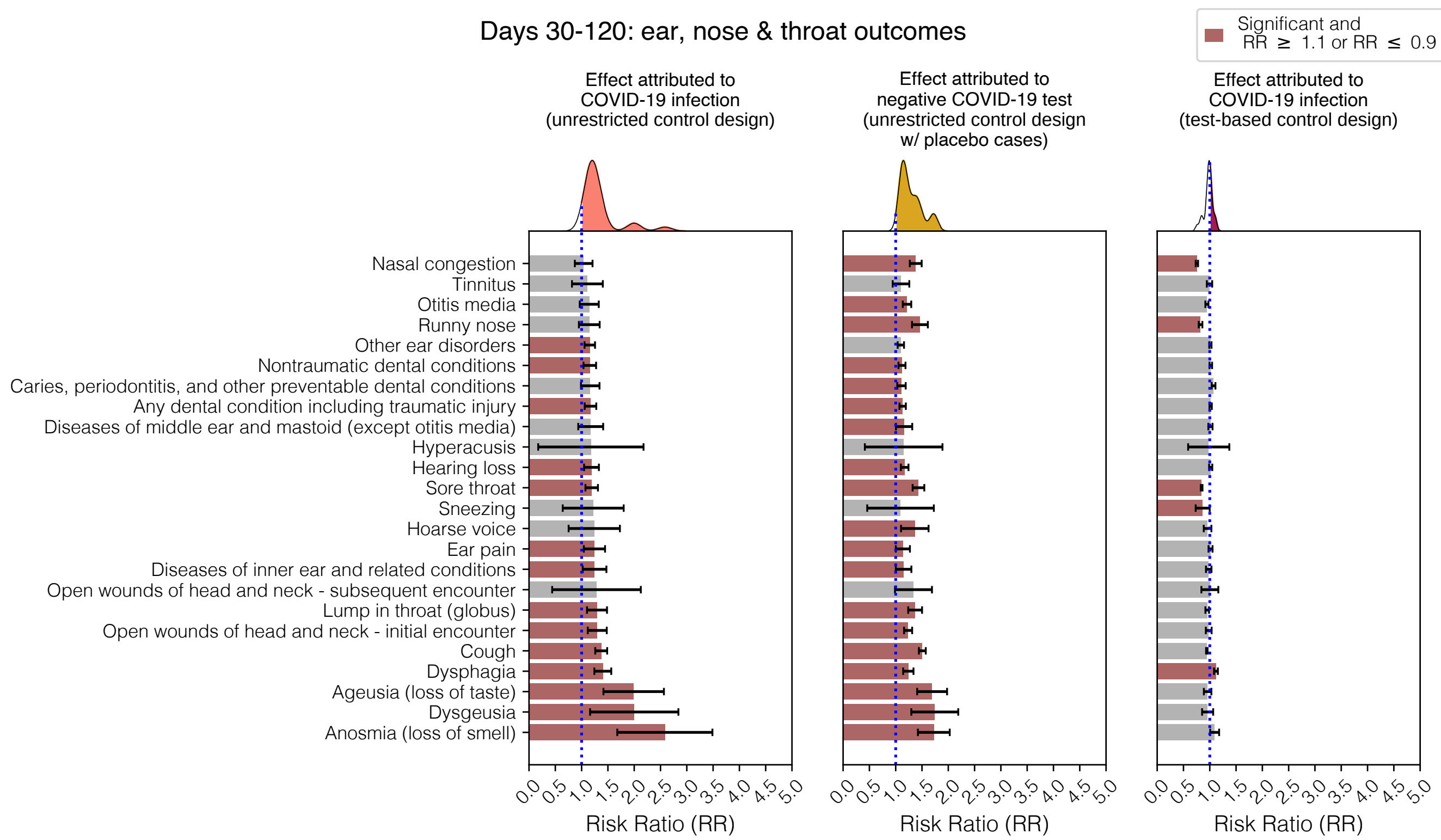


**Fig. S24.** Risk ratios for all outcomes related to ear, nose & throat on days 30–120 post-enrollment. Effects are shown for an unrestricted control design (left), the test-based control design (right), and an unrestricted control design with placebo cases (middle). Red bars indicate significant effects ($p < 0.05$ with Bonferroni correction for multiple comparisons; two-sided bootstrap) with $RR \geq 1.1$ or $RR \leq 0.9$.

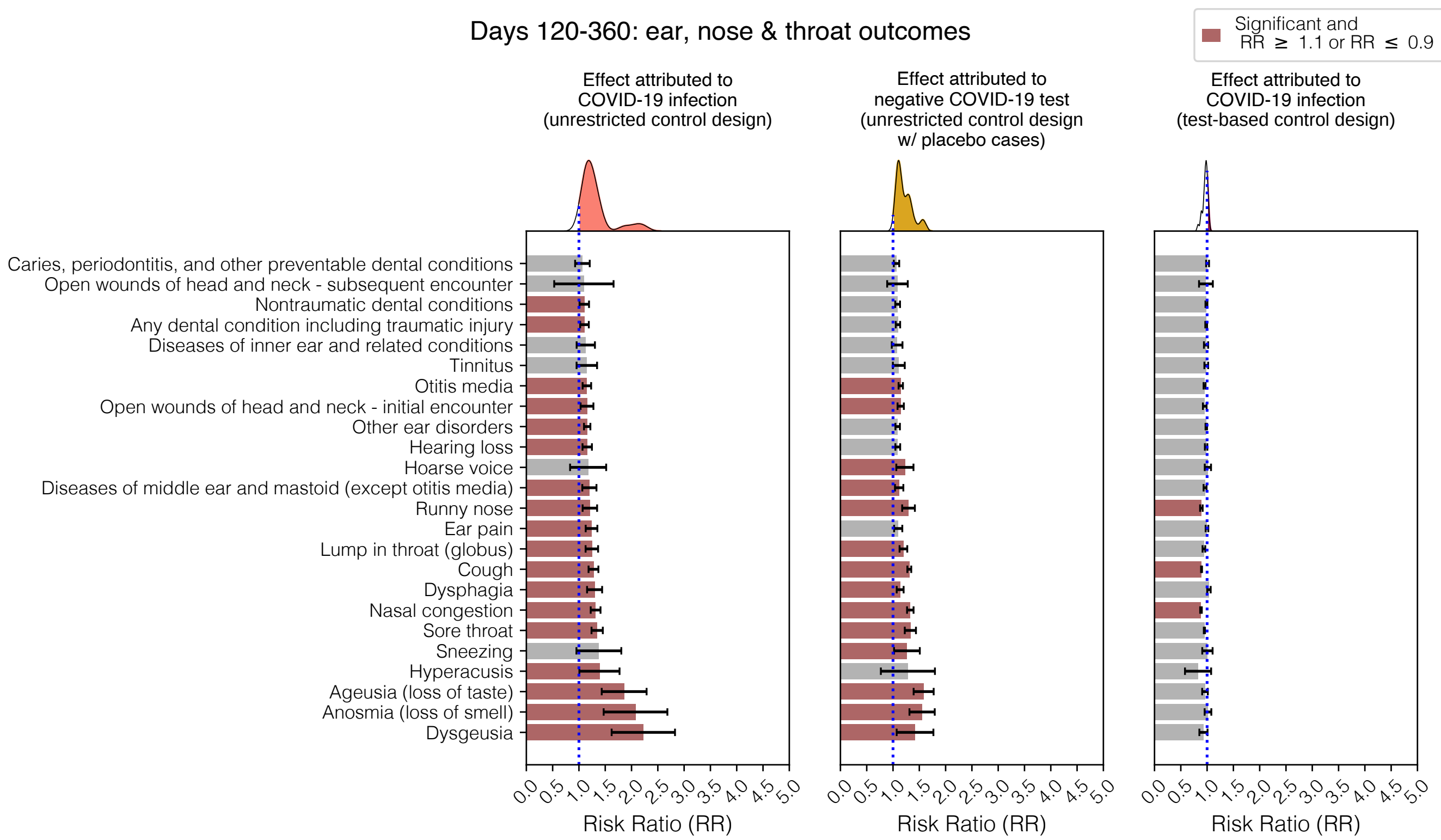


**Fig. S25.** Risk ratios for all outcomes related to ear, nose & throat on days 120–360 post-enrollment. Effects are shown for an unrestricted control design (left), the test-based control design (right), and an unrestricted control design with placebo cases (middle). Red bars indicate significant effects (p<0.05 with Bonferroni correction for multiple comparisons; two-sided bootstrap) with RR≥1.1 or RR≤0.9.

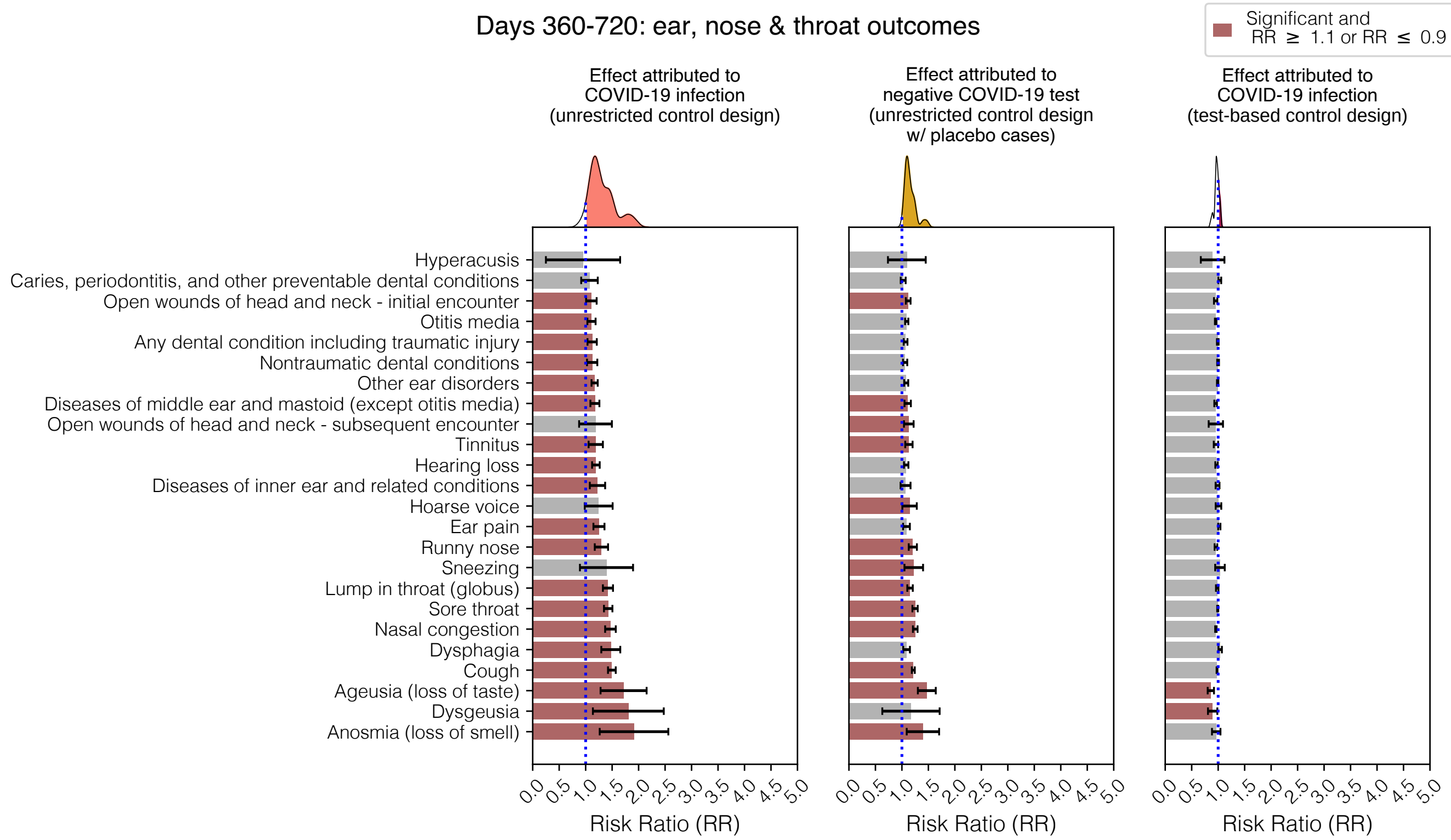


**Fig. S26.** Risk ratios for all outcomes related to ear, nose & throat on days 360–720 post-enrollment. Effects are shown for an unrestricted control design (left), the test-based control design (right), and an unrestricted control design with placebo cases (middle). Red bars indicate significant effects (p<0.05 with Bonferroni correction for multiple comparisons; two-sided bootstrap) with RR≥1.1 or RR≤0.9.

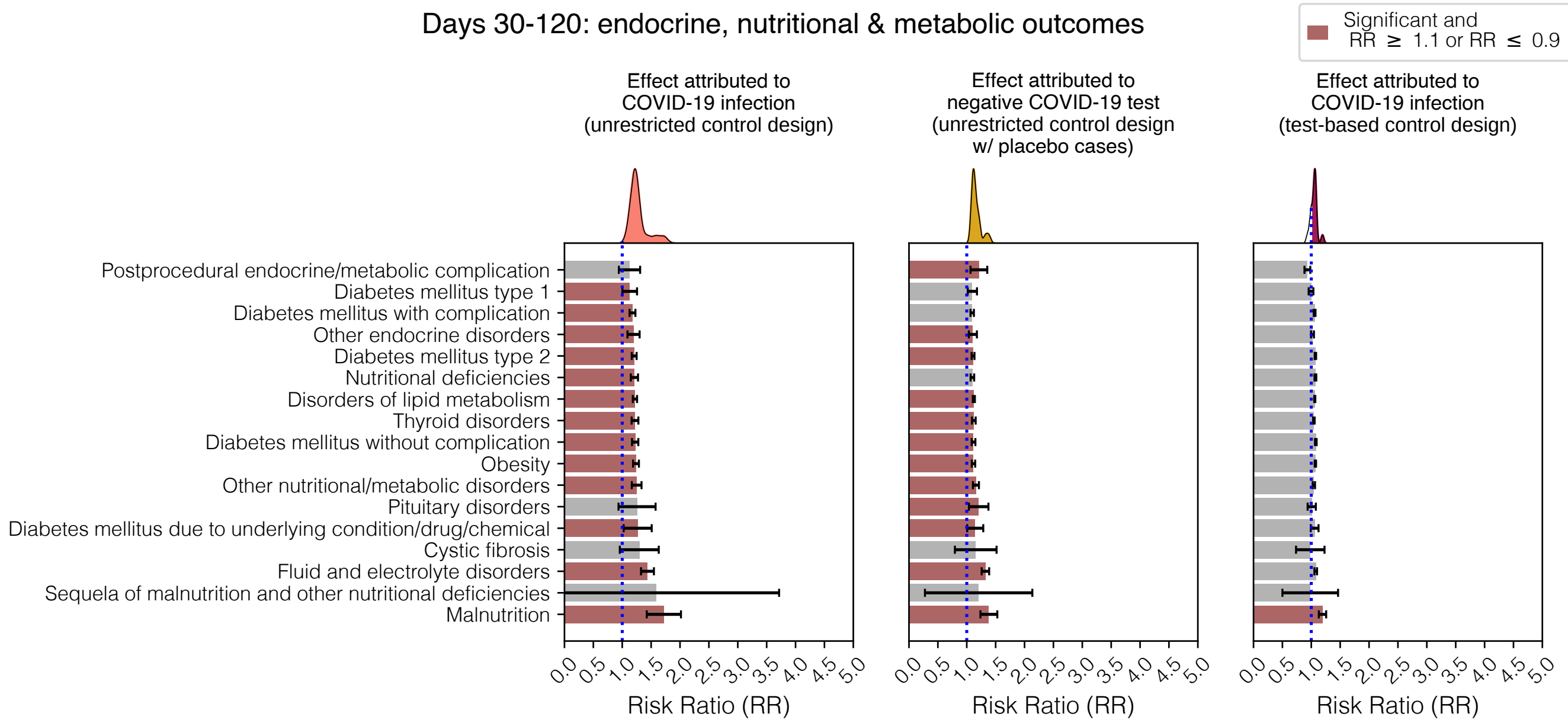


**Fig. S27.** Risk ratios for all outcomes related to the endocrine, nutritional & metabolic system on days 30–120 post-enrollment. Effects are shown for an unrestricted control design (left), the test-based control design (right), and an unrestricted control design with placebo cases (middle). Red bars indicate significant effects ($p<0.05$ with Bonferroni correction for multiple comparisons; two-sided bootstrap) with $RR \geq 1.1$ or $RR \leq 0.9$.

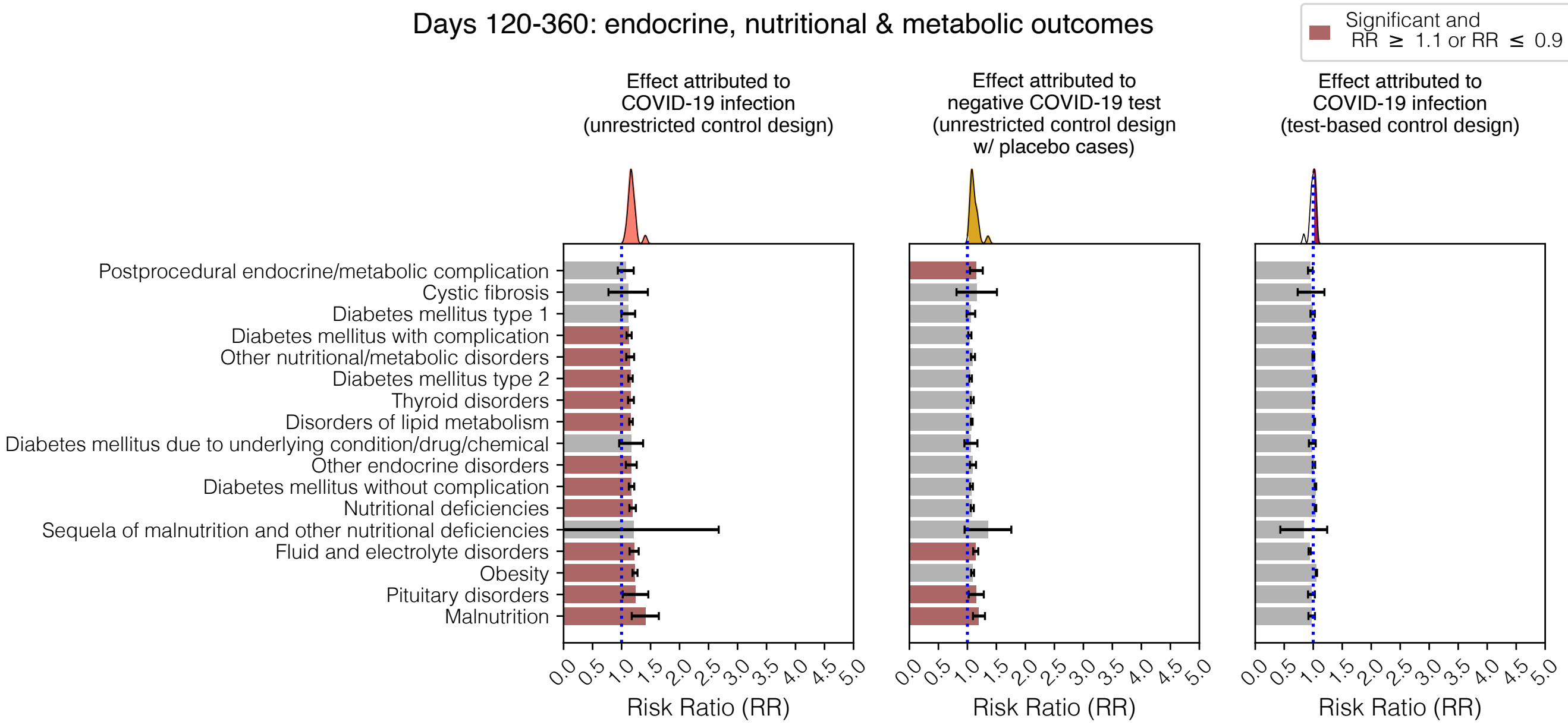


**Fig. S28.** Risk ratios for all outcomes related to the endocrine, nutritional & metabolic system on days 120–360 post-enrollment. Effects are shown for an unrestricted control design (left), the test-based control design (right), and an unrestricted control design with placebo cases (middle). Red bars indicate significant effects ($p<0.05$ with Bonferroni correction for multiple comparisons; two-sided bootstrap) with $RR \geq 1.1$ or $RR \leq 0.9$.

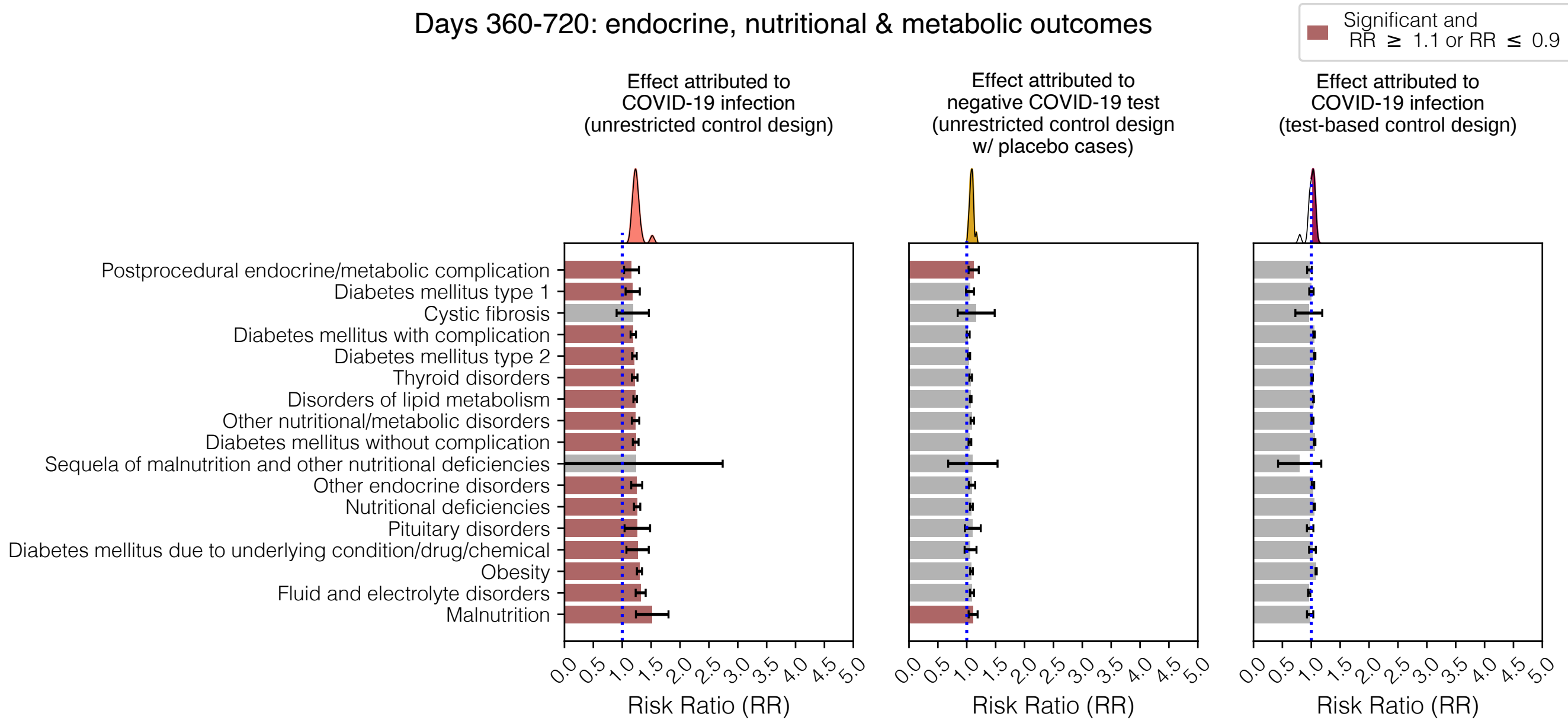


**Fig. S29.** Risk ratios for all outcomes related to the endocrine, nutritional & metabolic system on days 360–720 post-enrollment. Effects are shown for an unrestricted control design (left), the test-based control design (right), and an unrestricted control design with placebo cases (middle). Red bars indicate significant effects ($p<0.05$ with Bonferroni correction for multiple comparisons; two-sided bootstrap) with RR$\geq$1.1 or RR$\leq$0.9.

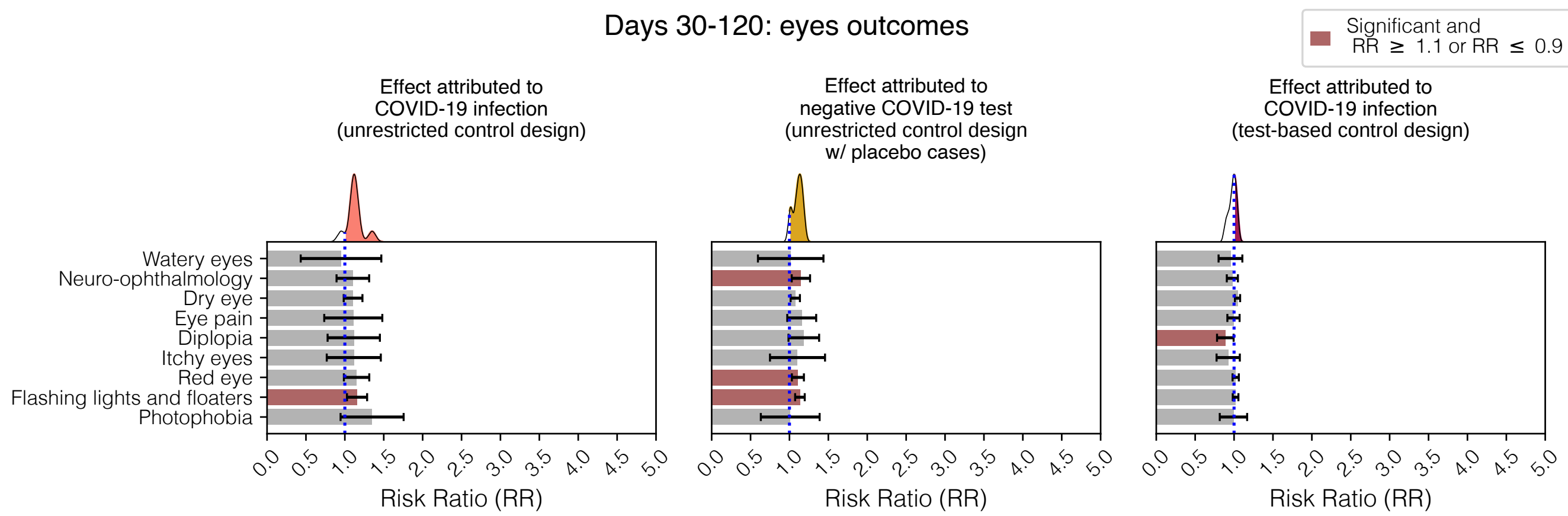


**Fig. S30.** Risk ratios for all outcomes related to eyes on days 30–120 post-enrollment. Effects are shown for an unrestricted control design (left), the test-based control design (right), and an unrestricted control design with placebo cases (middle). Red bars indicate significant effects ($p<0.05$ with Bonferroni correction for multiple comparisons; two-sided bootstrap) with $RR\geq1.1$ or $RR\leq0.9$.

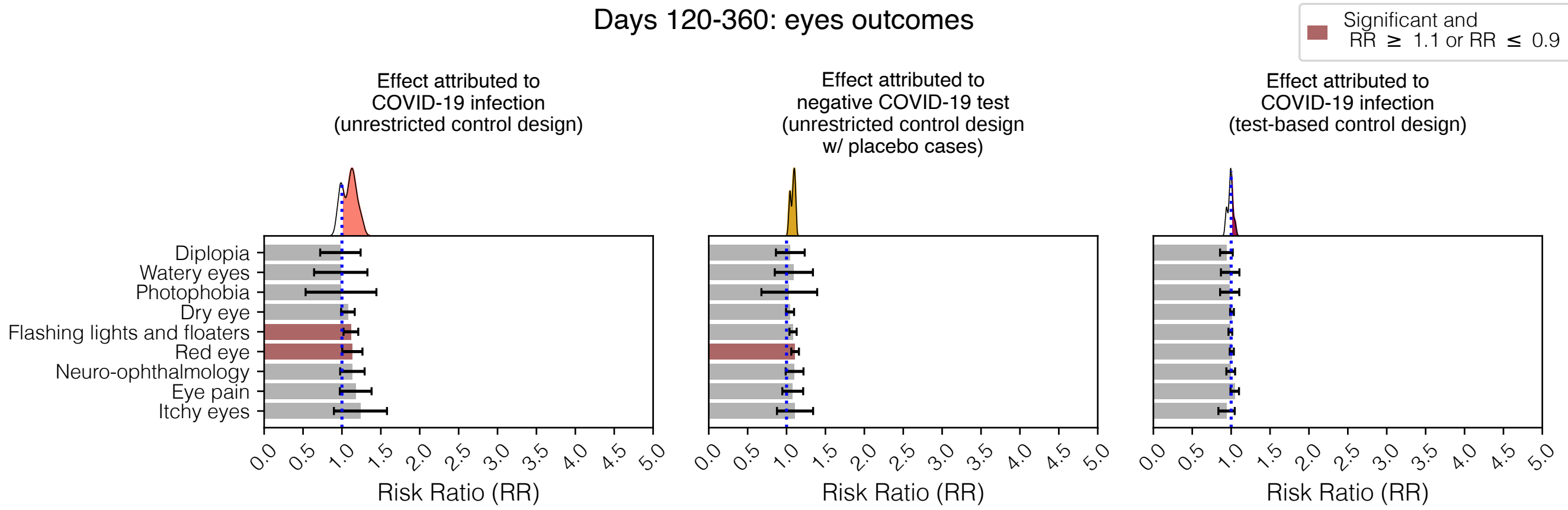


**Fig. S31.** Risk ratios for all outcomes related to eyes on days 120–360 post-enrollment. Effects are shown for an unrestricted control design (left), the test-based control design (right), and an unrestricted control design with placebo cases (middle). Red bars indicate significant effects ($p<0.05$ with Bonferroni correction for multiple comparisons; two-sided bootstrap) with $RR \geq 1.1$ or $RR \leq 0.9$.

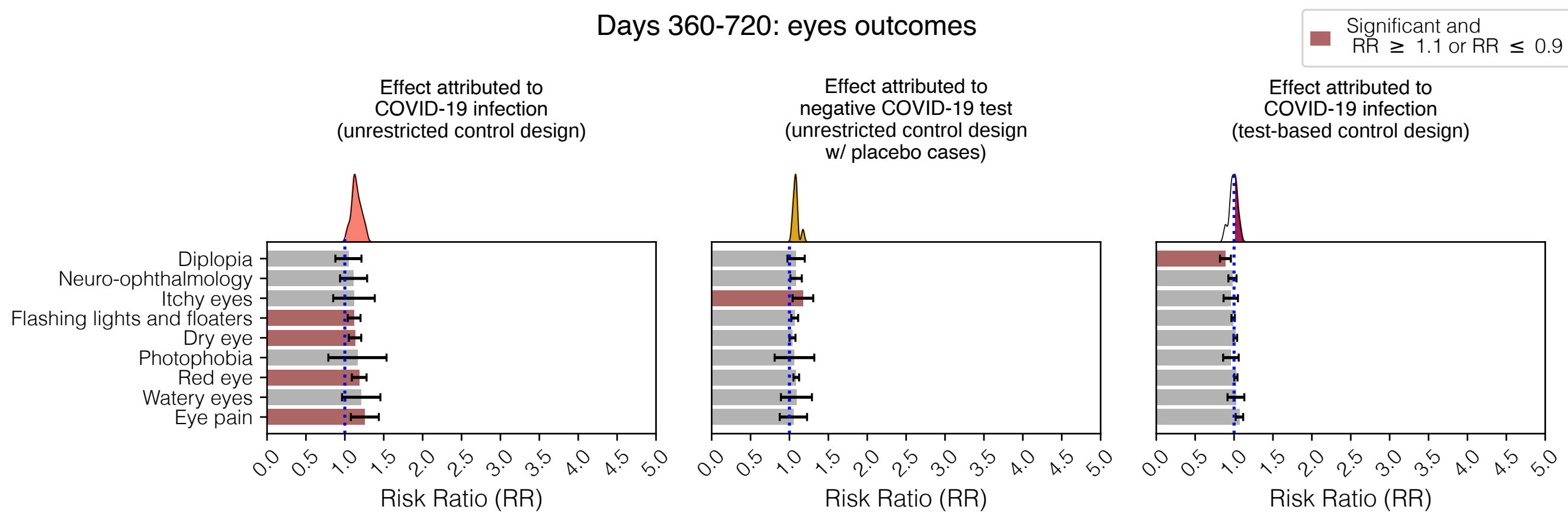


**Fig. S32.** Risk ratios for all outcomes related to eyes on days 360–720 post-enrollment. Effects are shown for an unrestricted control design (left), the test-based control design (right), and an unrestricted control design with placebo cases (middle). Red bars indicate significant effects ($p<0.05$ with Bonferroni correction for multiple comparisons; two-sided bootstrap) with $RR \geq 1.1$ or $RR \leq 0.9$.

 

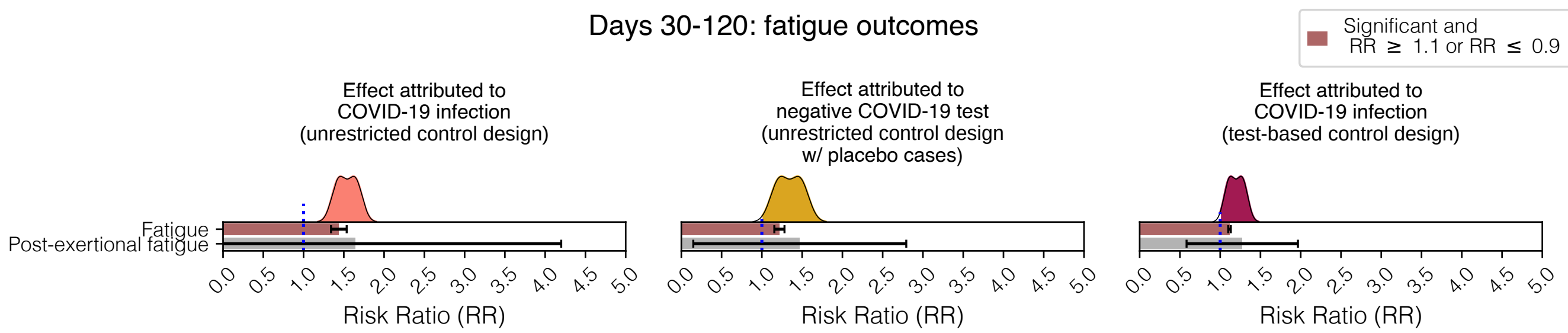


**Fig. S33.** Risk ratios for all outcomes related to fatigue on days 30–120 post-enrollment. Effects are shown for an unrestricted control design (left), the test-based control design (right), and an unrestricted control design with placebo cases (middle). Red bars indicate significant effects ($p<0.05$ with Bonferroni correction for multiple comparisons; two-sided bootstrap) with $RR\geq1.1$ or $RR\leq0.9$.

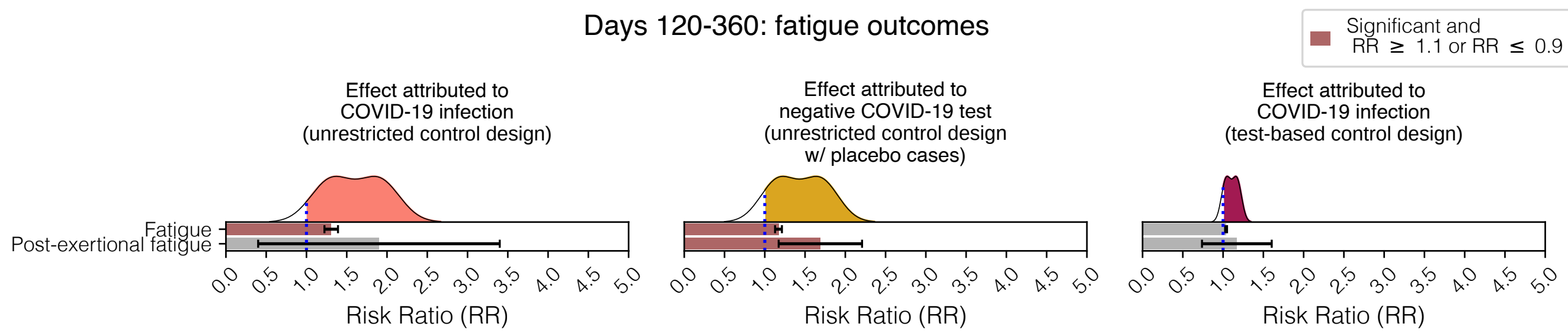


**Fig. S34.** Risk ratios for all outcomes related to fatigue on days 120–360 post-enrollment. Effects are shown for an unrestricted control design (left), the test-based control design (right), and an unrestricted control design with placebo cases (middle). Red bars indicate significant effects ($p<0.05$ with Bonferroni correction for multiple comparisons; two-sided bootstrap) with $RR \geq 1.1$ or $RR \leq 0.9$.

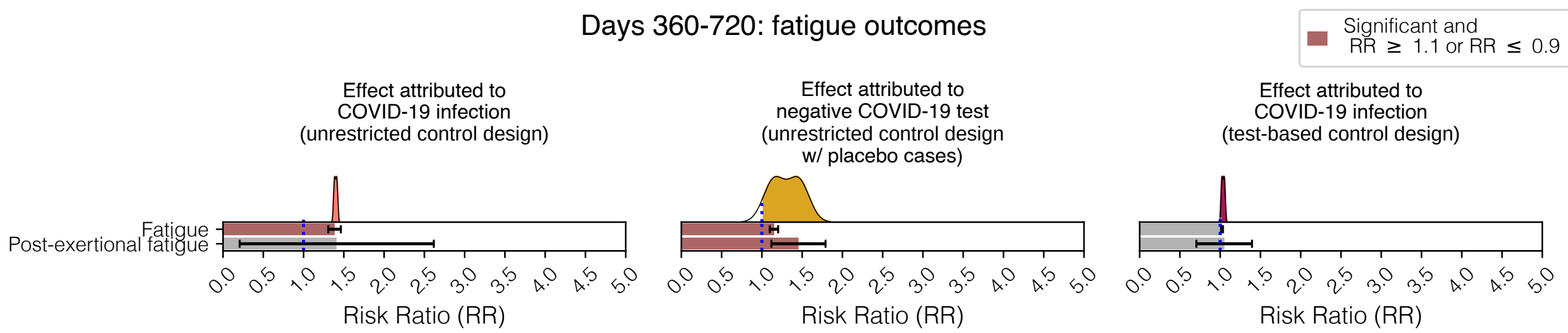


**Fig. S35.** Risk ratios for all outcomes related to fatigue on days 360–720 post-enrollment. Effects are shown for an unrestricted control design (left), the test-based control design (right), and an unrestricted control design with placebo cases (middle). Red bars indicate significant effects ($p<0.05$ with Bonferroni correction for multiple comparisons; two-sided bootstrap) with $RR\geq1.1$ or $RR\leq0.9$.

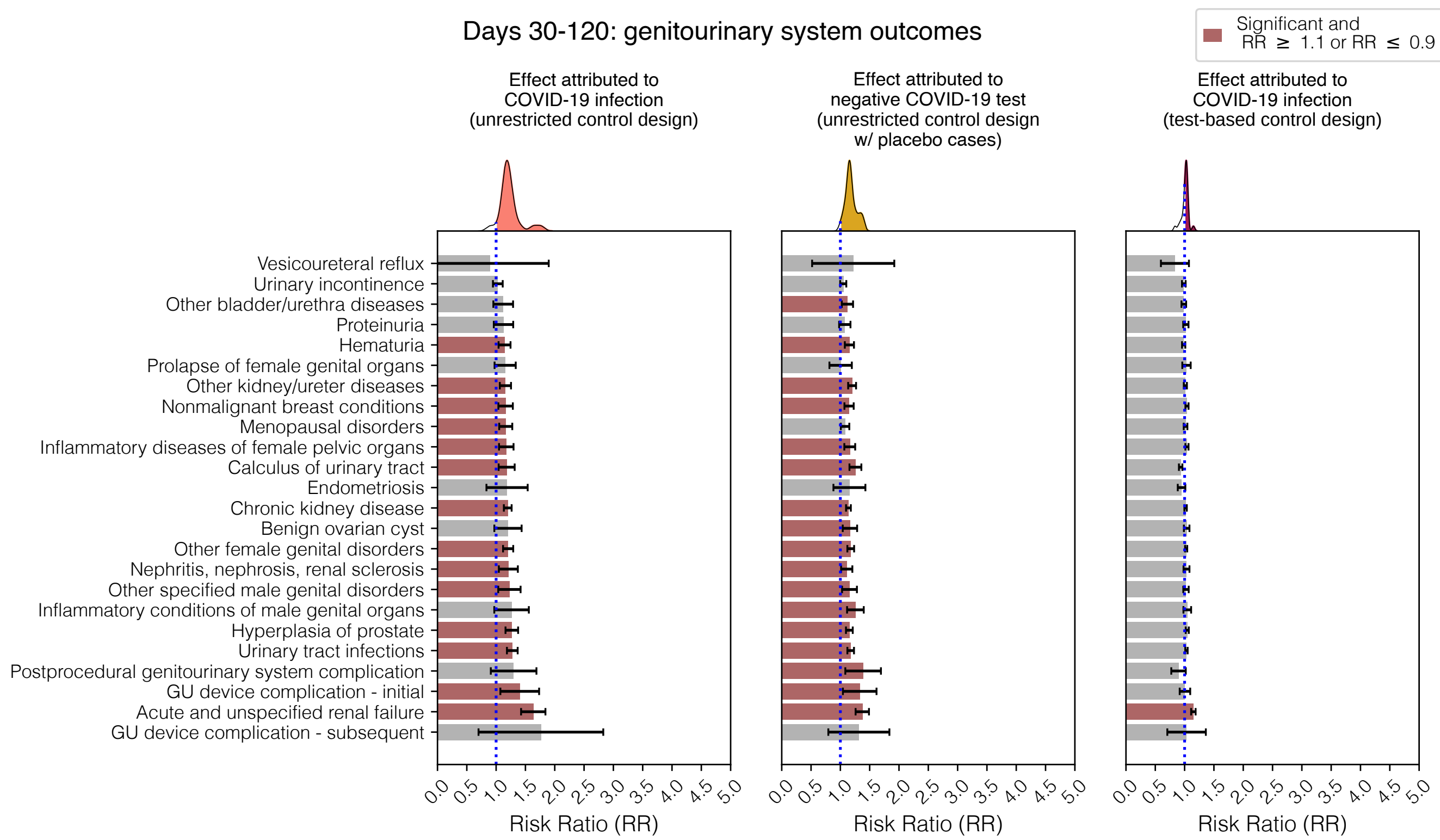


**Fig. S36.** Risk ratios for all outcomes related to the genitourinary system on days 30–120 post-enrollment. Effects are shown for an unrestricted control design (left), the test-based control design (right), and an unrestricted control design with placebo cases (middle). Red bars indicate significant effects ($p<0.05$ with Bonferroni correction for multiple comparisons; two-sided bootstrap) with $RR \geq 1.1$ or $RR \leq 0.9$.

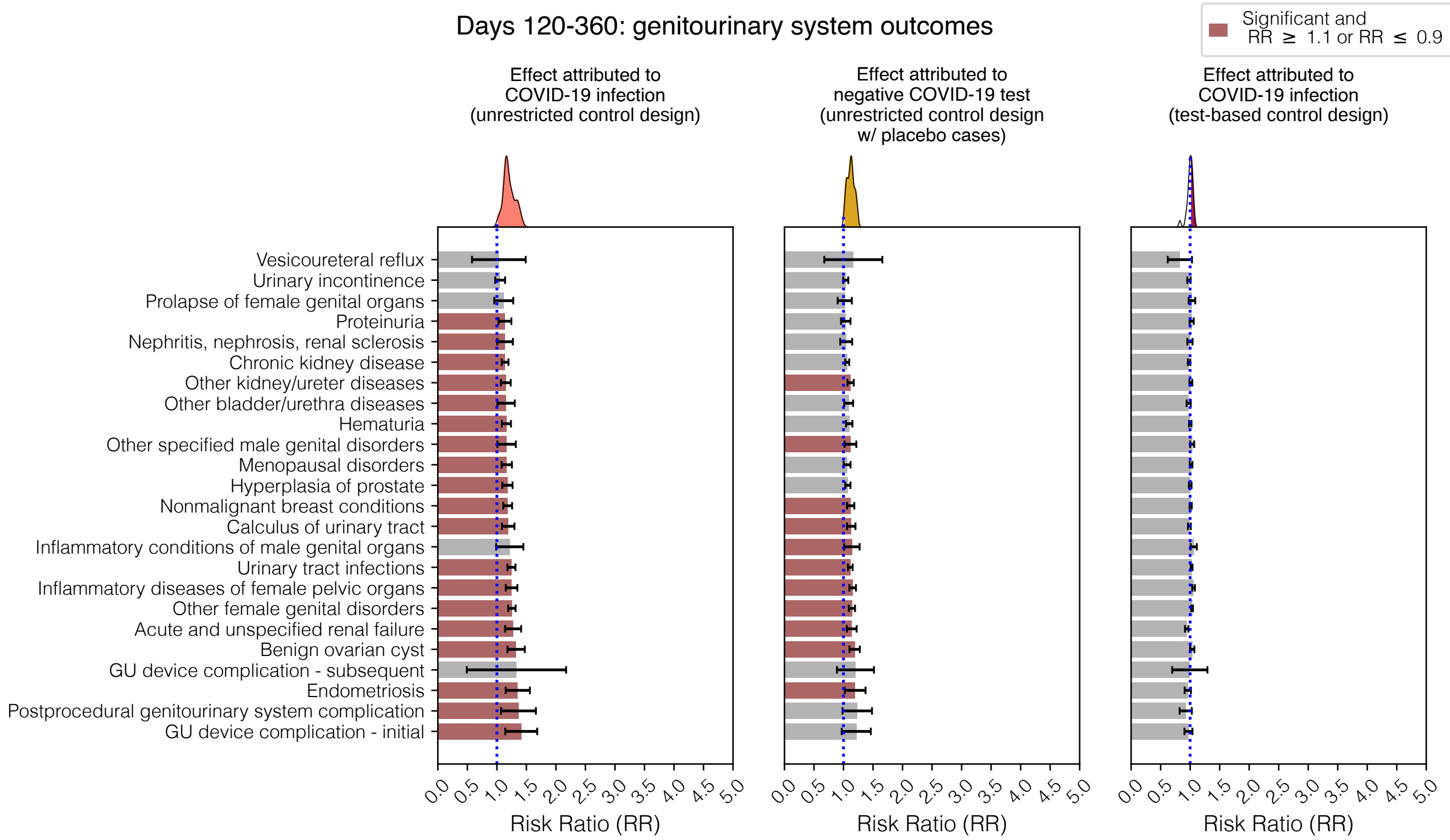


**Fig. S37.** Risk ratios for all outcomes related to the genitourinary system on days 120–360 post-enrollment. Effects are shown for an unrestricted control design (left), the test-based control design (right), and an unrestricted control design with placebo cases (middle). Red bars indicate significant effects ($p<0.05$ with Bonferroni correction for multiple comparisons; two-sided bootstrap) with $RR \geq 1.1$ or $RR \leq 0.9$.

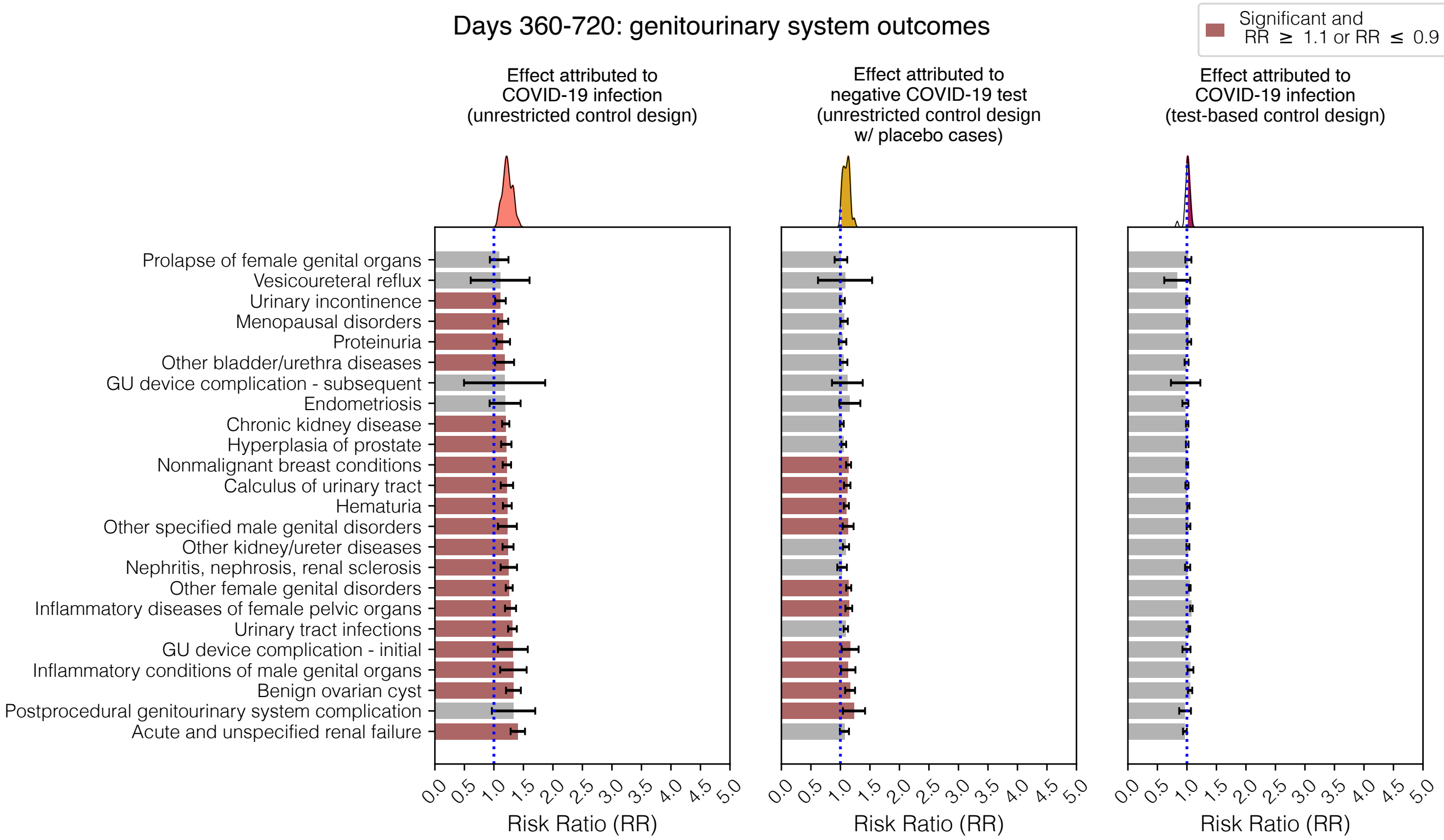


**Fig. S38.** Risk ratios for all outcomes related to the genitourinary system on days 360–720 post-enrollment. Effects are shown for an unrestricted control design (left), the test-based control design (right), and an unrestricted control design with placebo cases (middle). Red bars indicate significant effects ($p<0.05$ with Bonferroni correction for multiple comparisons; two-sided bootstrap) with $RR \geq 1.1$ or $RR \leq 0.9$.

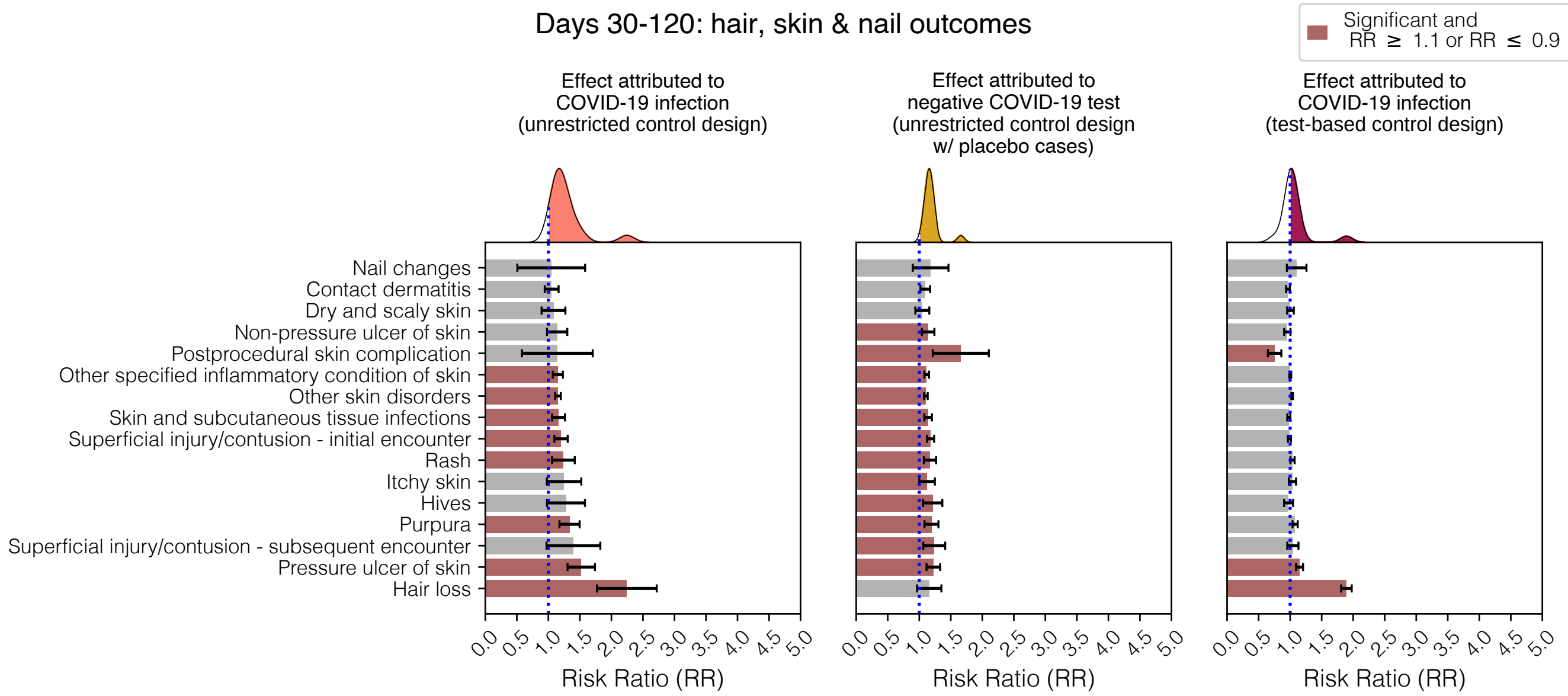


**Fig. S39.** Risk ratios for all outcomes related to hair, skin & nail on days 30–120 post-enrollment. Effects are shown for an unrestricted control design (left), the test-based control design (right), and an unrestricted control design with placebo cases (middle). Red bars indicate significant effects ($p<0.05$ with Bonferroni correction for multiple comparisons; two-sided bootstrap) with $RR\geq1.1$ or $RR\leq0.9$.

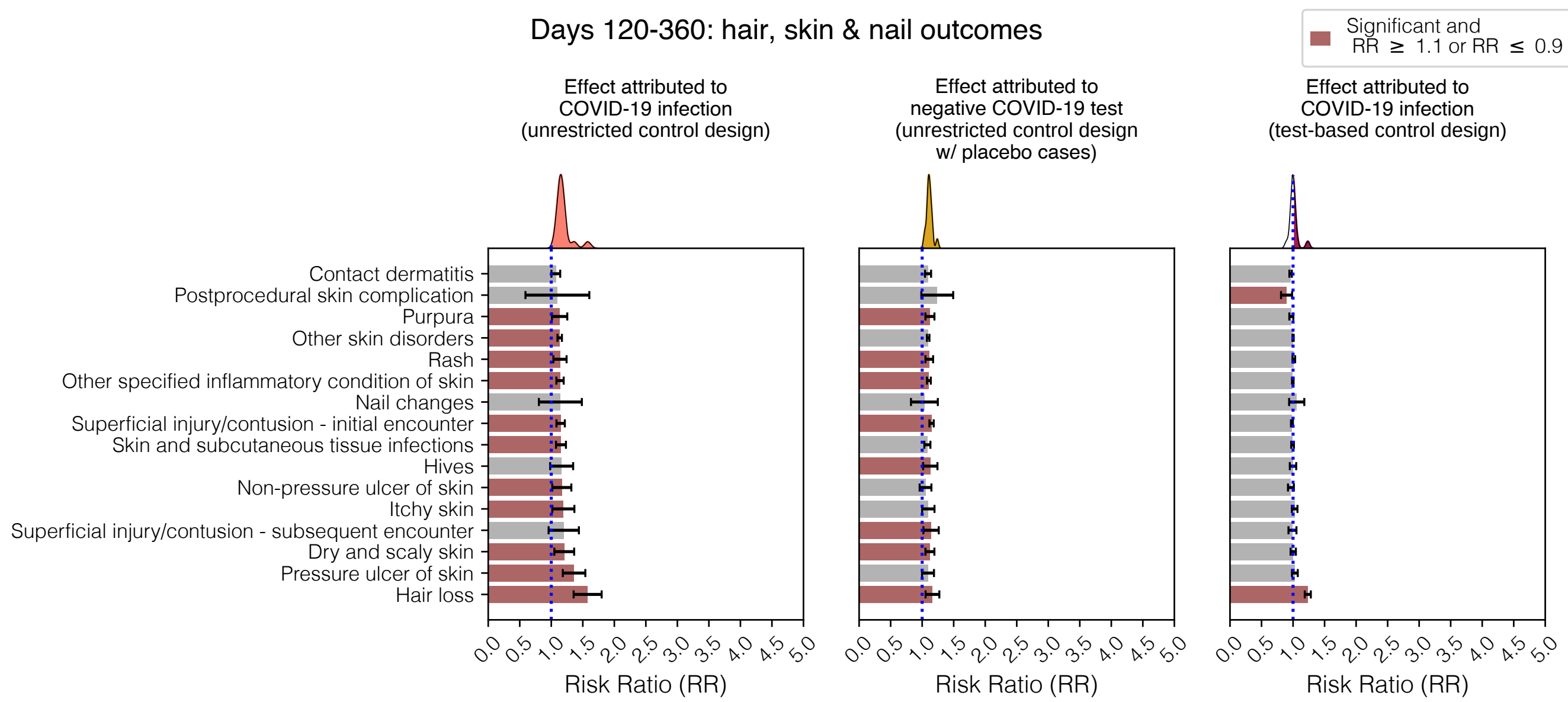


**Fig. S40.** Risk ratios for all outcomes related to hair, skin & nail on days 120–360 post-enrollment. Effects are shown for an unrestricted control design (left), the test-based control design (right), and an unrestricted control design with placebo cases (middle). Red bars indicate significant effects ($p<0.05$ with Bonferroni correction for multiple comparisons; two-sided bootstrap) with $RR \geq 1.1$ or $RR \leq 0.9$.

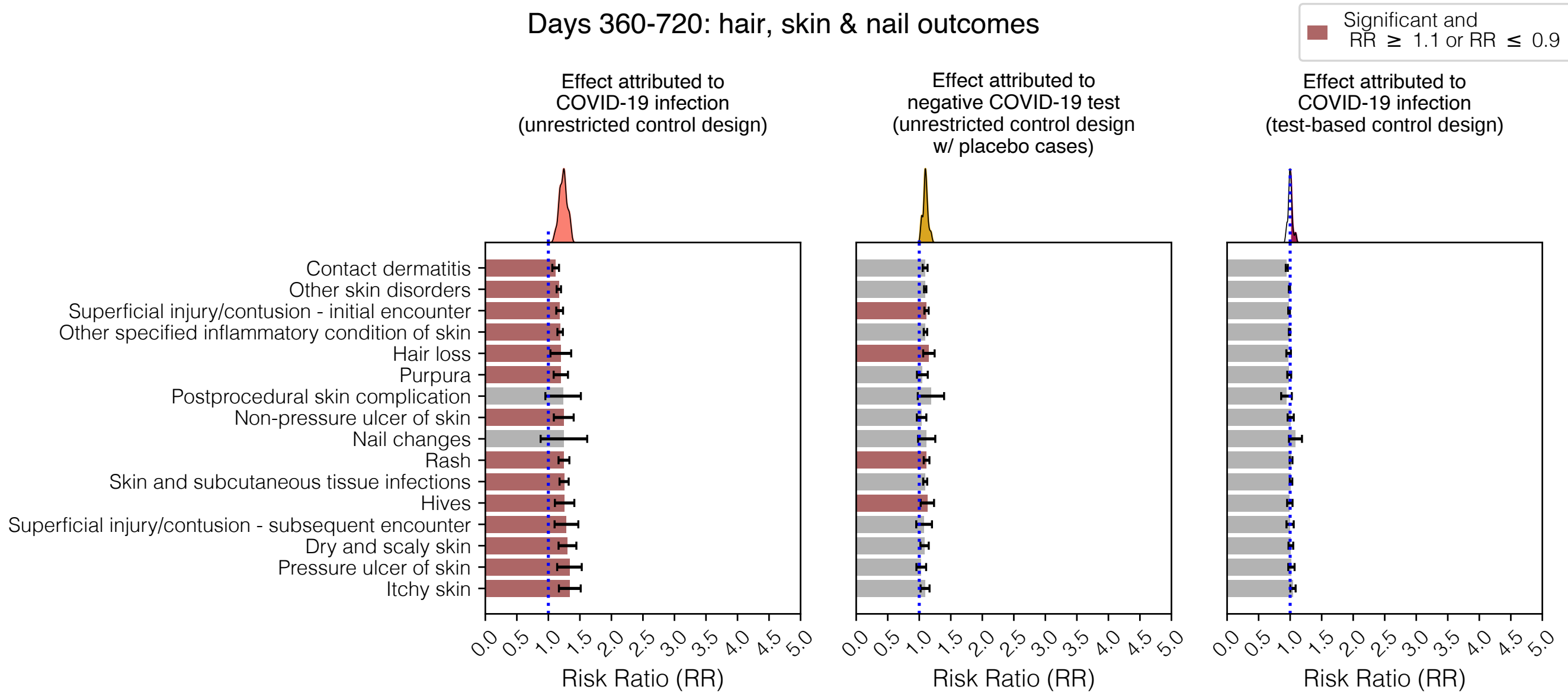


**Fig. S41.** Risk ratios for all outcomes related to hair, skin & nail on days 360–720 post-enrollment. Effects are shown for an unrestricted control design (left), the test-based control design (right), and an unrestricted control design with placebo cases (middle). Red bars indicate significant effects ($p<0.05$ with Bonferroni correction for multiple comparisons; two-sided bootstrap) with $RR\geq1.1$ or $RR\leq0.9$.

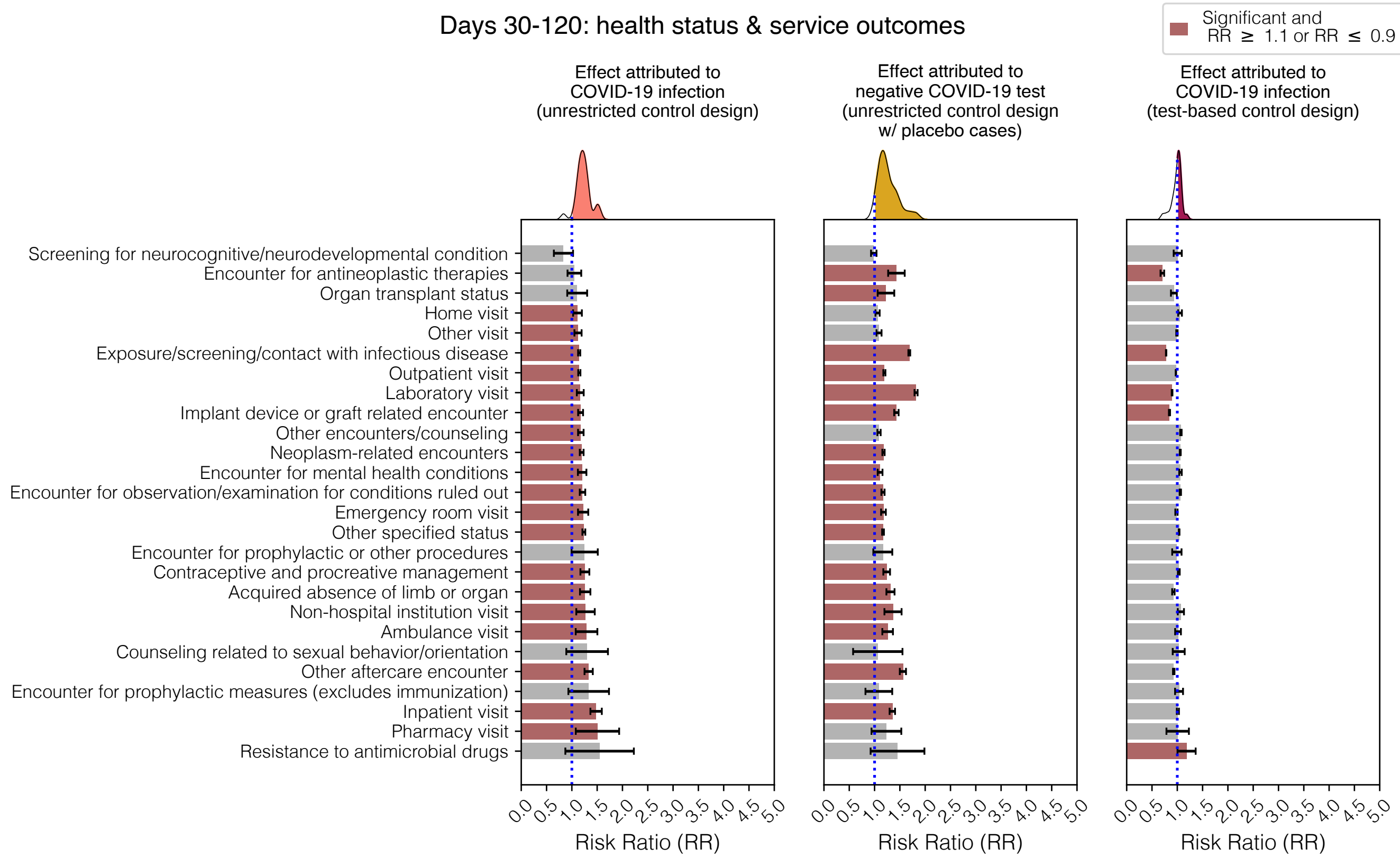


**Fig. S42.** Risk ratios for all outcomes related to health status & service on days 30–120 post-enrollment. Effects are shown for an unrestricted control design (left), the test-based control design (right), and an unrestricted control design with placebo cases (middle). Red bars indicate significant effects ($p<0.05$ with Bonferroni correction for multiple comparisons; two-sided bootstrap) with $RR \geq 1.1$ or $RR \leq 0.9$.

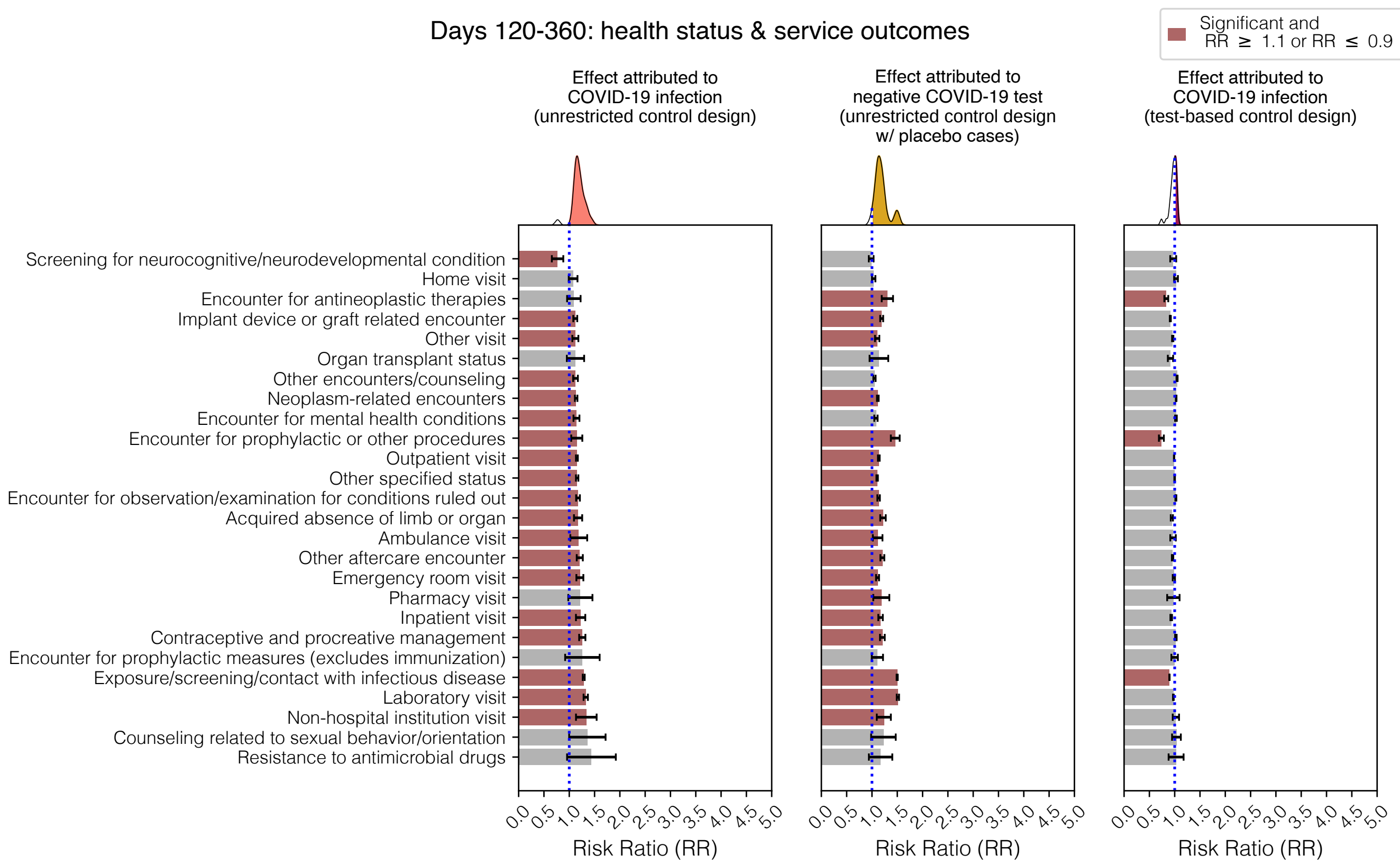


**Fig. S43.** Risk ratios for all outcomes related to health status & service on days 120–360 post-enrollment. Effects are shown for an unrestricted control design (left), the test-based control design (right), and an unrestricted control design with placebo cases (middle). Red bars indicate significant effects ($p<0.05$ with Bonferroni correction for multiple comparisons; two-sided bootstrap) with $RR \geq 1.1$ or $RR \leq 0.9$.

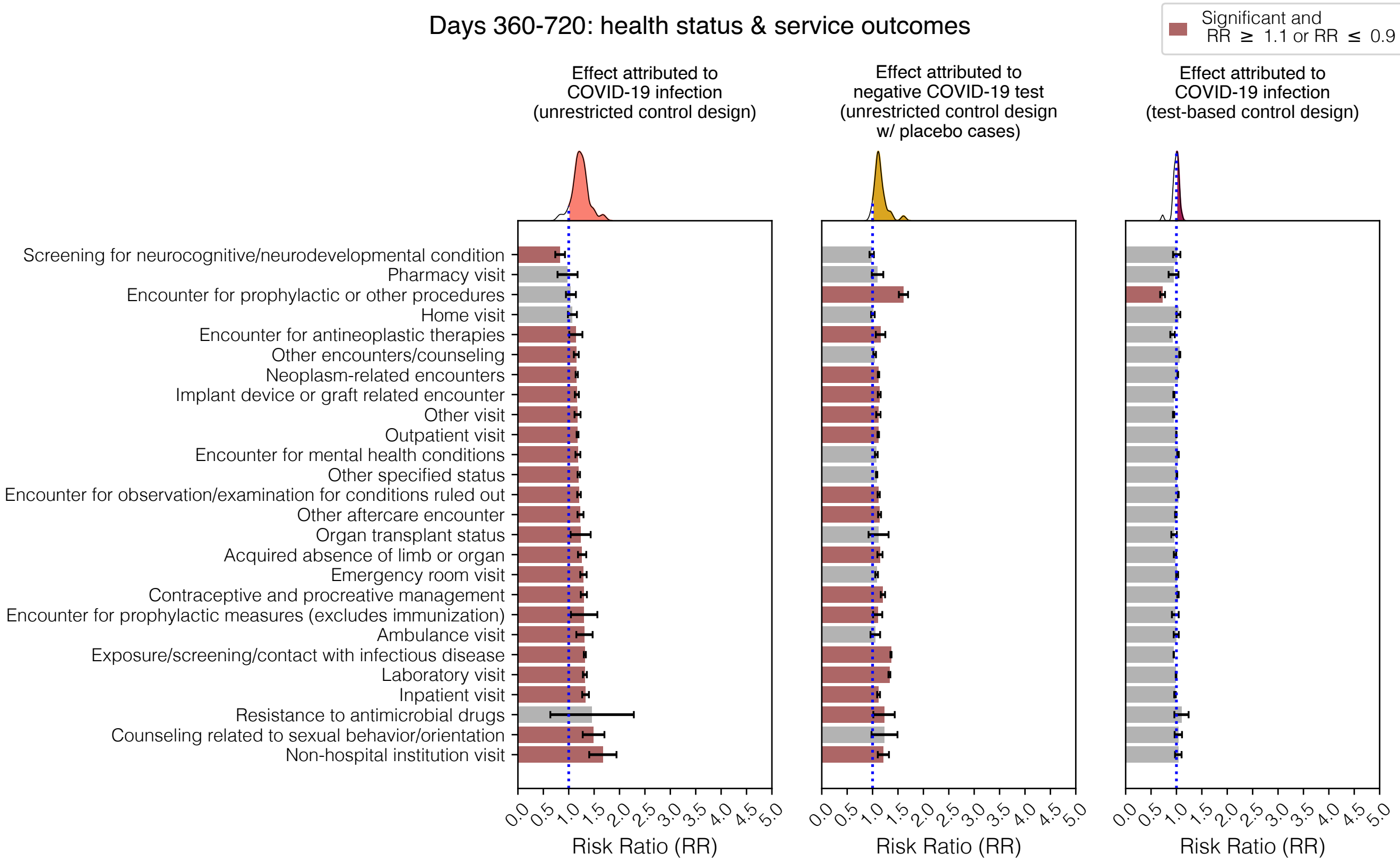


**Fig. S44.** Risk ratios for all outcomes related to health status & service on days 360–720 post-enrollment. Effects are shown for an unrestricted control design (left), the test-based control design (right), and an unrestricted control design with placebo cases (middle). Red bars indicate significant effects ($p<0.05$ with Bonferroni correction for multiple comparisons; two-sided bootstrap) with $RR\geq1.1$ or $RR\leq0.9$.

 

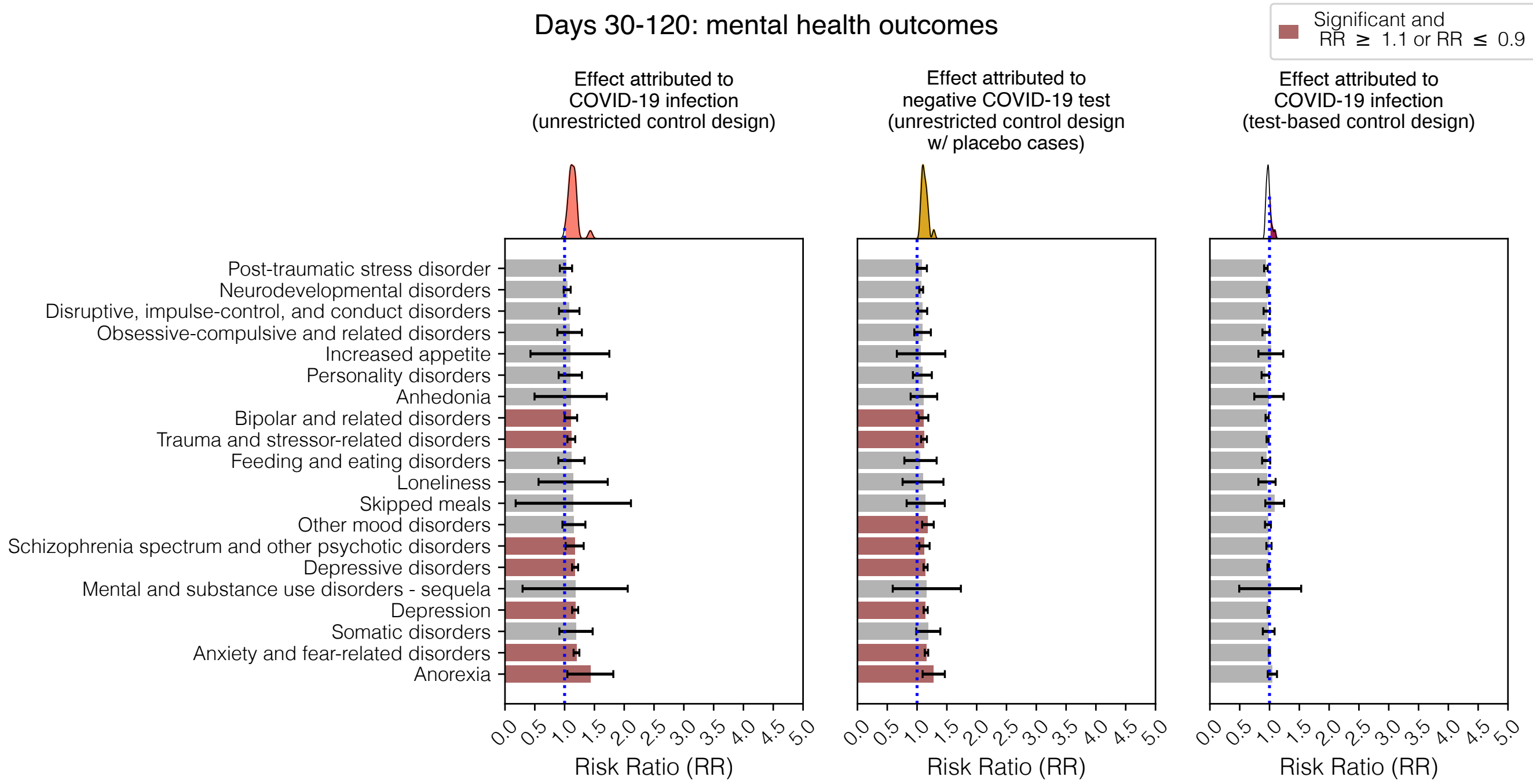


**Fig. S45.** Risk ratios for all outcomes related to mental health on days 30–120 post-enrollment. Effects are shown for an unrestricted control design (left), the test-based control design (right), and an unrestricted control design with placebo cases (middle). Red bars indicate significant effects ($p<0.05$ with Bonferroni correction for multiple comparisons; two-sided bootstrap) with $RR \geq 1.1$ or $RR \leq 0.9$.

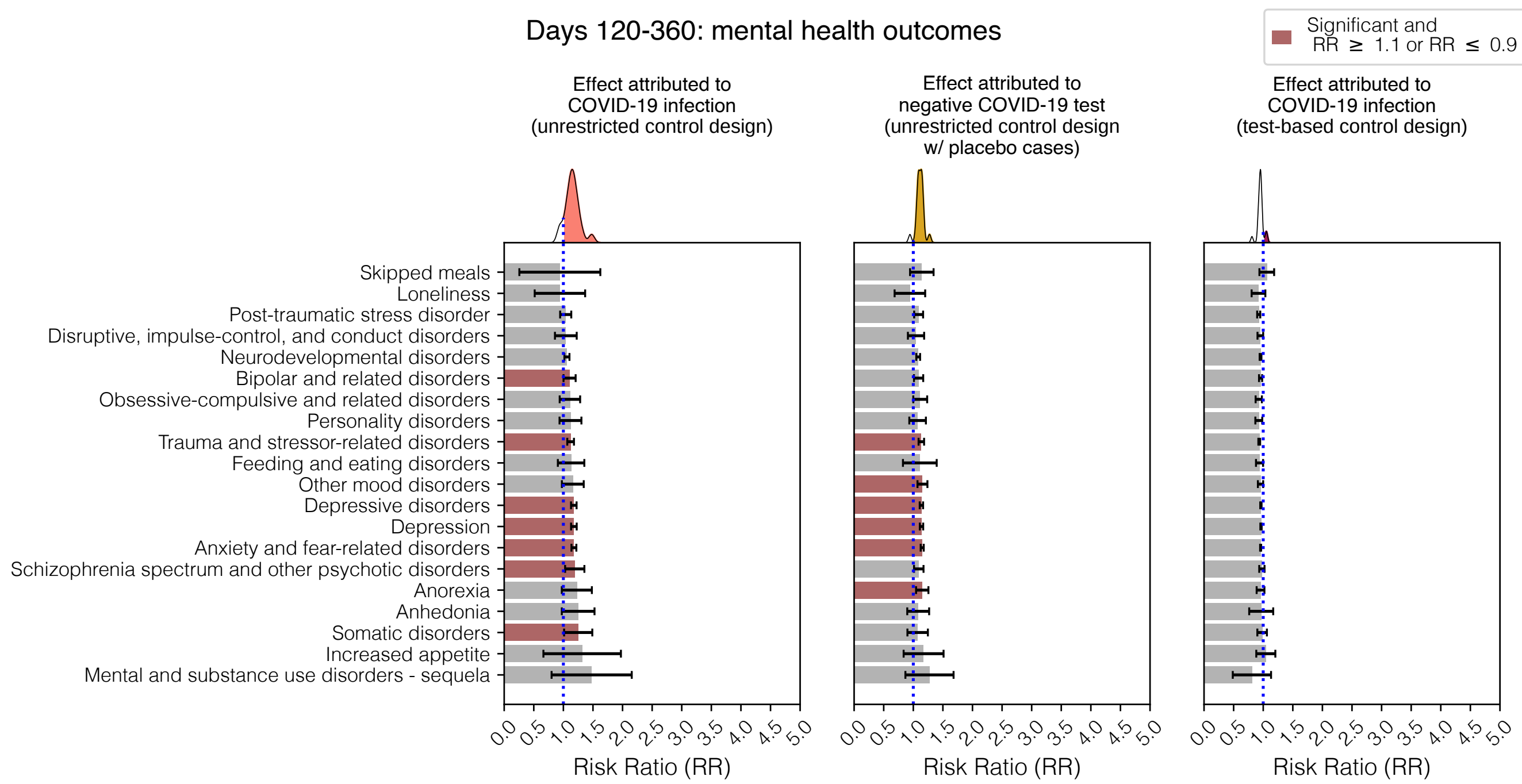


**Fig. S46.** Risk ratios for all outcomes related to mental health on days 120–360 post-enrollment. Effects are shown for an unrestricted control design (left), the test-based control design (right), and an unrestricted control design with placebo cases (middle). Red bars indicate significant effects ($p<0.05$ with Bonferroni correction for multiple comparisons; two-sided bootstrap) with RR$\geq$1.1 or RR$\leq$0.9.

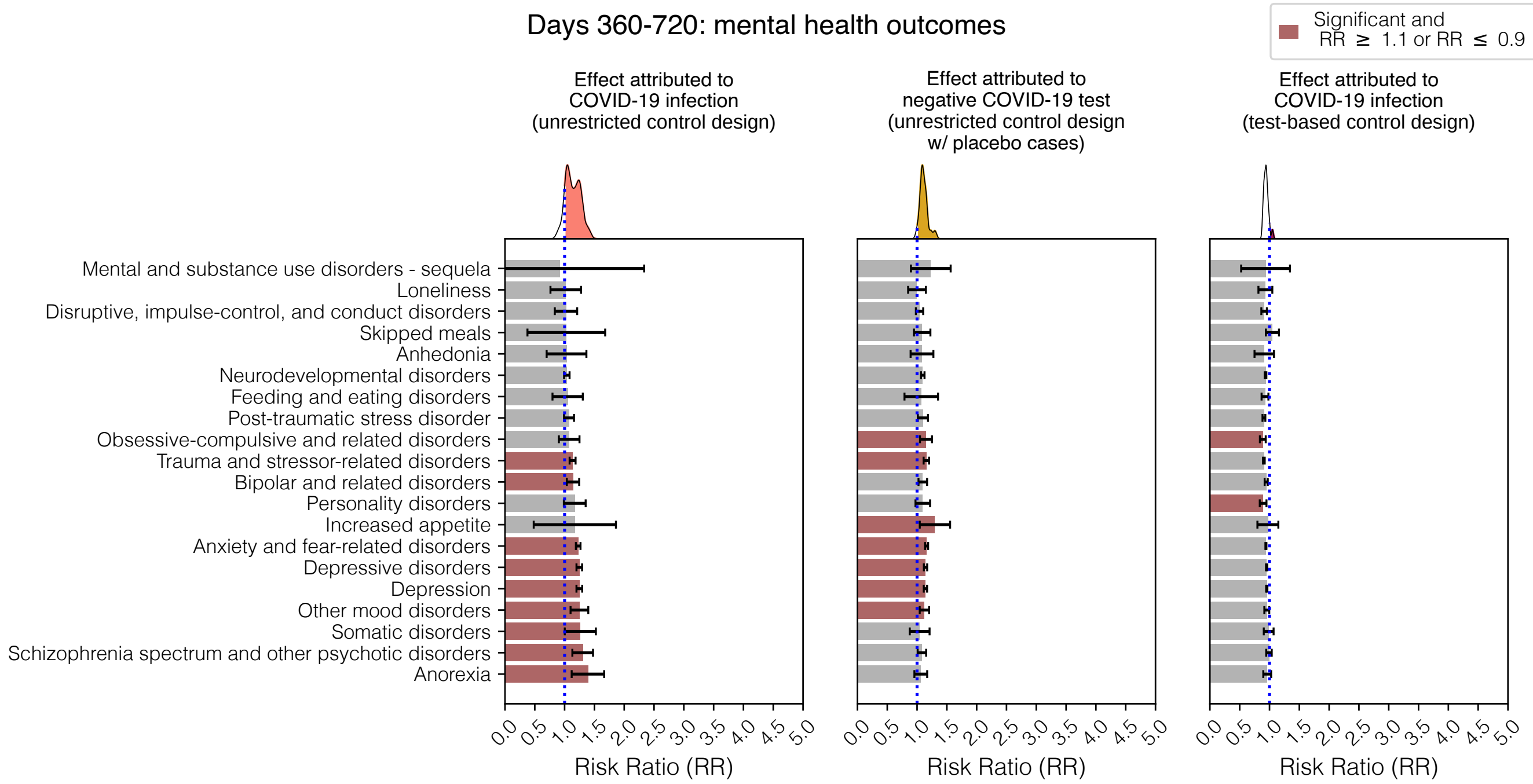


**Fig. S47.** Risk ratios for all outcomes related to mental health on days 360–720 post-enrollment. Effects are shown for an unrestricted control design (left), the test-based control design (right), and an unrestricted control design with placebo cases (middle). Red bars indicate significant effects ($p<0.05$ with Bonferroni correction for multiple comparisons; two-sided bootstrap) with $RR \geq 1.1$ or $RR \leq 0.9$.

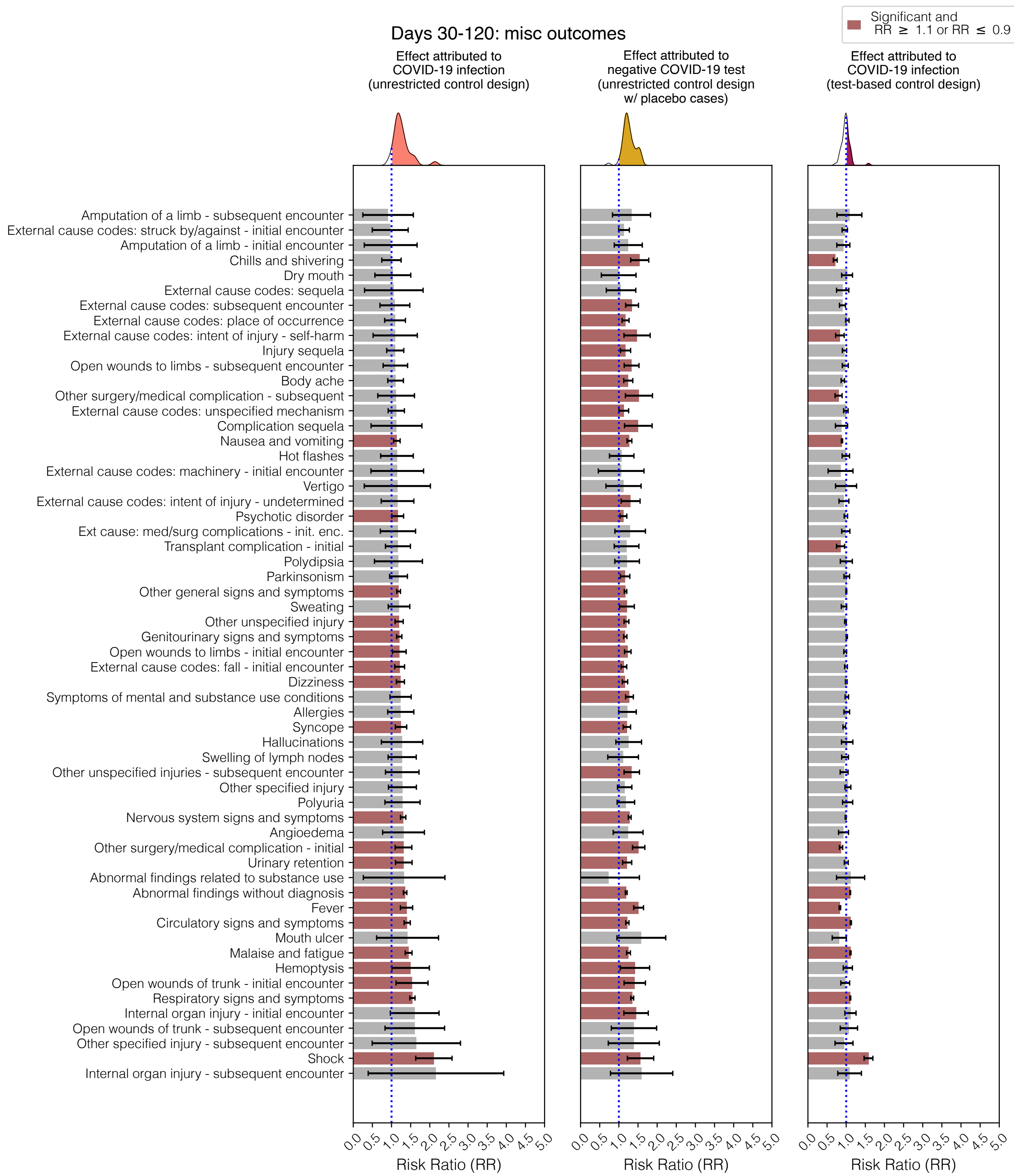


**Fig. S48.** Risk ratios for all miscellaneous outcomes on days 30–120 post-enrollment. Effects are shown for an unrestricted control design (left), the test-based control design (right), and an unrestricted control design with placebo cases (middle). Red bars indicate significant effects ($p<0.05$ with Bonferroni correction for multiple comparisons; two-sided bootstrap) with $RR\geq1.1$ or $RR\leq0.9$.

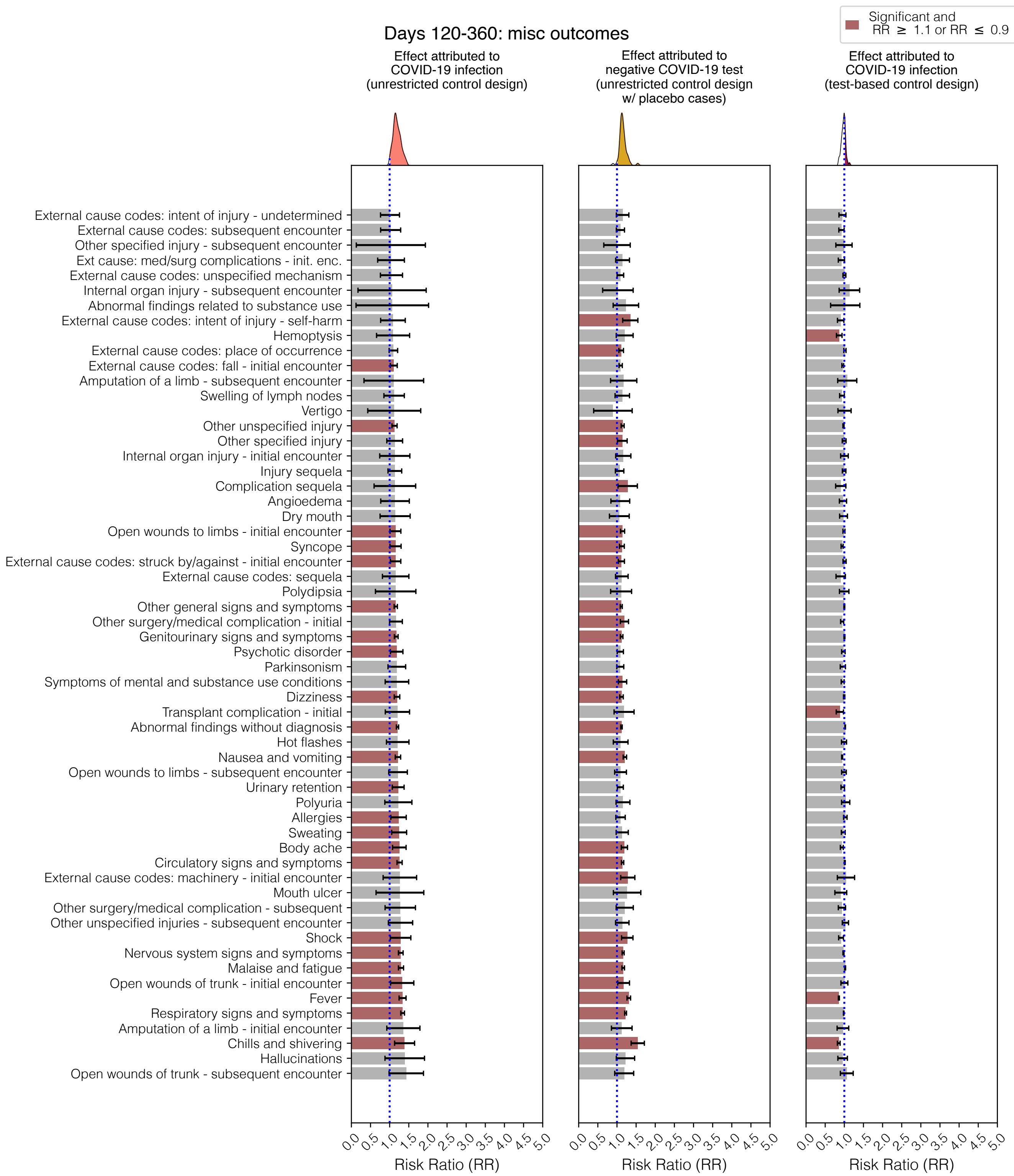


**Fig. S49.** Risk ratios for all miscellaneous outcomes on days 120–360 post-enrollment. Effects are shown for an unrestricted control design (left), the test-based control design (right), and an unrestricted control design with placebo cases (middle). Red bars indicate significant effects ($p<0.05$ with Bonferroni correction for multiple comparisons; two-sided bootstrap) with RR$\geq$1.1 or RR$\leq$0.9.

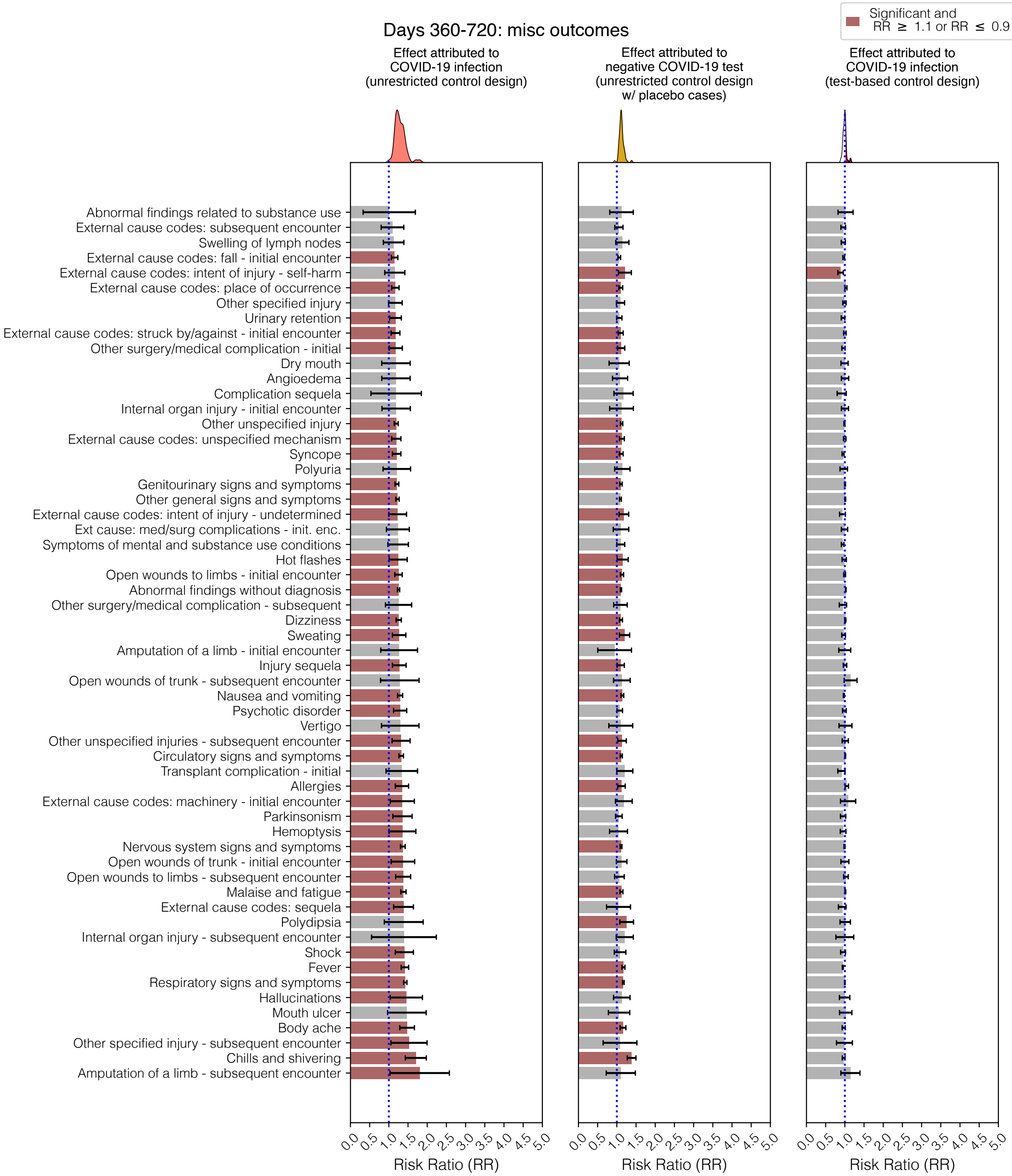


**Fig. S50.** Risk ratios for all miscellaneous outcomes on days 360–720 post-enrollment. Effects are shown for an unrestricted control design (left), the test-based control design (right), and an unrestricted control design with placebo cases (middle). Red bars indicate significant effects ($p<0.05$ with Bonferroni correction for multiple comparisons; two-sided bootstrap) with $RR \geq 1.1$ or $RR \leq 0.9$.

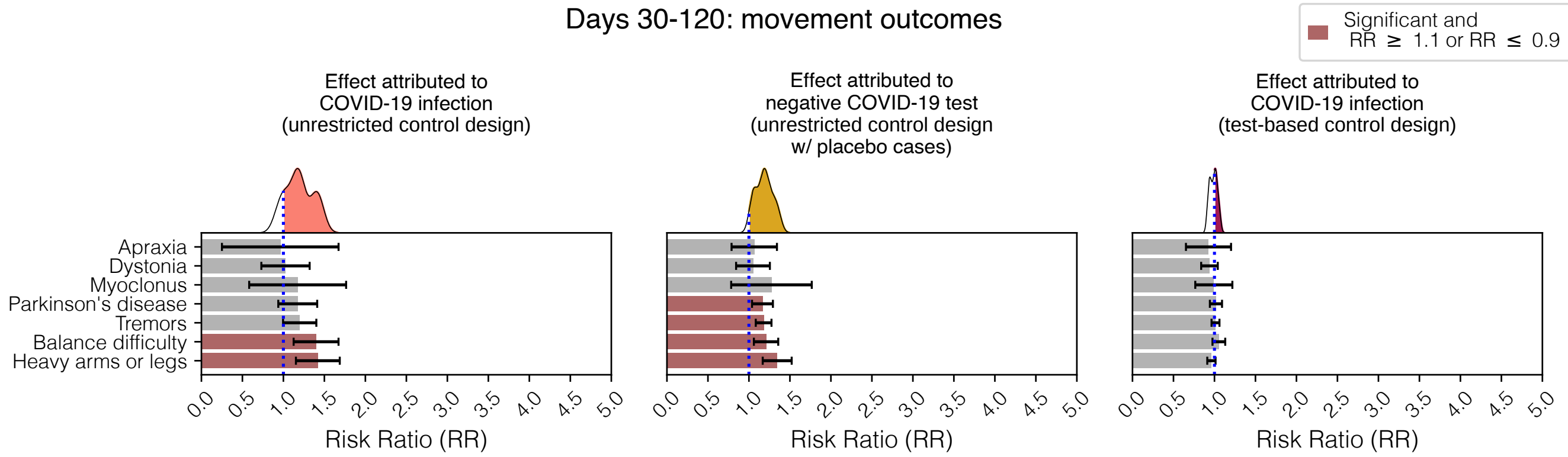


**Fig. S51.** Risk ratios for all outcomes related to movement on days 30–120 post-enrollment. Effects are shown for an unrestricted control design (left), the test-based control design (right), and an unrestricted control design with placebo cases (middle). Red bars indicate significant effects ($p<0.05$ with Bonferroni correction for multiple comparisons; two-sided bootstrap) with $RR\geq1.1$ or $RR\leq0.9$.

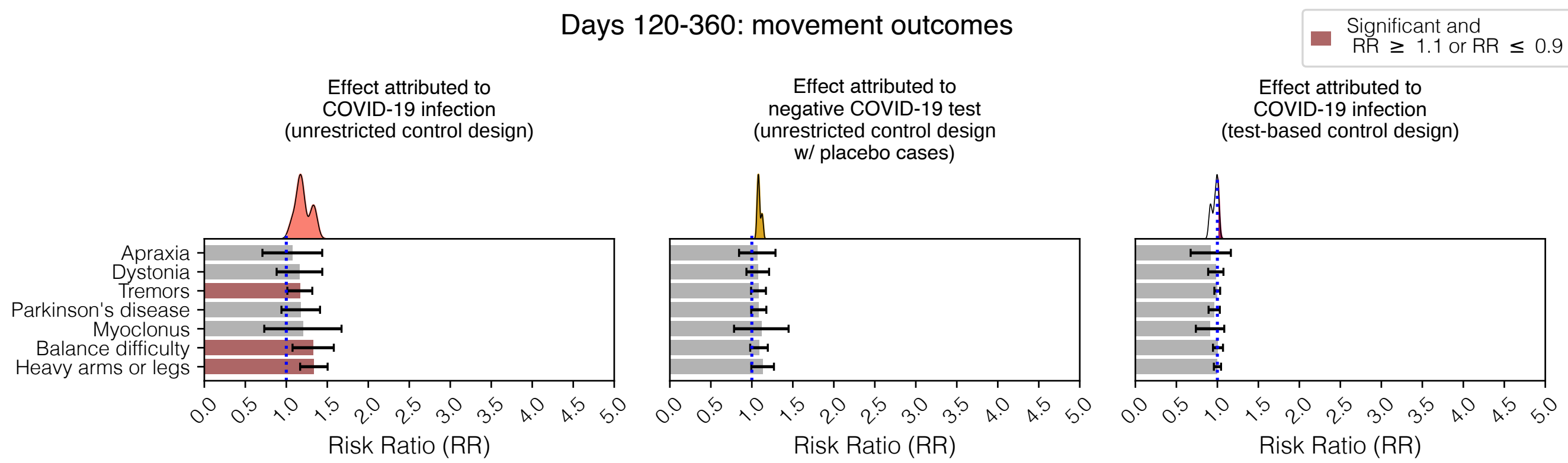


**Fig. S52.** Risk ratios for all outcomes related to movement on days 120–360 post-enrollment. Effects are shown for an unrestricted control design (left), the test-based control design (right), and an unrestricted control design with placebo cases (middle). Red bars indicate significant effects ($p<0.05$ with Bonferroni correction for multiple comparisons; two-sided bootstrap) with $RR \geq 1.1$ or $RR \leq 0.9$.

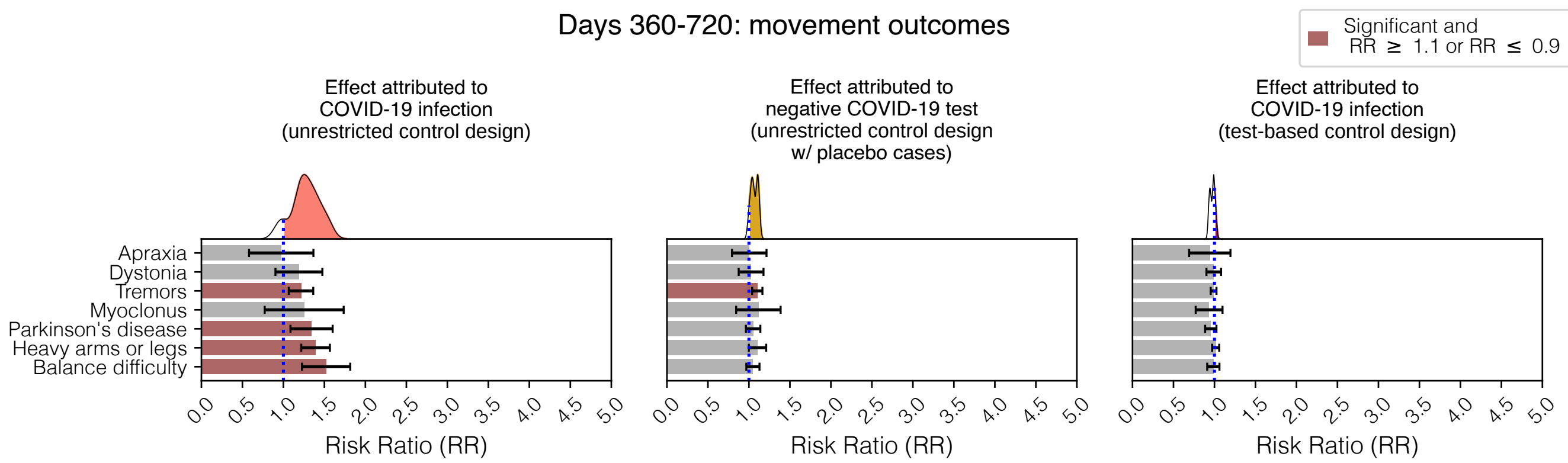


**Fig. S53.** Risk ratios for all outcomes related to movement on days 360–720 post-enrollment. Effects are shown for an unrestricted control design (left), the test-based control design (right), and an unrestricted control design with placebo cases (middle). Red bars indicate significant effects ($p<0.05$ with Bonferroni correction for multiple comparisons; two-sided bootstrap) with $RR \geq 1.1$ or $RR \leq 0.9$.

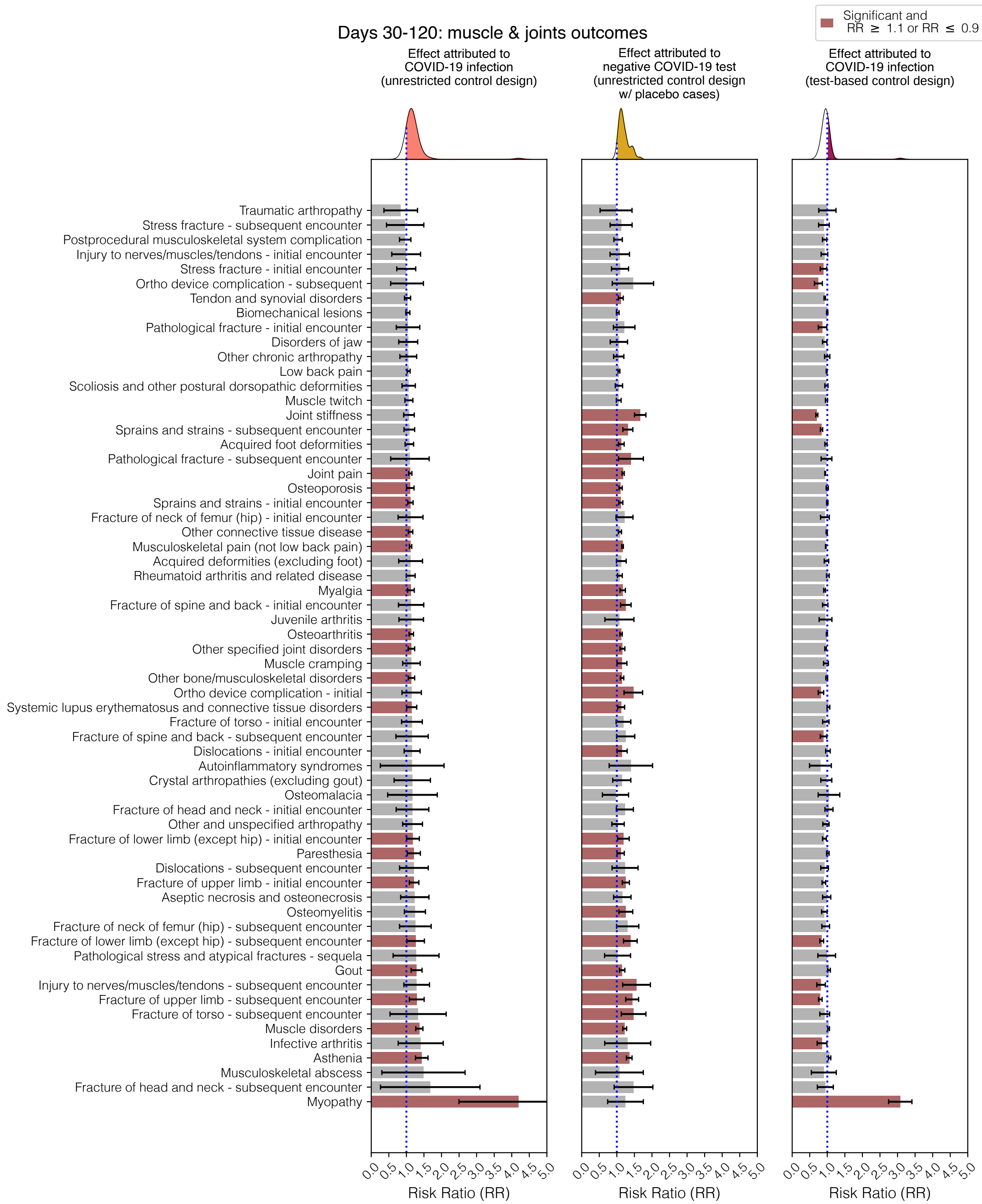


**Fig. S54.** Risk ratios for all outcomes related to muscle & joints on days 30–120 post-enrollment. Effects are shown for an unrestricted control design (left), the test-based control design (right), and an unrestricted control design with placebo cases (middle). Red bars indicate significant effects (p<0.05 with Bonferroni correction for multiple comparisons; two-sided bootstrap) with RR≥1.1 or RR≤0.9.

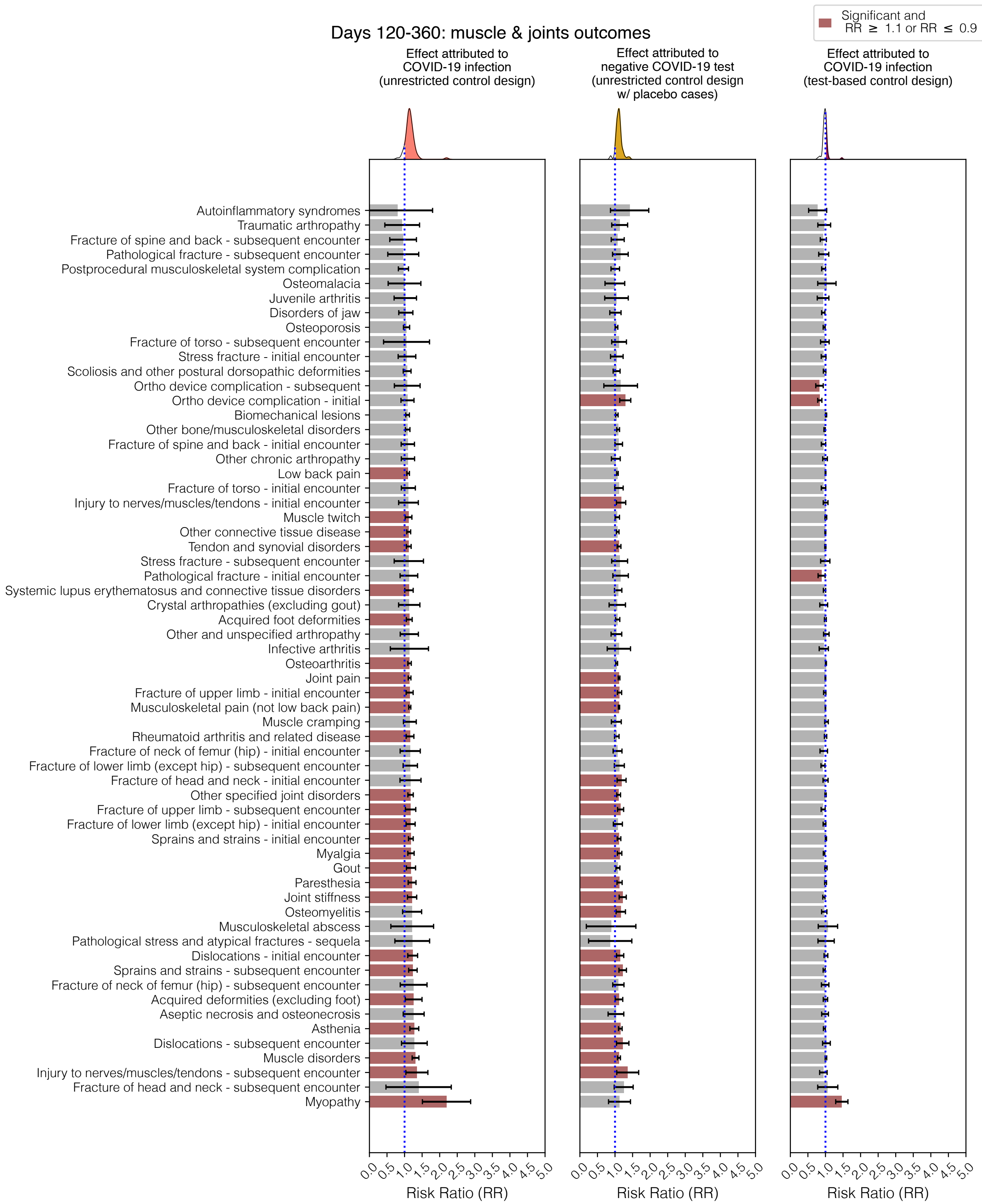


**Fig. S55.** Risk ratios for all outcomes related to muscle & joints on days 120–360 post-enrollment. Effects are shown for an unrestricted control design (left), the test-based control design (right), and an unrestricted control design with placebo cases (middle). Red bars indicate significant effects ($p<0.05$ with Bonferroni correction for multiple comparisons; two-sided bootstrap) with $RR \geq 1.1$ or $RR \leq 0.9$.

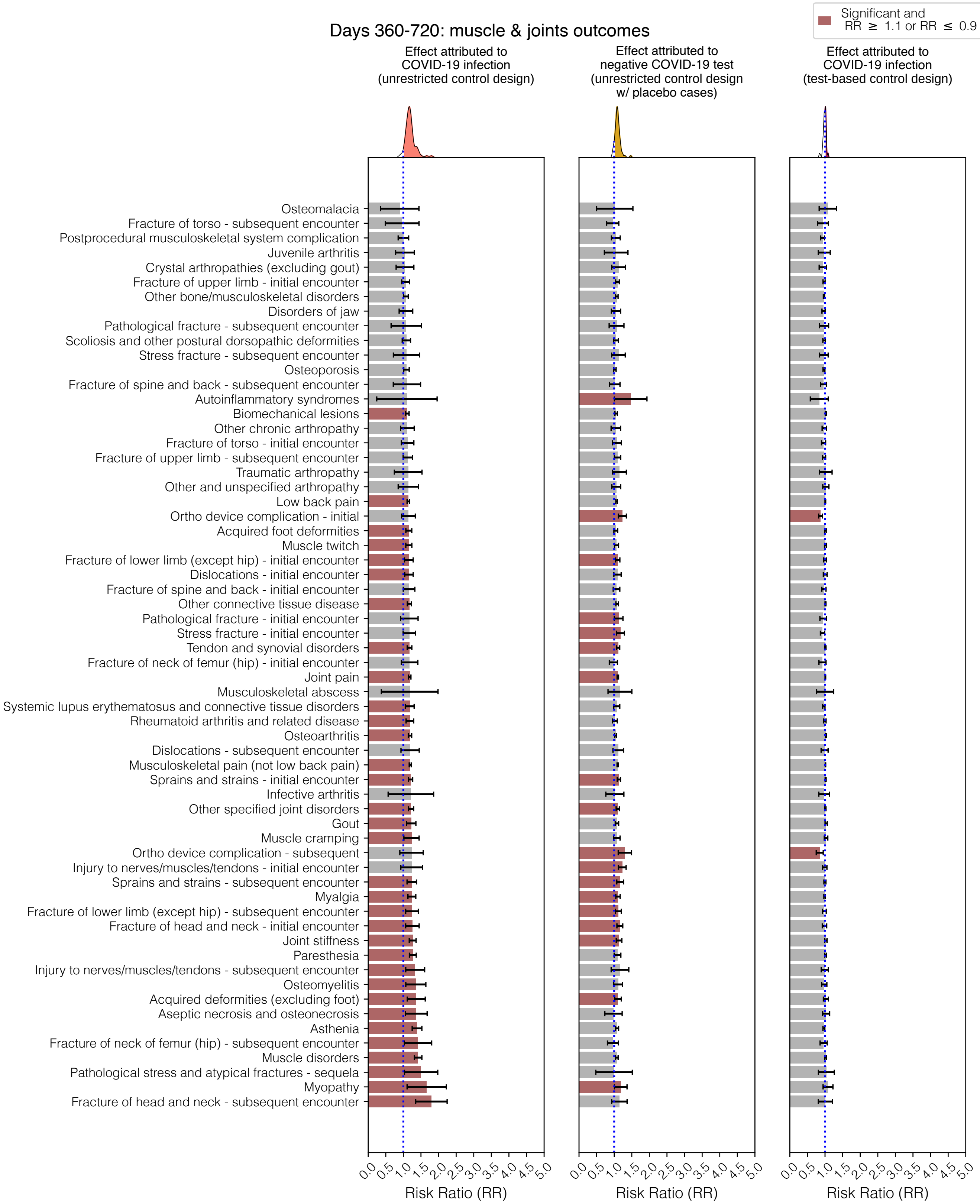


**Fig. S56.** Risk ratios for all outcomes related to muscle & joints on days 360–720 post-enrollment. Effects are shown for an unrestricted control design (left), the test-based control design (right), and an unrestricted control design with placebo cases (middle). Red bars indicate significant effects ($p<0.05$ with Bonferroni correction for multiple comparisons; two-sided bootstrap) with $RR \geq 1.1$ or $RR \leq 0.9$.

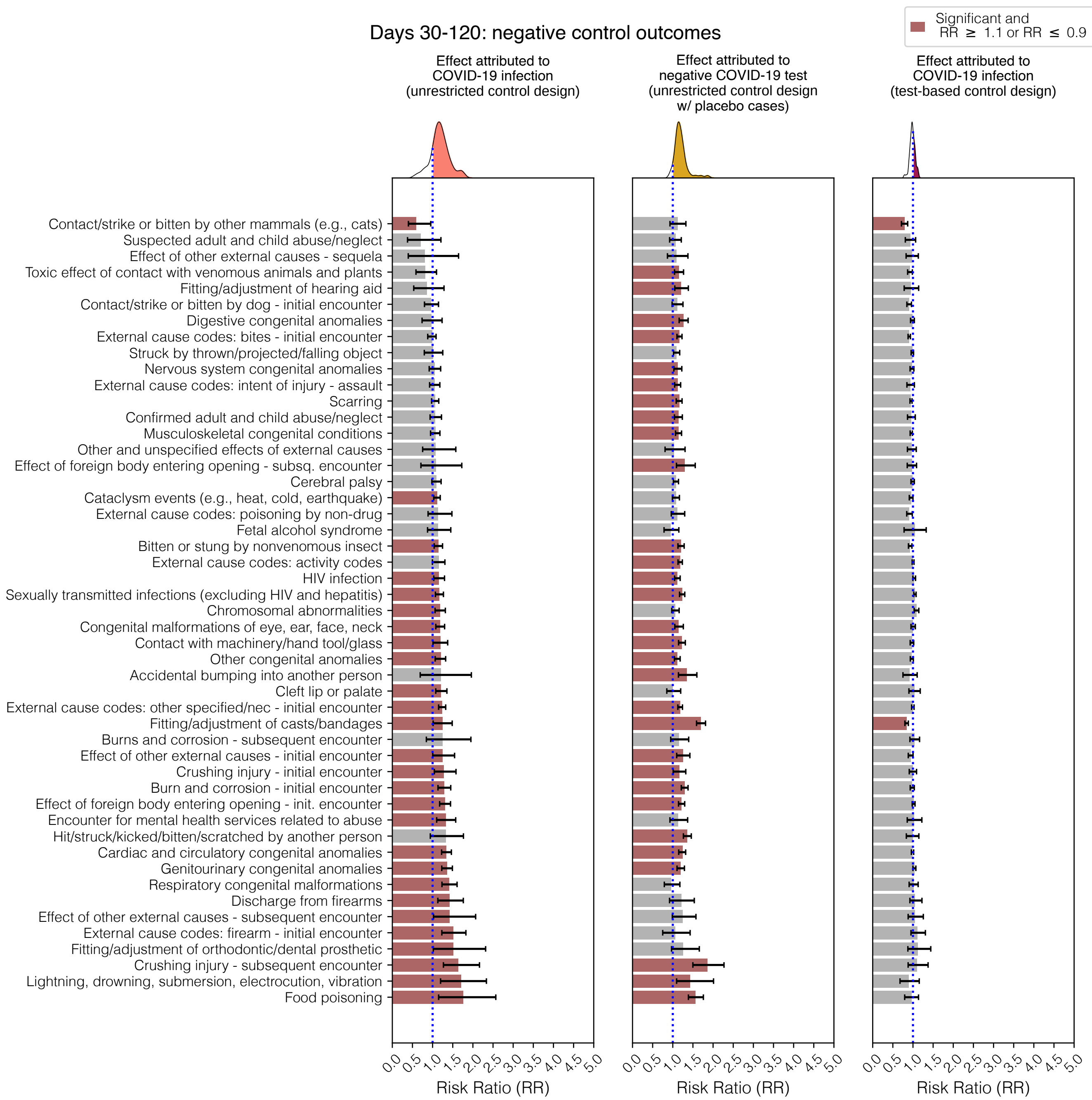


**Fig. S57.** Risk ratios for all negative control outcomes on days 30–120 post-enrollment. Effects are shown for an unrestricted control design (left), the test-based control design (right), and an unrestricted control design with placebo cases (middle). Red bars indicate significant effects ($p < 0.05$ unadjusted for multiple comparisons; two-sided bootstrap) with $RR \geq 1.1$ or $RR \leq 0.9$.

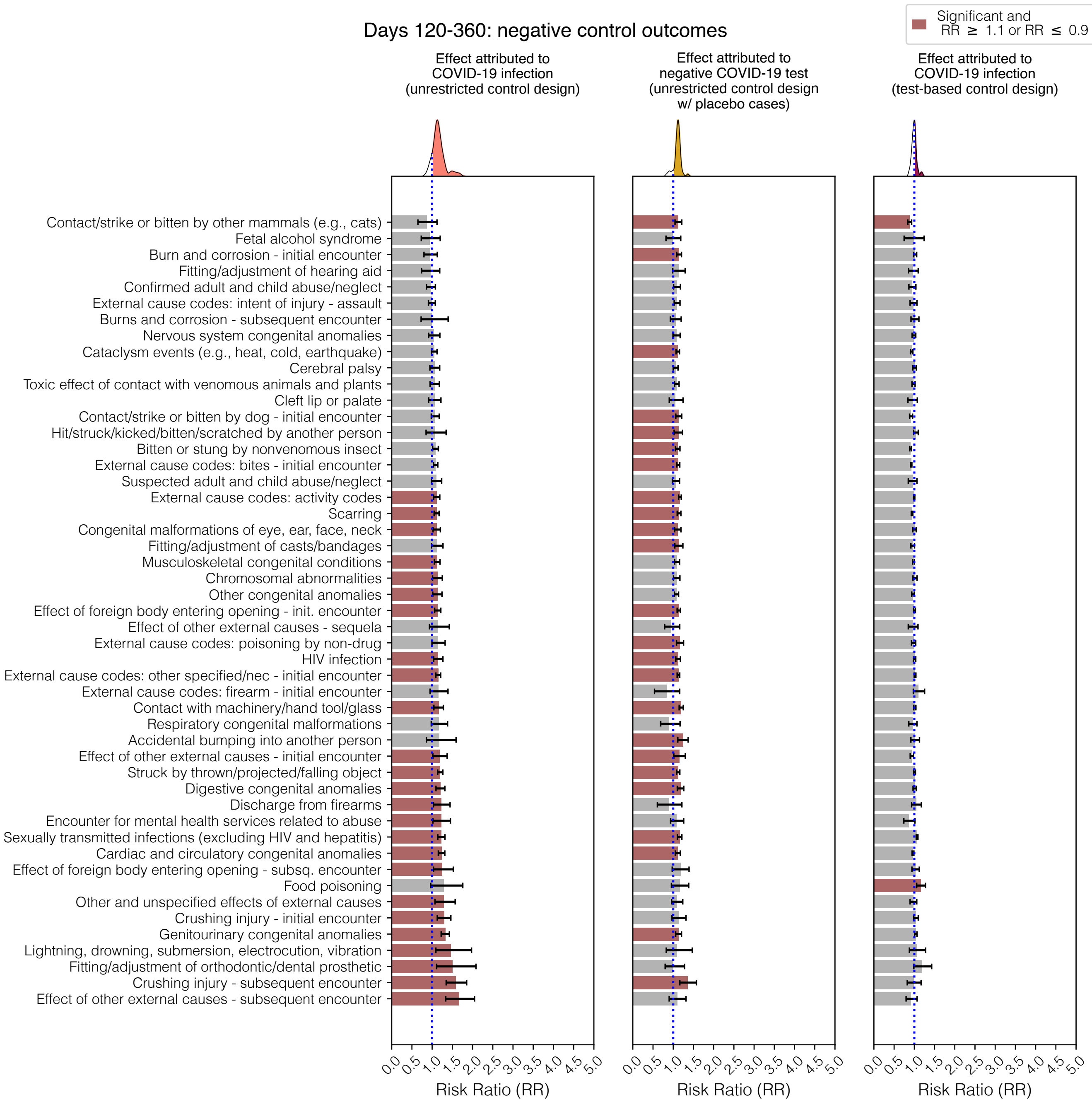


**Fig. S58.** Risk ratios for all negative control outcomes on days 120–360 post-enrollment. Effects are shown for an unrestricted control design (left), the test-based control design (right), and an unrestricted control design with placebo cases (middle). Red bars indicate significant effects ($p<0.05$ unadjusted for multiple comparisons; two-sided bootstrap) with $RR \geq 1.1$ or $RR \leq 0.9$.

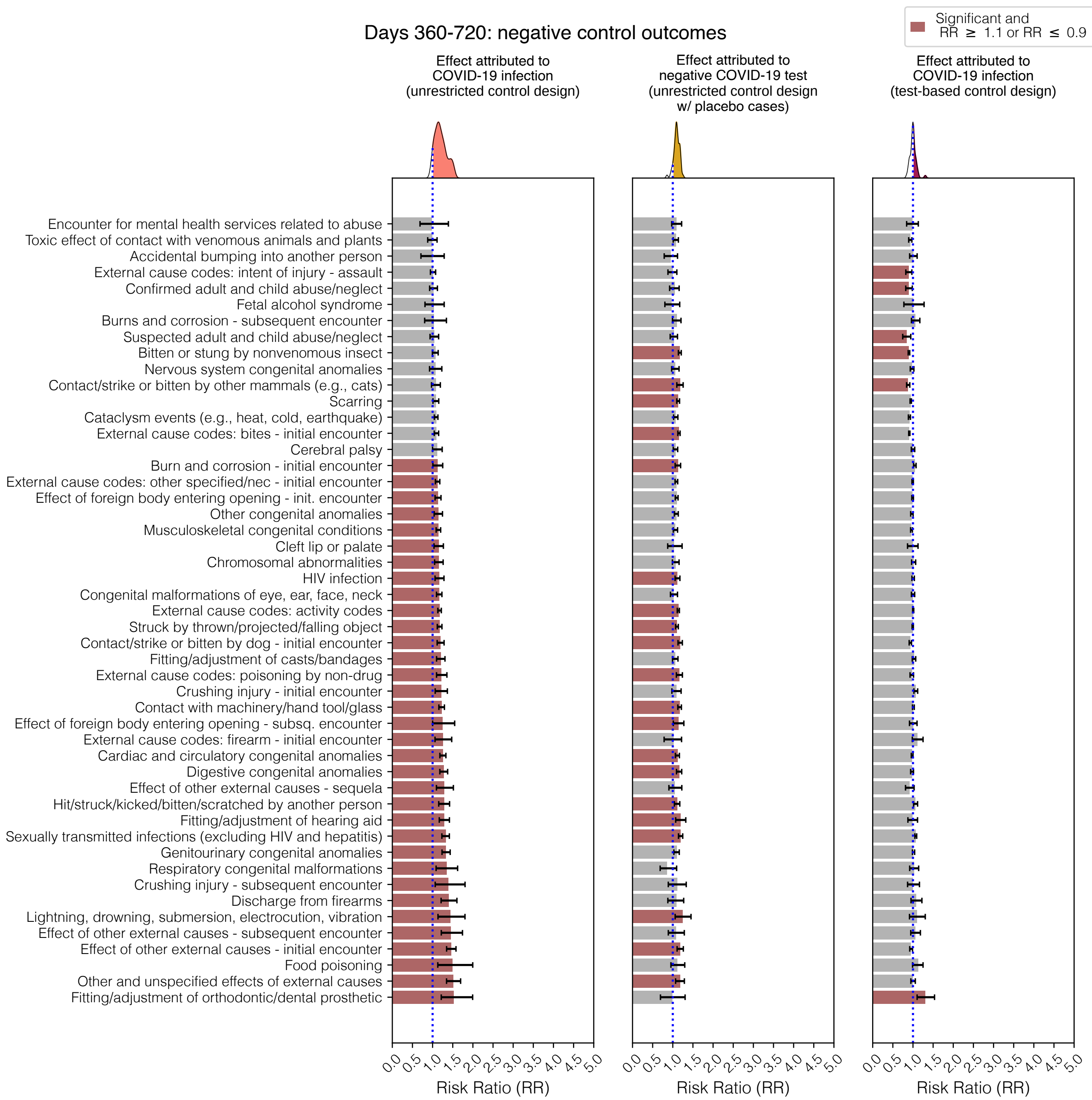


**Fig. S59.** Risk ratios for all negative control outcomes on days 360–720 post-enrollment. Effects are shown for an unrestricted control design (left), the test-based control design (right), and an unrestricted control design with placebo cases (middle). Red bars indicate significant effects ($p<0.05$ unadjusted for multiple comparisons; two-sided bootstrap) with $RR \geq 1.1$ or $RR \leq 0.9$.

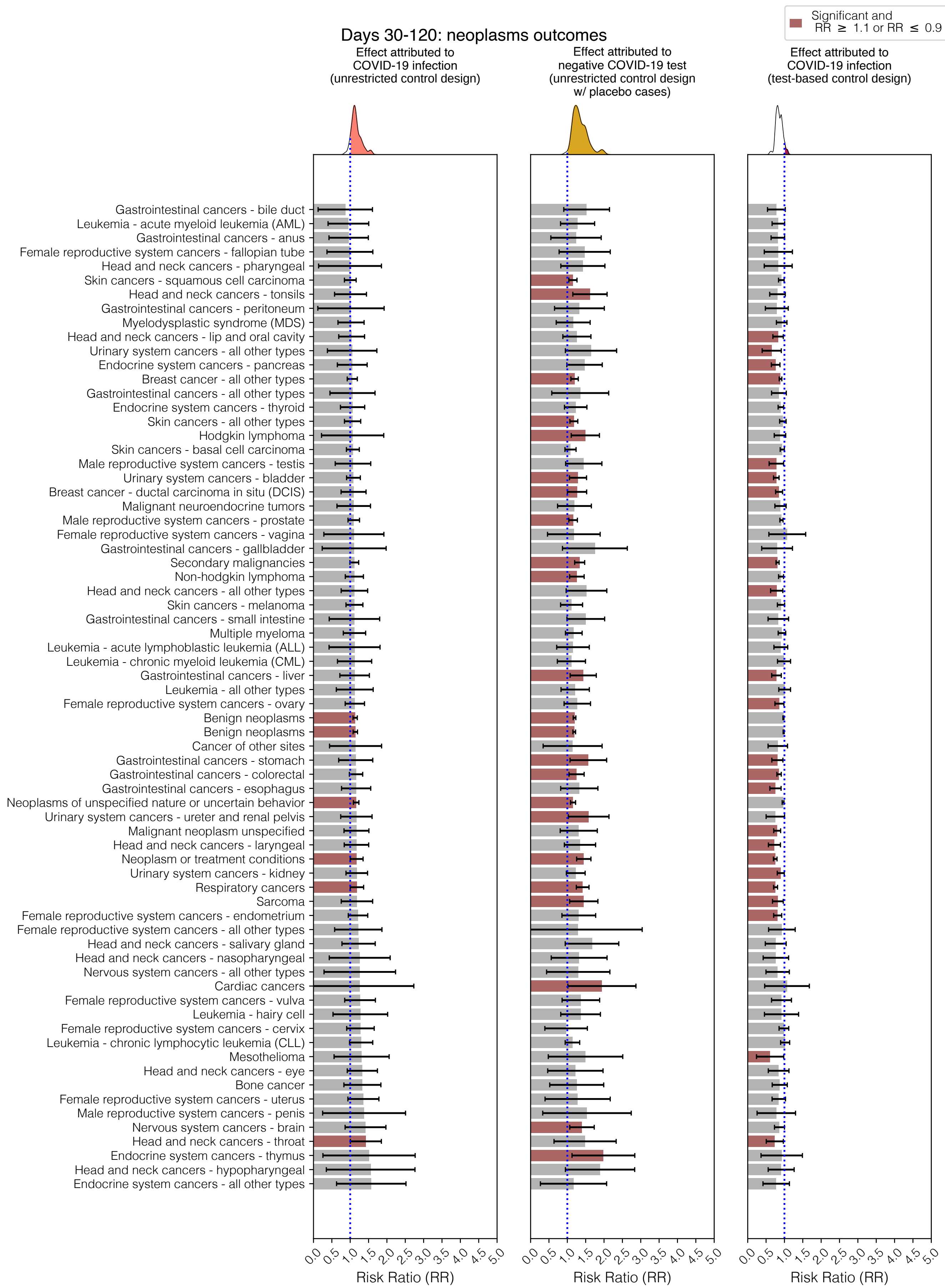


**Fig. S60.** Risk ratios for all outcomes related to neoplasms on days 30–120 post-enrollment. Effects are shown for an unrestricted control design (left), the test-based control design (right), and an unrestricted control design with placebo cases (middle). Red bars indicate significant effects ($p < 0.05$ with Bonferroni correction for multiple comparisons; two-sided bootstrap) with $RR \geq 1.1$ or $RR \leq 0.9$.

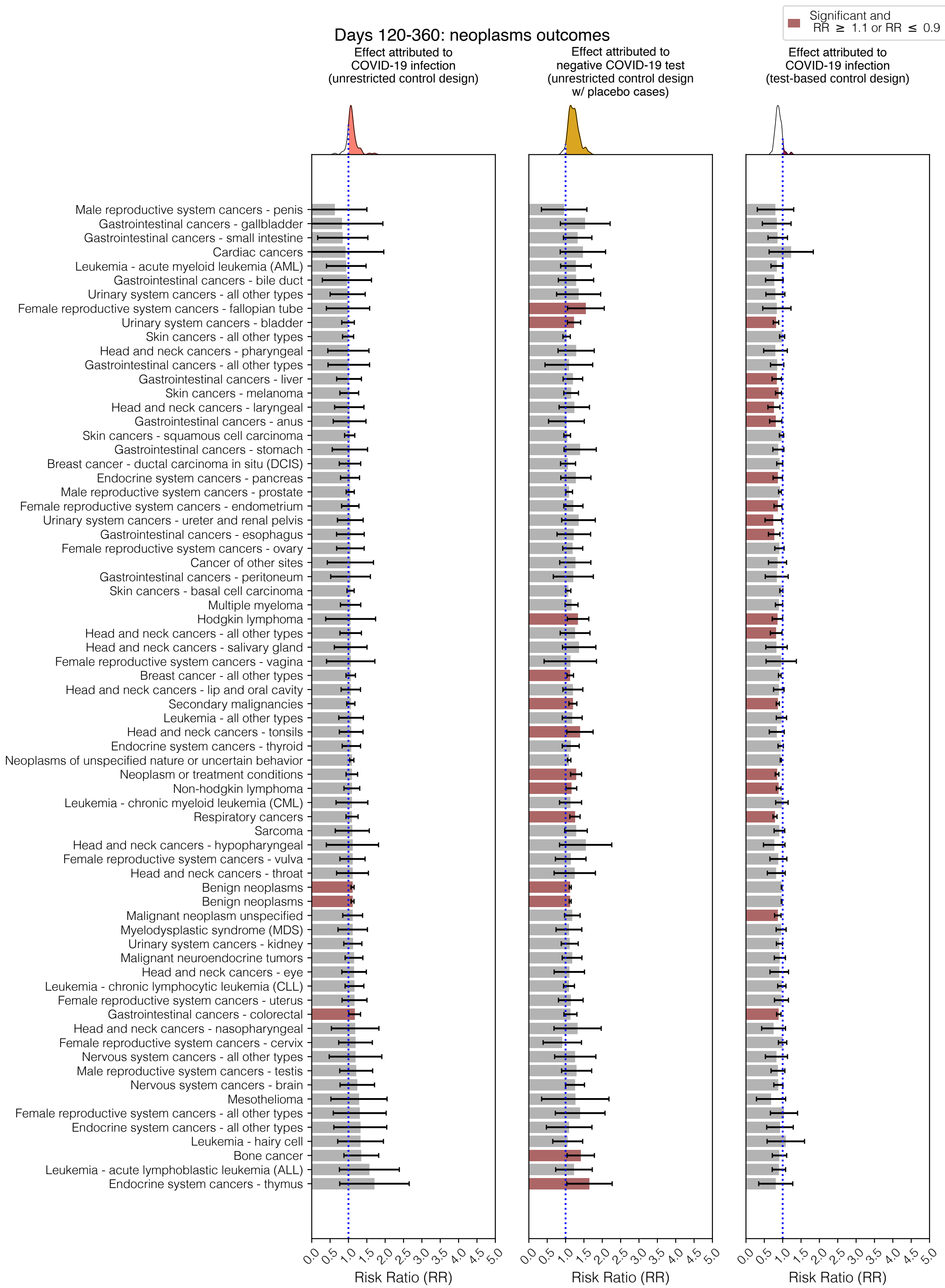


**Fig. S61.** Risk ratios for all outcomes related to neoplasms on days 120–360 post-enrollment. Effects are shown for an unrestricted control design (left), the test-based control design (right), and an unrestricted control design with placebo cases (middle). Red bars indicate significant effects ($p<0.05$ with Bonferroni correction for multiple comparisons; two-sided bootstrap) with $RR \geq 1.1$ or $RR \leq 0.9$.

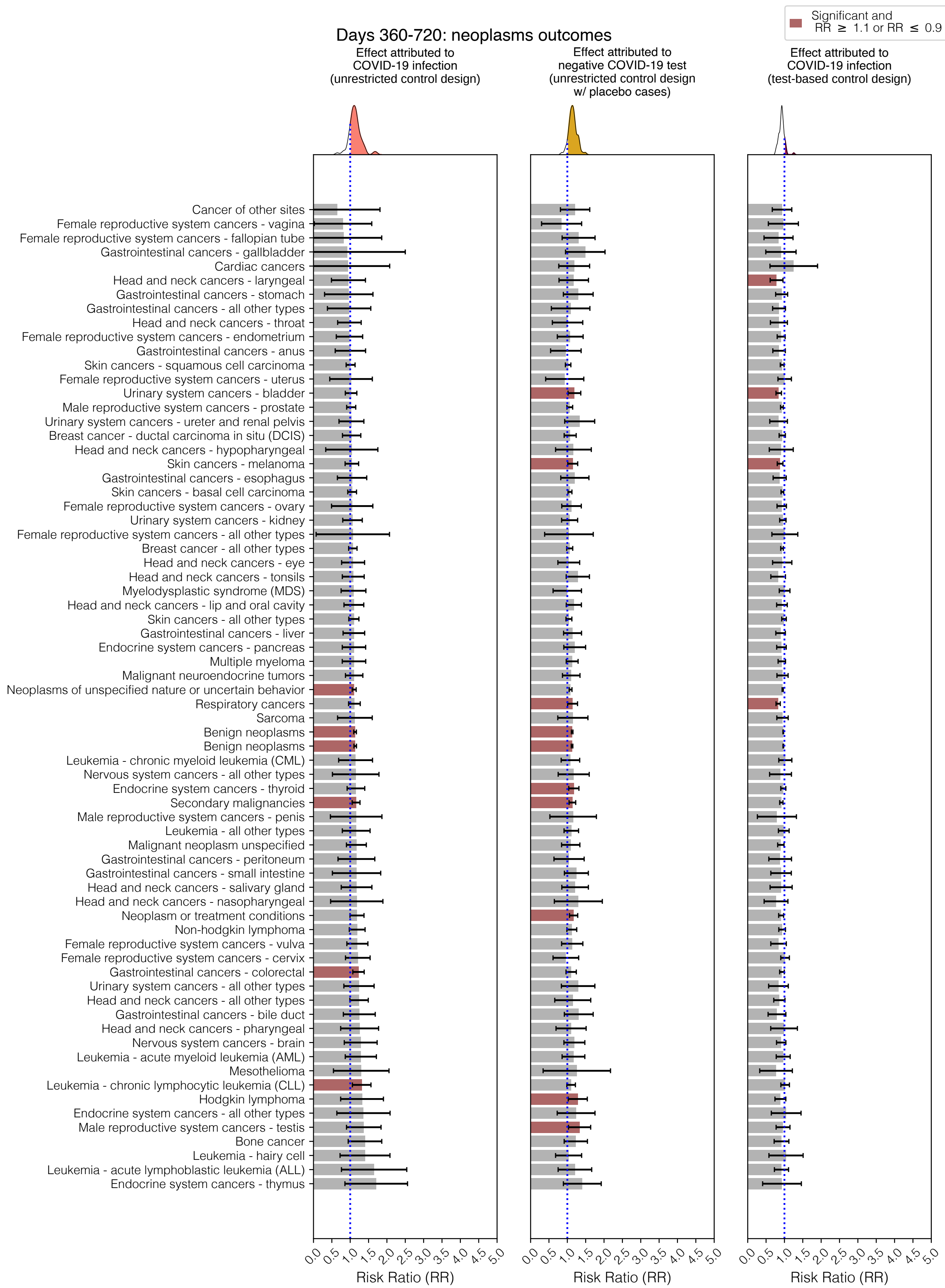


**Fig. S62.** Risk ratios for all outcomes related to neoplasms on days 360–720 post-enrollment. Effects are shown for an unrestricted control design (left), the test-based control design (right), and an unrestricted control design with placebo cases (middle). Red bars indicate significant effects ($p<0.05$ with Bonferroni correction for multiple comparisons; two-sided bootstrap) with $RR\geq1.1$ or $RR\leq0.9$.

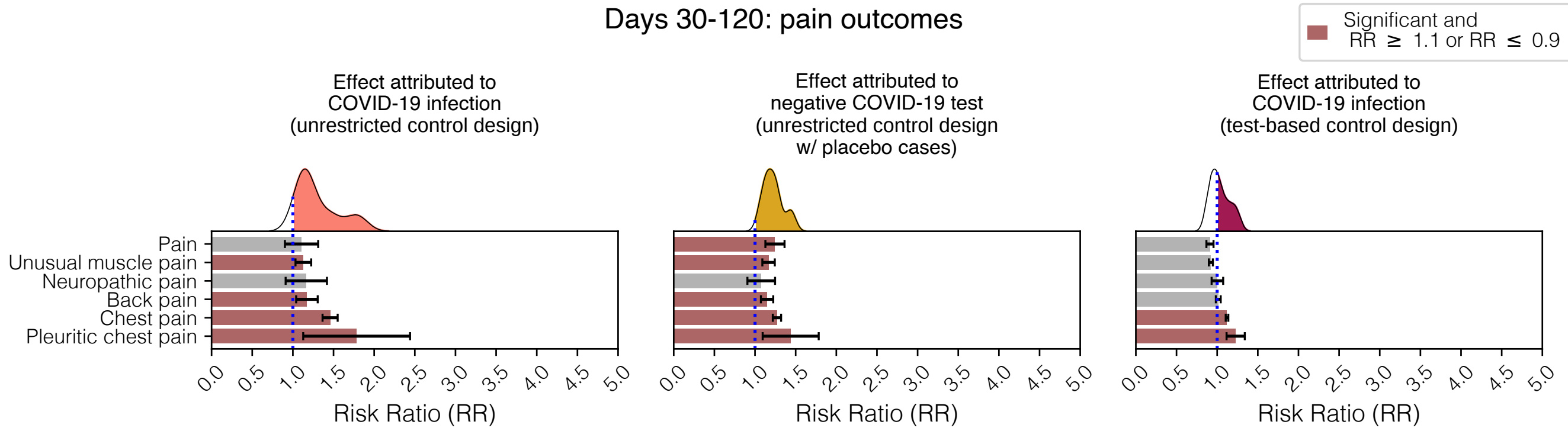


**Fig. S63.** Risk ratios for all outcomes related to pain on days 30–120 post-enrollment. Effects are shown for an unrestricted control design (left), the test-based control design (right), and an unrestricted control design with placebo cases (middle). Red bars indicate significant effects ($p<0.05$ with Bonferroni correction for multiple comparisons; two-sided bootstrap) with $RR\geq1.1$ or $RR\leq0.9$.

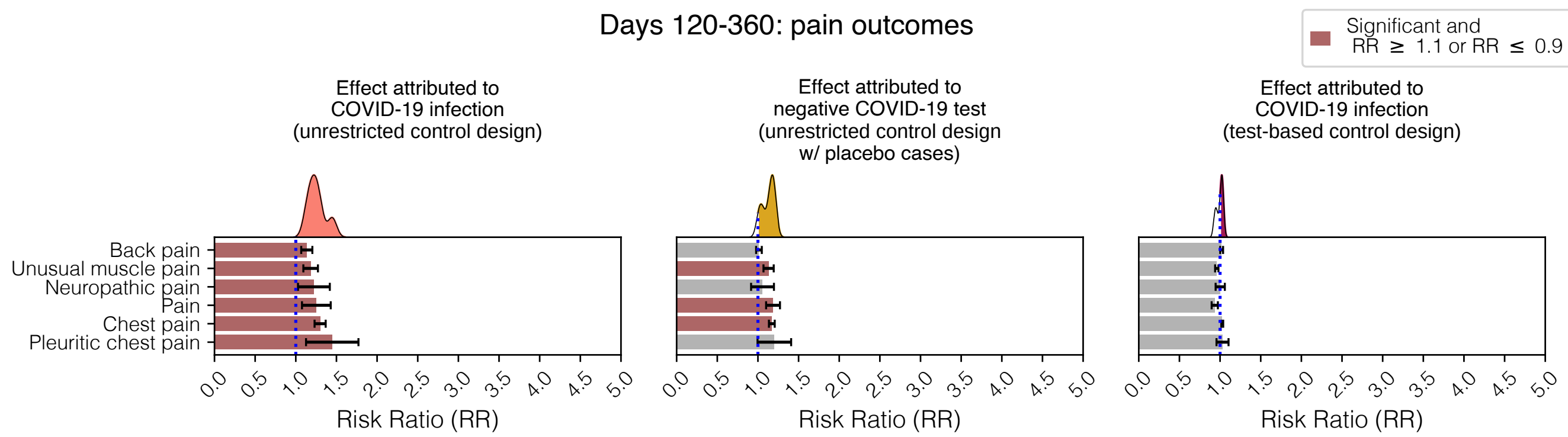


**Fig. S64.** Risk ratios for all outcomes related to pain on days 120–360 post-enrollment. Effects are shown for an unrestricted control design (left), the test-based control design (right), and an unrestricted control design with placebo cases (middle). Red bars indicate significant effects ($p<0.05$ with Bonferroni correction for multiple comparisons; two-sided bootstrap) with $RR \geq 1.1$ or $RR \leq 0.9$.

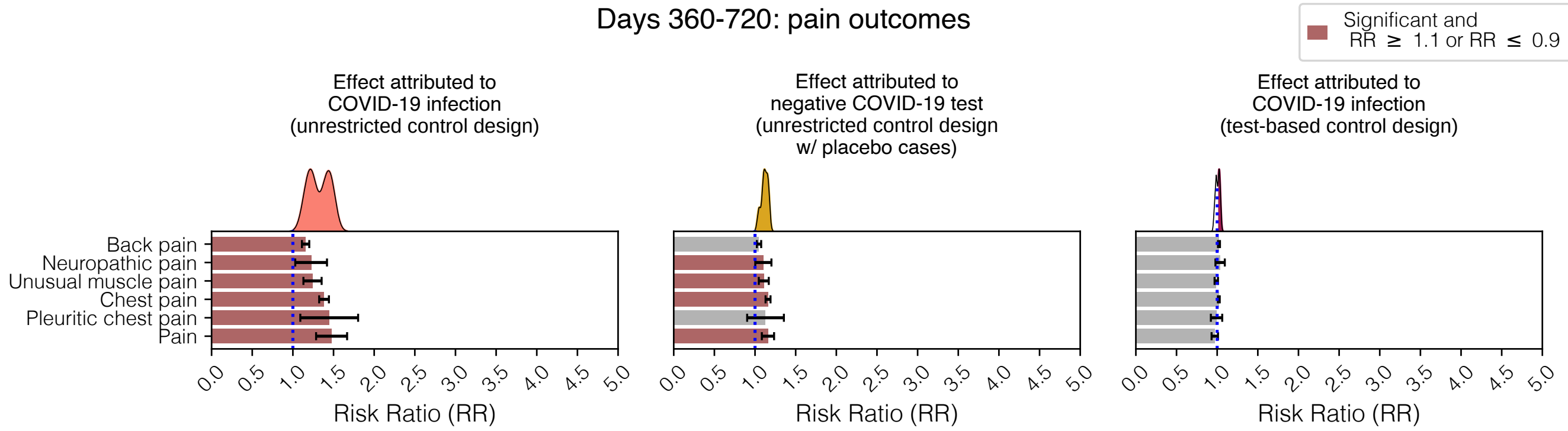


**Fig. S65.** Risk ratios for all outcomes related to pain on days 360–720 post-enrollment. Effects are shown for an unrestricted control design (left), the test-based control design (right), and an unrestricted control design with placebo cases (middle). Red bars indicate significant effects ($p<0.05$ with Bonferroni correction for multiple comparisons; two-sided bootstrap) with RR$\geq$1.1 or RR$\leq$0.9.

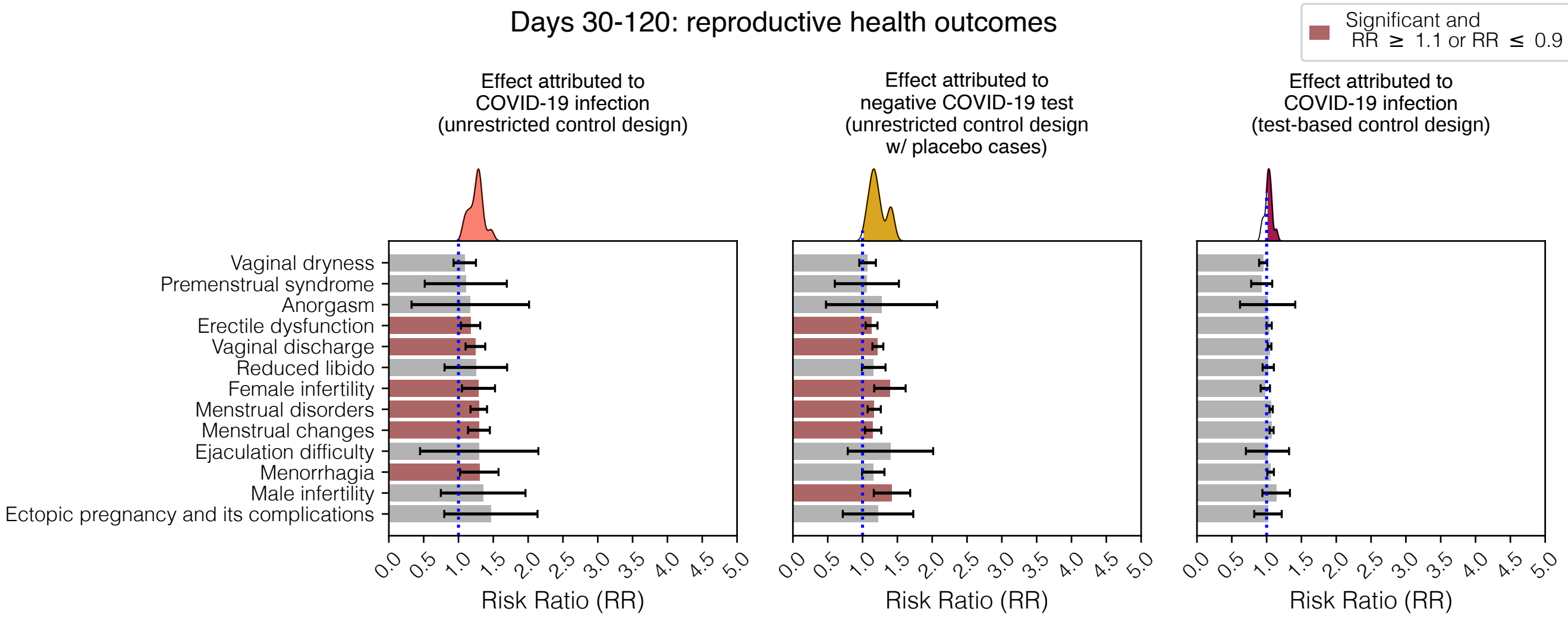


**Fig. S66.** Risk ratios for all outcomes related to reproductive health on days 30–120 post-enrollment. Effects are shown for an unrestricted control design (left), the test-based control design (right), and an unrestricted control design with placebo cases (middle). Red bars indicate significant effects ($p<0.05$ with Bonferroni correction for multiple comparisons; two-sided bootstrap) with $RR\geq1.1$ or $RR\leq0.9$.

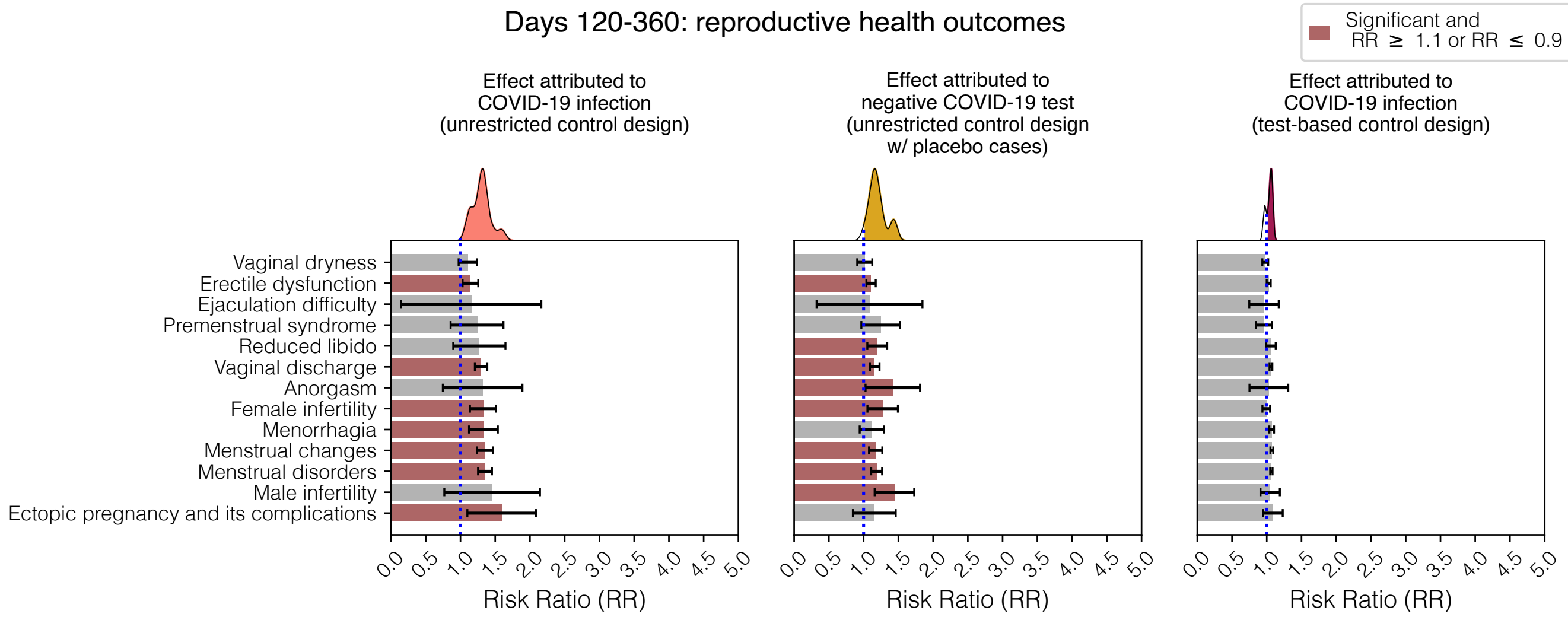


**Fig. S67.** Risk ratios for all outcomes related to reproductive health on days 120–360 post-enrollment. Effects are shown for an unrestricted control design (left), the test-based control design (right), and an unrestricted control design with placebo cases (middle). Red bars indicate significant effects ($p<0.05$ with Bonferroni correction for multiple comparisons; two-sided bootstrap) with $RR \geq 1.1$ or $RR \leq 0.9$.

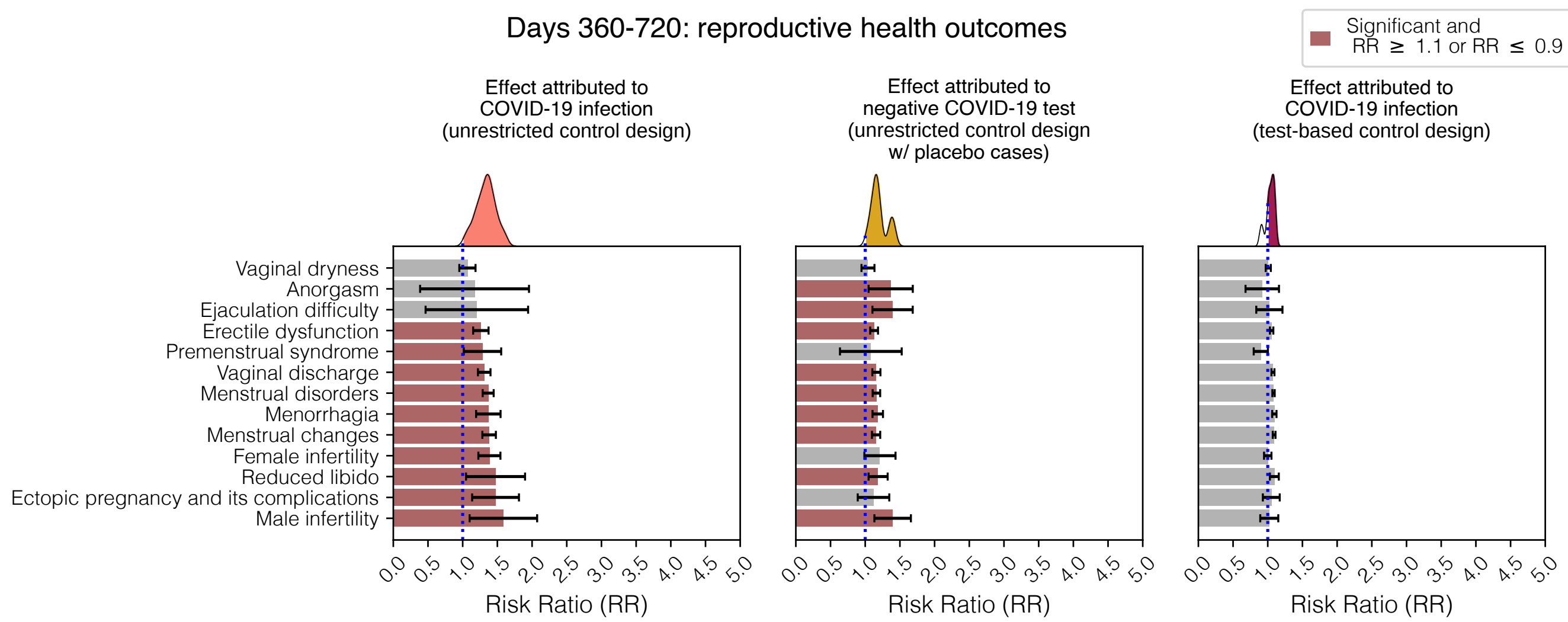


**Fig. S68.** Risk ratios for all outcomes related to reproductive health on days 360–720 post-enrollment. Effects are shown for an unrestricted control design (left), the test-based control design (right), and an unrestricted control design with placebo cases (middle). Red bars indicate significant effects ($p<0.05$ with Bonferroni correction for multiple comparisons; two-sided bootstrap) with $RR \geq 1.1$ or $RR \leq 0.9$.

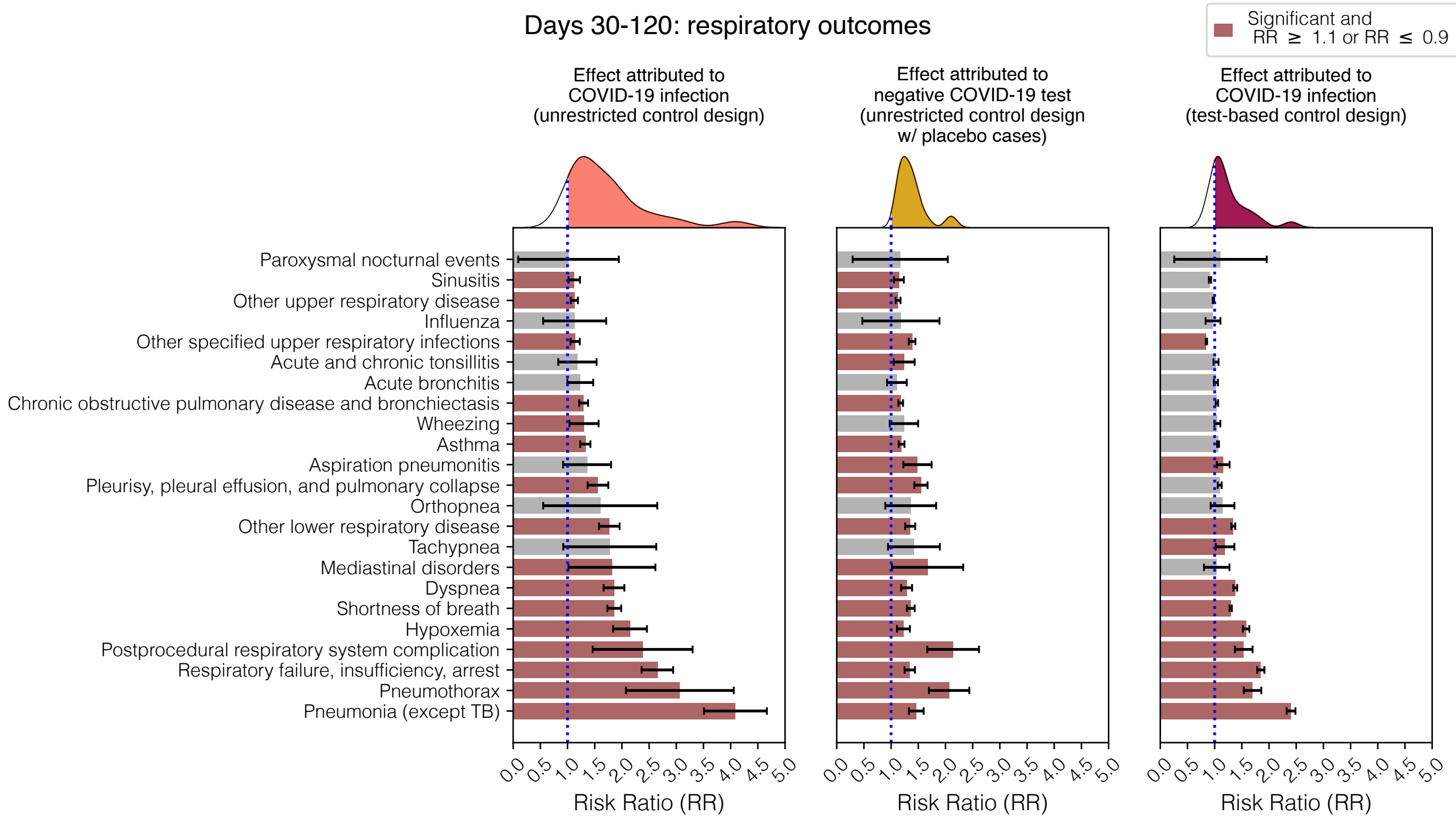


**Fig. S69.** Risk ratios for all outcomes related to the respiratory system on days 30–120 post-enrollment. Effects are shown for an unrestricted control design (left), the test-based control design (right), and an unrestricted control design with placebo cases (middle). Red bars indicate significant effects ($p<0.05$ with Bonferroni correction for multiple comparisons; two-sided bootstrap) with $RR \geq 1.1$ or $RR \leq 0.9$.

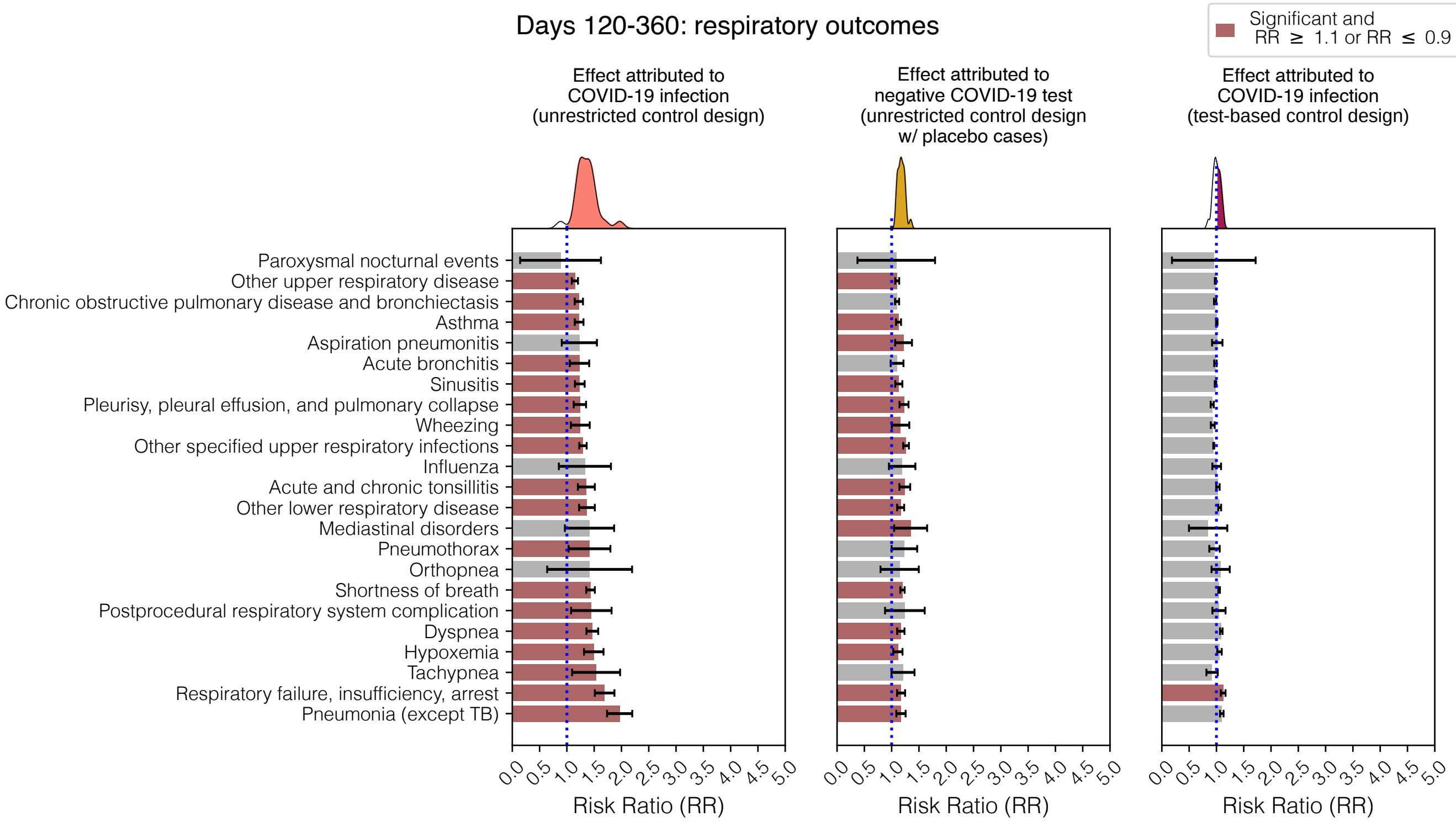


**Fig. S70.** Risk ratios for all outcomes related to the respiratory system on days 120–360 post-enrollment. Effects are shown for an unrestricted control design (left), the test-based control design (right), and an unrestricted control design with placebo cases (middle). Red bars indicate significant effects ($p<0.05$ with Bonferroni correction for multiple comparisons; two-sided bootstrap) with $RR\geq1.1$ or $RR\leq0.9$.

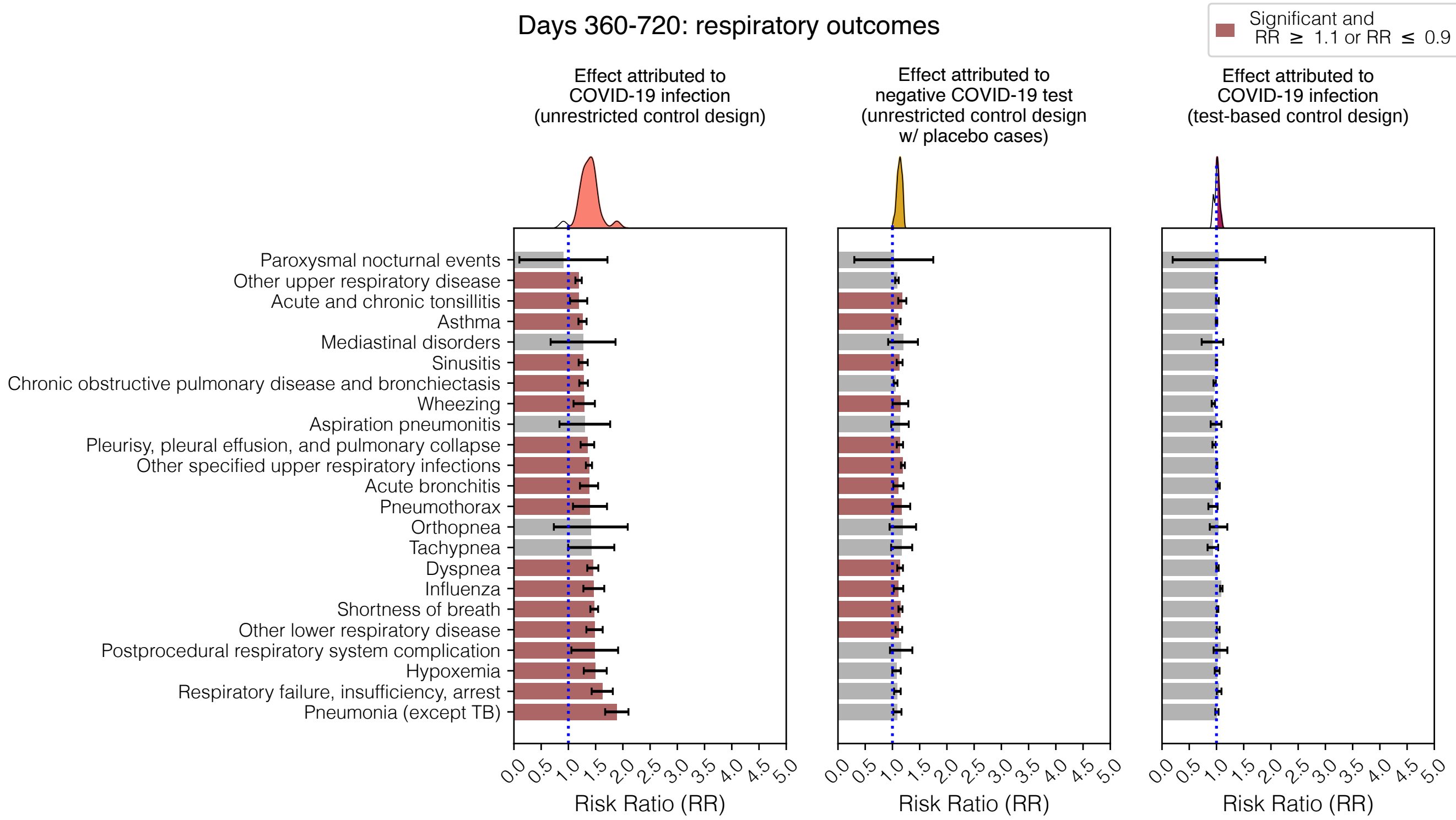


**Fig. S71.** Risk ratios for all outcomes related to the respiratory system on days 360–720 post-enrollment. Effects are shown for an unrestricted control design (left), the test-based control design (right), and an unrestricted control design with placebo cases (middle). Red bars indicate significant effects ($p<0.05$ with Bonferroni correction for multiple comparisons; two-sided bootstrap) with $RR \geq 1.1$ or $RR \leq 0.9$.

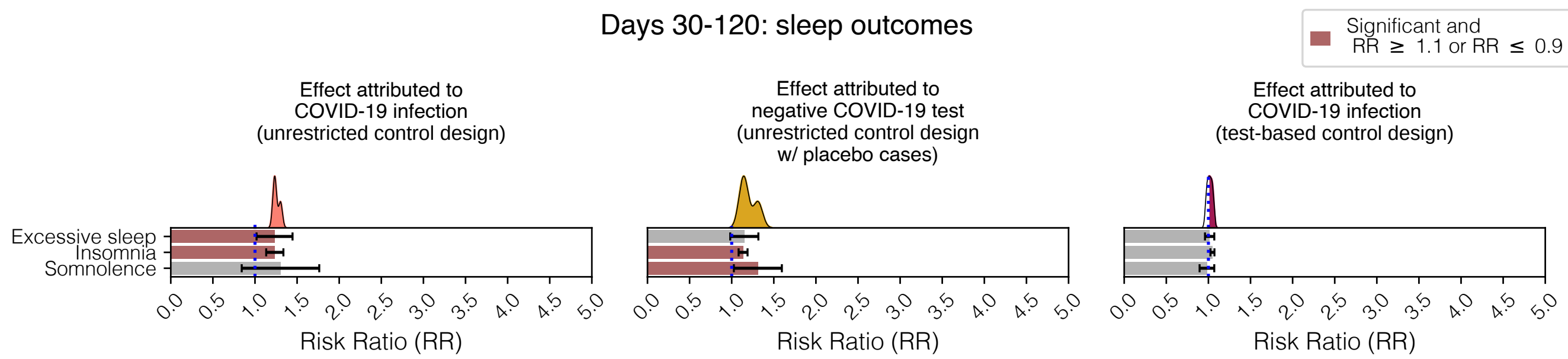


**Fig. S72.** Risk ratios for all outcomes related to sleep on days 30–120 post-enrollment. Effects are shown for an unrestricted control design (left), the test-based control design (right), and an unrestricted control design with placebo cases (middle). Red bars indicate significant effects ($p<0.05$ with Bonferroni correction for multiple comparisons; two-sided bootstrap) with $RR \geq 1.1$ or $RR \leq 0.9$.

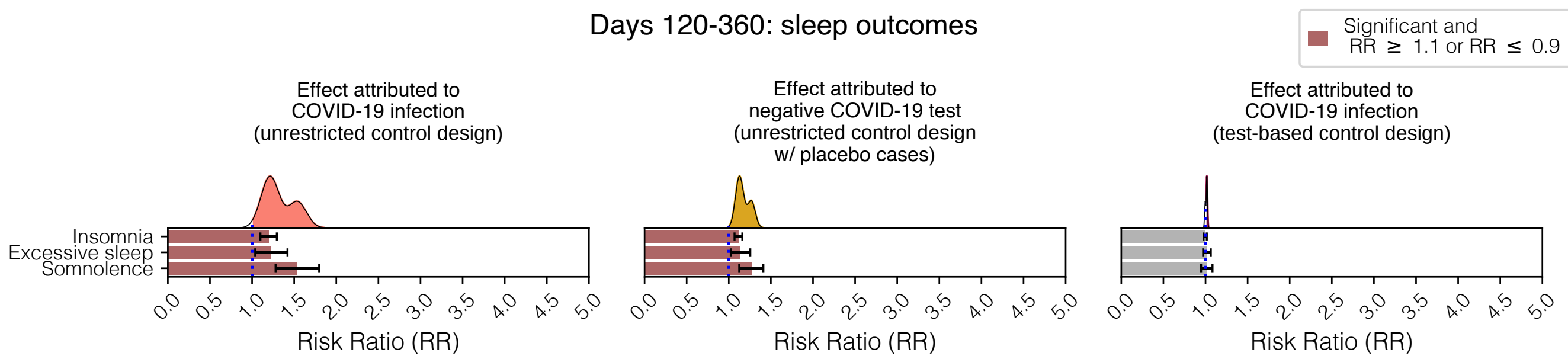


**Fig. S73.** Risk ratios for all outcomes related to sleep on days 120–360 post-enrollment. Effects are shown for an unrestricted control design (left), the test-based control design (right), and an unrestricted control design with placebo cases (middle). Red bars indicate significant effects ($p<0.05$ with Bonferroni correction for multiple comparisons; two-sided bootstrap) with $RR\geq1.1$ or $RR\leq0.9$.

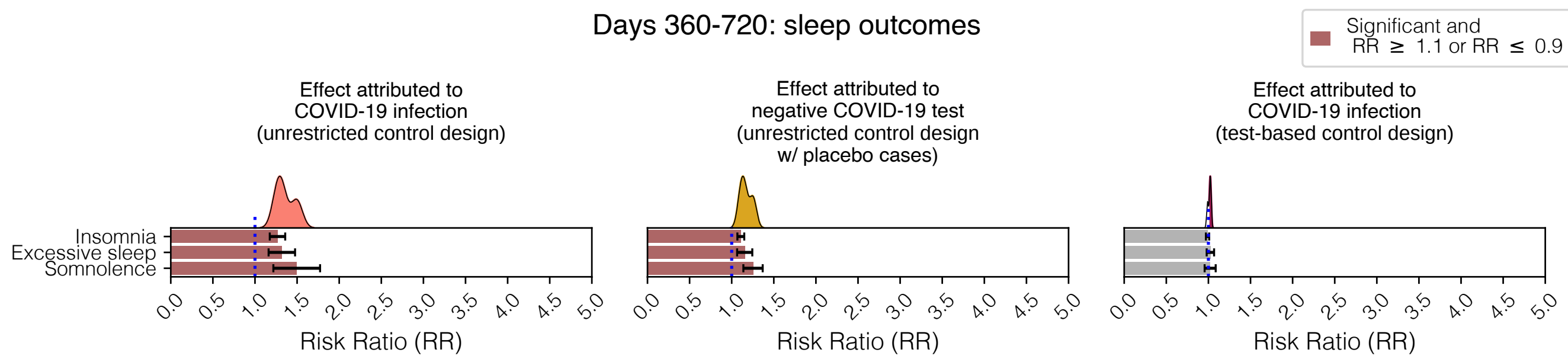


**Fig. S74.** Risk ratios for all outcomes related to sleep on days 360–720 post-enrollment. Effects are shown for an unrestricted control design (left), the test-based control design (right), and an unrestricted control design with placebo cases (middle). Red bars indicate significant effects ($p<0.05$ with Bonferroni correction for multiple comparisons; two-sided bootstrap) with $RR \geq 1.1$ or $RR \leq 0.9$.

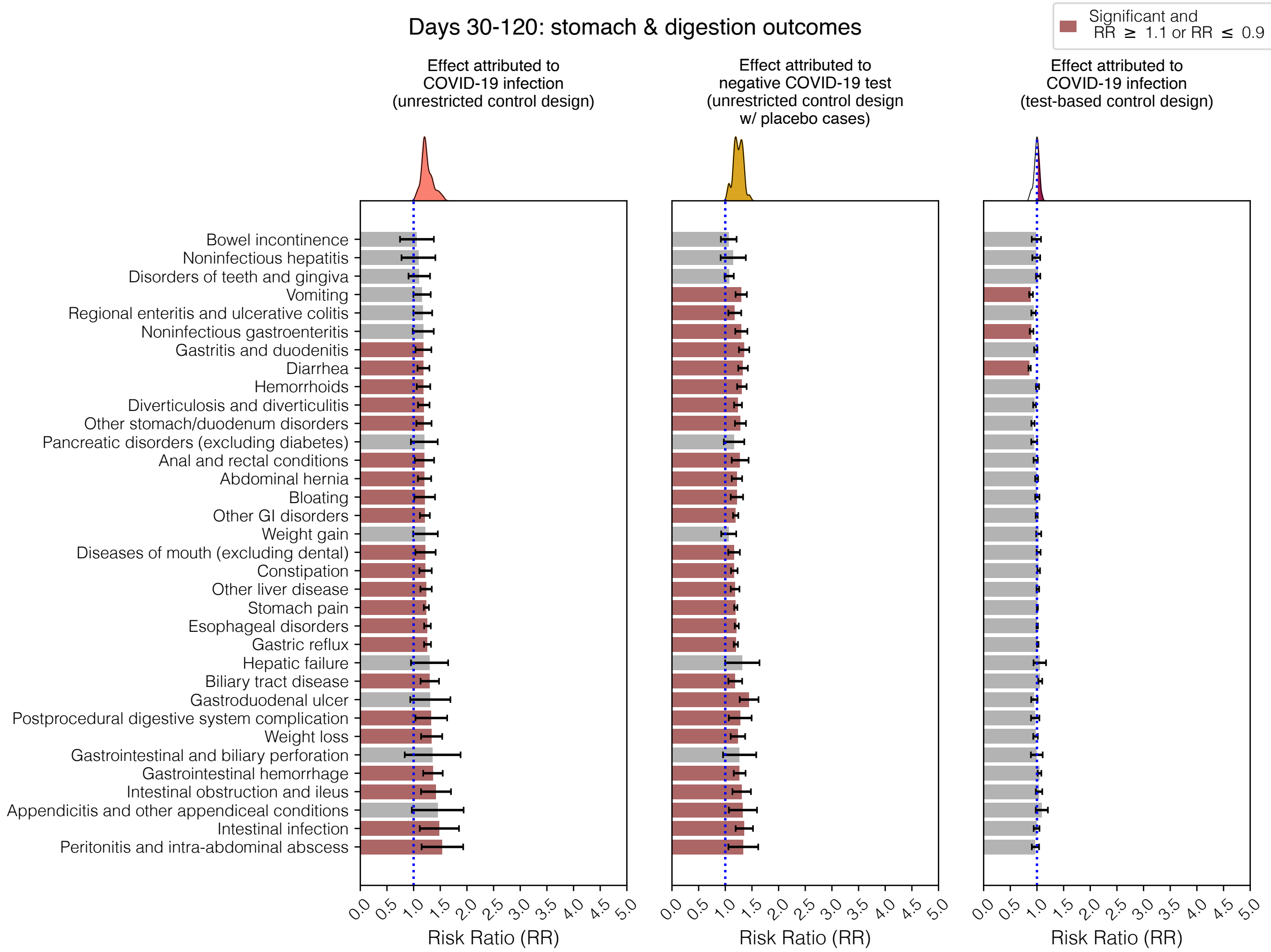


**Fig. S75.** Risk ratios for all outcomes related to stomach & digestion on days 30–120 post-enrollment. Effects are shown for an unrestricted control design (left), the test-based control design (right), and an unrestricted control design with placebo cases (middle). Red bars indicate significant effects (p<0.05 with Bonferroni correction for multiple comparisons; two-sided bootstrap) with RR≥1.1 or RR≤0.9.

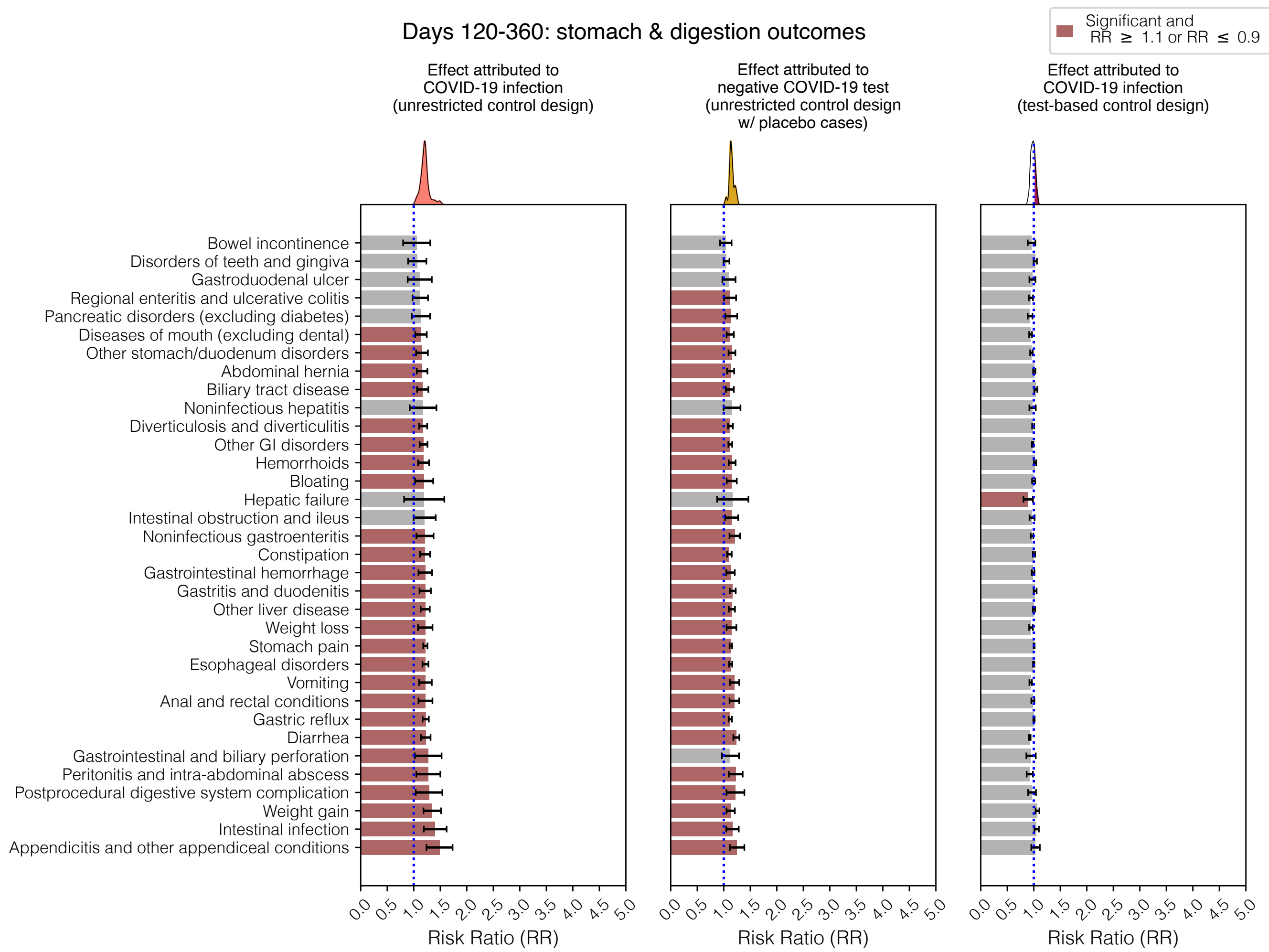


**Fig. S76.** Risk ratios for all outcomes related to stomach & digestion on days 120–360 post-enrollment. Effects are shown for an unrestricted control design (left), the test-based control design (right), and an unrestricted control design with placebo cases (middle). Red bars indicate significant effects ($p<0.05$ with Bonferroni correction for multiple comparisons; two-sided bootstrap) with RR$\geq$1.1 or RR$\leq$0.9.

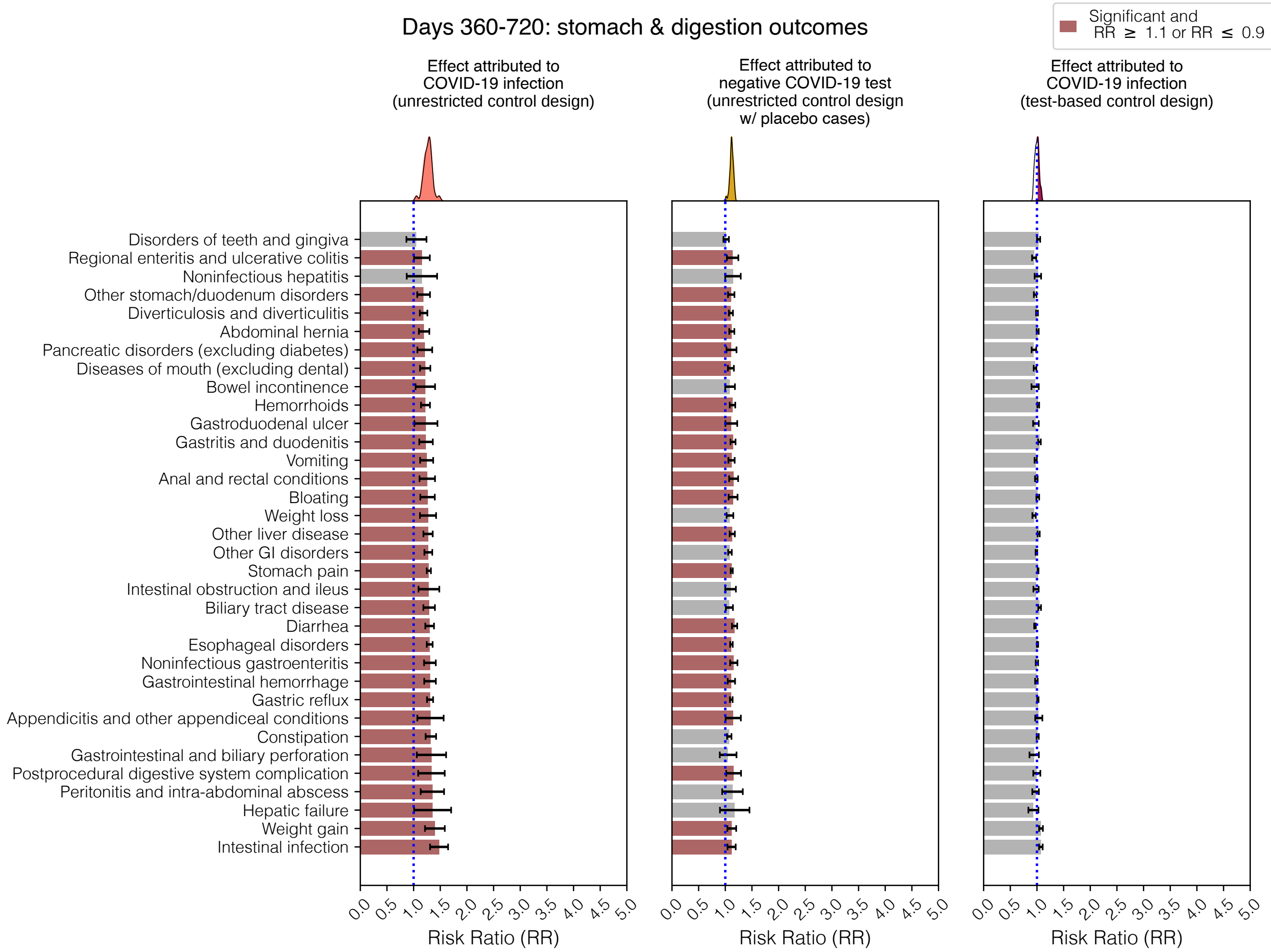


**Fig. S77.** Risk ratios for all outcomes related to stomach & digestion on days 360–720 post-enrollment. Effects are shown for an unrestricted control design (left), the test-based control design (right), and an unrestricted control design with placebo cases (middle). Red bars indicate significant effects (p<0.05 with Bonferroni correction for multiple comparisons; two-sided bootstrap) with RR≥1.1 or RR≤0.9.

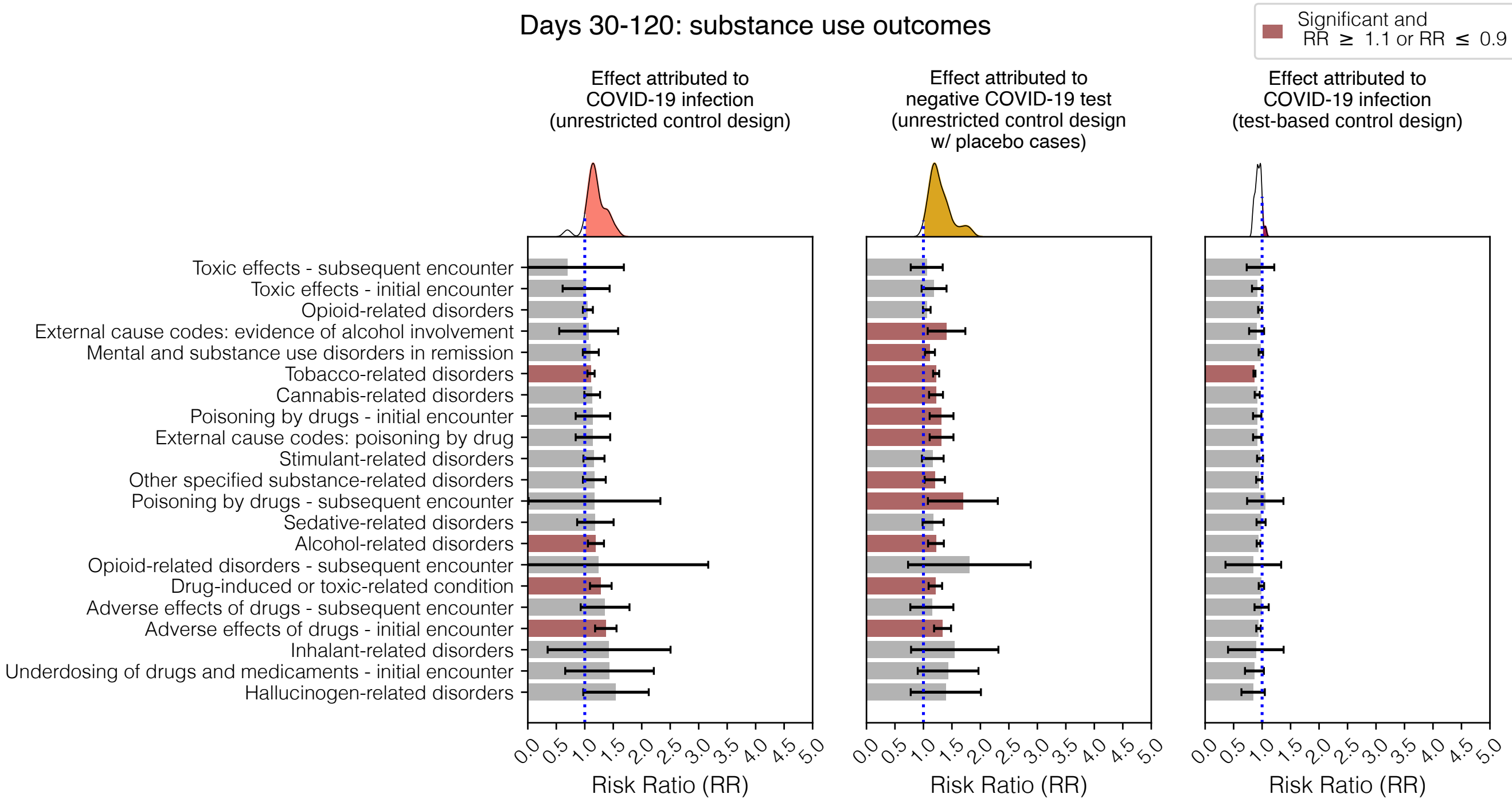


**Fig. S78.** Risk ratios for all outcomes related to substance use on days 30–120 post-enrollment. Effects are shown for an unrestricted control design (left), the test-based control design (right), and an unrestricted control design with placebo cases (middle). Red bars indicate significant effects (p<0.05 with Bonferroni correction for multiple comparisons; two-sided bootstrap) with RR≥1.1 or RR≤0.9.

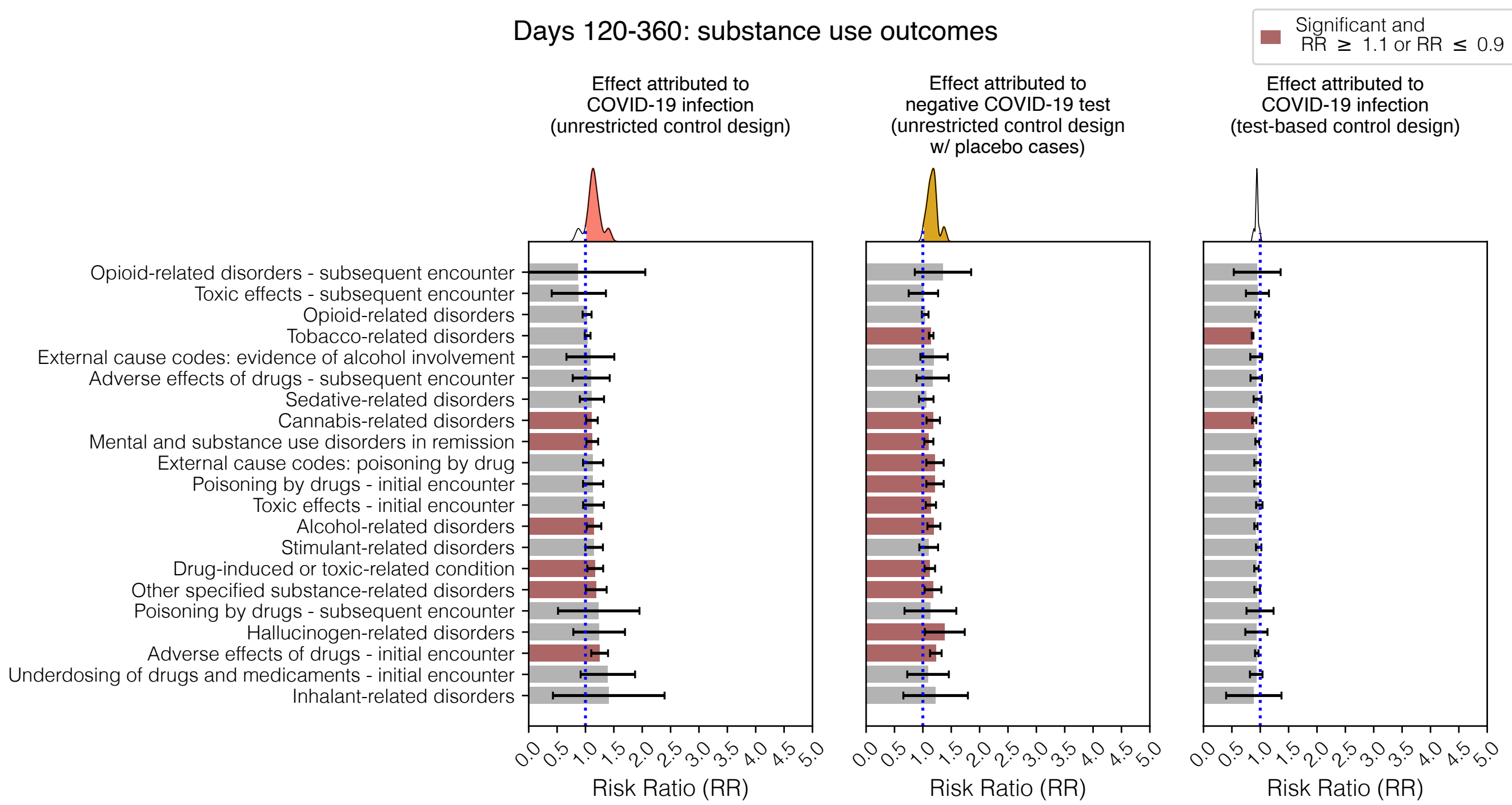


**Fig. S79.** Risk ratios for all outcomes related to substance use on days 120–360 post-enrollment. Effects are shown for an unrestricted control design (left), the test-based control design (right), and an unrestricted control design with placebo cases (middle). Red bars indicate significant effects (p<0.05 with Bonferroni correction for multiple comparisons; two-sided bootstrap) with RR≥1.1 or RR≤0.9.

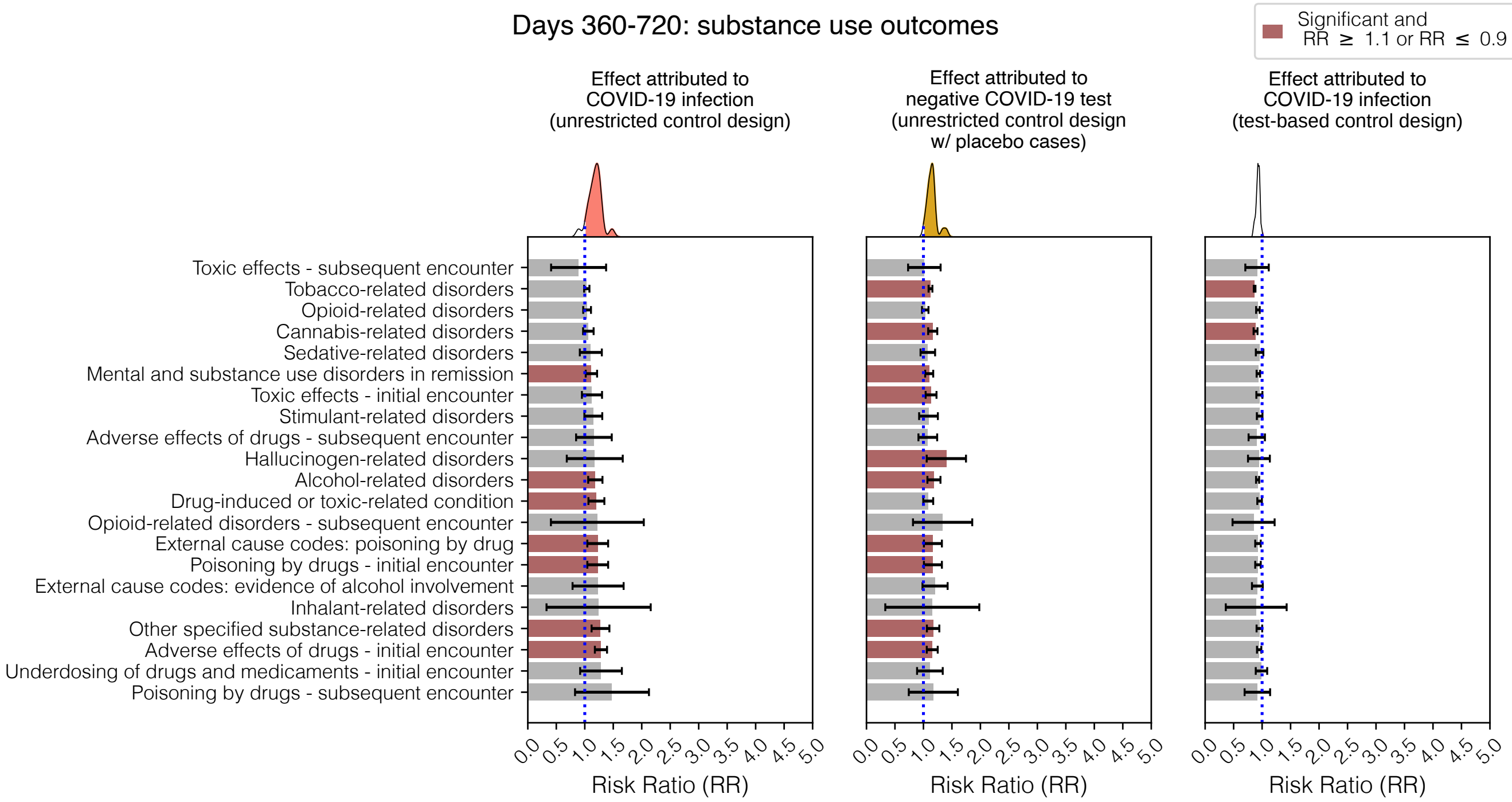


**Fig. S80.** Risk ratios for all outcomes related to substance use on days 360–720 post-enrollment. Effects are shown for an unrestricted control design (left), the test-based control design (right), and an unrestricted control design with placebo cases (middle). Red bars indicate significant effects ($p<0.05$ with Bonferroni correction for multiple comparisons; two-sided bootstrap) with RR$\geq$1.1 or RR$\leq$0.9.

 

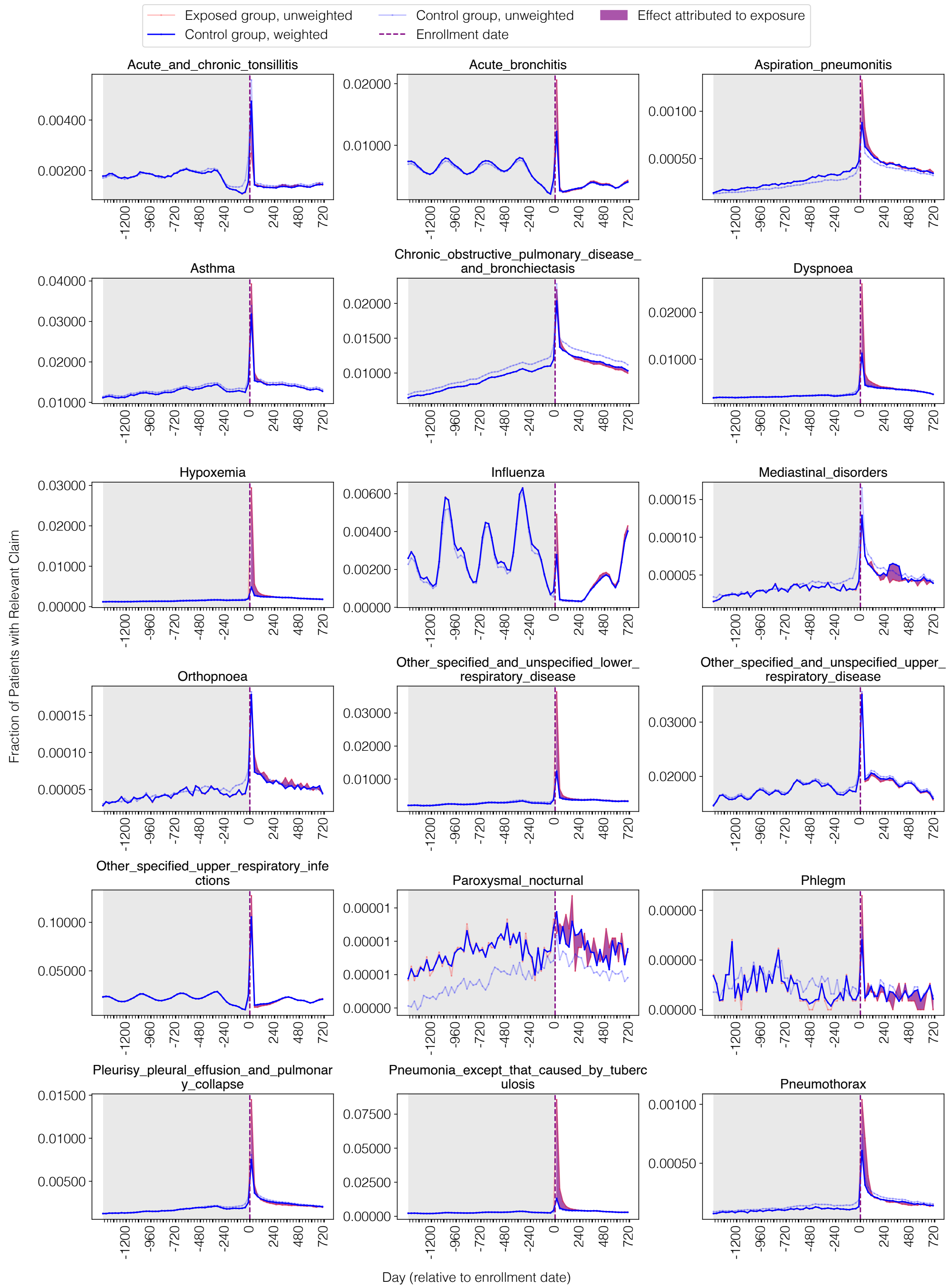


**Fig. S81.** Temporal trends in Breathing prevalence. The fraction of patients with relevant claims over time belonging for each outcome in the **test-based control design** experiment.

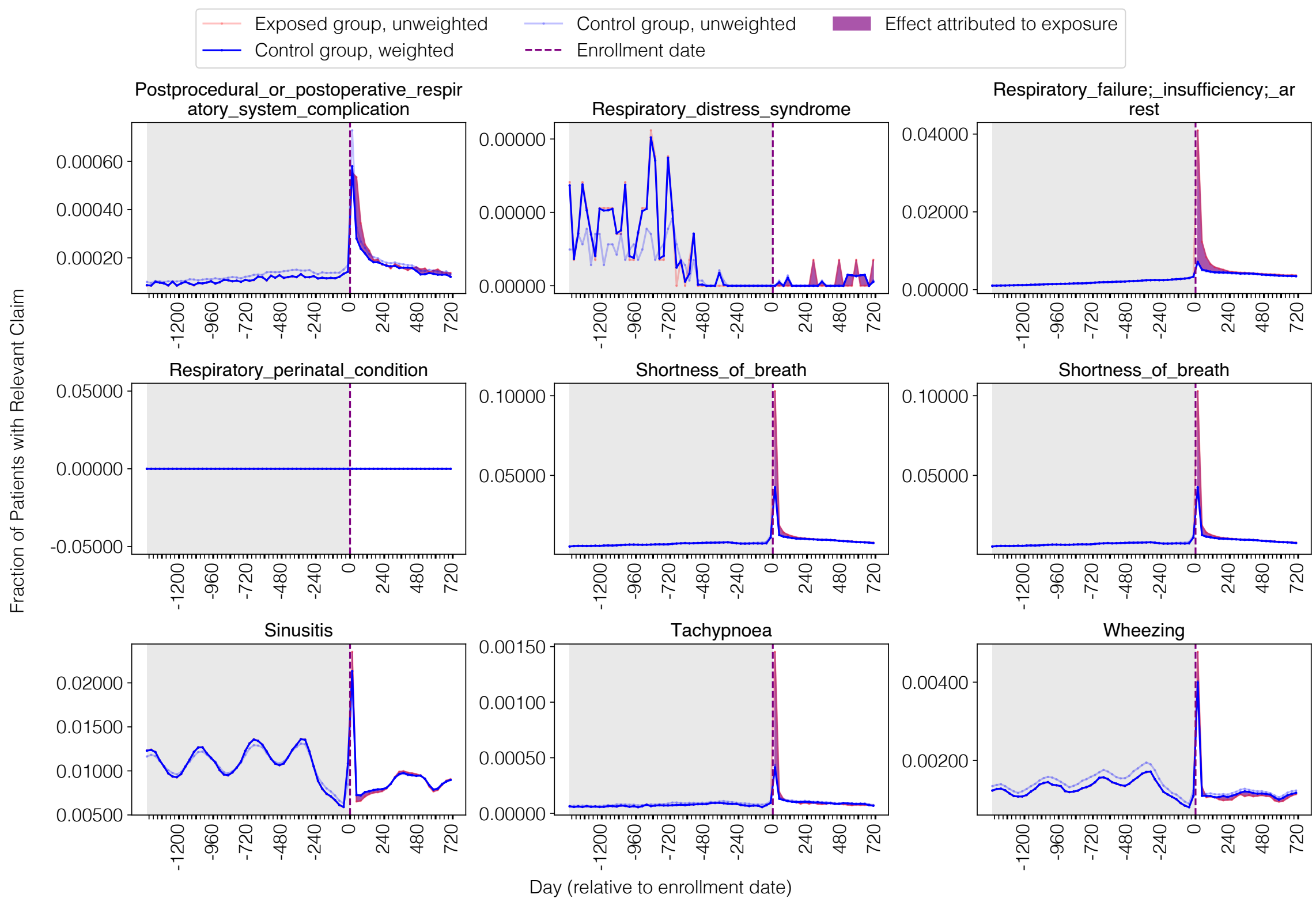


**Fig. S81.** (cont'd) Temporal trends in Breathing prevalence. The fraction of patients with relevant claims over time belonging for each outcome in the **test-based control design** experiment.

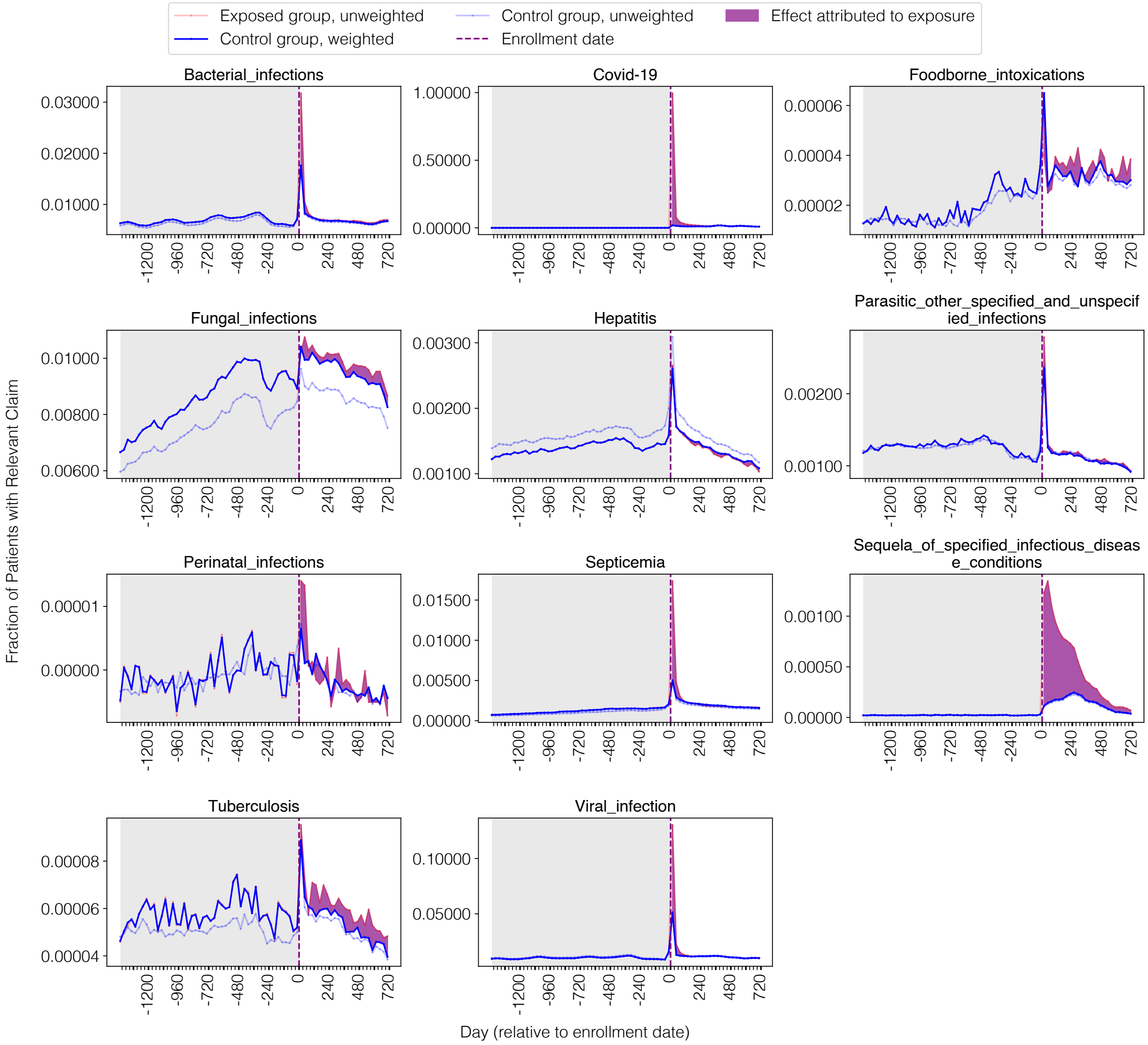


**Fig. S82.** Temporal trends in Certain Infectious and Parasitic Diseases prevalence. The fraction of patients with relevant claims over time belonging for each outcome in the **test-based control design** experiment.

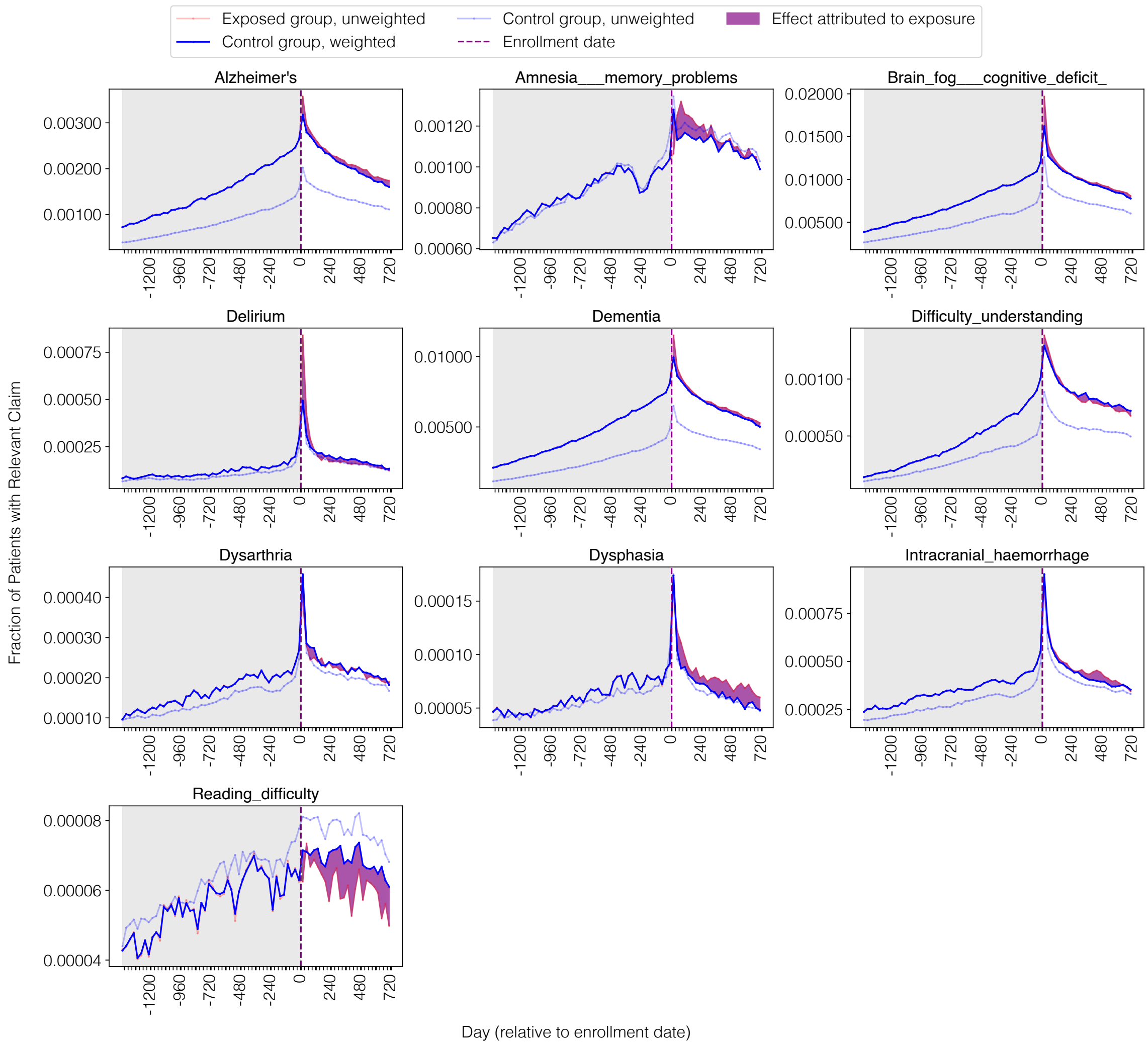


**Fig. S83.** Temporal trends in Cognitive health prevalence. The fraction of patients with relevant claims over time belonging for each outcome in the **test-based control design** experiment.

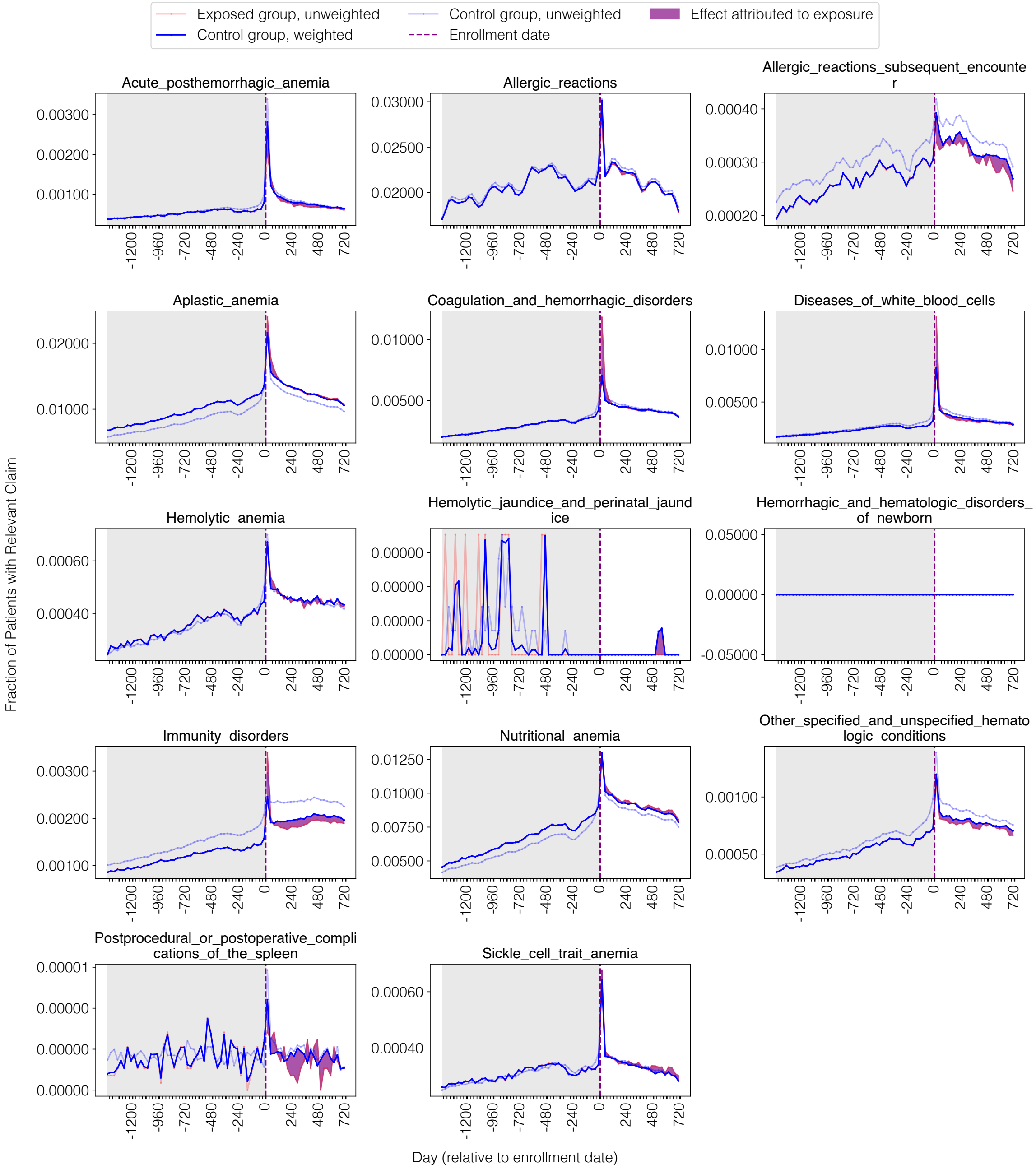


**Fig. S84.** Temporal trends in Diseases of the Blood and Blood Forming Organs and Certain Disorders Involving the Immune Mechanism prevalence. The fraction of patients with relevant claims over time belonging for each outcome in the **test-based control design** experiment.

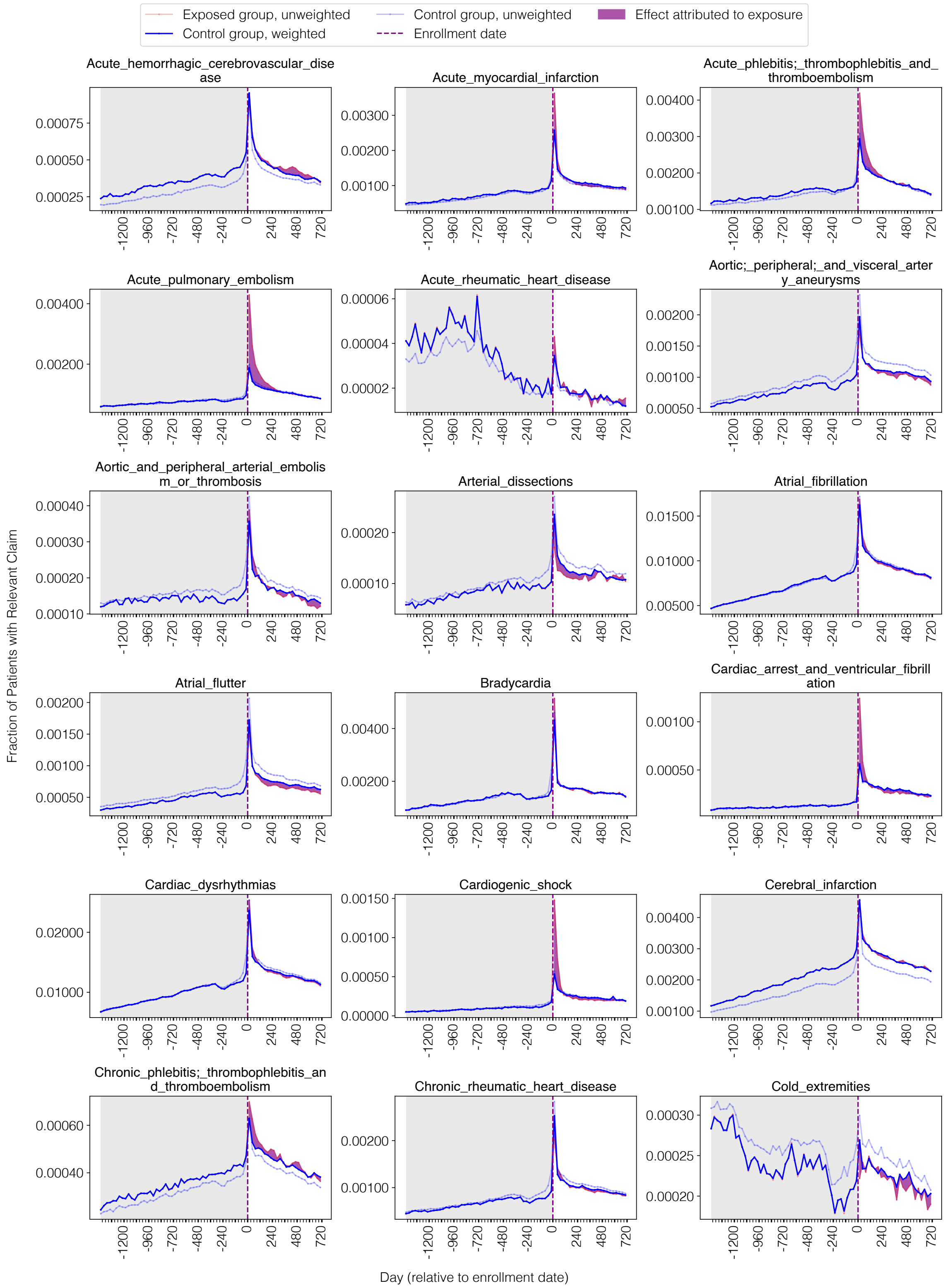


**Fig. S85.** Temporal trends in Diseases of the Circulatory System prevalence. The fraction of patients with relevant claims over time belonging for each outcome in the **test-based control design** experiment.

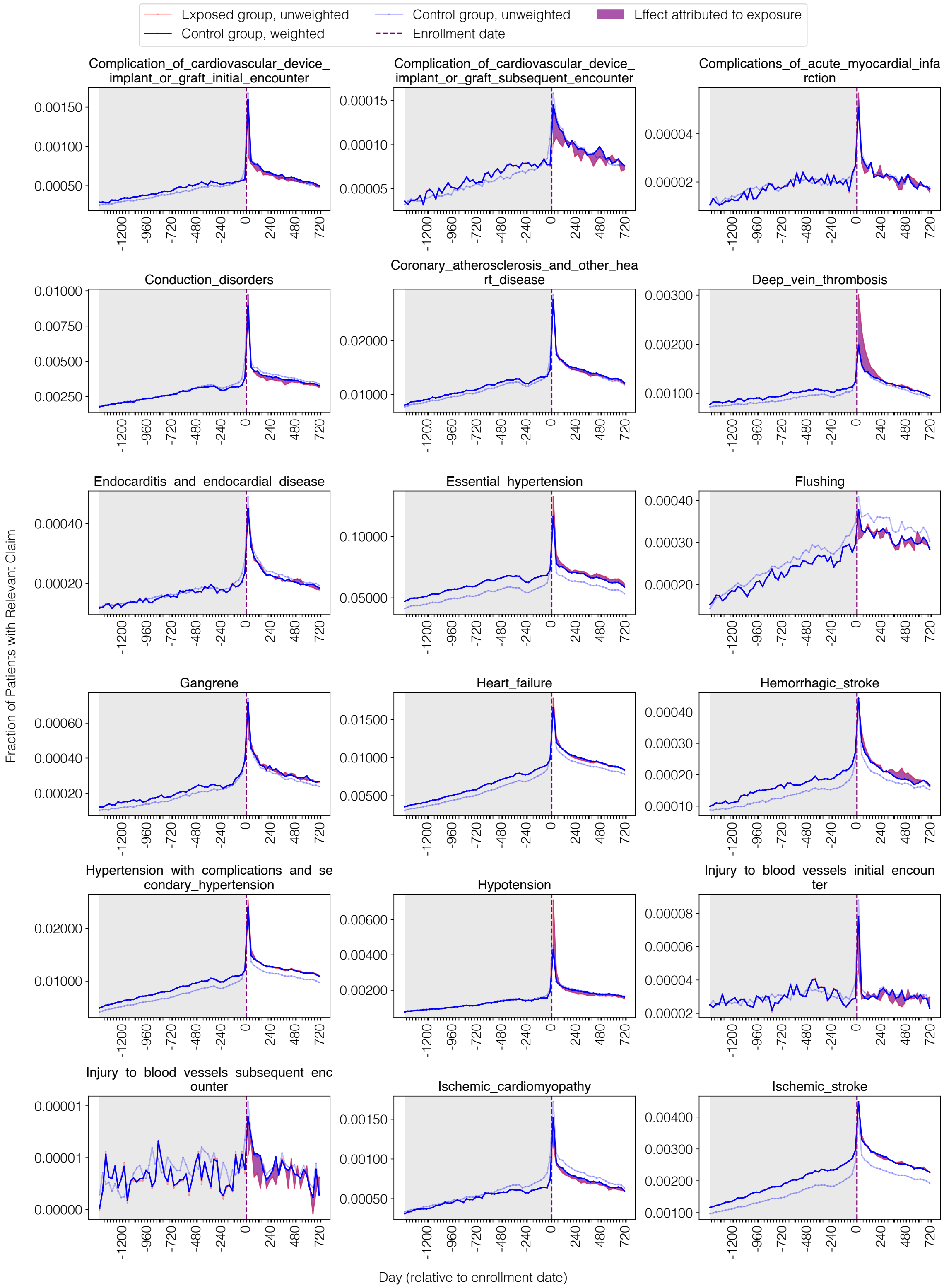


**Fig. S85.** (cont'd) Temporal trends in Diseases of the Circulatory System prevalence. The fraction of patients with relevant claims over time belonging for each outcome in the **test-based control design** experiment.

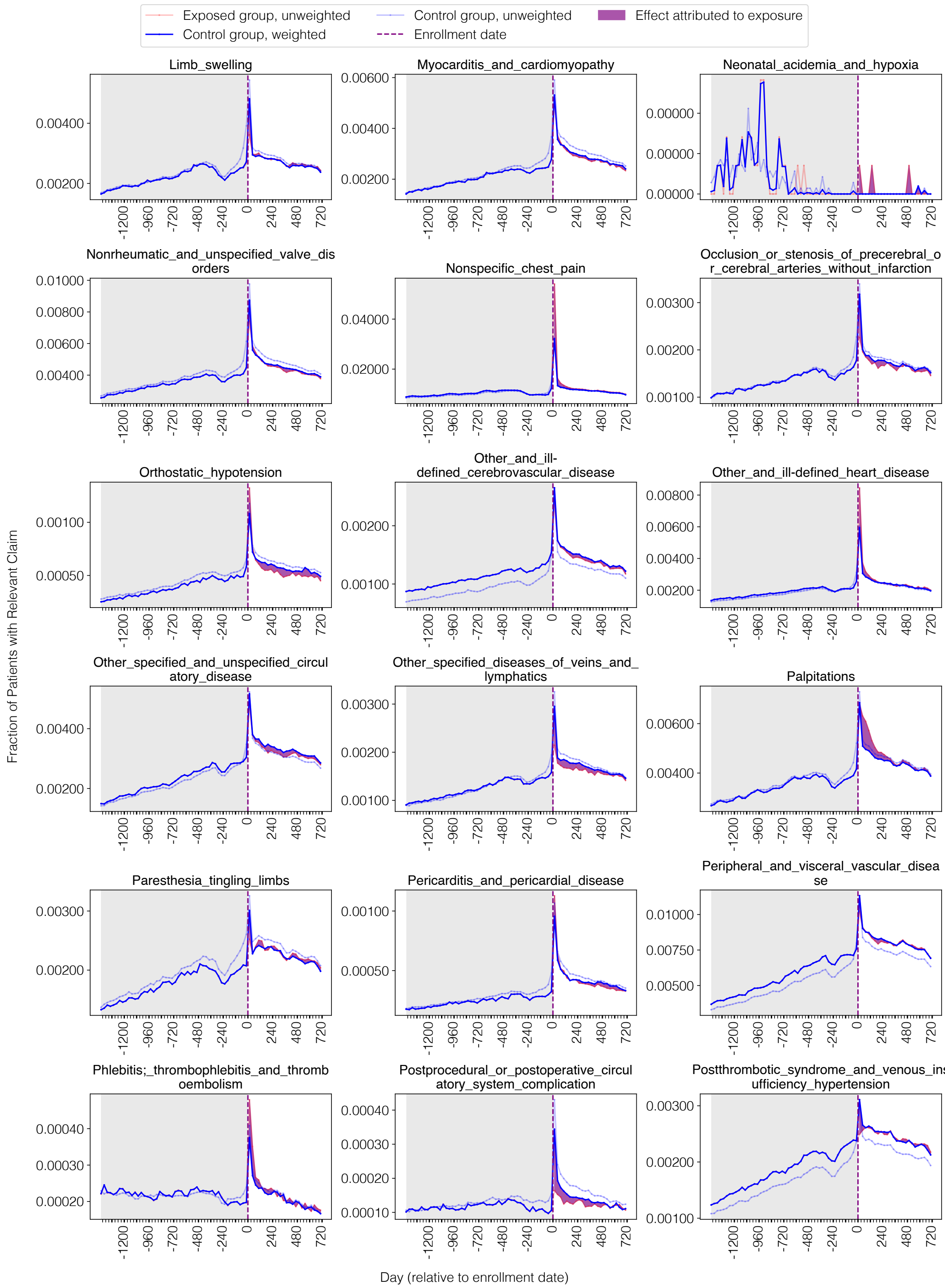


**Fig. S85.** (cont'd) Temporal trends in Diseases of the Circulatory System prevalence. The fraction of patients with relevant claims over time belonging for each outcome in the **test-based control design** experiment.

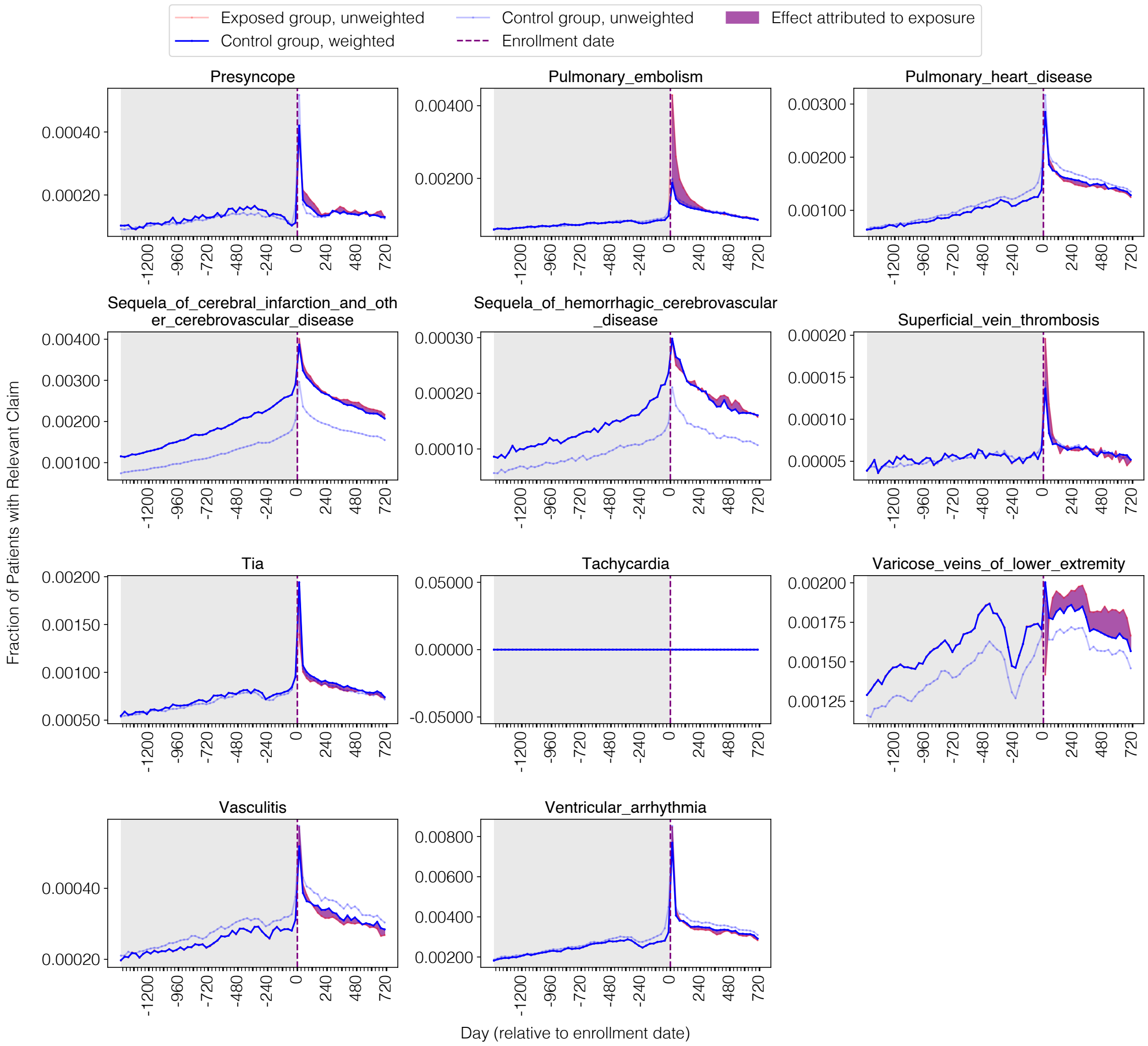


**Fig. S85.** (cont'd) Temporal trends in Diseases of the Circulatory System prevalence. The fraction of patients with relevant claims over time belonging for each outcome in the **test-based control design** experiment.

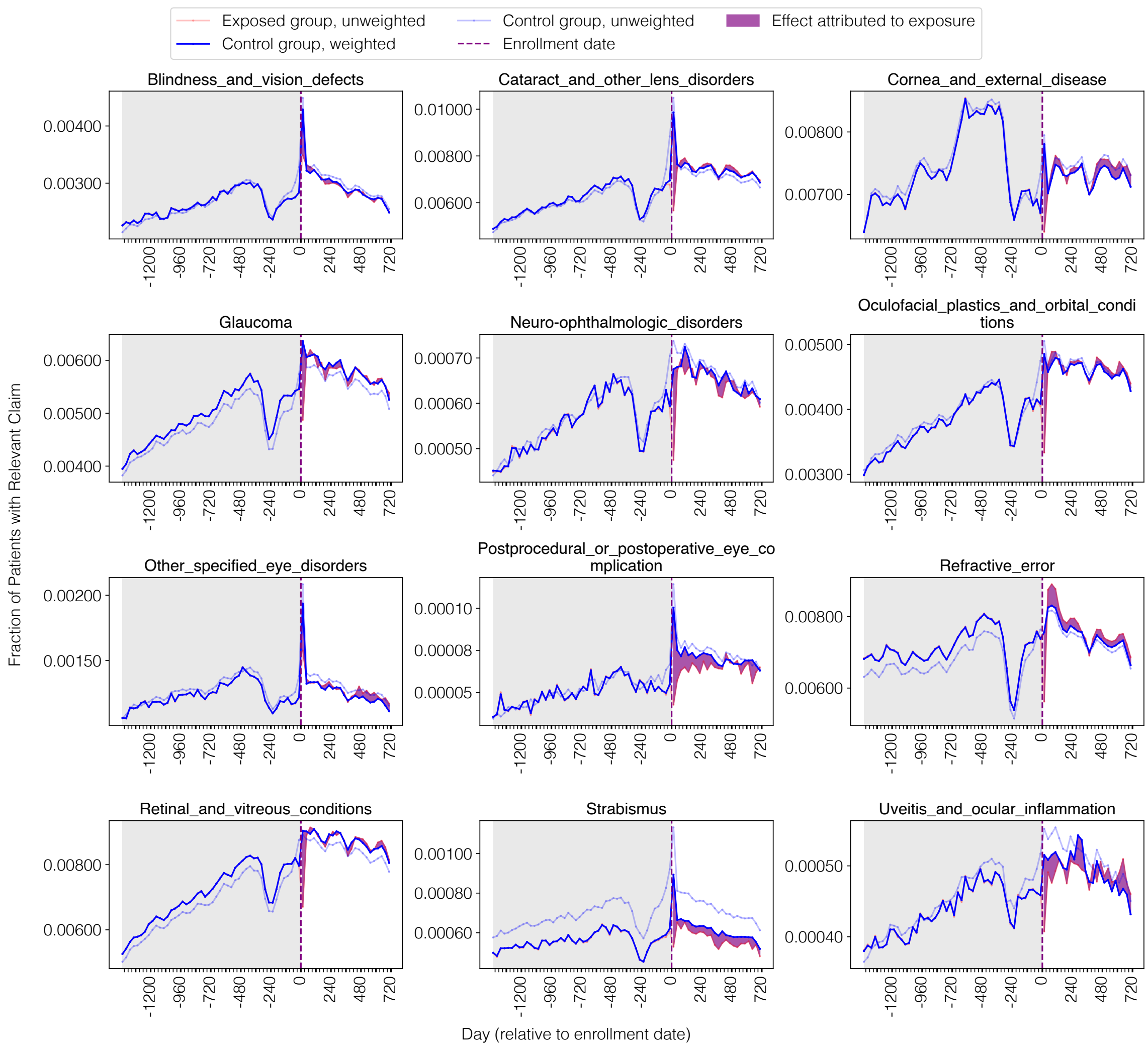


**Fig. S86.** Temporal trends in Diseases of the Eye and Adnexa prevalence. The fraction of patients with relevant claims over time belonging for each outcome in the **test-based control design** experiment.

 

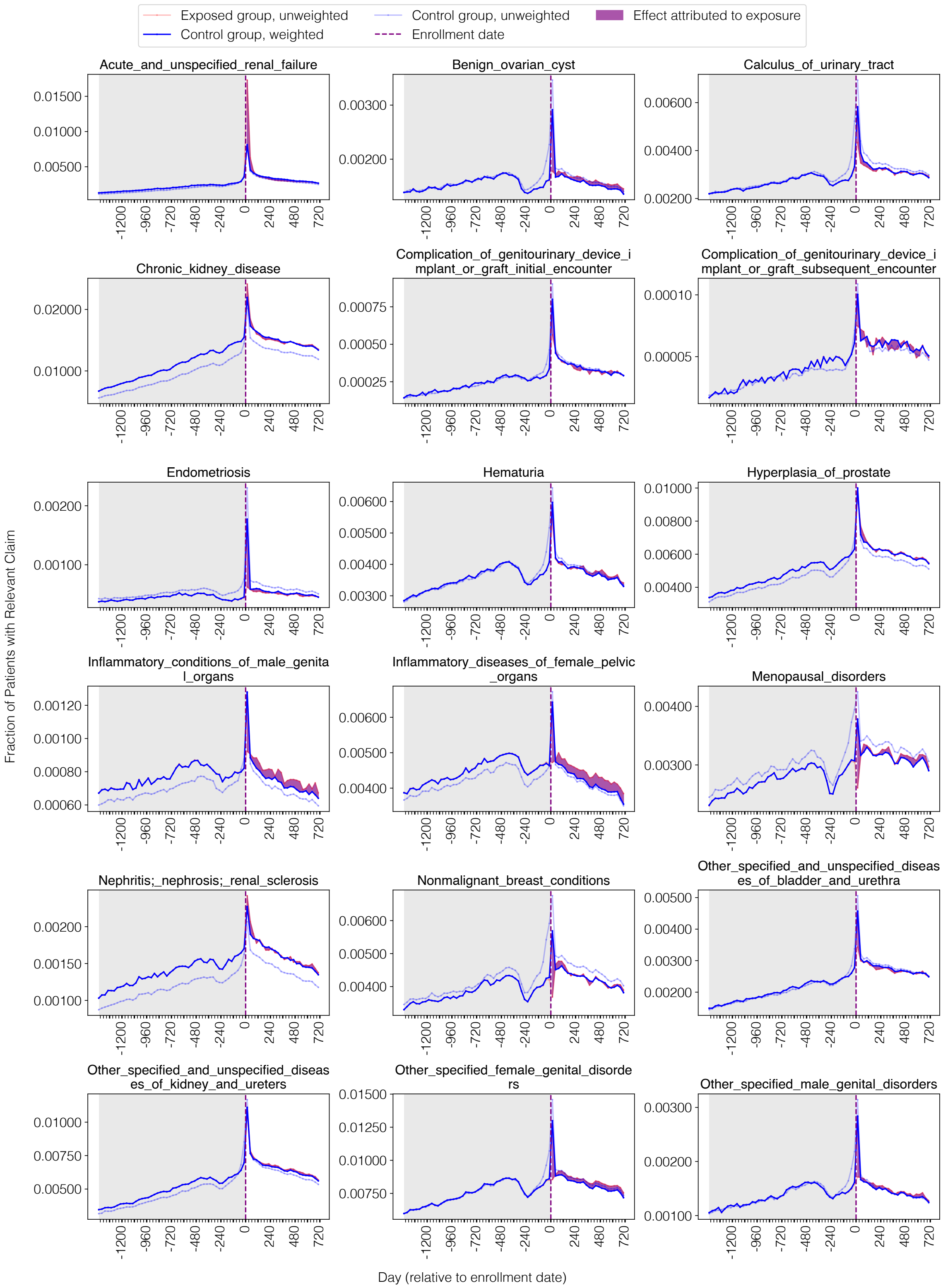


**Fig. S87.** Temporal trends in Diseases of the Genitourinary System prevalence. The fraction of patients with relevant claims over time belonging for each outcome in the **test-based control design** experiment.

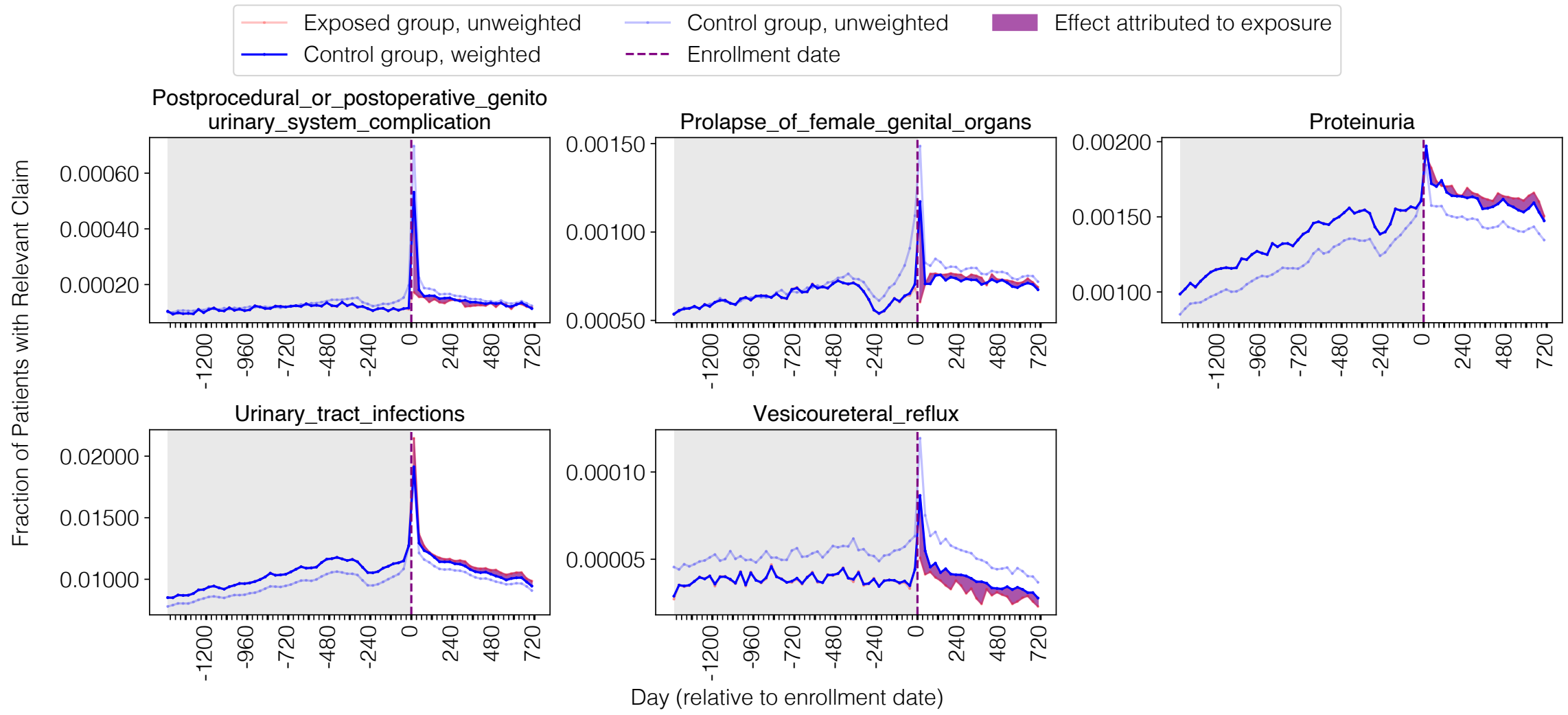


**Fig. S87.** (cont'd) Temporal trends in Diseases of the Genitourinary System prevalence. The fraction of patients with relevant claims over time belonging for each outcome in the **test-based control design** experiment.

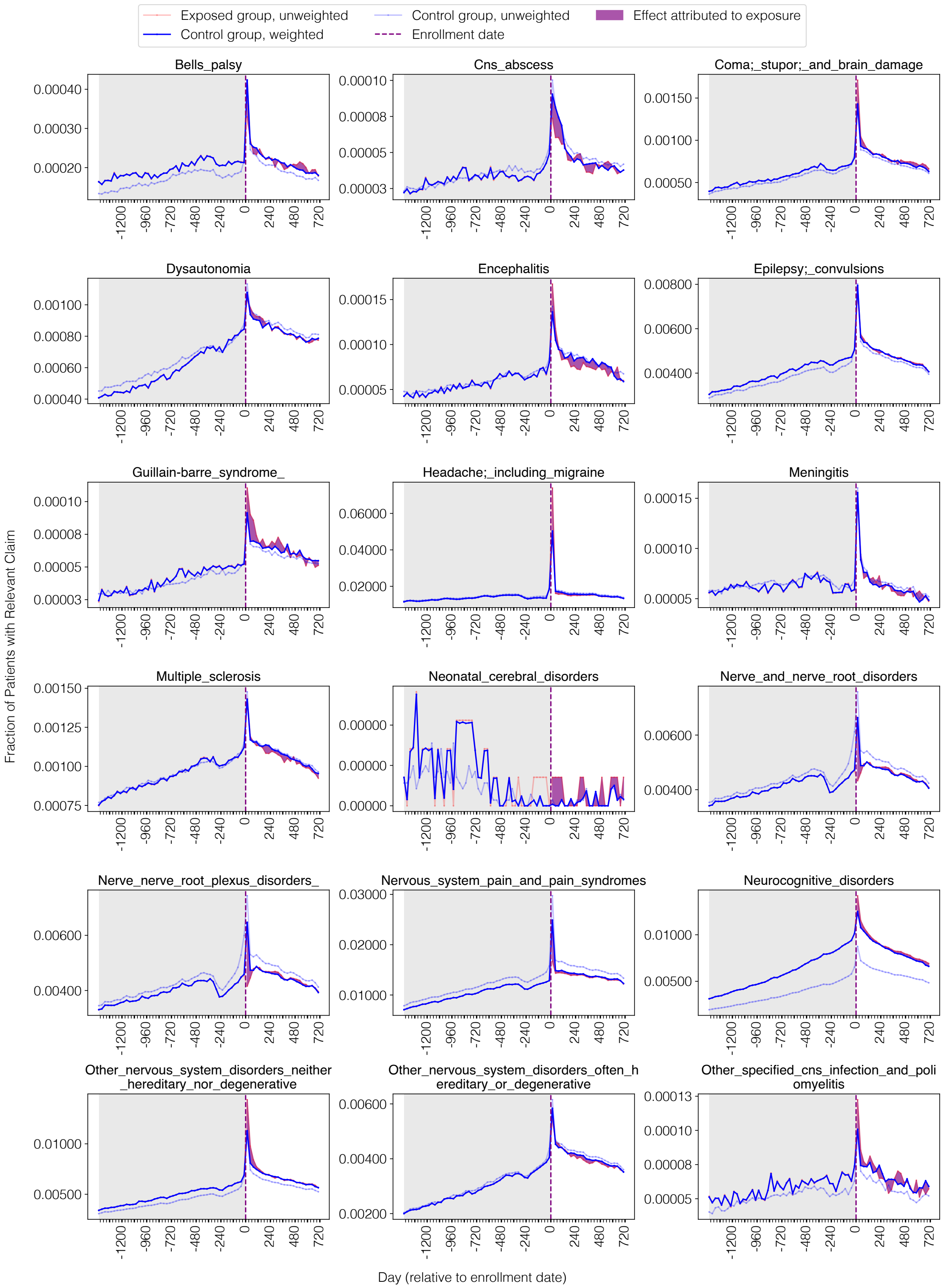


**Fig. S88.** Temporal trends in Diseases of the Nervous System prevalence. The fraction of patients with relevant claims over time belonging for each outcome in the **test-based control design** experiment.

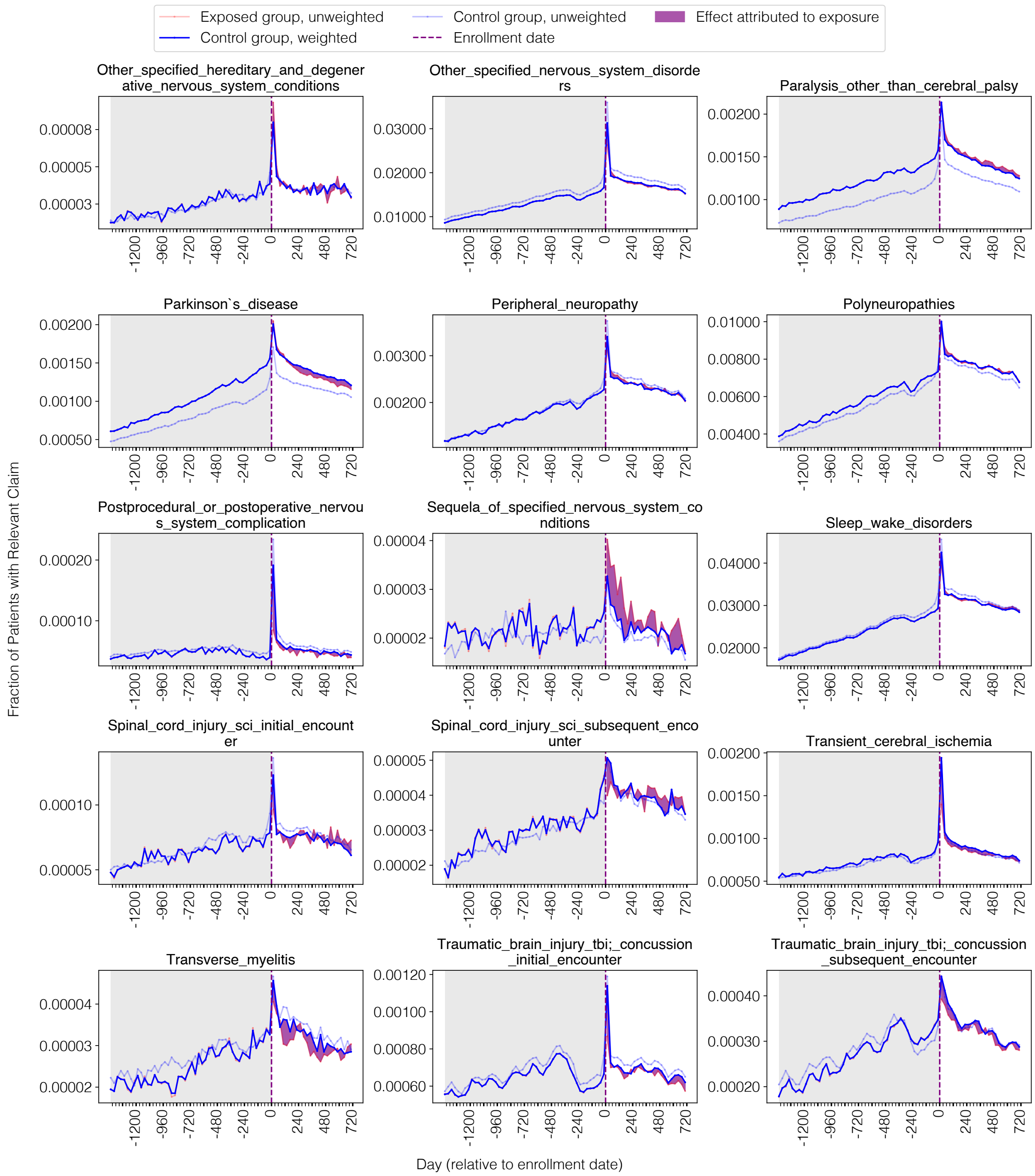


**Fig. S88.** (cont'd) Temporal trends in Diseases of the Nervous System prevalence. The fraction of patients with relevant claims over time belonging for each outcome in the **test-based control design** experiment.

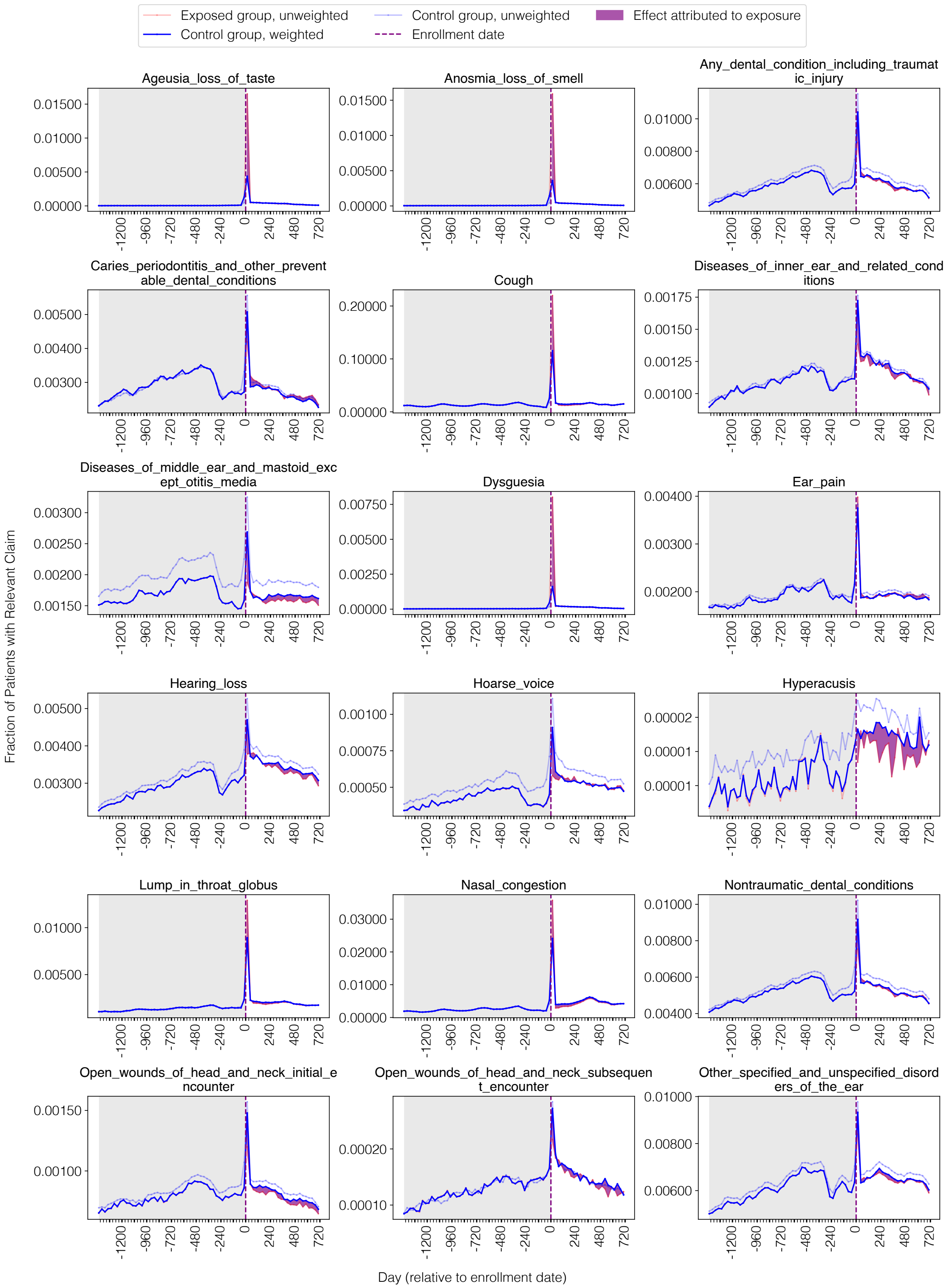


**Fig. S89.** Temporal trends in Ear, nose and throat prevalence. The fraction of patients with relevant claims over time belonging for each outcome in the **test-based control design** experiment.

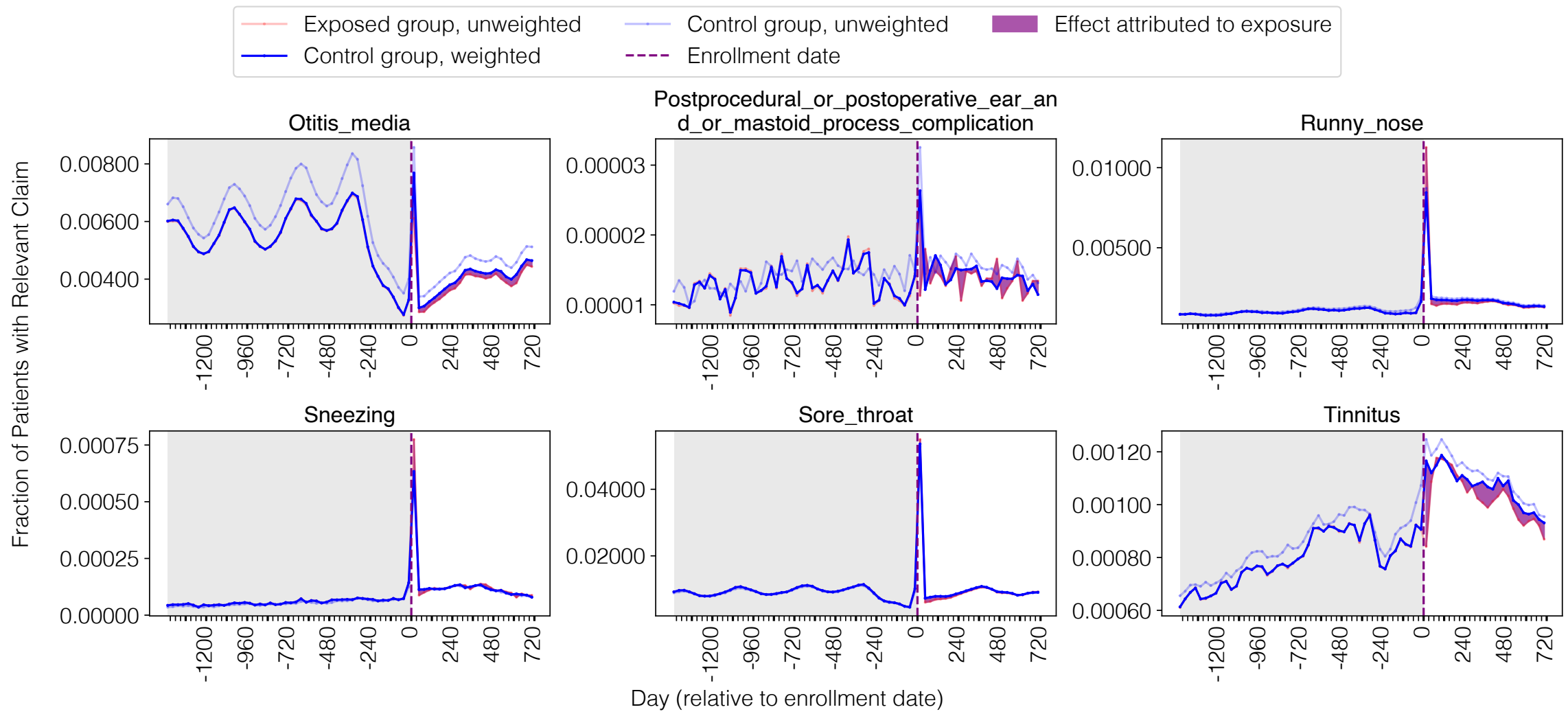


**Fig. S89.** (cont'd) Temporal trends in Ear, nose and throat prevalence. The fraction of patients with relevant claims over time belonging for each outcome in the **test-based control design** experiment.

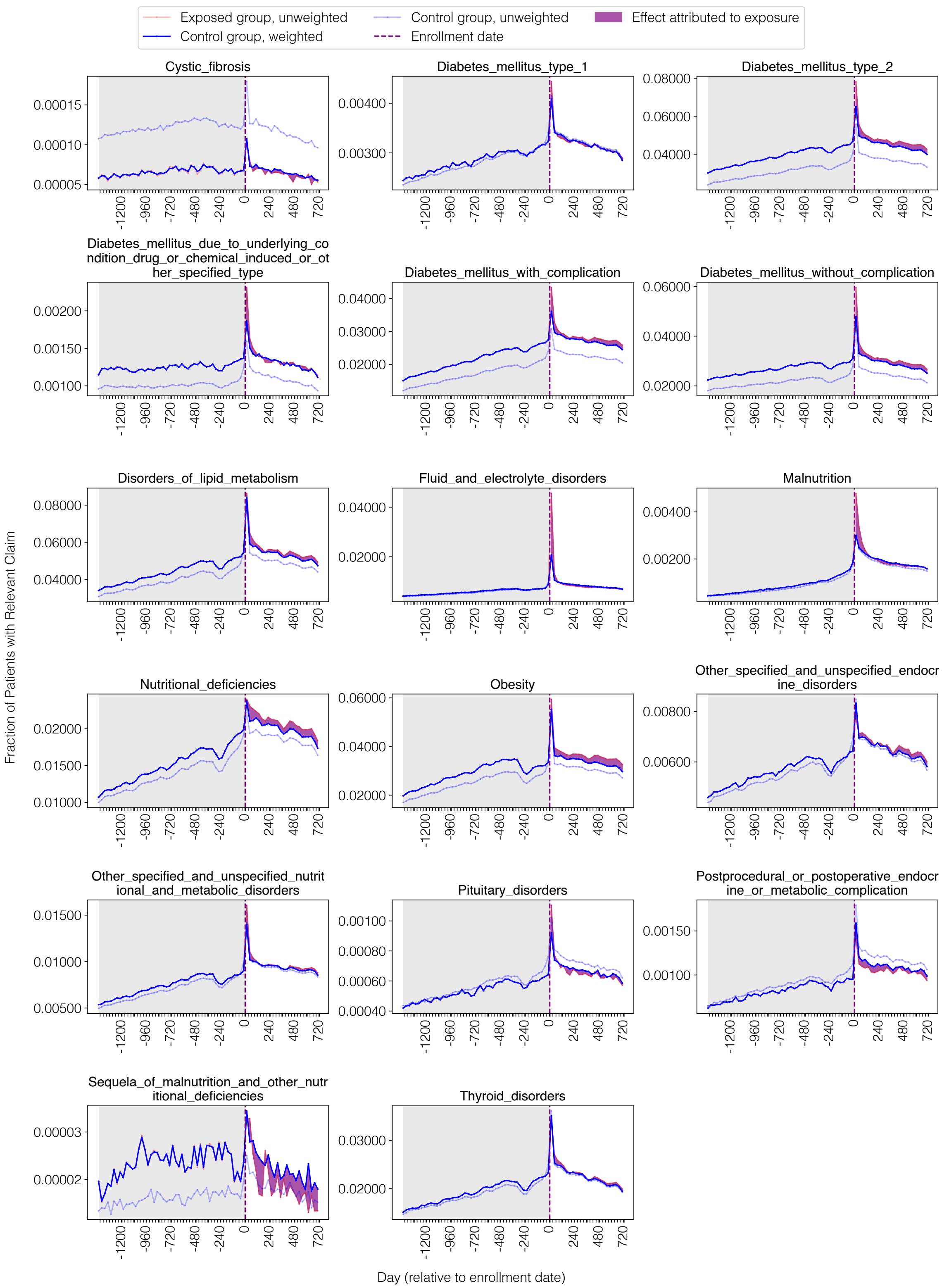


**Fig. S90.** Temporal trends in Endocrine, Nutritional and Metabolic Diseases prevalence. The fraction of patients with relevant claims over time belonging for each outcome in the **test-based control design** experiment.

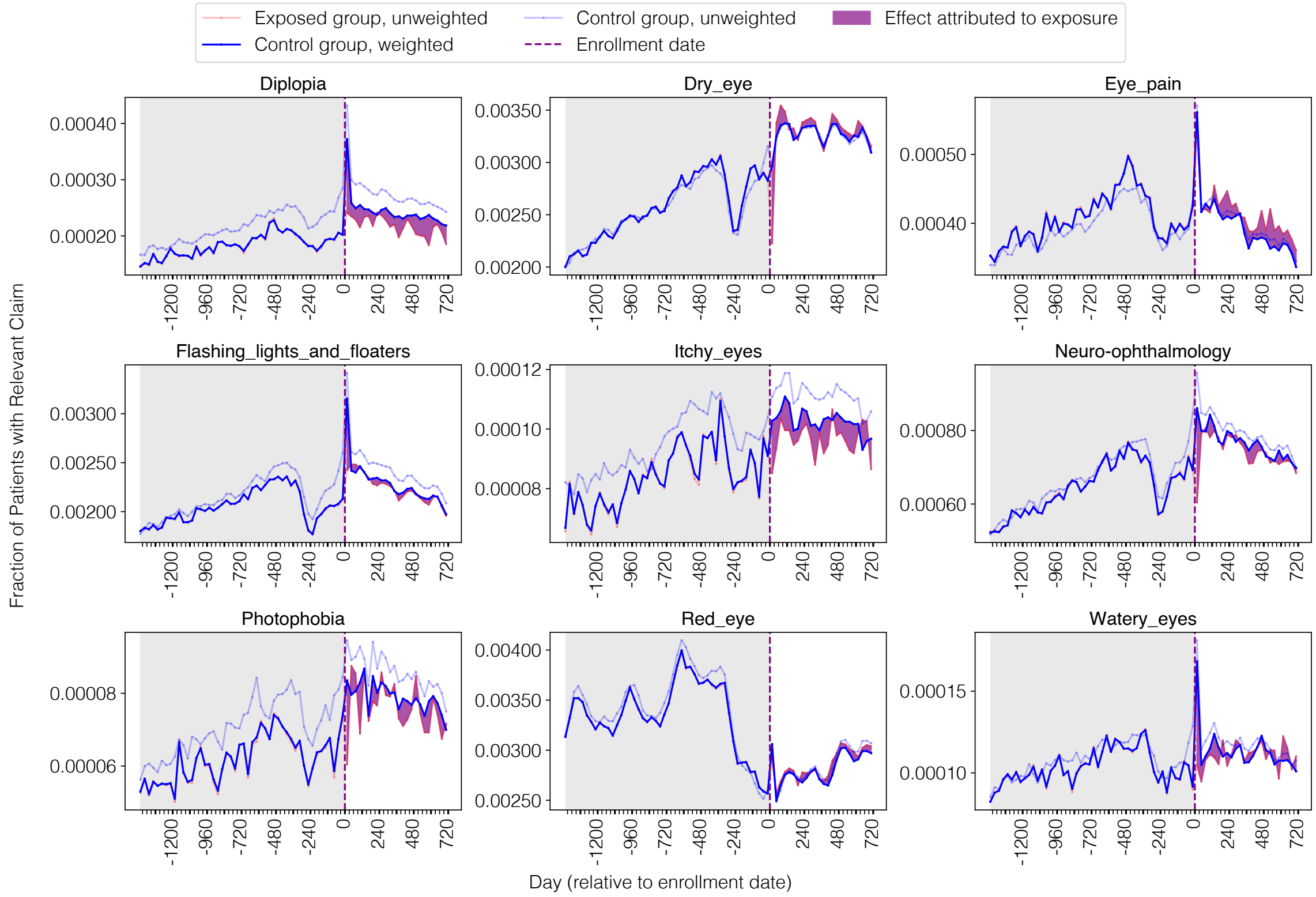


**Fig. S91.** Temporal trends in Eyes prevalence. The fraction of patients with relevant claims over time belonging for each outcome in the **test-based control design** experiment.

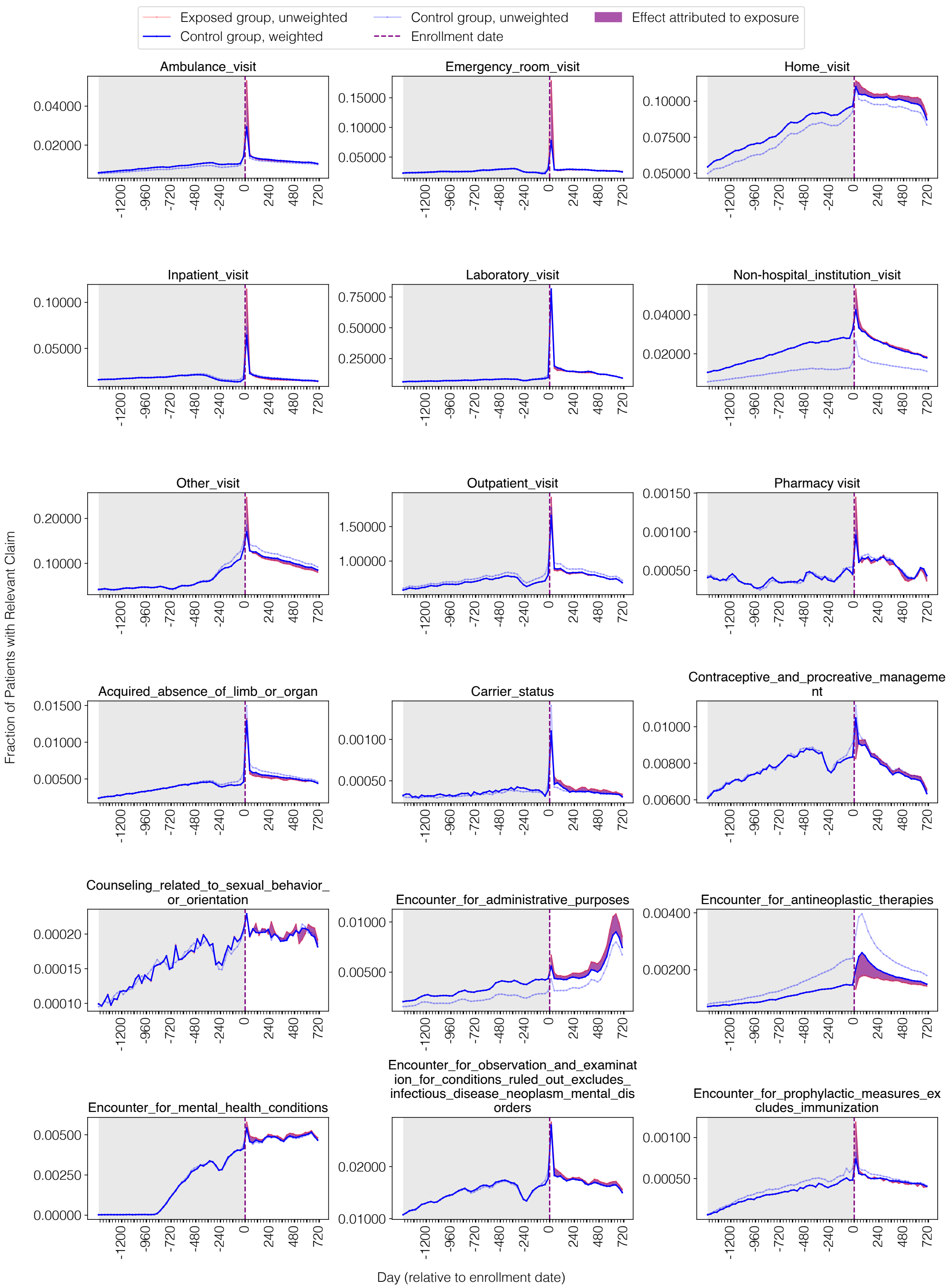


**Fig. S92.** Temporal trends in Factors Influencing Health Status and Contact with Health Services prevalence. The fraction of patients with relevant claims over time belonging for each outcome in the **test-based control design** experiment.

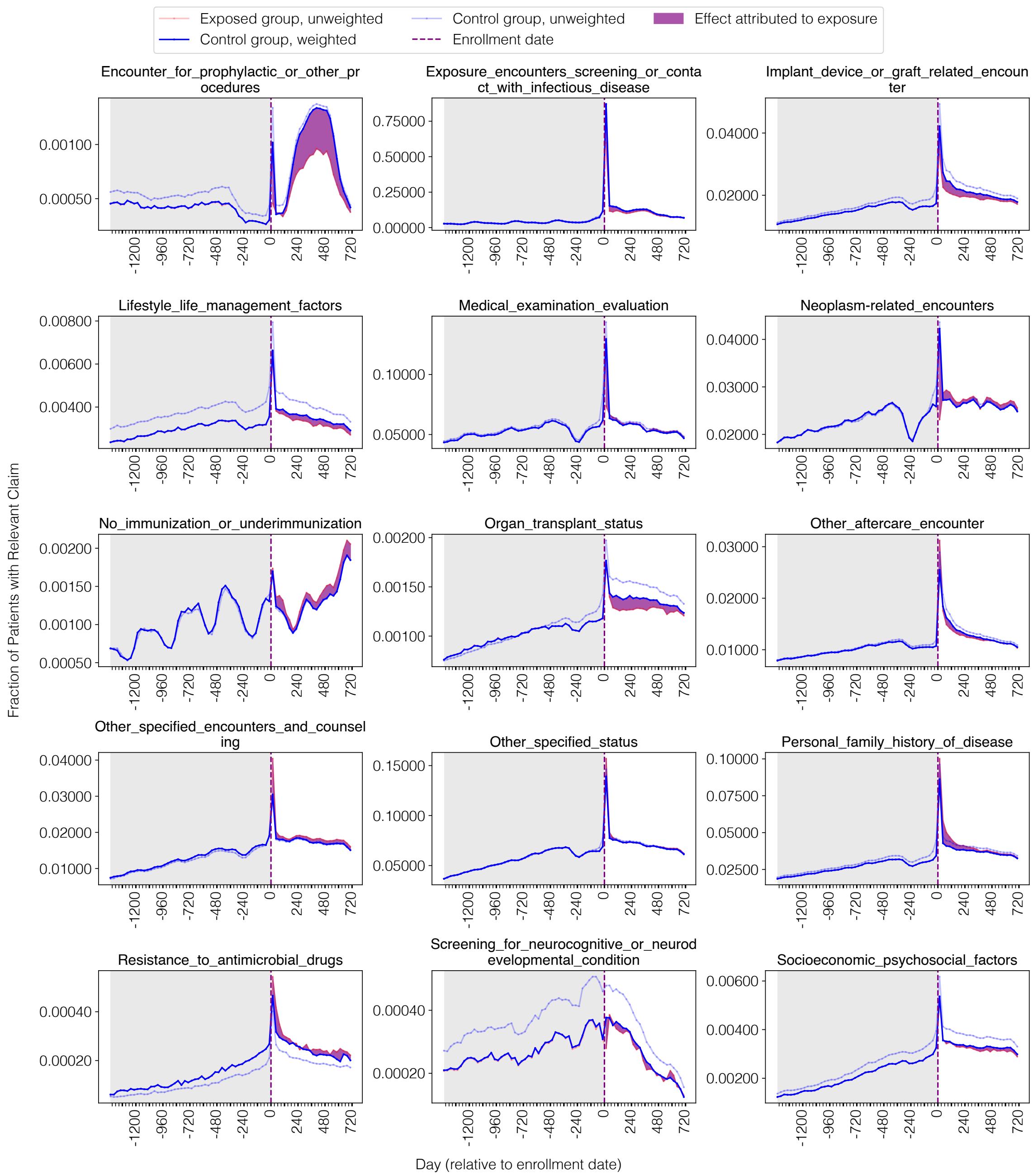


**Fig. S92.** (cont'd) Temporal trends in Factors Influencing Health Status and Contact with Health Services prevalence. The fraction of patients with relevant claims over time belonging for each outcome in the **test-based control design** experiment.

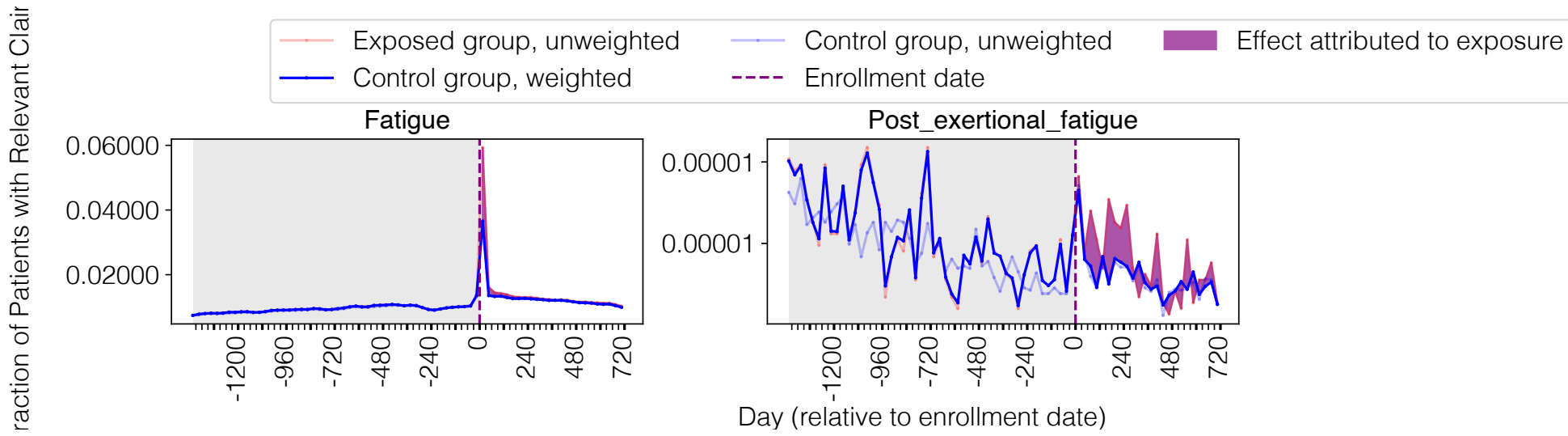


**Fig. S93.** Temporal trends in Fatigue prevalence. The fraction of patients with relevant claims over time belonging for each outcome in the **test-based control design** experiment.

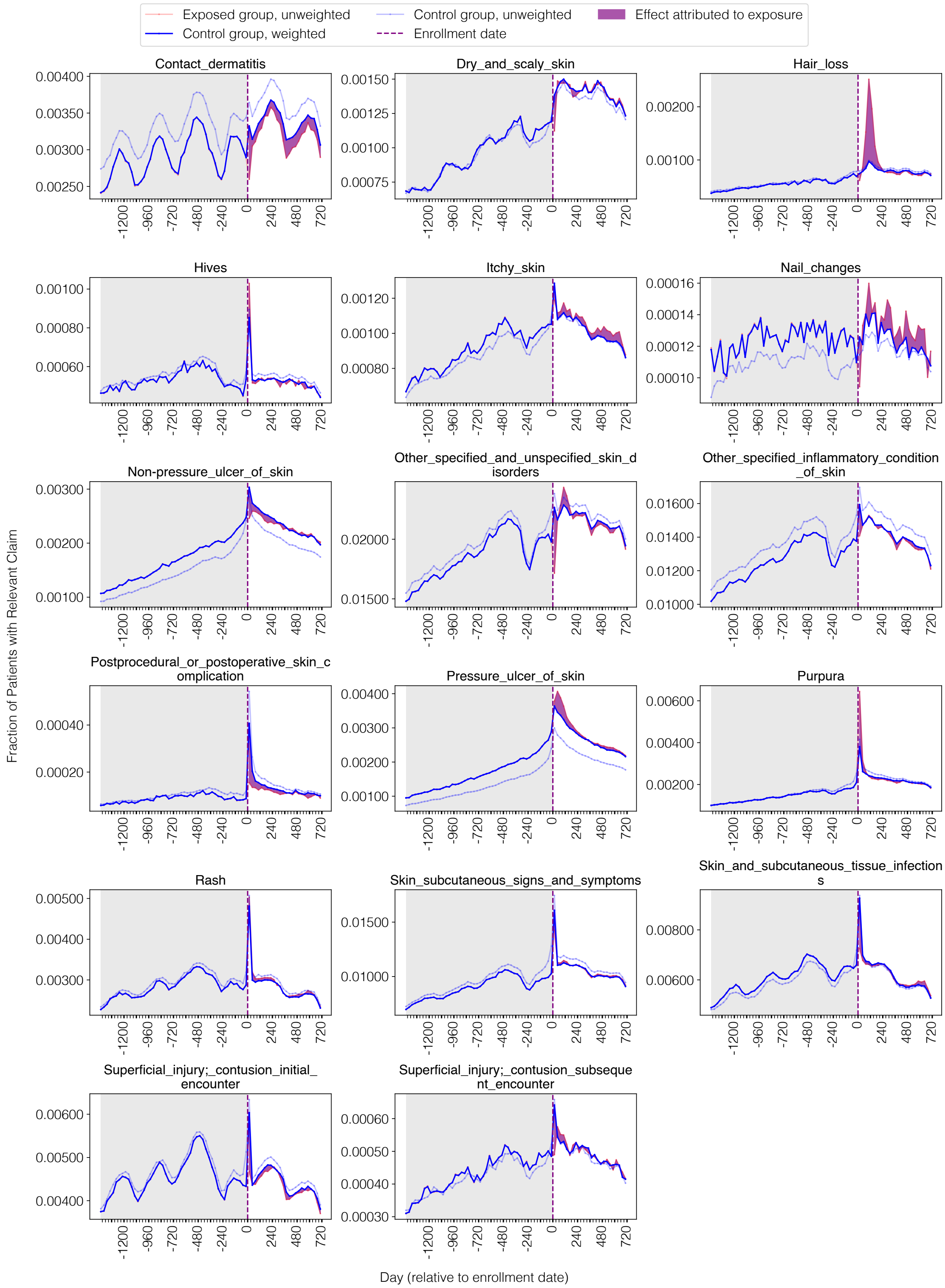


**Fig. S94.** Temporal trends in Hair, skin and nails prevalence. The fraction of patients with relevant claims over time belonging for each outcome in the **test-based control design** experiment.

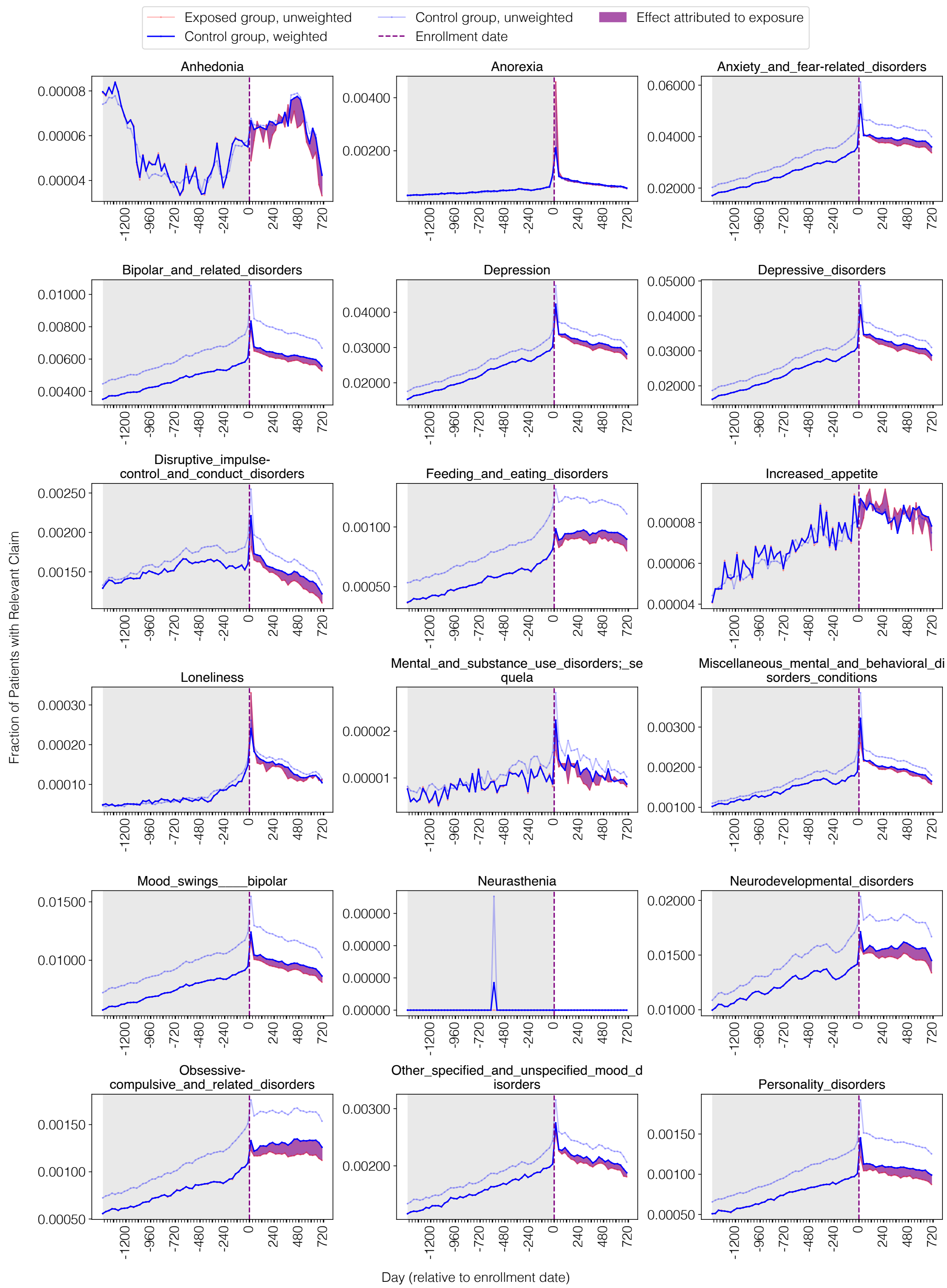


**Fig. S95.** Temporal trends in Mental health prevalence. The fraction of patients with relevant claims over time belonging for each outcome in the **test-based control design** experiment.

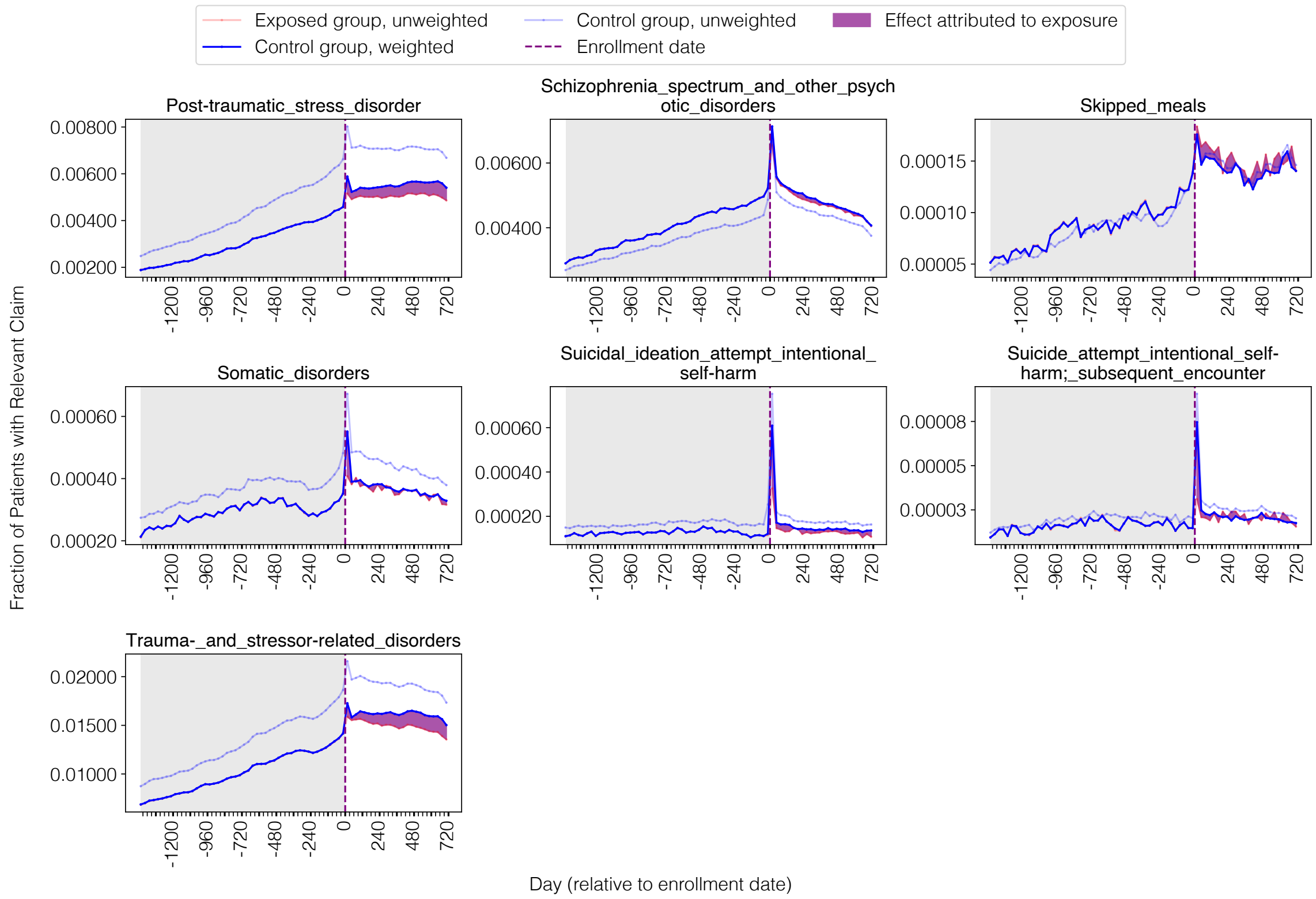


**Fig. S95.** (cont'd) Temporal trends in Mental health prevalence. The fraction of patients with relevant claims over time belonging for each outcome in the **test-based control design** experiment.

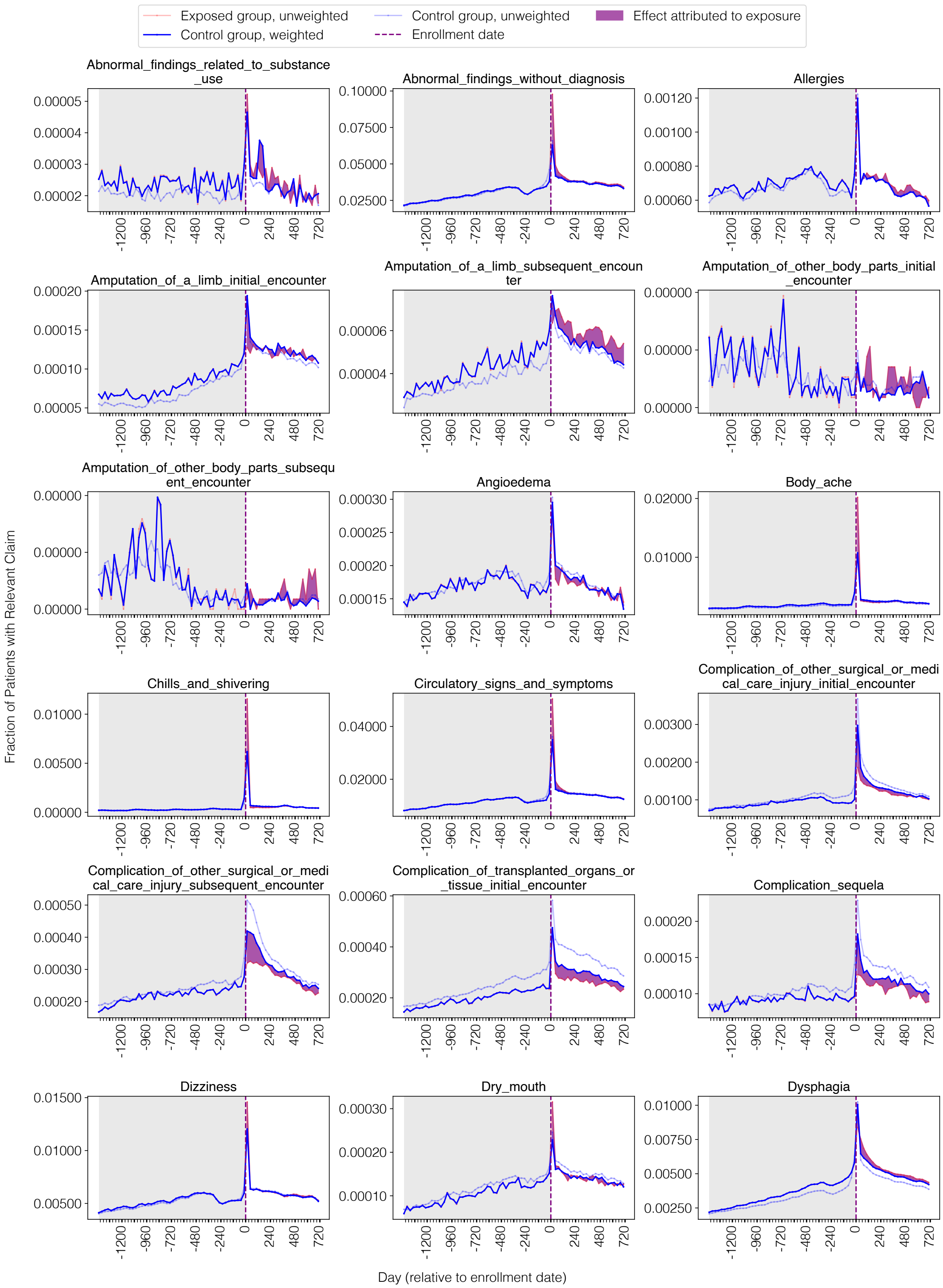


**Fig. S96.** Temporal trends in Misc prevalence. The fraction of patients with relevant claims over time belonging for each outcome in the **test-based control design** experiment.

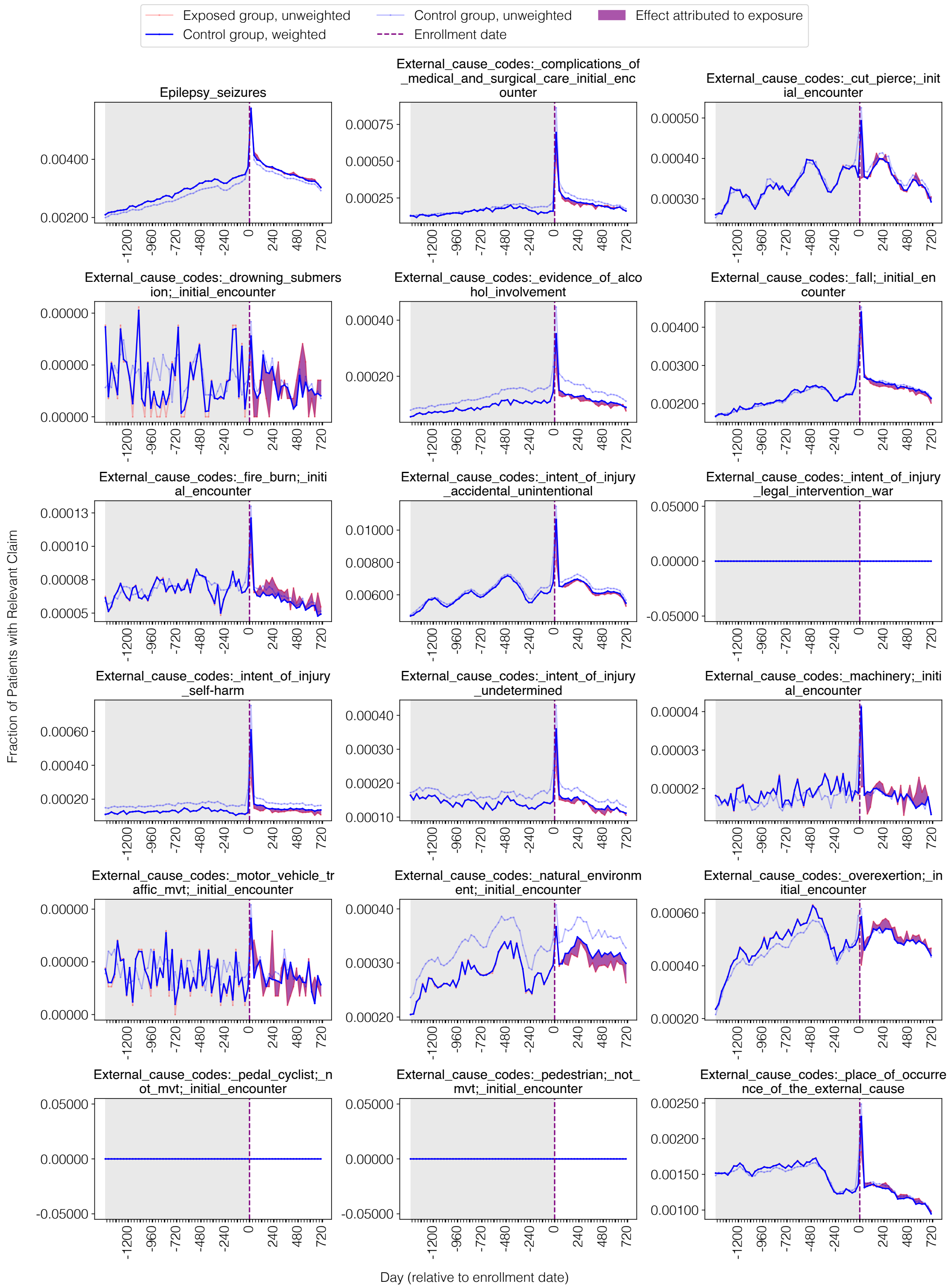


**Fig. S96.** (cont'd) Temporal trends in Misc prevalence. The fraction of patients with relevant claims over time belonging for each outcome in the **test-based control design** experiment.

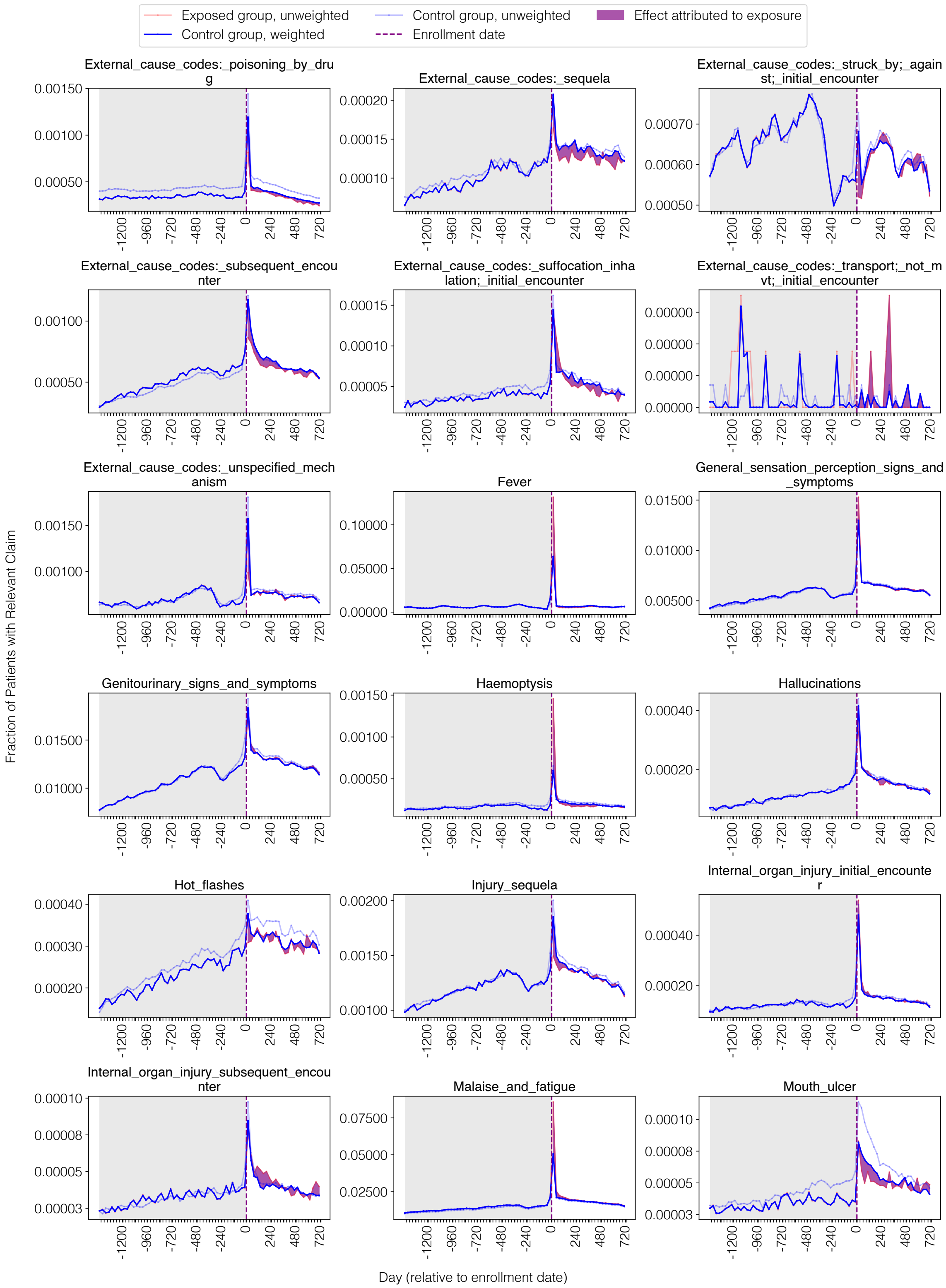


**Fig. S96.** (cont'd) Temporal trends in Misc prevalence. The fraction of patients with relevant claims over time belonging for each outcome in the **test-based control design** experiment.

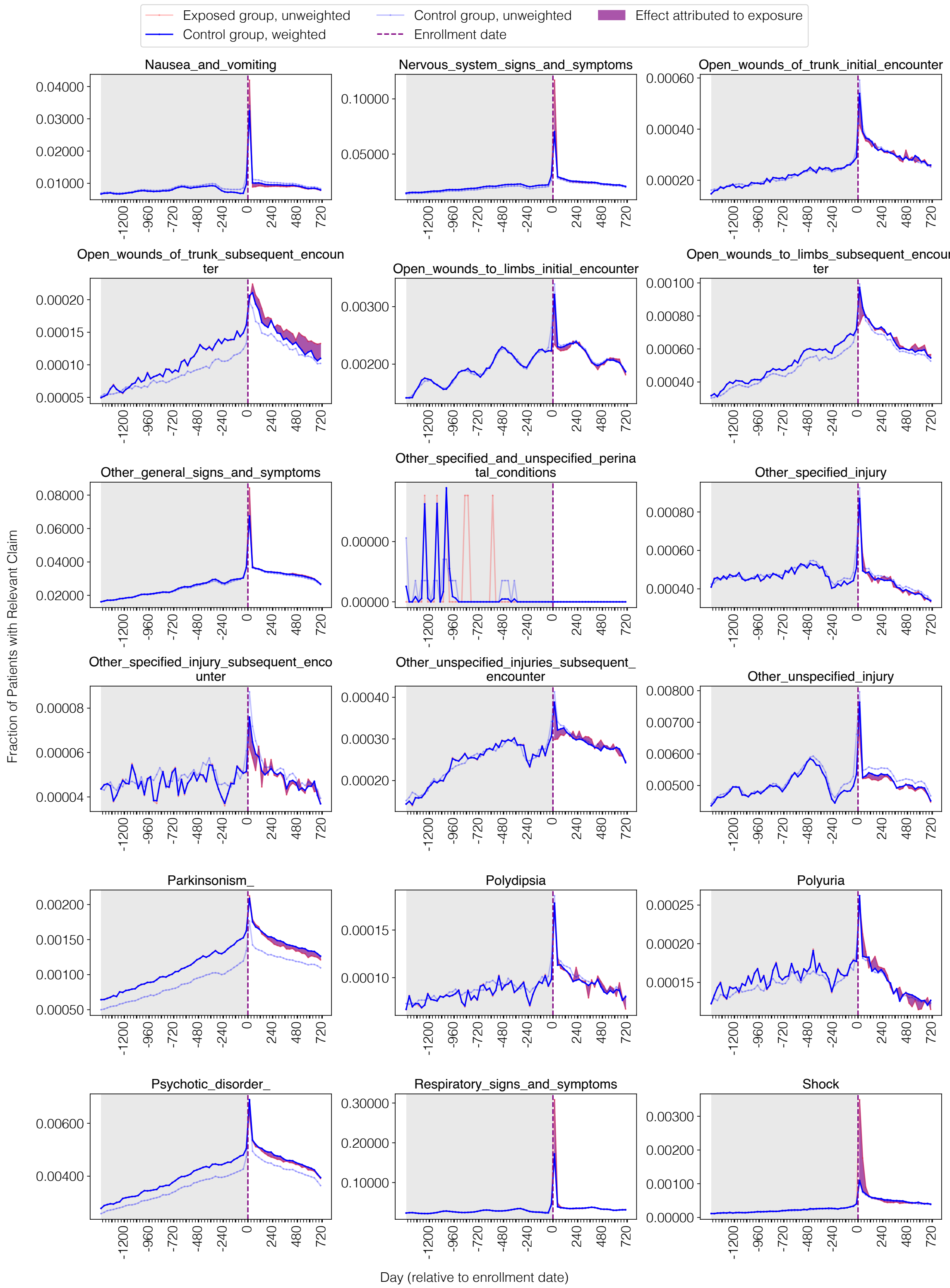


**Fig. S96.** (cont'd) Temporal trends in Misc prevalence. The fraction of patients with relevant claims over time belonging for each outcome in the **test-based control design** experiment.

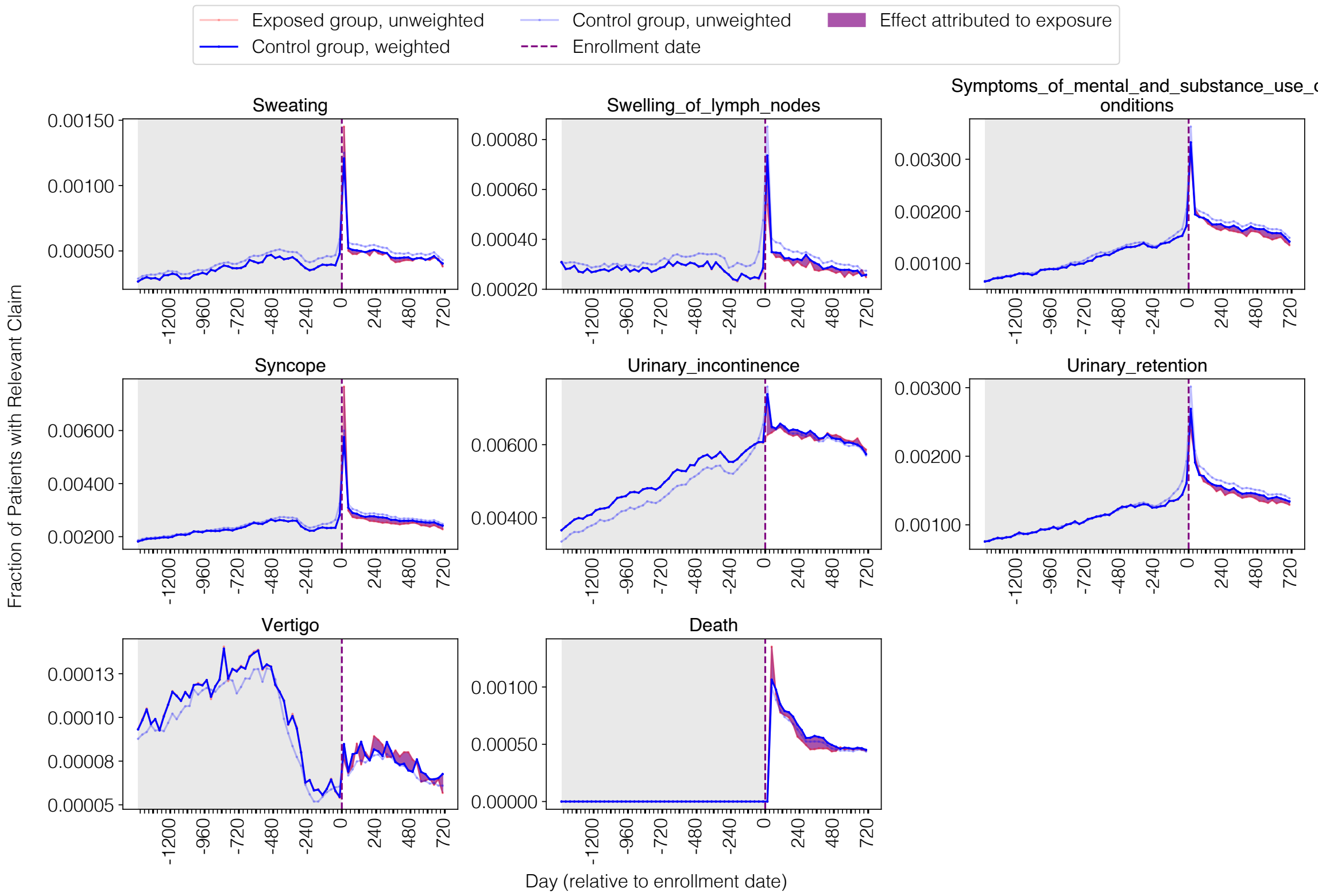


**Fig. S96.** (cont'd) Temporal trends in Misc prevalence. The fraction of patients with relevant claims over time belonging for each outcome in the **test-based control design** experiment.

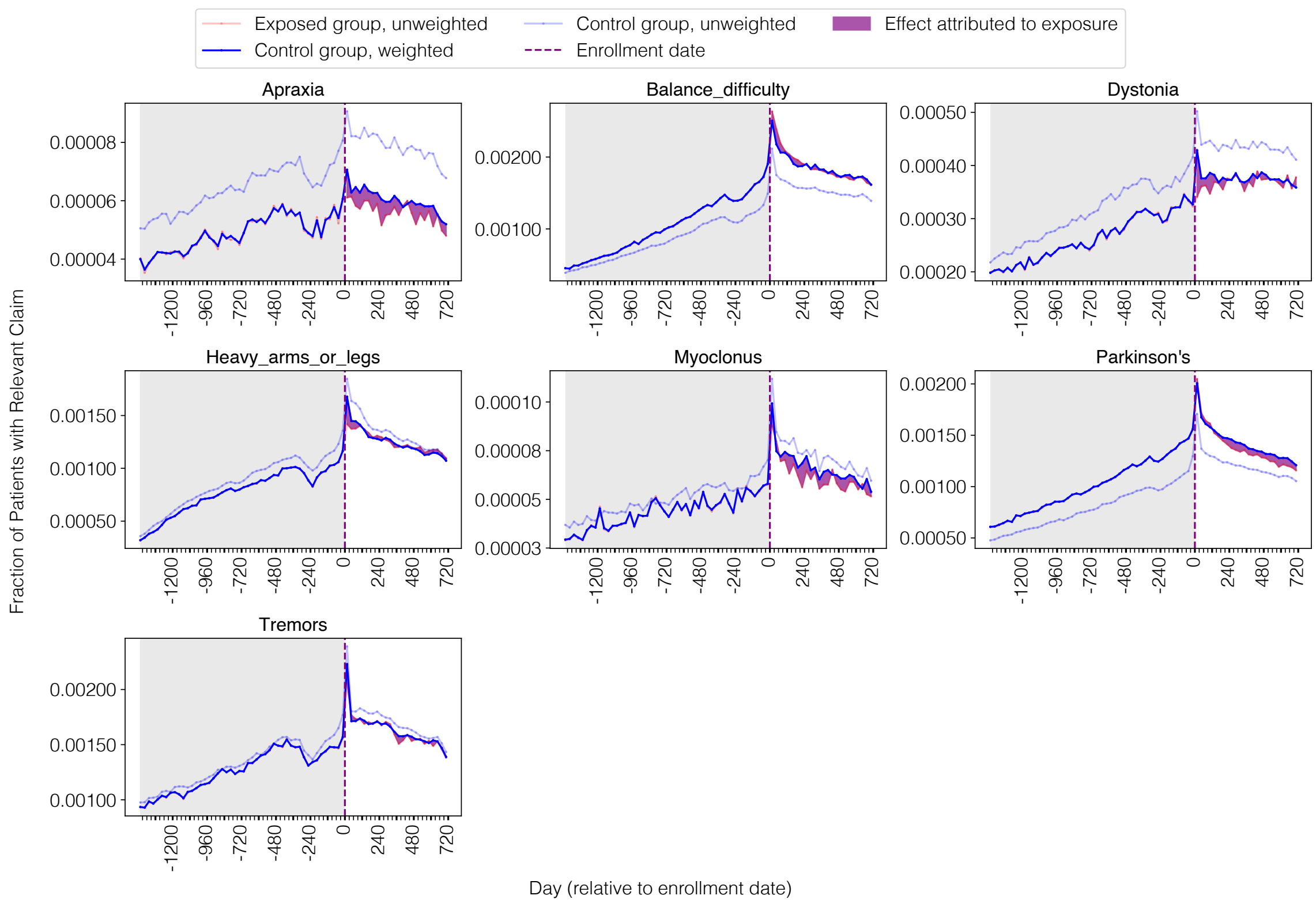


**Fig. S97.** Temporal trends in Movement prevalence. The fraction of patients with relevant claims over time belonging for each outcome in the **test-based control design** experiment.

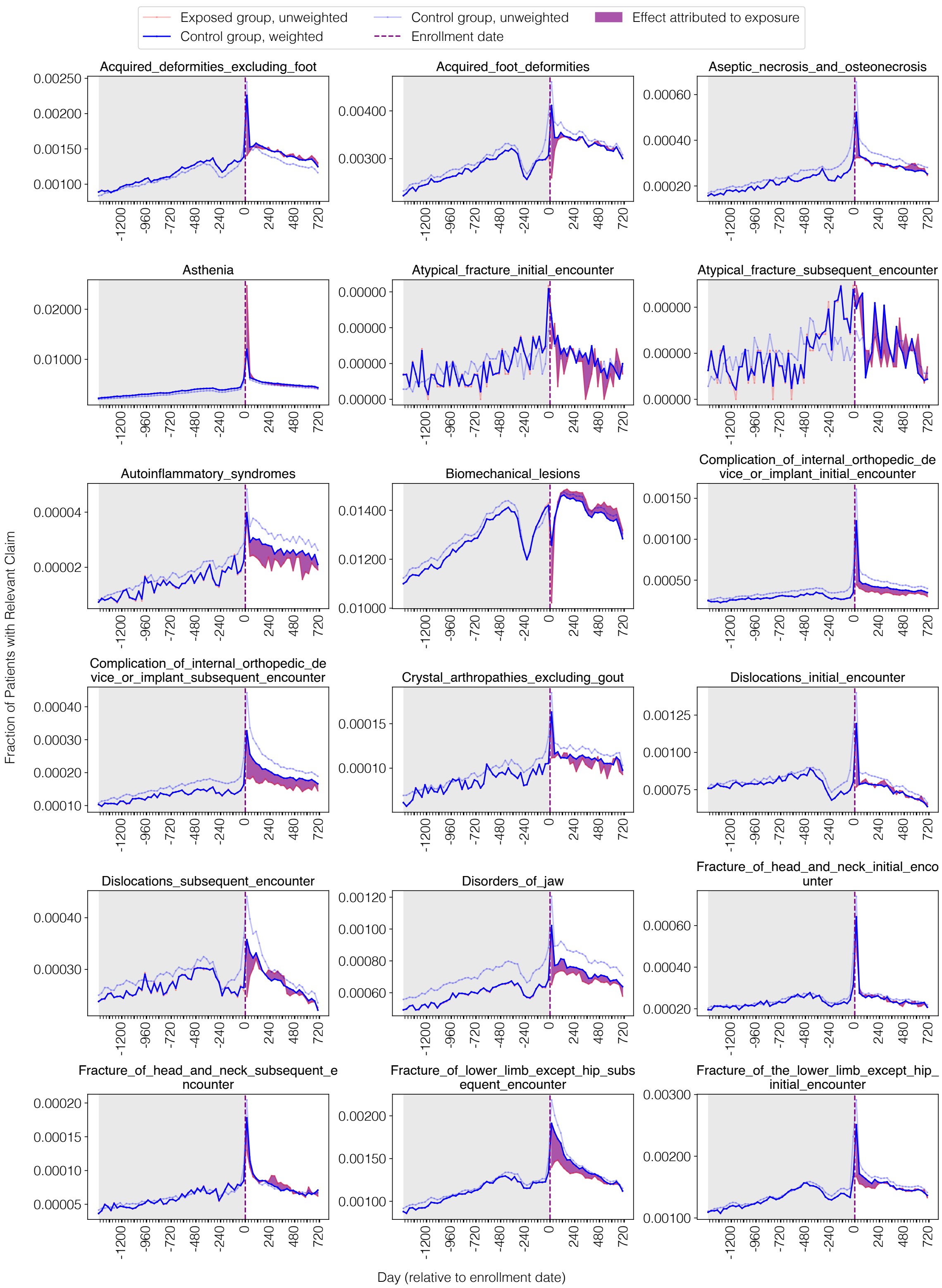


**Fig. S98.** Temporal trends in Muscles and joints prevalence. The fraction of patients with relevant claims over time belonging for each outcome in the **test-based control design** experiment.

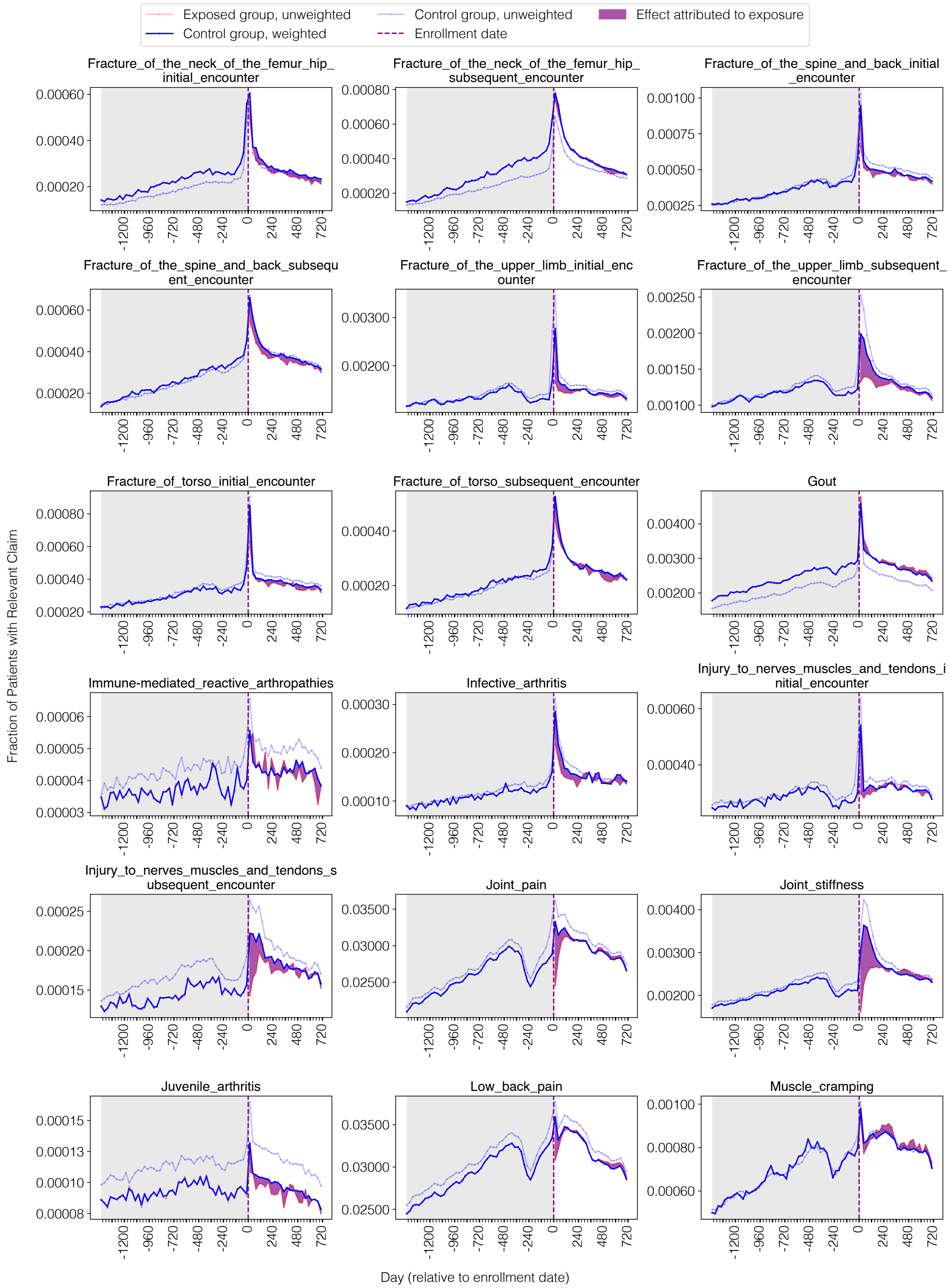


**Fig. S98.** (cont'd) Temporal trends in Muscles and joints prevalence. The fraction of patients with relevant claims over time belonging for each outcome in the **test-based control design** experiment.

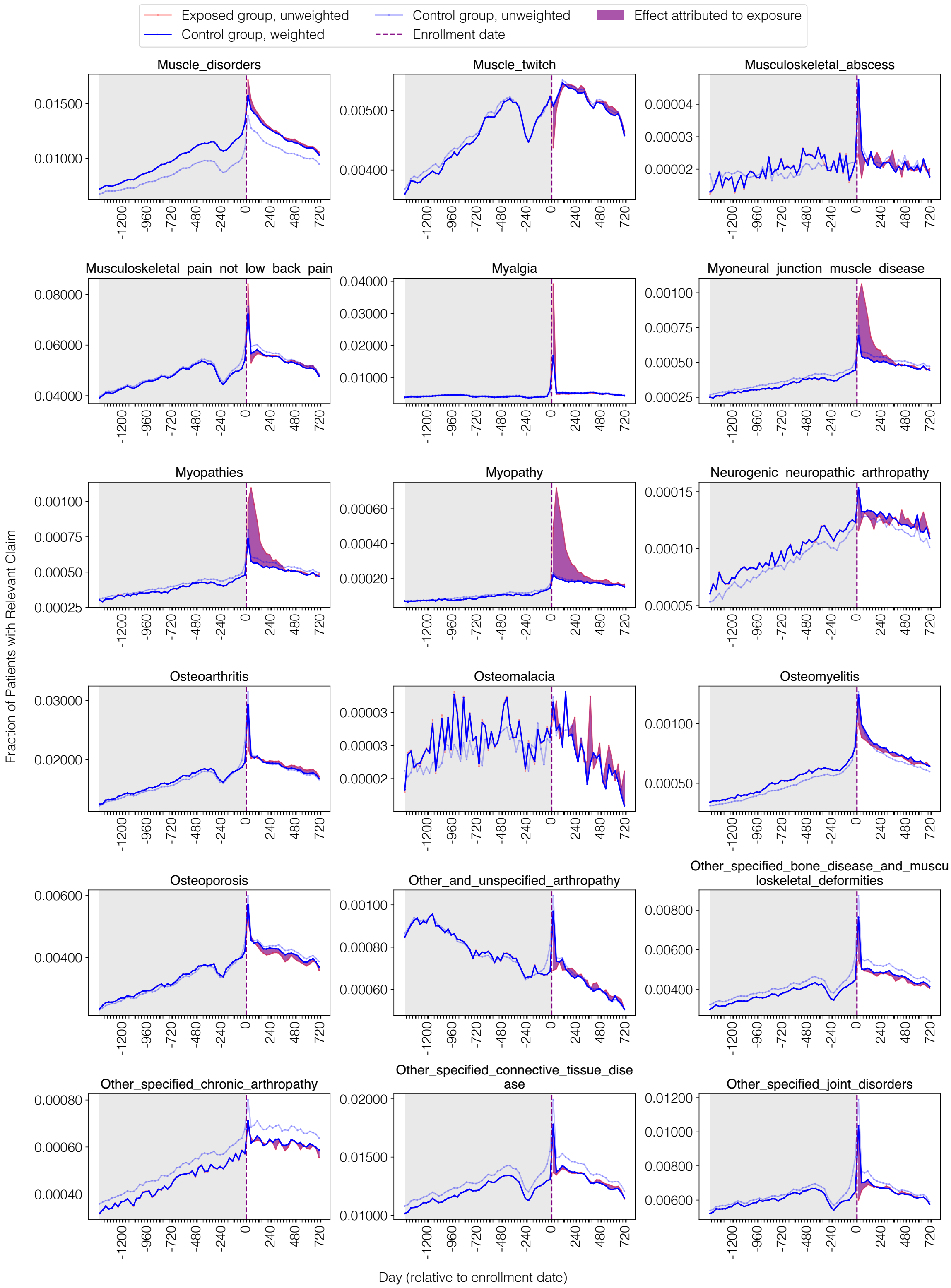


**Fig. S98.** (cont'd) Temporal trends in Muscles and joints prevalence. The fraction of patients with relevant claims over time belonging for each outcome in the **test-based control design** experiment.

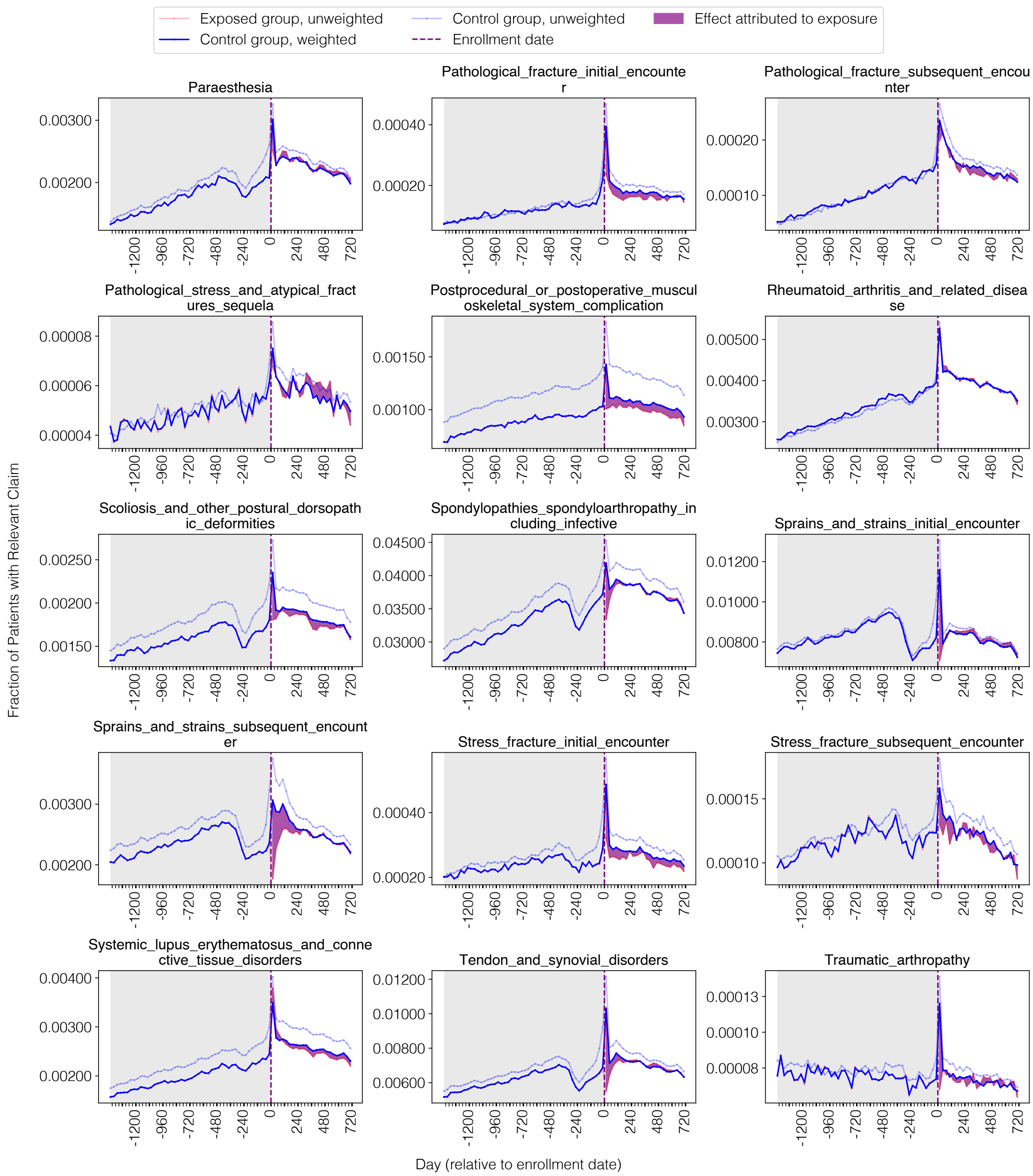


**Fig. S98.** (cont'd) Temporal trends in Muscles and joints prevalence. The fraction of patients with relevant claims over time belonging for each outcome in the **test-based control design** experiment.

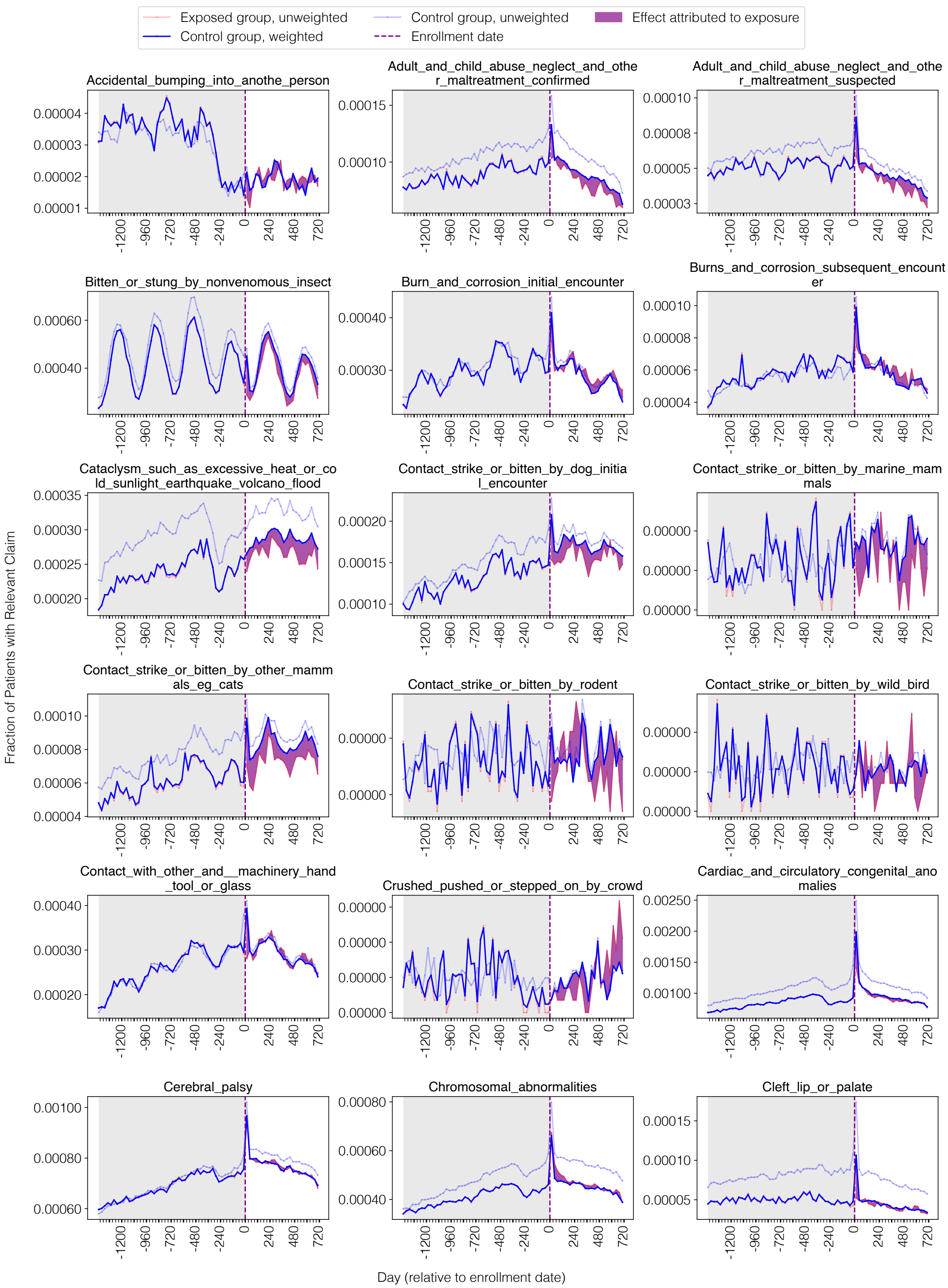


**Fig. S99.** Temporal trends in Negative prevalence. The fraction of patients with relevant claims over time belonging for each outcome in the **test-based control design** experiment.

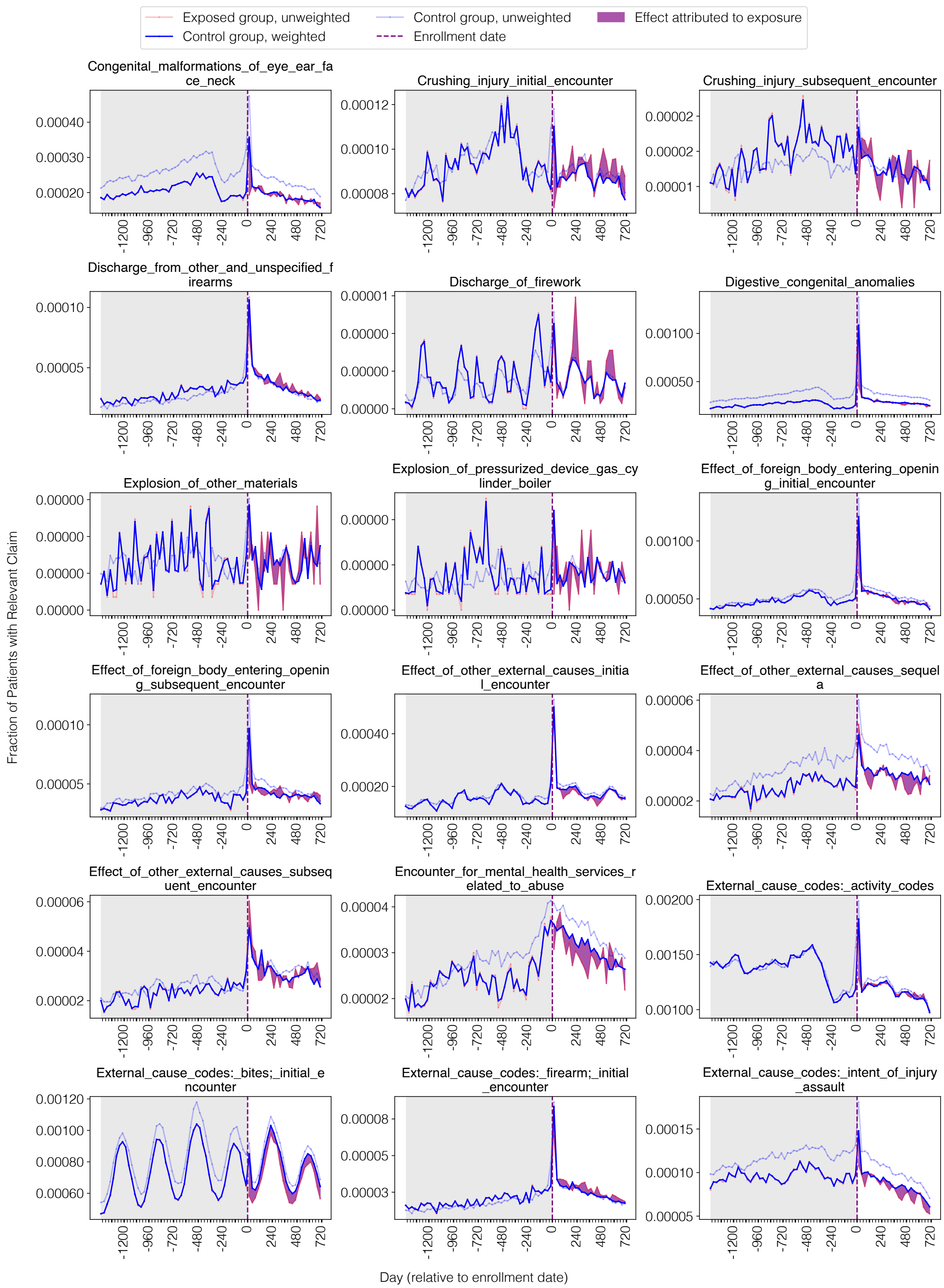


**Fig. S99.** (cont'd) Temporal trends in Negative prevalence. The fraction of patients with relevant claims over time belonging for each outcome in the **test-based control design** experiment.

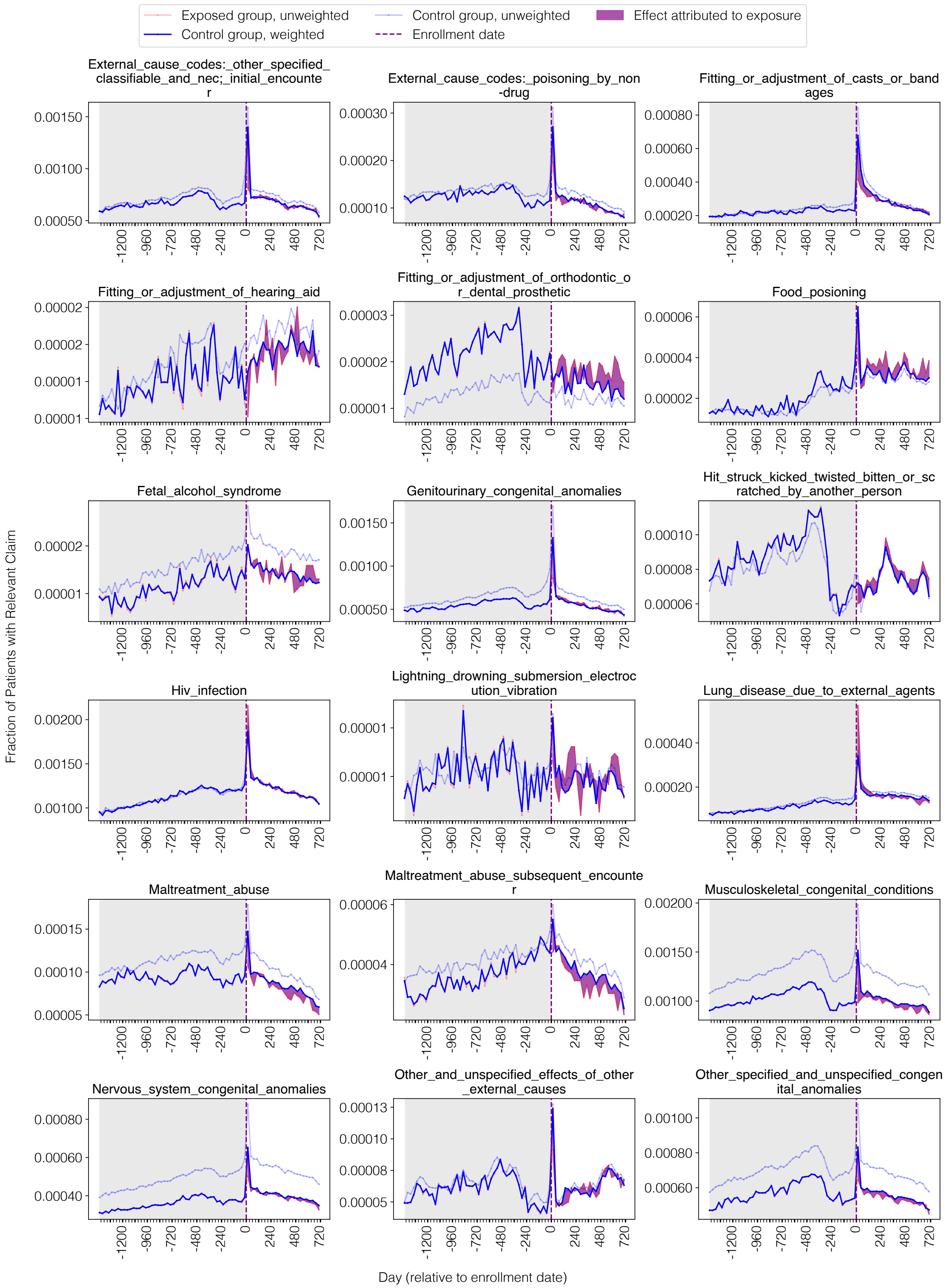


**Fig. S99.** (cont'd) Temporal trends in Negative prevalence. The fraction of patients with relevant claims over time belonging for each outcome in the **test-based control design** experiment.

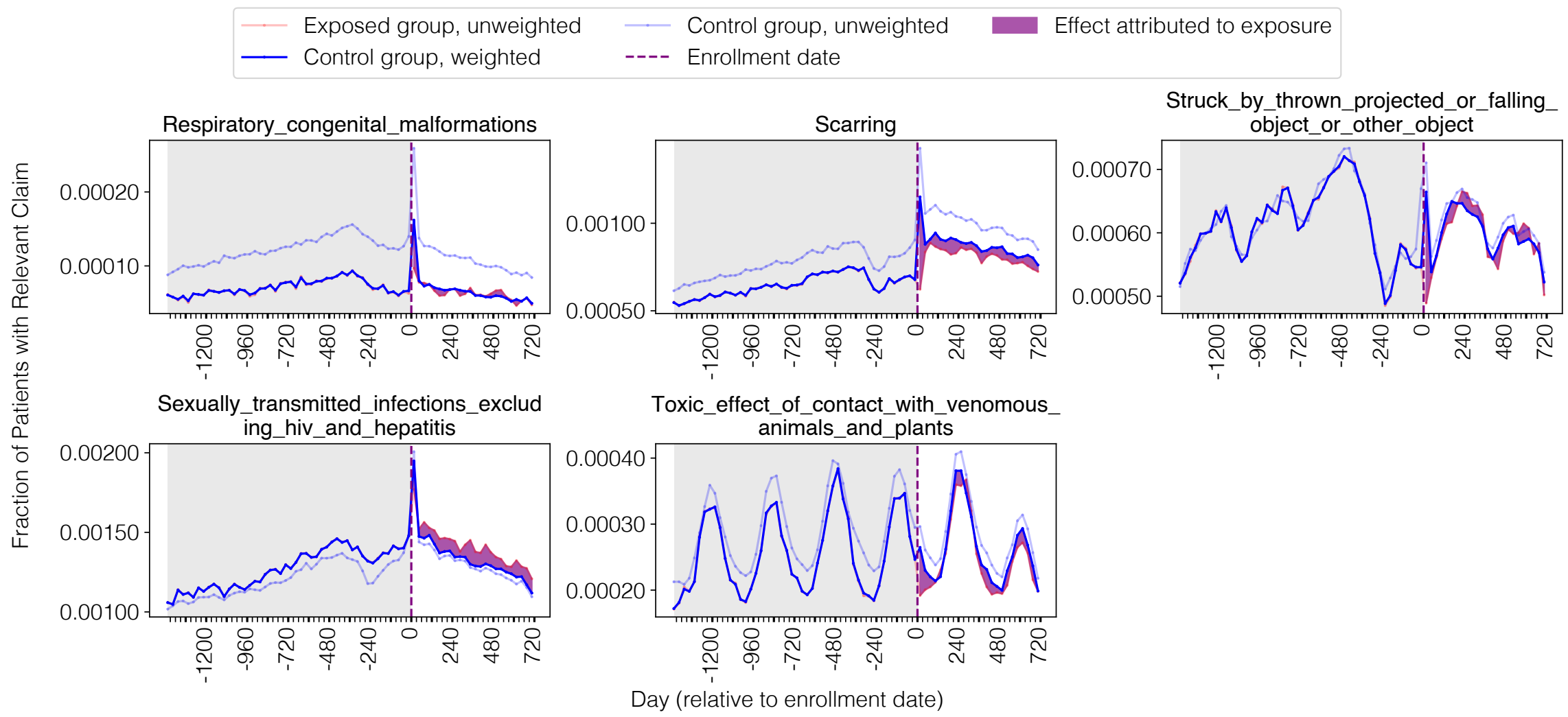


**Fig. S99.** (cont'd) Temporal trends in Negative prevalence. The fraction of patients with relevant claims over time belonging for each outcome in the **test-based control design** experiment.

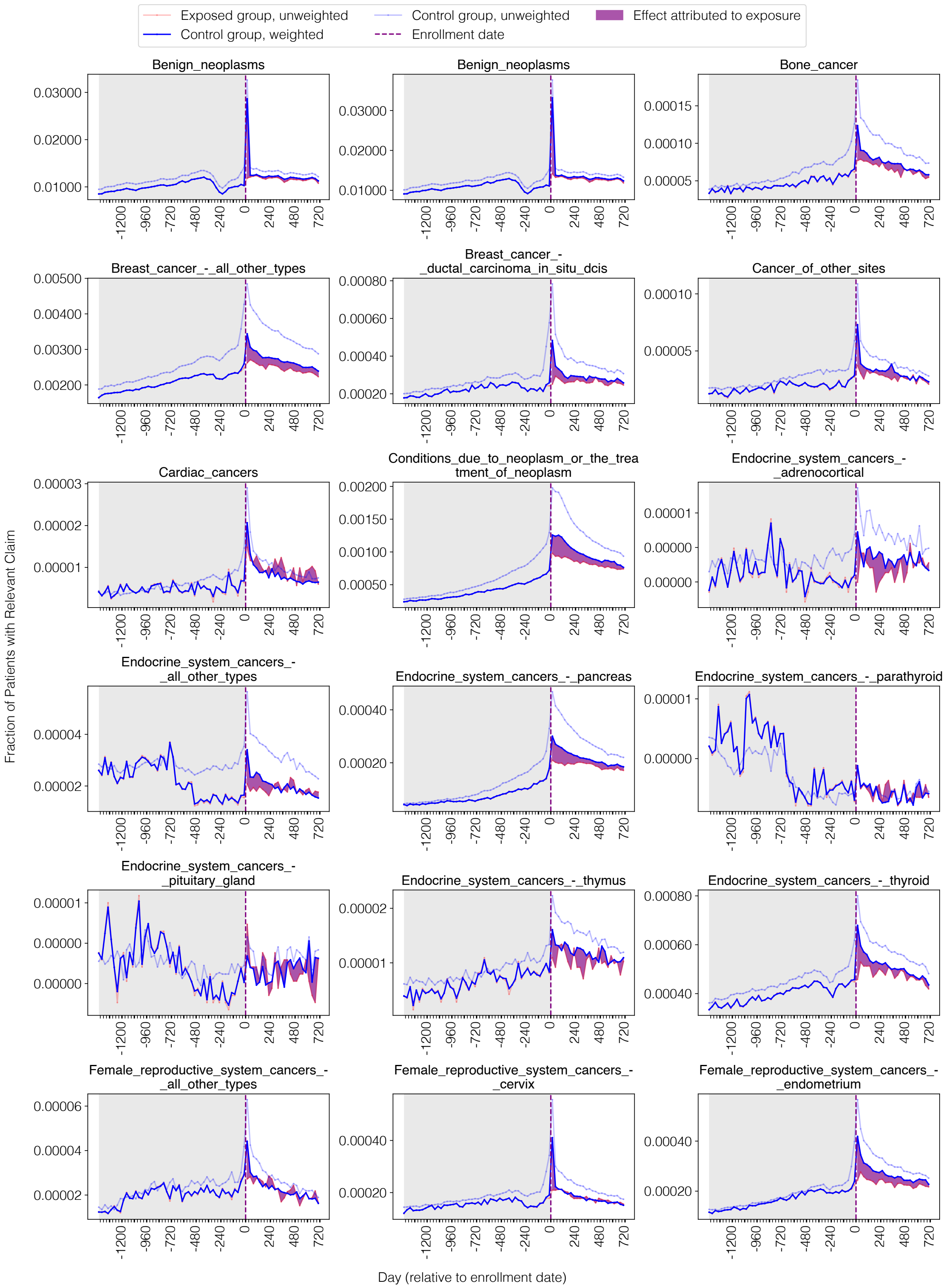


**Fig. S100.** Temporal trends in Neoplasms prevalence. The fraction of patients with relevant claims over time belonging for each outcome in the **test-based control design** experiment.

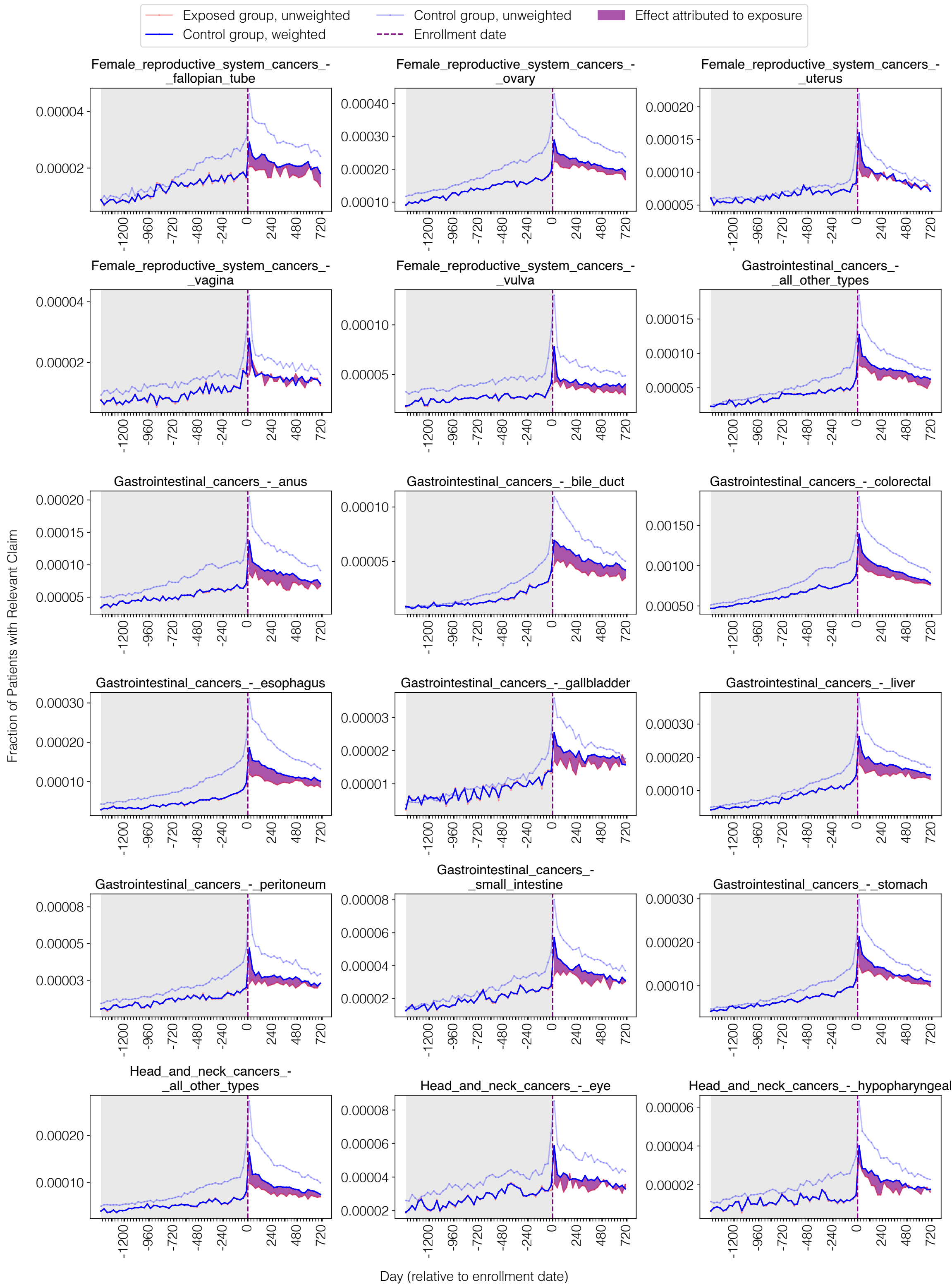


**Fig. S100.** (cont'd) Temporal trends in Neoplasms prevalence. The fraction of patients with relevant claims over time belonging for each outcome in the **test-based control design** experiment.

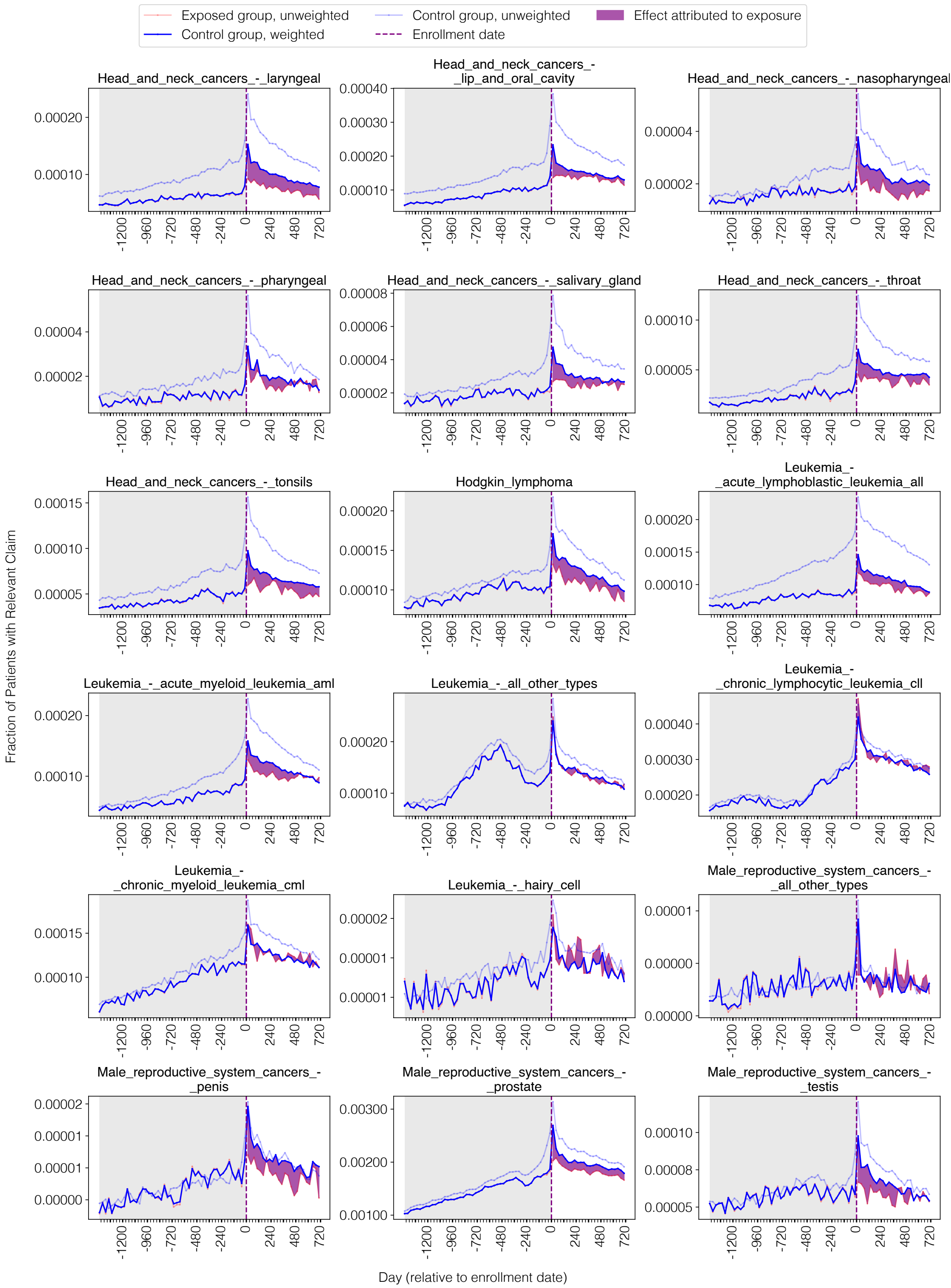


**Fig. S100.** (cont'd) Temporal trends in Neoplasms prevalence. The fraction of patients with relevant claims over time belonging for each outcome in the **test-based control design** experiment.

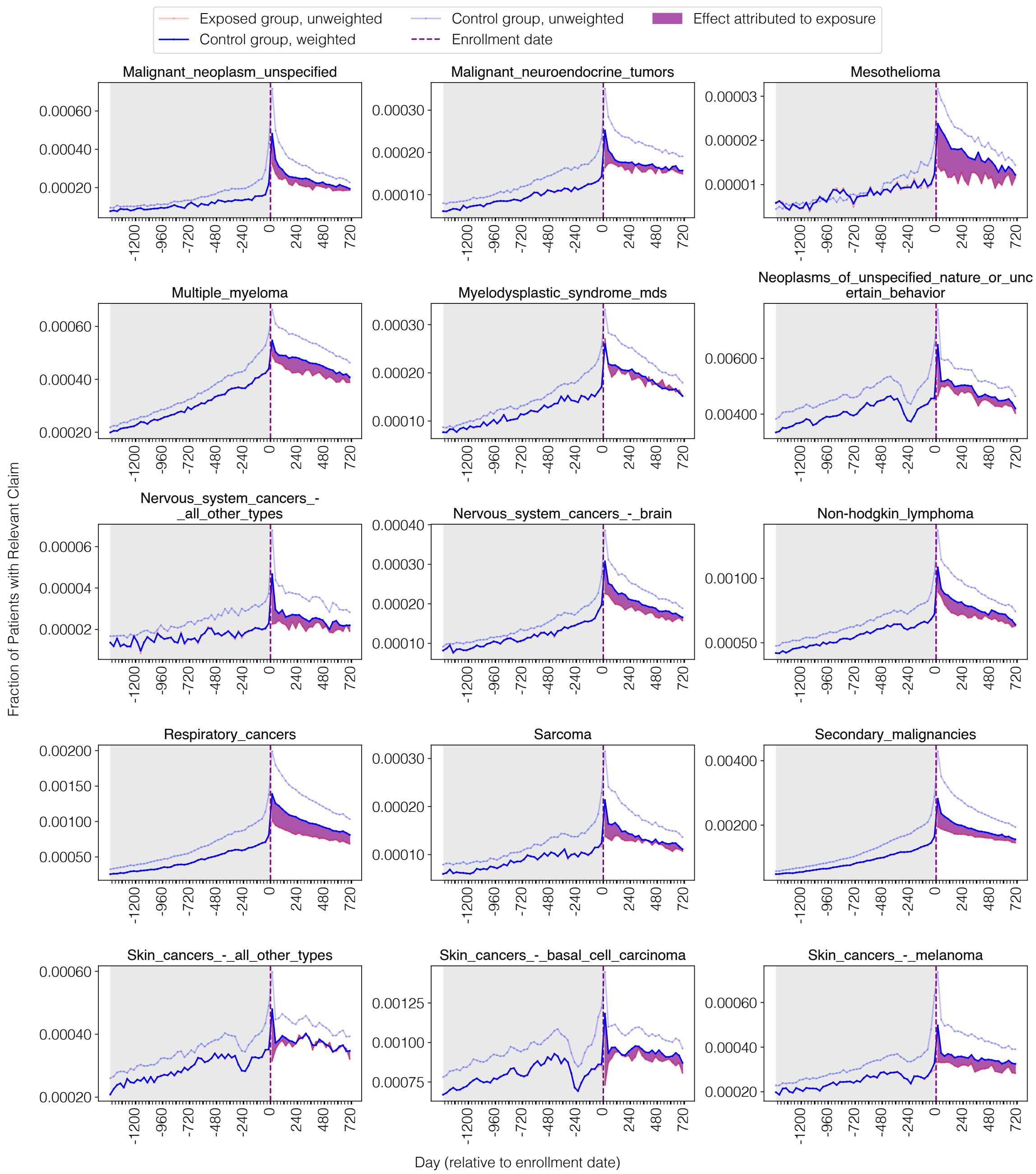


**Fig. S100.** (cont'd) Temporal trends in Neoplasms prevalence. The fraction of patients with relevant claims over time belonging for each outcome in the **test-based control design** experiment.

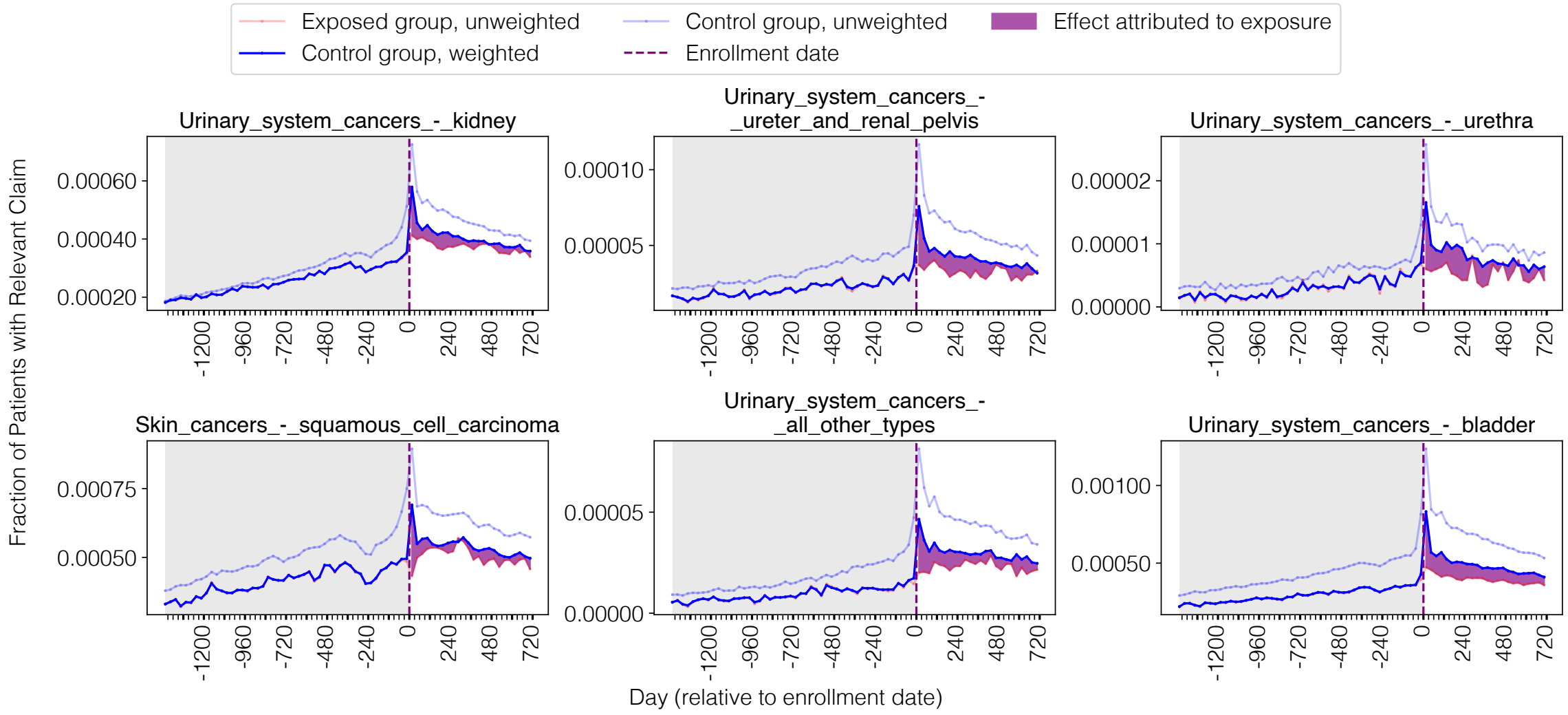


**Fig. S100.** (cont'd) Temporal trends in Neoplasms prevalence. The fraction of patients with relevant claims over time belonging for each outcome in the **test-based control design** experiment.

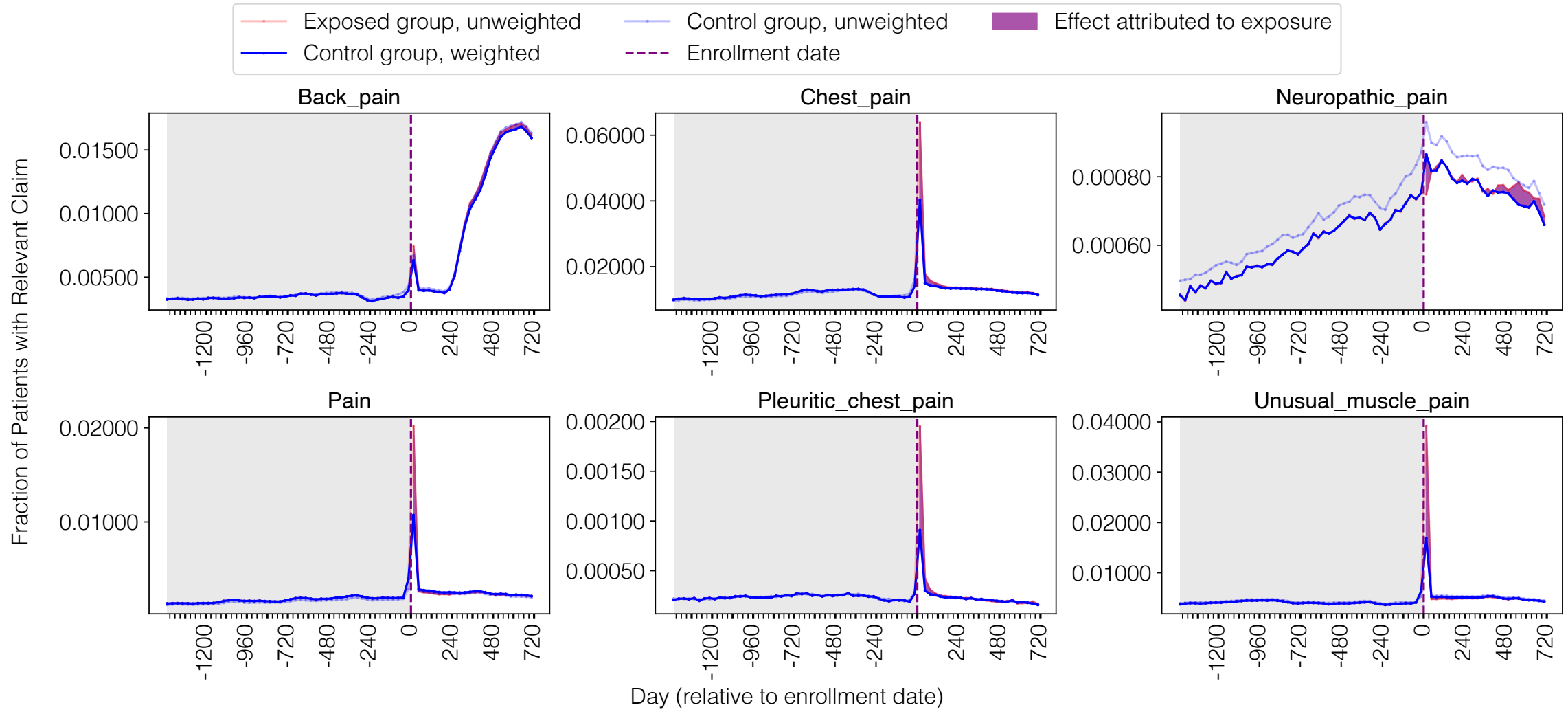


**Fig. S101.** Temporal trends in Pain prevalence. The fraction of patients with relevant claims over time belonging for each outcome in the **test-based control design** experiment.

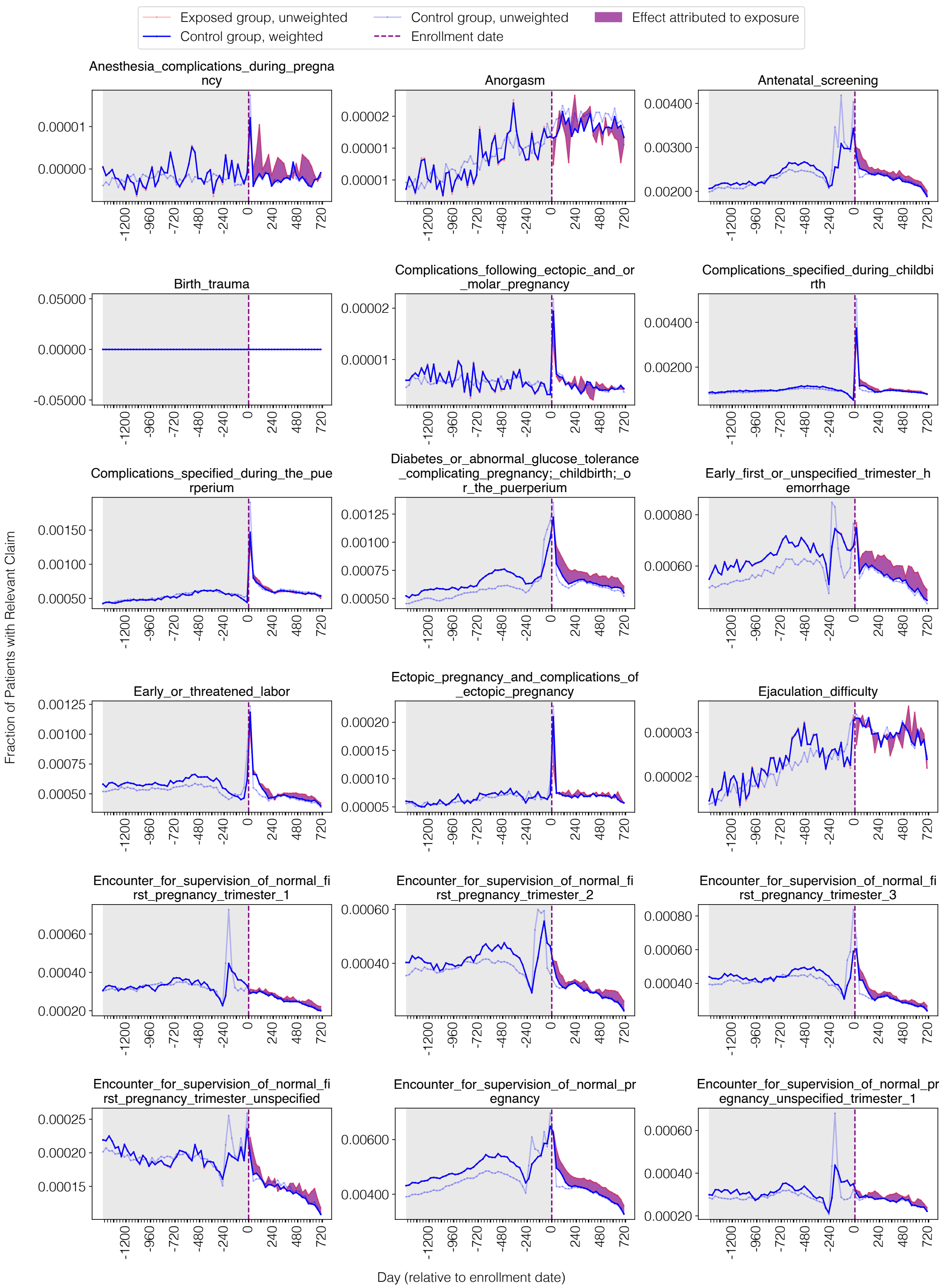


**Fig. S102.** Temporal trends in Reproductive health prevalence. The fraction of patients with relevant claims over time belonging for each outcome in the **test-based control design** experiment.

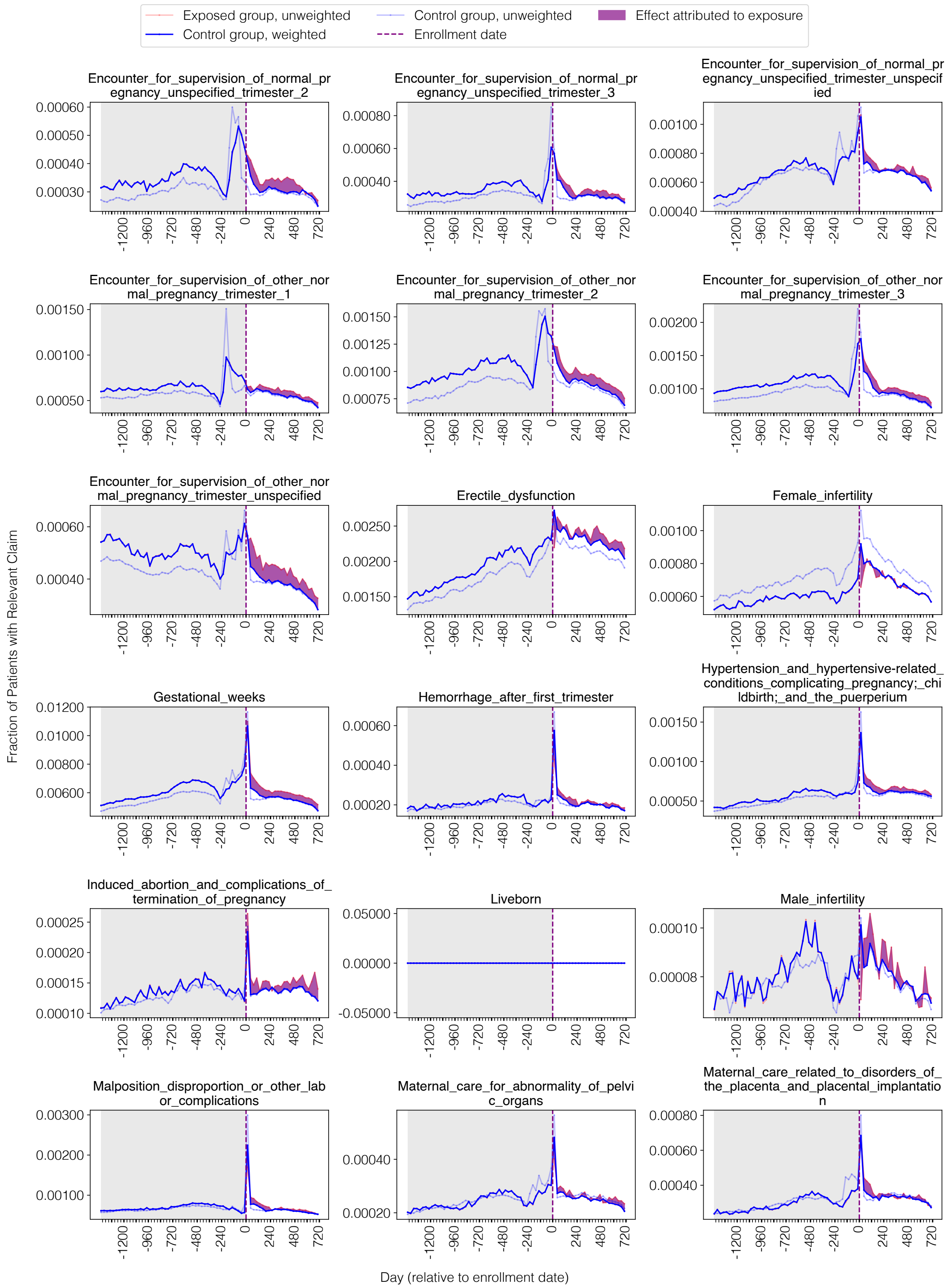


**Fig. S102.** (cont'd) Temporal trends in Reproductive health prevalence. The fraction of patients with relevant claims over time belonging for each outcome in the **test-based control design** experiment.

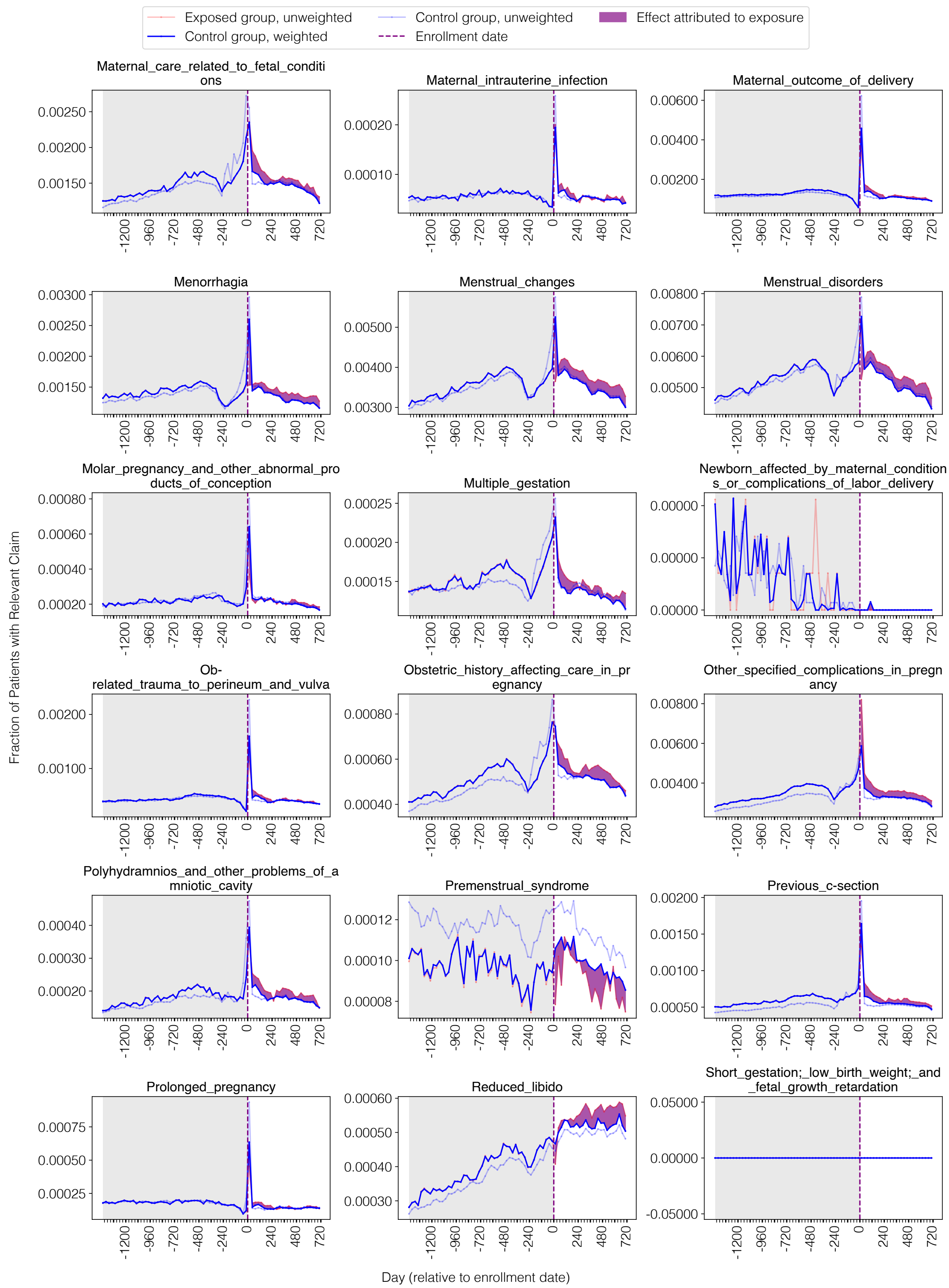


**Fig. S102.** (cont'd) Temporal trends in Reproductive health prevalence. The fraction of patients with relevant claims over time belonging for each outcome in the **test-based control design** experiment.

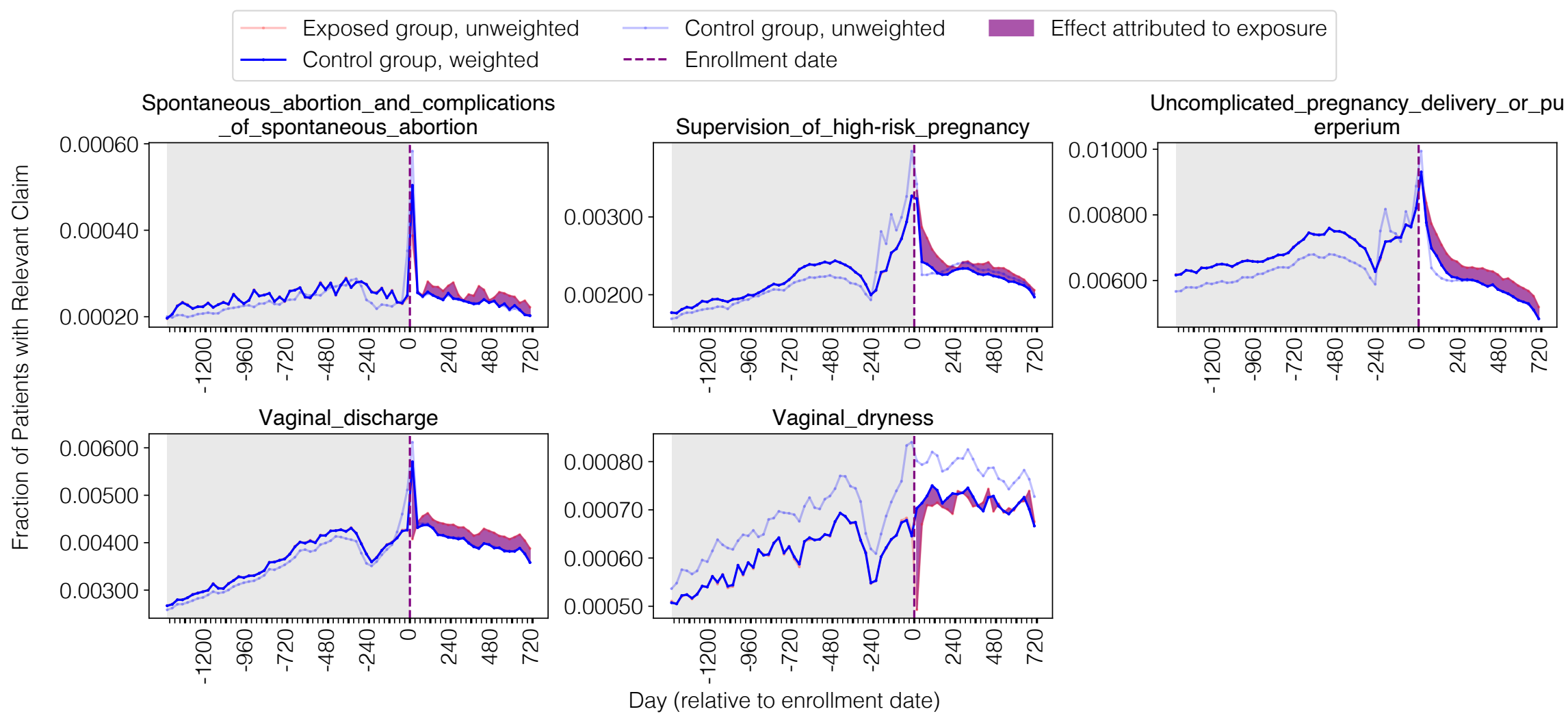


**Fig. S102.** (cont'd) Temporal trends in Reproductive health prevalence. The fraction of patients with relevant claims over time belonging for each outcome in the **test-based control design** experiment.

 

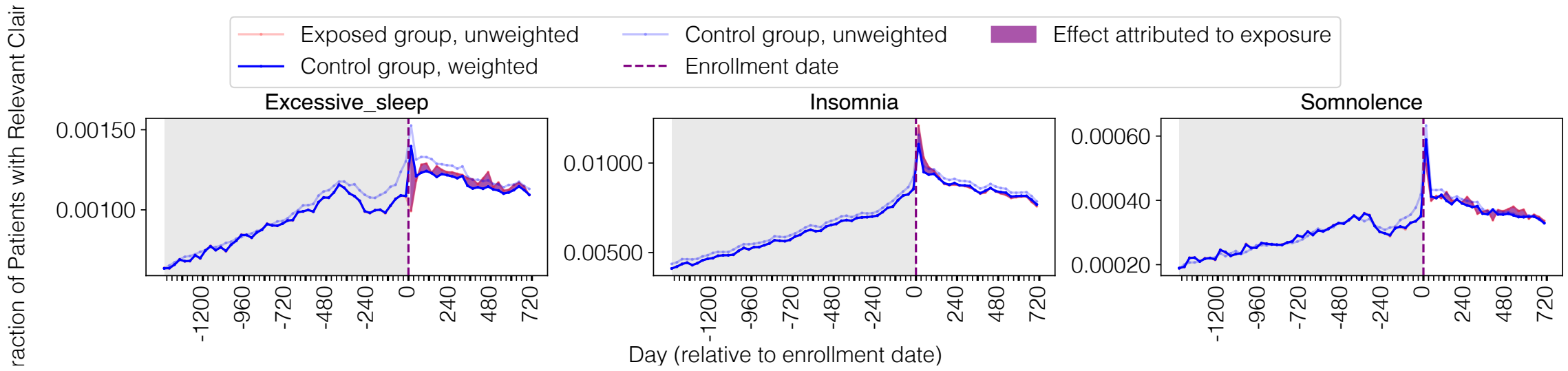


**Fig. S103.** Temporal trends in Sleep prevalence. The fraction of patients with relevant claims over time belonging for each outcome in the **test-based control design** experiment.

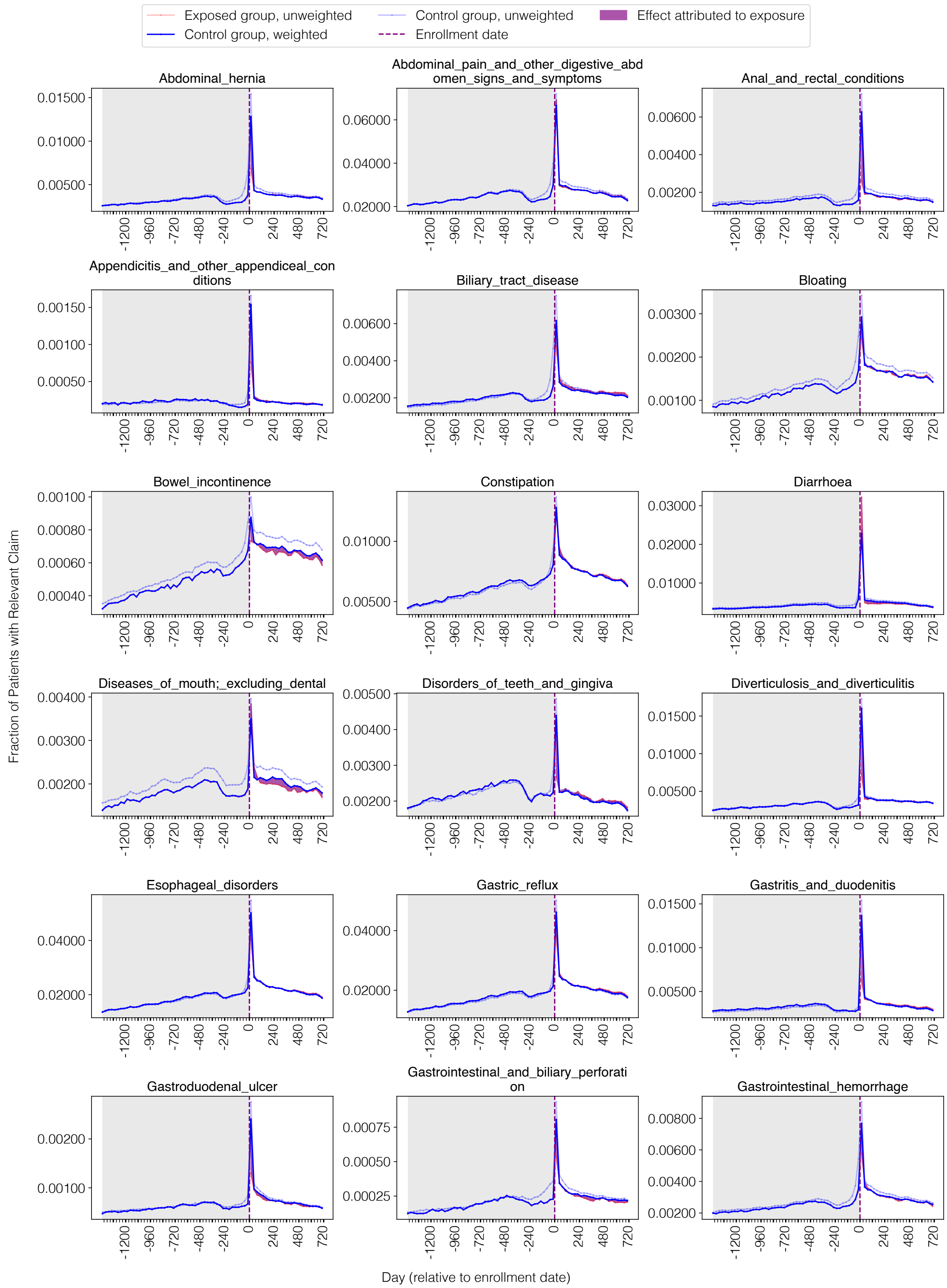


**Fig. S104.** Temporal trends in Stomach and digestion prevalence. The fraction of patients with relevant claims over time belonging for each outcome in the **test-based control design** experiment.

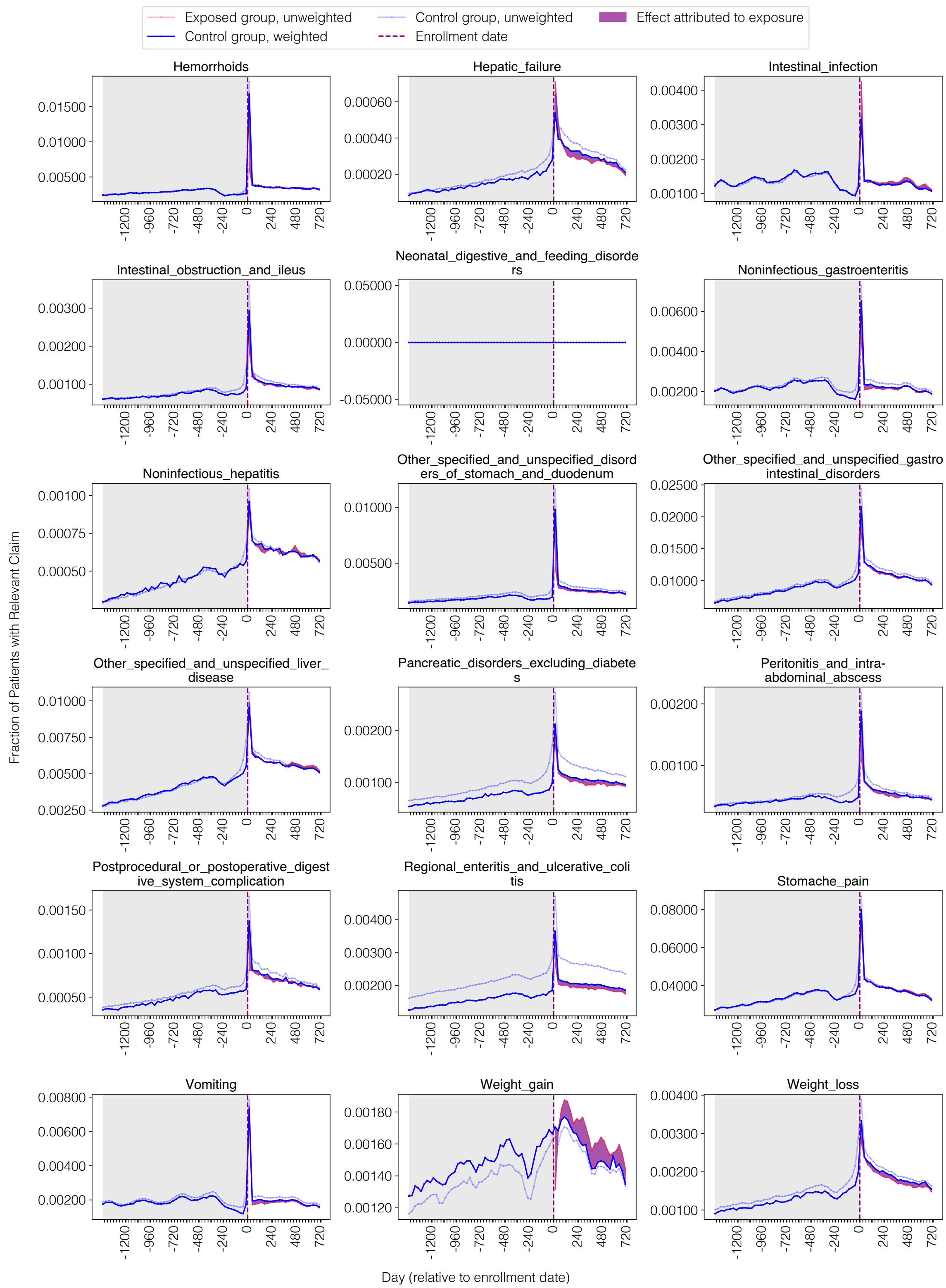


**Fig. S104.** (cont'd) Temporal trends in Stomach and digestion prevalence. The fraction of patients with relevant claims over time belonging for each outcome in the **test-based control design** experiment.

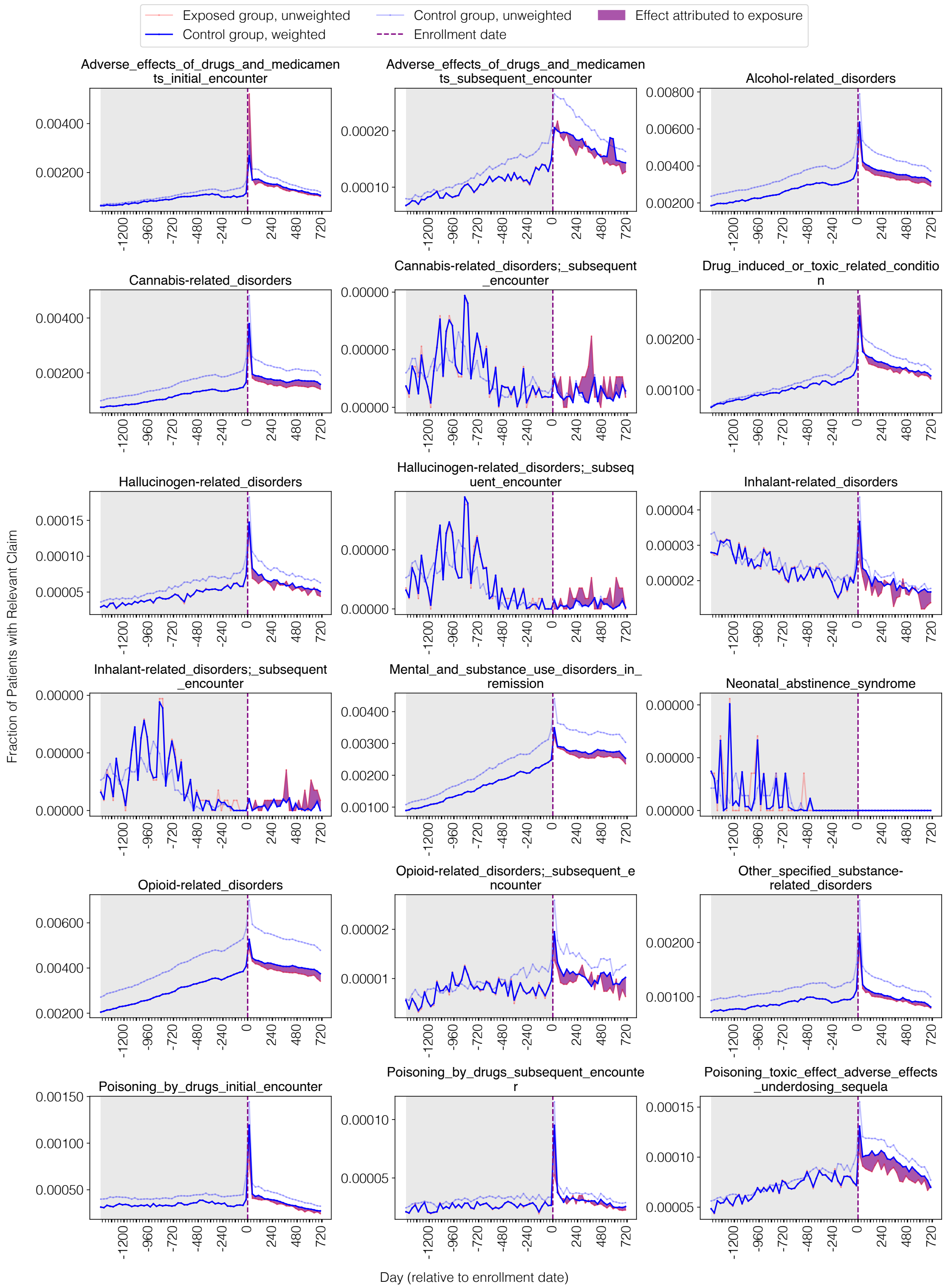


**Fig. S105.** Temporal trends in Substance use prevalence. The fraction of patients with relevant claims over time belonging for each outcome in the **test-based control design** experiment.

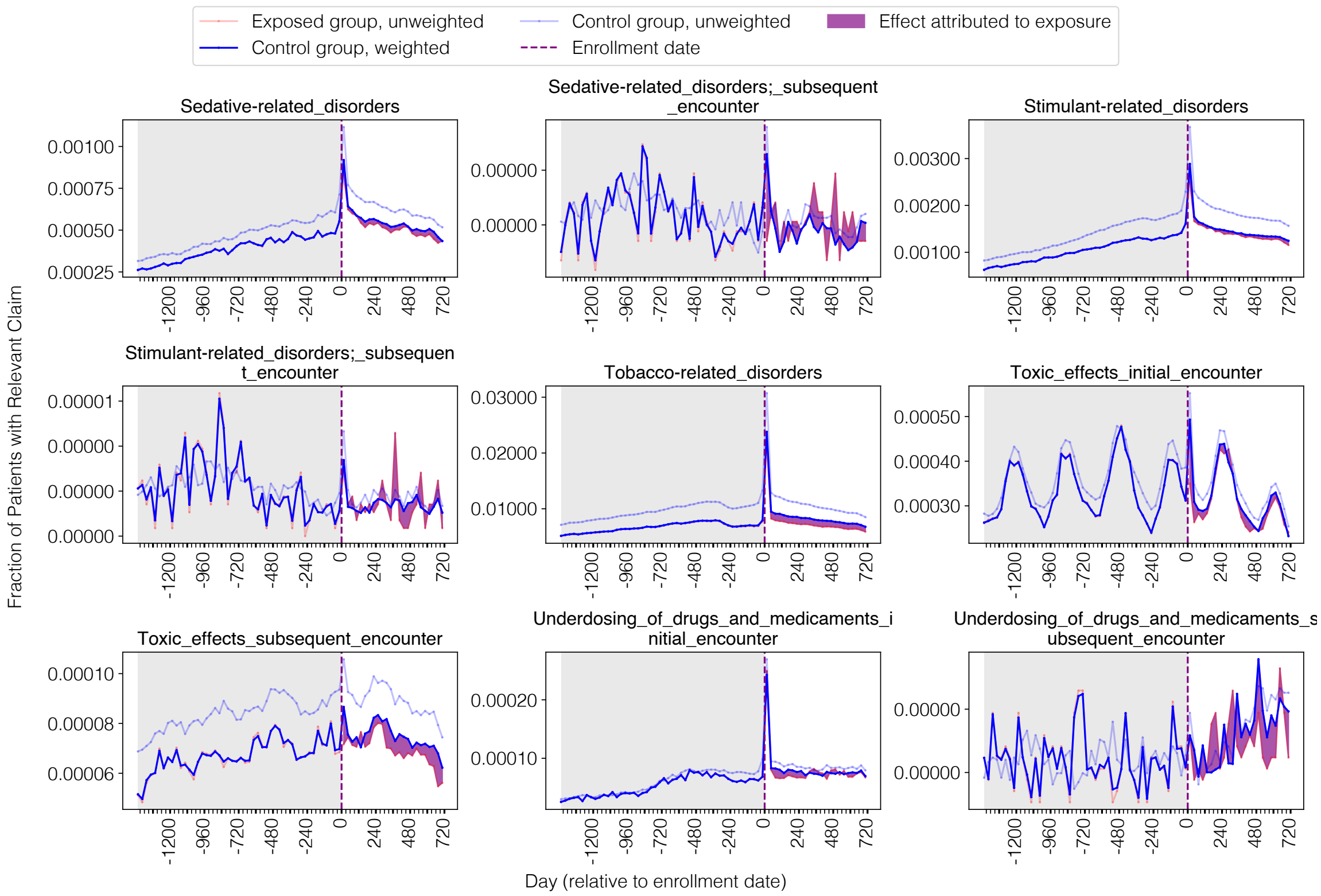


**Fig. S105.** (cont'd) Temporal trends in Substance use prevalence. The fraction of patients with relevant claims over time belonging for each outcome in the **test-based control design** experiment.

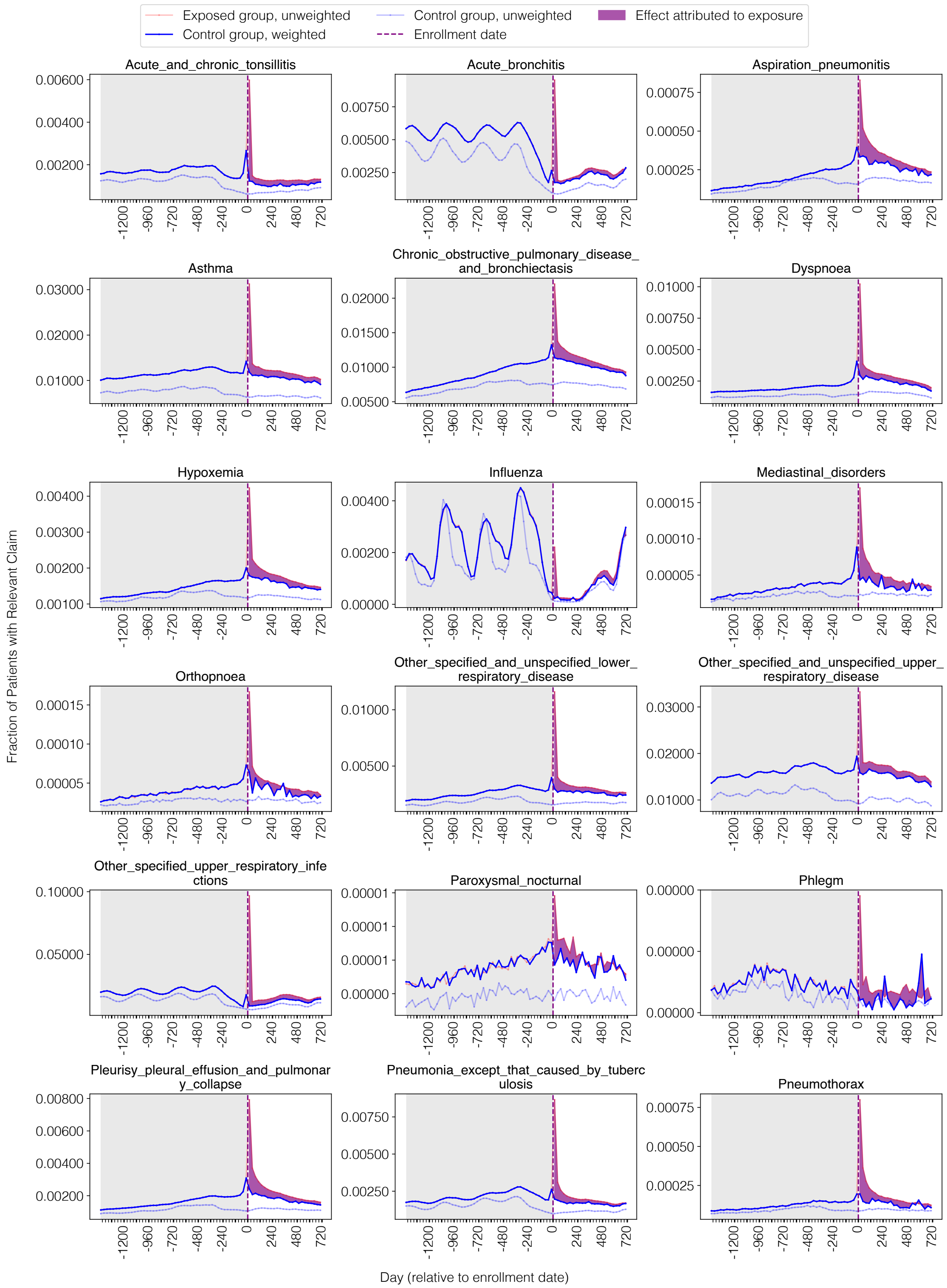


**Fig. S106.** Temporal trends in Breathing prevalence. The fraction of patients with relevant claims over time belonging for each outcome in the **unrestricted control design with placebo cases** (negative COVID-19 PCR test) experiment.

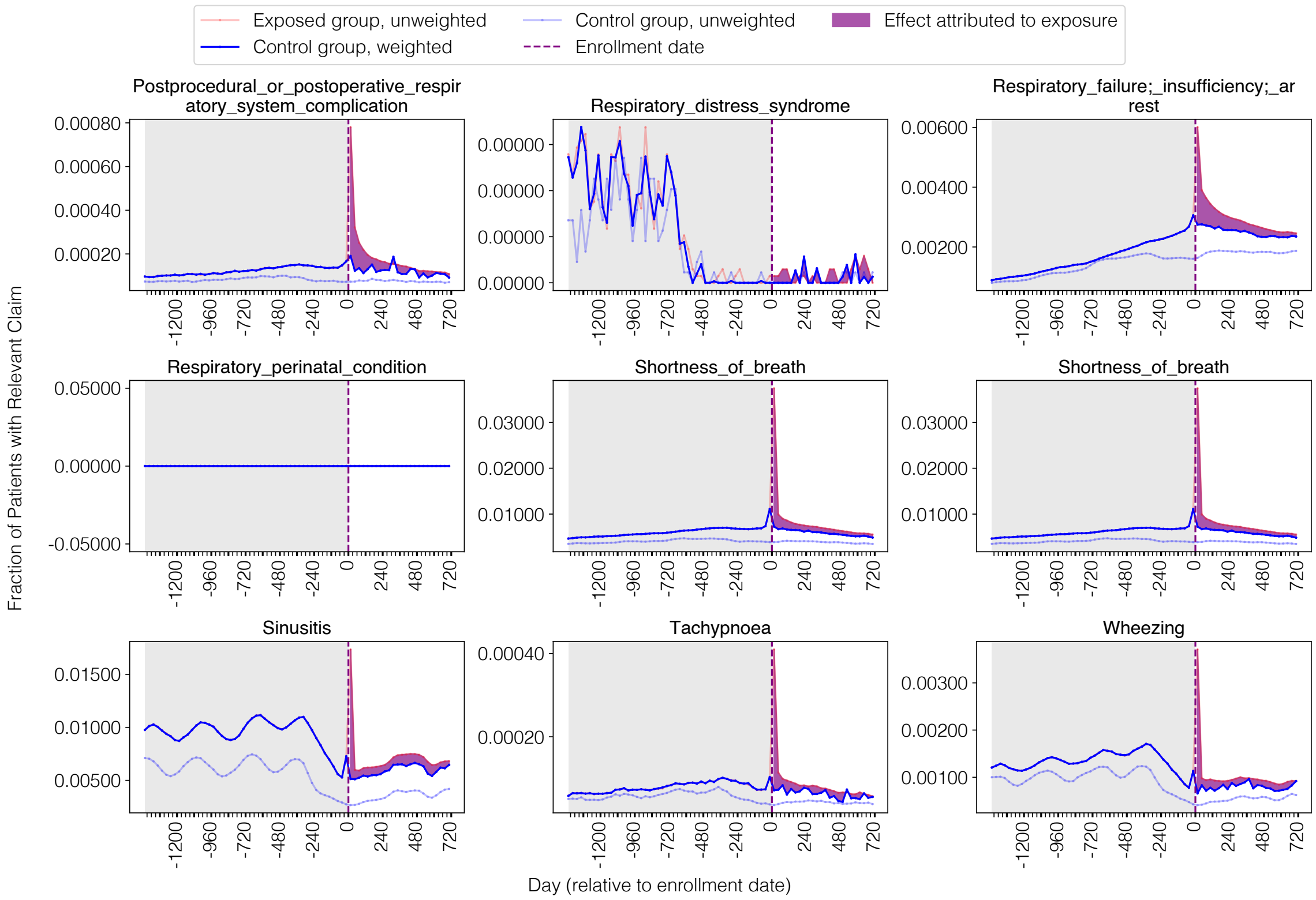


**Fig. S106.** (cont'd) Temporal trends in Breathing prevalence. The fraction of patients with relevant claims over time belonging for each outcome in the **unrestricted control design with placebo cases** (negative COVID-19 PCR test) experiment.

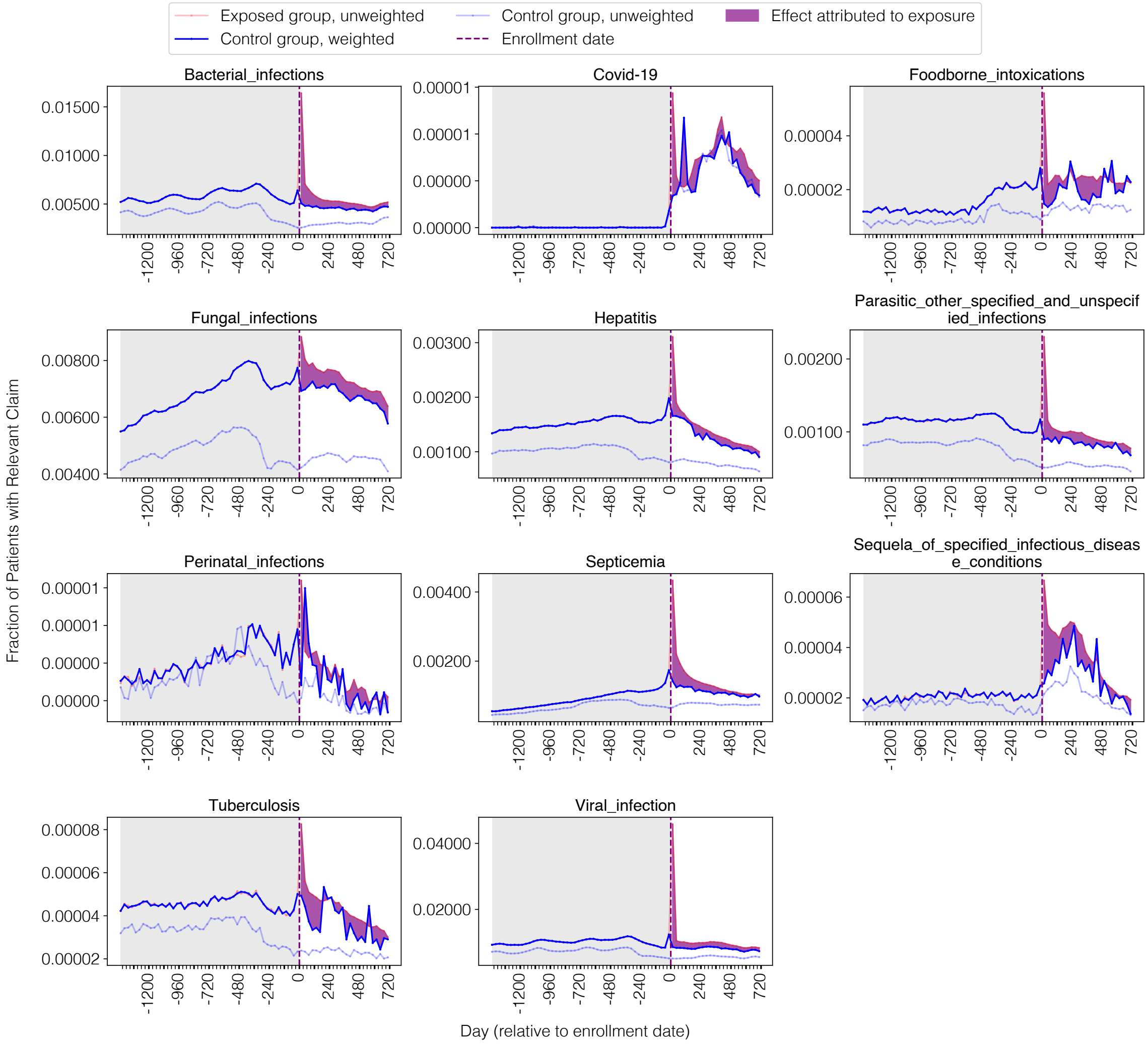


**Fig. S107.** Temporal trends in Certain Infectious and Parasitic Diseases prevalence. The fraction of patients with relevant claims over time belonging for each outcome in the **unrestricted control design with placebo cases** (negative COVID-19 PCR test) experiment.

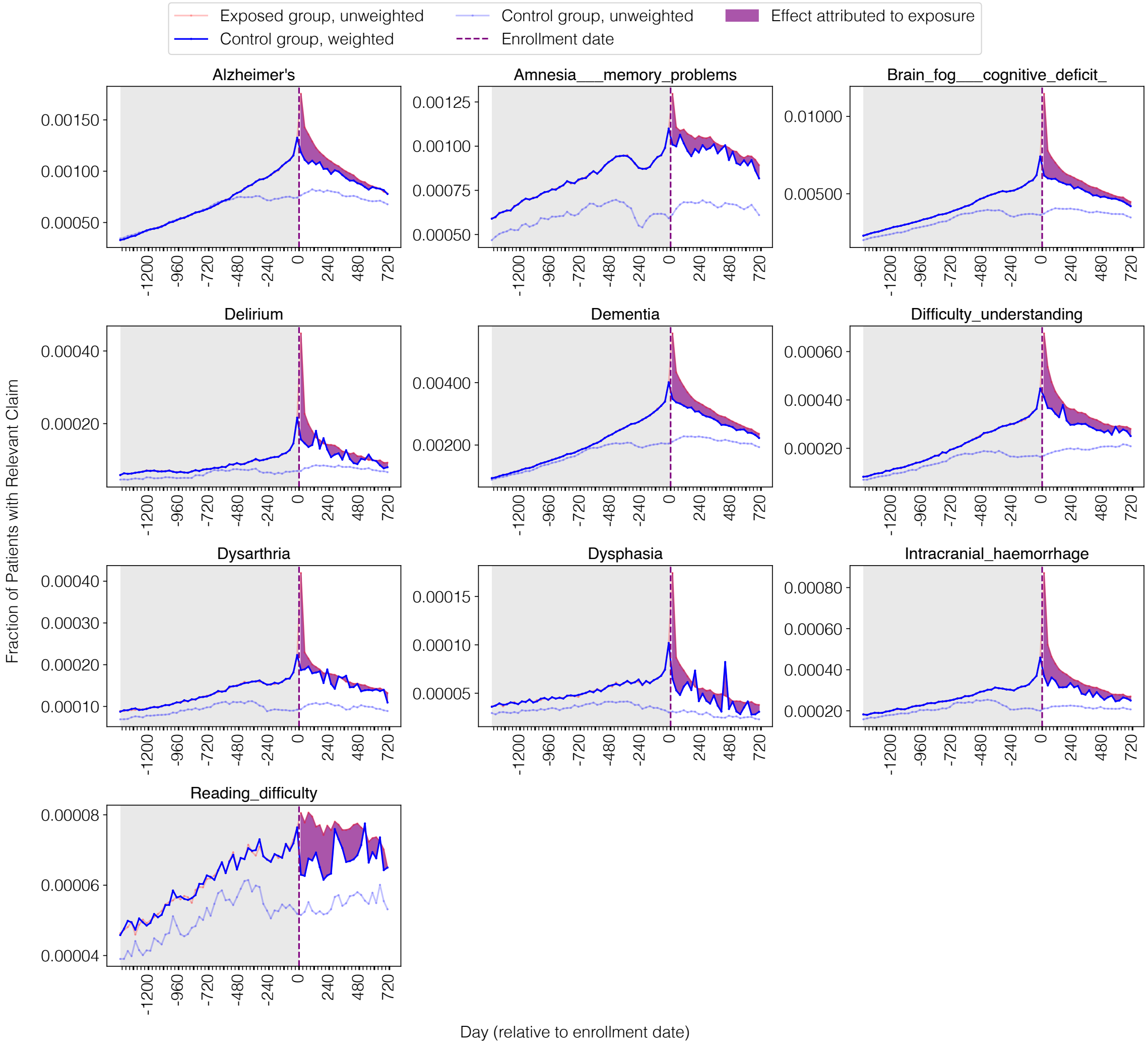


**Fig. S108.** Temporal trends in Cognitive health prevalence. The fraction of patients with relevant claims over time belonging for each outcome in the **unrestricted control design with placebo cases** (negative COVID-19 PCR test) experiment.

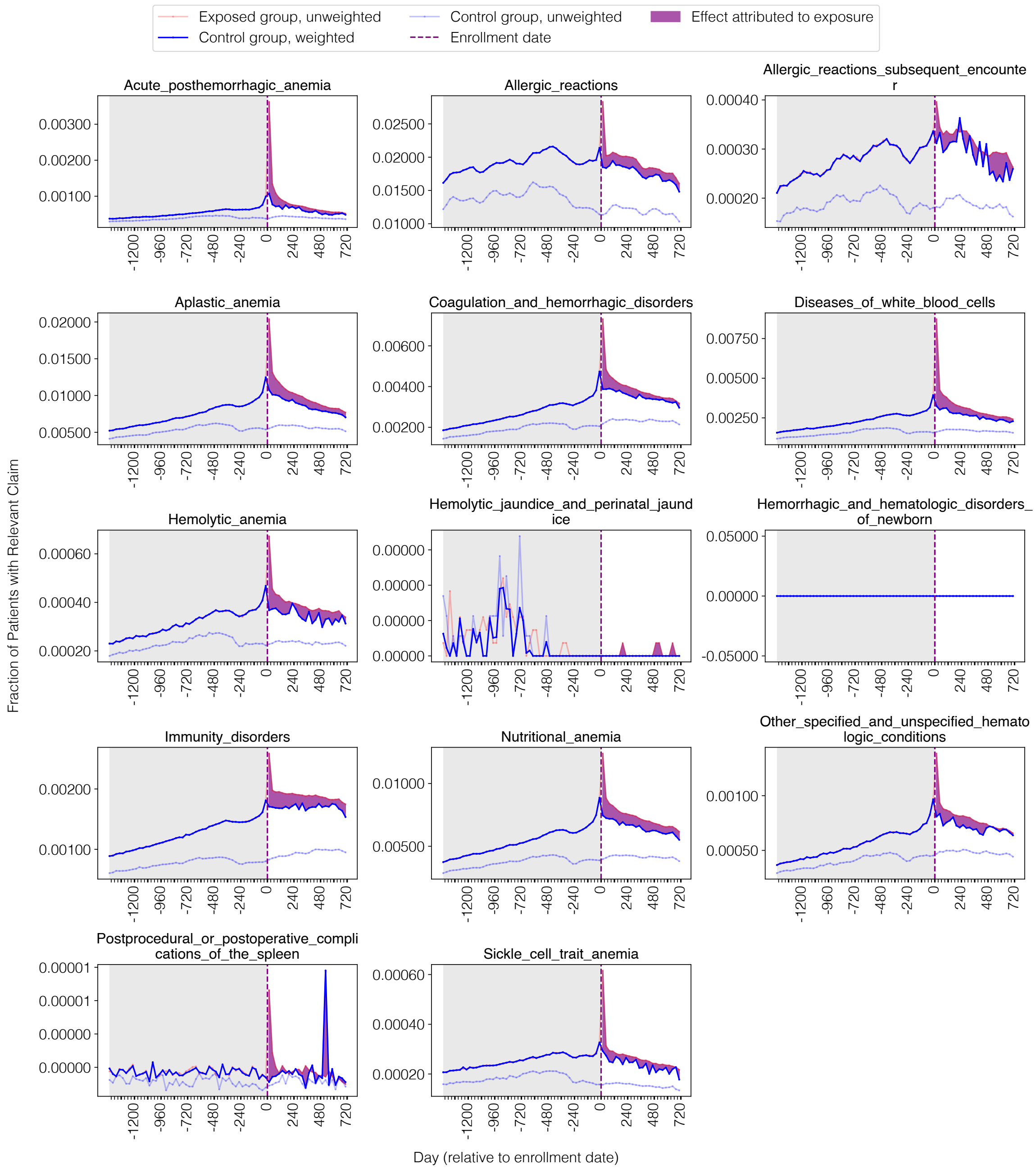


**Fig. S109.** Temporal trends in Diseases of the Blood and Blood Forming Organs and Certain Disorders Involving the Immune Mechanism prevalence. The fraction of patients with relevant claims over time belonging for each outcome in the **unrestricted control design with placebo cases** (negative COVID-19 PCR test) experiment.

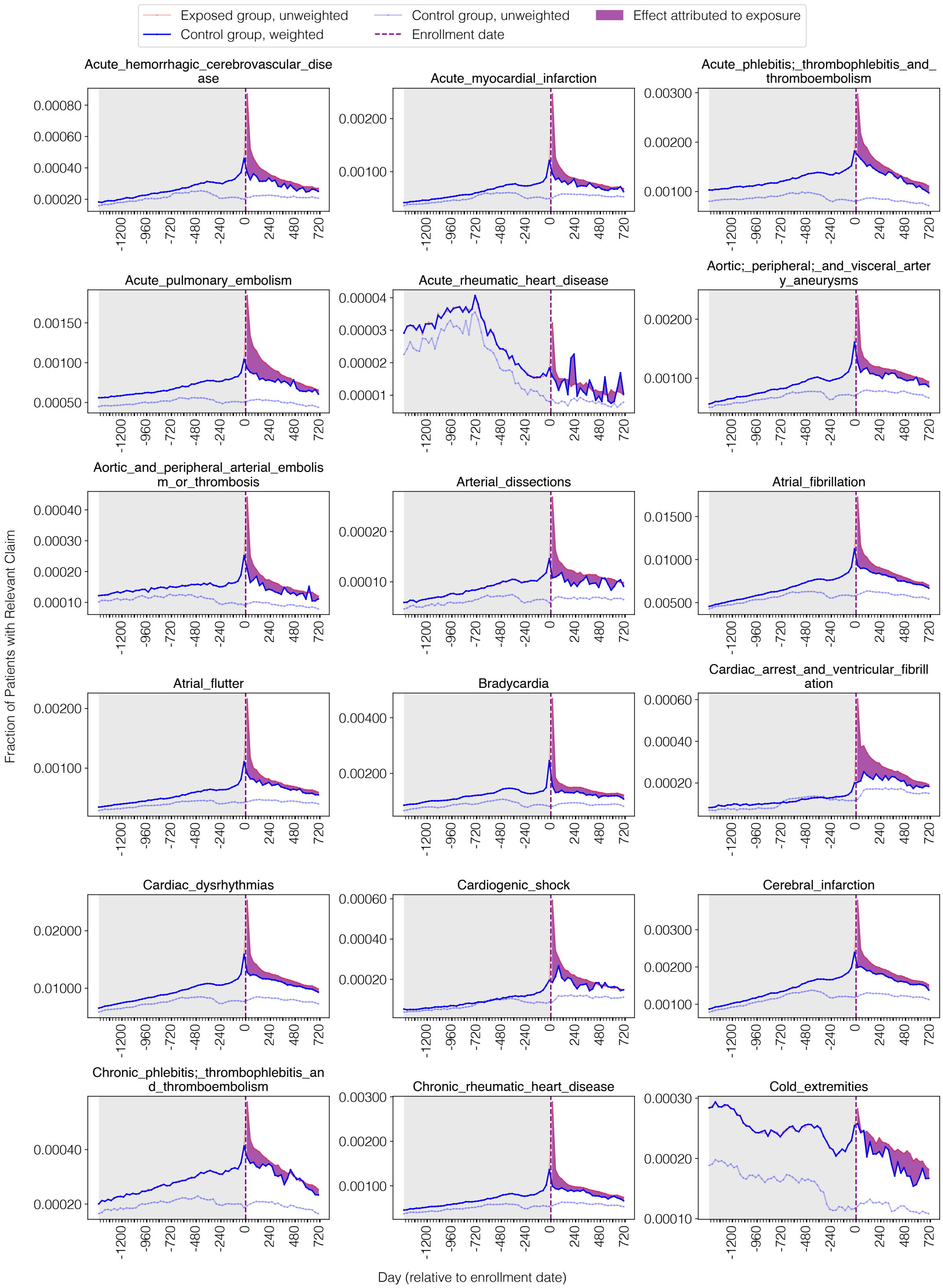


**Fig. S110.** Temporal trends in Diseases of the Circulatory System prevalence. The fraction of patients with relevant claims over time belonging for each outcome in the **unrestricted control design with placebo cases** (negative COVID-19 PCR test) experiment.

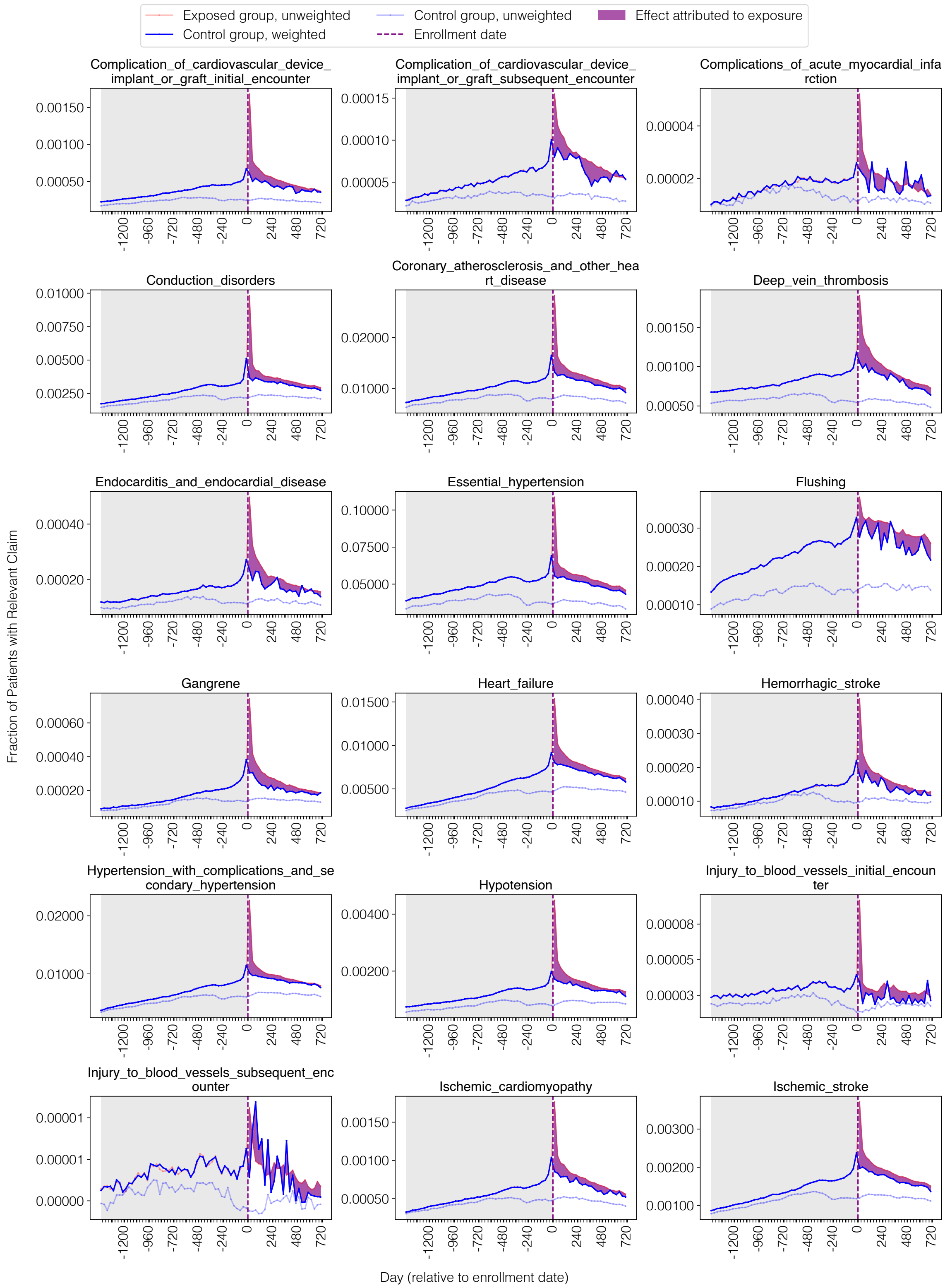


**Fig. S110.** (cont'd) Temporal trends in Diseases of the Circulatory System prevalence. The fraction of patients with relevant claims over time belonging for each outcome in the **unrestricted control design with placebo cases** (negative COVID-19 PCR test) experiment.

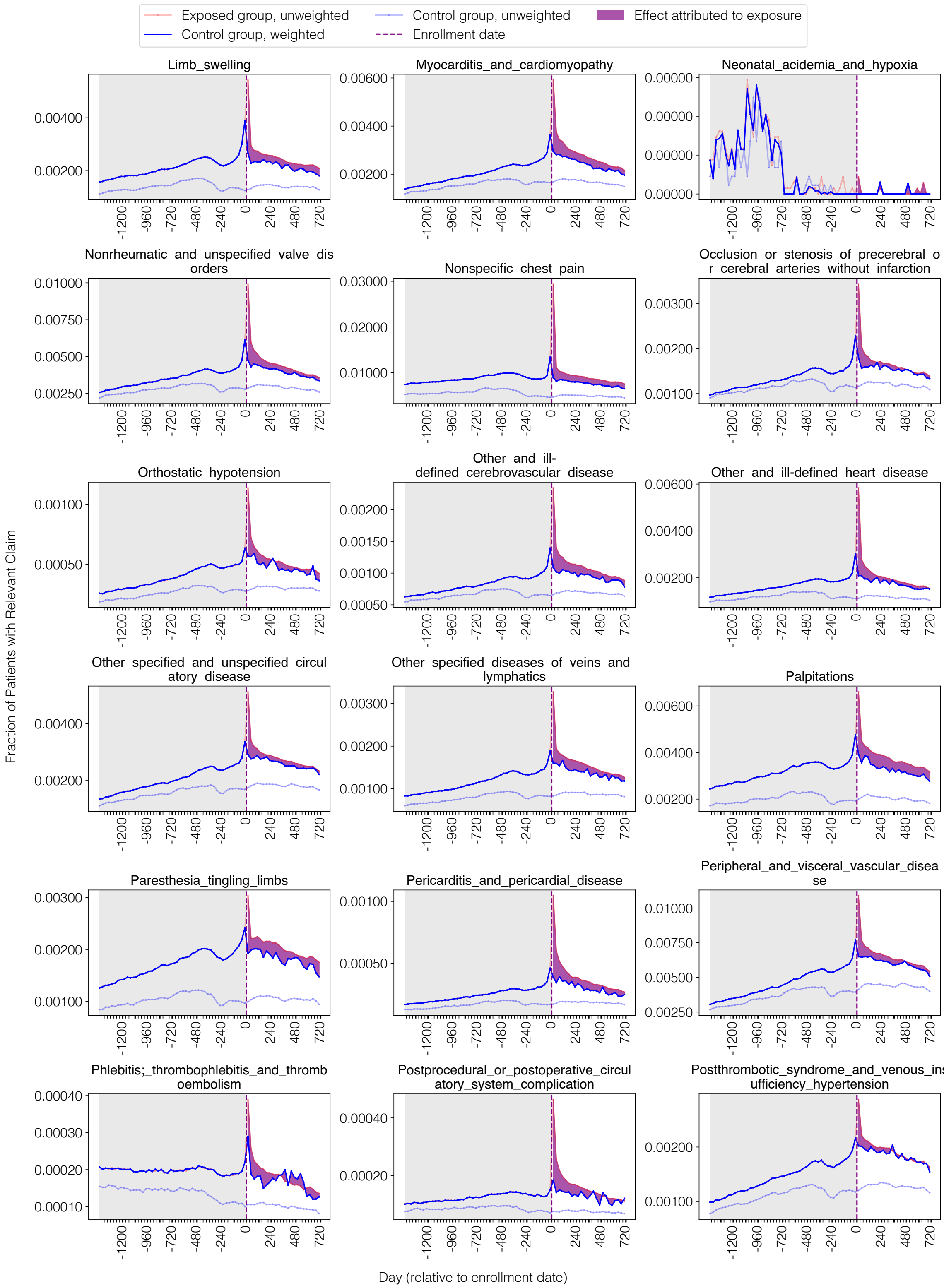


**Fig. S110.** (cont'd) Temporal trends in Diseases of the Circulatory System prevalence. The fraction of patients with relevant claims over time belonging for each outcome in the **unrestricted control design with placebo cases** (negative COVID-19 PCR test) experiment.

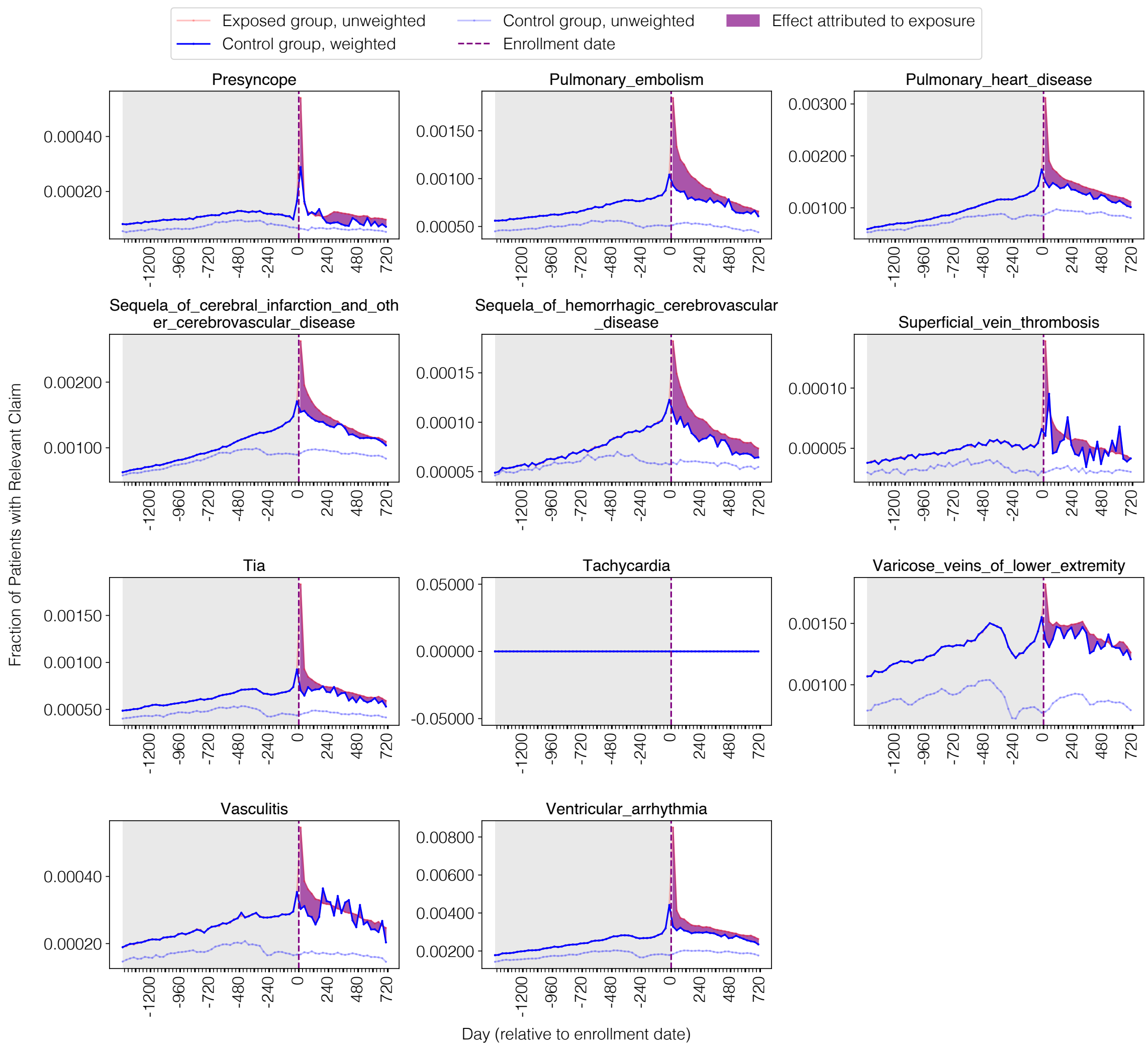


**Fig. S110.** (cont'd) Temporal trends in Diseases of the Circulatory System prevalence. The fraction of patients with relevant claims over time belonging for each outcome in the **unrestricted control design with placebo cases** (negative COVID-19 PCR test) experiment.

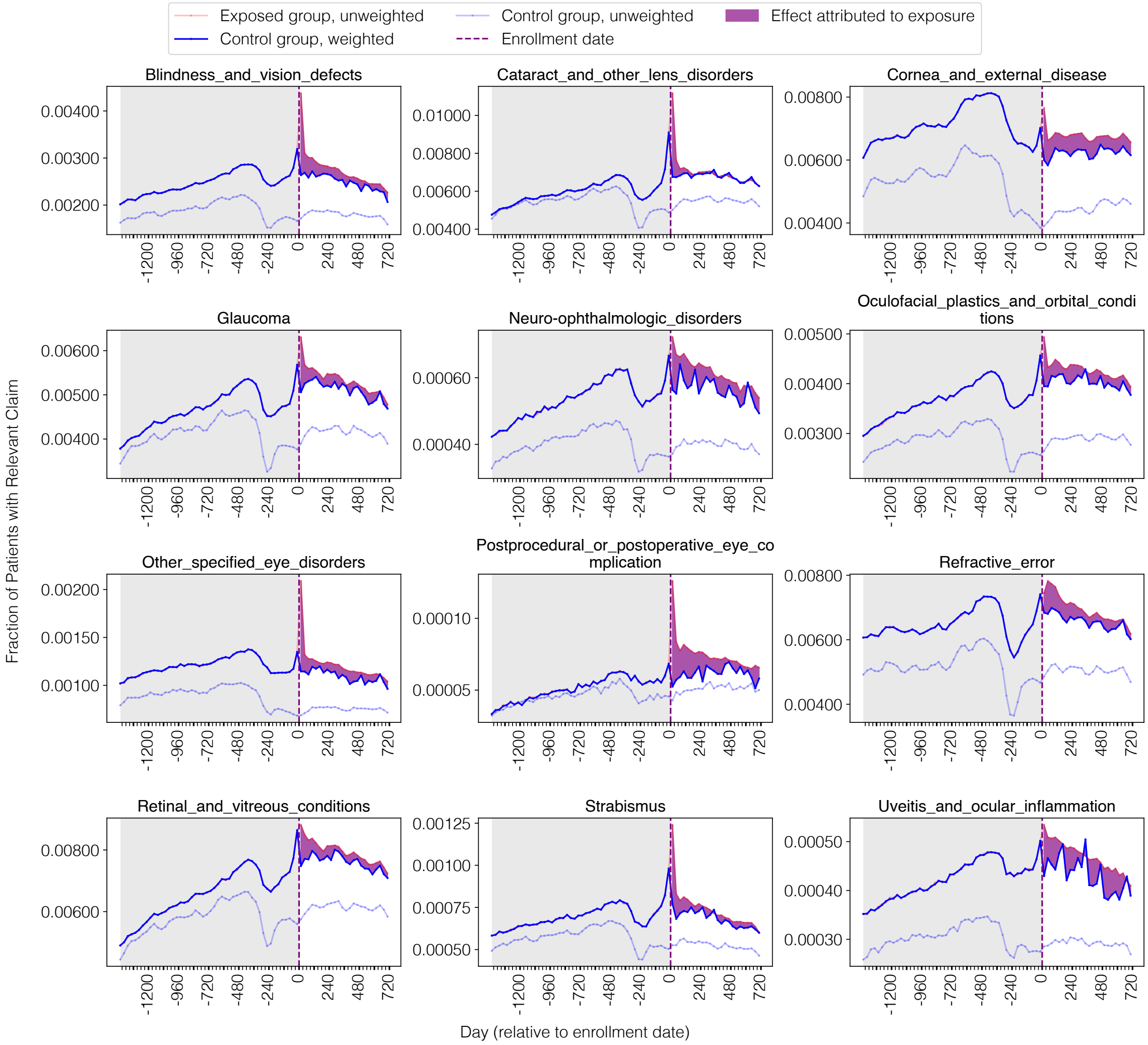


**Fig. S111.** Temporal trends in Diseases of the Eye and Adnexa prevalence. The fraction of patients with relevant claims over time belonging for each outcome in the **unrestricted control design with placebo cases** (negative COVID-19 PCR test) experiment.

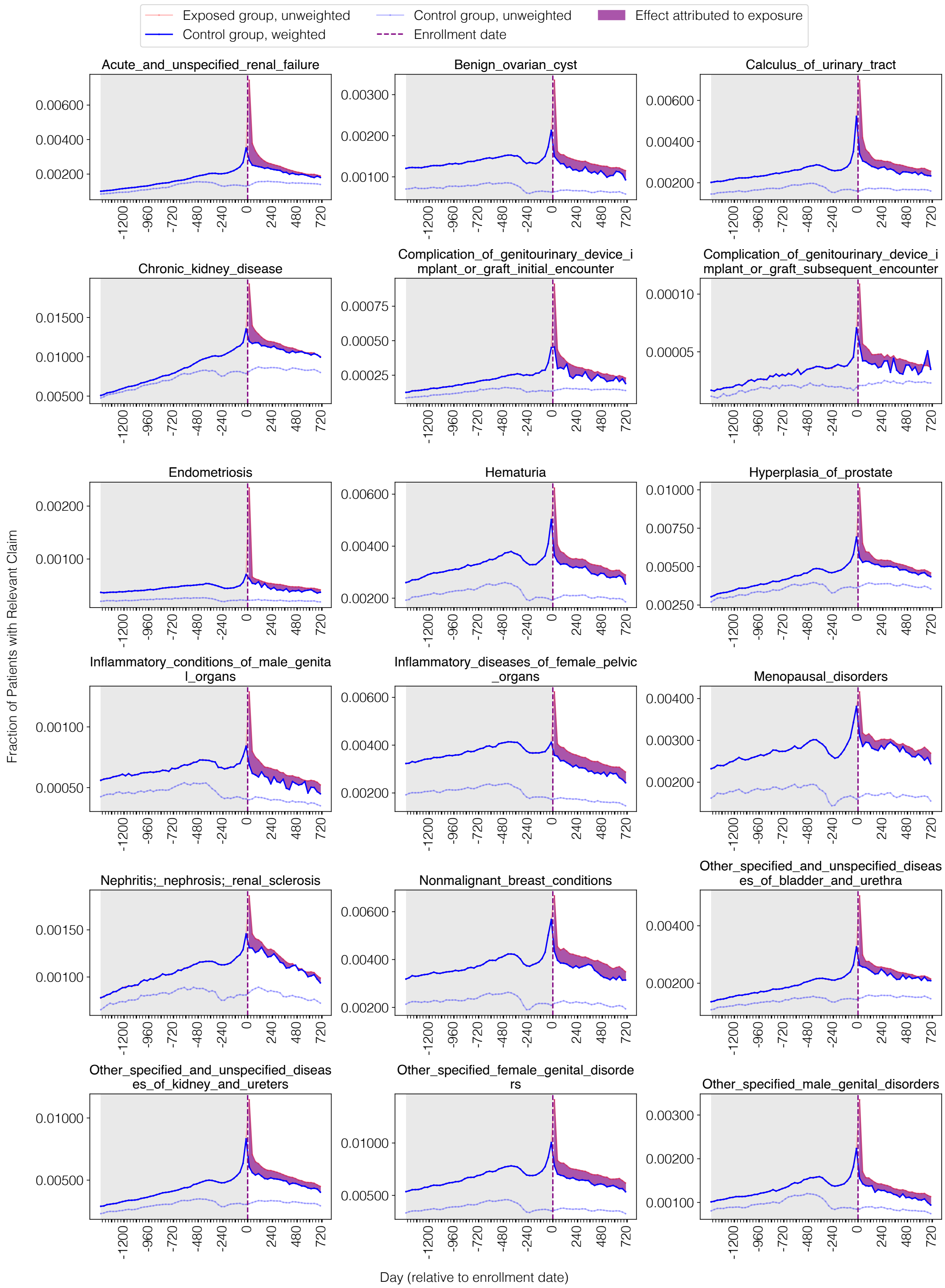


**Fig. S112.** Temporal trends in Diseases of the Genitourinary System prevalence. The fraction of patients with relevant claims over time belonging for each outcome in the **unrestricted control design with placebo cases** (negative COVID-19 PCR test) experiment.

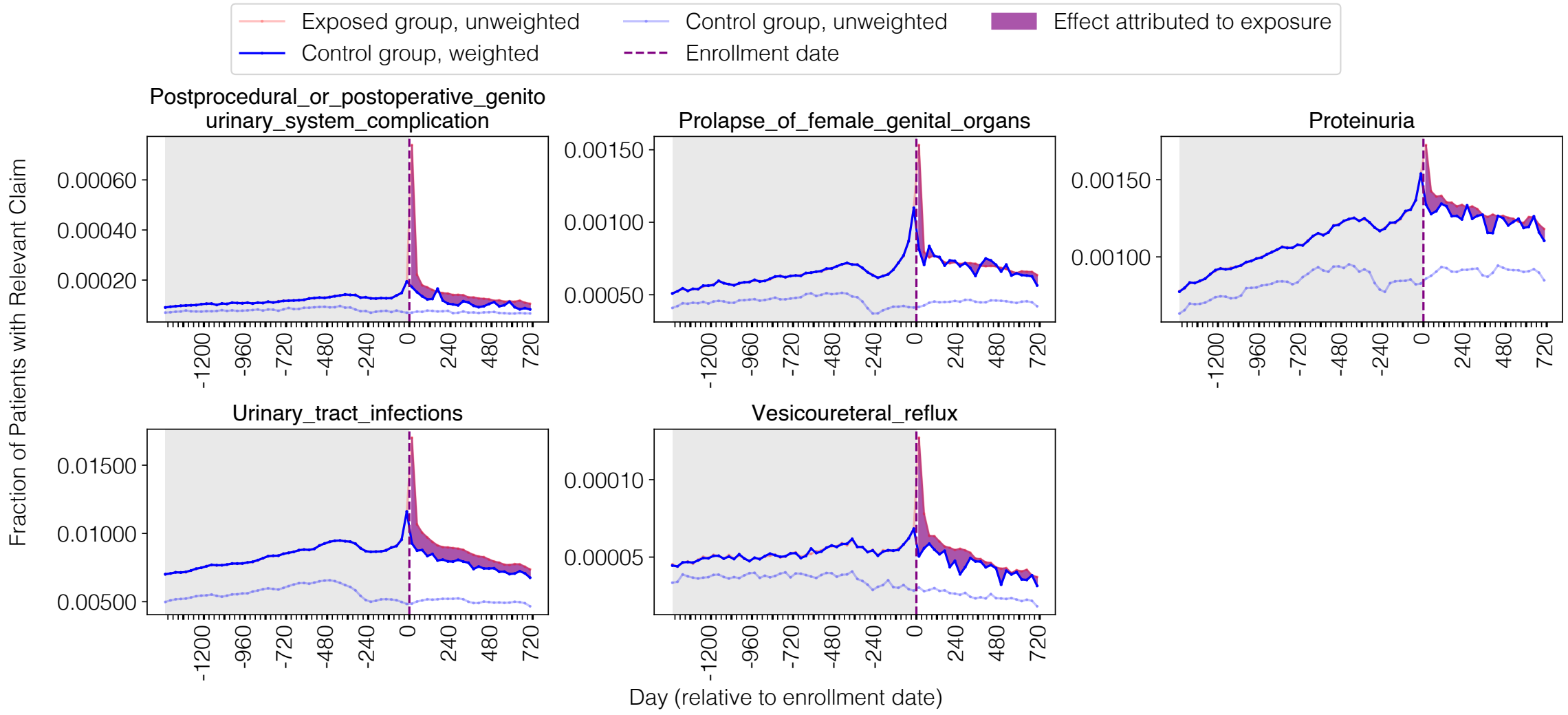


**Fig. S112.** (cont'd) Temporal trends in Diseases of the Genitourinary System prevalence. The fraction of patients with relevant claims over time belonging for each outcome in the **unrestricted control design with placebo cases** (negative COVID-19 PCR test) experiment.

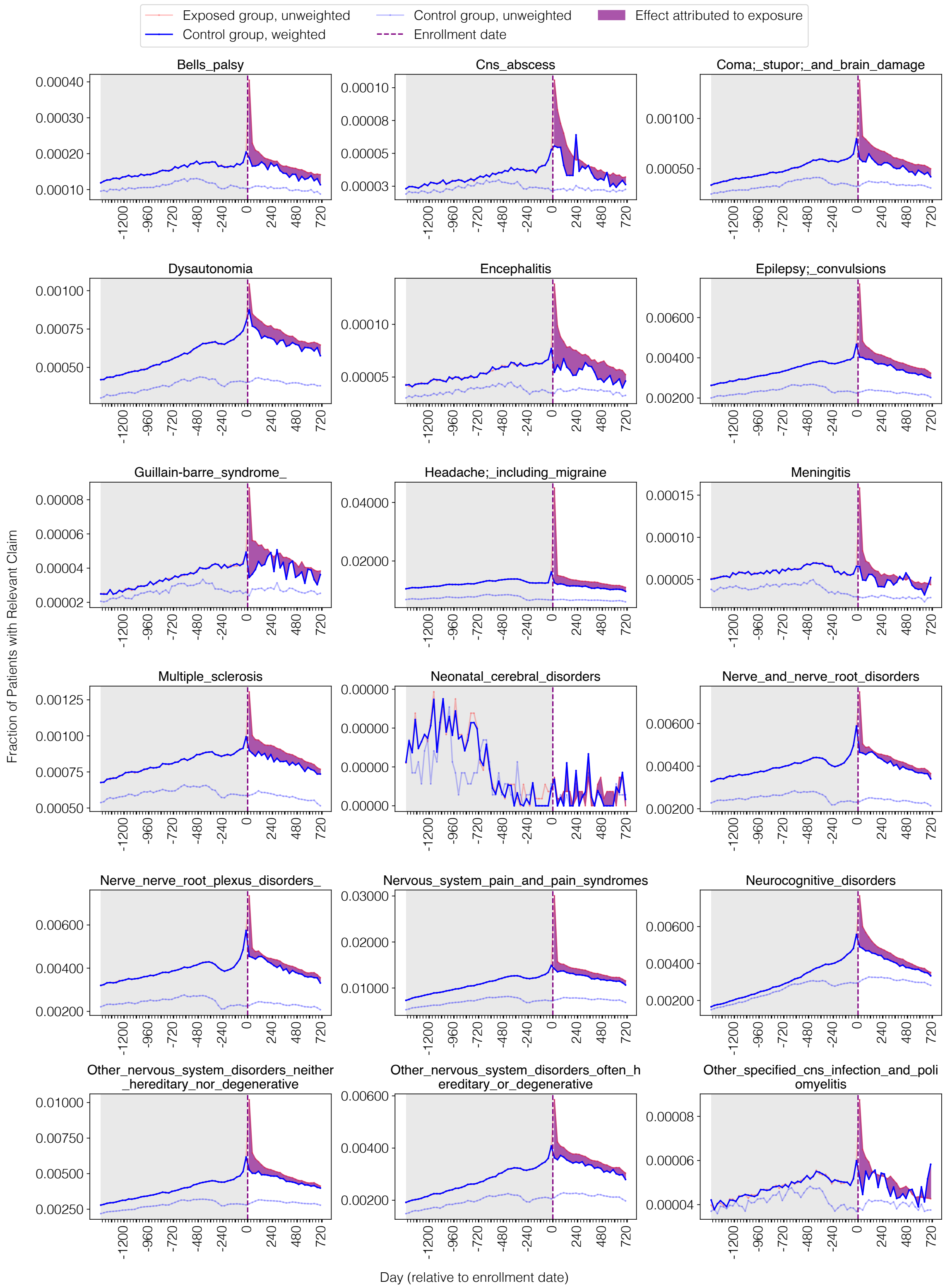


**Fig. S113.** Temporal trends in Diseases of the Nervous System prevalence. The fraction of patients with relevant claims over time belonging for each outcome in the **unrestricted control design with placebo cases** (negative COVID-19 PCR test) experiment.

 

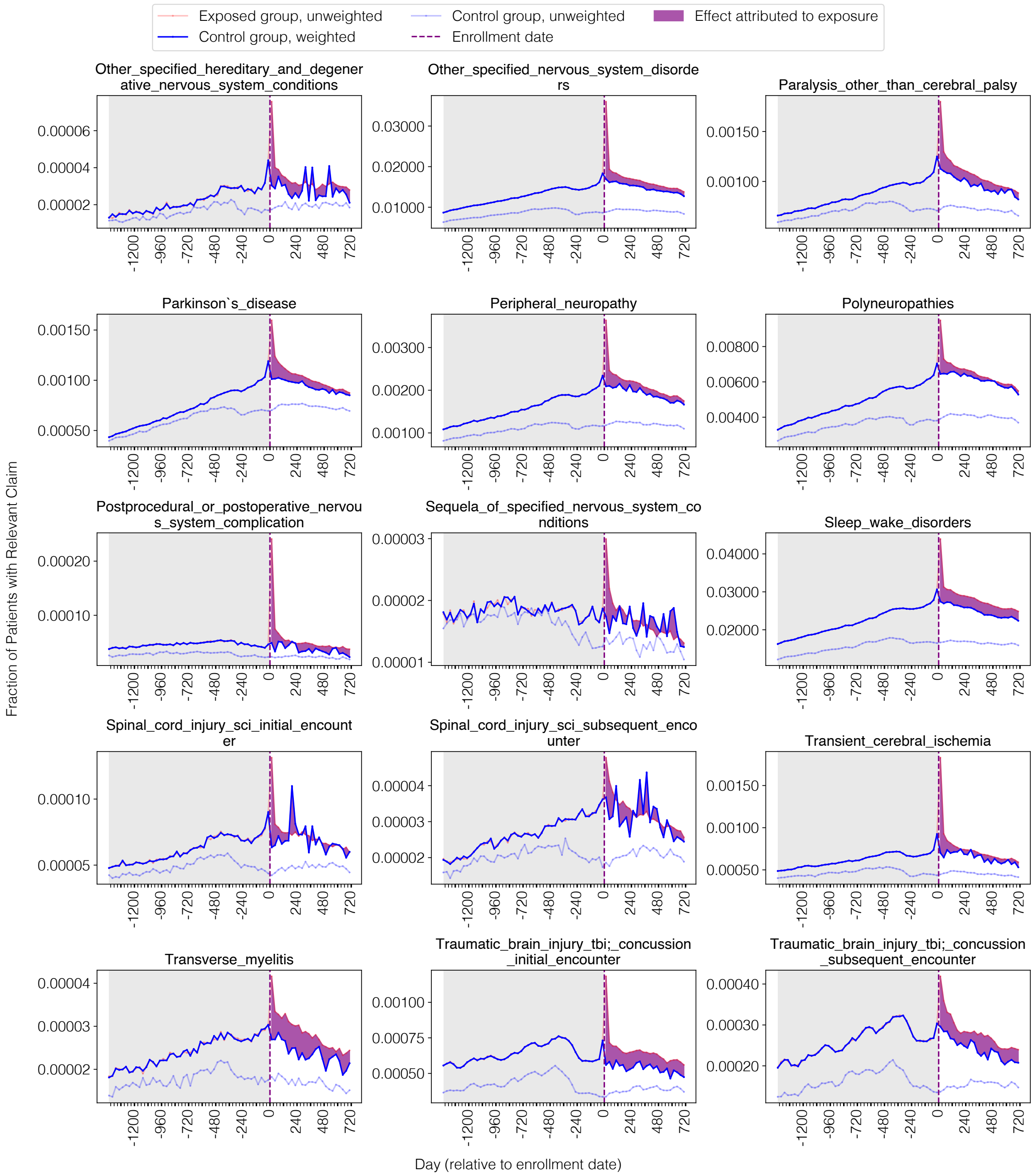


**Fig. S113.** (cont'd) Temporal trends in Diseases of the Nervous System prevalence. The fraction of patients with relevant claims over time belonging for each outcome in the **unrestricted control design with placebo cases** (negative COVID-19 PCR test) experiment.

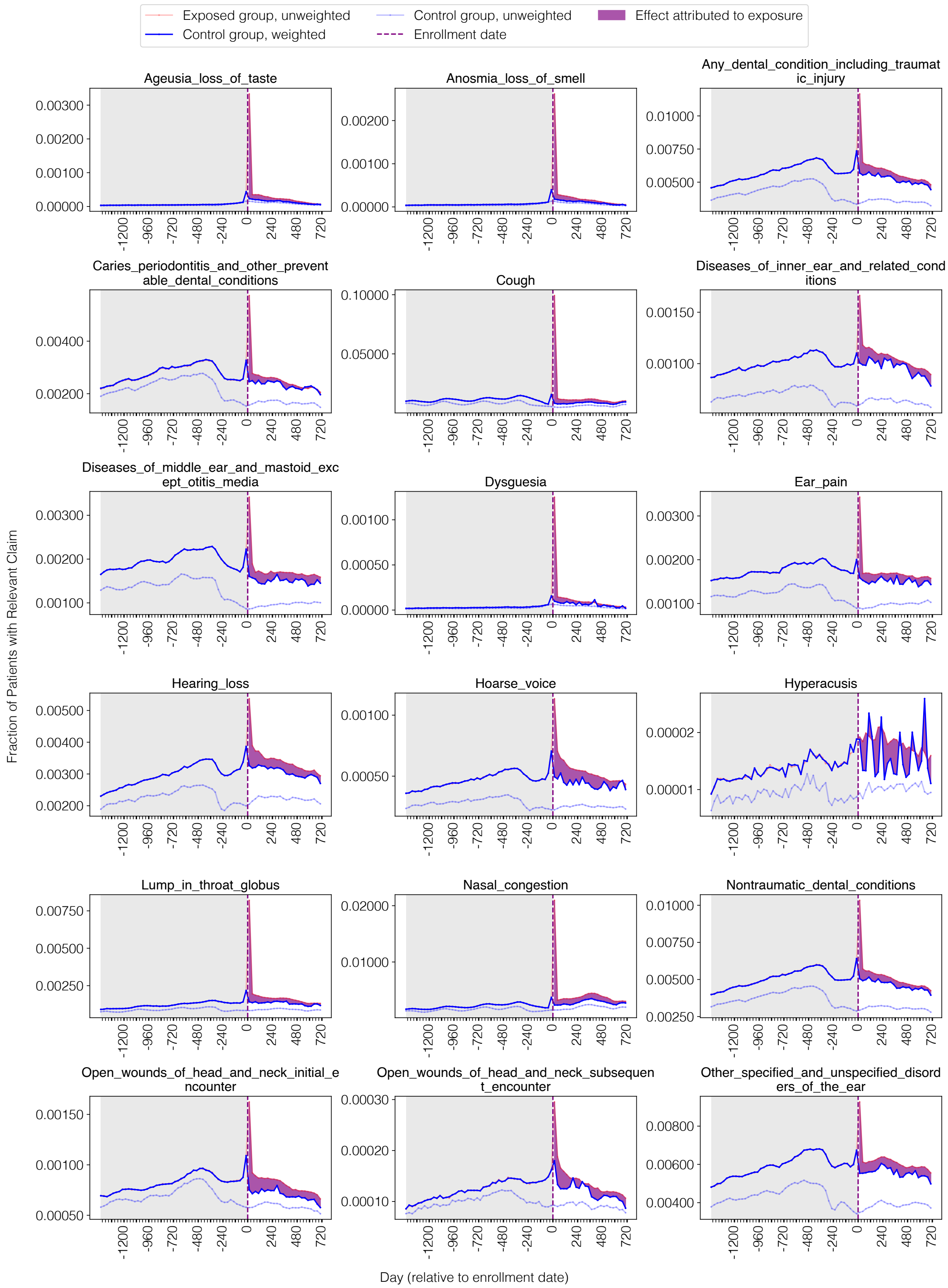


**Fig. S114.** Temporal trends in Ear, nose and throat prevalence. The fraction of patients with relevant claims over time belonging for each outcome in the **unrestricted control design with placebo cases** (negative COVID-19 PCR test) experiment.

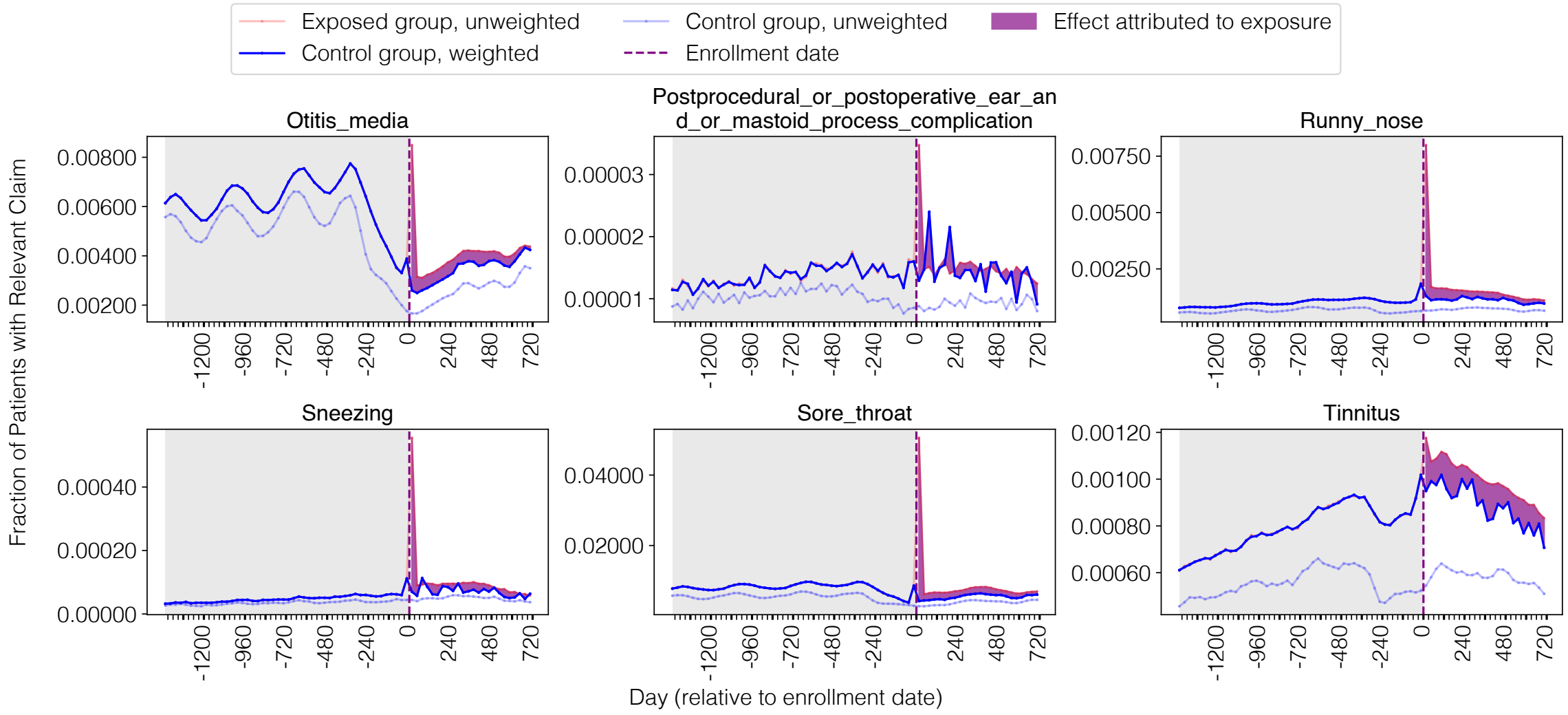


**Fig. S114.** (cont'd) Temporal trends in Ear, nose and throat prevalence. The fraction of patients with relevant claims over time belonging for each outcome in the **unrestricted control design with placebo cases** (negative COVID-19 PCR test) experiment.

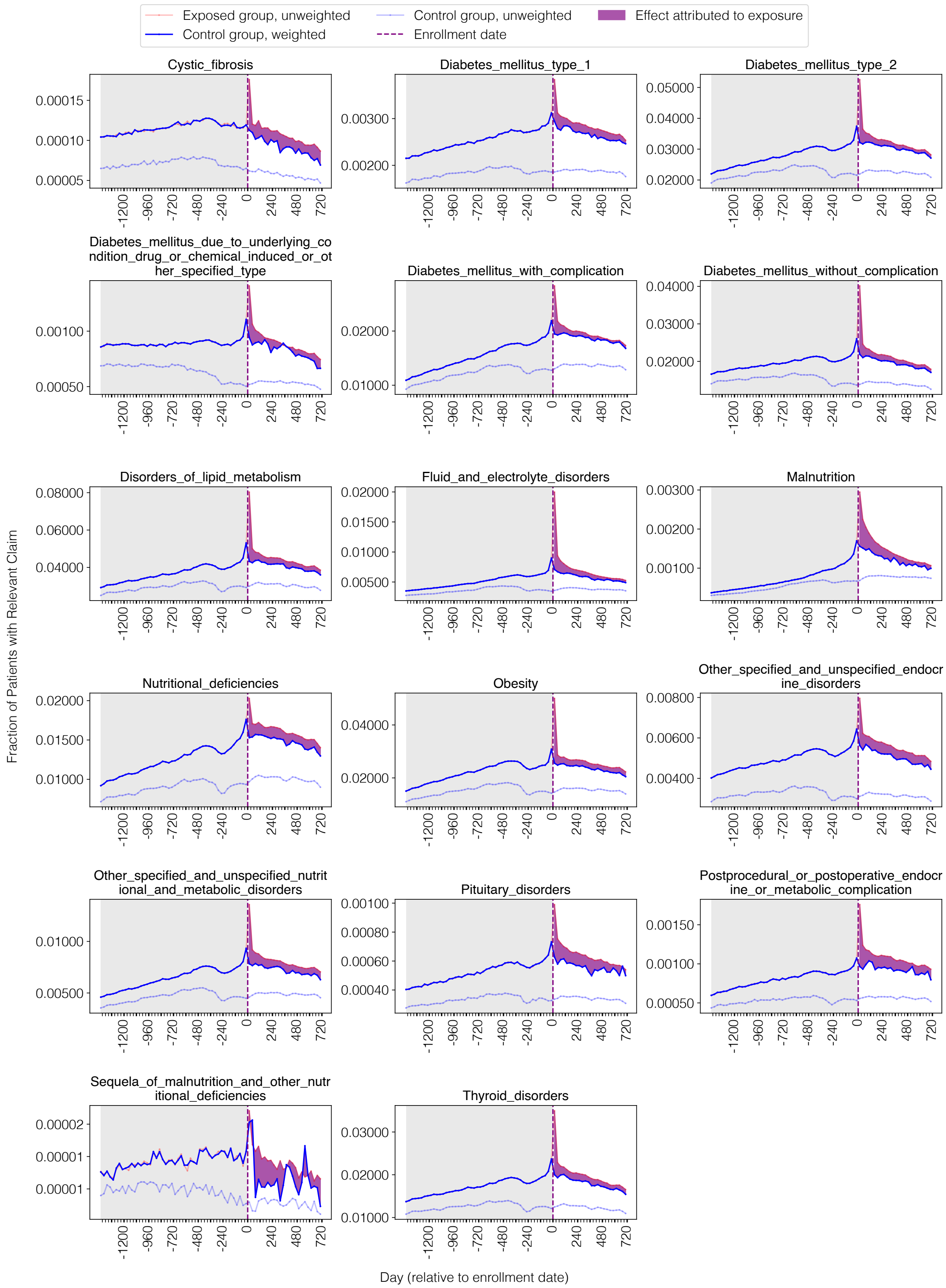


**Fig. S115.** Temporal trends in Endocrine, Nutritional and Metabolic Diseases prevalence. The fraction of patients with relevant claims over time belonging for each outcome in the **unrestricted control design with placebo cases** (negative COVID-19 PCR test) experiment.

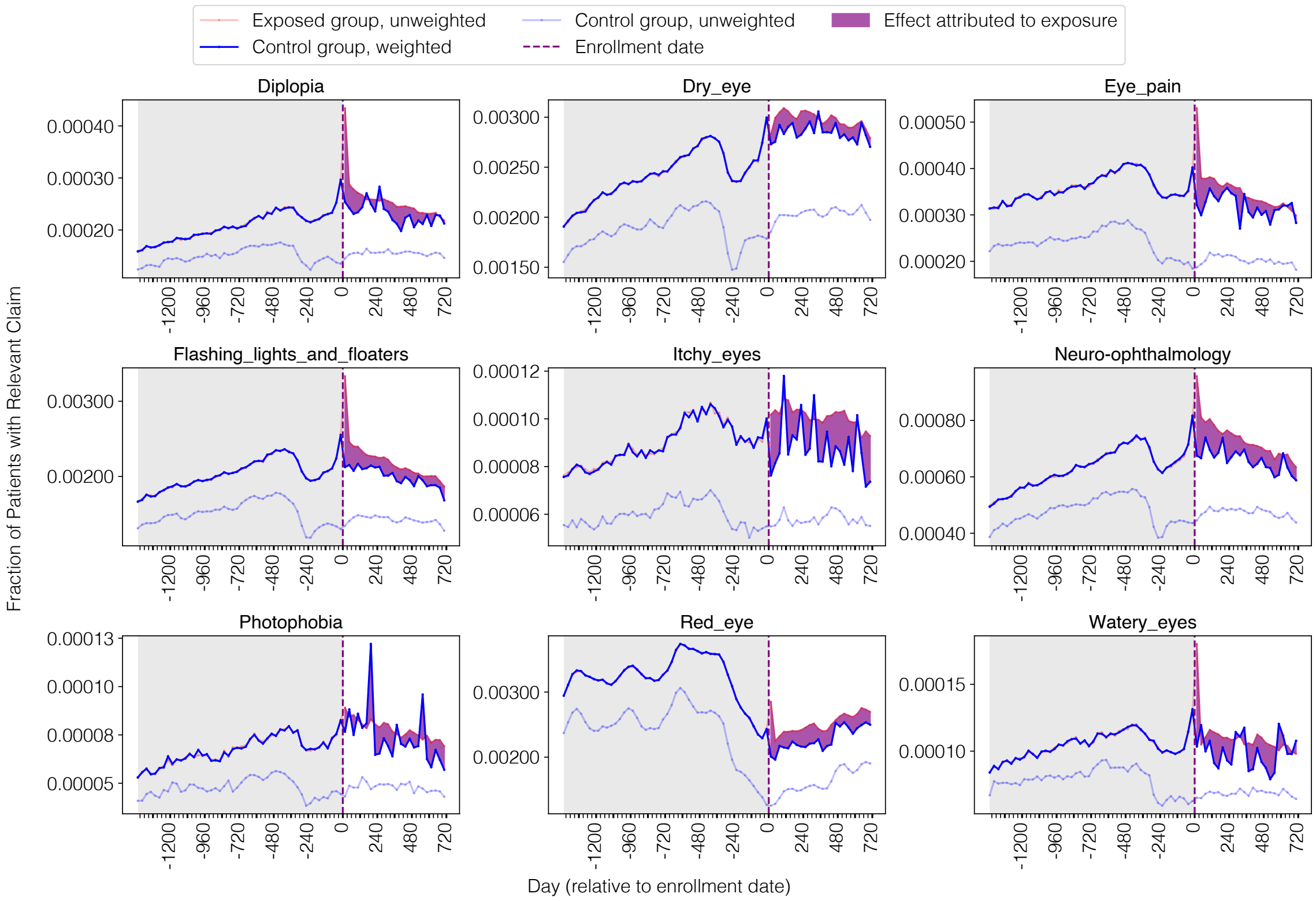


**Fig. S116.** Temporal trends in Eyes prevalence. The fraction of patients with relevant claims over time belonging for each outcome in the **unrestricted control design with placebo cases** (negative COVID-19 PCR test) experiment.

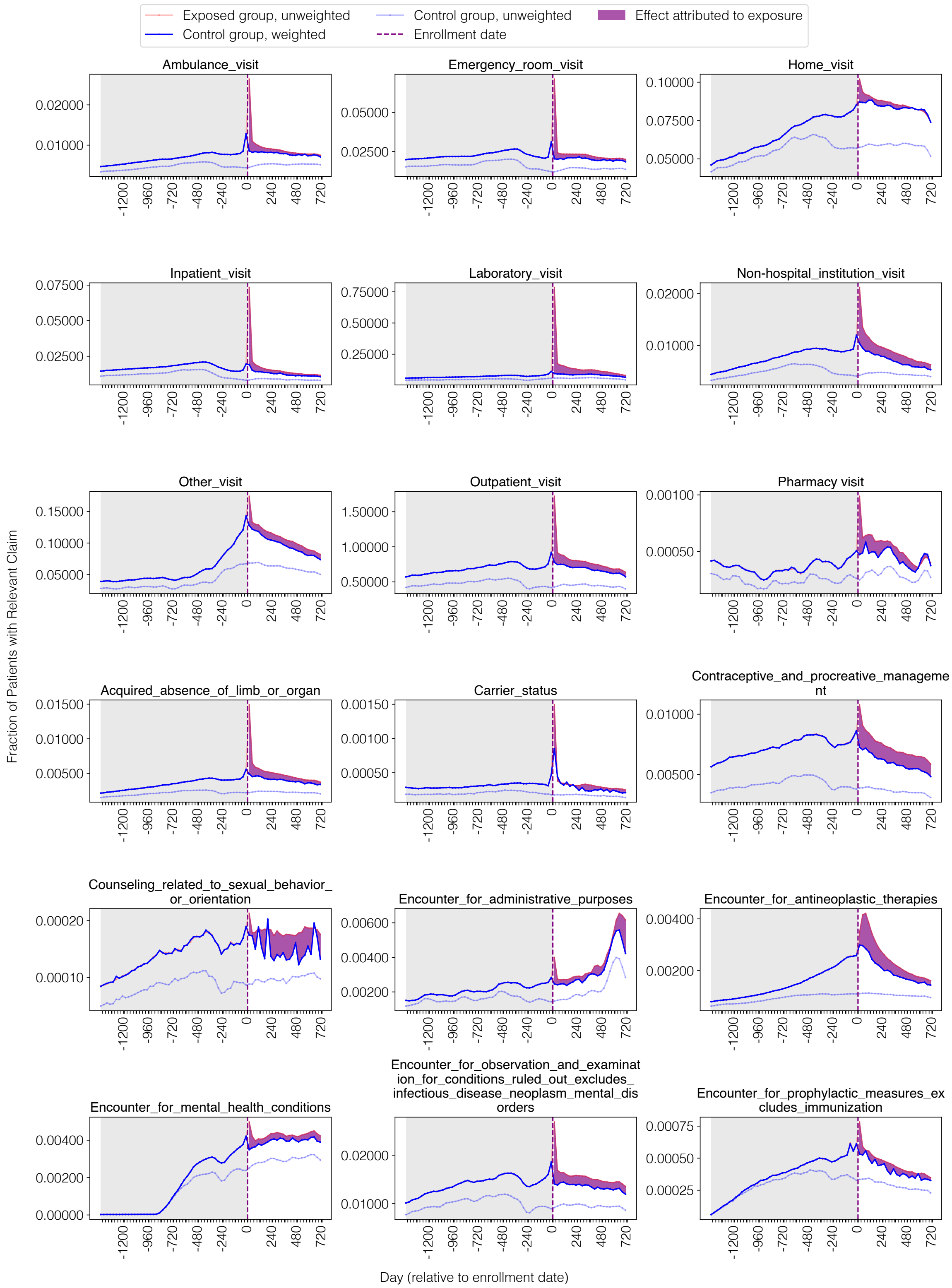


**Fig. S117.** Temporal trends in Factors Influencing Health Status and Contact with Health Services prevalence. The fraction of patients with relevant claims over time belonging for each outcome in the **unrestricted control design with placebo cases** (negative COVID-19 PCR test) experiment.

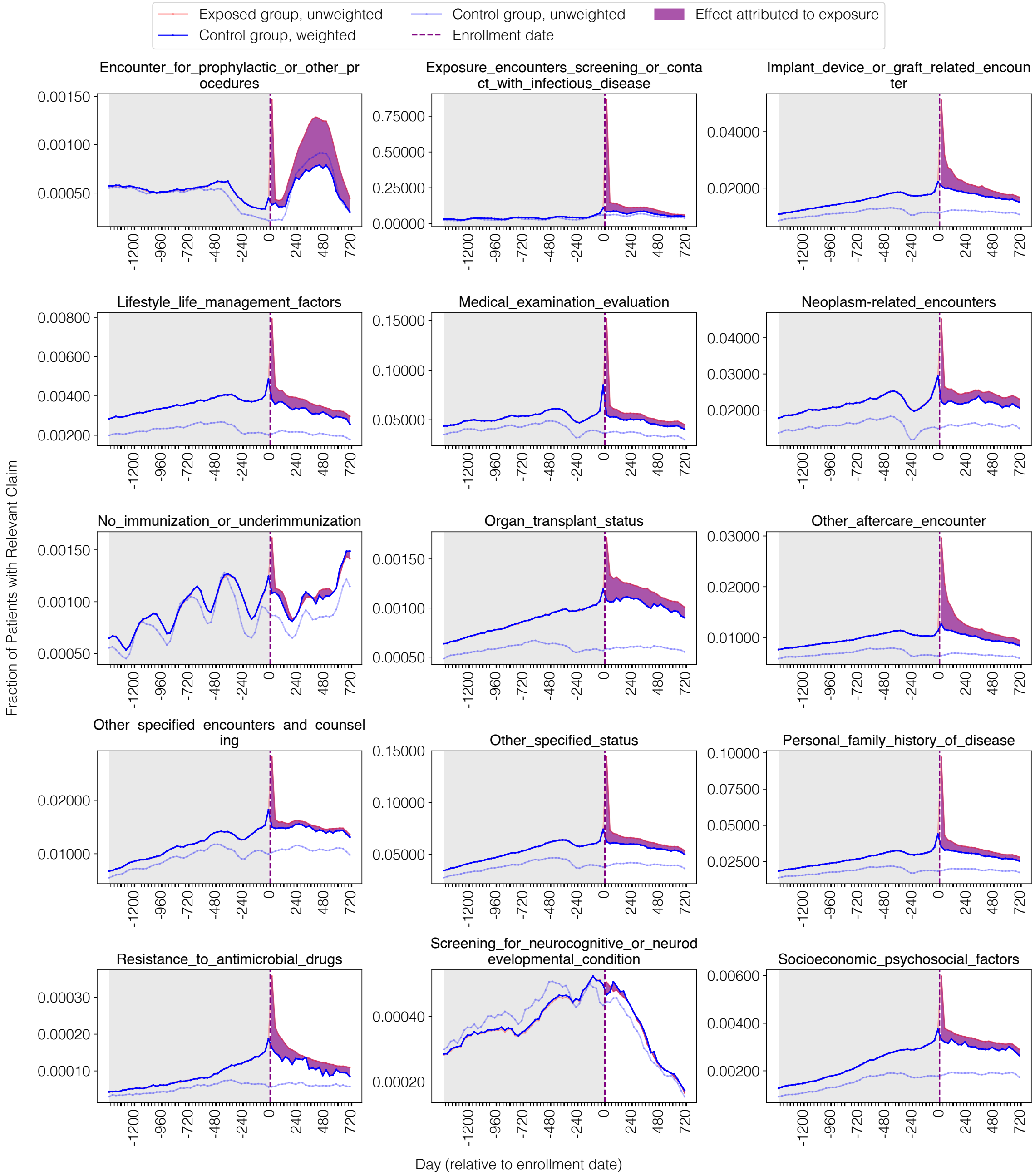


**Fig. S117.** (cont'd) Temporal trends in Factors Influencing Health Status and Contact with Health Services prevalence. The fraction of patients with relevant claims over time belonging for each outcome in the **unrestricted control design with placebo cases** (negative COVID-19 PCR test) experiment.

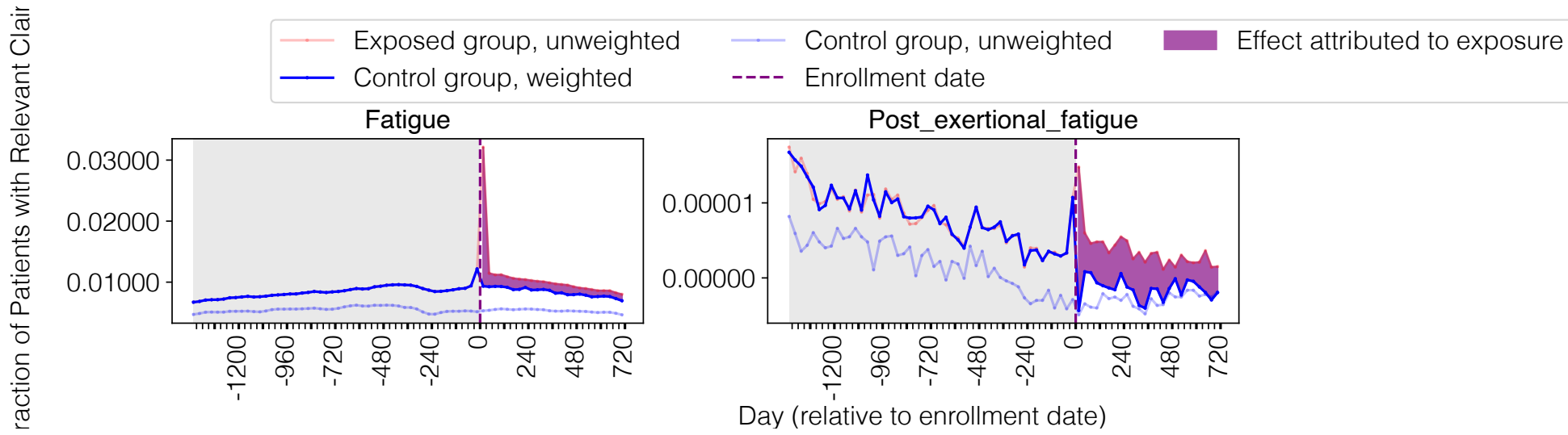


**Fig. S118.** Temporal trends in Fatigue prevalence. The fraction of patients with relevant claims over time belonging for each outcome in the **unrestricted control design with placebo cases** (negative COVID-19 PCR test) experiment.

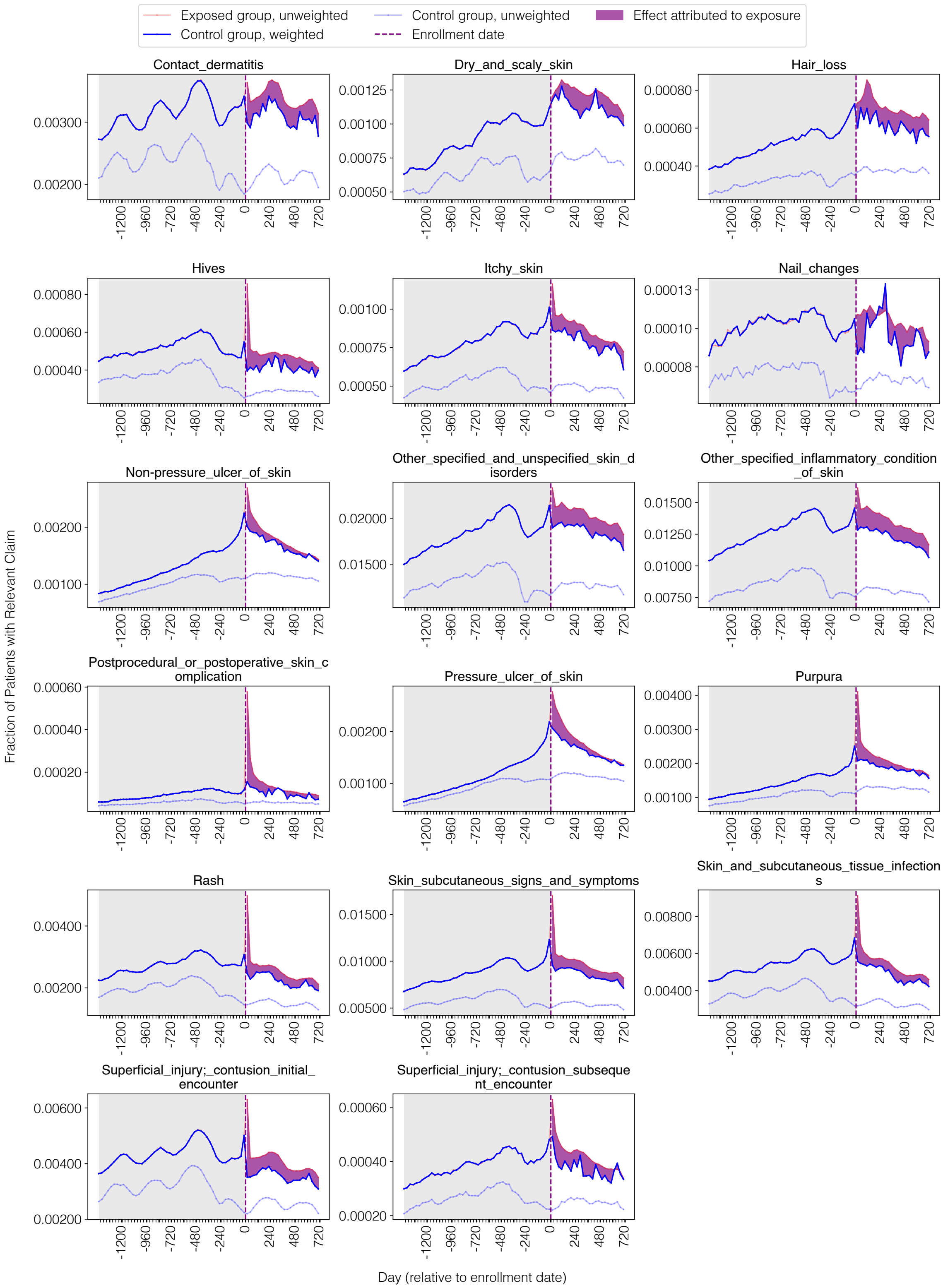


**Fig. S119.** Temporal trends in Hair, skin and nails prevalence. The fraction of patients with relevant claims over time belonging for each outcome in the **unrestricted control design with placebo cases** (negative COVID-19 PCR test) experiment.

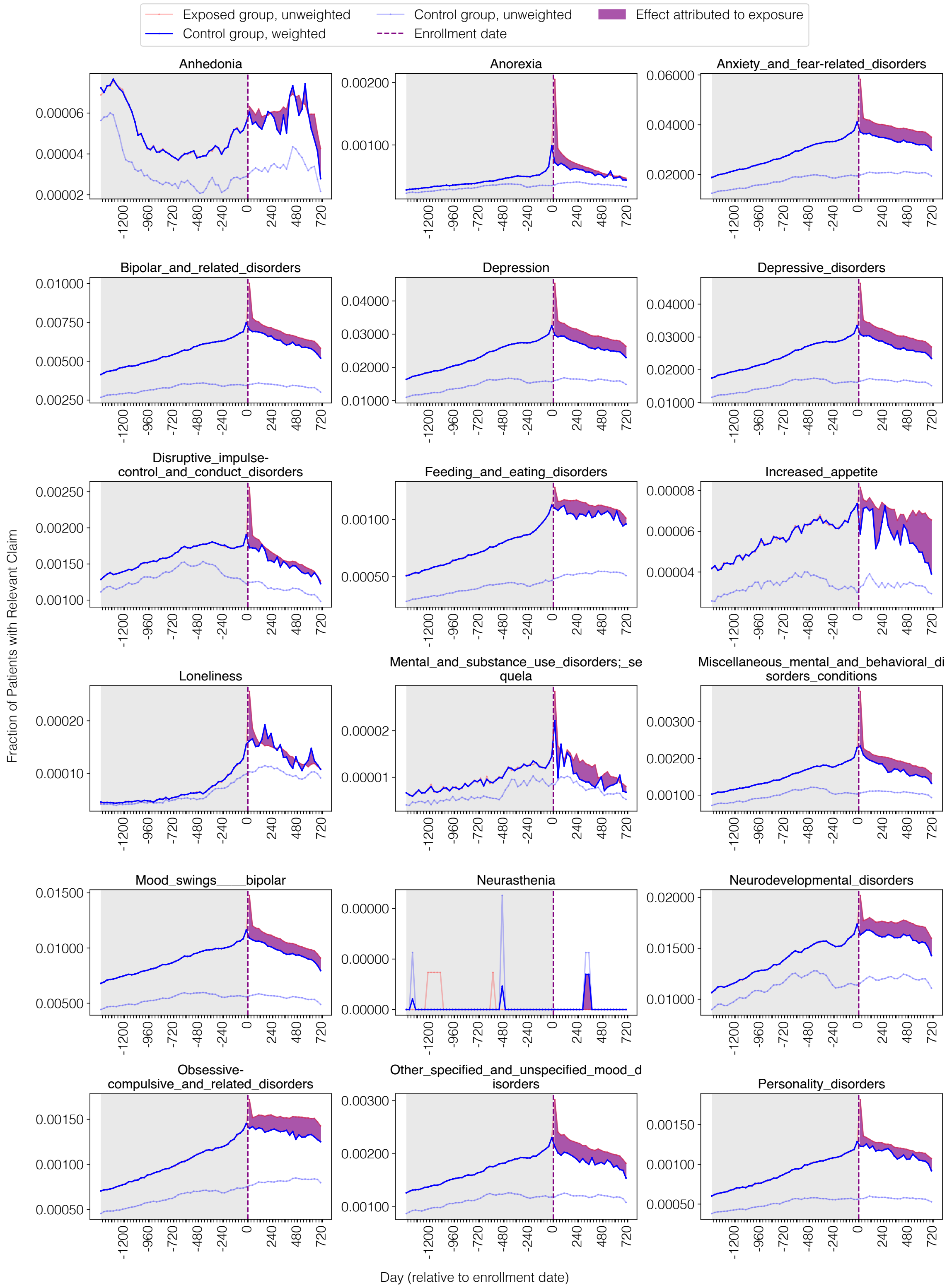


**Fig. S120.** Temporal trends in Mental health prevalence. The fraction of patients with relevant claims over time belonging for each outcome in the **unrestricted control design with placebo cases** (negative COVID-19 PCR test) experiment.

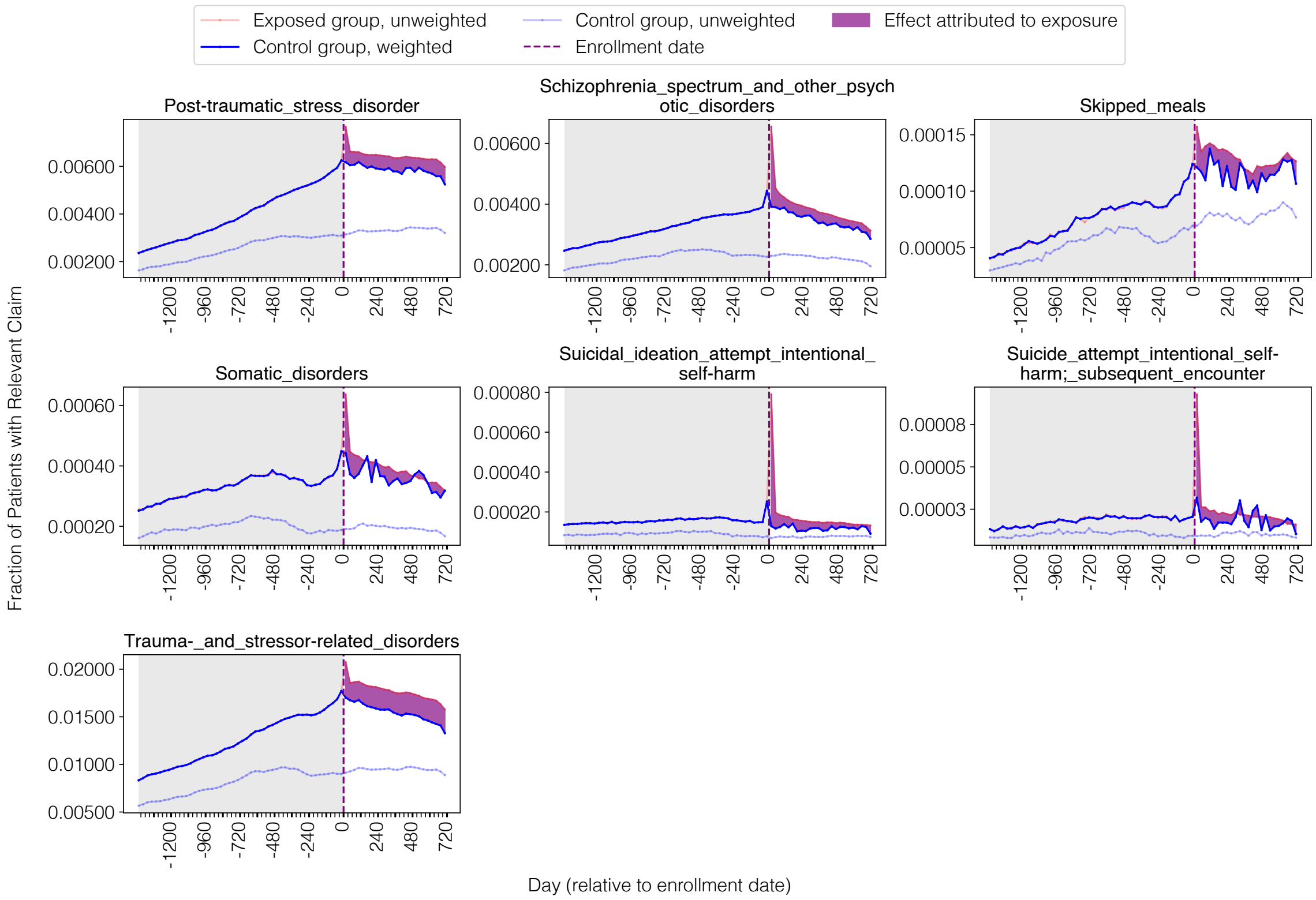


**Fig. S120.** (cont'd) Temporal trends in Mental health prevalence. The fraction of patients with relevant claims over time belonging for each outcome in the **unrestricted control design with placebo cases** (negative COVID-19 PCR test) experiment.

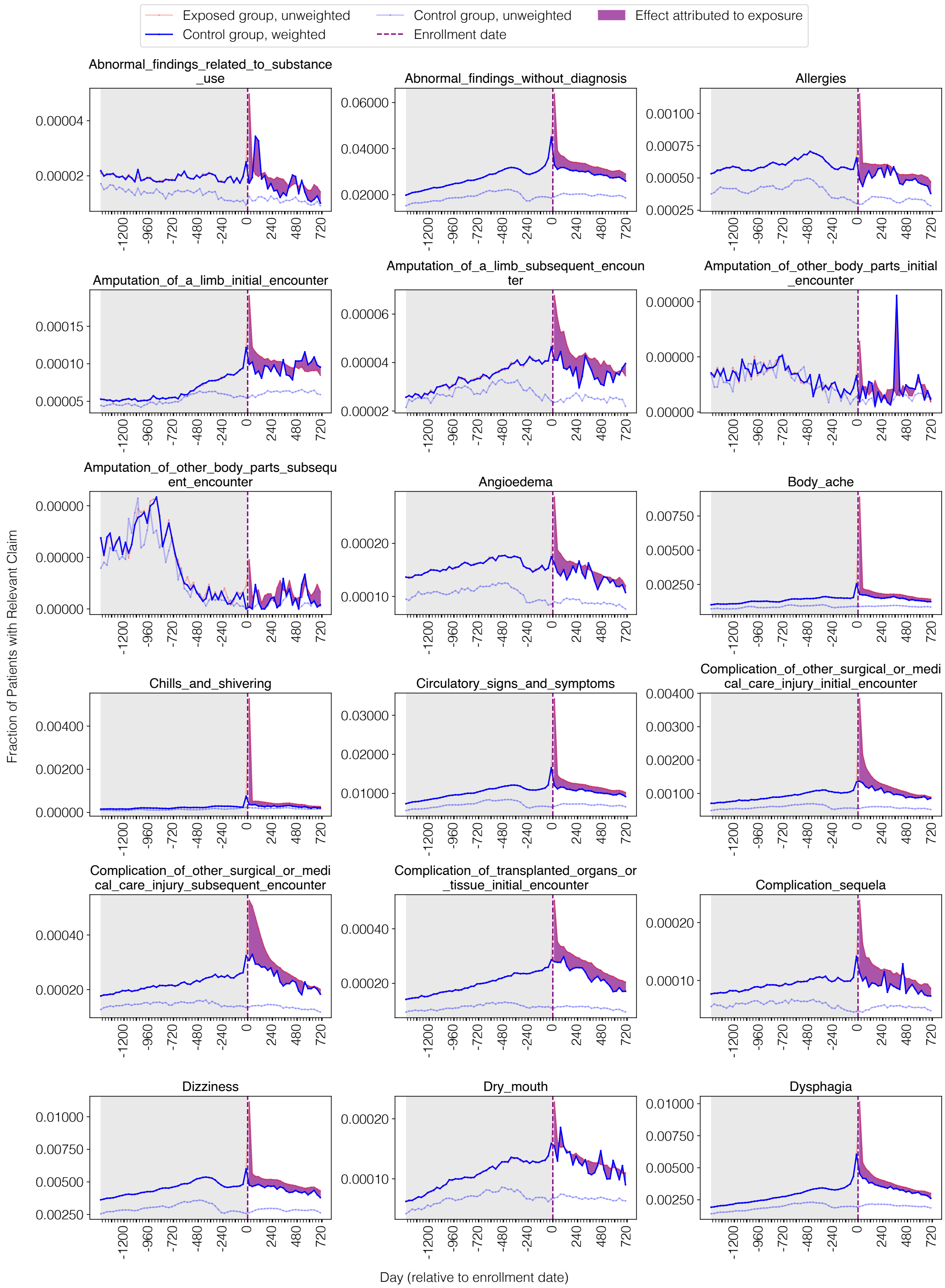


**Fig. S121.** Temporal trends in Misc prevalence. The fraction of patients with relevant claims over time belonging for each outcome in the **unrestricted control design with placebo cases** (negative COVID-19 PCR test) experiment.

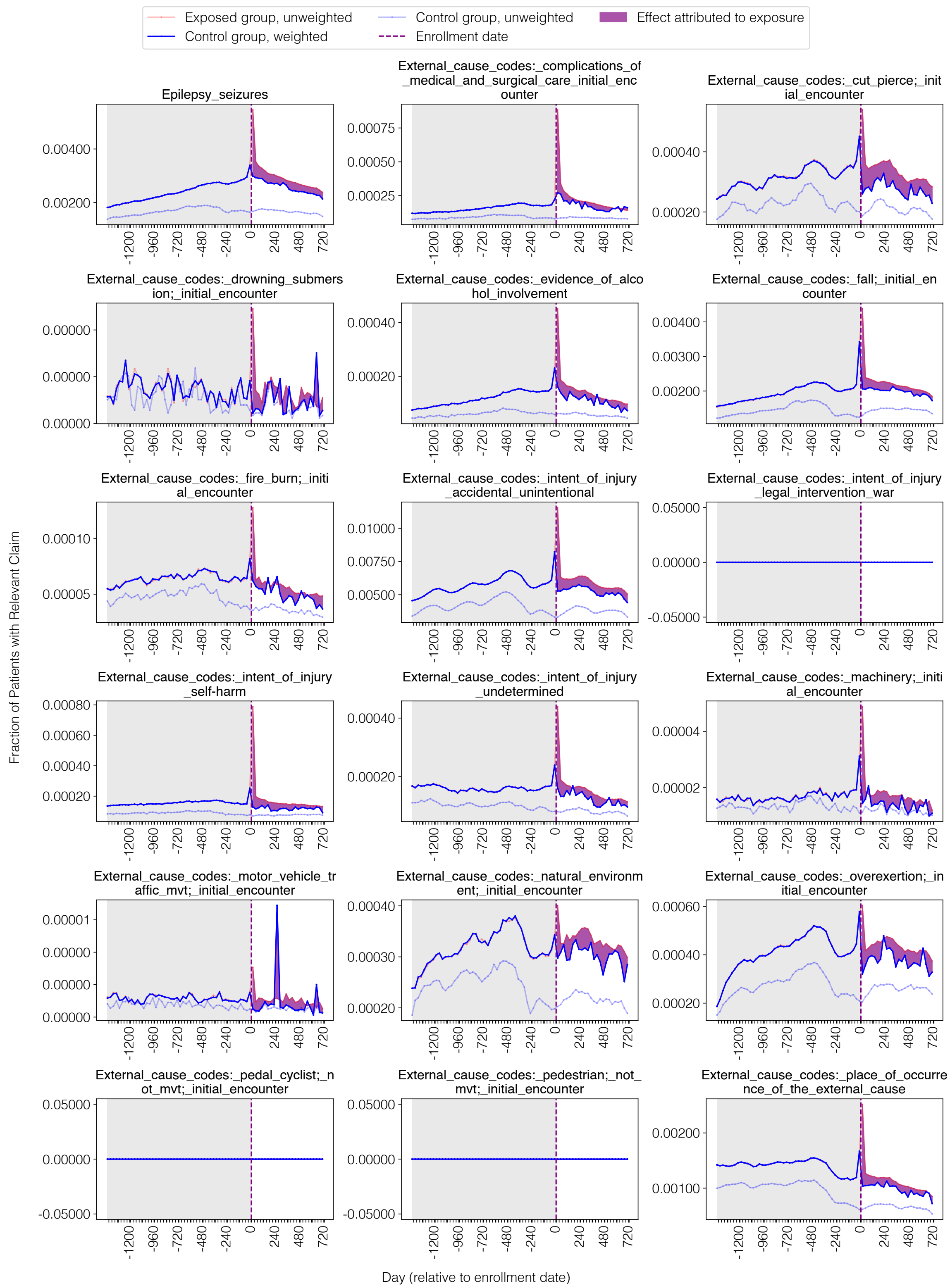


**Fig. S121.** (cont'd) Temporal trends in Misc prevalence. The fraction of patients with relevant claims over time belonging for each outcome in the **unrestricted control design with placebo cases** (negative COVID-19 PCR test) experiment.

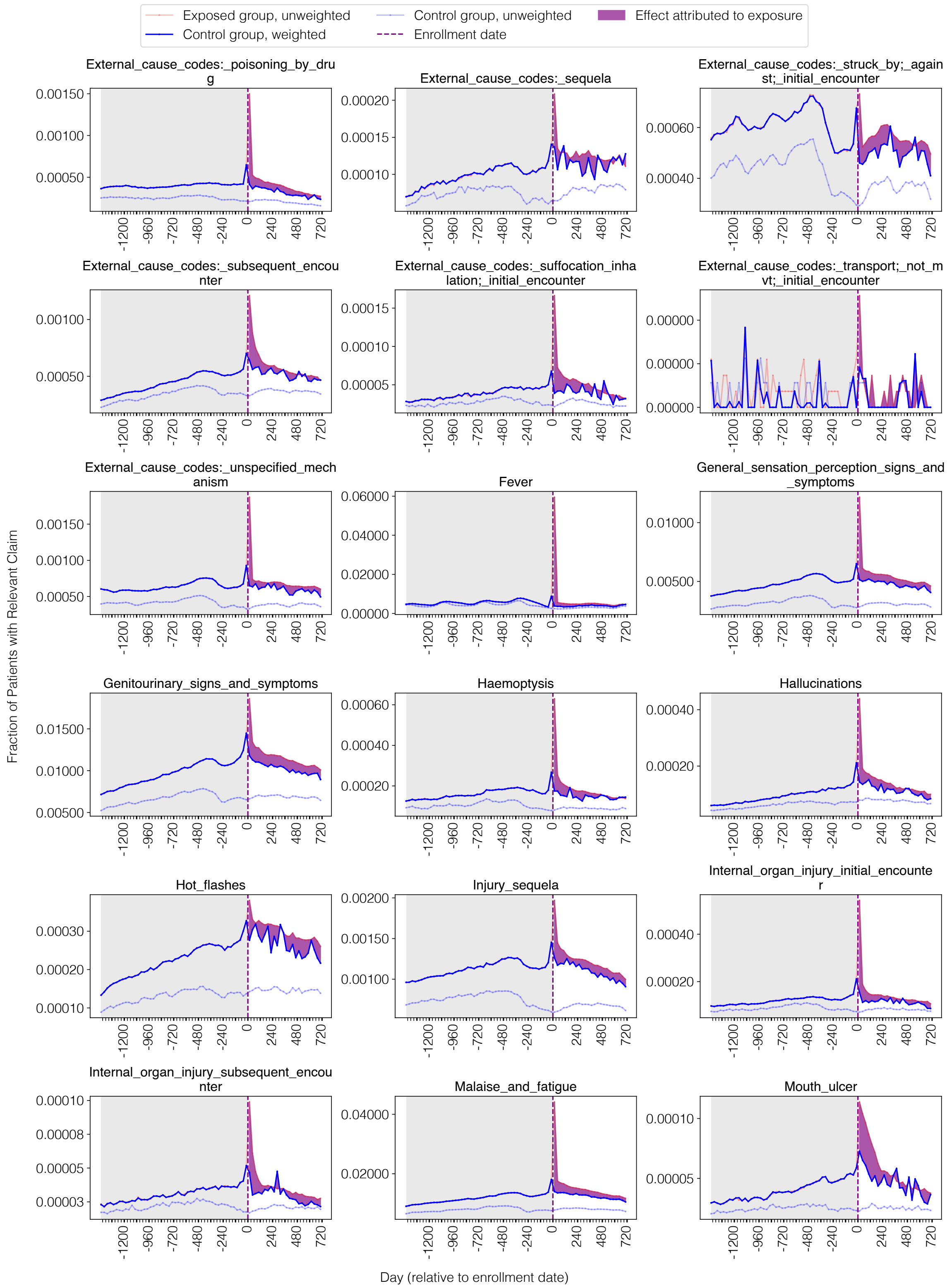


**Fig. S121.** (cont'd) Temporal trends in Misc prevalence. The fraction of patients with relevant claims over time belonging for each outcome in the **unrestricted control design with placebo cases** (negative COVID-19 PCR test) experiment.

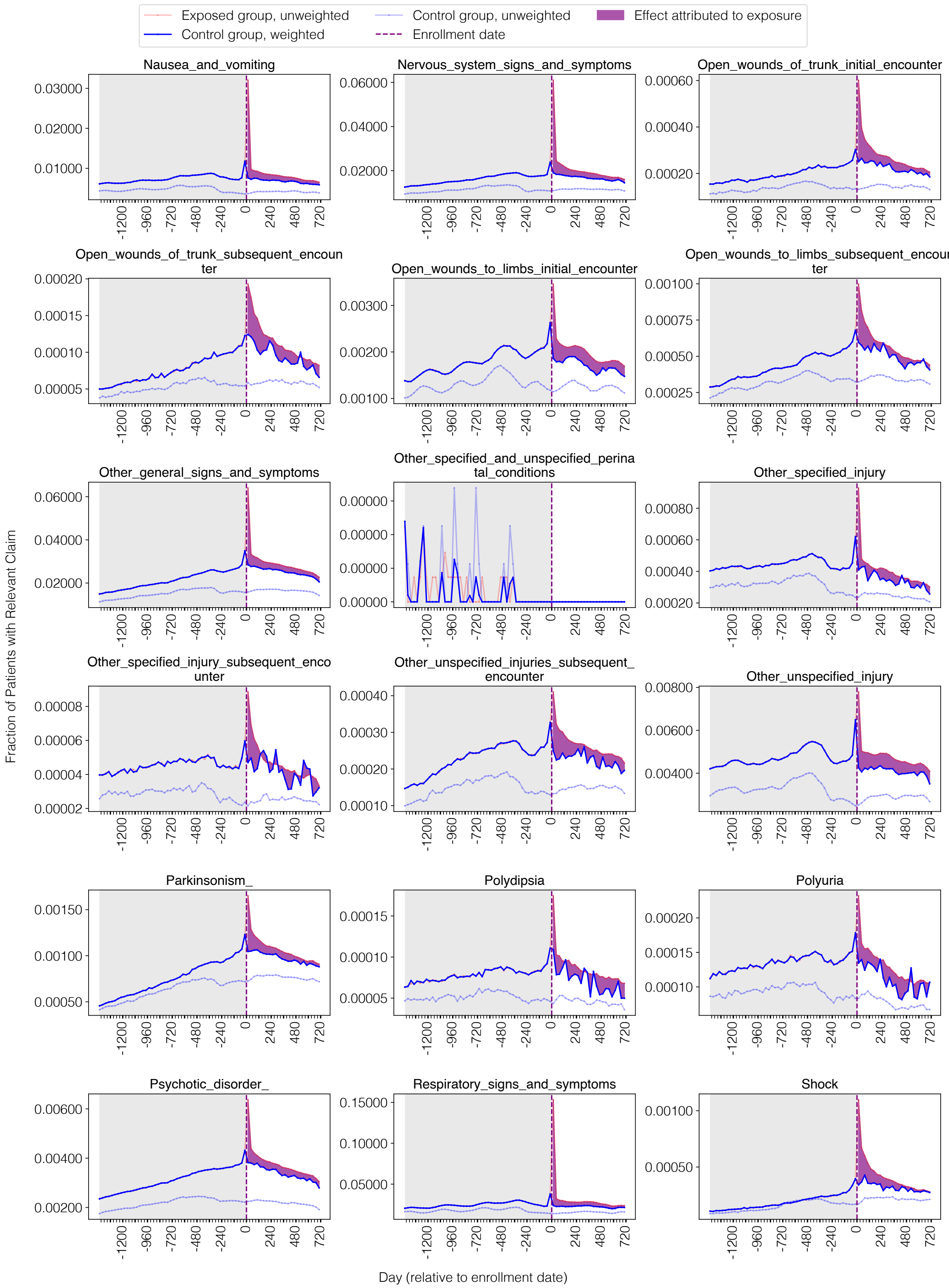


**Fig. S121.** (cont'd) Temporal trends in Misc prevalence. The fraction of patients with relevant claims over time belonging for each outcome in the **unrestricted control design with placebo cases** (negative COVID-19 PCR test) experiment.

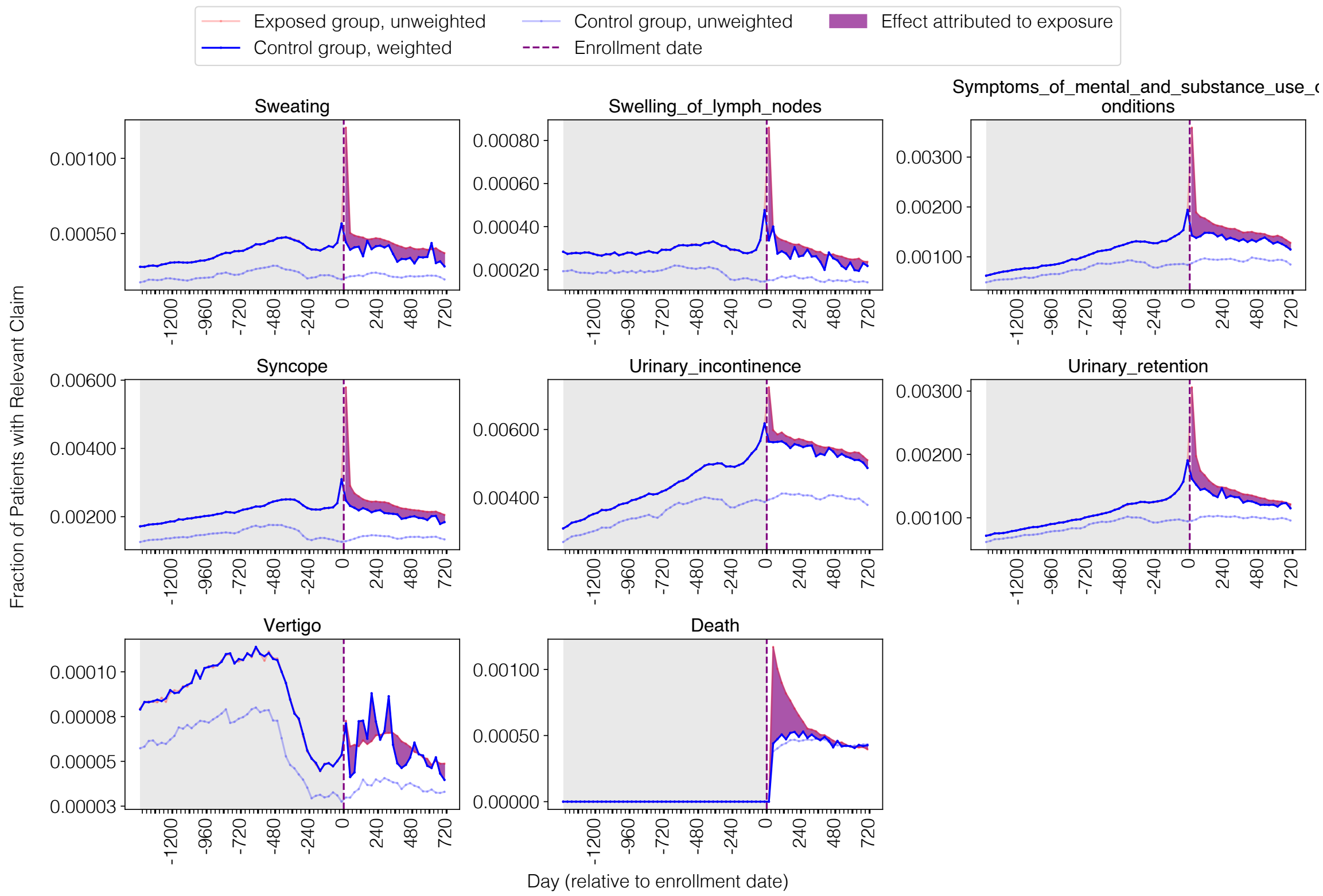


**Fig. S121.** (cont'd) Temporal trends in Misc prevalence. The fraction of patients with relevant claims over time belonging for each outcome in the **unrestricted control design with placebo cases** (negative COVID-19 PCR test) experiment.

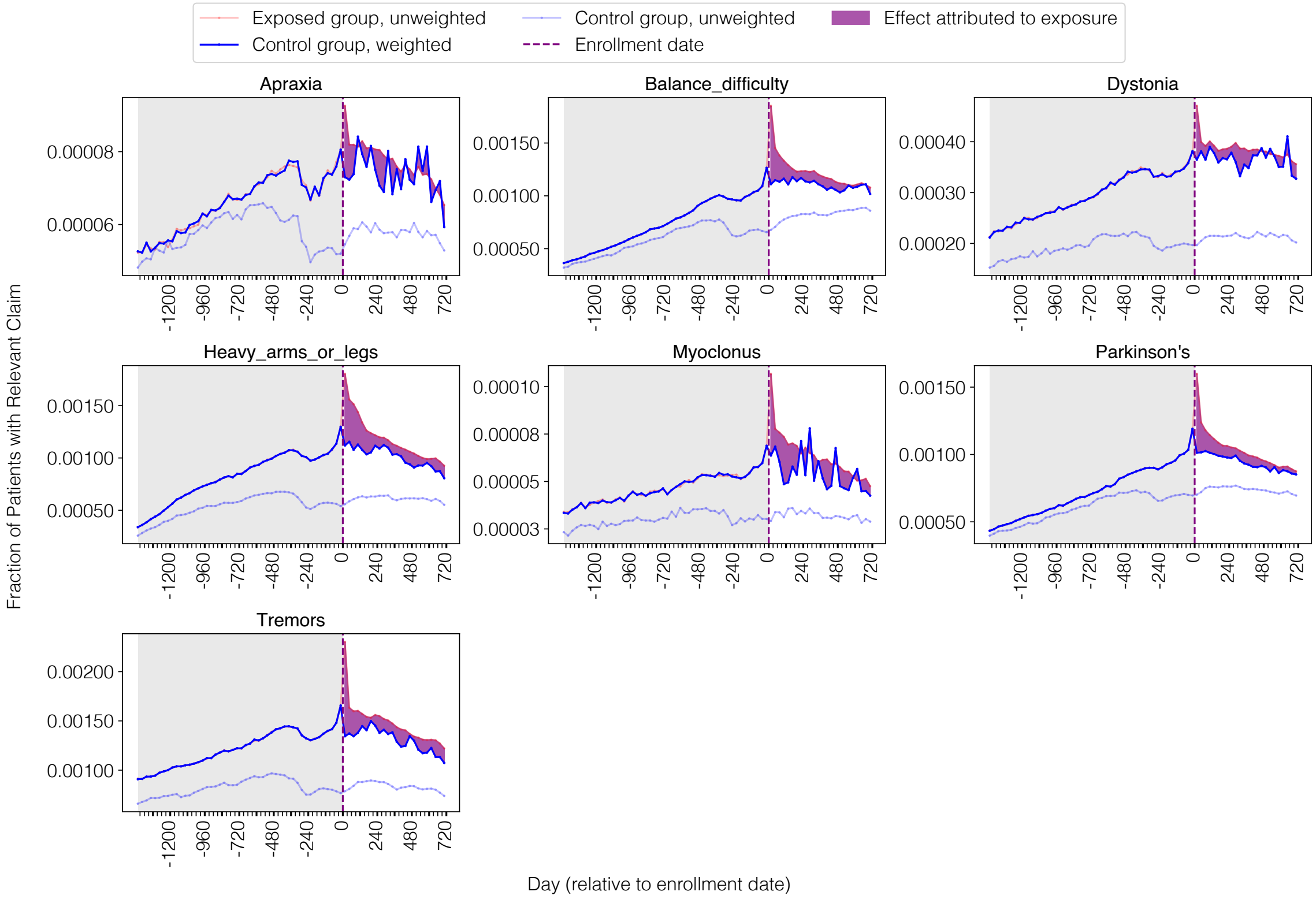


**Fig. S122.** Temporal trends in Movement prevalence. The fraction of patients with relevant claims over time belonging for each outcome in the **unrestricted control design with placebo cases** (negative COVID-19 PCR test) experiment.

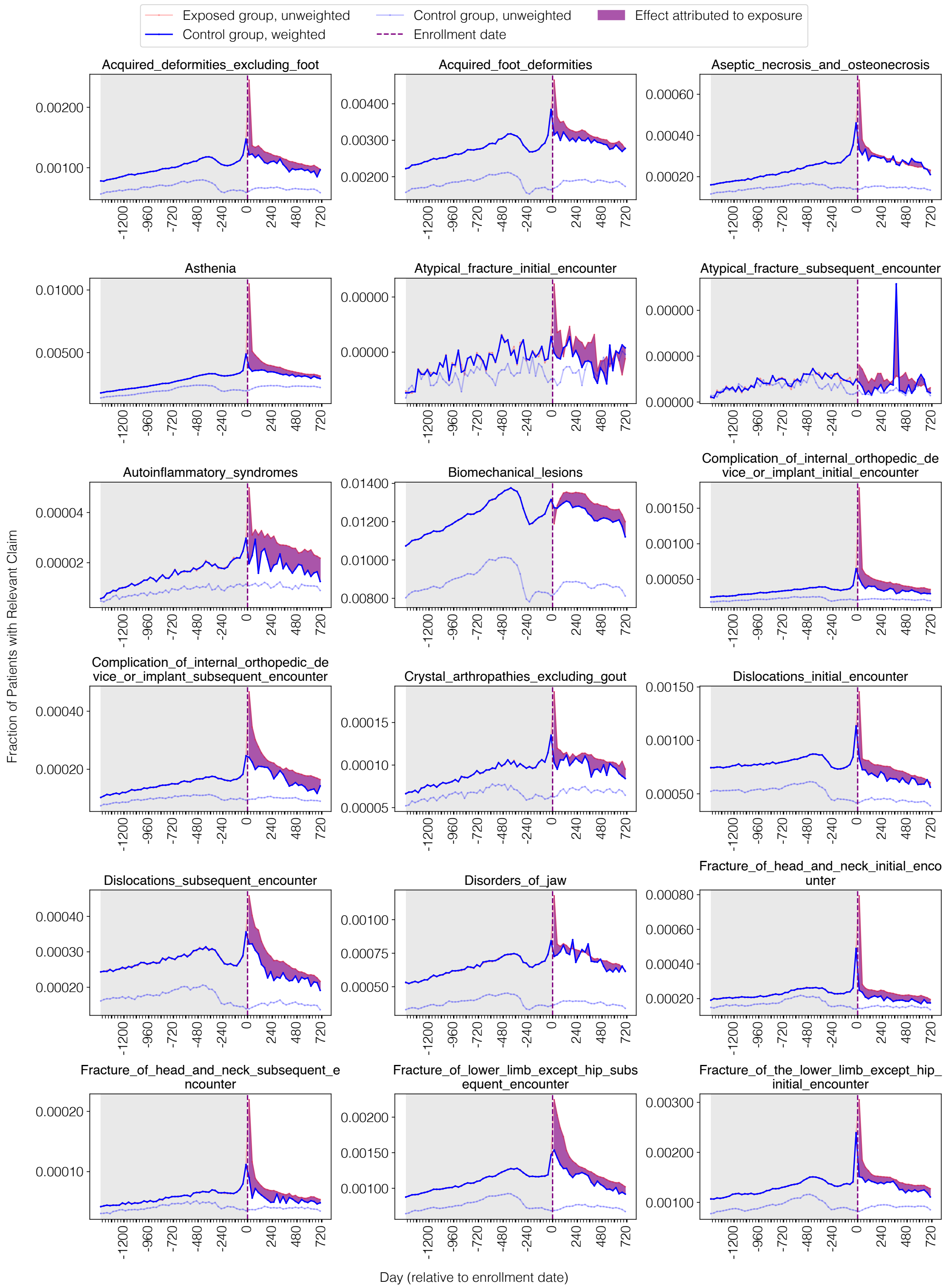


**Fig. S123.** Temporal trends in Muscles and joints prevalence. The fraction of patients with relevant claims over time belonging for each outcome in the **unrestricted control design with placebo cases** (negative COVID-19 PCR test) experiment.

 

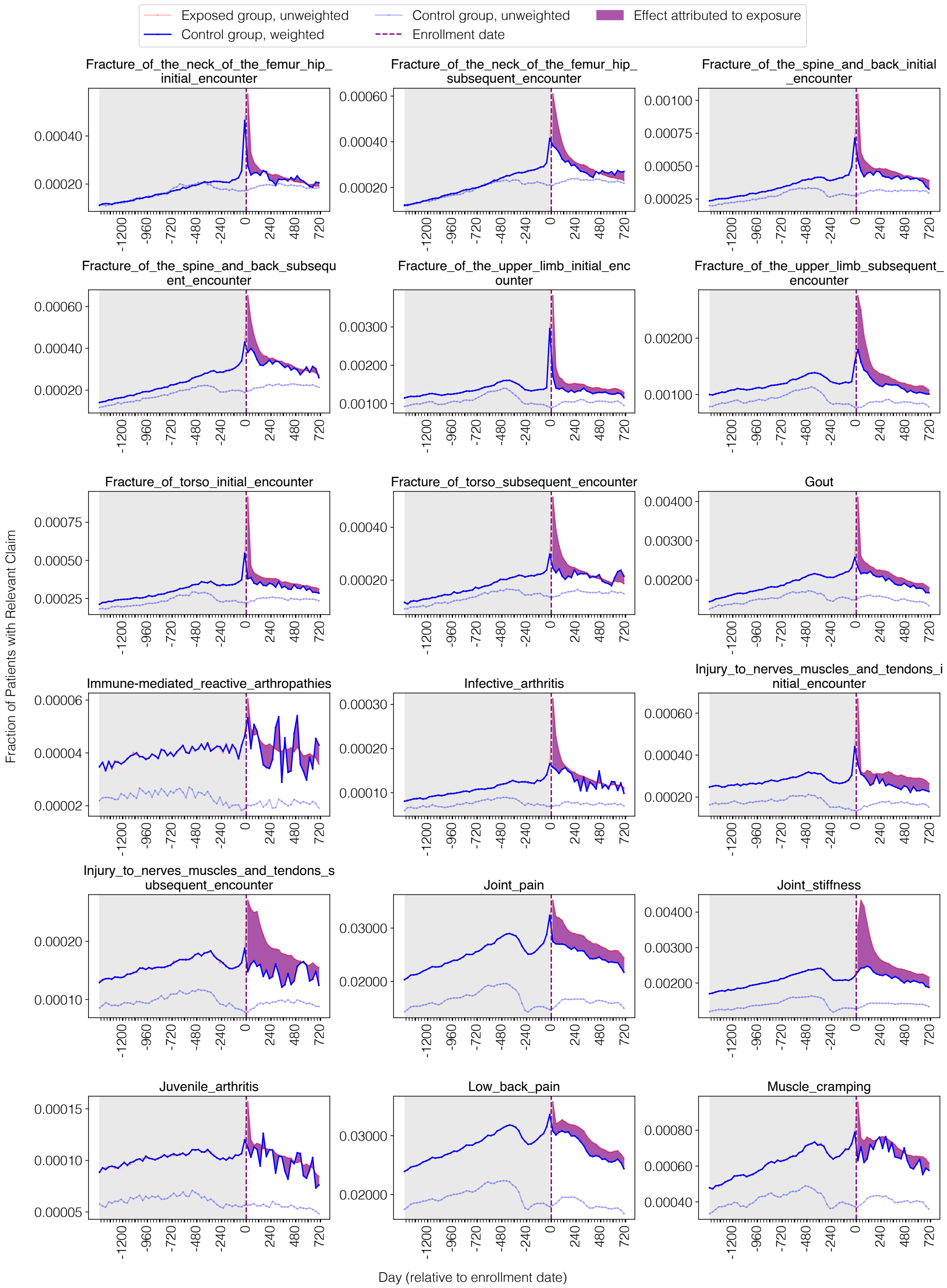


**Fig. S123.** (cont'd) Temporal trends in Muscles and joints prevalence. The fraction of patients with relevant claims over time belonging for each outcome in the **unrestricted control design with placebo cases** (negative COVID-19 PCR test) experiment.

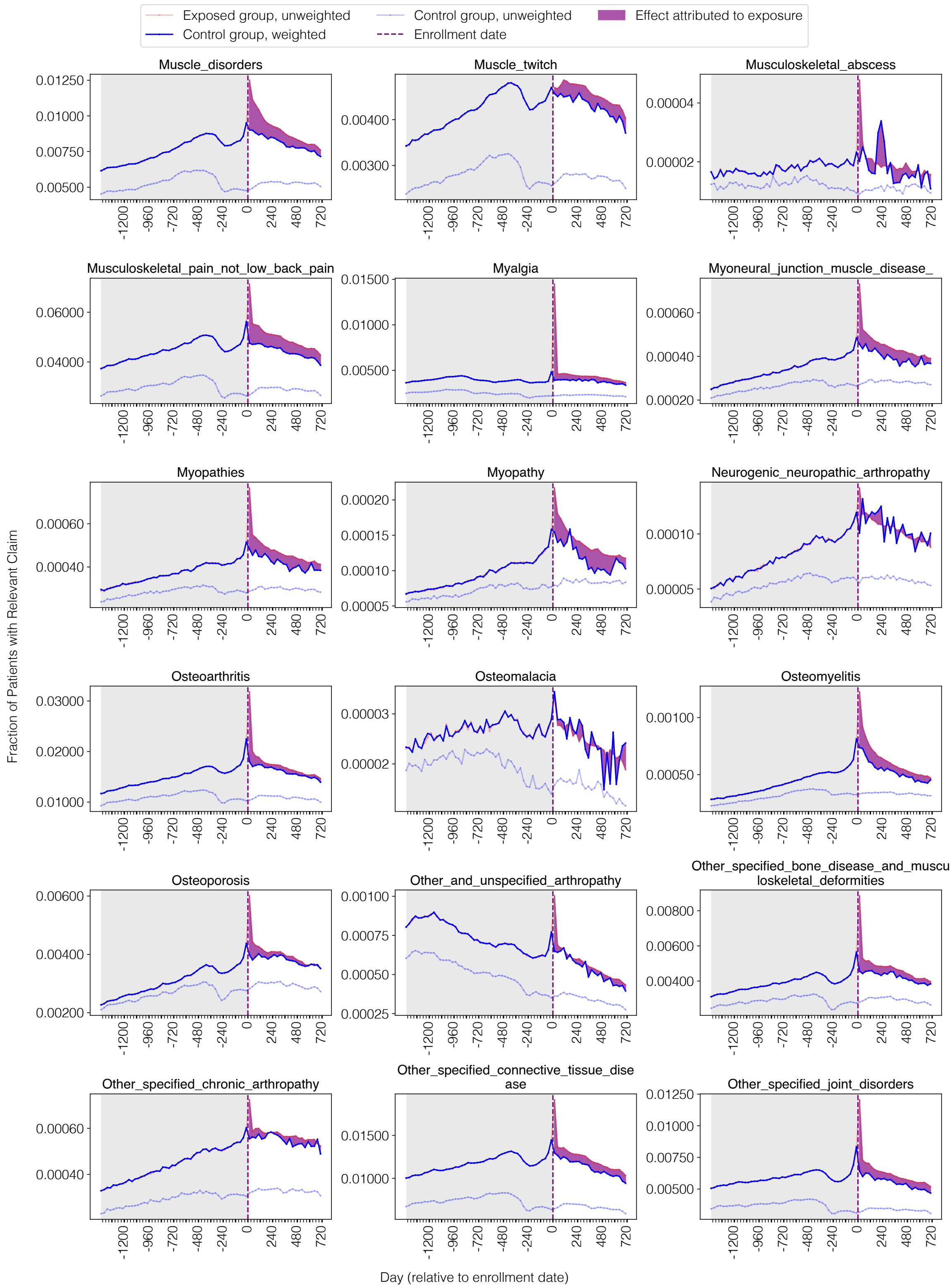


**Fig. S123.** (cont'd) Temporal trends in Muscles and joints prevalence. The fraction of patients with relevant claims over time belonging for each outcome in the **unrestricted control design with placebo cases** (negative COVID-19 PCR test) experiment.

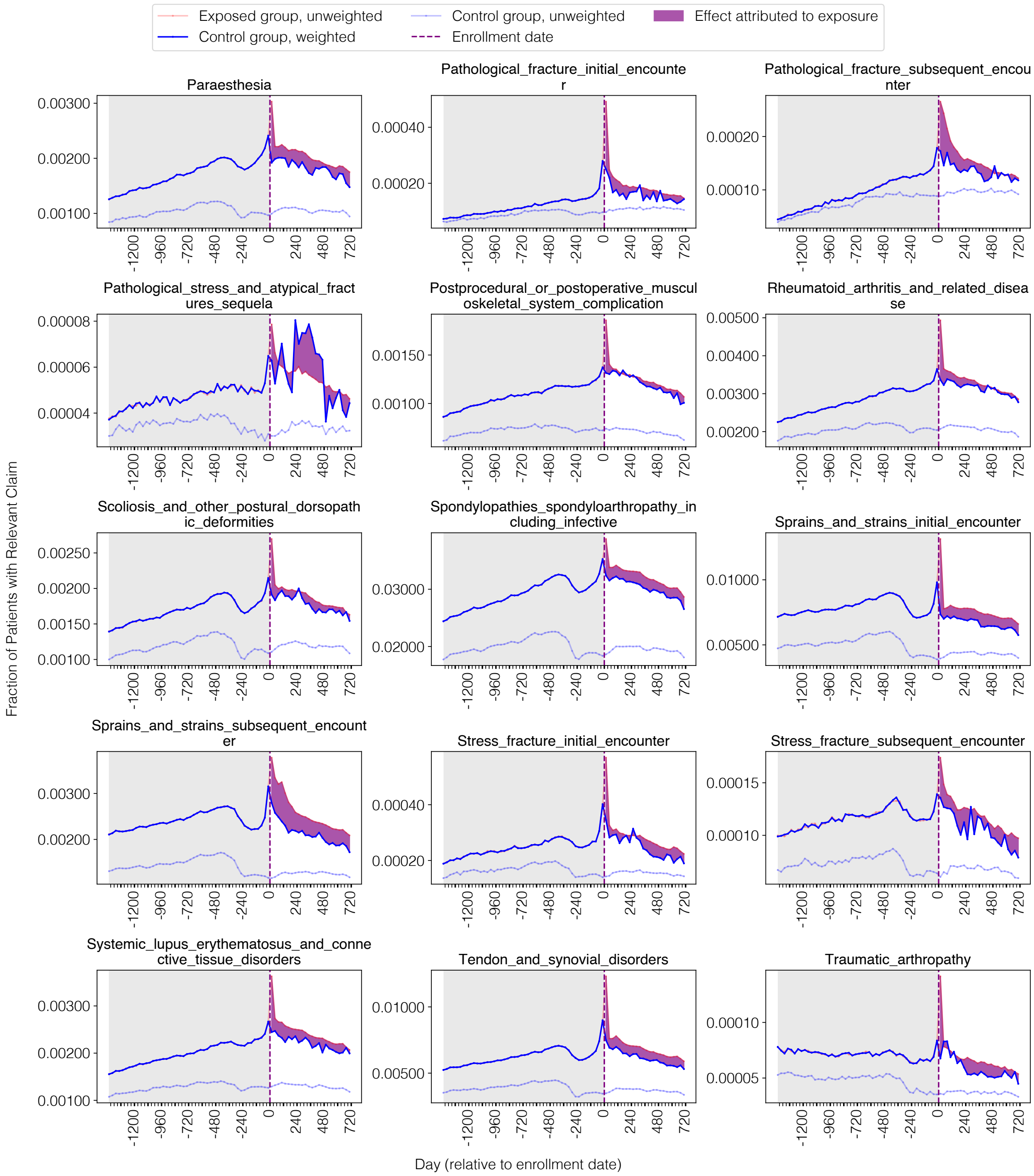


**Fig. S123.** (cont'd) Temporal trends in Muscles and joints prevalence. The fraction of patients with relevant claims over time belonging for each outcome in the **unrestricted control design with placebo cases** (negative COVID-19 PCR test) experiment.

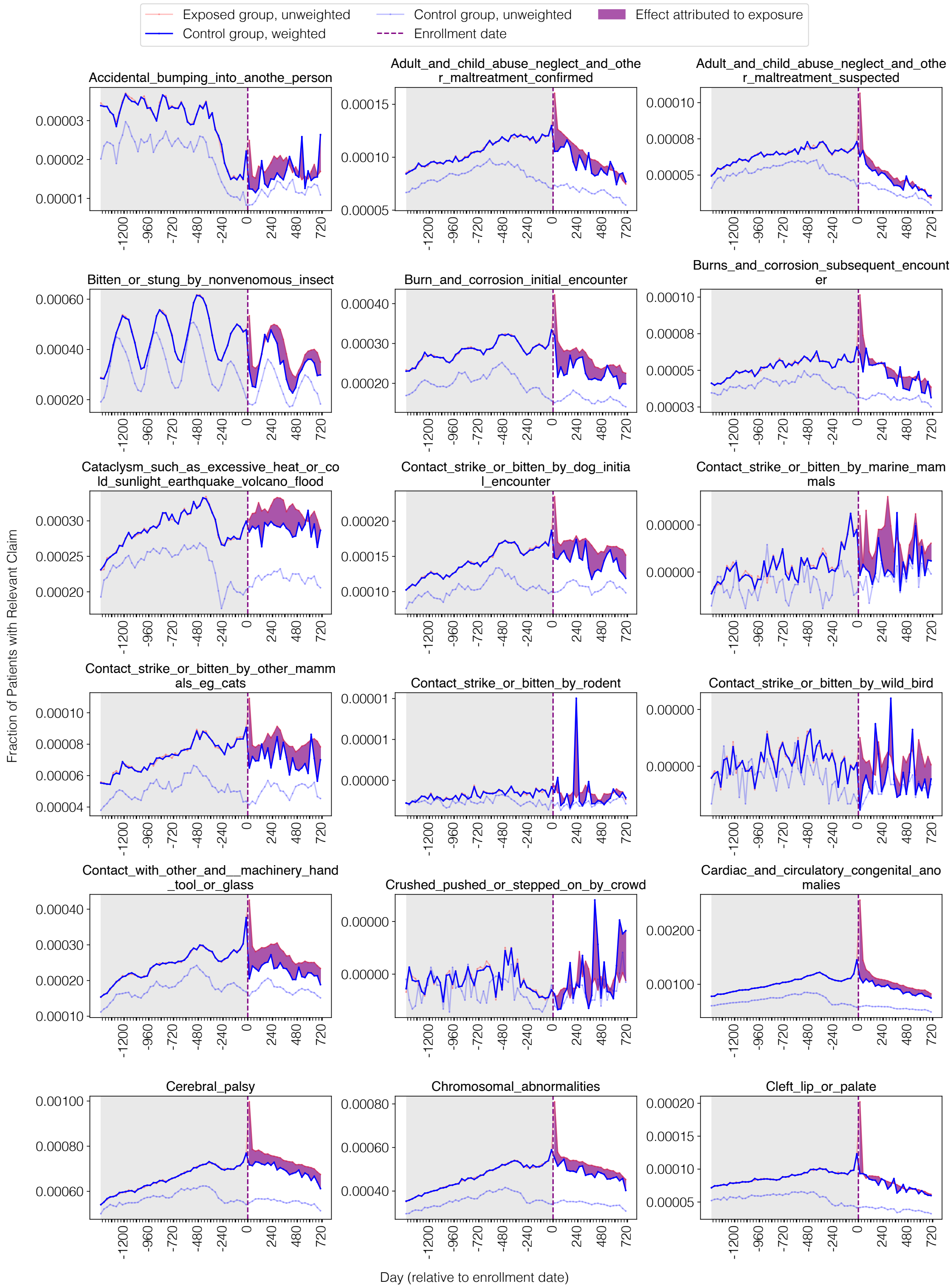


**Fig. S124.** Temporal trends in Negative prevalence. The fraction of patients with relevant claims over time belonging for each outcome in the **unrestricted control design with placebo cases** (negative COVID-19 PCR test) experiment.

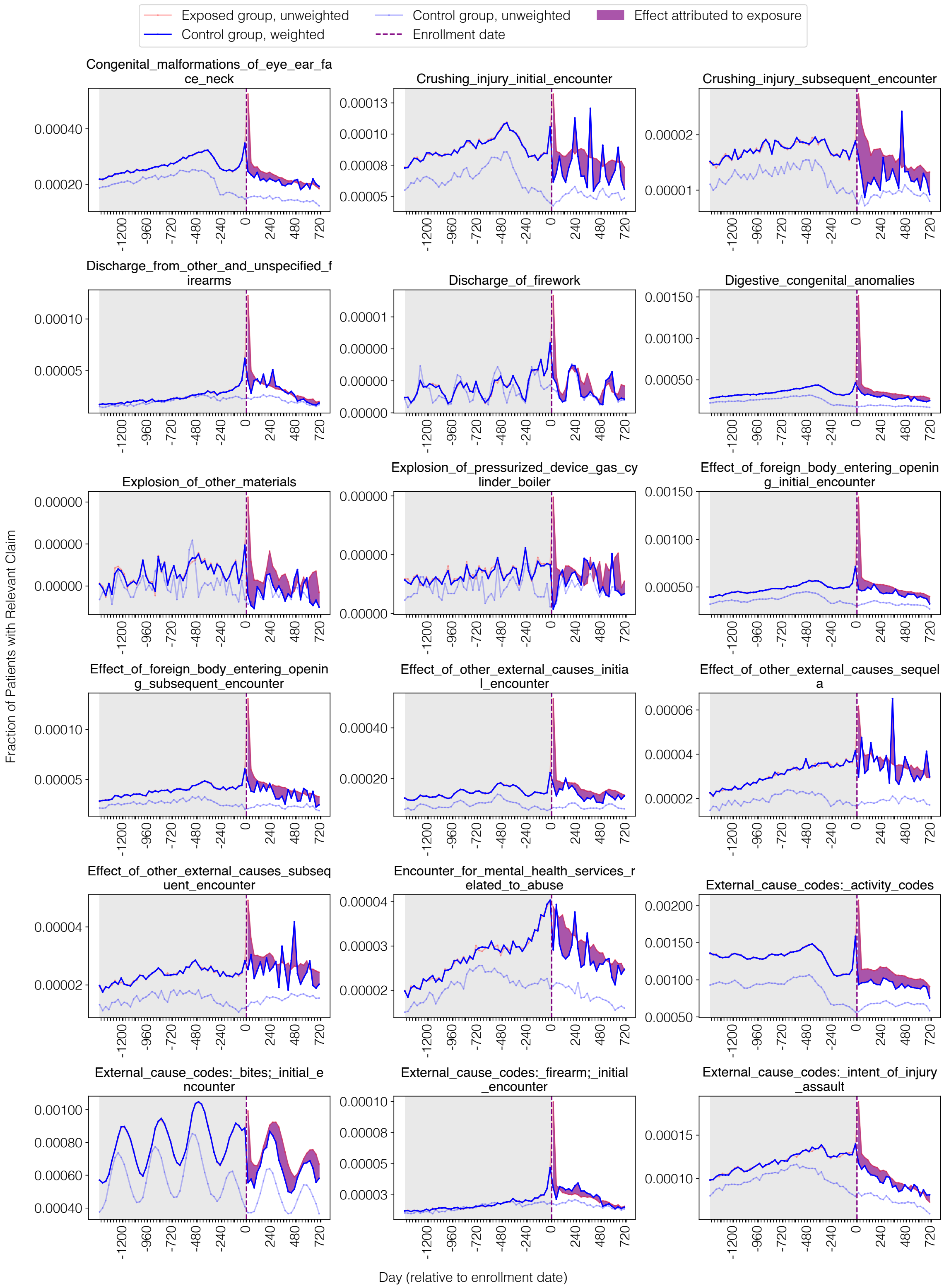


**Fig. S124.** (cont'd) Temporal trends in Negative prevalence. The fraction of patients with relevant claims over time belonging for each outcome in the **unrestricted control design with placebo cases** (negative COVID-19 PCR test) experiment.

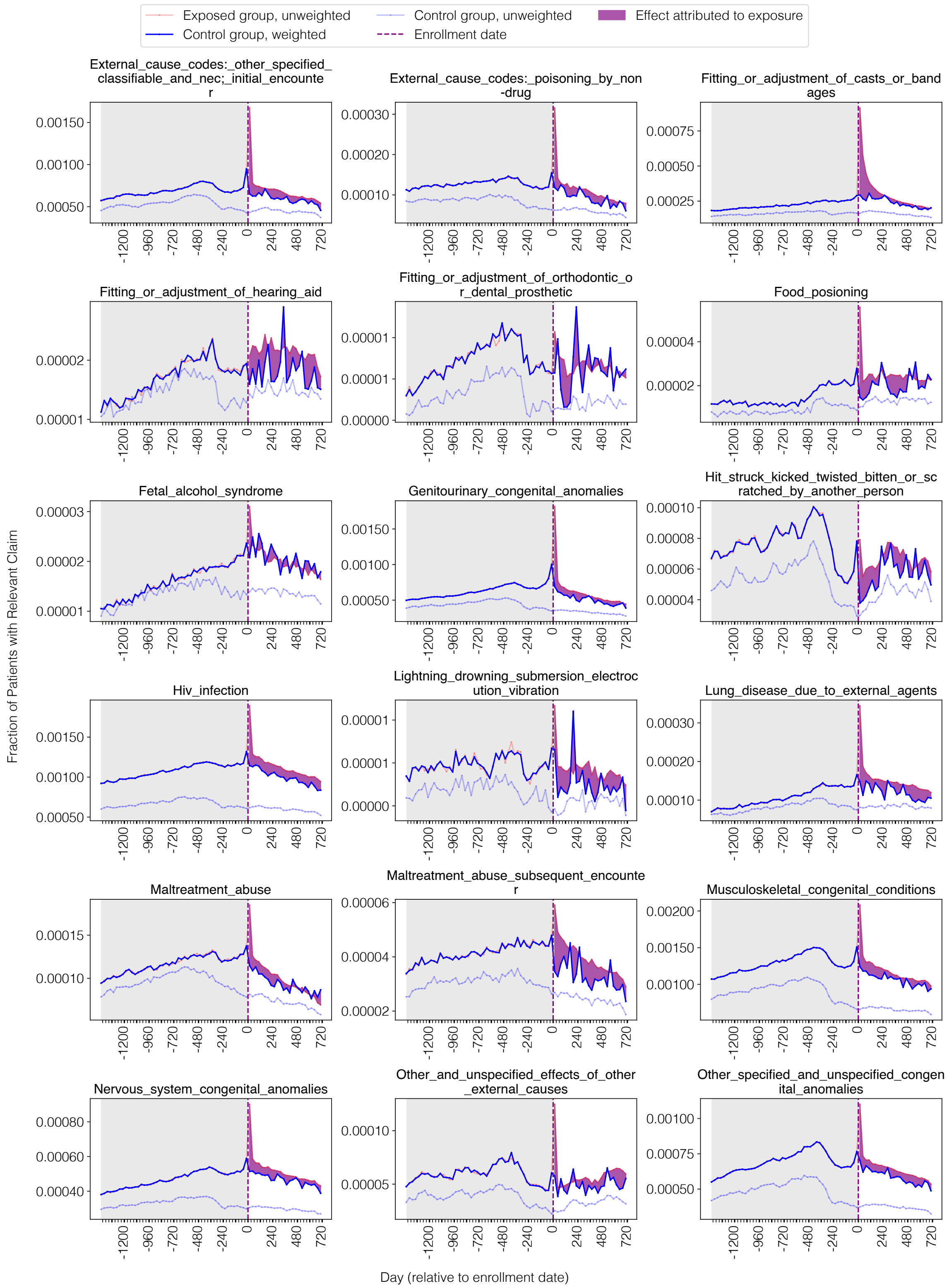


**Fig. S124.** (cont'd) Temporal trends in Negative prevalence. The fraction of patients with relevant claims over time belonging for each outcome in the **unrestricted control design with placebo cases** (negative COVID-19 PCR test) experiment.

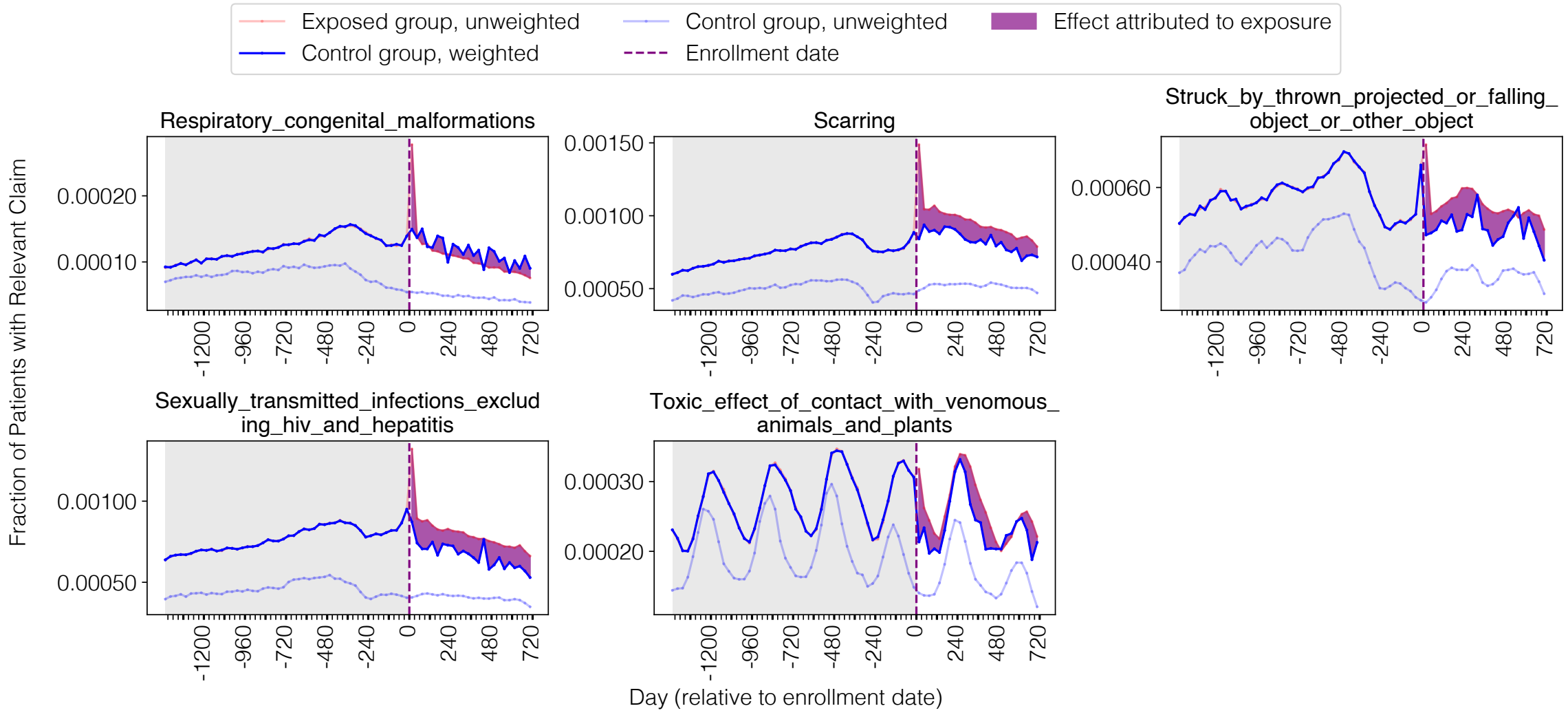


**Fig. S124.** (cont'd) Temporal trends in Negative prevalence. The fraction of patients with relevant claims over time belonging for each outcome in the **unrestricted control design with placebo cases** (negative COVID-19 PCR test) experiment.

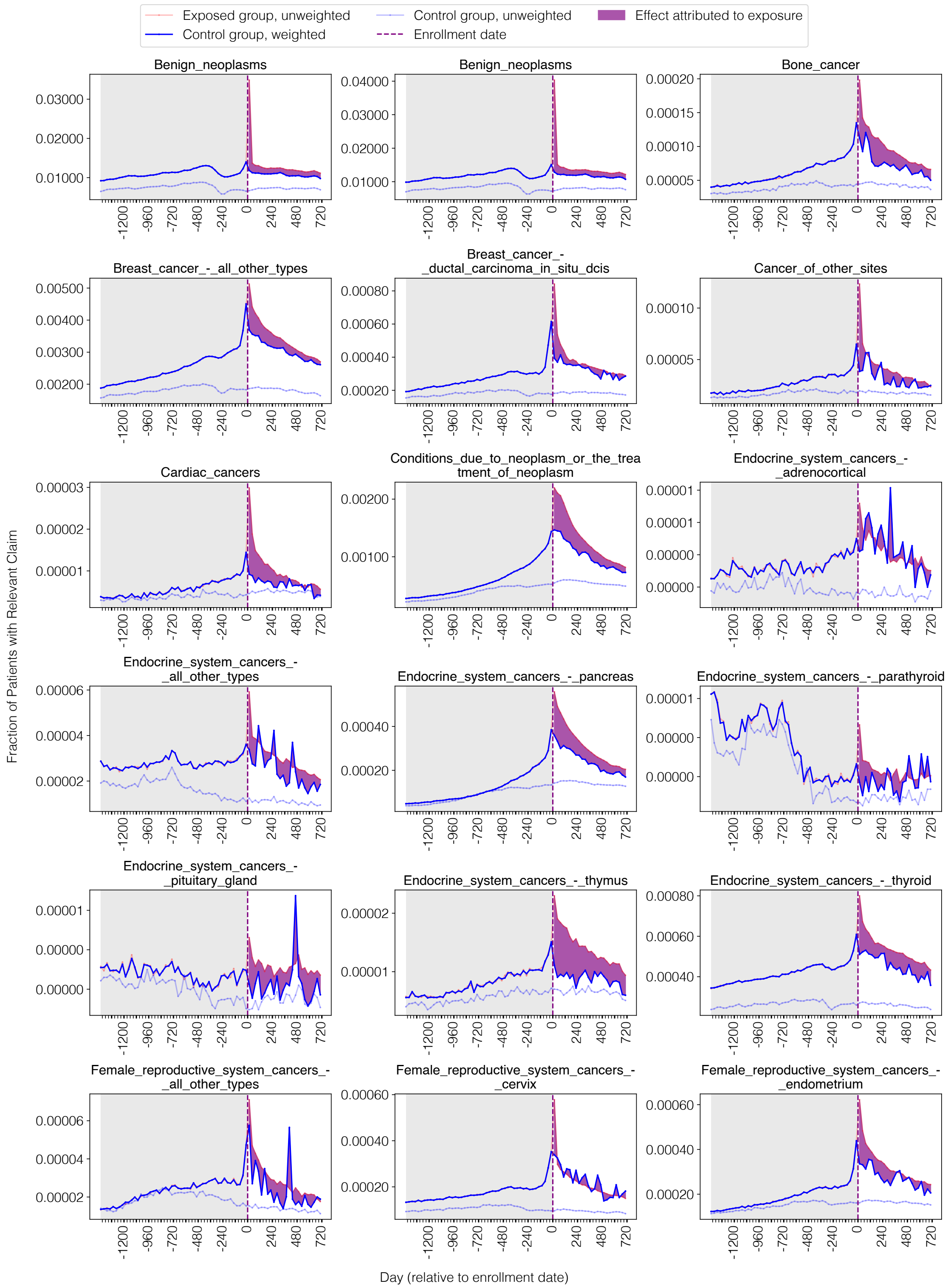


**Fig. S125.** Temporal trends in Neoplasms prevalence. The fraction of patients with relevant claims over time belonging for each outcome in the **unrestricted control design with placebo cases** (negative COVID-19 PCR test) experiment.

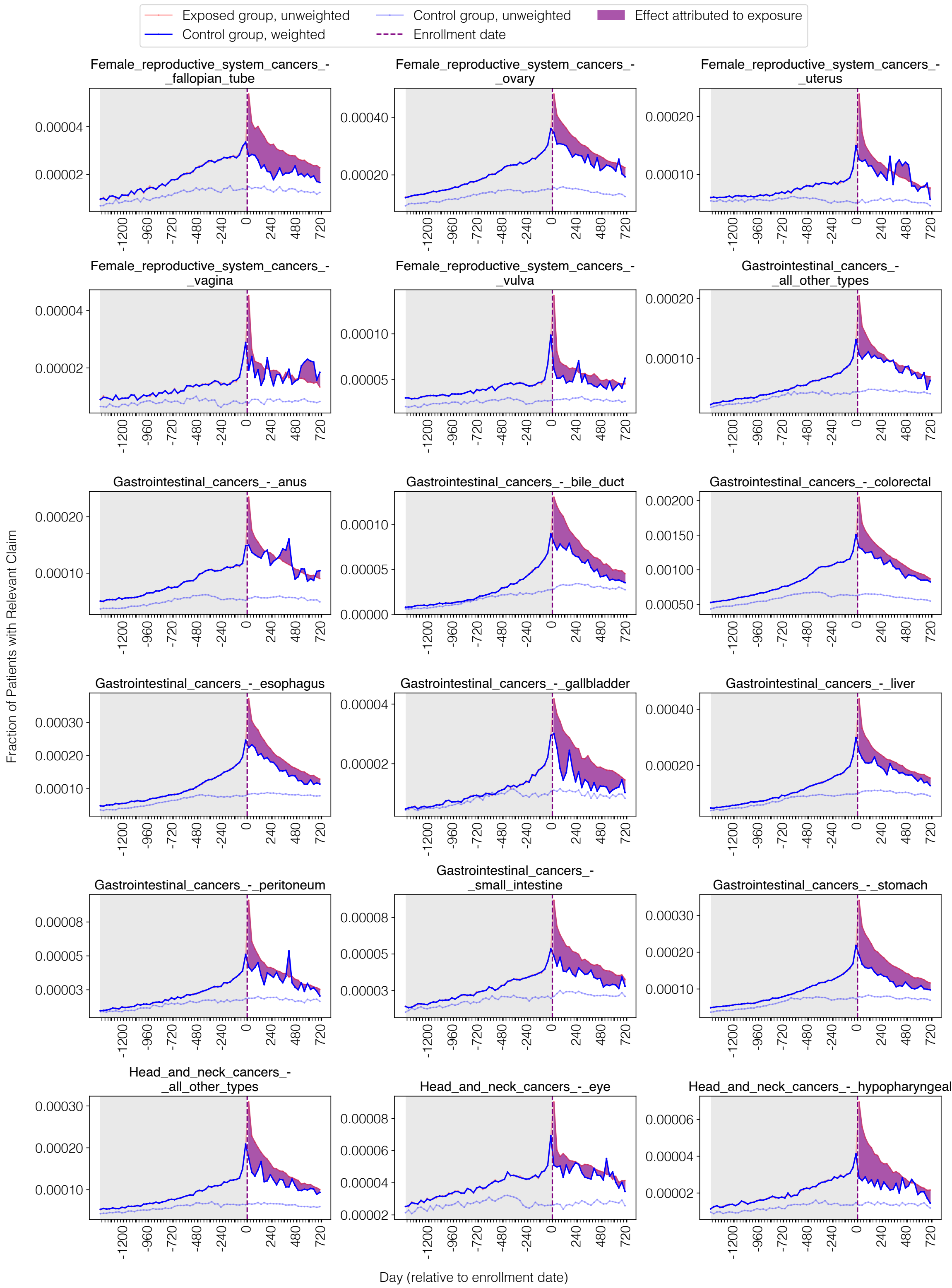


**Fig. S125.** (cont'd) Temporal trends in Neoplasms prevalence. The fraction of patients with relevant claims over time belonging for each outcome in the **unrestricted control design with placebo cases** (negative COVID-19 PCR test) experiment.

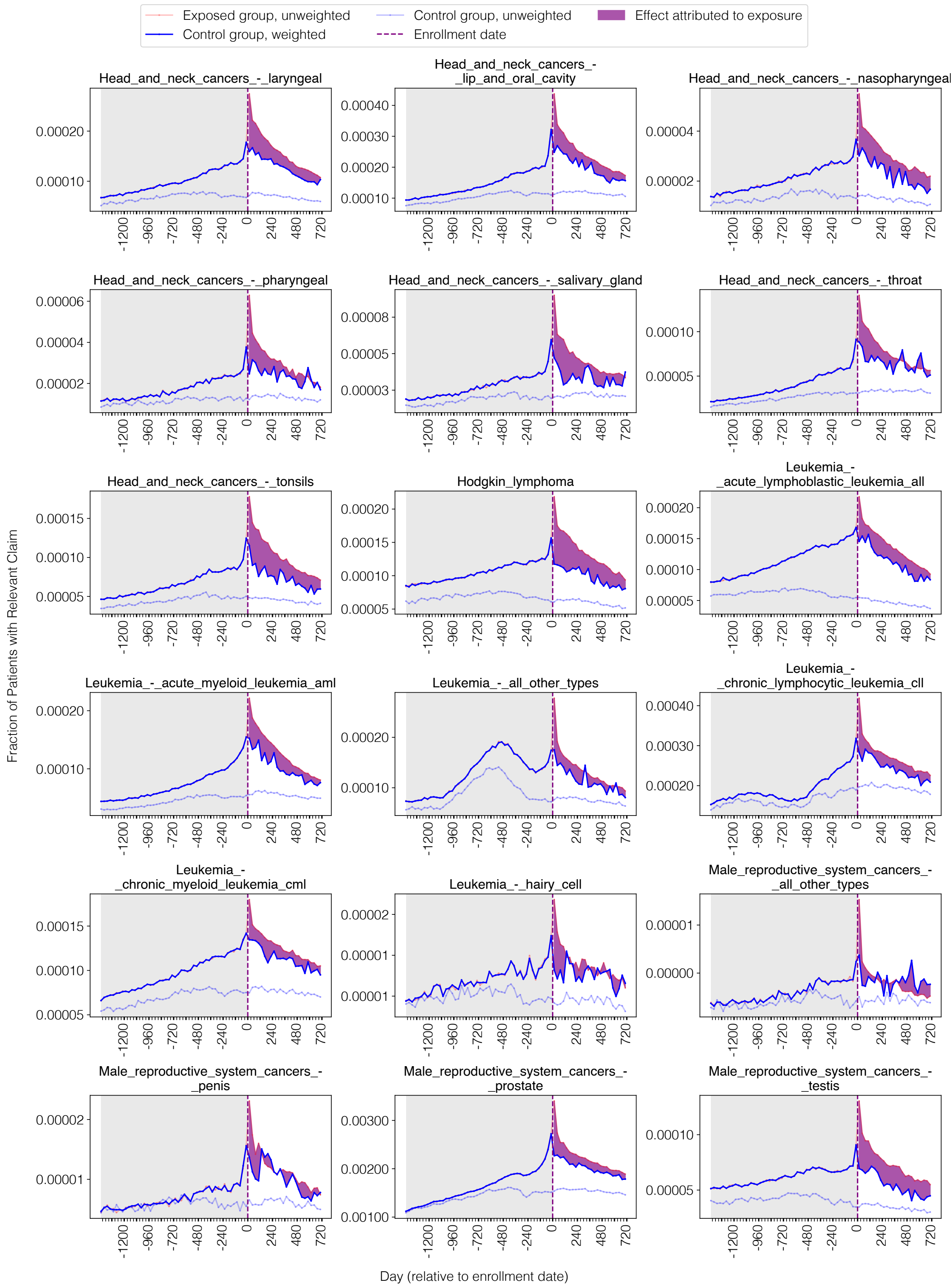


**Fig. S125.** (cont'd) Temporal trends in Neoplasms prevalence. The fraction of patients with relevant claims over time belonging for each outcome in the **unrestricted control design with placebo cases** (negative COVID-19 PCR test) experiment.

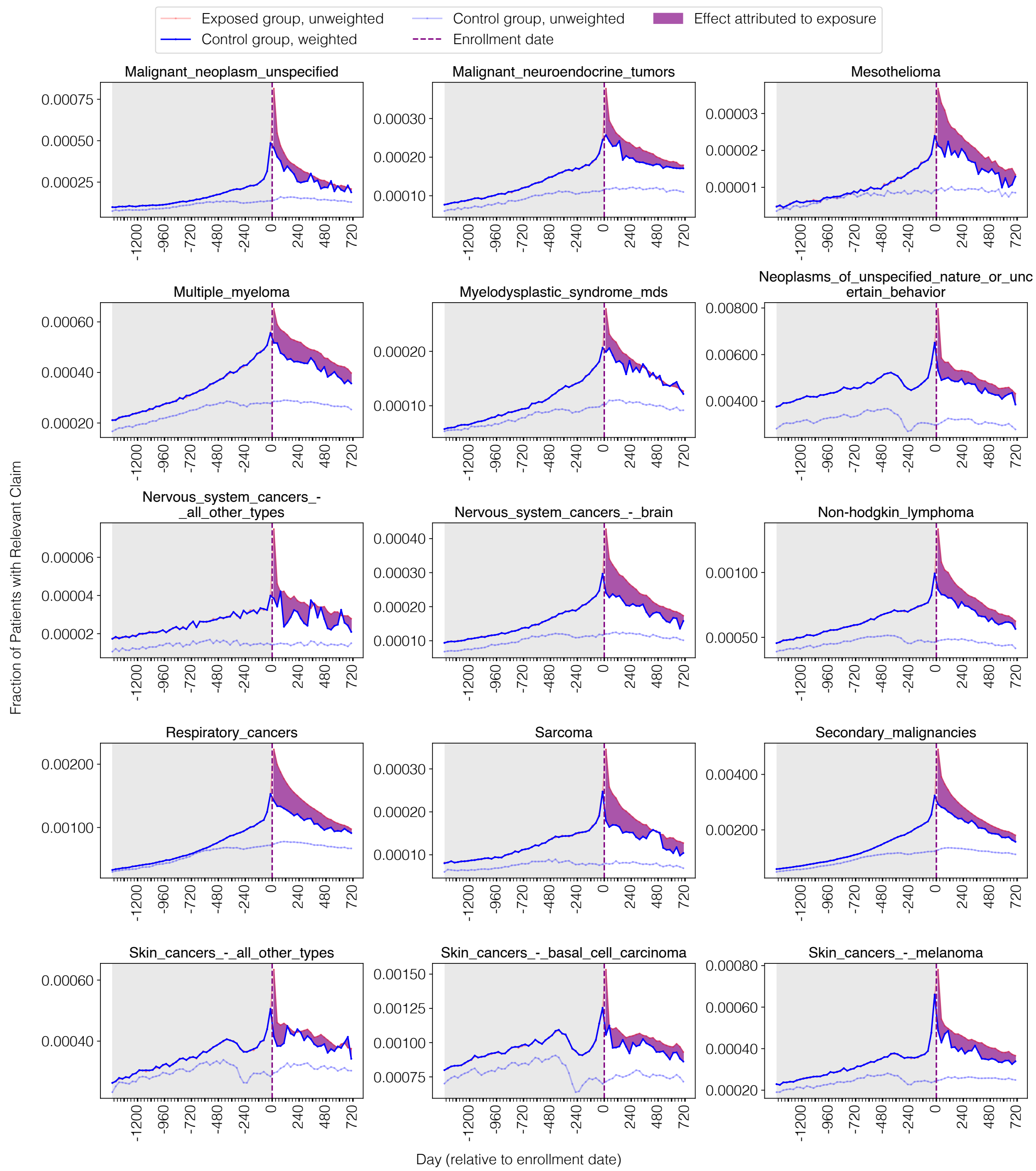


**Fig. S125.** (cont'd) Temporal trends in Neoplasms prevalence. The fraction of patients with relevant claims over time belonging for each outcome in the **unrestricted control design with placebo cases** (negative COVID-19 PCR test) experiment.

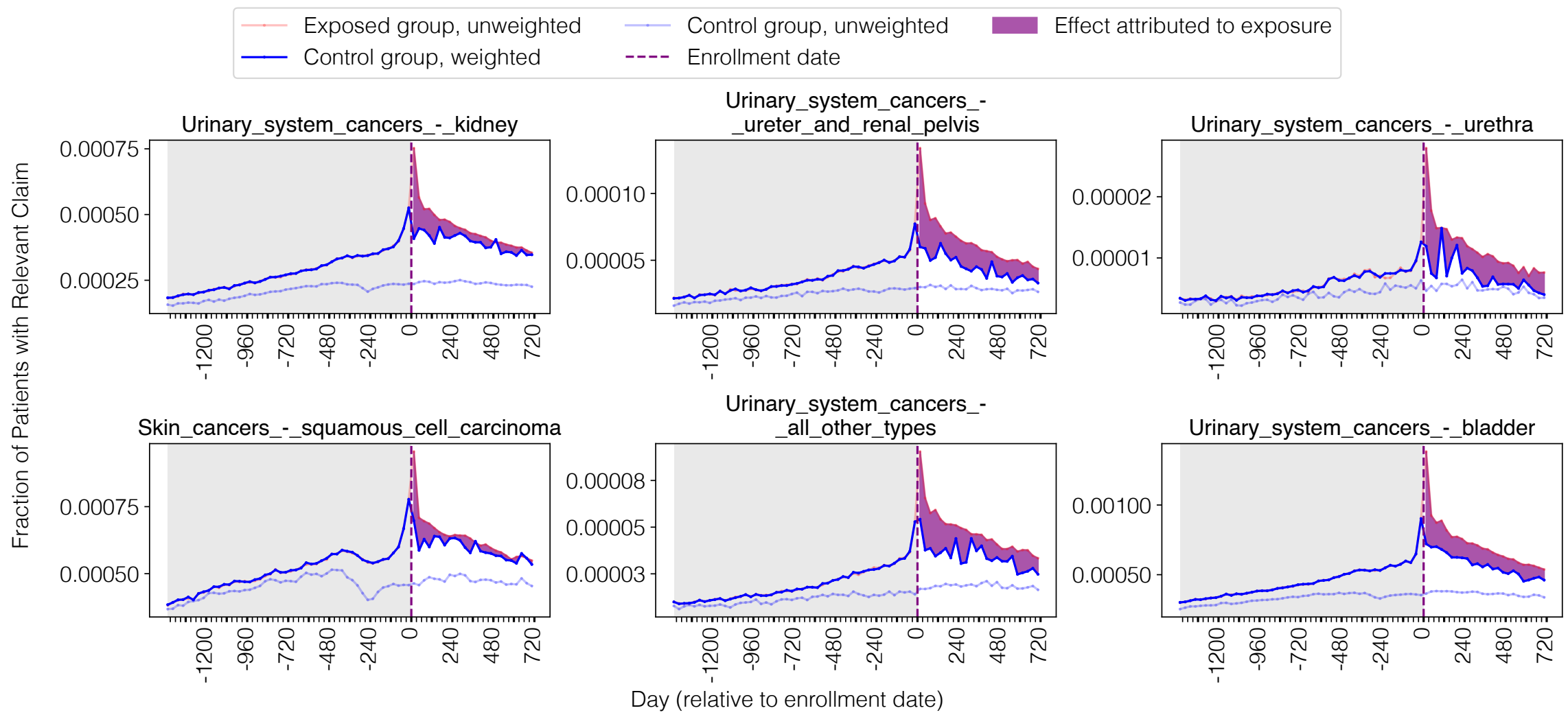


**Fig. S125.** (cont'd) Temporal trends in Neoplasms prevalence. The fraction of patients with relevant claims over time belonging for each outcome in the **unrestricted control design with placebo cases** (negative COVID-19 PCR test) experiment.

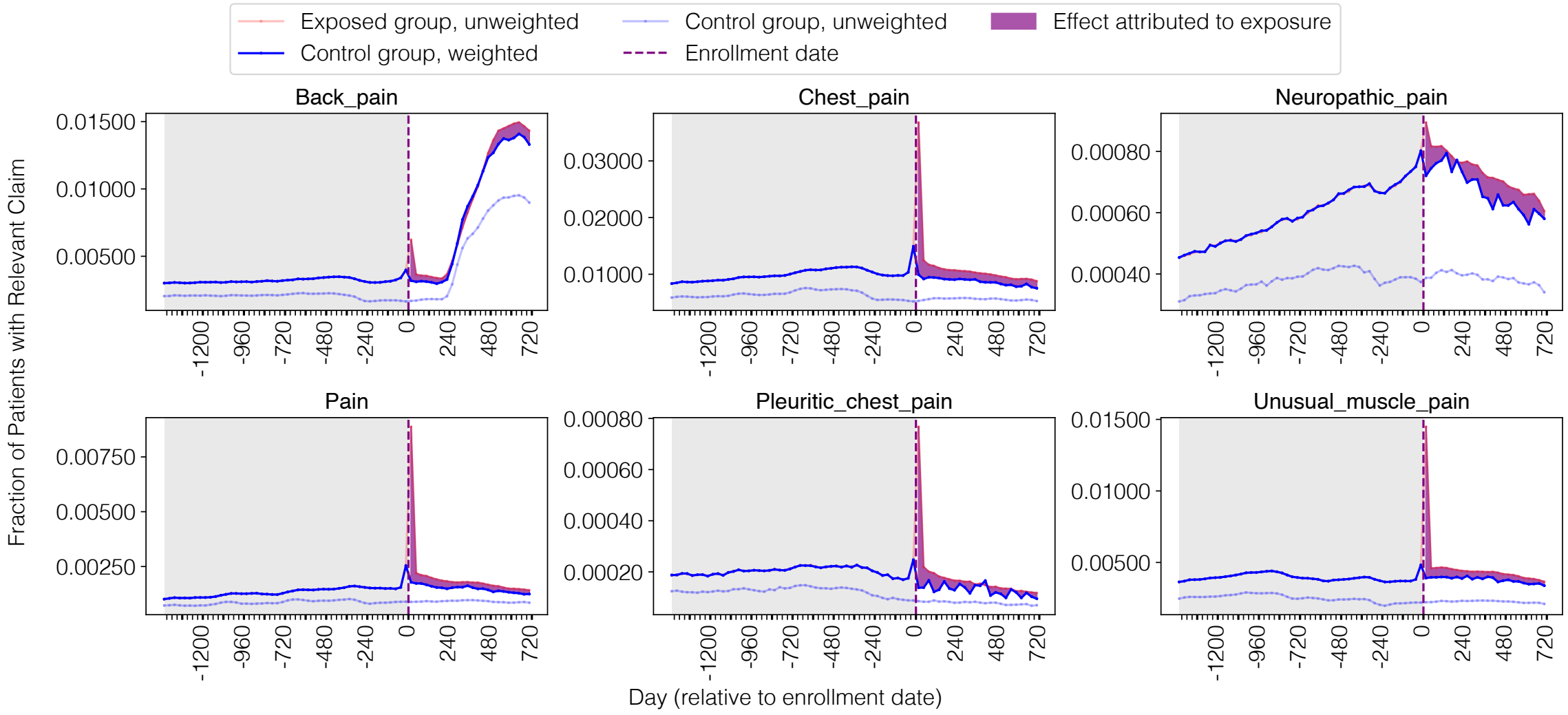


**Fig. S126.** Temporal trends in Pain prevalence. The fraction of patients with relevant claims over time belonging for each outcome in the **unrestricted control design with placebo cases** (negative COVID-19 PCR test) experiment.

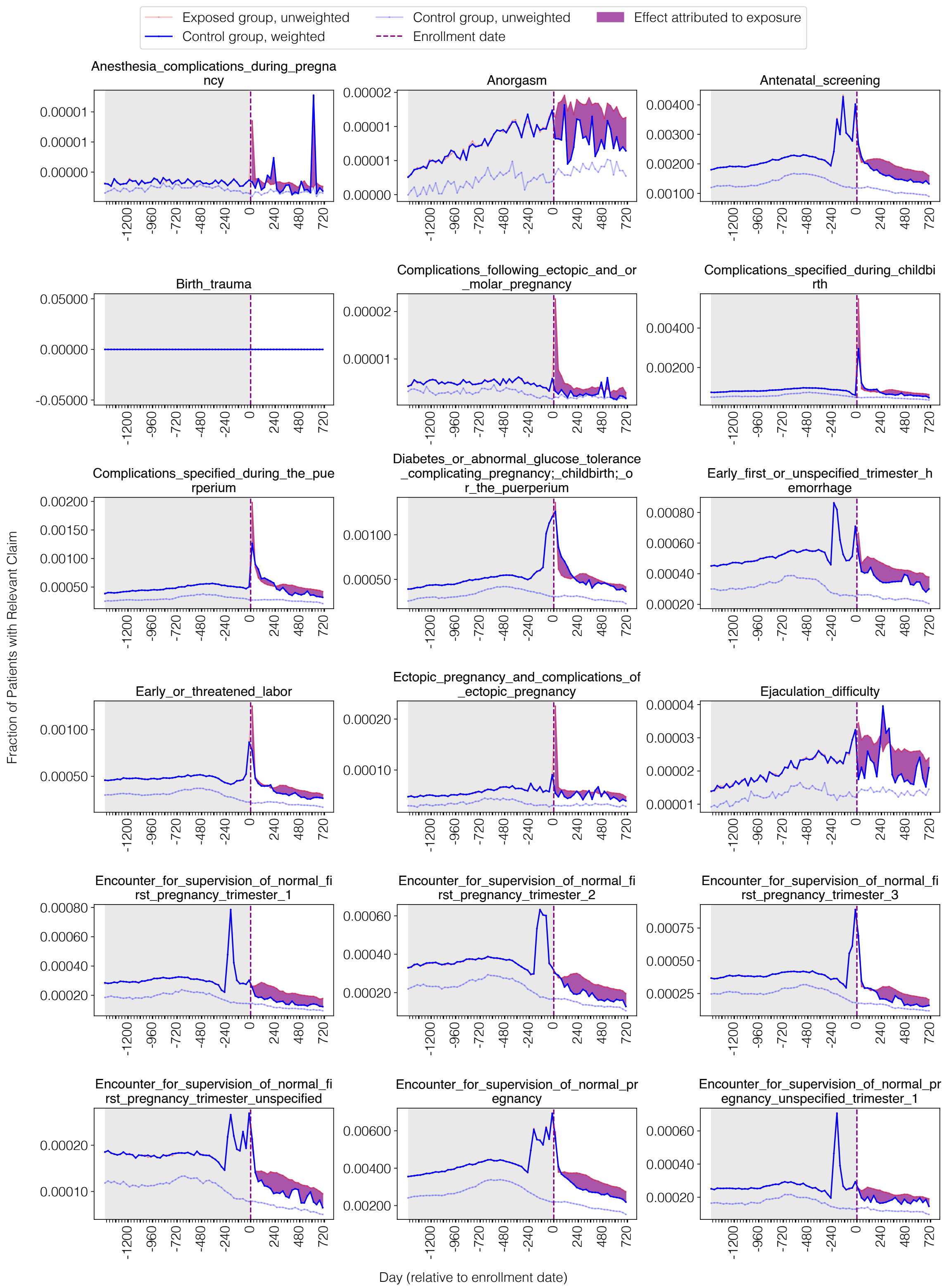


**Fig. S127.** Temporal trends in Reproductive health prevalence. The fraction of patients with relevant claims over time belonging for each outcome in the **unrestricted control design with placebo cases** (negative COVID-19 PCR test) experiment.

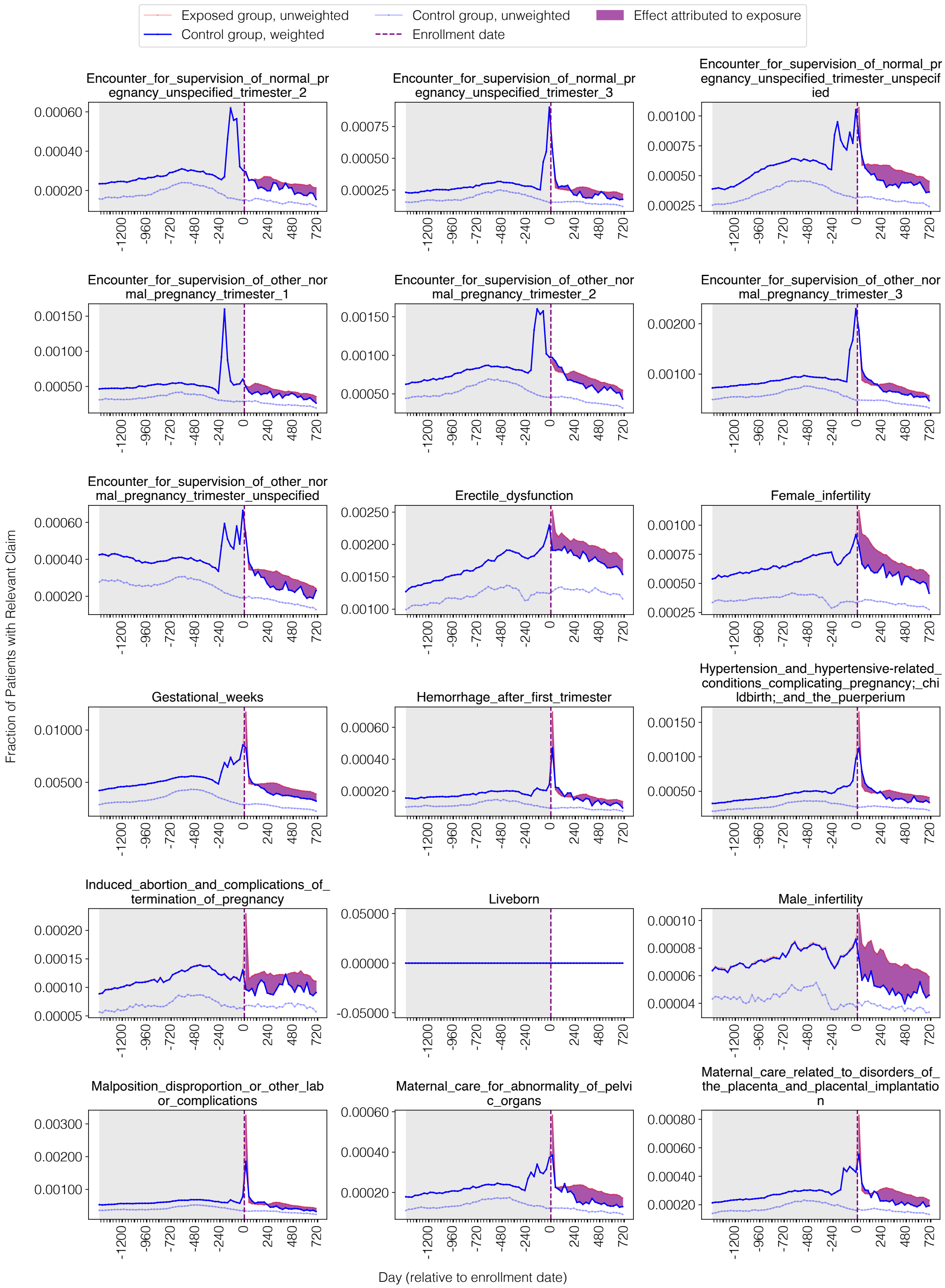


**Fig. S127.** (cont'd) Temporal trends in Reproductive health prevalence. The fraction of patients with relevant claims over time belonging for each outcome in the **unrestricted control design with placebo cases** (negative COVID-19 PCR test) experiment.

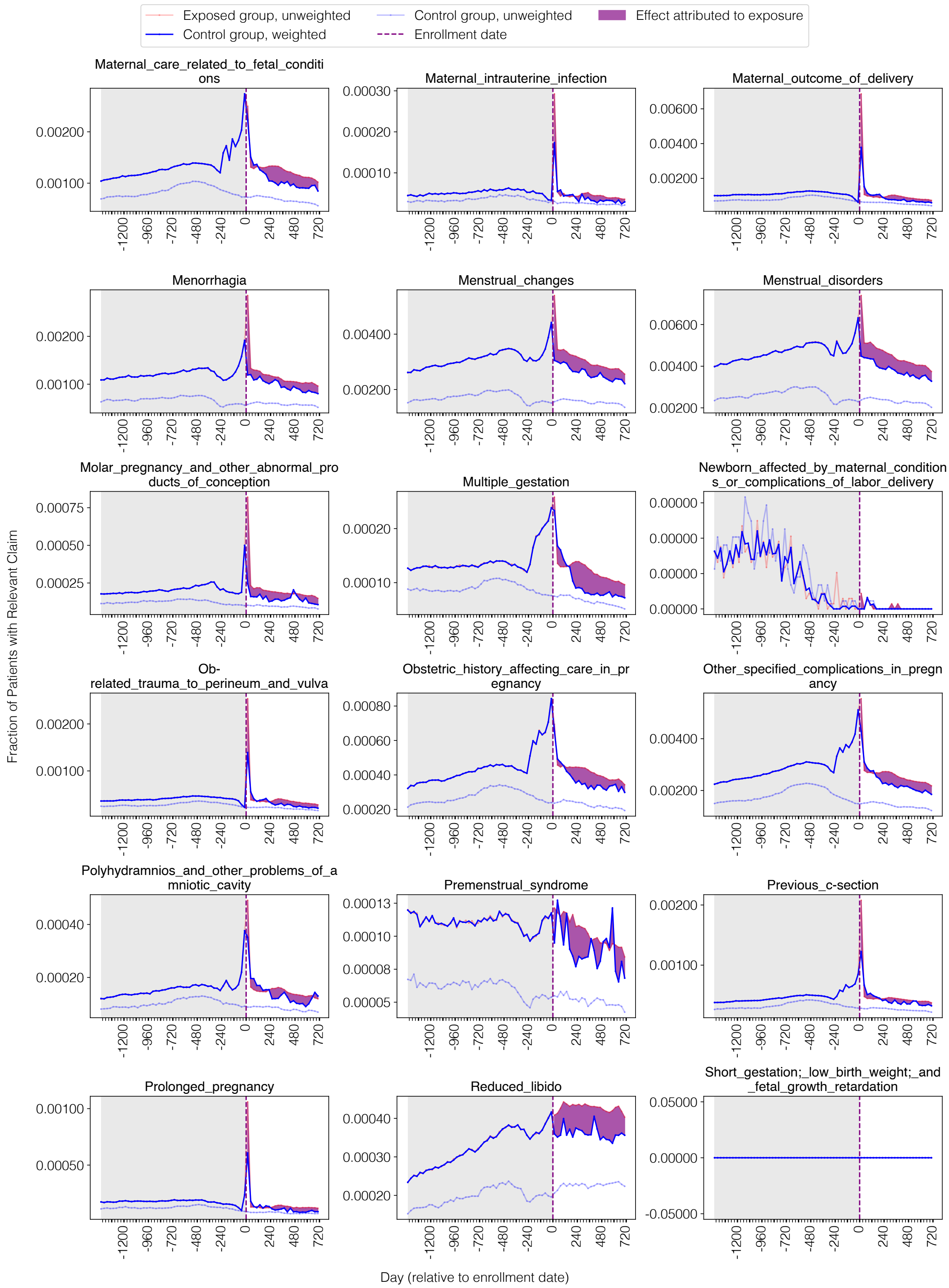


**Fig. S127.** (cont'd) Temporal trends in Reproductive health prevalence. The fraction of patients with relevant claims over time belonging for each outcome in the **unrestricted control design with placebo cases** (negative COVID-19 PCR test) experiment.

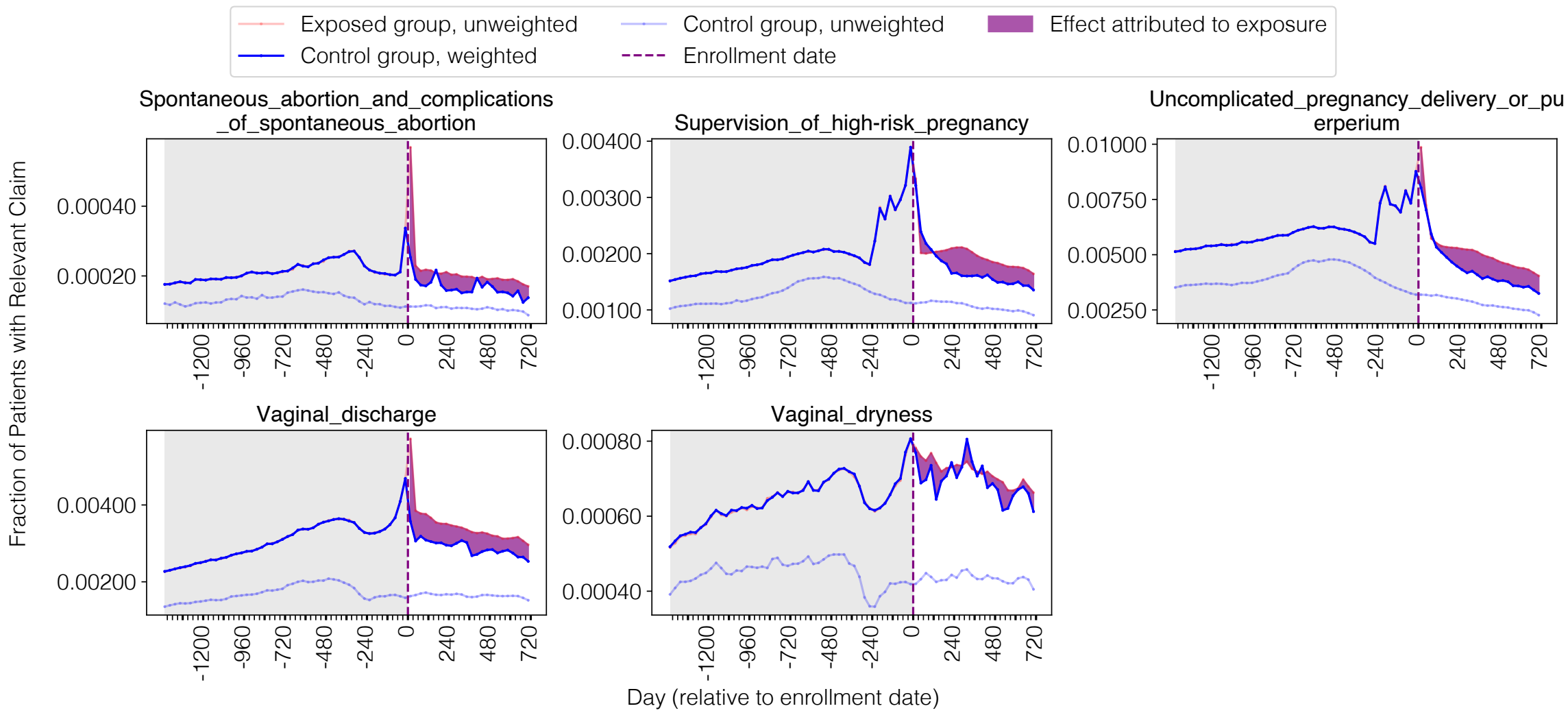


**Fig. S127.** (cont'd) Temporal trends in Reproductive health prevalence. The fraction of patients with relevant claims over time belonging for each outcome in the **unrestricted control design with placebo cases** (negative COVID-19 PCR test) experiment.

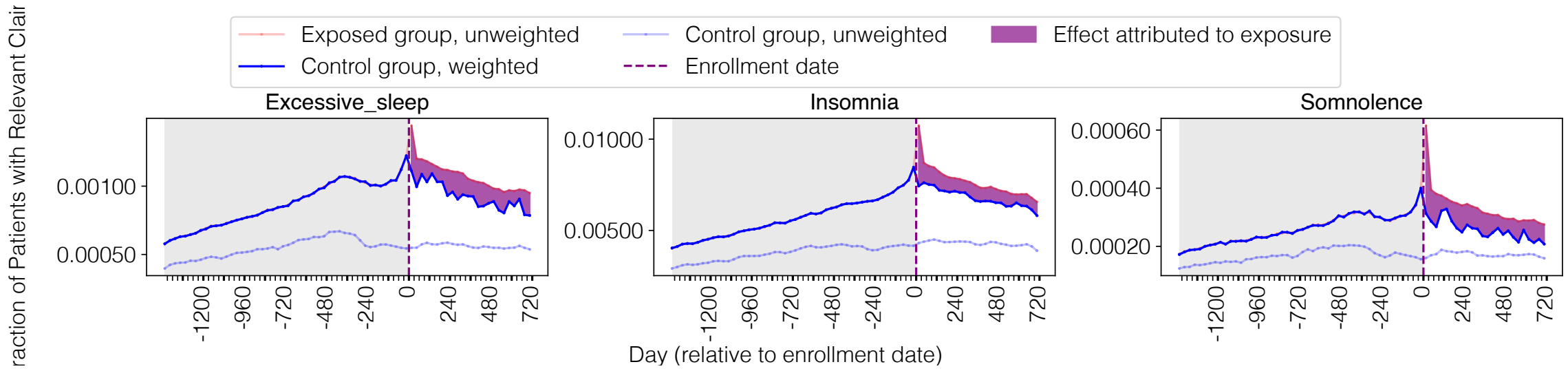


**Fig. S128.** Temporal trends in Sleep prevalence. The fraction of patients with relevant claims over time belonging for each outcome in the **unrestricted control design with placebo cases** (negative COVID-19 PCR test) experiment.

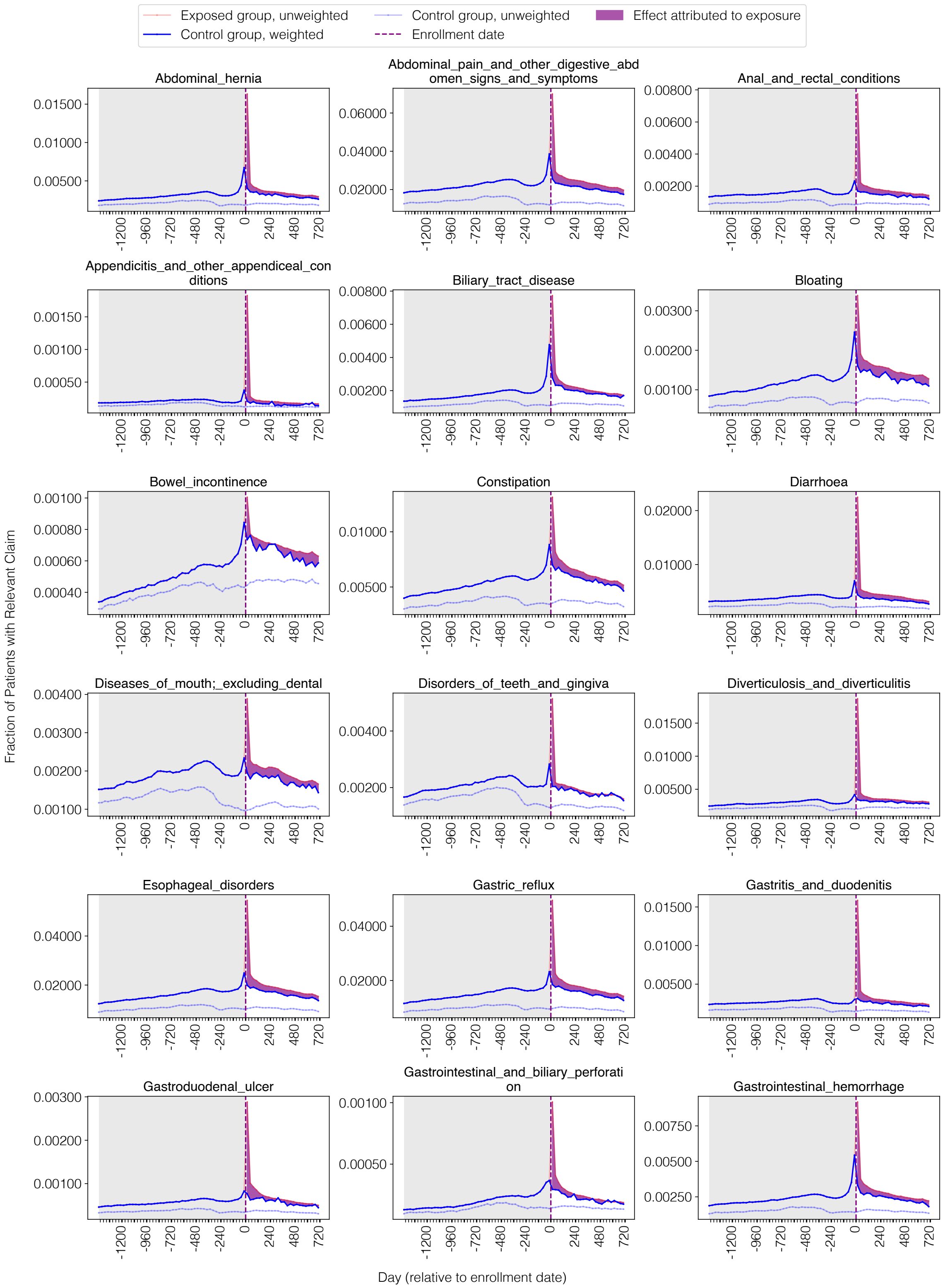


**Fig. S129.** Temporal trends in Stomach and digestion prevalence. The fraction of patients with relevant claims over time belonging for each outcome in the **unrestricted control design with placebo cases** (negative COVID-19 PCR test) experiment.

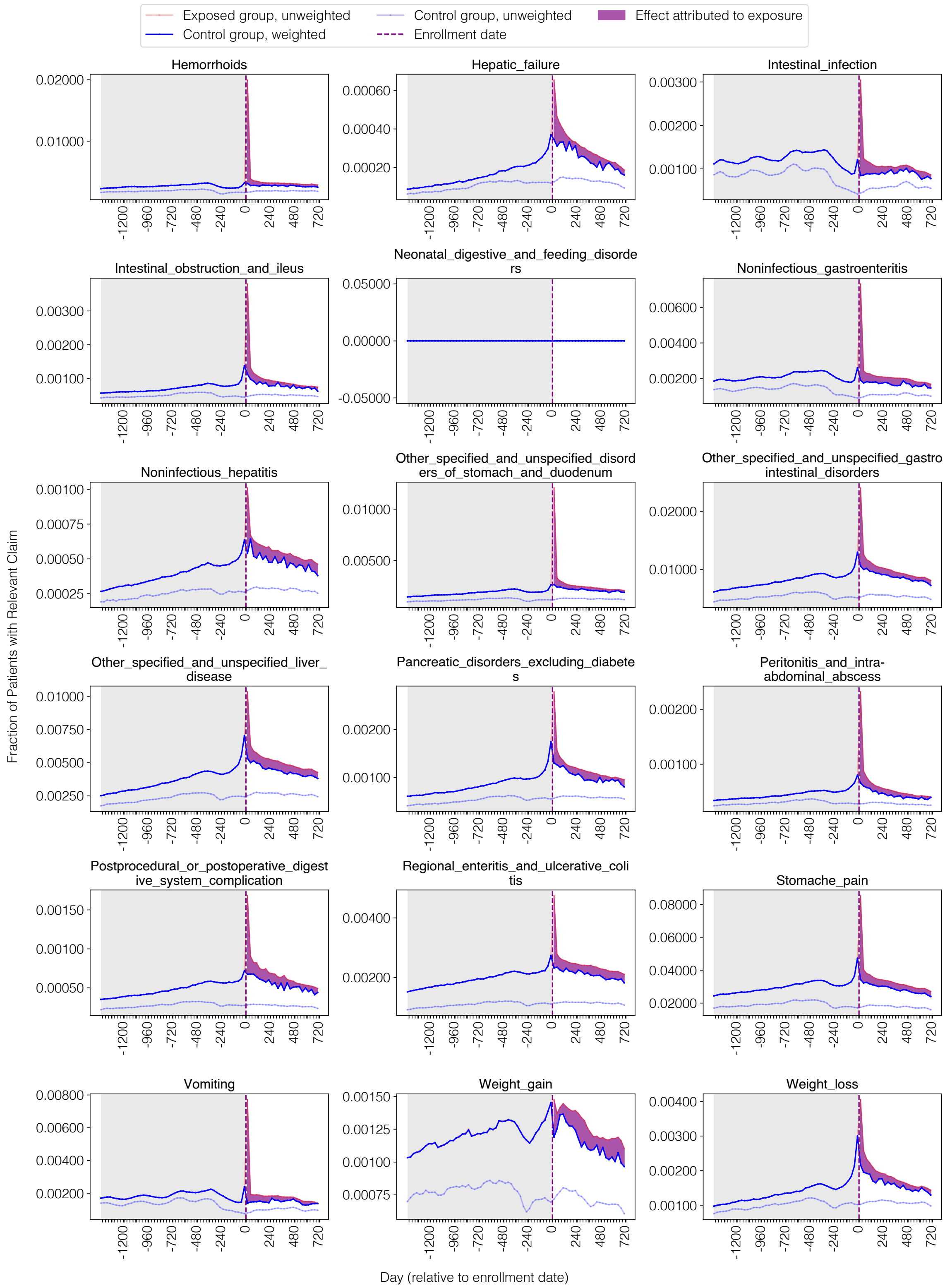


**Fig. S129.** (cont'd) Temporal trends in Stomach and digestion prevalence. The fraction of patients with relevant claims over time belonging for each outcome in the **unrestricted control design with placebo cases** (negative COVID-19 PCR test) experiment.

 

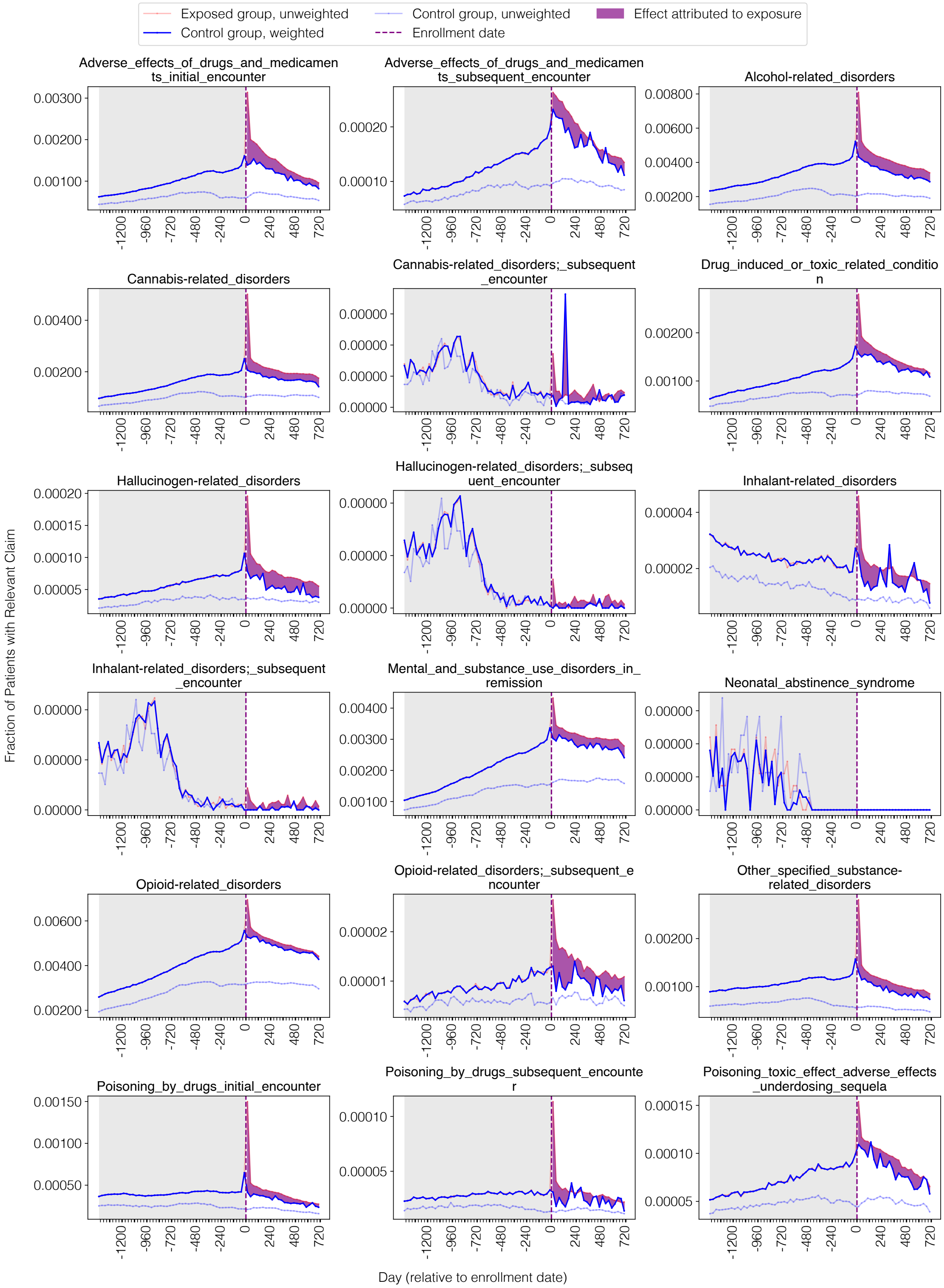


**Fig. S130.** Temporal trends in Substance use prevalence. The fraction of patients with relevant claims over time belonging for each outcome in the **unrestricted control design with placebo cases** (negative COVID-19 PCR test) experiment.

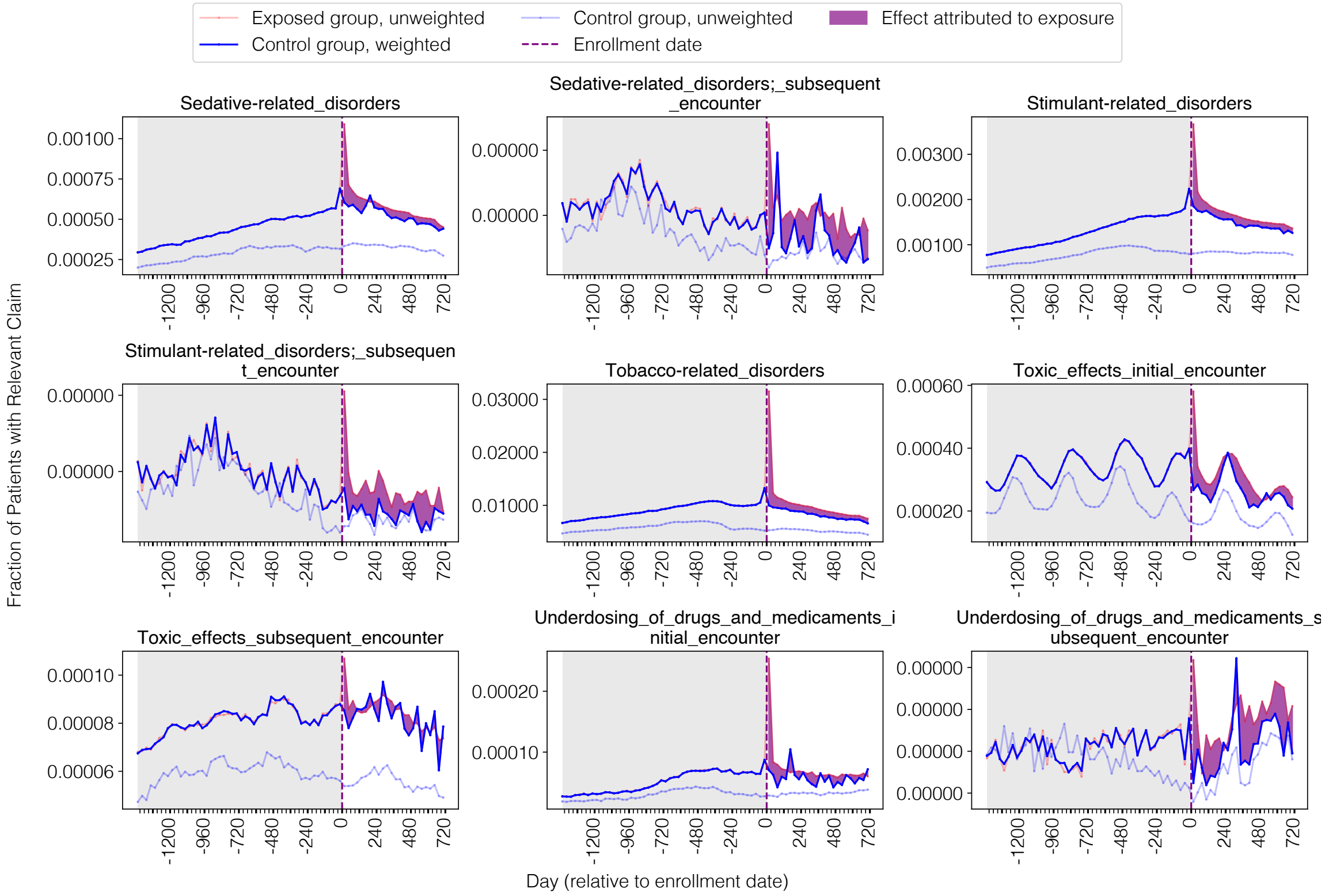


**Fig. S130.** (cont'd) Temporal trends in Substance use prevalence. The fraction of patients with relevant claims over time belonging for each outcome in the **unrestricted control design with placebo cases** (negative COVID-19 PCR test) experiment.

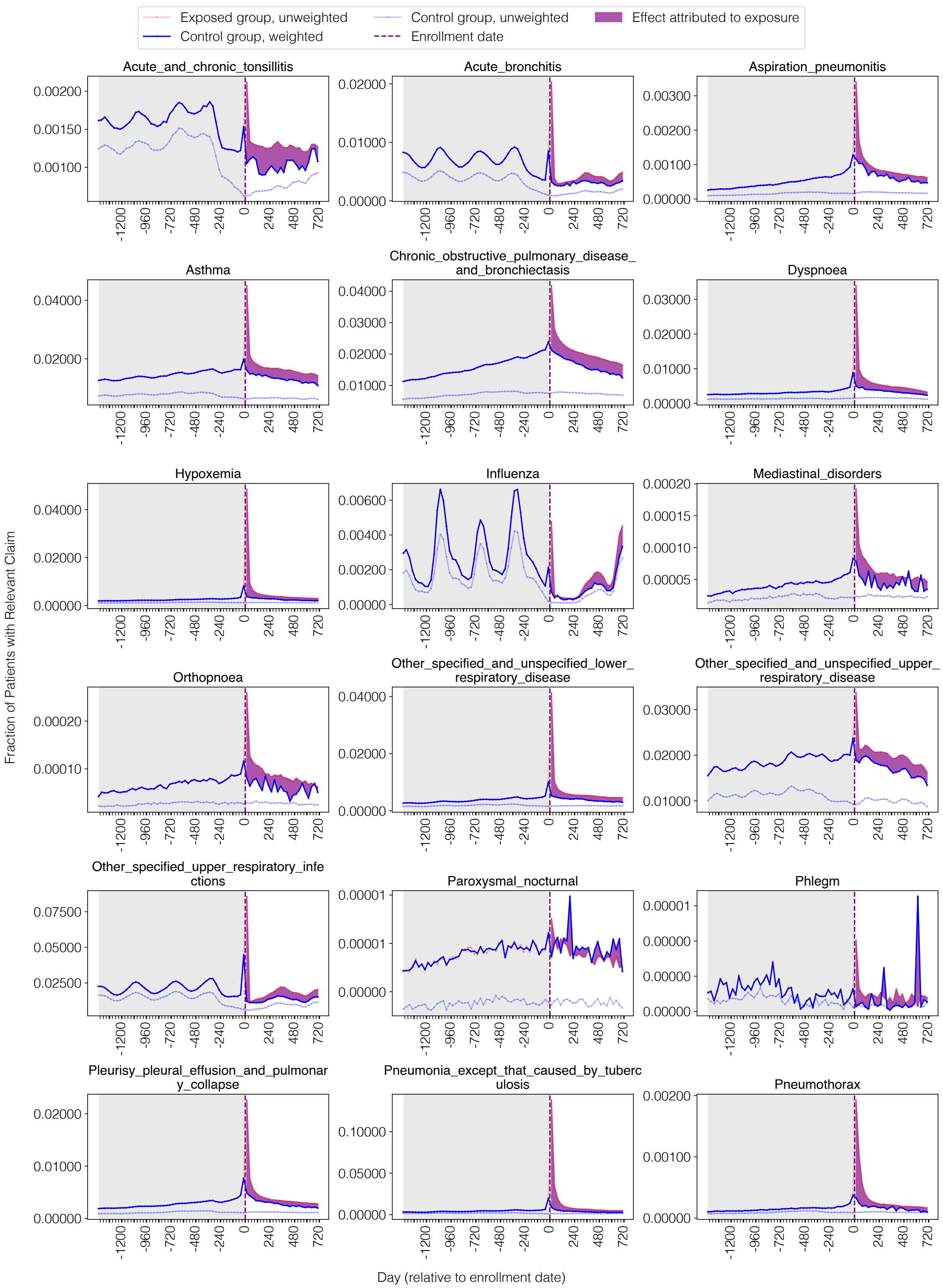


**Fig. S131.** Temporal trends in Breathing prevalence. The fraction of patients with relevant claims over time belonging for each outcome in the **unrestricted control design** experiment.

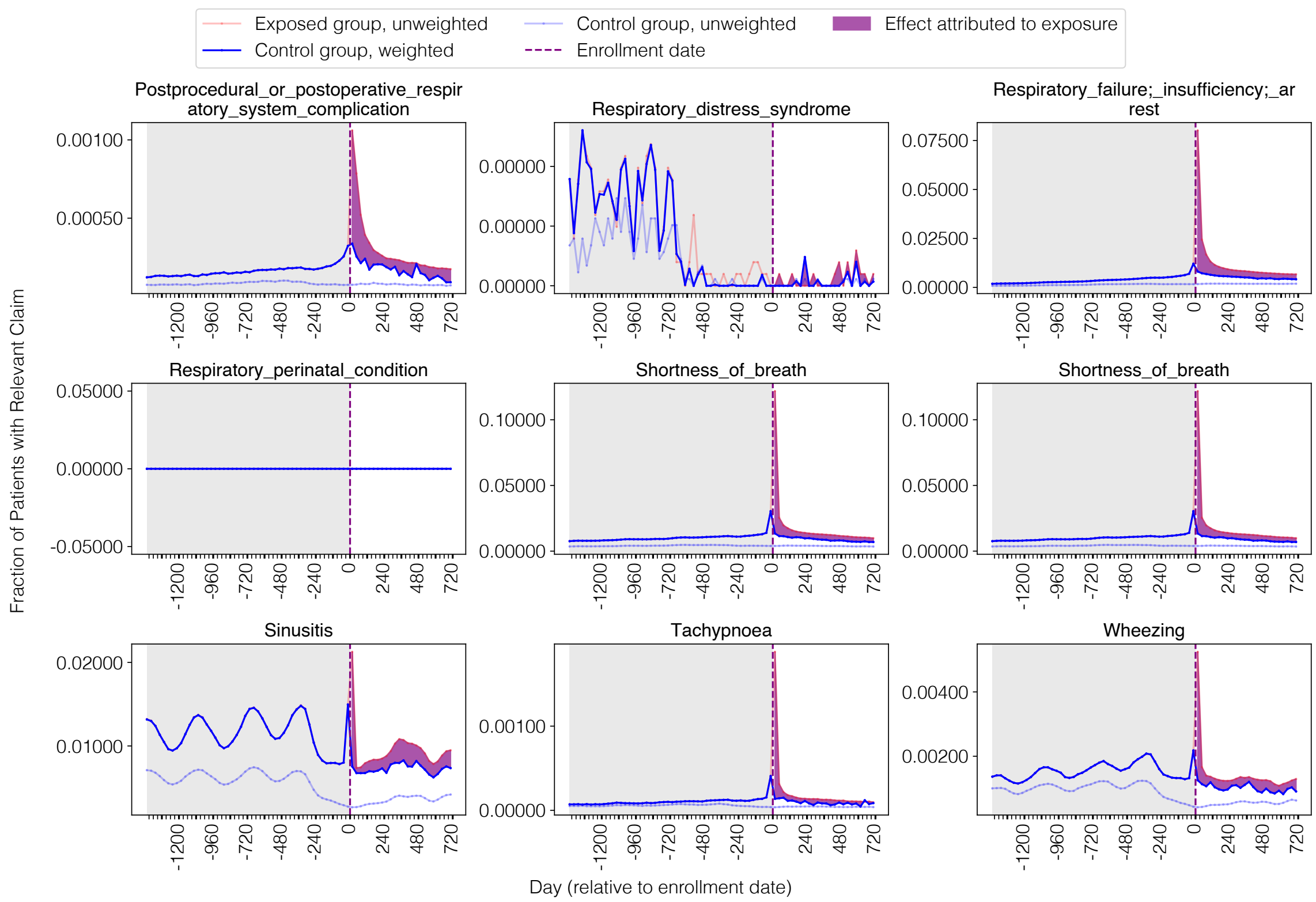


**Fig. S131.** (cont'd) Temporal trends in Breathing prevalence. The fraction of patients with relevant claims over time belonging for each outcome in the **unrestricted control design** experiment.

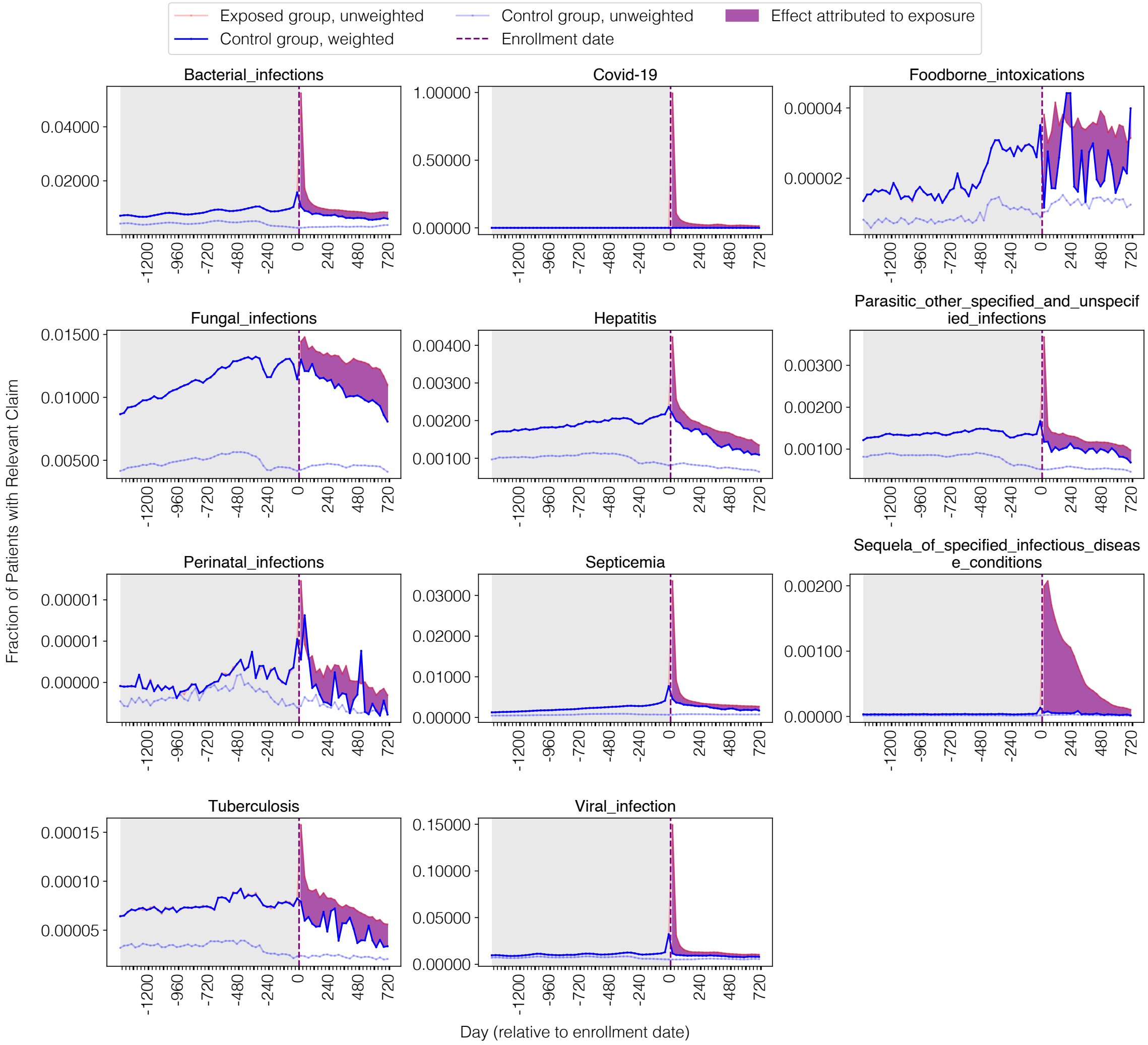


**Fig. S132.** Temporal trends in Certain Infectious and Parasitic Diseases prevalence. The fraction of patients with relevant claims over time belonging for each outcome in the **unrestricted control design** experiment.

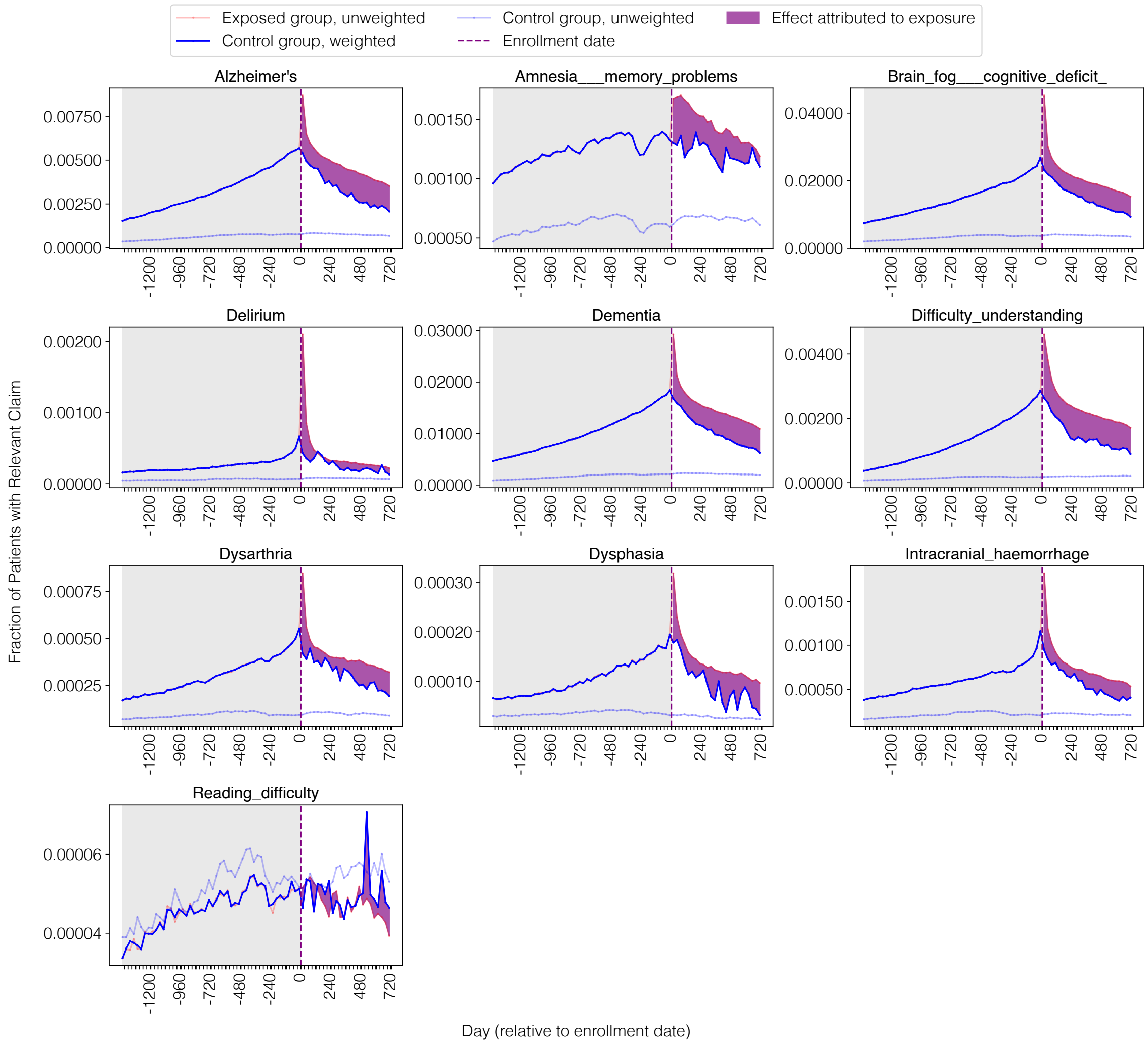


**Fig. S133.** Temporal trends in Cognitive health prevalence. The fraction of patients with relevant claims over time belonging for each outcome in the **unrestricted control design** experiment.

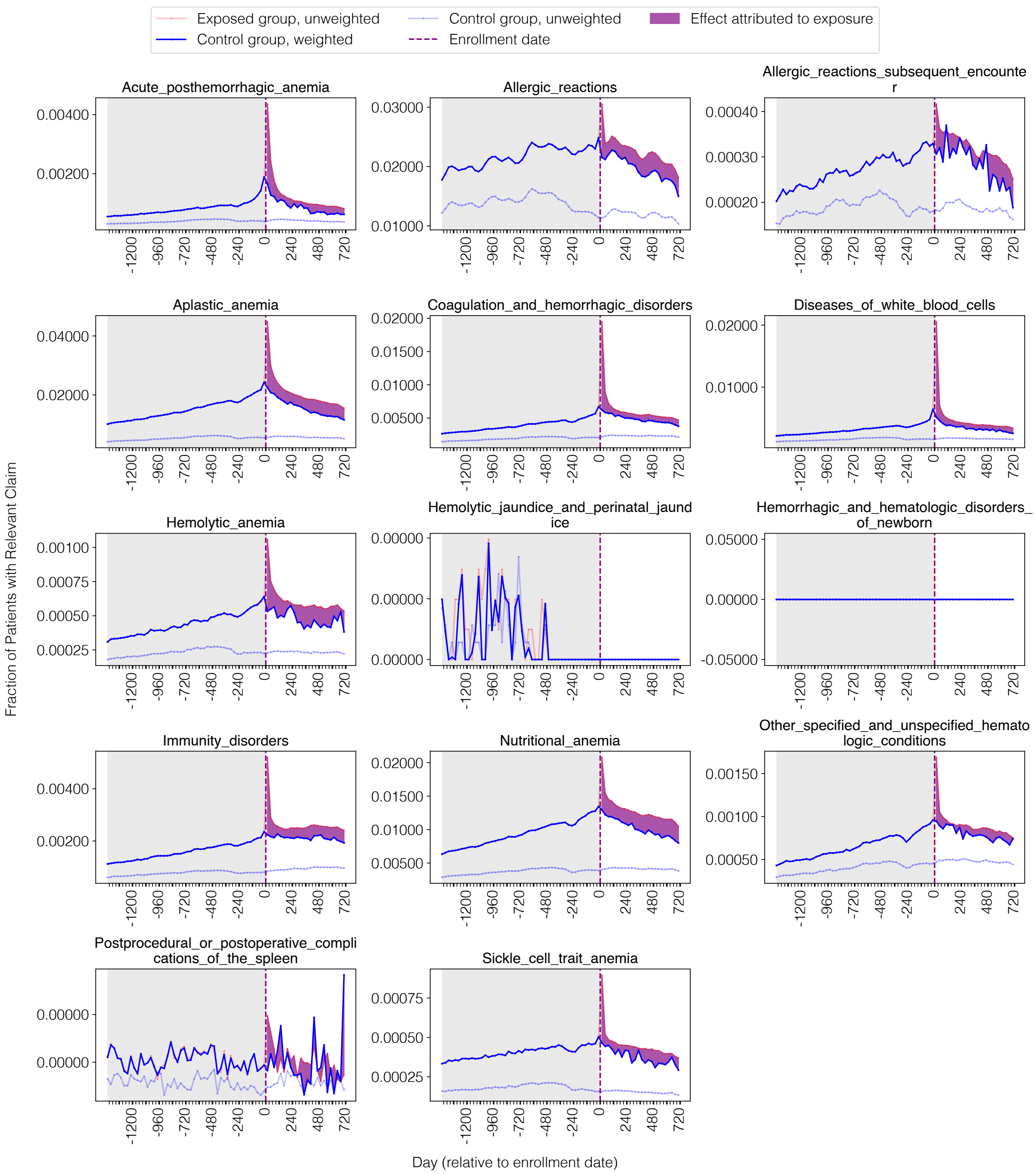


**Fig. S134.** Temporal trends in Diseases of the Blood and Blood Forming Organs and Certain Disorders Involving the Immune Mechanism prevalence. The fraction of patients with relevant claims over time belonging for each outcome in the **unrestricted control design** experiment.

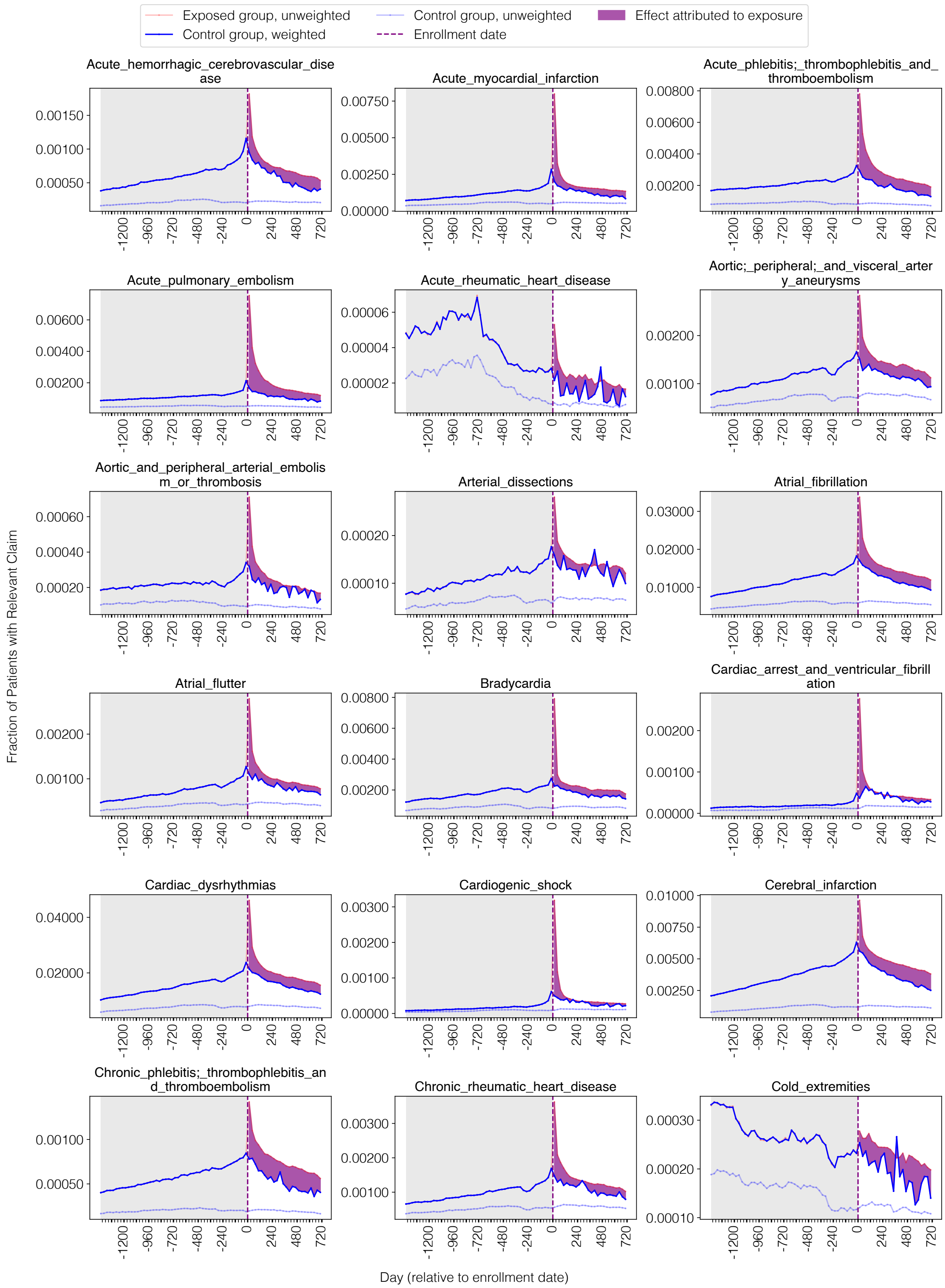


**Fig. S135.** Temporal trends in Diseases of the Circulatory System prevalence. The fraction of patients with relevant claims over time belonging for each outcome in the **unrestricted control design** experiment.

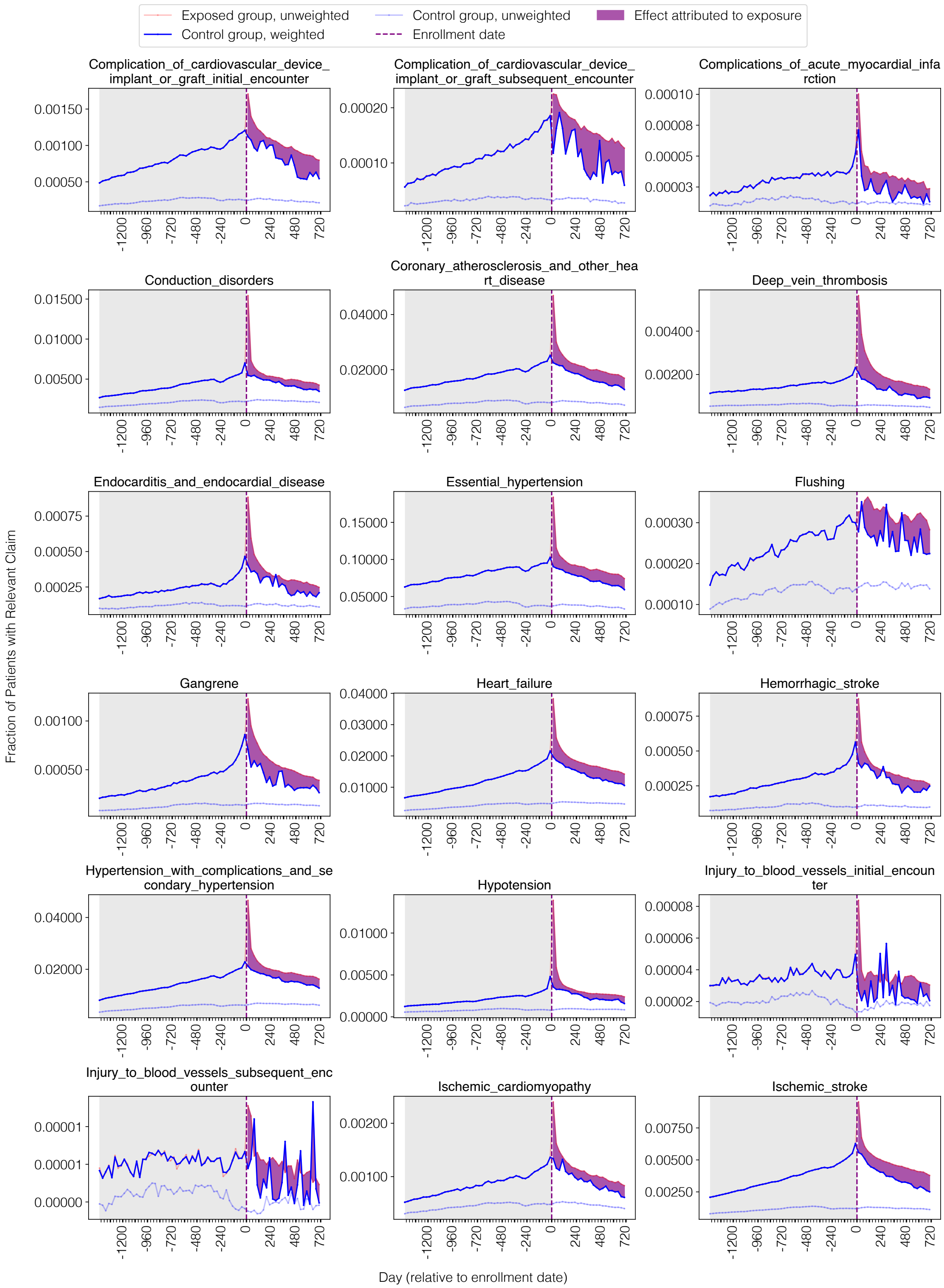


**Fig. S135.** (cont'd) Temporal trends in Diseases of the Circulatory System prevalence. The fraction of patients with relevant claims over time belonging for each outcome in the **unrestricted control design** experiment.

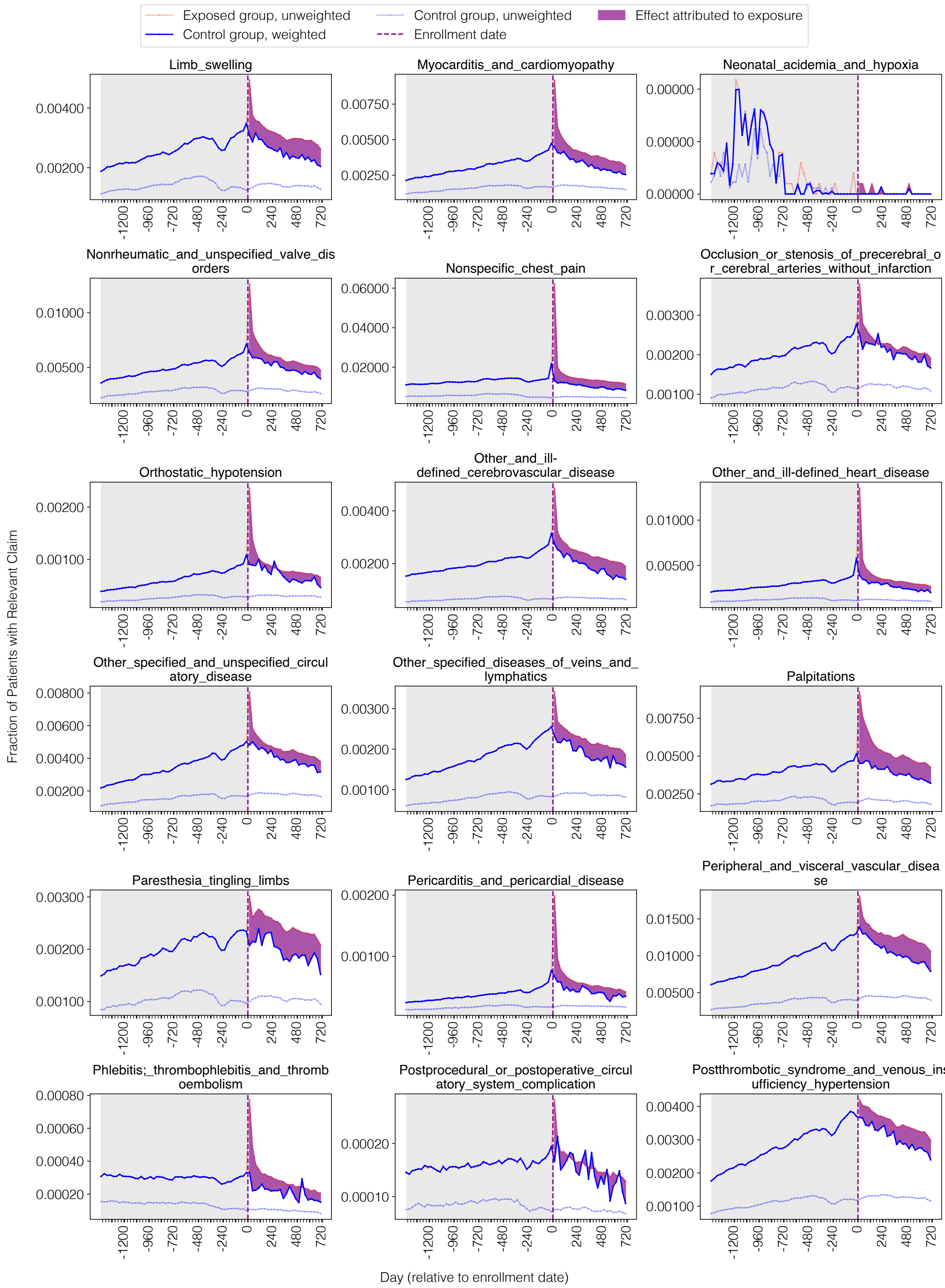


**Fig. S135.** (cont'd) Temporal trends in Diseases of the Circulatory System prevalence. The fraction of patients with relevant claims over time belonging for each outcome in the **unrestricted control design** experiment.

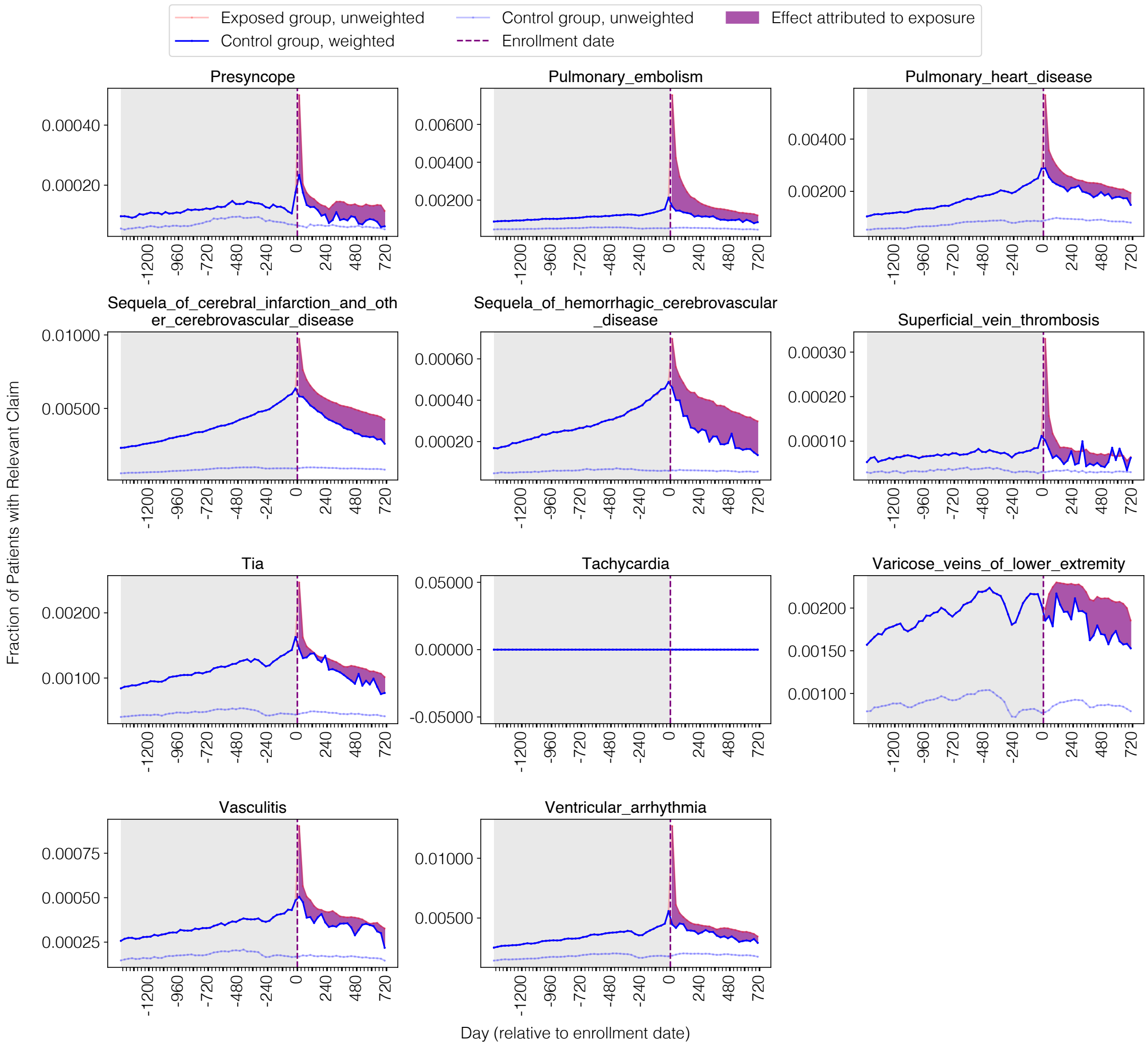


**Fig. S135.** (cont'd) Temporal trends in Diseases of the Circulatory System prevalence. The fraction of patients with relevant claims over time belonging for each outcome in the **unrestricted control design** experiment.

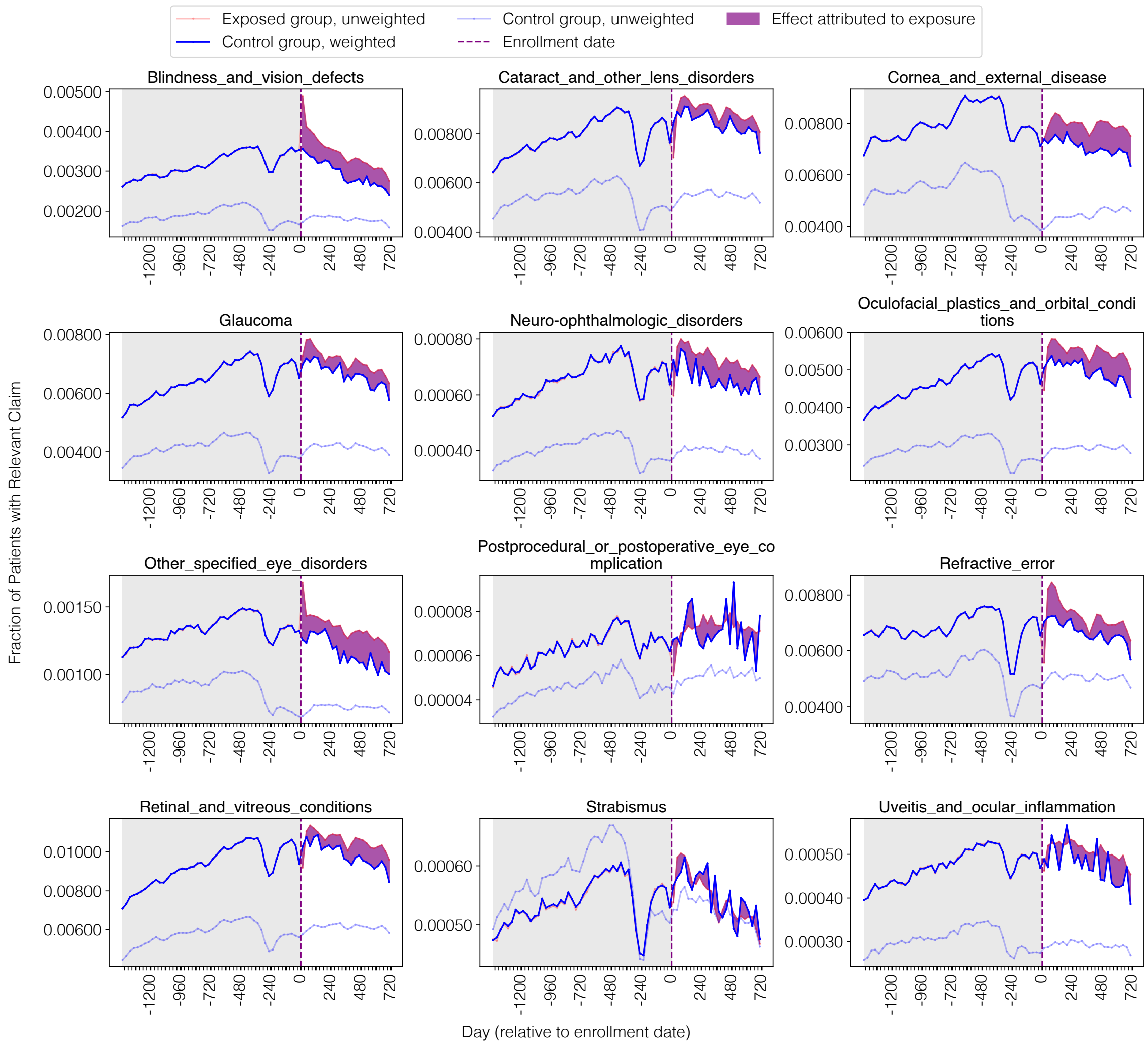


**Fig. S136.** Temporal trends in Diseases of the Eye and Adnexa prevalence. The fraction of patients with relevant claims over time belonging for each outcome in the **unrestricted control design** experiment.

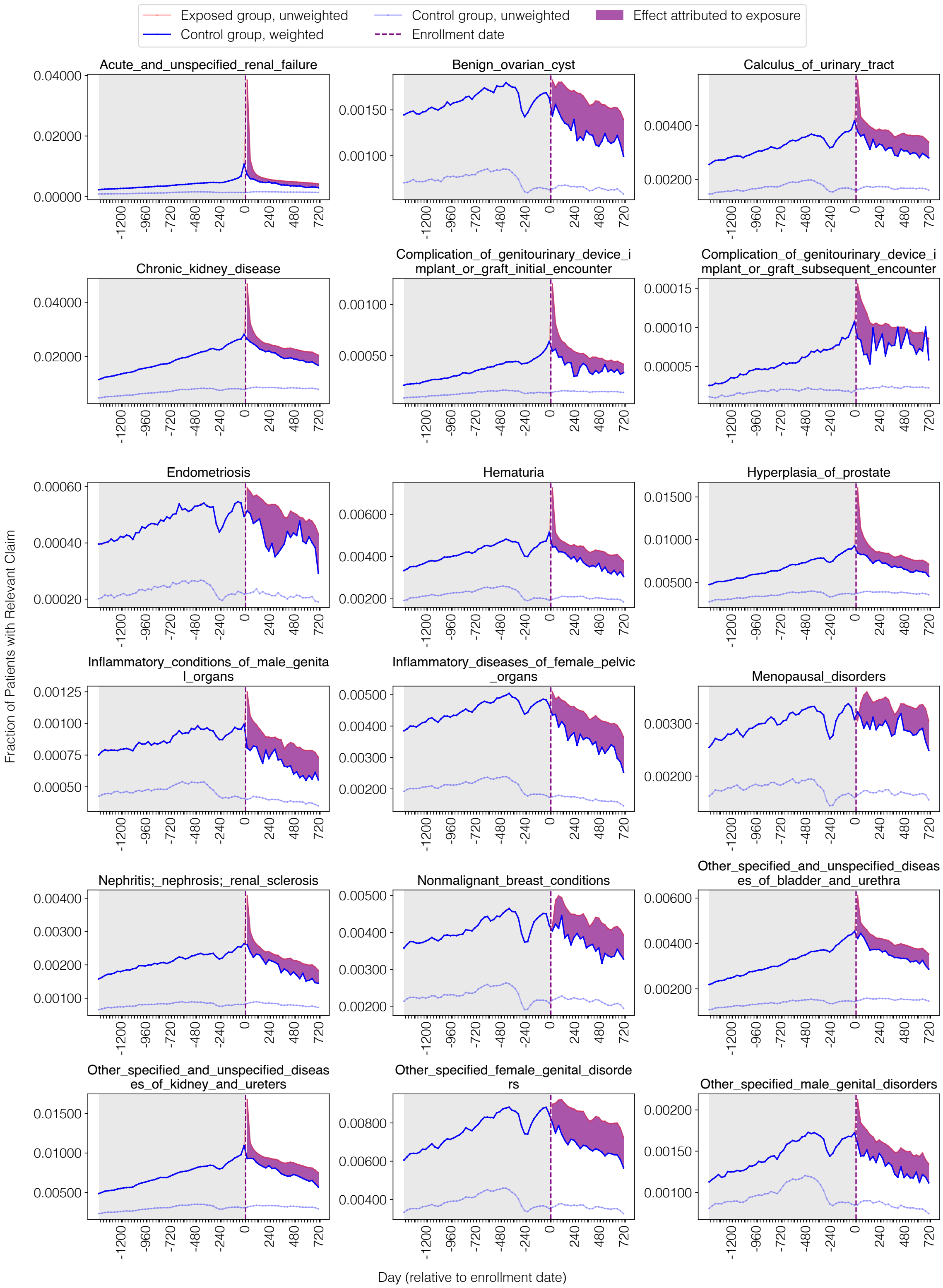


**Fig. S137.** Temporal trends in Diseases of the Genitourinary System prevalence. The fraction of patients with relevant claims over time belonging for each outcome in the **unrestricted control design** experiment.

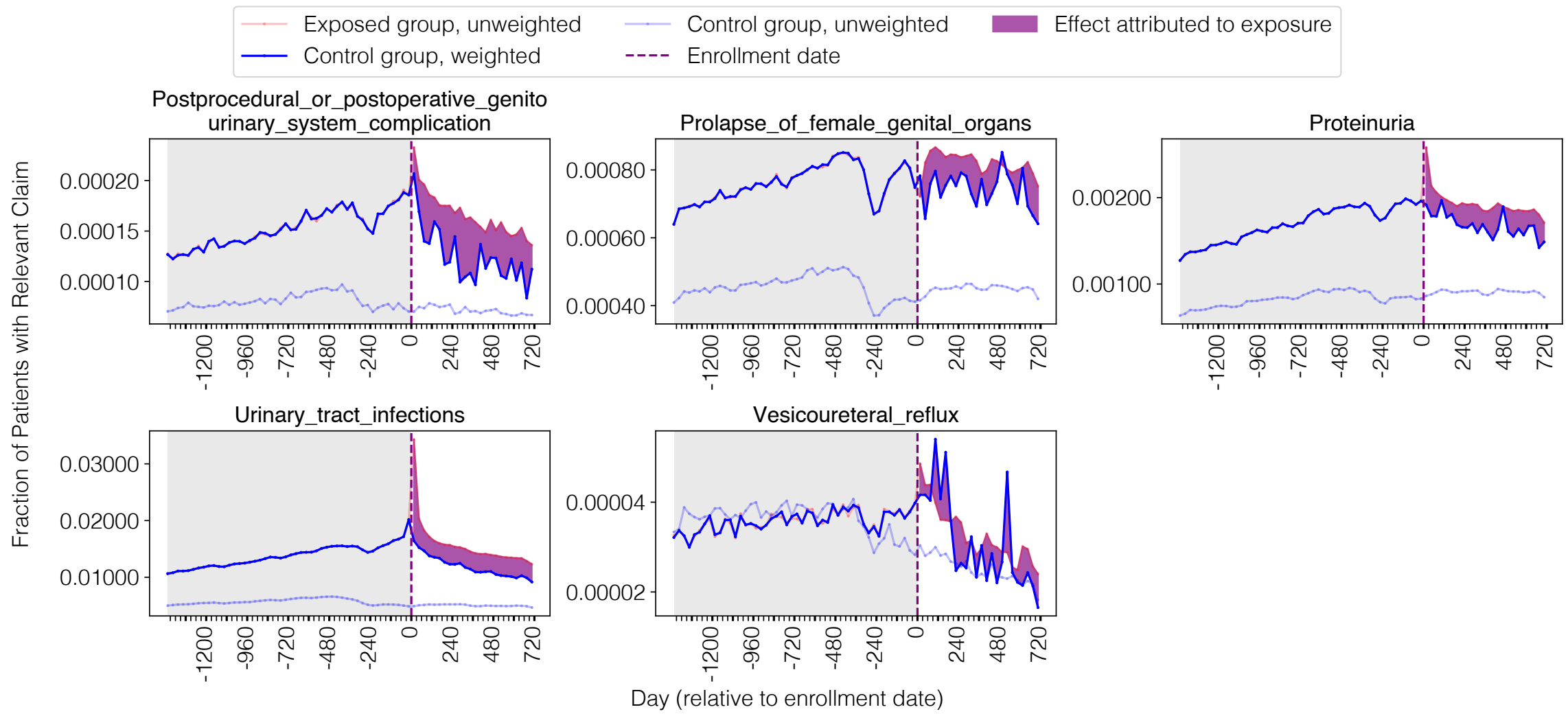


**Fig. S137.** (cont'd) Temporal trends in Diseases of the Genitourinary System prevalence. The fraction of patients with relevant claims over time belonging for each outcome in the **unrestricted control design** experiment.

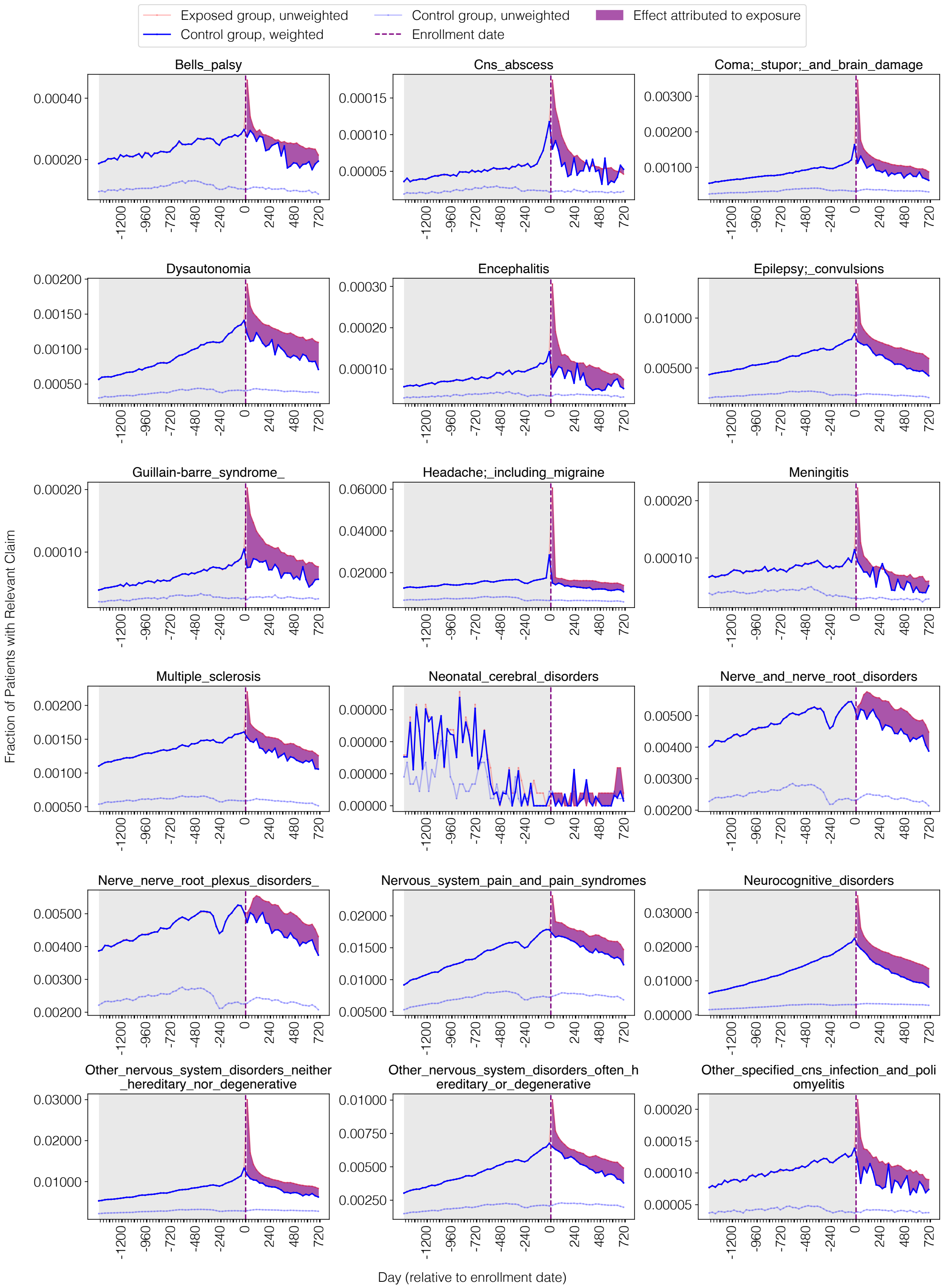


**Fig. S138.** Temporal trends in Diseases of the Nervous System prevalence. The fraction of patients with relevant claims over time belonging for each outcome in the **unrestricted control design** experiment.

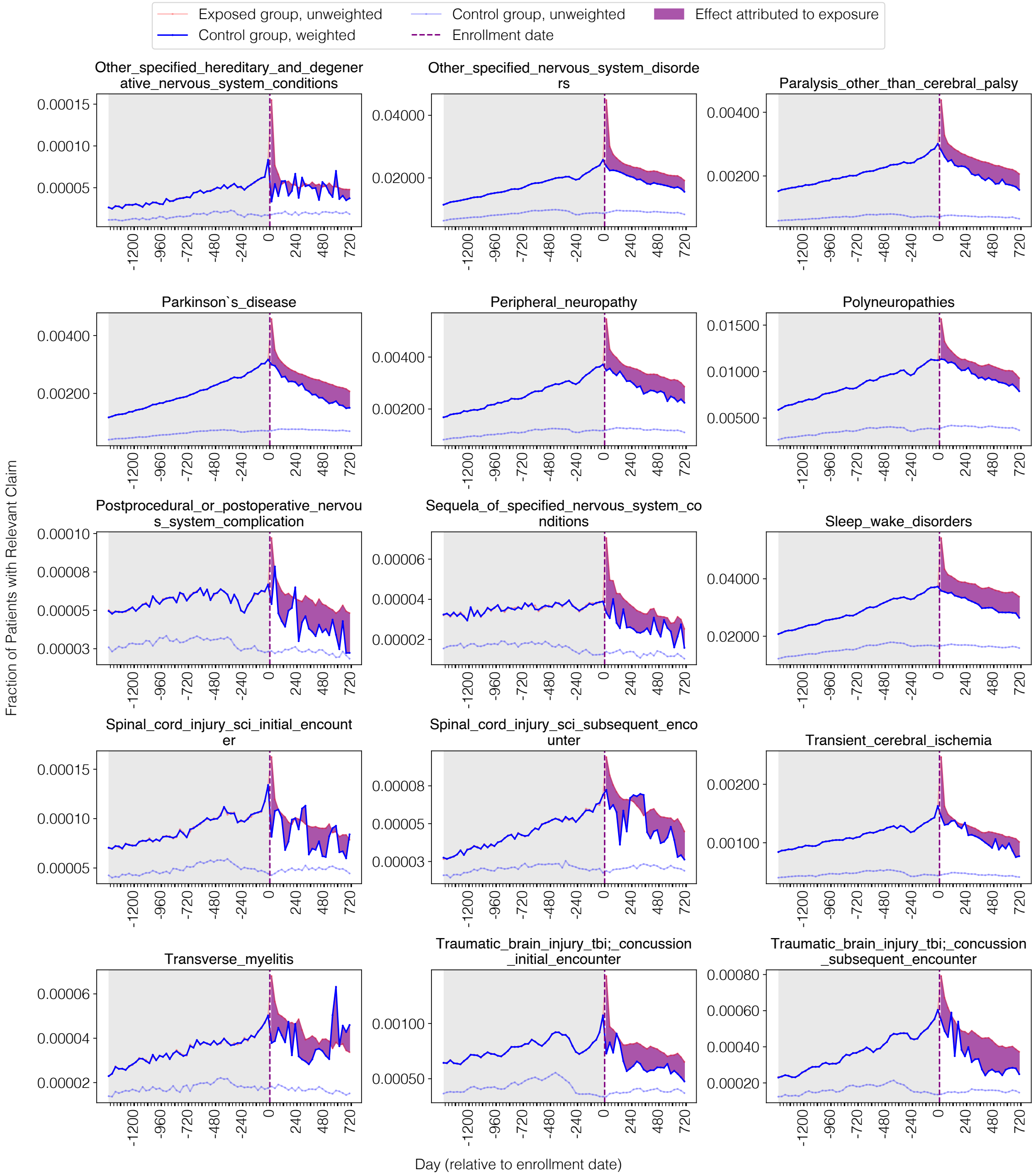


**Fig. S138.** (cont'd) Temporal trends in Diseases of the Nervous System prevalence. The fraction of patients with relevant claims over time belonging for each outcome in the **unrestricted control design** experiment.

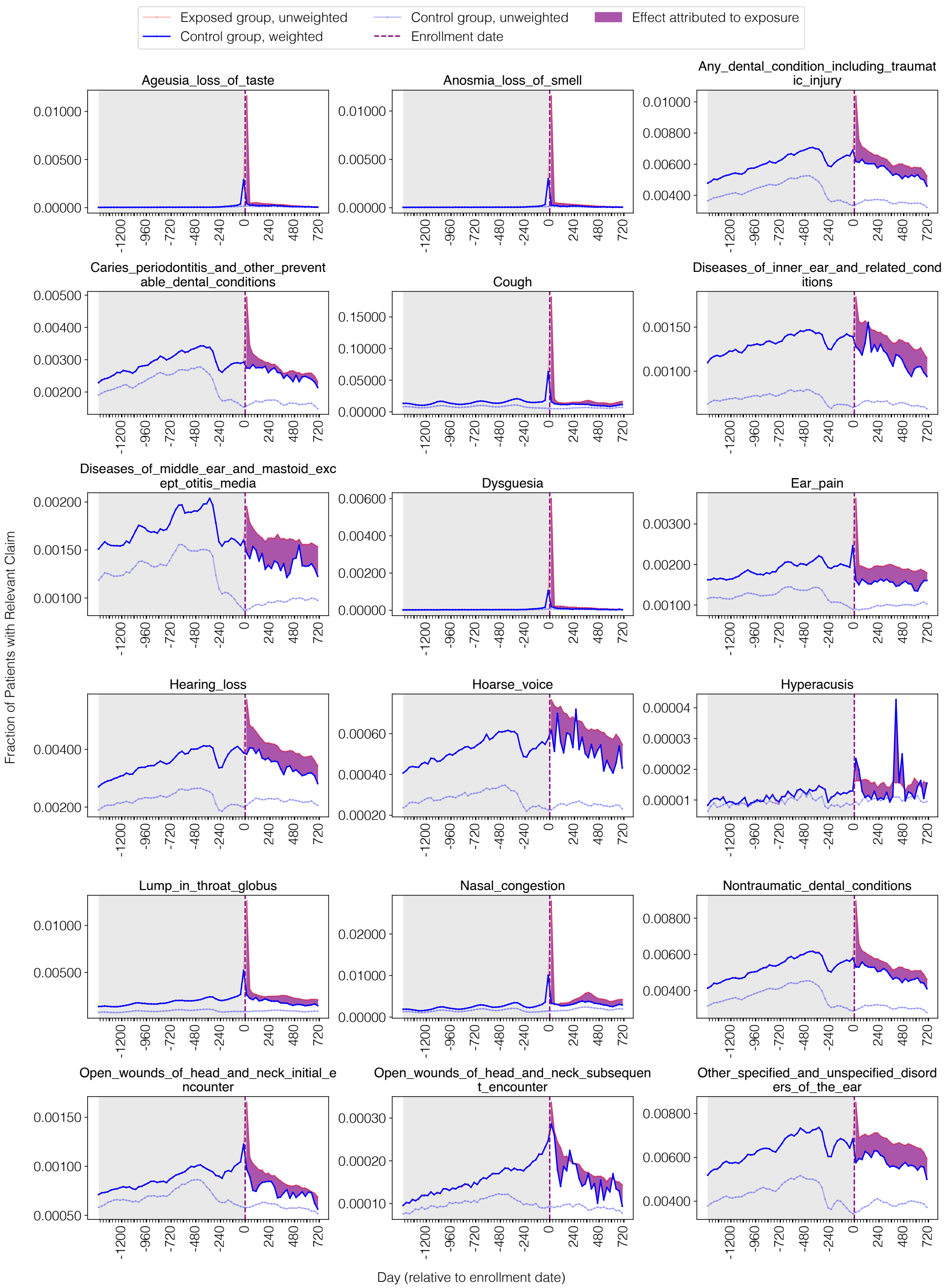


**Fig. S139.** Temporal trends in Ear, nose and throat prevalence. The fraction of patients with relevant claims over time belonging for each outcome in the **unrestricted control design** experiment.

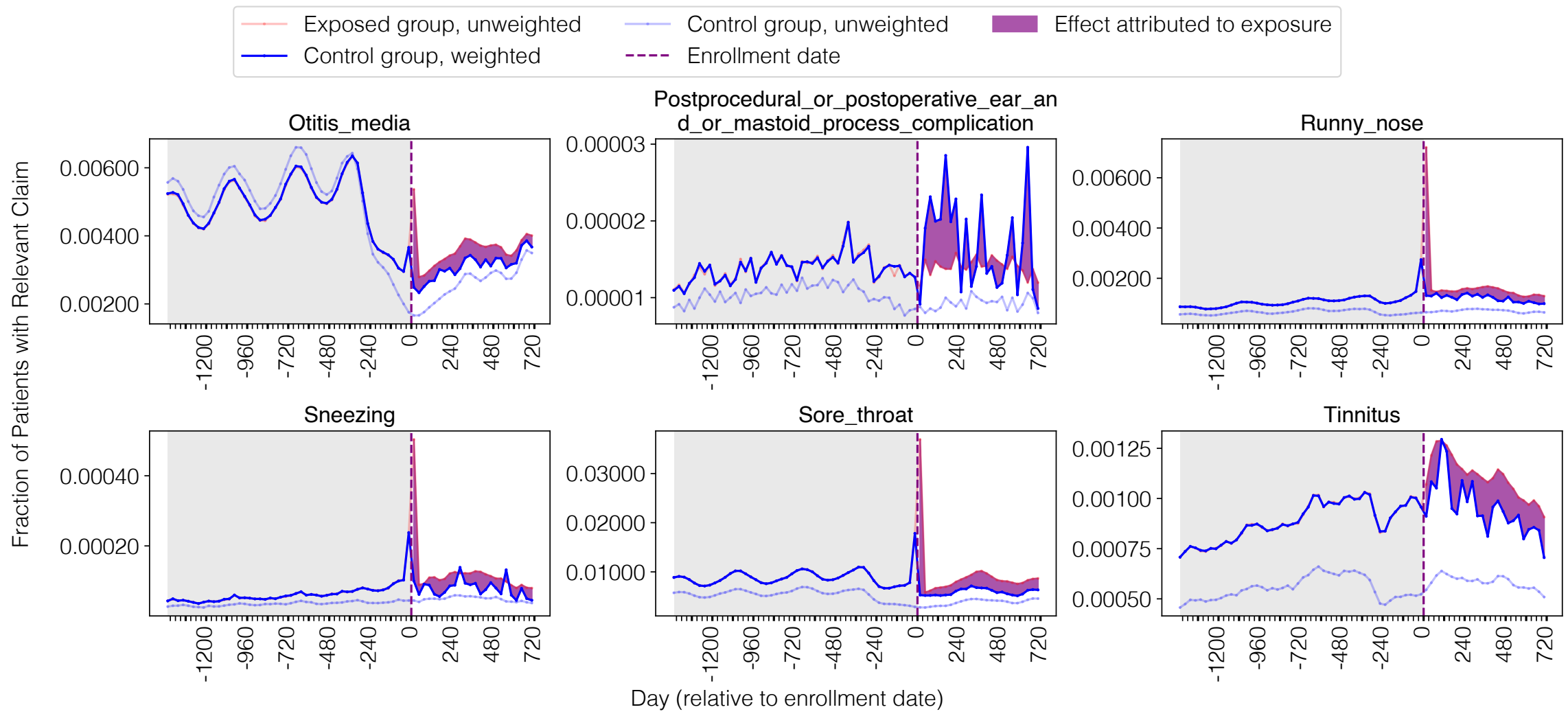


**Fig. S139.** (cont'd) Temporal trends in Ear, nose and throat prevalence. The fraction of patients with relevant claims over time belonging for each outcome in the **unrestricted control design** experiment.

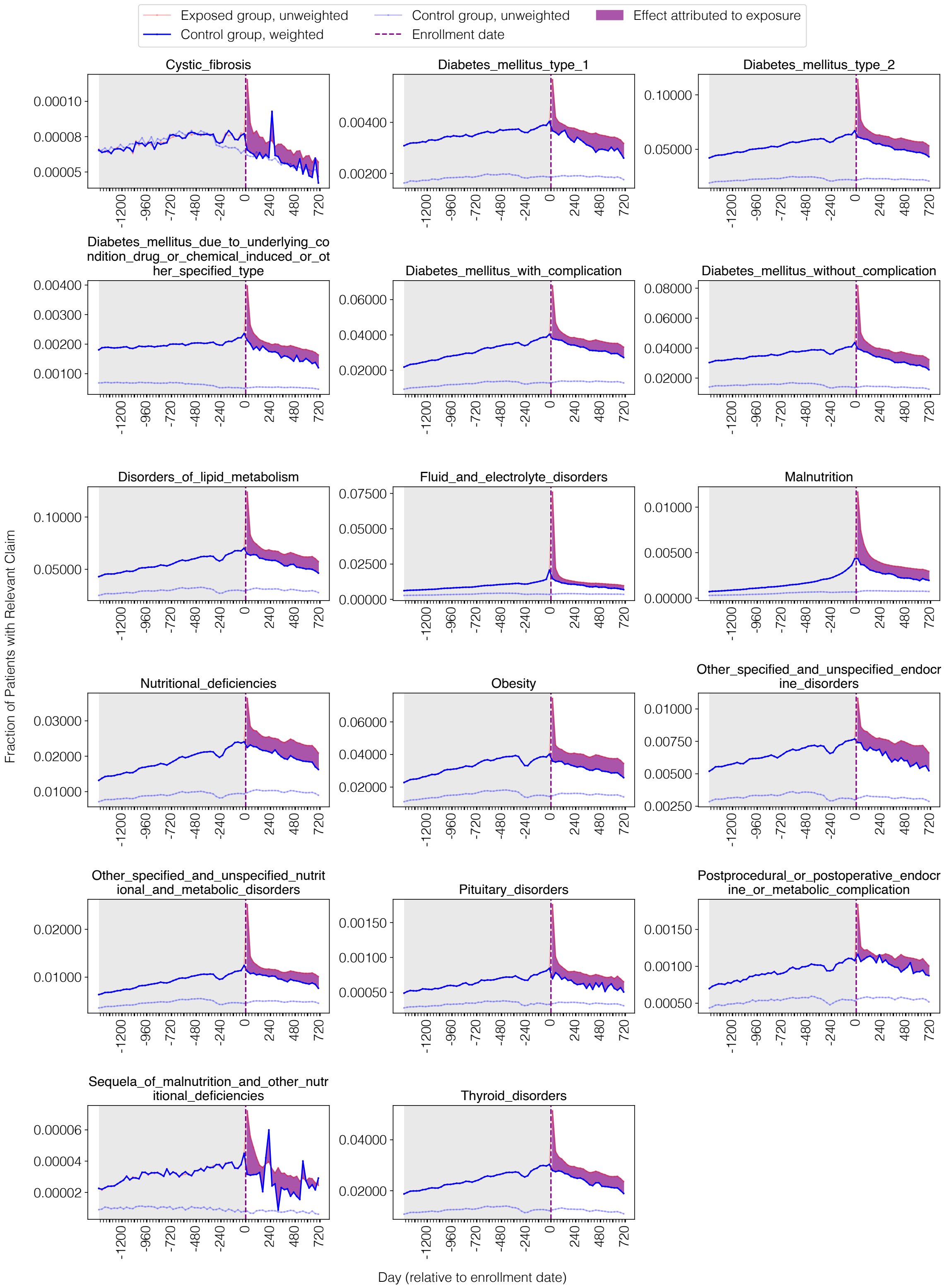


**Fig. S140.** Temporal trends in Endocrine, Nutritional and Metabolic Diseases prevalence. The fraction of patients with relevant claims over time belonging for each outcome in the **unrestricted control design** experiment.

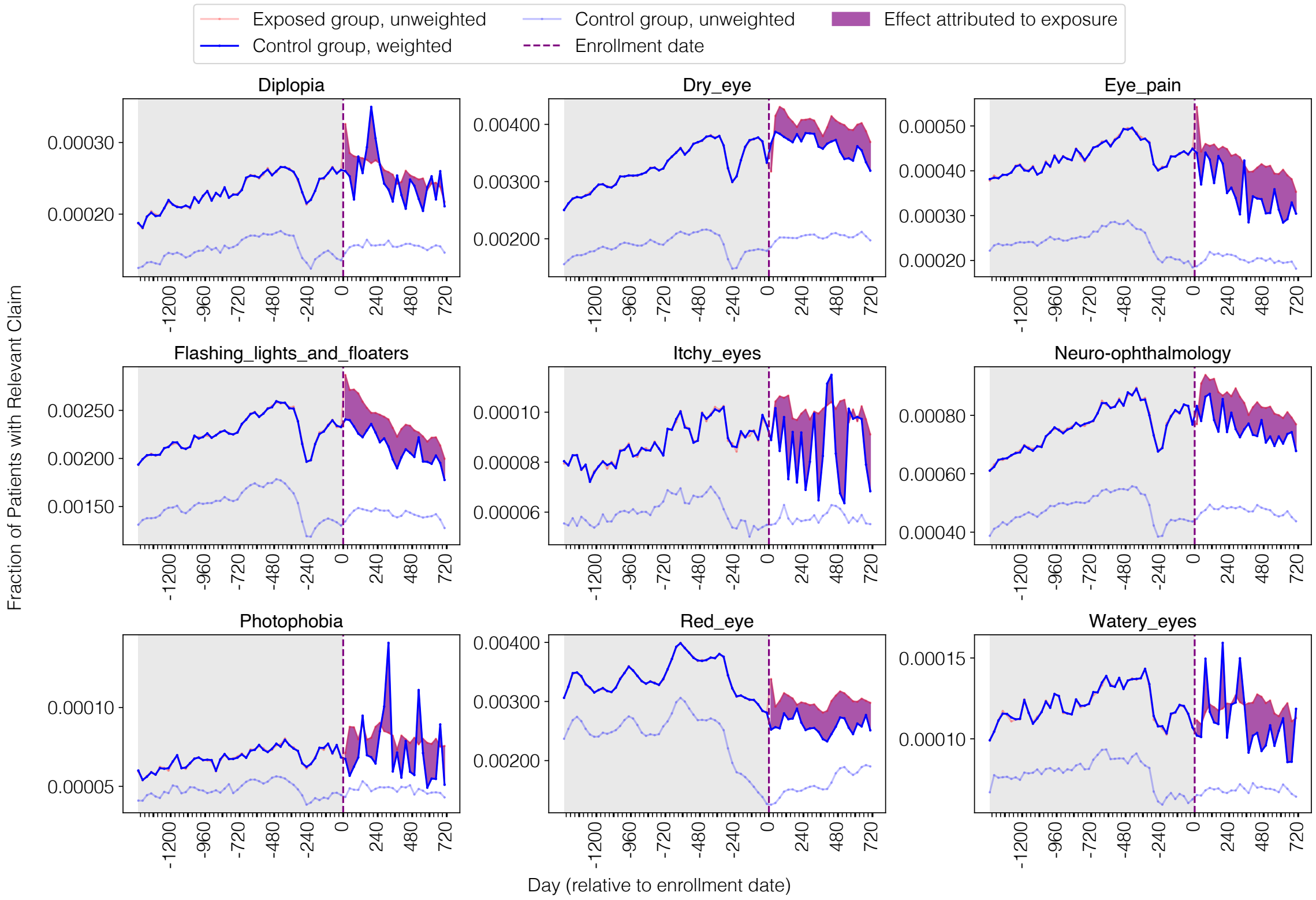


**Fig. S141.** Temporal trends in Eyes prevalence. The fraction of patients with relevant claims over time belonging for each outcome in the **unrestricted control design** experiment.

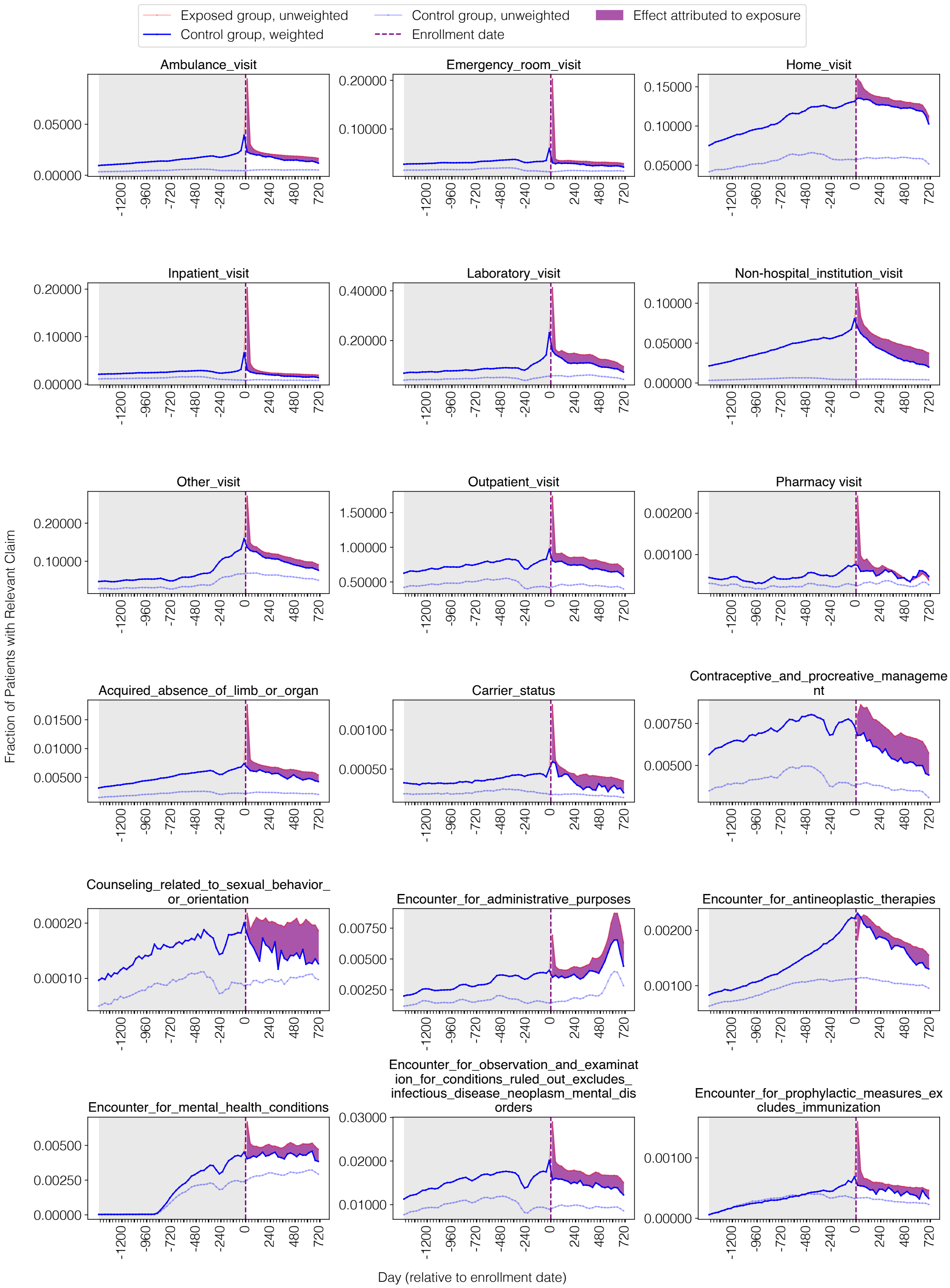


**Fig. S142.** Temporal trends in Factors Influencing Health Status and Contact with Health Services prevalence. The fraction of patients with relevant claims over time belonging for each outcome in the **unrestricted control design** experiment.

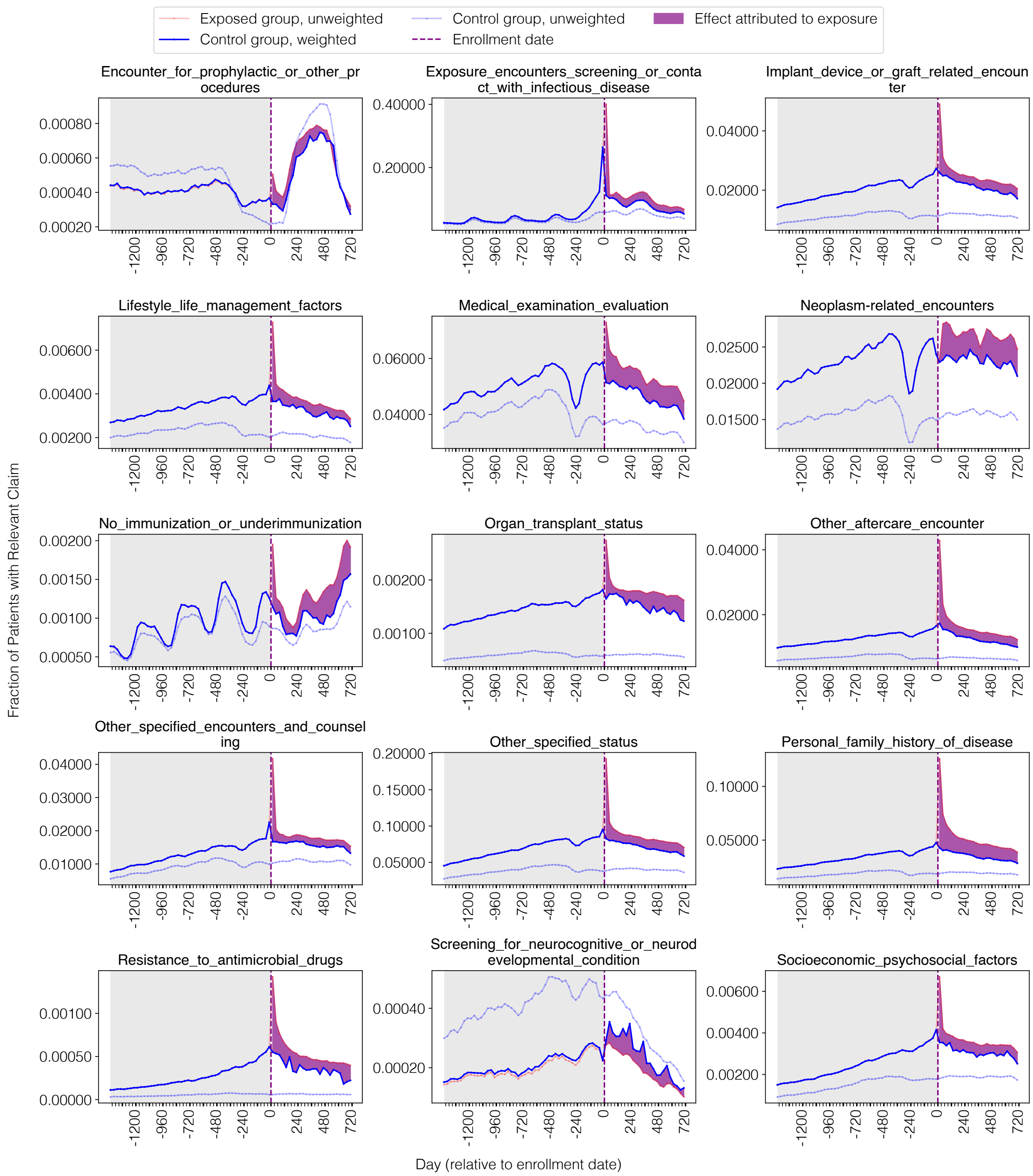


**Fig. S142.** (cont'd) Temporal trends in Factors Influencing Health Status and Contact with Health Services prevalence. The fraction of patients with relevant claims over time belonging for each outcome in the **unrestricted control design** experiment.

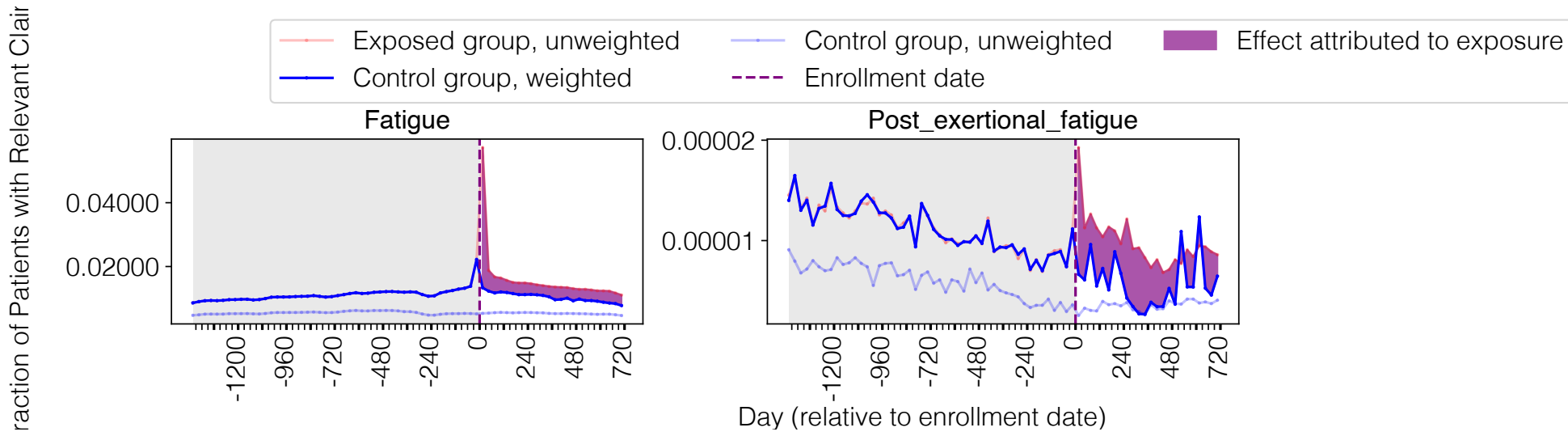


**Fig. S143.** Temporal trends in Fatigue prevalence. The fraction of patients with relevant claims over time belonging for each outcome in the **unrestricted control design** experiment.

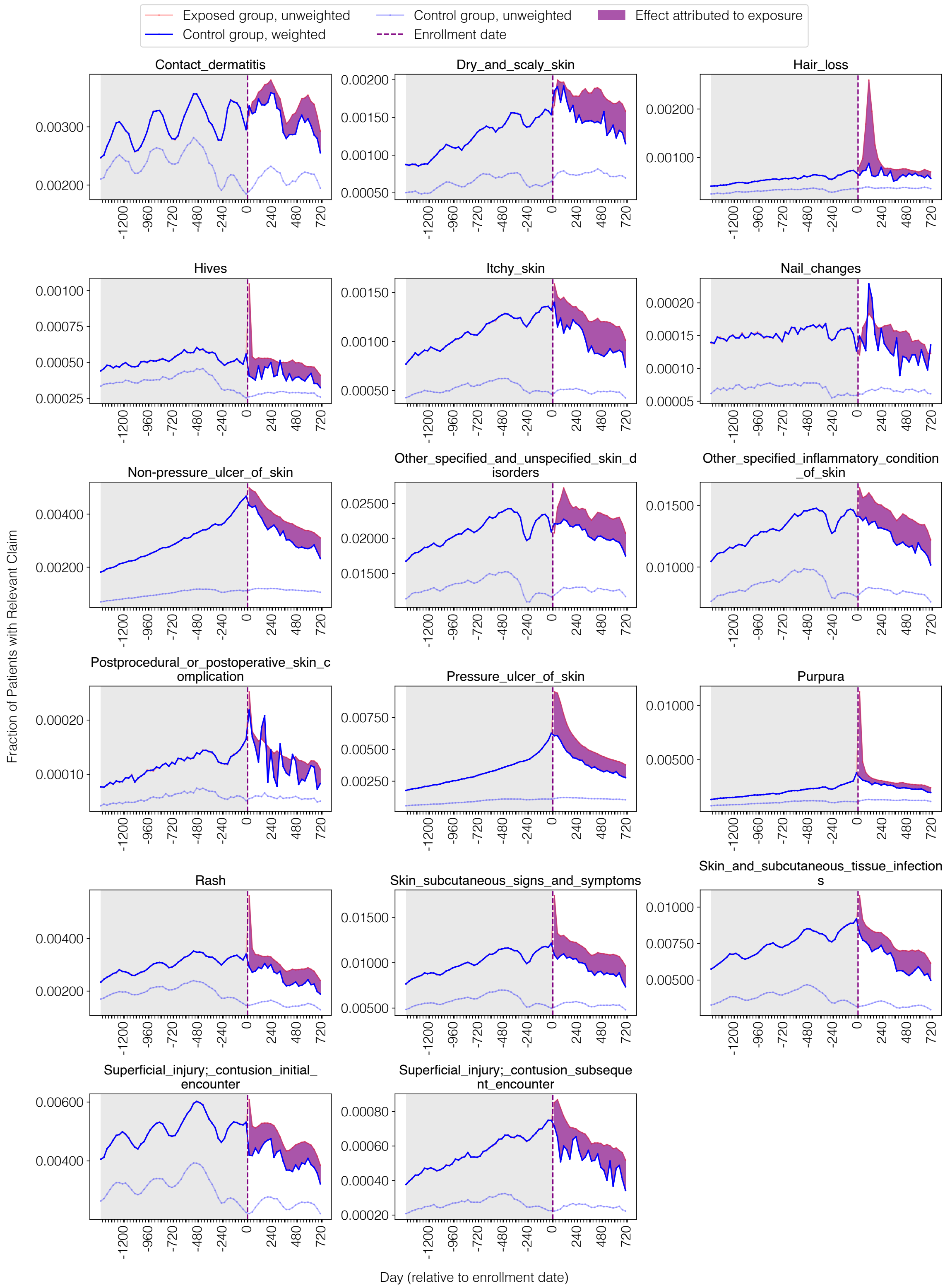


**Fig. S144.** Temporal trends in Hair, skin and nails prevalence. The fraction of patients with relevant claims over time belonging for each outcome in the **unrestricted control design** experiment.

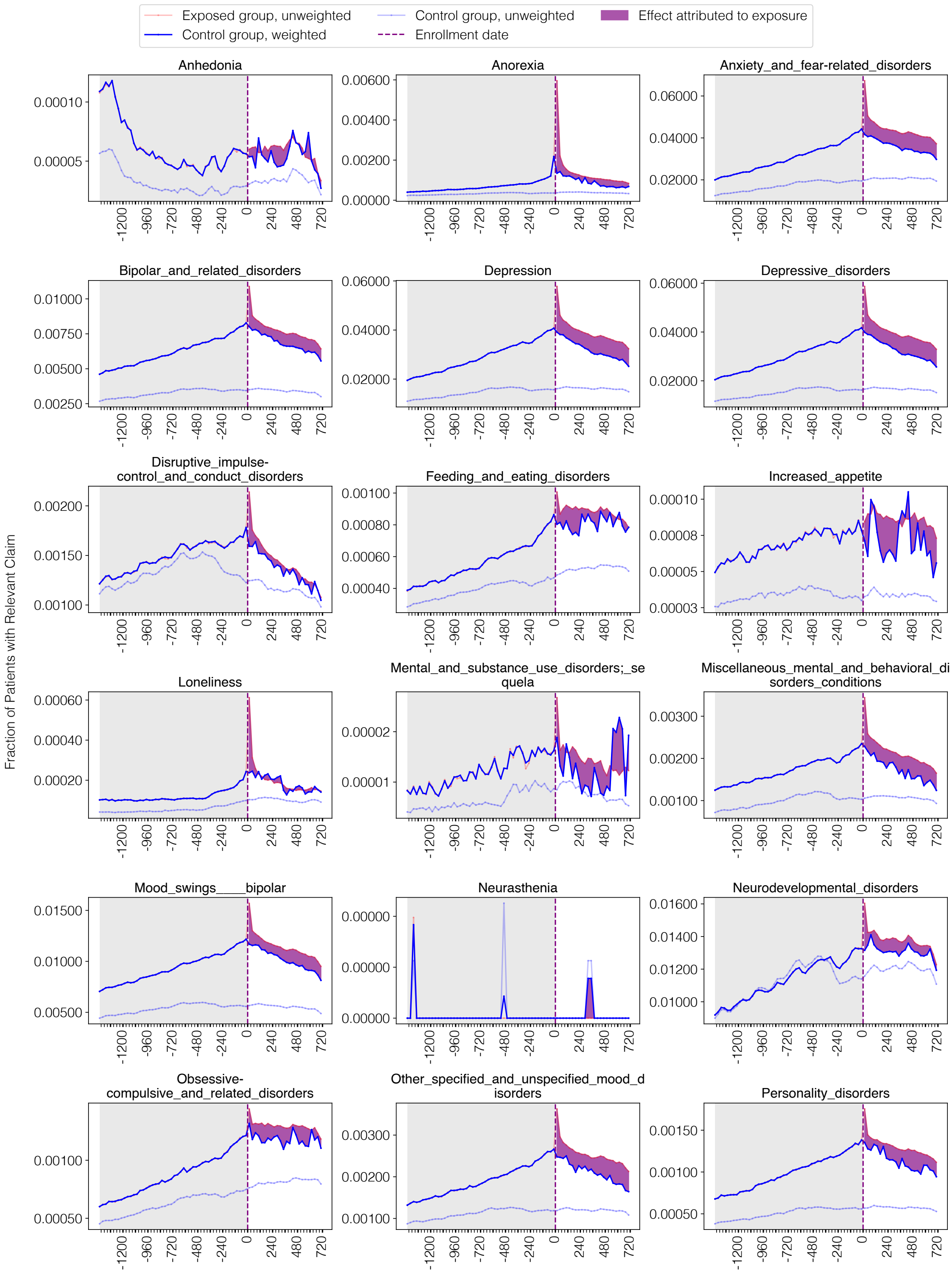


**Fig. S145.** Temporal trends in Mental health prevalence. The fraction of patients with relevant claims over time belonging for each outcome in the **unrestricted control design** experiment.

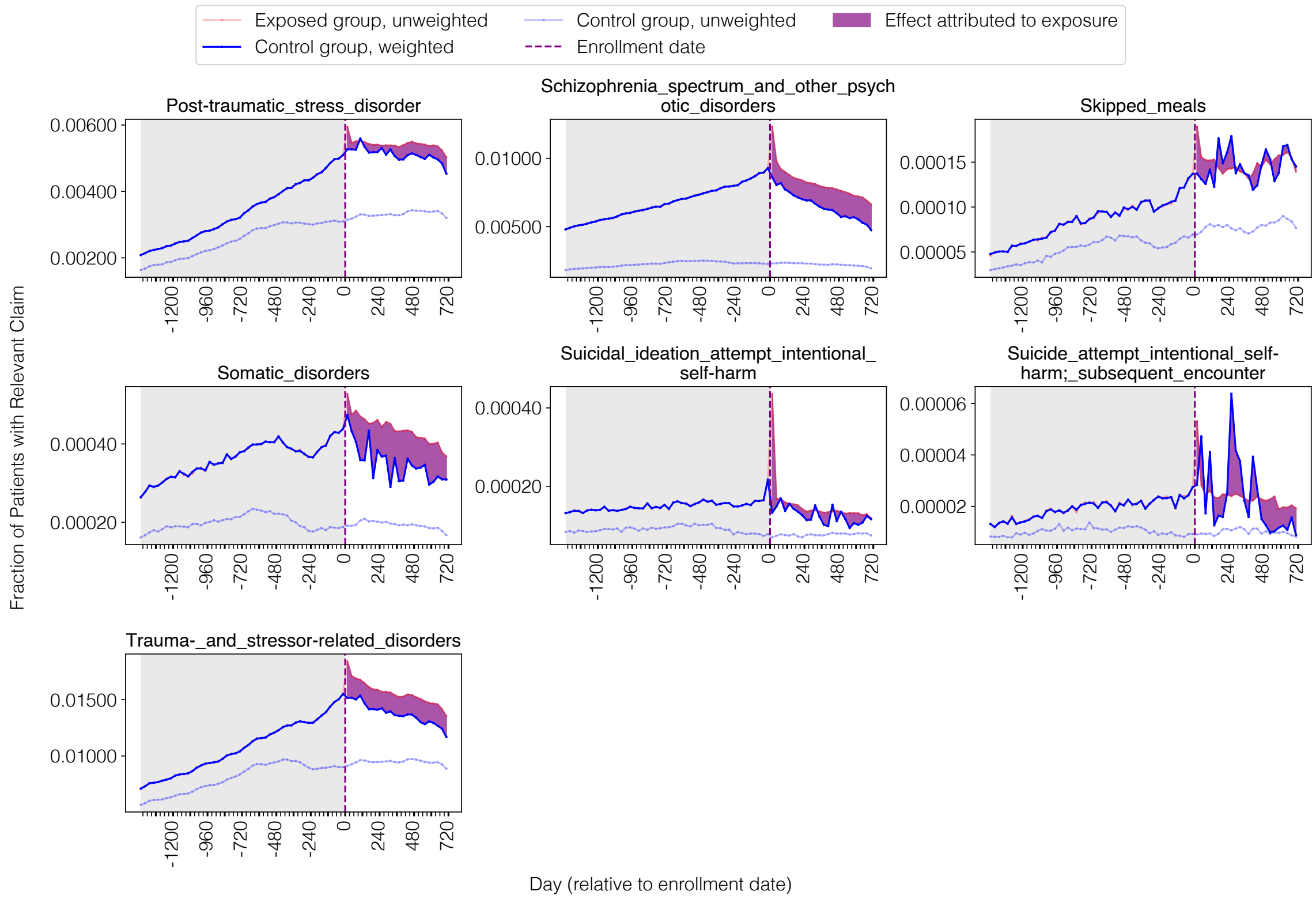


**Fig. S145.** (cont'd) Temporal trends in Mental health prevalence. The fraction of patients with relevant claims over time belonging for each outcome in the **unrestricted control design** experiment.

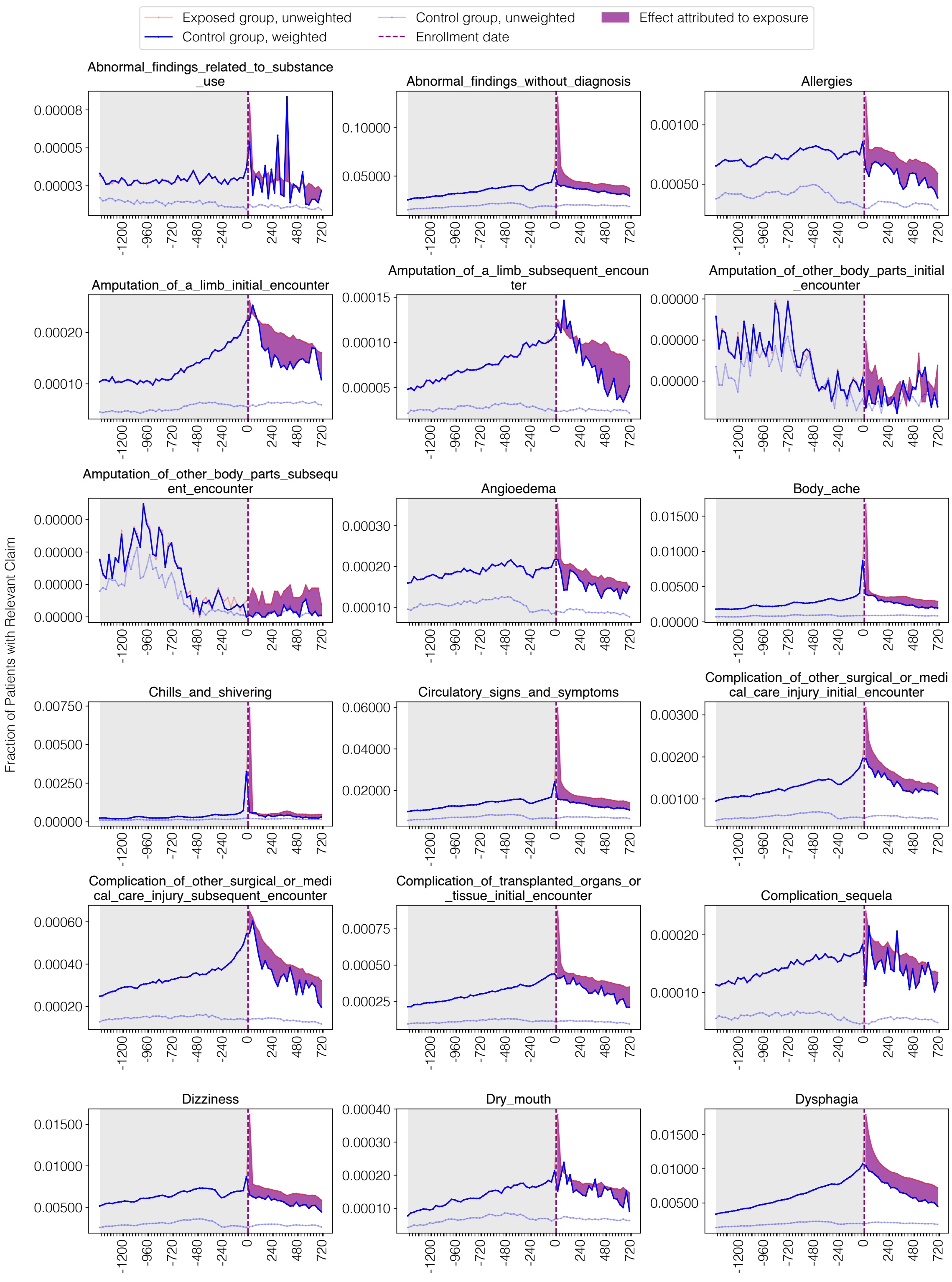


**Fig. S146.** Temporal trends in Misc prevalence. The fraction of patients with relevant claims over time belonging for each outcome in the **unrestricted control design** experiment.

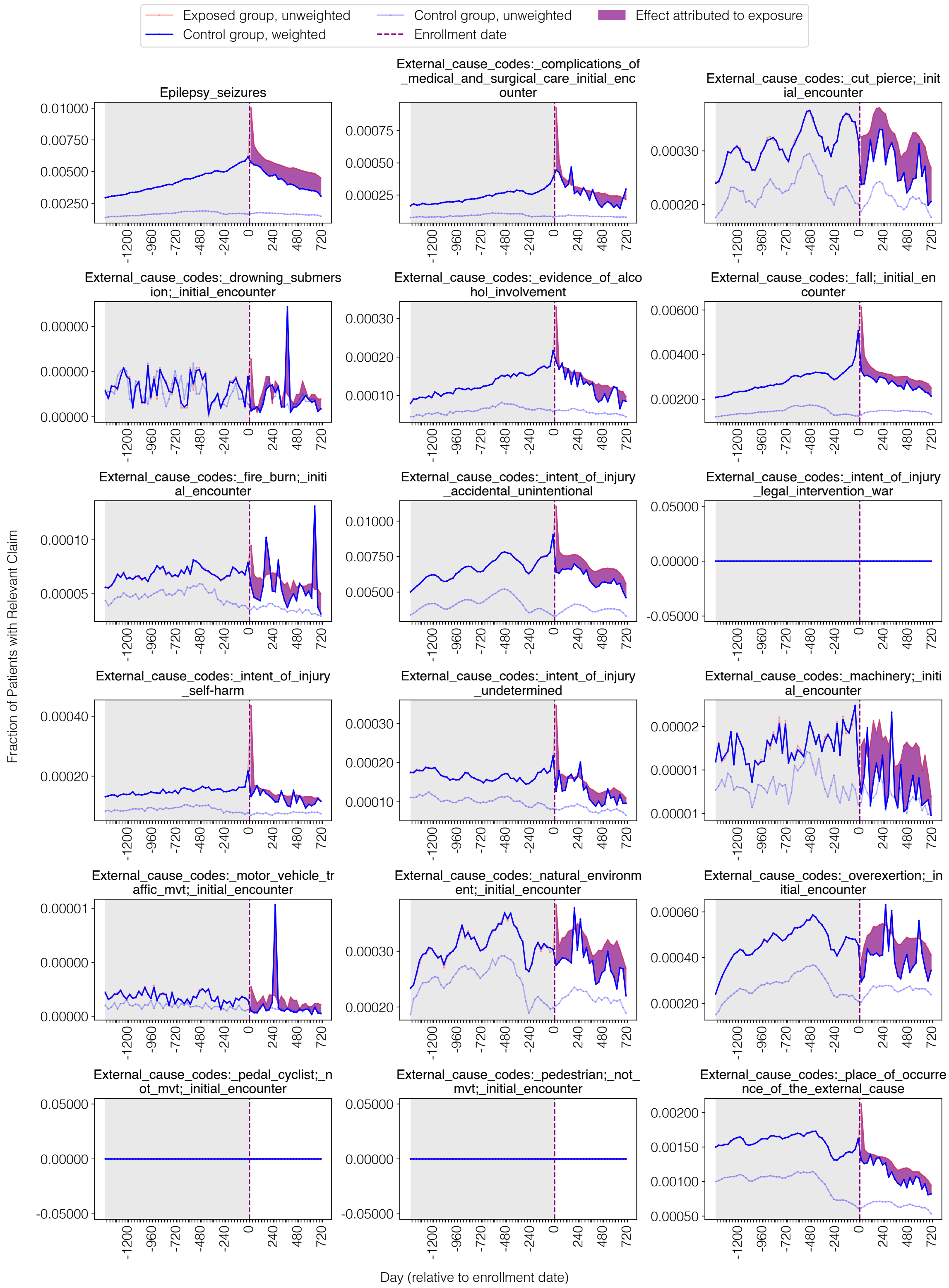


**Fig. S146.** (cont'd) Temporal trends in Misc prevalence. The fraction of patients with relevant claims over time belonging for each outcome in the **unrestricted control design** experiment.

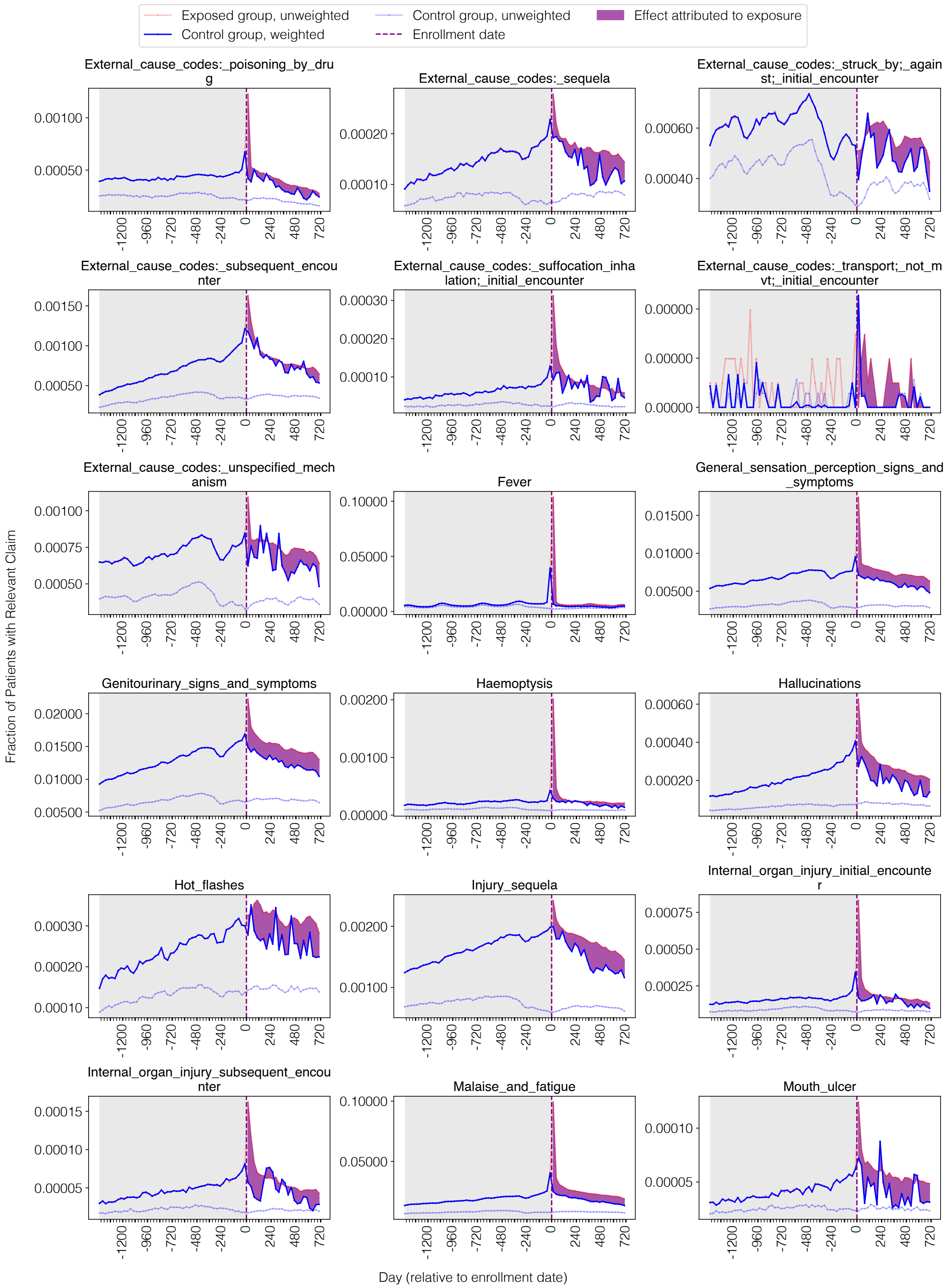


**Fig. S146.** (cont'd) Temporal trends in Misc prevalence. The fraction of patients with relevant claims over time belonging for each outcome in the **unrestricted control design** experiment.

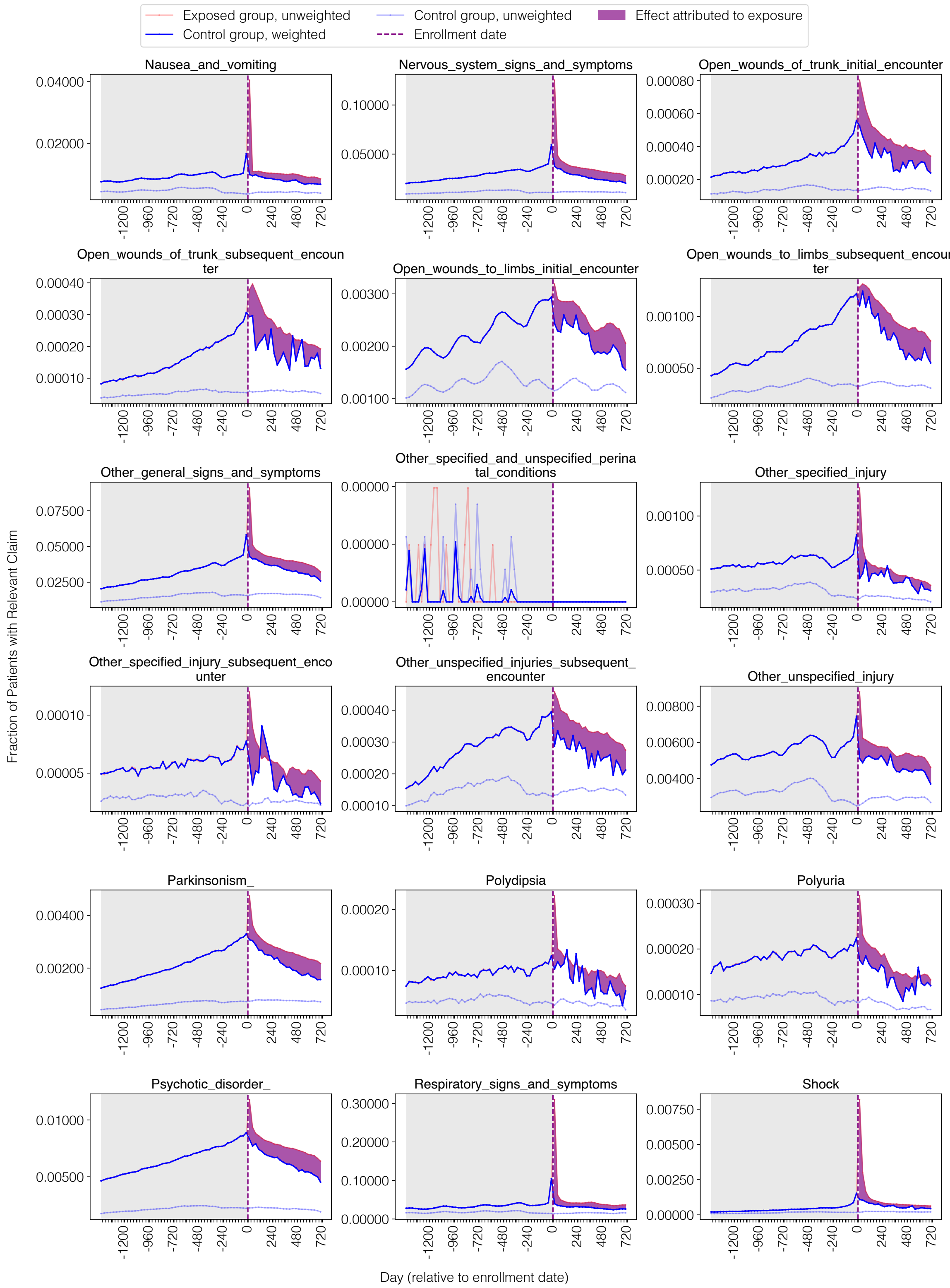


**Fig. S146.** (cont'd) Temporal trends in Misc prevalence. The fraction of patients with relevant claims over time belonging for each outcome in the **unrestricted control design** experiment.

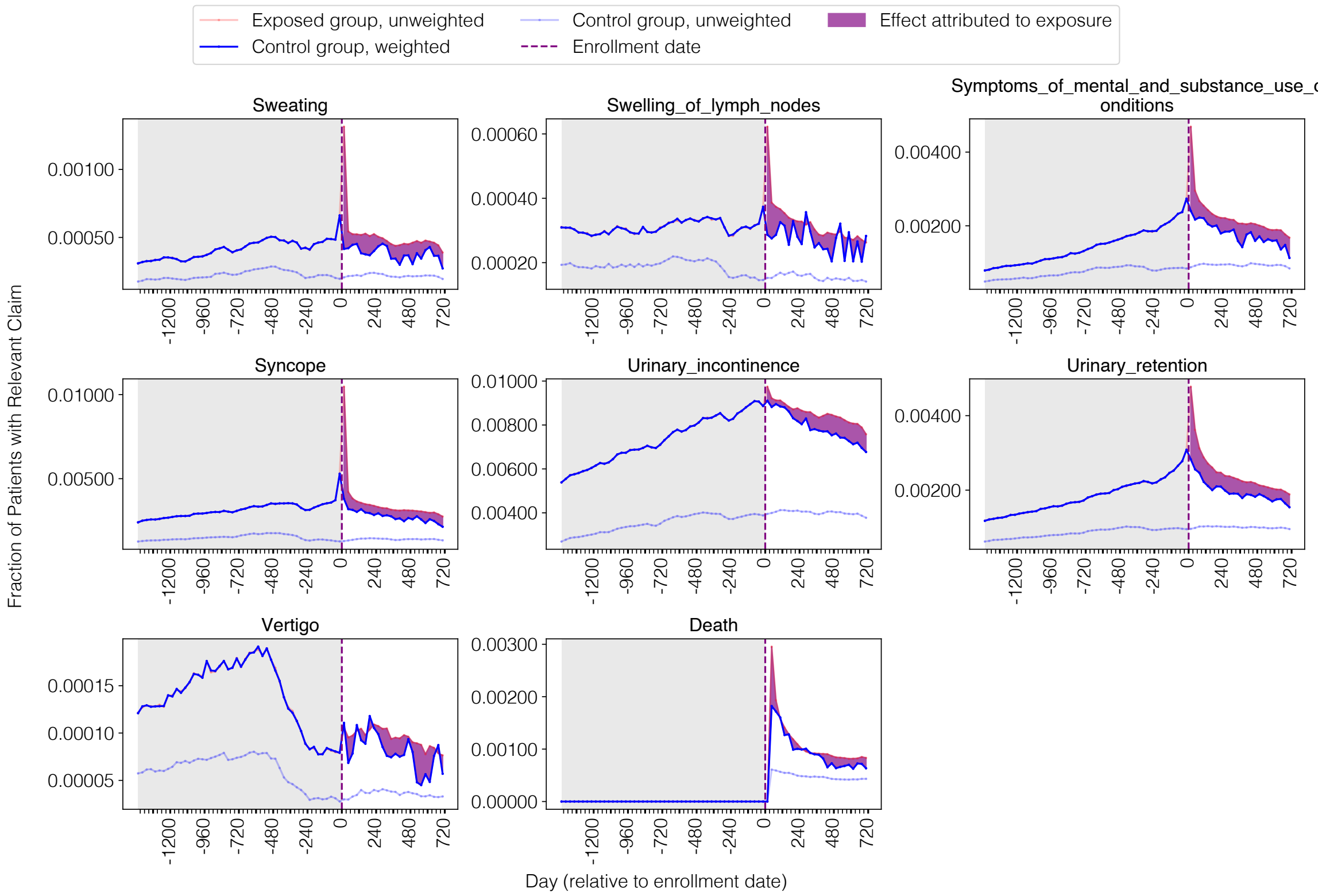


**Fig. S146.** (cont'd) Temporal trends in Misc prevalence. The fraction of patients with relevant claims over time belonging for each outcome in the **unrestricted control design** experiment.

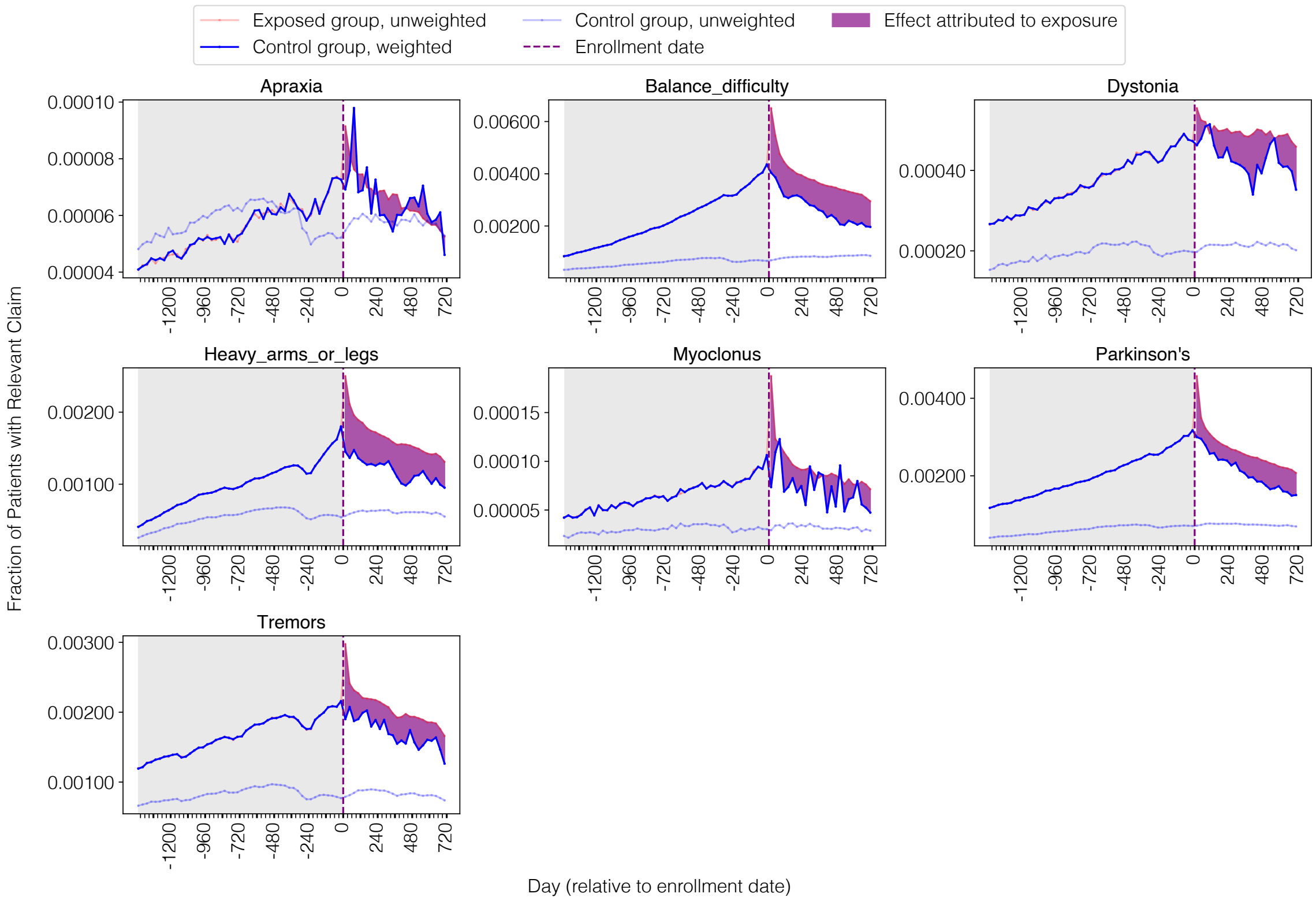


**Fig. S147.** Temporal trends in Movement prevalence. The fraction of patients with relevant claims over time belonging for each outcome in the **unrestricted control design** experiment.

 

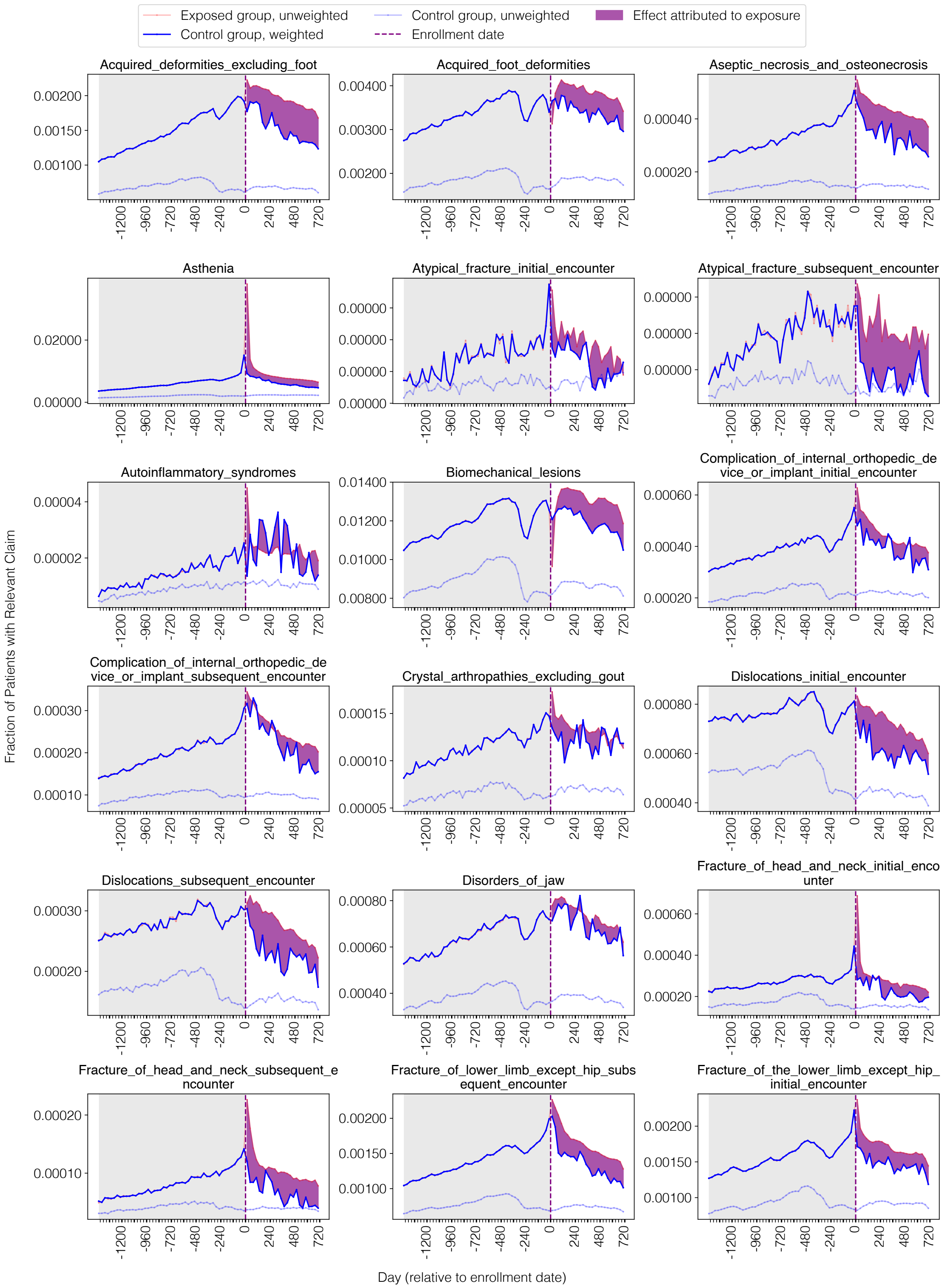


**Fig. S148.** Temporal trends in Muscles and joints prevalence. The fraction of patients with relevant claims over time belonging for each outcome in the **unrestricted control design** experiment.

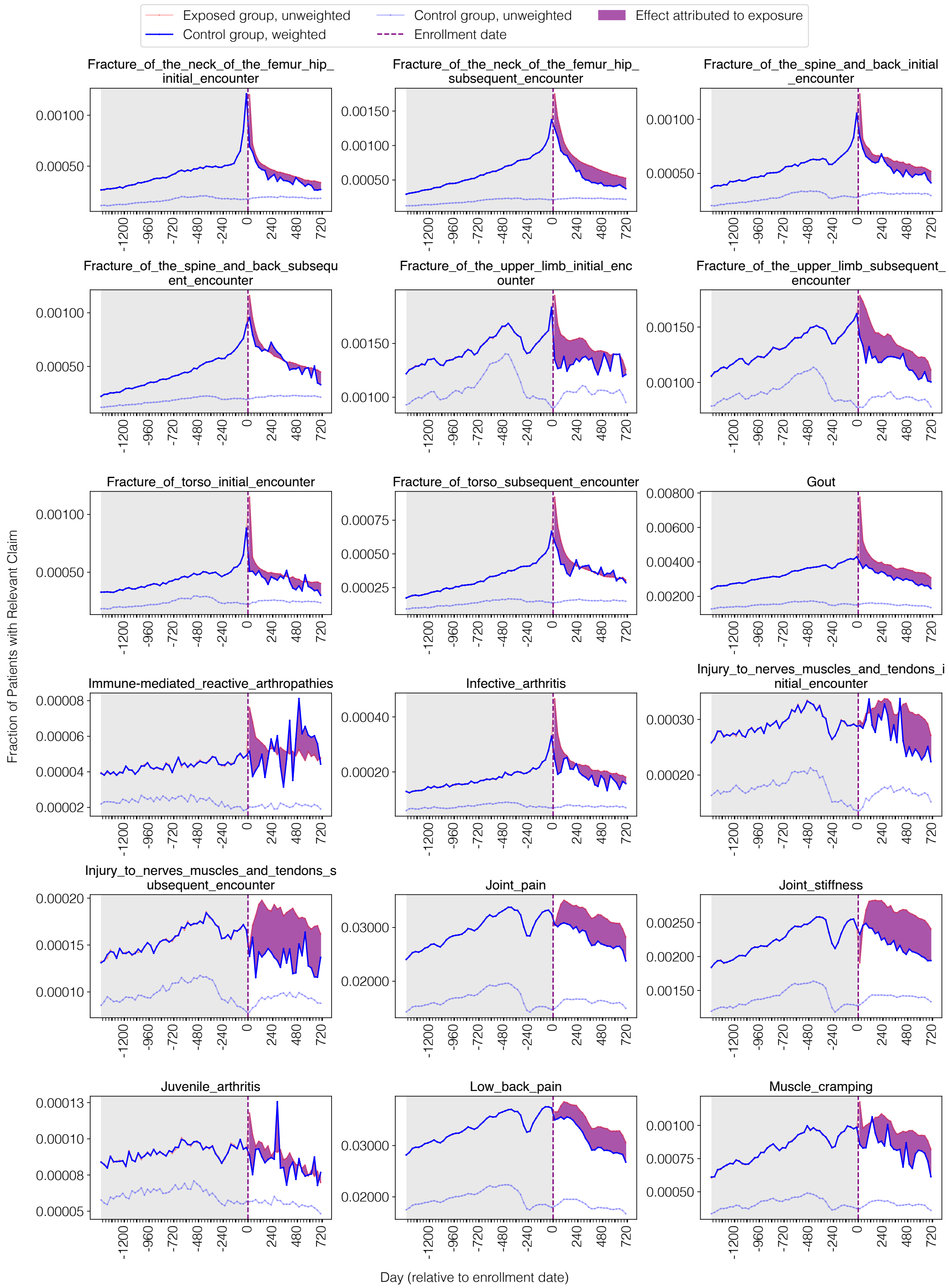


**Fig. S148.** (cont'd) Temporal trends in Muscles and joints prevalence. The fraction of patients with relevant claims over time belonging for each outcome in the **unrestricted control design** experiment.

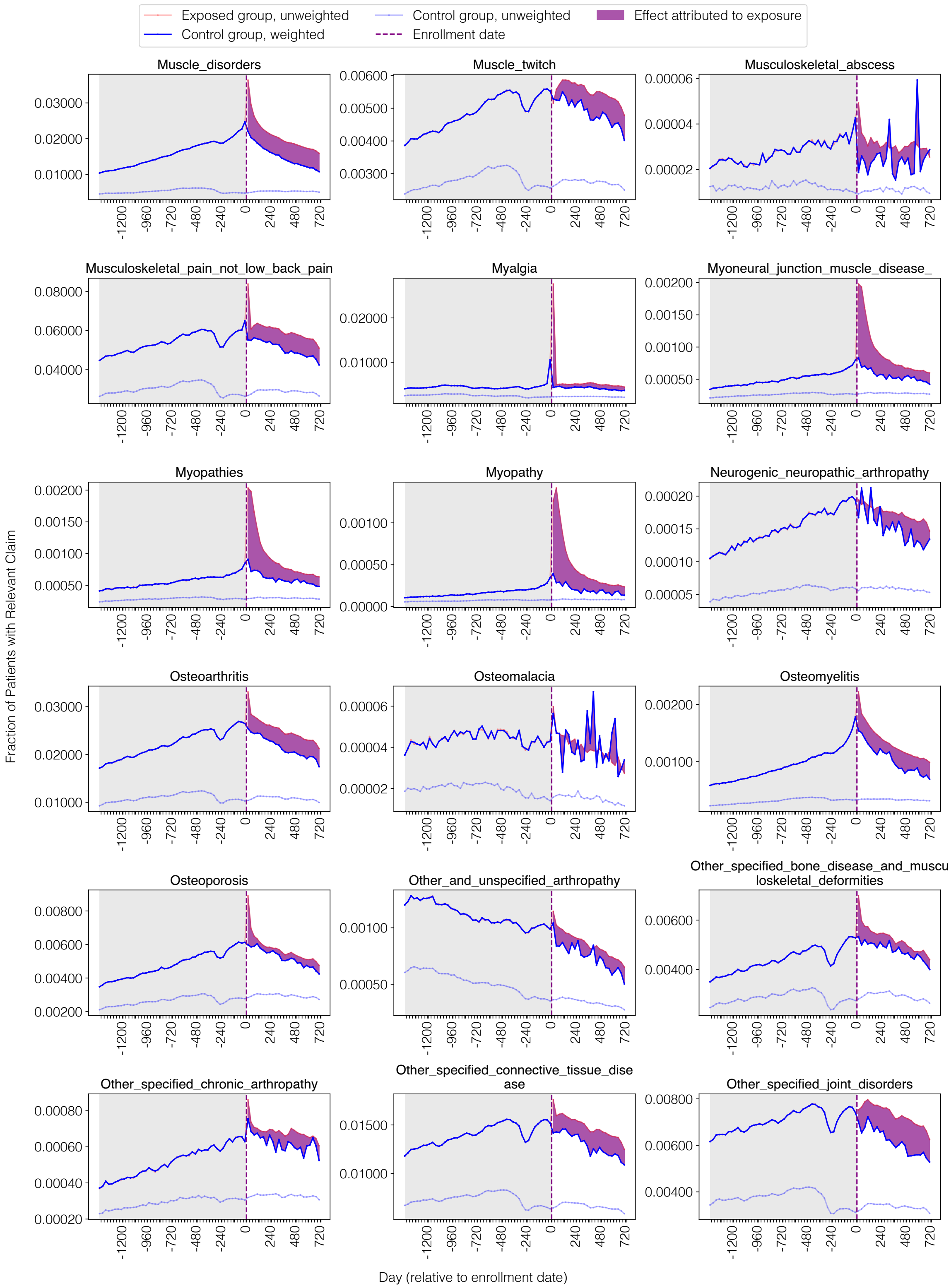


**Fig. S148.** (cont'd) Temporal trends in Muscles and joints prevalence. The fraction of patients with relevant claims over time belonging for each outcome in the **unrestricted control design** experiment.

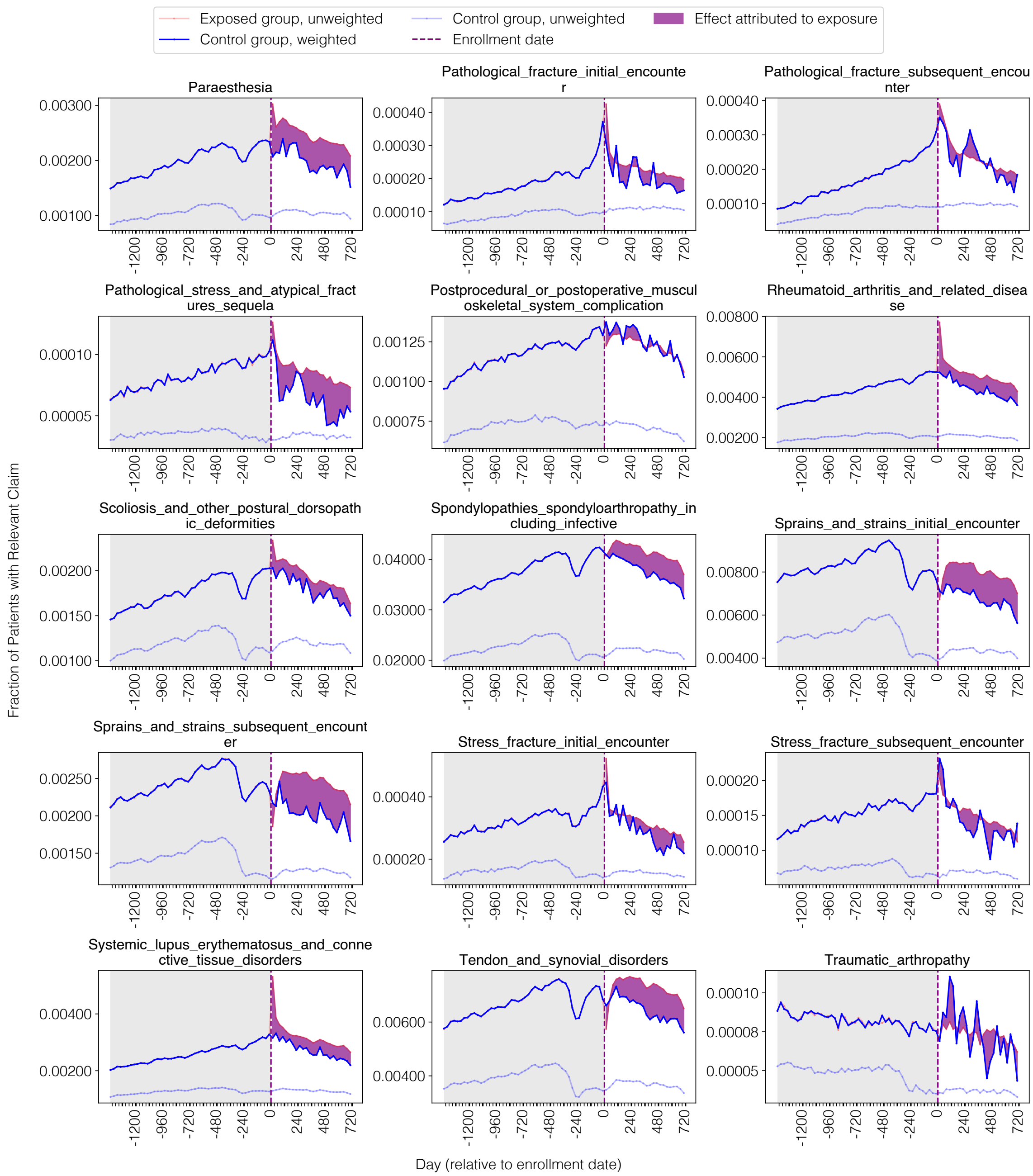


**Fig. S148.** (cont'd) Temporal trends in Muscles and joints prevalence. The fraction of patients with relevant claims over time belonging for each outcome in the **unrestricted control design** experiment.

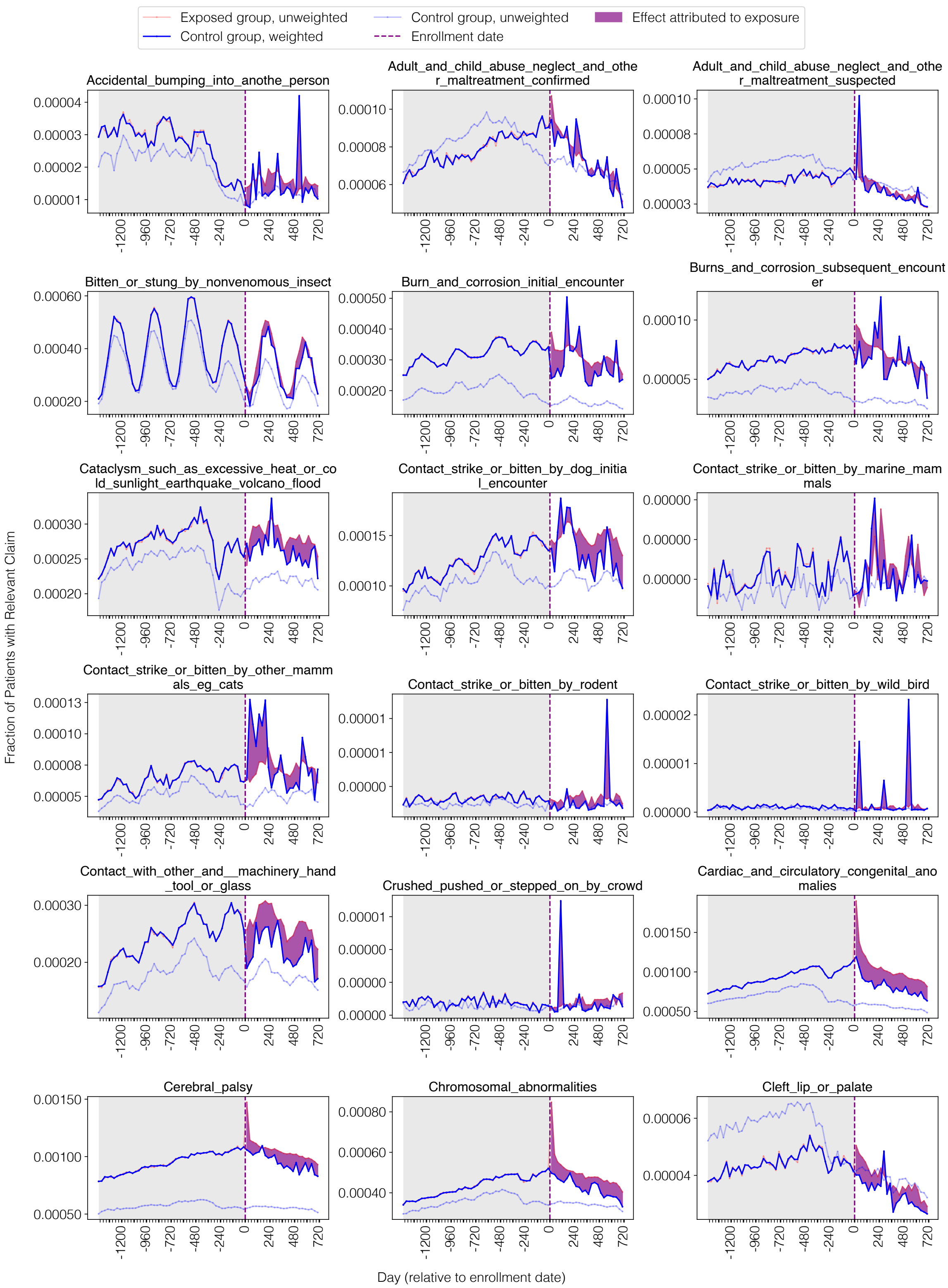


**Fig. S149.** Temporal trends in Negative prevalence. The fraction of patients with relevant claims over time belonging for each outcome in the **unrestricted control design** experiment.

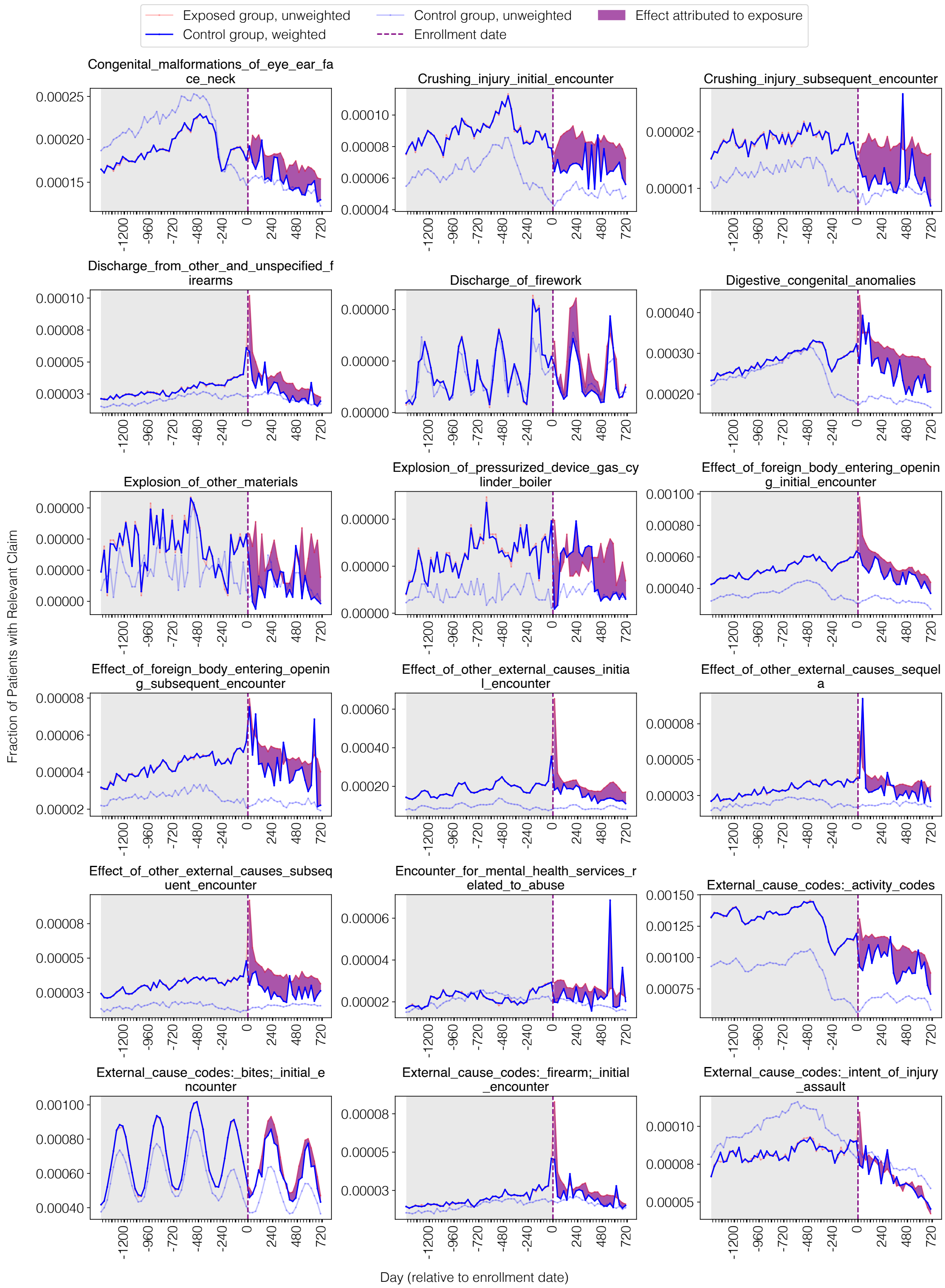


**Fig. S149.** (cont'd) Temporal trends in Negative prevalence. The fraction of patients with relevant claims over time belonging for each outcome in the **unrestricted control design** experiment.

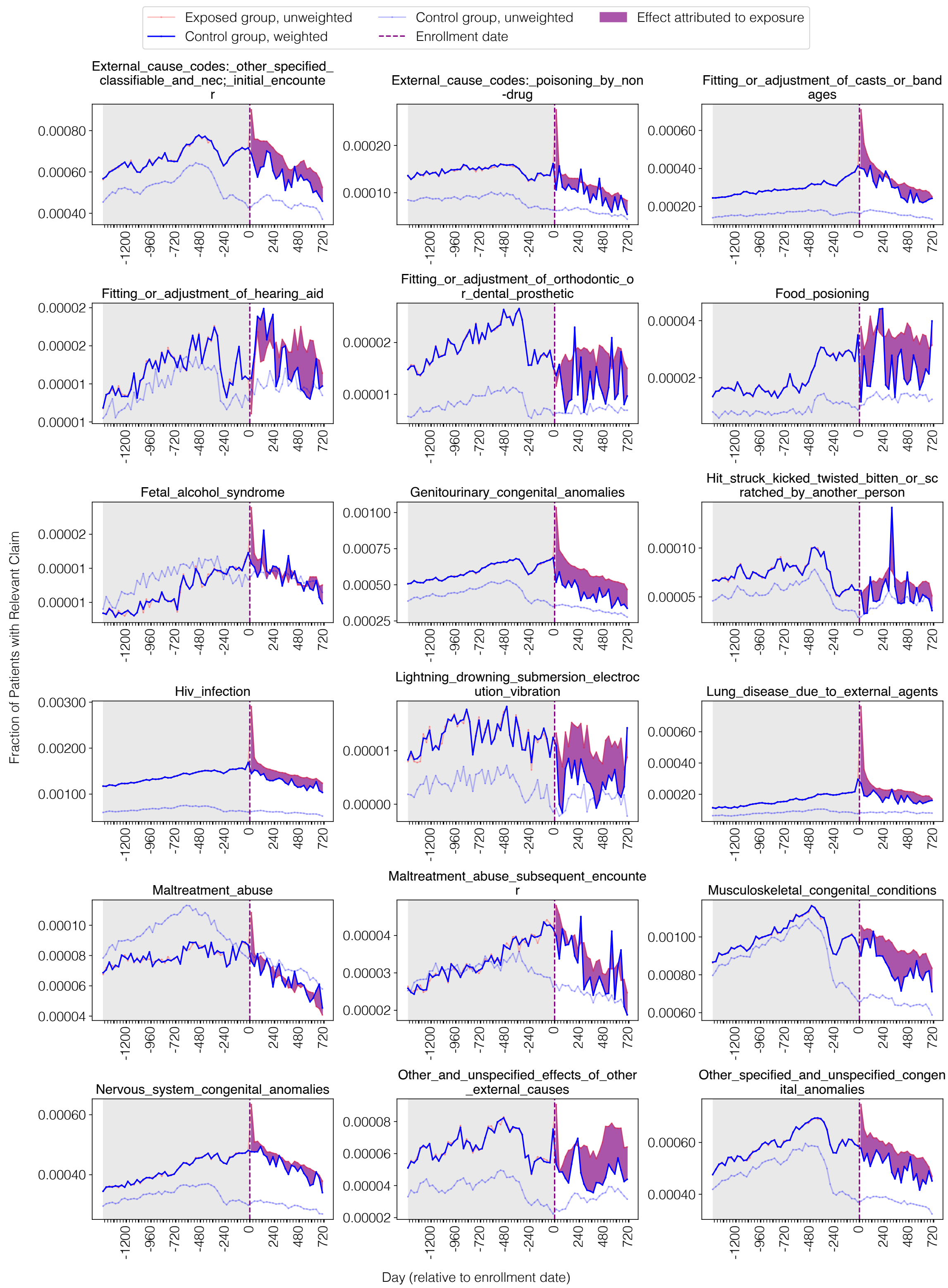


**Fig. S149.** (cont'd) Temporal trends in Negative prevalence. The fraction of patients with relevant claims over time belonging for each outcome in the **unrestricted control design** experiment.

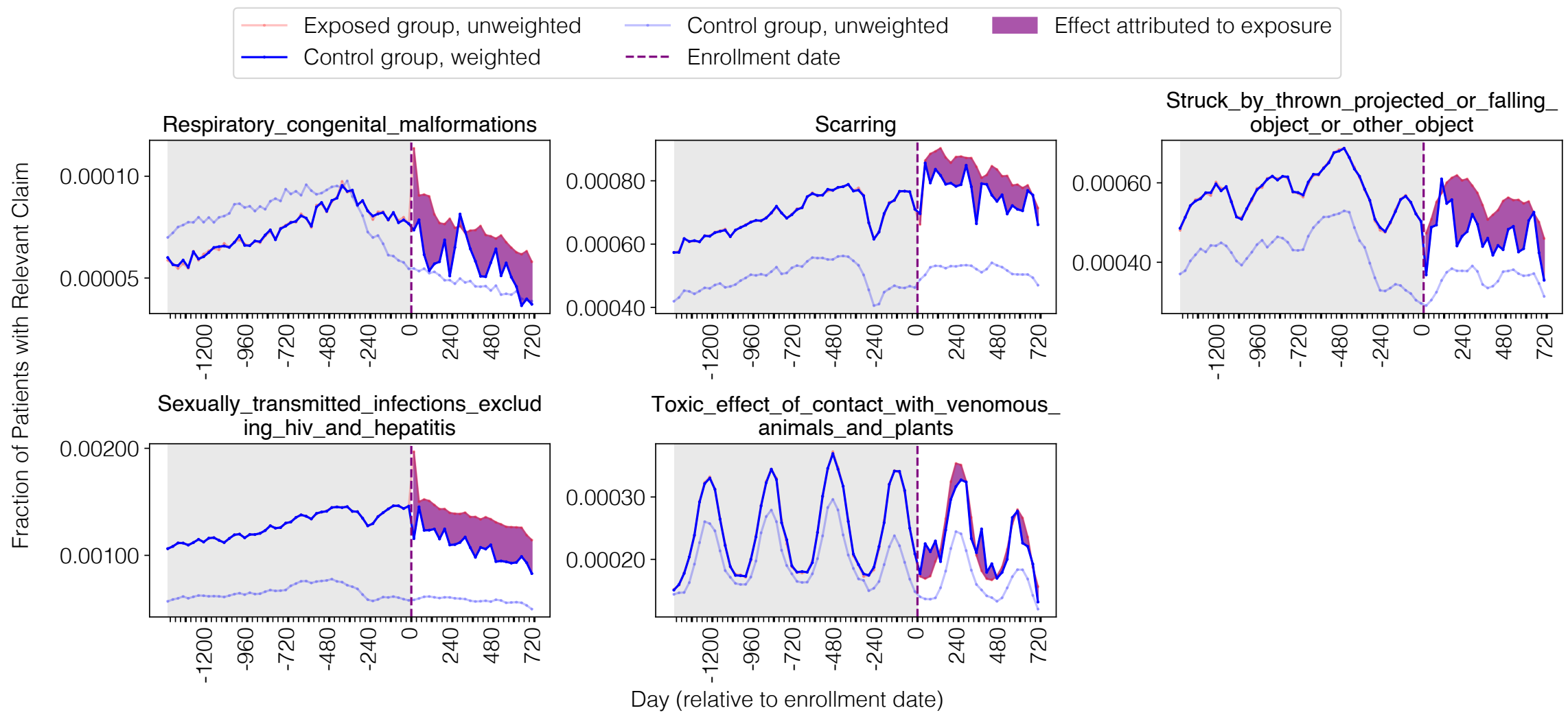


**Fig. S149.** (cont'd) Temporal trends in Negative prevalence. The fraction of patients with relevant claims over time belonging for each outcome in the **unrestricted control design** experiment.

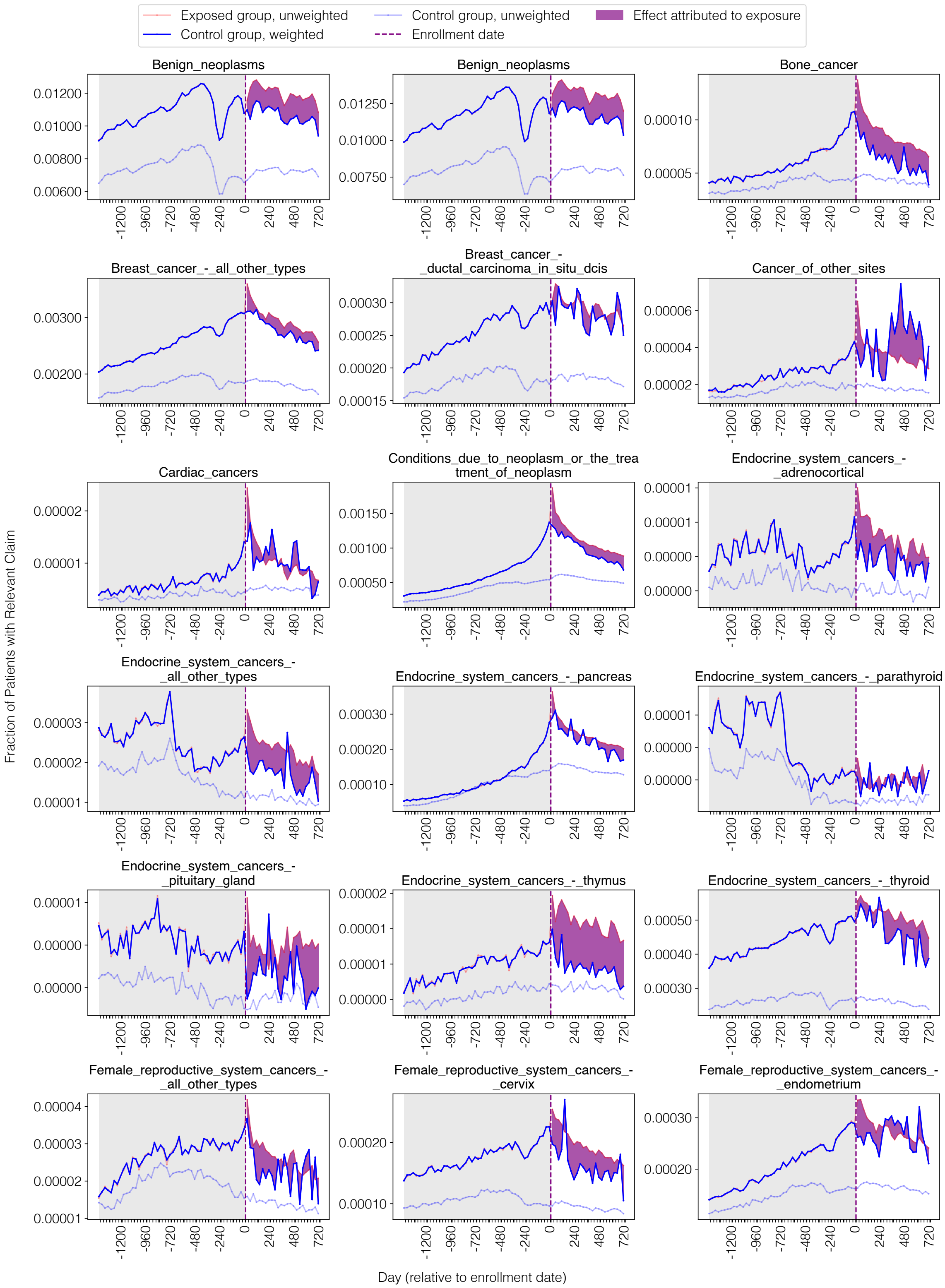


**Fig. S150.** Temporal trends in Neoplasms prevalence. The fraction of patients with relevant claims over time belonging for each outcome in the **unrestricted control design** experiment.

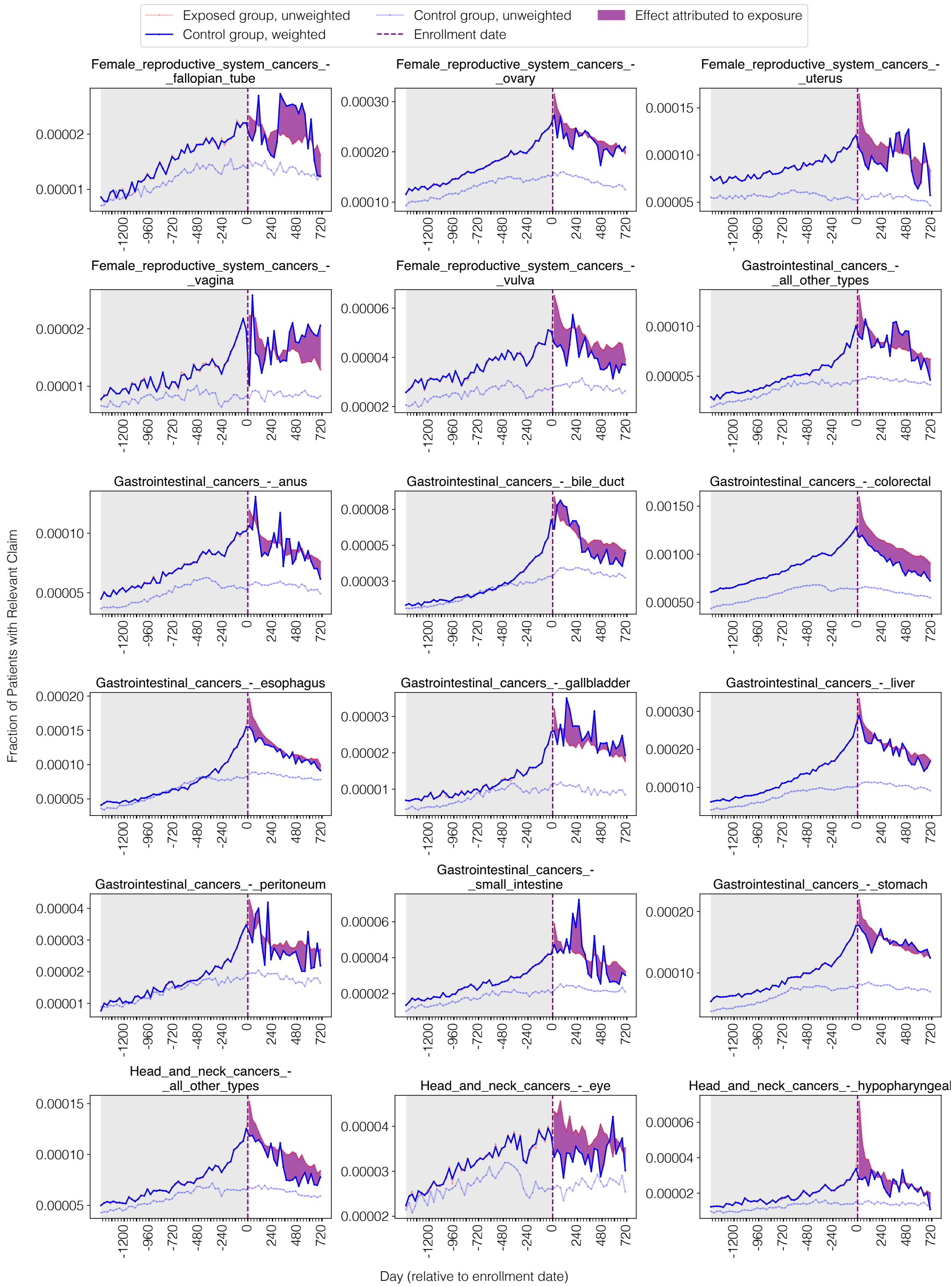


**Fig. S150.** (cont'd) Temporal trends in Neoplasms prevalence. The fraction of patients with relevant claims over time belonging for each outcome in the **unrestricted control design** experiment.

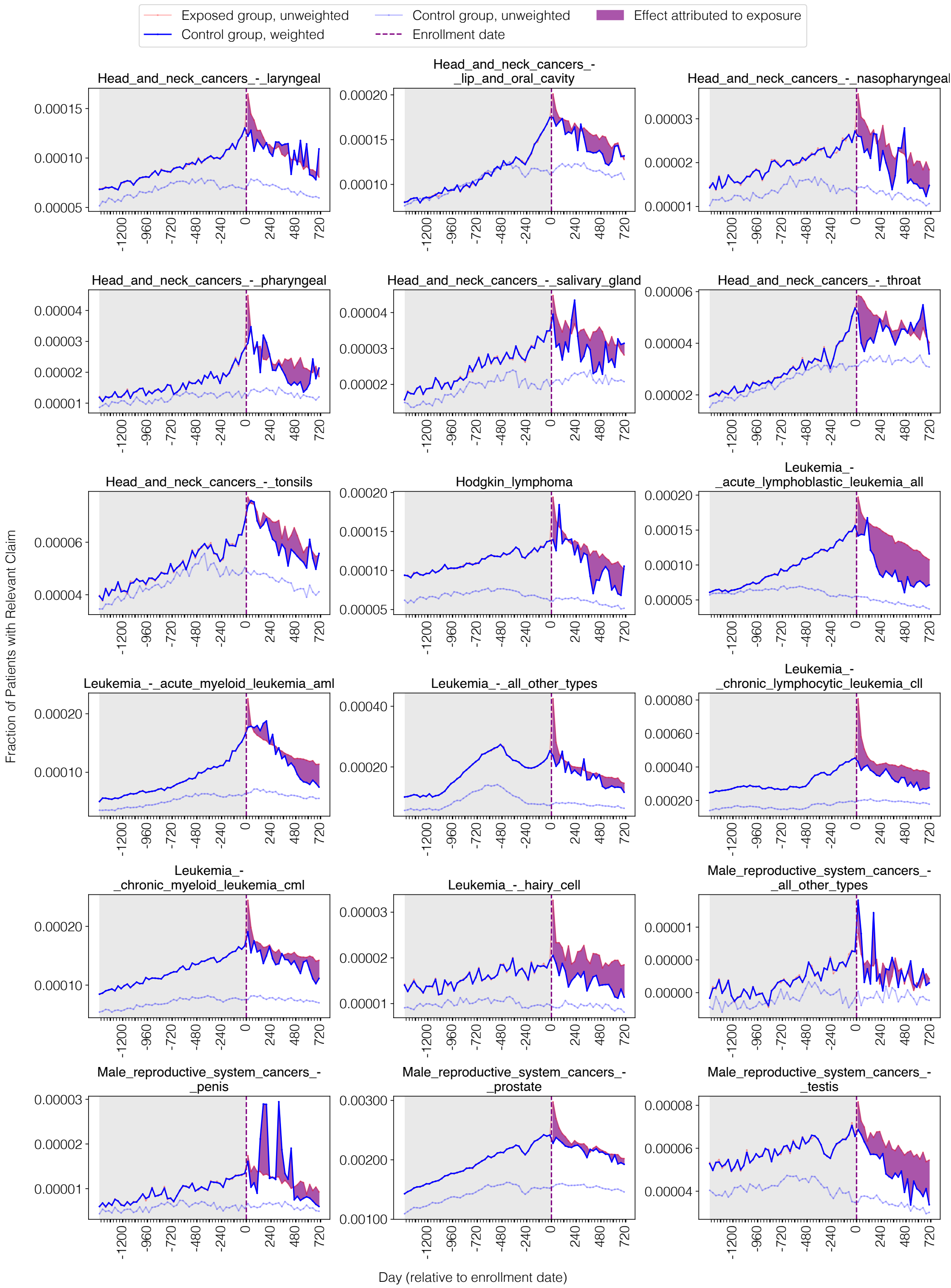


**Fig. S150.** (cont'd) Temporal trends in Neoplasms prevalence. The fraction of patients with relevant claims over time belonging for each outcome in the **unrestricted control design** experiment.

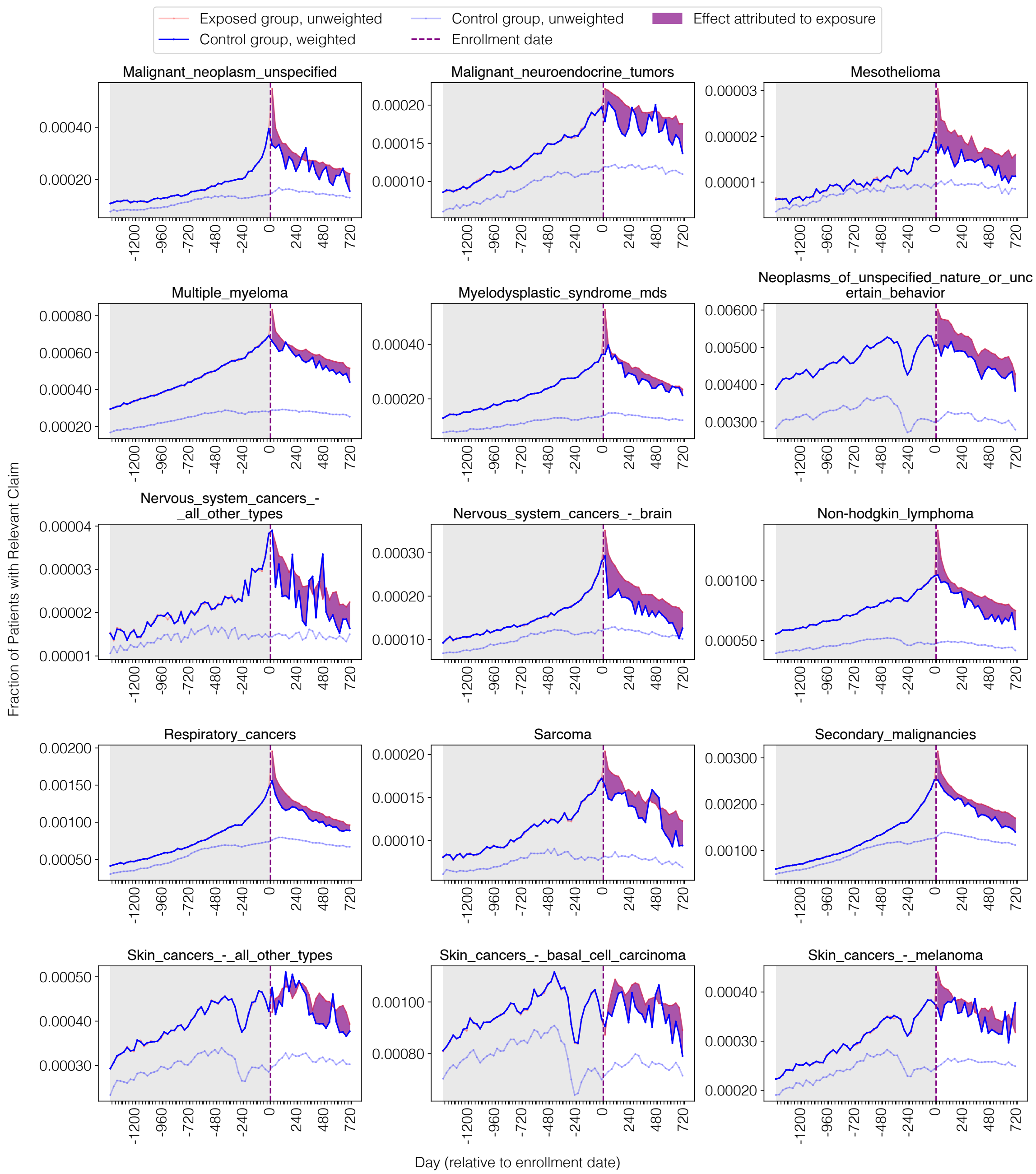


**Fig. S150.** (cont'd) Temporal trends in Neoplasms prevalence. The fraction of patients with relevant claims over time belonging for each outcome in the **unrestricted control design** experiment.

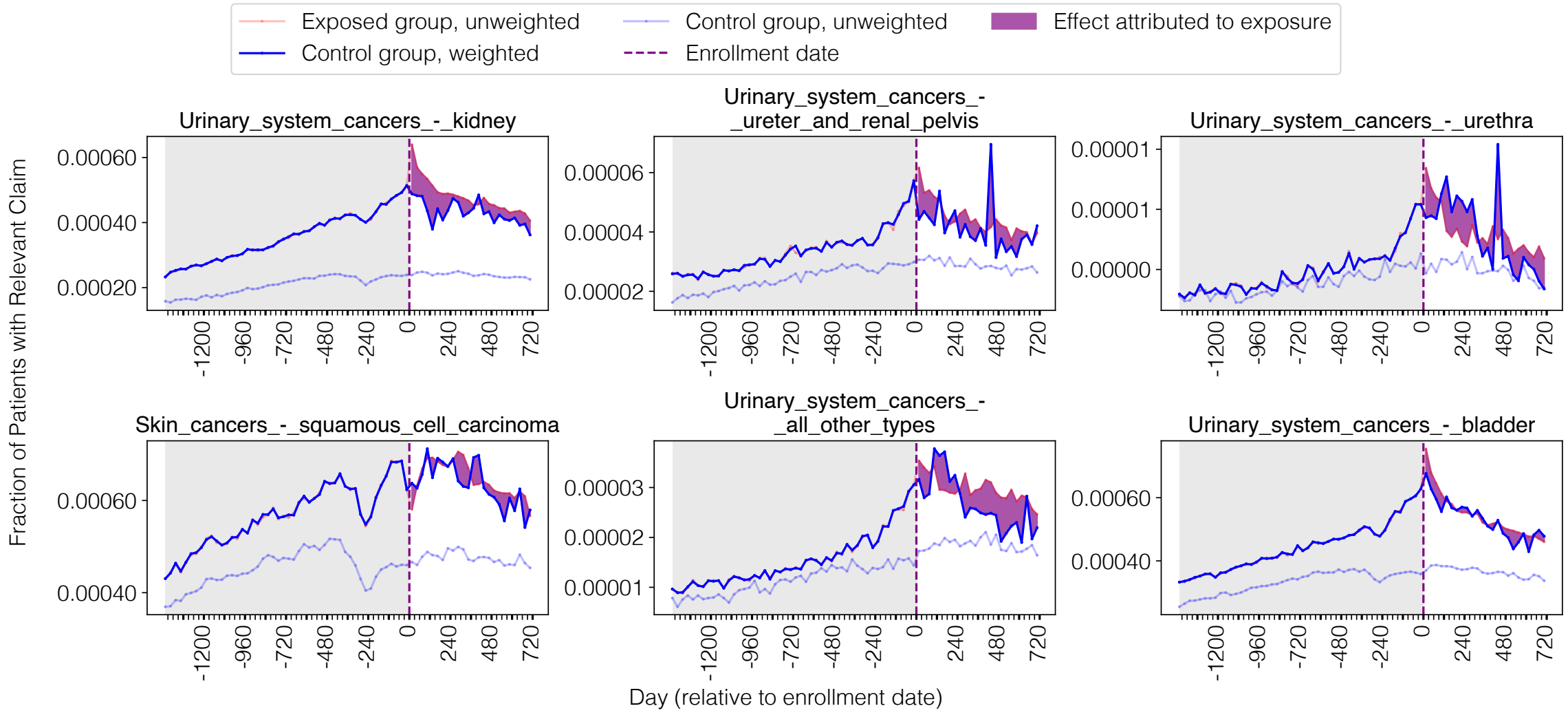


**Fig. S150.** (cont'd) Temporal trends in Neoplasms prevalence. The fraction of patients with relevant claims over time belonging for each outcome in the **unrestricted control design** experiment.

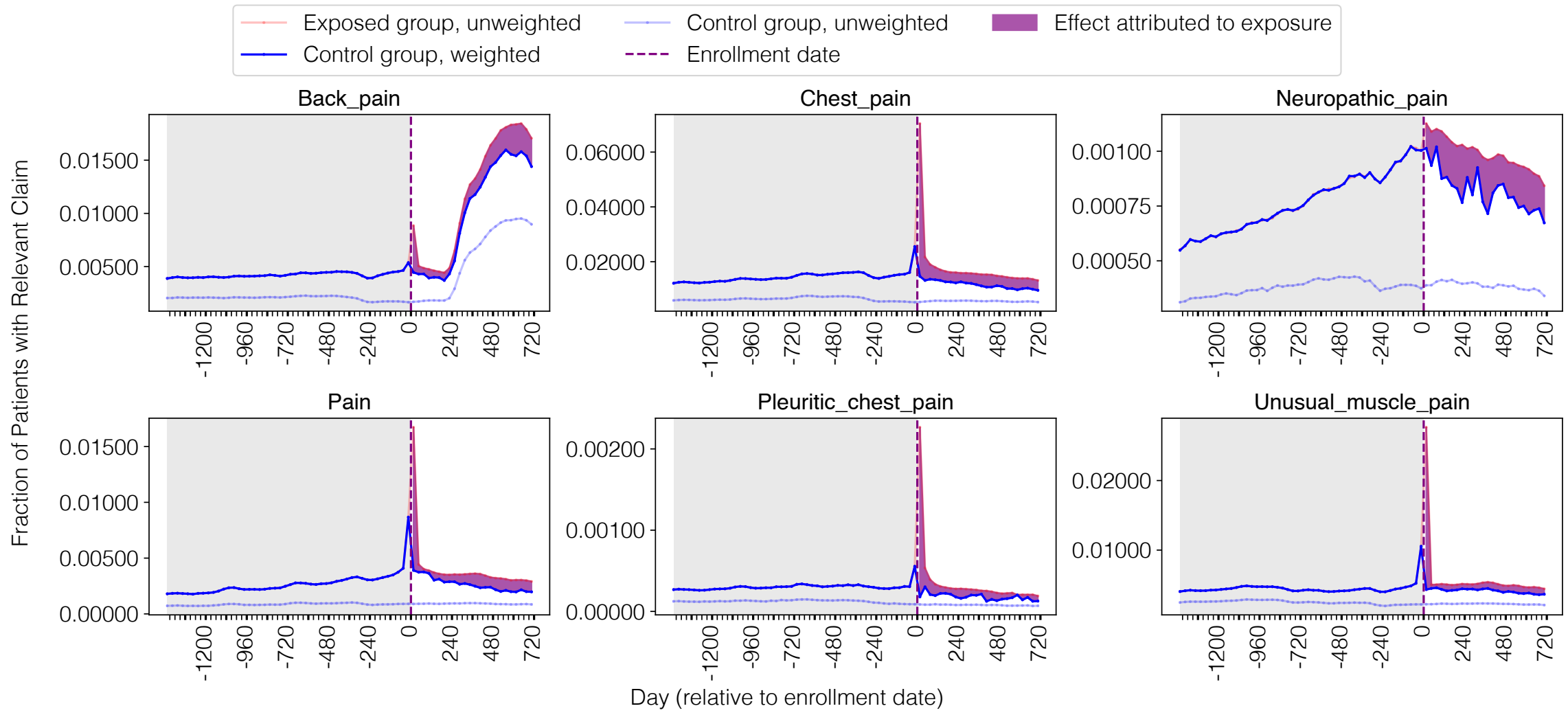


**Fig. S151.** Temporal trends in Pain prevalence. The fraction of patients with relevant claims over time belonging for each outcome in the **unrestricted control design** experiment.

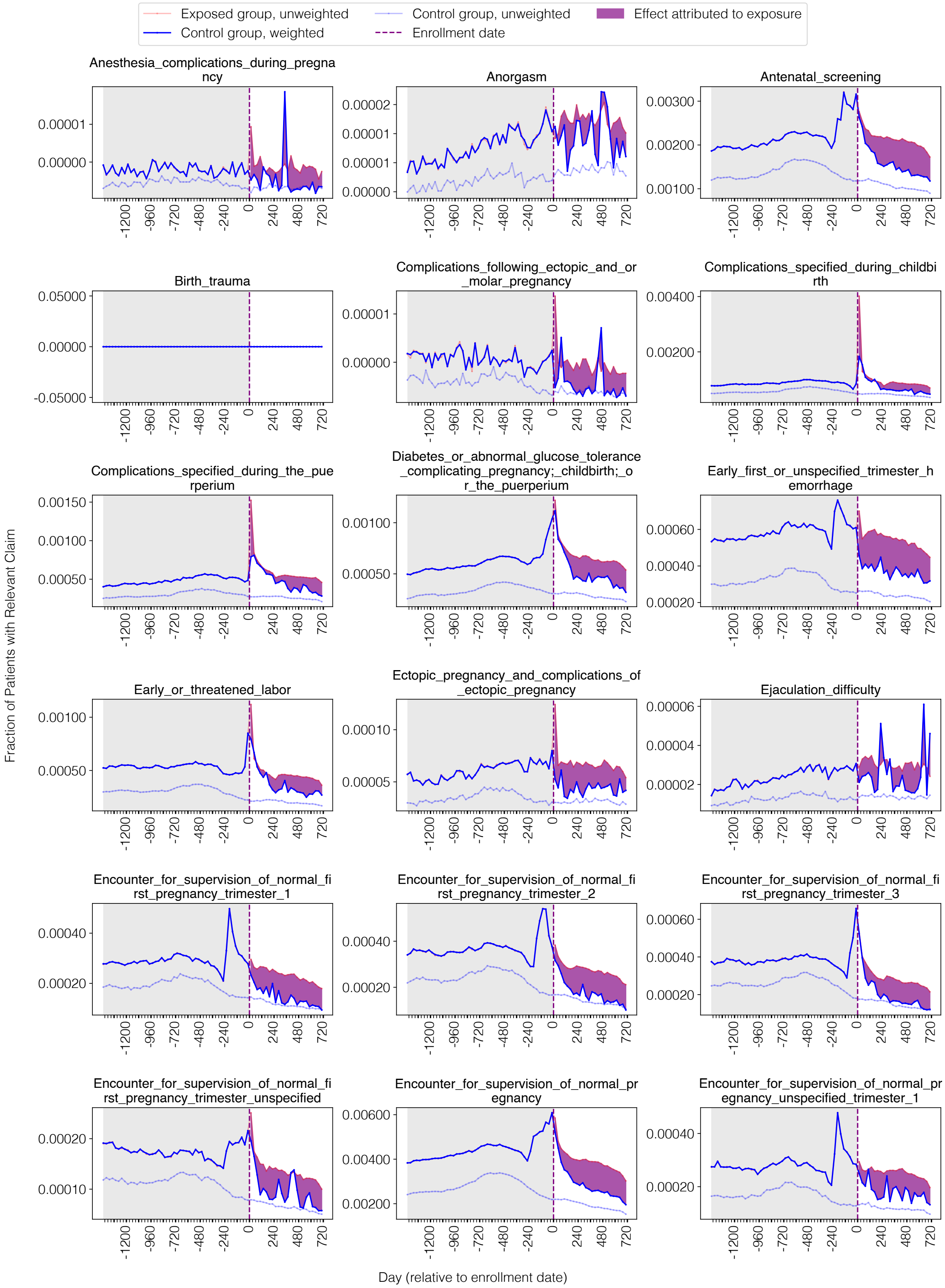


**Fig. S152.** Temporal trends in Reproductive health prevalence. The fraction of patients with relevant claims over time belonging for each outcome in the **unrestricted control design** experiment.

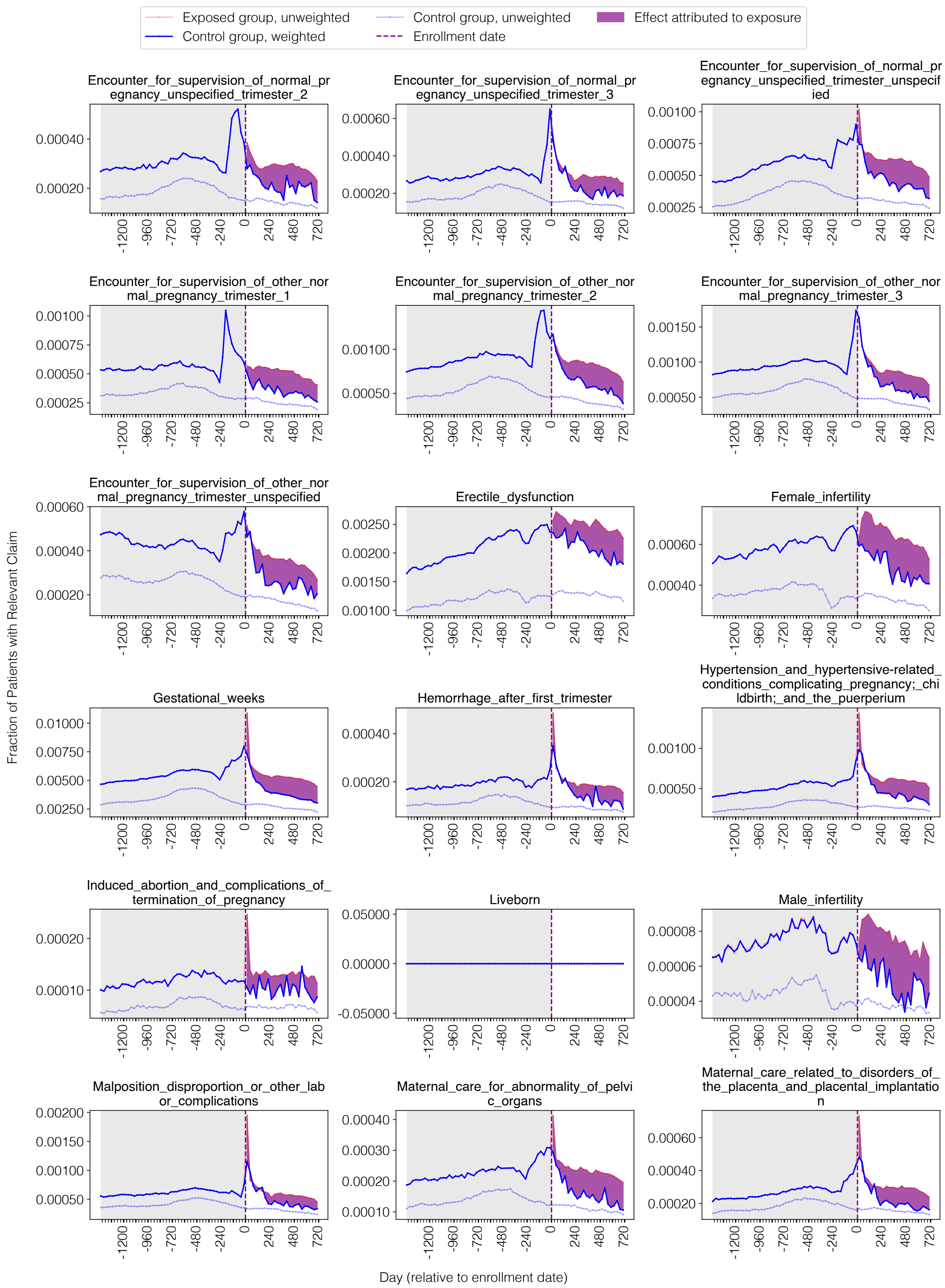


**Fig. S152.** (cont'd) Temporal trends in Reproductive health prevalence. The fraction of patients with relevant claims over time belonging for each outcome in the **unrestricted control design** experiment.

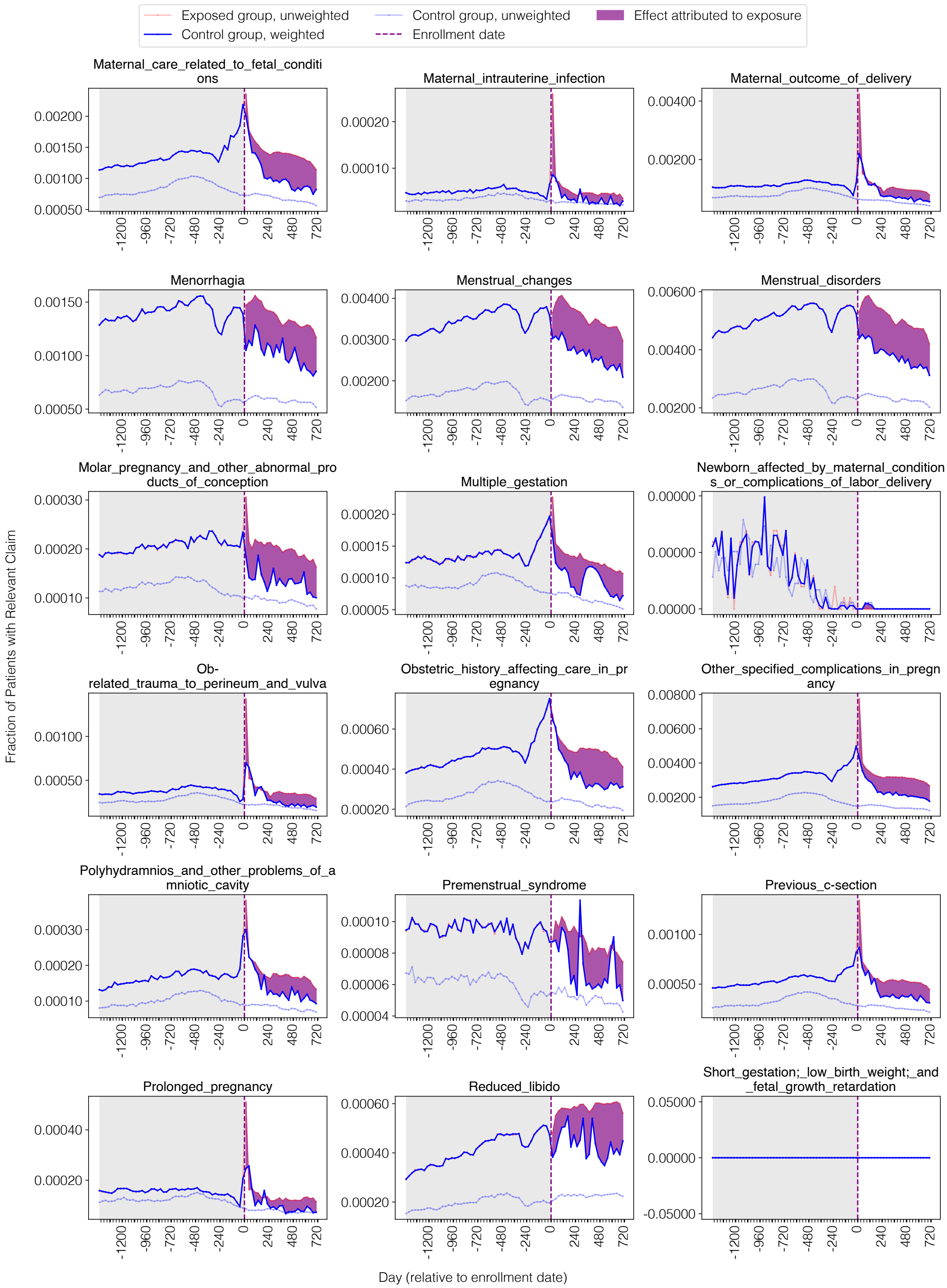


**Fig. S152.** (cont'd) Temporal trends in Reproductive health prevalence. The fraction of patients with relevant claims over time belonging for each outcome in the **unrestricted control design** experiment.

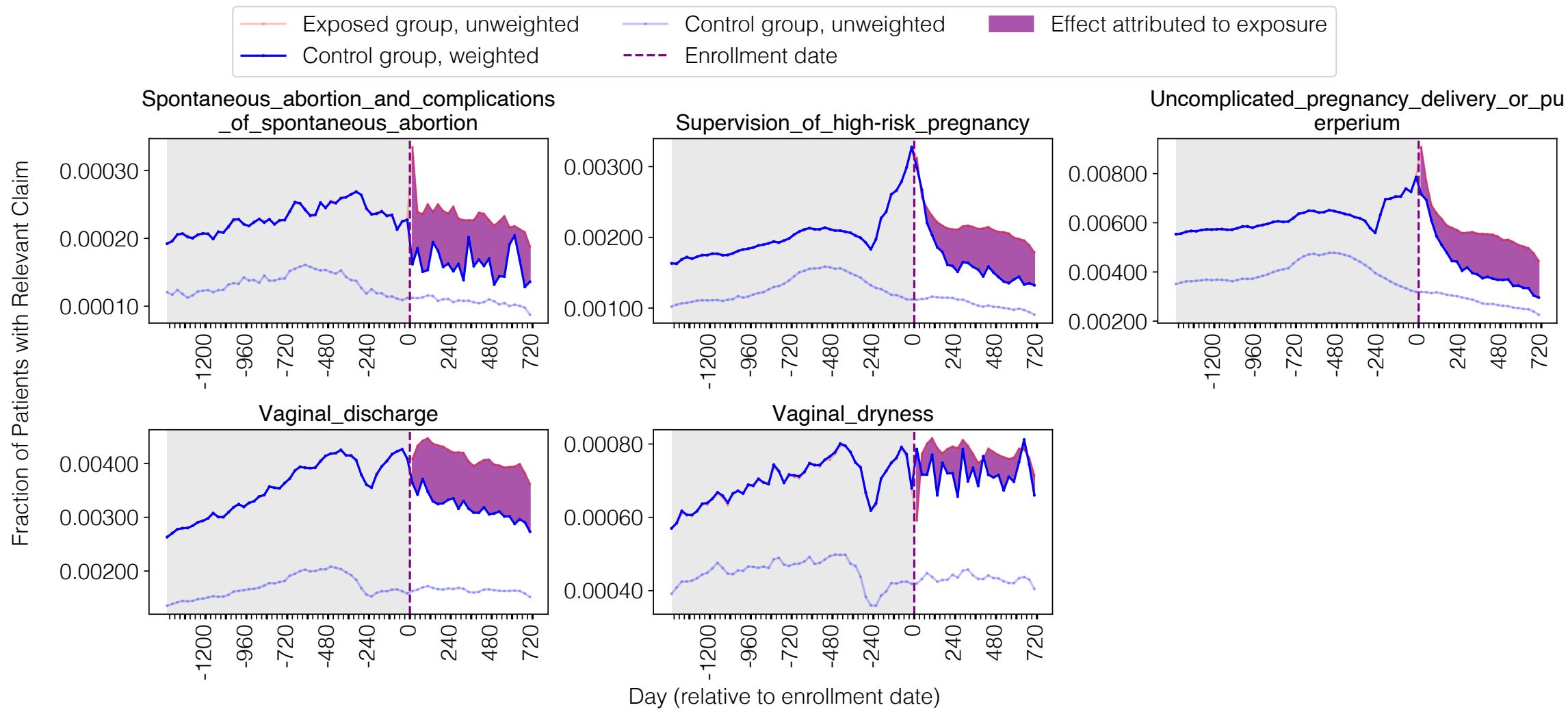


**Fig. S152.** (cont'd) Temporal trends in Reproductive health prevalence. The fraction of patients with relevant claims over time belonging for each outcome in the **unrestricted control design** experiment.

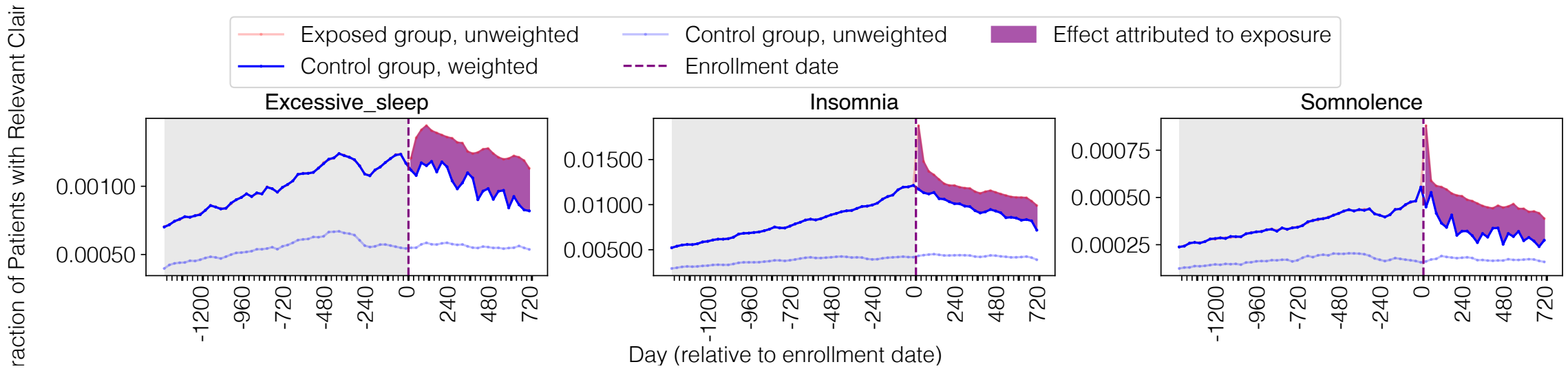


**Fig. S153.** Temporal trends in Sleep prevalence. The fraction of patients with relevant claims over time belonging for each outcome in the **unrestricted control design** experiment.

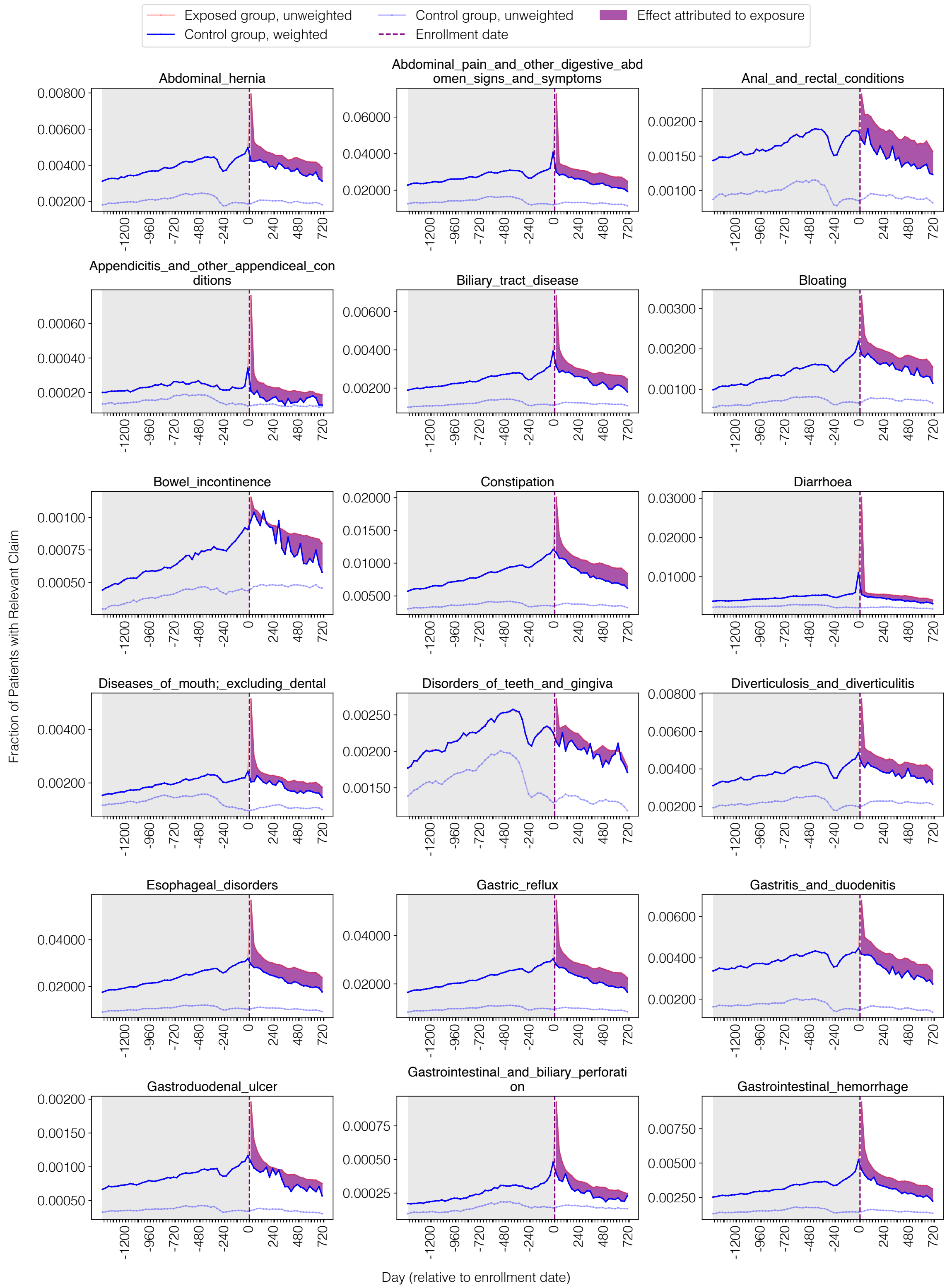


**Fig. S154.** Temporal trends in Stomach and digestion prevalence. The fraction of patients with relevant claims over time belonging for each outcome in the **unrestricted control design** experiment.

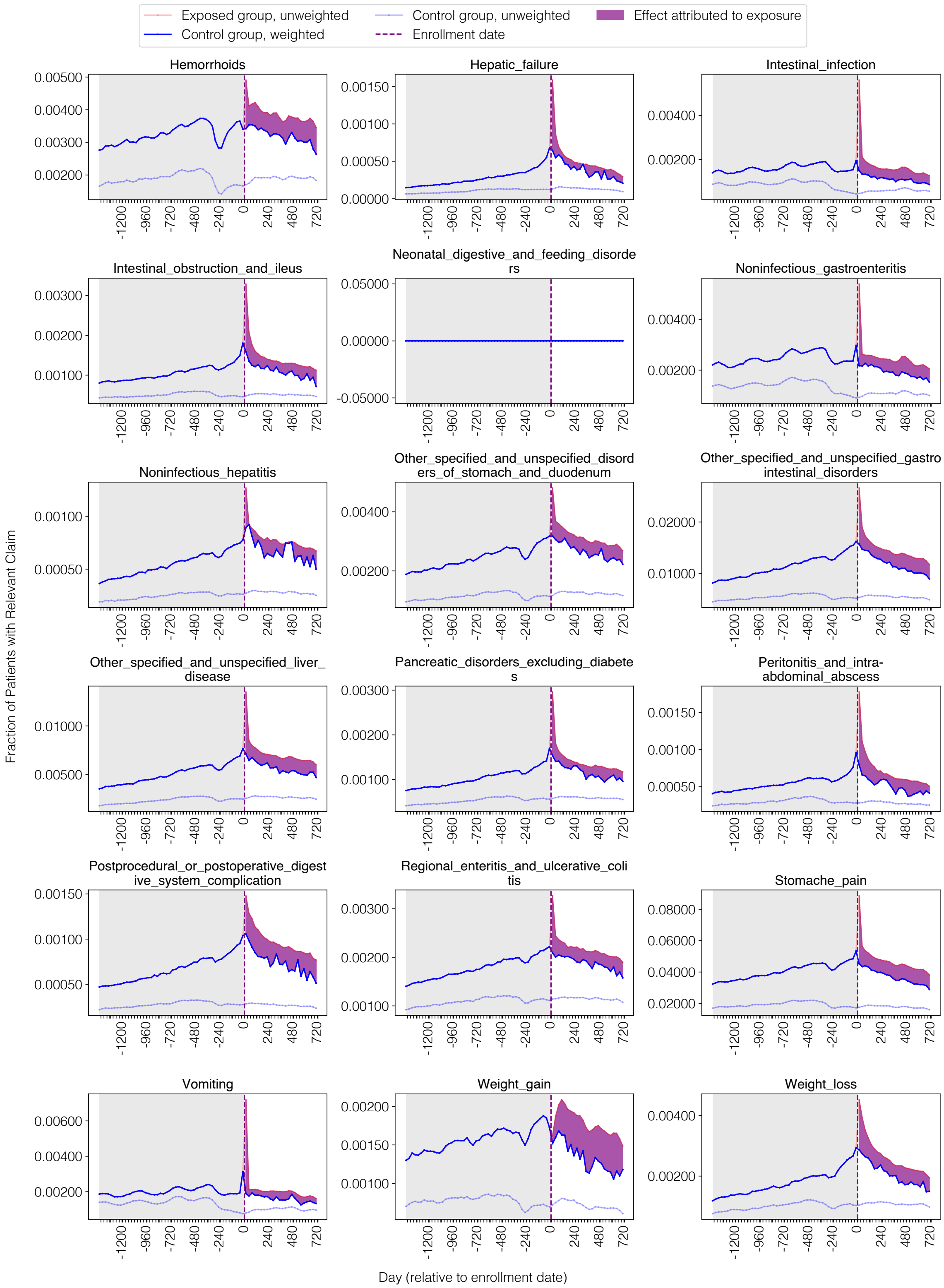


**Fig. S154.** (cont'd) Temporal trends in Stomach and digestion prevalence. The fraction of patients with relevant claims over time belonging for each outcome in the **unrestricted control design** experiment.

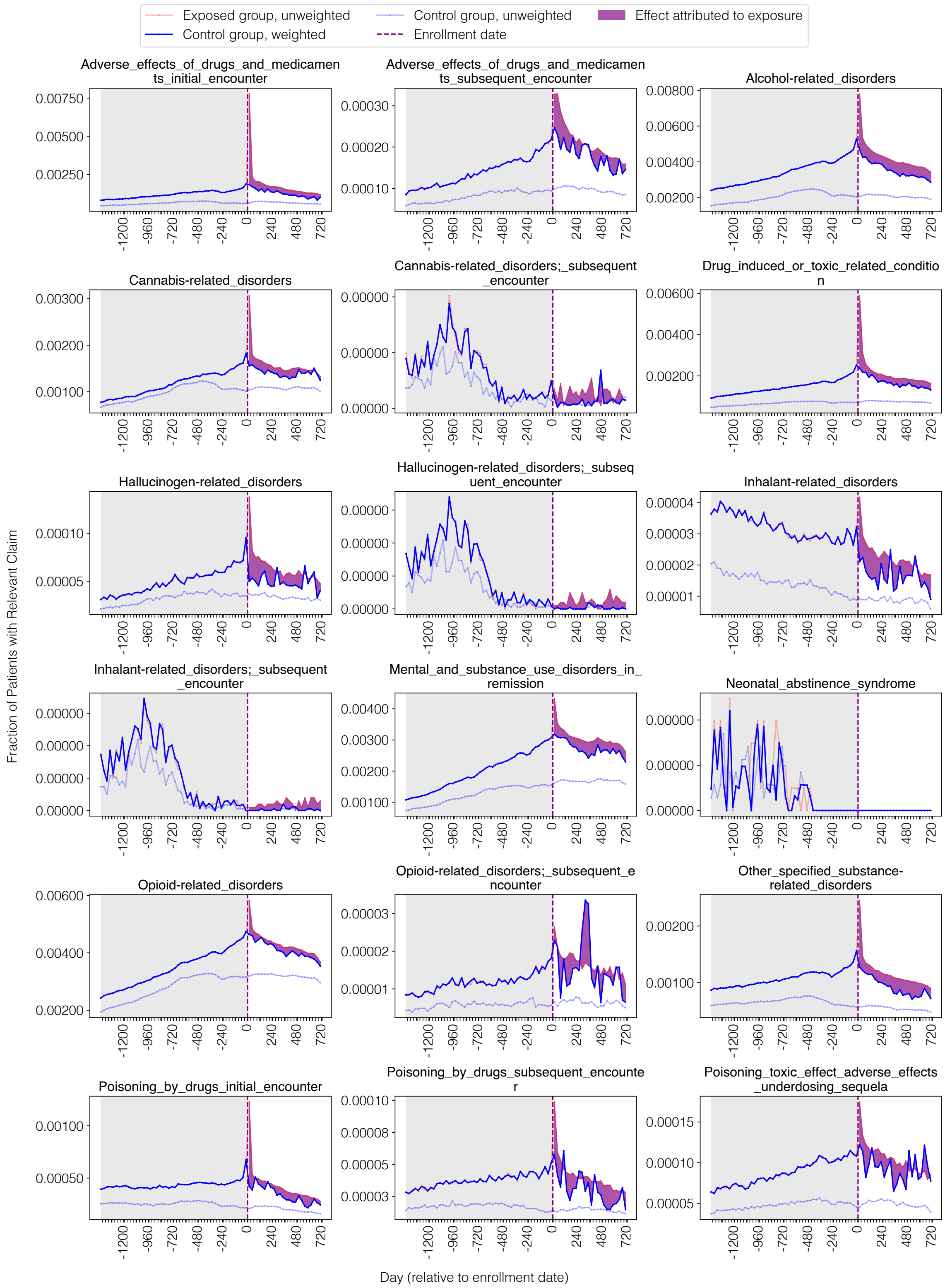


**Fig. S155.** Temporal trends in Substance use prevalence. The fraction of patients with relevant claims over time belonging for each outcome in the **unrestricted control design** experiment.

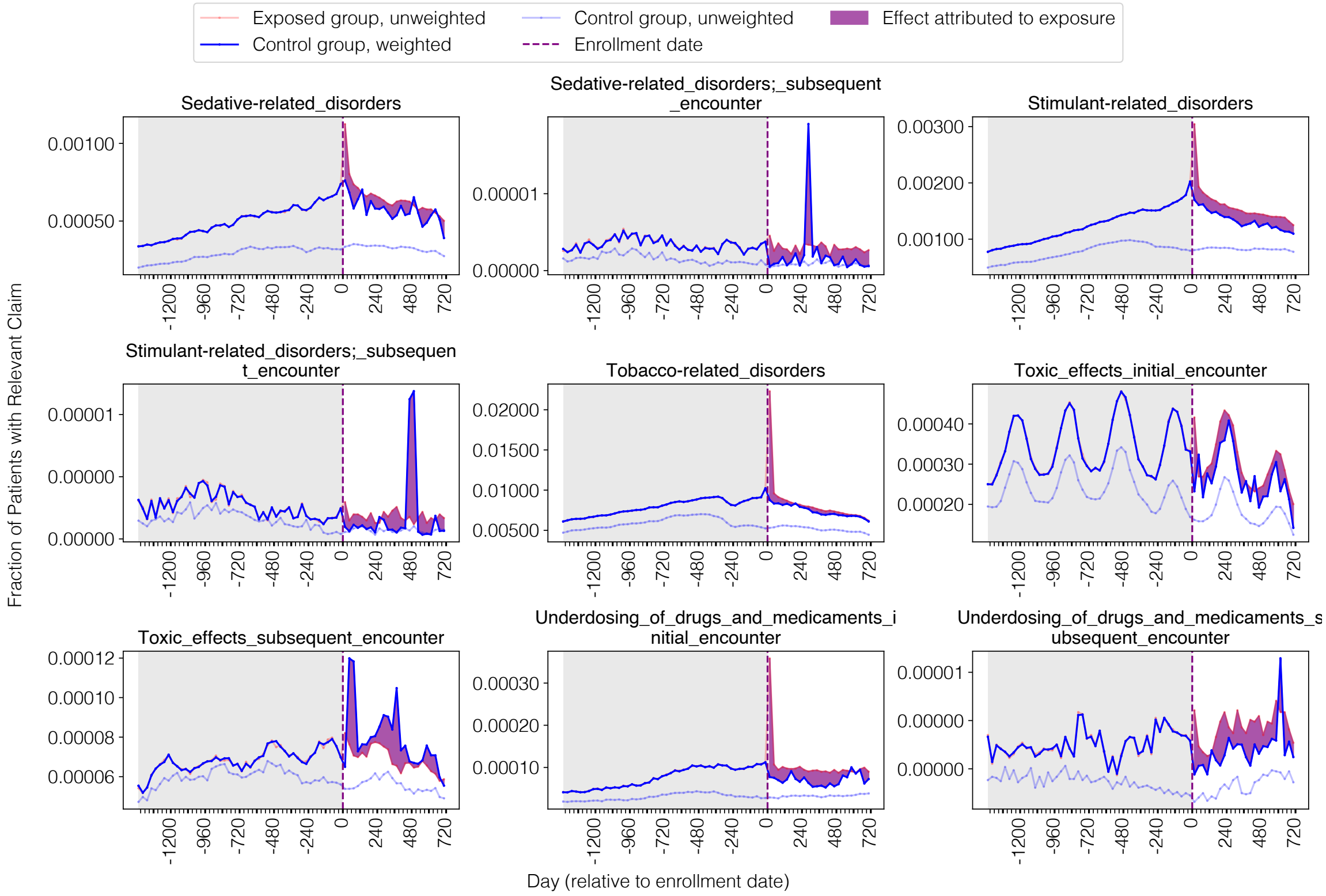


**Fig. S155.** (cont'd) Temporal trends in Substance use prevalence. The fraction of patients with relevant claims over time belonging for each outcome in the **unrestricted control design** experiment.

**Table S9. A comprehensive mapping of diagnosis groups used in the experiments to their corresponding ICD-10 and ICD-9 codes.**

| Diagnosis | ICD-10 | ICD-9 |
|---|---|---|
| Abdominal hernia | K4000,K4001,K4010,K4011,K4020,K4021,K4030,K4031,K4040,K4041<br>K4090,K4091,K4100,K4101,K4110,K4111,K4120,K4121,K4130,K4131<br>K4140,K4141,K4190,K4191,K420,K421,K429,K430,K431,K432,K433<br>K434,K435,K436,K437,K439,K440,K441,K449,K450,K451,K458,K460<br>K461,K469 | 55000,55001,55002,55003,55010,55011,<br>55012,55013,55090,55091<br>55092,55093,55100,55101,55102,55103,<br>5511,55120,55121,55129<br>5513,5518,5519,55200,55201,55202,55203,<br>5521,55220,55221,55229<br>5523,5528,5529,55300,55301,55302,55303,<br>5531,55320,55321,55329<br>5533,5538,5539 |
| Abdominal pain and other digestive abdomen signs and symptoms | G43D0,G43D1,R100,R1010,R1011,R1012,R1013,R102,R1030,R1031<br>R1032,R1033,R10811,R10812,R10813,R10814,R10815,R10816,R10817<br>R10819,R10821,R10822,R10823,R10824,R10825,R10826,R10827<br>R10829,R1083,R1084,R109,R12,R130,R140,R141,R142,R143,R150<br>R151,R152,R159,R160,R161,R162,R17,R180,R188,R1900,R1901,R1902<br>R1903,R1904,R1905,R1906,R1907,R1909,R1911,R1912,R1915,R192<br>R1930,R1931,R1932,R1933,R1934,R1935,R1936,R1937,R194,R195<br>R196,R197,R198 | 34620,34621,56882,6089,6259,7824,78499,<br>7871,78720,7873,7874<br>7875,78760,78761,78762,78763,7877,<br>78791,78799,78900,78901<br>78902,78903,78904,78905,78906,78907,<br>78909,7891,7892,78930<br>78931,78932,78933,78934,78935,78936,<br>78937,78939,78940,78941<br>78942,78943,78944,78945,78946,78947,<br>78949,78951,78959,78960<br>78961,78962,78963,78964,78965,78966,<br>78967,78969,7897,7899<br>7921 |
| Abnormal findings related to substance use | R780,R781,R782,R783,R784,R785,R786 | 7903,79099 |
| Abnormal findings without diagnosis | R700,R701,R710,R718,R7301,R7302,R7303,R7309,R739,R740,R7401<br>R7402,R748,R749,R75,R760,R7611,R7612,R768,R769,R770,R771,R772<br>R778,R779,R7871,R7879,R7881,R7889,R789,R790,R791,R7981,R7982<br>R7983,R7989,R799,R81,R830,R831,R832,R833,R834,R835,R836,R838<br>R839,R840,R841,R842,R843,R844,R845,R846,R847,R848,R849,R850<br>R851,R852,R853,R854,R855,R85610,R85611,R85612,R85613,R85614<br>R85615,R85616,R85618,R85619,R8569,R857,R8581,R8582,R8589,R859<br>R860,R861,R862,R863,R864,R865,R866,R867,R868,R869,R870,R871<br>R872,R873,R874,R875,R87610,R87611,R87612,R87613,R87614,R87615<br>R87616,R87618,R87619,R87620,R87621,R87622,R87623,R87624<br>R87625,R87628,R87629,R8769,R877,R87810,R87811,R87820,R87821<br>R8789,R879,R880,R888,R890,R891,R892,R893,R894,R895,R896,R897<br>R898,R899,R900,R9081,R9082,R9089,R911,R918,R920,R921,R922<br>R928,R930,R931,R932,R933,R934,R9341,R93421,R93422,R93429<br>R9349,R935,R936,R937,R938,R93811,R93812,R93813,R93819,R9389<br>R939,R9401,R9402,R9409,R94110,R94111,R94112,R94113,R94118<br>R94120,R94121,R94128,R94130,R94131,R94138,R942,R9430,R9431<br>R9439,R944,R945,R946,R947,R948,R970,R971,R972,R9720,R9721<br>R978 | 77183,7842,79001,79009,7901,79021,<br>79022,79029,7904,7905,7906<br>7907,79091,79092,79093,79095,79099,<br>7915,7920,7922,7924,7925<br>7929,7930,79311,79319,7932,7933,7934,<br>7935,7936,7937,79380<br>79381,79382,79389,79391,79399,79400,<br>79401,79402,79409,79410<br>79411,79412,79413,79414,79415,79416,<br>79417,79419,7942,79430<br>79431,79439,7944,7945,7946,7947,7948,<br>7949,79501,79502,79503<br>79504,79505,79506,79507,79508,79509,<br>79510,79511,79512,79513<br>79514,79515,79516,79518,79519,7952,<br>79539,7954,79551,79552<br>7956,79571,79579,79581,79582,79589,<br>7960,79670,79671,79672<br>79673,79674,79675,79676,79677,79678,<br>79679 |
| Acquired absence of limb or organ | Z89011,Z89012,Z89019,Z89021,Z89022,Z89029,Z89111,Z89112<br>Z89119,Z89121,Z89122,Z89129,Z89201,Z89202,Z89209,Z89211<br>Z89212,Z89219,Z89221,Z89222,Z89229,Z89231,Z89232,Z89239<br>Z89411,Z89412,Z89419,Z89421,Z89422,Z89429,Z89431,Z89432<br>Z89439,Z89441,Z89442,Z89449,Z89511,Z89512,Z89519,Z89521<br>Z89522,Z89529,Z89611,Z89612,Z89619,Z89621,Z89622,Z89629,Z899<br>Z9001,Z9002,Z9009,Z9010,Z9011,Z9012,Z9013,Z902,Z903,Z90410<br>Z90411,Z9049,Z905,Z906,Z90710,Z90711,Z90712,Z90721,Z90722<br>Z9079,Z9081,Z9089 | V4571,V4572,V4573,V4574,V4575,<br>V4576,V4577,V4578,V4579,V4960<br>V4961,V4962,V4963,V4964,V4965,<br>V4966,V4967,V4970,V4971,V4972<br>V4973,V4974,V4975,V4976,V4977,<br>V8801,V8802,V8803,V8811,V8812<br>V8821,V8822 |
| Acquired deformities (excluding foot) | M20001,M20002,M20009,M20011,M20012,M20019,M20021,M20022<br>M20029,M20031,M20032,M20039,M20091,M20092,M20099,M2100,M21021<br>M21022,M21029,M21051,M21052,M21059,M21061,M21062,M21069,M2110<br>M21121,M21122,M21129,M21151,M21152,M21159,M21161,M21162<br>M21169,M2120,M21211,M21212,M21219,M21221,M21222,M21229,M21231<br>M21232,M21239,M21241,M21242,M21249,M21251,M21252,M21259<br>M21261,M21262,M21269,M21271,M21272,M21279,M21331,M21332<br>M21339,M21511,M21512,M21519,M21521,M21522,M21529,M2170,M21721<br>M21722,M21729,M21731,M21732,M21733,M21734,M21739,M21751<br>M21752,M21759,M21761,M21762,M21763,M21764,M21769,M2180,M21821 | 71840,71841,71842,71843,71844,71845,<br>71846,71847,71848,71849<br>73600,73601,73602,73605,73606,73607,<br>73609,7361,73620,73621<br>73622,73629,73630,73631,73632,73639,<br>73641,73642,7366,73670<br>73681,73689,7369,7380,73819,7382,7383,<br>7386,7387,7388,7389 |

Continued on next page

Table S9 (continued).

| Diagnosis | ICD-10 | ICD-9 |
|---|---|---|
| | M21822,M21829,M21831,M21832,M21839,M21851,M21852,M21859 M21861,M21862,M21869,M2190,M21921,M21922,M21929,M21931,M21932 M21939,M21941,M21942,M21949,M21951,M21952,M21959,M21961 M21962,M21969,M2450,M24511,M24512,M24519,M24521,M24522,M24529 M24531,M24532,M24539,M24541,M24542,M24549,M24551,M24552 M24559,M24561,M24562,M24569,M24571,M24572,M24573,M2459,M950 M9510,M9511,M9512,M952,M953,M954,M955,M958,M959 | |
| Acquired foot deformities | M2010,M2011,M2012,M2020,M2021,M2022,M2030,M2031,M2032,M2040 M2041,M2042,M205X1,M205X2,M205X9,M2060,M2061,M2062,M21071 M21072,M21079,M21171,M21172,M21179,M21371,M21372,M21379,M2140 M2141,M2142,M21531,M21532,M21539,M21541,M21542,M21549,M21611 M21612,M21619,M21621,M21622,M21629,M216X1,M216X2,M216X9 M24574,M24575,M24576 | 71847,7271,734,7350,7351,7352,7354, 7358,7359,73671,73674 73679 |
| Acute and chronic tonsillitis | J0300,J0301,J0380,J0381,J0390,J0391,J3501,J3502,J3503,J351 J352,J353,J358,J359,J36 | 0340,463,47400,47401,47402,47410,47411, 47412,4742,4748,4749 475 |
| Acute and unspecified renal failure | N170,N171,N172,N178,N179,N19,N990,O904 | 5836,5837,5845,5846,5847,5848,5849,586, 66932,66934,9975 |
| Acute bronchitis | J200,J201,J202,J203,J204,J205,J206,J207,J208,J209,J210,J211 J218,J219,J40 | 04100,0415,04181,0791,0792,0793,0796, 07989,4660,46611,46619 490 |
| Acute hemorrhagic cerebrovascular disease | I6000,I6001,I6002,I6010,I6011,I6012,I602,I6020,I6021,I6022 I6030,I6031,I6032,I604,I6050,I6051,I6052,I606,I607,I608,I609 I610,I611,I612,I613,I614,I615,I616,I618,I619,I6200,I6201 I6202,I6203,I621,I629 | 430,431,4320,4321,4329 |
| Acute myocardial infarction | I2101,I2102,I2109,I2111,I2119,I2121,I2129,I213,I214,I219 I21A1,I21A9,I220,I221,I222,I228,I229 | 41001,41011,41021,41031,41041,41051, 41061,41071,41081,41091 |
| Acute phlebitis; thrombophlebitis and thromboembolism | I8000,I8001,I8002,I8003,I8010,I8011,I8012,I8013,I80201,I80202 I80203,I80209,I80211,I80212,I80213,I80219,I80221,I80222 I80223,I80229,I80231,I80232,I80233,I80239,I80241,I80242 I80243,I80249,I80251,I80252,I80253,I80259,I80291,I80292 I80293,I80299,I803,I808,I809,I81,I820,I821,I82210,I82220 I82290,I823,I82401,I82402,I82403,I82409,I82411,I82412,I82413 I82419,I82421,I82422,I82423,I82429,I82431,I82432,I82433 I82439,I82441,I82442,I82443,I82449,I82451,I82452,I82453 I82459,I82461,I82462,I82463,I82469,I82491,I82492,I82493 I82499,I824Y1,I824Y2,I824Y3,I824Y9,I824Z1,I824Z2,I824Z3 I824Z9,I82601,I82602,I82603,I82609,I82611,I82612,I82613 I82619,I82621,I82622,I82623,I82629,I82811,I82812,I82813 I82819,I82890,I8290,I82A11,I82A12,I82A13,I82A19,I82B11,I82B12 I82B13,I82B19,I82C11,I82C12,I82C13,I82C19,O2220,O2221,O2222 O2223,O2230,O2231,O2232,O2233,O8681,O870,O871 | 4510,45111,45119,4512,45181,45189,4519, 452,4530,4531,4532 4533,45340,45341,45342,4536,45381, 45382,45383,45384,45385 45386,45387,45389,67032,67034,67120, 67121,67122,67123,67124 67130,67131,67133,67142,67144 |
| Acute posthemorrhagic anemia | D62 | 2851 |
| Acute pulmonary embolism | I2601,I2602,I2609,I2690,I2692,I2693,I2694,I2699 | 4150,41512,41513,41519 |
| Acute rheumatic heart disease | I00,I010,I011,I012,I018,I019,I020,I029 | 390,3910,3911,3912,3918,3919,3920,3929 |
| Adverse effects of drugs and medicaments, initial encounter | T360X5A,T361X5A,T362X5A,T363X5A,T364X5A,T365X5A,T366X5A T367X5A,T368X5A,T3695XA,T370X5A,T371X5A,T372X5A,T373X5A T374X5A,T375X5A,T378X5A,T3795XA,T380X5A,T381X5A,T382X5A T383X5A,T384X5A,T385X5A,T386X5A,T387X5A,T38805A,T38815A T38895A,T38905A,T38995A,T39015A,T39095A,T391X5A,T392X5A T39315A,T39395A,T394X5A,T398X5A,T3995XA,T400X5A,T401X5A T402X5A,T403X5A,T40415A,T40425A,T40495A,T404X5A,T405X5A T40605A,T40695A,T40715A,T40725A,T407X5A,T408X5A,T40905A T40995A,T410X5A,T411X5A,T41205A,T41295A,T413X5A,T4145XA T415X5A,T420X5A,T421X5A,T422X5A,T423X5A,T424X5A,T425X5A T426X5A,T4275XA,T428X5A,T43015A,T43025A,T431X5A,T43205A | 99520,99522,99523,99529,E9300,E9301, E9302,E9303,E9304,E9305 E9306,E9308,E9310,E9314,E9315,E9316, E9317,E9319,E9320,E9321 E9322,E9323,E9324,E9327,E9328,E9329, E9330,E9331,E9334,E9335 E9336,E9337,E9338,E9339,E9340,E9342, E9344,E9345,E9346,E9348 E9349,E9350,E9351,E9352,E9353,E9355, E9356,E9357,E9360,E9361 E9362,E9363,E9364,E9370,E9378,E9379, E9382,E9383,E9384,E9389 E9390,E9391,E9392,E9393,E9394,E9396, E9397,E9398,E9399,E9400 E9401,E9410,E9411,E9412,E9413,E9419, E9420,E9421,E9422,E9424 E9425,E9426,E9427,E9429,E9430,E9431, E9432,E9433,E9434,E9435 E9436,E9438,E9439,E9442,E9444,E9445, E9447,E9450,E9452,E9453 E9454,E9455,E9456,E9457,E9458,E9460, E9461,E9462,E9463,E9464 |

Continued on next page

Table S9 (continued).

| Diagnosis | ICD-10 | ICD-9 |
|---|---|---|
| | T43215A,T43225A,T43295A,T433X5A,T434X5A,T43505A,T43595A T43605A,T43615A,T43625A,T43635A,T43655A,T43695A,T438X5A T4395XA,T440X5A,T441X5A,T442X5A,T443X5A,T444X5A,T445X5A T446X5A,T447X5A,T448X5A,T44905A,T44995A,T450X5A,T451X5A T452X5A,T453X5A,T454X5A,T45515A,T45525A,T45605A,T45615A T45625A,T45695A,T457X5A,T458X5A,T4595XA,T460X5A,T461X5A T462X5A,T463X5A,T464X5A,T465X5A,T466X5A,T467X5A,T468X5A T46905A,T46995A,T470X5A,T471X5A,T472X5A,T473X5A,T474X5A T475X5A,T476X5A,T477X5A,T478X5A,T4795XA,T480X5A,T481X5A T48205A,T48295A,T483X5A,T484X5A,T485X5A,T486X5A,T48905A T48995A,T490X5A,T491X5A,T492X5A,T493X5A,T494X5A,T495X5A T496X5A,T497X5A,T498X5A,T4995XA,T500X5A,T501X5A,T502X5A T503X5A,T504X5A,T505X5A,T506X5A,T507X5A,T508X5A,T50905A T50915A,T50995A,T50A15A,T50A25A,T50A95A,T50B15A,T50B95A T50Z15A,T50Z95A | E9465,E9466,E9467,E9468,E9469,E9470, E9472,E9478,E9479,E9486 E9488,E9489,E9490,E9496,E9499 |
| Adverse effects of drugs and medicaments, subsequent encounter | T360X5D,T361X5D,T362X5D,T363X5D,T364X5D,T365X5D,T366X5D T367X5D,T368X5D,T3695XD,T370X5D,T371X5D,T372X5D,T373X5D T374X5D,T375X5D,T378X5D,T3795XD,T380X5D,T381X5D,T382X5D T383X5D,T384X5D,T385X5D,T386X5D,T387X5D,T38805D,T38815D T38895D,T38905D,T38995D,T39015D,T39095D,T391X5D,T392X5D T39315D,T39395D,T394X5D,T398X5D,T3995XD,T400X5D,T401X5D T402X5D,T403X5D,T40415D,T40425D,T40495D,T404X5D,T405X5D T40605D,T40695D,T40715D,T40725D,T407X5D,T408X5D,T40905D T40995D,T410X5D,T411X5D,T41205D,T41295D,T413X5D,T4145XD T415X5D,T420X5D,T421X5D,T422X5D,T423X5D,T424X5D,T425X5D T426X5D,T4275XD,T428X5D,T43015D,T43025D,T431X5D,T43205D T43215D,T43225D,T43295D,T433X5D,T434X5D,T43505D,T43595D T43605D,T43615D,T43625D,T43635D,T43655D,T43695D,T438X5D T4395XD,T440X5D,T441X5D,T442X5D,T443X5D,T444X5D,T445X5D T446X5D,T447X5D,T448X5D,T44905D,T44995D,T450X5D,T451X5D T452X5D,T453X5D,T454X5D,T45515D,T45525D,T45605D,T45615D T45625D,T45695D,T457X5D,T458X5D,T4595XD,T460X5D,T461X5D T462X5D,T463X5D,T464X5D,T465X5D,T466X5D,T467X5D,T468X5D T46905D,T46995D,T470X5D,T471X5D,T472X5D,T473X5D,T474X5D T475X5D,T476X5D,T477X5D,T478X5D,T4795XD,T480X5D,T481X5D T48205D,T48295D,T483X5D,T484X5D,T485X5D,T486X5D,T48905D T48995D,T490X5D,T491X5D,T492X5D,T493X5D,T494X5D,T495X5D T496X5D,T497X5D,T498X5D,T4995XD,T500X5D,T501X5D,T502X5D T503X5D,T504X5D,T505X5D,T506X5D,T507X5D,T508X5D,T50905D T50915D,T50995D,T50A15D,T50A25D,T50A95D,T50B15D,T50B95D T50Z15D,T50Z95D | V5889 |
| Alcohol-related disorders | F1010,F10120,F10121,F10129,F10130,F10131,F10132,F10139,F1014 F10150,F10151,F10159,F10180,F10181,F10182,F10188,F1019,F1020 F10220,F10221,F10229,F10230,F10231,F10232,F10239,F1024,F10250 F10251,F10259,F1026,F1027,F10280,F10281,F10282,F10288,F1029 F1090,F1091,F10920,F10921,F10929,F10930,F10931,F10932,F10939 F1094,F10950,F10951,F10959,F1096,F1097,F10980,F10981,F10982 F10988,F1099,G312,G621,I426,K2920,K2921,K700,K7010,K7011,K702 K7030,K7031,K7040,K7041,K709,O354XX0,O354XX1,O354XX2,O354XX3 O354XX4,O354XX5,O354XX9,O99310,O99311,O99312,O99313,O99314 O99315 | 2910,2911,2912,2913,2914,2915,29181, 29182,29189,2919,30300 30301,30302,30303,30390,30391,30392, 30500,30501,30502,30503 3317,3575,4255,53530,53531,5710,5711, 5712,5713,5722,64840 64841,64843,64844,65541,65543 |
| Allergic reactions | B4481,H01111,H01112,H01113,H01114,H01115,H01116,H01119,H01131 H01132,H01133,H01134,H01135,H01136,H01139,H1045,H65111,H65112 H65113,H65114,H65115,H65116,H65117,H65119,H65411,H65412 H65413,H65419,J301,J302,J305,J3081,J3089,J309,K0855,K522 K5221,K5222,K5229,K9041,L200,L2081,L2082,L2083,L2084,L2089 L209,L230,L231,L232,L233,L234,L235,L236,L237,L2381,L2389,L239 L270,L271,L272,L278,L279,L300,L302,L308,L309,L500,L501,L502 L503,L504,L505,L506,L508,L509,L511,L512,L513,L518,L519,L560 L561,L562,L563,L564,M364,T7800XA,T7801XA,T7802XA,T7803XA T7804XA,T7805XA,T7806XA,T7807XA,T7808XA,T7809XA,T781XXA T782XXA,T783XXA,T7840XA,T7841XA,T7849XA,T788XXA | 37214,37331,37332,38102,38103,38104, 38105,38106,3813,4770 4771,4772,4778,4779,5186,52566,5583, 69012,6918,6923,6924,6925 6926,69272,69281,69282,69283,69284, 69289,6929,6930,6931,6938 6939,69510,69511,69512,69513,69514, 69515,69519,7080,7081,7082 7083,7084,7085,7088,7089,7136,78791, 9889,9950,9951,99521,9953 99560,99561,99562,99563,99564,99565, 99566,99567,99568,99569 9957,99589 |
| Allergic reactions, subsequent encounter | T7800XD,T7801XD,T7802XD,T7803XD,T7804XD,T7805XD,T7806XD T7807XD,T7808XD,T7809XD,T781XXD,T782XXD,T783XXD,T7840XD T7841XD,T7849XD,T788XXD | V5889 |
| Amputation of a limb, initial encounter | S48011A,S48012A,S48019A,S48021A,S48022A,S48029A,S48111A S48112A,S48119A,S48121A,S48122A,S48129A,S48911A,S48912A S48919A,S48921A,S48922A,S48929A,S58011A,S58012A,S58019A S58021A,S58022A,S58029A,S58111A,S58112A,S58119A,S58121A S58122A,S58129A,S58911A,S58912A,S58919A,S58921A,S58922A | 8850,8860,8870,8872,8874,8950,8960, 8970,8972,8974 |

Continued on next page

 

Table S9 (continued).

| Diagnosis | ICD-10 | ICD-9 |
|---|---|---|
| | S58929A,S68011A,S68012A,S68019A,S68021A,S68022A,S68029A S68110A,S68111A,S68112A,S68113A,S68114A,S68115A,S68116A S68117A,S68118A,S68119A,S68120A,S68121A,S68122A,S68123A S68124A,S68125A,S68126A,S68127A,S68128A,S68129A,S68411A S68412A,S68419A,S68421A,S68422A,S68429A,S68511A,S68512A S68519A,S68521A,S68522A,S68529A,S68610A,S68611A,S68612A S68613A,S68614A,S68615A,S68616A,S68617A,S68618A,S68619A S68620A,S68621A,S68622A,S68623A,S68624A,S68625A,S68626A S68627A,S68628A,S68629A,S68711A,S68712A,S68719A,S68721A S68722A,S68729A,S78011A,S78012A,S78019A,S78021A,S78022A S78029A,S78111A,S78112A,S78119A,S78121A,S78122A,S78129A S78911A,S78912A,S78919A,S78921A,S78922A,S78929A,S88011A S88012A,S88019A,S88021A,S88022A,S88029A,S88111A,S88112A S88119A,S88121A,S88122A,S88129A,S88911A,S88912A,S88919A S88921A,S88922A,S88929A,S98011A,S98012A,S98019A,S98021A S98022A,S98029A,S98111A,S98112A,S98119A,S98121A,S98122A S98129A,S98131A,S98132A,S98139A,S98141A,S98142A,S98149A S98211A,S98212A,S98219A,S98221A,S98222A,S98229A,S98311A S98312A,S98319A,S98321A,S98322A,S98329A,S98911A,S98912A S98919A,S98921A,S98922A,S98929A | |
| Amputation of a limb, subsequent encounter | S48011D,S48012D,S48019D,S48021D,S48022D,S48029D,S48111D S48112D,S48119D,S48121D,S48122D,S48129D,S48911D,S48912D S48919D,S48921D,S48922D,S48929D,S58011D,S58012D,S58019D S58021D,S58022D,S58029D,S58111D,S58112D,S58119D,S58121D S58122D,S58129D,S58911D,S58912D,S58919D,S58921D,S58922D S58929D,S68011D,S68012D,S68019D,S68021D,S68022D,S68029D S68110D,S68111D,S68112D,S68113D,S68114D,S68115D,S68116D S68117D,S68118D,S68119D,S68120D,S68121D,S68122D,S68123D S68124D,S68125D,S68126D,S68127D,S68128D,S68129D,S68411D S68412D,S68419D,S68421D,S68422D,S68429D,S68511D,S68512D S68519D,S68521D,S68522D,S68529D,S68610D,S68611D,S68612D S68613D,S68614D,S68615D,S68616D,S68617D,S68618D,S68619D S68620D,S68621D,S68622D,S68623D,S68624D,S68625D,S68626D S68627D,S68628D,S68629D,S68711D,S68712D,S68719D,S68721D S68722D,S68729D,S78011D,S78012D,S78019D,S78021D,S78022D S78029D,S78111D,S78112D,S78119D,S78121D,S78122D,S78129D S78911D,S78912D,S78919D,S78921D,S78922D,S78929D,S88011D S88012D,S88019D,S88021D,S88022D,S88029D,S88111D,S88112D S88119D,S88121D,S88122D,S88129D,S88911D,S88912D,S88919D S88921D,S88922D,S88929D,S98011D,S98012D,S98019D,S98021D S98022D,S98029D,S98111D,S98112D,S98119D,S98121D,S98122D S98129D,S98131D,S98132D,S98139D,S98141D,S98142D,S98149D S98211D,S98212D,S98219D,S98221D,S98222D,S98229D,S98311D S98312D,S98319D,S98321D,S98322D,S98329D,S98911D,S98912D S98919D,S98921D,S98922D,S98929D | V5889 |
| Amputation of other body parts, initial encounter | S08111A,S08112A,S08119A,S08121A,S08122A,S08129A,S08811A S08812A,S0889XA,S281XXA,S28211A,S28212A,S28219A,S28221A S28222A,S28229A,S38211A,S38212A,S38221A,S38222A,S38231A S38232A,S383XXA | 87201,87329,8738,8750,8780,8782,8788, 8790,8798 |
| Amputation of other body parts, subsequent encounter | S08111D,S08112D,S08119D,S08121D,S08122D,S08129D,S08811D S08812D,S0889XD,S281XXD,S28211D,S28212D,S28219D,S28221D S28222D,S28229D,S38211D,S38212D,S38221D,S38222D,S38231D S38232D,S383XXD | V5889 |
| Anal and rectal conditions | A511,A546,A563,A601,A609,A630,K594,K600,K601,K602,K603,K604 K605,K610,K611,K612,K613,K6131,K6139,K614,K615,K620,K621,K622 K623,K624,K626,K627,K6281,K6282,K6289,K629 | 05410,05419,07811,0911,0987,09952,5646, 5650,5651,566,5690 5691,5692,56941,56942,56943,56944, 56949 |
| Anesthesia complications during pregnancy | O29011,O29012,O29013,O29019,O29021,O29022,O29023,O29029 O29091,O29092,O29093,O29099,O29111,O29112,O29113,O29119 O29121,O29122,O29123,O29129,O29191,O29192,O29193,O29199 O29211,O29212,O29213,O29219,O29291,O29292,O29293,O29299 O293X1,O293X2,O293X3,O293X9,O2940,O2941,O2942,O2943,O295X1 O295X2,O295X3,O295X9,O2960,O2961,O2962,O2963,O298X1,O298X2 O298X3,O298X9,O2990,O2991,O2992,O2993,O740,O741,O742,O743 O744,O745,O746,O747,O748,O749 | 64680,64681,64683,66801,66802,66811, 66812,66821,66822,66881 66882,66891,66892 |
| Antenatal screening | Z36,Z360,Z361,Z362,Z363,Z364,Z365,Z3681,Z3682,Z3683,Z3684 Z3685,Z3686,Z3687,Z3688,Z3689,Z368A,Z369 | V289 |
| Anxiety and fear-related disorders | F064,F10180,F10280,F10980,F12180,F12280,F12980,F13180,F13280 F13980,F14180,F14280,F14980,F15180,F15280,F15980,F16180 F16280,F16980,F18180,F18280,F18980,F19180,F19280,F19980,F4000 F4001,F4002,F4010,F4011,F40210,F40218,F40220,F40228,F40230 F40231,F40232,F40233,F40240,F40241,F40242,F40243,F40248 F40290,F40291,F40298,F408,F409,F410,F411,F413,F418,F419,F930 | 29189,29289,29384,30000,30001,30002, 30009,30020,30021,30022 30023,30029,30410,30420,30430,30440, 30450,30460,30921,31323 |

Continued on next page

Table S9 (continued).

| Diagnosis | ICD-10 | ICD-9 |
|---|---|---|
| | F940 | |
| Aortic and peripheral<br>arterial embolism or<br>thrombosis | I7401,I7409,I7410,I7411,I7419,I742,I743,I744,I745,I748,I749<br>I75011,I75012,I75013,I75019,I75021,I75022,I75023,I75029,I7581<br>I7589,I76 | 44401,44409,4441,44421,44422,44481,<br>44489,4449,44501,44502<br>44581,44589,449 |
| Aortic; peripheral;<br>and visceral artery<br>aneurysms | A5201,I711,I7110,I7111,I7112,I7113,I712,I7120,I7121,I7122<br>I7123,I713,I7130,I7131,I7132,I7133,I714,I7140,I7141,I7142<br>I7143,I715,I7150,I7151,I7152,I716,I7160,I7161,I7162,I718,I719<br>I720,I721,I722,I723,I724,I725,I726,I728,I729,I790 | 0930,4411,4412,4413,4414,4415,4416,<br>4417,4419,4420,4421,4422<br>4423,44281,44282,44283,44289,4429 |
| Aplastic anemia | D600,D601,D608,D609,D6101,D6109,D611,D612,D613,D61810,D61811<br>D61818,D6182,D6189,D619,D630,D631,D638,D640,D641,D642,D643<br>D644,D6481,D6489,D649 | 28401,28409,28411,28412,28419,2842,<br>28481,28489,2849,2850<br>28521,28522,28529,2853,2858,2859 |
| Appendicitis and<br>other appendiceal<br>conditions | K352,K3520,K3521,K353,K3530,K3531,K3532,K3533,K3580,K3589<br>K35890,K35891,K36,K37,K380,K381,K382,K383,K388,K389 | 5400,5401,5409,541,542,5430,5439 |
| Arterial dissections | I2542,I670,I7100,I7101,I71010,I71011,I71012,I71019,I7102<br>I7103,I7770,I7771,I7772,I7773,I7774,I7775,I7776,I7777,I7779 | 41412,44100,44101,44102,44103,44321,<br>44322,44323,44324,44329 |
| Aseptic necrosis and<br>osteonecrosis | L97104,L97114,L97124,L97204,L97214,L97224,L97304,L97314<br>L97324,L97404,L97414,L97424,L97504,L97514,L97524,L97804<br>L97814,L97824,L97904,L97914,L97924,L98414,L98424,L98494,M8700<br>M87011,M87012,M87019,M87021,M87022,M87029,M87031,M87032<br>M87033,M87034,M87035,M87036,M87037,M87038,M87039,M87041<br>M87042,M87043,M87044,M87045,M87046,M87050,M87051,M87052<br>M87059,M87061,M87062,M87063,M87064,M87065,M87066,M87071<br>M87072,M87073,M87074,M87075,M87076,M87077,M87078,M87079,M8708<br>M8709,M8710,M87111,M87112,M87119,M87121,M87122,M87129,M87131<br>M87132,M87133,M87134,M87135,M87136,M87137,M87138,M87139<br>M87141,M87142,M87143,M87144,M87145,M87146,M87150,M87151<br>M87152,M87159,M87161,M87162,M87163,M87164,M87165,M87166<br>M87171,M87172,M87173,M87174,M87175,M87176,M87177,M87178<br>M87179,M87180,M87188,M8719,M8720,M87211,M87212,M87219,M87221<br>M87222,M87229,M87231,M87232,M87233,M87234,M87235,M87236<br>M87237,M87238,M87239,M87241,M87242,M87243,M87244,M87245<br>M87246,M87250,M87251,M87252,M87256,M87261,M87262,M87263<br>M87264,M87265,M87266,M87271,M87272,M87273,M87274,M87275<br>M87276,M87277,M87278,M87279,M8728,M8729,M8730,M87311,M87312<br>M87319,M87321,M87322,M87329,M87331,M87332,M87333,M87334<br>M87335,M87336,M87337,M87338,M87339,M87341,M87342,M87343<br>M87344,M87345,M87346,M87350,M87351,M87352,M87353,M87361<br>M87362,M87363,M87364,M87365,M87366,M87371,M87372,M87373<br>M87374,M87375,M87376,M87377,M87378,M87379,M8738,M8739,M8780<br>M87811,M87812,M87819,M87821,M87822,M87829,M87831,M87832<br>M87833,M87834,M87835,M87836,M87837,M87838,M87839,M87841<br>M87842,M87843,M87844,M87845,M87849,M87850,M87851,M87852<br>M87859,M87861,M87862,M87863,M87864,M87865,M87869,M87871<br>M87872,M87873,M87874,M87875,M87876,M87877,M87878,M87879,M8788<br>M8789,M879,M9050,M90511,M90512,M90519,M90521,M90522,M90529<br>M90531,M90532,M90539,M90541,M90542,M90549,M90551,M90552<br>M90559,M90561,M90562,M90569,M90571,M90572,M90579,M9058,M9059 | 70710,70711,70712,70713,70714,70715,<br>70719,7078,7079,7318<br>73340,73341,73342,73343,73344,73345,<br>73349 |
| Aspiration<br>pneumonitis | J690,J691,J698,J954,O29011,O29012,O29013,O29019,O740,O8901 | 5070,5071,5078,64680,64681,64683,66801,<br>66802,66804,99739 |
| Asthma | J4520,J4521,J4522,J4530,J4531,J4532,J4540,J4541,J4542,J4550<br>J4551,J4552,J45901,J45902,J45909,J45990,J45991,J45998 | 49300,49301,49302,49310,49311,49312,<br>49381,49382,49390,49391<br>49392 |
| Atypical fracture,<br>initial encounter | M84750A,M84751A,M84752A,M84753A,M84754A,M84755A,M84756A<br>M84757A,M84758A,M84759A | |
| Atypical fracture,<br>subsequent encounter | M84750D,M84750G,M84750K,M84750P,M84751D,M84751G,M84751K<br>M84751P,M84752D,M84752G,M84752K,M84752P,M84753D,M84753G<br>M84753K,M84753P,M84754D,M84754G,M84754K,M84754P,M84755D<br>M84755G,M84755K,M84755P,M84756D,M84756G,M84756K,M84756P<br>M84757D,M84757G,M84757K,M84757P,M84758D,M84758G,M84758K<br>M84758P,M84759D,M84759G,M84759K,M84759P | |
| Autoinflammatory<br>syndromes | M041,M042,M048,M049 | |
| Bacterial infections | A000,A001,A009,A0100,A0101,A0102,A0103,A0104,A0105,A0109,A011<br>A012,A013,A014,A020,A021,A0220,A0221,A0222,A0223,A0224,A0225 | 0010,0011,0019,0020,0021,0022,0023,<br>0029,0030,0031,00320,00321<br>00322,00323,00324,00329,0038,0039,0040,<br>0041,0042,0043,0048 |

Continued on next page

 

Table S9 (continued).

| Diagnosis | ICD-10 | ICD-9 |
|---|---|---|
| | A0229,A028,A029,A030,A031,A032,A033,A038,A039,A040,A041,A042 | 0049,00800,00801,00802,00803,00804, 00809,0082,00841,00843 |
| | A043,A044,A045,A046,A047,A0471,A0472,A048,A049,A200,A201,A202 | 00844,00845,00849,0085,0200,0201,0202, 0203,0204,0205,0208 |
| | A203,A207,A208,A209,A210,A211,A212,A213,A217,A218,A219,A220 | 0209,0210,0211,0212,0213,0218,0219, 0220,0221,0222,0223,0228 |
| | A221,A222,A227,A228,A229,A230,A231,A232,A233,A238,A239,A240 | 0229,0230,0231,0232,0233,0238,0239,024, 025,0260,0261,0269 |
| | A241,A242,A243,A249,A250,A251,A259,A260,A267,A268,A269,A270 | 0270,0271,0272,0278,0279,0300,0301, 0302,0303,0308,0309,0310 |
| | A2781,A2789,A279,A280,A281,A282,A288,A289,A300,A301,A302,A303 | 0311,0312,0318,0319,0320,0321,0322, 0323,03281,03282,03283 |
| | A304,A305,A308,A309,A310,A311,A312,A318,A319,A320,A3211,A3212 | 03284,03285,03289,0329,0330,0331,0338, 0339,0340,0341,035,0360 |
| | A327,A3281,A3282,A3289,A329,A33,A34,A35,A360,A361,A362,A363 | 0361,0362,0363,03640,03641,03642,03643, 03681,03682,03689,0369 |
| | A3681,A3682,A3683,A3684,A3685,A3686,A3689,A369,A3700,A3701 | 037,0380,03810,03811,03812,03819,0382, 0383,03840,03841,03842 |
| | A3710,A3711,A3780,A3781,A3790,A3791,A380,A381,A388,A389,A390 | 03843,03844,03849,0388,0389,0390,0391, 0392,0393,0398,0399 |
| | A391,A392,A393,A394,A3950,A3951,A3952,A3953,A3981,A3982,A3983 | 0400,0401,04042,04082,04089,04100, 04101,04102,04104,04109 |
| | A3984,A3989,A399,A400,A401,A403,A408,A409,A4101,A4102,A411 | 04110,04111,04112,04119,0412,0413, 04141,04142,04143,04149 |
| | A412,A413,A414,A4150,A4151,A4152,A4153,A4159,A4181,A4189,A419 | 0415,0416,0417,04181,04182,04183,04184, 04186,04189,0419,0739 |
| | A420,A421,A422,A427,A4281,A4282,A4289,A429,A430,A431,A438 | 0760,0761,0769,0770,07798,0783,07888, 07988,07998,0830,0880 |
| | A439,A440,A441,A448,A449,A46,A480,A481,A482,A483,A484,A4852 | 1000,10081,10089,1009,3200,3201,3202, 3203,3207,32082,32089 |
| | A488,A4901,A4902,A491,A492,A493,A498,A499,A70,A710,A711,A719 | 3209,390,3929,4660,481,4820,4821,4822, 48231,48232,48239,48240 |
| | A740,A7481,A7489,A749,A78,B950,B951,B952,B953,B954,B955,B9561 | 48241,48242,48249,48281,48282,48283, 48284,48289,4829,4830 |
| | B9562,B957,B958,B960,B961,B9620,B9621,B9622,B9623,B9629,B963 | 4831,4843,4845,56723,6390,71100,71101, 71102,71103,71104,71105 |
| | B964,B965,B966,B967,B9681,B9682,B9689,D5931,G000,G001,G002 | 71106,71107,71108,71109,7700,7712,7713, 77181,99591 |
| | G003,G008,G009,G01,G042,I00,I029,J020,J0300,J0301,J13,J14 J150,J151,J1520,J15211,J15212,J1529,J153,J154,J155,J156,J157 J158,J159,J160,J200,J201,J202,K652,M0000,M00011,M00012,M00019 M00021,M00022,M00029,M00031,M00032,M00039,M00041,M00042 M00049,M00051,M00052,M00059,M00061,M00062,M00069,M00071 M00072,M00079,M0008,M0009,M0010,M00111,M00112,M00119,M00121 M00122,M00129,M00131,M00132,M00139,M00141,M00142,M00149 M00151,M00152,M00159,M00161,M00162,M00169,M00171,M00172 M00179,M0018,M0019,M0020,M00211,M00212,M00219,M00221,M00222 M00229,M00231,M00232,M00239,M00241,M00242,M00249,M00251 M00252,M00259,M00261,M00262,M00269,M00271,M00272,M00279,M0028 M0029,M0080,M00811,M00812,M00819,M00821,M00822,M00829,M00831 M00832,M00839,M00841,M00842,M00849,M00851,M00852,M00859 M00861,M00862,M00869,M00871,M00872,M00879,M0088,M0089,M009 P0082,P231,P232,P233,P234,P235,P236,P360,P3610,P3619,P362 P3630,P3639,P364,P365,P368,P369,P372 | |
| Benign neoplasms | D100,D101,D102,D1030,D1039,D104,D105,D106,D107,D109,D110,D117 | 20940,20941,20942,20943,20950,20951, 20952,20953,20954,20955 |
| | D119,D120,D121,D122,D123,D124,D125,D126,D127,D128,D129,D130 | 20956,20957,20960,20961,20962,20963, 20964,20965,20966,20967 |
| | D131,D132,D1330,D1339,D134,D135,D136,D137,D139,D140,D141,D142 | 20969,2100,2101,2102,2103,2104,2105, 2106,2107,2108,2109,2110 |
| | D1430,D1431,D1432,D144,D150,D151,D152,D157,D159,D1600,D1601 | 2111,2112,2113,2114,2115,2116,2117, 2118,2119,2120,2121,2122 |
| | D1602,D1610,D1611,D1612,D1620,D1621,D1622,D1630,D1631,D1632 | 2123,2124,2125,2126,2127,2128,2129, 2130,2131,2132,2133,2134 |
| | D164,D165,D166,D167,D168,D169,D170,D171,D1720,D1721,D1722 | 2135,2136,2137,2138,2139,2140,2141, 2142,2143,2144,2148,2149 |
| | D1723,D1724,D1730,D1739,D174,D175,D176,D1771,D1772,D1779,D179 | 2150,2152,2153,2154,2155,2156,2157, 2159,2160,2161,2162,2163 |
| | D1800,D1801,D1802,D1803,D1809,D181,D190,D191,D197,D199,D200 | 2164,2165,2166,2167,2168,2169,217,2180, 2181,2182,2189,2190 |
| | D201,D210,D2110,D2111,D2112,D2120,D2121,D2122,D213,D214,D215 | 2191,2198,2199,220,2210,2211,2212,2218, 2219,2220,2221,2222 |
| | D216,D219,D220,D2210,D2211,D22111,D22112,D2212,D22121,D22122 | 2223,2224,2228,2229,2230,2231,2232, 2233,22381,22389,2239,2240 |
| | D2220,D2221,D2222,D2230,D2239,D224,D225,D2260,D2261,D2262 | 2241,2242,2243,2244,2245,2246,2247, 2248,2249,2250,2251,2252 |
| | D2270,D2271,D2272,D229,D230,D2310,D2311,D23111,D23112,D2312 | 2253,2254,2258,2259,226,2270,2271,2273, 2274,2275,2276,2278 |
| | D23121,D23122,D2320,D2321,D2322,D2330,D2339,D234,D235,D2360 | 2279,22800,22801,22802,22804,22809, 2281,2290,2298,2299 |
| | D2361,D2362,D2370,D2371,D2372,D239,D241,D242,D249,D250,D251 D252,D259,D260,D261,D267,D269,D270,D271,D279,D280,D281,D282 D287,D289,D290,D291,D2920,D2921,D2922,D2930,D2931,D2932,D294 D298,D299,D3000,D3001,D3002,D3010,D3011,D3012,D3020,D3021 D3022,D303,D304,D308,D309,D3100,D3101,D3102,D3110,D3111,D3112 D3120,D3121,D3122,D3130,D3131,D3132,D3140,D3141,D3142,D3150 D3151,D3152,D3160,D3161,D3162,D3190,D3191,D3192,D320,D321 | |

Continued on next page

Table S9 (continued).

| Diagnosis | ICD-10 | ICD-9 |
|---|---|---|
| | D329,D330,D331,D332,D333,D334,D337,D339,D34,D3500,D3501,D3502 D351,D352,D353,D354,D355,D356,D357,D359,D360,D3610,D3611 D3612,D3613,D3614,D3615,D3616,D3617,D367,D369,D3A00,D3A010 D3A011,D3A012,D3A019,D3A020,D3A021,D3A022,D3A023,D3A024 D3A025,D3A026,D3A029,D3A090,D3A091,D3A092,D3A093,D3A094 D3A095,D3A096,D3A098,D3A8,K317,K635 | |
| Benign ovarian cyst | N801,N830,N8300,N8301,N8302,N831,N8310,N8311,N8312,N8320 N83201,N83202,N83209,N8329,N83291,N83292,N83299 | 6171,6200,6201,6202 |
| Biliary tract disease | K563,K8000,K8001,K8010,K8011,K8012,K8013,K8018,K8019,K8020 K8021,K8030,K8031,K8032,K8033,K8034,K8035,K8036,K8037,K8040 K8041,K8042,K8043,K8044,K8045,K8046,K8047,K8050,K8051,K8060 K8061,K8062,K8063,K8064,K8065,K8066,K8067,K8070,K8071,K8080 K8081,K810,K811,K812,K819,K820,K821,K822,K823,K824,K828,K829 K82A1,K82A2,K830,K8301,K8309,K831,K832,K833,K834,K835,K838 K839,K87,K915 | 56031,57400,57401,57410,57411,57420, 57421,57430,57431,57440 57441,57450,57451,57460,57461,57470, 57471,57480,57481,57490 57491,5750,57510,57511,57512,5752,5753, 5754,5755,5756,5758 5759,5760,5761,5762,5763,5764,5765, 5768,5769,5778 |
| Biomechanical lesions | M9900,M9901,M9902,M9903,M9904,M9905,M9906,M9907,M9908,M9909 M9910,M9911,M9912,M9913,M9914,M9915,M9916,M9917,M9918,M9919 M9920,M9921,M9922,M9923,M9924,M9925,M9926,M9927,M9928,M9929 M9930,M9931,M9932,M9933,M9934,M9935,M9936,M9937,M9938,M9939 M9940,M9941,M9942,M9943,M9944,M9945,M9946,M9947,M9948,M9949 M9950,M9951,M9952,M9953,M9954,M9955,M9956,M9957,M9958,M9959 M9960,M9961,M9962,M9963,M9964,M9965,M9966,M9967,M9968,M9969 M9970,M9971,M9972,M9973,M9974,M9975,M9976,M9977,M9978,M9979 M9980,M9981,M9982,M9983,M9984,M9985,M9986,M9987,M9988,M9989 M999 | 7230,72401,72402,72409,73819,7382,7383, 7385,7386,7388,7389 7390,7391,7392,7393,7394,7395,7396, 7397,7398,7399,83900,83920 83921,83942,83949,83961,83969 |
| Bipolar and related disorders | F0633,F0634,F3010,F3011,F3012,F3013,F302,F303,F308,F309,F310 F3110,F3111,F3112,F3113,F312,F3130,F3131,F3132,F314,F315 F3160,F3161,F3162,F3163,F3164,F3171,F3173,F3175,F3177,F3181 F3189,F319,F340 | 29383,29600,29601,29602,29603,29604, 29605,29640,29641,29642 29643,29644,29645,29650,29651,29652, 29653,29654,29655,29660 29661,29662,29663,29664,29665,2967, 29680,29681,29689,30110 30113 |
| Birth trauma | P100,P101,P102,P103,P104,P108,P109,P110,P111,P112,P113,P114 P115,P119,P120,P121,P122,P123,P124,P1281,P1289,P129,P130,P131 P132,P133,P134,P138,P139,P140,P141,P142,P143,P148,P149,P150 P151,P152,P153,P154,P155,P156,P158,P159 | 7670,76711,76719,7672,7673,7674,7675, 7676,7677,7678,7679 77210,7722 |
| Blindness and vision defects | H53001,H53002,H53003,H53009,H53011,H53012,H53013,H53019 H53021,H53022,H53023,H53029,H53031,H53032,H53033,H53039 H53041,H53042,H53043,H53049,H5310,H5311,H53121,H53122,H53123 H53129,H53131,H53132,H53133,H53139,H53141,H53142,H53143 H53149,H5315,H5316,H5319,H532,H5330,H5331,H5332,H5333,H5334 H5340,H53411,H53412,H53413,H53419,H53421,H53422,H53423,H53429 H53431,H53432,H53433,H53439,H53451,H53452,H53453,H53459 H53461,H53462,H53469,H5347,H53481,H53482,H53483,H53489,H5350 H5351,H5352,H5353,H5354,H5355,H5359,H5360,H5361,H5362,H5363 H5369,H5371,H5372,H538,H539,H540,H540X33,H540X34,H540X35 H540X43,H540X44,H540X45,H540X53,H540X54,H540X55,H5410,H5411 H541131,H541132,H541141,H541142,H541151,H541152,H5412,H541213 H541214,H541215,H541223,H541224,H541225,H542,H542X11,H542X12 H542X21,H542X22,H543,H5440,H5441,H54413A,H54414A,H54415A H5442,H5442A3,H5442A4,H5442A5,H5450,H5451,H54511A,H54512A H5452,H5452A1,H5452A2,H5460,H5461,H5462,H547,H548,Z4531 | 36800,36801,36802,36803,36810,36811, 36812,36813,36814,36815 36816,3682,36830,36831,36832,36833, 36834,36840,36841,36842 36843,36844,36845,36846,36847,36851, 36852,36853,36854,36855 36859,36860,36861,36862,36863,36869, 3688,3689,36900,36901 36910,36920,36925,3693,3694,36960, 36963,36966,36969,36970 36971,3698,3699,V410,V5309 |
| Bone cancer | C4000,C4001,C4002,C4010,C4011,C4012,C4020,C4021,C4022,C4030 C4031,C4032,C4080,C4081,C4082,C4090,C4091,C4092,C410,C411 C412,C413,C414,C419 | 1700,1701,1702,1703,1704,1705,1706, 1707,1708,1709 |
| Breast cancer - all other types | C50011,C50012,C50019,C50021,C50022,C50029,C50111,C50112 C50119,C50121,C50122,C50129,C50211,C50212,C50219,C50221 C50222,C50229,C50311,C50312,C50319,C50321,C50322,C50329 C50411,C50412,C50419,C50421,C50422,C50429,C50511,C50512 C50519,C50521,C50522,C50529,C50611,C50612,C50619,C50621 C50622,C50629,C50811,C50812,C50819,C50821,C50822,C50829 C50911,C50912,C50919,C50921,C50922,C50929,D0500,D0501,D0502 D0580,D0581,D0582,D0590,D0591,D0592 | 1740,1741,1742,1743,1744,1745,1746, 1748,1749,1750,1759,2330 |

Continued on next page

Table S9 (continued).

| Diagnosis | ICD-10 | ICD-9 |
|---|---|---|
| Breast cancer - ductal carcinoma in situ (DCIS) | D0510,D0511,D0512 | 2330 |
| Burn and corrosion, | T2000XA,T20011A,T20012A,T20019A,T2002XA,T2003XA,T2004XA | 9400,9401,9402,9403,9404,9405,9409, 94100,94101,94103,94104 |
| initial encounter | T2005XA,T2006XA,T2007XA,T2009XA,T2010XA,T20111A,T20112A | 94105,94106,94107,94108,94109,94110, 94111,94113,94114,94115 |
| | T20119A,T2012XA,T2013XA,T2014XA,T2015XA,T2016XA,T2017XA | 94116,94117,94118,94119,94120,94121, 94123,94124,94125,94126 |
| | T2019XA,T2020XA,T20211A,T20212A,T20219A,T2022XA,T2023XA | 94127,94128,94129,94130,94131,94133, 94134,94135,94136,94137 |
| | T2024XA,T2025XA,T2026XA,T2027XA,T2029XA,T2030XA,T20311A | 94138,94139,94140,94141,94143,94144, 94145,94146,94147,94148 |
| | T20312A,T20319A,T2032XA,T2033XA,T2034XA,T2035XA,T2036XA | 94149,94150,94151,94152,94153,94154, 94155,94156,94157,94158 |
| | T2037XA,T2039XA,T2040XA,T20411A,T20412A,T20419A,T2042XA | 94159,94200,94201,94202,94203,94204, 94205,94209,94210,94211 |
| | T2043XA,T2044XA,T2045XA,T2046XA,T2047XA,T2049XA,T2050XA | 94212,94213,94214,94215,94219,94220, 94221,94222,94223,94224 |
| | T20511A,T20512A,T20519A,T2052XA,T2053XA,T2054XA,T2055XA | 94225,94229,94230,94231,94232,94233, 94234,94235,94239,94240 |
| | T2056XA,T2057XA,T2059XA,T2060XA,T20611A,T20612A,T20619A | 94241,94242,94243,94244,94245,94249, 94250,94251,94252,94253 |
| | T2062XA,T2063XA,T2064XA,T2065XA,T2066XA,T2067XA,T2069XA | 94254,94255,94259,94300,94301,94302, 94303,94304,94305,94306 |
| | T2070XA,T20711A,T20712A,T20719A,T2072XA,T2073XA,T2074XA | 94309,94310,94311,94312,94313,94314, 94315,94316,94319,94320 |
| | T2075XA,T2076XA,T2077XA,T2079XA,T2100XA,T2101XA,T2102XA | 94321,94322,94323,94324,94325,94326, 94329,94330,94331,94332 |
| | T2103XA,T2104XA,T2105XA,T2106XA,T2107XA,T2109XA,T2110XA | 94333,94334,94335,94336,94339,94340, 94341,94342,94343,94344 |
| | T2111XA,T2112XA,T2113XA,T2114XA,T2115XA,T2116XA,T2117XA | 94345,94346,94349,94351,94352,94353, 94354,94355,94356,94359 |
| | T2119XA,T2120XA,T2121XA,T2122XA,T2123XA,T2124XA,T2125XA | 94400,94401,94402,94403,94404,94405, 94406,94407,94408,94410 |
| | T2126XA,T2127XA,T2129XA,T2130XA,T2131XA,T2132XA,T2133XA | 94411,94412,94413,94414,94415,94416, 94417,94418,94420,94421 |
| | T2134XA,T2135XA,T2136XA,T2137XA,T2139XA,T2140XA,T2141XA | 94422,94423,94424,94425,94426,94427, 94428,94430,94431,94432 |
| | T2142XA,T2143XA,T2144XA,T2145XA,T2146XA,T2147XA,T2149XA | 94433,94434,94435,94436,94437,94438, 94440,94441,94442,94443 |
| | T2150XA,T2151XA,T2152XA,T2153XA,T2154XA,T2155XA,T2156XA | 94444,94445,94446,94447,94448,94450, 94451,94452,94453,94454 |
| | T2157XA,T2159XA,T2160XA,T2161XA,T2162XA,T2163XA,T2164XA | 94455,94456,94457,94458,94500,94501, 94502,94503,94504,94505 |
| | T2165XA,T2166XA,T2167XA,T2169XA,T2170XA,T2171XA,T2172XA | 94506,94509,94510,94511,94512,94513, 94514,94515,94516,94519 |
| | T2173XA,T2174XA,T2175XA,T2176XA,T2177XA,T2179XA,T2200XA | 94520,94521,94522,94523,94524,94525, 94526,94529,94530,94531 |
| | T22011A,T22012A,T22019A,T22021A,T22022A,T22029A,T22031A | 94532,94533,94534,94535,94536,94539, 94540,94541,94542,94543 |
| | T22032A,T22039A,T22041A,T22042A,T22049A,T22051A,T22052A | 94544,94545,94546,94549,94550,94551, 94552,94553,94554,94555 |
| | T22059A,T22061A,T22062A,T22069A,T22091A,T22092A,T22099A | 94556,94559,9470,9471,9472,9473,9474, 9478,9479,94800,94810 |
| | T2210XA,T22111A,T22112A,T22119A,T22121A,T22122A,T22129A | 94811,94820,94821,94822,94830,94831, 94832,94833,94840,94841 |
| | T22131A,T22132A,T22139A,T22141A,T22142A,T22149A,T22151A | 94842,94843,94844,94850,94851,94852, 94853,94854,94855,94860 |
| | T22152A,T22159A,T22161A,T22162A,T22169A,T22191A,T22192A | 94861,94862,94863,94864,94865,94866, 94870,94871,94872,94873 |
| | T22199A,T2220XA,T22211A,T22212A,T22219A,T22221A,T22222A | 94874,94875,94876,94877,94880,94881, 94882,94883,94884,94885 |
| | T22229A,T22231A,T22232A,T22239A,T22241A,T22242A,T22249A | 94886,94887,94888,94890,94891,94892, 94893,94894,94895,94896 |
| | T22251A,T22252A,T22259A,T22261A,T22262A,T22269A,T22291A | 94897,94898,94899,9490 |
| | T22292A,T22299A,T2230XA,T22311A,T22312A,T22319A,T22321A | |
| | T22322A,T22329A,T22331A,T22332A,T22339A,T22341A,T22342A | |
| | T22349A,T22351A,T22352A,T22359A,T22361A,T22362A,T22369A | |
| | T22391A,T22392A,T22399A,T2240XA,T22411A,T22412A,T22419A | |
| | T22421A,T22422A,T22429A,T22431A,T22432A,T22439A,T22441A | |
| | T22442A,T22449A,T22451A,T22452A,T22459A,T22461A,T22462A | |
| | T22469A,T22491A,T22492A,T22499A,T2250XA,T22511A,T22512A | |
| | T22519A,T22521A,T22522A,T22529A,T22531A,T22532A,T22539A | |
| | T22541A,T22542A,T22549A,T22551A,T22552A,T22559A,T22561A | |
| | T22562A,T22569A,T22591A,T22592A,T22599A,T2260XA,T22611A | |
| | T22612A,T22619A,T22621A,T22622A,T22629A,T22631A,T22632A | |
| | T22639A,T22641A,T22642A,T22649A,T22651A,T22652A,T22659A | |
| | T22661A,T22662A,T22669A,T22691A,T22692A,T22699A,T2270XA | |
| | T22711A,T22712A,T22719A,T22721A,T22722A,T22729A,T22731A | |
| | T22732A,T22739A,T22741A,T22742A,T22749A,T22751A,T22752A | |
| | T22759A,T22761A,T22762A,T22769A,T22791A,T22792A,T22799A | |
| | T23001A,T23002A,T23009A,T23011A,T23012A,T23019A,T23021A | |
| | T23022A,T23029A,T23031A,T23032A,T23039A,T23041A,T23042A | |
| | T23049A,T23051A,T23052A,T23059A,T23061A,T23062A,T23069A | |
| | T23071A,T23072A,T23079A,T23091A,T23092A,T23099A,T23101A | |
| | T23102A,T23109A,T23111A,T23112A,T23119A,T23121A,T23122A | |

Continued on next page

Table S9 (continued).

| Diagnosis | ICD-10 | ICD-9 |
|---|---|---|
| | T23129A,T23131A,T23132A,T23139A,T23141A,T23142A,T23149A<br>T23151A,T23152A,T23159A,T23161A,T23162A,T23169A,T23171A<br>T23172A,T23179A,T23191A,T23192A,T23199A,T23201A,T23202A<br>T23209A,T23211A,T23212A,T23219A,T23221A,T23222A,T23229A<br>T23231A,T23232A,T23239A,T23241A,T23242A,T23249A,T23251A<br>T23252A,T23259A,T23261A,T23262A,T23269A,T23271A,T23272A<br>T23279A,T23291A,T23292A,T23299A,T23301A,T23302A,T23309A<br>T23311A,T23312A,T23319A,T23321A,T23322A,T23329A,T23331A<br>T23332A,T23339A,T23341A,T23342A,T23349A,T23351A,T23352A<br>T23359A,T23361A,T23362A,T23369A,T23371A,T23372A,T23379A<br>T23391A,T23392A,T23399A,T23401A,T23402A,T23409A,T23411A<br>T23412A,T23419A,T23421A,T23422A,T23429A,T23431A,T23432A<br>T23439A,T23441A,T23442A,T23449A,T23451A,T23452A,T23459A<br>T23461A,T23462A,T23469A,T23471A,T23472A,T23479A,T23491A<br>T23492A,T23499A,T23501A,T23502A,T23509A,T23511A,T23512A<br>T23519A,T23521A,T23522A,T23529A,T23531A,T23532A,T23539A<br>T23541A,T23542A,T23549A,T23551A,T23552A,T23559A,T23561A<br>T23562A,T23569A,T23571A,T23572A,T23579A,T23591A,T23592A<br>T23599A,T23601A,T23602A,T23609A,T23611A,T23612A,T23619A<br>T23621A,T23622A,T23629A,T23631A,T23632A,T23639A,T23641A<br>T23642A,T23649A,T23651A,T23652A,T23659A,T23661A,T23662A<br>T23669A,T23671A,T23672A,T23679A,T23691A,T23692A,T23699A<br>T23701A,T23702A,T23709A,T23711A,T23712A,T23719A,T23721A<br>T23722A,T23729A,T23731A,T23732A,T23739A,T23741A,T23742A<br>T23749A,T23751A,T23752A,T23759A,T23761A,T23762A,T23769A<br>T23771A,T23772A,T23779A,T23791A,T23792A,T23799A,T24001A<br>T24002A,T24009A,T24011A,T24012A,T24019A,T24021A,T24022A<br>T24029A,T24031A,T24032A,T24039A,T24091A,T24092A,T24099A<br>T24101A,T24102A,T24109A,T24111A,T24112A,T24119A,T24121A<br>T24122A,T24129A,T24131A,T24132A,T24139A,T24191A,T24192A<br>T24199A,T24201A,T24202A,T24209A,T24211A,T24212A,T24219A<br>T24221A,T24222A,T24229A,T24231A,T24232A,T24239A,T24291A<br>T24292A,T24299A,T24301A,T24302A,T24309A,T24311A,T24312A<br>T24319A,T24321A,T24322A,T24329A,T24331A,T24332A,T24339A<br>T24391A,T24392A,T24399A,T24401A,T24402A,T24409A,T24411A<br>T24412A,T24419A,T24421A,T24422A,T24429A,T24431A,T24432A<br>T24439A,T24491A,T24492A,T24499A,T24501A,T24502A,T24509A<br>T24511A,T24512A,T24519A,T24521A,T24522A,T24529A,T24531A<br>T24532A,T24539A,T24591A,T24592A,T24599A,T24601A,T24602A<br>T24609A,T24611A,T24612A,T24619A,T24621A,T24622A,T24629A<br>T24631A,T24632A,T24639A,T24691A,T24692A,T24699A,T24701A<br>T24702A,T24709A,T24711A,T24712A,T24719A,T24721A,T24722A<br>T24729A,T24731A,T24732A,T24739A,T24791A,T24792A,T24799A<br>T25011A,T25012A,T25019A,T25021A,T25022A,T25029A,T25031A<br>T25032A,T25039A,T25091A,T25092A,T25099A,T25111A,T25112A<br>T25119A,T25121A,T25122A,T25129A,T25131A,T25132A,T25139A<br>T25191A,T25192A,T25199A,T25211A,T25212A,T25219A,T25221A<br>T25222A,T25229A,T25231A,T25232A,T25239A,T25291A,T25292A<br>T25299A,T25311A,T25312A,T25319A,T25321A,T25322A,T25329A<br>T25331A,T25332A,T25339A,T25391A,T25392A,T25399A,T25411A<br>T25412A,T25419A,T25421A,T25422A,T25429A,T25431A,T25432A<br>T25439A,T25491A,T25492A,T25499A,T25511A,T25512A,T25519A<br>T25521A,T25522A,T25529A,T25531A,T25532A,T25539A,T25591A<br>T25592A,T25599A,T25611A,T25612A,T25619A,T25621A,T25622A<br>T25629A,T25631A,T25632A,T25639A,T25691A,T25692A,T25699A<br>T25711A,T25712A,T25719A,T25721A,T25722A,T25729A,T25731A<br>T25732A,T25739A,T25791A,T25792A,T25799A,T2600XA,T2601XA<br>T2602XA,T2610XA,T2611XA,T2612XA,T2620XA,T2621XA,T2622XA<br>T2630XA,T2631XA,T2632XA,T2640XA,T2641XA,T2642XA,T2650XA<br>T2651XA,T2652XA,T2660XA,T2661XA,T2662XA,T2670XA,T2671XA<br>T2672XA,T2680XA,T2681XA,T2682XA,T2690XA,T2691XA,T2692XA<br>T270XXA,T271XXA,T272XXA,T273XXA,T274XXA,T275XXA,T276XXA<br>T277XXA,T280XXA,T281XXA,T282XXA,T283XXA,T2840XA,T28411A<br>T28412A,T28419A,T2849XA,T285XXA,T286XXA,T287XXA,T288XXA<br>T2890XA,T28911A,T28912A,T28919A,T2899XA,T300,T304,T310,T3110<br>T3111,T3120,T3121,T3122,T3130,T3131,T3132,T3133,T3140,T3141<br>T3142,T3143,T3144,T3150,T3151,T3152,T3153,T3154,T3155,T3160<br>T3161,T3162,T3163,T3164,T3165,T3166,T3170,T3171,T3172,T3173<br>T3174,T3175,T3176,T3177,T3180,T3181,T3182,T3183,T3184,T3185<br>T3186,T3187,T3188,T3190,T3191,T3192,T3193,T3194,T3195,T3196<br>T3197,T3198,T3199,T320,T3210,T3211,T3220,T3221,T3222,T3230<br>T3231,T3232,T3233,T3240,T3241,T3242,T3243,T3244,T3250,T3251<br>T3252,T3253,T3254,T3255,T3260,T3261,T3262,T3263,T3264,T3265<br>T3266,T3270,T3271,T3272,T3273,T3274,T3275,T3276,T3277,T3280<br>T3281,T3282,T3283,T3284,T3285,T3286,T3287,T3288,T3290,T3291<br>T3292,T3293,T3294,T3295,T3296,T3297,T3298,T3299 | |
| Burns and corrosion, subsequent encounter | T2000XD,T20011D,T20012D,T20019D,T2002XD,T2003XD,T2004XD<br>T2005XD,T2006XD,T2007XD,T2009XD,T2010XD,T20111D,T20112D<br>T20119D,T2012XD,T2013XD,T2014XD,T2015XD,T2016XD,T2017XD<br>T2019XD,T2020XD,T20211D,T20212D,T20219D,T2022XD,T2023XD<br>T2024XD,T2025XD,T2026XD,T2027XD,T2029XD,T2030XD,T20311D<br>T20312D,T20319D,T2032XD,T2033XD,T2034XD,T2035XD,T2036XD<br>T2037XD,T2039XD,T2040XD,T20411D,T20412D,T20419D,T2042XD<br>T2043XD,T2044XD,T2045XD,T2046XD,T2047XD,T2049XD,T2050XD | V5889 |

Continued on next page

Table S9 (continued).

| Diagnosis | ICD-10 | ICD-9 |
|---|---|---|
| | T20511D,T20512D,T20519D,T2052XD,T2053XD,T2054XD,T2055XD | |
| | T2056XD,T2057XD,T2059XD,T2060XD,T20611D,T20612D,T20619D | |
| | T2062XD,T2063XD,T2064XD,T2065XD,T2066XD,T2067XD,T2069XD | |
| | T2070XD,T20711D,T20712D,T20719D,T2072XD,T2073XD,T2074XD | |
| | T2075XD,T2076XD,T2077XD,T2079XD,T2100XD,T2101XD,T2102XD | |
| | T2103XD,T2104XD,T2105XD,T2106XD,T2107XD,T2109XD,T2110XD | |
| | T2111XD,T2112XD,T2113XD,T2114XD,T2115XD,T2116XD,T2117XD | |
| | T2119XD,T2120XD,T2121XD,T2122XD,T2123XD,T2124XD,T2125XD | |
| | T2126XD,T2127XD,T2129XD,T2130XD,T2131XD,T2132XD,T2133XD | |
| | T2134XD,T2135XD,T2136XD,T2137XD,T2139XD,T2140XD,T2141XD | |
| | T2142XD,T2143XD,T2144XD,T2145XD,T2146XD,T2147XD,T2149XD | |
| | T2150XD,T2151XD,T2152XD,T2153XD,T2154XD,T2155XD,T2156XD | |
| | T2157XD,T2159XD,T2160XD,T2161XD,T2162XD,T2163XD,T2164XD | |
| | T2165XD,T2166XD,T2167XD,T2169XD,T2170XD,T2171XD,T2172XD | |
| | T2173XD,T2174XD,T2175XD,T2176XD,T2177XD,T2179XD,T2200XD | |
| | T22011D,T22012D,T22019D,T22021D,T22022D,T22029D,T22031D | |
| | T22032D,T22039D,T22041D,T22042D,T22049D,T22051D,T22052D | |
| | T22059D,T22061D,T22062D,T22069D,T22091D,T22092D,T22099D | |
| | T2210XD,T22111D,T22112D,T22119D,T22121D,T22122D,T22129D | |
| | T22131D,T22132D,T22139D,T22141D,T22142D,T22149D,T22151D | |
| | T22152D,T22159D,T22161D,T22162D,T22169D,T22191D,T22192D | |
| | T22199D,T2220XD,T22211D,T22212D,T22219D,T22221D,T22222D | |
| | T22229D,T22231D,T22232D,T22239D,T22241D,T22242D,T22249D | |
| | T22251D,T22252D,T22259D,T22261D,T22262D,T22269D,T22291D | |
| | T22292D,T22299D,T2230XD,T22311D,T22312D,T22319D,T22321D | |
| | T22322D,T22329D,T22331D,T22332D,T22339D,T22341D,T22342D | |
| | T22349D,T22351D,T22352D,T22359D,T22361D,T22362D,T22369D | |
| | T22391D,T22392D,T22399D,T2240XD,T22411D,T22412D,T22419D | |
| | T22421D,T22422D,T22429D,T22431D,T22432D,T22439D,T22441D | |
| | T22442D,T22449D,T22451D,T22452D,T22459D,T22461D,T22462D | |
| | T22469D,T22491D,T22492D,T22499D,T2250XD,T22511D,T22512D | |
| | T22519D,T22521D,T22522D,T22529D,T22531D,T22532D,T22539D | |
| | T22541D,T22542D,T22549D,T22551D,T22552D,T22559D,T22561D | |
| | T22562D,T22569D,T22591D,T22592D,T22599D,T2260XD,T22611D | |
| | T22612D,T22619D,T22621D,T22622D,T22629D,T22631D,T22632D | |
| | T22639D,T22641D,T22642D,T22649D,T22651D,T22652D,T22659D | |
| | T22661D,T22662D,T22669D,T22691D,T22692D,T22699D,T2270XD | |
| | T22711D,T22712D,T22719D,T22721D,T22722D,T22729D,T22731D | |
| | T22732D,T22739D,T22741D,T22742D,T22749D,T22751D,T22752D | |
| | T22759D,T22761D,T22762D,T22769D,T22791D,T22792D,T22799D | |
| | T23001D,T23002D,T23009D,T23011D,T23012D,T23019D,T23021D | |
| | T23022D,T23029D,T23031D,T23032D,T23039D,T23041D,T23042D | |
| | T23049D,T23051D,T23052D,T23059D,T23061D,T23062D,T23069D | |
| | T23071D,T23072D,T23079D,T23091D,T23092D,T23099D,T23101D | |
| | T23102D,T23109D,T23111D,T23112D,T23119D,T23121D,T23122D | |
| | T23129D,T23131D,T23132D,T23139D,T23141D,T23142D,T23149D | |
| | T23151D,T23152D,T23159D,T23161D,T23162D,T23169D,T23171D | |
| | T23172D,T23179D,T23191D,T23192D,T23199D,T23201D,T23202D | |
| | T23209D,T23211D,T23212D,T23219D,T23221D,T23222D,T23229D | |
| | T23231D,T23232D,T23239D,T23241D,T23242D,T23249D,T23251D | |
| | T23252D,T23259D,T23261D,T23262D,T23269D,T23271D,T23272D | |
| | T23279D,T23291D,T23292D,T23299D,T23301D,T23302D,T23309D | |
| | T23311D,T23312D,T23319D,T23321D,T23322D,T23329D,T23331D | |
| | T23332D,T23339D,T23341D,T23342D,T23349D,T23351D,T23352D | |
| | T23359D,T23361D,T23362D,T23369D,T23371D,T23372D,T23379D | |
| | T23391D,T23392D,T23399D,T23401D,T23402D,T23409D,T23411D | |
| | T23412D,T23419D,T23421D,T23422D,T23429D,T23431D,T23432D | |
| | T23439D,T23441D,T23442D,T23449D,T23451D,T23452D,T23459D | |
| | T23461D,T23462D,T23469D,T23471D,T23472D,T23479D,T23491D | |
| | T23492D,T23499D,T23501D,T23502D,T23509D,T23511D,T23512D | |
| | T23519D,T23521D,T23522D,T23529D,T23531D,T23532D,T23539D | |
| | T23541D,T23542D,T23549D,T23551D,T23552D,T23559D,T23561D | |
| | T23562D,T23569D,T23571D,T23572D,T23579D,T23591D,T23592D | |
| | T23599D,T23601D,T23602D,T23609D,T23611D,T23612D,T23619D | |
| | T23621D,T23622D,T23629D,T23631D,T23632D,T23639D,T23641D | |
| | T23642D,T23649D,T23651D,T23652D,T23659D,T23661D,T23662D | |
| | T23669D,T23671D,T23672D,T23679D,T23691D,T23692D,T23699D | |
| | T23701D,T23702D,T23709D,T23711D,T23712D,T23719D,T23721D | |
| | T23722D,T23729D,T23731D,T23732D,T23739D,T23741D,T23742D | |
| | T23749D,T23751D,T23752D,T23759D,T23761D,T23762D,T23769D | |
| | T23771D,T23772D,T23779D,T23791D,T23792D,T23799D,T24001D | |
| | T24002D,T24009D,T24011D,T24012D,T24019D,T24021D,T24022D | |
| | T24029D,T24031D,T24032D,T24039D,T24091D,T24092D,T24099D | |
| | T24101D,T24102D,T24109D,T24111D,T24112D,T24119D,T24121D | |
| | T24122D,T24129D,T24131D,T24132D,T24139D,T24191D,T24192D | |
| | T24199D,T24201D,T24202D,T24209D,T24211D,T24212D,T24219D | |
| | T24221D,T24222D,T24229D,T24231D,T24232D,T24239D,T24291D | |
| | T24292D,T24299D,T24301D,T24302D,T24309D,T24311D,T24312D | |
| | T24319D,T24321D,T24322D,T24329D,T24331D,T24332D,T24339D | |
| | T24391D,T24392D,T24399D,T24401D,T24402D,T24409D,T24411D | |
| | T24412D,T24419D,T24421D,T24422D,T24429D,T24431D,T24432D | |
| | T24439D,T24491D,T24492D,T24499D,T24501D,T24502D,T24509D | |
| | T24511D,T24512D,T24519D,T24521D,T24522D,T24529D,T24531D | |
| | T24532D,T24539D,T24591D,T24592D,T24599D,T24601D,T24602D | |
| | T24609D,T24611D,T24612D,T24619D,T24621D,T24622D,T24629D | |

Continued on next page

Table S9 (continued).

| Diagnosis | ICD-10 | ICD-9 |
|---|---|---|
| | T24631D,T24632D,T24639D,T24691D,T24692D,T24699D,T24701D<br>T24702D,T24709D,T24711D,T24712D,T24719D,T24721D,T24722D<br>T24729D,T24731D,T24732D,T24739D,T24791D,T24792D,T24799D<br>T25011D,T25012D,T25019D,T25021D,T25022D,T25029D,T25031D<br>T25032D,T25039D,T25091D,T25092D,T25099D,T25111D,T25112D<br>T25119D,T25121D,T25122D,T25129D,T25131D,T25132D,T25139D<br>T25191D,T25192D,T25199D,T25211D,T25212D,T25219D,T25221D<br>T25222D,T25229D,T25231D,T25232D,T25239D,T25291D,T25292D<br>T25299D,T25311D,T25312D,T25319D,T25321D,T25322D,T25329D<br>T25331D,T25332D,T25339D,T25391D,T25392D,T25399D,T25411D<br>T25412D,T25419D,T25421D,T25422D,T25429D,T25431D,T25432D<br>T25439D,T25491D,T25492D,T25499D,T25511D,T25512D,T25519D<br>T25521D,T25522D,T25529D,T25531D,T25532D,T25539D,T25591D<br>T25592D,T25599D,T25611D,T25612D,T25619D,T25621D,T25622D<br>T25629D,T25631D,T25632D,T25639D,T25691D,T25692D,T25699D<br>T25711D,T25712D,T25719D,T25721D,T25722D,T25729D,T25731D<br>T25732D,T25739D,T25791D,T25792D,T25799D,T2600XD,T2601XD<br>T2602XD,T2610XD,T2611XD,T2612XD,T2620XD,T2621XD,T2622XD<br>T2630XD,T2631XD,T2632XD,T2640XD,T2641XD,T2642XD,T2650XD<br>T2651XD,T2652XD,T2660XD,T2661XD,T2662XD,T2670XD,T2671XD<br>T2672XD,T2680XD,T2681XD,T2682XD,T2690XD,T2691XD,T2692XD<br>T270XXD,T271XXD,T272XXD,T273XXD,T274XXD,T275XXD,T276XXD<br>T277XXD,T280XXD,T281XXD,T282XXD,T283XXD,T2840XD,T28411D<br>T28412D,T28419D,T2849XD,T285XXD,T286XXD,T287XXD,T288XXD<br>T2890XD,T28911D,T28912D,T28919D,T2899XD | |
| CNS abscess | A066,A1781,A5482,B431,G060,G061,G062,G07 | 0065,01320,01330,01340,01350,09889,<br>1172,3240,3241,3249 |
| COVID-19 | J1282,U071,U099 | |
| COVID-19 acute | J1282,U071 | |
| COVID-19 long-term | U099 | |
| Calculus of urinary<br>tract | N132,N200,N201,N202,N209,N210,N211,N218,N219,N22 | 591,5920,5921,5929,5940,5941,5942,5948,<br>5949 |
| Cancer of other sites | C761,C762,C763,C7640,C7641,C7642,C7650,C7651,C7652,C768,D098 | 1951,1952,1953,1954,1955,1958,2348 |
| Cannabis-related<br>disorders | F1210,F12120,F12121,F12122,F12129,F1213,F12150,F12151,F12159<br>F12180,F12188,F1219,F1220,F12220,F12221,F12222,F12229,F1223<br>F12250,F12251,F12259,F12280,F12288,F1229,F1290,F1291,F12920<br>F12921,F12922,F12929,F1293,F12950,F12951,F12959,F12980,F12988<br>F1299,T40711A,T40712A,T40713A,T40714A,T40715A,T40721A,T40722A<br>T40723A,T40724A,T40725A,T407X1A,T407X2A,T407X3A,T407X4A<br>T407X5A | 29211,29212,2922,29281,29289,2929,<br>30430,30431,30432,30520<br>30521,30522,30523,9696,99529,E8541,<br>E9396,E9502,E9620,E9802 |
| Cardiac and<br>circulatory<br>congenital anomalies | P095,Q200,Q201,Q202,Q203,Q204,Q205,Q206,Q208,Q209,Q210,Q211<br>Q2110,Q2111,Q2112,Q2113,Q2114,Q2115,Q2116,Q2119,Q212,Q2120<br>Q2121,Q2122,Q2123,Q213,Q214,Q218,Q219,Q220,Q221,Q222,Q223<br>Q224,Q225,Q226,Q228,Q229,Q230,Q231,Q232,Q233,Q234,Q238,Q239<br>Q240,Q241,Q242,Q243,Q244,Q245,Q246,Q248,Q249,Q250,Q251,Q252<br>Q2521,Q2529,Q253,Q254,Q2540,Q2541,Q2542,Q2543,Q2544,Q2545<br>Q2546,Q2547,Q2548,Q2549,Q255,Q256,Q2571,Q2572,Q2579,Q258,Q259<br>Q260,Q261,Q262,Q263,Q264,Q265,Q266,Q268,Q269,Q270,Q271,Q272<br>Q2730,Q2731,Q2732,Q2733,Q2734,Q2739,Q274,Q278,Q279,Q280,Q281<br>Q282,Q283,Q288,Q289 | 7450,74510,74511,74512,74519,7452,7453,<br>7454,7455,74560,74561<br>74569,7457,7458,7459,74600,74601,74602,<br>74609,7461,7462,7463<br>7464,7465,7466,7467,74681,74682,74683,<br>74684,74685,74686,74687<br>74689,7469,7470,74710,74720,74721,<br>74722,74729,74731,74732<br>74739,74740,74741,74742,74749,7475,<br>74760,74761,74762,74763<br>74764,74769,74781,74789,7479 |
| Cardiac arrest and<br>ventricular<br>fibrillation | I462,I468,I469,I4901,I4902,I97120,I97121,I97710,I97711,O0336<br>O0386,O0486,O0736,O0881,O29111,O29112,O29113,O29119 | 42741,42742,4275,4294,63470,63471,<br>63472,63572,6387,6398,64680<br>64681,64683,9971 |
| Cardiac cancers | C380,C381,C382,C383,C388 | 1641,1642,1643,1648,1649 |
| Cardiac dysrhythmias | I470,I471,I472,I4720,I4721,I4729,I479,I480,I481,I4811,I4819<br>I482,I4820,I4821,I483,I484,I4891,I4892,I491,I492,I493,I4940<br>I4949,I495,I498,I499 | 4270,4271,4272,42731,42732,42760,42761,<br>42769,42781,42789,4279 |
| Carrier status | O99820,O99824,O99825,O99830,O99834,O99835,Z220,Z221,Z222<br>Z2231,Z22321,Z22322,Z22330,Z22338,Z2239,Z224,Z2250,Z2251<br>Z2252,Z2259,Z226,Z227,Z228,Z229 | 64781,64783,64784,64891,64893,64894,<br>V020,V021,V022,V023,V024<br>V0251,V0252,V0253,V0254,V0259,<br>V0260,V0261,V0262,V0269,V027<br>V028,V029 |

Continued on next page

Table S9 (continued).

| Diagnosis | ICD-10 | ICD-9 |
|---|---|---|
| Cataract and other lens disorders | E0836,E0936,E1036,E1136,E1336,H25011,H25012,H25013,H25019 H25031,H25032,H25033,H25039,H25041,H25042,H25043,H25049 H25091,H25092,H25093,H25099,H2510,H2511,H2512,H2513,H2520 H2521,H2522,H2523,H25811,H25812,H25813,H25819,H2589,H259 H26001,H26002,H26003,H26009,H26011,H26012,H26013,H26019 H26031,H26032,H26033,H26039,H26041,H26042,H26043,H26049 H26051,H26052,H26053,H26059,H26061,H26062,H26063,H26069,H2609 H26101,H26102,H26103,H26109,H26111,H26112,H26113,H26119 H26121,H26122,H26123,H26129,H26131,H26132,H26133,H26139,H2620 H26211,H26212,H26213,H26219,H26221,H26222,H26223,H26229 H26231,H26232,H26233,H26239,H2630,H2631,H2632,H2633,H2640 H26411,H26412,H26413,H26419,H26491,H26492,H26493,H26499,H268 H269,H2700,H2701,H2702,H2703,H2710,H27111,H27112,H27113 H27119,H27121,H27122,H27123,H27129,H27131,H27132,H27133 H27139,H278,H279,H28 | 24950,25050,25051,36600,36601,36602, 36603,36604,36609,36610 36612,36613,36614,36615,36616,36618, 36619,36620,36621,36622 36623,36630,36631,36632,36633,36634, 36641,36644,36645,36646 36650,36651,36652,36653,3668,3669, 37931,37932,37933,37934 37939 |
| Cerebral infarction | G43601,G43609,G43611,G43619,I6300,I63011,I63012,I63013,I63019 I6302,I63031,I63032,I63033,I63039,I6309,I6310,I63111,I63112 I63113,I63119,I6312,I63131,I63132,I63133,I63139,I6319,I6320 I63211,I63212,I63213,I63219,I6322,I63231,I63232,I63233,I63239 I6329,I6330,I63311,I63312,I63313,I63319,I63321,I63322,I63323 I63329,I63331,I63332,I63333,I63339,I63341,I63342,I63343 I63349,I6339,I6340,I63411,I63412,I63413,I63419,I63421,I63422 I63423,I63429,I63431,I63432,I63433,I63439,I63441,I63442 I63443,I63449,I6349,I6350,I63511,I63512,I63513,I63519,I63521 I63522,I63523,I63529,I63531,I63532,I63533,I63539,I63541 I63542,I63543,I63549,I6359,I636,I638,I6381,I6389,I639,I97810 I97811,I97820,I97821,R29700,R29701,R29702,R29703,R29704 R29705,R29706,R29707,R29708,R29709,R29710,R29711,R29712 R29713,R29714,R29715,R29716,R29717,R29718,R29719,R29720 R29721,R29722,R29723,R29724,R29725,R29726,R29727,R29728 R29729,R29730,R29731,R29732,R29733,R29734,R29735,R29736 R29737,R29738,R29739,R29740,R29741,R29742 | 34660,34661,34662,34663,43301,43311, 43321,43331,43381,43391 43401,43411,43491,99702 |
| Cerebral palsy | G800,G801,G802,G803,G804,G808,G809 | 33371,3430,3431,3432,3433,3434,3438, 3439 |
| Chromosomal abnormalities | Q900,Q901,Q902,Q909,Q910,Q911,Q912,Q913,Q914,Q915,Q916,Q917 Q920,Q921,Q922,Q925,Q9261,Q9262,Q927,Q928,Q929,Q930,Q931,Q932 Q933,Q934,Q935,Q9351,Q9359,Q937,Q9381,Q9382,Q9388,Q9389,Q939 Q950,Q951,Q952,Q953,Q955,Q958,Q959,Q960,Q961,Q962,Q963,Q964 Q968,Q969,Q970,Q971,Q972,Q973,Q978,Q979,Q980,Q981,Q983,Q984 Q985,Q986,Q987,Q988,Q989,Q990,Q991,Q992,Q998,Q999 | 7580,7581,7582,75831,75832,75833,75839, 7584,7585,7586,7587 75881,75889,7589,75983 |
| Chronic kidney disease | D631,E0822,E0922,E1022,E1122,E1322,I120,I129,I130,I1310,I1311 I132,N181,N182,N183,N1830,N1831,N1832,N184,N185,N186,N189 R880,Z4901,Z4902,Z4931,Z4932 | 24940,25040,25041,28521,40300,40301, 40310,40311,40390,40391 40400,40401,40402,40403,40410,40411, 40412,40413,40490,40491 40492,40493,58181,58381,5851,5852,5853, 5854,5855,5856,5859 7925,V561,V562,V5631,V5632 |
| Chronic obstructive pulmonary disease and bronchiectasis | J410,J411,J418,J42,J430,J431,J432,J438,J439,J440,J441,J449 J470,J471,J479 | 4910,4911,49120,49121,49122,4918,4919, 4920,4928,49320,49321 49322,4940,4941,496 |
| Chronic phlebitis; thrombophlebitis and thromboembolism | I82211,I82221,I82291,I82501,I82502,I82503,I82509,I82511 I82512,I82513,I82519,I82521,I82522,I82523,I82529,I82531 I82532,I82533,I82539,I82541,I82542,I82543,I82549,I82551 I82552,I82553,I82559,I82561,I82562,I82563,I82569,I82591 I82592,I82593,I82599,I825Y1,I825Y2,I825Y3,I825Y9,I825Z1 I825Z2,I825Z3,I825Z9,I82701,I82702,I82703,I82709,I82711 I82712,I82713,I82719,I82721,I82722,I82723,I82729,I82891,I8291 I82A21,I82A22,I82A23,I82A29,I82B21,I82B22,I82B23,I82B29 I82C21,I82C22,I82C23,I82C29 | 4532,45350,45351,45352,45371,45372, 45373,45374,45375,45376 45377,45379 |
| Chronic rheumatic heart disease | I050,I051,I052,I058,I059,I060,I061,I062,I068,I069,I070,I071 I072,I078,I079,I080,I081,I082,I083,I088,I089,I090,I091,I092 I0981,I0989,I099 | 393,3940,3941,3942,3949,3950,3951,3952, 3959,3960,3961,3962 3963,3968,3969,3970,3971,3979,3980, 39890,39891,39899 |
| Circulatory signs and symptoms | R000,R001,R002,R008,R009,R010,R011,R012,R030,R031,R0989,R58 | 42781,42789,4590,7850,7851,7852,7853, 7859,7867,7962,7963 |
| Cleft lip or palate | Q351,Q353,Q355,Q357,Q359,Q360,Q361,Q369,Q370,Q371,Q372,Q373 | 74900,74902,74910,74911,74912,74913, 74914,74920,74921,74922 |

Continued on next page

Table S9 (continued).

| Diagnosis | ICD-10 | ICD-9 |
|---|---|---|
| | Q374,Q375,Q378,Q379 | 74923,74924 |
| Coagulation and hemorrhagic disorders | D473,D65,D66,D67,D680,D6800,D6801,D68020,D68021,D68022,D68023<br>D68029,D6803,D6804,D6809,D681,D682,D68311,D68312,D68318,D6832<br>D684,D6851,D6852,D6859,D6861,D6862,D6869,D688,D689,D690,D691<br>D692,D693,D6941,D6942,D6949,D6951,D6959,D696,D698,D699,D7582<br>D75821,D75822,D75828,D75829,D75838,D75839,D7584,M362,O723 | 23871,2860,2861,2862,2863,2864,28652,<br>28653,28659,2866,2867<br>2869,2870,2871,2872,28731,28732,28733,<br>28739,28741,28749,2875<br>2878,2879,28981,28982,28984,66632,<br>66634,7132 |
| Coma; stupor; and brain damage | B150,B160,B162,B1711,B190,B1911,B1921,E035,E0801,E0811,E08641<br>E0901,E0911,E09641,E1011,E10641,E1101,E1111,E11641,E1301<br>E1311,E13641,E15,G931,K7041,K7111,K7201,K7211,K7291,R400,R401<br>R4020,R403 | 0700,07020,07021,07041,0706,07071,<br>24920,24930,25020,25030<br>25031,2510,3481,570,5713,5722,5728,<br>5733,78001,78003,78009 |
| Complication of cardiovascular device, implant or graft, initial encounter | T8201XA,T8202XA,T8203XA,T8209XA,T82110A,T82111A,T82118A<br>T82119A,T82120A,T82121A,T82128A,T82129A,T82190A,T82191A<br>T82198A,T82199A,T82211A,T82212A,T82213A,T82218A,T82221A<br>T82222A,T82223A,T82228A,T82310A,T82311A,T82312A,T82318A<br>T82319A,T82320A,T82321A,T82322A,T82328A,T82329A,T82330A<br>T82331A,T82332A,T82338A,T82339A,T82390A,T82391A,T82392A<br>T82398A,T82399A,T8241XA,T8242XA,T8243XA,T8249XA,T82510A<br>T82511A,T82512A,T82513A,T82514A,T82515A,T82518A,T82519A<br>T82520A,T82521A,T82522A,T82523A,T82524A,T82525A,T82528A<br>T82529A,T82530A,T82531A,T82532A,T82533A,T82534A,T82535A<br>T82538A,T82539A,T82590A,T82591A,T82592A,T82593A,T82594A<br>T82595A,T82598A,T82599A,T826XXA,T827XXA,T82817A,T82818A<br>T82827A,T82828A,T82837A,T82838A,T82847A,T82848A,T82855A<br>T82856A,T82857A,T82858A,T82867A,T82868A,T82897A,T82898A<br>T829XXA | 99600,99601,99602,99603,99604,99609,<br>9961,99661,99662,99671<br>99672,99673,99674 |
| Complication of cardiovascular device, implant or graft, subsequent encounter | T8201XD,T8202XD,T8203XD,T8209XD,T82110D,T82111D,T82118D<br>T82119D,T82120D,T82121D,T82128D,T82129D,T82190D,T82191D<br>T82198D,T82199D,T82211D,T82212D,T82213D,T82218D,T82221D<br>T82222D,T82223D,T82228D,T82310D,T82311D,T82312D,T82318D<br>T82319D,T82320D,T82321D,T82322D,T82328D,T82329D,T82330D<br>T82331D,T82332D,T82338D,T82339D,T82390D,T82391D,T82392D<br>T82398D,T82399D,T8241XD,T8242XD,T8243XD,T8249XD,T82510D<br>T82511D,T82512D,T82513D,T82514D,T82515D,T82518D,T82519D<br>T82520D,T82521D,T82522D,T82523D,T82524D,T82525D,T82528D<br>T82529D,T82530D,T82531D,T82532D,T82533D,T82534D,T82535D<br>T82538D,T82539D,T82590D,T82591D,T82592D,T82593D,T82594D<br>T82595D,T82598D,T82599D,T826XXD,T827XXD,T82817D,T82818D<br>T82827D,T82828D,T82837D,T82838D,T82847D,T82848D,T82855D<br>T82856D,T82857D,T82858D,T82867D,T82868D,T82897D,T82898D<br>T829XXD | V5889 |
| Complication of genitourinary device, implant or graft, initial encounter | T83010A,T83011A,T83012A,T83018A,T83020A,T83021A,T83022A<br>T83028A,T83030A,T83031A,T83032A,T83038A,T83090A,T83091A<br>T83092A,T83098A,T83110A,T83111A,T83112A,T83113A,T83118A<br>T83120A,T83121A,T83122A,T83123A,T83128A,T83190A,T83191A<br>T83192A,T83193A,T83198A,T8321XA,T8322XA,T8323XA,T8324XA<br>T8325XA,T8329XA,T8331XA,T8332XA,T8339XA,T83410A,T83411A<br>T83418A,T83420A,T83421A,T83428A,T83490A,T83491A,T83498A<br>T83510A,T83511A,T83512A,T83518A,T8351XA,T83590A,T83591A<br>T83592A,T83593A,T83598A,T8359XA,T8361XA,T8362XA,T8369XA<br>T836XXA,T83711A,T83712A,T83713A,T83714A,T83718A,T83719A<br>T83721A,T83722A,T83723A,T83724A,T83728A,T83729A,T8379XA<br>T8381XA,T8382XA,T8383XA,T8384XA,T8385XA,T8386XA,T8389XA<br>T839XXA | 62931,62932,99631,99632,99639,99664,<br>99665,99676 |
| Complication of genitourinary device, implant or graft, subsequent encounter | T83010D,T83011D,T83012D,T83018D,T83020D,T83021D,T83022D<br>T83028D,T83030D,T83031D,T83032D,T83038D,T83090D,T83091D<br>T83092D,T83098D,T83110D,T83111D,T83112D,T83113D,T83118D<br>T83120D,T83121D,T83122D,T83123D,T83128D,T83190D,T83191D<br>T83192D,T83193D,T83198D,T8321XD,T8322XD,T8323XD,T8324XD<br>T8325XD,T8329XD,T8331XD,T8332XD,T8339XD,T83410D,T83411D<br>T83418D,T83420D,T83421D,T83428D,T83490D,T83491D,T83498D<br>T83510D,T83511D,T83512D,T83518D,T8351XD,T83590D,T83591D<br>T83592D,T83593D,T83598D,T8359XD,T8361XD,T8362XD,T8369XD<br>T836XXD,T83711D,T83712D,T83713D,T83714D,T83718D,T83719D<br>T83721D,T83722D,T83723D,T83724D,T83728D,T83729D,T8379XD<br>T8381XD,T8382XD,T8383XD,T8384XD,T8385XD,T8386XD,T8389XD<br>T839XXD | V5889 |
| Complication of internal orthopedic device or implant, initial encounter | T84010A,T84011A,T84012A,T84013A,T84018A,T84019A,T84020A<br>T84021A,T84022A,T84023A,T84028A,T84029A,T84030A,T84031A<br>T84032A,T84033A,T84038A,T84039A,T84050A,T84051A,T84052A<br>T84053A,T84058A,T84059A,T84060A,T84061A,T84062A,T84063A<br>T84068A,T84069A,T84090A,T84091A,T84092A,T84093A,T84098A | 99641,99642,99643,99645,99646,99647,<br>99649,99666,99667,99677<br>99678 |

Continued on next page

 

Table S9 (continued).

| Diagnosis | ICD-10 | ICD-9 |
|---|---|---|
| | T84099A,T84110A,T84111A,T84112A,T84113A,T84114A,T84115A<br>T84116A,T84117A,T84119A,T84120A,T84121A,T84122A,T84123A<br>T84124A,T84125A,T84126A,T84127A,T84129A,T84190A,T84191A<br>T84192A,T84193A,T84194A,T84195A,T84196A,T84197A,T84199A<br>T84210A,T84213A,T84216A,T84218A,T84220A,T84223A,T84226A<br>T84228A,T84290A,T84293A,T84296A,T84298A,T84310A,T84318A<br>T84320A,T84328A,T84390A,T84398A,T84428A,T84498A,T8450XA<br>T8451XA,T8452XA,T8453XA,T8454XA,T8459XA,T8460XA,T84610A<br>T84611A,T84612A,T84613A,T84614A,T84615A,T84619A,T84620A<br>T84621A,T84622A,T84623A,T84624A,T84625A,T84629A,T8463XA<br>T8469XA,T847XXA,T8481XA,T8482XA,T8483XA,T8484XA,T8485XA<br>T8486XA,T8489XA,T849XXA | |
| Complication of internal orthopedic device or implant, subsequent encounter | T84010D,T84011D,T84012D,T84013D,T84018D,T84019D,T84020D<br>T84021D,T84022D,T84023D,T84028D,T84029D,T84030D,T84031D<br>T84032D,T84033D,T84038D,T84039D,T84050D,T84051D,T84052D<br>T84053D,T84058D,T84059D,T84060D,T84061D,T84062D,T84063D<br>T84068D,T84069D,T84090D,T84091D,T84092D,T84093D,T84098D<br>T84099D,T84110D,T84111D,T84112D,T84113D,T84114D,T84115D<br>T84116D,T84117D,T84119D,T84120D,T84121D,T84122D,T84123D<br>T84124D,T84125D,T84126D,T84127D,T84129D,T84190D,T84191D<br>T84192D,T84193D,T84194D,T84195D,T84196D,T84197D,T84199D<br>T84210D,T84213D,T84216D,T84218D,T84220D,T84223D,T84226D<br>T84228D,T84290D,T84293D,T84296D,T84298D,T84310D,T84318D<br>T84320D,T84328D,T84390D,T84398D,T84418D,T84428D,T84498D<br>T8450XD,T8451XD,T8452XD,T8453XD,T8454XD,T8459XD,T8460XD<br>T84610D,T84611D,T84612D,T84613D,T84614D,T84615D,T84619D<br>T84620D,T84621D,T84622D,T84623D,T84624D,T84625D,T84629D<br>T8463XD,T8469XD,T847XXD,T8481XD,T8482XD,T8483XD,T8484XD<br>T8485XD,T8486XD,T8489XD,T849XXD | V5889 |
| Complication of other surgical or medical care, injury, initial encounter | T800XXA,T801XXA,T80211A,T80212A,T80218A,T80219A,T8022XA<br>T8029XA,T8030XA,T80310A,T80311A,T80319A,T8039XA,T8040XA<br>T80410A,T80411A,T80419A,T8049XA,T8051XA,T8052XA,T8059XA<br>T8061XA,T8062XA,T8069XA,T80810A,T80818A,T8082XA,T8089XA<br>T8090XA,T80910A,T80911A,T80919A,T8092XA,T80A0XA,T80A10A<br>T80A11A,T80A19A,T80A9XA,T8110XA,T8111XA,T8112XA,T8119XA<br>T8130XA,T8131XA,T8132XA,T8133XA,T8140XA,T8141XA,T8142XA<br>T8143XA,T8144XA,T8149XA,T814XXA,T81500A,T81501A,T81502A<br>T81503A,T81504A,T81505A,T81506A,T81507A,T81508A,T81509A<br>T81510A,T81511A,T81512A,T81513A,T81514A,T81515A,T81516A<br>T81517A,T81518A,T81519A,T81520A,T81521A,T81522A,T81523A<br>T81524A,T81525A,T81526A,T81527A,T81528A,T81529A,T81530A<br>T81531A,T81532A,T81533A,T81534A,T81535A,T81536A,T81537A<br>T81538A,T81539A,T81590A,T81591A,T81592A,T81593A,T81594A<br>T81595A,T81596A,T81597A,T81598A,T81599A,T8160XA,T8161XA<br>T8169XA,T81710A,T81711A,T81718A,T81719A,T8172XA,T8181XA<br>T8182XA,T8183XA,T8189XA,T819XXA,T84410A,T84418A,T84420A<br>T84490A,T8501XA,T8502XA,T8503XA,T8509XA,T85110A,T85111A<br>T85112A,T85113A,T85118A,T85120A,T85121A,T85122A,T85123A<br>T85128A,T85190A,T85191A,T85192A,T85193A,T85199A,T8521XA<br>T8522XA,T8529XA,T85310A,T85311A,T85318A,T85320A,T85321A<br>T85328A,T85390A,T85391A,T85398A,T8541XA,T8542XA,T8543XA<br>T8544XA,T8549XA,T85510A,T85511A,T85518A,T85520A,T85521A<br>T85528A,T85590A,T85591A,T85598A,T85610A,T85611A,T85612A<br>T85613A,T85614A,T85615A,T85618A,T85620A,T85621A,T85622A<br>T85623A,T85624A,T85625A,T85628A,T85630A,T85631A,T85633A<br>T85635A,T85638A,T85690A,T85691A,T85692A,T85693A,T85694A<br>T85695A,T85698A,T8571XA,T8572XA,T85730A,T85731A,T85732A<br>T85733A,T85734A,T85735A,T85738A,T8579XA,T85810A,T85818A<br>T8581XA,T85820A,T85828A,T8582XA,T85830A,T85838A,T8583XA<br>T85840A,T85848A,T8584XA,T85850A,T85858A,T8585XA,T85860A<br>T85868A,T8586XA,T85890A,T85898A,T8589XA,T859XXA,T8730,T8731<br>T8732,T8733,T8734,T8740,T8741,T8742,T8743,T8744,T8750,T8751<br>T8752,T8753,T8754,T8781,T8789,T879,T880XXA,T881XXA,T882XXA<br>T883XXA,T884XXA,T8851XA,T8852XA,T8853XA,T8859XA,T886XXA<br>T887XXA,T888XXA,T889XXA | 9950,99522,99524,9954,99586,99589,9962,<br>99649,99651,99653<br>99654,99655,99656,99657,99659,99668,<br>99669,99670,99675,99679<br>9972,99760,99761,99762,99769,99771,<br>99772,99779,99800,99801<br>99802,99809,99830,99831,99832,99833,<br>9984,99859,9986,9987<br>99881,99889,9989,9990,9991,9992,99931,<br>99932,99933,99934,99939<br>99941,99942,99949,99951,99952,99959,<br>99960,99961,99962,99963<br>99969,99970,99971,99972,99973,99974,<br>99975,99976,99977,99978<br>99979,99980,99981,99982,99983,99984,<br>99985,99988,99989,9999 |
| Complication of other surgical or medical care, injury, subsequent encounter | T800XXD,T801XXD,T80211D,T80212D,T80218D,T80219D,T8022XD<br>T8029XD,T8030XD,T80310D,T80311D,T80319D,T8039XD,T8040XD<br>T80410D,T80411D,T80419D,T8049XD,T8051XD,T8052XD,T8059XD<br>T8061XD,T8062XD,T8069XD,T80810D,T80818D,T8082XD,T8089XD<br>T8090XD,T80910D,T80911D,T80919D,T8092XD,T80A0XD,T80A10D<br>T80A11D,T80A19D,T80A9XD,T8110XD,T8111XD,T8112XD,T8119XD<br>T8130XD,T8131XD,T8132XD,T8133XD,T8140XD,T8141XD,T8142XD<br>T8143XD,T8144XD,T8149XD,T814XXD,T81500D,T81501D,T81502D<br>T81503D,T81504D,T81505D,T81506D,T81507D,T81508D,T81509D<br>T81510D,T81511D,T81512D,T81513D,T81514D,T81515D,T81516D | V5889 |

Continued on next page

Table S9 (continued).

| Diagnosis | ICD-10 | ICD-9 |
|---|---|---|
| | T81517D,T81518D,T81519D,T81520D,T81521D,T81522D,T81523D T81524D,T81525D,T81526D,T81527D,T81528D,T81529D,T81530D T81531D,T81532D,T81533D,T81534D,T81535D,T81536D,T81537D T81538D,T81539D,T81590D,T81591D,T81592D,T81593D,T81594D T81595D,T81596D,T81597D,T81598D,T81599D,T8160XD,T8161XD T8169XD,T81710D,T81711D,T81718D,T81719D,T8172XD,T8181XD T8182XD,T8183XD,T8189XD,T819XXD,T84410D,T84420D,T84490D T8501XD,T8502XD,T8503XD,T8509XD,T85110D,T85111D,T85112D T85113D,T85118D,T85120D,T85121D,T85122D,T85123D,T85128D T85190D,T85191D,T85192D,T85193D,T85199D,T8521XD,T8522XD T8529XD,T85310D,T85311D,T85318D,T85320D,T85321D,T85328D T85390D,T85391D,T85398D,T8541XD,T8542XD,T8543XD,T8544XD T8549XD,T85510D,T85511D,T85518D,T85520D,T85521D,T85528D T85590D,T85591D,T85598D,T85610D,T85611D,T85612D,T85613D T85614D,T85615D,T85618D,T85620D,T85621D,T85622D,T85623D T85624D,T85625D,T85628D,T85630D,T85631D,T85633D,T85635D T85638D,T85690D,T85691D,T85692D,T85693D,T85694D,T85695D T85698D,T8571XD,T8572XD,T85730D,T85731D,T85732D,T85733D T85734D,T85735D,T85738D,T8579XD,T85810D,T85818D,T8581XD T85820D,T85828D,T8582XD,T85830D,T85838D,T8583XD,T85840D T85848D,T8584XD,T85850D,T85858D,T8585XD,T85860D,T85868D T8586XD,T85890D,T85898D,T8589XD,T859XXD,T880XXD,T881XXD T882XXD,T883XXD,T884XXD,T8851XD,T8852XD,T8853XD,T8859XD T886XXD,T887XXD,T888XXD,T889XXD | |
| Complication of transplanted organs or tissue, initial encounter | D89810,D89811,D89812,D89813,M3111,T8600,T8601,T8602,T8603 T8609,T8610,T8611,T8612,T8613,T8619,T8620,T8621,T8622,T8623 T86290,T86298,T8630,T8631,T8632,T8633,T8639,T8640,T8641,T8642 T8643,T8649,T865,T86810,T86811,T86812,T86818,T86819,T86820 T86821,T86822,T86828,T86829,T86830,T86831,T86832,T86838 T86839,T86840,T868401,T868402,T868403,T868409,T86841,T868411 T868412,T868413,T868419,T86842,T868421,T868422,T868423 T868429,T86848,T868481,T868482,T868483,T868489,T86849,T868491 T868492,T868493,T868499,T86850,T86851,T86852,T86858,T86859 T86890,T86891,T86892,T86898,T86899,T8690,T8691,T8692,T8693 T8699,T870X1,T870X2,T870X9,T871X1,T871X2,T871X9,T872 | 27950,27951,27952,27953,99651,99652, 99669,99679,99680,99681 99682,99683,99684,99685,99686,99687, 99688,99689,99694,99696 99699 |
| Complication, sequela | T800XXS,T801XXS,T80211S,T80212S,T80218S,T80219S,T8022XS T8029XS,T8030XS,T80310S,T80311S,T80319S,T8039XS,T8040XS T80410S,T80411S,T80419S,T8049XS,T8051XS,T8052XS,T8059XS T8061XS,T8062XS,T8069XS,T80810S,T80818S,T8082XS,T8089XS T8090XS,T80910S,T80911S,T80919S,T8092XS,T80A0XS,T80A10S T80A11S,T80A19S,T80A9XS,T8110XS,T8111XS,T8112XS,T8119XS T8130XS,T8131XS,T8132XS,T8133XS,T8140XS,T8141XS,T8142XS T8143XS,T8144XS,T8149XS,T814XXS,T81500S,T81501S,T81502S T81503S,T81504S,T81505S,T81506S,T81507S,T81508S,T81509S T81510S,T81511S,T81512S,T81513S,T81514S,T81515S,T81516S T81517S,T81518S,T81519S,T81520S,T81521S,T81522S,T81523S T81524S,T81525S,T81526S,T81527S,T81528S,T81529S,T81530S T81531S,T81532S,T81533S,T81534S,T81535S,T81536S,T81537S T81538S,T81539S,T81590S,T81591S,T81592S,T81593S,T81594S T81595S,T81596S,T81597S,T81598S,T81599S,T8160XS,T8161XS T8169XS,T81710S,T81711S,T81718S,T81719S,T8172XS,T8181XS T8182XS,T8183XS,T8189XS,T819XXS,T8201XS,T8202XS,T8203XS T8209XS,T82110S,T82111S,T82118S,T82119S,T82120S,T82121S T82128S,T82129S,T82190S,T82191S,T82198S,T82199S,T82211S T82212S,T82213S,T82218S,T82221S,T82222S,T82223S,T82228S T82310S,T82311S,T82312S,T82318S,T82319S,T82320S,T82321S T82322S,T82328S,T82329S,T82330S,T82331S,T82332S,T82338S T82339S,T82390S,T82391S,T82392S,T82398S,T82399S,T8241XS T8242XS,T8243XS,T8249XS,T82510S,T82511S,T82512S,T82513S T82514S,T82515S,T82518S,T82519S,T82520S,T82521S,T82522S T82523S,T82524S,T82525S,T82528S,T82529S,T82530S,T82531S T82532S,T82533S,T82534S,T82535S,T82538S,T82539S,T82590S T82591S,T82592S,T82593S,T82594S,T82595S,T82598S,T82599S T826XXS,T827XXS,T82817S,T82818S,T82827S,T82828S,T82837S T82838S,T82847S,T82848S,T82855S,T82856S,T82857S,T82858S T82867S,T82868S,T82897S,T82898S,T829XXS,T83010S,T83011S T83012S,T83018S,T83020S,T83021S,T83022S,T83028S,T83030S T83031S,T83032S,T83038S,T83090S,T83091S,T83092S,T83098S T83110S,T83111S,T83112S,T83113S,T83118S,T83120S,T83121S T83122S,T83123S,T83128S,T83190S,T83191S,T83192S,T83193S T83198S,T8321XS,T8322XS,T8323XS,T8324XS,T8325XS,T8329XS T8331XS,T8332XS,T8339XS,T83410S,T83411S,T83418S,T83420S T83421S,T83428S,T83490S,T83491S,T83498S,T83510S,T83511S T83512S,T83518S,T8351XS,T83590S,T83591S,T83592S,T83593S T83598S,T8359XS,T8361XS,T8362XS,T8369XS,T836XXS,T83711S T83712S,T83713S,T83714S,T83718S,T83719S,T83721S,T83722S T83723S,T83724S,T83728S,T83729S,T8379XS,T8381XS,T8382XS T8383XS,T8384XS,T8385XS,T8386XS,T8389XS,T839XXS,T84010S T84011S,T84012S,T84013S,T84018S,T84019S,T84020S,T84021S T84022S,T84023S,T84028S,T84029S,T84030S,T84031S,T84032S T84033S,T84038S,T84039S,T84050S,T84051S,T84052S,T84053S | 9093,9095 |

Continued on next page

**Table S9 (continued).**

| Diagnosis | ICD-10 | ICD-9 |
|---|---|---|
| | T84058S,T84059S,T84060S,T84061S,T84062S,T84063S,T84068S<br>T84069S,T84090S,T84091S,T84092S,T84093S,T84098S,T84099S<br>T84110S,T84111S,T84112S,T84113S,T84114S,T84115S,T84116S<br>T84117S,T84119S,T84120S,T84121S,T84122S,T84123S,T84124S<br>T84125S,T84126S,T84127S,T84129S,T84190S,T84191S,T84192S<br>T84193S,T84194S,T84195S,T84196S,T84197S,T84199S,T84210S<br>T84213S,T84216S,T84218S,T84220S,T84223S,T84226S,T84228S<br>T84290S,T84293S,T84296S,T84298S,T84310S,T84318S,T84320S<br>T84328S,T84390S,T84398S,T84410S,T84418S,T84420S,T84428S<br>T84490S,T84498S,T8450XS,T8451XS,T8452XS,T8453XS,T8454XS<br>T8459XS,T8460XS,T84610S,T84611S,T84612S,T84613S,T84614S<br>T84615S,T84619S,T84620S,T84621S,T84622S,T84623S,T84624S<br>T84625S,T84629S,T8463XS,T8469XS,T847XXS,T8481XS,T8482XS<br>T8483XS,T8484XS,T8485XS,T8486XS,T8489XS,T849XXS,T8501XS<br>T8502XS,T8503XS,T8509XS,T85110S,T85111S,T85112S,T85113S<br>T85118S,T85120S,T85121S,T85122S,T85123S,T85128S,T85190S<br>T85191S,T85192S,T85193S,T85199S,T8521XS,T8522XS,T8529XS<br>T85310S,T85311S,T85318S,T85320S,T85321S,T85328S,T85390S<br>T85391S,T85398S,T8541XS,T8542XS,T8543XS,T8544XS,T8549XS<br>T85510S,T85511S,T85518S,T85520S,T85521S,T85528S,T85590S<br>T85591S,T85598S,T85610S,T85611S,T85612S,T85613S,T85614S<br>T85615S,T85618S,T85620S,T85621S,T85622S,T85623S,T85624S<br>T85625S,T85628S,T85630S,T85631S,T85633S,T85635S,T85638S<br>T85690S,T85691S,T85692S,T85693S,T85694S,T85695S,T85698S<br>T8571XS,T8572XS,T85730S,T85731S,T85732S,T85733S,T85734S<br>T85735S,T85738S,T8579XS,T85810S,T85818S,T8581XS,T85820S<br>T85828S,T8582XS,T85830S,T85838S,T8583XS,T85840S,T85848S<br>T8584XS,T85850S,T85858S,T8585XS,T85860S,T85868S,T8586XS<br>T85890S,T85898S,T8589XS,T859XXS,T880XXS,T881XXS,T882XXS<br>T883XXS,T884XXS,T8851XS,T8852XS,T8853XS,T8859XS,T886XXS<br>T887XXS,T888XXS,T889XXS | |
| Complications | O080,O081,O082,O083,O084,O085,O086,O087,O0881,O0882,O0883 | 6390,6391,6392,6393,6394,6395,6396,<br>6398,6399 |
| following ectopic and or molar pregnancy | O0889,O089 | |
| Complications of acute myocardial infarction | I230,I231,I232,I233,I234,I235,I236,I237,I238,I241 | 4110,4295,4296,42971,42979 |
| Complications | A34,O1002,O1012,O1022,O1032,O1042,O1092,O114,O1204,O1214 | 6390,64131,64181,64191,64201,64211,<br>64221,64261,64671,64681 |
| specified during | O1224,O134,O1404,O1414,O1424,O1494,O151,O164,O2402,O2412 | 64701,64711,64721,64731,64741,64761,<br>64781,64791,64801,64821 |
| childbirth | O2432,O24420,O24424,O24425,O24429,O2482,O2492,O252,O2662 | 64831,64841,64881,64891,64901,64911,<br>64921,64931,64981,64982 |
| | O2672,O670,O678,O679,O711,O713,O714,O715,O716,O717,O7181 | 65681,65831,65921,65931,65971,65981,<br>65991,66451,66454,66511 |
| | O7189,O719,O730,O731,O740,O741,O742,O743,O744,O745,O746,O747 | 66531,66534,66541,66544,66551,66554,<br>66561,66564,66571,66572 |
| | O748,O749,O750,O751,O752,O753,O754,O755,O7581,O7582,O7589 | 66574,66581,66582,66584,66591,66592,<br>66594,66700,66702,66704 |
| | O759,O76,O770,O82,O860,O8600,O8601,O8602,O8603,O8604,O8609 | 66710,66712,66714,66801,66802,66811,<br>66812,66821,66822,66881 |
| | O8802,O8812,O8822,O8832,O8882,O9802,O9812,O9822,O9832,O9842 | 66882,66891,66892,66901,66902,66904,<br>66911,66912,66914,66941 |
| | O9852,O9862,O9872,O9882,O9892,O9902,O9912,O99214,O99284 | 66942,66944,66971,66981,66982,66984,<br>66991,66992,66994,67301 |
| | O99314,O99324,O99334,O99344,O99354,O9942,O9952,O9962,O9972 | 67302,67311,67312,67321,67322,67331,<br>67332,67381,67382,67401 |
| | O99814,O99824,O99834,O99844,O99892,O9A12,O9A22,O9A32,O9A42<br>O9A52 | 67402,67432,67434,V0251 |
| Complications | F53,F530,F531,M830,O1003,O1013,O1023,O1033,O1043,O1093,O115 | 2682,29389,64204,64214,64224,64244,<br>64264,64272,64274,64644 |
| specified during the | O1205,O1215,O1225,O135,O1405,O1415,O1425,O1495,O152,O165 | 64664,64682,64684,64702,64704,64712,<br>64714,64722,64724,64732 |
| puerperium | O2403,O2413,O2433,O24430,O24434,O24435,O24439,O2483,O2493 | 64734,64742,64744,64762,64764,64782,<br>64784,64792,64794,64802 |
| | O253,O2663,O2673,O712,O720,O721,O722,O723,O85,O8611,O8612 | 64804,64812,64814,64822,64824,64832,<br>64834,64842,64844,64882 |
| | O8613,O8619,O8620,O8621,O8622,O8629,O864,O8681,O8689,O870 | 64884,64894,64902,64904,64912,64914,<br>64922,64924,64934,66522 |
| | O871,O872,O873,O874,O878,O879,O8803,O8813,O8823,O8833,O8883 | 66524,66602,66604,66612,66614,66622,<br>66624,66632,66634,66802 |
| | O8901,O8909,O891,O892,O893,O894,O895,O896,O898,O899,O900,O901 | 66804,66814,66824,66882,66884,66892,<br>66894,66932,66934,67012 |
| | O902,O904,O905,O906,O9081,O9089,O909,O9102,O9103,O9112,O9113 | 67014,67022,67024,67032,67034,67082,<br>67084,67102,67104,67122 |
| | O9122,O9123,O9202,O9203,O9212,O9213,O9220,O9229,O923,O924 | 67124,67142,67144,67152,67154,67182,<br>67184,67192,67194,67202 |
| | O925,O926,O9270,O9279,O9803,O9813,O9823,O9833,O9843,O9853 | 67204,67302,67304,67312,67314,67322,<br>67324,67332,67334,67382 |
| | O9863,O9873,O9883,O9893,O9903,O9913,O99215,O99285,O99315 | 67384,67404,67412,67414,67422,67424,<br>67432,67444,67482,67484 |

Continued on next page

Table S9 (continued).

| Diagnosis | ICD-10 | ICD-9 |
|---|---|---|
| | O99325,O99335,O99345,O99355,O9943,O9953,O9963,O9973,O99815 | 67492,67494,67502,67504,67512,67514, 67522,67524,67582,67584 |
| | O99825,O99835,O99845,O99893,O9A13,O9A23,O9A33,O9A43,O9A53 | 67602,67604,67612,67614,67622,67632, 67634,67642,67644,67652 |
| | | 67654,67662,67664,67682,67684,67692, 67694,67904,V0251 |
| Conditions due to | D61810,D630,D6481,D701,E883,G131,G731,G893,H4742,H47521 | 27788,28411,28522,2853,28803,3383,3573, 35831,37752,37761 |
| neoplasm or the | H47522,H47529,H47631,H47632,H47639,J910,K1231,M360,M361 | 37771,51181,52801,7103,7138,73310, 73311,73312,73313,73314 |
| treatment of neoplasm | M8450XA,M84511A,M84512A,M84519A,M84521A,M84522A,M84529A | 73315,73316,73319,78079,78951,9093, 99981,V5889 |
| | M84531A,M84532A,M84533A,M84534A,M84539A,M84541A,M84542A M84549A,M84550A,M84551A,M84552A,M84553A,M84559A,M84561A M84562A,M84563A,M84564A,M84569A,M84571A,M84572A,M84573A M84574A,M84575A,M84576A,M8458XA,N5236,R180,R530,T80810A T80810D,T80810S | |
| Conduction disorders | I440,I441,I442,I4430,I4439,I444,I445,I4460,I4469,I447,I450 | 4260,42610,42611,42612,42613,4262,4263, 4264,42650,42651,42652 |
| | I4510,I4519,I452,I453,I454,I455,I456,I4581,I4589,I459,Z45010 | 42653,42654,4266,4267,42681,42682, 42689,4269,V4501,V5331 |
| | Z45018,Z950 | |
| Congenital | Q100,Q101,Q102,Q103,Q104,Q105,Q106,Q107,Q110,Q111,Q112,Q113 | 36514,74300,74303,74306,74310,74320, 74321,74322,74330,74335 |
| malformations of eye, | Q120,Q121,Q122,Q123,Q124,Q128,Q129,Q130,Q131,Q132,Q133,Q134 | 74336,74337,74339,74341,74343,74344, 74345,74346,74347,74349 |
| ear, face, neck | Q135,Q1381,Q1389,Q139,Q140,Q141,Q142,Q143,Q148,Q149,Q150,Q158 | 74351,74352,74353,74356,74357,74359, 74361,74362,74363,74365 |
| | Q159,Q160,Q161,Q162,Q163,Q164,Q165,Q169,Q170,Q171,Q172,Q173 | 74366,74369,7438,7439,74400,74401, 74402,74403,74404,74405 |
| | Q174,Q175,Q178,Q179,Q180,Q181,Q182,Q183,Q184,Q185,Q186,Q187 | 74409,7441,74421,74422,74423,74424, 74429,7443,74441,74442 |
| | Q188,Q189 | 74443,74446,74447,74449,7445,74481, 74482,74483,74484,74489 |
| | | 7449 |
| Contact dermatitis | L22,L230,L231,L232,L233,L234,L235,L236,L237,L2381,L2389,L239 | 6910,6920,6921,6922,6923,6924,6925, 6926,69270,69274,69279 |
| | L240,L241,L242,L243,L244,L245,L246,L247,L2481,L2489,L249 L24A0,L24A1,L24A2,L24A9,L24B0,L24B1,L24B2,L24B3,L250,L251 L252,L253,L254,L255,L258,L259,L568,L569,L578,L579,L580,L581 L589,L590,L598,L599 | 69281,69282,69283,69284,69289,6929 |
| Contraceptive and | Z30011,Z30012,Z30013,Z30014,Z30015,Z30016,Z30017,Z30018 | V2501,V2502,V2503,V2504,V2509, V2511,V2512,V2513,V252,V253 |
| procreative | Z30019,Z3002,Z3009,Z302,Z3040,Z3041,Z3042,Z30430,Z30431 | V2540,V2541,V2542,V2543,V2549,V255, V258,V259,V260,V261,V2621 |
| management | Z30432,Z30433,Z3044,Z3045,Z3046,Z3049,Z308,Z309,Z310,Z3141 | V2622,V2629,V2631,V2632,V2633, V2634,V2635,V2639,V2641,V2642 |
| | Z3142,Z31430,Z31438,Z31440,Z31441,Z31448,Z3149,Z315,Z3161 | V2649,V2651,V2652,V2681,V2682, V2689,V269,V4551,V4559 |
| | Z3162,Z3169,Z317,Z3181,Z3182,Z3183,Z3184,Z3189,Z319,Z975 Z9851,Z9852 | |
| Cornea and external | A1851,A1852,A3686,A5031,A5431,A5433,A740,B0052,B0053,B0181 | 01730,03281,0527,05321,05329,05442, 05443,05449,05571,0770 |
| disease | B0231,B0233,B0234,B0581,B300,B301,B302,B303,B308,B309,B6012 | 0771,0772,0773,0774,0778,07798,07799, 0903,09840,09843,13621 |
| | B6013,H00011,H00012,H00013,H00014,H00015,H00016,H00019,H00021 | 36014,37000,37001,37002,37003,37004, 37005,37006,37007,37020 |
| | H00022,H00023,H00024,H00025,H00026,H00029,H00031,H00032 | 37021,37022,37023,37024,37031,37032, 37033,37034,37035,37040 |
| | H00033,H00034,H00035,H00036,H00039,H0011,H0012,H0013,H0014 | 37044,37049,37050,37052,37054,37055, 37059,37060,37061,37062 |
| | H0015,H0016,H0019,H01001,H01002,H01003,H01004,H01005,H01006 | 37063,37064,3708,3709,37100,37101, 37102,37103,37104,37110 |
| | H01009,H0100A,H0100B,H01011,H01012,H01013,H01014,H01015 | 37111,37112,37113,37114,37115,37116, 37120,37121,37122,37123 |
| | H01016,H01019,H0101A,H0101B,H01021,H01022,H01023,H01024 | 37124,37130,37131,37132,37133,37140, 37141,37142,37143,37144 |
| | H01025,H01026,H01029,H0102A,H0102B,H01111,H01112,H01113 | 37145,37146,37148,37149,37150,37151, 37152,37153,37154,37155 |
| | H01114,H01115,H01116,H01119,H01121,H01122,H01123,H01124 | 37157,37160,37161,37162,37170,37171, 37172,37173,37181,37182 |
| | H01125,H01126,H01129,H01131,H01132,H01133,H01134,H01135 | 37189,3719,37200,37201,37202,37203, 37204,37205,37206,37210 |
| | H01136,H01139,H01141,H01142,H01143,H01144,H01145,H01146 | 37211,37212,37213,37214,37215,37220, 37221,37222,37230,37234 |
| | H01149,H018,H019,H02001,H02002,H02003,H02004,H02005,H02006 | 37239,37240,37241,37242,37243,37244, 37245,37250,37251,37252 |
| | H02009,H02011,H02012,H02013,H02014,H02015,H02016,H02019 | 37253,37254,37255,37256,37261,37262, 37263,37264,37271,37272 |
| | H02021,H02022,H02023,H02024,H02025,H02026,H02029,H02031 | 37273,37274,37275,37281,37289,3729, 37300,37301,37302,37311 |

Continued on next page

Table S9 (continued).

| Diagnosis | ICD-10 | ICD-9 |
|---|---|---|
| | H02032,H02033,H02034,H02035,H02036,H02039,H02041,H02042 | 37312,37313,3732,37331,37332,37333,37334,3738,3739,37400 |
| | H02043,H02044,H02045,H02046,H02049,H02051,H02052,H02053 | 37401,37402,37403,37404,37405,37410,37411,37412,37413,37414 |
| | H02054,H02055,H02056,H02059,H02101,H02102,H02103,H02104 | 37420,37421,37422,37423,37513,37514,37516,37520,37521,37522 |
| | H02105,H02106,H02109,H02111,H02112,H02113,H02114,H02115 | 37589,37900,37901,37902,37903,37904,37905,37906,37907,37909 |
| | H02116,H02119,H02121,H02122,H02123,H02124,H02125,H02126 | 37911,37912,37913,37914,37915,37916,37919,7102 |
| | H02129,H02131,H02132,H02133,H02134,H02135,H02136,H02139 | |
| | H02141,H02142,H02143,H02144,H02145,H02146,H02149,H02151 | |
| | H02152,H02153,H02154,H02155,H02156,H02159,H02201,H02202 | |
| | H02203,H02204,H02205,H02206,H02209,H0220A,H0220B,H0220C | |
| | H02211,H02212,H02213,H02214,H02215,H02216,H02219,H0221A | |
| | H0221B,H0221C,H02221,H02222,H02223,H02224,H02225,H02226 | |
| | H02229,H0222A,H0222B,H0222C,H02231,H02232,H02233,H02234 | |
| | H02235,H02236,H02239,H0223A,H0223B,H0223C,H02881,H02882 | |
| | H02883,H02884,H02885,H02886,H02889,H0288A,H0288B,H04141 | |
| | H04142,H04143,H04149,H04151,H04152,H04153,H04159,H04161 | |
| | H04162,H04163,H04169,H0419,H04201,H04202,H04203,H04209,H04211 | |
| | H04212,H04213,H04219,H04221,H04222,H04223,H04229,H10011 | |
| | H10012,H10013,H10019,H10021,H10022,H10023,H10029,H1010,H1011 | |
| | H1012,H1013,H10211,H10212,H10213,H10219,H10221,H10222,H10223 | |
| | H10229,H10231,H10232,H10233,H10239,H1030,H1031,H1032,H1033 | |
| | H10401,H10402,H10403,H10409,H10411,H10412,H10413,H10419 | |
| | H10421,H10422,H10423,H10429,H10431,H10432,H10433,H10439,H1044 | |
| | H1045,H10501,H10502,H10503,H10509,H10511,H10512,H10513,H10519 | |
| | H10521,H10522,H10523,H10529,H10531,H10532,H10533,H10539 | |
| | H10811,H10812,H10813,H10819,H10821,H10822,H10823,H10829,H1089 | |
| | H109,H11001,H11002,H11003,H11009,H11011,H11012,H11013,H11019 | |
| | H11021,H11022,H11023,H11029,H11031,H11032,H11033,H11039 | |
| | H11041,H11042,H11043,H11049,H11051,H11052,H11053,H11059 | |
| | H11061,H11062,H11063,H11069,H1110,H11111,H11112,H11113,H11119 | |
| | H11121,H11122,H11123,H11129,H11131,H11132,H11133,H11139 | |
| | H11141,H11142,H11143,H11149,H11151,H11152,H11153,H11159 | |
| | H11211,H11212,H11213,H11219,H11221,H11222,H11223,H11229 | |
| | H11231,H11232,H11233,H11239,H11241,H11242,H11243,H11249,H1130 | |
| | H1131,H1132,H1133,H11411,H11412,H11413,H11419,H11421,H11422 | |
| | H11423,H11429,H11431,H11432,H11433,H11439,H11441,H11442 | |
| | H11443,H11449,H11811,H11812,H11813,H11819,H11821,H11822 | |
| | H11823,H11829,H1189,H119,H15001,H15002,H15003,H15009,H15011 | |
| | H15012,H15013,H15019,H15021,H15022,H15023,H15029,H15031 | |
| | H15032,H15033,H15039,H15041,H15042,H15043,H15049,H15051 | |
| | H15052,H15053,H15059,H15091,H15092,H15093,H15099,H15101 | |
| | H15102,H15103,H15109,H15111,H15112,H15113,H15119,H15121 | |
| | H15122,H15123,H15129,H15811,H15812,H15813,H15819,H15821 | |
| | H15822,H15823,H15829,H15831,H15832,H15833,H15839,H15841 | |
| | H15842,H15843,H15849,H15851,H15852,H15853,H15859,H1589,H159 | |
| | H16001,H16002,H16003,H16009,H16011,H16012,H16013,H16019 | |
| | H16021,H16022,H16023,H16029,H16031,H16032,H16033,H16039 | |
| | H16041,H16042,H16043,H16049,H16051,H16052,H16053,H16059 | |
| | H16061,H16062,H16063,H16069,H16071,H16072,H16073,H16079 | |
| | H16101,H16102,H16103,H16109,H16111,H16112,H16113,H16119 | |
| | H16121,H16122,H16123,H16129,H16131,H16132,H16133,H16139 | |
| | H16141,H16142,H16143,H16149,H16201,H16202,H16203,H16209 | |
| | H16211,H16212,H16213,H16219,H16221,H16222,H16223,H16229 | |
| | H16231,H16232,H16233,H16239,H16241,H16242,H16243,H16249 | |
| | H16251,H16252,H16253,H16259,H16261,H16262,H16263,H16269 | |
| | H16291,H16292,H16293,H16299,H16301,H16302,H16303,H16309 | |
| | H16311,H16312,H16313,H16319,H16321,H16322,H16323,H16329 | |
| | H16331,H16332,H16333,H16339,H16391,H16392,H16393,H16399 | |
| | H16401,H16402,H16403,H16409,H16411,H16412,H16413,H16419 | |
| | H16421,H16422,H16423,H16429,H16431,H16432,H16433,H16439 | |
| | H16441,H16442,H16443,H16449,H168,H169,H1700,H1701,H1702,H1703 | |
| | H1710,H1711,H1712,H1713,H17811,H17812,H17813,H17819,H17821 | |
| | H17822,H17823,H17829,H1789,H179,H18001,H18002,H18003,H18009 | |
| | H18011,H18012,H18013,H18019,H18021,H18022,H18023,H18029 | |
| | H18031,H18032,H18033,H18039,H18041,H18042,H18043,H18049 | |
| | H18051,H18052,H18053,H18059,H18061,H18062,H18063,H18069,H1810 | |
| | H1811,H1812,H1813,H1820,H18211,H18212,H18213,H18219,H18221 | |
| | H18222,H18223,H18229,H18231,H18232,H18233,H18239,H1830,H18311 | |
| | H18312,H18313,H18319,H18321,H18322,H18323,H18329,H18331 | |
| | H18332,H18333,H18339,H1840,H18411,H18412,H18413,H18419,H18421 | |
| | H18422,H18423,H18429,H1843,H18441,H18442,H18443,H18449,H18451 | |
| | H18452,H18453,H18459,H18461,H18462,H18463,H18469,H1849,H1850 | |
| | H18501,H18502,H18503,H18509,H1851,H18511,H18512,H18513,H18519 | |
| | H1852,H18521,H18522,H18523,H18529,H1853,H18531,H18532,H18533 | |
| | H18539,H1854,H18541,H18542,H18543,H18549,H1855,H18551,H18552 | |
| | H18553,H18559,H1859,H18591,H18592,H18593,H18599,H18601,H18602 | |
| | H18603,H18609,H18611,H18612,H18613,H18619,H18621,H18622 | |
| | H18623,H18629,H1870,H18711,H18712,H18713,H18719,H18721,H18722 | |
| | H18723,H18729,H18731,H18732,H18733,H18739,H18791,H18792 | |
| | H18793,H18799,H18811,H18812,H18813,H18819,H18821,H18822 | |
| | H18823,H18829,H18831,H18832,H18833,H18839,H18891,H18892 | |

Continued on next page

Table S9 (continued).

| Diagnosis | ICD-10 | ICD-9 |
|---|---|---|
| | H18893,H18899,H189,M3501 | |
| Coronary atherosclerosis and other heart disease | I200,I201,I202,I208,I209,I240,I248,I249,I2510,I25110,I25111 I25112,I25118,I25119,I252,I255,I256,I25700,I25701,I25702 I25708,I25709,I25710,I25711,I25712,I25718,I25719,I25720 I25721,I25722,I25728,I25729,I25730,I25731,I25732,I25738 I25739,I25750,I25751,I25752,I25758,I25759,I25760,I25761 I25762,I25768,I25769,I25790,I25791,I25792,I25798,I25799 I25810,I25811,I25812,I2582,I2583,I2584,I2589,I259,Z951,Z955 Z9861 | 4111,41181,41189,412,4131,4139,41401, 41402,41403,41404,41405 41406,41407,4142,4143,4144,4148,4149, 4292,V4581,V4582 |
| Crushing injury, initial encounter | S070XXA,S071XXA,S078XXA,S079XXA,S170XXA,S178XXA,S179XXA S280XXA,S38001A,S38002A,S3801XA,S3802XA,S3803XA,S381XXA S471XXA,S472XXA,S479XXA,S5700XA,S5701XA,S5702XA,S5780XA S5781XA,S5782XA,S6700XA,S6701XA,S6702XA,S6710XA,S67190A S67191A,S67192A,S67193A,S67194A,S67195A,S67196A,S67197A S67198A,S6720XA,S6721XA,S6722XA,S6730XA,S6731XA,S6732XA S6740XA,S6741XA,S6742XA,S6790XA,S6791XA,S6792XA,S7700XA S7701XA,S7702XA,S7710XA,S7711XA,S7712XA,S7720XA,S7721XA S7722XA,S8700XA,S8701XA,S8702XA,S8780XA,S8781XA,S8782XA S9700XA,S9701XA,S9702XA,S97101A,S97102A,S97109A,S97111A S97112A,S97119A,S97121A,S97122A,S97129A,S9780XA,S9781XA S9782XA | 9251,9252,9260,92611,92619,92700,92701, 92702,92703,92710 92711,92720,92721,9273,9278,9279,92800, 92801,92810,92811 92820,92821,9283,9288,9289,9290,9299 |
| Crushing injury, subsequent encounter | S070XXD,S071XXD,S078XXD,S079XXD,S170XXD,S178XXD,S179XXD S280XXD,S38001D,S38002D,S3801XD,S3802XD,S3803XD,S381XXD S471XXD,S472XXD,S479XXD,S5700XD,S5701XD,S5702XD,S5780XD S5781XD,S5782XD,S6700XD,S6701XD,S6702XD,S6710XD,S67190D S67191D,S67192D,S67193D,S67194D,S67195D,S67196D,S67197D S67198D,S6720XD,S6721XD,S6722XD,S6730XD,S6731XD,S6732XD S6740XD,S6741XD,S6742XD,S6790XD,S6791XD,S6792XD,S7700XD S7701XD,S7702XD,S7710XD,S7711XD,S7712XD,S7720XD,S7721XD S7722XD,S8700XD,S8701XD,S8702XD,S8780XD,S8781XD,S8782XD S9700XD,S9701XD,S9702XD,S97101D,S97102D,S97109D,S97111D S97112D,S97119D,S97121D,S97122D,S97129D,S9780XD,S9781XD S9782XD | V5889 |
| Crystal arthropathies (excluding gout) | M1100,M11011,M11012,M11019,M11021,M11022,M11029,M11031,M11032 M11039,M11041,M11042,M11049,M11051,M11052,M11059,M11061 M11062,M11069,M11071,M11072,M11079,M1108,M1109,M1110,M11111 M11112,M11119,M11121,M11122,M11129,M11131,M11132,M11139 M11141,M11142,M11149,M11151,M11152,M11159,M11161,M11162 M11169,M11171,M11172,M11179,M1118,M1119,M1120,M11211,M11212 M11219,M11221,M11222,M11229,M11231,M11232,M11239,M11241 M11242,M11249,M11251,M11252,M11259,M11261,M11262,M11269 M11271,M11272,M11279,M1128,M1129,M1180,M11811,M11812,M11819 M11821,M11822,M11829,M11831,M11832,M11839,M11841,M11842 M11849,M11851,M11852,M11859,M11861,M11862,M11869,M11871 M11872,M11879,M1188,M1189,M119 | 71230,71231,71232,71233,71234,71235, 71236,71237,71238,71239 71280,71281,71282,71283,71284,71285, 71286,71287,71288,71289 71290,71291,71292,71293,71294,71295, 71296,71297,71298 |
| Cystic fibrosis | E840,E8411,E8419,E848,E849,P094 | 27700,27701,27702,27703,27709 |
| Depressive disorders | F0631,F0632,F0634,F320,F321,F322,F323,F324,F328,F3281,F3289 F329,F32A,F330,F331,F332,F333,F3341,F338,F339,F341,F530,O906 | 29383,29620,29621,29622,29623,29624, 29625,29630,29631,29632 29633,29634,29635,29682,29699,2980, 3004,30112,311,64842,64844 |
| Diabetes mellitus with complication | E0800,E0801,E0810,E0811,E0821,E0822,E0829,E08311,E08319 E08321,E083211,E083212,E083213,E083219,E08329,E083291,E083292 E083293,E083299,E08331,E083311,E083312,E083313,E083319,E08339 E083391,E083392,E083393,E083399,E08341,E083411,E083412 E083413,E083419,E08349,E083491,E083492,E083493,E083499,E08351 E083511,E083512,E083513,E083519,E083521,E083522,E083523 E083529,E083531,E083532,E083533,E083539,E083541,E083542 E083543,E083549,E083551,E083552,E083553,E083559,E08359 E083591,E083592,E083593,E083599,E0836,E0837X1,E0837X2,E0837X3 E0837X9,E0839,E0840,E0841,E0842,E0843,E0844,E0849,E0851,E0852 E0859,E08610,E08618,E08620,E08621,E08622,E08628,E08630,E08638 E08641,E08649,E0865,E0869,E088,E0900,E0901,E0910,E0911,E0921 E0922,E0929,E09311,E09319,E09321,E093211,E093212,E093213 E093219,E09329,E093291,E093292,E093293,E093299,E09331,E093311 E093312,E093313,E093319,E09339,E093391,E093392,E093393 E093399,E09341,E093411,E093412,E093413,E093419,E09349,E093491 E093492,E093493,E093499,E09351,E093511,E093512,E093513 E093519,E093521,E093522,E093523,E093529,E093531,E093532 | 24910,24920,24930,24940,24950,24960, 24970,24980,24990,25010 25011,25020,25030,25031,25040,25041, 25050,25051,25060,25061 25070,25071,25080,25081,25090,25091, 2511,34989,3535,3559,3572 36201,36202,36204,36205,36206,36207, 36641,44381,5238,5289 5363,58181,58381,7098,7135,71680,7854 |

Continued on next page

Table S9 (continued).

| Diagnosis | ICD-10 | ICD-9 |
|---|---|---|
| | E093533,E093539,E093541,E093542,E093543,E093549,E093551 | |
| | E093552,E093553,E093559,E09359,E093591,E093592,E093593 | |
| | E093599,E0936,E0937X1,E0937X2,E0937X3,E0937X9,E0939,E0940 | |
| | E0941,E0942,E0943,E0944,E0949,E0951,E0952,E0959,E09610,E09618 | |
| | E09620,E09621,E09622,E09628,E09630,E09638,E09641,E09649,E0965 | |
| | E0969,E098,E1010,E1011,E1021,E1022,E1029,E10311,E10319,E10321 | |
| | E103211,E103212,E103213,E103219,E10329,E103291,E103292 | |
| | E103293,E103299,E10331,E103311,E103312,E103313,E103319,E10339 | |
| | E103391,E103392,E103393,E103399,E10341,E103411,E103412 | |
| | E103413,E103419,E10349,E103491,E103492,E103493,E103499,E10351 | |
| | E103511,E103512,E103513,E103519,E103521,E103522,E103523 | |
| | E103529,E103531,E103532,E103533,E103539,E103541,E103542 | |
| | E103543,E103549,E103551,E103552,E103553,E103559,E10359 | |
| | E103591,E103592,E103593,E103599,E1036,E1037X1,E1037X2,E1037X3 | |
| | E1037X9,E1039,E1040,E1041,E1042,E1043,E1044,E1049,E1051,E1052 | |
| | E1059,E10610,E10618,E10620,E10621,E10622,E10628,E10630,E10638 | |
| | E10641,E10649,E1065,E1069,E108,E1100,E1101,E1110,E1111,E1121 | |
| | E1122,E1129,E11311,E11319,E11321,E113211,E113212,E113213 | |
| | E113219,E11329,E113291,E113292,E113293,E113299,E11331,E113311 | |
| | E113312,E113313,E113319,E11339,E113391,E113392,E113393 | |
| | E113399,E11341,E113411,E113412,E113413,E113419,E11349,E113491 | |
| | E113492,E113493,E113499,E11351,E113511,E113512,E113513 | |
| | E113519,E113521,E113522,E113523,E113529,E113531,E113532 | |
| | E113533,E113539,E113541,E113542,E113543,E113549,E113551 | |
| | E113552,E113553,E113559,E11359,E113591,E113592,E113593 | |
| | E113599,E1136,E1137X1,E1137X2,E1137X3,E1137X9,E1139,E1140 | |
| | E1141,E1142,E1143,E1144,E1149,E1151,E1152,E1159,E11610,E11618 | |
| | E11620,E11621,E11622,E11628,E11630,E11638,E11641,E11649,E1165 | |
| | E1169,E118,E1300,E1301,E1310,E1311,E1321,E1322,E1329,E13311 | |
| | E13319,E13321,E133211,E133212,E133213,E133219,E13329,E133291 | |
| | E133292,E133293,E133299,E13331,E133311,E133312,E133313 | |
| | E133319,E13339,E133391,E133392,E133393,E133399,E13341,E133411 | |
| | E133412,E133413,E133419,E13349,E133491,E133492,E133493 | |
| | E133499,E13351,E133511,E133512,E133513,E133519,E133521 | |
| | E133522,E133523,E133529,E133531,E133532,E133533,E133539 | |
| | E133541,E133542,E133543,E133549,E133551,E133552,E133553 | |
| | E133559,E13359,E133591,E133592,E133593,E133599,E1336,E1337X1 | |
| | E1337X2,E1337X3,E1337X9,E1339,E1340,E1341,E1342,E1343,E1344 | |
| | E1349,E1351,E1352,E1359,E13610,E13618,E13620,E13621,E13622 | |
| | E13628,E13630,E13638,E13641,E13649,E1365,E1369,E138 | |
| Diabetes mellitus | E089,E099,E109,E119,E139,E891,O24011,O24012,O24013,O24019 | 24900,25000,25001,2513,64800,64801, 64802,64803,64804,64881 |
| without complication | O2402,O2403,O24111,O24112,O24113,O24119,O2412,O2413,O24311 | 64882,64883,64884 |
| | O24312,O24313,O24319,O2432,O2433,O24410,O24414,O24415,O24419 | |
| | O24420,O24424,O24425,O24429,O24430,O24434,O24435,O24439 | |
| | O24811,O24812,O24813,O24819,O2482,O2483,O24911,O24912,O24913 | |
| | O24919,O2492,O2493,O99810,O99814,O99815 | |
| Diabetes or abnormal | O24011,O24012,O24013,O24019,O2402,O2403,O24111,O24112,O24113 | 64800,64801,64802,64803,64804,64881, 64882,64883,64884 |
| glucose tolerance | O24119,O2412,O2413,O24311,O24312,O24313,O24319,O2432,O2433 | |
| complicating | O24410,O24414,O24415,O24419,O24420,O24424,O24425,O24429 | |
| pregnancy; | O24430,O24434,O24435,O24439,O24811,O24812,O24813,O24819,O2482 | |
| childbirth; or the | O2483,O24911,O24912,O24913,O24919,O2492,O2493,O99810,O99814 | |
| puerperium | O99815 | |
| Digestive congenital | Q380,Q381,Q382,Q383,Q384,Q385,Q386,Q387,Q388,Q390,Q391,Q392 | 7500,75010,75011,75012,75013,75015, 75016,75019,75021,75022 |
| anomalies | Q393,Q394,Q395,Q396,Q398,Q399,Q400,Q401,Q402,Q403,Q408,Q409 | 75023,75024,75025,75026,75027,75029, 7503,7504,7505,7506,7507 |
| | Q410,Q411,Q412,Q418,Q419,Q420,Q421,Q422,Q423,Q428,Q429,Q430 | 7508,7509,7510,7511,7512,7513,7514, 7515,75160,75161,75162 |
| | Q431,Q432,Q433,Q434,Q435,Q436,Q437,Q438,Q439,Q440,Q441,Q442 | 75169,7517,7518,7519 |
| | Q443,Q444,Q445,Q446,Q447,Q450,Q451,Q452,Q453,Q458,Q459 | |
| Diseases of inner ear | H8000,H8001,H8002,H8003,H8010,H8011,H8012,H8013,H8020,H8021 | 38600,38601,38602,38603,38604,38610, 38611,38612,38619,3862 |
| and related | H8022,H8023,H8080,H8081,H8082,H8083,H8090,H8091,H8092,H8093 | 38630,38631,38632,38633,38634,38635, 38640,38641,38642,38643 |
| conditions | H8101,H8102,H8103,H8109,H8110,H8111,H8112,H8113,H8120,H8121 | 38648,38650,38651,38652,38653,38654, 38655,38656,38658,3868 |
| | H8122,H8123,H81311,H81312,H81313,H81319,H81391,H81392,H81393 | 3869,3870,3871,3872,3878,3879,38810, 38812 |
| | H81399,H814,H8141,H8142,H8143,H8149,H818X1,H818X2,H818X3 | |
| | H818X9,H8190,H8191,H8192,H8193,H821,H822,H823,H829,H8301 | |
| | H8302,H8303,H8309,H8311,H8312,H8313,H8319,H832X1,H832X2 | |
| | H832X3,H832X9,H833X1,H833X2,H833X3,H833X9,H838X1,H838X2 | |
| | H838X3,H838X9,H8390,H8391,H8392,H8393 | |
| Diseases of middle | A186,H68001,H68002,H68003,H68009,H68011,H68012,H68013,H68019 | 01740,38150,38151,38152,38160,38161, 38162,38163,3817,38181 |
| ear and mastoid | H68021,H68022,H68023,H68029,H68101,H68102,H68103,H68109 | 38189,3819,38300,38301,38302,3831, 38320,38321,38322,38330 |
| (except otitis media) | H68111,H68112,H68113,H68119,H68121,H68122,H68123,H68129 | 38331,38332,38333,38381,38389,3839, 38400,38401,38409,3841 |

Continued on next page

Table S9 (continued).

| Diagnosis | ICD-10 | ICD-9 |
|---|---|---|
| | H68131,H68132,H68133,H68139,H6900,H6901,H6902,H6903,H6980 | 38420,38421,38422,38423,38424,38425, 38481,38482,3849,38500 |
| | H6981,H6982,H6983,H6990,H6991,H6992,H6993,H70001,H70002 | 38501,38502,38503,38509,38510,38511, 38512,38513,38519,38521 |
| | H70003,H70009,H70011,H70012,H70013,H70019,H70091,H70092 | 38522,38523,38524,38530,38531,38532, 38533,38535,38589,3859 |
| | H70093,H70099,H7010,H7011,H7012,H7013,H70201,H70202,H70203 H70209,H70211,H70212,H70213,H70219,H70221,H70222,H70223 H70229,H70811,H70812,H70813,H70819,H70891,H70892,H70893 H70899,H7090,H7091,H7092,H7093,H7100,H7101,H7102,H7103,H7110 H7111,H7112,H7113,H7120,H7121,H7122,H7123,H7130,H7131,H7132 H7133,H7190,H7191,H7192,H7193,H7200,H7201,H7202,H7203,H7210 H7211,H7212,H7213,H722X1,H722X2,H722X3,H722X9,H72811,H72812 H72813,H72819,H72821,H72822,H72823,H72829,H7290,H7291,H7292 H7293,H73001,H73002,H73003,H73009,H73011,H73012,H73013,H73019 H73091,H73092,H73093,H73099,H7310,H7311,H7312,H7313,H7320 H7321,H7322,H7323,H73811,H73812,H73813,H73819,H73821,H73822 H73823,H73829,H73891,H73892,H73893,H73899,H7390,H7391,H7392 H7393,H7401,H7402,H7403,H7409,H7411,H7412,H7413,H7419,H7420 H7421,H7422,H7423,H74311,H74312,H74313,H74319,H74321,H74322 H74323,H74329,H74391,H74392,H74393,H74399,H7440,H7441,H7442 H7443,H748X1,H748X2,H748X3,H748X9,H7490,H7491,H7492,H7493 H7500,H7501,H7502,H7503,H7580,H7581,H7582,H7583,H9500,H9501 H9502,H9503,H95111,H95112,H95113,H95119,H95121,H95122,H95123 H95129,H95131,H95132,H95133,H95139,H95191,H95192,H95193 H95199 | |
| Diseases of mouth; | A690,B002,B084,B370,B3783,K098,K099,K110,K111,K1120,K1121 | 0542,0743,101,1120,5270,5271,5272,5273, 5274,5275,5276,5277 |
| excluding dental | K1122,K1123,K113,K114,K115,K116,K117,K118,K119,K120,K121,K122 | 5278,5279,52800,52801,52802,52809,5281, 5282,5283,5284,5285 |
| | K1230,K1231,K1232,K1233,K1239,K130,K131,K1321,K1322,K1323 | 5286,52871,52872,52879,5288,5289,5290, 5291,5292,5293,5294 |
| | K1324,K1329,K133,K134,K135,K136,K1370,K1379,K140,K141,K142 K143,K144,K145,K146,K148,K149,R682 | 5295,5296,5298,5299,6820 |
| Diseases of white | D700,D701,D702,D703,D704,D708,D709,D71,D720,D721,D7210,D72110 | 28800,28801,28802,28803,28804,28809, 2881,2882,2883,2884,28850 |
| blood cells | D72111,D72118,D72119,D7212,D7218,D7219,D72810,D72818,D72819 | 28851,28859,28860,28861,28862,28863, 28864,28865,28866,28869 |
| | D72820,D72821,D72822,D72823,D72824,D72825,D72828,D72829,D7289 D729,D7381,D761,D762,D763 | 2888,2889,28953 |
| Dislocations, initial | S0300XA,S0301XA,S0302XA,S0303XA,S030XXA,S031XXA,S032XXA | 8300,83100,83101,83102,83103,83104, 83109,83200,83201,83202 |
| encounter | S130XXA,S13100A,S13101A,S13110A,S13111A,S13120A,S13121A | 83203,83204,83209,8322,83300,83301, 83302,83303,83304,83305 |
| | S13130A,S13131A,S13140A,S13141A,S13150A,S13151A,S13160A | 83309,83400,83401,83402,83500,83501, 83502,83503,8363,83650 |
| | S13161A,S13170A,S13171A,S13180A,S13181A,S1320XA,S1329XA | 83651,83652,83653,83654,83659,8370, 83800,83802,83803,83804 |
| | S230XXA,S23100A,S23101A,S23110A,S23111A,S23120A,S23121A | 83805,83806,83809,83900,83901,83902, 83903,83904,83905,83906 |
| | S23122A,S23123A,S23130A,S23131A,S23132A,S23133A,S23140A | 83907,83908,83920,83921,83940,83941, 83942,83949,83961,83969 |
| | S23141A,S23142A,S23143A,S23150A,S23151A,S23152A,S23153A S23160A,S23161A,S23162A,S23163A,S23170A,S23171A,S2320XA S2329XA,S330XXA,S33100A,S33101A,S33110A,S33111A,S33120A S33121A,S33130A,S33131A,S33140A,S33141A,S332XXA,S3330XA S3339XA,S334XXA,S43001A,S43002A,S43003A,S43004A,S43005A S43006A,S43011A,S43012A,S43013A,S43014A,S43015A,S43016A S43021A,S43022A,S43023A,S43024A,S43025A,S43026A,S43031A S43032A,S43033A,S43034A,S43035A,S43036A,S43081A,S43082A S43083A,S43084A,S43085A,S43086A,S43101A,S43102A,S43109A S43111A,S43112A,S43119A,S43121A,S43122A,S43129A,S43131A S43132A,S43139A,S43141A,S43142A,S43149A,S43151A,S43152A S43159A,S43201A,S43202A,S43203A,S43204A,S43205A,S43206A S43211A,S43212A,S43213A,S43214A,S43215A,S43216A,S43221A S43222A,S43223A,S43224A,S43225A,S43226A,S43301A,S43302A S43303A,S43304A,S43305A,S43306A,S43311A,S43312A,S43313A S43314A,S43315A,S43316A,S43391A,S43392A,S43393A,S43394A S43395A,S43396A,S53001A,S53002A,S53003A,S53004A,S53005A S53006A,S53011A,S53012A,S53013A,S53014A,S53015A,S53016A S53021A,S53022A,S53023A,S53024A,S53025A,S53026A,S53031A S53032A,S53033A,S53091A,S53092A,S53093A,S53094A,S53095A S53096A,S53101A,S53102A,S53103A,S53104A,S53105A,S53106A S53111A,S53112A,S53113A,S53114A,S53115A,S53116A,S53121A S53122A,S53123A,S53124A,S53125A,S53126A,S53131A,S53132A S53133A,S53134A,S53135A,S53136A,S53141A,S53142A,S53143A S53144A,S53145A,S53146A,S53191A,S53192A,S53193A,S53194A S53195A,S53196A,S63001A,S63002A,S63003A,S63004A,S63005A S63006A,S63011A,S63012A,S63013A,S63014A,S63015A,S63016A S63021A,S63022A,S63023A,S63024A,S63025A,S63026A,S63031A S63032A,S63033A,S63034A,S63035A,S63036A,S63041A,S63042A S63043A,S63044A,S63045A,S63046A,S63051A,S63052A,S63053A S63054A,S63055A,S63056A,S63061A,S63062A,S63063A,S63064A | 8480,8485,87363 |

Continued on next page

Table S9 (continued).

| Diagnosis | ICD-10 | ICD-9 |
|---|---|---|
| | S63065A,S63066A,S63071A,S63072A,S63073A,S63074A,S63075A<br>S63076A,S63091A,S63092A,S63093A,S63094A,S63095A,S63096A<br>S63101A,S63102A,S63103A,S63104A,S63105A,S63106A,S63111A<br>S63112A,S63113A,S63114A,S63115A,S63116A,S63121A,S63122A<br>S63123A,S63124A,S63125A,S63126A,S63131A,S63132A,S63133A<br>S63134A,S63135A,S63136A,S63141A,S63142A,S63143A,S63144A<br>S63145A,S63146A,S63200A,S63201A,S63202A,S63203A,S63204A<br>S63205A,S63206A,S63207A,S63208A,S63209A,S63210A,S63211A<br>S63212A,S63213A,S63214A,S63215A,S63216A,S63217A,S63218A<br>S63219A,S63220A,S63221A,S63222A,S63223A,S63224A,S63225A<br>S63226A,S63227A,S63228A,S63229A,S63230A,S63231A,S63232A<br>S63233A,S63234A,S63235A,S63236A,S63237A,S63238A,S63239A<br>S63240A,S63241A,S63242A,S63243A,S63244A,S63245A,S63246A<br>S63247A,S63248A,S63249A,S63250A,S63251A,S63252A,S63253A<br>S63254A,S63255A,S63256A,S63257A,S63258A,S63259A,S63260A<br>S63261A,S63262A,S63263A,S63264A,S63265A,S63266A,S63267A<br>S63268A,S63269A,S63270A,S63271A,S63272A,S63273A,S63274A<br>S63275A,S63276A,S63277A,S63278A,S63279A,S63280A,S63281A<br>S63282A,S63283A,S63284A,S63285A,S63286A,S63287A,S63288A<br>S63289A,S63290A,S63291A,S63292A,S63293A,S63294A,S63295A<br>S63296A,S63297A,S63298A,S63299A,S73001A,S73002A,S73003A<br>S73004A,S73005A,S73006A,S73011A,S73012A,S73013A,S73014A<br>S73015A,S73016A,S73021A,S73022A,S73023A,S73024A,S73025A<br>S73026A,S73031A,S73032A,S73033A,S73034A,S73035A,S73036A<br>S73041A,S73042A,S73043A,S73044A,S73045A,S73046A,S83001A<br>S83002A,S83003A,S83004A,S83005A,S83006A,S83011A,S83012A<br>S83013A,S83014A,S83015A,S83016A,S83091A,S83092A,S83093A<br>S83094A,S83095A,S83096A,S83101A,S83102A,S83103A,S83104A<br>S83105A,S83106A,S83111A,S83112A,S83113A,S83114A,S83115A<br>S83116A,S83121A,S83122A,S83123A,S83124A,S83125A,S83126A<br>S83131A,S83132A,S83133A,S83134A,S83135A,S83136A,S83141A<br>S83142A,S83143A,S83144A,S83145A,S83146A,S83191A,S83192A<br>S83193A,S83194A,S83195A,S83196A,S9301XA,S9302XA,S9303XA<br>S9304XA,S9305XA,S9306XA,S93101A,S93102A,S93103A,S93104A<br>S93105A,S93106A,S93111A,S93112A,S93113A,S93114A,S93115A<br>S93116A,S93119A,S93121A,S93122A,S93123A,S93124A,S93125A<br>S93126A,S93129A,S93131A,S93132A,S93133A,S93134A,S93135A<br>S93136A,S93139A,S93141A,S93142A,S93143A,S93144A,S93145A<br>S93146A,S93149A,S93301A,S93302A,S93303A,S93304A,S93305A<br>S93306A,S93311A,S93312A,S93313A,S93314A,S93315A,S93316A<br>S93321A,S93322A,S93323A,S93324A,S93325A,S93326A,S93331A<br>S93332A,S93333A,S93334A,S93335A,S93336A | |
| Dislocations,<br>subsequent encounter | S0300XD,S0301XD,S0302XD,S0303XD,S030XXD,S031XXD,S032XXD<br>S130XXD,S13100D,S13101D,S13110D,S13111D,S13120D,S13121D<br>S13130D,S13131D,S13140D,S13141D,S13150D,S13151D,S13160D<br>S13161D,S13170D,S13171D,S13180D,S13181D,S1320XD,S1329XD<br>S230XXD,S23100D,S23101D,S23110D,S23111D,S23120D,S23121D<br>S23122D,S23123D,S23130D,S23131D,S23132D,S23133D,S23140D<br>S23141D,S23142D,S23143D,S23150D,S23151D,S23152D,S23153D<br>S23160D,S23161D,S23162D,S23163D,S23170D,S23171D,S2320XD<br>S2329XD,S330XXD,S33100D,S33101D,S33110D,S33111D,S33120D<br>S33121D,S33130D,S33131D,S33140D,S33141D,S332XXD,S3330XD<br>S3339XD,S334XXD,S43001D,S43002D,S43003D,S43004D,S43005D<br>S43006D,S43011D,S43012D,S43013D,S43014D,S43015D,S43016D<br>S43021D,S43022D,S43023D,S43024D,S43025D,S43026D,S43031D<br>S43032D,S43033D,S43034D,S43035D,S43036D,S43081D,S43082D<br>S43083D,S43084D,S43085D,S43086D,S43101D,S43102D,S43109D<br>S43111D,S43112D,S43119D,S43121D,S43122D,S43129D,S43131D<br>S43132D,S43139D,S43141D,S43142D,S43149D,S43151D,S43152D<br>S43159D,S43201D,S43202D,S43203D,S43204D,S43205D,S43206D<br>S43211D,S43212D,S43213D,S43214D,S43215D,S43216D,S43221D<br>S43222D,S43223D,S43224D,S43225D,S43226D,S43301D,S43302D<br>S43303D,S43304D,S43305D,S43306D,S43311D,S43312D,S43313D<br>S43314D,S43315D,S43316D,S43391D,S43392D,S43393D,S43394D<br>S43395D,S43396D,S53001D,S53002D,S53003D,S53004D,S53005D<br>S53006D,S53011D,S53012D,S53013D,S53014D,S53015D,S53016D<br>S53021D,S53022D,S53023D,S53024D,S53025D,S53026D,S53031D<br>S53032D,S53033D,S53091D,S53092D,S53093D,S53094D,S53095D<br>S53096D,S53101D,S53102D,S53103D,S53104D,S53105D,S53106D<br>S53111D,S53112D,S53113D,S53114D,S53115D,S53116D,S53121D<br>S53122D,S53123D,S53124D,S53125D,S53126D,S53131D,S53132D<br>S53133D,S53134D,S53135D,S53136D,S53141D,S53142D,S53143D<br>S53144D,S53145D,S53146D,S53191D,S53192D,S53193D,S53194D<br>S53195D,S53196D,S63001D,S63002D,S63003D,S63004D,S63005D<br>S63006D,S63011D,S63012D,S63013D,S63014D,S63015D,S63016D<br>S63021D,S63022D,S63023D,S63024D,S63025D,S63026D,S63031D<br>S63032D,S63033D,S63034D,S63035D,S63036D,S63041D,S63042D<br>S63043D,S63044D,S63045D,S63046D,S63051D,S63052D,S63053D<br>S63054D,S63055D,S63056D,S63061D,S63062D,S63063D,S63064D<br>S63065D,S63066D,S63071D,S63072D,S63073D,S63074D,S63075D<br>S63076D,S63091D,S63092D,S63093D,S63094D,S63095D,S63096D<br>S63101D,S63102D,S63103D,S63104D,S63105D,S63106D,S63111D<br>S63112D,S63113D,S63114D,S63115D,S63116D,S63121D,S63122D<br>S63123D,S63124D,S63125D,S63126D,S63131D,S63132D,S63133D | V5889 |

Continued on next page

Table S9 (continued).

| Diagnosis | ICD-10 | ICD-9 |
|---|---|---|
| | S63134D,S63135D,S63136D,S63141D,S63142D,S63143D,S63144D | |
| | S63145D,S63146D,S63200D,S63201D,S63202D,S63203D,S63204D | |
| | S63205D,S63206D,S63207D,S63208D,S63209D,S63210D,S63211D | |
| | S63212D,S63213D,S63214D,S63215D,S63216D,S63217D,S63218D | |
| | S63219D,S63220D,S63221D,S63222D,S63223D,S63224D,S63225D | |
| | S63226D,S63227D,S63228D,S63229D,S63230D,S63231D,S63232D | |
| | S63233D,S63234D,S63235D,S63236D,S63237D,S63238D,S63239D | |
| | S63240D,S63241D,S63242D,S63243D,S63244D,S63245D,S63246D | |
| | S63247D,S63248D,S63249D,S63250D,S63251D,S63252D,S63253D | |
| | S63254D,S63255D,S63256D,S63257D,S63258D,S63259D,S63260D | |
| | S63261D,S63262D,S63263D,S63264D,S63265D,S63266D,S63267D | |
| | S63268D,S63269D,S63270D,S63271D,S63272D,S63273D,S63274D | |
| | S63275D,S63276D,S63277D,S63278D,S63279D,S63280D,S63281D | |
| | S63282D,S63283D,S63284D,S63285D,S63286D,S63287D,S63288D | |
| | S63289D,S63290D,S63291D,S63292D,S63293D,S63294D,S63295D | |
| | S63296D,S63297D,S63298D,S63299D,S73001D,S73002D,S73003D | |
| | S73004D,S73005D,S73006D,S73011D,S73012D,S73013D,S73014D | |
| | S73015D,S73016D,S73021D,S73022D,S73023D,S73024D,S73025D | |
| | S73026D,S73031D,S73032D,S73033D,S73034D,S73035D,S73036D | |
| | S73041D,S73042D,S73043D,S73044D,S73045D,S73046D,S83001D | |
| | S83002D,S83003D,S83004D,S83005D,S83006D,S83011D,S83012D | |
| | S83013D,S83014D,S83015D,S83016D,S83091D,S83092D,S83093D | |
| | S83094D,S83095D,S83096D,S83101D,S83102D,S83103D,S83104D | |
| | S83105D,S83106D,S83111D,S83112D,S83113D,S83114D,S83115D | |
| | S83116D,S83121D,S83122D,S83123D,S83124D,S83125D,S83126D | |
| | S83131D,S83132D,S83133D,S83134D,S83135D,S83136D,S83141D | |
| | S83142D,S83143D,S83144D,S83145D,S83146D,S83191D,S83192D | |
| | S83193D,S83194D,S83195D,S83196D,S9301XD,S9302XD,S9303XD | |
| | S9304XD,S9305XD,S9306XD,S93101D,S93102D,S93103D,S93104D | |
| | S93105D,S93106D,S93111D,S93112D,S93113D,S93114D,S93115D | |
| | S93116D,S93119D,S93121D,S93122D,S93123D,S93124D,S93125D | |
| | S93126D,S93129D,S93131D,S93132D,S93133D,S93134D,S93135D | |
| | S93136D,S93139D,S93141D,S93142D,S93143D,S93144D,S93145D | |
| | S93146D,S93149D,S93301D,S93302D,S93303D,S93304D,S93305D | |
| | S93306D,S93311D,S93312D,S93313D,S93314D,S93315D,S93316D | |
| | S93321D,S93322D,S93323D,S93324D,S93325D,S93326D,S93331D | |
| | S93332D,S93333D,S93334D,S93335D,S93336D | |
| Disorders of jaw | K0820,K0821,K0822,K0823,K0824,K0825,K0826,M2600,M2601,M2602 | 52400,52401,52402,52403,52404,52405, 52406,52407,52409,52410 |
| | M2603,M2604,M2605,M2606,M2607,M2609,M2610,M2611,M2612,M2619 | 52411,52412,52419,52420,52421,52422, 52423,52424,52425,52426 |
| | M2620,M26211,M26212,M26213,M26219,M26220,M26221,M2623,M2624 | 52427,52428,52429,52430,52431,52432, 52433,52434,52435,52436 |
| | M2625,M2629,M2630,M2631,M2632,M2633,M2634,M2635,M2636,M2637 | 52437,52439,5244,52450,52451,52452, 52453,52454,52455,52456 |
| | M2639,M264,M2650,M2651,M2652,M2653,M2654,M2655,M2656,M2657 | 52457,52459,52460,52461,52462,52463, 52469,52470,52471,52472 |
| | M2659,M2660,M26601,M26602,M26603,M26609,M2661,M26611,M26612 | 52473,52474,52479,52481,52482,52489, 5249,52520,52521,52522 |
| | M26613,M26619,M2662,M26621,M26622,M26623,M26629,M2663,M26631 | 52523,52524,52525,52526,52571,52572, 52573,52579,5258,5262 |
| | M26632,M26633,M26639,M26641,M26642,M26643,M26649,M26651 | 5263,5264,5265,52661,52662,52663,52669, 52681,52689,5269 |
| | M26652,M26653,M26659,M2669,M2670,M2671,M2672,M2673,M2674 | |
| | M2679,M2681,M2682,M2689,M269,M270,M271,M272,M273,M2740,M2749 | |
| | M2751,M2752,M2753,M2759,M2761,M2762,M2763,M2769,M278,M279 | |
| Disorders of lipid metabolism | E780,E7800,E7801,E781,E782,E783,E784,E7841,E7849,E785 | 2720,2721,2722,2723,2724 |
| Disorders of teeth | K000,K001,K002,K003,K004,K005,K006,K007,K008,K009,K010,K011 | 5200,5201,5202,5203,5204,5205,5206, 5207,5208,5209,52100,52101 |
| and gingiva | K023,K0251,K0252,K0253,K0261,K0262,K0263,K027,K029,K030,K031 | 52102,52103,52104,52105,52106,52107, 52108,52110,52120,52130 |
| | K032,K033,K034,K035,K036,K037,K0381,K0389,K039,K040,K0401 | 52140,52141,52142,5215,5216,5217,52181, 52189,5219,5220,5221 |
| | K0402,K041,K042,K043,K044,K045,K046,K047,K048,K0490,K0499 | 5222,5223,5224,5225,5226,5227,5228, 5229,52300,52301,52310 |
| | K0500,K0501,K0510,K0511,K0520,K0521,K05211,K05212,K05213 | 52311,52320,52330,52331,52332,52333, 52340,52341,52342,5235 |
| | K05219,K0522,K05221,K05222,K05223,K05229,K0530,K0531,K05311 | 5236,5238,5239,52400,52401,52402,52403, 52404,52405,52406 |
| | K05312,K05313,K05319,K0532,K05321,K05322,K05323,K05329,K054 | 52407,52409,52410,52411,52412,52419, 52420,52421,52422,52423 |
| | K055,K056,K060,K06010,K06011,K06012,K06013,K06020,K06021 | 52424,52425,52426,52427,52428,52429, 52430,52431,52432,52433 |
| | K06022,K06023,K061,K062,K063,K068,K069,K080,K08101,K08102 | 52434,52435,52436,52437,52439,5244, 52450,52451,52452,52453 |
| | K08103,K08104,K08109,K08111,K08112,K08113,K08114,K08119 | 52454,52455,52456,52457,52459,52470, 52471,52472,52473,52474 |
| | K08121,K08122,K08123,K08124,K08129,K08131,K08132,K08133 | 52479,52481,52482,52489,5249,5250, 52510,52511,52512,52513 |
| | K08134,K08139,K08191,K08192,K08193,K08194,K08199,K0820,K0821 | 52519,52520,52521,52522,52523,52524, 52525,52526,5253,52540 |
| | K0822,K0823,K0824,K0825,K0826,K083,K08401,K08402,K08403 | 52541,52542,52543,52544,52550,52551, 52552,52553,52554,52560 |

Continued on next page

**Table S9 (continued).**

| Diagnosis | ICD-10 | ICD-9 |
|---|---|---|
| | K08404,K08409,K08411,K08412,K08413,K08414,K08419,K08421<br>K08422,K08423,K08424,K08429,K08431,K08432,K08433,K08434<br>K08439,K08491,K08492,K08493,K08494,K08499,K0850,K0851,K0852<br>K08530,K08531,K08539,K0854,K0855,K0856,K0859,K088,K0881,K0882<br>K0889,K089,K090,K091,M2600,M2601,M2602,M2603,M2604,M2605<br>M2606,M2607,M2609,M2610,M2611,M2612,M2619,M2620,M26211,M26212<br>M26213,M26219,M26220,M26221,M2623,M2624,M2625,M2629,M2630<br>M2631,M2632,M2633,M2634,M2635,M2636,M2637,M2639,M264,M2650<br>M2651,M2652,M2653,M2654,M2655,M2656,M2657,M2659,M2670,M2671<br>M2672,M2673,M2674,M2679,M2681,M2682,M2689,M269,M273,M2751<br>M2752,M2753,M2759,M2761,M2762,M2763,M2769,M350C,S032XXA<br>S032XXD,S032XXS | 52561,52562,52563,52564,52565,52566,<br>52567,52569,52571,52572<br>52573,52579,5258,5259,5260,5261,5265,<br>52661,52662,52663,52669<br>87363,9060,V5889 |
| Disruptive, impulse-control and conduct disorders | F602,F631,F632,F6381,F6389,F639,F910,F911,F912,F913,F918,F919 | 3017,31200,31210,31220,31230,31232,<br>31233,31234,31235,31239<br>31281,31282,31289,3129,31381 |
| Diverticulosis and diverticulitis | K5700,K5701,K5710,K5711,K5712,K5713,K5720,K5721,K5730,K5731<br>K5732,K5733,K5740,K5741,K5750,K5751,K5752,K5753,K5780,K5781<br>K5790,K5791,K5792,K5793 | 56200,56201,56202,56203,56210,56211,<br>56212,56213,5695 |
| Drug induced or toxic related condition | D521,D590,D592,D611,D612,D61811,D642,D702,E032,E064,E160,E231<br>E242,E273,E661,G210,G2111,G2119,G2401,G2402,G2409,G251,G254<br>G2561,G2570,G2571,G2579,G4440,G4441,G620,G622,G701,G7114,G720<br>G722,G92,G9200,G9201,G9202,G9203,G9204,G9205,G928,G929,H10211<br>H10212,H10213,H10219,H2630,H2631,H2632,H2633,H35381,H35382<br>H35383,H35389,H4060X0,H4060X1,H4060X2,H4060X3,H4060X4,H4061X0<br>H4061X1,H4061X2,H4061X3,H4061X4,H4062X0,H4062X1,H4062X2<br>H4062X3,H4062X4,H4063X0,H4063X1,H4063X2,H4063X3,H4063X4,H463<br>H9101,H9102,H9103,H9109,I427,I952,J702,J703,J704,K1232,K521<br>K5903,K5931,K710,K7110,K7111,K712,K713,K714,K7150,K7151,K716<br>K717,K718,K719,K853,K8530,K8531,K8532,L105,L244,L251,L432<br>L530,L640,M1020,M10211,M10212,M10219,M10221,M10222,M10229<br>M10231,M10232,M10239,M10241,M10242,M10249,M10251,M10252<br>M10259,M10261,M10262,M10269,M10271,M10272,M10279,M1028,M1029<br>M1A20X0,M1A20X1,M1A2110,M1A2111,M1A2120,M1A2121,M1A2190<br>M1A2191,M1A2210,M1A2211,M1A2220,M1A2221,M1A2290,M1A2291<br>M1A2310,M1A2311,M1A2320,M1A2321,M1A2390,M1A2391,M1A2410<br>M1A2411,M1A2420,M1A2421,M1A2490,M1A2491,M1A2510,M1A2511<br>M1A2520,M1A2521,M1A2590,M1A2591,M1A2610,M1A2611,M1A2620<br>M1A2621,M1A2690,M1A2691,M1A2710,M1A2711,M1A2720,M1A2721<br>M1A2790,M1A2791,M1A28X0,M1A28X1,M1A29X0,M1A29X1,M320,M342<br>M835,M8710,M87111,M87112,M87119,M87121,M87122,M87129,M87131<br>M87132,M87133,M87134,M87135,M87136,M87137,M87138,M87139<br>M87141,M87142,M87143,M87144,M87145,M87146,M87150,M87151<br>M87152,M87159,M87161,M87162,M87163,M87164,M87165,M87166<br>M87171,M87172,M87173,M87174,M87175,M87176,M87177,M87178<br>M87179,M87180,M87188,M8719,N140,N141,N1411,N1419,N142,N143<br>N144,N46021,N46121,N4833,N522,O771,P5841,P5842,P938,R330,R502 | 2442,2443,2454,2511,2537,2550,25541,<br>2682,27400,27401,27402<br>27403,27800,2812,2830,28319,28412,<br>28489,2850,28803,32371<br>32372,3321,3331,3333,3335,33372,33379,<br>33385,33392,33399,3393<br>34982,3576,3577,3582,35924,3594,36255,<br>36531,36532,36645,37206<br>37734,3898,4259,45829,5088,52802,5582,<br>5722,5733,5770,58389<br>6060,6061,6073,60784,65681,6923,6944,<br>6950,6970,70409,7100<br>7101,73340,73341,73342,73343,73344,<br>73345,73349,7741,7794<br>78060,78829,E9479 |
| Dysphagia | D501,R1310,R1311,R1312,R1313,R1314,R1319 | 2808,78720,78721,78722,78723,78724,<br>78729 |
| Early or threatened labor | O26872,O26873,O26879,O3430,O3431,O3432,O3433,O4200,O42011<br>O42012,O42013,O42019,O4210,O42111,O42112,O42113,O42119,O4290<br>O42911,O42912,O42913,O42919,O4700,O4702,O4703,O471,O479,O6000<br>O6002,O6003,O6010X0,O6010X1,O6010X2,O6010X3,O6010X4,O6010X5<br>O6010X9,O6012X0,O6012X1,O6012X2,O6012X3,O6012X4,O6012X5<br>O6012X9,O6013X0,O6013X1,O6013X2,O6013X3,O6013X4,O6013X5<br>O6013X9,O6014X0,O6014X1,O6014X2,O6014X3,O6014X4,O6014X5<br>O6014X9,O6020X0,O6020X1,O6020X2,O6020X3,O6020X4,O6020X5<br>O6020X9,O6022X0,O6022X1,O6022X2,O6022X3,O6022X4,O6022X5<br>O6022X9,O6023X0,O6023X1,O6023X2,O6023X3,O6023X4,O6023X5<br>O6023X9 | 64400,64403,64410,64413,64420,64421,<br>64970,64971,64973,65450<br>65451,65452,65453,65454,65810,65811,<br>65813,65820,65821,65823 |
| Early, first or unspecified trimester hemorrhage | O200,O208,O209,O26851,O26859,O4410,O4411,O4430,O4431,O4450<br>O4451,O45001,O45009,O45011,O45019,O45021,O45029,O45091,O45099<br>O458X1,O458X9,O4590,O4591,O46001,O46009,O46011,O46019,O46021<br>O46029,O46091,O46099,O468X1,O468X9,O4690,O4691 | 64003,64083,64093,64110,64111,64113,<br>64120,64121,64123,64130<br>64131,64133,64180,64181,64183,64190,<br>64191,64193,64950,64951<br>64953 |
| Ectopic pregnancy and | O000,O0000,O0001,O001,O0010,O00101,O00102,O00109,O0011,O00111 | 63300,63310,63320,63380,63390,65680,<br>65681,65683 |

Continued on next page

Table S9 (continued).

| Diagnosis | ICD-10 | ICD-9 |
|---|---|---|
| complications of ectopic pregnancy | O00112,O00119,O002,O0020,O00201,O00202,O00209,O0021,O00211 O00212,O00219,O008,O0080,O0081,O009,O0090,O0091,O3670X0 O3670X1,O3670X2,O3670X3,O3670X4,O3670X5,O3670X9,O3671X0 O3671X1,O3671X2,O3671X3,O3671X4,O3671X5,O3671X9,O3672X0 O3672X1,O3672X2,O3672X3,O3672X4,O3672X5,O3672X9,O3673X0 O3673X1,O3673X2,O3673X3,O3673X4,O3673X5,O3673X9 | |
| Effect of foreign body entering opening, initial encounter | T1500XA,T1501XA,T1502XA,T1510XA,T1511XA,T1512XA,T1580XA T1581XA,T1582XA,T1590XA,T1591XA,T1592XA,T161XXA,T162XXA T169XXA,T170XXA,T171XXA,T17200A,T17208A,T17210A,T17218A T17220A,T17228A,T17290A,T17298A,T17300A,T17308A,T17310A T17318A,T17320A,T17328A,T17390A,T17398A,T17400A,T17408A T17410A,T17418A,T17420A,T17428A,T17490A,T17498A,T17500A T17508A,T17510A,T17518A,T17520A,T17528A,T17590A,T17598A T17800A,T17808A,T17810A,T17818A,T17820A,T17828A,T17890A T17898A,T17900A,T17908A,T17910A,T17918A,T17920A,T17928A T17990A,T17998A,T180XXA,T18100A,T18108A,T18110A,T18118A T18120A,T18128A,T18190A,T18198A,T182XXA,T183XXA,T184XXA T185XXA,T188XXA,T189XXA,T190XXA,T191XXA,T192XXA,T193XXA T194XXA,T198XXA,T199XXA | 9300,9301,9302,9308,9309,931,932,9330, 9331,9340,9341,9348 9349,9350,9351,9352,936,937,938,9390, 9391,9392,9393,9399,E911 E912,E914,E915 |
| Effect of foreign body entering opening, subsequent encounter | T1500XD,T1501XD,T1502XD,T1510XD,T1511XD,T1512XD,T1580XD T1581XD,T1582XD,T1590XD,T1591XD,T1592XD,T161XXD,T162XXD T169XXD,T170XXD,T171XXD,T17200D,T17208D,T17210D,T17218D T17220D,T17228D,T17290D,T17298D,T17300D,T17308D,T17310D T17318D,T17320D,T17328D,T17390D,T17398D,T17400D,T17408D T17410D,T17418D,T17420D,T17428D,T17490D,T17498D,T17500D T17508D,T17510D,T17518D,T17520D,T17528D,T17590D,T17598D T17800D,T17808D,T17810D,T17818D,T17820D,T17828D,T17890D T17898D,T17900D,T17908D,T17910D,T17918D,T17920D,T17928D T17990D,T17998D,T180XXD,T18100D,T18108D,T18110D,T18118D T18120D,T18128D,T18190D,T18198D,T182XXD,T183XXD,T184XXD T185XXD,T188XXD,T189XXD,T190XXD,T191XXD,T192XXD,T193XXD T194XXD,T198XXD,T199XXD | V5889 |
| Effect of other external causes, initial encounter | T33011A,T33012A,T33019A,T3302XA,T3309XA,T331XXA,T332XXA T333XXA,T3340XA,T3341XA,T3342XA,T33511A,T33512A,T33519A T33521A,T33522A,T33529A,T33531A,T33532A,T33539A,T3360XA T3361XA,T3362XA,T3370XA,T3371XA,T3372XA,T33811A,T33812A T33819A,T33821A,T33822A,T33829A,T33831A,T33832A,T33839A T3390XA,T3399XA,T34011A,T34012A,T34019A,T3402XA,T3409XA T341XXA,T342XXA,T343XXA,T3440XA,T3441XA,T3442XA,T34511A T34512A,T34519A,T34521A,T34522A,T34529A,T34531A,T34532A T34539A,T3460XA,T3461XA,T3462XA,T3470XA,T3471XA,T3472XA T34811A,T34812A,T34819A,T34821A,T34822A,T34829A,T34831A T34832A,T34839A,T3490XA,T3499XA,T66XXXA,T6701XA,T6702XA T6709XA,T670XXA,T671XXA,T672XXA,T673XXA,T674XXA,T675XXA T676XXA,T677XXA,T678XXA,T679XXA,T68XXXA,T69011A,T69012A T69019A,T69021A,T69022A,T69029A,T691XXA,T698XXA,T699XXA T700XXA,T701XXA,T7020XA,T7029XA,T703XXA,T704XXA,T708XXA T709XXA,T71111A,T71112A,T71113A,T71114A,T71121A,T71122A T71123A,T71124A,T71131A,T71132A,T71133A,T71134A,T71141A T71143A,T71144A,T71151A,T71152A,T71153A,T71154A,T71161A T71162A,T71163A,T71164A,T71191A,T71192A,T71193A,T71194A T7120XA,T7121XA,T71221A,T71222A,T71223A,T71224A,T71231A T71232A,T71233A,T71234A,T7129XA,T719XXA,T730XXA,T731XXA T732XXA,T733XXA,T738XXA,T739XXA,T7500XA,T7501XA,T7509XA T751XXA,T7520XA,T7521XA,T7522XA,T7523XA,T7529XA,T753XXA T754XXA,T7581XA,T7582XA,T7589XA | 990,9910,9911,9912,9913,9914,9915,9916, 9918,9919,9920,9921 9922,9923,9924,9925,9926,9927,9928, 9929,9930,9931,9932,9933 9934,9938,9939,9940,9941,9942,9943, 9944,9945,9946,9947,9948 9949,E903,E9041,E9042,E9043,E9049, E9130,E9131,E9132,E9133 E9138,E9139,E9272,E9530,E9531,E9538, E963,E9830,E9831,E9838 |
| Effect of other external causes, sequela | T33011S,T33012S,T33019S,T3302XS,T3309XS,T331XXS,T332XXS T333XXS,T3340XS,T3341XS,T3342XS,T33511S,T33512S,T33519S T33521S,T33522S,T33529S,T33531S,T33532S,T33539S,T3360XS T3361XS,T3362XS,T3370XS,T3371XS,T3372XS,T33811S,T33812S T33819S,T33821S,T33822S,T33829S,T33831S,T33832S,T33839S T3390XS,T3399XS,T34011S,T34012S,T34019S,T3402XS,T3409XS T341XXS,T342XXS,T343XXS,T3440XS,T3441XS,T3442XS,T34511S T34512S,T34519S,T34521S,T34522S,T34529S,T34531S,T34532S T34539S,T3460XS,T3461XS,T3462XS,T3470XS,T3471XS,T3472XS T34811S,T34812S,T34819S,T34821S,T34822S,T34829S,T34831S T34832S,T34839S,T3490XS,T3499XS,T66XXXS,T6701XS,T6702XS T6709XS,T670XXS,T671XXS,T672XXS,T673XXS,T674XXS,T675XXS T676XXS,T677XXS,T678XXS,T679XXS,T68XXXS,T69011S,T69012S T69019S,T69021S,T69022S,T69029S,T691XXS,T698XXS,T699XXS T700XXS,T701XXS,T7020XS,T7029XS,T703XXS,T704XXS,T708XXS T709XXS,T71111S,T71112S,T71113S,T71114S,T71121S,T71122S T71123S,T71124S,T71131S,T71132S,T71133S,T71134S,T71141S T71143S,T71144S,T71151S,T71152S,T71153S,T71154S,T71161S | 9094,9099,E9298,E959,E969,E989 |

Continued on next page

Table S9 (continued).

| Diagnosis | ICD-10 | ICD-9 |
| --- | --- | --- |
| | T71162S,T71163S,T71164S,T71191S,T71192S,T71193S,T71194S T7120XS,T7121XS,T71221S,T71222S,T71223S,T71224S,T71231S T71232S,T71233S,T71234S,T7129XS,T719XXS,T730XXS,T731XXS T732XXS,T733XXS,T738XXS,T739XXS,T7401XS,T7402XS,T7411XS T7412XS,T7421XS,T7422XS,T7431XS,T7432XS,T744XXS,T7451XS T7452XS,T7461XS,T7462XS,T7491XS,T7492XS,T7500XS,T7501XS T7509XS,T751XXS,T7520XS,T7521XS,T7522XS,T7523XS,T7529XS T753XXS,T754XXS,T7581XS,T7582XS,T7589XS,T7601XS,T7602XS T7611XS,T7612XS,T7621XS,T7622XS,T7631XS,T7632XS,T7651XS T7652XS,T7661XS,T7662XS,T7691XS,T7692XS | |
| Effect of other external causes, subsequent encounter | T33011D,T33012D,T33019D,T3302XD,T3309XD,T331XXD,T332XXD T333XXD,T3340XD,T3341XD,T3342XD,T33511D,T33512D,T33519D T33521D,T33522D,T33529D,T33531D,T33532D,T33539D,T3360XD T3361XD,T3362XD,T3370XD,T3371XD,T3372XD,T33811D,T33812D T33819D,T33821D,T33822D,T33829D,T33831D,T33832D,T33839D T3390XD,T3399XD,T34011D,T34012D,T34019D,T3402XD,T3409XD T341XXD,T342XXD,T343XXD,T3440XD,T3441XD,T3442XD,T34511D T34512D,T34519D,T34521D,T34522D,T34529D,T34531D,T34532D T34539D,T3460XD,T3461XD,T3462XD,T3470XD,T3471XD,T3472XD T34811D,T34812D,T34819D,T34821D,T34822D,T34829D,T34831D T34832D,T34839D,T3490XD,T3499XD,T66XXXD,T6701XD,T6702XD T6709XD,T670XXD,T671XXD,T672XXD,T673XXD,T674XXD,T675XXD T676XXD,T677XXD,T678XXD,T679XXD,T68XXXD,T69011D,T69012D T69019D,T69021D,T69022D,T69029D,T691XXD,T698XXD,T699XXD T700XXD,T701XXD,T7020XD,T7029XD,T703XXD,T704XXD,T708XXD T709XXD,T71111D,T71112D,T71113D,T71114D,T71121D,T71122D T71123D,T71124D,T71131D,T71132D,T71133D,T71134D,T71141D T71143D,T71144D,T71151D,T71152D,T71153D,T71154D,T71161D T71162D,T71163D,T71164D,T71191D,T71192D,T71193D,T71194D T7120XD,T7121XD,T71221D,T71222D,T71223D,T71224D,T71231D T71232D,T71233D,T71234D,T7129XD,T719XXD,T730XXD,T731XXD T732XXD,T733XXD,T738XXD,T739XXD,T7500XD,T7501XD,T7509XD T751XXD,T7520XD,T7521XD,T7522XD,T7523XD,T7529XD,T753XXD T754XXD,T7581XD,T7582XD,T7589XD | V5889 |
| Encephalitis | A1782,A3212,A3981,A4282,A5042,A5214,A811,A830,A831,A832,A833 A834,A835,A836,A838,A839,A840,A841,A848,A8481,A8489,A849,A850 A851,A852,A858,A86,A922,A9231,B004,B0111,B020,B050,B0601 B1001,B1009,B262,B4081,B5742,B582,B6011,G0400,G0401,G0402 G042,G0430,G0431,G0432,G0439,G0481,G0482,G0489,G0490,G0491 G053,G054 | 01360,0270,0361,0398,0462,0498,0499, 0520,05319,0543,0550 05601,05821,05829,0620,0621,0622,0623, 0624,0625,0628,0629 0630,0631,0632,0638,0639,064,0662, 06641,0722,0861,09041,09481 1160,1300,13621,3209,32341,32342,32351, 32352,32361,32362 32381,32382,3239 |
| Encounter for administrative purposes | Z020,Z021,Z022,Z023,Z024,Z025,Z026,Z0271,Z0279,Z0281,Z0282 Z0283,Z0289,Z029 | V6801,V6809,V6889,V689,V703,V704, V705 |
| Encounter for antineoplastic therapies | Z510,Z5111,Z5112 | V580,V5811,V5812 |
| Encounter for mental health conditions | Z046,Z1330,Z1331,Z1332,Z1339 | V701 |
| Encounter for mental health services related to abuse | Z69010,Z69011,Z69020,Z69021,Z6911,Z6912,Z6981,Z6982 | V6111,V6112,V6121,V6122,V6283,V6549 |
| Encounter for observation and examination for conditions ruled out (excludes infectious disease, neoplasm, mental disorders) | Z036,Z0371,Z0372,Z0373,Z0374,Z0375,Z0379,Z03821,Z03822,Z03823 Z0383,Z0389,Z041,Z042,Z043,Z0441,Z0442,Z0471,Z0472,Z048,Z0481 Z0482,Z0489,Z049,Z050,Z052,Z053,Z0541,Z0542,Z0543,Z055,Z056 Z0571,Z0572,Z0573,Z058,Z059,Z130,Z131,Z1321,Z13220,Z13228 Z1329,Z135,Z136,Z1371,Z1379,Z13810,Z13811,Z13818,Z13820 Z13828,Z1383,Z1384,Z13850,Z13858,Z1388,Z1389,Z139,Z3200,Z3202 | V682,V713,V714,V715,V716,V7181, V7189,V719,V7240,V7241,V770 V771,V773,V774,V776,V777,V7791, V7799,V782,V783,V789,V790,V791 V799,V8001,V8009,V801,V802,V803, V811,V812,V813,V814,V815,V816 V820,V821,V824,V825,V826,V8271, V8279,V8281,V8289,V829,V8901 V8902,V8903,V8904,V8905,V8909 |
| Encounter for prophylactic or other procedures | Z4000,Z4001,Z4002,Z4003,Z4009,Z408,Z409,Z411,Z412,Z413,Z418 Z419,Z421,Z428 | V501,V502,V503,V5041,V5042,V5049, V508,V509,V510,V518 |
| Endocarditis and endocardial disease | A1884,A3282,A3951,A5203,A5206,A5483,B3321,B376,I011,I018,I019 I020,I099,I330,I339,I38,I39,M3211 | 01790,0270,03642,07422,09320,09321, 09322,09323,09324,09381 09382,09883,09884,09885,11281,3911, 3918,3919,3920,39890,4200 4210,4211,4219,4220,42490,42491,42499, 4258,7100 |

Continued on next page

Table S9 (continued).

| Diagnosis | ICD-10 | ICD-9 |
|---|---|---|
| Endocrine system cancers - adrenocortical | C7400,C7401,C7402 | 1940 |
| Endocrine system cancers - all other types | C7410,C7411,C7412,C7490,C7491,C7492,C752,C753,C754,C755,C758 C759 | 1940,1943,1944,1945,1946,1948,1949 |
| Endocrine system cancers - pancreas | C250,C251,C252,C253,C254,C257,C258,C259 | 1570,1571,1572,1573,1574,1578,1579 |
| Endocrine system cancers - parathyroid | C750 | 1941 |
| Endocrine system cancers - pituitary gland | C751 | 1943 |
| Endocrine system cancers - thymus | C37 | 1640 |
| Endocrine system cancers - thyroid | C73,D093,E3122,E3123 | 193,2348,25802,25803 |
| Endometriosis | N800,N8000,N8001,N8002,N8003,N801,N80101,N80102,N80103,N80109 N80111,N80112,N80113,N80119,N80121,N80122,N80123,N80129,N802 N80201,N80202,N80203,N80209,N80211,N80212,N80213,N80219 N80221,N80222,N80223,N80229,N803,N8030,N80311,N80312,N80319 N80321,N80322,N80329,N80331,N80332,N80333,N80339,N80341 N80342,N80343,N80349,N80351,N80352,N80353,N80359,N80361 N80362,N80363,N80369,N80371,N80372,N80373,N80379,N80381 N80382,N80383,N80389,N80391,N80392,N80399,N803A1,N803A2 N803A3,N803A9,N803B1,N803B2,N803B3,N803B9,N803C1,N803C2 N803C3,N803C9,N804,N8040,N8041,N8042,N805,N8050,N80511,N80512 N80519,N80521,N80522,N80529,N80531,N80532,N80539,N80541 N80542,N80549,N80551,N80552,N80559,N80561,N80562,N80569,N806 N808,N809,N80A0,N80A1,N80A2,N80A41,N80A42,N80A43,N80A49 N80A51,N80A52,N80A53,N80A59,N80A61,N80A62,N80A63,N80A69,N80B1 N80B2,N80B31,N80B32,N80B39,N80B4,N80B5,N80B6,N80C0,N80C10 N80C11,N80C19,N80C2,N80C3,N80C4,N80C9,N80D0,N80D1,N80D2,N80D3 N80D4,N80D5,N80D6,N80D9 | 6170,6171,6172,6173,6174,6175,6176, 6178,6179 |
| Epilepsy; convulsions | G40001,G40009,G40011,G40019,G40101,G40109,G40111,G40119 G40201,G40209,G40211,G40219,G40301,G40309,G40311,G40319 G40401,G40409,G40411,G40419,G4042,G40501,G40509,G40801,G40802 G40803,G40804,G40811,G40812,G40813,G40814,G40821,G40822 G40823,G40824,G40833,G40834,G4089,G40901,G40909,G40911,G40919 G40A01,G40A09,G40A11,G40A19,G40B01,G40B09,G40B11,G40B19,R5600 R5601,R561,R569 | 34500,34501,34510,34511,3452,3453, 34540,34541,34550,34551 34560,34561,34570,34571,34580,34581, 34590,34591,78031,78032 78033,78039 |
| Erectile dysfunction | N5201,N5202,N5203,N521,N522,N5231,N5232,N5233,N5234,N5235 N5236,N5237,N5239,N528,N529 | 60784 |
| Esophageal disorders | B3781,B5731,K200,K208,K2080,K2081,K209,K2090,K2091,K210,K2100 K2101,K219,K220,K2210,K2211,K222,K223,K224,K225,K226,K2270 K22710,K22711,K22719,K228,K2281,K2282,K2289,K229,K23 | 0861,11284,5300,53010,53011,53012, 53013,53019,53020,53021 5303,5304,5305,5306,5307,53081,53082, 53083,53085,53089,5309 |
| Essential hypertension | I10,O10011,O10012,O10013,O10019,O1002,O1003,O10911,O10912 O10913,O10919,O1092,O1093,O161,O162,O163,O164,O165,O169 | 4010,4011,4019,64200,64201,64203,64204, 64290,64291,64293 64294 |
| Exposure, encounters, screening or contact with infectious disease | G0402,G0432,Z03810,Z03818,Z051,Z110,Z111,Z112,Z113,Z114,Z1151 Z1152,Z1159,Z116,Z117,Z118,Z119,Z2001,Z2009,Z201,Z202,Z203 Z204,Z205,Z206,Z207,Z20810,Z20811,Z20818,Z20820,Z20821,Z20822 Z20828,Z2089,Z209,Z23 | 32351,32352,V010,V011,V012,V013, V014,V015,V016,V0171,V0179 V0181,V0182,V0183,V0184,V0189,V019, V059,V7182,V7183,V730,V732 V733,V734,V735,V7381,V7388,V7389, V7398,V740,V741,V742,V743 V745,V748,V750,V751,V752,V753,V754, V755,V756,V757,V758,V759 |
| Feeding and eating disorders | F5000,F5001,F5002,F502,F508,F5081,F5082,F5089,F509,F9821 F9829,F983 | 3071,30750,30751,30752,30753,30754, 30759 |
| Female infertility | E28310,E28319,E2839,N970,N971,N972,N978,N979 | 25631,25639,6280,6282,6283,6284,6288, 6289 |
| Female reproductive system cancers - all | C510,C511,C512,C518,C5710,C5711,C5712,C5720,C5721,C5722,C573 C574,C577,C578,C579,C58,D0730,D0739 | 181,1833,1834,1835,1839,1841,1842,1843, 1848,1849,23330,23339 |

Continued on next page

**Table S9 (continued).**

| Diagnosis | ICD-10 | ICD-9 |
|---|---|---|
| other types | | |
| Female reproductive system cancers - cervix | C530,C531,C538,C539,D060,D061,D067,D069 | 1800,1801,1808,1809,2331 |
| Female reproductive system cancers - endometrium | C541,D070 | 1820,2332 |
| Female reproductive system cancers - fallopian tube | C5700,C5701,C5702 | 1832 |
| Female reproductive system cancers - ovary | C561,C562,C563,C569 | 1830 |
| Female reproductive system cancers - uterus | C540,C542,C543,C548,C549,C55 | 179,1820,1821,1828 |
| Female reproductive system cancers - vagina | C52,D072 | 1840,23331 |
| Female reproductive system cancers - vulva | C519,D071 | 1844,23332 |
| Fetal alcohol syndrome | P043,Q860 | 76071 |
| Fever | R502,R5081,R5082,R5083,R5084,R509 | 78060,78061,78062,78063,78066,E9479 |
| Fluid and electrolyte disorders | E860,E861,E869,E870,E871,E872,E8720,E8721,E8722,E8729,E873 E874,E875,E876,E8770,E8771,E8779,E878 | 2760,2761,2762,2763,2764,27650,27651, 27652,27661,27669,2767 2768,2769 |
| Foodborne intoxications | A050,A051,A052,A053,A054,A055,A058,A059,A4851 | 0050,0051,0052,0054,00581,00589,0059, 04041 |
| Fracture of head and neck, initial encounter | S020XXA,S020XXB,S02101A,S02101B,S02102A,S02102B,S02109A S02109B,S0210XA,S0210XB,S02110A,S02110B,S02111A,S02111B S02112A,S02112B,S02113A,S02113B,S02118A,S02118B,S02119A S02119B,S0211AA,S0211AB,S0211BA,S0211BB,S0211CA,S0211CB S0211DA,S0211DB,S0211EA,S0211EB,S0211FA,S0211FB,S0211GA S0211GB,S0211HA,S0211HB,S02121A,S02121B,S02122A,S02122B S02129A,S02129B,S0219XA,S0219XB,S022XXA,S022XXB,S0230XA S0230XB,S0231XA,S0231XB,S0232XA,S0232XB,S023XXA,S023XXB S02400A,S02400B,S02401A,S02401B,S02402A,S02402B,S0240AA S0240AB,S0240BA,S0240BB,S0240CA,S0240CB,S0240DA,S0240DB S0240EA,S0240EB,S0240FA,S0240FB,S02411A,S02411B,S02412A S02412B,S02413A,S02413B,S0242XA,S0242XB,S025XXA,S025XXB S02600A,S02600B,S02601A,S02601B,S02602A,S02602B,S02609A S02609B,S02610A,S02610B,S02611A,S02611B,S02612A,S02612B S0261XA,S0261XB,S02620A,S02620B,S02621A,S02621B,S02622A S02622B,S0262XA,S0262XB,S02630A,S02630B,S02631A,S02631B S02632A,S02632B,S0263XA,S0263XB,S02640A,S02640B,S02641A S02641B,S02642A,S02642B,S0264XA,S0264XB,S02650A,S02650B S02651A,S02651B,S02652A,S02652B,S0265XA,S0265XB,S0266XA S0266XB,S02670A,S02670B,S02671A,S02671B,S02672A,S02672B S0267XA,S0267XB,S0269XA,S0269XB,S0280XA,S0280XB,S0281XA S0281XB,S0282XA,S0282XB,S02831A,S02831B,S02832A,S02832B S02839A,S02839B,S02841A,S02841B,S02842A,S02842B,S02849A S02849B,S0285XA,S0285XB,S028XXA,S028XXB,S0291XA,S0291XB S0292XA,S0292XB | 80000,80050,80100,80150,8020,8021, 80220,80221,80222,80223 80224,80225,80226,80227,80228,80230, 80231,80232,80233,80234 80235,80236,80237,80238,8024,8025,8026, 8027,8028,8029,80300 80350,87363 |
| Fracture of head and neck, subsequent encounter | S020XXD,S020XXG,S020XXK,S02101D,S02101G,S02101K,S02102D S02102G,S02102K,S02109D,S02109G,S02109K,S0210XD,S0210XG S0210XK,S02110D,S02110G,S02110K,S02111D,S02111G,S02111K S02112D,S02112G,S02112K,S02113D,S02113G,S02113K,S02118D S02118G,S02118K,S02119D,S02119G,S02119K,S0211AD,S0211AG S0211AK,S0211BD,S0211BG,S0211BK,S0211CD,S0211CG,S0211CK S0211DD,S0211DG,S0211DK,S0211ED,S0211EG,S0211EK,S0211FD S0211FG,S0211FK,S0211GD,S0211GG,S0211GK,S0211HD,S0211HG S0211HK,S02121D,S02121G,S02121K,S02122D,S02122G,S02122K S02129D,S02129G,S02129K,S0219XD,S0219XG,S0219XK,S022XXD S022XXG,S022XXK,S0230XD,S0230XG,S0230XK,S0231XD,S0231XG S0231XK,S0232XD,S0232XG,S0232XK,S023XXD,S023XXG,S023XXK S02400D,S02400G,S02400K,S02401D,S02401G,S02401K,S02402D | 73382,V5419 |

Continued on next page

Table S9 (continued).

| Diagnosis | ICD-10 | ICD-9 |
|---|---|---|
| | S02402G,S02402K,S0240AD,S0240AG,S0240AK,S0240BD,S0240BG | |
| | S0240BK,S0240CD,S0240CG,S0240CK,S0240DD,S0240DG,S0240DK | |
| | S0240ED,S0240EG,S0240EK,S0240FD,S0240FG,S0240FK,S02411D | |
| | S02411G,S02411K,S02412D,S02412G,S02412K,S02413D,S02413G | |
| | S02413K,S0242XD,S0242XG,S0242XK,S025XXD,S025XXG,S025XXK | |
| | S02600D,S02600G,S02600K,S02601D,S02601G,S02601K,S02602D | |
| | S02602G,S02602K,S02609D,S02609G,S02609K,S02610D,S02610G | |
| | S02610K,S02611D,S02611G,S02611K,S02612D,S02612G,S02612K | |
| | S0261XD,S0261XG,S0261XK,S02620D,S02620G,S02620K,S02621D | |
| | S02621G,S02621K,S02622D,S02622G,S02622K,S0262XD,S0262XG | |
| | S0262XK,S02630D,S02630G,S02630K,S02631D,S02631G,S02631K | |
| | S02632D,S02632G,S02632K,S0263XD,S0263XG,S0263XK,S02640D | |
| | S02640G,S02640K,S02641D,S02641G,S02641K,S02642D,S02642G | |
| | S02642K,S0264XD,S0264XG,S0264XK,S02650D,S02650G,S02650K | |
| | S02651D,S02651G,S02651K,S02652D,S02652G,S02652K,S0265XD | |
| | S0265XG,S0265XK,S0266XD,S0266XG,S0266XK,S02670D,S02670G | |
| | S02670K,S02671D,S02671G,S02671K,S02672D,S02672G,S02672K | |
| | S0267XD,S0267XG,S0267XK,S0269XD,S0269XG,S0269XK,S0280XD | |
| | S0280XG,S0280XK,S0281XD,S0281XG,S0281XK,S0282XD,S0282XG | |
| | S0282XK,S02831D,S02831G,S02831K,S02832D,S02832G,S02832K | |
| | S02839D,S02839G,S02839K,S02841D,S02841G,S02841K,S02842D | |
| | S02842G,S02842K,S02849D,S02849G,S02849K,S0285XD,S0285XG | |
| | S0285XK,S028XXD,S028XXG,S028XXK,S0291XD,S0291XG,S0291XK | |
| | S0292XD,S0292XG,S0292XK | |
| Fracture of lower limb (except hip), subsequent encounter | M9711XD,M9712XD,M9721XD,M9722XD,M978XXD,M979XXD,S72301D | 73381,73382,V5415,V5416,V5426,V5429 |
| | S72301E,S72301F,S72301G,S72301H,S72301J,S72301K,S72301M | |
| | S72301N,S72301P,S72301Q,S72301R,S72302D,S72302E,S72302F | |
| | S72302G,S72302H,S72302J,S72302K,S72302M,S72302N,S72302P | |
| | S72302Q,S72302R,S72309D,S72309E,S72309F,S72309G,S72309H | |
| | S72309J,S72309K,S72309M,S72309N,S72309P,S72309Q,S72309R | |
| | S72321D,S72321E,S72321F,S72321G,S72321H,S72321J,S72321K | |
| | S72321M,S72321N,S72321P,S72321Q,S72321R,S72322D,S72322E | |
| | S72322F,S72322G,S72322H,S72322J,S72322K,S72322M,S72322N | |
| | S72322P,S72322Q,S72322R,S72323D,S72323E,S72323F,S72323G | |
| | S72323H,S72323J,S72323K,S72323M,S72323N,S72323P,S72323Q | |
| | S72323R,S72324D,S72324E,S72324F,S72324G,S72324H,S72324J | |
| | S72324K,S72324M,S72324N,S72324P,S72324Q,S72324R,S72325D | |
| | S72325E,S72325F,S72325G,S72325H,S72325J,S72325K,S72325M | |
| | S72325N,S72325P,S72325Q,S72325R,S72326D,S72326E,S72326F | |
| | S72326G,S72326H,S72326J,S72326K,S72326M,S72326N,S72326P | |
| | S72326Q,S72326R,S72331D,S72331E,S72331F,S72331G,S72331H | |
| | S72331J,S72331K,S72331M,S72331N,S72331P,S72331Q,S72331R | |
| | S72332D,S72332E,S72332F,S72332G,S72332H,S72332J,S72332K | |
| | S72332M,S72332N,S72332P,S72332Q,S72332R,S72333D,S72333E | |
| | S72333F,S72333G,S72333H,S72333J,S72333K,S72333M,S72333N | |
| | S72333P,S72333Q,S72333R,S72334D,S72334E,S72334F,S72334G | |
| | S72334H,S72334J,S72334K,S72334M,S72334N,S72334P,S72334Q | |
| | S72334R,S72335D,S72335E,S72335F,S72335G,S72335H,S72335J | |
| | S72335K,S72335M,S72335N,S72335P,S72335Q,S72335R,S72336D | |
| | S72336E,S72336F,S72336G,S72336H,S72336J,S72336K,S72336M | |
| | S72336N,S72336P,S72336Q,S72336R,S72341D,S72341E,S72341F | |
| | S72341G,S72341H,S72341J,S72341K,S72341M,S72341N,S72341P | |
| | S72341Q,S72341R,S72342D,S72342E,S72342F,S72342G,S72342H | |
| | S72342J,S72342K,S72342M,S72342N,S72342P,S72342Q,S72342R | |
| | S72343D,S72343E,S72343F,S72343G,S72343H,S72343J,S72343K | |
| | S72343M,S72343N,S72343P,S72343Q,S72343R,S72344D,S72344E | |
| | S72344F,S72344G,S72344H,S72344J,S72344K,S72344M,S72344N | |
| | S72344P,S72344Q,S72344R,S72345D,S72345E,S72345F,S72345G | |
| | S72345H,S72345J,S72345K,S72345M,S72345N,S72345P,S72345Q | |
| | S72345R,S72346D,S72346E,S72346F,S72346G,S72346H,S72346J | |
| | S72346K,S72346M,S72346N,S72346P,S72346Q,S72346R,S72351D | |
| | S72351E,S72351F,S72351G,S72351H,S72351J,S72351K,S72351M | |
| | S72351N,S72351P,S72351Q,S72351R,S72352D,S72352E,S72352F | |
| | S72352G,S72352H,S72352J,S72352K,S72352M,S72352N,S72352P | |
| | S72352Q,S72352R,S72353D,S72353E,S72353F,S72353G,S72353H | |
| | S72353J,S72353K,S72353M,S72353N,S72353P,S72353Q,S72353R | |
| | S72354D,S72354E,S72354F,S72354G,S72354H,S72354J,S72354K | |
| | S72354M,S72354N,S72354P,S72354Q,S72354R,S72355D,S72355E | |
| | S72355F,S72355G,S72355H,S72355J,S72355K,S72355M,S72355N | |
| | S72355P,S72355Q,S72355R,S72356D,S72356E,S72356F,S72356G | |
| | S72356H,S72356J,S72356K,S72356M,S72356N,S72356P,S72356Q | |
| | S72356R,S72361D,S72361E,S72361F,S72361G,S72361H,S72361J | |
| | S72361K,S72361M,S72361N,S72361P,S72361Q,S72361R,S72362D | |
| | S72362E,S72362F,S72362G,S72362H,S72362J,S72362K,S72362M | |
| | S72362N,S72362P,S72362Q,S72362R,S72363D,S72363E,S72363F | |
| | S72363G,S72363H,S72363J,S72363K,S72363M,S72363N,S72363P | |
| | S72363Q,S72363R,S72364D,S72364E,S72364F,S72364G,S72364H | |
| | S72364J,S72364K,S72364M,S72364N,S72364P,S72364Q,S72364R | |
| | S72365D,S72365E,S72365F,S72365G,S72365H,S72365J,S72365K | |
| | S72365M,S72365N,S72365P,S72365Q,S72365R,S72366D,S72366E | |
| | S72366F,S72366G,S72366H,S72366J,S72366K,S72366M,S72366N | |
| | S72366P,S72366Q,S72366R,S72391D,S72391E,S72391F,S72391G | |
| | S72391H,S72391J,S72391K,S72391M,S72391N,S72391P,S72391Q | |
| | S72391R,S72392D,S72392E,S72392F,S72392G,S72392H,S72392J | |

Continued on next page

Table S9 (continued).

| Diagnosis | ICD-10 | ICD-9 |
|---|---|---|
| | S72392K,S72392M,S72392N,S72392P,S72392Q,S72392R,S72399D S72399E,S72399F,S72399G,S72399H,S72399J,S72399K,S72399M S72399N,S72399P,S72399Q,S72399R,S72401D,S72401E,S72401F S72401G,S72401H,S72401J,S72401K,S72401M,S72401N,S72401P S72401Q,S72401R,S72402D,S72402E,S72402F,S72402G,S72402H S72402J,S72402K,S72402M,S72402N,S72402P,S72402Q,S72402R S72409D,S72409E,S72409F,S72409G,S72409H,S72409J,S72409K S72409M,S72409N,S72409P,S72409Q,S72409R,S72411D,S72411E S72411F,S72411G,S72411H,S72411J,S72411K,S72411M,S72411N S72411P,S72411Q,S72411R,S72412D,S72412E,S72412F,S72412G S72412H,S72412J,S72412K,S72412M,S72412N,S72412P,S72412Q S72412R,S72413D,S72413E,S72413F,S72413G,S72413H,S72413J S72413K,S72413M,S72413N,S72413P,S72413Q,S72413R,S72414D S72414E,S72414F,S72414G,S72414H,S72414J,S72414K,S72414M S72414N,S72414P,S72414Q,S72414R,S72415D,S72415E,S72415F S72415G,S72415H,S72415J,S72415K,S72415M,S72415N,S72415P S72415Q,S72415R,S72416D,S72416E,S72416F,S72416G,S72416H S72416J,S72416K,S72416M,S72416N,S72416P,S72416Q,S72416R S72421D,S72421E,S72421F,S72421G,S72421H,S72421J,S72421K S72421M,S72421N,S72421P,S72421Q,S72421R,S72422D,S72422E S72422F,S72422G,S72422H,S72422J,S72422K,S72422M,S72422N S72422P,S72422Q,S72422R,S72423D,S72423E,S72423F,S72423G S72423H,S72423J,S72423K,S72423M,S72423N,S72423P,S72423Q S72423R,S72424D,S72424E,S72424F,S72424G,S72424H,S72424J S72424K,S72424M,S72424N,S72424P,S72424Q,S72424R,S72425D S72425E,S72425F,S72425G,S72425H,S72425J,S72425K,S72425M S72425N,S72425P,S72425Q,S72425R,S72426D,S72426E,S72426F S72426G,S72426H,S72426J,S72426K,S72426M,S72426N,S72426P S72426Q,S72426R,S72431D,S72431E,S72431F,S72431G,S72431H S72431J,S72431K,S72431M,S72431N,S72431P,S72431Q,S72431R S72432D,S72432E,S72432F,S72432G,S72432H,S72432J,S72432K S72432M,S72432N,S72432P,S72432Q,S72432R,S72433D,S72433E S72433F,S72433G,S72433H,S72433J,S72433K,S72433M,S72433N S72433P,S72433Q,S72433R,S72434D,S72434E,S72434F,S72434G S72434H,S72434J,S72434K,S72434M,S72434N,S72434P,S72434Q S72434R,S72435D,S72435E,S72435F,S72435G,S72435H,S72435J S72435K,S72435M,S72435N,S72435P,S72435Q,S72435R,S72436D S72436E,S72436F,S72436G,S72436H,S72436J,S72436K,S72436M S72436N,S72436P,S72436Q,S72436R,S72441D,S72441E,S72441F S72441G,S72441H,S72441J,S72441K,S72441M,S72441N,S72441P S72441Q,S72441R,S72442D,S72442E,S72442F,S72442G,S72442H S72442J,S72442K,S72442M,S72442N,S72442P,S72442Q,S72442R S72443D,S72443E,S72443F,S72443G,S72443H,S72443J,S72443K S72443M,S72443N,S72443P,S72443Q,S72443R,S72444D,S72444E S72444F,S72444G,S72444H,S72444J,S72444K,S72444M,S72444N S72444P,S72444Q,S72444R,S72445D,S72445E,S72445F,S72445G S72445H,S72445J,S72445K,S72445M,S72445N,S72445P,S72445Q S72445R,S72446D,S72446E,S72446F,S72446G,S72446H,S72446J S72446K,S72446M,S72446N,S72446P,S72446Q,S72446R,S72451D S72451E,S72451F,S72451G,S72451H,S72451J,S72451K,S72451M S72451N,S72451P,S72451Q,S72451R,S72452D,S72452E,S72452F S72452G,S72452H,S72452J,S72452K,S72452M,S72452N,S72452P S72452Q,S72452R,S72453D,S72453E,S72453F,S72453G,S72453H S72453J,S72453K,S72453M,S72453N,S72453P,S72453Q,S72453R S72454D,S72454E,S72454F,S72454G,S72454H,S72454J,S72454K S72454M,S72454N,S72454P,S72454Q,S72454R,S72455D,S72455E S72455F,S72455G,S72455H,S72455J,S72455K,S72455M,S72455N S72455P,S72455Q,S72455R,S72456D,S72456E,S72456F,S72456G S72456H,S72456J,S72456K,S72456M,S72456N,S72456P,S72456Q S72456R,S72461D,S72461E,S72461F,S72461G,S72461H,S72461J S72461K,S72461M,S72461N,S72461P,S72461Q,S72461R,S72462D S72462E,S72462F,S72462G,S72462H,S72462J,S72462K,S72462M S72462N,S72462P,S72462Q,S72462R,S72463D,S72463E,S72463F S72463G,S72463H,S72463J,S72463K,S72463M,S72463N,S72463P S72463Q,S72463R,S72464D,S72464E,S72464F,S72464G,S72464H S72464J,S72464K,S72464M,S72464N,S72464P,S72464Q,S72464R S72465D,S72465E,S72465F,S72465G,S72465H,S72465J,S72465K S72465M,S72465N,S72465P,S72465Q,S72465R,S72466D,S72466E S72466F,S72466G,S72466H,S72466J,S72466K,S72466M,S72466N S72466P,S72466Q,S72466R,S72471D,S72471G,S72471K,S72471P S72472D,S72472G,S72472K,S72472P,S72479D,S72479G,S72479K S72479P,S72491D,S72491E,S72491F,S72491G,S72491H,S72491J S72491K,S72491M,S72491N,S72491P,S72491Q,S72491R,S72492D S72492E,S72492F,S72492G,S72492H,S72492J,S72492K,S72492M S72492N,S72492P,S72492Q,S72492R,S72499D,S72499E,S72499F S72499G,S72499H,S72499J,S72499K,S72499M,S72499N,S72499P S72499Q,S72499R,S728X1D,S728X1E,S728X1F,S728X1G,S728X1H S728X1J,S728X1K,S728X1M,S728X1N,S728X1P,S728X1Q,S728X1R S728X2D,S728X2E,S728X2F,S728X2G,S728X2H,S728X2J,S728X2K S728X2M,S728X2N,S728X2P,S728X2Q,S728X2R,S728X9D,S728X9E S728X9F,S728X9G,S728X9H,S728X9J,S728X9K,S728X9M,S728X9N S728X9P,S728X9Q,S728X9R,S7290XD,S7290XE,S7290XF,S7290XG S7290XH,S7290XJ,S7290XK,S7290XM,S7290XN,S7290XP,S7290XQ S7290XR,S7291XD,S7291XE,S7291XF,S7291XG,S7291XH,S7291XJ S7291XK,S7291XM,S7291XN,S7291XP,S7291XQ,S7291XR,S7292XD | |

Continued on next page

Table S9 (continued).

| Diagnosis | ICD-10 | ICD-9 |
|---|---|---|
| | S7292XE,S7292XF,S7292XG,S7292XH,S7292XJ,S7292XK,S7292XM | |
| | S7292XN,S7292XP,S7292XQ,S7292XR,S79001D,S79001G,S79001K | |
| | S79001P,S79002D,S79002G,S79002K,S79002P,S79009D,S79009G | |
| | S79009K,S79009P,S79011D,S79011G,S79011K,S79011P,S79012D | |
| | S79012G,S79012K,S79012P,S79019D,S79019G,S79019K,S79019P | |
| | S79091D,S79091G,S79091K,S79091P,S79092D,S79092G,S79092K | |
| | S79092P,S79099D,S79099G,S79099K,S79099P,S79101D,S79101G | |
| | S79101K,S79101P,S79102D,S79102G,S79102K,S79102P,S79109D | |
| | S79109G,S79109K,S79109P,S79111D,S79111G,S79111K,S79111P | |
| | S79112D,S79112G,S79112K,S79112P,S79119D,S79119G,S79119K | |
| | S79119P,S79121D,S79121G,S79121K,S79121P,S79122D,S79122G | |
| | S79122K,S79122P,S79129D,S79129G,S79129K,S79129P,S79131D | |
| | S79131G,S79131K,S79131P,S79132D,S79132G,S79132K,S79132P | |
| | S79139D,S79139G,S79139K,S79139P,S79141D,S79141G,S79141K | |
| | S79141P,S79142D,S79142G,S79142K,S79142P,S79149D,S79149G | |
| | S79149K,S79149P,S79191D,S79191G,S79191K,S79191P,S79192D | |
| | S79192G,S79192K,S79192P,S79199D,S79199G,S79199K,S79199P | |
| | S82001D,S82001E,S82001F,S82001G,S82001H,S82001J,S82001K | |
| | S82001M,S82001N,S82001P,S82001Q,S82001R,S82002D,S82002E | |
| | S82002F,S82002G,S82002H,S82002J,S82002K,S82002M,S82002N | |
| | S82002P,S82002Q,S82002R,S82009D,S82009E,S82009F,S82009G | |
| | S82009H,S82009J,S82009K,S82009M,S82009N,S82009P,S82009Q | |
| | S82009R,S82011D,S82011E,S82011F,S82011G,S82011H,S82011J | |
| | S82011K,S82011M,S82011N,S82011P,S82011Q,S82011R,S82012D | |
| | S82012E,S82012F,S82012G,S82012H,S82012J,S82012K,S82012M | |
| | S82012N,S82012P,S82012Q,S82012R,S82013D,S82013E,S82013F | |
| | S82013G,S82013H,S82013J,S82013K,S82013M,S82013N,S82013P | |
| | S82013Q,S82013R,S82014D,S82014E,S82014F,S82014G,S82014H | |
| | S82014J,S82014K,S82014M,S82014N,S82014P,S82014Q,S82014R | |
| | S82015D,S82015E,S82015F,S82015G,S82015H,S82015J,S82015K | |
| | S82015M,S82015N,S82015P,S82015Q,S82015R,S82016D,S82016E | |
| | S82016F,S82016G,S82016H,S82016J,S82016K,S82016M,S82016N | |
| | S82016P,S82016Q,S82016R,S82021D,S82021E,S82021F,S82021G | |
| | S82021H,S82021J,S82021K,S82021M,S82021N,S82021P,S82021Q | |
| | S82021R,S82022D,S82022E,S82022F,S82022G,S82022H,S82022J | |
| | S82022K,S82022M,S82022N,S82022P,S82022Q,S82022R,S82023D | |
| | S82023E,S82023F,S82023G,S82023H,S82023J,S82023K,S82023M | |
| | S82023N,S82023P,S82023Q,S82023R,S82024D,S82024E,S82024F | |
| | S82024G,S82024H,S82024J,S82024K,S82024M,S82024N,S82024P | |
| | S82024Q,S82024R,S82025D,S82025E,S82025F,S82025G,S82025H | |
| | S82025J,S82025K,S82025M,S82025N,S82025P,S82025Q,S82025R | |
| | S82026D,S82026E,S82026F,S82026G,S82026H,S82026J,S82026K | |
| | S82026M,S82026N,S82026P,S82026Q,S82026R,S82031D,S82031E | |
| | S82031F,S82031G,S82031H,S82031J,S82031K,S82031M,S82031N | |
| | S82031P,S82031Q,S82031R,S82032D,S82032E,S82032F,S82032G | |
| | S82032H,S82032J,S82032K,S82032M,S82032N,S82032P,S82032Q | |
| | S82032R,S82033D,S82033E,S82033F,S82033G,S82033H,S82033J | |
| | S82033K,S82033M,S82033N,S82033P,S82033Q,S82033R,S82034D | |
| | S82034E,S82034F,S82034G,S82034H,S82034J,S82034K,S82034M | |
| | S82034N,S82034P,S82034Q,S82034R,S82035D,S82035E,S82035F | |
| | S82035G,S82035H,S82035J,S82035K,S82035M,S82035N,S82035P | |
| | S82035Q,S82035R,S82036D,S82036E,S82036F,S82036G,S82036H | |
| | S82036J,S82036K,S82036M,S82036N,S82036P,S82036Q,S82036R | |
| | S82041D,S82041E,S82041F,S82041G,S82041H,S82041J,S82041K | |
| | S82041M,S82041N,S82041P,S82041Q,S82041R,S82042D,S82042E | |
| | S82042F,S82042G,S82042H,S82042J,S82042K,S82042M,S82042N | |
| | S82042P,S82042Q,S82042R,S82043D,S82043E,S82043F,S82043G | |
| | S82043H,S82043J,S82043K,S82043M,S82043N,S82043P,S82043Q | |
| | S82043R,S82044D,S82044E,S82044F,S82044G,S82044H,S82044J | |
| | S82044K,S82044M,S82044N,S82044P,S82044Q,S82044R,S82045D | |
| | S82045E,S82045F,S82045G,S82045H,S82045J,S82045K,S82045M | |
| | S82045N,S82045P,S82045Q,S82045R,S82046D,S82046E,S82046F | |
| | S82046G,S82046H,S82046J,S82046K,S82046M,S82046N,S82046P | |
| | S82046Q,S82046R,S82091D,S82091E,S82091F,S82091G,S82091H | |
| | S82091J,S82091K,S82091M,S82091N,S82091P,S82091Q,S82091R | |
| | S82092D,S82092E,S82092F,S82092G,S82092H,S82092J,S82092K | |
| | S82092M,S82092N,S82092P,S82092Q,S82092R,S82099D,S82099E | |
| | S82099F,S82099G,S82099H,S82099J,S82099K,S82099M,S82099N | |
| | S82099P,S82099Q,S82099R,S82101D,S82101E,S82101F,S82101G | |
| | S82101H,S82101J,S82101K,S82101M,S82101N,S82101P,S82101Q | |
| | S82101R,S82102D,S82102E,S82102F,S82102G,S82102H,S82102J | |
| | S82102K,S82102M,S82102N,S82102P,S82102Q,S82102R,S82109D | |
| | S82109E,S82109F,S82109G,S82109H,S82109J,S82109K,S82109M | |
| | S82109N,S82109P,S82109Q,S82109R,S82111D,S82111E,S82111F | |
| | S82111G,S82111H,S82111J,S82111K,S82111M,S82111N,S82111P | |
| | S82111Q,S82111R,S82112D,S82112E,S82112F,S82112G,S82112H | |
| | S82112J,S82112K,S82112M,S82112N,S82112P,S82112Q,S82112R | |
| | S82113D,S82113E,S82113F,S82113G,S82113H,S82113J,S82113K | |
| | S82113M,S82113N,S82113P,S82113Q,S82113R,S82114D,S82114E | |
| | S82114F,S82114G,S82114H,S82114J,S82114K,S82114M,S82114N | |
| | S82114P,S82114Q,S82114R,S82115D,S82115E,S82115F,S82115G | |
| | S82115H,S82115J,S82115K,S82115M,S82115N,S82115P,S82115Q | |
| | S82115R,S82116D,S82116E,S82116F,S82116G,S82116H,S82116J | |
| | S82116K,S82116M,S82116N,S82116P,S82116Q,S82116R,S82121D | |
| | S82121E,S82121F,S82121G,S82121H,S82121J,S82121K,S82121M | |

Continued on next page

Table S9 (continued).

| Diagnosis | ICD-10 | ICD-9 |
|---|---|---|
| | S82121N,S82121P,S82121Q,S82121R,S82122D,S82122E,S82122F S82122G,S82122H,S82122J,S82122K,S82122M,S82122N,S82122P S82122Q,S82122R,S82123D,S82123E,S82123F,S82123G,S82123H S82123J,S82123K,S82123M,S82123N,S82123P,S82123Q,S82123R S82124D,S82124E,S82124F,S82124G,S82124H,S82124J,S82124K S82124M,S82124N,S82124P,S82124Q,S82124R,S82125D,S82125E S82125F,S82125G,S82125H,S82125J,S82125K,S82125M,S82125N S82125P,S82125Q,S82125R,S82126D,S82126E,S82126F,S82126G S82126H,S82126J,S82126K,S82126M,S82126N,S82126P,S82126Q S82126R,S82131D,S82131E,S82131F,S82131G,S82131H,S82131J S82131K,S82131M,S82131N,S82131P,S82131Q,S82131R,S82132D S82132E,S82132F,S82132G,S82132H,S82132J,S82132K,S82132M S82132N,S82132P,S82132Q,S82132R,S82133D,S82133E,S82133F S82133G,S82133H,S82133J,S82133K,S82133M,S82133N,S82133P S82133Q,S82133R,S82134D,S82134E,S82134F,S82134G,S82134H S82134J,S82134K,S82134M,S82134N,S82134P,S82134Q,S82134R S82135D,S82135E,S82135F,S82135G,S82135H,S82135J,S82135K S82135M,S82135N,S82135P,S82135Q,S82135R,S82136D,S82136E S82136F,S82136G,S82136H,S82136J,S82136K,S82136M,S82136N S82136P,S82136Q,S82136R,S82141D,S82141E,S82141F,S82141G S82141H,S82141J,S82141K,S82141M,S82141N,S82141P,S82141Q S82141R,S82142D,S82142E,S82142F,S82142G,S82142H,S82142J S82142K,S82142M,S82142N,S82142P,S82142Q,S82142R,S82143D S82143E,S82143F,S82143G,S82143H,S82143J,S82143K,S82143M S82143N,S82143P,S82143Q,S82143R,S82144D,S82144E,S82144F S82144G,S82144H,S82144J,S82144K,S82144M,S82144N,S82144P S82144Q,S82144R,S82145D,S82145E,S82145F,S82145G,S82145H S82145J,S82145K,S82145M,S82145N,S82145P,S82145Q,S82145R S82146D,S82146E,S82146F,S82146G,S82146H,S82146J,S82146K S82146M,S82146N,S82146P,S82146Q,S82146R,S82151D,S82151E S82151F,S82151G,S82151H,S82151J,S82151K,S82151M,S82151N S82151P,S82151Q,S82151R,S82152D,S82152E,S82152F,S82152G S82152H,S82152J,S82152K,S82152M,S82152N,S82152P,S82152Q S82152R,S82153D,S82153E,S82153F,S82153G,S82153H,S82153J S82153K,S82153M,S82153N,S82153P,S82153Q,S82153R,S82154D S82154E,S82154F,S82154G,S82154H,S82154J,S82154K,S82154M S82154N,S82154P,S82154Q,S82154R,S82155D,S82155E,S82155F S82155G,S82155H,S82155J,S82155K,S82155M,S82155N,S82155P S82155Q,S82155R,S82156D,S82156E,S82156F,S82156G,S82156H S82156J,S82156K,S82156M,S82156N,S82156P,S82156Q,S82156R S82161D,S82161G,S82161K,S82161P,S82162D,S82162G,S82162K S82162P,S82169D,S82169G,S82169K,S82169P,S82191D,S82191E S82191F,S82191G,S82191H,S82191J,S82191K,S82191M,S82191N S82191P,S82191Q,S82191R,S82192D,S82192E,S82192F,S82192G S82192H,S82192J,S82192K,S82192M,S82192N,S82192P,S82192Q S82192R,S82199D,S82199E,S82199F,S82199G,S82199H,S82199J S82199K,S82199M,S82199N,S82199P,S82199Q,S82199R,S82201D S82201E,S82201F,S82201G,S82201H,S82201J,S82201K,S82201M S82201N,S82201P,S82201Q,S82201R,S82202D,S82202E,S82202F S82202G,S82202H,S82202J,S82202K,S82202M,S82202N,S82202P S82202Q,S82202R,S82209D,S82209E,S82209F,S82209G,S82209H S82209J,S82209K,S82209M,S82209N,S82209P,S82209Q,S82209R S82221D,S82221E,S82221F,S82221G,S82221H,S82221J,S82221K S82221M,S82221N,S82221P,S82221Q,S82221R,S82222D,S82222E S82222F,S82222G,S82222H,S82222J,S82222K,S82222M,S82222N S82222P,S82222Q,S82222R,S82223D,S82223E,S82223F,S82223G S82223H,S82223J,S82223K,S82223M,S82223N,S82223P,S82223Q S82223R,S82224D,S82224E,S82224F,S82224G,S82224H,S82224J S82224K,S82224M,S82224N,S82224P,S82224Q,S82224R,S82225D S82225E,S82225F,S82225G,S82225H,S82225J,S82225K,S82225M S82225N,S82225P,S82225Q,S82225R,S82226D,S82226E,S82226F S82226G,S82226H,S82226J,S82226K,S82226M,S82226N,S82226P S82226Q,S82226R,S82231D,S82231E,S82231F,S82231G,S82231H S82231J,S82231K,S82231M,S82231N,S82231P,S82231Q,S82231R S82232D,S82232E,S82232F,S82232G,S82232H,S82232J,S82232K S82232M,S82232N,S82232P,S82232Q,S82232R,S82233D,S82233E S82233F,S82233G,S82233H,S82233J,S82233K,S82233M,S82233N S82233P,S82233Q,S82233R,S82234D,S82234E,S82234F,S82234G S82234H,S82234J,S82234K,S82234M,S82234N,S82234P,S82234Q S82234R,S82235D,S82235E,S82235F,S82235G,S82235H,S82235J S82235K,S82235M,S82235N,S82235P,S82235Q,S82235R,S82236D S82236E,S82236F,S82236G,S82236H,S82236J,S82236K,S82236M S82236N,S82236P,S82236Q,S82236R,S82241D,S82241E,S82241F S82241G,S82241H,S82241J,S82241K,S82241M,S82241N,S82241P S82241Q,S82241R,S82242D,S82242E,S82242F,S82242G,S82242H S82242J,S82242K,S82242M,S82242N,S82242P,S82242Q,S82242R S82243D,S82243E,S82243F,S82243G,S82243H,S82243J,S82243K S82243M,S82243N,S82243P,S82243Q,S82243R,S82244D,S82244E S82244F,S82244G,S82244H,S82244J,S82244K,S82244M,S82244N S82244P,S82244Q,S82244R,S82245D,S82245E,S82245F,S82245G S82245H,S82245J,S82245K,S82245M,S82245N,S82245P,S82245Q S82245R,S82246D,S82246E,S82246F,S82246G,S82246H,S82246J S82246K,S82246M,S82246N,S82246P,S82246Q,S82246R,S82251D S82251E,S82251F,S82251G,S82251H,S82251J,S82251K,S82251M S82251N,S82251P,S82251Q,S82251R,S82252D,S82252E,S82252F | |

Continued on next page

Table S9 (continued).

| Diagnosis | ICD-10 | ICD-9 |
|---|---|---|
| | S82252G,S82252H,S82252J,S82252K,S82252M,S82252N,S82252P | |
| | S82252Q,S82252R,S82253D,S82253E,S82253F,S82253G,S82253H | |
| | S82253J,S82253K,S82253M,S82253N,S82253P,S82253Q,S82253R | |
| | S82254D,S82254E,S82254F,S82254G,S82254H,S82254J,S82254K | |
| | S82254M,S82254N,S82254P,S82254Q,S82254R,S82255D,S82255E | |
| | S82255F,S82255G,S82255H,S82255J,S82255K,S82255M,S82255N | |
| | S82255P,S82255Q,S82255R,S82256D,S82256E,S82256F,S82256G | |
| | S82256H,S82256J,S82256K,S82256M,S82256N,S82256P,S82256Q | |
| | S82256R,S82261D,S82261E,S82261F,S82261G,S82261H,S82261J | |
| | S82261K,S82261M,S82261N,S82261P,S82261Q,S82261R,S82262D | |
| | S82262E,S82262F,S82262G,S82262H,S82262J,S82262K,S82262M | |
| | S82262N,S82262P,S82262Q,S82262R,S82263D,S82263E,S82263F | |
| | S82263G,S82263H,S82263J,S82263K,S82263M,S82263N,S82263P | |
| | S82263Q,S82263R,S82264D,S82264E,S82264F,S82264G,S82264H | |
| | S82264J,S82264K,S82264M,S82264N,S82264P,S82264Q,S82264R | |
| | S82265D,S82265E,S82265F,S82265G,S82265H,S82265J,S82265K | |
| | S82265M,S82265N,S82265P,S82265Q,S82265R,S82266D,S82266E | |
| | S82266F,S82266G,S82266H,S82266J,S82266K,S82266M,S82266N | |
| | S82266P,S82266Q,S82266R,S82291D,S82291E,S82291F,S82291G | |
| | S82291H,S82291J,S82291K,S82291M,S82291N,S82291P,S82291Q | |
| | S82291R,S82292D,S82292E,S82292F,S82292G,S82292H,S82292J | |
| | S82292K,S82292M,S82292N,S82292P,S82292Q,S82292R,S82299D | |
| | S82299E,S82299F,S82299G,S82299H,S82299J,S82299K,S82299M | |
| | S82299N,S82299P,S82299Q,S82299R,S82301D,S82301E,S82301F | |
| | S82301G,S82301H,S82301J,S82301K,S82301M,S82301N,S82301P | |
| | S82301Q,S82301R,S82302D,S82302E,S82302F,S82302G,S82302H | |
| | S82302J,S82302K,S82302M,S82302N,S82302P,S82302Q,S82302R | |
| | S82309D,S82309E,S82309F,S82309G,S82309H,S82309J,S82309K | |
| | S82309M,S82309N,S82309P,S82309Q,S82309R,S82311D,S82311G | |
| | S82311K,S82311P,S82312D,S82312G,S82312K,S82312P,S82319D | |
| | S82319G,S82319K,S82319P,S82391D,S82391E,S82391F,S82391G | |
| | S82391H,S82391J,S82391K,S82391M,S82391N,S82391P,S82391Q | |
| | S82391R,S82392D,S82392E,S82392F,S82392G,S82392H,S82392J | |
| | S82392K,S82392M,S82392N,S82392P,S82392Q,S82392R,S82399D | |
| | S82399E,S82399F,S82399G,S82399H,S82399J,S82399K,S82399M | |
| | S82399N,S82399P,S82399Q,S82399R,S82401D,S82401E,S82401F | |
| | S82401G,S82401H,S82401J,S82401K,S82401M,S82401N,S82401P | |
| | S82401Q,S82401R,S82402D,S82402E,S82402F,S82402G,S82402H | |
| | S82402J,S82402K,S82402M,S82402N,S82402P,S82402Q,S82402R | |
| | S82409D,S82409E,S82409F,S82409G,S82409H,S82409J,S82409K | |
| | S82409M,S82409N,S82409P,S82409Q,S82409R,S82421D,S82421E | |
| | S82421F,S82421G,S82421H,S82421J,S82421K,S82421M,S82421N | |
| | S82421P,S82421Q,S82421R,S82422D,S82422E,S82422F,S82422G | |
| | S82422H,S82422J,S82422K,S82422M,S82422N,S82422P,S82422Q | |
| | S82422R,S82423D,S82423E,S82423F,S82423G,S82423H,S82423J | |
| | S82423K,S82423M,S82423N,S82423P,S82423Q,S82423R,S82424D | |
| | S82424E,S82424F,S82424G,S82424H,S82424J,S82424K,S82424M | |
| | S82424N,S82424P,S82424Q,S82424R,S82425D,S82425E,S82425F | |
| | S82425G,S82425H,S82425J,S82425K,S82425M,S82425N,S82425P | |
| | S82425Q,S82425R,S82426D,S82426E,S82426F,S82426G,S82426H | |
| | S82426J,S82426K,S82426M,S82426N,S82426P,S82426Q,S82426R | |
| | S82431D,S82431E,S82431F,S82431G,S82431H,S82431J,S82431K | |
| | S82431M,S82431N,S82431P,S82431Q,S82431R,S82432D,S82432E | |
| | S82432F,S82432G,S82432H,S82432J,S82432K,S82432M,S82432N | |
| | S82432P,S82432Q,S82432R,S82433D,S82433E,S82433F,S82433G | |
| | S82433H,S82433J,S82433K,S82433M,S82433N,S82433P,S82433Q | |
| | S82433R,S82434D,S82434E,S82434F,S82434G,S82434H,S82434J | |
| | S82434K,S82434M,S82434N,S82434P,S82434Q,S82434R,S82435D | |
| | S82435E,S82435F,S82435G,S82435H,S82435J,S82435K,S82435M | |
| | S82435N,S82435P,S82435Q,S82435R,S82436D,S82436E,S82436F | |
| | S82436G,S82436H,S82436J,S82436K,S82436M,S82436N,S82436P | |
| | S82436Q,S82436R,S82441D,S82441E,S82441F,S82441G,S82441H | |
| | S82441J,S82441K,S82441M,S82441N,S82441P,S82441Q,S82441R | |
| | S82442D,S82442E,S82442F,S82442G,S82442H,S82442J,S82442K | |
| | S82442M,S82442N,S82442P,S82442Q,S82442R,S82443D,S82443E | |
| | S82443F,S82443G,S82443H,S82443J,S82443K,S82443M,S82443N | |
| | S82443P,S82443Q,S82443R,S82444D,S82444E,S82444F,S82444G | |
| | S82444H,S82444J,S82444K,S82444M,S82444N,S82444P,S82444Q | |
| | S82444R,S82445D,S82445E,S82445F,S82445G,S82445H,S82445J | |
| | S82445K,S82445M,S82445N,S82445P,S82445Q,S82445R,S82446D | |
| | S82446E,S82446F,S82446G,S82446H,S82446J,S82446K,S82446M | |
| | S82446N,S82446P,S82446Q,S82446R,S82451D,S82451E,S82451F | |
| | S82451G,S82451H,S82451J,S82451K,S82451M,S82451N,S82451P | |
| | S82451Q,S82451R,S82452D,S82452E,S82452F,S82452G,S82452H | |
| | S82452J,S82452K,S82452M,S82452N,S82452P,S82452Q,S82452R | |
| | S82453D,S82453E,S82453F,S82453G,S82453H,S82453J,S82453K | |
| | S82453M,S82453N,S82453P,S82453Q,S82453R,S82454D,S82454E | |
| | S82454F,S82454G,S82454H,S82454J,S82454K,S82454M,S82454N | |
| | S82454P,S82454Q,S82454R,S82455D,S82455E,S82455F,S82455G | |
| | S82455H,S82455J,S82455K,S82455M,S82455N,S82455P,S82455Q | |
| | S82455R,S82456D,S82456E,S82456F,S82456G,S82456H,S82456J | |
| | S82456K,S82456M,S82456N,S82456P,S82456Q,S82456R,S82461D | |
| | S82461E,S82461F,S82461G,S82461H,S82461J,S82461K,S82461M | |
| | S82461N,S82461P,S82461Q,S82461R,S82462D,S82462E,S82462F | |
| | S82462G,S82462H,S82462J,S82462K,S82462M,S82462N,S82462P | |

Continued on next page

Table S9 (continued).

| Diagnosis | ICD-10 | ICD-9 |
|---|---|---|
| | S82462Q,S82462R,S82463D,S82463E,S82463F,S82463G,S82463H | |
| | S82463J,S82463K,S82463M,S82463N,S82463P,S82463Q,S82463R | |
| | S82464D,S82464E,S82464F,S82464G,S82464H,S82464J,S82464K | |
| | S82464M,S82464N,S82464P,S82464Q,S82464R,S82465D,S82465E | |
| | S82465F,S82465G,S82465H,S82465J,S82465K,S82465M,S82465N | |
| | S82465P,S82465Q,S82465R,S82466D,S82466E,S82466F,S82466G | |
| | S82466H,S82466J,S82466K,S82466M,S82466N,S82466P,S82466Q | |
| | S82466R,S82491D,S82491E,S82491F,S82491G,S82491H,S82491J | |
| | S82491K,S82491M,S82491N,S82491P,S82491Q,S82491R,S82492D | |
| | S82492E,S82492F,S82492G,S82492H,S82492J,S82492K,S82492M | |
| | S82492N,S82492P,S82492Q,S82492R,S82499D,S82499E,S82499F | |
| | S82499G,S82499H,S82499J,S82499K,S82499M,S82499N,S82499P | |
| | S82499Q,S82499R,S8251XD,S8251XE,S8251XF,S8251XG,S8251XH | |
| | S8251XJ,S8251XK,S8251XM,S8251XN,S8251XP,S8251XQ,S8251XR | |
| | S8252XD,S8252XE,S8252XF,S8252XG,S8252XH,S8252XJ,S8252XK | |
| | S8252XM,S8252XN,S8252XP,S8252XQ,S8252XR,S8253XD,S8253XE | |
| | S8253XF,S8253XG,S8253XH,S8253XJ,S8253XK,S8253XM,S8253XN | |
| | S8253XP,S8253XQ,S8253XR,S8254XD,S8254XE,S8254XF,S8254XG | |
| | S8254XH,S8254XJ,S8254XK,S8254XM,S8254XN,S8254XP,S8254XQ | |
| | S8254XR,S8255XD,S8255XE,S8255XF,S8255XG,S8255XH,S8255XJ | |
| | S8255XK,S8255XM,S8255XN,S8255XP,S8255XQ,S8255XR,S8256XD | |
| | S8256XE,S8256XF,S8256XG,S8256XH,S8256XJ,S8256XK,S8256XM | |
| | S8256XN,S8256XP,S8256XQ,S8256XR,S8261XD,S8261XE,S8261XF | |
| | S8261XG,S8261XH,S8261XJ,S8261XK,S8261XM,S8261XN,S8261XP | |
| | S8261XQ,S8261XR,S8262XD,S8262XE,S8262XF,S8262XG,S8262XH | |
| | S8262XJ,S8262XK,S8262XM,S8262XN,S8262XP,S8262XQ,S8262XR | |
| | S8263XD,S8263XE,S8263XF,S8263XG,S8263XH,S8263XJ,S8263XK | |
| | S8263XM,S8263XN,S8263XP,S8263XQ,S8263XR,S8264XD,S8264XE | |
| | S8264XF,S8264XG,S8264XH,S8264XJ,S8264XK,S8264XM,S8264XN | |
| | S8264XP,S8264XQ,S8264XR,S8265XD,S8265XE,S8265XF,S8265XG | |
| | S8265XH,S8265XJ,S8265XK,S8265XM,S8265XN,S8265XP,S8265XQ | |
| | S8265XR,S8266XD,S8266XE,S8266XF,S8266XG,S8266XH,S8266XJ | |
| | S8266XK,S8266XM,S8266XN,S8266XP,S8266XQ,S8266XR,S82811D | |
| | S82811G,S82811K,S82811P,S82812D,S82812G,S82812K,S82812P | |
| | S82819D,S82819G,S82819K,S82819P,S82821D,S82821G,S82821K | |
| | S82821P,S82822D,S82822G,S82822K,S82822P,S82829D,S82829G | |
| | S82829K,S82829P,S82831D,S82831E,S82831F,S82831G,S82831H | |
| | S82831J,S82831K,S82831M,S82831N,S82831P,S82831Q,S82831R | |
| | S82832D,S82832E,S82832F,S82832G,S82832H,S82832J,S82832K | |
| | S82832M,S82832N,S82832P,S82832Q,S82832R,S82839D,S82839E | |
| | S82839F,S82839G,S82839H,S82839J,S82839K,S82839M,S82839N | |
| | S82839P,S82839Q,S82839R,S82841D,S82841E,S82841F,S82841G | |
| | S82841H,S82841J,S82841K,S82841M,S82841N,S82841P,S82841Q | |
| | S82841R,S82842D,S82842E,S82842F,S82842G,S82842H,S82842J | |
| | S82842K,S82842M,S82842N,S82842P,S82842Q,S82842R,S82843D | |
| | S82843E,S82843F,S82843G,S82843H,S82843J,S82843K,S82843M | |
| | S82843N,S82843P,S82843Q,S82843R,S82844D,S82844E,S82844F | |
| | S82844G,S82844H,S82844J,S82844K,S82844M,S82844N,S82844P | |
| | S82844Q,S82844R,S82845D,S82845E,S82845F,S82845G,S82845H | |
| | S82845J,S82845K,S82845M,S82845N,S82845P,S82845Q,S82845R | |
| | S82846D,S82846E,S82846F,S82846G,S82846H,S82846J,S82846K | |
| | S82846M,S82846N,S82846P,S82846Q,S82846R,S82851D,S82851E | |
| | S82851F,S82851G,S82851H,S82851J,S82851K,S82851M,S82851N | |
| | S82851P,S82851Q,S82851R,S82852D,S82852E,S82852F,S82852G | |
| | S82852H,S82852J,S82852K,S82852M,S82852N,S82852P,S82852Q | |
| | S82852R,S82853D,S82853E,S82853F,S82853G,S82853H,S82853J | |
| | S82853K,S82853M,S82853N,S82853P,S82853Q,S82853R,S82854D | |
| | S82854E,S82854F,S82854G,S82854H,S82854J,S82854K,S82854M | |
| | S82854N,S82854P,S82854Q,S82854R,S82855D,S82855E,S82855F | |
| | S82855G,S82855H,S82855J,S82855K,S82855M,S82855N,S82855P | |
| | S82855Q,S82855R,S82856D,S82856E,S82856F,S82856G,S82856H | |
| | S82856J,S82856K,S82856M,S82856N,S82856P,S82856Q,S82856R | |
| | S82861D,S82861E,S82861F,S82861G,S82861H,S82861J,S82861K | |
| | S82861M,S82861N,S82861P,S82861Q,S82861R,S82862D,S82862E | |
| | S82862F,S82862G,S82862H,S82862J,S82862K,S82862M,S82862N | |
| | S82862P,S82862Q,S82862R,S82863D,S82863E,S82863F,S82863G | |
| | S82863H,S82863J,S82863K,S82863M,S82863N,S82863P,S82863Q | |
| | S82863R,S82864D,S82864E,S82864F,S82864G,S82864H,S82864J | |
| | S82864K,S82864M,S82864N,S82864P,S82864Q,S82864R,S82865D | |
| | S82865E,S82865F,S82865G,S82865H,S82865J,S82865K,S82865M | |
| | S82865N,S82865P,S82865Q,S82865R,S82866D,S82866E,S82866F | |
| | S82866G,S82866H,S82866J,S82866K,S82866M,S82866N,S82866P | |
| | S82866Q,S82866R,S82871D,S82871E,S82871F,S82871G,S82871H | |
| | S82871J,S82871K,S82871M,S82871N,S82871P,S82871Q,S82871R | |
| | S82872D,S82872E,S82872F,S82872G,S82872H,S82872J,S82872K | |
| | S82872M,S82872N,S82872P,S82872Q,S82872R,S82873D,S82873E | |
| | S82873F,S82873G,S82873H,S82873J,S82873K,S82873M,S82873N | |
| | S82873P,S82873Q,S82873R,S82874D,S82874E,S82874F,S82874G | |
| | S82874H,S82874J,S82874K,S82874M,S82874N,S82874P,S82874Q | |
| | S82874R,S82875D,S82875E,S82875F,S82875G,S82875H,S82875J | |
| | S82875K,S82875M,S82875N,S82875P,S82875Q,S82875R,S82876D | |
| | S82876E,S82876F,S82876G,S82876H,S82876J,S82876K,S82876M | |
| | S82876N,S82876P,S82876Q,S82876R,S82891D,S82891E,S82891F | |
| | S82891G,S82891H,S82891J,S82891K,S82891M,S82891N,S82891P | |
| | S82891Q,S82891R,S82892D,S82892E,S82892F,S82892G,S82892H | |

Continued on next page

Table S9 (continued).

| Diagnosis | ICD-10 | ICD-9 |
|---|---|---|
| | S82892J,S82892K,S82892M,S82892N,S82892P,S82892Q,S82892R | |
| | S82899D,S82899E,S82899F,S82899G,S82899H,S82899J,S82899K | |
| | S82899M,S82899N,S82899P,S82899Q,S82899R,S8290XD,S8290XE | |
| | S8290XF,S8290XG,S8290XH,S8290XJ,S8290XK,S8290XM,S8290XN | |
| | S8290XP,S8290XQ,S8290XR,S8291XD,S8291XE,S8291XF,S8291XG | |
| | S8291XH,S8291XJ,S8291XK,S8291XM,S8291XN,S8291XP,S8291XQ | |
| | S8291XR,S8292XD,S8292XE,S8292XF,S8292XG,S8292XH,S8292XJ | |
| | S8292XK,S8292XM,S8292XN,S8292XP,S8292XQ,S8292XR,S89001D | |
| | S89001G,S89001K,S89001P,S89002D,S89002G,S89002K,S89002P | |
| | S89009D,S89009G,S89009K,S89009P,S89011D,S89011G,S89011K | |
| | S89011P,S89012D,S89012G,S89012K,S89012P,S89019D,S89019G | |
| | S89019K,S89019P,S89021D,S89021G,S89021K,S89021P,S89022D | |
| | S89022G,S89022K,S89022P,S89029D,S89029G,S89029K,S89029P | |
| | S89031D,S89031G,S89031K,S89031P,S89032D,S89032G,S89032K | |
| | S89032P,S89039D,S89039G,S89039K,S89039P,S89041D,S89041G | |
| | S89041K,S89041P,S89042D,S89042G,S89042K,S89042P,S89049D | |
| | S89049G,S89049K,S89049P,S89091D,S89091G,S89091K,S89091P | |
| | S89092D,S89092G,S89092K,S89092P,S89099D,S89099G,S89099K | |
| | S89099P,S89101D,S89101G,S89101K,S89101P,S89102D,S89102G | |
| | S89102K,S89102P,S89109D,S89109G,S89109K,S89109P,S89111D | |
| | S89111G,S89111K,S89111P,S89112D,S89112G,S89112K,S89112P | |
| | S89119D,S89119G,S89119K,S89119P,S89121D,S89121G,S89121K | |
| | S89121P,S89122D,S89122G,S89122K,S89122P,S89129D,S89129G | |
| | S89129K,S89129P,S89131D,S89131G,S89131K,S89131P,S89132D | |
| | S89132G,S89132K,S89132P,S89139D,S89139G,S89139K,S89139P | |
| | S89141D,S89141G,S89141K,S89141P,S89142D,S89142G,S89142K | |
| | S89142P,S89149D,S89149G,S89149K,S89149P,S89191D,S89191G | |
| | S89191K,S89191P,S89192D,S89192G,S89192K,S89192P,S89199D | |
| | S89199G,S89199K,S89199P,S89201D,S89201G,S89201K,S89201P | |
| | S89202D,S89202G,S89202K,S89202P,S89209D,S89209G,S89209K | |
| | S89209P,S89211D,S89211G,S89211K,S89211P,S89212D,S89212G | |
| | S89212K,S89212P,S89219D,S89219G,S89219K,S89219P,S89221D | |
| | S89221G,S89221K,S89221P,S89222D,S89222G,S89222K,S89222P | |
| | S89229D,S89229G,S89229K,S89229P,S89291D,S89291G,S89291K | |
| | S89291P,S89292D,S89292G,S89292K,S89292P,S89299D,S89299G | |
| | S89299K,S89299P,S89301D,S89301G,S89301K,S89301P,S89302D | |
| | S89302G,S89302K,S89302P,S89309D,S89309G,S89309K,S89309P | |
| | S89311D,S89311G,S89311K,S89311P,S89312D,S89312G,S89312K | |
| | S89312P,S89319D,S89319G,S89319K,S89319P,S89321D,S89321G | |
| | S89321K,S89321P,S89322D,S89322G,S89322K,S89322P,S89329D | |
| | S89329G,S89329K,S89329P,S89391D,S89391G,S89391K,S89391P | |
| | S89392D,S89392G,S89392K,S89392P,S89399D,S89399G,S89399K | |
| | S89399P,S92001D,S92001G,S92001K,S92001P,S92002D,S92002G | |
| | S92002K,S92002P,S92009D,S92009G,S92009K,S92009P,S92011D | |
| | S92011G,S92011K,S92011P,S92012D,S92012G,S92012K,S92012P | |
| | S92013D,S92013G,S92013K,S92013P,S92014D,S92014G,S92014K | |
| | S92014P,S92015D,S92015G,S92015K,S92015P,S92016D,S92016G | |
| | S92016K,S92016P,S92021D,S92021G,S92021K,S92021P,S92022D | |
| | S92022G,S92022K,S92022P,S92023D,S92023G,S92023K,S92023P | |
| | S92024D,S92024G,S92024K,S92024P,S92025D,S92025G,S92025K | |
| | S92025P,S92026D,S92026G,S92026K,S92026P,S92031D,S92031G | |
| | S92031K,S92031P,S92032D,S92032G,S92032K,S92032P,S92033D | |
| | S92033G,S92033K,S92033P,S92034D,S92034G,S92034K,S92034P | |
| | S92035D,S92035G,S92035K,S92035P,S92036D,S92036G,S92036K | |
| | S92036P,S92041D,S92041G,S92041K,S92041P,S92042D,S92042G | |
| | S92042K,S92042P,S92043D,S92043G,S92043K,S92043P,S92044D | |
| | S92044G,S92044K,S92044P,S92045D,S92045G,S92045K,S92045P | |
| | S92046D,S92046G,S92046K,S92046P,S92051D,S92051G,S92051K | |
| | S92051P,S92052D,S92052G,S92052K,S92052P,S92053D,S92053G | |
| | S92053K,S92053P,S92054D,S92054G,S92054K,S92054P,S92055D | |
| | S92055G,S92055K,S92055P,S92056D,S92056G,S92056K,S92056P | |
| | S92061D,S92061G,S92061K,S92061P,S92062D,S92062G,S92062K | |
| | S92062P,S92063D,S92063G,S92063K,S92063P,S92064D,S92064G | |
| | S92064K,S92064P,S92065D,S92065G,S92065K,S92065P,S92066D | |
| | S92066G,S92066K,S92066P,S92101D,S92101G,S92101K,S92101P | |
| | S92102D,S92102G,S92102K,S92102P,S92109D,S92109G,S92109K | |
| | S92109P,S92111D,S92111G,S92111K,S92111P,S92112D,S92112G | |
| | S92112K,S92112P,S92113D,S92113G,S92113K,S92113P,S92114D | |
| | S92114G,S92114K,S92114P,S92115D,S92115G,S92115K,S92115P | |
| | S92116D,S92116G,S92116K,S92116P,S92121D,S92121G,S92121K | |
| | S92121P,S92122D,S92122G,S92122K,S92122P,S92123D,S92123G | |
| | S92123K,S92123P,S92124D,S92124G,S92124K,S92124P,S92125D | |
| | S92125G,S92125K,S92125P,S92126D,S92126G,S92126K,S92126P | |
| | S92131D,S92131G,S92131K,S92131P,S92132D,S92132G,S92132K | |
| | S92132P,S92133D,S92133G,S92133K,S92133P,S92134D,S92134G | |
| | S92134K,S92134P,S92135D,S92135G,S92135K,S92135P,S92136D | |
| | S92136G,S92136K,S92136P,S92141D,S92141G,S92141K,S92141P | |
| | S92142D,S92142G,S92142K,S92142P,S92143D,S92143G,S92143K | |
| | S92143P,S92144D,S92144G,S92144K,S92144P,S92145D,S92145G | |
| | S92145K,S92145P,S92146D,S92146G,S92146K,S92146P,S92151D | |
| | S92151G,S92151K,S92151P,S92152D,S92152G,S92152K,S92152P | |
| | S92153D,S92153G,S92153K,S92153P,S92154D,S92154G,S92154K | |
| | S92154P,S92155D,S92155G,S92155K,S92155P,S92156D,S92156G | |
| | S92156K,S92156P,S92191D,S92191G,S92191K,S92191P,S92192D | |
| | S92192G,S92192K,S92192P,S92199D,S92199G,S92199K,S92199P | |

Continued on next page

Table S9 (continued).

| Diagnosis | ICD-10 | ICD-9 |
|---|---|---|
| | S92201D,S92201G,S92201K,S92201P,S92202D,S92202G,S92202K S92202P,S92209D,S92209G,S92209K,S92209P,S92211D,S92211G S92211K,S92211P,S92212D,S92212G,S92212K,S92212P,S92213D S92213G,S92213K,S92213P,S92214D,S92214G,S92214K,S92214P S92215D,S92215G,S92215K,S92215P,S92216D,S92216G,S92216K S92216P,S92221D,S92221G,S92221K,S92221P,S92222D,S92222G S92222K,S92222P,S92223D,S92223G,S92223K,S92223P,S92224D S92224G,S92224K,S92224P,S92225D,S92225G,S92225K,S92225P S92226D,S92226G,S92226K,S92226P,S92231D,S92231G,S92231K S92231P,S92232D,S92232G,S92232K,S92232P,S92233D,S92233G S92233K,S92233P,S92234D,S92234G,S92234K,S92234P,S92235D S92235G,S92235K,S92235P,S92236D,S92236G,S92236K,S92236P S92241D,S92241G,S92241K,S92241P,S92242D,S92242G,S92242K S92242P,S92243D,S92243G,S92243K,S92243P,S92244D,S92244G S92244K,S92244P,S92245D,S92245G,S92245K,S92245P,S92246D S92246G,S92246K,S92246P,S92251D,S92251G,S92251K,S92251P S92252D,S92252G,S92252K,S92252P,S92253D,S92253G,S92253K S92253P,S92254D,S92254G,S92254K,S92254P,S92255D,S92255G S92255K,S92255P,S92256D,S92256G,S92256K,S92256P,S92301D S92301G,S92301K,S92301P,S92302D,S92302G,S92302K,S92302P S92309D,S92309G,S92309K,S92309P,S92311D,S92311G,S92311K S92311P,S92312D,S92312G,S92312K,S92312P,S92313D,S92313G S92313K,S92313P,S92314D,S92314G,S92314K,S92314P,S92315D S92315G,S92315K,S92315P,S92316D,S92316G,S92316K,S92316P S92321D,S92321G,S92321K,S92321P,S92322D,S92322G,S92322K S92322P,S92323D,S92323G,S92323K,S92323P,S92324D,S92324G S92324K,S92324P,S92325D,S92325G,S92325K,S92325P,S92326D S92326G,S92326K,S92326P,S92331D,S92331G,S92331K,S92331P S92332D,S92332G,S92332K,S92332P,S92333D,S92333G,S92333K S92333P,S92334D,S92334G,S92334K,S92334P,S92335D,S92335G S92335K,S92335P,S92336D,S92336G,S92336K,S92336P,S92341D S92341G,S92341K,S92341P,S92342D,S92342G,S92342K,S92342P S92343D,S92343G,S92343K,S92343P,S92344D,S92344G,S92344K S92344P,S92345D,S92345G,S92345K,S92345P,S92346D,S92346G S92346K,S92346P,S92351D,S92351G,S92351K,S92351P,S92352D S92352G,S92352K,S92352P,S92353D,S92353G,S92353K,S92353P S92354D,S92354G,S92354K,S92354P,S92355D,S92355G,S92355K S92355P,S92356D,S92356G,S92356K,S92356P,S92401D,S92401G S92401K,S92401P,S92402D,S92402G,S92402K,S92402P,S92403D S92403G,S92403K,S92403P,S92404D,S92404G,S92404K,S92404P S92405D,S92405G,S92405K,S92405P,S92406D,S92406G,S92406K S92406P,S92411D,S92411G,S92411K,S92411P,S92412D,S92412G S92412K,S92412P,S92413D,S92413G,S92413K,S92413P,S92414D S92414G,S92414K,S92414P,S92415D,S92415G,S92415K,S92415P S92416D,S92416G,S92416K,S92416P,S92421D,S92421G,S92421K S92421P,S92422D,S92422G,S92422K,S92422P,S92423D,S92423G S92423K,S92423P,S92424D,S92424G,S92424K,S92424P,S92425D S92425G,S92425K,S92425P,S92426D,S92426G,S92426K,S92426P S92491D,S92491G,S92491K,S92491P,S92492D,S92492G,S92492K S92492P,S92499D,S92499G,S92499K,S92499P,S92501D,S92501G S92501K,S92501P,S92502D,S92502G,S92502K,S92502P,S92503D S92503G,S92503K,S92503P,S92504D,S92504G,S92504K,S92504P S92505D,S92505G,S92505K,S92505P,S92506D,S92506G,S92506K S92506P,S92511D,S92511G,S92511K,S92511P,S92512D,S92512G S92512K,S92512P,S92513D,S92513G,S92513K,S92513P,S92514D S92514G,S92514K,S92514P,S92515D,S92515G,S92515K,S92515P S92516D,S92516G,S92516K,S92516P,S92521D,S92521G,S92521K S92521P,S92522D,S92522G,S92522K,S92522P,S92523D,S92523G S92523K,S92523P,S92524D,S92524G,S92524K,S92524P,S92525D S92525G,S92525K,S92525P,S92526D,S92526G,S92526K,S92526P S92531D,S92531G,S92531K,S92531P,S92532D,S92532G,S92532K S92532P,S92533D,S92533G,S92533K,S92533P,S92534D,S92534G S92534K,S92534P,S92535D,S92535G,S92535K,S92535P,S92536D S92536G,S92536K,S92536P,S92591D,S92591G,S92591K,S92591P S92592D,S92592G,S92592K,S92592P,S92599D,S92599G,S92599K S92599P,S92811D,S92811G,S92811K,S92811P,S92812D,S92812G S92812K,S92812P,S92819D,S92819G,S92819K,S92819P,S92901D S92901G,S92901K,S92901P,S92902D,S92902G,S92902K,S92902P S92909D,S92909G,S92909K,S92909P,S92911D,S92911G,S92911K S92911P,S92912D,S92912G,S92912K,S92912P,S92919D,S92919G S92919K,S92919P,S99001D,S99001G,S99001K,S99001P,S99002D S99002G,S99002K,S99002P,S99009D,S99009G,S99009K,S99009P S99011D,S99011G,S99011K,S99011P,S99012D,S99012G,S99012K S99012P,S99019D,S99019G,S99019K,S99019P,S99021D,S99021G S99021K,S99021P,S99022D,S99022G,S99022K,S99022P,S99029D S99029G,S99029K,S99029P,S99031D,S99031G,S99031K,S99031P S99032D,S99032G,S99032K,S99032P,S99039D,S99039G,S99039K S99039P,S99041D,S99041G,S99041K,S99041P,S99042D,S99042G S99042K,S99042P,S99049D,S99049G,S99049K,S99049P,S99091D S99091G,S99091K,S99091P,S99092D,S99092G,S99092K,S99092P S99099D,S99099G,S99099K,S99099P,S99101D,S99101G,S99101K S99101P,S99102D,S99102G,S99102K,S99102P,S99109D,S99109G S99109K,S99109P,S99111D,S99111G,S99111K,S99111P,S99112D S99112G,S99112K,S99112P,S99119D,S99119G,S99119K,S99119P S99121D,S99121G,S99121K,S99121P,S99122D,S99122G,S99122K | |

Continued on next page

Table S9 (continued).

| Diagnosis | ICD-10 | ICD-9 |
|---|---|---|
| | S99122P,S99129D,S99129G,S99129K,S99129P,S99131D,S99131G<br>S99131K,S99131P,S99132D,S99132G,S99132K,S99132P,S99139D<br>S99139G,S99139K,S99139P,S99141D,S99141G,S99141K,S99141P<br>S99142D,S99142G,S99142K,S99142P,S99149D,S99149G,S99149K<br>S99149P,S99191D,S99191G,S99191K,S99191P,S99192D,S99192G<br>S99192K,S99192P,S99199D,S99199G,S99199K,S99199P,S99201D<br>S99201G,S99201K,S99201P,S99202D,S99202G,S99202K,S99202P<br>S99209D,S99209G,S99209K,S99209P,S99211D,S99211G,S99211K<br>S99211P,S99212D,S99212G,S99212K,S99212P,S99219D,S99219G<br>S99219K,S99219P,S99221D,S99221G,S99221K,S99221P,S99222D<br>S99222G,S99222K,S99222P,S99229D,S99229G,S99229K,S99229P<br>S99231D,S99231G,S99231K,S99231P,S99232D,S99232G,S99232K<br>S99232P,S99239D,S99239G,S99239K,S99239P,S99241D,S99241G<br>S99241K,S99241P,S99242D,S99242G,S99242K,S99242P,S99249D<br>S99249G,S99249K,S99249P,S99291D,S99291G,S99291K,S99291P<br>S99292D,S99292G,S99292K,S99292P,S99299D,S99299G,S99299K<br>S99299P,T84042D,T84043D,T84048D,T84049D | |
| Fracture of the lower limb (except hip), initial encounter | M9711XA,M9712XA,M9721XA,M9722XA,M978XXA,M979XXA,S72301A<br>S72301B,S72301C,S72302A,S72302B,S72302C,S72309A,S72309B<br>S72309C,S72321A,S72321B,S72321C,S72322A,S72322B,S72322C<br>S72323A,S72323B,S72323C,S72324A,S72324B,S72324C,S72325A<br>S72325B,S72325C,S72326A,S72326B,S72326C,S72331A,S72331B<br>S72331C,S72332A,S72332B,S72332C,S72333A,S72333B,S72333C<br>S72334A,S72334B,S72334C,S72335A,S72335B,S72335C,S72336A<br>S72336B,S72336C,S72341A,S72341B,S72341C,S72342A,S72342B<br>S72342C,S72343A,S72343B,S72343C,S72344A,S72344B,S72344C<br>S72345A,S72345B,S72345C,S72346A,S72346B,S72346C,S72351A<br>S72351B,S72351C,S72352A,S72352B,S72352C,S72353A,S72353B<br>S72353C,S72354A,S72354B,S72354C,S72355A,S72355B,S72355C<br>S72356A,S72356B,S72356C,S72361A,S72361B,S72361C,S72362A<br>S72362B,S72362C,S72363A,S72363B,S72363C,S72364A,S72364B<br>S72364C,S72365A,S72365B,S72365C,S72366A,S72366B,S72366C<br>S72391A,S72391B,S72391C,S72392A,S72392B,S72392C,S72399A<br>S72399B,S72399C,S72401A,S72401B,S72401C,S72402A,S72402B<br>S72402C,S72409A,S72409B,S72409C,S72411A,S72411B,S72411C<br>S72412A,S72412B,S72412C,S72413A,S72413B,S72413C,S72414A<br>S72414B,S72414C,S72415A,S72415B,S72415C,S72416A,S72416B<br>S72416C,S72421A,S72421B,S72421C,S72422A,S72422B,S72422C<br>S72423A,S72423B,S72423C,S72424A,S72424B,S72424C,S72425A<br>S72425B,S72425C,S72426A,S72426B,S72426C,S72431A,S72431B<br>S72431C,S72432A,S72432B,S72432C,S72433A,S72433B,S72433C<br>S72434A,S72434B,S72434C,S72435A,S72435B,S72435C,S72436A<br>S72436B,S72436C,S72441A,S72441B,S72441C,S72442A,S72442B<br>S72442C,S72443A,S72443B,S72443C,S72444A,S72444B,S72444C<br>S72445A,S72445B,S72445C,S72446A,S72446B,S72446C,S72451A<br>S72451B,S72451C,S72452A,S72452B,S72452C,S72453A,S72453B<br>S72453C,S72454A,S72454B,S72454C,S72455A,S72455B,S72455C<br>S72456A,S72456B,S72456C,S72461A,S72461B,S72461C,S72462A<br>S72462B,S72462C,S72463A,S72463B,S72463C,S72464A,S72464B<br>S72464C,S72465A,S72465B,S72465C,S72466A,S72466B,S72466C<br>S72471A,S72472A,S72479A,S72491A,S72491B,S72491C,S72492A<br>S72492B,S72492C,S72499A,S72499B,S72499C,S728X1A,S728X1B<br>S728X1C,S728X2A,S728X2B,S728X2C,S728X9A,S728X9B,S728X9C<br>S7290XA,S7290XB,S7290XC,S7291XA,S7291XB,S7291XC,S7292XA<br>S7292XB,S7292XC,S79001A,S79002A,S79009A,S79011A,S79012A<br>S79019A,S79091A,S79092A,S79099A,S79101A,S79102A,S79109A<br>S79111A,S79112A,S79119A,S79121A,S79122A,S79129A,S79131A<br>S79132A,S79139A,S79141A,S79142A,S79149A,S79191A,S79192A<br>S79199A,S82001A,S82001B,S82001C,S82002A,S82002B,S82002C<br>S82009A,S82009B,S82009C,S82011A,S82011B,S82011C,S82012A<br>S82012B,S82012C,S82013A,S82013B,S82013C,S82014A,S82014B<br>S82014C,S82015A,S82015B,S82015C,S82016A,S82016B,S82016C<br>S82021A,S82021B,S82021C,S82022A,S82022B,S82022C,S82023A<br>S82023B,S82023C,S82024A,S82024B,S82024C,S82025A,S82025B<br>S82025C,S82026A,S82026B,S82026C,S82031A,S82031B,S82031C<br>S82032A,S82032B,S82032C,S82033A,S82033B,S82033C,S82034A<br>S82034B,S82034C,S82035A,S82035B,S82035C,S82036A,S82036B<br>S82036C,S82041A,S82041B,S82041C,S82042A,S82042B,S82042C<br>S82043A,S82043B,S82043C,S82044A,S82044B,S82044C,S82045A<br>S82045B,S82045C,S82046A,S82046B,S82046C,S82091A,S82091B<br>S82091C,S82092A,S82092B,S82092C,S82099A,S82099B,S82099C<br>S82101A,S82101B,S82101C,S82102A,S82102B,S82102C,S82109A<br>S82109B,S82109C,S82111A,S82111B,S82111C,S82112A,S82112B<br>S82112C,S82113A,S82113B,S82113C,S82114A,S82114B,S82114C<br>S82115A,S82115B,S82115C,S82116A,S82116B,S82116C,S82121A<br>S82121B,S82121C,S82122A,S82122B,S82122C,S82123A,S82123B<br>S82123C,S82124A,S82124B,S82124C,S82125A,S82125B,S82125C<br>S82126A,S82126B,S82126C,S82131A,S82131B,S82131C,S82132A | 82001,82100,82101,82110,82111,82120,<br>82121,82122,82123,82129<br>82130,82131,82132,82133,82139,8220,<br>8221,82300,82301,82310<br>82311,82320,82321,82330,82331,82340,<br>82341,82380,82381,82390<br>82391,8240,8241,8242,8243,8244,8245,<br>8246,8247,8248,8249,8250<br>8251,82520,82521,82522,82523,82524,<br>82525,82529,82530,82531<br>82532,82533,82534,82535,82539,8260,<br>8261,8270,8271,99644 |

Continued on next page

Table S9 (continued).

| Diagnosis | ICD-10 | ICD-9 |
|---|---|---|
| | S82132B,S82132C,S82133A,S82133B,S82133C,S82134A,S82134B | |
| | S82134C,S82135A,S82135B,S82135C,S82136A,S82136B,S82136C | |
| | S82141A,S82141B,S82141C,S82142A,S82142B,S82142C,S82143A | |
| | S82143B,S82143C,S82144A,S82144B,S82144C,S82145A,S82145B | |
| | S82145C,S82146A,S82146B,S82146C,S82151A,S82151B,S82151C | |
| | S82152A,S82152B,S82152C,S82153A,S82153B,S82153C,S82154A | |
| | S82154B,S82154C,S82155A,S82155B,S82155C,S82156A,S82156B | |
| | S82156C,S82161A,S82162A,S82169A,S82191A,S82191B,S82191C | |
| | S82192A,S82192B,S82192C,S82199A,S82199B,S82199C,S82201A | |
| | S82201B,S82201C,S82202A,S82202B,S82202C,S82209A,S82209B | |
| | S82209C,S82221A,S82221B,S82221C,S82222A,S82222B,S82222C | |
| | S82223A,S82223B,S82223C,S82224A,S82224B,S82224C,S82225A | |
| | S82225B,S82225C,S82226A,S82226B,S82226C,S82231A,S82231B | |
| | S82231C,S82232A,S82232B,S82232C,S82233A,S82233B,S82233C | |
| | S82234A,S82234B,S82234C,S82235A,S82235B,S82235C,S82236A | |
| | S82236B,S82236C,S82241A,S82241B,S82241C,S82242A,S82242B | |
| | S82242C,S82243A,S82243B,S82243C,S82244A,S82244B,S82244C | |
| | S82245A,S82245B,S82245C,S82246A,S82246B,S82246C,S82251A | |
| | S82251B,S82251C,S82252A,S82252B,S82252C,S82253A,S82253B | |
| | S82253C,S82254A,S82254B,S82254C,S82255A,S82255B,S82255C | |
| | S82256A,S82256B,S82256C,S82261A,S82261B,S82261C,S82262A | |
| | S82262B,S82262C,S82263A,S82263B,S82263C,S82264A,S82264B | |
| | S82264C,S82265A,S82265B,S82265C,S82266A,S82266B,S82266C | |
| | S82291A,S82291B,S82291C,S82292A,S82292B,S82292C,S82299A | |
| | S82299B,S82299C,S82301A,S82301B,S82301C,S82302A,S82302B | |
| | S82302C,S82309A,S82309B,S82309C,S82311A,S82312A,S82319A | |
| | S82391A,S82391B,S82391C,S82392A,S82392B,S82392C,S82399A | |
| | S82399B,S82399C,S82401A,S82401B,S82401C,S82402A,S82402B | |
| | S82402C,S82409A,S82409B,S82409C,S82421A,S82421B,S82421C | |
| | S82422A,S82422B,S82422C,S82423A,S82423B,S82423C,S82424A | |
| | S82424B,S82424C,S82425A,S82425B,S82425C,S82426A,S82426B | |
| | S82426C,S82431A,S82431B,S82431C,S82432A,S82432B,S82432C | |
| | S82433A,S82433B,S82433C,S82434A,S82434B,S82434C,S82435A | |
| | S82435B,S82435C,S82436A,S82436B,S82436C,S82441A,S82441B | |
| | S82441C,S82442A,S82442B,S82442C,S82443A,S82443B,S82443C | |
| | S82444A,S82444B,S82444C,S82445A,S82445B,S82445C,S82446A | |
| | S82446B,S82446C,S82451A,S82451B,S82451C,S82452A,S82452B | |
| | S82452C,S82453A,S82453B,S82453C,S82454A,S82454B,S82454C | |
| | S82455A,S82455B,S82455C,S82456A,S82456B,S82456C,S82461A | |
| | S82461B,S82461C,S82462A,S82462B,S82462C,S82463A,S82463B | |
| | S82463C,S82464A,S82464B,S82464C,S82465A,S82465B,S82465C | |
| | S82466A,S82466B,S82466C,S82491A,S82491B,S82491C,S82492A | |
| | S82492B,S82492C,S82499A,S82499B,S82499C,S8251XA,S8251XB | |
| | S8251XC,S8252XA,S8252XB,S8252XC,S8253XA,S8253XB,S8253XC | |
| | S8254XA,S8254XB,S8254XC,S8255XA,S8255XB,S8255XC,S8256XA | |
| | S8256XB,S8256XC,S8261XA,S8261XB,S8261XC,S8262XA,S8262XB | |
| | S8262XC,S8263XA,S8263XB,S8263XC,S8264XA,S8264XB,S8264XC | |
| | S8265XA,S8265XB,S8265XC,S8266XA,S8266XB,S8266XC,S82811A | |
| | S82812A,S82819A,S82821A,S82822A,S82829A,S82831A,S82831B | |
| | S82831C,S82832A,S82832B,S82832C,S82839A,S82839B,S82839C | |
| | S82841A,S82841B,S82841C,S82842A,S82842B,S82842C,S82843A | |
| | S82843B,S82843C,S82844A,S82844B,S82844C,S82845A,S82845B | |
| | S82845C,S82846A,S82846B,S82846C,S82851A,S82851B,S82851C | |
| | S82852A,S82852B,S82852C,S82853A,S82853B,S82853C,S82854A | |
| | S82854B,S82854C,S82855A,S82855B,S82855C,S82856A,S82856B | |
| | S82856C,S82861A,S82861B,S82861C,S82862A,S82862B,S82862C | |
| | S82863A,S82863B,S82863C,S82864A,S82864B,S82864C,S82865A | |
| | S82865B,S82865C,S82866A,S82866B,S82866C,S82871A,S82871B | |
| | S82871C,S82872A,S82872B,S82872C,S82873A,S82873B,S82873C | |
| | S82874A,S82874B,S82874C,S82875A,S82875B,S82875C,S82876A | |
| | S82876B,S82876C,S82891A,S82891B,S82891C,S82892A,S82892B | |
| | S82892C,S82899A,S82899B,S82899C,S8290XA,S8290XB,S8290XC | |
| | S8291XA,S8291XB,S8291XC,S8292XA,S8292XB,S8292XC,S89001A | |
| | S89002A,S89009A,S89011A,S89012A,S89019A,S89021A,S89022A | |
| | S89029A,S89031A,S89032A,S89039A,S89041A,S89042A,S89049A | |
| | S89091A,S89092A,S89099A,S89101A,S89102A,S89109A,S89111A | |
| | S89112A,S89119A,S89121A,S89122A,S89129A,S89131A,S89132A | |
| | S89139A,S89141A,S89142A,S89149A,S89191A,S89192A,S89199A | |
| | S89201A,S89202A,S89209A,S89211A,S89212A,S89219A,S89221A | |
| | S89222A,S89229A,S89291A,S89292A,S89299A,S89301A,S89302A | |
| | S89309A,S89311A,S89312A,S89319A,S89321A,S89322A,S89329A | |
| | S89391A,S89392A,S89399A,S92001A,S92001B,S92002A,S92002B | |
| | S92009A,S92009B,S92011A,S92011B,S92012A,S92012B,S92013A | |
| | S92013B,S92014A,S92014B,S92015A,S92015B,S92016A,S92016B | |
| | S92021A,S92021B,S92022A,S92022B,S92023A,S92023B,S92024A | |
| | S92024B,S92025A,S92025B,S92026A,S92026B,S92031A,S92031B | |
| | S92032A,S92032B,S92033A,S92033B,S92034A,S92034B,S92035A | |
| | S92035B,S92036A,S92036B,S92041A,S92041B,S92042A,S92042B | |
| | S92043A,S92043B,S92044A,S92044B,S92045A,S92045B,S92046A | |
| | S92046B,S92051A,S92051B,S92052A,S92052B,S92053A,S92053B | |
| | S92054A,S92054B,S92055A,S92055B,S92056A,S92056B,S92061A | |
| | S92061B,S92062A,S92062B,S92063A,S92063B,S92064A,S92064B | |
| | S92065A,S92065B,S92066A,S92066B,S92101A,S92101B,S92102A | |
| | S92102B,S92109A,S92109B,S92111A,S92111B,S92112A,S92112B | |
| | S92113A,S92113B,S92114A,S92114B,S92115A,S92115B,S92116A | |

Continued on next page

Table S9 (continued).

| Diagnosis | ICD-10 | ICD-9 |
|---|---|---|
| | S92116B,S92121A,S92121B,S92122A,S92122B,S92123A,S92123B | |
| | S92124A,S92124B,S92125A,S92125B,S92126A,S92126B,S92131A | |
| | S92131B,S92132A,S92132B,S92133A,S92133B,S92134A,S92134B | |
| | S92135A,S92135B,S92136A,S92136B,S92141A,S92141B,S92142A | |
| | S92142B,S92143A,S92143B,S92144A,S92144B,S92145A,S92145B | |
| | S92146A,S92146B,S92151A,S92151B,S92152A,S92152B,S92153A | |
| | S92153B,S92154A,S92154B,S92155A,S92155B,S92156A,S92156B | |
| | S92191A,S92191B,S92192A,S92192B,S92199A,S92199B,S92201A | |
| | S92201B,S92202A,S92202B,S92209A,S92209B,S92211A,S92211B | |
| | S92212A,S92212B,S92213A,S92213B,S92214A,S92214B,S92215A | |
| | S92215B,S92216A,S92216B,S92221A,S92221B,S92222A,S92222B | |
| | S92223A,S92223B,S92224A,S92224B,S92225A,S92225B,S92226A | |
| | S92226B,S92231A,S92231B,S92232A,S92232B,S92233A,S92233B | |
| | S92234A,S92234B,S92235A,S92235B,S92236A,S92236B,S92241A | |
| | S92241B,S92242A,S92242B,S92243A,S92243B,S92244A,S92244B | |
| | S92245A,S92245B,S92246A,S92246B,S92251A,S92251B,S92252A | |
| | S92252B,S92253A,S92253B,S92254A,S92254B,S92255A,S92255B | |
| | S92256A,S92256B,S92301A,S92301B,S92302A,S92302B,S92309A | |
| | S92309B,S92311A,S92311B,S92312A,S92312B,S92313A,S92313B | |
| | S92314A,S92314B,S92315A,S92315B,S92316A,S92316B,S92321A | |
| | S92321B,S92322A,S92322B,S92323A,S92323B,S92324A,S92324B | |
| | S92325A,S92325B,S92326A,S92326B,S92331A,S92331B,S92332A | |
| | S92332B,S92333A,S92333B,S92334A,S92334B,S92335A,S92335B | |
| | S92336A,S92336B,S92341A,S92341B,S92342A,S92342B,S92343A | |
| | S92343B,S92344A,S92344B,S92345A,S92345B,S92346A,S92346B | |
| | S92351A,S92351B,S92352A,S92352B,S92353A,S92353B,S92354A | |
| | S92354B,S92355A,S92355B,S92356A,S92356B,S92401A,S92401B | |
| | S92402A,S92402B,S92403A,S92403B,S92404A,S92404B,S92405A | |
| | S92405B,S92406A,S92406B,S92411A,S92411B,S92412A,S92412B | |
| | S92413A,S92413B,S92414A,S92414B,S92415A,S92415B,S92416A | |
| | S92416B,S92421A,S92421B,S92422A,S92422B,S92423A,S92423B | |
| | S92424A,S92424B,S92425A,S92425B,S92426A,S92426B,S92491A | |
| | S92491B,S92492A,S92492B,S92499A,S92499B,S92501A,S92501B | |
| | S92502A,S92502B,S92503A,S92503B,S92504A,S92504B,S92505A | |
| | S92505B,S92506A,S92506B,S92511A,S92511B,S92512A,S92512B | |
| | S92513A,S92513B,S92514A,S92514B,S92515A,S92515B,S92516A | |
| | S92516B,S92521A,S92521B,S92522A,S92522B,S92523A,S92523B | |
| | S92524A,S92524B,S92525A,S92525B,S92526A,S92526B,S92531A | |
| | S92531B,S92532A,S92532B,S92533A,S92533B,S92534A,S92534B | |
| | S92535A,S92535B,S92536A,S92536B,S92591A,S92591B,S92592A | |
| | S92592B,S92599A,S92599B,S92811A,S92811B,S92812A,S92812B | |
| | S92819A,S92819B,S92901A,S92901B,S92902A,S92902B,S92909A | |
| | S92909B,S92911A,S92911B,S92912A,S92912B,S92919A,S92919B | |
| | S99001A,S99001B,S99002A,S99002B,S99009A,S99009B,S99011A | |
| | S99011B,S99012A,S99012B,S99019A,S99019B,S99021A,S99021B | |
| | S99022A,S99022B,S99029A,S99029B,S99031A,S99031B,S99032A | |
| | S99032B,S99039A,S99039B,S99041A,S99041B,S99042A,S99042B | |
| | S99049A,S99049B,S99091A,S99091B,S99092A,S99092B,S99099A | |
| | S99099B,S99101A,S99101B,S99102A,S99102B,S99109A,S99109B | |
| | S99111A,S99111B,S99112A,S99112B,S99119A,S99119B,S99121A | |
| | S99121B,S99122A,S99122B,S99129A,S99129B,S99131A,S99131B | |
| | S99132A,S99132B,S99139A,S99139B,S99141A,S99141B,S99142A | |
| | S99142B,S99149A,S99149B,S99191A,S99191B,S99192A,S99192B | |
| | S99199A,S99199B,S99201A,S99201B,S99202A,S99202B,S99209A | |
| | S99209B,S99211A,S99211B,S99212A,S99212B,S99219A,S99219B | |
| | S99221A,S99221B,S99222A,S99222B,S99229A,S99229B,S99231A | |
| | S99231B,S99232A,S99232B,S99239A,S99239B,S99241A,S99241B | |
| | S99242A,S99242B,S99249A,S99249B,S99291A,S99291B,S99292A | |
| | S99292B,S99299A,S99299B,T84042A,T84043A,T84048A,T84049A | |
| Fracture of the neck | M9701XA,M9702XA,S72001A,S72001B,S72001C,S72002A,S72002B | 82000,82001,82002,82003,82009,82010, 82011,82012,82013,82019 |
| of the femur (hip), | S72002C,S72009A,S72009B,S72009C,S72011A,S72011B,S72011C | 82020,82021,82022,82030,82031,82032, 8208,8209,99644 |
| initial encounter | S72012A,S72012B,S72012C,S72019A,S72019B,S72019C,S72021A | |
| | S72021B,S72021C,S72022A,S72022B,S72022C,S72023A,S72023B | |
| | S72023C,S72024A,S72024B,S72024C,S72025A,S72025B,S72025C | |
| | S72026A,S72026B,S72026C,S72031A,S72031B,S72031C,S72032A | |
| | S72032B,S72032C,S72033A,S72033B,S72033C,S72034A,S72034B | |
| | S72034C,S72035A,S72035B,S72035C,S72036A,S72036B,S72036C | |
| | S72041A,S72041B,S72041C,S72042A,S72042B,S72042C,S72043A | |
| | S72043B,S72043C,S72044A,S72044B,S72044C,S72045A,S72045B | |
| | S72045C,S72046A,S72046B,S72046C,S72051A,S72051B,S72051C | |
| | S72052A,S72052B,S72052C,S72059A,S72059B,S72059C,S72061A | |
| | S72061B,S72061C,S72062A,S72062B,S72062C,S72063A,S72063B | |
| | S72063C,S72064A,S72064B,S72064C,S72065A,S72065B,S72065C | |
| | S72066A,S72066B,S72066C,S72091A,S72091B,S72091C,S72092A | |
| | S72092B,S72092C,S72099A,S72099B,S72099C,S72101A,S72101B | |
| | S72101C,S72102A,S72102B,S72102C,S72109A,S72109B,S72109C | |
| | S72111A,S72111B,S72111C,S72112A,S72112B,S72112C,S72113A | |
| | S72113B,S72113C,S72114A,S72114B,S72114C,S72115A,S72115B | |
| | S72115C,S72116A,S72116B,S72116C,S72121A,S72121B,S72121C | |
| | S72122A,S72122B,S72122C,S72123A,S72123B,S72123C,S72124A | |
| | S72124B,S72124C,S72125A,S72125B,S72125C,S72126A,S72126B | |
| | S72126C,S72131A,S72131B,S72131C,S72132A,S72132B,S72132C | |

Continued on next page

Table S9 (continued).

| Diagnosis | ICD-10 | ICD-9 |
| --- | --- | --- |
| | S72133A,S72133B,S72133C,S72134A,S72134B,S72134C,S72135A<br>S72135B,S72135C,S72136A,S72136B,S72136C,S72141A,S72141B<br>S72141C,S72142A,S72142B,S72142C,S72143A,S72143B,S72143C<br>S72144A,S72144B,S72144C,S72145A,S72145B,S72145C,S72146A<br>S72146B,S72146C,S7221XA,S7221XB,S7221XC,S7222XA,S7222XB<br>S7222XC,S7223XA,S7223XB,S7223XC,S7224XA,S7224XB,S7224XC<br>S7225XA,S7225XB,S7225XC,S7226XA,S7226XB,S7226XC,T84040A<br>T84041A | |
| Fracture of the neck of the femur (hip), subsequent encounter | M9701XD,M9702XD,S72001D,S72001E,S72001F,S72001G,S72001H<br>S72001J,S72001K,S72001M,S72001N,S72001P,S72001Q,S72001R<br>S72002D,S72002E,S72002F,S72002G,S72002H,S72002J,S72002K<br>S72002M,S72002N,S72002P,S72002Q,S72002R,S72009D,S72009E<br>S72009F,S72009G,S72009H,S72009J,S72009K,S72009M,S72009N<br>S72009P,S72009Q,S72009R,S72011D,S72011E,S72011F,S72011G<br>S72011H,S72011J,S72011K,S72011M,S72011N,S72011P,S72011Q<br>S72011R,S72012D,S72012E,S72012F,S72012G,S72012H,S72012J<br>S72012K,S72012M,S72012N,S72012P,S72012Q,S72012R,S72019D<br>S72019E,S72019F,S72019G,S72019H,S72019J,S72019K,S72019M<br>S72019N,S72019P,S72019Q,S72019R,S72021D,S72021E,S72021F<br>S72021G,S72021H,S72021J,S72021K,S72021M,S72021N,S72021P<br>S72021Q,S72021R,S72022D,S72022E,S72022F,S72022G,S72022H<br>S72022J,S72022K,S72022M,S72022N,S72022P,S72022Q,S72022R<br>S72023D,S72023E,S72023F,S72023G,S72023H,S72023J,S72023K<br>S72023M,S72023N,S72023P,S72023Q,S72023R,S72024D,S72024E<br>S72024F,S72024G,S72024H,S72024J,S72024K,S72024M,S72024N<br>S72024P,S72024Q,S72024R,S72025D,S72025E,S72025F,S72025G<br>S72025H,S72025J,S72025K,S72025M,S72025N,S72025P,S72025Q<br>S72025R,S72026D,S72026E,S72026F,S72026G,S72026H,S72026J<br>S72026K,S72026M,S72026N,S72026P,S72026Q,S72026R,S72031D<br>S72031E,S72031F,S72031G,S72031H,S72031J,S72031K,S72031M<br>S72031N,S72031P,S72031Q,S72031R,S72032D,S72032E,S72032F<br>S72032G,S72032H,S72032J,S72032K,S72032M,S72032N,S72032P<br>S72032Q,S72032R,S72033D,S72033E,S72033F,S72033G,S72033H<br>S72033J,S72033K,S72033M,S72033N,S72033P,S72033Q,S72033R<br>S72034D,S72034E,S72034F,S72034G,S72034H,S72034J,S72034K<br>S72034M,S72034N,S72034P,S72034Q,S72034R,S72035D,S72035E<br>S72035F,S72035G,S72035H,S72035J,S72035K,S72035M,S72035N<br>S72035P,S72035Q,S72035R,S72036D,S72036E,S72036F,S72036G<br>S72036H,S72036J,S72036K,S72036M,S72036N,S72036P,S72036Q<br>S72036R,S72041D,S72041E,S72041F,S72041G,S72041H,S72041J<br>S72041K,S72041M,S72041N,S72041P,S72041Q,S72041R,S72042D<br>S72042E,S72042F,S72042G,S72042H,S72042J,S72042K,S72042M<br>S72042N,S72042P,S72042Q,S72042R,S72043D,S72043E,S72043F<br>S72043G,S72043H,S72043J,S72043K,S72043M,S72043N,S72043P<br>S72043Q,S72043R,S72044D,S72044E,S72044F,S72044G,S72044H<br>S72044J,S72044K,S72044M,S72044N,S72044P,S72044Q,S72044R<br>S72045D,S72045E,S72045F,S72045G,S72045H,S72045J,S72045K<br>S72045M,S72045N,S72045P,S72045Q,S72045R,S72046D,S72046E<br>S72046F,S72046G,S72046H,S72046J,S72046K,S72046M,S72046N<br>S72046P,S72046Q,S72046R,S72051D,S72051E,S72051F,S72051G<br>S72051H,S72051J,S72051K,S72051M,S72051N,S72051P,S72051Q<br>S72051R,S72052D,S72052E,S72052F,S72052G,S72052H,S72052J<br>S72052K,S72052M,S72052N,S72052P,S72052Q,S72052R,S72059D<br>S72059E,S72059F,S72059G,S72059H,S72059J,S72059K,S72059M<br>S72059N,S72059P,S72059Q,S72059R,S72061D,S72061E,S72061F<br>S72061G,S72061H,S72061J,S72061K,S72061M,S72061N,S72061P<br>S72061Q,S72061R,S72062D,S72062E,S72062F,S72062G,S72062H<br>S72062J,S72062K,S72062M,S72062N,S72062P,S72062Q,S72062R<br>S72063D,S72063E,S72063F,S72063G,S72063H,S72063J,S72063K<br>S72063M,S72063N,S72063P,S72063Q,S72063R,S72064D,S72064E<br>S72064F,S72064G,S72064H,S72064J,S72064K,S72064M,S72064N<br>S72064P,S72064Q,S72064R,S72065D,S72065E,S72065F,S72065G<br>S72065H,S72065J,S72065K,S72065M,S72065N,S72065P,S72065Q<br>S72065R,S72066D,S72066E,S72066F,S72066G,S72066H,S72066J<br>S72066K,S72066M,S72066N,S72066P,S72066Q,S72066R,S72091D<br>S72091E,S72091F,S72091G,S72091H,S72091J,S72091K,S72091M<br>S72091N,S72091P,S72091Q,S72091R,S72092D,S72092E,S72092F<br>S72092G,S72092H,S72092J,S72092K,S72092M,S72092N,S72092P<br>S72092Q,S72092R,S72099D,S72099E,S72099F,S72099G,S72099H<br>S72099J,S72099K,S72099M,S72099N,S72099P,S72099Q,S72099R<br>S72101D,S72101E,S72101F,S72101G,S72101H,S72101J,S72101K<br>S72101M,S72101N,S72101P,S72101Q,S72101R,S72102D,S72102E<br>S72102F,S72102G,S72102H,S72102J,S72102K,S72102M,S72102N<br>S72102P,S72102Q,S72102R,S72109D,S72109E,S72109F,S72109G<br>S72109H,S72109J,S72109K,S72109M,S72109N,S72109P,S72109Q<br>S72109R,S72111D,S72111E,S72111F,S72111G,S72111H,S72111J<br>S72111K,S72111M,S72111N,S72111P,S72111Q,S72111R,S72112D<br>S72112E,S72112F,S72112G,S72112H,S72112J,S72112K,S72112M<br>S72112N,S72112P,S72112Q,S72112R,S72113D,S72113E,S72113F<br>S72113G,S72113H,S72113J,S72113K,S72113M,S72113N,S72113P<br>S72113Q,S72113R,S72114D,S72114E,S72114F,S72114G,S72114H<br>S72114J,S72114K,S72114M,S72114N,S72114P,S72114Q,S72114R<br>S72115D,S72115E,S72115F,S72115G,S72115H,S72115J,S72115K<br>S72115M,S72115N,S72115P,S72115Q,S72115R,S72116D,S72116E | 73381,73382,V5413,V5415,V5423 |

Continued on next page

Table S9 (continued).

| Diagnosis | ICD-10 | ICD-9 |
|---|---|---|
| | S72116F,S72116G,S72116H,S72116J,S72116K,S72116M,S72116N | |
| | S72116P,S72116Q,S72116R,S72121D,S72121E,S72121F,S72121G | |
| | S72121H,S72121J,S72121K,S72121M,S72121N,S72121P,S72121Q | |
| | S72121R,S72122D,S72122E,S72122F,S72122G,S72122H,S72122J | |
| | S72122K,S72122M,S72122N,S72122P,S72122Q,S72122R,S72123D | |
| | S72123E,S72123F,S72123G,S72123H,S72123J,S72123K,S72123M | |
| | S72123N,S72123P,S72123Q,S72123R,S72124D,S72124E,S72124F | |
| | S72124G,S72124H,S72124J,S72124K,S72124M,S72124N,S72124P | |
| | S72124Q,S72124R,S72125D,S72125E,S72125F,S72125G,S72125H | |
| | S72125J,S72125K,S72125M,S72125N,S72125P,S72125Q,S72125R | |
| | S72126D,S72126E,S72126F,S72126G,S72126H,S72126J,S72126K | |
| | S72126M,S72126N,S72126P,S72126Q,S72126R,S72131D,S72131E | |
| | S72131F,S72131G,S72131H,S72131J,S72131K,S72131M,S72131N | |
| | S72131P,S72131Q,S72131R,S72132D,S72132E,S72132F,S72132G | |
| | S72132H,S72132J,S72132K,S72132M,S72132N,S72132P,S72132Q | |
| | S72132R,S72133D,S72133E,S72133F,S72133G,S72133H,S72133J | |
| | S72133K,S72133M,S72133N,S72133P,S72133Q,S72133R,S72134D | |
| | S72134E,S72134F,S72134G,S72134H,S72134J,S72134K,S72134M | |
| | S72134N,S72134P,S72134Q,S72134R,S72135D,S72135E,S72135F | |
| | S72135G,S72135H,S72135J,S72135K,S72135M,S72135N,S72135P | |
| | S72135Q,S72135R,S72136D,S72136E,S72136F,S72136G,S72136H | |
| | S72136J,S72136K,S72136M,S72136N,S72136P,S72136Q,S72136R | |
| | S72141D,S72141E,S72141F,S72141G,S72141H,S72141J,S72141K | |
| | S72141M,S72141N,S72141P,S72141Q,S72141R,S72142D,S72142E | |
| | S72142F,S72142G,S72142H,S72142J,S72142K,S72142M,S72142N | |
| | S72142P,S72142Q,S72142R,S72143D,S72143E,S72143F,S72143G | |
| | S72143H,S72143J,S72143K,S72143M,S72143N,S72143P,S72143Q | |
| | S72143R,S72144D,S72144E,S72144F,S72144G,S72144H,S72144J | |
| | S72144K,S72144M,S72144N,S72144P,S72144Q,S72144R,S72145D | |
| | S72145E,S72145F,S72145G,S72145H,S72145J,S72145K,S72145M | |
| | S72145N,S72145P,S72145Q,S72145R,S72146D,S72146E,S72146F | |
| | S72146G,S72146H,S72146J,S72146K,S72146M,S72146N,S72146P | |
| | S72146Q,S72146R,S7221XD,S7221XE,S7221XF,S7221XG,S7221XH | |
| | S7221XJ,S7221XK,S7221XM,S7221XN,S7221XP,S7221XQ,S7221XR | |
| | S7222XD,S7222XE,S7222XF,S7222XG,S7222XH,S7222XJ,S7222XK | |
| | S7222XM,S7222XN,S7222XP,S7222XQ,S7222XR,S7223XD,S7223XE | |
| | S7223XF,S7223XG,S7223XH,S7223XJ,S7223XK,S7223XM,S7223XN | |
| | S7223XP,S7223XQ,S7223XR,S7224XD,S7224XE,S7224XF,S7224XG | |
| | S7224XH,S7224XJ,S7224XK,S7224XM,S7224XN,S7224XP,S7224XQ | |
| | S7224XR,S7225XD,S7225XE,S7225XF,S7225XG,S7225XH,S7225XJ | |
| | S7225XK,S7225XM,S7225XN,S7225XP,S7225XQ,S7225XR,S7226XD | |
| | S7226XE,S7226XF,S7226XG,S7226XH,S7226XJ,S7226XK,S7226XM | |
| | S7226XN,S7226XP,S7226XQ,S7226XR,T84040D,T84041D | |
| Fracture of the spine | S12000A,S12000B,S12001A,S12001B,S1201XA,S1201XB,S1202XA | 80500,80501,80502,80503,80504,80505,<br>80506,80507,80511,80512 |
| and back, initial | S1202XB,S12030A,S12030B,S12031A,S12031B,S12040A,S12040B | 80513,80514,80515,80516,80517,8052,<br>8053,8054,8055,8056,8057 |
| encounter | S12041A,S12041B,S12090A,S12090B,S12091A,S12091B,S12100A | 8075,80849,80859,8088,8089 |
| | S12100B,S12101A,S12101B,S12110A,S12110B,S12111A,S12111B | |
| | S12112A,S12112B,S12120A,S12120B,S12121A,S12121B,S12130A | |
| | S12130B,S12131A,S12131B,S1214XA,S1214XB,S12150A,S12150B | |
| | S12151A,S12151B,S12190A,S12190B,S12191A,S12191B,S12200A | |
| | S12200B,S12201A,S12201B,S12230A,S12230B,S12231A,S12231B | |
| | S1224XA,S1224XB,S12250A,S12250B,S12251A,S12251B,S12290A | |
| | S12290B,S12291A,S12291B,S12300A,S12300B,S12301A,S12301B | |
| | S12330A,S12330B,S12331A,S12331B,S1234XA,S1234XB,S12350A | |
| | S12350B,S12351A,S12351B,S12390A,S12390B,S12391A,S12391B | |
| | S12400A,S12400B,S12401A,S12401B,S12430A,S12430B,S12431A | |
| | S12431B,S1244XA,S1244XB,S12450A,S12450B,S12451A,S12451B | |
| | S12490A,S12490B,S12491A,S12491B,S12500A,S12500B,S12501A | |
| | S12501B,S12530A,S12530B,S12531A,S12531B,S1254XA,S1254XB | |
| | S12550A,S12550B,S12551A,S12551B,S12590A,S12590B,S12591A | |
| | S12591B,S12600A,S12600B,S12601A,S12601B,S12630A,S12630B | |
| | S12631A,S12631B,S1264XA,S1264XB,S12650A,S12650B,S12651A | |
| | S12651B,S12690A,S12690B,S12691A,S12691B,S128XXA,S129XXA | |
| | S22000A,S22000B,S22001A,S22001B,S22002A,S22002B,S22008A | |
| | S22008B,S22009A,S22009B,S22010A,S22010B,S22011A,S22011B | |
| | S22012A,S22012B,S22018A,S22018B,S22019A,S22019B,S22020A | |
| | S22020B,S22021A,S22021B,S22022A,S22022B,S22028A,S22028B | |
| | S22029A,S22029B,S22030A,S22030B,S22031A,S22031B,S22032A | |
| | S22032B,S22038A,S22038B,S22039A,S22039B,S22040A,S22040B | |
| | S22041A,S22041B,S22042A,S22042B,S22048A,S22048B,S22049A | |
| | S22049B,S22050A,S22050B,S22051A,S22051B,S22052A,S22052B | |
| | S22058A,S22058B,S22059A,S22059B,S22060A,S22060B,S22061A | |
| | S22061B,S22062A,S22062B,S22068A,S22068B,S22069A,S22069B | |
| | S22070A,S22070B,S22071A,S22071B,S22072A,S22072B,S22078A | |
| | S22078B,S22079A,S22079B,S22080A,S22080B,S22081A,S22081B | |
| | S22082A,S22082B,S22088A,S22088B,S22089A,S22089B,S32000A | |
| | S32000B,S32001A,S32001B,S32002A,S32002B,S32008A,S32008B | |
| | S32009A,S32009B,S32010A,S32010B,S32011A,S32011B,S32012A | |
| | S32012B,S32018A,S32018B,S32019A,S32019B,S32020A,S32020B | |
| | S32021A,S32021B,S32022A,S32022B,S32028A,S32028B,S32029A | |
| | S32029B,S32030A,S32030B,S32031A,S32031B,S32032A,S32032B | |
| | S32038A,S32038B,S32039A,S32039B,S32040A,S32040B,S32041A | |

Continued on next page

**Table S9 (continued).**

| Diagnosis | ICD-10 | ICD-9 |
|---|---|---|
| | S32041B,S32042A,S32042B,S32048A,S32048B,S32049A,S32049B<br>S32050A,S32050B,S32051A,S32051B,S32052A,S32052B,S32058A<br>S32058B,S32059A,S32059B,S3210XA,S3210XB,S32110A,S32110B<br>S32111A,S32111B,S32112A,S32112B,S32119A,S32119B,S32120A<br>S32120B,S32121A,S32121B,S32122A,S32122B,S32129A,S32129B<br>S32130A,S32130B,S32131A,S32131B,S32132A,S32132B,S32139A<br>S32139B,S3214XA,S3214XB,S3215XA,S3215XB,S3216XA,S3216XB<br>S3217XA,S3217XB,S3219XA,S3219XB,S322XXA,S322XXB,S329XXA<br>S329XXB | |
| Fracture of the spine and back, subsequent encounter | S12000D,S12000G,S12000K,S12001D,S12001G,S12001K,S1201XD<br>S1201XG,S1201XK,S1202XD,S1202XG,S1202XK,S12030D,S12030G<br>S12030K,S12031D,S12031G,S12031K,S12040D,S12040G,S12040K<br>S12041D,S12041G,S12041K,S12090D,S12090G,S12090K,S12091D<br>S12091G,S12091K,S12100D,S12100G,S12100K,S12101D,S12101G<br>S12101K,S12110D,S12110G,S12110K,S12111D,S12111G,S12111K<br>S12112D,S12112G,S12112K,S12120D,S12120G,S12120K,S12121D<br>S12121G,S12121K,S12130D,S12130G,S12130K,S12131D,S12131G<br>S12131K,S1214XD,S1214XG,S1214XK,S12150D,S12150G,S12150K<br>S12151D,S12151G,S12151K,S12190D,S12190G,S12190K,S12191D<br>S12191G,S12191K,S12200D,S12200G,S12200K,S12201D,S12201G<br>S12201K,S12230D,S12230G,S12230K,S12231D,S12231G,S12231K<br>S1224XD,S1224XG,S1224XK,S12250D,S12250G,S12250K,S12251D<br>S12251G,S12251K,S12290D,S12290G,S12290K,S12291D,S12291G<br>S12291K,S12300D,S12300G,S12300K,S12301D,S12301G,S12301K<br>S12330D,S12330G,S12330K,S12331D,S12331G,S12331K,S1234XD<br>S1234XG,S1234XK,S12350D,S12350G,S12350K,S12351D,S12351G<br>S12351K,S12390D,S12390G,S12390K,S12391D,S12391G,S12391K<br>S12400D,S12400G,S12400K,S12401D,S12401G,S12401K,S12430D<br>S12430G,S12430K,S12431D,S12431G,S12431K,S1244XD,S1244XG<br>S1244XK,S12450D,S12450G,S12450K,S12451D,S12451G,S12451K<br>S12490D,S12490G,S12490K,S12491D,S12491G,S12491K,S12500D<br>S12500G,S12500K,S12501D,S12501G,S12501K,S12530D,S12530G<br>S12530K,S12531D,S12531G,S12531K,S1254XD,S1254XG,S1254XK<br>S12550D,S12550G,S12550K,S12551D,S12551G,S12551K,S12590D<br>S12590G,S12590K,S12591D,S12591G,S12591K,S12600D,S12600G<br>S12600K,S12601D,S12601G,S12601K,S12630D,S12630G,S12630K<br>S12631D,S12631G,S12631K,S1264XD,S1264XG,S1264XK,S12650D<br>S12650G,S12650K,S12651D,S12651G,S12651K,S12690D,S12690G<br>S12690K,S12691D,S12691G,S12691K,S128XXD,S129XXD,S22000D<br>S22000G,S22000K,S22001D,S22001G,S22001K,S22002D,S22002G<br>S22002K,S22008D,S22008G,S22008K,S22009D,S22009G,S22009K<br>S22010D,S22010G,S22010K,S22011D,S22011G,S22011K,S22012D<br>S22012G,S22012K,S22018D,S22018G,S22018K,S22019D,S22019G<br>S22019K,S22020D,S22020G,S22020K,S22021D,S22021G,S22021K<br>S22022D,S22022G,S22022K,S22028D,S22028G,S22028K,S22029D<br>S22029G,S22029K,S22030D,S22030G,S22030K,S22031D,S22031G<br>S22031K,S22032D,S22032G,S22032K,S22038D,S22038G,S22038K<br>S22039D,S22039G,S22039K,S22040D,S22040G,S22040K,S22041D<br>S22041G,S22041K,S22042D,S22042G,S22042K,S22048D,S22048G<br>S22048K,S22049D,S22049G,S22049K,S22050D,S22050G,S22050K<br>S22051D,S22051G,S22051K,S22052D,S22052G,S22052K,S22058D<br>S22058G,S22058K,S22059D,S22059G,S22059K,S22060D,S22060G<br>S22060K,S22061D,S22061G,S22061K,S22062D,S22062G,S22062K<br>S22068D,S22068G,S22068K,S22069D,S22069G,S22069K,S22070D<br>S22070G,S22070K,S22071D,S22071G,S22071K,S22072D,S22072G<br>S22072K,S22078D,S22078G,S22078K,S22079D,S22079G,S22079K<br>S22080D,S22080G,S22080K,S22081D,S22081G,S22081K,S22082D<br>S22082G,S22082K,S22088D,S22088G,S22088K,S22089D,S22089G<br>S22089K,S32000D,S32000G,S32000K,S32001D,S32001G,S32001K<br>S32002D,S32002G,S32002K,S32008D,S32008G,S32008K,S32009D<br>S32009G,S32009K,S32010D,S32010G,S32010K,S32011D,S32011G<br>S32011K,S32012D,S32012G,S32012K,S32018D,S32018G,S32018K<br>S32019D,S32019G,S32019K,S32020D,S32020G,S32020K,S32021D<br>S32021G,S32021K,S32022D,S32022G,S32022K,S32028D,S32028G<br>S32028K,S32029D,S32029G,S32029K,S32030D,S32030G,S32030K<br>S32031D,S32031G,S32031K,S32032D,S32032G,S32032K,S32038D<br>S32038G,S32038K,S32039D,S32039G,S32039K,S32040D,S32040G<br>S32040K,S32041D,S32041G,S32041K,S32042D,S32042G,S32042K<br>S32048D,S32048G,S32048K,S32049D,S32049G,S32049K,S32050D<br>S32050G,S32050K,S32051D,S32051G,S32051K,S32052D,S32052G<br>S32052K,S32058D,S32058G,S32058K,S32059D,S32059G,S32059K<br>S3210XD,S3210XG,S3210XK,S32110D,S32110G,S32110K,S32111D<br>S32111G,S32111K,S32112D,S32112G,S32112K,S32119D,S32119G<br>S32119K,S32120D,S32120G,S32120K,S32121D,S32121G,S32121K<br>S32122D,S32122G,S32122K,S32129D,S32129G,S32129K,S32130D<br>S32130G,S32130K,S32131D,S32131G,S32131K,S32132D,S32132G<br>S32132K,S32139D,S32139G,S32139K,S3214XD,S3214XG,S3214XK<br>S3215XD,S3215XG,S3215XK,S3216XD,S3216XG,S3216XK,S3217XD<br>S3217XG,S3217XK,S3219XD,S3219XG,S3219XK,S322XXD,S322XXG<br>S322XXK,S329XXD,S329XXG,S329XXK | 73382,V5417,V5419 |
| Fracture of the upper | M9731XA,M9732XA,M9741XA,M9742XA,S42001A,S42001B,S42002A | 81000,81001,81002,81003,81010,81011,<br>81012,81013,81100,81101 |

Continued on next page

Table S9 (continued).

| Diagnosis | ICD-10 | ICD-9 |
|---|---|---|
| limb, initial | S42002B,S42009A,S42009B,S42011A,S42011B,S42012A,S42012B | 81102,81103,81109,81110,81111,81112, 81113,81119,81200,81201 |
| encounter | S42013A,S42013B,S42014A,S42014B,S42015A,S42015B,S42016A | 81202,81203,81209,81210,81211,81212, 81213,81219,81220,81221 |
| | S42016B,S42017A,S42017B,S42018A,S42018B,S42019A,S42019B | 81230,81231,81240,81241,81242,81243, 81244,81249,81250,81251 |
| | S42021A,S42021B,S42022A,S42022B,S42023A,S42023B,S42024A | 81252,81253,81254,81259,81301,81302, 81303,81304,81305,81306 |
| | S42024B,S42025A,S42025B,S42026A,S42026B,S42031A,S42031B | 81307,81311,81312,81313,81314,81315, 81316,81317,81321,81322 |
| | S42032A,S42032B,S42033A,S42033B,S42034A,S42034B,S42035A | 81331,81332,81341,81342,81343,81345, 81346,81351,81352,81353 |
| | S42035B,S42036A,S42036B,S42101A,S42101B,S42102A,S42102B | 81380,81390,81400,81401,81402,81403, 81404,81405,81406,81407 |
| | S42109A,S42109B,S42111A,S42111B,S42112A,S42112B,S42113A | 81408,81409,81410,81411,81412,81413, 81414,81415,81416,81417 |
| | S42113B,S42114A,S42114B,S42115A,S42115B,S42116A,S42116B | 81418,81419,81500,81501,81502,81503, 81504,81509,81510,81511 |
| | S42121A,S42121B,S42122A,S42122B,S42123A,S42123B,S42124A | 81512,81513,81514,81519,81600,81601, 81602,81603,81610,81611 |
| | S42124B,S42125A,S42125B,S42126A,S42126B,S42131A,S42131B | 81612,81613,8170,8181 |
| | S42132A,S42132B,S42133A,S42133B,S42134A,S42134B,S42135A | |
| | S42135B,S42136A,S42136B,S42141A,S42141B,S42142A,S42142B | |
| | S42143A,S42143B,S42144A,S42144B,S42145A,S42145B,S42146A | |
| | S42146B,S42151A,S42151B,S42152A,S42152B,S42153A,S42153B | |
| | S42154A,S42154B,S42155A,S42155B,S42156A,S42156B,S42191A | |
| | S42191B,S42192A,S42192B,S42199A,S42199B,S42201A,S42201B | |
| | S42202A,S42202B,S42209A,S42209B,S42211A,S42211B,S42212A | |
| | S42212B,S42213A,S42213B,S42214A,S42214B,S42215A,S42215B | |
| | S42216A,S42216B,S42221A,S42221B,S42222A,S42222B,S42223A | |
| | S42223B,S42224A,S42224B,S42225A,S42225B,S42226A,S42226B | |
| | S42231A,S42231B,S42232A,S42232B,S42239A,S42239B,S42241A | |
| | S42241B,S42242A,S42242B,S42249A,S42249B,S42251A,S42251B | |
| | S42252A,S42252B,S42253A,S42253B,S42254A,S42254B,S42255A | |
| | S42255B,S42256A,S42256B,S42261A,S42261B,S42262A,S42262B | |
| | S42263A,S42263B,S42264A,S42264B,S42265A,S42265B,S42266A | |
| | S42266B,S42271A,S42272A,S42279A,S42291A,S42291B,S42292A | |
| | S42292B,S42293A,S42293B,S42294A,S42294B,S42295A,S42295B | |
| | S42296A,S42296B,S42301A,S42301B,S42302A,S42302B,S42309A | |
| | S42309B,S42311A,S42312A,S42319A,S42321A,S42321B,S42322A | |
| | S42322B,S42323A,S42323B,S42324A,S42324B,S42325A,S42325B | |
| | S42326A,S42326B,S42331A,S42331B,S42332A,S42332B,S42333A | |
| | S42333B,S42334A,S42334B,S42335A,S42335B,S42336A,S42336B | |
| | S42341A,S42341B,S42342A,S42342B,S42343A,S42343B,S42344A | |
| | S42344B,S42345A,S42345B,S42346A,S42346B,S42351A,S42351B | |
| | S42352A,S42352B,S42353A,S42353B,S42354A,S42354B,S42355A | |
| | S42355B,S42356A,S42356B,S42361A,S42361B,S42362A,S42362B | |
| | S42363A,S42363B,S42364A,S42364B,S42365A,S42365B,S42366A | |
| | S42366B,S42391A,S42391B,S42392A,S42392B,S42399A,S42399B | |
| | S42401A,S42401B,S42402A,S42402B,S42409A,S42409B,S42411A | |
| | S42411B,S42412A,S42412B,S42413A,S42413B,S42414A,S42414B | |
| | S42415A,S42415B,S42416A,S42416B,S42421A,S42421B,S42422A | |
| | S42422B,S42423A,S42423B,S42424A,S42424B,S42425A,S42425B | |
| | S42426A,S42426B,S42431A,S42431B,S42432A,S42432B,S42433A | |
| | S42433B,S42434A,S42434B,S42435A,S42435B,S42436A,S42436B | |
| | S42441A,S42441B,S42442A,S42442B,S42443A,S42443B,S42444A | |
| | S42444B,S42445A,S42445B,S42446A,S42446B,S42447A,S42447B | |
| | S42448A,S42448B,S42449A,S42449B,S42451A,S42451B,S42452A | |
| | S42452B,S42453A,S42453B,S42454A,S42454B,S42455A,S42455B | |
| | S42456A,S42456B,S42461A,S42461B,S42462A,S42462B,S42463A | |
| | S42463B,S42464A,S42464B,S42465A,S42465B,S42466A,S42466B | |
| | S42471A,S42471B,S42472A,S42472B,S42473A,S42473B,S42474A | |
| | S42474B,S42475A,S42475B,S42476A,S42476B,S42481A,S42482A | |
| | S42489A,S42491A,S42491B,S42492A,S42492B,S42493A,S42493B | |
| | S42494A,S42494B,S42495A,S42495B,S42496A,S42496B,S4290XA | |
| | S4290XB,S4291XA,S4291XB,S4292XA,S4292XB,S49001A,S49002A | |
| | S49009A,S49011A,S49012A,S49019A,S49021A,S49022A,S49029A | |
| | S49031A,S49032A,S49039A,S49041A,S49042A,S49049A,S49091A | |
| | S49092A,S49099A,S49101A,S49102A,S49109A,S49111A,S49112A | |
| | S49119A,S49121A,S49122A,S49129A,S49131A,S49132A,S49139A | |
| | S49141A,S49142A,S49149A,S49191A,S49192A,S49199A,S52001A | |
| | S52001B,S52001C,S52002A,S52002B,S52002C,S52009A,S52009B | |
| | S52009C,S52011A,S52012A,S52019A,S52021A,S52021B,S52021C | |
| | S52022A,S52022B,S52022C,S52023A,S52023B,S52023C,S52024A | |
| | S52024B,S52024C,S52025A,S52025B,S52025C,S52026A,S52026B | |
| | S52026C,S52031A,S52031B,S52031C,S52032A,S52032B,S52032C | |
| | S52033A,S52033B,S52033C,S52034A,S52034B,S52034C,S52035A | |
| | S52035B,S52035C,S52036A,S52036B,S52036C,S52041A,S52041B | |
| | S52041C,S52042A,S52042B,S52042C,S52043A,S52043B,S52043C | |
| | S52044A,S52044B,S52044C,S52045A,S52045B,S52045C,S52046A | |
| | S52046B,S52046C,S52091A,S52091B,S52091C,S52092A,S52092B | |
| | S52092C,S52099A,S52099B,S52099C,S52101A,S52101B,S52101C | |
| | S52102A,S52102B,S52102C,S52109A,S52109B,S52109C,S52111A | |
| | S52112A,S52119A,S52121A,S52121B,S52121C,S52122A,S52122B | |
| | S52122C,S52123A,S52123B,S52123C,S52124A,S52124B,S52124C | |
| | S52125A,S52125B,S52125C,S52126A,S52126B,S52126C,S52131A | |

Continued on next page

Table S9 (continued).

| Diagnosis | ICD-10 | ICD-9 |
|---|---|---|
| | S52131B,S52131C,S52132A,S52132B,S52132C,S52133A,S52133B | |
| | S52133C,S52134A,S52134B,S52134C,S52135A,S52135B,S52135C | |
| | S52136A,S52136B,S52136C,S52181A,S52181B,S52181C,S52182A | |
| | S52182B,S52182C,S52189A,S52189B,S52189C,S52201A,S52201B | |
| | S52201C,S52202A,S52202B,S52202C,S52209A,S52209B,S52209C | |
| | S52211A,S52212A,S52219A,S52221A,S52221B,S52221C,S52222A | |
| | S52222B,S52222C,S52223A,S52223B,S52223C,S52224A,S52224B | |
| | S52224C,S52225A,S52225B,S52225C,S52226A,S52226B,S52226C | |
| | S52231A,S52231B,S52231C,S52232A,S52232B,S52232C,S52233A | |
| | S52233B,S52233C,S52234A,S52234B,S52234C,S52235A,S52235B | |
| | S52235C,S52236A,S52236B,S52236C,S52241A,S52241B,S52241C | |
| | S52242A,S52242B,S52242C,S52243A,S52243B,S52243C,S52244A | |
| | S52244B,S52244C,S52245A,S52245B,S52245C,S52246A,S52246B | |
| | S52246C,S52251A,S52251B,S52251C,S52252A,S52252B,S52252C | |
| | S52253A,S52253B,S52253C,S52254A,S52254B,S52254C,S52255A | |
| | S52255B,S52255C,S52256A,S52256B,S52256C,S52261A,S52261B | |
| | S52261C,S52262A,S52262B,S52262C,S52263A,S52263B,S52263C | |
| | S52264A,S52264B,S52264C,S52265A,S52265B,S52265C,S52266A | |
| | S52266B,S52266C,S52271A,S52271B,S52271C,S52272A,S52272B | |
| | S52272C,S52279A,S52279B,S52279C,S52281A,S52281B,S52281C | |
| | S52282A,S52282B,S52282C,S52283A,S52283B,S52283C,S52291A | |
| | S52291B,S52291C,S52292A,S52292B,S52292C,S52299A,S52299B | |
| | S52299C,S52301A,S52301B,S52301C,S52302A,S52302B,S52302C | |
| | S52309A,S52309B,S52309C,S52311A,S52312A,S52319A,S52321A | |
| | S52321B,S52321C,S52322A,S52322B,S52322C,S52323A,S52323B | |
| | S52323C,S52324A,S52324B,S52324C,S52325A,S52325B,S52325C | |
| | S52326A,S52326B,S52326C,S52331A,S52331B,S52331C,S52332A | |
| | S52332B,S52332C,S52333A,S52333B,S52333C,S52334A,S52334B | |
| | S52334C,S52335A,S52335B,S52335C,S52336A,S52336B,S52336C | |
| | S52341A,S52341B,S52341C,S52342A,S52342B,S52342C,S52343A | |
| | S52343B,S52343C,S52344A,S52344B,S52344C,S52345A,S52345B | |
| | S52345C,S52346A,S52346B,S52346C,S52351A,S52351B,S52351C | |
| | S52352A,S52352B,S52352C,S52353A,S52353B,S52353C,S52354A | |
| | S52354B,S52354C,S52355A,S52355B,S52355C,S52356A,S52356B | |
| | S52356C,S52361A,S52361B,S52361C,S52362A,S52362B,S52362C | |
| | S52363A,S52363B,S52363C,S52364A,S52364B,S52364C,S52365A | |
| | S52365B,S52365C,S52366A,S52366B,S52366C,S52371A,S52371B | |
| | S52371C,S52372A,S52372B,S52372C,S52379A,S52379B,S52379C | |
| | S52381A,S52381B,S52381C,S52382A,S52382B,S52382C,S52389A | |
| | S52389B,S52389C,S52391A,S52391B,S52391C,S52392A,S52392B | |
| | S52392C,S52399A,S52399B,S52399C,S52501A,S52501B,S52501C | |
| | S52502A,S52502B,S52502C,S52509A,S52509B,S52509C,S52511A | |
| | S52511B,S52511C,S52512A,S52512B,S52512C,S52513A,S52513B | |
| | S52513C,S52514A,S52514B,S52514C,S52515A,S52515B,S52515C | |
| | S52516A,S52516B,S52516C,S52521A,S52522A,S52529A,S52531A | |
| | S52531B,S52531C,S52532A,S52532B,S52532C,S52539A,S52539B | |
| | S52539C,S52541A,S52541B,S52541C,S52542A,S52542B,S52542C | |
| | S52549A,S52549B,S52549C,S52551A,S52551B,S52551C,S52552A | |
| | S52552B,S52552C,S52559A,S52559B,S52559C,S52561A,S52561B | |
| | S52561C,S52562A,S52562B,S52562C,S52569A,S52569B,S52569C | |
| | S52571A,S52571B,S52571C,S52572A,S52572B,S52572C,S52579A | |
| | S52579B,S52579C,S52591A,S52591B,S52591C,S52592A,S52592B | |
| | S52592C,S52599A,S52599B,S52599C,S52601A,S52601B,S52601C | |
| | S52602A,S52602B,S52602C,S52609A,S52609B,S52609C,S52611A | |
| | S52611B,S52611C,S52612A,S52612B,S52612C,S52613A,S52613B | |
| | S52613C,S52614A,S52614B,S52614C,S52615A,S52615B,S52615C | |
| | S52616A,S52616B,S52616C,S52621A,S52622A,S52629A,S52691A | |
| | S52691B,S52691C,S52692A,S52692B,S52692C,S52699A,S52699B | |
| | S52699C,S5290XA,S5290XB,S5290XC,S5291XA,S5291XB,S5291XC | |
| | S5292XA,S5292XB,S5292XC,S59001A,S59002A,S59009A,S59011A | |
| | S59012A,S59019A,S59021A,S59022A,S59029A,S59031A,S59032A | |
| | S59039A,S59041A,S59042A,S59049A,S59091A,S59092A,S59099A | |
| | S59101A,S59102A,S59109A,S59111A,S59112A,S59119A,S59121A | |
| | S59122A,S59129A,S59131A,S59132A,S59139A,S59141A,S59142A | |
| | S59149A,S59191A,S59192A,S59199A,S59201A,S59202A,S59209A | |
| | S59211A,S59212A,S59219A,S59221A,S59222A,S59229A,S59231A | |
| | S59232A,S59239A,S59241A,S59242A,S59249A,S59291A,S59292A | |
| | S59299A,S62001A,S62001B,S62002A,S62002B,S62009A,S62009B | |
| | S62011A,S62011B,S62012A,S62012B,S62013A,S62013B,S62014A | |
| | S62014B,S62015A,S62015B,S62016A,S62016B,S62021A,S62021B | |
| | S62022A,S62022B,S62023A,S62023B,S62024A,S62024B,S62025A | |
| | S62025B,S62026A,S62026B,S62031A,S62031B,S62032A,S62032B | |
| | S62033A,S62033B,S62034A,S62034B,S62035A,S62035B,S62036A | |
| | S62036B,S62101A,S62101B,S62102A,S62102B,S62109A,S62109B | |
| | S62111A,S62111B,S62112A,S62112B,S62113A,S62113B,S62114A | |
| | S62114B,S62115A,S62115B,S62116A,S62116B,S62121A,S62121B | |
| | S62122A,S62122B,S62123A,S62123B,S62124A,S62124B,S62125A | |
| | S62125B,S62126A,S62126B,S62131A,S62131B,S62132A,S62132B | |
| | S62133A,S62133B,S62134A,S62134B,S62135A,S62135B,S62136A | |
| | S62136B,S62141A,S62141B,S62142A,S62142B,S62143A,S62143B | |
| | S62144A,S62144B,S62145A,S62145B,S62146A,S62146B,S62151A | |
| | S62151B,S62152A,S62152B,S62153A,S62153B,S62154A,S62154B | |
| | S62155A,S62155B,S62156A,S62156B,S62161A,S62161B,S62162A | |
| | S62162B,S62163A,S62163B,S62164A,S62164B,S62165A,S62165B | |
| | S62166A,S62166B,S62171A,S62171B,S62172A,S62172B,S62173A | |

Continued on next page

Table S9 (continued).

| Diagnosis | ICD-10 | ICD-9 |
|---|---|---|
| | S62173B,S62174A,S62174B,S62175A,S62175B,S62176A,S62176B<br>S62181A,S62181B,S62182A,S62182B,S62183A,S62183B,S62184A<br>S62184B,S62185A,S62185B,S62186A,S62186B,S62201A,S62201B<br>S62202A,S62202B,S62209A,S62209B,S62211A,S62211B,S62212A<br>S62212B,S62213A,S62213B,S62221A,S62221B,S62222A,S62222B<br>S62223A,S62223B,S62224A,S62224B,S62225A,S62225B,S62226A<br>S62226B,S62231A,S62231B,S62232A,S62232B,S62233A,S62233B<br>S62234A,S62234B,S62235A,S62235B,S62236A,S62236B,S62241A<br>S62241B,S62242A,S62242B,S62243A,S62243B,S62244A,S62244B<br>S62245A,S62245B,S62246A,S62246B,S62251A,S62251B,S62252A<br>S62252B,S62253A,S62253B,S62254A,S62254B,S62255A,S62255B<br>S62256A,S62256B,S62291A,S62291B,S62292A,S62292B,S62299A<br>S62299B,S62300A,S62300B,S62301A,S62301B,S62302A,S62302B<br>S62303A,S62303B,S62304A,S62304B,S62305A,S62305B,S62306A<br>S62306B,S62307A,S62307B,S62308A,S62308B,S62309A,S62309B<br>S62310A,S62310B,S62311A,S62311B,S62312A,S62312B,S62313A<br>S62313B,S62314A,S62314B,S62315A,S62315B,S62316A,S62316B<br>S62317A,S62317B,S62318A,S62318B,S62319A,S62319B,S62320A<br>S62320B,S62321A,S62321B,S62322A,S62322B,S62323A,S62323B<br>S62324A,S62324B,S62325A,S62325B,S62326A,S62326B,S62327A<br>S62327B,S62328A,S62328B,S62329A,S62329B,S62330A,S62330B<br>S62331A,S62331B,S62332A,S62332B,S62333A,S62333B,S62334A<br>S62334B,S62335A,S62335B,S62336A,S62336B,S62337A,S62337B<br>S62338A,S62338B,S62339A,S62339B,S62340A,S62340B,S62341A<br>S62341B,S62342A,S62342B,S62343A,S62343B,S62344A,S62344B<br>S62345A,S62345B,S62346A,S62346B,S62347A,S62347B,S62348A<br>S62348B,S62349A,S62349B,S62350A,S62350B,S62351A,S62351B<br>S62352A,S62352B,S62353A,S62353B,S62354A,S62354B,S62355A<br>S62355B,S62356A,S62356B,S62357A,S62357B,S62358A,S62358B<br>S62359A,S62359B,S62360A,S62360B,S62361A,S62361B,S62362A<br>S62362B,S62363A,S62363B,S62364A,S62364B,S62365A,S62365B<br>S62366A,S62366B,S62367A,S62367B,S62368A,S62368B,S62369A<br>S62369B,S62390A,S62390B,S62391A,S62391B,S62392A,S62392B<br>S62393A,S62393B,S62394A,S62394B,S62395A,S62395B,S62396A<br>S62396B,S62397A,S62397B,S62398A,S62398B,S62399A,S62399B<br>S62501A,S62501B,S62502A,S62502B,S62509A,S62509B,S62511A<br>S62511B,S62512A,S62512B,S62513A,S62513B,S62514A,S62514B<br>S62515A,S62515B,S62516A,S62516B,S62521A,S62521B,S62522A<br>S62522B,S62523A,S62523B,S62524A,S62524B,S62525A,S62525B<br>S62526A,S62526B,S62600A,S62600B,S62601A,S62601B,S62602A<br>S62602B,S62603A,S62603B,S62604A,S62604B,S62605A,S62605B<br>S62606A,S62606B,S62607A,S62607B,S62608A,S62608B,S62609A<br>S62609B,S62610A,S62610B,S62611A,S62611B,S62612A,S62612B<br>S62613A,S62613B,S62614A,S62614B,S62615A,S62615B,S62616A<br>S62616B,S62617A,S62617B,S62618A,S62618B,S62619A,S62619B<br>S62620A,S62620B,S62621A,S62621B,S62622A,S62622B,S62623A<br>S62623B,S62624A,S62624B,S62625A,S62625B,S62626A,S62626B<br>S62627A,S62627B,S62628A,S62628B,S62629A,S62629B,S62630A<br>S62630B,S62631A,S62631B,S62632A,S62632B,S62633A,S62633B<br>S62634A,S62634B,S62635A,S62635B,S62636A,S62636B,S62637A<br>S62637B,S62638A,S62638B,S62639A,S62639B,S62640A,S62640B<br>S62641A,S62641B,S62642A,S62642B,S62643A,S62643B,S62644A<br>S62644B,S62645A,S62645B,S62646A,S62646B,S62647A,S62647B<br>S62648A,S62648B,S62649A,S62649B,S62650A,S62650B,S62651A<br>S62651B,S62652A,S62652B,S62653A,S62653B,S62654A,S62654B<br>S62655A,S62655B,S62656A,S62656B,S62657A,S62657B,S62658A<br>S62658B,S62659A,S62659B,S62660A,S62660B,S62661A,S62661B<br>S62662A,S62662B,S62663A,S62663B,S62664A,S62664B,S62665A<br>S62665B,S62666A,S62666B,S62667A,S62667B,S62668A,S62668B<br>S62669A,S62669B,S6290XA,S6290XB,S6291XA,S6291XB,S6292XA<br>S6292XB | |
| Fracture of the upper limb, subsequent encounter | M9731XD,M9732XD,M9741XD,M9742XD,S42001D,S42001G,S42001K<br>S42001P,S42002D,S42002G,S42002K,S42002P,S42009D,S42009G<br>S42009K,S42009P,S42011D,S42011G,S42011K,S42011P,S42012D<br>S42012G,S42012K,S42012P,S42013D,S42013G,S42013K,S42013P<br>S42014D,S42014G,S42014K,S42014P,S42015D,S42015G,S42015K<br>S42015P,S42016D,S42016G,S42016K,S42016P,S42017D,S42017G<br>S42017K,S42017P,S42018D,S42018G,S42018K,S42018P,S42019D<br>S42019G,S42019K,S42019P,S42021D,S42021G,S42021K,S42021P<br>S42022D,S42022G,S42022K,S42022P,S42023D,S42023G,S42023K<br>S42023P,S42024D,S42024G,S42024K,S42024P,S42025D,S42025G<br>S42025K,S42025P,S42026D,S42026G,S42026K,S42026P,S42031D<br>S42031G,S42031K,S42031P,S42032D,S42032G,S42032K,S42032P<br>S42033D,S42033G,S42033K,S42033P,S42034D,S42034G,S42034K<br>S42034P,S42035D,S42035G,S42035K,S42035P,S42036D,S42036G<br>S42036K,S42036P,S42101D,S42101G,S42101K,S42101P,S42102D<br>S42102G,S42102K,S42102P,S42109D,S42109G,S42109K,S42109P<br>S42111D,S42111G,S42111K,S42111P,S42112D,S42112G,S42112K<br>S42112P,S42113D,S42113G,S42113K,S42113P,S42114D,S42114G<br>S42114K,S42114P,S42115D,S42115G,S42115K,S42115P,S42116D<br>S42116G,S42116K,S42116P,S42121D,S42121G,S42121K,S42121P<br>S42122D,S42122G,S42122K,S42122P,S42123D,S42123G,S42123K<br>S42123P,S42124D,S42124G,S42124K,S42124P,S42125D,S42125G<br>S42125K,S42125P,S42126D,S42126G,S42126K,S42126P,S42131D | 73381,73382,V5410,V5411,V5412,V5419 |

Continued on next page

Table S9 (continued).

| Diagnosis | ICD-10 | ICD-9 |
|---|---|---|
| | S42131G,S42131K,S42131P,S42132D,S42132G,S42132K,S42132P | |
| | S42133D,S42133G,S42133K,S42133P,S42134D,S42134G,S42134K | |
| | S42134P,S42135D,S42135G,S42135K,S42135P,S42136D,S42136G | |
| | S42136K,S42136P,S42141D,S42141G,S42141K,S42141P,S42142D | |
| | S42142G,S42142K,S42142P,S42143D,S42143G,S42143K,S42143P | |
| | S42144D,S42144G,S42144K,S42144P,S42145D,S42145G,S42145K | |
| | S42145P,S42146D,S42146G,S42146K,S42146P,S42151D,S42151G | |
| | S42151K,S42151P,S42152D,S42152G,S42152K,S42152P,S42153D | |
| | S42153G,S42153K,S42153P,S42154D,S42154G,S42154K,S42154P | |
| | S42155D,S42155G,S42155K,S42155P,S42156D,S42156G,S42156K | |
| | S42156P,S42191D,S42191G,S42191K,S42191P,S42192D,S42192G | |
| | S42192K,S42192P,S42199D,S42199G,S42199K,S42199P,S42201D | |
| | S42201G,S42201K,S42201P,S42202D,S42202G,S42202K,S42202P | |
| | S42209D,S42209G,S42209K,S42209P,S42211D,S42211G,S42211K | |
| | S42211P,S42212D,S42212G,S42212K,S42212P,S42213D,S42213G | |
| | S42213K,S42213P,S42214D,S42214G,S42214K,S42214P,S42215D | |
| | S42215G,S42215K,S42215P,S42216D,S42216G,S42216K,S42216P | |
| | S42221D,S42221G,S42221K,S42221P,S42222D,S42222G,S42222K | |
| | S42222P,S42223D,S42223G,S42223K,S42223P,S42224D,S42224G | |
| | S42224K,S42224P,S42225D,S42225G,S42225K,S42225P,S42226D | |
| | S42226G,S42226K,S42226P,S42231D,S42231G,S42231K,S42231P | |
| | S42232D,S42232G,S42232K,S42232P,S42239D,S42239G,S42239K | |
| | S42239P,S42241D,S42241G,S42241K,S42241P,S42242D,S42242G | |
| | S42242K,S42242P,S42249D,S42249G,S42249K,S42249P,S42251D | |
| | S42251G,S42251K,S42251P,S42252D,S42252G,S42252K,S42252P | |
| | S42253D,S42253G,S42253K,S42253P,S42254D,S42254G,S42254K | |
| | S42254P,S42255D,S42255G,S42255K,S42255P,S42256D,S42256G | |
| | S42256K,S42256P,S42261D,S42261G,S42261K,S42261P,S42262D | |
| | S42262G,S42262K,S42262P,S42263D,S42263G,S42263K,S42263P | |
| | S42264D,S42264G,S42264K,S42264P,S42265D,S42265G,S42265K | |
| | S42265P,S42266D,S42266G,S42266K,S42266P,S42271D,S42271G | |
| | S42271K,S42271P,S42272D,S42272G,S42272K,S42272P,S42279D | |
| | S42279G,S42279K,S42279P,S42291D,S42291G,S42291K,S42291P | |
| | S42292D,S42292G,S42292K,S42292P,S42293D,S42293G,S42293K | |
| | S42293P,S42294D,S42294G,S42294K,S42294P,S42295D,S42295G | |
| | S42295K,S42295P,S42296D,S42296G,S42296K,S42296P,S42301D | |
| | S42301G,S42301K,S42301P,S42302D,S42302G,S42302K,S42302P | |
| | S42309D,S42309G,S42309K,S42309P,S42311D,S42311G,S42311K | |
| | S42311P,S42312D,S42312G,S42312K,S42312P,S42319D,S42319G | |
| | S42319K,S42319P,S42321D,S42321G,S42321K,S42321P,S42322D | |
| | S42322G,S42322K,S42322P,S42323D,S42323G,S42323K,S42323P | |
| | S42324D,S42324G,S42324K,S42324P,S42325D,S42325G,S42325K | |
| | S42325P,S42326D,S42326G,S42326K,S42326P,S42331D,S42331G | |
| | S42331K,S42331P,S42332D,S42332G,S42332K,S42332P,S42333D | |
| | S42333G,S42333K,S42333P,S42334D,S42334G,S42334K,S42334P | |
| | S42335D,S42335G,S42335K,S42335P,S42336D,S42336G,S42336K | |
| | S42336P,S42341D,S42341G,S42341K,S42341P,S42342D,S42342G | |
| | S42342K,S42342P,S42343D,S42343G,S42343K,S42343P,S42344D | |
| | S42344G,S42344K,S42344P,S42345D,S42345G,S42345K,S42345P | |
| | S42346D,S42346G,S42346K,S42346P,S42351D,S42351G,S42351K | |
| | S42351P,S42352D,S42352G,S42352K,S42352P,S42353D,S42353G | |
| | S42353K,S42353P,S42354D,S42354G,S42354K,S42354P,S42355D | |
| | S42355G,S42355K,S42355P,S42356D,S42356G,S42356K,S42356P | |
| | S42361D,S42361G,S42361K,S42361P,S42362D,S42362G,S42362K | |
| | S42362P,S42363D,S42363G,S42363K,S42363P,S42364D,S42364G | |
| | S42364K,S42364P,S42365D,S42365G,S42365K,S42365P,S42366D | |
| | S42366G,S42366K,S42366P,S42391D,S42391G,S42391K,S42391P | |
| | S42392D,S42392G,S42392K,S42392P,S42399D,S42399G,S42399K | |
| | S42399P,S42401D,S42401G,S42401K,S42401P,S42402D,S42402G | |
| | S42402K,S42402P,S42409D,S42409G,S42409K,S42409P,S42411D | |
| | S42411G,S42411K,S42411P,S42412D,S42412G,S42412K,S42412P | |
| | S42413D,S42413G,S42413K,S42413P,S42414D,S42414G,S42414K | |
| | S42414P,S42415D,S42415G,S42415K,S42415P,S42416D,S42416G | |
| | S42416K,S42416P,S42421D,S42421G,S42421K,S42421P,S42422D | |
| | S42422G,S42422K,S42422P,S42423D,S42423G,S42423K,S42423P | |
| | S42424D,S42424G,S42424K,S42424P,S42425D,S42425G,S42425K | |
| | S42425P,S42426D,S42426G,S42426K,S42426P,S42431D,S42431G | |
| | S42431K,S42431P,S42432D,S42432G,S42432K,S42432P,S42433D | |
| | S42433G,S42433K,S42433P,S42434D,S42434G,S42434K,S42434P | |
| | S42435D,S42435G,S42435K,S42435P,S42436D,S42436G,S42436K | |
| | S42436P,S42441D,S42441G,S42441K,S42441P,S42442D,S42442G | |
| | S42442K,S42442P,S42443D,S42443G,S42443K,S42443P,S42444D | |
| | S42444G,S42444K,S42444P,S42445D,S42445G,S42445K,S42445P | |
| | S42446D,S42446G,S42446K,S42446P,S42447D,S42447G,S42447K | |
| | S42447P,S42448D,S42448G,S42448K,S42448P,S42449D,S42449G | |
| | S42449K,S42449P,S42451D,S42451G,S42451K,S42451P,S42452D | |
| | S42452G,S42452K,S42452P,S42453D,S42453G,S42453K,S42453P | |
| | S42454D,S42454G,S42454K,S42454P,S42455D,S42455G,S42455K | |
| | S42455P,S42456D,S42456G,S42456K,S42456P,S42461D,S42461G | |
| | S42461K,S42461P,S42462D,S42462G,S42462K,S42462P,S42463D | |
| | S42463G,S42463K,S42463P,S42464D,S42464G,S42464K,S42464P | |
| | S42465D,S42465G,S42465K,S42465P,S42466D,S42466G,S42466K | |
| | S42466P,S42471D,S42471G,S42471K,S42471P,S42472D,S42472G | |
| | S42472K,S42472P,S42473D,S42473G,S42473K,S42473P,S42474D | |
| | S42474G,S42474K,S42474P,S42475D,S42475G,S42475K,S42475P | |

Continued on next page

Table S9 (continued).

| Diagnosis | ICD-10 | ICD-9 |
|---|---|---|
| | S42476D,S42476G,S42476K,S42476P,S42481D,S42481G,S42481K | |
| | S42481P,S42482D,S42482G,S42482K,S42482P,S42489D,S42489G | |
| | S42489K,S42489P,S42491D,S42491G,S42491K,S42491P,S42492D | |
| | S42492G,S42492K,S42492P,S42493D,S42493G,S42493K,S42493P | |
| | S42494D,S42494G,S42494K,S42494P,S42495D,S42495G,S42495K | |
| | S42495P,S42496D,S42496G,S42496K,S42496P,S4290XD,S4290XG | |
| | S4290XK,S4290XP,S4291XD,S4291XG,S4291XK,S4291XP,S4292XD | |
| | S4292XG,S4292XK,S4292XP,S49001D,S49001G,S49001K,S49001P | |
| | S49002D,S49002G,S49002K,S49002P,S49009D,S49009G,S49009K | |
| | S49009P,S49011D,S49011G,S49011K,S49011P,S49012D,S49012G | |
| | S49012K,S49012P,S49019D,S49019G,S49019K,S49019P,S49021D | |
| | S49021G,S49021K,S49021P,S49022D,S49022G,S49022K,S49022P | |
| | S49029D,S49029G,S49029K,S49029P,S49031D,S49031G,S49031K | |
| | S49031P,S49032D,S49032G,S49032K,S49032P,S49039D,S49039G | |
| | S49039K,S49039P,S49041D,S49041G,S49041K,S49041P,S49042D | |
| | S49042G,S49042K,S49042P,S49049D,S49049G,S49049K,S49049P | |
| | S49091D,S49091G,S49091K,S49091P,S49092D,S49092G,S49092K | |
| | S49092P,S49099D,S49099G,S49099K,S49099P,S49101D,S49101G | |
| | S49101K,S49101P,S49102D,S49102G,S49102K,S49102P,S49109D | |
| | S49109G,S49109K,S49109P,S49111D,S49111G,S49111K,S49111P | |
| | S49112D,S49112G,S49112K,S49112P,S49119D,S49119G,S49119K | |
| | S49119P,S49121D,S49121G,S49121K,S49121P,S49122D,S49122G | |
| | S49122K,S49122P,S49129D,S49129G,S49129K,S49129P,S49131D | |
| | S49131G,S49131K,S49131P,S49132D,S49132G,S49132K,S49132P | |
| | S49139D,S49139G,S49139K,S49139P,S49141D,S49141G,S49141K | |
| | S49141P,S49142D,S49142G,S49142K,S49142P,S49149D,S49149G | |
| | S49149K,S49149P,S49191D,S49191G,S49191K,S49191P,S49192D | |
| | S49192G,S49192K,S49192P,S49199D,S49199G,S49199K,S49199P | |
| | S52001D,S52001E,S52001F,S52001G,S52001H,S52001J,S52001K | |
| | S52001M,S52001N,S52001P,S52001Q,S52001R,S52002D,S52002E | |
| | S52002F,S52002G,S52002H,S52002J,S52002K,S52002M,S52002N | |
| | S52002P,S52002Q,S52002R,S52009D,S52009E,S52009F,S52009G | |
| | S52009H,S52009J,S52009K,S52009M,S52009N,S52009P,S52009Q | |
| | S52009R,S52011D,S52011G,S52011K,S52011P,S52012D,S52012G | |
| | S52012K,S52012P,S52019D,S52019G,S52019K,S52019P,S52021D | |
| | S52021E,S52021F,S52021G,S52021H,S52021J,S52021K,S52021M | |
| | S52021N,S52021P,S52021Q,S52021R,S52022D,S52022E,S52022F | |
| | S52022G,S52022H,S52022J,S52022K,S52022M,S52022N,S52022P | |
| | S52022Q,S52022R,S52023D,S52023E,S52023F,S52023G,S52023H | |
| | S52023J,S52023K,S52023M,S52023N,S52023P,S52023Q,S52023R | |
| | S52024D,S52024E,S52024F,S52024G,S52024H,S52024J,S52024K | |
| | S52024M,S52024N,S52024P,S52024Q,S52024R,S52025D,S52025E | |
| | S52025F,S52025G,S52025H,S52025J,S52025K,S52025M,S52025N | |
| | S52025P,S52025Q,S52025R,S52026D,S52026E,S52026F,S52026G | |
| | S52026H,S52026J,S52026K,S52026M,S52026N,S52026P,S52026Q | |
| | S52026R,S52031D,S52031E,S52031F,S52031G,S52031H,S52031J | |
| | S52031K,S52031M,S52031N,S52031P,S52031Q,S52031R,S52032D | |
| | S52032E,S52032F,S52032G,S52032H,S52032J,S52032K,S52032M | |
| | S52032N,S52032P,S52032Q,S52032R,S52033D,S52033E,S52033F | |
| | S52033G,S52033H,S52033J,S52033K,S52033M,S52033N,S52033P | |
| | S52033Q,S52033R,S52034D,S52034E,S52034F,S52034G,S52034H | |
| | S52034J,S52034K,S52034M,S52034N,S52034P,S52034Q,S52034R | |
| | S52035D,S52035E,S52035F,S52035G,S52035H,S52035J,S52035K | |
| | S52035M,S52035N,S52035P,S52035Q,S52035R,S52036D,S52036E | |
| | S52036F,S52036G,S52036H,S52036J,S52036K,S52036M,S52036N | |
| | S52036P,S52036Q,S52036R,S52041D,S52041E,S52041F,S52041G | |
| | S52041H,S52041J,S52041K,S52041M,S52041N,S52041P,S52041Q | |
| | S52041R,S52042D,S52042E,S52042F,S52042G,S52042H,S52042J | |
| | S52042K,S52042M,S52042N,S52042P,S52042Q,S52042R,S52043D | |
| | S52043E,S52043F,S52043G,S52043H,S52043J,S52043K,S52043M | |
| | S52043N,S52043P,S52043Q,S52043R,S52044D,S52044E,S52044F | |
| | S52044G,S52044H,S52044J,S52044K,S52044M,S52044N,S52044P | |
| | S52044Q,S52044R,S52045D,S52045E,S52045F,S52045G,S52045H | |
| | S52045J,S52045K,S52045M,S52045N,S52045P,S52045Q,S52045R | |
| | S52046D,S52046E,S52046F,S52046G,S52046H,S52046J,S52046K | |
| | S52046M,S52046N,S52046P,S52046Q,S52046R,S52091D,S52091E | |
| | S52091F,S52091G,S52091H,S52091J,S52091K,S52091M,S52091N | |
| | S52091P,S52091Q,S52091R,S52092D,S52092E,S52092F,S52092G | |
| | S52092H,S52092J,S52092K,S52092M,S52092N,S52092P,S52092Q | |
| | S52092R,S52099D,S52099E,S52099F,S52099G,S52099H,S52099J | |
| | S52099K,S52099M,S52099N,S52099P,S52099Q,S52099R,S52101D | |
| | S52101E,S52101F,S52101G,S52101H,S52101J,S52101K,S52101M | |
| | S52101N,S52101P,S52101Q,S52101R,S52102D,S52102E,S52102F | |
| | S52102G,S52102H,S52102J,S52102K,S52102M,S52102N,S52102P | |
| | S52102Q,S52102R,S52109D,S52109E,S52109F,S52109G,S52109H | |
| | S52109J,S52109K,S52109M,S52109N,S52109P,S52109Q,S52109R | |
| | S52111D,S52111G,S52111K,S52111P,S52112D,S52112G,S52112K | |
| | S52112P,S52119D,S52119G,S52119K,S52119P,S52121D,S52121E | |
| | S52121F,S52121G,S52121H,S52121J,S52121K,S52121M,S52121N | |
| | S52121P,S52121Q,S52121R,S52122D,S52122E,S52122F,S52122G | |
| | S52122H,S52122J,S52122K,S52122M,S52122N,S52122P,S52122Q | |
| | S52122R,S52123D,S52123E,S52123F,S52123G,S52123H,S52123J | |
| | S52123K,S52123M,S52123N,S52123P,S52123Q,S52123R,S52124D | |
| | S52124E,S52124F,S52124G,S52124H,S52124J,S52124K,S52124M | |
| | S52124N,S52124P,S52124Q,S52124R,S52125D,S52125E,S52125F | |

Continued on next page

Table S9 (continued).

| Diagnosis | ICD-10 | ICD-9 |
|---|---|---|
| | S52125G,S52125H,S52125J,S52125K,S52125M,S52125N,S52125P<br>S52125Q,S52125R,S52126D,S52126E,S52126F,S52126G,S52126H<br>S52126J,S52126K,S52126M,S52126N,S52126P,S52126Q,S52126R<br>S52131D,S52131E,S52131F,S52131G,S52131H,S52131J,S52131K<br>S52131M,S52131N,S52131P,S52131Q,S52131R,S52132D,S52132E<br>S52132F,S52132G,S52132H,S52132J,S52132K,S52132M,S52132N<br>S52132P,S52132Q,S52132R,S52133D,S52133E,S52133F,S52133G<br>S52133H,S52133J,S52133K,S52133M,S52133N,S52133P,S52133Q<br>S52133R,S52134D,S52134E,S52134F,S52134G,S52134H,S52134J<br>S52134K,S52134M,S52134N,S52134P,S52134Q,S52134R,S52135D<br>S52135E,S52135F,S52135G,S52135H,S52135J,S52135K,S52135M<br>S52135N,S52135P,S52135Q,S52135R,S52136D,S52136E,S52136F<br>S52136G,S52136H,S52136J,S52136K,S52136M,S52136N,S52136P<br>S52136Q,S52136R,S52181D,S52181E,S52181F,S52181G,S52181H<br>S52181J,S52181K,S52181M,S52181N,S52181P,S52181Q,S52181R<br>S52182D,S52182E,S52182F,S52182G,S52182H,S52182J,S52182K<br>S52182M,S52182N,S52182P,S52182Q,S52182R,S52189D,S52189E<br>S52189F,S52189G,S52189H,S52189J,S52189K,S52189M,S52189N<br>S52189P,S52189Q,S52189R,S52201D,S52201E,S52201F,S52201G<br>S52201H,S52201J,S52201K,S52201M,S52201N,S52201P,S52201Q<br>S52201R,S52202D,S52202E,S52202F,S52202G,S52202H,S52202J<br>S52202K,S52202M,S52202N,S52202P,S52202Q,S52202R,S52209D<br>S52209E,S52209F,S52209G,S52209H,S52209J,S52209K,S52209M<br>S52209N,S52209P,S52209Q,S52209R,S52211D,S52211G,S52211K<br>S52211P,S52212D,S52212G,S52212K,S52212P,S52219D,S52219G<br>S52219K,S52219P,S52221D,S52221E,S52221F,S52221G,S52221H<br>S52221J,S52221K,S52221M,S52221N,S52221P,S52221Q,S52221R<br>S52222D,S52222E,S52222F,S52222G,S52222H,S52222J,S52222K<br>S52222M,S52222N,S52222P,S52222Q,S52222R,S52223D,S52223E<br>S52223F,S52223G,S52223H,S52223J,S52223K,S52223M,S52223N<br>S52223P,S52223Q,S52223R,S52224D,S52224E,S52224F,S52224G<br>S52224H,S52224J,S52224K,S52224M,S52224N,S52224P,S52224Q<br>S52224R,S52225D,S52225E,S52225F,S52225G,S52225H,S52225J<br>S52225K,S52225M,S52225N,S52225P,S52225Q,S52225R,S52226D<br>S52226E,S52226F,S52226G,S52226H,S52226J,S52226K,S52226M<br>S52226N,S52226P,S52226Q,S52226R,S52231D,S52231E,S52231F<br>S52231G,S52231H,S52231J,S52231K,S52231M,S52231N,S52231P<br>S52231Q,S52231R,S52232D,S52232E,S52232F,S52232G,S52232H<br>S52232J,S52232K,S52232M,S52232N,S52232P,S52232Q,S52232R<br>S52233D,S52233E,S52233F,S52233G,S52233H,S52233J,S52233K<br>S52233M,S52233N,S52233P,S52233Q,S52233R,S52234D,S52234E<br>S52234F,S52234G,S52234H,S52234J,S52234K,S52234M,S52234N<br>S52234P,S52234Q,S52234R,S52235D,S52235E,S52235F,S52235G<br>S52235H,S52235J,S52235K,S52235M,S52235N,S52235P,S52235Q<br>S52235R,S52236D,S52236E,S52236F,S52236G,S52236H,S52236J<br>S52236K,S52236M,S52236N,S52236P,S52236Q,S52236R,S52241D<br>S52241E,S52241F,S52241G,S52241H,S52241J,S52241K,S52241M<br>S52241N,S52241P,S52241Q,S52241R,S52242D,S52242E,S52242F<br>S52242G,S52242H,S52242J,S52242K,S52242M,S52242N,S52242P<br>S52242Q,S52242R,S52243D,S52243E,S52243F,S52243G,S52243H<br>S52243J,S52243K,S52243M,S52243N,S52243P,S52243Q,S52243R<br>S52244D,S52244E,S52244F,S52244G,S52244H,S52244J,S52244K<br>S52244M,S52244N,S52244P,S52244Q,S52244R,S52245D,S52245E<br>S52245F,S52245G,S52245H,S52245J,S52245K,S52245M,S52245N<br>S52245P,S52245Q,S52245R,S52246D,S52246E,S52246F,S52246G<br>S52246H,S52246J,S52246K,S52246M,S52246N,S52246P,S52246Q<br>S52246R,S52251D,S52251E,S52251F,S52251G,S52251H,S52251J<br>S52251K,S52251M,S52251N,S52251P,S52251Q,S52251R,S52252D<br>S52252E,S52252F,S52252G,S52252H,S52252J,S52252K,S52252M<br>S52252N,S52252P,S52252Q,S52252R,S52253D,S52253E,S52253F<br>S52253G,S52253H,S52253J,S52253K,S52253M,S52253N,S52253P<br>S52253Q,S52253R,S52254D,S52254E,S52254F,S52254G,S52254H<br>S52254J,S52254K,S52254M,S52254N,S52254P,S52254Q,S52254R<br>S52255D,S52255E,S52255F,S52255G,S52255H,S52255J,S52255K<br>S52255M,S52255N,S52255P,S52255Q,S52255R,S52256D,S52256E<br>S52256F,S52256G,S52256H,S52256J,S52256K,S52256M,S52256N<br>S52256P,S52256Q,S52256R,S52261D,S52261E,S52261F,S52261G<br>S52261H,S52261J,S52261K,S52261M,S52261N,S52261P,S52261Q<br>S52261R,S52262D,S52262E,S52262F,S52262G,S52262H,S52262J<br>S52262K,S52262M,S52262N,S52262P,S52262Q,S52262R,S52263D<br>S52263E,S52263F,S52263G,S52263H,S52263J,S52263K,S52263M<br>S52263N,S52263P,S52263Q,S52263R,S52264D,S52264E,S52264F<br>S52264G,S52264H,S52264J,S52264K,S52264M,S52264N,S52264P<br>S52264Q,S52264R,S52265D,S52265E,S52265F,S52265G,S52265H<br>S52265J,S52265K,S52265M,S52265N,S52265P,S52265Q,S52265R<br>S52266D,S52266E,S52266F,S52266G,S52266H,S52266J,S52266K<br>S52266M,S52266N,S52266P,S52266Q,S52266R,S52271D,S52271E<br>S52271F,S52271G,S52271H,S52271J,S52271K,S52271M,S52271N<br>S52271P,S52271Q,S52271R,S52272D,S52272E,S52272F,S52272G<br>S52272H,S52272J,S52272K,S52272M,S52272N,S52272P,S52272Q<br>S52272R,S52279D,S52279E,S52279F,S52279G,S52279H,S52279J<br>S52279K,S52279M,S52279N,S52279P,S52279Q,S52279R,S52281D<br>S52281E,S52281F,S52281G,S52281H,S52281J,S52281K,S52281M<br>S52281N,S52281P,S52281Q,S52281R,S52282D,S52282E,S52282F<br>S52282G,S52282H,S52282J,S52282K,S52282M,S52282N,S52282P | |

Continued on next page

Table S9 (continued).

| Diagnosis | ICD-10 | ICD-9 |
|---|---|---|
| | S52282Q,S52282R,S52283D,S52283E,S52283F,S52283G,S52283H S52283J,S52283K,S52283M,S52283N,S52283P,S52283Q,S52283R S52291D,S52291E,S52291F,S52291G,S52291H,S52291J,S52291K S52291M,S52291N,S52291P,S52291Q,S52291R,S52292D,S52292E S52292F,S52292G,S52292H,S52292J,S52292K,S52292M,S52292N S52292P,S52292Q,S52292R,S52299D,S52299E,S52299F,S52299G S52299H,S52299J,S52299K,S52299M,S52299N,S52299P,S52299Q S52299R,S52301D,S52301E,S52301F,S52301G,S52301H,S52301J S52301K,S52301M,S52301N,S52301P,S52301Q,S52301R,S52302D S52302E,S52302F,S52302G,S52302H,S52302J,S52302K,S52302M S52302N,S52302P,S52302Q,S52302R,S52309D,S52309E,S52309F S52309G,S52309H,S52309J,S52309K,S52309M,S52309N,S52309P S52309Q,S52309R,S52311D,S52311G,S52311K,S52311P,S52312D S52312G,S52312K,S52312P,S52319D,S52319G,S52319K,S52319P S52321D,S52321E,S52321F,S52321G,S52321H,S52321J,S52321K S52321M,S52321N,S52321P,S52321Q,S52321R,S52322D,S52322E S52322F,S52322G,S52322H,S52322J,S52322K,S52322M,S52322N S52322P,S52322Q,S52322R,S52323D,S52323E,S52323F,S52323G S52323H,S52323J,S52323K,S52323M,S52323N,S52323P,S52323Q S52323R,S52324D,S52324E,S52324F,S52324G,S52324H,S52324J S52324K,S52324M,S52324N,S52324P,S52324Q,S52324R,S52325D S52325E,S52325F,S52325G,S52325H,S52325J,S52325K,S52325M S52325N,S52325P,S52325Q,S52325R,S52326D,S52326E,S52326F S52326G,S52326H,S52326J,S52326K,S52326M,S52326N,S52326P S52326Q,S52326R,S52331D,S52331E,S52331F,S52331G,S52331H S52331J,S52331K,S52331M,S52331N,S52331P,S52331Q,S52331R S52332D,S52332E,S52332F,S52332G,S52332H,S52332J,S52332K S52332M,S52332N,S52332P,S52332Q,S52332R,S52333D,S52333E S52333F,S52333G,S52333H,S52333J,S52333K,S52333M,S52333N S52333P,S52333Q,S52333R,S52334D,S52334E,S52334F,S52334G S52334H,S52334J,S52334K,S52334M,S52334N,S52334P,S52334Q S52334R,S52335D,S52335E,S52335F,S52335G,S52335H,S52335J S52335K,S52335M,S52335N,S52335P,S52335Q,S52335R,S52336D S52336E,S52336F,S52336G,S52336H,S52336J,S52336K,S52336M S52336N,S52336P,S52336Q,S52336R,S52341D,S52341E,S52341F S52341G,S52341H,S52341J,S52341K,S52341M,S52341N,S52341P S52341Q,S52341R,S52342D,S52342E,S52342F,S52342G,S52342H S52342J,S52342K,S52342M,S52342N,S52342P,S52342Q,S52342R S52343D,S52343E,S52343F,S52343G,S52343H,S52343J,S52343K S52343M,S52343N,S52343P,S52343Q,S52343R,S52344D,S52344E S52344F,S52344G,S52344H,S52344J,S52344K,S52344M,S52344N S52344P,S52344Q,S52344R,S52345D,S52345E,S52345F,S52345G S52345H,S52345J,S52345K,S52345M,S52345N,S52345P,S52345Q S52345R,S52346D,S52346E,S52346F,S52346G,S52346H,S52346J S52346K,S52346M,S52346N,S52346P,S52346Q,S52346R,S52351D S52351E,S52351F,S52351G,S52351H,S52351J,S52351K,S52351M S52351N,S52351P,S52351Q,S52351R,S52352D,S52352E,S52352F S52352G,S52352H,S52352J,S52352K,S52352M,S52352N,S52352P S52352Q,S52352R,S52353D,S52353E,S52353F,S52353G,S52353H S52353J,S52353K,S52353M,S52353N,S52353P,S52353Q,S52353R S52354D,S52354E,S52354F,S52354G,S52354H,S52354J,S52354K S52354M,S52354N,S52354P,S52354Q,S52354R,S52355D,S52355E S52355F,S52355G,S52355H,S52355J,S52355K,S52355M,S52355N S52355P,S52355Q,S52355R,S52356D,S52356E,S52356F,S52356G S52356H,S52356J,S52356K,S52356M,S52356N,S52356P,S52356Q S52356R,S52361D,S52361E,S52361F,S52361G,S52361H,S52361J S52361K,S52361M,S52361N,S52361P,S52361Q,S52361R,S52362D S52362E,S52362F,S52362G,S52362H,S52362J,S52362K,S52362M S52362N,S52362P,S52362Q,S52362R,S52363D,S52363E,S52363F S52363G,S52363H,S52363J,S52363K,S52363M,S52363N,S52363P S52363Q,S52363R,S52364D,S52364E,S52364F,S52364G,S52364H S52364J,S52364K,S52364M,S52364N,S52364P,S52364Q,S52364R S52365D,S52365E,S52365F,S52365G,S52365H,S52365J,S52365K S52365M,S52365N,S52365P,S52365Q,S52365R,S52366D,S52366E S52366F,S52366G,S52366H,S52366J,S52366K,S52366M,S52366N S52366P,S52366Q,S52366R,S52371D,S52371E,S52371F,S52371G S52371H,S52371J,S52371K,S52371M,S52371N,S52371P,S52371Q S52371R,S52372D,S52372E,S52372F,S52372G,S52372H,S52372J S52372K,S52372M,S52372N,S52372P,S52372Q,S52372R,S52379D S52379E,S52379F,S52379G,S52379H,S52379J,S52379K,S52379M S52379N,S52379P,S52379Q,S52379R,S52381D,S52381E,S52381F S52381G,S52381H,S52381J,S52381K,S52381M,S52381N,S52381P S52381Q,S52381R,S52382D,S52382E,S52382F,S52382G,S52382H S52382J,S52382K,S52382M,S52382N,S52382P,S52382Q,S52382R S52389D,S52389E,S52389F,S52389G,S52389H,S52389J,S52389K S52389M,S52389N,S52389P,S52389Q,S52389R,S52391D,S52391E S52391F,S52391G,S52391H,S52391J,S52391K,S52391M,S52391N S52391P,S52391Q,S52391R,S52392D,S52392E,S52392F,S52392G S52392H,S52392J,S52392K,S52392M,S52392N,S52392P,S52392Q S52392R,S52399D,S52399E,S52399F,S52399G,S52399H,S52399J S52399K,S52399M,S52399N,S52399P,S52399Q,S52399R,S52501D S52501E,S52501F,S52501G,S52501H,S52501J,S52501K,S52501M S52501N,S52501P,S52501Q,S52501R,S52502D,S52502E,S52502F S52502G,S52502H,S52502J,S52502K,S52502M,S52502N,S52502P S52502Q,S52502R,S52509D,S52509E,S52509F,S52509G,S52509H | |

Continued on next page

Table S9 (continued).

| Diagnosis | ICD-10 | ICD-9 |
|---|---|---|
| | S52509J,S52509K,S52509M,S52509N,S52509P,S52509Q,S52509R | |
| | S52511D,S52511E,S52511F,S52511G,S52511H,S52511J,S52511K | |
| | S52511M,S52511N,S52511P,S52511Q,S52511R,S52512D,S52512E | |
| | S52512F,S52512G,S52512H,S52512J,S52512K,S52512M,S52512N | |
| | S52512P,S52512Q,S52512R,S52513D,S52513E,S52513F,S52513G | |
| | S52513H,S52513J,S52513K,S52513M,S52513N,S52513P,S52513Q | |
| | S52513R,S52514D,S52514E,S52514F,S52514G,S52514H,S52514J | |
| | S52514K,S52514M,S52514N,S52514P,S52514Q,S52514R,S52515D | |
| | S52515E,S52515F,S52515G,S52515H,S52515J,S52515K,S52515M | |
| | S52515N,S52515P,S52515Q,S52515R,S52516D,S52516E,S52516F | |
| | S52516G,S52516H,S52516J,S52516K,S52516M,S52516N,S52516P | |
| | S52516Q,S52516R,S52521D,S52521G,S52521K,S52521P,S52522D | |
| | S52522G,S52522K,S52522P,S52529D,S52529G,S52529K,S52529P | |
| | S52531D,S52531E,S52531F,S52531G,S52531H,S52531J,S52531K | |
| | S52531M,S52531N,S52531P,S52531Q,S52531R,S52532D,S52532E | |
| | S52532F,S52532G,S52532H,S52532J,S52532K,S52532M,S52532N | |
| | S52532P,S52532Q,S52532R,S52539D,S52539E,S52539F,S52539G | |
| | S52539H,S52539J,S52539K,S52539M,S52539N,S52539P,S52539Q | |
| | S52539R,S52541D,S52541E,S52541F,S52541G,S52541H,S52541J | |
| | S52541K,S52541M,S52541N,S52541P,S52541Q,S52541R,S52542D | |
| | S52542E,S52542F,S52542G,S52542H,S52542J,S52542K,S52542M | |
| | S52542N,S52542P,S52542Q,S52542R,S52549D,S52549E,S52549F | |
| | S52549G,S52549H,S52549J,S52549K,S52549M,S52549N,S52549P | |
| | S52549Q,S52549R,S52551D,S52551E,S52551F,S52551G,S52551H | |
| | S52551J,S52551K,S52551M,S52551N,S52551P,S52551Q,S52551R | |
| | S52552D,S52552E,S52552F,S52552G,S52552H,S52552J,S52552K | |
| | S52552M,S52552N,S52552P,S52552Q,S52552R,S52559D,S52559E | |
| | S52559F,S52559G,S52559H,S52559J,S52559K,S52559M,S52559N | |
| | S52559P,S52559Q,S52559R,S52561D,S52561E,S52561F,S52561G | |
| | S52561H,S52561J,S52561K,S52561M,S52561N,S52561P,S52561Q | |
| | S52561R,S52562D,S52562E,S52562F,S52562G,S52562H,S52562J | |
| | S52562K,S52562M,S52562N,S52562P,S52562Q,S52562R,S52569D | |
| | S52569E,S52569F,S52569G,S52569H,S52569J,S52569K,S52569M | |
| | S52569N,S52569P,S52569Q,S52569R,S52571D,S52571E,S52571F | |
| | S52571G,S52571H,S52571J,S52571K,S52571M,S52571N,S52571P | |
| | S52571Q,S52571R,S52572D,S52572E,S52572F,S52572G,S52572H | |
| | S52572J,S52572K,S52572M,S52572N,S52572P,S52572Q,S52572R | |
| | S52579D,S52579E,S52579F,S52579G,S52579H,S52579J,S52579K | |
| | S52579M,S52579N,S52579P,S52579Q,S52579R,S52591D,S52591E | |
| | S52591F,S52591G,S52591H,S52591J,S52591K,S52591M,S52591N | |
| | S52591P,S52591Q,S52591R,S52592D,S52592E,S52592F,S52592G | |
| | S52592H,S52592J,S52592K,S52592M,S52592N,S52592P,S52592Q | |
| | S52592R,S52599D,S52599E,S52599F,S52599G,S52599H,S52599J | |
| | S52599K,S52599M,S52599N,S52599P,S52599Q,S52599R,S52601D | |
| | S52601E,S52601F,S52601G,S52601H,S52601J,S52601K,S52601M | |
| | S52601N,S52601P,S52601Q,S52601R,S52602D,S52602E,S52602F | |
| | S52602G,S52602H,S52602J,S52602K,S52602M,S52602N,S52602P | |
| | S52602Q,S52602R,S52609D,S52609E,S52609F,S52609G,S52609H | |
| | S52609J,S52609K,S52609M,S52609N,S52609P,S52609Q,S52609R | |
| | S52611D,S52611E,S52611F,S52611G,S52611H,S52611J,S52611K | |
| | S52611M,S52611N,S52611P,S52611Q,S52611R,S52612D,S52612E | |
| | S52612F,S52612G,S52612H,S52612J,S52612K,S52612M,S52612N | |
| | S52612P,S52612Q,S52612R,S52613D,S52613E,S52613F,S52613G | |
| | S52613H,S52613J,S52613K,S52613M,S52613N,S52613P,S52613Q | |
| | S52613R,S52614D,S52614E,S52614F,S52614G,S52614H,S52614J | |
| | S52614K,S52614M,S52614N,S52614P,S52614Q,S52614R,S52615D | |
| | S52615E,S52615F,S52615G,S52615H,S52615J,S52615K,S52615M | |
| | S52615N,S52615P,S52615Q,S52615R,S52616D,S52616E,S52616F | |
| | S52616G,S52616H,S52616J,S52616K,S52616M,S52616N,S52616P | |
| | S52616Q,S52616R,S52621D,S52621G,S52621K,S52621P,S52622D | |
| | S52622G,S52622K,S52622P,S52629D,S52629G,S52629K,S52629P | |
| | S52691D,S52691E,S52691F,S52691G,S52691H,S52691J,S52691K | |
| | S52691M,S52691N,S52691P,S52691Q,S52691R,S52692D,S52692E | |
| | S52692F,S52692G,S52692H,S52692J,S52692K,S52692M,S52692N | |
| | S52692P,S52692Q,S52692R,S52699D,S52699E,S52699F,S52699G | |
| | S52699H,S52699J,S52699K,S52699M,S52699N,S52699P,S52699Q | |
| | S52699R,S5290XD,S5290XE,S5290XF,S5290XG,S5290XH,S5290XJ | |
| | S5290XK,S5290XM,S5290XN,S5290XP,S5290XQ,S5290XR,S5291XD | |
| | S5291XE,S5291XF,S5291XG,S5291XH,S5291XJ,S5291XK,S5291XM | |
| | S5291XN,S5291XP,S5291XQ,S5291XR,S5292XD,S5292XE,S5292XF | |
| | S5292XG,S5292XH,S5292XJ,S5292XK,S5292XM,S5292XN,S5292XP | |
| | S5292XQ,S5292XR,S59001D,S59001G,S59001K,S59001P,S59002D | |
| | S59002G,S59002K,S59002P,S59009D,S59009G,S59009K,S59009P | |
| | S59011D,S59011G,S59011K,S59011P,S59012D,S59012G,S59012K | |
| | S59012P,S59019D,S59019G,S59019K,S59019P,S59021D,S59021G | |
| | S59021K,S59021P,S59022D,S59022G,S59022K,S59022P,S59029D | |
| | S59029G,S59029K,S59029P,S59031D,S59031G,S59031K,S59031P | |
| | S59032D,S59032G,S59032K,S59032P,S59039D,S59039G,S59039K | |
| | S59039P,S59041D,S59041G,S59041K,S59041P,S59042D,S59042G | |
| | S59042K,S59042P,S59049D,S59049G,S59049K,S59049P,S59091D | |
| | S59091G,S59091K,S59091P,S59092D,S59092G,S59092K,S59092P | |
| | S59099D,S59099G,S59099K,S59099P,S59101D,S59101G,S59101K | |
| | S59101P,S59102D,S59102G,S59102K,S59102P,S59109D,S59109G | |
| | S59109K,S59109P,S59111D,S59111G,S59111K,S59111P,S59112D | |
| | S59112G,S59112K,S59112P,S59119D,S59119G,S59119K,S59119P | |

Continued on next page

Table S9 (continued).

| Diagnosis | ICD-10 | ICD-9 |
|---|---|---|
| | S59121D,S59121G,S59121K,S59121P,S59122D,S59122G,S59122K | |
| | S59122P,S59129D,S59129G,S59129K,S59129P,S59131D,S59131G | |
| | S59131K,S59131P,S59132D,S59132G,S59132K,S59132P,S59139D | |
| | S59139G,S59139K,S59139P,S59141D,S59141G,S59141K,S59141P | |
| | S59142D,S59142G,S59142K,S59142P,S59149D,S59149G,S59149K | |
| | S59149P,S59191D,S59191G,S59191K,S59191P,S59192D,S59192G | |
| | S59192K,S59192P,S59199D,S59199G,S59199K,S59199P,S59201D | |
| | S59201G,S59201K,S59201P,S59202D,S59202G,S59202K,S59202P | |
| | S59209D,S59209G,S59209K,S59209P,S59211D,S59211G,S59211K | |
| | S59211P,S59212D,S59212G,S59212K,S59212P,S59219D,S59219G | |
| | S59219K,S59219P,S59221D,S59221G,S59221K,S59221P,S59222D | |
| | S59222G,S59222K,S59222P,S59229D,S59229G,S59229K,S59229P | |
| | S59231D,S59231G,S59231K,S59231P,S59232D,S59232G,S59232K | |
| | S59232P,S59239D,S59239G,S59239K,S59239P,S59241D,S59241G | |
| | S59241K,S59241P,S59242D,S59242G,S59242K,S59242P,S59249D | |
| | S59249G,S59249K,S59249P,S59291D,S59291G,S59291K,S59291P | |
| | S59292D,S59292G,S59292K,S59292P,S59299D,S59299G,S59299K | |
| | S59299P,S62001D,S62001G,S62001K,S62001P,S62002D,S62002G | |
| | S62002K,S62002P,S62009D,S62009G,S62009K,S62009P,S62011D | |
| | S62011G,S62011K,S62011P,S62012D,S62012G,S62012K,S62012P | |
| | S62013D,S62013G,S62013K,S62013P,S62014D,S62014G,S62014K | |
| | S62014P,S62015D,S62015G,S62015K,S62015P,S62016D,S62016G | |
| | S62016K,S62016P,S62021D,S62021G,S62021K,S62021P,S62022D | |
| | S62022G,S62022K,S62022P,S62023D,S62023G,S62023K,S62023P | |
| | S62024D,S62024G,S62024K,S62024P,S62025D,S62025G,S62025K | |
| | S62025P,S62026D,S62026G,S62026K,S62026P,S62031D,S62031G | |
| | S62031K,S62031P,S62032D,S62032G,S62032K,S62032P,S62033D | |
| | S62033G,S62033K,S62033P,S62034D,S62034G,S62034K,S62034P | |
| | S62035D,S62035G,S62035K,S62035P,S62036D,S62036G,S62036K | |
| | S62036P,S62101D,S62101G,S62101K,S62101P,S62102D,S62102G | |
| | S62102K,S62102P,S62109D,S62109G,S62109K,S62109P,S62111D | |
| | S62111G,S62111K,S62111P,S62112D,S62112G,S62112K,S62112P | |
| | S62113D,S62113G,S62113K,S62113P,S62114D,S62114G,S62114K | |
| | S62114P,S62115D,S62115G,S62115K,S62115P,S62116D,S62116G | |
| | S62116K,S62116P,S62121D,S62121G,S62121K,S62121P,S62122D | |
| | S62122G,S62122K,S62122P,S62123D,S62123G,S62123K,S62123P | |
| | S62124D,S62124G,S62124K,S62124P,S62125D,S62125G,S62125K | |
| | S62125P,S62126D,S62126G,S62126K,S62126P,S62131D,S62131G | |
| | S62131K,S62131P,S62132D,S62132G,S62132K,S62132P,S62133D | |
| | S62133G,S62133K,S62133P,S62134D,S62134G,S62134K,S62134P | |
| | S62135D,S62135G,S62135K,S62135P,S62136D,S62136G,S62136K | |
| | S62136P,S62141D,S62141G,S62141K,S62141P,S62142D,S62142G | |
| | S62142K,S62142P,S62143D,S62143G,S62143K,S62143P,S62144D | |
| | S62144G,S62144K,S62144P,S62145D,S62145G,S62145K,S62145P | |
| | S62146D,S62146G,S62146K,S62146P,S62151D,S62151G,S62151K | |
| | S62151P,S62152D,S62152G,S62152K,S62152P,S62153D,S62153G | |
| | S62153K,S62153P,S62154D,S62154G,S62154K,S62154P,S62155D | |
| | S62155G,S62155K,S62155P,S62156D,S62156G,S62156K,S62156P | |
| | S62161D,S62161G,S62161K,S62161P,S62162D,S62162G,S62162K | |
| | S62162P,S62163D,S62163G,S62163K,S62163P,S62164D,S62164G | |
| | S62164K,S62164P,S62165D,S62165G,S62165K,S62165P,S62166D | |
| | S62166G,S62166K,S62166P,S62171D,S62171G,S62171K,S62171P | |
| | S62172D,S62172G,S62172K,S62172P,S62173D,S62173G,S62173K | |
| | S62173P,S62174D,S62174G,S62174K,S62174P,S62175D,S62175G | |
| | S62175K,S62175P,S62176D,S62176G,S62176K,S62176P,S62181D | |
| | S62181G,S62181K,S62181P,S62182D,S62182G,S62182K,S62182P | |
| | S62183D,S62183G,S62183K,S62183P,S62184D,S62184G,S62184K | |
| | S62184P,S62185D,S62185G,S62185K,S62185P,S62186D,S62186G | |
| | S62186K,S62186P,S62201D,S62201G,S62201K,S62201P,S62202D | |
| | S62202G,S62202K,S62202P,S62209D,S62209G,S62209K,S62209P | |
| | S62211D,S62211G,S62211K,S62211P,S62212D,S62212G,S62212K | |
| | S62212P,S62213D,S62213G,S62213K,S62213P,S62221D,S62221G | |
| | S62221K,S62221P,S62222D,S62222G,S62222K,S62222P,S62223D | |
| | S62223G,S62223K,S62223P,S62224D,S62224G,S62224K,S62224P | |
| | S62225D,S62225G,S62225K,S62225P,S62226D,S62226G,S62226K | |
| | S62226P,S62231D,S62231G,S62231K,S62231P,S62232D,S62232G | |
| | S62232K,S62232P,S62233D,S62233G,S62233K,S62233P,S62234D | |
| | S62234G,S62234K,S62234P,S62235D,S62235G,S62235K,S62235P | |
| | S62236D,S62236G,S62236K,S62236P,S62241D,S62241G,S62241K | |
| | S62241P,S62242D,S62242G,S62242K,S62242P,S62243D,S62243G | |
| | S62243K,S62243P,S62244D,S62244G,S62244K,S62244P,S62245D | |
| | S62245G,S62245K,S62245P,S62246D,S62246G,S62246K,S62246P | |
| | S62251D,S62251G,S62251K,S62251P,S62252D,S62252G,S62252K | |
| | S62252P,S62253D,S62253G,S62253K,S62253P,S62254D,S62254G | |
| | S62254K,S62254P,S62255D,S62255G,S62255K,S62255P,S62256D | |
| | S62256G,S62256K,S62256P,S62291D,S62291G,S62291K,S62291P | |
| | S62292D,S62292G,S62292K,S62292P,S62299D,S62299G,S62299K | |
| | S62299P,S62300D,S62300G,S62300K,S62300P,S62301D,S62301G | |
| | S62301K,S62301P,S62302D,S62302G,S62302K,S62302P,S62303D | |
| | S62303G,S62303K,S62303P,S62304D,S62304G,S62304K,S62304P | |
| | S62305D,S62305G,S62305K,S62305P,S62306D,S62306G,S62306K | |
| | S62306P,S62307D,S62307G,S62307K,S62307P,S62308D,S62308G | |
| | S62308K,S62308P,S62309D,S62309G,S62309K,S62309P,S62310D | |
| | S62310G,S62310K,S62310P,S62311D,S62311G,S62311K,S62311P | |
| | S62312D,S62312G,S62312K,S62312P,S62313D,S62313G,S62313K | |

Continued on next page

Table S9 (continued).

| Diagnosis | ICD-10 | ICD-9 |
|---|---|---|
| | S62313P,S62314D,S62314G,S62314K,S62314P,S62315D,S62315G | |
| | S62315K,S62315P,S62316D,S62316G,S62316K,S62316P,S62317D | |
| | S62317G,S62317K,S62317P,S62318D,S62318G,S62318K,S62318P | |
| | S62319D,S62319G,S62319K,S62319P,S62320D,S62320G,S62320K | |
| | S62320P,S62321D,S62321G,S62321K,S62321P,S62322D,S62322G | |
| | S62322K,S62322P,S62323D,S62323G,S62323K,S62323P,S62324D | |
| | S62324G,S62324K,S62324P,S62325D,S62325G,S62325K,S62325P | |
| | S62326D,S62326G,S62326K,S62326P,S62327D,S62327G,S62327K | |
| | S62327P,S62328D,S62328G,S62328K,S62328P,S62329D,S62329G | |
| | S62329K,S62329P,S62330D,S62330G,S62330K,S62330P,S62331D | |
| | S62331G,S62331K,S62331P,S62332D,S62332G,S62332K,S62332P | |
| | S62333D,S62333G,S62333K,S62333P,S62334D,S62334G,S62334K | |
| | S62334P,S62335D,S62335G,S62335K,S62335P,S62336D,S62336G | |
| | S62336K,S62336P,S62337D,S62337G,S62337K,S62337P,S62338D | |
| | S62338G,S62338K,S62338P,S62339D,S62339G,S62339K,S62339P | |
| | S62340D,S62340G,S62340K,S62340P,S62341D,S62341G,S62341K | |
| | S62341P,S62342D,S62342G,S62342K,S62342P,S62343D,S62343G | |
| | S62343K,S62343P,S62344D,S62344G,S62344K,S62344P,S62345D | |
| | S62345G,S62345K,S62345P,S62346D,S62346G,S62346K,S62346P | |
| | S62347D,S62347G,S62347K,S62347P,S62348D,S62348G,S62348K | |
| | S62348P,S62349D,S62349G,S62349K,S62349P,S62350D,S62350G | |
| | S62350K,S62350P,S62351D,S62351G,S62351K,S62351P,S62352D | |
| | S62352G,S62352K,S62352P,S62353D,S62353G,S62353K,S62353P | |
| | S62354D,S62354G,S62354K,S62354P,S62355D,S62355G,S62355K | |
| | S62355P,S62356D,S62356G,S62356K,S62356P,S62357D,S62357G | |
| | S62357K,S62357P,S62358D,S62358G,S62358K,S62358P,S62359D | |
| | S62359G,S62359K,S62359P,S62360D,S62360G,S62360K,S62360P | |
| | S62361D,S62361G,S62361K,S62361P,S62362D,S62362G,S62362K | |
| | S62362P,S62363D,S62363G,S62363K,S62363P,S62364D,S62364G | |
| | S62364K,S62364P,S62365D,S62365G,S62365K,S62365P,S62366D | |
| | S62366G,S62366K,S62366P,S62367D,S62367G,S62367K,S62367P | |
| | S62368D,S62368G,S62368K,S62368P,S62369D,S62369G,S62369K | |
| | S62369P,S62390D,S62390G,S62390K,S62390P,S62391D,S62391G | |
| | S62391K,S62391P,S62392D,S62392G,S62392K,S62392P,S62393D | |
| | S62393G,S62393K,S62393P,S62394D,S62394G,S62394K,S62394P | |
| | S62395D,S62395G,S62395K,S62395P,S62396D,S62396G,S62396K | |
| | S62396P,S62397D,S62397G,S62397K,S62397P,S62398D,S62398G | |
| | S62398K,S62398P,S62399D,S62399G,S62399K,S62399P,S62501D | |
| | S62501G,S62501K,S62501P,S62502D,S62502G,S62502K,S62502P | |
| | S62509D,S62509G,S62509K,S62509P,S62511D,S62511G,S62511K | |
| | S62511P,S62512D,S62512G,S62512K,S62512P,S62513D,S62513G | |
| | S62513K,S62513P,S62514D,S62514G,S62514K,S62514P,S62515D | |
| | S62515G,S62515K,S62515P,S62516D,S62516G,S62516K,S62516P | |
| | S62521D,S62521G,S62521K,S62521P,S62522D,S62522G,S62522K | |
| | S62522P,S62523D,S62523G,S62523K,S62523P,S62524D,S62524G | |
| | S62524K,S62524P,S62525D,S62525G,S62525K,S62525P,S62526D | |
| | S62526G,S62526K,S62526P,S62600D,S62600G,S62600K,S62600P | |
| | S62601D,S62601G,S62601K,S62601P,S62602D,S62602G,S62602K | |
| | S62602P,S62603D,S62603G,S62603K,S62603P,S62604D,S62604G | |
| | S62604K,S62604P,S62605D,S62605G,S62605K,S62605P,S62606D | |
| | S62606G,S62606K,S62606P,S62607D,S62607G,S62607K,S62607P | |
| | S62608D,S62608G,S62608K,S62608P,S62609D,S62609G,S62609K | |
| | S62609P,S62610D,S62610G,S62610K,S62610P,S62611D,S62611G | |
| | S62611K,S62611P,S62612D,S62612G,S62612K,S62612P,S62613D | |
| | S62613G,S62613K,S62613P,S62614D,S62614G,S62614K,S62614P | |
| | S62615D,S62615G,S62615K,S62615P,S62616D,S62616G,S62616K | |
| | S62616P,S62617D,S62617G,S62617K,S62617P,S62618D,S62618G | |
| | S62618K,S62618P,S62619D,S62619G,S62619K,S62619P,S62620D | |
| | S62620G,S62620K,S62620P,S62621D,S62621G,S62621K,S62621P | |
| | S62622D,S62622G,S62622K,S62622P,S62623D,S62623G,S62623K | |
| | S62623P,S62624D,S62624G,S62624K,S62624P,S62625D,S62625G | |
| | S62625K,S62625P,S62626D,S62626G,S62626K,S62626P,S62627D | |
| | S62627G,S62627K,S62627P,S62628D,S62628G,S62628K,S62628P | |
| | S62629D,S62629G,S62629K,S62629P,S62630D,S62630G,S62630K | |
| | S62630P,S62631D,S62631G,S62631K,S62631P,S62632D,S62632G | |
| | S62632K,S62632P,S62633D,S62633G,S62633K,S62633P,S62634D | |
| | S62634G,S62634K,S62634P,S62635D,S62635G,S62635K,S62635P | |
| | S62636D,S62636G,S62636K,S62636P,S62637D,S62637G,S62637K | |
| | S62637P,S62638D,S62638G,S62638K,S62638P,S62639D,S62639G | |
| | S62639K,S62639P,S62640D,S62640G,S62640K,S62640P,S62641D | |
| | S62641G,S62641K,S62641P,S62642D,S62642G,S62642K,S62642P | |
| | S62643D,S62643G,S62643K,S62643P,S62644D,S62644G,S62644K | |
| | S62644P,S62645D,S62645G,S62645K,S62645P,S62646D,S62646G | |
| | S62646K,S62646P,S62647D,S62647G,S62647K,S62647P,S62648D | |
| | S62648G,S62648K,S62648P,S62649D,S62649G,S62649K,S62649P | |
| | S62650D,S62650G,S62650K,S62650P,S62651D,S62651G,S62651K | |
| | S62651P,S62652D,S62652G,S62652K,S62652P,S62653D,S62653G | |
| | S62653K,S62653P,S62654D,S62654G,S62654K,S62654P,S62655D | |
| | S62655G,S62655K,S62655P,S62656D,S62656G,S62656K,S62656P | |
| | S62657D,S62657G,S62657K,S62657P,S62658D,S62658G,S62658K | |
| | S62658P,S62659D,S62659G,S62659K,S62659P,S62660D,S62660G | |
| | S62660K,S62660P,S62661D,S62661G,S62661K,S62661P,S62662D | |
| | S62662G,S62662K,S62662P,S62663D,S62663G,S62663K,S62663P | |
| | S62664D,S62664G,S62664K,S62664P,S62665D,S62665G,S62665K | |
| | S62665P,S62666D,S62666G,S62666K,S62666P,S62667D,S62667G | |

Continued on next page

Table S9 (continued).

| Diagnosis | ICD-10 | ICD-9 |
|---|---|---|
| | S62667K,S62667P,S62668D,S62668G,S62668K,S62668P,S62669D S62669G,S62669K,S62669P,S6290XD,S6290XG,S6290XK,S6290XP S6291XD,S6291XG,S6291XK,S6291XP,S6292XD,S6292XG,S6292XK S6292XP | |
| Fracture of torso, | M96A1,M96A2,M96A3,M96A4,M96A9,S2220XA,S2220XB,S2221XA,S2221XB | 80700,80701,80702,80703,80704,80705, 80706,80707,80708,80709 |
| initial encounter | S2222XA,S2222XB,S2223XA,S2223XB,S2224XA,S2224XB,S2231XA | 80711,80712,80713,80714,80715,80716, 80717,80718,80719,8072 |
| | S2231XB,S2232XA,S2232XB,S2239XA,S2239XB,S2241XA,S2241XB | 8073,8074,8080,8081,8082,8083,80841, 80842,80843,80844,80849 |
| | S2242XA,S2242XB,S2243XA,S2243XB,S2249XA,S2249XB,S225XXA | 80851,80852,80853,80854,80859,8090, 8091 |
| | S225XXB,S229XXA,S229XXB,S32301A,S32301B,S32302A,S32302B S32309A,S32309B,S32311A,S32311B,S32312A,S32312B,S32313A S32313B,S32314A,S32314B,S32315A,S32315B,S32316A,S32316B S32391A,S32391B,S32392A,S32392B,S32399A,S32399B,S32401A S32401B,S32402A,S32402B,S32409A,S32409B,S32411A,S32411B S32412A,S32412B,S32413A,S32413B,S32414A,S32414B,S32415A S32415B,S32416A,S32416B,S32421A,S32421B,S32422A,S32422B S32423A,S32423B,S32424A,S32424B,S32425A,S32425B,S32426A S32426B,S32431A,S32431B,S32432A,S32432B,S32433A,S32433B S32434A,S32434B,S32435A,S32435B,S32436A,S32436B,S32441A S32441B,S32442A,S32442B,S32443A,S32443B,S32444A,S32444B S32445A,S32445B,S32446A,S32446B,S32451A,S32451B,S32452A S32452B,S32453A,S32453B,S32454A,S32454B,S32455A,S32455B S32456A,S32456B,S32461A,S32461B,S32462A,S32462B,S32463A S32463B,S32464A,S32464B,S32465A,S32465B,S32466A,S32466B S32471A,S32471B,S32472A,S32472B,S32473A,S32473B,S32474A S32474B,S32475A,S32475B,S32476A,S32476B,S32481A,S32481B S32482A,S32482B,S32483A,S32483B,S32484A,S32484B,S32485A S32485B,S32486A,S32486B,S32491A,S32491B,S32492A,S32492B S32499A,S32499B,S32501A,S32501B,S32502A,S32502B,S32509A S32509B,S32511A,S32511B,S32512A,S32512B,S32519A,S32519B S32591A,S32591B,S32592A,S32592B,S32599A,S32599B,S32601A S32601B,S32602A,S32602B,S32609A,S32609B,S32611A,S32611B S32612A,S32612B,S32613A,S32613B,S32614A,S32614B,S32615A S32615B,S32616A,S32616B,S32691A,S32691B,S32692A,S32692B S32699A,S32699B,S32810A,S32810B,S32811A,S32811B,S3282XA S3282XB,S3289XA,S3289XB | |
| Fracture of torso, subsequent encounter | S2220XD,S2220XG,S2220XK,S2221XD,S2221XG,S2221XK,S2222XD S2222XG,S2222XK,S2223XD,S2223XG,S2223XK,S2224XD,S2224XG S2224XK,S2231XD,S2231XG,S2231XK,S2232XD,S2232XG,S2232XK S2239XD,S2239XG,S2239XK,S2241XD,S2241XG,S2241XK,S2242XD S2242XG,S2242XK,S2243XD,S2243XG,S2243XK,S2249XD,S2249XG S2249XK,S225XXD,S225XXG,S225XXK,S229XXD,S229XXG,S229XXK S32301D,S32301G,S32301K,S32302D,S32302G,S32302K,S32309D S32309G,S32309K,S32311D,S32311G,S32311K,S32312D,S32312G S32312K,S32313D,S32313G,S32313K,S32314D,S32314G,S32314K S32315D,S32315G,S32315K,S32316D,S32316G,S32316K,S32391D S32391G,S32391K,S32392D,S32392G,S32392K,S32399D,S32399G S32399K,S32401D,S32401G,S32401K,S32402D,S32402G,S32402K S32409D,S32409G,S32409K,S32411D,S32411G,S32411K,S32412D S32412G,S32412K,S32413D,S32413G,S32413K,S32414D,S32414G S32414K,S32415D,S32415G,S32415K,S32416D,S32416G,S32416K S32421D,S32421G,S32421K,S32422D,S32422G,S32422K,S32423D S32423G,S32423K,S32424D,S32424G,S32424K,S32425D,S32425G S32425K,S32426D,S32426G,S32426K,S32431D,S32431G,S32431K S32432D,S32432G,S32432K,S32433D,S32433G,S32433K,S32434D S32434G,S32434K,S32435D,S32435G,S32435K,S32436D,S32436G S32436K,S32441D,S32441G,S32441K,S32442D,S32442G,S32442K S32443D,S32443G,S32443K,S32444D,S32444G,S32444K,S32445D S32445G,S32445K,S32446D,S32446G,S32446K,S32451D,S32451G S32451K,S32452D,S32452G,S32452K,S32453D,S32453G,S32453K S32454D,S32454G,S32454K,S32455D,S32455G,S32455K,S32456D S32456G,S32456K,S32461D,S32461G,S32461K,S32462D,S32462G S32462K,S32463D,S32463G,S32463K,S32464D,S32464G,S32464K S32465D,S32465G,S32465K,S32466D,S32466G,S32466K,S32471D S32471G,S32471K,S32472D,S32472G,S32472K,S32473D,S32473G S32473K,S32474D,S32474G,S32474K,S32475D,S32475G,S32475K S32476D,S32476G,S32476K,S32481D,S32481G,S32481K,S32482D S32482G,S32482K,S32483D,S32483G,S32483K,S32484D,S32484G S32484K,S32485D,S32485G,S32485K,S32486D,S32486G,S32486K S32491D,S32491G,S32491K,S32492D,S32492G,S32492K,S32499D S32499G,S32499K,S32501D,S32501G,S32501K,S32502D,S32502G S32502K,S32509D,S32509G,S32509K,S32511D,S32511G,S32511K S32512D,S32512G,S32512K,S32519D,S32519G,S32519K,S32591D S32591G,S32591K,S32592D,S32592G,S32592K,S32599D,S32599G S32599K,S32601D,S32601G,S32601K,S32602D,S32602G,S32602K S32609D,S32609G,S32609K,S32611D,S32611G,S32611K,S32612D S32612G,S32612K,S32613D,S32613G,S32613K,S32614D,S32614G S32614K,S32615D,S32615G,S32615K,S32616D,S32616G,S32616K S32691D,S32691G,S32691K,S32692D,S32692G,S32692K,S32699D S32699G,S32699K,S32810D,S32810G,S32810K,S32811D,S32811G | 73382,V5413,V5419 |

Continued on next page

Table S9 (continued).

| Diagnosis | ICD-10 | ICD-9 |
|---|---|---|
| | S32811K,S3282XD,S3282XG,S3282XK,S3289XD,S3289XG,S3289XK | |
| Fungal infections | B350,B351,B352,B353,B354,B355,B356,B358,B359,B360,B361,B362<br>B363,B368,B369,B370,B371,B372,B373,B3731,B3732,B3741,B3742<br>B3749,B375,B376,B377,B3781,B3782,B3783,B3784,B3789,B379,B380<br>B381,B382,B383,B384,B387,B3881,B3889,B389,B390,B391,B392,B393<br>B394,B395,B399,B400,B401,B402,B403,B407,B4081,B4089,B409,B410<br>B417,B418,B419,B420,B421,B427,B4281,B4282,B4289,B429,B430<br>B431,B432,B438,B439,B440,B441,B442,B447,B4481,B4489,B449,B450<br>B451,B452,B453,B457,B458,B459,B460,B461,B462,B463,B464,B465<br>B468,B469,B470,B471,B479,B480,B481,B482,B483,B484,B488,B49<br>B59,P375 | 0394,0399,1100,1101,1102,1103,1104,<br>1105,1106,1108,1109,1110<br>1111,1112,1113,1118,1119,1120,1121,<br>1122,1123,1124,1125,11281<br>11282,11283,11284,11285,11289,1129,<br>1140,1141,1142,1143,1144<br>1145,1149,11500,11505,11509,11510,<br>11590,1160,1161,1162,1170<br>1171,1172,1173,1174,1175,1176,1177,<br>1178,1179,118,1363,3210<br>4846,5186,7717,99591 |
| Gangrene | E0852,E0952,E1052,E1152,E1352,I70261,I70262,I70263,I70268<br>I70269,I70361,I70362,I70363,I70368,I70369,I70461,I70462<br>I70463,I70468,I70469,I70561,I70562,I70563,I70568,I70569<br>I70661,I70662,I70663,I70668,I70669,I70761,I70762,I70763<br>I70768,I70769,I7301,I96,J850,K4010,K4011,K4040,K4041,K4110<br>K4111,K4140,K4141,K421,K431,K434,K437,K441,K451,K461,K82A1<br>N493 | 24970,25070,25071,44024,44030,44031,<br>44032,4430,44381,5130<br>55000,55001,55002,55003,55100,55101,<br>55102,55103,5511,55120<br>55121,55129,5513,5518,5519,6084,7854 |
| Gastritis and<br>duodenitis | K2900,K2901,K2920,K2921,K2930,K2931,K2940,K2941,K2950,K2951<br>K2960,K2961,K2970,K2971,K2980,K2981,K2990,K2991 | 53500,53501,53510,53511,53520,53521,<br>53530,53531,53540,53541<br>53550,53551,53560,53561 |
| Gastroduodenal ulcer | K251,K253,K255,K257,K259,K261,K263,K265,K267,K269,K271,K273<br>K275,K277,K279,K281,K283,K285,K287,K289 | 53110,53130,53150,53170,53190,53210,<br>53230,53250,53270,53290<br>53310,53330,53350,53370,53390,53410,<br>53430,53450,53470,53490 |
| Gastrointestinal and<br>biliary perforation | K223,K251,K255,K261,K265,K271,K275,K281,K285,K3532,K3533<br>K5700,K5701,K5720,K5721,K5740,K5741,K5780,K5781,K631,K822<br>K82A2,K832 | 5304,53110,53150,53210,53250,53310,<br>53350,53410,53450,56201<br>56203,56211,56213,5695,56983,5754,5763 |
| Gastrointestinal<br>cancers - all other types | C240,C241,C248,C249,C261,C269,C480,C488,C49A0,C49A9,D017,D019 | 1561,1562,1568,1569,1580,1588,1591,<br>1598,1599,2309 |
| Gastrointestinal<br>cancers - anus | C210,C211,C212,C218,D013 | 1542,1543,1548,2305,2306 |
| Gastrointestinal<br>cancers - bile duct | C221 | 1551 |
| Gastrointestinal<br>cancers - colorectal | C180,C181,C182,C183,C184,C185,C186,C187,C188,C189,C19,C20<br>C260,C49A4,C49A5,D010,D011,D012,D0140 | 1530,1531,1532,1533,1534,1535,1536,<br>1537,1538,1539,1540,1541<br>1590,2303,2304,2307 |
| Gastrointestinal<br>cancers - esophagus | C153,C154,C155,C158,C159,C49A1,D001 | 1500,1501,1502,1503,1504,1505,1508,<br>1509,2301 |
| Gastrointestinal<br>cancers - gallbladder | C23 | 1560 |
| Gastrointestinal<br>cancers - liver | C220,C222,C223,C224,C227,C228,C229,D015 | 1550,1552,2308 |
| Gastrointestinal<br>cancers - peritoneum | C481,C482,C488 | 1588,1589 |
| Gastrointestinal<br>cancers - small intestine | C170,C171,C172,C173,C178,C179,C49A3,D0149 | 1520,1521,1522,1523,1528,1529,2307 |
| Gastrointestinal<br>cancers - stomach | C160,C161,C162,C163,C164,C165,C166,C168,C169,C49A2,D002 | 1510,1511,1512,1513,1514,1515,1516,<br>1518,1519,2302 |
| Gastrointestinal<br>hemorrhage | I8501,I8511,K2081,K2091,K2101,K2211,K250,K252,K254,K256,K260<br>K262,K264,K266,K270,K272,K274,K276,K280,K282,K284,K286,K2901<br>K2921,K2931,K2941,K2951,K2961,K2971,K2981,K2991,K31811,K50011<br>K50111,K50811,K50911,K51011,K51211,K51311,K51411,K51511<br>K51811,K51911,K5521,K5701,K5711,K5713,K5721,K5731,K5733,K5741 | 4560,45620,53021,53100,53120,53140,<br>53160,53200,53220,53240<br>53260,53300,53320,53340,53360,53400,<br>53420,53440,53460,53501<br>53511,53521,53531,53541,53551,53561,<br>53783,5550,5551,5552,5559<br>5562,5563,5564,5565,5566,5568,5569,<br>56202,56203,56212,56213<br>5693,5695,56985,5780,5781,5789 |

Continued on next page

Table S9 (continued).

| Diagnosis | ICD-10 | ICD-9 |
|---|---|---|
| | K5751,K5753,K5781,K5791,K5793,K625,K920,K921,K922 | |
| General sensation perception signs and symptoms | R42,R440,R441,R442,R443,R448,R449 | 36816,7801,7804,79989 |
| Genitourinary | Q5001,Q5002,Q501,Q502,Q5031,Q5032,Q5039,Q504,Q505,Q506,Q510 | 7520,75210,75211,75219,7522,75231, 75232,75233,75234,75235 |
| congenital anomalies | Q5110,Q5111,Q512,Q5120,Q5121,Q5122,Q5128,Q513,Q514,Q515,Q516 | 75236,75239,75240,75241,75242,75243, 75244,75245,75246,75247 |
| | Q517,Q51810,Q51811,Q51818,Q51820,Q51821,Q51828,Q519,Q520 | 75249,75251,75252,75261,75262,75263, 75264,75265,75269,7527 |
| | Q5210,Q5211,Q5212,Q52120,Q52121,Q52122,Q52123,Q52124,Q52129 | 75281,75289,7529,7530,75310,75311, 75312,75313,75314,75315 |
| | Q522,Q523,Q524,Q525,Q526,Q5270,Q5271,Q5279,Q528,Q529,Q5300 | 75316,75317,75319,75320,75321,75322, 75323,75329,7533,7534 |
| | Q5301,Q5302,Q5310,Q5311,Q53111,Q53112,Q5312,Q5313,Q5320,Q5321 | 7535,7536,7537,7538,7539 |
| | Q53211,Q53212,Q5322,Q5323,Q539,Q540,Q541,Q542,Q543,Q544,Q548 | |
| | Q549,Q550,Q551,Q5520,Q5521,Q5522,Q5523,Q5529,Q553,Q554,Q555 | |
| | Q5561,Q5562,Q5563,Q5564,Q5569,Q557,Q558,Q559,Q560,Q561,Q562 | |
| | Q563,Q564,Q600,Q601,Q602,Q603,Q604,Q605,Q606,Q6100,Q6101 | |
| | Q6102,Q6111,Q6119,Q612,Q613,Q614,Q615,Q618,Q619,Q620,Q6210 | |
| | Q6211,Q6212,Q622,Q6231,Q6232,Q6239,Q624,Q625,Q6260,Q6261 | |
| | Q6262,Q6263,Q6269,Q627,Q628,Q630,Q631,Q632,Q633,Q638,Q639 | |
| | Q640,Q6410,Q6411,Q6412,Q6419,Q642,Q6431,Q6432,Q6433,Q6439 | |
| | Q644,Q645,Q646,Q6470,Q6471,Q6472,Q6473,Q6474,Q6475,Q6479,Q648 | |
| | Q649 | |
| Genitourinary signs | N398,N399,R300,R301,R309,R330,R338,R339,R34,R350,R351,R358 | 59989,5999,60882,7881,78820,78821, 78829,78841,78842,78843 |
| and symptoms | R3581,R3589,R360,R361,R369,R390,R3911,R3912,R3913,R3914,R3915 | 7885,78861,78862,78863,78864,78865, 78869,7887,7888,78899,7911 |
| | R3916,R3919,R39191,R39192,R39198,R392,R3982,R3983,R3984,R3989 | 7912,7913,7914,7916,7917,7919,7960 |
| | R399,R820,R821,R822,R823,R824,R825,R826,R827,R8271,R8279,R828 | |
| | R8281,R8289,R8290,R8291,R8299,R82991,R82992,R82993,R82994 | |
| | R82998 | |
| Glaucoma | H21521,H21522,H21523,H21529,H21531,H21532,H21533,H21539 | 36042,36471,36473,36476,36477,36500, 36501,36502,36503,36504 |
| | H21541,H21542,H21543,H21549,H21551,H21552,H21553,H21559 | 36505,36506,36510,36511,36512,36513, 36515,36520,36521,36522 |
| | H40001,H40002,H40003,H40009,H40011,H40012,H40013,H40019 | 36523,36524,36531,36532,36544,36552, 36559,36560,36561,36562 |
| | H40021,H40022,H40023,H40029,H40031,H40032,H40033,H40039 | 36564,36565,36570,36571,36572,36573, 36574,36581,36582,36583 |
| | H40041,H40042,H40043,H40049,H40051,H40052,H40053,H40059 | 36589,3659 |
| | H40061,H40062,H40063,H40069,H4010X0,H4010X1,H4010X2,H4010X3 | |
| | H4010X4,H401110,H401111,H401112,H401113,H401114,H401120 | |
| | H401121,H401122,H401123,H401124,H401130,H401131,H401132 | |
| | H401133,H401134,H401190,H401191,H401192,H401193,H401194 | |
| | H4011X0,H4011X1,H4011X2,H4011X3,H4011X4,H401210,H401211 | |
| | H401212,H401213,H401214,H401220,H401221,H401222,H401223 | |
| | H401224,H401230,H401231,H401232,H401233,H401234,H401290 | |
| | H401291,H401292,H401293,H401294,H401310,H401311,H401312 | |
| | H401313,H401314,H401320,H401321,H401322,H401323,H401324 | |
| | H401330,H401331,H401332,H401333,H401334,H401390,H401391 | |
| | H401392,H401393,H401394,H401410,H401411,H401412,H401413 | |
| | H401414,H401420,H401421,H401422,H401423,H401424,H401430 | |
| | H401431,H401432,H401433,H401434,H401490,H401491,H401492 | |
| | H401493,H401494,H40151,H40152,H40153,H40159,H4020X0,H4020X1 | |
| | H4020X2,H4020X3,H4020X4,H40211,H40212,H40213,H40219,H402210 | |
| | H402211,H402212,H402213,H402214,H402220,H402221,H402222 | |
| | H402223,H402224,H402230,H402231,H402232,H402233,H402234 | |
| | H402290,H402291,H402292,H402293,H402294,H40231,H40232,H40233 | |
| | H40239,H40241,H40242,H40243,H40249,H4030X0,H4030X1,H4030X2 | |
| | H4030X3,H4030X4,H4031X0,H4031X1,H4031X2,H4031X3,H4031X4 | |
| | H4032X0,H4032X1,H4032X2,H4032X3,H4032X4,H4033X0,H4033X1 | |
| | H4033X2,H4033X3,H4033X4,H4040X0,H4040X1,H4040X2,H4040X3 | |
| | H4040X4,H4041X0,H4041X1,H4041X2,H4041X3,H4041X4,H4042X0 | |
| | H4042X1,H4042X2,H4042X3,H4042X4,H4043X0,H4043X1,H4043X2 | |
| | H4043X3,H4043X4,H4050X0,H4050X1,H4050X2,H4050X3,H4050X4 | |
| | H4051X0,H4051X1,H4051X2,H4051X3,H4051X4,H4052X0,H4052X1 | |
| | H4052X2,H4052X3,H4052X4,H4053X0,H4053X1,H4053X2,H4053X3 | |
| | H4053X4,H4060X0,H4060X1,H4060X2,H4060X3,H4060X4,H4061X0 | |
| | H4061X1,H4061X2,H4061X3,H4061X4,H4062X0,H4062X1,H4062X2 | |
| | H4062X3,H4062X4,H4063X0,H4063X1,H4063X2,H4063X3,H4063X4 | |
| | H40811,H40812,H40813,H40819,H40821,H40822,H40823,H40829 | |
| | H40831,H40832,H40833,H40839,H4089,H409,H42,H44511,H44512 | |
| | H44513,H44519 | |
| Gout | M1000,M10011,M10012,M10019,M10021,M10022,M10029,M10031,M10032 | 27400,27401,27402,27403,27410,27489, 2749,9849 |
| | M10039,M10041,M10042,M10049,M10051,M10052,M10059,M10061 | |
| | M10062,M10069,M10071,M10072,M10079,M1008,M1009,M1010,M10111 | |
| | M10112,M10119,M10121,M10122,M10129,M10131,M10132,M10139 | |
| | M10141,M10142,M10149,M10151,M10152,M10159,M10161,M10162 | |

Continued on next page

Table S9 (continued).

| Diagnosis | ICD-10 | ICD-9 |
|---|---|---|
| | M10169,M10171,M10172,M10179,M1018,M1019,M1020,M10211,M10212<br>M10219,M10221,M10222,M10229,M10231,M10232,M10239,M10241<br>M10242,M10249,M10251,M10252,M10259,M10261,M10262,M10269<br>M10271,M10272,M10279,M1028,M1029,M1030,M10311,M10312,M10319<br>M10321,M10322,M10329,M10331,M10332,M10339,M10341,M10342<br>M10349,M10351,M10352,M10359,M10361,M10362,M10369,M10371<br>M10372,M10379,M1038,M1039,M1040,M10411,M10412,M10419,M10421<br>M10422,M10429,M10431,M10432,M10439,M10441,M10442,M10449<br>M10451,M10452,M10459,M10461,M10462,M10469,M10471,M10472<br>M10479,M1048,M1049,M109,M1A00X0,M1A00X1,M1A0110,M1A0111<br>M1A0120,M1A0121,M1A0190,M1A0191,M1A0210,M1A0211,M1A0220<br>M1A0221,M1A0290,M1A0291,M1A0310,M1A0311,M1A0320,M1A0321<br>M1A0390,M1A0391,M1A0410,M1A0411,M1A0420,M1A0421,M1A0490<br>M1A0491,M1A0510,M1A0511,M1A0520,M1A0521,M1A0590,M1A0591<br>M1A0610,M1A0611,M1A0620,M1A0621,M1A0690,M1A0691,M1A0710<br>M1A0711,M1A0720,M1A0721,M1A0790,M1A0791,M1A08X0,M1A08X1<br>M1A09X0,M1A09X1,M1A10X0,M1A10X1,M1A1110,M1A1111,M1A1120<br>M1A1121,M1A1190,M1A1191,M1A1210,M1A1211,M1A1220,M1A1221<br>M1A1290,M1A1291,M1A1310,M1A1311,M1A1320,M1A1321,M1A1390<br>M1A1391,M1A1410,M1A1411,M1A1420,M1A1421,M1A1490,M1A1491<br>M1A1510,M1A1511,M1A1520,M1A1521,M1A1590,M1A1591,M1A1610<br>M1A1611,M1A1620,M1A1621,M1A1690,M1A1691,M1A1710,M1A1711<br>M1A1720,M1A1721,M1A1790,M1A1791,M1A18X0,M1A18X1,M1A19X0<br>M1A19X1,M1A20X0,M1A20X1,M1A2110,M1A2111,M1A2120,M1A2121<br>M1A2190,M1A2191,M1A2210,M1A2211,M1A2220,M1A2221,M1A2290<br>M1A2291,M1A2310,M1A2311,M1A2320,M1A2321,M1A2390,M1A2391<br>M1A2410,M1A2411,M1A2420,M1A2421,M1A2490,M1A2491,M1A2510<br>M1A2511,M1A2520,M1A2521,M1A2590,M1A2591,M1A2610,M1A2611<br>M1A2620,M1A2621,M1A2690,M1A2691,M1A2710,M1A2711,M1A2720<br>M1A2721,M1A2790,M1A2791,M1A28X0,M1A28X1,M1A29X0,M1A29X1<br>M1A30X0,M1A30X1,M1A3110,M1A3111,M1A3120,M1A3121,M1A3190<br>M1A3191,M1A3210,M1A3211,M1A3220,M1A3221,M1A3290,M1A3291<br>M1A3310,M1A3311,M1A3320,M1A3321,M1A3390,M1A3391,M1A3410<br>M1A3411,M1A3420,M1A3421,M1A3490,M1A3491,M1A3510,M1A3511<br>M1A3520,M1A3521,M1A3590,M1A3591,M1A3610,M1A3611,M1A3620<br>M1A3621,M1A3690,M1A3691,M1A3710,M1A3711,M1A3720,M1A3721<br>M1A3790,M1A3791,M1A38X0,M1A38X1,M1A39X0,M1A39X1,M1A40X0<br>M1A40X1,M1A4110,M1A4111,M1A4120,M1A4121,M1A4190,M1A4191<br>M1A4210,M1A4211,M1A4220,M1A4221,M1A4290,M1A4291,M1A4310<br>M1A4311,M1A4320,M1A4321,M1A4390,M1A4391,M1A4410,M1A4411<br>M1A4420,M1A4421,M1A4490,M1A4491,M1A4510,M1A4511,M1A4520<br>M1A4521,M1A4590,M1A4591,M1A4610,M1A4611,M1A4620,M1A4621<br>M1A4690,M1A4691,M1A4710,M1A4711,M1A4720,M1A4721,M1A4790<br>M1A4791,M1A48X0,M1A48X1,M1A49X0,M1A49X1,M1A9XX0,M1A9XX1 | |
| HIV infection | B20,B9735,O98711,O98712,O98713,O98719,O9872,O9873,Z21 | 042,07953,64760,64761,64763,64764,V08 |
| Hallucinogen-related disorders | F1610,F16120,F16121,F16122,F16129,F1614,F16150,F16151,F16159<br>F16180,F16183,F16188,F1619,F1620,F16220,F16221,F16229,F1624<br>F16250,F16251,F16259,F16280,F16283,F16288,F1629,F1690,F1691<br>F16920,F16921,F16929,F1694,F16950,F16951,F16959,F16980,F16983<br>F16988,F1699,T408X1A,T408X2A,T408X3A,T408X4A,T40901A,T40902A<br>T40903A,T40904A,T40905A,T40991A,T40992A,T40993A,T40994A<br>T40995A,T43641A,T43642A,T43643A,T43644A | 29211,29212,2922,29281,29284,29289,<br>2929,30450,30451,30452<br>30530,30531,30532,30533,9696,99529,<br>E8541,E9396,E9503,E9620<br>E9802 |
| Head and neck cancers - all other types | C148,C300,C301,C310,C311,C312,C313,C318,C319,C760 | 1498,1499,1600,1601,1602,1603,1604,<br>1605,1608,1609,1950 |
| Head and neck cancers - eye | C6900,C6901,C6902,C6910,C6911,C6912,C6920,C6921,C6922,C6930<br>C6931,C6932,C6940,C6941,C6942,C6950,C6951,C6952,C6960,C6961<br>C6962,C6980,C6981,C6982,C6990,C6991,C6992,D0920,D0921,D0922 | 1900,1901,1902,1903,1904,1905,1906,<br>1907,1908,1909,2340 |
| Head and neck cancers - hypopharyngeal | C12,C130,C131,C132,C138,C139 | 1480,1481,1482,1483,1488,1489 |
| Head and neck cancers - laryngeal | C320,C321,C322,C323,C328,C329,D020 | 1610,1611,1612,1613,1618,1619,2310 |
| Head and neck cancers - lip and oral cavity | C000,C001,C002,C003,C004,C005,C006,C008,C009,C01,C020,C021<br>C022,C023,C024,C028,C029,C030,C031,C039,C040,C041,C048,C049<br>C050,C051,C052,C058,C059,C060,C061,C062,C0680,C0689,C069<br>D0000,D0001,D0002,D0003,D0004,D0005,D0006,D0007 | 1400,1401,1403,1404,1405,1406,1408,<br>1409,1410,1411,1412,1413<br>1414,1416,1418,1419,1430,1431,1438,<br>1439,1440,1441,1448,1449<br>1450,1451,1452,1453,1454,1455,1456,<br>1458,1459,2300 |
| Head and neck cancers - nasopharyngeal | C110,C111,C112,C113,C118,C119 | 1470,1471,1472,1473,1478,1479 |
| Head and neck cancers - pharyngeal | C140,C142,D0008 | 1490,1491,2300 |

Continued on next page

Table S9 (continued).

| Diagnosis | ICD-10 | ICD-9 |
|---|---|---|
| Head and neck cancers - salivary gland | C07,C080,C081,C089 | 1420,1421,1422,1428,1429 |
| Head and neck cancers - throat | C100,C101,C102,C103,C104,C108,C109 | 1463,1464,1466,1467,1468,1469 |
| Head and neck cancers - tonsils | C090,C091,C098,C099 | 1460,1461,1462 |
| Headache; including migraine | G43001,G43009,G43011,G43019,G43101,G43109,G43111,G43119<br>G43401,G43409,G43411,G43419,G43501,G43509,G43511,G43519<br>G43601,G43609,G43611,G43619,G43701,G43709,G43711,G43719<br>G43801,G43809,G43811,G43819,G43821,G43829,G43831,G43839<br>G43901,G43909,G43911,G43919,G43A0,G43A1,G43B0,G43B1,G43C0<br>G43C1,G43D0,G43D1,G44001,G44009,G44011,G44019,G44021,G44029<br>G44031,G44039,G44041,G44049,G44051,G44059,G44091,G44099,G441<br>G44201,G44209,G44211,G44219,G44221,G44229,G44301,G44309<br>G44311,G44319,G44321,G44329,G4440,G4441,G4451,G4452,G4453<br>G4459,G4481,G4482,G4483,G4484,G4485,G4486,G4489,R51,R510,R519 | 30781,33900,33901,33902,33903,33904,<br>33905,33909,33910,33911<br>33912,33920,33921,33922,3393,33941,<br>33942,33943,33944,33981<br>33982,33983,33984,33985,33989,34600,<br>34601,34602,34603,34610<br>34611,34612,34613,34620,34621,34622,<br>34630,34631,34632,34633<br>34640,34641,34642,34643,34650,34651,<br>34652,34653,34660,34661<br>34662,34663,34670,34671,34672,34673,<br>34680,34681,34682,34683<br>34690,34691,34692,34693,7840 |
| Hearing loss | F804,H900,H9011,H9012,H902,H903,H9041,H9042,H905,H906,H9071<br>H9072,H908,H90A11,H90A12,H90A21,H90A22,H90A31,H90A32,H9101<br>H9102,H9103,H9109,H9110,H9111,H9112,H9113,H9120,H9121,H9122<br>H9123,H913,H918X1,H918X2,H918X3,H918X9,H9190,H9191,H9192<br>H9193,H93011,H93012,H93013,H93019,P096,Z45320,Z45321,Z45328 | 31534,38801,38802,38811,3882,38900,<br>38901,38902,38903,38904<br>38905,38906,38908,38910,38911,38912,<br>38913,38914,38915,38916<br>38917,38918,38920,38921,38922,3897,<br>3898,3899,V5309 |
| Heart failure | I0981,I110,I130,I132,I501,I5020,I5021,I5022,I5023,I5030,I5031<br>I5032,I5033,I5040,I5041,I5042,I5043,I50810,I50811,I50812<br>I50813,I50814,I5082,I5083,I5084,I5089,I509,I97130,I97131<br>O29121,O29122,O29123,O29129,Z95811,Z95812 | 39891,40201,40211,40291,40401,40403,<br>40411,40413,40491,40493<br>4280,4281,42820,42821,42822,42823,<br>42830,42831,42832,42833<br>42840,42841,42842,42843,4289,4294,<br>64680,64681,64683,9971<br>V4321,V4322 |
| Hematuria | N020,N021,N022,N023,N024,N025,N026,N027,N028,N029,N02A,N3001<br>N3011,N3021,N3031,N3041,N3081,N3091,R310,R311,R312,R3121<br>R3129,R319 | 5811,5812,5813,58189,5819,5950,5951,<br>5952,5953,5954,59581<br>59582,59589,5959,59970,59971,59972 |
| Hemolytic anemia | D550,D551,D552,D5521,D5529,D553,D558,D559,D560,D561,D562,D563<br>D564,D565,D568,D569,D580,D581,D582,D588,D589,D590,D591,D5910<br>D5911,D5912,D5913,D5919,D592,D593,D5930,D5931,D5932,D5939<br>D594,D595,D596,D598,D599 | 2820,2821,2822,2823,28240,28243,28244,<br>28245,28246,28247,28249<br>2827,2828,2829,2830,28310,28311,28319,<br>2832,2839 |
| Hemolytic jaundice and perinatal jaundice | P550,P551,P558,P559,P560,P5690,P5699,P570,P578,P579,P580,P581<br>P582,P583,P5841,P5842,P585,P588,P589,P590,P591,P5920,P5929<br>P593,P598,P599 | 7730,7731,7732,7733,7734,7740,7741,<br>7742,77430,77439,7744,7745<br>7746,7747 |
| Hemorrhage after first trimester | O26852,O26853,O4412,O4413,O4432,O4433,O4452,O4453,O45002<br>O45003,O45012,O45013,O45022,O45023,O45092,O45093,O458X2<br>O458X3,O4592,O4593,O46002,O46003,O46012,O46013,O46022,O46023<br>O46092,O46093,O468X2,O468X3,O4692,O4693,O670,O678,O679,O720<br>O721,O722 | 64111,64113,64121,64123,64131,64133,<br>64181,64183,64191,64193<br>64951,64953,66602,66604,66612,66614,<br>66622,66624 |
| Hemorrhagic and hematologic disorders of newborn | P500,P501,P502,P503,P504,P505,P508,P509,P510,P518,P519,P520<br>P521,P5221,P5222,P523,P524,P525,P526,P528,P529,P53,P540,P541<br>P542,P543,P544,P545,P546,P548,P549,P60,P610,P611,P612,P613<br>P614,P615,P616,P618,P619 | 7670,7720,77210,77211,77212,77213,<br>77214,7722,7723,7724,7725<br>7726,7728,7729,7760,7761,7762,7763,<br>7764,7765,7766,7767,7768<br>7769 |
| Hemorrhoids | K640,K641,K642,K643,K644,K645,K648,K649,O2240,O2241,O2242<br>O2243,O872 | 4550,4551,4552,4553,4554,4555,4556,<br>4557,4558,4559,67180,67181<br>67182,67183,67184 |
| Hepatic failure | K7040,K7041,K7200,K7201,K7210,K7211,K7290,K7291,K9182 | 570,5713,5722,5728,99749 |
| Hepatitis | A5145,B0081,B150,B159,B160,B161,B162,B169,B170,B1710,B1711<br>B172,B178,B179,B180,B181,B182,B188,B189,B190,B1910,B1911 | 05471,0700,0701,07020,07021,07030,<br>07031,07032,07033,07041<br>07049,07051,07052,07053,07054,07059,<br>0706,07070,07071,0709 |

Continued on next page

Table S9 (continued).

| Diagnosis | ICD-10 | ICD-9 |
| --- | --- | --- |
| | B1920,B1921,B199,B251,B2681,B581,K7010,K7011,O98411,O98412 | 07271,0785,09162,1305,5711,5731,64760, 64761,64762,64763,64764 |
| | O98413,O98419,O9842,O9843,P353 | 7712 |
| Hodgkin lymphoma | C8100,C8101,C8102,C8103,C8104,C8105,C8106,C8107,C8108,C8109 | 20100,20101,20102,20103,20104,20105, 20106,20107,20108,20110 |
| | C8110,C8111,C8112,C8113,C8114,C8115,C8116,C8117,C8118,C8119 | 20111,20112,20113,20114,20115,20116, 20117,20118,20120,20121 |
| | C8120,C8121,C8122,C8123,C8124,C8125,C8126,C8127,C8128,C8129 | 20122,20123,20124,20125,20126,20127, 20128,20140,20141,20142 |
| | C8130,C8131,C8132,C8133,C8134,C8135,C8136,C8137,C8138,C8139 | 20143,20144,20145,20146,20147,20148, 20150,20151,20152,20153 |
| | C8140,C8141,C8142,C8143,C8144,C8145,C8146,C8147,C8148,C8149 | 20154,20155,20156,20157,20158,20160, 20161,20162,20163,20164 |
| | C8170,C8171,C8172,C8173,C8174,C8175,C8176,C8177,C8178,C8179 | 20165,20166,20167,20168,20170,20171, 20172,20173,20174,20175 |
| | C8190,C8191,C8192,C8193,C8194,C8195,C8196,C8197,C8198,C8199 | 20176,20177,20178,20190,20191,20192, 20193,20194,20195,20196 |
| | | 20197,20198 |
| Hyperplasia of prostate | N400,N401,N402,N403 | 60000,60001,60010,60011 |
| Hypertension and | O10011,O10012,O10013,O10019,O1002,O1003,O10111,O10112,O10113 | 64200,64201,64203,64204,64210,64211, 64213,64214,64220,64221 |
| hypertensive-related | O10119,O1012,O1013,O10211,O10212,O10213,O10219,O1022,O1023 | 64223,64224,64230,64231,64232,64233, 64234,64240,64241,64242 |
| conditions | O10311,O10312,O10313,O10319,O1032,O1033,O10411,O10412,O10413 | 64243,64244,64250,64251,64252,64253, 64254,64260,64261,64263 |
| complicating | O10419,O1042,O1043,O10911,O10912,O10913,O10919,O1092,O1093 | 64264,64270,64271,64272,64273,64274, 64290,64291,64293,64294 |
| pregnancy; childbirth; and the puerperium | O111,O112,O113,O114,O115,O119,O131,O132,O133,O134,O135,O139 O1400,O1402,O1403,O1404,O1405,O1410,O1412,O1413,O1414,O1415 O1420,O1422,O1423,O1424,O1425,O1490,O1492,O1493,O1494,O1495 O1500,O1502,O1503,O151,O152,O159,O161,O162,O163,O164,O165 O169 | |
| Hypertension with | H35031,H35032,H35033,I110,I119,I120,I129,I130,I1310,I1311 | 36211,40200,40201,40210,40211,40290, 40291,40300,40301,40310 |
| complications and | I132,I150,I151,I152,I158,I159,I160,I161,I169,I674,I973,O10111 | 40311,40390,40391,40400,40401,40402, 40403,40410,40411,40412 |
| secondary | O10112,O10113,O10119,O1012,O1013,O10211,O10212,O10213,O10219 | 40413,40490,40491,40492,40493,40501, 40509,40511,40519,40591 |
| hypertension | O1022,O1023,O10311,O10312,O10313,O10319,O1032,O1033,O10411 | 40599,4372,64210,64211,64213,64214, 64220,64221,64223,64224 |
| | O10412,O10413,O10419,O1042,O1043,O111,O112,O113,O114,O115 | 64230,64231,64232,64233,64234,64270, 64271,64273,99791 |
| | O119,O131,O132,O133,O134,O135,O139 | |
| Hypotension | I950,I951,I952,I953,I9581,I9589,I959,O2650,O2651,O2652,O2653 | 4580,4581,45821,45829,4588,4589,66920, 66921,66922,66923,66924 |
| Immune-mediated | A3984,M0210,M02111,M02112,M02119,M02121,M02122,M02129,M02131 | 03682,0993,71110,71111,71112,71113, 71114,71115,71116,71117 |
| reactive | M02132,M02139,M02141,M02142,M02149,M02151,M02152,M02159 | 71118,71119,71130,71131,71132,71133, 71134,71135,71136,71137 |
| arthropathies | M02161,M02162,M02169,M02171,M02172,M02179,M0218,M0219,M0230 | 71138,71139,71140,71141,71142,71143, 71144,71145,71146,71147 |
| | M02311,M02312,M02319,M02321,M02322,M02329,M02331,M02332 | 71148,71149,71150,71151,71152,71153, 71154,71155,71156,71157 |
| | M02339,M02341,M02342,M02349,M02351,M02352,M02359,M02361 | 71158,71159,71180,71181,71182,71183, 71184,71185,71186,71187 |
| | M02362,M02369,M02371,M02372,M02379,M0238,M0239,M0280,M02811 | 71188,71189,7131,7137,71680,71681, 71682,71683,71684,71685 |
| | M02812,M02819,M02821,M02822,M02829,M02831,M02832,M02839 M02841,M02842,M02849,M02851,M02852,M02859,M02861,M02862 M02869,M02871,M02872,M02879,M0288,M0289,M029,M0760,M07611 M07612,M07619,M07621,M07622,M07629,M07631,M07632,M07639 M07641,M07642,M07649,M07651,M07652,M07659,M07661,M07662 M07669,M07671,M07672,M07679,M0768,M0769 | 71686,71687,71688,71689 |
| Immunity disorders | D800,D801,D802,D803,D804,D805,D806,D807,D808,D809,D810,D811 | 135,2662,2730,2731,2732,2772,2776, 27900,27901,27902,27903 |
| | D812,D813,D8130,D8131,D8132,D8139,D814,D815,D816,D817,D81810 | 27904,27905,27906,27909,27910,27911, 27912,27913,27919,2792 |
| | D81818,D81819,D8182,D8189,D819,D820,D821,D822,D823,D824,D828 | 2793,27941,27950,27951,27952,27953, 2798,2799,28989 |
| | D829,D830,D831,D832,D838,D839,D840,D841,D848,D8481,D84821 D84822,D8489,D849,D860,D861,D862,D863,D8681,D8682,D8683,D8684 D8685,D8686,D8687,D8689,D869,D890,D891,D892,D893,D8940,D8941 D8942,D8943,D8944,D8949,D89810,D89811,D89812,D89813,D8982 D89831,D89832,D89833,D89834,D89835,D89839,D8989,D899 | |
| Implant, device or | Z44001,Z44002,Z44009,Z44011,Z44012,Z44019,Z44021,Z44022 | V410,V412,V422,V430,V431,V4321, V4322,V433,V434,V435,V4360 |
| graft related | Z44029,Z44101,Z44102,Z44109,Z44111,Z44112,Z44119,Z44121 | V4361,V4362,V4363,V4364,V4365, V4366,V4369,V437,V4381,V4382 |

Continued on next page

Table S9 (continued).

| Diagnosis | ICD-10 | ICD-9 |
|---|---|---|
| encounter | Z44122,Z44129,Z4420,Z4421,Z4422,Z4430,Z4431,Z4432,Z448,Z449 | V4383,V4389,V4500,V4501,V4502, V4509,V452,V453,V454,V4561 |
| | Z45010,Z45018,Z4502,Z4509,Z451,Z452,Z4531,Z45320,Z45321 | V4569,V4581,V4582,V4584,V4585, V4586,V4589,V4982,V520,V521 |
| | Z45328,Z4541,Z4542,Z4549,Z45811,Z45812,Z45819,Z4582,Z4589 | V522,V523,V524,V528,V529,V5301, V5302,V5309,V531,V532,V5331 |
| | Z459,Z460,Z461,Z462,Z463,Z464,Z4651,Z4659,Z466,Z4681,Z4682 | V5332,V5339,V534,V5350,V5351,V5359, V536,V537,V538,V5390,V5391 |
| | Z4689,Z469,Z950,Z951,Z952,Z953,Z954,Z955,Z95810,Z95811,Z95812 Z95818,Z95820,Z95828,Z959,Z960,Z961,Z9620,Z9621,Z9622,Z9629 Z963,Z9641,Z9649,Z965,Z9660,Z96611,Z96612,Z96619,Z96621 Z96622,Z96629,Z96631,Z96632,Z96639,Z96641,Z96642,Z96643 Z96649,Z96651,Z96652,Z96653,Z96659,Z96661,Z96662,Z96669 Z96691,Z96692,Z96693,Z96698,Z967,Z9681,Z9682,Z9689,Z969,Z970 Z9710,Z9711,Z9712,Z9713,Z9714,Z9715,Z9716,Z972,Z973,Z974,Z978 Z980,Z981,Z982,Z983,Z9841,Z9842,Z9849,Z9861,Z9862,Z98810 Z98811,Z98818,Z9882,Z9883,Z9884,Z9889,Z98890 | V5399,V585,V5881,V5882,V6546 |
| Induced abortion and | O045,O046,O047,O0480,O0481,O0482,O0483,O0484,O0485,O0486 | 63502,63512,63522,63532,63542,63552, 63562,63572,63582,63590 |
| complications of | O0487,O0488,O0489,O070,O071,O072,O0730,O0731,O0732,O0733 | 6380,6381,6382,6383,6384,6385,6386, 6387,6388,6389 |
| termination of pregnancy | O0734,O0735,O0736,O0737,O0738,O0739,O074,Z332 | |
| Infective arthritis | A0104,A0223,A1801,A1802,A1803,A3983,A5442,A6923,B0682,B2685 | 0020,00323,01500,01510,01520,01560, 01570,01580,01590,03682 |
| | B4282,M0000,M00011,M00012,M00019,M00021,M00022,M00029,M00031 | 0412,05671,07279,08881,09850,1171, 71100,71101,71102,71103 |
| | M00032,M00039,M00041,M00042,M00049,M00051,M00052,M00059 | 71104,71105,71106,71107,71108,71109, 71140,71141,71142,71143 |
| | M00061,M00062,M00069,M00071,M00072,M00079,M0008,M0009,M0010 | 71144,71145,71146,71147,71148,71149, 71150,71151,71152,71153 |
| | M00111,M00112,M00119,M00121,M00122,M00129,M00131,M00132 | 71154,71155,71156,71157,71158,71159, 71160,71161,71162,71163 |
| | M00139,M00141,M00142,M00149,M00151,M00152,M00159,M00161 | 71164,71165,71166,71167,71168,71169, 71170,71171,71172,71173 |
| | M00162,M00169,M00171,M00172,M00179,M0018,M0019,M0020,M00211 | 71174,71175,71176,71177,71178,71179, 71180,71181,71182,71183 |
| | M00212,M00219,M00221,M00222,M00229,M00231,M00232,M00239 | 71184,71185,71186,71187,71188,71189, 72081,73088,73740 |
| | M00241,M00242,M00249,M00251,M00252,M00259,M00261,M00262 M00269,M00271,M00272,M00279,M0028,M0029,M0080,M00811,M00812 M00819,M00821,M00822,M00829,M00831,M00832,M00839,M00841 M00842,M00849,M00851,M00852,M00859,M00861,M00862,M00869 M00871,M00872,M00879,M0088,M0089,M009,M01X0,M01X11,M01X12 M01X19,M01X21,M01X22,M01X29,M01X31,M01X32,M01X39,M01X41 M01X42,M01X49,M01X51,M01X52,M01X59,M01X61,M01X62,M01X69 M01X71,M01X72,M01X79,M01X8,M01X9 | |
| Inflammatory | A1814,A1815,A5422,A5423,A5902,A6001,A6002,B260,B3742,B3881 | 01650,05413,05419,0720,09812,09813, 09814,09832,09833,09834 |
| conditions of male | N410,N411,N412,N413,N414,N418,N419,N431,N451,N452,N453,N454 | 1122,1141,13103,6010,6011,6012,6013, 6014,6018,6019,6031,6040 |
| genital organs | N476,N481,N4821,N4822,N4829,N490,N491,N492,N493,N498,N499 | 60490,60499,6071,6072,6080,6084 |
| Inflammatory diseases | A1816,A1817,A1818,A5142,A5402,A5403,A5424,A5602,A5611,A5901 | 01660,01670,05411,05412,05419,09189, 0980,09815,09816,09835 |
| of female pelvic | A6003,A6004,B373,B3731,B3732,N7001,N7002,N7003,N7011,N7012 | 09836,09953,09954,1121,13101,6140,6141, 6142,6143,6144,6145 |
| organs | N7013,N7091,N7092,N7093,N710,N711,N719,N72,N730,N731,N732 | 6146,6147,6148,6149,6150,6151,6159, 6160,61610,61611,6162,6163 |
| | N733,N734,N735,N736,N738,N739,N74,N750,N751,N758,N759,N760 | 6164,61650,61651,61681,61689,6169, 64660,64661,64663,64664 |
| | N761,N762,N763,N764,N765,N766,N7681,N7682,N7689,N770,N771 O23511,O23512,O23513,O23519,O23591,O23592,O23593,O23599,O8611 O8619 | |
| Influenza | J09X1,J09X2,J09X3,J09X9,J1000,J1001,J1008,J101,J102,J1081 | 4870,4871,4878,48801,48802,48809,48811, 48812,48819,48881 |
| | J1082,J1083,J1089,J1100,J1108,J111,J112,J1181,J1182,J1183 J1189 | 48882,48889 |
| Inhalant-related | F1810,F18120,F18121,F18129,F1814,F18150,F18151,F18159,F1817 | 29211,29212,2922,29281,29282,29284, 29289,2929,30460,30590 |
| disorders | F18180,F18188,F1819,F1820,F18220,F18221,F18229,F1824,F18250 | 30591,30592,30593,9682,99522,E8551, E9382,E9528,E9620,E9804 |
| | F18251,F18259,F1827,F18280,F18288,F1829,F1890,F1891,F18920 F18921,F18929,F1894,F18950,F18951,F18959,F1897,F18980,F18988 F1899,T410X1A,T410X2A,T410X3A,T410X4A,T410X5A | |
| Injury to blood | S090XXA,S15001A,S15002A,S15009A,S15011A,S15012A,S15019A | 90000,90001,90002,90003,9001,90081, 90082,90089,9009,9010,9011 |
| vessels, initial | S15021A,S15022A,S15029A,S15091A,S15092A,S15099A,S15101A | 9012,9013,90140,90141,90142,90181, 90182,90189,9019,9020,90210 |
| encounter | S15102A,S15109A,S15111A,S15112A,S15119A,S15121A,S15122A | 90211,90219,90220,90221,90222,90223, 90224,90225,90226,90227 |

Continued on next page

Table S9 (continued).

| Diagnosis | ICD-10 | ICD-9 |
|---|---|---|
| | S15129A,S15191A,S15192A,S15199A,S15201A,S15202A,S15209A | 90229,90231,90232,90233,90234,90240, 90241,90242,90249,90251 |
| | S15211A,S15212A,S15219A,S15221A,S15222A,S15229A,S15291A | 90252,90253,90254,90255,90256,90259, 90281,90282,90289,9029 |
| | S15292A,S15299A,S15301A,S15302A,S15309A,S15311A,S15312A | 90300,90301,90302,9031,9032,9033,9034, 9035,9038,9039,9040 |
| | S15319A,S15321A,S15322A,S15329A,S15391A,S15392A,S15399A | 9041,9042,9043,90441,90442,90450,90451, 90452,90453,90454,9046 |
| | S158XXA,S159XXA,S2500XA,S2501XA,S2502XA,S2509XA,S25101A | 9047,9048 |
| | S25102A,S25109A,S25111A,S25112A,S25119A,S25121A,S25122A | |
| | S25129A,S25191A,S25192A,S25199A,S2520XA,S2521XA,S2522XA | |
| | S2529XA,S25301A,S25302A,S25309A,S25311A,S25312A,S25319A | |
| | S25321A,S25322A,S25329A,S25391A,S25392A,S25399A,S25401A | |
| | S25402A,S25409A,S25411A,S25412A,S25419A,S25421A,S25422A | |
| | S25429A,S25491A,S25492A,S25499A,S25501A,S25502A,S25509A | |
| | S25511A,S25512A,S25519A,S25591A,S25592A,S25599A,S25801A | |
| | S25802A,S25809A,S25811A,S25812A,S25819A,S25891A,S25892A | |
| | S25899A,S2590XA,S2591XA,S2599XA,S3500XA,S3501XA,S3502XA | |
| | S3509XA,S3510XA,S3511XA,S3512XA,S3519XA,S35211A,S35212A | |
| | S35218A,S35219A,S35221A,S35222A,S35228A,S35229A,S35231A | |
| | S35232A,S35238A,S35239A,S35291A,S35292A,S35298A,S35299A | |
| | S35311A,S35318A,S35319A,S35321A,S35328A,S35329A,S35331A | |
| | S35338A,S35339A,S35341A,S35348A,S35349A,S35401A,S35402A | |
| | S35403A,S35404A,S35405A,S35406A,S35411A,S35412A,S35413A | |
| | S35414A,S35415A,S35416A,S35491A,S35492A,S35493A,S35494A | |
| | S35495A,S35496A,S3550XA,S35511A,S35512A,S35513A,S35514A | |
| | S35515A,S35516A,S35531A,S35532A,S35533A,S35534A,S35535A | |
| | S35536A,S3559XA,S358X1A,S358X8A,S358X9A,S3590XA,S3591XA | |
| | S3599XA,S45001A,S45002A,S45009A,S45011A,S45012A,S45019A | |
| | S45091A,S45092A,S45099A,S45101A,S45102A,S45109A,S45111A | |
| | S45112A,S45119A,S45191A,S45192A,S45199A,S45201A,S45202A | |
| | S45209A,S45211A,S45212A,S45219A,S45291A,S45292A,S45299A | |
| | S45301A,S45302A,S45309A,S45311A,S45312A,S45319A,S45391A | |
| | S45392A,S45399A,S45801A,S45802A,S45809A,S45811A,S45812A | |
| | S45819A,S45891A,S45892A,S45899A,S45901A,S45902A,S45909A | |
| | S45911A,S45912A,S45919A,S45991A,S45992A,S45999A,S55001A | |
| | S55002A,S55009A,S55011A,S55012A,S55019A,S55091A,S55092A | |
| | S55099A,S55101A,S55102A,S55109A,S55111A,S55112A,S55119A | |
| | S55191A,S55192A,S55199A,S55201A,S55202A,S55209A,S55211A | |
| | S55212A,S55219A,S55291A,S55292A,S55299A,S55801A,S55802A | |
| | S55809A,S55811A,S55812A,S55819A,S55891A,S55892A,S55899A | |
| | S55901A,S55902A,S55909A,S55911A,S55912A,S55919A,S55991A | |
| | S55992A,S55999A,S65001A,S65002A,S65009A,S65011A,S65012A | |
| | S65019A,S65091A,S65092A,S65099A,S65101A,S65102A,S65109A | |
| | S65111A,S65112A,S65119A,S65191A,S65192A,S65199A,S65201A | |
| | S65202A,S65209A,S65211A,S65212A,S65219A,S65291A,S65292A | |
| | S65299A,S65301A,S65302A,S65309A,S65311A,S65312A,S65319A | |
| | S65391A,S65392A,S65399A,S65401A,S65402A,S65409A,S65411A | |
| | S65412A,S65419A,S65491A,S65492A,S65499A,S65500A,S65501A | |
| | S65502A,S65503A,S65504A,S65505A,S65506A,S65507A,S65508A | |
| | S65509A,S65510A,S65511A,S65512A,S65513A,S65514A,S65515A | |
| | S65516A,S65517A,S65518A,S65519A,S65590A,S65591A,S65592A | |
| | S65593A,S65594A,S65595A,S65596A,S65597A,S65598A,S65599A | |
| | S65801A,S65802A,S65809A,S65811A,S65812A,S65819A,S65891A | |
| | S65892A,S65899A,S65901A,S65902A,S65909A,S65911A,S65912A | |
| | S65919A,S65991A,S65992A,S65999A,S75001A,S75002A,S75009A | |
| | S75011A,S75012A,S75019A,S75021A,S75022A,S75029A,S75091A | |
| | S75092A,S75099A,S75101A,S75102A,S75109A,S75111A,S75112A | |
| | S75119A,S75121A,S75122A,S75129A,S75191A,S75192A,S75199A | |
| | S75201A,S75202A,S75209A,S75211A,S75212A,S75219A,S75221A | |
| | S75222A,S75229A,S75291A,S75292A,S75299A,S75801A,S75802A | |
| | S75809A,S75811A,S75812A,S75819A,S75891A,S75892A,S75899A | |
| | S75901A,S75902A,S75909A,S75911A,S75912A,S75919A,S75991A | |
| | S75992A,S75999A,S85001A,S85002A,S85009A,S85011A,S85012A | |
| | S85019A,S85091A,S85092A,S85099A,S85101A,S85102A,S85109A | |
| | S85111A,S85112A,S85119A,S85121A,S85122A,S85129A,S85131A | |
| | S85132A,S85139A,S85141A,S85142A,S85149A,S85151A,S85152A | |
| | S85159A,S85161A,S85162A,S85169A,S85171A,S85172A,S85179A | |
| | S85181A,S85182A,S85189A,S85201A,S85202A,S85209A,S85211A | |
| | S85212A,S85219A,S85291A,S85292A,S85299A,S85301A,S85302A | |
| | S85309A,S85311A,S85312A,S85319A,S85391A,S85392A,S85399A | |
| | S85401A,S85402A,S85409A,S85411A,S85412A,S85419A,S85491A | |
| | S85492A,S85499A,S85501A,S85502A,S85509A,S85511A,S85512A | |
| | S85519A,S85591A,S85592A,S85599A,S85801A,S85802A,S85809A | |
| | S85811A,S85812A,S85819A,S85891A,S85892A,S85899A,S85901A | |
| | S85902A,S85909A,S85911A,S85912A,S85919A,S85991A,S85992A | |
| | S85999A,S95001A,S95002A,S95009A,S95011A,S95012A,S95019A | |
| | S95091A,S95092A,S95099A,S95101A,S95102A,S95109A,S95111A | |
| | S95112A,S95119A,S95191A,S95192A,S95199A,S95201A,S95202A | |
| | S95209A,S95211A,S95212A,S95219A,S95291A,S95292A,S95299A | |
| | S95801A,S95802A,S95809A,S95811A,S95812A,S95819A,S95891A | |
| | S95892A,S95899A,S95901A,S95902A,S95909A,S95911A,S95912A | |
| | S95919A,S95991A,S95992A,S95999A | |
| Injury to blood | S090XXD,S15001D,S15002D,S15009D,S15011D,S15012D,S15019D | V5889 |

Continued on next page

Table S9 (continued).

| Diagnosis | ICD-10 | ICD-9 |
|---|---|---|
| vessels, subsequent encounter | S15021D,S15022D,S15029D,S15091D,S15092D,S15099D,S15101D<br>S15102D,S15109D,S15111D,S15112D,S15119D,S15121D,S15122D<br>S15129D,S15191D,S15192D,S15199D,S15201D,S15202D,S15209D<br>S15211D,S15212D,S15219D,S15221D,S15222D,S15229D,S15291D<br>S15292D,S15299D,S15301D,S15302D,S15309D,S15311D,S15312D<br>S15319D,S15321D,S15322D,S15329D,S15391D,S15392D,S15399D<br>S158XXD,S159XXD,S2500XD,S2501XD,S2502XD,S2509XD,S25101D<br>S25102D,S25109D,S25111D,S25112D,S25119D,S25121D,S25122D<br>S25129D,S25191D,S25192D,S25199D,S2520XD,S2521XD,S2522XD<br>S2529XD,S25301D,S25302D,S25309D,S25311D,S25312D,S25319D<br>S25321D,S25322D,S25329D,S25391D,S25392D,S25399D,S25401D<br>S25402D,S25409D,S25411D,S25412D,S25419D,S25421D,S25422D<br>S25429D,S25491D,S25492D,S25499D,S25501D,S25502D,S25509D<br>S25511D,S25512D,S25519D,S25591D,S25592D,S25599D,S25801D<br>S25802D,S25809D,S25811D,S25812D,S25819D,S25891D,S25892D<br>S25899D,S2590XD,S2591XD,S2599XD,S3500XD,S3501XD,S3502XD<br>S3509XD,S3510XD,S3511XD,S3512XD,S3519XD,S35211D,S35212D<br>S35218D,S35219D,S35221D,S35222D,S35228D,S35229D,S35231D<br>S35232D,S35238D,S35239D,S35291D,S35292D,S35298D,S35299D<br>S35311D,S35318D,S35319D,S35321D,S35328D,S35329D,S35331D<br>S35338D,S35339D,S35341D,S35348D,S35349D,S35401D,S35402D<br>S35403D,S35404D,S35405D,S35406D,S35411D,S35412D,S35413D<br>S35414D,S35415D,S35416D,S35491D,S35492D,S35493D,S35494D<br>S35495D,S35496D,S3550XD,S35511D,S35512D,S35513D,S35514D<br>S35515D,S35516D,S35531D,S35532D,S35533D,S35534D,S35535D<br>S35536D,S3559XD,S358X1D,S358X8D,S358X9D,S3590XD,S3591XD<br>S3599XD,S45001D,S45002D,S45009D,S45011D,S45012D,S45019D<br>S45091D,S45092D,S45099D,S45101D,S45102D,S45109D,S45111D<br>S45112D,S45119D,S45191D,S45192D,S45199D,S45201D,S45202D<br>S45209D,S45211D,S45212D,S45219D,S45291D,S45292D,S45299D<br>S45301D,S45302D,S45309D,S45311D,S45312D,S45319D,S45391D<br>S45392D,S45399D,S45801D,S45802D,S45809D,S45811D,S45812D<br>S45819D,S45891D,S45892D,S45899D,S45901D,S45902D,S45909D<br>S45911D,S45912D,S45919D,S45991D,S45992D,S45999D,S55001D<br>S55002D,S55009D,S55011D,S55012D,S55019D,S55091D,S55092D<br>S55099D,S55101D,S55102D,S55109D,S55111D,S55112D,S55119D<br>S55191D,S55192D,S55199D,S55201D,S55202D,S55209D,S55211D<br>S55212D,S55219D,S55291D,S55292D,S55299D,S55801D,S55802D<br>S55809D,S55811D,S55812D,S55819D,S55891D,S55892D,S55899D<br>S55901D,S55902D,S55909D,S55911D,S55912D,S55919D,S55991D<br>S55992D,S55999D,S65001D,S65002D,S65009D,S65011D,S65012D<br>S65019D,S65091D,S65092D,S65099D,S65101D,S65102D,S65109D<br>S65111D,S65112D,S65119D,S65191D,S65192D,S65199D,S65201D<br>S65202D,S65209D,S65211D,S65212D,S65219D,S65291D,S65292D<br>S65299D,S65301D,S65302D,S65309D,S65311D,S65312D,S65319D<br>S65391D,S65392D,S65399D,S65401D,S65402D,S65409D,S65411D<br>S65412D,S65419D,S65491D,S65492D,S65499D,S65500D,S65501D<br>S65502D,S65503D,S65504D,S65505D,S65506D,S65507D,S65508D<br>S65509D,S65510D,S65511D,S65512D,S65513D,S65514D,S65515D<br>S65516D,S65517D,S65518D,S65519D,S65590D,S65591D,S65592D<br>S65593D,S65594D,S65595D,S65596D,S65597D,S65598D,S65599D<br>S65801D,S65802D,S65809D,S65811D,S65812D,S65819D,S65891D<br>S65892D,S65899D,S65901D,S65902D,S65909D,S65911D,S65912D<br>S65919D,S65991D,S65992D,S65999D,S75001D,S75002D,S75009D<br>S75011D,S75012D,S75019D,S75021D,S75022D,S75029D,S75091D<br>S75092D,S75099D,S75101D,S75102D,S75109D,S75111D,S75112D<br>S75119D,S75121D,S75122D,S75129D,S75191D,S75192D,S75199D<br>S75201D,S75202D,S75209D,S75211D,S75212D,S75219D,S75221D<br>S75222D,S75229D,S75291D,S75292D,S75299D,S75801D,S75802D<br>S75809D,S75811D,S75812D,S75819D,S75891D,S75892D,S75899D<br>S75901D,S75902D,S75909D,S75911D,S75912D,S75919D,S75991D<br>S75992D,S75999D,S85001D,S85002D,S85009D,S85011D,S85012D<br>S85019D,S85091D,S85092D,S85099D,S85101D,S85102D,S85109D<br>S85111D,S85112D,S85119D,S85121D,S85122D,S85129D,S85131D<br>S85132D,S85139D,S85141D,S85142D,S85149D,S85151D,S85152D<br>S85159D,S85161D,S85162D,S85169D,S85171D,S85172D,S85179D<br>S85181D,S85182D,S85189D,S85201D,S85202D,S85209D,S85211D<br>S85212D,S85219D,S85291D,S85292D,S85299D,S85301D,S85302D<br>S85309D,S85311D,S85312D,S85319D,S85391D,S85392D,S85399D<br>S85401D,S85402D,S85409D,S85411D,S85412D,S85419D,S85491D<br>S85492D,S85499D,S85501D,S85502D,S85509D,S85511D,S85512D<br>S85519D,S85591D,S85592D,S85599D,S85801D,S85802D,S85809D<br>S85811D,S85812D,S85819D,S85891D,S85892D,S85899D,S85901D<br>S85902D,S85909D,S85911D,S85912D,S85919D,S85991D,S85992D<br>S85999D,S95001D,S95002D,S95009D,S95011D,S95012D,S95019D<br>S95091D,S95092D,S95099D,S95101D,S95102D,S95109D,S95111D<br>S95112D,S95119D,S95191D,S95192D,S95199D,S95201D,S95202D<br>S95209D,S95211D,S95212D,S95219D,S95291D,S95292D,S95299D<br>S95801D,S95802D,S95809D,S95811D,S95812D,S95819D,S95891D<br>S95892D,S95899D,S95901D,S95902D,S95909D,S95911D,S95912D<br>S95919D,S95991D,S95992D,S95999D | |
| Injury to nerves, | S04011A,S04012A,S04019A,S0402XA,S04031A,S04032A,S04039A | 9500,9501,9502,9503,9510,9511,9512,<br>9513,9514,9515,9516,9517 |

Continued on next page

Table S9 (continued).

| Diagnosis | ICD-10 | ICD-9 |
|---|---|---|
| muscles and tendons, | S04041A,S04042A,S04049A,S0410XA,S0411XA,S0412XA,S0420XA | 9518,9519,9530,9531,9532,9533,9534,9535,9540,9541,9548,9549 |
| initial encounter | S0421XA,S0422XA,S0430XA,S0431XA,S0432XA,S0440XA,S0441XA | 9550,9551,9552,9553,9554,9555,9556,9557,9558,9559,9560,9561 |
| | S0442XA,S0450XA,S0451XA,S0452XA,S0460XA,S0461XA,S0462XA | 9562,9563,9564,9565,9568,9569,9570,9571,9578,9579,95901,95909 |
| | S0470XA,S0471XA,S0472XA,S04811A,S04812A,S04819A,S04891A | 95911,95912,95919,9592,9593,9594,9596,9597 |
| | S04892A,S04899A,S049XXA,S0910XA,S0919XA,S142XXA,S143XXA | |
| | S144XXA,S145XXA,S148XXA,S149XXA,S168XXA,S169XXA,S242XXA | |
| | S243XXA,S244XXA,S248XXA,S249XXA,S29001A,S29002A,S29009A | |
| | S29091A,S29092A,S29099A,S3421XA,S3422XA,S344XXA,S345XXA | |
| | S346XXA,S348XXA,S349XXA,S39001A,S39002A,S39003A,S39091A | |
| | S39092A,S39093A,S4400XA,S4401XA,S4402XA,S4410XA,S4411XA | |
| | S4412XA,S4420XA,S4421XA,S4422XA,S4430XA,S4431XA,S4432XA | |
| | S4440XA,S4441XA,S4442XA,S4450XA,S4451XA,S4452XA,S448X1A | |
| | S448X2A,S448X9A,S4490XA,S4491XA,S4492XA,S46001A,S46002A | |
| | S46009A,S46091A,S46092A,S46099A,S46101A,S46102A,S46109A | |
| | S46191A,S46192A,S46199A,S46201A,S46202A,S46209A,S46291A | |
| | S46292A,S46299A,S46301A,S46302A,S46309A,S46391A,S46392A | |
| | S46399A,S46801A,S46802A,S46809A,S46891A,S46892A,S46899A | |
| | S46901A,S46902A,S46909A,S46991A,S46992A,S46999A,S5400XA | |
| | S5401XA,S5402XA,S5410XA,S5411XA,S5412XA,S5420XA,S5421XA | |
| | S5422XA,S5430XA,S5431XA,S5432XA,S548X1A,S548X2A,S548X9A | |
| | S5490XA,S5491XA,S5492XA,S56001A,S56002A,S56009A,S56091A | |
| | S56092A,S56099A,S56101A,S56102A,S56103A,S56104A,S56105A | |
| | S56106A,S56107A,S56108A,S56109A,S56191A,S56192A,S56193A | |
| | S56194A,S56195A,S56196A,S56197A,S56198A,S56199A,S56201A | |
| | S56202A,S56209A,S56291A,S56292A,S56299A,S56301A,S56302A | |
| | S56309A,S56391A,S56392A,S56399A,S56401A,S56402A,S56403A | |
| | S56404A,S56405A,S56406A,S56407A,S56408A,S56409A,S56491A | |
| | S56492A,S56493A,S56494A,S56495A,S56496A,S56497A,S56498A | |
| | S56499A,S56501A,S56502A,S56509A,S56591A,S56592A,S56599A | |
| | S56801A,S56802A,S56809A,S56891A,S56892A,S56899A,S56901A | |
| | S56902A,S56909A,S56991A,S56992A,S56999A,S6400XA,S6401XA | |
| | S6402XA,S6410XA,S6411XA,S6412XA,S6420XA,S6421XA,S6422XA | |
| | S6430XA,S6431XA,S6432XA,S6440XA,S64490A,S64491A,S64492A | |
| | S64493A,S64494A,S64495A,S64496A,S64497A,S64498A,S648X1A | |
| | S648X2A,S648X9A,S6490XA,S6491XA,S6492XA,S66001A,S66002A | |
| | S66009A,S66091A,S66092A,S66099A,S66100A,S66101A,S66102A | |
| | S66103A,S66104A,S66105A,S66106A,S66107A,S66108A,S66109A | |
| | S66190A,S66191A,S66192A,S66193A,S66194A,S66195A,S66196A | |
| | S66197A,S66198A,S66199A,S66201A,S66202A,S66209A,S66291A | |
| | S66292A,S66299A,S66300A,S66301A,S66302A,S66303A,S66304A | |
| | S66305A,S66306A,S66307A,S66308A,S66309A,S66390A,S66391A | |
| | S66392A,S66393A,S66394A,S66395A,S66396A,S66397A,S66398A | |
| | S66399A,S66401A,S66402A,S66409A,S66491A,S66492A,S66499A | |
| | S66500A,S66501A,S66502A,S66503A,S66504A,S66505A,S66506A | |
| | S66507A,S66508A,S66509A,S66590A,S66591A,S66592A,S66593A | |
| | S66594A,S66595A,S66596A,S66597A,S66598A,S66599A,S66801A | |
| | S66802A,S66809A,S66891A,S66892A,S66899A,S66901A,S66902A | |
| | S66909A,S66991A,S66992A,S66999A,S7400XA,S7401XA,S7402XA | |
| | S7410XA,S7411XA,S7412XA,S7420XA,S7421XA,S7422XA,S748X1A | |
| | S748X2A,S748X9A,S7490XA,S7491XA,S7492XA,S76001A,S76002A | |
| | S76009A,S76091A,S76092A,S76099A,S76101A,S76102A,S76109A | |
| | S76191A,S76192A,S76199A,S76201A,S76202A,S76209A,S76291A | |
| | S76292A,S76299A,S76301A,S76302A,S76309A,S76391A,S76392A | |
| | S76399A,S76801A,S76802A,S76809A,S76891A,S76892A,S76899A | |
| | S76901A,S76902A,S76909A,S76991A,S76992A,S76999A,S8400XA | |
| | S8401XA,S8402XA,S8410XA,S8411XA,S8412XA,S8420XA,S8421XA | |
| | S8422XA,S84801A,S84802A,S84809A,S8490XA,S8491XA,S8492XA | |
| | S86001A,S86002A,S86009A,S86091A,S86092A,S86099A,S86101A | |
| | S86102A,S86109A,S86191A,S86192A,S86199A,S86201A,S86202A | |
| | S86209A,S86291A,S86292A,S86299A,S86301A,S86302A,S86309A | |
| | S86391A,S86392A,S86399A,S86801A,S86802A,S86809A,S86891A | |
| | S86892A,S86899A,S86901A,S86902A,S86909A,S86991A,S86992A | |
| | S86999A,S9400XA,S9401XA,S9402XA,S9410XA,S9411XA,S9412XA | |
| | S9420XA,S9421XA,S9422XA,S9430XA,S9431XA,S9432XA,S948X1A | |
| | S948X2A,S948X9A,S9490XA,S9491XA,S9492XA,S96001A,S96002A | |
| | S96009A,S96091A,S96092A,S96099A,S96101A,S96102A,S96109A | |
| | S96191A,S96192A,S96199A,S96201A,S96202A,S96209A,S96291A | |
| | S96292A,S96299A,S96801A,S96802A,S96809A,S96891A,S96892A | |
| | S96899A,S96901A,S96902A,S96909A,S96991A,S96992A,S96999A | |
| Injury to nerves, muscles and tendons, subsequent encounter | S04011D,S04012D,S04019D,S0402XD,S04031D,S04032D,S04039D | V5889 |
| | S04041D,S04042D,S04049D,S0410XD,S0411XD,S0412XD,S0420XD | |
| | S0421XD,S0422XD,S0430XD,S0431XD,S0432XD,S0440XD,S0441XD | |
| | S0442XD,S0450XD,S0451XD,S0452XD,S0460XD,S0461XD,S0462XD | |
| | S0470XD,S0471XD,S0472XD,S04811D,S04812D,S04819D,S04891D | |
| | S04892D,S04899D,S049XXD,S0910XD,S0919XD,S142XXD,S143XXD | |
| | S144XXD,S145XXD,S148XXD,S149XXD,S168XXD,S169XXD,S242XXD | |
| | S243XXD,S244XXD,S248XXD,S249XXD,S29001D,S29002D,S29009D | |
| | S29091D,S29092D,S29099D,S3421XD,S3422XD,S344XXD,S345XXD | |
| | S346XXD,S348XXD,S349XXD,S39001D,S39002D,S39003D,S39091D | |
| | S39092D,S39093D,S4400XD,S4401XD,S4402XD,S4410XD,S4411XD | |

Continued on next page

Table S9 (continued).

| Diagnosis | ICD-10 | ICD-9 |
|---|---|---|
| | S4412XD,S4420XD,S4421XD,S4422XD,S4430XD,S4431XD,S4432XD | |
| | S4440XD,S4441XD,S4442XD,S4450XD,S4451XD,S4452XD,S448X1D | |
| | S448X2D,S448X9D,S4490XD,S4491XD,S4492XD,S46001D,S46002D | |
| | S46009D,S46091D,S46092D,S46099D,S46101D,S46102D,S46109D | |
| | S46191D,S46192D,S46199D,S46201D,S46202D,S46209D,S46291D | |
| | S46292D,S46299D,S46301D,S46302D,S46309D,S46391D,S46392D | |
| | S46399D,S46801D,S46802D,S46809D,S46891D,S46892D,S46899D | |
| | S46901D,S46902D,S46909D,S46991D,S46992D,S46999D,S5400XD | |
| | S5401XD,S5402XD,S5410XD,S5411XD,S5412XD,S5420XD,S5421XD | |
| | S5422XD,S5430XD,S5431XD,S5432XD,S548X1D,S548X2D,S548X9D | |
| | S5490XD,S5491XD,S5492XD,S56001D,S56002D,S56009D,S56091D | |
| | S56092D,S56099D,S56101D,S56102D,S56103D,S56104D,S56105D | |
| | S56106D,S56107D,S56108D,S56109D,S56191D,S56192D,S56193D | |
| | S56194D,S56195D,S56196D,S56197D,S56198D,S56199D,S56201D | |
| | S56202D,S56209D,S56291D,S56292D,S56299D,S56301D,S56302D | |
| | S56309D,S56391D,S56392D,S56399D,S56401D,S56402D,S56403D | |
| | S56404D,S56405D,S56406D,S56407D,S56408D,S56409D,S56491D | |
| | S56492D,S56493D,S56494D,S56495D,S56496D,S56497D,S56498D | |
| | S56499D,S56501D,S56502D,S56509D,S56591D,S56592D,S56599D | |
| | S56801D,S56802D,S56809D,S56891D,S56892D,S56899D,S56901D | |
| | S56902D,S56909D,S56991D,S56992D,S56999D,S6400XD,S6401XD | |
| | S6402XD,S6410XD,S6411XD,S6412XD,S6420XD,S6421XD,S6422XD | |
| | S6430XD,S6431XD,S6432XD,S6440XD,S64490D,S64491D,S64492D | |
| | S64493D,S64494D,S64495D,S64496D,S64497D,S64498D,S648X1D | |
| | S648X2D,S648X9D,S6490XD,S6491XD,S6492XD,S66001D,S66002D | |
| | S66009D,S66091D,S66092D,S66099D,S66100D,S66101D,S66102D | |
| | S66103D,S66104D,S66105D,S66106D,S66107D,S66108D,S66109D | |
| | S66190D,S66191D,S66192D,S66193D,S66194D,S66195D,S66196D | |
| | S66197D,S66198D,S66199D,S66201D,S66202D,S66209D,S66291D | |
| | S66292D,S66299D,S66300D,S66301D,S66302D,S66303D,S66304D | |
| | S66305D,S66306D,S66307D,S66308D,S66309D,S66390D,S66391D | |
| | S66392D,S66393D,S66394D,S66395D,S66396D,S66397D,S66398D | |
| | S66399D,S66401D,S66402D,S66409D,S66491D,S66492D,S66499D | |
| | S66500D,S66501D,S66502D,S66503D,S66504D,S66505D,S66506D | |
| | S66507D,S66508D,S66509D,S66590D,S66591D,S66592D,S66593D | |
| | S66594D,S66595D,S66596D,S66597D,S66598D,S66599D,S66801D | |
| | S66802D,S66809D,S66891D,S66892D,S66899D,S66901D,S66902D | |
| | S66909D,S66991D,S66992D,S66999D,S7400XD,S7401XD,S7402XD | |
| | S7410XD,S7411XD,S7412XD,S7420XD,S7421XD,S7422XD,S748X1D | |
| | S748X2D,S748X9D,S7490XD,S7491XD,S7492XD,S76001D,S76002D | |
| | S76009D,S76091D,S76092D,S76099D,S76101D,S76102D,S76109D | |
| | S76191D,S76192D,S76199D,S76201D,S76202D,S76209D,S76291D | |
| | S76292D,S76299D,S76301D,S76302D,S76309D,S76391D,S76392D | |
| | S76399D,S76801D,S76802D,S76809D,S76891D,S76892D,S76899D | |
| | S76901D,S76902D,S76909D,S76991D,S76992D,S76999D,S8400XD | |
| | S8401XD,S8402XD,S8410XD,S8411XD,S8412XD,S8420XD,S8421XD | |
| | S8422XD,S84801D,S84802D,S84809D,S8490XD,S8491XD,S8492XD | |
| | S86001D,S86002D,S86009D,S86091D,S86092D,S86099D,S86101D | |
| | S86102D,S86109D,S86191D,S86192D,S86199D,S86201D,S86202D | |
| | S86209D,S86291D,S86292D,S86299D,S86301D,S86302D,S86309D | |
| | S86391D,S86392D,S86399D,S86801D,S86802D,S86809D,S86891D | |
| | S86892D,S86899D,S86901D,S86902D,S86909D,S86991D,S86992D | |
| | S86999D,S9400XD,S9401XD,S9402XD,S9410XD,S9411XD,S9412XD | |
| | S9420XD,S9421XD,S9422XD,S9430XD,S9431XD,S9432XD,S948X1D | |
| | S948X2D,S948X9D,S9490XD,S9491XD,S9492XD,S96001D,S96002D | |
| | S96009D,S96091D,S96092D,S96099D,S96101D,S96102D,S96109D | |
| | S96191D,S96192D,S96199D,S96201D,S96202D,S96209D,S96291D | |
| | S96292D,S96299D,S96801D,S96802D,S96809D,S96891D,S96892D | |
| | S96899D,S96901D,S96902D,S96909D,S96991D,S96992D,S96999D | |
| Injury, sequela | M9701XS,M9702XS,M9711XS,M9712XS,M9721XS,M9722XS,M9731XS | 9050,9051,9052,9053,9054,9056,9057,<br>9059,9060,9061,9062,9063 |
| | M9732XS,M9741XS,M9742XS,M978XXS,M979XXS,S0000XS,S0001XS | 9064,9065,9066,9067,9068,9070,9071,<br>9072,9073,9074,9075,9080 |
| | S0002XS,S0003XS,S0004XS,S0005XS,S0006XS,S0007XS,S0010XS | 9081,9082,9083,9084,9085,9086,9089,<br>9093,9099 |
| | S0011XS,S0012XS,S00201S,S00202S,S00209S,S00211S,S00212S | |
| | S00219S,S00221S,S00222S,S00229S,S00241S,S00242S,S00249S | |
| | S00251S,S00252S,S00259S,S00261S,S00262S,S00269S,S00271S | |
| | S00272S,S00279S,S0030XS,S0031XS,S0032XS,S0033XS,S0034XS | |
| | S0035XS,S0036XS,S0037XS,S00401S,S00402S,S00409S,S00411S | |
| | S00412S,S00419S,S00421S,S00422S,S00429S,S00431S,S00432S | |
| | S00439S,S00441S,S00442S,S00449S,S00451S,S00452S,S00459S | |
| | S00461S,S00462S,S00469S,S00471S,S00472S,S00479S,S00501S | |
| | S00502S,S00511S,S00512S,S00521S,S00522S,S00531S,S00532S | |
| | S00541S,S00542S,S00551S,S00552S,S00561S,S00562S,S00571S | |
| | S00572S,S0080XS,S0081XS,S0082XS,S0083XS,S0084XS,S0085XS | |
| | S0086XS,S0087XS,S0090XS,S0091XS,S0092XS,S0093XS,S0094XS | |
| | S0095XS,S0096XS,S0097XS,S0100XS,S0101XS,S0102XS,S0103XS | |
| | S0104XS,S0105XS,S01101S,S01102S,S01109S,S01111S,S01112S | |
| | S01119S,S01121S,S01122S,S01129S,S01131S,S01132S,S01139S | |
| | S01141S,S01142S,S01149S,S01151S,S01152S,S01159S,S0120XS | |
| | S0121XS,S0122XS,S0123XS,S0124XS,S0125XS,S01301S,S01302S | |
| | S01309S,S01311S,S01312S,S01319S,S01321S,S01322S,S01329S | |
| | S01331S,S01332S,S01339S,S01341S,S01342S,S01349S,S01351S | |

Continued on next page

Table S9 (continued).

| Diagnosis | ICD-10 | ICD-9 |
|---|---|---|
| | S01352S,S01359S,S01401S,S01402S,S01409S,S01411S,S01412S | |
| | S01419S,S01421S,S01422S,S01429S,S01431S,S01432S,S01439S | |
| | S01441S,S01442S,S01449S,S01451S,S01452S,S01459S,S01501S | |
| | S01502S,S01511S,S01512S,S01521S,S01522S,S01531S,S01532S | |
| | S01541S,S01542S,S01551S,S01552S,S0180XS,S0181XS,S0182XS | |
| | S0183XS,S0184XS,S0185XS,S0190XS,S0191XS,S0192XS,S0193XS | |
| | S0194XS,S0195XS,S020XXS,S02101S,S02102S,S02109S,S0210XS | |
| | S02110S,S02111S,S02112S,S02113S,S02118S,S02119S,S0211AS | |
| | S0211BS,S0211CS,S0211DS,S0211ES,S0211FS,S0211GS,S0211HS | |
| | S02121S,S02122S,S02129S,S0219XS,S022XXS,S0230XS,S0231XS | |
| | S0232XS,S023XXS,S02400S,S02401S,S02402S,S0240AS,S0240BS | |
| | S0240CS,S0240DS,S0240ES,S0240FS,S02411S,S02412S,S02413S | |
| | S0242XS,S025XXS,S02600S,S02601S,S02602S,S02609S,S02610S | |
| | S02611S,S02612S,S0261XS,S02620S,S02621S,S02622S,S0262XS | |
| | S02630S,S02631S,S02632S,S0263XS,S02640S,S02641S,S02642S | |
| | S0264XS,S02650S,S02651S,S02652S,S0265XS,S0266XS,S02670S | |
| | S02671S,S02672S,S0267XS,S0269XS,S0280XS,S0281XS,S0282XS | |
| | S02831S,S02832S,S02839S,S02841S,S02842S,S02849S,S0285XS | |
| | S028XXS,S0291XS,S0292XS,S0300XS,S0301XS,S0302XS,S0303XS | |
| | S030XXS,S031XXS,S032XXS,S0340XS,S0341XS,S0342XS,S0343XS | |
| | S034XXS,S038XXS,S039XXS,S04011S,S04012S,S04019S,S0402XS | |
| | S04031S,S04032S,S04039S,S04041S,S04042S,S04049S,S0410XS | |
| | S0411XS,S0412XS,S0420XS,S0421XS,S0422XS,S0430XS,S0431XS | |
| | S0432XS,S0440XS,S0441XS,S0442XS,S0450XS,S0451XS,S0452XS | |
| | S0460XS,S0461XS,S0462XS,S0470XS,S0471XS,S0472XS,S04811S | |
| | S04812S,S04819S,S04891S,S04892S,S04899S,S049XXS,S0500XS | |
| | S0501XS,S0502XS,S0510XS,S0511XS,S0512XS,S0520XS,S0521XS | |
| | S0522XS,S0530XS,S0531XS,S0532XS,S0540XS,S0541XS,S0542XS | |
| | S0550XS,S0551XS,S0552XS,S0560XS,S0561XS,S0562XS,S0570XS | |
| | S0571XS,S0572XS,S058X1S,S058X2S,S058X9S,S0590XS,S0591XS | |
| | S0592XS,S060X0S,S060X1S,S060X2S,S060X3S,S060X4S,S060X5S | |
| | S060X6S,S060X7S,S060X8S,S060X9S,S060XAS,S061X0S,S061X1S | |
| | S061X2S,S061X3S,S061X4S,S061X5S,S061X6S,S061X7S,S061X8S | |
| | S061X9S,S061XAS,S062X0S,S062X1S,S062X2S,S062X3S,S062X4S | |
| | S062X5S,S062X6S,S062X7S,S062X8S,S062X9S,S062XAS,S06300S | |
| | S06301S,S06302S,S06303S,S06304S,S06305S,S06306S,S06307S | |
| | S06308S,S06309S,S0630AS,S06310S,S06311S,S06312S,S06313S | |
| | S06314S,S06315S,S06316S,S06317S,S06318S,S06319S,S0631AS | |
| | S06320S,S06321S,S06322S,S06323S,S06324S,S06325S,S06326S | |
| | S06327S,S06328S,S06329S,S0632AS,S06330S,S06331S,S06332S | |
| | S06333S,S06334S,S06335S,S06336S,S06337S,S06338S,S06339S | |
| | S0633AS,S06340S,S06341S,S06342S,S06343S,S06344S,S06345S | |
| | S06346S,S06347S,S06348S,S06349S,S0634AS,S06350S,S06351S | |
| | S06352S,S06353S,S06354S,S06355S,S06356S,S06357S,S06358S | |
| | S06359S,S0635AS,S06360S,S06361S,S06362S,S06363S,S06364S | |
| | S06365S,S06366S,S06367S,S06368S,S06369S,S0636AS,S06370S | |
| | S06371S,S06372S,S06373S,S06374S,S06375S,S06376S,S06377S | |
| | S06378S,S06379S,S0637AS,S06380S,S06381S,S06382S,S06383S | |
| | S06384S,S06385S,S06386S,S06387S,S06388S,S06389S,S0638AS | |
| | S064X0S,S064X1S,S064X2S,S064X3S,S064X4S,S064X5S,S064X6S | |
| | S064X7S,S064X8S,S064X9S,S064XAS,S065X0S,S065X1S,S065X2S | |
| | S065X3S,S065X4S,S065X5S,S065X6S,S065X7S,S065X8S,S065X9S | |
| | S065XAS,S066X0S,S066X1S,S066X2S,S066X3S,S066X4S,S066X5S | |
| | S066X6S,S066X7S,S066X8S,S066X9S,S066XAS,S06810S,S06811S | |
| | S06812S,S06813S,S06814S,S06815S,S06816S,S06817S,S06818S | |
| | S06819S,S0681AS,S06820S,S06821S,S06822S,S06823S,S06824S | |
| | S06825S,S06826S,S06827S,S06828S,S06829S,S0682AS,S06890S | |
| | S06891S,S06892S,S06893S,S06894S,S06895S,S06896S,S06897S | |
| | S06898S,S06899S,S0689AS,S068A0S,S068A1S,S068A2S,S068A3S | |
| | S068A4S,S068A5S,S068A6S,S068A9S,S068AAS,S069X0S,S069X1S | |
| | S069X2S,S069X3S,S069X4S,S069X5S,S069X6S,S069X7S,S069X8S | |
| | S069X9S,S069XAS,S06A0XS,S06A1XS,S070XXS,S071XXS,S078XXS | |
| | S079XXS,S080XXS,S08111S,S08112S,S08119S,S08121S,S08122S | |
| | S08129S,S08811S,S08812S,S0889XS,S090XXS,S0910XS,S0911XS | |
| | S0912XS,S0919XS,S0920XS,S0921XS,S0922XS,S09301S,S09302S | |
| | S09309S,S09311S,S09312S,S09313S,S09319S,S09391S,S09392S | |
| | S09399S,S098XXS,S0990XS,S0991XS,S0992XS,S0993XS,S100XXS | |
| | S1010XS,S1011XS,S1012XS,S1014XS,S1015XS,S1016XS,S1017XS | |
| | S1080XS,S1081XS,S1082XS,S1083XS,S1084XS,S1085XS,S1086XS | |
| | S1087XS,S1090XS,S1091XS,S1092XS,S1093XS,S1094XS,S1095XS | |
| | S1096XS,S1097XS,S11011S,S11012S,S11013S,S11014S,S11015S | |
| | S11019S,S11021S,S11022S,S11023S,S11024S,S11025S,S11029S | |
| | S11031S,S11032S,S11033S,S11034S,S11035S,S11039S,S1110XS | |
| | S1111XS,S1112XS,S1113XS,S1114XS,S1115XS,S1120XS,S1121XS | |
| | S1122XS,S1123XS,S1124XS,S1125XS,S1180XS,S1181XS,S1182XS | |
| | S1183XS,S1184XS,S1185XS,S1189XS,S1190XS,S1191XS,S1192XS | |
| | S1193XS,S1194XS,S1195XS,S12000S,S12001S,S1201XS,S1202XS | |
| | S12030S,S12031S,S12040S,S12041S,S12090S,S12091S,S12100S | |
| | S12101S,S12110S,S12111S,S12112S,S12120S,S12121S,S12130S | |
| | S12131S,S1214XS,S12150S,S12151S,S12190S,S12191S,S12200S | |
| | S12201S,S12230S,S12231S,S1224XS,S12250S,S12251S,S12290S | |
| | S12291S,S12300S,S12301S,S12330S,S12331S,S1234XS,S12350S | |
| | S12351S,S12390S,S12391S,S12400S,S12401S,S12430S,S12431S | |
| | S1244XS,S12450S,S12451S,S12490S,S12491S,S12500S,S12501S | |
| | S12530S,S12531S,S1254XS,S12550S,S12551S,S12590S,S12591S | |

Continued on next page

Table S9 (continued).

| Diagnosis | ICD-10 | ICD-9 |
|---|---|---|
| | S12600S,S12601S,S12630S,S12631S,S1264XS,S12650S,S12651S | |
| | S12690S,S12691S,S128XXS,S129XXS,S130XXS,S13100S,S13101S | |
| | S13110S,S13111S,S13120S,S13121S,S13130S,S13131S,S13140S | |
| | S13141S,S13150S,S13151S,S13160S,S13161S,S13170S,S13171S | |
| | S13180S,S13181S,S1320XS,S1329XS,S134XXS,S135XXS,S138XXS | |
| | S139XXS,S140XXS,S14101S,S14102S,S14103S,S14104S,S14105S | |
| | S14106S,S14107S,S14108S,S14109S,S14111S,S14112S,S14113S | |
| | S14114S,S14115S,S14116S,S14117S,S14118S,S14119S,S14121S | |
| | S14122S,S14123S,S14124S,S14125S,S14126S,S14127S,S14128S | |
| | S14129S,S14131S,S14132S,S14133S,S14134S,S14135S,S14136S | |
| | S14137S,S14138S,S14139S,S14141S,S14142S,S14143S,S14144S | |
| | S14145S,S14146S,S14147S,S14148S,S14149S,S14151S,S14152S | |
| | S14153S,S14154S,S14155S,S14156S,S14157S,S14158S,S14159S | |
| | S142XXS,S143XXS,S144XXS,S145XXS,S148XXS,S149XXS,S15001S | |
| | S15002S,S15009S,S15011S,S15012S,S15019S,S15021S,S15022S | |
| | S15029S,S15091S,S15092S,S15099S,S15101S,S15102S,S15109S | |
| | S15111S,S15112S,S15119S,S15121S,S15122S,S15129S,S15191S | |
| | S15192S,S15199S,S15201S,S15202S,S15209S,S15211S,S15212S | |
| | S15219S,S15221S,S15222S,S15229S,S15291S,S15292S,S15299S | |
| | S15301S,S15302S,S15309S,S15311S,S15312S,S15319S,S15321S | |
| | S15322S,S15329S,S15391S,S15392S,S15399S,S158XXS,S159XXS | |
| | S161XXS,S162XXS,S168XXS,S169XXS,S170XXS,S178XXS,S179XXS | |
| | S1980XS,S1981XS,S1982XS,S1983XS,S1984XS,S1985XS,S1989XS | |
| | S199XXS,S2000XS,S2001XS,S2002XS,S20101S,S20102S,S20109S | |
| | S20111S,S20112S,S20119S,S20121S,S20122S,S20129S,S20141S | |
| | S20142S,S20149S,S20151S,S20152S,S20159S,S20161S,S20162S | |
| | S20169S,S20171S,S20172S,S20179S,S2020XS,S20211S,S20212S | |
| | S20213S,S20214S,S20219S,S20221S,S20222S,S20223S,S20224S | |
| | S20229S,S20301S,S20302S,S20303S,S20304S,S20309S,S20311S | |
| | S20312S,S20313S,S20314S,S20319S,S20321S,S20322S,S20323S | |
| | S20324S,S20329S,S20341S,S20342S,S20343S,S20344S,S20349S | |
| | S20351S,S20352S,S20353S,S20354S,S20359S,S20361S,S20362S | |
| | S20363S,S20364S,S20369S,S20371S,S20372S,S20373S,S20374S | |
| | S20379S,S20401S,S20402S,S20409S,S20411S,S20412S,S20419S | |
| | S20421S,S20422S,S20429S,S20441S,S20442S,S20449S,S20451S | |
| | S20452S,S20459S,S20461S,S20462S,S20469S,S20471S,S20472S | |
| | S20479S,S2090XS,S2091XS,S2092XS,S2094XS,S2095XS,S2096XS | |
| | S2097XS,S21001S,S21002S,S21009S,S21011S,S21012S,S21019S | |
| | S21021S,S21022S,S21029S,S21031S,S21032S,S21039S,S21041S | |
| | S21042S,S21049S,S21051S,S21052S,S21059S,S21101S,S21102S | |
| | S21109S,S21111S,S21112S,S21119S,S21121S,S21122S,S21129S | |
| | S21131S,S21132S,S21139S,S21141S,S21142S,S21149S,S21151S | |
| | S21152S,S21159S,S21201S,S21202S,S21209S,S21211S,S21212S | |
| | S21219S,S21221S,S21222S,S21229S,S21231S,S21232S,S21239S | |
| | S21241S,S21242S,S21249S,S21251S,S21252S,S21259S,S21301S | |
| | S21302S,S21309S,S21311S,S21312S,S21319S,S21321S,S21322S | |
| | S21329S,S21331S,S21332S,S21339S,S21341S,S21342S,S21349S | |
| | S21351S,S21352S,S21359S,S21401S,S21402S,S21409S,S21411S | |
| | S21412S,S21419S,S21421S,S21422S,S21429S,S21431S,S21432S | |
| | S21439S,S21441S,S21442S,S21449S,S21451S,S21452S,S21459S | |
| | S2190XS,S2191XS,S2192XS,S2193XS,S2194XS,S2195XS,S22000S | |
| | S22001S,S22002S,S22008S,S22009S,S22010S,S22011S,S22012S | |
| | S22018S,S22019S,S22020S,S22021S,S22022S,S22028S,S22029S | |
| | S22030S,S22031S,S22032S,S22038S,S22039S,S22040S,S22041S | |
| | S22042S,S22048S,S22049S,S22050S,S22051S,S22052S,S22058S | |
| | S22059S,S22060S,S22061S,S22062S,S22068S,S22069S,S22070S | |
| | S22071S,S22072S,S22078S,S22079S,S22080S,S22081S,S22082S | |
| | S22088S,S22089S,S2220XS,S2221XS,S2222XS,S2223XS,S2224XS | |
| | S2231XS,S2232XS,S2239XS,S2241XS,S2242XS,S2243XS,S2249XS | |
| | S225XXS,S229XXS,S230XXS,S23100S,S23101S,S23110S,S23111S | |
| | S23120S,S23121S,S23122S,S23123S,S23130S,S23131S,S23132S | |
| | S23133S,S23140S,S23141S,S23142S,S23143S,S23150S,S23151S | |
| | S23152S,S23153S,S23160S,S23161S,S23162S,S23163S,S23170S | |
| | S23171S,S2320XS,S2329XS,S233XXS,S2341XS,S23420S,S23421S | |
| | S23428S,S23429S,S238XXS,S239XXS,S240XXS,S24101S,S24102S | |
| | S24103S,S24104S,S24109S,S24111S,S24112S,S24113S,S24114S | |
| | S24119S,S24131S,S24132S,S24133S,S24134S,S24139S,S24141S | |
| | S24142S,S24143S,S24144S,S24149S,S24151S,S24152S,S24153S | |
| | S24154S,S24159S,S242XXS,S243XXS,S244XXS,S248XXS,S249XXS | |
| | S2500XS,S2501XS,S2502XS,S2509XS,S25101S,S25102S,S25109S | |
| | S25111S,S25112S,S25119S,S25121S,S25122S,S25129S,S25191S | |
| | S25192S,S25199S,S2520XS,S2521XS,S2522XS,S2529XS,S25301S | |
| | S25302S,S25309S,S25311S,S25312S,S25319S,S25321S,S25322S | |
| | S25329S,S25391S,S25392S,S25399S,S25401S,S25402S,S25409S | |
| | S25411S,S25412S,S25419S,S25421S,S25422S,S25429S,S25491S | |
| | S25492S,S25499S,S25501S,S25502S,S25509S,S25511S,S25512S | |
| | S25519S,S25591S,S25592S,S25599S,S25801S,S25802S,S25809S | |
| | S25811S,S25812S,S25819S,S25891S,S25892S,S25899S,S2590XS | |
| | S2591XS,S2599XS,S2600XS,S2601XS,S26020S,S26021S,S26022S | |
| | S2609XS,S2610XS,S2611XS,S2612XS,S2619XS,S2690XS,S2691XS | |
| | S2692XS,S2699XS,S270XXS,S271XXS,S272XXS,S27301S,S27302S | |
| | S27309S,S27311S,S27312S,S27319S,S27321S,S27322S,S27329S | |
| | S27331S,S27332S,S27339S,S27391S,S27392S,S27399S,S27401S | |
| | S27402S,S27409S,S27411S,S27412S,S27419S,S27421S,S27422S | |
| | S27429S,S27431S,S27432S,S27439S,S27491S,S27492S,S27499S | |

Continued on next page

 

Table S9 (continued).

| Diagnosis | ICD-10 | ICD-9 |
|---|---|---|
| | S2750XS,S2751XS,S2752XS,S2753XS,S2759XS,S2760XS,S2763XS<br>S2769XS,S27802S,S27803S,S27808S,S27809S,S27812S,S27813S<br>S27818S,S27819S,S27892S,S27893S,S27898S,S27899S,S279XXS<br>S280XXS,S281XXS,S28211S,S28212S,S28219S,S28221S,S28222S<br>S28229S,S29001S,S29002S,S29009S,S29011S,S29012S,S29019S<br>S29021S,S29022S,S29029S,S29091S,S29092S,S29099S,S298XXS<br>S299XXS,S300XXS,S301XXS,S30201S,S30202S,S3021XS,S3022XS<br>S3023XS,S303XXS,S30810S,S30811S,S30812S,S30813S,S30814S<br>S30815S,S30816S,S30817S,S30820S,S30821S,S30822S,S30823S<br>S30824S,S30825S,S30826S,S30827S,S30840S,S30841S,S30842S<br>S30843S,S30844S,S30845S,S30846S,S30850S,S30851S,S30852S<br>S30853S,S30854S,S30855S,S30856S,S30857S,S30860S,S30861S<br>S30862S,S30863S,S30864S,S30865S,S30866S,S30867S,S30870S<br>S30871S,S30872S,S30873S,S30874S,S30875S,S30876S,S30877S<br>S3091XS,S3092XS,S3093XS,S3094XS,S3095XS,S3096XS,S3097XS<br>S3098XS,S31000S,S31001S,S31010S,S31011S,S31020S,S31021S<br>S31030S,S31031S,S31040S,S31041S,S31050S,S31051S,S31100S<br>S31101S,S31102S,S31103S,S31104S,S31105S,S31109S,S31110S<br>S31111S,S31112S,S31113S,S31114S,S31115S,S31119S,S31120S<br>S31121S,S31122S,S31123S,S31124S,S31125S,S31129S,S31130S<br>S31131S,S31132S,S31133S,S31134S,S31135S,S31139S,S31140S<br>S31141S,S31142S,S31143S,S31144S,S31145S,S31149S,S31150S<br>S31151S,S31152S,S31153S,S31154S,S31155S,S31159S,S3120XS<br>S3121XS,S3122XS,S3123XS,S3124XS,S3125XS,S3130XS,S3131XS<br>S3132XS,S3133XS,S3134XS,S3135XS,S3140XS,S3141XS,S3142XS<br>S3143XS,S3144XS,S3145XS,S31501S,S31502S,S31511S,S31512S<br>S31521S,S31522S,S31531S,S31532S,S31541S,S31542S,S31551S<br>S31552S,S31600S,S31601S,S31602S,S31603S,S31604S,S31605S<br>S31609S,S31610S,S31611S,S31612S,S31613S,S31614S,S31615S<br>S31619S,S31620S,S31621S,S31622S,S31623S,S31624S,S31625S<br>S31629S,S31630S,S31631S,S31632S,S31633S,S31634S,S31635S<br>S31639S,S31640S,S31641S,S31642S,S31643S,S31644S,S31645S<br>S31649S,S31650S,S31651S,S31652S,S31653S,S31654S,S31655S<br>S31659S,S31801S,S31802S,S31803S,S31804S,S31805S,S31809S<br>S31811S,S31812S,S31813S,S31814S,S31815S,S31819S,S31821S<br>S31822S,S31823S,S31824S,S31825S,S31829S,S31831S,S31832S<br>S31833S,S31834S,S31835S,S31839S,S32000S,S32001S,S32002S<br>S32008S,S32009S,S32010S,S32011S,S32012S,S32018S,S32019S<br>S32020S,S32021S,S32022S,S32028S,S32029S,S32030S,S32031S<br>S32032S,S32038S,S32039S,S32040S,S32041S,S32042S,S32048S<br>S32049S,S32050S,S32051S,S32052S,S32058S,S32059S,S3210XS<br>S32110S,S32111S,S32112S,S32119S,S32120S,S32121S,S32122S<br>S32129S,S32130S,S32131S,S32132S,S32139S,S3214XS,S3215XS<br>S3216XS,S3217XS,S3219XS,S322XXS,S32301S,S32302S,S32309S<br>S32311S,S32312S,S32313S,S32314S,S32315S,S32316S,S32391S<br>S32392S,S32399S,S32401S,S32402S,S32409S,S32411S,S32412S<br>S32413S,S32414S,S32415S,S32416S,S32421S,S32422S,S32423S<br>S32424S,S32425S,S32426S,S32431S,S32432S,S32433S,S32434S<br>S32435S,S32436S,S32441S,S32442S,S32443S,S32444S,S32445S<br>S32446S,S32451S,S32452S,S32453S,S32454S,S32455S,S32456S<br>S32461S,S32462S,S32463S,S32464S,S32465S,S32466S,S32471S<br>S32472S,S32473S,S32474S,S32475S,S32476S,S32481S,S32482S<br>S32483S,S32484S,S32485S,S32486S,S32491S,S32492S,S32499S<br>S32501S,S32502S,S32509S,S32511S,S32512S,S32519S,S32591S<br>S32592S,S32599S,S32601S,S32602S,S32609S,S32611S,S32612S<br>S32613S,S32614S,S32615S,S32616S,S32691S,S32692S,S32699S<br>S32810S,S32811S,S3282XS,S3289XS,S329XXS,S330XXS,S33100S<br>S33101S,S33110S,S33111S,S33120S,S33121S,S33130S,S33131S<br>S33140S,S33141S,S332XXS,S3330XS,S3339XS,S334XXS,S335XXS<br>S336XXS,S338XXS,S339XXS,S3401XS,S3402XS,S34101S,S34102S<br>S34103S,S34104S,S34105S,S34109S,S34111S,S34112S,S34113S<br>S34114S,S34115S,S34119S,S34121S,S34122S,S34123S,S34124S<br>S34125S,S34129S,S34131S,S34132S,S34139S,S3421XS,S3422XS<br>S343XXS,S344XXS,S345XXS,S346XXS,S348XXS,S349XXS,S3500XS<br>S3501XS,S3502XS,S3509XS,S3510XS,S3511XS,S3512XS,S3519XS<br>S35211S,S35212S,S35218S,S35219S,S35221S,S35222S,S35228S<br>S35229S,S35231S,S35232S,S35238S,S35239S,S35291S,S35292S<br>S35298S,S35299S,S35311S,S35318S,S35319S,S35321S,S35328S<br>S35329S,S35331S,S35338S,S35339S,S35341S,S35348S,S35349S<br>S35401S,S35402S,S35403S,S35404S,S35405S,S35406S,S35411S<br>S35412S,S35413S,S35414S,S35415S,S35416S,S35491S,S35492S<br>S35493S,S35494S,S35495S,S35496S,S3550XS,S35511S,S35512S<br>S35513S,S35514S,S35515S,S35516S,S35531S,S35532S,S35533S<br>S35534S,S35535S,S35536S,S3559XS,S358X1S,S358X8S,S358X9S<br>S3590XS,S3591XS,S3599XS,S3600XS,S36020S,S36021S,S36029S<br>S36030S,S36031S,S36032S,S36039S,S3609XS,S36112S,S36113S<br>S36114S,S36115S,S36116S,S36118S,S36119S,S36122S,S36123S<br>S36128S,S36129S,S3613XS,S36200S,S36201S,S36202S,S36209S<br>S36220S,S36221S,S36222S,S36229S,S36230S,S36231S,S36232S<br>S36239S,S36240S,S36241S,S36242S,S36249S,S36250S,S36251S<br>S36252S,S36259S,S36260S,S36261S,S36262S,S36269S,S36290S<br>S36291S,S36292S,S36299S,S3630XS,S3632XS,S3633XS,S3639XS<br>S36400S,S36408S,S36409S,S36410S,S36418S,S36419S,S36420S<br>S36428S,S36429S,S36430S,S36438S,S36439S,S36490S,S36498S<br>S36499S,S36500S,S36501S,S36502S,S36503S,S36508S,S36509S | |

Continued on next page

Table S9 (continued).

| Diagnosis | ICD-10 | ICD-9 |
|---|---|---|
| | S36510S,S36511S,S36512S,S36513S,S36518S,S36519S,S36520S | |
| | S36521S,S36522S,S36523S,S36528S,S36529S,S36530S,S36531S | |
| | S36532S,S36533S,S36538S,S36539S,S36590S,S36591S,S36592S | |
| | S36593S,S36598S,S36599S,S3660XS,S3661XS,S3662XS,S3663XS | |
| | S3669XS,S3681XS,S36892S,S36893S,S36898S,S36899S,S3690XS | |
| | S3692XS,S3693XS,S3699XS,S37001S,S37002S,S37009S,S37011S | |
| | S37012S,S37019S,S37021S,S37022S,S37029S,S37031S,S37032S | |
| | S37039S,S37041S,S37042S,S37049S,S37051S,S37052S,S37059S | |
| | S37061S,S37062S,S37069S,S37091S,S37092S,S37099S,S3710XS | |
| | S3712XS,S3713XS,S3719XS,S3720XS,S3722XS,S3723XS,S3729XS | |
| | S3730XS,S3732XS,S3733XS,S3739XS,S37401S,S37402S,S37409S | |
| | S37421S,S37422S,S37429S,S37431S,S37432S,S37439S,S37491S | |
| | S37492S,S37499S,S37501S,S37502S,S37509S,S37511S,S37512S | |
| | S37519S,S37521S,S37522S,S37529S,S37531S,S37532S,S37539S | |
| | S37591S,S37592S,S37599S,S3760XS,S3762XS,S3763XS,S3769XS | |
| | S37812S,S37813S,S37818S,S37819S,S37822S,S37823S,S37828S | |
| | S37829S,S37892S,S37893S,S37898S,S37899S,S3790XS,S3792XS | |
| | S3793XS,S3799XS,S38001S,S38002S,S3801XS,S3802XS,S3803XS | |
| | S381XXS,S38211S,S38212S,S38221S,S38222S,S38231S,S38232S | |
| | S383XXS,S39001S,S39002S,S39003S,S39011S,S39012S,S39013S | |
| | S39021S,S39022S,S39023S,S39091S,S39092S,S39093S,S3981XS | |
| | S3982XS,S3983XS,S39840S,S39848S,S3991XS,S3992XS,S3993XS | |
| | S3994XS,S40011S,S40012S,S40019S,S40021S,S40022S,S40029S | |
| | S40211S,S40212S,S40219S,S40221S,S40222S,S40229S,S40241S | |
| | S40242S,S40249S,S40251S,S40252S,S40259S,S40261S,S40262S | |
| | S40269S,S40271S,S40272S,S40279S,S40811S,S40812S,S40819S | |
| | S40821S,S40822S,S40829S,S40841S,S40842S,S40849S,S40851S | |
| | S40852S,S40859S,S40861S,S40862S,S40869S,S40871S,S40872S | |
| | S40879S,S40911S,S40912S,S40919S,S40921S,S40922S,S40929S | |
| | S41001S,S41002S,S41009S,S41011S,S41012S,S41019S,S41021S | |
| | S41022S,S41029S,S41031S,S41032S,S41039S,S41041S,S41042S | |
| | S41049S,S41051S,S41052S,S41059S,S41101S,S41102S,S41109S | |
| | S41111S,S41112S,S41119S,S41121S,S41122S,S41129S,S41131S | |
| | S41132S,S41139S,S41141S,S41142S,S41149S,S41151S,S41152S | |
| | S41159S,S42001S,S42002S,S42009S,S42011S,S42012S,S42013S | |
| | S42014S,S42015S,S42016S,S42017S,S42018S,S42019S,S42021S | |
| | S42022S,S42023S,S42024S,S42025S,S42026S,S42031S,S42032S | |
| | S42033S,S42034S,S42035S,S42036S,S42101S,S42102S,S42109S | |
| | S42111S,S42112S,S42113S,S42114S,S42115S,S42116S,S42121S | |
| | S42122S,S42123S,S42124S,S42125S,S42126S,S42131S,S42132S | |
| | S42133S,S42134S,S42135S,S42136S,S42141S,S42142S,S42143S | |
| | S42144S,S42145S,S42146S,S42151S,S42152S,S42153S,S42154S | |
| | S42155S,S42156S,S42191S,S42192S,S42199S,S42201S,S42202S | |
| | S42209S,S42211S,S42212S,S42213S,S42214S,S42215S,S42216S | |
| | S42221S,S42222S,S42223S,S42224S,S42225S,S42226S,S42231S | |
| | S42232S,S42239S,S42241S,S42242S,S42249S,S42251S,S42252S | |
| | S42253S,S42254S,S42255S,S42256S,S42261S,S42262S,S42263S | |
| | S42264S,S42265S,S42266S,S42271S,S42272S,S42279S,S42291S | |
| | S42292S,S42293S,S42294S,S42295S,S42296S,S42301S,S42302S | |
| | S42309S,S42311S,S42312S,S42319S,S42321S,S42322S,S42323S | |
| | S42324S,S42325S,S42326S,S42331S,S42332S,S42333S,S42334S | |
| | S42335S,S42336S,S42341S,S42342S,S42343S,S42344S,S42345S | |
| | S42346S,S42351S,S42352S,S42353S,S42354S,S42355S,S42356S | |
| | S42361S,S42362S,S42363S,S42364S,S42365S,S42366S,S42391S | |
| | S42392S,S42399S,S42401S,S42402S,S42409S,S42411S,S42412S | |
| | S42413S,S42414S,S42415S,S42416S,S42421S,S42422S,S42423S | |
| | S42424S,S42425S,S42426S,S42431S,S42432S,S42433S,S42434S | |
| | S42435S,S42436S,S42441S,S42442S,S42443S,S42444S,S42445S | |
| | S42446S,S42447S,S42448S,S42449S,S42451S,S42452S,S42453S | |
| | S42454S,S42455S,S42456S,S42461S,S42462S,S42463S,S42464S | |
| | S42465S,S42466S,S42471S,S42472S,S42473S,S42474S,S42475S | |
| | S42476S,S42481S,S42482S,S42489S,S42491S,S42492S,S42493S | |
| | S42494S,S42495S,S42496S,S4290XS,S4291XS,S4292XS,S43001S | |
| | S43002S,S43003S,S43004S,S43005S,S43006S,S43011S,S43012S | |
| | S43013S,S43014S,S43015S,S43016S,S43021S,S43022S,S43023S | |
| | S43024S,S43025S,S43026S,S43031S,S43032S,S43033S,S43034S | |
| | S43035S,S43036S,S43081S,S43082S,S43083S,S43084S,S43085S | |
| | S43086S,S43101S,S43102S,S43109S,S43111S,S43112S,S43119S | |
| | S43121S,S43122S,S43129S,S43131S,S43132S,S43139S,S43141S | |
| | S43142S,S43149S,S43151S,S43152S,S43159S,S43201S,S43202S | |
| | S43203S,S43204S,S43205S,S43206S,S43211S,S43212S,S43213S | |
| | S43214S,S43215S,S43216S,S43221S,S43222S,S43223S,S43224S | |
| | S43225S,S43226S,S43301S,S43302S,S43303S,S43304S,S43305S | |
| | S43306S,S43311S,S43312S,S43313S,S43314S,S43315S,S43316S | |
| | S43391S,S43392S,S43393S,S43394S,S43395S,S43396S,S43401S | |
| | S43402S,S43409S,S43411S,S43412S,S43419S,S43421S,S43422S | |
| | S43429S,S43431S,S43432S,S43439S,S43491S,S43492S,S43499S | |
| | S4350XS,S4351XS,S4352XS,S4360XS,S4361XS,S4362XS,S4380XS | |
| | S4381XS,S4382XS,S4390XS,S4391XS,S4392XS,S4400XS,S4401XS | |
| | S4402XS,S4410XS,S4411XS,S4412XS,S4420XS,S4421XS,S4422XS | |
| | S4430XS,S4431XS,S4432XS,S4440XS,S4441XS,S4442XS,S4450XS | |
| | S4451XS,S4452XS,S448X1S,S448X2S,S448X9S,S4490XS,S4491XS | |
| | S4492XS,S45001S,S45002S,S45009S,S45011S,S45012S,S45019S | |
| | S45091S,S45092S,S45099S,S45101S,S45102S,S45109S,S45111S | |
| | S45112S,S45119S,S45191S,S45192S,S45199S,S45201S,S45202S | |

Continued on next page

Table S9 (continued).

| Diagnosis | ICD-10 | ICD-9 |
| --- | --- | --- |
| | S45209S,S45211S,S45212S,S45219S,S45291S,S45292S,S45299S | |
| | S45301S,S45302S,S45309S,S45311S,S45312S,S45319S,S45391S | |
| | S45392S,S45399S,S45801S,S45802S,S45809S,S45811S,S45812S | |
| | S45819S,S45891S,S45892S,S45899S,S45901S,S45902S,S45909S | |
| | S45911S,S45912S,S45919S,S45991S,S45992S,S45999S,S46001S | |
| | S46002S,S46009S,S46011S,S46012S,S46019S,S46021S,S46022S | |
| | S46029S,S46091S,S46092S,S46099S,S46101S,S46102S,S46109S | |
| | S46111S,S46112S,S46119S,S46121S,S46122S,S46129S,S46191S | |
| | S46192S,S46199S,S46201S,S46202S,S46209S,S46211S,S46212S | |
| | S46219S,S46221S,S46222S,S46229S,S46291S,S46292S,S46299S | |
| | S46301S,S46302S,S46309S,S46311S,S46312S,S46319S,S46321S | |
| | S46322S,S46329S,S46391S,S46392S,S46399S,S46801S,S46802S | |
| | S46809S,S46811S,S46812S,S46819S,S46821S,S46822S,S46829S | |
| | S46891S,S46892S,S46899S,S46901S,S46902S,S46909S,S46911S | |
| | S46912S,S46919S,S46921S,S46922S,S46929S,S46991S,S46992S | |
| | S46999S,S471XXS,S472XXS,S479XXS,S48011S,S48012S,S48019S | |
| | S48021S,S48022S,S48029S,S48111S,S48112S,S48119S,S48121S | |
| | S48122S,S48129S,S48911S,S48912S,S48919S,S48921S,S48922S | |
| | S48929S,S49001S,S49002S,S49009S,S49011S,S49012S,S49019S | |
| | S49021S,S49022S,S49029S,S49031S,S49032S,S49039S,S49041S | |
| | S49042S,S49049S,S49091S,S49092S,S49099S,S49101S,S49102S | |
| | S49109S,S49111S,S49112S,S49119S,S49121S,S49122S,S49129S | |
| | S49131S,S49132S,S49139S,S49141S,S49142S,S49149S,S49191S | |
| | S49192S,S49199S,S4980XS,S4981XS,S4982XS,S4990XS,S4991XS | |
| | S4992XS,S5000XS,S5001XS,S5002XS,S5010XS,S5011XS,S5012XS | |
| | S50311S,S50312S,S50319S,S50321S,S50322S,S50329S,S50341S | |
| | S50342S,S50349S,S50351S,S50352S,S50359S,S50361S,S50362S | |
| | S50369S,S50371S,S50372S,S50379S,S50811S,S50812S,S50819S | |
| | S50821S,S50822S,S50829S,S50841S,S50842S,S50849S,S50851S | |
| | S50852S,S50859S,S50861S,S50862S,S50869S,S50871S,S50872S | |
| | S50879S,S50901S,S50902S,S50909S,S50911S,S50912S,S50919S | |
| | S51001S,S51002S,S51009S,S51011S,S51012S,S51019S,S51021S | |
| | S51022S,S51029S,S51031S,S51032S,S51039S,S51041S,S51042S | |
| | S51049S,S51051S,S51052S,S51059S,S51801S,S51802S,S51809S | |
| | S51811S,S51812S,S51819S,S51821S,S51822S,S51829S,S51831S | |
| | S51832S,S51839S,S51841S,S51842S,S51849S,S51851S,S51852S | |
| | S51859S,S52001S,S52002S,S52009S,S52011S,S52012S,S52019S | |
| | S52021S,S52022S,S52023S,S52024S,S52025S,S52026S,S52031S | |
| | S52032S,S52033S,S52034S,S52035S,S52036S,S52041S,S52042S | |
| | S52043S,S52044S,S52045S,S52046S,S52091S,S52092S,S52099S | |
| | S52101S,S52102S,S52109S,S52111S,S52112S,S52119S,S52121S | |
| | S52122S,S52123S,S52124S,S52125S,S52126S,S52131S,S52132S | |
| | S52133S,S52134S,S52135S,S52136S,S52181S,S52182S,S52189S | |
| | S52201S,S52202S,S52209S,S52211S,S52212S,S52219S,S52221S | |
| | S52222S,S52223S,S52224S,S52225S,S52226S,S52231S,S52232S | |
| | S52233S,S52234S,S52235S,S52236S,S52241S,S52242S,S52243S | |
| | S52244S,S52245S,S52246S,S52251S,S52252S,S52253S,S52254S | |
| | S52255S,S52256S,S52261S,S52262S,S52263S,S52264S,S52265S | |
| | S52266S,S52271S,S52272S,S52279S,S52281S,S52282S,S52283S | |
| | S52291S,S52292S,S52299S,S52301S,S52302S,S52309S,S52311S | |
| | S52312S,S52319S,S52321S,S52322S,S52323S,S52324S,S52325S | |
| | S52326S,S52331S,S52332S,S52333S,S52334S,S52335S,S52336S | |
| | S52341S,S52342S,S52343S,S52344S,S52345S,S52346S,S52351S | |
| | S52352S,S52353S,S52354S,S52355S,S52356S,S52361S,S52362S | |
| | S52363S,S52364S,S52365S,S52366S,S52371S,S52372S,S52379S | |
| | S52381S,S52382S,S52389S,S52391S,S52392S,S52399S,S52501S | |
| | S52502S,S52509S,S52511S,S52512S,S52513S,S52514S,S52515S | |
| | S52516S,S52521S,S52522S,S52529S,S52531S,S52532S,S52539S | |
| | S52541S,S52542S,S52549S,S52551S,S52552S,S52559S,S52561S | |
| | S52562S,S52569S,S52571S,S52572S,S52579S,S52591S,S52592S | |
| | S52599S,S52601S,S52602S,S52609S,S52611S,S52612S,S52613S | |
| | S52614S,S52615S,S52616S,S52621S,S52622S,S52629S,S52691S | |
| | S52692S,S52699S,S5290XS,S5291XS,S5292XS,S53001S,S53002S | |
| | S53003S,S53004S,S53005S,S53006S,S53011S,S53012S,S53013S | |
| | S53014S,S53015S,S53016S,S53021S,S53022S,S53023S,S53024S | |
| | S53025S,S53026S,S53031S,S53032S,S53033S,S53091S,S53092S | |
| | S53093S,S53094S,S53095S,S53096S,S53101S,S53102S,S53103S | |
| | S53104S,S53105S,S53106S,S53111S,S53112S,S53113S,S53114S | |
| | S53115S,S53116S,S53121S,S53122S,S53123S,S53124S,S53125S | |
| | S53126S,S53131S,S53132S,S53133S,S53134S,S53135S,S53136S | |
| | S53141S,S53142S,S53143S,S53144S,S53145S,S53146S,S53191S | |
| | S53192S,S53193S,S53194S,S53195S,S53196S,S5320XS,S5321XS | |
| | S5322XS,S5330XS,S5331XS,S5332XS,S53401S,S53402S,S53409S | |
| | S53411S,S53412S,S53419S,S53421S,S53422S,S53429S,S53431S | |
| | S53432S,S53439S,S53441S,S53442S,S53449S,S53491S,S53492S | |
| | S53499S,S5400XS,S5401XS,S5402XS,S5410XS,S5411XS,S5412XS | |
| | S5420XS,S5421XS,S5422XS,S5430XS,S5431XS,S5432XS,S548X1S | |
| | S548X2S,S548X9S,S5490XS,S5491XS,S5492XS,S55001S,S55002S | |
| | S55009S,S55011S,S55012S,S55019S,S55091S,S55092S,S55099S | |
| | S55101S,S55102S,S55109S,S55111S,S55112S,S55119S,S55191S | |
| | S55192S,S55199S,S55201S,S55202S,S55209S,S55211S,S55212S | |
| | S55219S,S55291S,S55292S,S55299S,S55801S,S55802S,S55809S | |
| | S55811S,S55812S,S55819S,S55891S,S55892S,S55899S,S55901S | |
| | S55902S,S55909S,S55911S,S55912S,S55919S,S55991S,S55992S | |
| | S55999S,S56001S,S56002S,S56009S,S56011S,S56012S,S56019S | |

Continued on next page

Table S9 (continued).

| Diagnosis | ICD-10 | ICD-9 |
|---|---|---|
| | S56021S,S56022S,S56029S,S56091S,S56092S,S56099S,S56101S | |
| | S56102S,S56103S,S56104S,S56105S,S56106S,S56107S,S56108S | |
| | S56109S,S56111S,S56112S,S56113S,S56114S,S56115S,S56116S | |
| | S56117S,S56118S,S56119S,S56121S,S56122S,S56123S,S56124S | |
| | S56125S,S56126S,S56127S,S56128S,S56129S,S56191S,S56192S | |
| | S56193S,S56194S,S56195S,S56196S,S56197S,S56198S,S56199S | |
| | S56201S,S56202S,S56209S,S56211S,S56212S,S56219S,S56221S | |
| | S56222S,S56229S,S56291S,S56292S,S56299S,S56301S,S56302S | |
| | S56309S,S56311S,S56312S,S56319S,S56321S,S56322S,S56329S | |
| | S56391S,S56392S,S56399S,S56401S,S56402S,S56403S,S56404S | |
| | S56405S,S56406S,S56407S,S56408S,S56409S,S56411S,S56412S | |
| | S56413S,S56414S,S56415S,S56416S,S56417S,S56418S,S56419S | |
| | S56421S,S56422S,S56423S,S56424S,S56425S,S56426S,S56427S | |
| | S56428S,S56429S,S56491S,S56492S,S56493S,S56494S,S56495S | |
| | S56496S,S56497S,S56498S,S56499S,S56501S,S56502S,S56509S | |
| | S56511S,S56512S,S56519S,S56521S,S56522S,S56529S,S56591S | |
| | S56592S,S56599S,S56801S,S56802S,S56809S,S56811S,S56812S | |
| | S56819S,S56821S,S56822S,S56829S,S56891S,S56892S,S56899S | |
| | S56901S,S56902S,S56909S,S56911S,S56912S,S56919S,S56921S | |
| | S56922S,S56929S,S56991S,S56992S,S56999S,S5700XS,S5701XS | |
| | S5702XS,S5780XS,S5781XS,S5782XS,S58011S,S58012S,S58019S | |
| | S58021S,S58022S,S58029S,S58111S,S58112S,S58119S,S58121S | |
| | S58122S,S58129S,S58911S,S58912S,S58919S,S58921S,S58922S | |
| | S58929S,S59001S,S59002S,S59009S,S59011S,S59012S,S59019S | |
| | S59021S,S59022S,S59029S,S59031S,S59032S,S59039S,S59041S | |
| | S59042S,S59049S,S59091S,S59092S,S59099S,S59101S,S59102S | |
| | S59109S,S59111S,S59112S,S59119S,S59121S,S59122S,S59129S | |
| | S59131S,S59132S,S59139S,S59141S,S59142S,S59149S,S59191S | |
| | S59192S,S59199S,S59201S,S59202S,S59209S,S59211S,S59212S | |
| | S59219S,S59221S,S59222S,S59229S,S59231S,S59232S,S59239S | |
| | S59241S,S59242S,S59249S,S59291S,S59292S,S59299S,S59801S | |
| | S59802S,S59809S,S59811S,S59812S,S59819S,S59901S,S59902S | |
| | S59909S,S59911S,S59912S,S59919S,S6000XS,S60011S,S60012S | |
| | S60019S,S60021S,S60022S,S60029S,S60031S,S60032S,S60039S | |
| | S60041S,S60042S,S60049S,S60051S,S60052S,S60059S,S6010XS | |
| | S60111S,S60112S,S60119S,S60121S,S60122S,S60129S,S60131S | |
| | S60132S,S60139S,S60141S,S60142S,S60149S,S60151S,S60152S | |
| | S60159S,S60211S,S60212S,S60219S,S60221S,S60222S,S60229S | |
| | S60311S,S60312S,S60319S,S60321S,S60322S,S60329S,S60341S | |
| | S60342S,S60349S,S60351S,S60352S,S60359S,S60361S,S60362S | |
| | S60369S,S60371S,S60372S,S60379S,S60391S,S60392S,S60399S | |
| | S60410S,S60411S,S60412S,S60413S,S60414S,S60415S,S60416S | |
| | S60417S,S60418S,S60419S,S60420S,S60421S,S60422S,S60423S | |
| | S60424S,S60425S,S60426S,S60427S,S60428S,S60429S,S60440S | |
| | S60441S,S60442S,S60443S,S60444S,S60445S,S60446S,S60447S | |
| | S60448S,S60449S,S60450S,S60451S,S60452S,S60453S,S60454S | |
| | S60455S,S60456S,S60457S,S60458S,S60459S,S60460S,S60461S | |
| | S60462S,S60463S,S60464S,S60465S,S60466S,S60467S,S60468S | |
| | S60469S,S60470S,S60471S,S60472S,S60473S,S60474S,S60475S | |
| | S60476S,S60477S,S60478S,S60479S,S60511S,S60512S,S60519S | |
| | S60521S,S60522S,S60529S,S60541S,S60542S,S60549S,S60551S | |
| | S60552S,S60559S,S60561S,S60562S,S60569S,S60571S,S60572S | |
| | S60579S,S60811S,S60812S,S60819S,S60821S,S60822S,S60829S | |
| | S60841S,S60842S,S60849S,S60851S,S60852S,S60859S,S60861S | |
| | S60862S,S60869S,S60871S,S60872S,S60879S,S60911S,S60912S | |
| | S60919S,S60921S,S60922S,S60929S,S60931S,S60932S,S60939S | |
| | S60940S,S60941S,S60942S,S60943S,S60944S,S60945S,S60946S | |
| | S60947S,S60948S,S60949S,S61001S,S61002S,S61009S,S61011S | |
| | S61012S,S61019S,S61021S,S61022S,S61029S,S61031S,S61032S | |
| | S61039S,S61041S,S61042S,S61049S,S61051S,S61052S,S61059S | |
| | S61101S,S61102S,S61109S,S61111S,S61112S,S61119S,S61121S | |
| | S61122S,S61129S,S61131S,S61132S,S61139S,S61141S,S61142S | |
| | S61149S,S61151S,S61152S,S61159S,S61200S,S61201S,S61202S | |
| | S61203S,S61204S,S61205S,S61206S,S61207S,S61208S,S61209S | |
| | S61210S,S61211S,S61212S,S61213S,S61214S,S61215S,S61216S | |
| | S61217S,S61218S,S61219S,S61220S,S61221S,S61222S,S61223S | |
| | S61224S,S61225S,S61226S,S61227S,S61228S,S61229S,S61230S | |
| | S61231S,S61232S,S61233S,S61234S,S61235S,S61236S,S61237S | |
| | S61238S,S61239S,S61240S,S61241S,S61242S,S61243S,S61244S | |
| | S61245S,S61246S,S61247S,S61248S,S61249S,S61250S,S61251S | |
| | S61252S,S61253S,S61254S,S61255S,S61256S,S61257S,S61258S | |
| | S61259S,S61300S,S61301S,S61302S,S61303S,S61304S,S61305S | |
| | S61306S,S61307S,S61308S,S61309S,S61310S,S61311S,S61312S | |
| | S61313S,S61314S,S61315S,S61316S,S61317S,S61318S,S61319S | |
| | S61320S,S61321S,S61322S,S61323S,S61324S,S61325S,S61326S | |
| | S61327S,S61328S,S61329S,S61330S,S61331S,S61332S,S61333S | |
| | S61334S,S61335S,S61336S,S61337S,S61338S,S61339S,S61340S | |
| | S61341S,S61342S,S61343S,S61344S,S61345S,S61346S,S61347S | |
| | S61348S,S61349S,S61350S,S61351S,S61352S,S61353S,S61354S | |
| | S61355S,S61356S,S61357S,S61358S,S61359S,S61401S,S61402S | |
| | S61409S,S61411S,S61412S,S61419S,S61421S,S61422S,S61429S | |
| | S61431S,S61432S,S61439S,S61441S,S61442S,S61449S,S61451S | |
| | S61452S,S61459S,S61501S,S61502S,S61509S,S61511S,S61512S | |
| | S61519S,S61521S,S61522S,S61529S,S61531S,S61532S,S61539S | |
| | S61541S,S61542S,S61549S,S61551S,S61552S,S61559S,S62001S | |

Continued on next page

Table S9 (continued).

| Diagnosis | ICD-10 | ICD-9 |
|---|---|---|
| | S62002S,S62009S,S62011S,S62012S,S62013S,S62014S,S62015S<br>S62016S,S62021S,S62022S,S62023S,S62024S,S62025S,S62026S<br>S62031S,S62032S,S62033S,S62034S,S62035S,S62036S,S62101S<br>S62102S,S62109S,S62111S,S62112S,S62113S,S62114S,S62115S<br>S62116S,S62121S,S62122S,S62123S,S62124S,S62125S,S62126S<br>S62131S,S62132S,S62133S,S62134S,S62135S,S62136S,S62141S<br>S62142S,S62143S,S62144S,S62145S,S62146S,S62151S,S62152S<br>S62153S,S62154S,S62155S,S62156S,S62161S,S62162S,S62163S<br>S62164S,S62165S,S62166S,S62171S,S62172S,S62173S,S62174S<br>S62175S,S62176S,S62181S,S62182S,S62183S,S62184S,S62185S<br>S62186S,S62201S,S62202S,S62209S,S62211S,S62212S,S62213S<br>S62221S,S62222S,S62223S,S62224S,S62225S,S62226S,S62231S<br>S62232S,S62233S,S62234S,S62235S,S62236S,S62241S,S62242S<br>S62243S,S62244S,S62245S,S62246S,S62251S,S62252S,S62253S<br>S62254S,S62255S,S62256S,S62291S,S62292S,S62299S,S62300S<br>S62301S,S62302S,S62303S,S62304S,S62305S,S62306S,S62307S<br>S62308S,S62309S,S62310S,S62311S,S62312S,S62313S,S62314S<br>S62315S,S62316S,S62317S,S62318S,S62319S,S62320S,S62321S<br>S62322S,S62323S,S62324S,S62325S,S62326S,S62327S,S62328S<br>S62329S,S62330S,S62331S,S62332S,S62333S,S62334S,S62335S<br>S62336S,S62337S,S62338S,S62339S,S62340S,S62341S,S62342S<br>S62343S,S62344S,S62345S,S62346S,S62347S,S62348S,S62349S<br>S62350S,S62351S,S62352S,S62353S,S62354S,S62355S,S62356S<br>S62357S,S62358S,S62359S,S62360S,S62361S,S62362S,S62363S<br>S62364S,S62365S,S62366S,S62367S,S62368S,S62369S,S62390S<br>S62391S,S62392S,S62393S,S62394S,S62395S,S62396S,S62397S<br>S62398S,S62399S,S62501S,S62502S,S62509S,S62511S,S62512S<br>S62513S,S62514S,S62515S,S62516S,S62521S,S62522S,S62523S<br>S62524S,S62525S,S62526S,S62600S,S62601S,S62602S,S62603S<br>S62604S,S62605S,S62606S,S62607S,S62608S,S62609S,S62610S<br>S62611S,S62612S,S62613S,S62614S,S62615S,S62616S,S62617S<br>S62618S,S62619S,S62620S,S62621S,S62622S,S62623S,S62624S<br>S62625S,S62626S,S62627S,S62628S,S62629S,S62630S,S62631S<br>S62632S,S62633S,S62634S,S62635S,S62636S,S62637S,S62638S<br>S62639S,S62640S,S62641S,S62642S,S62643S,S62644S,S62645S<br>S62646S,S62647S,S62648S,S62649S,S62650S,S62651S,S62652S<br>S62653S,S62654S,S62655S,S62656S,S62657S,S62658S,S62659S<br>S62660S,S62661S,S62662S,S62663S,S62664S,S62665S,S62666S<br>S62667S,S62668S,S62669S,S6290XS,S6291XS,S6292XS,S63001S<br>S63002S,S63003S,S63004S,S63005S,S63006S,S63011S,S63012S<br>S63013S,S63014S,S63015S,S63016S,S63021S,S63022S,S63023S<br>S63024S,S63025S,S63026S,S63031S,S63032S,S63033S,S63034S<br>S63035S,S63036S,S63041S,S63042S,S63043S,S63044S,S63045S<br>S63046S,S63051S,S63052S,S63053S,S63054S,S63055S,S63056S<br>S63061S,S63062S,S63063S,S63064S,S63065S,S63066S,S63071S<br>S63072S,S63073S,S63074S,S63075S,S63076S,S63091S,S63092S<br>S63093S,S63094S,S63095S,S63096S,S63101S,S63102S,S63103S<br>S63104S,S63105S,S63106S,S63111S,S63112S,S63113S,S63114S<br>S63115S,S63116S,S63121S,S63122S,S63123S,S63124S,S63125S<br>S63126S,S63131S,S63132S,S63133S,S63134S,S63135S,S63136S<br>S63141S,S63142S,S63143S,S63144S,S63145S,S63146S,S63200S<br>S63201S,S63202S,S63203S,S63204S,S63205S,S63206S,S63207S<br>S63208S,S63209S,S63210S,S63211S,S63212S,S63213S,S63214S<br>S63215S,S63216S,S63217S,S63218S,S63219S,S63220S,S63221S<br>S63222S,S63223S,S63224S,S63225S,S63226S,S63227S,S63228S<br>S63229S,S63230S,S63231S,S63232S,S63233S,S63234S,S63235S<br>S63236S,S63237S,S63238S,S63239S,S63240S,S63241S,S63242S<br>S63243S,S63244S,S63245S,S63246S,S63247S,S63248S,S63249S<br>S63250S,S63251S,S63252S,S63253S,S63254S,S63255S,S63256S<br>S63257S,S63258S,S63259S,S63260S,S63261S,S63262S,S63263S<br>S63264S,S63265S,S63266S,S63267S,S63268S,S63269S,S63270S<br>S63271S,S63272S,S63273S,S63274S,S63275S,S63276S,S63277S<br>S63278S,S63279S,S63280S,S63281S,S63282S,S63283S,S63284S<br>S63285S,S63286S,S63287S,S63288S,S63289S,S63290S,S63291S<br>S63292S,S63293S,S63294S,S63295S,S63296S,S63297S,S63298S<br>S63299S,S63301S,S63302S,S63309S,S63311S,S63312S,S63319S<br>S63321S,S63322S,S63329S,S63331S,S63332S,S63339S,S63391S<br>S63392S,S63399S,S63400S,S63401S,S63402S,S63403S,S63404S<br>S63405S,S63406S,S63407S,S63408S,S63409S,S63410S,S63411S<br>S63412S,S63413S,S63414S,S63415S,S63416S,S63417S,S63418S<br>S63419S,S63420S,S63421S,S63422S,S63423S,S63424S,S63425S<br>S63426S,S63427S,S63428S,S63429S,S63430S,S63431S,S63432S<br>S63433S,S63434S,S63435S,S63436S,S63437S,S63438S,S63439S<br>S63490S,S63491S,S63492S,S63493S,S63494S,S63495S,S63496S<br>S63497S,S63498S,S63499S,S63501S,S63502S,S63509S,S63511S<br>S63512S,S63519S,S63521S,S63522S,S63529S,S63591S,S63592S<br>S63599S,S63601S,S63602S,S63609S,S63610S,S63611S,S63612S<br>S63613S,S63614S,S63615S,S63616S,S63617S,S63618S,S63619S<br>S63621S,S63622S,S63629S,S63630S,S63631S,S63632S,S63633S<br>S63634S,S63635S,S63636S,S63637S,S63638S,S63639S,S63641S<br>S63642S,S63649S,S63650S,S63651S,S63652S,S63653S,S63654S<br>S63655S,S63656S,S63657S,S63658S,S63659S,S63681S,S63682S<br>S63689S,S63690S,S63691S,S63692S,S63693S,S63694S,S63695S<br>S63696S,S63697S,S63698S,S63699S,S638X1S,S638X2S,S638X9S<br>S6390XS,S6391XS,S6392XS,S6400XS,S6401XS,S6402XS,S6410XS | |

Continued on next page

Table S9 (continued).

| Diagnosis | ICD-10 | ICD-9 |
|---|---|---|
| | S6411XS,S6412XS,S6420XS,S6421XS,S6422XS,S6430XS,S6431XS | |
| | S6432XS,S6440XS,S64490S,S64491S,S64492S,S64493S,S64494S | |
| | S64495S,S64496S,S64497S,S64498S,S648X1S,S648X2S,S648X9S | |
| | S6490XS,S6491XS,S6492XS,S65001S,S65002S,S65009S,S65011S | |
| | S65012S,S65019S,S65091S,S65092S,S65099S,S65101S,S65102S | |
| | S65109S,S65111S,S65112S,S65119S,S65191S,S65192S,S65199S | |
| | S65201S,S65202S,S65209S,S65211S,S65212S,S65219S,S65291S | |
| | S65292S,S65299S,S65301S,S65302S,S65309S,S65311S,S65312S | |
| | S65319S,S65391S,S65392S,S65399S,S65401S,S65402S,S65409S | |
| | S65411S,S65412S,S65419S,S65491S,S65492S,S65499S,S65500S | |
| | S65501S,S65502S,S65503S,S65504S,S65505S,S65506S,S65507S | |
| | S65508S,S65509S,S65510S,S65511S,S65512S,S65513S,S65514S | |
| | S65515S,S65516S,S65517S,S65518S,S65519S,S65590S,S65591S | |
| | S65592S,S65593S,S65594S,S65595S,S65596S,S65597S,S65598S | |
| | S65599S,S65801S,S65802S,S65809S,S65811S,S65812S,S65819S | |
| | S65891S,S65892S,S65899S,S65901S,S65902S,S65909S,S65911S | |
| | S65912S,S65919S,S65991S,S65992S,S65999S,S66001S,S66002S | |
| | S66009S,S66011S,S66012S,S66019S,S66021S,S66022S,S66029S | |
| | S66091S,S66092S,S66099S,S66100S,S66101S,S66102S,S66103S | |
| | S66104S,S66105S,S66106S,S66107S,S66108S,S66109S,S66110S | |
| | S66111S,S66112S,S66113S,S66114S,S66115S,S66116S,S66117S | |
| | S66118S,S66119S,S66120S,S66121S,S66122S,S66123S,S66124S | |
| | S66125S,S66126S,S66127S,S66128S,S66129S,S66190S,S66191S | |
| | S66192S,S66193S,S66194S,S66195S,S66196S,S66197S,S66198S | |
| | S66199S,S66201S,S66202S,S66209S,S66211S,S66212S,S66219S | |
| | S66221S,S66222S,S66229S,S66291S,S66292S,S66299S,S66300S | |
| | S66301S,S66302S,S66303S,S66304S,S66305S,S66306S,S66307S | |
| | S66308S,S66309S,S66310S,S66311S,S66312S,S66313S,S66314S | |
| | S66315S,S66316S,S66317S,S66318S,S66319S,S66320S,S66321S | |
| | S66322S,S66323S,S66324S,S66325S,S66326S,S66327S,S66328S | |
| | S66329S,S66390S,S66391S,S66392S,S66393S,S66394S,S66395S | |
| | S66396S,S66397S,S66398S,S66399S,S66401S,S66402S,S66409S | |
| | S66411S,S66412S,S66419S,S66421S,S66422S,S66429S,S66491S | |
| | S66492S,S66499S,S66500S,S66501S,S66502S,S66503S,S66504S | |
| | S66505S,S66506S,S66507S,S66508S,S66509S,S66510S,S66511S | |
| | S66512S,S66513S,S66514S,S66515S,S66516S,S66517S,S66518S | |
| | S66519S,S66520S,S66521S,S66522S,S66523S,S66524S,S66525S | |
| | S66526S,S66527S,S66528S,S66529S,S66590S,S66591S,S66592S | |
| | S66593S,S66594S,S66595S,S66596S,S66597S,S66598S,S66599S | |
| | S66801S,S66802S,S66809S,S66811S,S66812S,S66819S,S66821S | |
| | S66822S,S66829S,S66891S,S66892S,S66899S,S66901S,S66902S | |
| | S66909S,S66911S,S66912S,S66919S,S66921S,S66922S,S66929S | |
| | S66991S,S66992S,S66999S,S6700XS,S6701XS,S6702XS,S6710XS | |
| | S67190S,S67191S,S67192S,S67193S,S67194S,S67195S,S67196S | |
| | S67197S,S67198S,S6720XS,S6721XS,S6722XS,S6730XS,S6731XS | |
| | S6732XS,S6740XS,S6741XS,S6742XS,S6790XS,S6791XS,S6792XS | |
| | S68011S,S68012S,S68019S,S68021S,S68022S,S68029S,S68110S | |
| | S68111S,S68112S,S68113S,S68114S,S68115S,S68116S,S68117S | |
| | S68118S,S68119S,S68120S,S68121S,S68122S,S68123S,S68124S | |
| | S68125S,S68126S,S68127S,S68128S,S68129S,S68411S,S68412S | |
| | S68419S,S68421S,S68422S,S68429S,S68511S,S68512S,S68519S | |
| | S68521S,S68522S,S68529S,S68610S,S68611S,S68612S,S68613S | |
| | S68614S,S68615S,S68616S,S68617S,S68618S,S68619S,S68620S | |
| | S68621S,S68622S,S68623S,S68624S,S68625S,S68626S,S68627S | |
| | S68628S,S68629S,S68711S,S68712S,S68719S,S68721S,S68722S | |
| | S68729S,S6980XS,S6981XS,S6982XS,S6990XS,S6991XS,S6992XS | |
| | S7000XS,S7001XS,S7002XS,S7010XS,S7011XS,S7012XS,S70211S | |
| | S70212S,S70219S,S70221S,S70222S,S70229S,S70241S,S70242S | |
| | S70249S,S70251S,S70252S,S70259S,S70261S,S70262S,S70269S | |
| | S70271S,S70272S,S70279S,S70311S,S70312S,S70319S,S70321S | |
| | S70322S,S70329S,S70341S,S70342S,S70349S,S70351S,S70352S | |
| | S70359S,S70361S,S70362S,S70369S,S70371S,S70372S,S70379S | |
| | S70911S,S70912S,S70919S,S70921S,S70922S,S70929S,S71001S | |
| | S71002S,S71009S,S71011S,S71012S,S71019S,S71021S,S71022S | |
| | S71029S,S71031S,S71032S,S71039S,S71041S,S71042S,S71049S | |
| | S71051S,S71052S,S71059S,S71101S,S71102S,S71109S,S71111S | |
| | S71112S,S71119S,S71121S,S71122S,S71129S,S71131S,S71132S | |
| | S71139S,S71141S,S71142S,S71149S,S71151S,S71152S,S71159S | |
| | S72001S,S72002S,S72009S,S72011S,S72012S,S72019S,S72021S | |
| | S72022S,S72023S,S72024S,S72025S,S72026S,S72031S,S72032S | |
| | S72033S,S72034S,S72035S,S72036S,S72041S,S72042S,S72043S | |
| | S72044S,S72045S,S72046S,S72051S,S72052S,S72059S,S72061S | |
| | S72062S,S72063S,S72064S,S72065S,S72066S,S72091S,S72092S | |
| | S72099S,S72101S,S72102S,S72109S,S72111S,S72112S,S72113S | |
| | S72114S,S72115S,S72116S,S72121S,S72122S,S72123S,S72124S | |
| | S72125S,S72126S,S72131S,S72132S,S72133S,S72134S,S72135S | |
| | S72136S,S72141S,S72142S,S72143S,S72144S,S72145S,S72146S | |
| | S7221XS,S7222XS,S7223XS,S7224XS,S7225XS,S7226XS,S72301S | |
| | S72302S,S72309S,S72321S,S72322S,S72323S,S72324S,S72325S | |
| | S72326S,S72331S,S72332S,S72333S,S72334S,S72335S,S72336S | |
| | S72341S,S72342S,S72343S,S72344S,S72345S,S72346S,S72351S | |
| | S72352S,S72353S,S72354S,S72355S,S72356S,S72361S,S72362S | |
| | S72363S,S72364S,S72365S,S72366S,S72391S,S72392S,S72399S | |
| | S72401S,S72402S,S72409S,S72411S,S72412S,S72413S,S72414S | |
| | S72415S,S72416S,S72421S,S72422S,S72423S,S72424S,S72425S | |

Continued on next page

Table S9 (continued).

| Diagnosis | ICD-10 | ICD-9 |
|---|---|---|
| | S72426S,S72431S,S72432S,S72433S,S72434S,S72435S,S72436S | |
| | S72441S,S72442S,S72443S,S72444S,S72445S,S72446S,S72451S | |
| | S72452S,S72453S,S72454S,S72455S,S72456S,S72461S,S72462S | |
| | S72463S,S72464S,S72465S,S72466S,S72471S,S72472S,S72479S | |
| | S72491S,S72492S,S72499S,S728X1S,S728X2S,S728X9S,S7290XS | |
| | S7291XS,S7292XS,S73001S,S73002S,S73003S,S73004S,S73005S | |
| | S73006S,S73011S,S73012S,S73013S,S73014S,S73015S,S73016S | |
| | S73021S,S73022S,S73023S,S73024S,S73025S,S73026S,S73031S | |
| | S73032S,S73033S,S73034S,S73035S,S73036S,S73041S,S73042S | |
| | S73043S,S73044S,S73045S,S73046S,S73101S,S73102S,S73109S | |
| | S73111S,S73112S,S73119S,S73121S,S73122S,S73129S,S73191S | |
| | S73192S,S73199S,S7400XS,S7401XS,S7402XS,S7410XS,S7411XS | |
| | S7412XS,S7420XS,S7421XS,S7422XS,S748X1S,S748X2S,S748X9S | |
| | S7490XS,S7491XS,S7492XS,S75001S,S75002S,S75009S,S75011S | |
| | S75012S,S75019S,S75021S,S75022S,S75029S,S75091S,S75092S | |
| | S75099S,S75101S,S75102S,S75109S,S75111S,S75112S,S75119S | |
| | S75121S,S75122S,S75129S,S75191S,S75192S,S75199S,S75201S | |
| | S75202S,S75209S,S75211S,S75212S,S75219S,S75221S,S75222S | |
| | S75229S,S75291S,S75292S,S75299S,S75801S,S75802S,S75809S | |
| | S75811S,S75812S,S75819S,S75891S,S75892S,S75899S,S75901S | |
| | S75902S,S75909S,S75911S,S75912S,S75919S,S75991S,S75992S | |
| | S75999S,S76001S,S76002S,S76009S,S76011S,S76012S,S76019S | |
| | S76021S,S76022S,S76029S,S76091S,S76092S,S76099S,S76101S | |
| | S76102S,S76109S,S76111S,S76112S,S76119S,S76121S,S76122S | |
| | S76129S,S76191S,S76192S,S76199S,S76201S,S76202S,S76209S | |
| | S76211S,S76212S,S76219S,S76221S,S76222S,S76229S,S76291S | |
| | S76292S,S76299S,S76301S,S76302S,S76309S,S76311S,S76312S | |
| | S76319S,S76321S,S76322S,S76329S,S76391S,S76392S,S76399S | |
| | S76801S,S76802S,S76809S,S76811S,S76812S,S76819S,S76821S | |
| | S76822S,S76829S,S76891S,S76892S,S76899S,S76901S,S76902S | |
| | S76909S,S76911S,S76912S,S76919S,S76921S,S76922S,S76929S | |
| | S76991S,S76992S,S76999S,S7700XS,S7701XS,S7702XS,S7710XS | |
| | S7711XS,S7712XS,S7720XS,S7721XS,S7722XS,S78011S,S78012S | |
| | S78019S,S78021S,S78022S,S78029S,S78111S,S78112S,S78119S | |
| | S78121S,S78122S,S78129S,S78911S,S78912S,S78919S,S78921S | |
| | S78922S,S78929S,S79001S,S79002S,S79009S,S79011S,S79012S | |
| | S79019S,S79091S,S79092S,S79099S,S79101S,S79102S,S79109S | |
| | S79111S,S79112S,S79119S,S79121S,S79122S,S79129S,S79131S | |
| | S79132S,S79139S,S79141S,S79142S,S79149S,S79191S,S79192S | |
| | S79199S,S79811S,S79812S,S79819S,S79821S,S79822S,S79829S | |
| | S79911S,S79912S,S79919S,S79921S,S79922S,S79929S,S8000XS | |
| | S8001XS,S8002XS,S8010XS,S8011XS,S8012XS,S80211S,S80212S | |
| | S80219S,S80221S,S80222S,S80229S,S80241S,S80242S,S80249S | |
| | S80251S,S80252S,S80259S,S80261S,S80262S,S80269S,S80271S | |
| | S80272S,S80279S,S80811S,S80812S,S80819S,S80821S,S80822S | |
| | S80829S,S80841S,S80842S,S80849S,S80851S,S80852S,S80859S | |
| | S80861S,S80862S,S80869S,S80871S,S80872S,S80879S,S80911S | |
| | S80912S,S80919S,S80921S,S80922S,S80929S,S81001S,S81002S | |
| | S81009S,S81011S,S81012S,S81019S,S81021S,S81022S,S81029S | |
| | S81031S,S81032S,S81039S,S81041S,S81042S,S81049S,S81051S | |
| | S81052S,S81059S,S81801S,S81802S,S81809S,S81811S,S81812S | |
| | S81819S,S81821S,S81822S,S81829S,S81831S,S81832S,S81839S | |
| | S81841S,S81842S,S81849S,S81851S,S81852S,S81859S,S82001S | |
| | S82002S,S82009S,S82011S,S82012S,S82013S,S82014S,S82015S | |
| | S82016S,S82021S,S82022S,S82023S,S82024S,S82025S,S82026S | |
| | S82031S,S82032S,S82033S,S82034S,S82035S,S82036S,S82041S | |
| | S82042S,S82043S,S82044S,S82045S,S82046S,S82091S,S82092S | |
| | S82099S,S82101S,S82102S,S82109S,S82111S,S82112S,S82113S | |
| | S82114S,S82115S,S82116S,S82121S,S82122S,S82123S,S82124S | |
| | S82125S,S82126S,S82131S,S82132S,S82133S,S82134S,S82135S | |
| | S82136S,S82141S,S82142S,S82143S,S82144S,S82145S,S82146S | |
| | S82151S,S82152S,S82153S,S82154S,S82155S,S82156S,S82161S | |
| | S82162S,S82169S,S82191S,S82192S,S82199S,S82201S,S82202S | |
| | S82209S,S82221S,S82222S,S82223S,S82224S,S82225S,S82226S | |
| | S82231S,S82232S,S82233S,S82234S,S82235S,S82236S,S82241S | |
| | S82242S,S82243S,S82244S,S82245S,S82246S,S82251S,S82252S | |
| | S82253S,S82254S,S82255S,S82256S,S82261S,S82262S,S82263S | |
| | S82264S,S82265S,S82266S,S82291S,S82292S,S82299S,S82301S | |
| | S82302S,S82309S,S82311S,S82312S,S82319S,S82391S,S82392S | |
| | S82399S,S82401S,S82402S,S82409S,S82421S,S82422S,S82423S | |
| | S82424S,S82425S,S82426S,S82431S,S82432S,S82433S,S82434S | |
| | S82435S,S82436S,S82441S,S82442S,S82443S,S82444S,S82445S | |
| | S82446S,S82451S,S82452S,S82453S,S82454S,S82455S,S82456S | |
| | S82461S,S82462S,S82463S,S82464S,S82465S,S82466S,S82491S | |
| | S82492S,S82499S,S8251XS,S8252XS,S8253XS,S8254XS,S8255XS | |
| | S8256XS,S8261XS,S8262XS,S8263XS,S8264XS,S8265XS,S8266XS | |
| | S82811S,S82812S,S82819S,S82821S,S82822S,S82829S,S82831S | |
| | S82832S,S82839S,S82841S,S82842S,S82843S,S82844S,S82845S | |
| | S82846S,S82851S,S82852S,S82853S,S82854S,S82855S,S82856S | |
| | S82861S,S82862S,S82863S,S82864S,S82865S,S82866S,S82871S | |
| | S82872S,S82873S,S82874S,S82875S,S82876S,S82891S,S82892S | |
| | S82899S,S8290XS,S8291XS,S8292XS,S83001S,S83002S,S83003S | |
| | S83004S,S83005S,S83006S,S83011S,S83012S,S83013S,S83014S | |
| | S83015S,S83016S,S83091S,S83092S,S83093S,S83094S,S83095S | |
| | S83096S,S83101S,S83102S,S83103S,S83104S,S83105S,S83106S | |

Continued on next page

Table S9 (continued).

| Diagnosis | ICD-10 | ICD-9 |
|---|---|---|
| | S83111S,S83112S,S83113S,S83114S,S83115S,S83116S,S83121S | |
| | S83122S,S83123S,S83124S,S83125S,S83126S,S83131S,S83132S | |
| | S83133S,S83134S,S83135S,S83136S,S83141S,S83142S,S83143S | |
| | S83144S,S83145S,S83146S,S83191S,S83192S,S83193S,S83194S | |
| | S83195S,S83196S,S83200S,S83201S,S83202S,S83203S,S83204S | |
| | S83205S,S83206S,S83207S,S83209S,S83211S,S83212S,S83219S | |
| | S83221S,S83222S,S83229S,S83231S,S83232S,S83239S,S83241S | |
| | S83242S,S83249S,S83251S,S83252S,S83259S,S83261S,S83262S | |
| | S83269S,S83271S,S83272S,S83279S,S83281S,S83282S,S83289S | |
| | S8330XS,S8331XS,S8332XS,S83401S,S83402S,S83409S,S83411S | |
| | S83412S,S83419S,S83421S,S83422S,S83429S,S83501S,S83502S | |
| | S83509S,S83511S,S83512S,S83519S,S83521S,S83522S,S83529S | |
| | S8360XS,S8361XS,S8362XS,S838X1S,S838X2S,S838X9S,S8390XS | |
| | S8391XS,S8392XS,S8400XS,S8401XS,S8402XS,S8410XS,S8411XS | |
| | S8412XS,S8420XS,S8421XS,S8422XS,S84801S,S84802S,S84809S | |
| | S8490XS,S8491XS,S8492XS,S85001S,S85002S,S85009S,S85011S | |
| | S85012S,S85019S,S85091S,S85092S,S85099S,S85101S,S85102S | |
| | S85109S,S85111S,S85112S,S85119S,S85121S,S85122S,S85129S | |
| | S85131S,S85132S,S85139S,S85141S,S85142S,S85149S,S85151S | |
| | S85152S,S85159S,S85161S,S85162S,S85169S,S85171S,S85172S | |
| | S85179S,S85181S,S85182S,S85189S,S85201S,S85202S,S85209S | |
| | S85211S,S85212S,S85219S,S85291S,S85292S,S85299S,S85301S | |
| | S85302S,S85309S,S85311S,S85312S,S85319S,S85391S,S85392S | |
| | S85399S,S85401S,S85402S,S85409S,S85411S,S85412S,S85419S | |
| | S85491S,S85492S,S85499S,S85501S,S85502S,S85509S,S85511S | |
| | S85512S,S85519S,S85591S,S85592S,S85599S,S85801S,S85802S | |
| | S85809S,S85811S,S85812S,S85819S,S85891S,S85892S,S85899S | |
| | S85901S,S85902S,S85909S,S85911S,S85912S,S85919S,S85991S | |
| | S85992S,S85999S,S86001S,S86002S,S86009S,S86011S,S86012S | |
| | S86019S,S86021S,S86022S,S86029S,S86091S,S86092S,S86099S | |
| | S86101S,S86102S,S86109S,S86111S,S86112S,S86119S,S86121S | |
| | S86122S,S86129S,S86191S,S86192S,S86199S,S86201S,S86202S | |
| | S86209S,S86211S,S86212S,S86219S,S86221S,S86222S,S86229S | |
| | S86291S,S86292S,S86299S,S86301S,S86302S,S86309S,S86311S | |
| | S86312S,S86319S,S86321S,S86322S,S86329S,S86391S,S86392S | |
| | S86399S,S86801S,S86802S,S86809S,S86811S,S86812S,S86819S | |
| | S86821S,S86822S,S86829S,S86891S,S86892S,S86899S,S86901S | |
| | S86902S,S86909S,S86911S,S86912S,S86919S,S86921S,S86922S | |
| | S86929S,S86991S,S86992S,S86999S,S8700XS,S8701XS,S8702XS | |
| | S8780XS,S8781XS,S8782XS,S88011S,S88012S,S88019S,S88021S | |
| | S88022S,S88029S,S88111S,S88112S,S88119S,S88121S,S88122S | |
| | S88129S,S88911S,S88912S,S88919S,S88921S,S88922S,S88929S | |
| | S89001S,S89002S,S89009S,S89011S,S89012S,S89019S,S89021S | |
| | S89022S,S89029S,S89031S,S89032S,S89039S,S89041S,S89042S | |
| | S89049S,S89091S,S89092S,S89099S,S89101S,S89102S,S89109S | |
| | S89111S,S89112S,S89119S,S89121S,S89122S,S89129S,S89131S | |
| | S89132S,S89139S,S89141S,S89142S,S89149S,S89191S,S89192S | |
| | S89199S,S89201S,S89202S,S89209S,S89211S,S89212S,S89219S | |
| | S89221S,S89222S,S89229S,S89291S,S89292S,S89299S,S89301S | |
| | S89302S,S89309S,S89311S,S89312S,S89319S,S89321S,S89322S | |
| | S89329S,S89391S,S89392S,S89399S,S8980XS,S8981XS,S8982XS | |
| | S8990XS,S8991XS,S8992XS,S9000XS,S9001XS,S9002XS,S90111S | |
| | S90112S,S90119S,S90121S,S90122S,S90129S,S90211S,S90212S | |
| | S90219S,S90221S,S90222S,S90229S,S9030XS,S9031XS,S9032XS | |
| | S90411S,S90412S,S90413S,S90414S,S90415S,S90416S,S90421S | |
| | S90422S,S90423S,S90424S,S90425S,S90426S,S90441S,S90442S | |
| | S90443S,S90444S,S90445S,S90446S,S90451S,S90452S,S90453S | |
| | S90454S,S90455S,S90456S,S90461S,S90462S,S90463S,S90464S | |
| | S90465S,S90466S,S90471S,S90472S,S90473S,S90474S,S90475S | |
| | S90476S,S90511S,S90512S,S90519S,S90521S,S90522S,S90529S | |
| | S90541S,S90542S,S90549S,S90551S,S90552S,S90559S,S90561S | |
| | S90562S,S90569S,S90571S,S90572S,S90579S,S90811S,S90812S | |
| | S90819S,S90821S,S90822S,S90829S,S90841S,S90842S,S90849S | |
| | S90851S,S90852S,S90859S,S90861S,S90862S,S90869S,S90871S | |
| | S90872S,S90879S,S90911S,S90912S,S90919S,S90921S,S90922S | |
| | S90929S,S90931S,S90932S,S90933S,S90934S,S90935S,S90936S | |
| | S91001S,S91002S,S91009S,S91011S,S91012S,S91019S,S91021S | |
| | S91022S,S91029S,S91031S,S91032S,S91039S,S91041S,S91042S | |
| | S91049S,S91051S,S91052S,S91059S,S91101S,S91102S,S91103S | |
| | S91104S,S91105S,S91106S,S91109S,S91111S,S91112S,S91113S | |
| | S91114S,S91115S,S91116S,S91119S,S91121S,S91122S,S91123S | |
| | S91124S,S91125S,S91126S,S91129S,S91131S,S91132S,S91133S | |
| | S91134S,S91135S,S91136S,S91139S,S91141S,S91142S,S91143S | |
| | S91144S,S91145S,S91146S,S91149S,S91151S,S91152S,S91153S | |
| | S91154S,S91155S,S91156S,S91159S,S91201S,S91202S,S91203S | |
| | S91204S,S91205S,S91206S,S91209S,S91211S,S91212S,S91213S | |
| | S91214S,S91215S,S91216S,S91219S,S91221S,S91222S,S91223S | |
| | S91224S,S91225S,S91226S,S91229S,S91231S,S91232S,S91233S | |
| | S91234S,S91235S,S91236S,S91239S,S91241S,S91242S,S91243S | |
| | S91244S,S91245S,S91246S,S91249S,S91251S,S91252S,S91253S | |
| | S91254S,S91255S,S91256S,S91259S,S91301S,S91302S,S91309S | |
| | S91311S,S91312S,S91319S,S91321S,S91322S,S91329S,S91331S | |
| | S91332S,S91339S,S91341S,S91342S,S91349S,S91351S,S91352S | |
| | S91359S,S92001S,S92002S,S92009S,S92011S,S92012S,S92013S | |
| | S92014S,S92015S,S92016S,S92021S,S92022S,S92023S,S92024S | |

Continued on next page

Table S9 (continued).

| Diagnosis | ICD-10 | ICD-9 |
|---|---|---|
| | S92025S,S92026S,S92031S,S92032S,S92033S,S92034S,S92035S | |
| | S92036S,S92041S,S92042S,S92043S,S92044S,S92045S,S92046S | |
| | S92051S,S92052S,S92053S,S92054S,S92055S,S92056S,S92061S | |
| | S92062S,S92063S,S92064S,S92065S,S92066S,S92101S,S92102S | |
| | S92109S,S92111S,S92112S,S92113S,S92114S,S92115S,S92116S | |
| | S92121S,S92122S,S92123S,S92124S,S92125S,S92126S,S92131S | |
| | S92132S,S92133S,S92134S,S92135S,S92136S,S92141S,S92142S | |
| | S92143S,S92144S,S92145S,S92146S,S92151S,S92152S,S92153S | |
| | S92154S,S92155S,S92156S,S92191S,S92192S,S92199S,S92201S | |
| | S92202S,S92209S,S92211S,S92212S,S92213S,S92214S,S92215S | |
| | S92216S,S92221S,S92222S,S92223S,S92224S,S92225S,S92226S | |
| | S92231S,S92232S,S92233S,S92234S,S92235S,S92236S,S92241S | |
| | S92242S,S92243S,S92244S,S92245S,S92246S,S92251S,S92252S | |
| | S92253S,S92254S,S92255S,S92256S,S92301S,S92302S,S92309S | |
| | S92311S,S92312S,S92313S,S92314S,S92315S,S92316S,S92321S | |
| | S92322S,S92323S,S92324S,S92325S,S92326S,S92331S,S92332S | |
| | S92333S,S92334S,S92335S,S92336S,S92341S,S92342S,S92343S | |
| | S92344S,S92345S,S92346S,S92351S,S92352S,S92353S,S92354S | |
| | S92355S,S92356S,S92401S,S92402S,S92403S,S92404S,S92405S | |
| | S92406S,S92411S,S92412S,S92413S,S92414S,S92415S,S92416S | |
| | S92421S,S92422S,S92423S,S92424S,S92425S,S92426S,S92491S | |
| | S92492S,S92499S,S92501S,S92502S,S92503S,S92504S,S92505S | |
| | S92506S,S92511S,S92512S,S92513S,S92514S,S92515S,S92516S | |
| | S92521S,S92522S,S92523S,S92524S,S92525S,S92526S,S92531S | |
| | S92532S,S92533S,S92534S,S92535S,S92536S,S92591S,S92592S | |
| | S92599S,S92811S,S92812S,S92819S,S92901S,S92902S,S92909S | |
| | S92911S,S92912S,S92919S,S9301XS,S9302XS,S9303XS,S9304XS | |
| | S9305XS,S9306XS,S93101S,S93102S,S93103S,S93104S,S93105S | |
| | S93106S,S93111S,S93112S,S93113S,S93114S,S93115S,S93116S | |
| | S93119S,S93121S,S93122S,S93123S,S93124S,S93125S,S93126S | |
| | S93129S,S93131S,S93132S,S93133S,S93134S,S93135S,S93136S | |
| | S93139S,S93141S,S93142S,S93143S,S93144S,S93145S,S93146S | |
| | S93149S,S93301S,S93302S,S93303S,S93304S,S93305S,S93306S | |
| | S93311S,S93312S,S93313S,S93314S,S93315S,S93316S,S93321S | |
| | S93322S,S93323S,S93324S,S93325S,S93326S,S93331S,S93332S | |
| | S93333S,S93334S,S93335S,S93336S,S93401S,S93402S,S93409S | |
| | S93411S,S93412S,S93419S,S93421S,S93422S,S93429S,S93431S | |
| | S93432S,S93439S,S93491S,S93492S,S93499S,S93501S,S93502S | |
| | S93503S,S93504S,S93505S,S93506S,S93509S,S93511S,S93512S | |
| | S93513S,S93514S,S93515S,S93516S,S93519S,S93521S,S93522S | |
| | S93523S,S93524S,S93525S,S93526S,S93529S,S93601S,S93602S | |
| | S93609S,S93611S,S93612S,S93619S,S93621S,S93622S,S93629S | |
| | S93691S,S93692S,S93699S,S9400XS,S9401XS,S9402XS,S9410XS | |
| | S9411XS,S9412XS,S9420XS,S9421XS,S9422XS,S9430XS,S9431XS | |
| | S9432XS,S948X1S,S948X2S,S948X9S,S9490XS,S9491XS,S9492XS | |
| | S95001S,S95002S,S95009S,S95011S,S95012S,S95019S,S95091S | |
| | S95092S,S95099S,S95101S,S95102S,S95109S,S95111S,S95112S | |
| | S95119S,S95191S,S95192S,S95199S,S95201S,S95202S,S95209S | |
| | S95211S,S95212S,S95219S,S95291S,S95292S,S95299S,S95801S | |
| | S95802S,S95809S,S95811S,S95812S,S95819S,S95891S,S95892S | |
| | S95899S,S95901S,S95902S,S95909S,S95911S,S95912S,S95919S | |
| | S95991S,S95992S,S95999S,S96001S,S96002S,S96009S,S96011S | |
| | S96012S,S96019S,S96021S,S96022S,S96029S,S96091S,S96092S | |
| | S96099S,S96101S,S96102S,S96109S,S96111S,S96112S,S96119S | |
| | S96121S,S96122S,S96129S,S96191S,S96192S,S96199S,S96201S | |
| | S96202S,S96209S,S96211S,S96212S,S96219S,S96221S,S96222S | |
| | S96229S,S96291S,S96292S,S96299S,S96801S,S96802S,S96809S | |
| | S96811S,S96812S,S96819S,S96821S,S96822S,S96829S,S96891S | |
| | S96892S,S96899S,S96901S,S96902S,S96909S,S96911S,S96912S | |
| | S96919S,S96921S,S96922S,S96929S,S96991S,S96992S,S96999S | |
| | S9700XS,S9701XS,S9702XS,S97101S,S97102S,S97109S,S97111S | |
| | S97112S,S97119S,S97121S,S97122S,S97129S,S9780XS,S9781XS | |
| | S9782XS,S98011S,S98012S,S98019S,S98021S,S98022S,S98029S | |
| | S98111S,S98112S,S98119S,S98121S,S98122S,S98129S,S98131S | |
| | S98132S,S98139S,S98141S,S98142S,S98149S,S98211S,S98212S | |
| | S98219S,S98221S,S98222S,S98229S,S98311S,S98312S,S98319S | |
| | S98321S,S98322S,S98329S,S98911S,S98912S,S98919S,S98921S | |
| | S98922S,S98929S,S99001S,S99002S,S99009S,S99011S,S99012S | |
| | S99019S,S99021S,S99022S,S99029S,S99031S,S99032S,S99039S | |
| | S99041S,S99042S,S99049S,S99091S,S99092S,S99099S,S99101S | |
| | S99102S,S99109S,S99111S,S99112S,S99119S,S99121S,S99122S | |
| | S99129S,S99131S,S99132S,S99139S,S99141S,S99142S,S99149S | |
| | S99191S,S99192S,S99199S,S99201S,S99202S,S99209S,S99211S | |
| | S99212S,S99219S,S99221S,S99222S,S99229S,S99231S,S99232S | |
| | S99239S,S99241S,S99242S,S99249S,S99291S,S99292S,S99299S | |
| | S99811S,S99812S,S99819S,S99821S,S99822S,S99829S,S99911S | |
| | S99912S,S99919S,S99921S,S99922S,S99929S,T07XXXS,T148XXS | |
| | T1490XS,T1491XS,T1500XS,T1501XS,T1502XS,T1510XS,T1511XS | |
| | T1512XS,T1580XS,T1581XS,T1582XS,T1590XS,T1591XS,T1592XS | |
| | T161XXS,T162XXS,T169XXS,T170XXS,T171XXS,T17200S,T17208S | |
| | T17210S,T17218S,T17220S,T17228S,T17290S,T17298S,T17300S | |
| | T17308S,T17310S,T17318S,T17320S,T17328S,T17390S,T17398S | |
| | T17400S,T17408S,T17410S,T17418S,T17420S,T17428S,T17490S | |
| | T17498S,T17500S,T17508S,T17510S,T17518S,T17520S,T17528S | |
| | T17590S,T17598S,T17800S,T17808S,T17810S,T17818S,T17820S | |

Continued on next page

Table S9 (continued).

| Diagnosis | ICD-10 | ICD-9 |
|---|---|---|
| | T17828S,T17890S,T17898S,T17900S,T17908S,T17910S,T17918S | |
| | T17920S,T17928S,T17990S,T17998S,T180XXS,T18100S,T18108S | |
| | T18110S,T18118S,T18120S,T18128S,T18190S,T18198S,T182XXS | |
| | T183XXS,T184XXS,T185XXS,T188XXS,T189XXS,T190XXS,T191XXS | |
| | T192XXS,T193XXS,T194XXS,T198XXS,T199XXS,T2000XS,T20011S | |
| | T20012S,T20019S,T2002XS,T2003XS,T2004XS,T2005XS,T2006XS | |
| | T2007XS,T2009XS,T2010XS,T20111S,T20112S,T20119S,T2012XS | |
| | T2013XS,T2014XS,T2015XS,T2016XS,T2017XS,T2019XS,T2020XS | |
| | T20211S,T20212S,T20219S,T2022XS,T2023XS,T2024XS,T2025XS | |
| | T2026XS,T2027XS,T2029XS,T2030XS,T20311S,T20312S,T20319S | |
| | T2032XS,T2033XS,T2034XS,T2035XS,T2036XS,T2037XS,T2039XS | |
| | T2040XS,T20411S,T20412S,T20419S,T2042XS,T2043XS,T2044XS | |
| | T2045XS,T2046XS,T2047XS,T2049XS,T2050XS,T20511S,T20512S | |
| | T20519S,T2052XS,T2053XS,T2054XS,T2055XS,T2056XS,T2057XS | |
| | T2059XS,T2060XS,T20611S,T20612S,T20619S,T2062XS,T2063XS | |
| | T2064XS,T2065XS,T2066XS,T2067XS,T2069XS,T2070XS,T20711S | |
| | T20712S,T20719S,T2072XS,T2073XS,T2074XS,T2075XS,T2076XS | |
| | T2077XS,T2079XS,T2100XS,T2101XS,T2102XS,T2103XS,T2104XS | |
| | T2105XS,T2106XS,T2107XS,T2109XS,T2110XS,T2111XS,T2112XS | |
| | T2113XS,T2114XS,T2115XS,T2116XS,T2117XS,T2119XS,T2120XS | |
| | T2121XS,T2122XS,T2123XS,T2124XS,T2125XS,T2126XS,T2127XS | |
| | T2129XS,T2130XS,T2131XS,T2132XS,T2133XS,T2134XS,T2135XS | |
| | T2136XS,T2137XS,T2139XS,T2140XS,T2141XS,T2142XS,T2143XS | |
| | T2144XS,T2145XS,T2146XS,T2147XS,T2149XS,T2150XS,T2151XS | |
| | T2152XS,T2153XS,T2154XS,T2155XS,T2156XS,T2157XS,T2159XS | |
| | T2160XS,T2161XS,T2162XS,T2163XS,T2164XS,T2165XS,T2166XS | |
| | T2167XS,T2169XS,T2170XS,T2171XS,T2172XS,T2173XS,T2174XS | |
| | T2175XS,T2176XS,T2177XS,T2179XS,T2200XS,T22011S,T22012S | |
| | T22019S,T22021S,T22022S,T22029S,T22031S,T22032S,T22039S | |
| | T22041S,T22042S,T22049S,T22051S,T22052S,T22059S,T22061S | |
| | T22062S,T22069S,T22091S,T22092S,T22099S,T2210XS,T22111S | |
| | T22112S,T22119S,T22121S,T22122S,T22129S,T22131S,T22132S | |
| | T22139S,T22141S,T22142S,T22149S,T22151S,T22152S,T22159S | |
| | T22161S,T22162S,T22169S,T22191S,T22192S,T22199S,T2220XS | |
| | T22211S,T22212S,T22219S,T22221S,T22222S,T22229S,T22231S | |
| | T22232S,T22239S,T22241S,T22242S,T22249S,T22251S,T22252S | |
| | T22259S,T22261S,T22262S,T22269S,T22291S,T22292S,T22299S | |
| | T2230XS,T22311S,T22312S,T22319S,T22321S,T22322S,T22329S | |
| | T22331S,T22332S,T22339S,T22341S,T22342S,T22349S,T22351S | |
| | T22352S,T22359S,T22361S,T22362S,T22369S,T22391S,T22392S | |
| | T22399S,T2240XS,T22411S,T22412S,T22419S,T22421S,T22422S | |
| | T22429S,T22431S,T22432S,T22439S,T22441S,T22442S,T22449S | |
| | T22451S,T22452S,T22459S,T22461S,T22462S,T22469S,T22491S | |
| | T22492S,T22499S,T2250XS,T22511S,T22512S,T22519S,T22521S | |
| | T22522S,T22529S,T22531S,T22532S,T22539S,T22541S,T22542S | |
| | T22549S,T22551S,T22552S,T22559S,T22561S,T22562S,T22569S | |
| | T22591S,T22592S,T22599S,T2260XS,T22611S,T22612S,T22619S | |
| | T22621S,T22622S,T22629S,T22631S,T22632S,T22639S,T22641S | |
| | T22642S,T22649S,T22651S,T22652S,T22659S,T22661S,T22662S | |
| | T22669S,T22691S,T22692S,T22699S,T2270XS,T22711S,T22712S | |
| | T22719S,T22721S,T22722S,T22729S,T22731S,T22732S,T22739S | |
| | T22741S,T22742S,T22749S,T22751S,T22752S,T22759S,T22761S | |
| | T22762S,T22769S,T22791S,T22792S,T22799S,T23001S,T23002S | |
| | T23009S,T23011S,T23012S,T23019S,T23021S,T23022S,T23029S | |
| | T23031S,T23032S,T23039S,T23041S,T23042S,T23049S,T23051S | |
| | T23052S,T23059S,T23061S,T23062S,T23069S,T23071S,T23072S | |
| | T23079S,T23091S,T23092S,T23099S,T23101S,T23102S,T23109S | |
| | T23111S,T23112S,T23119S,T23121S,T23122S,T23129S,T23131S | |
| | T23132S,T23139S,T23141S,T23142S,T23149S,T23151S,T23152S | |
| | T23159S,T23161S,T23162S,T23169S,T23171S,T23172S,T23179S | |
| | T23191S,T23192S,T23199S,T23201S,T23202S,T23209S,T23211S | |
| | T23212S,T23219S,T23221S,T23222S,T23229S,T23231S,T23232S | |
| | T23239S,T23241S,T23242S,T23249S,T23251S,T23252S,T23259S | |
| | T23261S,T23262S,T23269S,T23271S,T23272S,T23279S,T23291S | |
| | T23292S,T23299S,T23301S,T23302S,T23309S,T23311S,T23312S | |
| | T23319S,T23321S,T23322S,T23329S,T23331S,T23332S,T23339S | |
| | T23341S,T23342S,T23349S,T23351S,T23352S,T23359S,T23361S | |
| | T23362S,T23369S,T23371S,T23372S,T23379S,T23391S,T23392S | |
| | T23399S,T23401S,T23402S,T23409S,T23411S,T23412S,T23419S | |
| | T23421S,T23422S,T23429S,T23431S,T23432S,T23439S,T23441S | |
| | T23442S,T23449S,T23451S,T23452S,T23459S,T23461S,T23462S | |
| | T23469S,T23471S,T23472S,T23479S,T23491S,T23492S,T23499S | |
| | T23501S,T23502S,T23509S,T23511S,T23512S,T23519S,T23521S | |
| | T23522S,T23529S,T23531S,T23532S,T23539S,T23541S,T23542S | |
| | T23549S,T23551S,T23552S,T23559S,T23561S,T23562S,T23569S | |
| | T23571S,T23572S,T23579S,T23591S,T23592S,T23599S,T23601S | |
| | T23602S,T23609S,T23611S,T23612S,T23619S,T23621S,T23622S | |
| | T23629S,T23631S,T23632S,T23639S,T23641S,T23642S,T23649S | |
| | T23651S,T23652S,T23659S,T23661S,T23662S,T23669S,T23671S | |
| | T23672S,T23679S,T23691S,T23692S,T23699S,T23701S,T23702S | |
| | T23709S,T23711S,T23712S,T23719S,T23721S,T23722S,T23729S | |
| | T23731S,T23732S,T23739S,T23741S,T23742S,T23749S,T23751S | |
| | T23752S,T23759S,T23761S,T23762S,T23769S,T23771S,T23772S | |
| | T23779S,T23791S,T23792S,T23799S,T24001S,T24002S,T24009S | |
| | T24011S,T24012S,T24019S,T24021S,T24022S,T24029S,T24031S | |

Continued on next page

Table S9 (continued).

| Diagnosis | ICD-10 | ICD-9 |
| --- | --- | --- |
| | T24032S,T24039S,T24091S,T24092S,T24099S,T24101S,T24102S<br>T24109S,T24111S,T24112S,T24119S,T24121S,T24122S,T24129S<br>T24131S,T24132S,T24139S,T24191S,T24192S,T24199S,T24201S<br>T24202S,T24209S,T24211S,T24212S,T24219S,T24221S,T24222S<br>T24229S,T24231S,T24232S,T24239S,T24291S,T24292S,T24299S<br>T24301S,T24302S,T24309S,T24311S,T24312S,T24319S,T24321S<br>T24322S,T24329S,T24331S,T24332S,T24339S,T24391S,T24392S<br>T24399S,T24401S,T24402S,T24409S,T24411S,T24412S,T24419S<br>T24421S,T24422S,T24429S,T24431S,T24432S,T24439S,T24491S<br>T24492S,T24499S,T24501S,T24502S,T24509S,T24511S,T24512S<br>T24519S,T24521S,T24522S,T24529S,T24531S,T24532S,T24539S<br>T24591S,T24592S,T24599S,T24601S,T24602S,T24609S,T24611S<br>T24612S,T24619S,T24621S,T24622S,T24629S,T24631S,T24632S<br>T24639S,T24691S,T24692S,T24699S,T24701S,T24702S,T24709S<br>T24711S,T24712S,T24719S,T24721S,T24722S,T24729S,T24731S<br>T24732S,T24739S,T24791S,T24792S,T24799S,T25011S,T25012S<br>T25019S,T25021S,T25022S,T25029S,T25031S,T25032S,T25039S<br>T25091S,T25092S,T25099S,T25111S,T25112S,T25119S,T25121S<br>T25122S,T25129S,T25131S,T25132S,T25139S,T25191S,T25192S<br>T25199S,T25211S,T25212S,T25219S,T25221S,T25222S,T25229S<br>T25231S,T25232S,T25239S,T25291S,T25292S,T25299S,T25311S<br>T25312S,T25319S,T25321S,T25322S,T25329S,T25331S,T25332S<br>T25339S,T25391S,T25392S,T25399S,T25411S,T25412S,T25419S<br>T25421S,T25422S,T25429S,T25431S,T25432S,T25439S,T25491S<br>T25492S,T25499S,T25511S,T25512S,T25519S,T25521S,T25522S<br>T25529S,T25531S,T25532S,T25539S,T25591S,T25592S,T25599S<br>T25611S,T25612S,T25619S,T25621S,T25622S,T25629S,T25631S<br>T25632S,T25639S,T25691S,T25692S,T25699S,T25711S,T25712S<br>T25719S,T25721S,T25722S,T25729S,T25731S,T25732S,T25739S<br>T25791S,T25792S,T25799S,T2600XS,T2601XS,T2602XS,T2610XS<br>T2611XS,T2612XS,T2620XS,T2621XS,T2622XS,T2630XS,T2631XS<br>T2632XS,T2640XS,T2641XS,T2642XS,T2650XS,T2651XS,T2652XS<br>T2660XS,T2661XS,T2662XS,T2670XS,T2671XS,T2672XS,T2680XS<br>T2681XS,T2682XS,T2690XS,T2691XS,T2692XS,T270XXS,T271XXS<br>T272XXS,T273XXS,T274XXS,T275XXS,T276XXS,T277XXS,T280XXS<br>T281XXS,T282XXS,T283XXS,T2840XS,T28411S,T28412S,T28419S<br>T2849XS,T285XXS,T286XXS,T287XXS,T288XXS,T2890XS,T28911S<br>T28912S,T28919S,T2899XS,T7800XS,T7801XS,T7802XS,T7803XS<br>T7804XS,T7805XS,T7806XS,T7807XS,T7808XS,T7809XS,T781XXS<br>T782XXS,T783XXS,T7840XS,T7841XS,T7849XS,T790XXS,T791XXS<br>T792XXS,T794XXS,T795XXS,T796XXS,T797XXS,T798XXS,T799XXS<br>T79A0XS,T79A11S,T79A12S,T79A19S,T79A21S,T79A22S,T79A29S<br>T79A3XS,T79A9XS,T84040S,T84041S,T84042S,T84043S,T84048S<br>T84049S | |
| Internal organ injury, initial encounter | S2600XA,S2601XA,S26020A,S26021A,S26022A,S2609XA,S2610XA<br>S2611XA,S2612XA,S2619XA,S2690XA,S2691XA,S2692XA,S2699XA<br>S270XXA,S271XXA,S272XXA,S27301A,S27302A,S27309A,S27311A<br>S27312A,S27319A,S27321A,S27322A,S27329A,S27331A,S27332A<br>S27339A,S27391A,S27392A,S27399A,S27401A,S27402A,S27409A<br>S27411A,S27412A,S27419A,S27421A,S27422A,S27429A,S27431A<br>S27432A,S27439A,S27491A,S27492A,S27499A,S2750XA,S2751XA<br>S2752XA,S2753XA,S2759XA,S2760XA,S2763XA,S2769XA,S27802A<br>S27803A,S27808A,S27809A,S27812A,S27813A,S27818A,S27819A<br>S27892A,S27893A,S27898A,S27899A,S279XXA,S3600XA,S36020A<br>S36021A,S36029A,S36030A,S36031A,S36032A,S36039A,S3609XA<br>S36112A,S36113A,S36114A,S36115A,S36116A,S36118A,S36119A<br>S36122A,S36123A,S36128A,S36129A,S3613XA,S36200A,S36201A<br>S36202A,S36209A,S36220A,S36221A,S36222A,S36229A,S36230A<br>S36231A,S36232A,S36239A,S36240A,S36241A,S36242A,S36249A<br>S36250A,S36251A,S36252A,S36259A,S36260A,S36261A,S36262A<br>S36269A,S36290A,S36291A,S36292A,S36299A,S3630XA,S3632XA<br>S3633XA,S3639XA,S36400A,S36408A,S36409A,S36410A,S36418A<br>S36419A,S36420A,S36428A,S36429A,S36430A,S36438A,S36439A<br>S36490A,S36498A,S36499A,S36500A,S36501A,S36502A,S36503A<br>S36508A,S36509A,S36510A,S36511A,S36512A,S36513A,S36518A<br>S36519A,S36520A,S36521A,S36522A,S36523A,S36528A,S36529A<br>S36530A,S36531A,S36532A,S36533A,S36538A,S36539A,S36590A<br>S36591A,S36592A,S36593A,S36598A,S36599A,S3660XA,S3661XA<br>S3662XA,S3663XA,S3669XA,S3681XA,S36892A,S36893A,S36898A<br>S36899A,S3690XA,S3692XA,S3693XA,S3699XA,S37001A,S37002A<br>S37009A,S37011A,S37012A,S37019A,S37021A,S37022A,S37029A<br>S37031A,S37032A,S37039A,S37041A,S37042A,S37049A,S37051A<br>S37052A,S37059A,S37061A,S37062A,S37069A,S37091A,S37092A<br>S37099A,S3710XA,S3712XA,S3713XA,S3719XA,S3720XA,S3722XA<br>S3723XA,S3729XA,S3730XA,S3732XA,S3733XA,S3739XA,S37401A<br>S37402A,S37409A,S37421A,S37422A,S37429A,S37431A,S37432A<br>S37439A,S37491A,S37492A,S37499A,S37501A,S37502A,S37509A<br>S37511A,S37512A,S37519A,S37521A,S37522A,S37529A,S37531A | 8600,8602,8604,86100,86101,86102,86103,<br>86120,86121,86122,8620<br>86221,86222,86229,8628,8630,86320,<br>86321,86329,86340,86341<br>86342,86343,86344,86345,86346,86349,<br>86381,86382,86383,86384<br>86385,86389,86400,86401,86402,86403,<br>86404,86405,86409,86500<br>86501,86502,86503,86504,86509,86600,<br>86601,86602,86603,8670<br>8672,8674,8676,8678,86800,86801,86802,<br>86803,86804,86809,8690 |

Continued on next page

Table S9 (continued).

| Diagnosis | ICD-10 | ICD-9 |
| --- | --- | --- |
| | S37532A,S37539A,S37591A,S37592A,S37599A,S3760XA,S3762XA<br>S3763XA,S3769XA,S37812A,S37813A,S37818A,S37819A,S37822A<br>S37823A,S37828A,S37829A,S37892A,S37893A,S37898A,S37899A<br>S3790XA,S3792XA,S3793XA,S3799XA,U070 | |
| Internal organ injury, subsequent encounter | S2600XD,S2601XD,S26020D,S26021D,S26022D,S2609XD,S2610XD<br>S2611XD,S2612XD,S2619XD,S2690XD,S2691XD,S2692XD,S2699XD<br>S270XXD,S271XXD,S272XXD,S27301D,S27302D,S27309D,S27311D<br>S27312D,S27319D,S27321D,S27322D,S27329D,S27331D,S27332D<br>S27339D,S27391D,S27392D,S27399D,S27401D,S27402D,S27409D<br>S27411D,S27412D,S27419D,S27421D,S27422D,S27429D,S27431D<br>S27432D,S27439D,S27491D,S27492D,S27499D,S2750XD,S2751XD<br>S2752XD,S2753XD,S2759XD,S2760XD,S2763XD,S2769XD,S27802D<br>S27803D,S27808D,S27809D,S27812D,S27813D,S27818D,S27819D<br>S27892D,S27893D,S27898D,S27899D,S279XXD,S3600XD,S36020D<br>S36021D,S36029D,S36030D,S36031D,S36032D,S36039D,S3609XD<br>S36112D,S36113D,S36114D,S36115D,S36116D,S36118D,S36119D<br>S36122D,S36123D,S36128D,S36129D,S3613XD,S36200D,S36201D<br>S36202D,S36209D,S36220D,S36221D,S36222D,S36229D,S36230D<br>S36231D,S36232D,S36239D,S36240D,S36241D,S36242D,S36249D<br>S36250D,S36251D,S36252D,S36259D,S36260D,S36261D,S36262D<br>S36269D,S36290D,S36291D,S36292D,S36299D,S3630XD,S3632XD<br>S3633XD,S3639XD,S36400D,S36408D,S36409D,S36410D,S36418D<br>S36419D,S36420D,S36428D,S36429D,S36430D,S36438D,S36439D<br>S36490D,S36498D,S36499D,S36500D,S36501D,S36502D,S36503D<br>S36508D,S36509D,S36510D,S36511D,S36512D,S36513D,S36518D<br>S36519D,S36520D,S36521D,S36522D,S36523D,S36528D,S36529D<br>S36530D,S36531D,S36532D,S36533D,S36538D,S36539D,S36590D<br>S36591D,S36592D,S36593D,S36598D,S36599D,S3660XD,S3661XD<br>S3662XD,S3663XD,S3669XD,S3681XD,S36892D,S36893D,S36898D<br>S36899D,S3690XD,S3692XD,S3693XD,S3699XD,S37001D,S37002D<br>S37009D,S37011D,S37012D,S37019D,S37021D,S37022D,S37029D<br>S37031D,S37032D,S37039D,S37041D,S37042D,S37049D,S37051D<br>S37052D,S37059D,S37061D,S37062D,S37069D,S37091D,S37092D<br>S37099D,S3710XD,S3712XD,S3713XD,S3719XD,S3720XD,S3722XD<br>S3723XD,S3729XD,S3730XD,S3732XD,S3733XD,S3739XD,S37401D<br>S37402D,S37409D,S37421D,S37422D,S37429D,S37431D,S37432D<br>S37439D,S37491D,S37492D,S37499D,S37501D,S37502D,S37509D<br>S37511D,S37512D,S37519D,S37521D,S37522D,S37529D,S37531D<br>S37532D,S37539D,S37591D,S37592D,S37599D,S3760XD,S3762XD<br>S3763XD,S3769XD,S37812D,S37813D,S37818D,S37819D,S37822D<br>S37823D,S37828D,S37829D,S37892D,S37893D,S37898D,S37899D<br>S3790XD,S3792XD,S3793XD,S3799XD | V5889 |
| Intestinal infection | A000,A001,A009,A0100,A0109,A011,A012,A013,A014,A020,A0229<br>A029,A030,A031,A032,A033,A038,A039,A040,A041,A042,A043,A044<br>A045,A046,A047,A0471,A0472,A048,A049,A060,A061,A062,A063,A064<br>A069,A070,A071,A072,A073,A074,A078,A079,A080,A0811,A0819,A082<br>A0831,A0832,A0839,A084,A088,A09,A1832,A213,A222,B3782 | 0010,0011,0019,0020,0021,0022,0023,<br>0029,0030,00329,0039,0040<br>0041,0042,0043,0048,0049,0060,0061,<br>0062,0063,0068,0069,0070<br>0071,0072,0073,0074,0075,0078,0079,<br>00800,00801,00802,00803<br>00804,00809,0082,00841,00843,00844,<br>00845,00849,0085,00861<br>00862,00863,00864,00865,00866,00867,<br>00869,0088,0090,01480<br>0211,0222,11285,1365 |
| Intestinal obstruction and ileus | K315,K4000,K4001,K4030,K4031,K4100,K4101,K4130,K4131,K420<br>K430,K433,K436,K440,K450,K460,K50012,K50112,K50812,K50912<br>K51012,K51212,K51312,K51412,K51512,K51812,K51912,K560,K561<br>K562,K563,K5641,K5649,K565,K5650,K5651,K5652,K5660,K56600<br>K56601,K56609,K5669,K56690,K56691,K56699,K567,K913,K9130<br>K9131,K9132 | 5372,5373,55010,55011,55012,55013,<br>55200,55201,55202,55203<br>5521,55220,55221,55229,5523,5528,5529,<br>5550,5551,5552,5559<br>5562,5563,5564,5565,5566,5568,5569,<br>5600,5601,5602,56030,56031<br>56032,56039,56081,56089,5609,99749 |
| Juvenile arthritis | M0800,M08011,M08012,M08019,M08021,M08022,M08029,M08031,M08032<br>M08039,M08041,M08042,M08049,M08051,M08052,M08059,M08061<br>M08062,M08069,M08071,M08072,M08079,M0808,M0809,M080A,M081<br>M0820,M08211,M08212,M08219,M08221,M08222,M08229,M08231,M08232<br>M08239,M08241,M08242,M08249,M08251,M08252,M08259,M08261<br>M08262,M08269,M08271,M08272,M08279,M0828,M0829,M082A,M083<br>M0840,M08411,M08412,M08419,M08421,M08422,M08429,M08431,M08432<br>M08439,M08441,M08442,M08449,M08451,M08452,M08459,M08461<br>M08462,M08469,M08471,M08472,M08479,M0848,M084A,M0880,M08811<br>M08812,M08819,M08821,M08822,M08829,M08831,M08832,M08839<br>M08841,M08842,M08849,M08851,M08852,M08859,M08861,M08862<br>M08869,M08871,M08872,M08879,M0888,M0889,M0890,M08911,M08912<br>M08919,M08921,M08922,M08929,M08931,M08932,M08939,M08941<br>M08942,M08949,M08951,M08952,M08959,M08961,M08962,M08969<br>M08971,M08972,M08979,M0898,M0899,M089A | 71430,71432,7200 |
| Leukemia - acute lymphoblastic leukemia (ALL) | C9100,C9101,C9102 | 20400,20401,20402 |

Continued on next page

 

Table S9 (continued).

| Diagnosis | ICD-10 | ICD-9 |
|---|---|---|
| Leukemia - acute myeloid leukemia (AML) | C9200,C9201,C9202,C9240,C9241,C9242,C9250,C9251,C9252,C9260 C9261,C9262,C92A0,C92A1,C92A2,C9300,C9301,C9302,C9400,C9401 C9402,C9420,C9421,C9422 | 20500,20501,20502,20600,20601,20602, 20700,20701,20702,20720 20721,20722 |
| Leukemia - all other types | C9130,C9131,C9132,C9150,C9151,C9152,C9160,C9161,C9162,C9190 C9191,C9192,C91A0,C91A1,C91A2,C91Z0,C91Z1,C91Z2,C9290,C9291 C9292,C92Z0,C92Z1,C92Z2,C9330,C9331,C9332,C9390,C9391,C9392 C93Z0,C93Z1,C93Z2,C9430,C9431,C9432,C9440,C9441,C9442,C946 C9480,C9481,C9482,C9500,C9501,C9502,C9510,C9511,C9512,C9590 C9591,C9592 | 20480,20481,20482,20490,20491,20492, 20580,20581,20582,20590 20591,20592,20620,20621,20622,20680, 20681,20682,20690,20691 20692,20780,20781,20782,20800,20801, 20802,20810,20811,20812 20820,20821,20822,20880,20881,20882, 20890,20891,20892,23879 |
| Leukemia - chronic lymphocytic leukemia (CLL) | C9110,C9111,C9112 | 20410,20411,20412 |
| Leukemia - chronic myeloid leukemia (CML) | C9210,C9211,C9212,C9220,C9221,C9222,C9310,C9311,C9312 | 20510,20511,20512,20520,20521,20522, 20610,20611,20612 |
| Leukemia - hairy cell | C9140,C9141,C9142 | 20240 |
| Lifestyle life management factors | Z720,Z723,Z724,Z7251,Z7252,Z7253,Z726,Z72810,Z72811,Z72820 Z72821,Z72823,Z7289,Z729,Z730,Z731,Z732,Z733,Z734,Z735,Z736 Z73810,Z73811,Z73812,Z73819,Z7382,Z7389,Z739 | V4985,V624,V6289,V690,V691,V692, V693,V694,V695,V698,V699 V7101,V7102 |
| Liveborn | Z3800,Z3801,Z381,Z382,Z3830,Z3831,Z384,Z385,Z3861,Z3862,Z3863 Z3864,Z3865,Z3866,Z3868,Z3869,Z387,Z388 | V3000,V3001,V301,V302,V3100,V3101, V311,V312,V3200,V3201,V321 V322,V3300,V3301,V331,V332,V3400, V3401,V341,V342,V3500,V3501 V351,V352,V3600,V3601,V361,V362, V3700,V3701,V371,V372,V3900 V391,V392 |
| Low back pain | M5106,M5116,M5117,M5126,M5127,M5136,M5137,M5146,M5147,M5186 M5187,M51A0,M51A1,M51A2,M51A3,M51A4,M51A5,M532X6,M532X7 M532X8,M533,M5386,M5387,M5388,M5416,M5417,M5418,M5430,M5431 M5432,M5440,M5441,M5442,M545,M5450,M5451,M5459,M62830 | 71888,72210,72232,72252,72273,72293, 7242,7243,7244,7246,72470 72471,72479,7248,7292 |
| Lung disease due to external agents | J60,J61,J620,J628,J630,J631,J632,J633,J634,J635,J636,J64,J65 J660,J661,J662,J668,J670,J671,J672,J673,J674,J675,J676,J677 J678,J679,J680,J681,J682,J683,J684,J688,J689,J691,J698,J700 J701,J702,J703,J704,J705,J708,J709,U070 | 4950,4951,4952,4953,4954,4955,4956, 4957,4958,4959,500,501,502 503,504,505,5060,5061,5062,5063,5064, 5069,5071,5078,5080,5081 5082,5088,5089 |
| Malaise and fatigue | G933,R530,R531,R5381,R5382,R5383 | 78071,78079,7993 |
| Male infertility | N4601,N46021,N46022,N46023,N46024,N46025,N46029,N4611,N46121 N46122,N46123,N46124,N46125,N46129,N468,N469 | 6060,6061,6068,6069 |
| Male reproductive system cancers - all other types | C6300,C6301,C6302,C6310,C6311,C6312,C632,C637,C638,C639,D0760 D0761,D0769 | 1875,1876,1877,1878,1879,2336 |
| Male reproductive system cancers - penis | C600,C601,C602,C608,C609,D074 | 1871,1872,1873,1874,1878,2335 |
| Male reproductive system cancers - prostate | C61,D075 | 185,2334 |
| Male reproductive system cancers - testis | C6200,C6201,C6202,C6210,C6211,C6212,C6290,C6291,C6292 | 1860,1869 |
| Malignant neoplasm, unspecified | C800,C801,C802,D099 | 1990,1991,1992,2349 |
| Malignant neuroendocrine tumors | C7A00,C7A010,C7A011,C7A012,C7A019,C7A020,C7A021,C7A022,C7A023 C7A024,C7A025,C7A026,C7A029,C7A090,C7A091,C7A092,C7A093 C7A094,C7A095,C7A096,C7A098,C7A1,C7A8,C7B00,C7B01,C7B02,C7B03 C7B04,C7B09,C7B1,C7B8 | 20900,20901,20902,20903,20910,20911, 20912,20913,20914,20915 20916,20917,20920,20921,20922,20923, 20924,20925,20926,20927 20929,20930,20970,20971,20972,20973, 20974,20975,20979 |
| Malnutrition | E40,E41,E42,E43,E440,E441,E45,E46,O2510,O2511,O2512,O2513 | 260,261,262,2630,2631,2632,2638,2639, 64890,64891,64893,64894 |

Continued on next page

Table S9 (continued).

| Diagnosis | ICD-10 | ICD-9 |
|---|---|---|
| | O252,O253 | |
| Malposition, disproportion or other labor complications | O320XX0,O320XX1,O320XX2,O320XX3,O320XX4,O320XX5,O320XX9<br>O321XX0,O321XX1,O321XX2,O321XX3,O321XX4,O321XX5,O321XX9<br>O322XX0,O322XX1,O322XX2,O322XX3,O322XX4,O322XX5,O322XX9<br>O323XX0,O323XX1,O323XX2,O323XX3,O323XX4,O323XX5,O323XX9<br>O324XX0,O324XX1,O324XX2,O324XX3,O324XX4,O324XX5,O324XX9<br>O326XX0,O326XX1,O326XX2,O326XX3,O326XX4,O326XX5,O326XX9<br>O328XX0,O328XX1,O328XX2,O328XX3,O328XX4,O328XX5,O328XX9<br>O329XX0,O329XX1,O329XX2,O329XX3,O329XX4,O329XX5,O329XX9,O330<br>O331,O332,O333XX0,O333XX1,O333XX2,O333XX3,O333XX4,O333XX5<br>O333XX9,O334XX0,O334XX1,O334XX2,O334XX3,O334XX4,O334XX5<br>O334XX9,O335XX0,O335XX1,O335XX2,O335XX3,O335XX4,O335XX5<br>O335XX9,O336XX0,O336XX1,O336XX2,O336XX3,O336XX4,O336XX5<br>O336XX9,O337,O337XX0,O337XX1,O337XX2,O337XX3,O337XX4,O337XX5<br>O337XX9,O338,O339,O610,O611,O618,O619,O620,O621,O622,O623<br>O624,O628,O629,O630,O631,O632,O639,O640XX0,O640XX1,O640XX2<br>O640XX3,O640XX4,O640XX5,O640XX9,O641XX0,O641XX1,O641XX2<br>O641XX3,O641XX4,O641XX5,O641XX9,O642XX0,O642XX1,O642XX2<br>O642XX3,O642XX4,O642XX5,O642XX9,O643XX0,O643XX1,O643XX2<br>O643XX3,O643XX4,O643XX5,O643XX9,O644XX0,O644XX1,O644XX2<br>O644XX3,O644XX4,O644XX5,O644XX9,O645XX0,O645XX1,O645XX2<br>O645XX3,O645XX4,O645XX5,O645XX9,O648XX0,O648XX1,O648XX2<br>O648XX3,O648XX4,O648XX5,O648XX9,O649XX0,O649XX1,O649XX2<br>O649XX3,O649XX4,O649XX5,O649XX9,O650,O651,O652,O653,O654,O655<br>O658,O659,O660,O661,O662,O663,O6640,O6641,O665,O666,O668,O669<br>O68,O690XX0,O690XX1,O690XX2,O690XX3,O690XX4,O690XX5,O690XX9<br>O691XX0,O691XX1,O691XX2,O691XX3,O691XX4,O691XX5,O691XX9<br>O692XX0,O692XX1,O692XX2,O692XX3,O692XX4,O692XX5,O692XX9<br>O693XX0,O693XX1,O693XX2,O693XX3,O693XX4,O693XX5,O693XX9<br>O694XX0,O694XX1,O694XX2,O694XX3,O694XX4,O694XX5,O694XX9<br>O695XX0,O695XX1,O695XX2,O695XX3,O695XX4,O695XX5,O695XX9<br>O6981X0,O6981X1,O6981X2,O6981X3,O6981X4,O6981X5,O6981X9<br>O6982X0,O6982X1,O6982X2,O6982X3,O6982X4,O6982X5,O6982X9<br>O6989X0,O6989X1,O6989X2,O6989X3,O6989X4,O6989X5,O6989X9<br>O699XX0,O699XX1,O699XX2,O699XX3,O699XX4,O699XX5,O699XX9,O771<br>O778,O779 | 64871,64872,65201,65203,65211,65221,<br>65223,65231,65233,65241<br>65243,65251,65253,65261,65281,65283,<br>65291,65293,65301,65311<br>65313,65321,65323,65331,65333,65341,<br>65343,65351,65353,65361<br>65363,65371,65373,65381,65383,65391,<br>65393,65491,65631,65681<br>65901,65911,66001,66011,66021,66031,<br>66041,66051,66061,66071<br>66081,66091,66101,66111,66121,66131,<br>66141,66191,66201,66211<br>66221,66231,66301,66311,66321,66331,<br>66341,66351,66361,66381<br>66391,66951 |
| Maltreatment abuse | O9A311,O9A312,O9A313,O9A319,O9A32,O9A33,O9A411,O9A412,O9A413<br>O9A419,O9A42,O9A43,O9A511,O9A512,O9A513,O9A519,O9A52,O9A53<br>T7401XA,T7402XA,T7411XA,T7412XA,T7421XA,T7422XA,T7431XA<br>T7432XA,T744XXA,T7451XA,T7452XA,T7461XA,T7462XA,T7491XA<br>T7492XA,T7601XA,T7602XA,T7611XA,T7612XA,T7621XA,T7622XA<br>T7631XA,T7632XA,T7651XA,T7652XA,T7661XA,T7662XA,T7691XA<br>T7692XA | 64890,64891,64893,64894,99550,99551,<br>99552,99553,99554,99555<br>99580,99581,99582,99583,99584,E9040 |
| Maltreatment abuse, subsequent encounter | T7401XD,T7402XD,T7411XD,T7412XD,T7421XD,T7422XD,T7431XD<br>T7432XD,T744XXD,T7451XD,T7452XD,T7461XD,T7462XD,T7491XD<br>T7492XD,T7601XD,T7602XD,T7611XD,T7612XD,T7621XD,T7622XD<br>T7631XD,T7632XD,T7651XD,T7652XD,T7661XD,T7662XD,T7691XD<br>T7692XD | V5889 |
| Maternal care for abnormality of pelvic organs | O3400,O3401,O3402,O3403,O3410,O3411,O3412,O3413,O3429,O3440<br>O3441,O3442,O3443,O34511,O34512,O34513,O34519,O34521,O34522<br>O34523,O34529,O34531,O34532,O34533,O34539,O34591,O34592<br>O34593,O34599,O3460,O3461,O3462,O3463,O3470,O3471,O3472,O3473<br>O3480,O3481,O3482,O3483,O3490,O3491,O3492,O3493,O7100,O7102<br>O7103 | 65400,65401,65402,65403,65404,65410,<br>65411,65412,65413,65414<br>65430,65431,65432,65433,65434,65440,<br>65441,65442,65443,65444<br>65460,65461,65462,65463,65464,65470,<br>65471,65472,65473,65474<br>65480,65481,65482,65483,65484,65490,<br>65491,65492,65493,65494<br>66500,66501,66503 |
| Maternal care related to disorders of the placenta and placental implantation | O3670X0,O3670X1,O3670X2,O3670X3,O3670X4,O3670X5,O3670X9<br>O3671X0,O3671X1,O3671X2,O3671X3,O3671X4,O3671X5,O3671X9<br>O3672X0,O3672X1,O3672X2,O3672X3,O3672X4,O3672X5,O3672X9<br>O3673X0,O3673X1,O3673X2,O3673X3,O3673X4,O3673X5,O3673X9<br>O43011,O43012,O43013,O43019,O43021,O43022,O43023,O43029<br>O43101,O43102,O43103,O43109,O43111,O43112,O43113,O43119<br>O43121,O43122,O43123,O43129,O43191,O43192,O43193,O43199<br>O43211,O43212,O43213,O43219,O43221,O43222,O43223,O43229<br>O43231,O43232,O43233,O43239,O43811,O43812,O43813,O43819<br>O43891,O43892,O43893,O43899,O4390,O4391,O4392,O4393,O4400<br>O4401,O4402,O4403,O4410,O4411,O4412,O4413,O4420,O4421,O4422<br>O4423,O4430,O4431,O4432,O4433,O4440,O4441,O4442,O4443,O4450<br>O4451,O4452,O4453,O45001,O45002,O45003,O45009,O45011,O45012 | 64100,64101,64103,64110,64111,64113,<br>64120,64121,64123,64130<br>64131,64133,65600,65601,65603,65670,<br>65671,65673,65680,65681<br>65683,66380,66381,66700,66702,66704 |

Continued on next page

Table S9 (continued).

| Diagnosis | ICD-10 | ICD-9 |
|---|---|---|
| | O45013,O45019,O45021,O45022,O45023,O45029,O45091,O45092 O45093,O45099,O458X1,O458X2,O458X3,O458X9,O4590,O4591,O4592 O4593 | |
| Maternal care related | O3500X0,O3500X1,O3500X2,O3500X3,O3500X4,O3500X5,O3500X9 | 65501,65503,65511,65513,65521,65523, 65531,65533,65541,65543 |
| to fetal conditions | O3501X0,O3501X1,O3501X2,O3501X3,O3501X4,O3501X5,O3501X9 | 65551,65553,65561,65563,65571,65573, 65581,65583,65591,65593 |
| | O3502X0,O3502X1,O3502X2,O3502X3,O3502X4,O3502X5,O3502X9 | 65610,65611,65613,65620,65621,65623, 65650,65651,65653,65660 |
| | O3503X0,O3503X1,O3503X2,O3503X3,O3503X4,O3503X5,O3503X9 | 65661,65663,65680,65681,65683,65690, 65691,65693,67800,67801 |
| | O3504X0,O3504X1,O3504X2,O3504X3,O3504X4,O3504X5,O3504X9 | 67803,67910,67911,67912,67913,67914, V2387 |
| | O3505X0,O3505X1,O3505X2,O3505X3,O3505X4,O3505X5,O3505X9 O3506X0,O3506X1,O3506X2,O3506X3,O3506X4,O3506X5,O3506X9 O3507X0,O3507X1,O3507X2,O3507X3,O3507X4,O3507X5,O3507X9 O3508X0,O3508X1,O3508X2,O3508X3,O3508X4,O3508X5,O3508X9 O3509X0,O3509X1,O3509X2,O3509X3,O3509X4,O3509X5,O3509X9 O350XX0,O350XX1,O350XX2,O350XX3,O350XX4,O350XX5,O350XX9 O3510X0,O3510X1,O3510X2,O3510X3,O3510X4,O3510X5,O3510X9 O3511X0,O3511X1,O3511X2,O3511X3,O3511X4,O3511X5,O3511X9 O3512X0,O3512X1,O3512X2,O3512X3,O3512X4,O3512X5,O3512X9 O3513X0,O3513X1,O3513X2,O3513X3,O3513X4,O3513X5,O3513X9 O3514X0,O3514X1,O3514X2,O3514X3,O3514X4,O3514X5,O3514X9 O3515X0,O3515X1,O3515X2,O3515X3,O3515X4,O3515X5,O3515X9 O3519X0,O3519X1,O3519X2,O3519X3,O3519X4,O3519X5,O3519X9 O351XX0,O351XX1,O351XX2,O351XX3,O351XX4,O351XX5,O351XX9 O352XX0,O352XX1,O352XX2,O352XX3,O352XX4,O352XX5,O352XX9 O353XX0,O353XX1,O353XX2,O353XX3,O353XX4,O353XX5,O353XX9 O354XX0,O354XX1,O354XX2,O354XX3,O354XX4,O354XX5,O354XX9 O355XX0,O355XX1,O355XX2,O355XX3,O355XX4,O355XX5,O355XX9 O356XX0,O356XX1,O356XX2,O356XX3,O356XX4,O356XX5,O356XX9 O357XX0,O357XX1,O357XX2,O357XX3,O357XX4,O357XX5,O357XX9 O358XX0,O358XX1,O358XX2,O358XX3,O358XX4,O358XX5,O358XX9 O359XX0,O359XX1,O359XX2,O359XX3,O359XX4,O359XX5,O359XX9 O35AXX0,O35AXX1,O35AXX2,O35AXX3,O35AXX4,O35AXX5,O35AXX9 O35BXX0,O35BXX1,O35BXX2,O35BXX3,O35BXX4,O35BXX5,O35BXX9 O35CXX0,O35CXX1,O35CXX2,O35CXX3,O35CXX4,O35CXX5,O35CXX9 O35DXX0,O35DXX1,O35DXX2,O35DXX3,O35DXX4,O35DXX5,O35DXX9 O35EXX0,O35EXX1,O35EXX2,O35EXX3,O35EXX4,O35EXX5,O35EXX9 O35FXX0,O35FXX1,O35FXX2,O35FXX3,O35FXX4,O35FXX5,O35FXX9 O35GXX0,O35GXX1,O35GXX2,O35GXX3,O35GXX4,O35GXX5,O35GXX9 O35HXX0,O35HXX1,O35HXX2,O35HXX3,O35HXX4,O35HXX5,O35HXX9 O360110,O360111,O360112,O360113,O360114,O360115,O360119 O360120,O360121,O360122,O360123,O360124,O360125,O360129 O360130,O360131,O360132,O360133,O360134,O360135,O360139 O360190,O360191,O360192,O360193,O360194,O360195,O360199 O360910,O360911,O360912,O360913,O360914,O360915,O360919 O360920,O360921,O360922,O360923,O360924,O360925,O360929 O360930,O360931,O360932,O360933,O360934,O360935,O360939 O360990,O360991,O360992,O360993,O360994,O360995,O360999 O361110,O361111,O361112,O361113,O361114,O361115,O361119 O361120,O361121,O361122,O361123,O361124,O361125,O361129 O361130,O361131,O361132,O361133,O361134,O361135,O361139 O361190,O361191,O361192,O361193,O361194,O361195,O361199 O361910,O361911,O361912,O361913,O361914,O361915,O361919 O361920,O361921,O361922,O361923,O361924,O361925,O361929 O361930,O361931,O361932,O361933,O361934,O361935,O361939 O361990,O361991,O361992,O361993,O361994,O361995,O361999 O3620X0,O3620X1,O3620X2,O3620X3,O3620X4,O3620X5,O3620X9 O3621X0,O3621X1,O3621X2,O3621X3,O3621X4,O3621X5,O3621X9 O3622X0,O3622X1,O3622X2,O3622X3,O3622X4,O3622X5,O3622X9 O3623X0,O3623X1,O3623X2,O3623X3,O3623X4,O3623X5,O3623X9 O365110,O365111,O365112,O365113,O365114,O365115,O365119 O365120,O365121,O365122,O365123,O365124,O365125,O365129 O365130,O365131,O365132,O365133,O365134,O365135,O365139 O365190,O365191,O365192,O365193,O365194,O365195,O365199 O365910,O365911,O365912,O365913,O365914,O365915,O365919 O365920,O365921,O365922,O365923,O365924,O365925,O365929 O365930,O365931,O365932,O365933,O365934,O365935,O365939 O365990,O365991,O365992,O365993,O365994,O365995,O365999 O3660X0,O3660X1,O3660X2,O3660X3,O3660X4,O3660X5,O3660X9 O3661X0,O3661X1,O3661X2,O3661X3,O3661X4,O3661X5,O3661X9 O3662X0,O3662X1,O3662X2,O3662X3,O3662X4,O3662X5,O3662X9 O3663X0,O3663X1,O3663X2,O3663X3,O3663X4,O3663X5,O3663X9 O3680X0,O3680X1,O3680X2,O3680X3,O3680X4,O3680X5,O3680X9 O368120,O368121,O368122,O368123,O368124,O368125,O368129 O368130,O368131,O368132,O368133,O368134,O368135,O368139 O368190,O368191,O368192,O368193,O368194,O368195,O368199 O368210,O368211,O368212,O368213,O368214,O368215,O368219 O368220,O368221,O368222,O368223,O368224,O368225,O368229 O368230,O368231,O368232,O368233,O368234,O368235,O368239 O368290,O368291,O368292,O368293,O368294,O368295,O368299 O368310,O368311,O368312,O368313,O368314,O368315,O368319 | |

Continued on next page

Table S9 (continued).

| Diagnosis | ICD-10 | ICD-9 |
|---|---|---|
| | O368320,O368321,O368322,O368323,O368324,O368325,O368329 O368330,O368331,O368332,O368333,O368334,O368335,O368339 O368390,O368391,O368392,O368393,O368394,O368395,O368399 O368910,O368911,O368912,O368913,O368914,O368915,O368919 O368920,O368921,O368922,O368923,O368924,O368925,O368929 O368930,O368931,O368932,O368933,O368934,O368935,O368939 O368990,O368991,O368992,O368993,O368994,O368995,O368999 O3690X0,O3690X1,O3690X2,O3690X3,O3690X4,O3690X5,O3690X9 O3691X0,O3691X1,O3691X2,O3691X3,O3691X4,O3691X5,O3691X9 O3692X0,O3692X1,O3692X2,O3692X3,O3692X4,O3692X5,O3692X9 O3693X0,O3693X1,O3693X2,O3693X3,O3693X4,O3693X5,O3693X9 | |
| Maternal intrauterine infection | O411010,O411011,O411012,O411013,O411014,O411015,O411019 O411020,O411021,O411022,O411023,O411024,O411025,O411029 O411030,O411031,O411032,O411033,O411034,O411035,O411039 O411090,O411091,O411092,O411093,O411094,O411095,O411099 O411210,O411211,O411212,O411213,O411214,O411215,O411219 O411220,O411221,O411222,O411223,O411224,O411225,O411229 O411230,O411231,O411232,O411233,O411234,O411235,O411239 O411290,O411291,O411292,O411293,O411294,O411295,O411299 O411410,O411411,O411412,O411413,O411414,O411415,O411419 O411420,O411421,O411422,O411423,O411424,O411425,O411429 O411430,O411431,O411432,O411433,O411434,O411435,O411439 O411490,O411491,O411492,O411493,O411494,O411495,O411499 | 65840,65841,65843 |
| Mediastinal disorders | J853,J985,J9851,J9859 | 5131,5192,5193 |
| Medical examination evaluation | Z0000,Z0001,Z00110,Z00111,Z00121,Z00129,Z002,Z003,Z005,Z006 Z0070,Z0071,Z008,Z0100,Z0101,Z01020,Z01021,Z0110,Z01110 Z01118,Z0112,Z0120,Z0121,Z0130,Z0131,Z01411,Z01419,Z0142 Z01810,Z01811,Z01812,Z01818,Z0182,Z0183,Z0184,Z0189 | V202,V2031,V2032,V210,V211,V212, V700,V706,V707,V708,V720 V7211,V7212,V7219,V722,V7231,V7232, V7261,V7263,V7281,V7282 V7283,V7284,V7285,V7286 |
| Meningitis | A0101,A0221,A170,A171,A203,A2781,A3211,A390,A4281,A5041,A5141 A5213,A5481,A6921,A870,A871,A872,A878,A879,B003,B010,B021 B051,B0602,B261,B2702,B2712,B2782,B2792,B375,B384,B4281,B451 B5741,D8681,G000,G001,G002,G003,G008,G009,G01,G02,G030,G031 G032,G038,G039 | 0020,00321,01300,01310,0208,0270,0360, 0398,0470,0471,0478 0479,0490,0491,0527,0530,05472,05579, 05609,0721,075,0861 08881,09042,09181,0942,09882,10081, 11283,1142,1171,1175,135 3200,3201,3202,3203,3207,32082,32089, 3209,3210,3211,3212,3218 3220,3221,3222,3229,99709 |
| Menopausal disorders | E28310,E28319,E2839,N924,N950,N951,N952,N958,N959 | 25631,25639,6270,6271,6272,6273,6278, 6279 |
| Menstrual disorders | N897,N910,N911,N912,N913,N914,N915,N920,N921,N922,N923,N925 N926,N944,N945,N946 | 6253,6260,6261,6262,6263,6264,6265, 6266,6268,6269 |
| Mental and substance use disorders in remission | F1011,F1021,F1111,F1121,F1211,F1221,F1311,F1321,F1411,F1421 F1511,F1521,F1611,F1621,F17201,F17211,F17221,F17291,F1811 F1821,F1911,F1921,F304,F3170,F3172,F3174,F3176,F3178,F325 F3340,F3342 | 29285,29606,29626,29630,29636,29646, 29656,29666,2967,30393 30403,30413,30423,30433,30443,30453, 30463,3051 |
| Mental and substance use disorders; sequela | T1491XS,T360X2S,T361X2S,T362X2S,T363X2S,T364X2S,T365X2S T366X2S,T367X2S,T368X2S,T3692XS,T370X2S,T371X2S,T372X2S T373X2S,T374X2S,T375X2S,T378X2S,T3792XS,T380X2S,T381X2S T382X2S,T383X2S,T384X2S,T385X2S,T386X2S,T387X2S,T38802S T38812S,T38892S,T38902S,T38992S,T39012S,T39092S,T391X2S T392X2S,T39312S,T39392S,T394X2S,T398X2S,T3992XS,T400X1S T400X2S,T400X3S,T400X4S,T400X5S,T401X1S,T401X2S,T401X3S T401X4S,T402X1S,T402X2S,T402X3S,T402X4S,T402X5S,T403X1S T403X2S,T403X3S,T403X4S,T403X5S,T40411S,T40412S,T40413S T40414S,T40415S,T40421S,T40422S,T40423S,T40424S,T40425S T40491S,T40492S,T40493S,T40494S,T40495S,T404X1S,T404X2S T404X3S,T404X4S,T404X5S,T405X1S,T405X2S,T405X3S,T405X4S T405X5S,T40601S,T40602S,T40603S,T40604S,T40605S,T40691S T40692S,T40693S,T40694S,T40695S,T40711S,T40712S,T40713S T40714S,T40715S,T40721S,T40722S,T40723S,T40724S,T40725S T407X1S,T407X2S,T407X3S,T407X4S,T407X5S,T408X1S,T408X2S T408X3S,T408X4S,T40901S,T40902S,T40903S,T40904S,T40905S T40991S,T40992S,T40993S,T40994S,T40995S,T410X1S,T410X2S T410X3S,T410X4S,T410X5S,T411X2S,T41202S,T41292S,T413X2S T4142XS,T415X2S,T420X2S,T422X2S,T423X2S,T424X2S,T425X2S T426X1S,T426X2S,T426X3S,T426X4S,T426X5S,T4271XS,T4272XS T4273XS,T4274XS,T4275XS,T428X2S,T43012S,T43022S,T431X2S T43202S,T43205S,T43212S,T43222S,T43292S,T433X2S,T434X2S T43502S,T43592S,T43601S,T43602S,T43603S,T43604S,T43605S T43611S,T43612S,T43613S,T43614S,T43615S,T43621S,T43622S T43623S,T43624S,T43625S,T43631S,T43632S,T43633S,T43634S T43635S,T43641S,T43642S,T43643S,T43644S,T43651S,T43652S | 9090,9091,9094,9095,E9292,E9351,E9352, E9363,E9378,E9379,E9382 E9390,E9396,E9397,E959,E969,E989 |

Continued on next page

Table S9 (continued).

| Diagnosis | ICD-10 | ICD-9 |
|---|---|---|
| | T43653S,T43654S,T43655S,T43691S,T43692S,T43693S,T43694S<br>T43695S,T438X2S,T4392XS,T440X2S,T441X2S,T442X2S,T443X2S<br>T444X2S,T445X2S,T446X2S,T447X2S,T448X2S,T44902S,T44992S<br>T450X2S,T451X2S,T452X2S,T453X2S,T454X2S,T45512S,T45522S<br>T45602S,T45612S,T45622S,T45692S,T457X2S,T458X2S,T4592XS<br>T460X2S,T461X2S,T462X2S,T463X2S,T464X2S,T465X2S,T466X2S<br>T467X2S,T468X2S,T46902S,T46992S,T470X2S,T471X2S,T472X2S<br>T473X2S,T474X2S,T475X2S,T476X2S,T477X2S,T478X2S,T4792XS<br>T480X2S,T481X2S,T48202S,T48292S,T483X2S,T484X2S,T485X2S<br>T486X2S,T48902S,T48992S,T490X2S,T491X2S,T492X2S,T493X2S<br>T494X2S,T495X2S,T496X2S,T497X2S,T498X2S,T4992XS,T500X2S<br>T501X2S,T502X2S,T503X2S,T504X2S,T505X2S,T506X2S,T507X2S<br>T508X2S,T50902S,T50912S,T50992S,T50A12S,T50A22S,T50A92S<br>T50B12S,T50B92S,T50Z12S,T50Z92S,T510X2S,T511X2S,T512X2S<br>T513X2S,T518X2S,T5192XS,T520X2S,T521X2S,T522X2S,T523X2S<br>T524X2S,T528X2S,T5292XS,T530X2S,T531X2S,T532X2S,T533X2S<br>T534X2S,T535X2S,T536X2S,T537X2S,T5392XS,T540X2S,T541X2S<br>T542X2S,T543X2S,T5492XS,T550X2S,T551X2S,T560X2S,T561X2S<br>T562X2S,T563X2S,T564X2S,T565X2S,T566X2S,T567X2S,T56812S<br>T56892S,T5692XS,T570X2S,T571X2S,T572X2S,T573X2S,T578X2S<br>T5792XS,T5802XS,T5812XS,T582X2S,T588X2S,T5892XS,T590X2S<br>T591X2S,T592X2S,T593X2S,T594X2S,T595X2S,T596X2S,T597X2S<br>T59812S,T59892S,T5992XS,T600X2S,T601X2S,T602X2S,T603X2S<br>T604X2S,T608X2S,T6092XS,T6102XS,T6112XS,T61772S,T61782S<br>T618X2S,T6192XS,T620X2S,T621X2S,T622X2S,T628X2S,T6292XS<br>T63002S,T63012S,T63022S,T63032S,T63042S,T63062S,T63072S<br>T63082S,T63092S,T63112S,T63122S,T63192S,T632X2S,T63302S<br>T63312S,T63322S,T63332S,T63392S,T63412S,T63422S,T63432S<br>T63442S,T63452S,T63462S,T63482S,T63512S,T63592S,T63612S<br>T63622S,T63632S,T63692S,T63712S,T63792S,T63812S,T63822S<br>T63832S,T63892S,T6392XS,T6402XS,T6482XS,T650X2S,T651X2S<br>T65212S,T65222S,T65292S,T653X2S,T654X2S,T655X2S,T656X2S<br>T65812S,T65822S,T65832S,T65892S,T6592XS,T71112S,T71122S<br>T71132S,T71152S,T71162S,T71192S,T71222S,T71232S,X710XXS<br>X711XXS,X712XXS,X713XXS,X718XXS,X719XXS,X72XXXS,X730XXS<br>X731XXS,X732XXS,X738XXS,X739XXS,X7401XS,X7402XS,X7409XS<br>X748XXS,X749XXS,X75XXXS,X76XXXS,X770XXS,X771XXS,X772XXS<br>X773XXS,X778XXS,X779XXS,X780XXS,X781XXS,X782XXS,X788XXS<br>X789XXS,X79XXXS,X80XXXS,X810XXS,X811XXS,X818XXS,X828XXS<br>X830XXS,X831XXS,X832XXS,X838XXS | |
| Mesothelioma | C450,C451,C452,C457,C459 | 1588,1639,1641,1958,1991 |
| Miscellaneous mental | F068,F079,F09,F488,F489,F520,F521,F5221,F5222,F5231,F5232 | 2948,3005,30089,3009,3021,3022,3023,<br>3024,30250,3026,30270 |
| and behavioral | F524,F525,F526,F528,F529,F54,F550,F551,F552,F553,F554,F558 | 30271,30272,30273,30274,30275,30276,<br>30279,30281,30282,30283 |
| disorders conditions | F59,F630,F640,F641,F642,F648,F649,F650,F651,F652,F653,F654 | 30284,30285,30289,3029,30590,30591,<br>30592,30593,30651,3068 |
| | F6550,F6551,F6552,F6581,F6589,F659,F66,F938,F939,F989,F99 | 3109,31231,3130,3133,31382,31389,3139,<br>316,64840,64841,64842 |
| | O99340,O99341,O99342,O99343,O99344,O99345,R37 | 64843,64844,V402,V409,V417 |
| Molar pregnancy and other abnormal products of conception | O010,O011,O019,O020,O021,O0281,O0289,O029 | 630,6310,6318,632 |
| Multiple gestation | O30001,O30002,O30003,O30009,O30011,O30012,O30013,O30019 | 64600,64601,64603,65100,65101,65103,<br>65110,65111,65113,65120 |
| | O30021,O30022,O30023,O30029,O30031,O30032,O30033,O30039 | 65121,65123,65130,65131,65133,65160,<br>65161,65163,65170,65171 |
| | O30041,O30042,O30043,O30049,O30091,O30092,O30093,O30099 | 65173,65180,65181,65183,65190,65191,<br>65193,67810,67811,67813 |
| | O30101,O30102,O30103,O30109,O30111,O30112,O30113,O30119 | V9100,V9101,V9102,V9103,V9109,<br>V9110,V9111,V9112,V9119,V9120 |
| | O30121,O30122,O30123,O30129,O30131,O30132,O30133,O30139 | V9121,V9122,V9129,V9190,V9191,<br>V9192,V9199 |
| | O30191,O30192,O30193,O30199,O30201,O30202,O30203,O30209<br>O30211,O30212,O30213,O30219,O30221,O30222,O30223,O30229<br>O30231,O30232,O30233,O30239,O30291,O30292,O30293,O30299<br>O30801,O30802,O30803,O30809,O30811,O30812,O30813,O30819<br>O30821,O30822,O30823,O30829,O30831,O30832,O30833,O30839<br>O30891,O30892,O30893,O30899,O3090,O3091,O3092,O3093,O3100X0<br>O3100X1,O3100X2,O3100X3,O3100X4,O3100X5,O3100X9,O3101X0<br>O3101X1,O3101X2,O3101X3,O3101X4,O3101X5,O3101X9,O3102X0<br>O3102X1,O3102X2,O3102X3,O3102X4,O3102X5,O3102X9,O3103X0<br>O3103X1,O3103X2,O3103X3,O3103X4,O3103X5,O3103X9,O3110X0<br>O3110X1,O3110X2,O3110X3,O3110X4,O3110X5,O3110X9,O3111X0<br>O3111X1,O3111X2,O3111X3,O3111X4,O3111X5,O3111X9,O3112X0<br>O3112X1,O3112X2,O3112X3,O3112X4,O3112X5,O3112X9,O3113X0<br>O3113X1,O3113X2,O3113X3,O3113X4,O3113X5,O3113X9,O3120X0<br>O3120X1,O3120X2,O3120X3,O3120X4,O3120X5,O3120X9,O3121X0<br>O3121X1,O3121X2,O3121X3,O3121X4,O3121X5,O3121X9,O3122X0<br>O3122X1,O3122X2,O3122X3,O3122X4,O3122X5,O3122X9,O3123X0<br>O3123X1,O3123X2,O3123X3,O3123X4,O3123X5,O3123X9,O3130X0 | |

Continued on next page

Table S9 (continued).

| Diagnosis | ICD-10 | ICD-9 |
|---|---|---|
| | O3130X1,O3130X2,O3130X3,O3130X4,O3130X5,O3130X9,O3131X0<br>O3131X1,O3131X2,O3131X3,O3131X4,O3131X5,O3131X9,O3132X0<br>O3132X1,O3132X2,O3132X3,O3132X4,O3132X5,O3132X9,O3133X0<br>O3133X1,O3133X2,O3133X3,O3133X4,O3133X5,O3133X9,O318X10<br>O318X11,O318X12,O318X13,O318X14,O318X15,O318X19,O318X20<br>O318X21,O318X22,O318X23,O318X24,O318X25,O318X29,O318X30<br>O318X31,O318X32,O318X33,O318X34,O318X35,O318X39,O318X90<br>O318X91,O318X92,O318X93,O318X94,O318X95,O318X99 | |
| Multiple myeloma | C9000,C9001,C9002,C9010,C9011,C9012,C9020,C9021,C9022,C9030<br>C9031,C9032 | 20300,20301,20302,20310,20311,20312,<br>20380,20381,20382 |
| Multiple sclerosis | G35 | 340 |
| Muscle disorders | B5882,B6981,K6812,M60000,M60001,M60002,M60003,M60004,M60005<br>M60009,M60011,M60012,M60019,M60021,M60022,M60029,M60031<br>M60032,M60039,M60041,M60042,M60043,M60044,M60045,M60046<br>M60051,M60052,M60059,M60061,M60062,M60069,M60070,M60071<br>M60072,M60073,M60074,M60075,M60076,M60077,M60078,M6008,M6009<br>M6010,M60111,M60112,M60119,M60121,M60122,M60129,M60131,M60132<br>M60139,M60141,M60142,M60149,M60151,M60152,M60159,M60161<br>M60162,M60169,M60171,M60172,M60179,M6018,M6019,M6080,M60811<br>M60812,M60819,M60821,M60822,M60829,M60831,M60832,M60839<br>M60841,M60842,M60849,M60851,M60852,M60859,M60861,M60862<br>M60869,M60871,M60872,M60879,M6088,M6089,M609,M6100,M61011<br>M61012,M61019,M61021,M61022,M61029,M61031,M61032,M61039<br>M61041,M61042,M61049,M61051,M61052,M61059,M61061,M61062<br>M61069,M61071,M61072,M61079,M6108,M6109,M6110,M61111,M61112<br>M61119,M61121,M61122,M61129,M61131,M61132,M61139,M61141<br>M61142,M61143,M61144,M61145,M61146,M61151,M61152,M61159<br>M61161,M61162,M61169,M61171,M61172,M61173,M61174,M61175<br>M61176,M61177,M61178,M61179,M6118,M6119,M6120,M61211,M61212<br>M61219,M61221,M61222,M61229,M61231,M61232,M61239,M61241<br>M61242,M61249,M61251,M61252,M61259,M61261,M61262,M61269<br>M61271,M61272,M61279,M6128,M6129,M6130,M61311,M61312,M61319<br>M61321,M61322,M61329,M61331,M61332,M61339,M61341,M61342<br>M61349,M61351,M61352,M61359,M61361,M61362,M61369,M61371<br>M61372,M61379,M6138,M6139,M6140,M61411,M61412,M61419,M61421<br>M61422,M61429,M61431,M61432,M61439,M61441,M61442,M61449<br>M61451,M61452,M61459,M61461,M61462,M61469,M61471,M61472<br>M61479,M6148,M6149,M6150,M61511,M61512,M61519,M61521,M61522<br>M61529,M61531,M61532,M61539,M61541,M61542,M61549,M61551<br>M61552,M61559,M61561,M61562,M61569,M61571,M61572,M61579,M6158<br>M6159,M619,M6200,M62011,M62012,M62019,M62021,M62022,M62029<br>M62031,M62032,M62039,M62041,M62042,M62049,M62051,M62052<br>M62059,M62061,M62062,M62069,M62071,M62072,M62079,M6208,M6210<br>M62111,M62112,M62119,M62121,M62122,M62129,M62131,M62132<br>M62139,M62141,M62142,M62149,M62151,M62152,M62159,M62161<br>M62162,M62169,M62171,M62172,M62179,M6218,M6220,M62211,M62212<br>M62219,M62221,M62222,M62229,M62231,M62232,M62239,M62241<br>M62242,M62249,M62251,M62252,M62259,M62261,M62262,M62269<br>M62271,M62272,M62279,M6228,M623,M6240,M62411,M62412,M62419<br>M62421,M62422,M62429,M62431,M62432,M62439,M62441,M62442<br>M62449,M62451,M62452,M62459,M62461,M62462,M62469,M62471<br>M62472,M62479,M6248,M6249,M6250,M62511,M62512,M62519,M62521<br>M62522,M62529,M62531,M62532,M62539,M62541,M62542,M62549<br>M62551,M62552,M62559,M62561,M62562,M62569,M62571,M62572<br>M62579,M6258,M6259,M625A0,M625A1,M625A2,M625A9,M6281,M6282<br>M62831,M62838,M6284,M6289,M629,M6380,M63811,M63812,M63819<br>M63821,M63822,M63829,M63831,M63832,M63839,M63841,M63842<br>M63849,M63851,M63852,M63859,M63861,M63862,M63869,M63871<br>M63872,M63879,M6388,M6389 | 04081,1231,1307,56731,7280,72810,72811,<br>72812,72819,7282,7283<br>72881,72883,72884,72885,72887,72888,<br>72889,7289,7291 |
| Musculoskeletal abscess | M6500,M65011,M65012,M65019,M65021,M65022,M65029,M65031,M65032<br>M65039,M65041,M65042,M65049,M65051,M65052,M65059,M65061<br>M65062,M65069,M65071,M65072,M65079,M6508,M7100,M71011,M71012<br>M71019,M71021,M71022,M71029,M71031,M71032,M71039,M71041<br>M71042,M71049,M71051,M71052,M71059,M71061,M71062,M71069<br>M71071,M71072,M71079,M7108,M7109 | 72789 |
| Musculoskeletal congenital conditions | Q6500,Q6501,Q6502,Q651,Q652,Q6530,Q6531,Q6532,Q654,Q655,Q656<br>Q6581,Q6582,Q6589,Q659,Q660,Q6600,Q6601,Q6602,Q661,Q6610<br>Q6611,Q6612,Q662,Q6621,Q66211,Q66212,Q66219,Q6622,Q66221<br>Q66222,Q66229,Q663,Q6630,Q6631,Q6632,Q664,Q6640,Q6641,Q6642<br>Q6650,Q6651,Q6652,Q666,Q667,Q6670,Q6671,Q6672,Q6680,Q6681<br>Q6682,Q6689,Q669,Q6690,Q6691,Q6692,Q670,Q671,Q672,Q673,Q674 | 7175,7540,7541,7542,75430,75431,75432,<br>75433,75440,75441,75442<br>75443,75444,75451,75452,75453,75459,<br>75461,75462,75469,75470<br>75471,75479,75481,75482,75489,75500,<br>75501,75502,75510,75511<br>75512,75513,75514,75520,75521,75523,<br>75525,75526,75527,75528<br>75529,75530,75531,75532,75533,75534,<br>75535,75536,75537,75538<br>75539,7554,75550,75551,75552,75553,<br>75554,75556,75557,75558 |

Continued on next page

**Table** S9 **(continued).**

| Diagnosis | ICD-10 | ICD-9 |
|---|---|---|
| | Q675,Q676,Q677,Q678,Q680,Q681,Q682,Q683,Q684,Q685,Q686,Q688 | 75559,75561,75562,75563,75564,75565, 75566,75567,75569,7558 |
| | Q690,Q691,Q692,Q699,Q7000,Q7001,Q7002,Q7003,Q7010,Q7011,Q7012 | 7559,7560,75612,75613,75614,75615, 75616,75617,75619,7562,7563 |
| | Q7013,Q7020,Q7021,Q7022,Q7023,Q7030,Q7031,Q7032,Q7033,Q704 | 7564,75650,75651,75652,75653,75654, 75655,75656,75659,7566 |
| | Q709,Q7100,Q7101,Q7102,Q7103,Q7110,Q7111,Q7112,Q7113,Q7120 | 75670,75671,75672,75673,75679,75681, 75682,75683,75689,7569 |
| | Q7121,Q7122,Q7123,Q7130,Q7131,Q7132,Q7133,Q7140,Q7141,Q7142 | |
| | Q7143,Q7150,Q7151,Q7152,Q7153,Q7160,Q7161,Q7162,Q7163,Q71811 | |
| | Q71812,Q71813,Q71819,Q71891,Q71892,Q71893,Q71899,Q7190,Q7191 | |
| | Q7192,Q7193,Q7200,Q7201,Q7202,Q7203,Q7210,Q7211,Q7212,Q7213 | |
| | Q7220,Q7221,Q7222,Q7223,Q7230,Q7231,Q7232,Q7233,Q7240,Q7241 | |
| | Q7242,Q7243,Q7250,Q7251,Q7252,Q7253,Q7260,Q7261,Q7262,Q7263 | |
| | Q7270,Q7271,Q7272,Q7273,Q72811,Q72812,Q72813,Q72819,Q72891 | |
| | Q72892,Q72893,Q72899,Q7290,Q7291,Q7292,Q7293,Q730,Q731,Q738 | |
| | Q740,Q741,Q742,Q743,Q748,Q749,Q750,Q751,Q752,Q753,Q754,Q755 | |
| | Q758,Q759,Q760,Q761,Q762,Q763,Q76411,Q76412,Q76413,Q76414 | |
| | Q76415,Q76419,Q76425,Q76426,Q76427,Q76428,Q76429,Q7649,Q765 | |
| | Q766,Q767,Q768,Q769,Q770,Q771,Q772,Q773,Q774,Q775,Q776,Q777 | |
| | Q778,Q779,Q780,Q781,Q782,Q783,Q784,Q785,Q786,Q788,Q789,Q790 | |
| | Q791,Q792,Q793,Q794,Q7951,Q7959,Q796,Q7960,Q7961,Q7962,Q7963 | |
| | Q7969,Q798,Q799 | |
| Musculoskeletal pain, | M2550,M25511,M25512,M25519,M25521,M25522,M25529,M25531,M25532 | 71940,71941,71942,71943,71944,71945, 71946,71947,71950,71951 |
| not low back pain | M25539,M25541,M25542,M25549,M25551,M25552,M25559,M25561 | 71952,71953,71954,71955,71956,71957, 7238,7241,7245,7291,7295 |
| | M25562,M25569,M25571,M25572,M25579,M2559,M2560,M25611,M25612 | |
| | M25619,M25621,M25622,M25629,M25631,M25632,M25639,M25641 | |
| | M25642,M25649,M25651,M25652,M25659,M25661,M25662,M25669 | |
| | M25671,M25672,M25673,M25674,M25675,M25676,M2569,M546,M5481 | |
| | M5489,M549,M791,M7910,M7911,M7912,M7918,M79601,M79602,M79603 | |
| | M79604,M79605,M79606,M79609,M79621,M79622,M79629,M79631 | |
| | M79632,M79639,M79641,M79642,M79643,M79644,M79645,M79646 | |
| | M79651,M79652,M79659,M79661,M79662,M79669,M79671,M79672 | |
| | M79673,M79674,M79675,M79676 | |
| Myelodysplastic syndrome (MDS) | D460,D461,D4620,D4621,D4622,D464,D469,D46A,D46B,D46C,D46Z | 23872,23873,23874,23875 |
| Myocarditis and | A3681,A381,A3950,A3952,B2682,B3320,B3322,B3324,B5881,D8685 | 03282,0341,03640,03643,07279,07420, 07423,07889,1303,135,3912 |
| cardiomyopathy | I012,I090,I255,I400,I401,I408,I409,I41,I420,I421,I422,I423 | 3980,4148,4220,42290,42291,42292,42293, 42299,4250,42511,42518 |
| | I424,I425,I426,I427,I428,I429,I43,I514,J1082,J1182,O903 | 4252,4253,4254,4255,4258,4259,4290, 4878,67452,67454 |
| Myopathies | G7000,G7001,G701,G702,G7080,G7081,G7089,G709,G710,G7100,G7101 | 35800,35801,3581,3582,35830,35831, 35839,3588,3589,3590,3591 |
| | G7102,G71031,G71032,G71033,G710340,G710341,G710342,G710349 | 35921,35922,35923,35924,35929,3593, 3594,3595,3596,35971,35979 |
| | G71035,G71038,G71039,G7109,G7111,G7112,G7113,G7114,G7119,G712 | 35981,35989,3599,7102,7140 |
| | G7120,G7121,G71220,G71228,G7129,G713,G718,G719,G720,G721,G722 | |
| | G723,G7241,G7249,G7281,G7289,G729,G731,G733,G737,M0540,M05411 | |
| | M05412,M05419,M05421,M05422,M05429,M05431,M05432,M05439 | |
| | M05441,M05442,M05449,M05451,M05452,M05459,M05461,M05462 | |
| | M05469,M05471,M05472,M05479,M0549,M3503 | |
| Nausea and vomiting | G43A0,G43A1,K910,O212,O218,O219,R110,R1110,R1111,R1112,R1113 | 07882,34620,34621,5362,5643,56987, 64323,64383,64393,78701 |
| | R1114,R1115,R112 | 78702,78703,78704 |
| Neonatal abstinence syndrome | P0414,P0415,P0416,P0417,P041A,P042,P0440,P0441,P0442,P0449 | 76072,76073,76075,76079,7795 |
| | P0481,P961,P962 | |
| Neonatal acidemia and hypoxia | P190,P191,P192,P199,P84 | 7682,7683,7684,7689,77088,77581 |
| Neonatal cerebral | P90,P910,P911,P912,P913,P914,P915,P9160,P9161,P9162,P9163 | 76870,76871,76872,76873,7790,7791,7792, 7797 |
| disorders | P918,P91811,P91819,P91821,P91822,P91823,P91829,P9188,P919 | |
| Neonatal digestive | P760,P761,P762,P768,P769,P771,P772,P773,P779,P780,P781,P782 | 7771,7772,7773,7774,77750,77751,77752, 77753,7776,7778,7779 |
| and feeding disorders | P783,P7881,P7882,P7883,P7884,P7889,P789,P9201,P9209,P921,P922 | 77931,77932,77933,77934 |
| | P923,P924,P925,P926,P928,P929 | |
| Neoplasm-related | Z08,Z120,Z1210,Z1211,Z1212,Z1213,Z122,Z1231,Z1239,Z124,Z125 | V5842,V6701,V671,V672,V6759,V676, V679,V760,V7610,V7612,V7619 |
| encounters | Z126,Z1271,Z1272,Z1273,Z1279,Z1281,Z1282,Z1283,Z1289,Z129 | V762,V763,V7641,V7642,V7643,V7644, V7645,V7646,V7647,V7649 |
| | Z483 | V7650,V7651,V7652,V7681,V769 |
| Neoplasms of | D3701,D3702,D37030,D37031,D37032,D37039,D3704,D3705,D3709 | 20710,20711,20712,2350,2351,2352,2353, 2354,2355,2356,2357 |

Continued on next page

Table S9 (continued).

| Diagnosis | ICD-10 | ICD-9 |
|---|---|---|
| unspecified nature or uncertain behavior | D371,D372,D373,D374,D375,D376,D378,D379,D380,D381,D382,D383<br>D384,D385,D386,D390,D3910,D3911,D3912,D392,D398,D399,D400<br>D4010,D4011,D4012,D408,D409,D4100,D4101,D4102,D4110,D4111<br>D4112,D4120,D4121,D4122,D413,D414,D418,D419,D420,D421,D429<br>D430,D431,D432,D433,D434,D438,D439,D440,D4410,D4411,D4412<br>D442,D443,D444,D445,D446,D447,D449,D45,D470,D4701,D4702,D4709<br>D471,D472,D473,D474,D479,D47Z1,D47Z2,D47Z9,D480,D481,D482<br>D483,D484,D485,D4860,D4861,D4862,D487,D489,D490,D491,D492<br>D493,D494,D495,D49511,D49512,D49519,D4959,D496,D497,D4981<br>D4989,D499 | 2358,2359,2360,2361,2362,2363,2364,<br>2365,2366,2367,23690,23691<br>23699,2370,2371,2372,2373,2374,2375,<br>2376,2379,2380,2381,2382<br>2383,2384,2385,23871,23876,23877,23879,<br>2388,2389,2390,2391<br>2392,2393,2394,2395,2396,2397,23981,<br>23989,2399,2731,28989 |
| Nephritis; nephrosis; renal sclerosis | A3684,A5144,B2683,B520,B5883,E0821,E0921,E1021,E1121,E1321<br>M3504,M350A,N000,N001,N002,N003,N004,N005,N006,N007,N008,N009<br>N00A,N010,N011,N012,N013,N014,N015,N016,N017,N018,N019,N01A<br>N020,N021,N022,N023,N024,N025,N026,N027,N028,N029,N02A,N030<br>N031,N032,N033,N034,N035,N036,N037,N038,N039,N03A,N040,N041<br>N042,N043,N044,N045,N046,N047,N048,N049,N04A,N050,N051,N052<br>N053,N054,N055,N056,N057,N058,N059,N05A,N060,N061,N062,N063<br>N064,N065,N066,N067,N068,N069,N06A,N070,N071,N072,N073,N074<br>N075,N076,N077,N078,N079,N07A,N08,N140,N141,N1411,N1419,N142<br>N143,N144,N150,N158,N159,N16,N269 | 03289,07279,0849,09189,1307,24940,<br>25040,25041,5800,5804,58089<br>5809,5810,5811,5812,5813,58181,58189,<br>5819,5820,5821,5822,5824<br>58289,5829,5830,5831,5832,5834,58381,<br>58389,5839,587,59081<br>5909,7102 |
| Nerve and nerve root disorders | A1783,B0221,B0222,E0841,E0941,E1041,E1141,E1341,G500,G501<br>G508,G509,G510,G511,G512,G513,G5131,G5132,G5133,G5139,G514<br>G518,G519,G520,G521,G522,G523,G527,G528,G529,G53,G540,G541<br>G542,G543,G544,G545,G546,G547,G548,G549,G55,G5600,G5601,G5602<br>G5603,G5610,G5611,G5612,G5613,G5620,G5621,G5622,G5623,G5630<br>G5631,G5632,G5633,G5640,G5641,G5642,G5643,G5680,G5681,G5682<br>G5683,G5690,G5691,G5692,G5693,G5700,G5701,G5702,G5703,G5710<br>G5711,G5712,G5713,G5720,G5721,G5722,G5723,G5730,G5731,G5732<br>G5733,G5740,G5741,G5742,G5743,G5750,G5751,G5752,G5753,G5760<br>G5761,G5762,G5763,G5770,G5771,G5772,G5773,G5780,G5781,G5782<br>G5783,G5790,G5791,G5792,G5793,G580,G587,G588,G589,G59 | 01362,01790,05311,05312,24960,25060,<br>25061,3501,3502,3508,3509<br>3510,3511,3518,3519,3520,3521,3522,<br>3523,3524,3525,3526,3529<br>3530,3531,3532,3533,3534,3535,3536,<br>3538,3539,3540,3541,3542<br>3543,3544,3545,3548,3549,3550,3551,<br>3552,3553,3554,3555,3556<br>35571,35579,3558,3559 |
| Nervous system cancers - all other types | C470,C4710,C4711,C4712,C4720,C4721,C4722,C473,C474,C475,C476<br>C478,C479,C720,C721,C7220,C7221,C7222,C7230,C7231,C7232,C7240<br>C7241,C7242,C7250,C7259,C729 | 1710,1712,1713,1714,1715,1716,1717,<br>1718,1719,1920,1922,1928<br>1929 |
| Nervous system cancers - brain | C700,C701,C709,C710,C711,C712,C713,C714,C715,C716,C717,C718<br>C719 | 1910,1911,1912,1913,1914,1915,1916,<br>1917,1918,1919,1921,1923 |
| Nervous system congenital anomalies | Q000,Q001,Q002,Q010,Q011,Q012,Q018,Q019,Q02,Q030,Q031,Q038<br>Q039,Q040,Q041,Q042,Q043,Q044,Q045,Q046,Q048,Q049,Q050,Q051<br>Q052,Q053,Q054,Q055,Q056,Q057,Q058,Q059,Q060,Q061,Q062,Q063<br>Q064,Q068,Q069,Q0700,Q0701,Q0702,Q0703,Q078,Q079,Q8500,Q8501<br>Q8502,Q8503,Q8509 | 23770,23771,23772,23773,23779,7400,<br>7401,7402,74100,74101<br>74102,74103,74190,74191,74192,74193,<br>7420,7421,7422,7423,7424<br>74251,74253,74259,7428,7429 |
| Nervous system pain and pain syndromes | G890,G8911,G8912,G8918,G8921,G8922,G8928,G8929,G893,G894,G902<br>G9050,G90511,G90512,G90513,G90519,G90521,G90522,G90523,G90529<br>G9059 | 33720,33721,33722,33729,3379,3380,<br>33811,33812,33818,33821<br>33822,33828,33829,3383,3384 |
| Nervous system signs and symptoms | R250,R251,R253,R258,R259,R260,R261,R262,R2681,R2689,R269,R270<br>R278,R279,R290,R291,R292,R295,R29810,R29818,R2990,R402110<br>R402111,R402112,R402113,R402114,R402120,R402121,R402122<br>R402123,R402124,R402130,R402131,R402132,R402133,R402134<br>R402140,R402141,R402142,R402143,R402144,R402210,R402211<br>R402212,R402213,R402214,R402220,R402221,R402222,R402223<br>R402224,R402230,R402231,R402232,R402233,R402234,R402240<br>R402241,R402242,R402243,R402244,R402250,R402251,R402252<br>R402253,R402254,R402310,R402311,R402312,R402313,R402314<br>R402320,R402321,R402322,R402323,R402324,R402330,R402331<br>R402332,R402333,R402334,R402340,R402341,R402342,R402343<br>R402344,R402350,R402351,R402352,R402353,R402354,R402360<br>R402361,R402362,R402363,R402364,R40241,R402410,R402411<br>R402412,R402413,R402414,R40242,R402420,R402421,R402422<br>R402423,R402424,R40243,R402430,R402431,R402432,R402433<br>R402434,R40244,R402440,R402441,R402442,R402443,R402444,R404 | 31501,36816,7197,78001,78002,78072,<br>78093,78097,78099,7810<br>7811,7812,7813,7814,7816,7817,7818,<br>78194,78199,7840,7843<br>78451,78452,78459,78460,78461,78469,<br>7961,797,79951,79952<br>79953,79954,79955,79959,NoDx,V414,<br>V415,V6289 |

Continued on next page

Table S9 (continued).

| Diagnosis | ICD-10 | ICD-9 |
|---|---|---|
| | R410,R411,R412,R413,R414,R4181,R4182,R4183,R41840,R41841 | |
| | R41842,R41843,R41844,R4189,R419,R430,R431,R432,R438,R439 | |
| | R4701,R4702,R471,R4781,R4782,R4789,R479,R480,R481,R482,R483 | |
| | R488,R489,R51,R510,R519,R532 | |
| Neuro-ophthalmology | A3982,A5044,H4600,H4601,H4602,H4603,H4610,H4611,H4612,H4613 | 03681,0905,27787,37700,37701,37702, 37703,37704,37710,37711 |
| | H462,H463,H468,H469,H47011,H47012,H47013,H47019,H47021,H47022 | 37712,37713,37714,37715,37716,37721, 37722,37723,37724,37730 |
| | H47023,H47029,H47031,H47032,H47033,H47039,H47091,H47092 | 37731,37732,37733,37734,37739,37741, 37742,37743,37749,37751 |
| | H47093,H47099,H4710,H4711,H4712,H4713,H47141,H47142,H47143 | 37752,37753,37754,37761,37762,37763, 37771,37772,37773,37775 |
| | H47149,H4720,H47211,H47212,H47213,H47219,H4722,H47231,H47232 | 3779,37850,37851,37852,37853,37854, 37855,37856,37885,37886 |
| | H47233,H47239,H47291,H47292,H47293,H47299,H47311,H47312 | 37887,3789 |
| | H47313,H47319,H47321,H47322,H47323,H47329,H47331,H47332 | |
| | H47333,H47339,H47391,H47392,H47393,H47399,H4741,H4742,H4743 | |
| | H4749,H47511,H47512,H47519,H47521,H47522,H47529,H47531,H47532 | |
| | H47539,H47611,H47612,H47619,H47621,H47622,H47629,H47631 | |
| | H47632,H47639,H47641,H47642,H47649,H479,H4900,H4901,H4902 | |
| | H4903,H4910,H4911,H4912,H4913,H4920,H4921,H4922,H4923,H4930 | |
| | H4931,H4932,H4933,H4940,H4941,H4942,H4943,H49811,H49812 | |
| | H49813,H49819,H49881,H49882,H49883,H49889,H499,H5120,H5121 | |
| | H5122,H5123,H518,H519 | |
| Neurocognitive | F0150,F0151,F01511,F01518,F0152,F0153,F0154,F01A0,F01A11 | 2900,29010,29011,29013,29021,29040, 29041,2908,2909,29282,2930 |
| disorders | F01A18,F01A2,F01A3,F01A4,F01B0,F01B11,F01B18,F01B2,F01B3 | 2931,2940,29410,29411,29420,29421, 30460,3102,31081,31089,3308 |
| | F01B4,F01C0,F01C11,F01C18,F01C2,F01C3,F01C4,F0280,F0281 | 3310,33111,33119,3312,33182,33183,3319 |
| | F02811,F02818,F0282,F0283,F0284,F02A0,F02A11,F02A18,F02A2 | |
| | F02A3,F02A4,F02B0,F02B11,F02B18,F02B2,F02B3,F02B4,F02C0 | |
| | F02C11,F02C18,F02C2,F02C3,F02C4,F0390,F0391,F03911,F03918 | |
| | F0392,F0393,F0394,F03A0,F03A11,F03A18,F03A2,F03A3,F03A4,F03B0 | |
| | F03B11,F03B18,F03B2,F03B3,F03B4,F03C0,F03C11,F03C18,F03C2 | |
| | F03C3,F03C4,F04,F05,F0670,F0671,F0781,F0789,F1917,F1927,F1997 | |
| | F482,G300,G301,G308,G309,G3101,G3109,G311,G3181,G3183,G3184 | |
| | G319 | |
| Neurodevelopmental | F70,F71,F72,F73,F78,F78A1,F78A9,F79,F800,F801,F802,F804,F8081 | 29900,29901,29910,29980,29981,29990, 3070,30720,30721,30722 |
| disorders | F8082,F8089,F809,F810,F812,F8181,F8189,F819,F82,F840,F842 | 30723,3073,30929,31389,3139,31400, 31401,3142,3149,31500,31502 |
| | F843,F845,F848,F849,F88,F89,F900,F901,F902,F908,F909,F948 | 31509,3151,3152,31531,31532,31534, 31535,31539,3154,3158,3159 |
| | F949,F950,F951,F952,F958,F959,F984,F985,F988 | 317,3180,3181,3182,319,3308,V400 |
| Neurogenic neuropathic arthropathy | G980,M1460,M14611,M14612,M14619,M14621,M14622,M14629,M14631 | 34989,7135 |
| | M14632,M14639,M14641,M14642,M14649,M14651,M14652,M14659 | |
| | M14661,M14662,M14669,M14671,M14672,M14679,M1468,M1469 | |
| Newborn affected by | P000,P001,P002,P003,P004,P005,P006,P007,P0081,P0082,P0089 | 7600,7601,7602,7603,7604,7605,76062, 76063,76070,76071,76072 |
| maternal conditions | P009,P010,P011,P012,P013,P014,P015,P016,P017,P018,P019,P020 | 76073,76074,76075,76078,76079,7608, 7609,7610,7611,7612,7613 |
| or complications of | P021,P0220,P0229,P023,P024,P025,P0260,P0269,P027,P0270,P0278 | 7614,7615,7616,7617,7618,7619,7620, 7621,7622,7623,7624,7625 |
| labor delivery | P028,P029,P030,P031,P032,P033,P034,P035,P036,P03810,P03811 | 7626,7627,7628,7629,7630,7631,7632, 7633,7634,7635,7636,7637 |
| | P03819,P0382,P0389,P039,P040,P041,P0411,P0412,P0413,P0414 | 76381,76382,76383,76384,76389,7639, 7795 |
| | P0415,P0416,P0417,P0418,P0419,P041A,P042,P043,P0440,P0441 | |
| | P0442,P0449,P045,P046,P048,P0481,P0489,P049,P961 | |
| Non-Hodgkin lymphoma | C8200,C8201,C8202,C8203,C8204,C8205,C8206,C8207,C8208,C8209 | 20000,20001,20002,20003,20004,20005, 20006,20007,20008,20010 |
| | C8210,C8211,C8212,C8213,C8214,C8215,C8216,C8217,C8218,C8219 | 20011,20012,20013,20014,20015,20016, 20017,20018,20020,20021 |
| | C8220,C8221,C8222,C8223,C8224,C8225,C8226,C8227,C8228,C8229 | 20022,20023,20024,20025,20026,20027, 20028,20030,20040,20041 |
| | C8230,C8231,C8232,C8233,C8234,C8235,C8236,C8237,C8238,C8239 | 20042,20043,20044,20045,20046,20047, 20048,20050,20051,20052 |
| | C8240,C8241,C8242,C8243,C8244,C8245,C8246,C8247,C8248,C8249 | 20053,20054,20055,20056,20057,20058, 20060,20061,20062,20063 |
| | C8250,C8251,C8252,C8253,C8254,C8255,C8256,C8257,C8258,C8259 | 20064,20065,20066,20067,20068,20070, 20071,20072,20073,20074 |
| | C8260,C8261,C8262,C8263,C8264,C8265,C8266,C8267,C8268,C8269 | 20075,20076,20077,20078,20080,20081, 20082,20083,20084,20085 |
| | C8280,C8281,C8282,C8283,C8284,C8285,C8286,C8287,C8288,C8289 | 20086,20087,20088,20200,20201,20202, 20203,20204,20205,20206 |
| | C8290,C8291,C8292,C8293,C8294,C8295,C8296,C8297,C8298,C8299 | 20207,20208,20210,20211,20212,20213, 20214,20215,20216,20217 |
| | C8300,C8301,C8302,C8303,C8304,C8305,C8306,C8307,C8308,C8309 | 20218,20220,20221,20222,20223,20224, 20225,20226,20227,20228 |
| | C8310,C8311,C8312,C8313,C8314,C8315,C8316,C8317,C8318,C8319 | 20250,20260,20270,20271,20272,20273, 20274,20275,20276,20277 |

Continued on next page

Table S9 (continued).

| Diagnosis | ICD-10 | ICD-9 |
|---|---|---|
| | C8330,C8331,C8332,C8333,C8334,C8335,C8336,C8337,C8338,C8339 | 20278,20280,20281,20282,20283,20284, 20285,20286,20287,20288 |
| | C8350,C8351,C8352,C8353,C8354,C8355,C8356,C8357,C8358,C8359 | 20290,20291,20292,20293,20294,20295, 20296,20297,20298,20380 |
| | C8370,C8371,C8372,C8373,C8374,C8375,C8376,C8377,C8378,C8379 C8380,C8381,C8382,C8383,C8384,C8385,C8386,C8387,C8388,C8389 C8390,C8391,C8392,C8393,C8394,C8395,C8396,C8397,C8398,C8399 C8400,C8401,C8402,C8403,C8404,C8405,C8406,C8407,C8408,C8409 C8410,C8411,C8412,C8413,C8414,C8415,C8416,C8417,C8418,C8419 C8440,C8441,C8442,C8443,C8444,C8445,C8446,C8447,C8448,C8449 C8460,C8461,C8462,C8463,C8464,C8465,C8466,C8467,C8468,C8469 C8470,C8471,C8472,C8473,C8474,C8475,C8476,C8477,C8478,C8479 C847A,C8490,C8491,C8492,C8493,C8494,C8495,C8496,C8497,C8498 C8499,C84A0,C84A1,C84A2,C84A3,C84A4,C84A5,C84A6,C84A7,C84A8 C84A9,C84Z0,C84Z1,C84Z2,C84Z3,C84Z4,C84Z5,C84Z6,C84Z7,C84Z8 C84Z9,C8510,C8511,C8512,C8513,C8514,C8515,C8516,C8517,C8518 C8519,C8520,C8521,C8522,C8523,C8524,C8525,C8526,C8527,C8528 C8529,C8580,C8581,C8582,C8583,C8584,C8585,C8586,C8587,C8588 C8589,C8590,C8591,C8592,C8593,C8594,C8595,C8596,C8597,C8598 C8599,C860,C861,C862,C863,C864,C865,C866,C880,C882,C883,C884 C888,C889,C960,C962,C9620,C9621,C9629,C965,C966,C969,C96Z | 20381,23879,2733,27789 |
| Non-pressure ulcer of | L97101,L97102,L97103,L97104,L97105,L97106,L97108,L97109 | 70710,70711,70712,70713,70714,70715, 70719,7078,7079 |
| skin | L97111,L97112,L97113,L97114,L97115,L97116,L97118,L97119 L97121,L97122,L97123,L97124,L97125,L97126,L97128,L97129 L97201,L97202,L97203,L97204,L97205,L97206,L97208,L97209 L97211,L97212,L97213,L97214,L97215,L97216,L97218,L97219 L97221,L97222,L97223,L97224,L97225,L97226,L97228,L97229 L97301,L97302,L97303,L97304,L97305,L97306,L97308,L97309 L97311,L97312,L97313,L97314,L97315,L97316,L97318,L97319 L97321,L97322,L97323,L97324,L97325,L97326,L97328,L97329 L97401,L97402,L97403,L97404,L97405,L97406,L97408,L97409 L97411,L97412,L97413,L97414,L97415,L97416,L97418,L97419 L97421,L97422,L97423,L97424,L97425,L97426,L97428,L97429 L97501,L97502,L97503,L97504,L97505,L97506,L97508,L97509 L97511,L97512,L97513,L97514,L97515,L97516,L97518,L97519 L97521,L97522,L97523,L97524,L97525,L97526,L97528,L97529 L97801,L97802,L97803,L97804,L97805,L97806,L97808,L97809 L97811,L97812,L97813,L97814,L97815,L97816,L97818,L97819 L97821,L97822,L97823,L97824,L97825,L97826,L97828,L97829 L97901,L97902,L97903,L97904,L97905,L97906,L97908,L97909 L97911,L97912,L97913,L97914,L97915,L97916,L97918,L97919 L97921,L97922,L97923,L97924,L97925,L97926,L97928,L97929 L98411,L98412,L98413,L98414,L98415,L98416,L98418,L98419 L98421,L98422,L98423,L98424,L98425,L98426,L98428,L98429 L98491,L98492,L98493,L98494,L98495,L98496,L98498,L98499 | |
| Noninfectious | K520,K521,K522,K5221,K5222,K5229,K523,K5281,K5282,K52831 | 53570,53571,5581,5582,5583,55841,55842, 5589,78791 |
| gastroenteritis | K52832,K52838,K52839,K5289,K529 | |
| Noninfectious hepatitis | K712,K713,K714,K7150,K7151,K716,K730,K731,K732,K738,K739,K752 K753,K754,K7581 | 57140,57141,57142,57149,5718,5733 |
| Nonmalignant breast | N6001,N6002,N6009,N6011,N6012,N6019,N6021,N6022,N6029,N6031 | 6100,6101,6102,6103,6104,6108,6109, 6110,6111,6112,6113,6114 |
| conditions | N6032,N6039,N6041,N6042,N6049,N6081,N6082,N6089,N6091,N6092 | 6115,6116,61171,61172,61179,61181, 61182,61189,6119,6120,6121 |
| | N6099,N61,N610,N611,N6120,N6121,N6122,N6123,N62,N63,N630 | 67500,67501,67502,67503,67504,67510, 67511,67512,67513,67514 |
| | N6310,N6311,N6312,N6313,N6314,N6315,N6320,N6321,N6322,N6323 | 67520,67521,67522,67523,67524,67582, 67584,67600,67601,67602 |
| | N6324,N6325,N6331,N6332,N6341,N6342,N640,N641,N642,N643,N644 N6451,N6452,N6453,N6459,N6481,N6482,N6489,N649,N650,N651 O91011,O91012,O91013,O91019,O9102,O9103,O91111,O91112,O91113 O91119,O9112,O9113,O91211,O91212,O91213,O91219,O9122,O9123 O92011,O92012,O92013,O92019,O9202,O9203 | 67603,67604 |
| Nonrheumatic and unspecified valve disorders | I340,I341,I342,I348,I3481,I3489,I349,I350,I351,I352,I358,I359 I360,I361,I362,I368,I369,I370,I371,I372,I378,I379,Z952,Z953 Z954 | 4240,4241,4242,4243,V422,V433 |
| Nonspecific chest pain | R072,R0789,R079 | 78650,78651,78659 |
| Nutritional anemia | D500,D501,D508,D509,D510,D511,D512,D513,D518,D519,D520,D521 | 2800,2801,2808,2809,2810,2811,2812, 2813,2814,2818,2819 |
| | D528,D529,D530,D531,D532,D538,D539 | |
| Nutritional | E500,E501,E502,E503,E504,E505,E506,E507,E508,E509,E5111,E5112 | 2640,2641,2642,2643,2644,2645,2646, 2647,2648,2649,2650,2651 |
| deficiencies | E512,E518,E519,E52,E530,E531,E538,E539,E54,E550,E559,E560 | 2652,2660,2661,2662,2669,267,2680,2689, 2690,2691,2692,2693 |
| | E561,E568,E569,E58,E59,E60,E610,E611,E612,E613,E614,E615,E616 E617,E618,E619,E630,E631,E638,E639 | 2698,2699 |

Continued on next page

**Table S9** (continued).

| Diagnosis | ICD-10 | ICD-9 |
|---|---|---|
| OB-related trauma to | O700,O701,O702,O7020,O7021,O7022,O7023,O703,O704,O709,O7182 | 66401,66404,66411,66414,66421,66424,<br>66431,66434,66441,66444 |
| perineum and vulva | | 66461,66464,66481,66484 |
| Obesity | E6601,E6609,E661,E662,E668,E669,O99210,O99211,O99212,O99213 | 27800,27801,27803,64910,64911,64912,<br>64913,64914,V8530,V8531 |
| | O99214,O99215,Z6830,Z6831,Z6832,Z6833,Z6834,Z6835,Z6836,Z6837 | V8532,V8533,V8534,V8535,V8536,<br>V8537,V8538,V8539,V8541,V8542 |
| | Z6838,Z6839,Z6841,Z6842,Z6843,Z6844,Z6845,Z6854 | V8543,V8544,V8545,V8554 |
| Obsessive-compulsive and related disorders | F42,F422,F423,F424,F428,F429,F4522,F633 | 3003,3007,3079,31239 |
| Obstetric history | O09211,O09212,O09213,O09219,O09291,O09292,O09293,O09299,O2620 | 64630,64631,64633,64680,64681,64683,<br>V2341,V2349,V235 |
| affecting care in pregnancy | O2621,O2622,O2623,O2630,O2631,O2632,O2633 | |
| Occlusion or stenosis | I6501,I6502,I6503,I6509,I651,I6521,I6522,I6523,I6529,I658 | 43300,43310,43320,43380,43390,43400,<br>43410,43490 |
| of precerebral or cerebral arteries without infarction | I659,I6601,I6602,I6603,I6609,I6611,I6612,I6613,I6619,I6621<br>I6622,I6623,I6629,I663,I668,I669 | |
| Oculofacial plastics | H00031,H00032,H00033,H00034,H00035,H00036,H00039,H01111 | 36081,36089,3609,37313,37331,37332,<br>37333,37334,3738,3739 |
| and orbital | H01112,H01113,H01114,H01115,H01116,H01119,H01121,H01122 | 37400,37401,37402,37403,37404,37405,<br>37410,37411,37412,37413 |
| conditions | H01123,H01124,H01125,H01126,H01129,H01131,H01132,H01133 | 37414,37420,37421,37422,37423,37430,<br>37431,37432,37433,37434 |
| | H01134,H01135,H01136,H01139,H01141,H01142,H01143,H01144 | 37441,37443,37444,37445,37446,37450,<br>37451,37452,37453,37454 |
| | H01145,H01146,H01149,H018,H019,H02001,H02002,H02003,H02004 | 37455,37456,37481,37482,37483,37484,<br>37485,37486,37487,37489 |
| | H02005,H02006,H02009,H02011,H02012,H02013,H02014,H02015 | 3749,37500,37501,37502,37503,37511,<br>37512,37513,37514,37515 |
| | H02016,H02019,H02021,H02022,H02023,H02024,H02025,H02026 | 37516,37520,37521,37522,37530,37531,<br>37532,37533,37541,37542 |
| | H02029,H02031,H02032,H02033,H02034,H02035,H02036,H02039 | 37543,37551,37552,37553,37554,37555,<br>37556,37557,37561,37569 |
| | H02041,H02042,H02043,H02044,H02045,H02046,H02049,H02051 | 37581,37589,3759,37600,37601,37602,<br>37603,37604,37610,37611 |
| | H02052,H02053,H02054,H02055,H02056,H02059,H02101,H02102 | 37612,37630,37631,37632,37633,37634,<br>37635,37636,37640,37642 |
| | H02103,H02104,H02105,H02106,H02109,H02111,H02112,H02113 | 37643,37645,37646,37647,37650,37651,<br>37652,3766,37681,37682 |
| | H02114,H02115,H02116,H02119,H02121,H02122,H02123,H02124 | 37689,3769 |
| | H02125,H02126,H02129,H02131,H02132,H02133,H02134,H02135 | |
| | H02136,H02139,H02141,H02142,H02143,H02144,H02145,H02146 | |
| | H02149,H02151,H02152,H02153,H02154,H02155,H02156,H02159 | |
| | H02201,H02202,H02203,H02204,H02205,H02206,H02209,H0220A | |
| | H0220B,H0220C,H02211,H02212,H02213,H02214,H02215,H02216 | |
| | H02219,H0221A,H0221B,H0221C,H02221,H02222,H02223,H02224 | |
| | H02225,H02226,H02229,H0222A,H0222B,H0222C,H02231,H02232 | |
| | H02233,H02234,H02235,H02236,H02239,H0223A,H0223B,H0223C,H0230 | |
| | H0231,H0232,H0233,H0234,H0235,H0236,H02401,H02402,H02403 | |
| | H02409,H02411,H02412,H02413,H02419,H02421,H02422,H02423 | |
| | H02429,H02431,H02432,H02433,H02439,H02511,H02512,H02513 | |
| | H02514,H02515,H02516,H02519,H02521,H02522,H02523,H02524 | |
| | H02525,H02526,H02529,H02531,H02532,H02533,H02534,H02535 | |
| | H02536,H02539,H0259,H0260,H0261,H0262,H0263,H0264,H0265,H0266 | |
| | H0270,H02711,H02712,H02713,H02714,H02715,H02716,H02719,H02721 | |
| | H02722,H02723,H02724,H02725,H02726,H02729,H02731,H02732 | |
| | H02733,H02734,H02735,H02736,H02739,H0279,H02811,H02812,H02813 | |
| | H02814,H02815,H02816,H02819,H02821,H02822,H02823,H02824 | |
| | H02825,H02826,H02829,H02831,H02832,H02833,H02834,H02835 | |
| | H02836,H02839,H02841,H02842,H02843,H02844,H02845,H02846 | |
| | H02849,H02851,H02852,H02853,H02854,H02855,H02856,H02859 | |
| | H02861,H02862,H02863,H02864,H02865,H02866,H02869,H02871 | |
| | H02872,H02873,H02874,H02875,H02876,H02879,H0289,H029,H04001 | |
| | H04002,H04003,H04009,H04011,H04012,H04013,H04019,H04021 | |
| | H04022,H04023,H04029,H04031,H04032,H04033,H04039,H04111 | |
| | H04112,H04113,H04119,H04121,H04122,H04123,H04129,H04131 | |
| | H04132,H04133,H04139,H04141,H04142,H04143,H04149,H04151 | |
| | H04152,H04153,H04159,H04161,H04162,H04163,H04169,H0419,H04201 | |
| | H04202,H04203,H04209,H04211,H04212,H04213,H04219,H04221 | |
| | H04222,H04223,H04229,H04301,H04302,H04303,H04309,H04311 | |
| | H04312,H04313,H04319,H04321,H04322,H04323,H04329,H04331 | |
| | H04332,H04333,H04339,H04411,H04412,H04413,H04419,H04421 | |
| | H04422,H04423,H04429,H04431,H04432,H04433,H04439,H04511 | |
| | H04512,H04513,H04519,H04521,H04522,H04523,H04529,H04531 | |
| | H04532,H04533,H04539,H04541,H04542,H04543,H04549,H04551 | |
| | H04552,H04553,H04559,H04561,H04562,H04563,H04569,H04571 | |
| | H04572,H04573,H04579,H04611,H04612,H04613,H04619,H0469,H04811 | |
| | H04812,H04813,H04819,H0489,H049,H0500,H05011,H05012,H05013 | |

Continued on next page

Table S9 (continued).

| Diagnosis | ICD-10 | ICD-9 |
|---|---|---|
| | H05019,H05021,H05022,H05023,H05029,H05031,H05032,H05033<br>H05039,H05041,H05042,H05043,H05049,H0510,H05111,H05112,H05113<br>H05119,H05121,H05122,H05123,H05129,H0520,H05211,H05212,H05213<br>H05219,H05221,H05222,H05223,H05229,H05231,H05232,H05233<br>H05239,H05241,H05242,H05243,H05249,H05251,H05252,H05253<br>H05259,H05261,H05262,H05263,H05269,H0530,H05311,H05312,H05313<br>H05319,H05321,H05322,H05323,H05329,H05331,H05332,H05333<br>H05339,H05341,H05342,H05343,H05349,H05351,H05352,H05353<br>H05359,H05401,H05402,H05403,H05409,H05411,H05412,H05413<br>H05419,H05421,H05422,H05423,H05429,H0550,H0551,H0552,H0553<br>H05811,H05812,H05813,H05819,H05821,H05822,H05823,H05829,H0589<br>H059,H44821,H44822,H44823,H44829,H4489,H449,H57811,H57812<br>H57813,H57819 | |
| Open wounds of head and neck, initial encounter | S0100XA,S0101XA,S0102XA,S0103XA,S0104XA,S0105XA,S01101A<br>S01102A,S01109A,S01111A,S01112A,S01119A,S01121A,S01122A<br>S01129A,S01131A,S01132A,S01139A,S01141A,S01142A,S01149A<br>S01151A,S01152A,S01159A,S0120XA,S0121XA,S0122XA,S0123XA<br>S0124XA,S0125XA,S01301A,S01302A,S01309A,S01311A,S01312A<br>S01319A,S01321A,S01322A,S01329A,S01331A,S01332A,S01339A<br>S01341A,S01342A,S01349A,S01351A,S01352A,S01359A,S01401A<br>S01402A,S01409A,S01411A,S01412A,S01419A,S01421A,S01422A<br>S01429A,S01431A,S01432A,S01439A,S01441A,S01442A,S01449A<br>S01451A,S01452A,S01459A,S01501A,S01502A,S01511A,S01512A<br>S01521A,S01522A,S01531A,S01532A,S01541A,S01542A,S01551A<br>S01552A,S0180XA,S0181XA,S0182XA,S0183XA,S0184XA,S0185XA<br>S0190XA,S0191XA,S0192XA,S0193XA,S0194XA,S0195XA,S0520XA<br>S0521XA,S0522XA,S0530XA,S0531XA,S0532XA,S0540XA,S0541XA<br>S0542XA,S0550XA,S0551XA,S0552XA,S0560XA,S0561XA,S0562XA<br>S0570XA,S0571XA,S0572XA,S080XXA,S0912XA,S0920XA,S0921XA<br>S0922XA,S11011A,S11012A,S11013A,S11014A,S11015A,S11019A<br>S11021A,S11022A,S11023A,S11024A,S11025A,S11029A,S11031A<br>S11032A,S11033A,S11034A,S11035A,S11039A,S1110XA,S1111XA<br>S1112XA,S1113XA,S1114XA,S1115XA,S1120XA,S1121XA,S1122XA<br>S1123XA,S1124XA,S1125XA,S1180XA,S1181XA,S1182XA,S1183XA<br>S1184XA,S1185XA,S1189XA,S1190XA,S1191XA,S1192XA,S1193XA<br>S1194XA,S1195XA,S162XXA | 8700,8702,8703,8704,8708,8709,8710,<br>8711,8712,8713,8715,8716<br>8717,87200,87201,87202,87210,87261,<br>87271,8730,8731,87320<br>87330,87341,87342,87343,87344,87349,<br>87351,87352,87353,87354<br>87359,87360,87361,87362,87364,87365,<br>87370,87371,87372,87374<br>87375,8738,8739,87401,87402,87411,<br>87412,8742,8743,8744,8745<br>8748,8749 |
| Open wounds of head and neck, subsequent encounter | S0100XD,S0101XD,S0102XD,S0103XD,S0104XD,S0105XD,S01101D<br>S01102D,S01109D,S01111D,S01112D,S01119D,S01121D,S01122D<br>S01129D,S01131D,S01132D,S01139D,S01141D,S01142D,S01149D<br>S01151D,S01152D,S01159D,S0120XD,S0121XD,S0122XD,S0123XD<br>S0124XD,S0125XD,S01301D,S01302D,S01309D,S01311D,S01312D<br>S01319D,S01321D,S01322D,S01329D,S01331D,S01332D,S01339D<br>S01341D,S01342D,S01349D,S01351D,S01352D,S01359D,S01401D<br>S01402D,S01409D,S01411D,S01412D,S01419D,S01421D,S01422D<br>S01429D,S01431D,S01432D,S01439D,S01441D,S01442D,S01449D<br>S01451D,S01452D,S01459D,S01501D,S01502D,S01511D,S01512D<br>S01521D,S01522D,S01531D,S01532D,S01541D,S01542D,S01551D<br>S01552D,S0180XD,S0181XD,S0182XD,S0183XD,S0184XD,S0185XD<br>S0190XD,S0191XD,S0192XD,S0193XD,S0194XD,S0195XD,S0520XD<br>S0521XD,S0522XD,S0530XD,S0531XD,S0532XD,S0540XD,S0541XD<br>S0542XD,S0550XD,S0551XD,S0552XD,S0560XD,S0561XD,S0562XD<br>S0570XD,S0571XD,S0572XD,S080XXD,S0912XD,S0920XD,S0921XD<br>S0922XD,S11011D,S11012D,S11013D,S11014D,S11015D,S11019D<br>S11021D,S11022D,S11023D,S11024D,S11025D,S11029D,S11031D<br>S11032D,S11033D,S11034D,S11035D,S11039D,S1110XD,S1111XD<br>S1112XD,S1113XD,S1114XD,S1115XD,S1120XD,S1121XD,S1122XD<br>S1123XD,S1124XD,S1125XD,S1180XD,S1181XD,S1182XD,S1183XD<br>S1184XD,S1185XD,S1189XD,S1190XD,S1191XD,S1192XD,S1193XD<br>S1194XD,S1195XD,S162XXD | V5889 |
| Open wounds of trunk, initial encounter | S21001A,S21002A,S21009A,S21011A,S21012A,S21019A,S21021A<br>S21022A,S21029A,S21031A,S21032A,S21039A,S21041A,S21042A<br>S21049A,S21051A,S21052A,S21059A,S21101A,S21102A,S21109A<br>S21111A,S21112A,S21119A,S21121A,S21122A,S21129A,S21131A<br>S21132A,S21139A,S21141A,S21142A,S21149A,S21151A,S21152A<br>S21159A,S21201A,S21202A,S21209A,S21211A,S21212A,S21219A<br>S21221A,S21222A,S21229A,S21231A,S21232A,S21239A,S21241A<br>S21242A,S21249A,S21251A,S21252A,S21259A,S21301A,S21302A<br>S21309A,S21311A,S21312A,S21319A,S21321A,S21322A,S21329A<br>S21331A,S21332A,S21339A,S21341A,S21342A,S21349A,S21351A<br>S21352A,S21359A,S21401A,S21402A,S21409A,S21411A,S21412A<br>S21419A,S21421A,S21422A,S21429A,S21431A,S21432A,S21439A<br>S21441A,S21442A,S21449A,S21451A,S21452A,S21459A,S2190XA<br>S2191XA,S2192XA,S2193XA,S2194XA,S2195XA,S29021A,S29022A<br>S29029A,S31000A,S31001A,S31010A,S31011A,S31020A,S31021A<br>S31030A,S31031A,S31040A,S31041A,S31050A,S31051A,S31100A | 8629,86813,86814,8750,8751,8760,8761,<br>8770,8771,8780,8781,8782<br>8783,8784,8785,8786,8787,8788,8789,<br>8790,8791,8792,8793,8794<br>8795,8796,8797,8798,8799 |

Continued on next page

Table S9 (continued).

| Diagnosis | ICD-10 | ICD-9 |
|---|---|---|
| | S31101A,S31102A,S31103A,S31104A,S31105A,S31109A,S31110A<br>S31111A,S31112A,S31113A,S31114A,S31115A,S31119A,S31120A<br>S31121A,S31122A,S31123A,S31124A,S31125A,S31129A,S31130A<br>S31131A,S31132A,S31133A,S31134A,S31135A,S31139A,S31140A<br>S31141A,S31142A,S31143A,S31144A,S31145A,S31149A,S31150A<br>S31151A,S31152A,S31153A,S31154A,S31155A,S31159A,S3120XA<br>S3121XA,S3122XA,S3123XA,S3124XA,S3125XA,S3130XA,S3131XA<br>S3132XA,S3133XA,S3134XA,S3135XA,S3140XA,S3141XA,S3142XA<br>S3143XA,S3144XA,S3145XA,S31501A,S31502A,S31511A,S31512A<br>S31521A,S31522A,S31531A,S31532A,S31541A,S31542A,S31551A<br>S31552A,S31600A,S31601A,S31602A,S31603A,S31604A,S31605A<br>S31609A,S31610A,S31611A,S31612A,S31613A,S31614A,S31615A<br>S31619A,S31620A,S31621A,S31622A,S31623A,S31624A,S31625A<br>S31629A,S31630A,S31631A,S31632A,S31633A,S31634A,S31635A<br>S31639A,S31640A,S31641A,S31642A,S31643A,S31644A,S31645A<br>S31649A,S31650A,S31651A,S31652A,S31653A,S31654A,S31655A<br>S31659A,S31801A,S31802A,S31803A,S31804A,S31805A,S31809A<br>S31811A,S31812A,S31813A,S31814A,S31815A,S31819A,S31821A<br>S31822A,S31823A,S31824A,S31825A,S31829A,S31831A,S31832A<br>S31833A,S31834A,S31835A,S31839A,S39021A,S39022A,S39023A | |
| Open wounds of trunk, subsequent encounter | S21001D,S21002D,S21009D,S21011D,S21012D,S21019D,S21021D<br>S21022D,S21029D,S21031D,S21032D,S21039D,S21041D,S21042D<br>S21049D,S21051D,S21052D,S21059D,S21101D,S21102D,S21109D<br>S21111D,S21112D,S21119D,S21121D,S21122D,S21129D,S21131D<br>S21132D,S21139D,S21141D,S21142D,S21149D,S21151D,S21152D<br>S21159D,S21201D,S21202D,S21209D,S21211D,S21212D,S21219D<br>S21221D,S21222D,S21229D,S21231D,S21232D,S21239D,S21241D<br>S21242D,S21249D,S21251D,S21252D,S21259D,S21301D,S21302D<br>S21309D,S21311D,S21312D,S21319D,S21321D,S21322D,S21329D<br>S21331D,S21332D,S21339D,S21341D,S21342D,S21349D,S21351D<br>S21352D,S21359D,S21401D,S21402D,S21409D,S21411D,S21412D<br>S21419D,S21421D,S21422D,S21429D,S21431D,S21432D,S21439D<br>S21441D,S21442D,S21449D,S21451D,S21452D,S21459D,S2190XD<br>S2191XD,S2192XD,S2193XD,S2194XD,S2195XD,S29021D,S29022D<br>S29029D,S31000D,S31001D,S31010D,S31011D,S31020D,S31021D<br>S31030D,S31031D,S31040D,S31041D,S31050D,S31051D,S31100D<br>S31101D,S31102D,S31103D,S31104D,S31105D,S31109D,S31110D<br>S31111D,S31112D,S31113D,S31114D,S31115D,S31119D,S31120D<br>S31121D,S31122D,S31123D,S31124D,S31125D,S31129D,S31130D<br>S31131D,S31132D,S31133D,S31134D,S31135D,S31139D,S31140D<br>S31141D,S31142D,S31143D,S31144D,S31145D,S31149D,S31150D<br>S31151D,S31152D,S31153D,S31154D,S31155D,S31159D,S3120XD<br>S3121XD,S3122XD,S3123XD,S3124XD,S3125XD,S3130XD,S3131XD<br>S3132XD,S3133XD,S3134XD,S3135XD,S3140XD,S3141XD,S3142XD<br>S3143XD,S3144XD,S3145XD,S31501D,S31502D,S31511D,S31512D<br>S31521D,S31522D,S31531D,S31532D,S31541D,S31542D,S31551D<br>S31552D,S31600D,S31601D,S31602D,S31603D,S31604D,S31605D<br>S31609D,S31610D,S31611D,S31612D,S31613D,S31614D,S31615D<br>S31619D,S31620D,S31621D,S31622D,S31623D,S31624D,S31625D<br>S31629D,S31630D,S31631D,S31632D,S31633D,S31634D,S31635D<br>S31639D,S31640D,S31641D,S31642D,S31643D,S31644D,S31645D<br>S31649D,S31650D,S31651D,S31652D,S31653D,S31654D,S31655D<br>S31659D,S31801D,S31802D,S31803D,S31804D,S31805D,S31809D<br>S31811D,S31812D,S31813D,S31814D,S31815D,S31819D,S31821D<br>S31822D,S31823D,S31824D,S31825D,S31829D,S31831D,S31832D<br>S31833D,S31834D,S31835D,S31839D,S39021D,S39022D,S39023D | V5889 |
| Open wounds to limbs, initial encounter | S41001A,S41002A,S41009A,S41011A,S41012A,S41019A,S41021A<br>S41022A,S41029A,S41031A,S41032A,S41039A,S41041A,S41042A<br>S41049A,S41051A,S41052A,S41059A,S41101A,S41102A,S41109A<br>S41111A,S41112A,S41119A,S41121A,S41122A,S41129A,S41131A<br>S41132A,S41139A,S41141A,S41142A,S41149A,S41151A,S41152A<br>S41159A,S46021A,S46022A,S46029A,S46121A,S46122A,S46129A<br>S46221A,S46222A,S46229A,S46321A,S46322A,S46329A,S46821A<br>S46822A,S46829A,S46921A,S46922A,S46929A,S51001A,S51002A<br>S51009A,S51011A,S51012A,S51019A,S51021A,S51022A,S51029A<br>S51031A,S51032A,S51039A,S51041A,S51042A,S51049A,S51051A<br>S51052A,S51059A,S51801A,S51802A,S51809A,S51811A,S51812A<br>S51819A,S51821A,S51822A,S51829A,S51831A,S51832A,S51839A<br>S51841A,S51842A,S51849A,S51851A,S51852A,S51859A,S56021A<br>S56022A,S56029A,S56121A,S56122A,S56123A,S56124A,S56125A<br>S56126A,S56127A,S56128A,S56129A,S56221A,S56222A,S56229A<br>S56321A,S56322A,S56329A,S56421A,S56422A,S56423A,S56424A<br>S56425A,S56426A,S56427A,S56428A,S56429A,S56521A,S56522A<br>S56529A,S56821A,S56822A,S56829A,S56921A,S56922A,S56929A<br>S61001A,S61002A,S61009A,S61011A,S61012A,S61019A,S61021A<br>S61022A,S61029A,S61031A,S61032A,S61039A,S61041A,S61042A<br>S61049A,S61051A,S61052A,S61059A,S61101A,S61102A,S61109A<br>S61111A,S61112A,S61119A,S61121A,S61122A,S61129A,S61131A<br>S61132A,S61139A,S61141A,S61142A,S61149A,S61151A,S61152A<br>S61159A,S61200A,S61201A,S61202A,S61203A,S61204A,S61205A | 88000,88001,88002,88003,88009,88010,<br>88011,88012,88013,88019<br>88020,88100,88101,88102,88110,88111,<br>88112,88120,88122,8820<br>8821,8822,8830,8831,8840,8841,8900,<br>8901,8902,8910,8911,8912<br>8920,8921,8930,8931,8941 |

Continued on next page

Table S9 (continued).

| Diagnosis | ICD-10 | ICD-9 |
|---|---|---|
| | S61206A,S61207A,S61208A,S61209A,S61210A,S61211A,S61212A S61213A,S61214A,S61215A,S61216A,S61217A,S61218A,S61219A S61220A,S61221A,S61222A,S61223A,S61224A,S61225A,S61226A S61227A,S61228A,S61229A,S61230A,S61231A,S61232A,S61233A S61234A,S61235A,S61236A,S61237A,S61238A,S61239A,S61240A S61241A,S61242A,S61243A,S61244A,S61245A,S61246A,S61247A S61248A,S61249A,S61250A,S61251A,S61252A,S61253A,S61254A S61255A,S61256A,S61257A,S61258A,S61259A,S61300A,S61301A S61302A,S61303A,S61304A,S61305A,S61306A,S61307A,S61308A S61309A,S61310A,S61311A,S61312A,S61313A,S61314A,S61315A S61316A,S61317A,S61318A,S61319A,S61320A,S61321A,S61322A S61323A,S61324A,S61325A,S61326A,S61327A,S61328A,S61329A S61330A,S61331A,S61332A,S61333A,S61334A,S61335A,S61336A S61337A,S61338A,S61339A,S61340A,S61341A,S61342A,S61343A S61344A,S61345A,S61346A,S61347A,S61348A,S61349A,S61350A S61351A,S61352A,S61353A,S61354A,S61355A,S61356A,S61357A S61358A,S61359A,S61401A,S61402A,S61409A,S61411A,S61412A S61419A,S61421A,S61422A,S61429A,S61431A,S61432A,S61439A S61441A,S61442A,S61449A,S61451A,S61452A,S61459A,S61501A S61502A,S61509A,S61511A,S61512A,S61519A,S61521A,S61522A S61529A,S61531A,S61532A,S61539A,S61541A,S61542A,S61549A S61551A,S61552A,S61559A,S66021A,S66022A,S66029A,S66120A S66121A,S66122A,S66123A,S66124A,S66125A,S66126A,S66127A S66128A,S66129A,S66221A,S66222A,S66229A,S66320A,S66321A S66322A,S66323A,S66324A,S66325A,S66326A,S66327A,S66328A S66329A,S66421A,S66422A,S66429A,S66520A,S66521A,S66522A S66523A,S66524A,S66525A,S66526A,S66527A,S66528A,S66529A S66821A,S66822A,S66829A,S66921A,S66922A,S66929A,S71001A S71002A,S71009A,S71011A,S71012A,S71019A,S71021A,S71022A S71029A,S71031A,S71032A,S71039A,S71041A,S71042A,S71049A S71051A,S71052A,S71059A,S71101A,S71102A,S71109A,S71111A S71112A,S71119A,S71121A,S71122A,S71129A,S71131A,S71132A S71139A,S71141A,S71142A,S71149A,S71151A,S71152A,S71159A S76021A,S76022A,S76029A,S76121A,S76122A,S76129A,S76221A S76222A,S76229A,S76321A,S76322A,S76329A,S76821A,S76822A S76829A,S76921A,S76922A,S76929A,S81001A,S81002A,S81009A S81011A,S81012A,S81019A,S81021A,S81022A,S81029A,S81031A S81032A,S81039A,S81041A,S81042A,S81049A,S81051A,S81052A S81059A,S81801A,S81802A,S81809A,S81811A,S81812A,S81819A S81821A,S81822A,S81829A,S81831A,S81832A,S81839A,S81841A S81842A,S81849A,S81851A,S81852A,S81859A,S86021A,S86022A S86029A,S86121A,S86122A,S86129A,S86221A,S86222A,S86229A S86321A,S86322A,S86329A,S86821A,S86822A,S86829A,S86921A S86922A,S86929A,S91001A,S91002A,S91009A,S91011A,S91012A S91019A,S91021A,S91022A,S91029A,S91031A,S91032A,S91039A S91041A,S91042A,S91049A,S91051A,S91052A,S91059A,S91101A S91102A,S91103A,S91104A,S91105A,S91106A,S91109A,S91111A S91112A,S91113A,S91114A,S91115A,S91116A,S91119A,S91121A S91122A,S91123A,S91124A,S91125A,S91126A,S91129A,S91131A S91132A,S91133A,S91134A,S91135A,S91136A,S91139A,S91141A S91142A,S91143A,S91144A,S91145A,S91146A,S91149A,S91151A S91152A,S91153A,S91154A,S91155A,S91156A,S91159A,S91201A S91202A,S91203A,S91204A,S91205A,S91206A,S91209A,S91211A S91212A,S91213A,S91214A,S91215A,S91216A,S91219A,S91221A S91222A,S91223A,S91224A,S91225A,S91226A,S91229A,S91231A S91232A,S91233A,S91234A,S91235A,S91236A,S91239A,S91241A S91242A,S91243A,S91244A,S91245A,S91246A,S91249A,S91251A S91252A,S91253A,S91254A,S91255A,S91256A,S91259A,S91301A S91302A,S91309A,S91311A,S91312A,S91319A,S91321A,S91322A S91329A,S91331A,S91332A,S91339A,S91341A,S91342A,S91349A S91351A,S91352A,S91359A,S96021A,S96022A,S96029A,S96121A S96122A,S96129A,S96221A,S96222A,S96229A,S96821A,S96822A S96829A,S96921A,S96922A,S96929A | |
| Open wounds to limbs, subsequent encounter | S41001D,S41002D,S41009D,S41011D,S41012D,S41019D,S41021D S41022D,S41029D,S41031D,S41032D,S41039D,S41041D,S41042D S41049D,S41051D,S41052D,S41059D,S41101D,S41102D,S41109D S41111D,S41112D,S41119D,S41121D,S41122D,S41129D,S41131D S41132D,S41139D,S41141D,S41142D,S41149D,S41151D,S41152D S41159D,S46021D,S46022D,S46029D,S46121D,S46122D,S46129D S46221D,S46222D,S46229D,S46321D,S46322D,S46329D,S46821D S46822D,S46829D,S46921D,S46922D,S46929D,S51001D,S51002D S51009D,S51011D,S51012D,S51019D,S51021D,S51022D,S51029D S51031D,S51032D,S51039D,S51041D,S51042D,S51049D,S51051D S51052D,S51059D,S51801D,S51802D,S51809D,S51811D,S51812D S51819D,S51821D,S51822D,S51829D,S51831D,S51832D,S51839D S51841D,S51842D,S51849D,S51851D,S51852D,S51859D,S56021D S56022D,S56029D,S56121D,S56122D,S56123D,S56124D,S56125D S56126D,S56127D,S56128D,S56129D,S56221D,S56222D,S56229D S56321D,S56322D,S56329D,S56421D,S56422D,S56423D,S56424D S56425D,S56426D,S56427D,S56428D,S56429D,S56521D,S56522D S56529D,S56821D,S56822D,S56829D,S56921D,S56922D,S56929D S61001D,S61002D,S61009D,S61011D,S61012D,S61019D,S61021D S61022D,S61029D,S61031D,S61032D,S61039D,S61041D,S61042D S61049D,S61051D,S61052D,S61059D,S61101D,S61102D,S61109D | V5889 |

Continued on next page

**Table S9 (continued).**

| Diagnosis | ICD-10 | ICD-9 |
|---|---|---|
| | S61111D,S61112D,S61119D,S61121D,S61122D,S61129D,S61131D<br>S61132D,S61139D,S61141D,S61142D,S61149D,S61151D,S61152D<br>S61159D,S61200D,S61201D,S61202D,S61203D,S61204D,S61205D<br>S61206D,S61207D,S61208D,S61209D,S61210D,S61211D,S61212D<br>S61213D,S61214D,S61215D,S61216D,S61217D,S61218D,S61219D<br>S61220D,S61221D,S61222D,S61223D,S61224D,S61225D,S61226D<br>S61227D,S61228D,S61229D,S61230D,S61231D,S61232D,S61233D<br>S61234D,S61235D,S61236D,S61237D,S61238D,S61239D,S61240D<br>S61241D,S61242D,S61243D,S61244D,S61245D,S61246D,S61247D<br>S61248D,S61249D,S61250D,S61251D,S61252D,S61253D,S61254D<br>S61255D,S61256D,S61257D,S61258D,S61259D,S61300D,S61301D<br>S61302D,S61303D,S61304D,S61305D,S61306D,S61307D,S61308D<br>S61309D,S61310D,S61311D,S61312D,S61313D,S61314D,S61315D<br>S61316D,S61317D,S61318D,S61319D,S61320D,S61321D,S61322D<br>S61323D,S61324D,S61325D,S61326D,S61327D,S61328D,S61329D<br>S61330D,S61331D,S61332D,S61333D,S61334D,S61335D,S61336D<br>S61337D,S61338D,S61339D,S61340D,S61341D,S61342D,S61343D<br>S61344D,S61345D,S61346D,S61347D,S61348D,S61349D,S61350D<br>S61351D,S61352D,S61353D,S61354D,S61355D,S61356D,S61357D<br>S61358D,S61359D,S61401D,S61402D,S61409D,S61411D,S61412D<br>S61419D,S61421D,S61422D,S61429D,S61431D,S61432D,S61439D<br>S61441D,S61442D,S61449D,S61451D,S61452D,S61459D,S61501D<br>S61502D,S61509D,S61511D,S61512D,S61519D,S61521D,S61522D<br>S61529D,S61531D,S61532D,S61539D,S61541D,S61542D,S61549D<br>S61551D,S61552D,S61559D,S66021D,S66022D,S66029D,S66120D<br>S66121D,S66122D,S66123D,S66124D,S66125D,S66126D,S66127D<br>S66128D,S66129D,S66221D,S66222D,S66229D,S66320D,S66321D<br>S66322D,S66323D,S66324D,S66325D,S66326D,S66327D,S66328D<br>S66329D,S66421D,S66422D,S66429D,S66520D,S66521D,S66522D<br>S66523D,S66524D,S66525D,S66526D,S66527D,S66528D,S66529D<br>S66821D,S66822D,S66829D,S66921D,S66922D,S66929D,S71001D<br>S71002D,S71009D,S71011D,S71012D,S71019D,S71021D,S71022D<br>S71029D,S71031D,S71032D,S71039D,S71041D,S71042D,S71049D<br>S71051D,S71052D,S71059D,S71101D,S71102D,S71109D,S71111D<br>S71112D,S71119D,S71121D,S71122D,S71129D,S71131D,S71132D<br>S71139D,S71141D,S71142D,S71149D,S71151D,S71152D,S71159D<br>S76021D,S76022D,S76029D,S76121D,S76122D,S76129D,S76221D<br>S76222D,S76229D,S76321D,S76322D,S76329D,S76821D,S76822D<br>S76829D,S76921D,S76922D,S76929D,S81001D,S81002D,S81009D<br>S81011D,S81012D,S81019D,S81021D,S81022D,S81029D,S81031D<br>S81032D,S81039D,S81041D,S81042D,S81049D,S81051D,S81052D<br>S81059D,S81801D,S81802D,S81809D,S81811D,S81812D,S81819D<br>S81821D,S81822D,S81829D,S81831D,S81832D,S81839D,S81841D<br>S81842D,S81849D,S81851D,S81852D,S81859D,S86021D,S86022D<br>S86029D,S86121D,S86122D,S86129D,S86221D,S86222D,S86229D<br>S86321D,S86322D,S86329D,S86821D,S86822D,S86829D,S86921D<br>S86922D,S86929D,S91001D,S91002D,S91009D,S91011D,S91012D<br>S91019D,S91021D,S91022D,S91029D,S91031D,S91032D,S91039D<br>S91041D,S91042D,S91049D,S91051D,S91052D,S91059D,S91101D<br>S91102D,S91103D,S91104D,S91105D,S91106D,S91109D,S91111D<br>S91112D,S91113D,S91114D,S91115D,S91116D,S91119D,S91121D<br>S91122D,S91123D,S91124D,S91125D,S91126D,S91129D,S91131D<br>S91132D,S91133D,S91134D,S91135D,S91136D,S91139D,S91141D<br>S91142D,S91143D,S91144D,S91145D,S91146D,S91149D,S91151D<br>S91152D,S91153D,S91154D,S91155D,S91156D,S91159D,S91201D<br>S91202D,S91203D,S91204D,S91205D,S91206D,S91209D,S91211D<br>S91212D,S91213D,S91214D,S91215D,S91216D,S91219D,S91221D<br>S91222D,S91223D,S91224D,S91225D,S91226D,S91229D,S91231D<br>S91232D,S91233D,S91234D,S91235D,S91236D,S91239D,S91241D<br>S91242D,S91243D,S91244D,S91245D,S91246D,S91249D,S91251D<br>S91252D,S91253D,S91254D,S91255D,S91256D,S91259D,S91301D<br>S91302D,S91309D,S91311D,S91312D,S91319D,S91321D,S91322D<br>S91329D,S91331D,S91332D,S91339D,S91341D,S91342D,S91349D<br>S91351D,S91352D,S91359D,S96021D,S96022D,S96029D,S96121D<br>S96122D,S96129D,S96221D,S96222D,S96229D,S96821D,S96822D<br>S96829D,S96921D,S96922D,S96929D | |
| Opioid-related disorders | F1110,F11120,F11121,F11122,F11129,F1113,F1114,F11150,F11151<br>F11159,F11181,F11182,F11188,F1119,F1120,F11220,F11221,F11222<br>F11229,F1123,F1124,F11250,F11251,F11259,F11281,F11282,F11288<br>F1129,F1190,F1191,F11920,F11921,F11922,F11929,F1193,F1194<br>F11950,F11951,F11959,F11981,F11982,F11988,F1199,T400X1A<br>T400X2A,T400X3A,T400X4A,T400X5A,T401X1A,T401X2A,T401X3A<br>T401X4A,T402X1A,T402X2A,T402X3A,T402X4A,T402X5A,T403X1A<br>T403X2A,T403X3A,T403X4A,T403X5A,T40411A,T40412A,T40413A<br>T40414A,T40415A,T40422A,T40423A,T40424A,T40425A,T40491A<br>T40492A,T40493A,T40494A,T40495A,T404X1A,T404X2A,T404X3A<br>T404X4A,T404X5A,T40601A,T40602A,T40603A,T40604A,T40605A<br>T40691A,T40692A,T40693A,T40694A,T40695A | 2920,29211,29212,2922,29281,29282,<br>29284,29285,29289,2929<br>30400,30401,30402,30550,30551,30552,<br>30553,96500,96501,96502<br>96509,99529,E8500,E8501,E8502,E9351,<br>E9352,E9500,E9504,E9620<br>E9800,E9804 |
| Osteoarthritis | M150,M151,M152,M153,M154,M158,M159,M160,M1610,M1611,M1612 | 71500,71504,71509,71510,71511,71512,<br>71513,71514,71515,71516 |

Continued on next page

Table S9 (continued).

| Diagnosis | ICD-10 | ICD-9 |
|---|---|---|
| | M162,M1630,M1631,M1632,M164,M1650,M1651,M1652,M166,M167,M169 | 71517,71520,71521,71522,71523,71524, 71525,71526,71527,71528 |
| | M170,M1710,M1711,M1712,M172,M1730,M1731,M1732,M174,M175,M179 | 71530,71534,71535,71536,71580,71589, 71590,71594,71595,71596 |
| | M180,M1810,M1811,M1812,M182,M1830,M1831,M1832,M184,M1850 M1851,M1852,M189,M19011,M19012,M19019,M19021,M19022,M19029 M19031,M19032,M19039,M19041,M19042,M19049,M19071,M19072 M19079,M1909,M19111,M19112,M19119,M19121,M19122,M19129,M19131 M19132,M19139,M19141,M19142,M19149,M19171,M19172,M19179,M1919 M19211,M19212,M19219,M19221,M19222,M19229,M19231,M19232 M19239,M19241,M19242,M19249,M19271,M19272,M19279,M1929,M1990 M1991,M1992,M1993 | |
| Osteomalacia | M830,M831,M832,M833,M834,M835,M838,M839 | 2682 |
| Osteomyelitis | A0105,A0224,A5443,H05021,H05022,H05023,H05029,M4620,M4621 | 0020,00324,09859,37603,73000,73001, 73002,73003,73004,73005 |
| | M4622,M4623,M4624,M4625,M4626,M4627,M4628,M8600,M86011,M86012 | 73006,73007,73008,73009,73010,73011, 73012,73013,73014,73015 |
| | M86019,M86021,M86022,M86029,M86031,M86032,M86039,M86041 | 73016,73017,73018,73019,73020,73028, 73030,73090,73098 |
| | M86042,M86049,M86051,M86052,M86059,M86061,M86062,M86069 M86071,M86072,M86079,M8608,M8609,M8610,M86111,M86112,M86119 M86121,M86122,M86129,M86131,M86132,M86139,M86141,M86142 M86149,M86151,M86152,M86159,M86161,M86162,M86169,M86171 M86172,M86179,M8618,M8619,M8620,M86211,M86212,M86219,M86221 M86222,M86229,M86231,M86232,M86239,M86241,M86242,M86249 M86251,M86252,M86259,M86261,M86262,M86269,M86271,M86272 M86279,M8628,M8629,M8630,M86311,M86312,M86319,M86321,M86322 M86329,M86331,M86332,M86339,M86341,M86342,M86349,M86351 M86352,M86359,M86361,M86362,M86369,M86371,M86372,M86379,M8638 M8639,M8640,M86411,M86412,M86419,M86421,M86422,M86429,M86431 M86432,M86439,M86441,M86442,M86449,M86451,M86452,M86459 M86461,M86462,M86469,M86471,M86472,M86479,M8648,M8649,M8650 M86511,M86512,M86519,M86521,M86522,M86529,M86531,M86532 M86539,M86541,M86542,M86549,M86551,M86552,M86559,M86561 M86562,M86569,M86571,M86572,M86579,M8658,M8659,M8660,M86611 M86612,M86619,M86621,M86622,M86629,M86631,M86632,M86639 M86641,M86642,M86649,M86651,M86652,M86659,M86661,M86662 M86669,M86671,M86672,M86679,M8668,M8669,M868X0,M868X1,M868X2 M868X3,M868X4,M868X5,M868X6,M868X7,M868X8,M868X9,M869 | |
| Osteoporosis | M8000XA,M80011A,M80012A,M80019A,M80021A,M80022A,M80029A | 73300,73301,73302,73303,73309,73310, 73311,73312,73313,73314 |
| | M80031A,M80032A,M80039A,M80041A,M80042A,M80049A,M80051A M80052A,M80059A,M80061A,M80062A,M80069A,M80071A,M80072A M80079A,M8008XA,M800AXA,M8080XA,M80811A,M80812A,M80819A M80821A,M80822A,M80829A,M80831A,M80832A,M80839A,M80841A M80842A,M80849A,M80851A,M80852A,M80859A,M80861A,M80862A M80869A,M80871A,M80872A,M80879A,M8088XA,M808AXA,M810,M816 M818 | 73315,73316,73319 |
| Other aftercare | Z09,Z430,Z431,Z432,Z433,Z434,Z435,Z436,Z437,Z438,Z439,Z471 | V420,V421,V426,V427,V4281,V4289, V429,V5401,V5481,V5482,V5489 |
| encounter | Z472,Z4731,Z4732,Z4733,Z4781,Z4782,Z4789,Z4800,Z4801,Z4802 | V550,V551,V552,V553,V554,V555,V556, V557,V558,V559,V561,V562 |
| | Z4803,Z481,Z4821,Z4822,Z4823,Z4824,Z48280,Z48288,Z48290 | V5631,V5632,V5830,V5831,V5832, V5841,V5844,V5849,V5871,V5872 |
| | Z48298,Z48810,Z48811,Z48812,Z48813,Z48814,Z48815,Z48816 | V5873,V5874,V5875,V5876,V5877, V5883,V5889,V589,V667,V6700 |
| | Z48817,Z4889,Z4901,Z4902,Z4931,Z4932,Z515,Z516,Z5181,Z5189 | V6709,V671,V672,V673,V674,V6751, V6759,V676,V679 |
| Other and ill-defined | A5204,A5205,G460,G461,G462,G463,G464,G465,G466,G467,G468,I671 | 0463,09389,34839,4358,4359,436,4370, 4371,4373,4374,4375,4376 |
| cerebrovascular disease | I672,I673,I675,I676,I677,I6781,I6782,I6783,I67841,I67848 I67850,I67858,I6789,I679,I680,I682,I688 | 4378,4379 |
| Other and ill-defined | I253,I2541,I510,I511,I512,I513,I515,I517,I5181,I5189,I519,I52 | 41410,41411,41419,4291,4293,4295,4296, 42971,42979,42981,42982 |
| heart disease | I5A,M0530,M05311,M05312,M05319,M05321,M05322,M05329,M05331 M05332,M05339,M05341,M05342,M05349,M05351,M05352,M05359 M05361,M05362,M05369,M05371,M05372,M05379,M0539 | 42983,42989,4299,7142 |
| Other general signs | R252,R293,R294,R296,R29890,R29891,R29898,R2991,R490,R491 | 33819,5277,71965,72982,72989,78064, 78065,7808,78091,78092 |
| and symptoms | R4921,R4922,R498,R499,R52,R54,R590,R591,R599,R600,R601,R609 | 78094,78096,78099,7810,7815,78191, 78192,78193,78199,7823,7830 |
| | R61,R620,R6250,R6251,R6252,R6259,R627,R630,R631,R632,R633 | 7831,78321,78322,7833,78340,78341, 78342,78343,7835,7836,7837 |
| | R6330,R6331,R6332,R6339,R634,R635,R636,R638,R64,R6510,R6511 | 7839,78440,78441,78442,78443,78444, 78449,78492,7856,7964,7969 |
| | R680,R6811,R6812,R6813,R6819,R682,R683,R6881,R6882,R6883 | 797,7980,7994,79981,79982,79989,7999, 99593,99594,V419 |
| | R6884,R6889,R69,R99 | |

Continued on next page

**Table S9 (continued).**

| Diagnosis | ICD-10 | ICD-9 |
|---|---|---|
| Other nervous system disorders (neither hereditary nor degenerative) | A1789,A179,B2701,B2711,B2781,B2791,E0840,E0844,E0940,E0944 E1040,E1044,E1140,E1144,E1340,E1344,G08,G130,G131,G132,G138 G360,G361,G368,G369,G370,G371,G372,G373,G374,G375,G378,G379 G460,G461,G462,G463,G464,G465,G466,G467,G468,G64,G908,G909 G90A,G910,G911,G912,G913,G914,G918,G919,G92,G9200,G9201,G9202 G9203,G9204,G9205,G928,G929,G930,G932,G933,G9331,G9332,G9339 G9340,G9341,G9349,G935,G936,G937,G9381,G9382,G9389,G939,G94 G950,G9511,G9519,G9520,G9529,G9581,G9589,G959,G960,G9600 G9601,G9602,G9608,G9609,G9611,G9612,G9619,G96191,G96198,G968 G96810,G96811,G96819,G9689,G969,G980,G988,G998,I673,I674 J1081,J1181,K7682,M3506,M3507 | 01380,01790,0463,075,24960,25060,25061, 32371,32372,3239,325 3313,3314,3315,3317,33181,3360,3361, 3368,3369,3379,3410,3411 34120,34121,3418,3419,3480,3482,34830, 34831,34839,3484,3485 34881,34882,34889,3489,3492,34939, 34981,34982,34989,3499,3535 3572,3573,35789,38861,4358,4372,4378, 4878,78079 |
| Other nervous system disorders (often hereditary or degenerative) | E7500,E7501,E7502,E7509,E7510,E7511,E7519,E7523,E7525,E7526 E7529,E754,G10,G110,G111,G1110,G1111,G1119,G112,G113,G114 G118,G119,G120,G121,G1220,G1221,G1222,G1223,G1224,G1225,G1229 G128,G129,G210,G2111,G2119,G212,G213,G214,G218,G219,G230,G231 G232,G238,G239,G2401,G2402,G2409,G241,G242,G243,G244,G245 G248,G249,G250,G251,G252,G253,G254,G255,G2561,G2569,G2570 G2571,G2579,G2581,G2582,G2583,G2589,G259,G26,G312,G3181,G3182 G3184,G3185,G3189,G319,G320,G3281,G3289,G9001,G9009,G901,G903 G904,G990,G992 | 3300,3301,3308,3316,3317,33183,33189, 3319,3320,3321,3330,3331 3332,3333,3334,3335,3336,33372,33379, 33381,33382,33383,33384 33385,33389,33390,33391,33392,33393, 33394,33399,3340,3341 3342,3343,3344,3348,3349,3350,33510, 33511,33519,33520,33521 33522,33524,33529,3359,3362,3363,33700, 33701,33709,3371,3373 34989,7428 |
| Other specified CNS infection and poliomyelitis | A5210,A5211,A5212,A5217,A5219,A522,A523,A800,A801,A802,A8030 A8039,A804,A809,A8100,A8101,A8109,A812,A8181,A8182,A8183 A8189,A819,A820,A821,A829,A880,A881,A888,A89,G14 | 04510,04520,04590,0460,04611,04619, 0463,04671,04672,04679 0468,0469,048,0498,0499,071,07881,0940, 0941,0943,09482,09483 09489,0949,138 |
| Other specified and unspecified circulatory disease | A5200,A5202,A5209,E0851,E0951,E1051,E1151,E1351,I770,I771 I772,I773,I774,I775,I776,I77810,I77811,I77812,I77819,I7782 I7789,I779,I780,I781,I788,I789,I791,I798,I998,I999 | 0931,09389,0939,24970,25070,25071, 44381,4470,4471,4472,4473 4474,4475,4476,44770,44771,44772,44773, 4478,4479,4480,4481 4489,45989,4599 |
| Other specified and unspecified congenital anomalies | P092,P093,Q800,Q801,Q802,Q803,Q804,Q808,Q809,Q810,Q811,Q812 Q818,Q819,Q820,Q821,Q822,Q823,Q824,Q825,Q826,Q828,Q829,Q830 Q831,Q832,Q833,Q838,Q839,Q840,Q841,Q842,Q843,Q844,Q845,Q846 Q848,Q849,Q851,Q858,Q8581,Q8582,Q8583,Q8589,Q859,Q860,Q861 Q862,Q868,Q870,Q871,Q8711,Q8719,Q872,Q873,Q8740,Q87410,Q87418 Q8742,Q8743,Q875,Q8781,Q8782,Q8789,Q8901,Q8909,Q891,Q892,Q893 Q894,Q897,Q898,Q899 | 75555,7560,7570,7571,7572,75731,75732, 75733,75739,7574,7575 7576,7578,7579,7590,7591,7592,7593, 7594,7595,7596,7597,75981 75982,75989,7599,76071,76077,76079 |
| Other specified and unspecified diseases of bladder and urethra | A1812,N310,N311,N312,N318,N319,N320,N321,N322,N323,N3281 N3289,N329,N33,N35010,N35011,N35012,N35013,N35014,N35016 N35021,N35028,N35111,N35112,N35113,N35114,N35116,N35119,N3512 N358,N35811,N35812,N35813,N35814,N35816,N35819,N3582,N359 N35911,N35912,N35913,N35914,N35916,N35919,N3592,N360,N361 N362,N3641,N3642,N3643,N3644,N365,N368,N369,N37,N99110,N99111 N99112,N99113,N99114,N99115,N99116,N9912 | 01610,5960,5961,5962,5963,5964,59651, 59652,59653,59654,59655 59659,5966,5967,59689,5969,59800,59801, 5981,5982,5988,5989 5991,5992,5993,5994,5995,59981,59982, 59984,5999 |
| Other specified and unspecified diseases of kidney and ureters | A1811,A5421,N130,N131,N132,N1330,N1339,N134,N135,N138,N139 N23,N250,N251,N2581,N2589,N259,N261,N262,N270,N271,N279,N280 N281,N2881,N2882,N2883,N2884,N2885,N2886,N2889,N289,N29 O26831,O26832,O26833,O26839 | 01600,01620,09819,40591,587,5880,5881, 58881,58889,5889,5890 5891,5899,5903,591,5930,5931,5932,5933, 5934,5935,59373,59381 59382,59389,5939,59960,59969,64620, 64621,64622,64623,7880 |
| Other specified and unspecified disorders of stomach and duodenum | K30,K310,K311,K312,K313,K314,K316,K317,K31811,K31819,K3182 K3183,K3184,K3189,K319,K31A0,K31A11,K31A12,K31A13,K31A14 K31A15,K31A19,K31A21,K31A22,K31A29 | 2111,5360,5361,5363,5368,5369,5370, 5371,5374,5375,5376,53781 53782,53783,53784,53789,5379 |
| Other specified and unspecified disorders of the ear | A186,B3784,H6000,H6001,H6002,H6003,H6010,H6011,H6012,H6013 H6020,H6021,H6022,H6023,H60311,H60312,H60313,H60319,H60321 H60322,H60323,H60329,H60331,H60332,H60333,H60339,H60391 H60392,H60393,H60399,H6040,H6041,H6042,H6043,H60501,H60502 | 01740,11282,31532,38000,38001,38002, 38003,38010,38011,38012 38013,38014,38015,38021,38022,38023, 38030,38031,38032,38039 3804,38050,38051,38052,38053,38081, 38089,3809,38800,38830 38831,38832,38840,38841,38842,38844, 3885,38860,38869,38870 |

Continued on next page

Table S9 (continued).

| Diagnosis | ICD-10 | ICD-9 |
|---|---|---|
| | H60503,H60509,H60511,H60512,H60513,H60519,H60521,H60522<br>H60523,H60529,H60531,H60532,H60533,H60539,H60541,H60542<br>H60543,H60549,H60551,H60552,H60553,H60559,H60591,H60592<br>H60593,H60599,H6060,H6061,H6062,H6063,H608X1,H608X2,H608X3<br>H608X9,H6090,H6091,H6092,H6093,H61001,H61002,H61003,H61009<br>H61011,H61012,H61013,H61019,H61021,H61022,H61023,H61029<br>H61031,H61032,H61033,H61039,H61101,H61102,H61103,H61109<br>H61111,H61112,H61113,H61119,H61121,H61122,H61123,H61129<br>H61191,H61192,H61193,H61199,H6120,H6121,H6122,H6123,H61301<br>H61302,H61303,H61309,H61311,H61312,H61313,H61319,H61321<br>H61322,H61323,H61329,H61391,H61392,H61393,H61399,H61811<br>H61812,H61813,H61819,H61891,H61892,H61893,H61899,H6190,H6191<br>H6192,H6193,H6240,H6241,H6242,H6243,H628X1,H628X2,H628X3<br>H628X9,H9201,H9202,H9203,H9209,H9210,H9211,H9212,H9213,H9220<br>H9221,H9222,H9223,H93091,H93092,H93093,H93099,H9311,H9312<br>H9313,H9319,H93211,H93212,H93213,H93219,H93221,H93222,H93223<br>H93229,H93231,H93232,H93233,H93239,H93241,H93242,H93243<br>H93249,H9325,H93291,H93292,H93293,H93299,H933X1,H933X2,H933X3<br>H933X9,H938X1,H938X2,H938X3,H938X9,H9390,H9391,H9392,H9393<br>H93A1,H93A2,H93A3,H93A9,H9400,H9401,H9402,H9403,H9480,H9481<br>H9482,H9483 | 3888,3889,V413 |
| Other specified and<br>unspecified endocrine<br>disorders | A187,A1882,A391,E15,E160,E161,E162,E163,E164,E168,E169,E200<br>E201,E208,E209,E210,E211,E212,E213,E214,E215,E241,E242,E243<br>E244,E248,E249,E250,E258,E259,E2601,E2602,E2609,E261,E2681<br>E2689,E269,E270,E271,E272,E273,E2740,E2749,E275,E278,E279<br>E280,E281,E282,E28310,E28319,E2839,E288,E289,E290,E291,E298<br>E299,E300,E301,E308,E309,E310,E311,E3120,E3121,E3122,E3123<br>E318,E319,E320,E321,E328,E329,E340,E341,E342,E343,E3430,E3431<br>E34321,E34322,E34328,E34329,E3439,E344,E3450,E3451,E3452,E348<br>E349,E35 | 01760,01790,0363,2468,2510,2511,2512,<br>2514,2515,2518,2519<br>25200,25201,25202,25208,2521,2528,2529,<br>2530,2538,2540,2541<br>2548,2549,2550,25510,25511,25512,25513,<br>25514,2552,2553,25541<br>25542,2556,2558,2559,2560,2561,25631,<br>25639,2564,2568,2569<br>2570,2572,2578,2579,25801,25802,25803,<br>2581,2588,2589,2590<br>2591,2592,2593,2594,25950,25951,25952,<br>2598,2599,27549 |
| Other specified and<br>unspecified<br>gastrointestinal<br>disorders | A1883,B5732,K550,K55011,K55012,K55019,K55021,K55022,K55029<br>K55031,K55032,K55039,K55041,K55042,K55049,K55051,K55052<br>K55059,K55061,K55062,K55069,K551,K5520,K5521,K5530,K5531<br>K5532,K5533,K558,K559,K580,K581,K582,K588,K589,K5900,K5901<br>K5902,K5903,K5904,K5909,K591,K592,K593,K5931,K5939,K598,K5981<br>K5989,K599,K632,K633,K634,K6381,K6389,K639,K660,K661,K668<br>K669,K689,K900,K901,K902,K904,K9041,K9049,K9081,K9089,K909<br>K9281,K9289,K929,M3508 | 01480,0402,0861,538,5570,5571,5579,<br>56400,56401,56402,56409<br>5641,5645,5647,56481,56489,5649,56739,<br>5680,56881,56889,5689<br>56981,56982,56984,56985,56986,56989,<br>5699,5790,5791,5792,5798<br>5799 |
| Other specified and<br>unspecified<br>hematologic<br>conditions | A1885,B510,D730,D731,D732,D733,D734,D735,D7389,D739,D740,D748<br>D749,D750,D751,D7581,D7589,D759,D75A,D77,M363 | 01770,0849,2890,2894,28950,28951,28959,<br>2896,2897,28983,28989<br>2899,7132 |
| Other specified and<br>unspecified liver<br>disease | A5274,B670,B675,K700,K702,K7030,K7031,K709,K710,K7110,K7111<br>K717,K718,K719,K740,K7400,K7401,K7402,K741,K742,K743,K744<br>K745,K7460,K7469,K751,K7589,K759,K760,K761,K762,K763,K764<br>K765,K766,K767,K7681,K7682,K7689,K769,K77,O26611,O26612<br>O26613,O26619,O2662,O2663 | 0952,0953,1220,1225,570,5710,5712,5713,<br>5715,5716,5718,5719<br>5721,5722,5723,5724,5730,5733,5734,<br>5735,5738,5739,64670,64671<br>64673,64684 |
| Other specified and<br>unspecified lower<br>respiratory disease | A065,A157,A158,A159,B400,B401,B402,B410,B420,B440,B441,B450<br>B460,B671,J182,J22,J810,J811,J82,J8281,J8282,J8283,J8289<br>J8401,J8402,J8403,J8409,J8410,J84111,J84112,J84113,J84114<br>J84115,J84116,J84117,J8417,J84170,J84178,J842,J8481,J8482<br>J8483,J84841,J84842,J84843,J84848,J8489,J849,J852,J9801,J9809<br>J984,J986,J988,J989,J99,M0510,M05111,M05112,M05119,M05121<br>M05122,M05129,M05131,M05132,M05139,M05141,M05142,M05149<br>M05151,M05152,M05159,M05161,M05162,M05169,M05171,M05172<br>M05179,M0519 | 0064,01090,01280,1160,1161,1171,1173,<br>1175,1177,1221,4846,5130<br>514,515,5160,5161,5162,51630,51631,<br>51632,51633,51634,51635<br>51636,51637,5164,5165,51661,51662,<br>51663,51664,51669,5168,5169<br>5178,5183,5184,51889,51911,51919,5194,<br>5198,5199,71481 |
| Other specified and<br>unspecified mood<br>disorders | F0630,F348,F3481,F3489,F349,F39 | 29383,29690,29699 |
| Other specified and<br>unspecified | D813,D8130,D8131,D8132,D8139,D815,D81810,D81818,D81819,E65<br>E663,E670,E671,E672,E673,E678,E700,E701,E7020,E7021,E7029 | 2662,2700,2701,2702,2703,2704,2705,<br>2706,2707,2708,2709,2710<br>2711,2712,2713,2714,2718,2719,2725,<br>2726,2727,2728,2729,2734 |

Continued on next page

**Table S9 (continued).**

| Diagnosis | ICD-10 | ICD-9 |
|---|---|---|
| nutritional and metabolic disorders | E7030,E70310,E70311,E70318,E70319,E70320,E70321,E70328,E70329<br>E70330,E70331,E70338,E70339,E7039,E7040,E7041,E7049,E705,E708<br>E7081,E7089,E709,E710,E71110,E71111,E71118,E71120,E71121<br>E71128,E7119,E712,E7130,E71310,E71311,E71312,E71313,E71314<br>E71318,E7132,E7139,E7140,E7141,E7142,E7143,E71440,E71448<br>E7150,E71510,E71511,E71518,E71520,E71521,E71522,E71528,E71529<br>E7153,E71540,E71541,E71542,E71548,E7200,E7201,E7202,E7203<br>E7204,E7209,E7210,E7211,E7212,E7219,E7220,E7221,E7222,E7223<br>E7229,E723,E724,E7250,E7251,E7252,E7253,E7259,E728,E7281<br>E7289,E729,E730,E731,E738,E739,E7400,E7401,E7402,E7403,E7404<br>E7409,E7410,E7411,E7412,E7419,E7420,E7421,E7429,E7431,E7439<br>E744,E748,E74810,E74818,E74819,E7489,E749,E7500,E7501,E7502<br>E7509,E7510,E7511,E7519,E7521,E7522,E7523,E75240,E75241<br>E75242,E75243,E75244,E75248,E75249,E7525,E7526,E7529,E753<br>E754,E755,E756,E7601,E7602,E7603,E761,E76210,E76211,E76219<br>E7622,E7629,E763,E768,E769,E770,E771,E778,E779,E786,E7870<br>E7871,E7872,E7879,E7881,E7889,E789,E790,E791,E792,E798,E799<br>E800,E801,E8020,E8021,E8029,E803,E804,E805,E806,E807,E8300<br>E8301,E8309,E8310,E83110,E83111,E83118,E83119,E8319,E832<br>E8330,E8331,E8332,E8339,E8340,E8341,E8342,E8349,E8350,E8351<br>E8352,E8359,E8381,E8389,E839,E850,E851,E852,E853,E854,E858<br>E8581,E8582,E8589,E859,E8801,E8802,E8809,E881,E882,E883,E8840<br>E8841,E8842,E8849,E8881,E8889,E889,P091 | 2738,27501,27502,27503,27509,2751,2752,<br>2753,27540,27541,27542<br>27549,2755,2758,2759,2771,2772,27730,<br>27731,27739,2774,2775<br>2776,2777,27781,27782,27783,27784,<br>27785,27786,27787,27788<br>27789,2779,27802,2781,2782,2783,2784,<br>2788,3300,3301,6868<br>75989,7906 |
| Other specified and unspecified perinatal conditions | P080,P081,P0821,P0822,P09,P091,P092,P093,P094,P095,P096,P098<br>P099,P221,P290,P2911,P2912,P292,P293,P2930,P2938,P294,P2981<br>P2989,P299,P700,P701,P702,P703,P704,P708,P709,P710,P711,P712<br>P713,P714,P718,P719,P720,P721,P722,P728,P729,P740,P741,P742<br>P7421,P7422,P743,P7431,P7432,P744,P7441,P74421,P74422,P7449<br>P745,P746,P748,P749,P800,P808,P809,P810,P818,P819,P830,P831<br>P832,P8330,P8339,P834,P835,P836,P838,P8381,P8388,P839,P930<br>P938,P940,P941,P942,P948,P949,P95,P960,P963,P965,P9681,P9682<br>P9683,P9689,P969 | 74783,7608,7660,7661,76621,76622,7706,<br>7750,7751,7752,7753<br>7754,7755,7756,7757,77589,7759,7780,<br>7781,7782,7783,7784,7785<br>7786,7787,7788,7789,7794,77981,77982,<br>77983,77984,77985,77989<br>7799,7966 |
| Other specified and unspecified skin disorders | A5132,B000,B001,B372,B383,B403,B421,B430,B432,B452,B463,B781<br>L300,L301,L302,L308,L309,L560,L561,L562,L563,L564,L565,L570<br>L571,L572,L573,L574,L575,L600,L601,L602,L603,L604,L605,L608<br>L609,L62,L630,L631,L632,L638,L639,L640,L648,L649,L650,L651<br>L652,L658,L659,L660,L662,L663,L664,L668,L669,L670,L671,L678<br>L679,L680,L681,L682,L683,L688,L689,L720,L7211,L7212,L722,L723<br>L728,L729,L730,L731,L739,L740,L741,L742,L743,L744,L74510<br>L74511,L74512,L74513,L74519,L7452,L748,L749,L750,L751,L752<br>L758,L759,L80,L810,L811,L812,L813,L814,L815,L816,L817,L818<br>L819,L820,L821,L83,L84,L850,L851,L852,L853,L858,L859,L86,L870<br>L871,L872,L878,L879,L900,L901,L902,L903,L904,L905,L906,L908<br>L909,L910,L918,L919,L921,L922,L923,L928,L929,L940,L941,L942<br>L943,L944,L946,L948,L949,L980,L985,L986,L987,L988,L989,L99 | 0540,05473,09182,1123,1141,1160,1171,<br>1172,1175,1177,1360,6861<br>6868,69272,69273,69275,69282,6929,700,<br>7010,7011,7012,7013<br>7014,7015,7018,7019,7020,70211,70219,<br>7030,7038,7039,70400<br>70401,70402,70409,7041,7042,7043,70441,<br>70442,7048,7049,7050<br>7051,70521,70522,70581,70582,70589,<br>7059,7061,7062,7068,7069<br>70900,70901,70909,7092,7093,7094,7098,<br>7099 |
| Other specified and unspecified upper respiratory disease | A5005,J300,J301,J302,J305,J3081,J3089,J309,J310,J311,J312<br>J330,J331,J338,J339,J340,J341,J342,J343,J3481,J3489,J349,J370<br>J371,J3800,J3801,J3802,J381,J382,J383,J384,J385,J386,J387<br>J392,J393,J398,J399 | 0900,470,4710,4711,4718,4719,4720,4721,<br>4722,4760,4761,4770<br>4771,4772,4778,4779,4780,47811,47819,<br>47825,47826,47829,47830<br>47831,47832,47833,47834,4784,4785,4786,<br>47871,47874,47875<br>47879,4788,4789,51919 |
| Other specified bone disease and musculoskeletal deformities | A5002,A5051,A5055,A5056,A5146,A5216,A5277,B672,L4052,M830<br>M831,M832,M833,M834,M838,M839,M8480,M84811,M84812,M84819<br>M84821,M84822,M84829,M84831,M84832,M84833,M84834,M84839<br>M84841,M84842,M84849,M84851,M84852,M84859,M84861,M84862<br>M84863,M84864,M84869,M84871,M84872,M84879,M8488,M849,M8500<br>M85011,M85012,M85019,M85021,M85022,M85029,M85031,M85032<br>M85039,M85041,M85042,M85049,M85051,M85052,M85059,M85061<br>M85062,M85069,M85071,M85072,M85079,M8508,M8509,M8510,M85111<br>M85112,M85119,M85121,M85122,M85129,M85131,M85132,M85139<br>M85141,M85142,M85149,M85151,M85152,M85159,M85161,M85162<br>M85169,M85171,M85172,M85179,M8518,M8519,M852,M8530,M85311<br>M85312,M85319,M85321,M85322,M85329,M85331,M85332,M85339<br>M85341,M85342,M85349,M85351,M85352,M85359,M85361,M85362<br>M85369,M85371,M85372,M85379,M8538,M8539,M8540,M85411,M85412 | 0900,0905,09161,0940,0955,1223,2682,<br>6960,7135,73081,73082<br>73083,73084,73085,73086,73087,73088,<br>73089,7310,7311,7312,7313<br>7318,7321,7322,7323,7324,7325,7326,<br>7327,7328,7329,73321,73322<br>73329,7333,7335,7336,7337,73390,73391,<br>73392,73399,73811,73812<br>V454 |

Continued on next page

Table S9 (continued).

| Diagnosis | ICD-10 | ICD-9 |
|---|---|---|
| | M85419,M85421,M85422,M85429,M85431,M85432,M85439,M85441<br>M85442,M85449,M85451,M85452,M85459,M85461,M85462,M85469<br>M85471,M85472,M85479,M8548,M8550,M85511,M85512,M85519,M85521<br>M85522,M85529,M85531,M85532,M85539,M85541,M85542,M85549<br>M85551,M85552,M85559,M85561,M85562,M85569,M85571,M85572<br>M85579,M8558,M8559,M8560,M85611,M85612,M85619,M85621,M85622<br>M85629,M85631,M85632,M85639,M85641,M85642,M85649,M85651<br>M85652,M85659,M85661,M85662,M85669,M85671,M85672,M85679,M8568<br>M8569,M8580,M85811,M85812,M85819,M85821,M85822,M85829,M85831<br>M85832,M85839,M85841,M85842,M85849,M85851,M85852,M85859<br>M85861,M85862,M85869,M85871,M85872,M85879,M8588,M8589,M859<br>M880,M881,M88811,M88812,M88819,M88821,M88822,M88829,M88831<br>M88832,M88839,M88841,M88842,M88849,M88851,M88852,M88859<br>M88861,M88862,M88869,M88871,M88872,M88879,M8888,M8889,M889<br>M8900,M89011,M89012,M89019,M89021,M89022,M89029,M89031,M89032<br>M89039,M89041,M89042,M89049,M89051,M89052,M89059,M89061<br>M89062,M89069,M89071,M89072,M89079,M8908,M8909,M89121,M89122<br>M89123,M89124,M89125,M89126,M89127,M89128,M89129,M89131<br>M89132,M89133,M89134,M89138,M89139,M89151,M89152,M89153<br>M89154,M89155,M89156,M89157,M89158,M89159,M89160,M89161<br>M89162,M89163,M89164,M89165,M89166,M89167,M89168,M89169,M8918<br>M8920,M89211,M89212,M89219,M89221,M89222,M89229,M89231,M89232<br>M89233,M89234,M89239,M89241,M89242,M89249,M89251,M89252<br>M89259,M89261,M89262,M89263,M89264,M89269,M89271,M89272<br>M89279,M8928,M8929,M8930,M89311,M89312,M89319,M89321,M89322<br>M89329,M89331,M89332,M89333,M89334,M89339,M89341,M89342<br>M89349,M89351,M89352,M89359,M89361,M89362,M89363,M89364<br>M89369,M89371,M89372,M89379,M8938,M8939,M8940,M89411,M89412<br>M89419,M89421,M89422,M89429,M89431,M89432,M89439,M89441<br>M89442,M89449,M89451,M89452,M89459,M89461,M89462,M89469<br>M89471,M89472,M89479,M8948,M8949,M8950,M89511,M89512,M89519<br>M89521,M89522,M89529,M89531,M89532,M89539,M89541,M89542<br>M89549,M89551,M89552,M89559,M89561,M89562,M89569,M89571<br>M89572,M89579,M8958,M8959,M8970,M89711,M89712,M89719,M89721<br>M89722,M89729,M89731,M89732,M89739,M89741,M89742,M89749<br>M89751,M89752,M89759,M89761,M89762,M89769,M89771,M89772<br>M89779,M8978,M8979,M898X0,M898X1,M898X2,M898X3,M898X4,M898X5<br>M898X6,M898X7,M898X8,M898X9,M899,M9060,M90611,M90612,M90619<br>M90621,M90622,M90629,M90631,M90632,M90639,M90641,M90642<br>M90649,M90651,M90652,M90659,M90661,M90662,M90669,M90671<br>M90672,M90679,M9068,M9069,M9080,M90811,M90812,M90819,M90821<br>M90822,M90829,M90831,M90832,M90839,M90841,M90842,M90849<br>M90851,M90852,M90859,M90861,M90862,M90869,M90871,M90872<br>M90879,M9088,M9089,M910,M9110,M9111,M9112,M9120,M9121,M9122<br>M9130,M9131,M9132,M9140,M9141,M9142,M9180,M9181,M9182,M9190<br>M9191,M9192,M9200,M9201,M9202,M9210,M9211,M9212,M92201,M92202<br>M92209,M92211,M92212,M92219,M92221,M92222,M92229,M92291<br>M92292,M92299,M9230,M9231,M9232,M9240,M9241,M9242,M9250<br>M92501,M92502,M92503,M92509,M9251,M92511,M92512,M92513,M92519<br>M9252,M92521,M92522,M92523,M92529,M92591,M92592,M92593,M92599<br>M9260,M9261,M9262,M9270,M9271,M9272,M928,M929,M93001,M93002<br>M93003,M93004,M93011,M93012,M93013,M93014,M93021,M93022<br>M93023,M93024,M93031,M93032,M93033,M93034,M93041,M93042<br>M93043,M93044,M93051,M93052,M93053,M93054,M93061,M93062<br>M93063,M93064,M93071,M93072,M93073,M93074,M931,M9320,M93211<br>M93212,M93219,M93221,M93222,M93229,M93231,M93232,M93239<br>M93241,M93242,M93249,M93251,M93252,M93259,M93261,M93262<br>M93269,M93271,M93272,M93279,M9328,M9329,M9380,M93811,M93812<br>M93819,M93821,M93822,M93829,M93831,M93832,M93839,M93841<br>M93842,M93849,M93851,M93852,M93859,M93861,M93862,M93869<br>M93871,M93872,M93879,M9388,M9389,M9390,M93911,M93912,M93919<br>M93921,M93922,M93929,M93931,M93932,M93939,M93941,M93942<br>M93949,M93951,M93952,M93959,M93961,M93962,M93969,M93971<br>M93972,M93979,M9398,M9399,M940,M941,M9420,M94211,M94212<br>M94219,M94221,M94222,M94229,M94231,M94232,M94239,M94241<br>M94242,M94249,M94251,M94252,M94259,M94261,M94262,M94269<br>M94271,M94272,M94279,M9428,M9429,M94351,M94352,M94359,M948X0<br>M948X1,M948X2,M948X3,M948X4,M948X5,M948X6,M948X7,M948X8<br>M948X9,M949,M960 | |
| Other specified chronic arthropathy | L4050,L4051,L4054,L4059,M1200,M12011,M12012,M12019,M12021<br>M12022,M12029,M12031,M12032,M12039,M12041,M12042,M12049<br>M12051,M12052,M12059,M12061,M12062,M12069,M12071,M12072<br>M12079,M1208,M1209 | 6960,7144 |
| Other specified complications in pregnancy | O1200,O1201,O1202,O1203,O1210,O1211,O1212,O1213,O1220,O1221<br>O1222,O1223,O210,O211,O212,O218,O219,O2200,O2201,O2202,O2203<br>O2210,O2211,O2212,O2213,O2220,O2221,O2222,O2223,O2230,O2231<br>O2232,O2233,O2240,O2241,O2242,O2243,O2250,O2251,O2252,O2253<br>O228X1,O228X2,O228X3,O228X9,O2290,O2291,O2292,O2293,O2300 | 64303,64313,64323,64383,64393,64610,<br>64611,64612,64613,64614<br>64620,64621,64622,64623,64640,64641,<br>64642,64643,64660,64661<br>64662,64663,64670,64671,64673,64680,<br>64681,64682,64683,64690<br>64693,64700,64701,64703,64710,64711,<br>64713,64720,64721,64723<br>64730,64731,64733,64740,64760,64761,<br>64763,64780,64781,64783 |

Continued on next page

**Table S9 (continued).**

| Diagnosis | ICD-10 | ICD-9 |
|---|---|---|
| | O2301,O2302,O2303,O2310,O2311,O2312,O2313,O2320,O2321,O2322 | 64790,64791,64793,64820,64821,64823, 64830,64831,64833,64840 |
| | O2323,O2330,O2331,O2332,O2333,O2340,O2341,O2342,O2343,O23511 | 64841,64843,64890,64891,64893,64900, 64901,64903,64910,64911 |
| | O23512,O23513,O23519,O23521,O23522,O23523,O23529,O23591 | 64913,64920,64921,64923,64930,64931, 64933,64960,64961,64962 |
| | O23592,O23593,O23599,O2390,O2391,O2392,O2393,O2510,O2511 | 64963,64964,65641,65643,66920,66921, 66922,66923,66924,67100 |
| | O2512,O2513,O2600,O2601,O2602,O2603,O2610,O2611,O2612,O2613 | 67101,67103,67110,67111,67112,67113, 67114,67120,67121,67123 |
| | O2640,O2641,O2642,O2643,O2650,O2651,O2652,O2653,O26611,O26612 | 67130,67131,67133,67150,67151,67153, 67180,67181,67183,67190 |
| | O26613,O26619,O26711,O26712,O26713,O26719,O26811,O26812 | 67191,67193,67300,67301,67303,67310, 67311,67313,67320,67321 |
| | O26813,O26819,O26821,O26822,O26823,O26829,O26831,O26832 | 67323,67330,67331,67333,67380,67381, 67382,67383,67400,67401 |
| | O26833,O26839,O26841,O26842,O26843,O26849,O2686,O26891,O26892 | 67403,67452,67454,67500,67501,67503, 67510,67511,67513,67520 |
| | O26893,O26899,O2690,O2691,O2692,O2693,O280,O281,O282,O283 | 67521,67523,67600,67601,67603,67610, 67611,67613,677,67903 |
| | O284,O285,O288,O289,O364XX0,O364XX1,O364XX2,O364XX3,O364XX4 O364XX5,O364XX9,O88011,O88012,O88013,O88019,O88111,O88112 O88113,O88119,O88211,O88212,O88213,O88219,O88311,O88312 O88313,O88319,O88811,O88812,O88813,O88819,O903,O91011,O91012 O91013,O91019,O91111,O91112,O91113,O91119,O91211,O91212 O91213,O91219,O92011,O92012,O92013,O92019,O92111,O92112 O92113,O92119,O94,O98011,O98012,O98013,O98019,O98111,O98112 O98113,O98119,O98211,O98212,O98213,O98219,O98311,O98312 O98313,O98319,O98411,O98412,O98413,O98419,O98511,O98512 O98513,O98519,O98611,O98612,O98613,O98619,O98711,O98712 O98713,O98719,O98811,O98812,O98813,O98819,O98911,O98912 O98913,O98919,O99011,O99012,O99013,O99019,O99111,O99112 O99113,O99119,O99210,O99211,O99212,O99213,O99280,O99281 O99282,O99283,O99310,O99311,O99312,O99313,O99320,O99321 O99322,O99323,O99330,O99331,O99332,O99333,O99340,O99341 O99342,O99343,O99350,O99351,O99352,O99353,O99411,O99412 O99413,O99419,O99511,O99512,O99513,O99519,O99611,O99612 O99613,O99619,O99711,O99712,O99713,O99719,O99820,O99830 O99840,O99841,O99842,O99843,O9989,O99891,O9A111,O9A112,O9A113 O9A119,O9A211,O9A212,O9A213,O9A219,O9A311,O9A312,O9A313 O9A319,O9A411,O9A412,O9A413,O9A419,O9A511,O9A512,O9A513 O9A519 | 7965,V0251 |
| Other specified | A1809,L945,M5400,M5401,M5402,M5403,M5404,M5405,M5406,M5407 | 01590,6962,7236,7248,7260,72610,72619, 7262,72631,72632,72633 |
| connective tissue | M5408,M5409,M6020,M60211,M60212,M60219,M60221,M60222,M60229 | 72639,7264,7265,72660,72662,72665, 72670,72672,72673,7272,7273 |
| disease | M60231,M60232,M60239,M60241,M60242,M60249,M60251,M60252 | 72740,72749,72782,72789,7279,7286, 72871,72879,72882,72886 |
| | M60259,M60261,M60262,M60269,M60271,M60272,M60279,M6028,M7010 | 72889,7290,7291,7292,72930,72931,7294, 7296,72971,72972,72973 |
| | M7011,M7012,M7020,M7021,M7022,M7030,M7031,M7032,M7040,M7041 M7042,M7050,M7051,M7052,M7060,M7061,M7062,M7070,M7071,M7072 M7080,M70811,M70812,M70819,M70821,M70822,M70829,M70831,M70832 M70839,M70841,M70842,M70849,M70851,M70852,M70859,M70861 M70862,M70869,M70871,M70872,M70879,M7088,M7089,M7090,M70911 M70912,M70919,M70921,M70922,M70929,M70931,M70932,M70939 M70941,M70942,M70949,M70951,M70952,M70959,M70961,M70962 M70969,M70971,M70972,M70979,M7098,M7099,M7100,M71011,M71012 M71019,M71021,M71022,M71029,M71031,M71032,M71039,M71041 M71042,M71049,M71051,M71052,M71059,M71061,M71062,M71069 M71071,M71072,M71079,M7108,M7109,M7110,M71111,M71112,M71119 M71121,M71122,M71129,M71131,M71132,M71139,M71141,M71142 M71149,M71151,M71152,M71159,M71161,M71162,M71169,M71171 M71172,M71179,M7118,M7119,M7130,M71311,M71312,M71319,M71321 M71322,M71329,M71331,M71332,M71339,M71341,M71342,M71349 M71351,M71352,M71359,M71371,M71372,M71379,M7138,M7139,M7140 M71421,M71422,M71429,M71431,M71432,M71439,M71441,M71442 M71449,M71451,M71452,M71459,M71461,M71462,M71469,M71471 M71472,M71479,M7148,M7149,M7150,M71521,M71522,M71529,M71531 M71532,M71539,M71541,M71542,M71549,M71551,M71552,M71559 M71561,M71562,M71569,M71571,M71572,M71579,M7158,M7180,M71811 M71812,M71819,M71821,M71822,M71829,M71831,M71832,M71839 M71841,M71842,M71849,M71851,M71852,M71859,M71861,M71862 M71869,M71871,M71872,M71879,M7188,M7189,M719,M720,M721,M722 M724,M726,M728,M729,M7500,M7501,M7502,M7540,M7541,M7542,M7550 M7551,M7552,M7580,M7581,M7582,M7590,M7591,M7592,M7620,M7621 M7622,M7630,M7631,M7632,M7640,M7641,M7642,M76811,M76812 M76819,M7700,M7701,M7702,M7710,M7711,M7712,M7720,M7721,M7722 M7730,M7731,M7732,M7740,M7741,M7742,M790,M792,M793,M794,M795 M797,M7981,M7989,M799,M79A11,M79A12,M79A19,M79A21,M79A22 M79A29,M79A3,M79A9 | 72979,72981,72990,72992,72999 |
| Other specified | I8500,I8501,I8510,I8511,I860,I861,I862,I863,I864,I868,I878 | 2891,2892,2893,4560,4561,45620,45621, 4563,4564,4565,4566,4568 |
| diseases of veins and | I879,I880,I881,I888,I889,I890,I891,I898,I899,O2210,O2211 | 4571,4572,4578,4579,45981,45989,67110, 67111,67112,67113,67114 |

Continued on next page

Table S9 (continued).

| Diagnosis | ICD-10 | ICD-9 |
|---|---|---|
| lymphatics | O2212,O2213 | |
| Other specified | Z322,Z323,Z710,Z713,Z7141,Z7142,Z7151,Z7152,Z716,Z717,Z7181 | V200,V201,V4612,V6519,V653,V6540, V6542,V6544,V6549,V658,V659 |
| encounters and counseling | Z7182,Z7183,Z7184,Z7185,Z7187,Z7188,Z7189,Z719,Z760,Z761,Z762 Z7689,Z9912 | V681 |
| Other specified eye | A1850,A1854,A1859,A211,A5001,A5030,A5039,A5143,A5271,A5430 | 01730,0213,05320,05322,05329,05440, 05441,05444,05449,0760 |
| disorders | A5432,A5439,A710,A711,A719,B0050,B0051,B0059,B0230,B0232 | 0761,0769,0900,0905,09150,09151,09152, 0950,09841,09842,09849 |
| | B0239,B5800,B5809,B691,B7300,B7301,B7309,H2100,H2101,H2102 | 1231,1253,1301,1307,36013,36020,36021, 36023,36024,36029,36030 |
| | H2103,H211X1,H211X2,H211X3,H211X9,H21211,H21212,H21213,H21219 | 36031,36032,36033,36034,36040,36041, 36043,36044,36050,36051 |
| | H21221,H21222,H21223,H21229,H21231,H21232,H21233,H21239 | 36052,36053,36054,36055,36059,36060, 36061,36062,36063,36064 |
| | H21241,H21242,H21243,H21249,H21251,H21252,H21253,H21259 | 36065,36069,36411,36441,36442,36451, 36452,36453,36454,36455 |
| | H21261,H21262,H21263,H21269,H21271,H21272,H21273,H21279,H2129 | 36456,36457,36459,36460,36461,36462, 36463,36464,36470,36471 |
| | H21301,H21302,H21303,H21309,H21311,H21312,H21313,H21319 | 36472,36473,36474,36475,36476,36477, 36481,36482,36489,3649 |
| | H21321,H21322,H21323,H21329,H21331,H21332,H21333,H21339 | 37881,37882,37883,37884,37940,37941, 37942,37943,37945,37946 |
| | H21341,H21342,H21343,H21349,H21351,H21352,H21353,H21359,H2140 | 37949,37950,37951,37952,37953,37955, 37956,37957,37959,3798 |
| | H2141,H2142,H2143,H21501,H21502,H21503,H21509,H21511,H21512 H21513,H21519,H21521,H21522,H21523,H21529,H21531,H21532 H21533,H21539,H21541,H21542,H21543,H21549,H21551,H21552 H21553,H21559,H21561,H21562,H21563,H21569,H2181,H2182,H2189 H219,H22,H4420,H4421,H4422,H4423,H442A1,H442A2,H442A3,H442A9 H442B1,H442B2,H442B3,H442B9,H442C1,H442C2,H442C3,H442C9 H442D1,H442D2,H442D3,H442D9,H442E1,H442E2,H442E3,H442E9,H4430 H44311,H44312,H44313,H44319,H44321,H44322,H44323,H44329 H44391,H44392,H44393,H44399,H4440,H44411,H44412,H44413,H44419 H44421,H44422,H44423,H44429,H44431,H44432,H44433,H44439 H44441,H44442,H44443,H44449,H4450,H44521,H44522,H44523,H44529 H44531,H44532,H44533,H44539,H44601,H44602,H44603,H44609 H44611,H44612,H44613,H44619,H44621,H44622,H44623,H44629 H44631,H44632,H44633,H44639,H44641,H44642,H44643,H44649 H44651,H44652,H44653,H44659,H44691,H44692,H44693,H44699 H44701,H44702,H44703,H44709,H44711,H44712,H44713,H44719 H44721,H44722,H44723,H44729,H44731,H44732,H44733,H44739 H44741,H44742,H44743,H44749,H44751,H44752,H44753,H44759 H44791,H44792,H44793,H44799,H44811,H44812,H44813,H44819,H510 H5111,H5112,H5500,H5501,H5502,H5503,H5504,H5509,H5581,H5582 H5589,H5700,H5701,H5702,H5703,H5704,H57051,H57052,H57053 H57059,H5709,H5710,H5711,H5712,H5713,H578,H5789,H579 | 37990,37991,V411 |
| Other specified | B373,B3731,B3732,N820,N821,N822,N823,N824,N825,N828,N829 | 1121,6185,6190,6191,6192,6198,6199, 6203,6204,6205,6206,6207 |
| female genital | N8331,N83311,N83312,N83319,N8332,N83321,N83322,N83329,N8333 | 6208,6209,6210,6211,6212,62130,62131, 62132,62133,62134,62135 |
| disorders | N83331,N83332,N83339,N834,N8340,N8341,N8342,N8351,N83511 | 6214,6215,6216,6217,6218,6219,6220, 62210,62211,62212,6222 |
| | N83512,N83519,N8352,N83521,N83522,N83529,N8353,N836,N837,N838 | 6223,6224,6225,6226,6227,6228,6229, 6230,6231,6232,6233,6234 |
| | N839,N840,N841,N842,N843,N848,N849,N8500,N8501,N8502,N852 | 6235,6236,6237,6238,6239,62401,62402, 62409,6241,6242,6243 |
| | N853,N854,N855,N856,N857,N858,N859,N85A,N86,N870,N871,N879 | 6246,6248,6249,6250,6251,6252,6254, 6255,62570,62571,62579 |
| | N880,N881,N882,N883,N884,N888,N889,N890,N891,N893,N894,N895 | 6258,6267,6268,6269,6273,6290,6291, 62920,62921,62922,62923 |
| | N896,N898,N899,N900,N901,N903,N904,N905,N906,N9060,N9061 N9069,N907,N90810,N90811,N90812,N90813,N90818,N9089,N909,N930 N931,N938,N939,N940,N941,N9410,N9411,N9412,N9419,N942,N943 N94810,N94818,N94819,N9489,N949,N952,N96,N992,N993 | 62929,62981,62989,6299 |
| Other specified | B653,E08620,E09620,E10620,E11620,E13620,L100,L101,L102,L103 | 0578,1203,24980,25080,25081,6829,68601, 69010,69011,69012 |
| inflammatory | L104,L105,L1081,L1089,L109,L110,L111,L118,L119,L120,L121,L122 | 69018,6918,69271,69276,69277,6930,6931, 6938,6939,6940,6941 |
| condition of skin | L1230,L1231,L1235,L128,L129,L130,L131,L138,L139,L14,L200 | 6942,6943,6944,6945,69460,69461,6948, 6949,6950,69510,69511 |
| | L2081,L2082,L2083,L2084,L2089,L209,L210,L211,L218,L219,L26 | 69512,69513,69514,69515,69519,6952, 6953,6954,69550,69551 |
| | L270,L271,L272,L278,L279,L280,L281,L282,L290,L291,L292,L293 | 69552,69553,69554,69555,69556,69557, 69558,69559,69589,6959 |
| | L298,L299,L304,L305,L400,L401,L402,L403,L404,L4050,L4051 | 6960,6961,6962,6963,6964,6965,6968, 6970,6971,6978,6980,6981 |
| | L4052,L4053,L4054,L4059,L408,L409,L410,L411,L413,L414,L415 | 6982,6983,6984,6988,6989,7011,7018, 7028,7048,70583,7060,7061 |
| | L418,L419,L42,L430,L431,L432,L433,L438,L439,L440,L441,L442 | 7063,7080,7081,7082,7083,7084,7085, 7088,7089,7091,7098 |
| | L443,L444,L448,L449,L45,L490,L491,L492,L493,L494,L495,L496 L497,L498,L499,L500,L501,L502,L503,L504,L505,L506,L508,L509 | |

Continued on next page

Table S9 (continued).

| Diagnosis | ICD-10 | ICD-9 |
|---|---|---|
| | L510,L511,L512,L513,L518,L519,L52,L530,L531,L532,L533,L538 | |
| | L539,L54,L550,L551,L552,L559,L661,L700,L701,L702,L703,L704 | |
| | L705,L708,L709,L710,L711,L718,L719,L732,L738,L88,L920,L930 | |
| | L931,L932,L945,L950,L951,L958,L959,L981,L982,L983 | |
| Other specified | S058X1A,S058X2A,S058X9A,S09311A,S09312A,S09313A,S09319A | 8719,87262,87263,87264,87269,87272, 87273,87274,8738,8739,9580 |
| injury | S09391A,S09392A,S09399A,S098XXA,S1980XA,S1981XA,S1982XA | 9581,9582,9584,9585,9586,9587,9588, 95890,95891,95892,95893 |
| | S1983XA,S1984XA,S1985XA,S1989XA,S298XXA,S3981XA,S3982XA | 95899,95901,95909,95911,95912,95913, 95914,95919,9592,9593 |
| | S3983XA,S39840A,S39848A,S4980XA,S4981XA,S4982XA,S59801A | 9594,9595,9596,9597 |
| | S59802A,S59809A,S59811A,S59812A,S59819A,S6980XA,S6981XA | |
| | S6982XA,S79811A,S79812A,S79819A,S79821A,S79822A,S79829A | |
| | S8980XA,S8981XA,S8982XA,S99811A,S99812A,S99819A,S99821A | |
| | S99822A,S99829A,T790XXA,T791XXA,T792XXA,T794XXA,T795XXA | |
| | T796XXA,T797XXA,T798XXA,T799XXA,T79A0XA,T79A11A,T79A12A | |
| | T79A19A,T79A21A,T79A22A,T79A29A,T79A3XA,T79A9XA | |
| Other specified | S058X1D,S058X2D,S058X9D,S09311D,S09312D,S09313D,S09319D | V5889 |
| injury, subsequent | S09391D,S09392D,S09399D,S098XXD,S1980XD,S1981XD,S1982XD | |
| encounter | S1983XD,S1984XD,S1985XD,S1989XD,S298XXD,S3981XD,S3982XD | |
| | S3983XD,S39840D,S39848D,S4980XD,S4981XD,S4982XD,S59801D | |
| | S59802D,S59809D,S59811D,S59812D,S59819D,S6980XD,S6981XD | |
| | S6982XD,S79811D,S79812D,S79819D,S79821D,S79822D,S79829D | |
| | S8980XD,S8981XD,S8982XD,S99811D,S99812D,S99819D,S99821D | |
| | S99822D,S99829D,T790XXD,T791XXD,T792XXD,T794XXD,T795XXD | |
| | T796XXD,T797XXD,T798XXD,T799XXD,T79A0XD,T79A11D,T79A12D | |
| | T79A19D,T79A21D,T79A22D,T79A29D,T79A3XD,T79A9XD | |
| Other specified joint | E08610,E08618,E09610,E09618,E10610,E10618,E11610,E11618 | 24960,24980,25060,25061,25080,25081, 7131,7132,7135,7136,7138 |
| disorders | E13610,E13618,M0200,M02011,M02012,M02019,M02021,M02022,M02029 | 71600,71601,71602,71603,71604,71605, 71606,71607,71608,71609 |
| | M02031,M02032,M02039,M02041,M02042,M02049,M02051,M02052 | 71620,71621,71622,71623,71624,71625, 71626,71627,71628,71629 |
| | M02059,M02061,M02062,M02069,M02071,M02072,M02079,M0208,M0209 | 71640,71641,71642,71643,71644,71645, 71646,71647,71648,71649 |
| | M0220,M02211,M02212,M02219,M02221,M02222,M02229,M02231,M02232 | 71650,71660,71661,71662,71663,71664, 71665,71666,71667,71668 |
| | M02239,M02241,M02242,M02249,M02251,M02252,M02259,M02261 | 71680,71681,71682,71683,71684,71685, 71686,71687,71688,71689 |
| | M02262,M02269,M02271,M02272,M02279,M0228,M0229,M1210,M12111 | 71690,7171,7172,7173,71740,71742,71743, 71749,7175,7176,7177 |
| | M12112,M12119,M12121,M12122,M12129,M12131,M12132,M12139 | 71785,71789,7179,71800,71801,71802, 71803,71804,71805,71807 |
| | M12141,M12142,M12149,M12151,M12152,M12159,M12161,M12162 | 71810,71811,71812,71813,71814,71815, 71817,71818,71820,71821 |
| | M12169,M12171,M12172,M12179,M1218,M1219,M1230,M12311,M12312 | 71822,71823,71824,71825,71826,71827, 71828,71829,71830,71831 |
| | M12319,M12321,M12322,M12329,M12331,M12332,M12339,M12341 | 71832,71833,71834,71835,71836,71837, 71850,71851,71852,71853 |
| | M12342,M12349,M12351,M12352,M12359,M12361,M12362,M12369 | 71854,71855,71856,71857,71865,71871, 71872,71873,71874,71875 |
| | M12371,M12372,M12379,M1238,M1239,M1240,M12411,M12412,M12419 | 71877,71880,71881,71882,71883,71884, 71885,71886,71887,71888 |
| | M12421,M12422,M12429,M12431,M12432,M12439,M12441,M12442 | 71890,71900,71901,71902,71903,71904, 71905,71906,71907,71908 |
| | M12449,M12451,M12452,M12459,M12461,M12462,M12469,M12471 | 71910,71911,71912,71913,71914,71915, 71916,71917,71918,71930 |
| | M12472,M12479,M1248,M1249,M1280,M12811,M12812,M12819,M12821 | 71931,71932,71933,71934,71935,71936, 71937,71938,71939,71980 |
| | M12822,M12829,M12831,M12832,M12839,M12841,M12842,M12849 | 71981,71982,71983,71984,71985,71986, 71987,71988,71990,7218 |
| | M12851,M12852,M12859,M12861,M12862,M12869,M12871,M12872 | 7262,72630,7264,7265,72660,72670,72691, 7284,73070,73071,73072 |
| | M12879,M1288,M1289,M129,M130,M1310,M13111,M13112,M13119 | 73073,73074,73075,73076,73077,73078, 73079,99952 |
| | M13121,M13122,M13129,M13131,M13132,M13139,M13141,M13142 | |
| | M13149,M13151,M13152,M13159,M13161,M13162,M13169,M13171 | |
| | M13172,M13179,M1380,M13811,M13812,M13819,M13821,M13822,M13829 | |
| | M13831,M13832,M13839,M13841,M13842,M13849,M13851,M13852 | |
| | M13859,M13861,M13862,M13869,M13871,M13872,M13879,M1388,M1389 | |
| | M1480,M14811,M14812,M14819,M14821,M14822,M14829,M14831,M14832 | |
| | M14839,M14841,M14842,M14849,M14851,M14852,M14859,M14861 | |
| | M14862,M14869,M14871,M14872,M14879,M1488,M1489,M2200,M2201 | |
| | M2202,M2210,M2211,M2212,M222X1,M222X2,M222X9,M223X1,M223X2 | |
| | M223X9,M2240,M2241,M2242,M228X1,M228X2,M228X9,M2290,M2291 | |
| | M2292,M23000,M23001,M23002,M23003,M23004,M23005,M23006,M23007 | |
| | M23009,M23011,M23012,M23019,M23021,M23022,M23029,M23031 | |
| | M23032,M23039,M23041,M23042,M23049,M23051,M23052,M23059 | |
| | M23061,M23062,M23069,M23200,M23201,M23202,M23203,M23204 | |
| | M23205,M23206,M23207,M23209,M23211,M23212,M23219,M23221 | |
| | M23222,M23229,M23231,M23232,M23239,M23241,M23242,M23249 | |
| | M23251,M23252,M23259,M23261,M23262,M23269,M23300,M23301 | |
| | M23302,M23303,M23304,M23305,M23306,M23307,M23309,M23311 | |
| | M23312,M23319,M23321,M23322,M23329,M23331,M23332,M23339 | |

Continued on next page

Table S9 (continued).

| Diagnosis | ICD-10 | ICD-9 |
|---|---|---|
| | M23341,M23342,M23349,M23351,M23352,M23359,M23361,M23362 | |
| | M23369,M2340,M2341,M2342,M2350,M2351,M2352,M23601,M23602 | |
| | M23609,M23611,M23612,M23619,M23621,M23622,M23629,M23631 | |
| | M23632,M23639,M23641,M23642,M23649,M23671,M23672,M23679 | |
| | M238X1,M238X2,M238X9,M2390,M2391,M2392,M2400,M24011,M24012 | |
| | M24019,M24021,M24022,M24029,M24031,M24032,M24039,M24041 | |
| | M24042,M24049,M24051,M24052,M24059,M24071,M24072,M24073 | |
| | M24074,M24075,M24076,M2408,M2410,M24111,M24112,M24119,M24121 | |
| | M24122,M24129,M24131,M24132,M24139,M24141,M24142,M24149 | |
| | M24151,M24152,M24159,M24171,M24172,M24173,M24174,M24175 | |
| | M24176,M2419,M2420,M24211,M24212,M24219,M24221,M24222,M24229 | |
| | M24231,M24232,M24239,M24241,M24242,M24249,M24251,M24252 | |
| | M24259,M24271,M24272,M24273,M24274,M24275,M24276,M2428,M2429 | |
| | M2430,M24311,M24312,M24319,M24321,M24322,M24329,M24331,M24332 | |
| | M24339,M24341,M24342,M24349,M24351,M24352,M24359,M24361 | |
| | M24362,M24369,M24371,M24372,M24373,M24374,M24375,M24376,M2439 | |
| | M2440,M24411,M24412,M24419,M24421,M24422,M24429,M24431,M24432 | |
| | M24439,M24441,M24442,M24443,M24444,M24445,M24446,M24451 | |
| | M24452,M24459,M24461,M24462,M24469,M24471,M24472,M24473 | |
| | M24474,M24475,M24476,M24477,M24478,M24479,M2449,M2460,M24611 | |
| | M24612,M24619,M24621,M24622,M24629,M24631,M24632,M24639 | |
| | M24641,M24642,M24649,M24651,M24652,M24659,M24661,M24662 | |
| | M24669,M24671,M24672,M24673,M24674,M24675,M24676,M2469,M247 | |
| | M2480,M24811,M24812,M24819,M24821,M24822,M24829,M24831,M24832 | |
| | M24839,M24841,M24842,M24849,M24851,M24852,M24859,M24871 | |
| | M24872,M24873,M24874,M24875,M24876,M2489,M249,M2500,M25011 | |
| | M25012,M25019,M25021,M25022,M25029,M25031,M25032,M25039 | |
| | M25041,M25042,M25049,M25051,M25052,M25059,M25061,M25062 | |
| | M25069,M25071,M25072,M25073,M25074,M25075,M25076,M2508,M2510 | |
| | M25111,M25112,M25119,M25121,M25122,M25129,M25131,M25132 | |
| | M25139,M25141,M25142,M25149,M25151,M25152,M25159,M25161 | |
| | M25162,M25169,M25171,M25172,M25173,M25174,M25175,M25176,M2518 | |
| | M2520,M25211,M25212,M25219,M25221,M25222,M25229,M25231,M25232 | |
| | M25239,M25241,M25242,M25249,M25251,M25252,M25259,M25261 | |
| | M25262,M25269,M25271,M25272,M25279,M2528,M2530,M25311,M25312 | |
| | M25319,M25321,M25322,M25329,M25331,M25332,M25339,M25341 | |
| | M25342,M25349,M25351,M25352,M25359,M25361,M25362,M25369 | |
| | M25371,M25372,M25373,M25374,M25375,M25376,M2539,M2540,M25411 | |
| | M25412,M25419,M25421,M25422,M25429,M25431,M25432,M25439 | |
| | M25441,M25442,M25449,M25451,M25452,M25459,M25461,M25462 | |
| | M25469,M25471,M25472,M25473,M25474,M25475,M25476,M2548,M2570 | |
| | M25711,M25712,M25719,M25721,M25722,M25729,M25731,M25732 | |
| | M25739,M25741,M25742,M25749,M25751,M25752,M25759,M25761 | |
| | M25762,M25769,M25771,M25772,M25773,M25774,M25775,M25776,M2578 | |
| | M2580,M25811,M25812,M25819,M25821,M25822,M25829,M25831,M25832 | |
| | M25839,M25841,M25842,M25849,M25851,M25852,M25859,M25861 | |
| | M25862,M25869,M25871,M25872,M25879,M259,M3505,M361,M362,M363 | |
| | M364,M8960,M89611,M89612,M89619,M89621,M89622,M89629,M89631 | |
| | M89632,M89639,M89641,M89642,M89649,M89651,M89652,M89659 | |
| | M89661,M89662,M89669,M89671,M89672,M89679,M8968,M8969 | |
| Other specified male | N420,N421,N423,N4230,N4231,N4232,N4239,N4281,N4282,N4283 | 6003,6020,6021,6022,6023,6028,6029,<br>6030,6031,6038,6039,605 |
| genital disorders | N4289,N429,N430,N431,N432,N433,N4340,N4341,N4342,N4400,N4401 | 6070,6073,60781,60782,60783,60785,<br>60789,6079,6081,60820,60821 |
| | N4402,N4403,N4404,N441,N442,N448,N470,N471,N472,N473,N474 | 60822,60823,60824,6083,60881,60883,<br>60884,60885,60886,60887 |
| | N475,N477,N478,N480,N4830,N4831,N4832,N4833,N4839,N485,N486 | 60889,6089 |
| | N4881,N4882,N4883,N4889,N489,N500,N501,N503,N508,N50811 | |
| | N50812,N50819,N5082,N5089,N509,N51,N5311,N5312,N5313,N5314 | |
| | N5319,N538,N539 | |
| Other specified | Z1401,Z1402,Z141,Z148,Z1801,Z1809,Z1810,Z1811,Z1812,Z182 | NoDx,V074,V0751,V0752,V0759,V140,<br>V141,V142,V143,V144,V145 |
| status | Z1831,Z1832,Z1833,Z1839,Z1881,Z1883,Z1889,Z189,Z52000,Z52001 | V146,V147,V148,V149,V1501,V1502,<br>V1503,V1504,V1505,V1506,V1507 |
| | Z52008,Z52010,Z52011,Z52018,Z52090,Z52091,Z52098,Z5210,Z5211 | V1508,V1509,V1581,V1584,V1586,<br>V1589,V4031,V440,V441,V442,V443 |
| | Z5219,Z5220,Z5221,Z5229,Z523,Z524,Z525,Z526,Z52810,Z52811 | V444,V4450,V4451,V4452,V4459,V446,<br>V447,V448,V449,V4511,V4512 |
| | Z52812,Z52813,Z52819,Z5289,Z529,Z5301,Z5309,Z531,Z5320,Z5321 | V460,V4611,V462,V463,V468,V469,<br>V4981,V4983,V4984,V4986,V4987 |
| | Z5329,Z5331,Z5332,Z5333,Z5339,Z538,Z539,Z66,Z6710,Z6711,Z6720 | V4989,V5861,V5862,V5863,V5864,<br>V5865,V5866,V5867,V5868,V5869 |
| | Z6721,Z6730,Z6731,Z6740,Z6741,Z6790,Z6791,Z681,Z6820,Z6821 | V5901,V5902,V5909,V591,V592,V593,<br>V594,V595,V596,V5970,V5971 |
| | Z6822,Z6823,Z6824,Z6825,Z6826,Z6827,Z6828,Z6829,Z6851,Z6852 | V5972,V5973,V5974,V598,V599,V604,<br>V605,V6089,V609,V626,V631 |
| | Z6853,Z711,Z712,Z7401,Z7409,Z741,Z742,Z743,Z748,Z749,Z750 | V632,V638,V639,V6406,V641,V642,<br>V643,V650,V6511,V652,V655,V658 |
| | Z751,Z752,Z753,Z754,Z755,Z758,Z759,Z763,Z764,Z765,Z7681,Z7682 | V8301,V8302,V8381,V8389,V850,V851,<br>V8521,V8522,V8523,V8524 |
| | Z77010,Z77011,Z77012,Z77018,Z77020,Z77021,Z77028,Z77090 | V8525,V8551,V8552,V8553,V8701,<br>V8702,V8709,V8711,V8712,V8719 |
| | Z77098,Z77110,Z77111,Z77112,Z77118,Z77120,Z77121,Z77122 | V872,V8731,V8732,V8739,V9001,V9009,<br>V9010,V9011,V9012,V902 |

Continued on next page

**Table S9 (continued).**

| Diagnosis | ICD-10 | ICD-9 |
|---|---|---|
| | Z77123,Z77128,Z7721,Z7722,Z7729,Z779,Z780,Z781,Z789,Z7901 Z7902,Z791,Z792,Z793,Z794,Z7951,Z7952,Z7960,Z7961,Z79620 Z79621,Z79622,Z79623,Z79624,Z79630,Z79631,Z79632,Z79633 Z79634,Z7964,Z7969,Z79810,Z79811,Z79818,Z7982,Z7983,Z7984 Z7985,Z79890,Z79891,Z79899,Z880,Z881,Z882,Z883,Z884,Z885,Z886 Z887,Z888,Z889,Z91010,Z91011,Z91012,Z91013,Z91014,Z91018 Z9102,Z91030,Z91038,Z91040,Z91041,Z91048,Z9109,Z9111,Z91110 Z91118,Z91119,Z91120,Z91128,Z91130,Z91138,Z9114,Z9115,Z9119 Z91190,Z91198,Z91199,Z9183,Z91841,Z91842,Z91843,Z91849,Z9189 Z91A10,Z91A18,Z91A20,Z91A28,Z91A3,Z91A4,Z91A5,Z91A9,Z930,Z931 Z932,Z933,Z934,Z9350,Z9351,Z9352,Z9359,Z936,Z938,Z939,Z990 Z9911,Z992,Z993,Z9981,Z9989 | V9031,V9032,V9033,V9039,V9081, V9083,V9089,V909 |
| Other specified substance-related disorders | F1910,F19120,F19121,F19122,F19129,F19130,F19131,F19132,F19139 F1914,F19150,F19151,F19159,F1916,F1917,F19180,F19181,F19182 F19188,F1919,F1920,F19220,F19221,F19222,F19229,F19230,F19231 F19232,F19239,F1924,F19250,F19251,F19259,F1926,F1927,F19280 F19281,F19282,F19288,F1929,F1990,F1991,F19920,F19921,F19922 F19929,F19930,F19931,F19932,F19939,F1994,F19950,F19951,F19959 F1996,F1997,F19980,F19981,F19982,F19988,F1999,O355XX0,O355XX1 O355XX2,O355XX3,O355XX4,O355XX5,O355XX9,O99320,O99321,O99322 O99323,O99324,O99325 | 2920,29211,29212,2922,29281,29282, 29283,29284,29285,29289 2929,30460,30461,30462,30580,30581, 30582,30583,64830,64831 64832,64833,64834,65551,65553 |
| Other specified upper respiratory infections | A155,A360,A361,A362,A5003,A5273,A545,A564,B002,B085,J00,J020 J028,J029,J040,J0410,J0411,J042,J0430,J0431,J050,J0510,J0511 J060,J069,J390,J391 | 01130,01220,01230,0320,0321,0323,0340, 0542,0740,0900,0958 0986,09951,460,462,46400,46401,46410, 46411,46420,46421,46430 46431,4644,46450,46451,4650,4658,4659, 47821,47822,47824 |
| Other unspecified injuries, subsequent encounter | S0590XD,S0591XD,S0592XD,S09301D,S09302D,S09309D,S0990XD S0991XD,S0992XD,S0993XD,S199XXD,S299XXD,S3991XD,S3992XD S3993XD,S3994XD,S4990XD,S4991XD,S4992XD,S59901D,S59902D S59909D,S59911D,S59912D,S59919D,S6990XD,S6991XD,S6992XD S79911D,S79912D,S79919D,S79921D,S79922D,S79929D,S8990XD S8991XD,S8992XD,S99911D,S99912D,S99919D,S99921D,S99922D S99929D,T07XXXD,T148XXD,T1490XD,T1491XD | V5889 |
| Other unspecified injury | O9A211,O9A212,O9A213,O9A219,O9A22,O9A23,R4588,S0590XA,S0591XA S0592XA,S09301A,S09302A,S09309A,S0990XA,S0991XA,S0992XA S0993XA,S199XXA,S299XXA,S3991XA,S3992XA,S3993XA,S3994XA S4990XA,S4991XA,S4992XA,S59901A,S59902A,S59909A,S59911A S59912A,S59919A,S6990XA,S6991XA,S6992XA,S79911A,S79912A S79919A,S79921A,S79922A,S79929A,S8990XA,S8991XA,S8992XA S99911A,S99912A,S99919A,S99921A,S99922A,S99929A,T07,T07XXXA T148,T148XXA,T1490,T1490XA,T1491,T1491XA | 64890,64891,64893,64894,8719,87262, 87263,87264,87272,87273 87274,8728,8738,8739,9189,9219,95901, 95909,95911,95912,95914 95919,9592,9593,9594,9595,9596,9597, 9598,9599,E9589 |
| Otitis media | A380,B053,H6500,H6501,H6502,H6503,H6504,H6505,H6506,H6507 H65111,H65112,H65113,H65114,H65115,H65116,H65117,H65119 H65191,H65192,H65193,H65194,H65195,H65196,H65197,H65199,H6520 H6521,H6522,H6523,H6530,H6531,H6532,H6533,H65411,H65412 H65413,H65419,H65491,H65492,H65493,H65499,H6590,H6591,H6592 H6593,H66001,H66002,H66003,H66004,H66005,H66006,H66007,H66009 H66011,H66012,H66013,H66014,H66015,H66016,H66017,H66019,H6610 H6611,H6612,H6613,H6620,H6621,H6622,H6623,H663X1,H663X2 H663X3,H663X9,H6640,H6641,H6642,H6643,H6690,H6691,H6692,H6693 H671,H672,H673,H679,J1083,J1183 | 0341,0552,38100,38101,38102,38103, 38104,38105,38106,38110 38119,38120,38129,3813,3814,38200, 38201,38202,3821,3822,3823 3824,3829,4878 |
| Pancreatic disorders (excluding diabetes) | B252,B263,K850,K8500,K8501,K8502,K851,K8510,K8511,K8512,K852 K8520,K8521,K8522,K853,K8530,K8531,K8532,K858,K8580,K8581 K8582,K859,K8590,K8591,K8592,K860,K861,K862,K863,K868,K8681 K8689,K869,K903 | 0723,0785,5770,5771,5772,5778,5779, 5794 |
| Paralysis (other than cerebral palsy) | G041,G8100,G8101,G8102,G8103,G8104,G8110,G8111,G8112,G8113 G8114,G8190,G8191,G8192,G8193,G8194,G8220,G8221,G8222,G8250 G8251,G8252,G8253,G8254,G830,G8310,G8311,G8312,G8313,G8314 G8320,G8321,G8322,G8323,G8324,G8330,G8331,G8332,G8333,G8334 G834,G835,G8381,G8382,G8383,G8384,G8389,G839 | 34200,34201,34202,34210,34211,34212, 34280,34281,34282,34290 34291,34292,34400,34401,34402,34403, 34404,34409,3441,3442 34430,34431,34432,34440,34441,34442, 3445,34460,34461,34481 34489,3449 |
| Parasitic, other specified and unspecified | A060,A061,A062,A063,A064,A065,A066,A067,A0681,A0682,A0689 A069,A070,A071,A072,A073,A074,A078,A079,A09,A5900,A5901,A5902 A5903,A5909,A598,A599,A65,A660,A661,A662,A663,A664,A665,A666 | 0060,0061,0062,0063,0064,0065,0066, 0068,0069,0070,0071,0072 0073,0074,0075,0078,0079,0090,080,0810, 0811,0812,0819,0820 0821,0822,0823,08240,08241,08249,0828, 0831,0832,0838,0839 |

Continued on next page

Table S9 (continued).

| Diagnosis | ICD-10 | ICD-9 |
|---|---|---|
| infections | A667,A668,A669,A670,A671,A672,A673,A679,A680,A681,A689,A690 | 0840,0841,0842,0843,0844,0845,0846,<br>0848,0849,0850,0851,0852 |
| | A691,A6920,A6921,A6922,A6923,A6929,A698,A699,A750,A751,A752 | 0853,0854,0855,0859,0860,0861,0862,<br>0863,0864,0865,0869,0870 |
| | A753,A759,A770,A771,A772,A773,A7740,A7741,A7749,A778,A779 | 0871,0879,08881,08882,0889,101,1020,<br>1021,1022,1023,1024,1025 |
| | A790,A791,A7981,A7982,A7989,A799,B500,B508,B509,B510,B518 | 1026,1027,1028,1029,1030,1031,1032,<br>1033,1039,1040,1048,1049 |
| | B519,B520,B528,B529,B530,B531,B538,B54,B550,B551,B552,B559 | 1200,1201,1202,1203,1208,1209,1210,<br>1211,1212,1213,1214,1215 |
| | B560,B561,B569,B570,B571,B572,B5730,B5731,B5732,B5739,B5740 | 1216,1218,1219,1220,1221,1222,1223,<br>1224,1225,1226,1227,1228 |
| | B5741,B5742,B5749,B575,B5800,B5801,B5809,B581,B582,B583,B5881 | 1229,1230,1231,1232,1233,1234,1235,<br>1236,1238,1239,124,1250 |
| | B5882,B5883,B5889,B589,B600,B6000,B6001,B6002,B6003,B6009 | 1251,1252,1253,1255,1256,1257,1259,<br>1260,1261,1262,1263,1268 |
| | B6010,B6011,B6012,B6013,B6019,B602,B608,B64,B650,B651,B652 | 1269,1270,1271,1272,1273,1274,1275,<br>1276,1277,1278,1279,1280 |
| | B653,B658,B659,B660,B661,B662,B663,B664,B665,B668,B669,B670 | 1281,1288,1289,129,1300,1301,1302,1303,<br>1304,1305,1307,1309 |
| | B671,B672,B6731,B6732,B6739,B674,B675,B6761,B6769,B677,B678 | 13100,13101,13102,13103,13109,1318,<br>1319,1320,1321,1322,1323 |
| | B6790,B6799,B680,B681,B689,B690,B691,B6981,B6989,B699,B700 | 1329,1330,1338,1340,1341,1342,1348,<br>1349,13621,13629,1365,1368 |
| | B701,B710,B711,B718,B719,B72,B7300,B7301,B7302,B7309,B731 | 1369,3211,3212,3218,3708,37215,4848,<br>5281,58181,64740,64741 |
| | B740,B741,B742,B743,B744,B748,B749,B75,B760,B761,B768,B769<br>B770,B7781,B7789,B779,B780,B781,B787,B789,B79,B80,B810,B811<br>B812,B813,B814,B818,B820,B829,B830,B831,B832,B833,B834,B838<br>B839,B850,B851,B852,B853,B854,B86,B870,B871,B872,B873,B874<br>B8781,B8782,B8789,B879,B880,B881,B882,B883,B888,B889,B89,B998<br>B999,G02,O98611,O98612,O98613,O98619,O9862,O9863,P238,P371<br>P373,P374,P378,P379 | 64742,64744,64781,64783,6868,7700,7712 |
| Parkinson‘s disease | G20 | 3320 |
| Pathological | M8000XA,M80011A,M80012A,M80019A,M80021A,M80022A,M80029A | 73310,73311,73312,73313,73314,73315,<br>73316,73319 |
| fracture, initial<br>encounter | M80031A,M80032A,M80039A,M80041A,M80042A,M80049A,M80051A<br>M80052A,M80059A,M80061A,M80062A,M80069A,M80071A,M80072A<br>M80079A,M8008XA,M800AXA,M8080XA,M80811A,M80812A,M80819A<br>M80821A,M80822A,M80829A,M80831A,M80832A,M80839A,M80841A<br>M80842A,M80849A,M80851A,M80852A,M80859A,M80861A,M80862A<br>M80869A,M80871A,M80872A,M80879A,M8088XA,M808AXA,M8440XA<br>M84411A,M84412A,M84419A,M84421A,M84422A,M84429A,M84431A<br>M84432A,M84433A,M84434A,M84439A,M84441A,M84442A,M84443A<br>M84444A,M84445A,M84446A,M84451A,M84452A,M84453A,M84454A<br>M84459A,M84461A,M84462A,M84463A,M84464A,M84469A,M84471A<br>M84472A,M84473A,M84474A,M84475A,M84476A,M84477A,M84478A<br>M84479A,M8448XA,M8450XA,M84511A,M84512A,M84519A,M84521A<br>M84522A,M84529A,M84531A,M84532A,M84533A,M84534A,M84539A<br>M84541A,M84542A,M84549A,M84550A,M84551A,M84552A,M84553A<br>M84559A,M84561A,M84562A,M84563A,M84564A,M84569A,M84571A<br>M84572A,M84573A,M84574A,M84575A,M84576A,M8458XA,M8460XA<br>M84611A,M84612A,M84619A,M84621A,M84622A,M84629A,M84631A<br>M84632A,M84633A,M84634A,M84639A,M84641A,M84642A,M84649A<br>M84650A,M84651A,M84652A,M84653A,M84659A,M84661A,M84662A<br>M84663A,M84664A,M84669A,M84671A,M84672A,M84673A,M84674A<br>M84675A,M84676A,M8468XA | |
| Pathological | M8000XD,M8000XG,M8000XK,M8000XP,M80011D,M80011G,M80011K | 73381,73382,V5420,V5421,V5422,V5423,<br>V5424,V5425,V5426,V5427 |
| fracture, subsequent<br>encounter | M80011P,M80012D,M80012G,M80012K,M80012P,M80019D,M80019G<br>M80019K,M80019P,M80021D,M80021G,M80021K,M80021P,M80022D<br>M80022G,M80022K,M80022P,M80029D,M80029G,M80029K,M80029P<br>M80031D,M80031G,M80031K,M80031P,M80032D,M80032G,M80032K<br>M80032P,M80039D,M80039G,M80039K,M80039P,M80041D,M80041G<br>M80041K,M80041P,M80042D,M80042G,M80042K,M80042P,M80049D<br>M80049G,M80049K,M80049P,M80051D,M80051G,M80051K,M80051P<br>M80052D,M80052G,M80052K,M80052P,M80059D,M80059G,M80059K<br>M80059P,M80061D,M80061G,M80061K,M80061P,M80062D,M80062G<br>M80062K,M80062P,M80069D,M80069G,M80069K,M80069P,M80071D<br>M80071G,M80071K,M80071P,M80072D,M80072G,M80072K,M80072P<br>M80079D,M80079G,M80079K,M80079P,M8008XD,M8008XG,M8008XK<br>M8008XP,M800AXD,M800AXG,M800AXK,M800AXP,M8080XD,M8080XG<br>M8080XK,M8080XP,M80811D,M80811G,M80811K,M80811P,M80812D<br>M80812G,M80812K,M80812P,M80819D,M80819G,M80819K,M80819P<br>M80821D,M80821G,M80821K,M80821P,M80822D,M80822G,M80822K<br>M80822P,M80829D,M80829G,M80829K,M80829P,M80831D,M80831G<br>M80831K,M80831P,M80832D,M80832G,M80832K,M80832P,M80839D<br>M80839G,M80839K,M80839P,M80841D,M80841G,M80841K,M80841P<br>M80842D,M80842G,M80842K,M80842P,M80849D,M80849G,M80849K<br>M80849P,M80851D,M80851G,M80851K,M80851P,M80852D,M80852G<br>M80852K,M80852P,M80859D,M80859G,M80859K,M80859P,M80861D<br>M80861G,M80861K,M80861P,M80862D,M80862G,M80862K,M80862P<br>M80869D,M80869G,M80869K,M80869P,M80871D,M80871G,M80871K | V5429 |

Continued on next page

Table S9 (continued).

| Diagnosis | ICD-10 | ICD-9 |
| --- | --- | --- |
| | M80871P,M80872D,M80872G,M80872K,M80872P,M80879D,M80879G | |
| | M80879K,M80879P,M8088XD,M8088XG,M8088XK,M8088XP,M808AXD | |
| | M808AXG,M808AXK,M808AXP,M8440XD,M8440XG,M8440XK,M8440XP | |
| | M84411D,M84411G,M84411K,M84411P,M84412D,M84412G,M84412K | |
| | M84412P,M84419D,M84419G,M84419K,M84419P,M84421D,M84421G | |
| | M84421K,M84421P,M84422D,M84422G,M84422K,M84422P,M84429D | |
| | M84429G,M84429K,M84429P,M84431D,M84431G,M84431K,M84431P | |
| | M84432D,M84432G,M84432K,M84432P,M84433D,M84433G,M84433K | |
| | M84433P,M84434D,M84434G,M84434K,M84434P,M84439D,M84439G | |
| | M84439K,M84439P,M84441D,M84441G,M84441K,M84441P,M84442D | |
| | M84442G,M84442K,M84442P,M84443D,M84443G,M84443K,M84443P | |
| | M84444D,M84444G,M84444K,M84444P,M84445D,M84445G,M84445K | |
| | M84445P,M84446D,M84446G,M84446K,M84446P,M84451D,M84451G | |
| | M84451K,M84451P,M84452D,M84452G,M84452K,M84452P,M84453D | |
| | M84453G,M84453K,M84453P,M84454D,M84454G,M84454K,M84454P | |
| | M84459D,M84459G,M84459K,M84459P,M84461D,M84461G,M84461K | |
| | M84461P,M84462D,M84462G,M84462K,M84462P,M84463D,M84463G | |
| | M84463K,M84463P,M84464D,M84464G,M84464K,M84464P,M84469D | |
| | M84469G,M84469K,M84469P,M84471D,M84471G,M84471K,M84471P | |
| | M84472D,M84472G,M84472K,M84472P,M84473D,M84473G,M84473K | |
| | M84473P,M84474D,M84474G,M84474K,M84474P,M84475D,M84475G | |
| | M84475K,M84475P,M84476D,M84476G,M84476K,M84476P,M84477D | |
| | M84477G,M84477K,M84477P,M84478D,M84478G,M84478K,M84478P | |
| | M84479D,M84479G,M84479K,M84479P,M8448XD,M8448XG,M8448XK | |
| | M8448XP,M8450XD,M8450XG,M8450XK,M8450XP,M84511D,M84511G | |
| | M84511K,M84511P,M84512D,M84512G,M84512K,M84512P,M84519D | |
| | M84519G,M84519K,M84519P,M84521D,M84521G,M84521K,M84521P | |
| | M84522D,M84522G,M84522K,M84522P,M84529D,M84529G,M84529K | |
| | M84529P,M84531D,M84531G,M84531K,M84531P,M84532D,M84532G | |
| | M84532K,M84532P,M84533D,M84533G,M84533K,M84533P,M84534D | |
| | M84534G,M84534K,M84534P,M84539D,M84539G,M84539K,M84539P | |
| | M84541D,M84541G,M84541K,M84541P,M84542D,M84542G,M84542K | |
| | M84542P,M84549D,M84549G,M84549K,M84549P,M84550D,M84550G | |
| | M84550K,M84550P,M84551D,M84551G,M84551K,M84551P,M84552D | |
| | M84552G,M84552K,M84552P,M84553D,M84553G,M84553K,M84553P | |
| | M84559D,M84559G,M84559K,M84559P,M84561D,M84561G,M84561K | |
| | M84561P,M84562D,M84562G,M84562K,M84562P,M84563D,M84563G | |
| | M84563K,M84563P,M84564D,M84564G,M84564K,M84564P,M84569D | |
| | M84569G,M84569K,M84569P,M84571D,M84571G,M84571K,M84571P | |
| | M84572D,M84572G,M84572K,M84572P,M84573D,M84573G,M84573K | |
| | M84573P,M84574D,M84574G,M84574K,M84574P,M84575D,M84575G | |
| | M84575K,M84575P,M84576D,M84576G,M84576K,M84576P,M8458XD | |
| | M8458XG,M8458XK,M8458XP,M8460XD,M8460XG,M8460XK,M8460XP | |
| | M84611D,M84611G,M84611K,M84611P,M84612D,M84612G,M84612K | |
| | M84612P,M84619D,M84619G,M84619K,M84619P,M84621D,M84621G | |
| | M84621K,M84621P,M84622D,M84622G,M84622K,M84622P,M84629D | |
| | M84629G,M84629K,M84629P,M84631D,M84631G,M84631K,M84631P | |
| | M84632D,M84632G,M84632K,M84632P,M84633D,M84633G,M84633K | |
| | M84633P,M84634D,M84634G,M84634K,M84634P,M84639D,M84639G | |
| | M84639K,M84639P,M84641D,M84641G,M84641K,M84641P,M84642D | |
| | M84642G,M84642K,M84642P,M84649D,M84649G,M84649K,M84649P | |
| | M84650D,M84650G,M84650K,M84650P,M84651D,M84651G,M84651K | |
| | M84651P,M84652D,M84652G,M84652K,M84652P,M84653D,M84653G | |
| | M84653K,M84653P,M84659D,M84659G,M84659K,M84659P,M84661D | |
| | M84661G,M84661K,M84661P,M84662D,M84662G,M84662K,M84662P | |
| | M84663D,M84663G,M84663K,M84663P,M84664D,M84664G,M84664K | |
| | M84664P,M84669D,M84669G,M84669K,M84669P,M84671D,M84671G | |
| | M84671K,M84671P,M84672D,M84672G,M84672K,M84672P,M84673D | |
| | M84673G,M84673K,M84673P,M84674D,M84674G,M84674K,M84674P | |
| | M84675D,M84675G,M84675K,M84675P,M84676D,M84676G,M84676K | |
| | M84676P,M8468XD,M8468XG,M8468XK,M8468XP | |
| Pathological, stress and atypical fractures, sequela | M4840XS,M4841XS,M4842XS,M4843XS,M4844XS,M4845XS,M4846XS | 9051,9052,9053,9054,9055 |
| | M4847XS,M4848XS,M4850XS,M4851XS,M4852XS,M4853XS,M4854XS | |
| | M4855XS,M4856XS,M4857XS,M4858XS,M8000XS,M80011S,M80012S | |
| | M80019S,M80021S,M80022S,M80029S,M80031S,M80032S,M80039S | |
| | M80041S,M80042S,M80049S,M80051S,M80052S,M80059S,M80061S | |
| | M80062S,M80069S,M80071S,M80072S,M80079S,M8008XS,M800AXS | |
| | M8080XS,M80811S,M80812S,M80819S,M80821S,M80822S,M80829S | |
| | M80831S,M80832S,M80839S,M80841S,M80842S,M80849S,M80851S | |
| | M80852S,M80859S,M80861S,M80862S,M80869S,M80871S,M80872S | |
| | M80879S,M8088XS,M808AXS,M8430XS,M84311S,M84312S,M84319S | |
| | M84321S,M84322S,M84329S,M84331S,M84332S,M84333S,M84334S | |
| | M84339S,M84341S,M84342S,M84343S,M84344S,M84345S,M84346S | |
| | M84350S,M84351S,M84352S,M84353S,M84359S,M84361S,M84362S | |
| | M84363S,M84364S,M84369S,M84371S,M84372S,M84373S,M84374S | |
| | M84375S,M84376S,M84377S,M84378S,M84379S,M8438XS,M8440XS | |
| | M84411S,M84412S,M84419S,M84421S,M84422S,M84429S,M84431S | |
| | M84432S,M84433S,M84434S,M84439S,M84441S,M84442S,M84443S | |
| | M84444S,M84445S,M84446S,M84451S,M84452S,M84453S,M84454S | |
| | M84459S,M84461S,M84462S,M84463S,M84464S,M84469S,M84471S | |
| | M84472S,M84473S,M84474S,M84475S,M84476S,M84477S,M84478S | |
| | M84479S,M8448XS,M8450XS,M84511S,M84512S,M84519S,M84521S | |
| | M84522S,M84529S,M84531S,M84532S,M84533S,M84534S,M84539S | |
| | M84541S,M84542S,M84549S,M84550S,M84551S,M84552S,M84553S | |

Continued on next page

Table S9 (continued).

| Diagnosis | ICD-10 | ICD-9 |
|---|---|---|
| | M84559S,M84561S,M84562S,M84563S,M84564S,M84569S,M84571S M84572S,M84573S,M84574S,M84575S,M84576S,M8458XS,M8460XS M84611S,M84612S,M84619S,M84621S,M84622S,M84629S,M84631S M84632S,M84633S,M84634S,M84639S,M84641S,M84642S,M84649S M84650S,M84651S,M84652S,M84653S,M84659S,M84661S,M84662S M84663S,M84664S,M84669S,M84671S,M84672S,M84673S,M84674S M84675S,M84676S,M8468XS,M84750S,M84751S,M84752S,M84753S M84754S,M84755S,M84756S,M84757S,M84758S,M84759S | |
| Pericarditis and pericardial disease | A3953,B3323,I010,I092,I300,I301,I308,I309,I310,I311,I312,I313 I3131,I3139,I314,I318,I319,I32,M3212 | 03641,07421,3910,393,4200,42090,42091, 42099,4230,4231,4232 4233,4238,4239,7100 |
| Perinatal infections | A33,A4851,A5001,A5002,A5003,A5004,A5005,A5006,A5007,A5008 A5009,A501,A502,A5030,A5031,A5032,A5039,A5040,A5041,A5042 A5043,A5044,A5045,A5049,A5051,A5052,A5053,A5054,A5055,A5056 A5057,A5059,A506,A507,A509,P230,P231,P232,P233,P234,P235,P236 P238,P239,P350,P351,P352,P353,P354,P358,P359,P360,P3610,P3619 P362,P3630,P3639,P364,P365,P368,P369,P370,P371,P372,P373,P374 P375,P378,P379,P381,P389,P390,P391,P392,P393,P394,P398,P399 | 04041,0900,0901,0902,0903,09040,09041, 09042,09049,0905,0906 0907,0909,7700,7710,7711,7712,7713, 7714,7715,7716,7717,77181 77182,77189 |
| Peripheral and visceral vascular disease | I700,I701,I70201,I70202,I70203,I70208,I70209,I70211,I70212 I70213,I70218,I70219,I70221,I70222,I70223,I70228,I70229 I70231,I70232,I70233,I70234,I70235,I70238,I70239,I70241 I70242,I70243,I70244,I70245,I70248,I70249,I7025,I70261,I70262 I70263,I70268,I70269,I70291,I70292,I70293,I70298,I70299 I70301,I70302,I70303,I70308,I70309,I70311,I70312,I70313 I70318,I70319,I70321,I70322,I70323,I70328,I70329,I70331 I70332,I70333,I70334,I70335,I70338,I70339,I70341,I70342 I70343,I70344,I70345,I70348,I70349,I7035,I70361,I70362,I70363 I70368,I70369,I70391,I70392,I70393,I70398,I70399,I70401 I70402,I70403,I70408,I70409,I70411,I70412,I70413,I70418 I70419,I70421,I70422,I70423,I70428,I70429,I70431,I70432 I70433,I70434,I70435,I70438,I70439,I70441,I70442,I70443 I70444,I70445,I70448,I70449,I7045,I70461,I70462,I70463,I70468 I70469,I70491,I70492,I70493,I70498,I70499,I70501,I70502 I70503,I70508,I70509,I70511,I70512,I70513,I70518,I70519 I70521,I70522,I70523,I70528,I70529,I70531,I70532,I70533 I70534,I70535,I70538,I70539,I70541,I70542,I70543,I70544 I70545,I70548,I70549,I7055,I70561,I70562,I70563,I70568,I70569 I70591,I70592,I70593,I70598,I70599,I70601,I70602,I70603 I70608,I70609,I70611,I70612,I70613,I70618,I70619,I70621 I70622,I70623,I70628,I70629,I70631,I70632,I70633,I70634 I70635,I70638,I70639,I70641,I70642,I70643,I70644,I70645 I70648,I70649,I7065,I70661,I70662,I70663,I70668,I70669,I70691 I70692,I70693,I70698,I70699,I70701,I70702,I70703,I70708 I70709,I70711,I70712,I70713,I70718,I70719,I70721,I70722 I70723,I70728,I70729,I70731,I70732,I70733,I70734,I70735 I70738,I70739,I70741,I70742,I70743,I70744,I70745,I70748 I70749,I7075,I70761,I70762,I70763,I70768,I70769,I70791,I70792 I70793,I70798,I70799,I708,I7090,I7091,I7092,I7300,I7301,I731 I7381,I7389,I739,K550,K55011,K55012,K55019,K55021,K55022 K55029,K55031,K55032,K55039,K55041,K55042,K55049,K55051 K55052,K55059,K55061,K55062,K55069,K551,K5520,K5521,Z95820 Z9862 | 4400,4401,44020,44021,44022,44023, 44024,44029,44030,44031 44032,4404,4408,4409,4430,4431,44382, 44389,4439,5570,5571 56984,56985,70711,70712,70713,70714, 70715,70719,7079,7854 V434,V4589 |
| Peritonitis and intra-abdominal abscess | A1831,A5485,A7481,K352,K3520,K3521,K353,K3530,K3531,K3532 K3533,K50014,K50114,K50814,K50914,K51014,K51214,K51314,K51414 K51514,K51814,K51914,K5700,K5720,K5740,K5780,K610,K611,K612 K613,K6131,K6139,K614,K615,K630,K650,K651,K652,K653,K654,K658 K659,K67,K6811,K6812,K6819,K750 | 01400,07988,09886,5400,5401,5550,5551, 5552,5559,5562,5563 5564,5565,5566,5568,5569,56201,56211, 566,5670,56721,56722 56723,56729,56731,56738,56781,56782, 56789,5679,5695,5720 99859 |
| Personal family history of disease | Z1501,Z1502,Z1503,Z1504,Z1509,Z1581,Z1589,Z170,Z171,Z191,Z192 Z800,Z801,Z802,Z803,Z8041,Z8042,Z8043,Z8049,Z8051,Z8052,Z8059 Z806,Z807,Z808,Z809,Z810,Z811,Z812,Z813,Z814,Z818,Z820,Z821 Z822,Z823,Z8241,Z8249,Z825,Z8261,Z8262,Z8269,Z8271,Z8279,Z828 Z830,Z831,Z832,Z833,Z8341,Z8342,Z83430,Z83438,Z8349,Z83511 Z83518,Z8352,Z836,Z8371,Z8379,Z840,Z841,Z842,Z843,Z8481,Z8482 Z8489,Z8500,Z8501,Z85020,Z85028,Z85030,Z85038,Z85040,Z85048 Z8505,Z85060,Z85068,Z8507,Z8509,Z85110,Z85118,Z8512,Z8520 | 30250,V1000,V1001,V1002,V1003,V1004, V1005,V1006,V1007,V1009 V1011,V1012,V1020,V1021,V1022, V1029,V103,V1040,V1041,V1042 V1043,V1044,V1045,V1046,V1047, V1048,V1049,V1050,V1051,V1052 V1053,V1059,V1060,V1072,V1079, V1081,V1082,V1083,V1084,V1085 V1086,V1087,V1088,V1089,V1090, V1091,V114,V118,V1200,V1201 V1202,V1203,V1204,V1209,V121,V1221, V1229,V123,V1240,V1241 V1242,V1249,V1250,V1251,V1252, V1253,V1254,V1255,V1259,V1260 V1261,V1269,V1270,V1271,V1272, V1279,V1300,V1301,V1302,V1303 |

Continued on next page

Table S9 (continued).

| Diagnosis | ICD-10 | ICD-9 |
|---|---|---|
| | Z8521,Z8522,Z85230,Z85238,Z8529,Z853,Z8540,Z8541,Z8542,Z8543 | V1309,V131,V1321,V1322,V1323,V1324, V1329,V133,V1351,V1352 |
| | Z8544,Z8545,Z8546,Z8547,Z8548,Z8549,Z8550,Z8551,Z85520,Z85528 | V1359,V1361,V1362,V1363,V1364, V1365,V1366,V1367,V1368,V1369 |
| | Z8553,Z8554,Z8559,Z856,Z8571,Z8572,Z8579,Z85810,Z85818,Z85819 | V1381,V1389,V1521,V1522,V153,V1541, V1542,V1549,V1551,V1552 |
| | Z85820,Z85821,Z85828,Z85830,Z85831,Z85840,Z85841,Z85848 | V1553,V1559,V157,V1580,V1582,V1587, V1588,V1589,V160,V161,V162 |
| | Z85850,Z85858,Z8589,Z859,Z86000,Z86001,Z86002,Z86003,Z86004 | V163,V1640,V1641,V1642,V1643,V1649, V1651,V1652,V1659,V166 |
| | Z86005,Z86006,Z86007,Z86008,Z86010,Z86011,Z86012,Z86018,Z8603 | V167,V168,V169,V170,V171,V172,V173, V1741,V1749,V175,V176,V177 |
| | Z8611,Z8612,Z8613,Z8614,Z8615,Z8616,Z8619,Z862,Z8631,Z8632 | V1781,V1789,V180,V1811,V1819,V182, V183,V184,V1851,V1859,V1861 |
| | Z8639,Z8651,Z8659,Z8661,Z8669,Z86711,Z86718,Z8672,Z8673,Z8674 | V1869,V187,V188,V189,V190,V1911, V1919,V192,V193,V194,V195 |
| | Z8679,Z8701,Z8709,Z8711,Z8719,Z872,Z87310,Z87311,Z87312,Z8739 | V196,V197,V198,V4583,V4588,V8401, V8402,V8403,V8404,V8409 |
| | Z87410,Z87411,Z87412,Z8742,Z87430,Z87438,Z87440,Z87441,Z87442 | V8481,V8489,V860,V861,V8741,V8742, V8743,V8744,V8745,V8746 |
| | Z87448,Z8751,Z8759,Z8761,Z8768,Z87710,Z87718,Z87720,Z87721 Z87728,Z87730,Z87731,Z87732,Z87738,Z8774,Z8775,Z8776,Z87760 Z87761,Z87762,Z87763,Z87768,Z87790,Z87798,Z8781,Z87820,Z87821 Z87828,Z87890,Z87891,Z87892,Z87898,Z91410,Z91411,Z91412 Z91419,Z9142,Z9149,Z915,Z9151,Z9152,Z9181,Z9182,Z920,Z9221 Z9222,Z9223,Z92240,Z92241,Z9225,Z9229,Z923,Z9281,Z9282,Z9283 Z9284,Z92850,Z92858,Z92859,Z9286,Z9289,Z9886,Z98870,Z98871 Z98891 | V8749 |
| Personality disorders | F070,F21,F600,F601,F602,F603,F604,F605,F606,F607,F6081,F6089 | 30019,3010,30120,30122,3013,3014,30150, 30159,3016,3017,30181 |
| | F609,F688,F69 | 30182,30183,30184,30189,3019,3100,3101 |
| Pituitary disorders | E220,E221,E222,E228,E229,E230,E231,E232,E233,E236,E237,E240 | 2530,2531,2532,2533,2535,2536,2537, 2538,2539,2550,6281 |
| Pleurisy, pleural | A156,J860,J869,J90,J910,J918,J920,J929,J940,J941,J942,J948 | 01200,5100,5109,5110,5111,51181,51189, 5119,5180,5181,5182 |
| effusion and pulmonary collapse | J949,J9811,J9819,J982,J983,R091 | 53084 |
| Pneumonia (except | A0103,A0222,A202,A212,A221,A310,A3701,A3711,A3781,A3791,A430 | 0020,00322,0203,0204,0205,0212,0221, 0310,0330,0331,0338,0339 |
| that caused by | A481,A5004,A5272,A5484,B012,B052,B0681,B250,B371,B380,B381 | 0391,0521,0551,05679,0785,0900,0951, 09889,1124,1140,1144,1145 |
| tuberculosis) | B382,B390,B391,B392,B583,B59,B7781,J09X1,J1000,J1001,J1008 | 11505,1270,1304,1363,4800,4801,4802, 4803,4808,4809,481,4820 |
| | J1100,J1108,J120,J121,J122,J123,J1281,J1282,J1289,J129,J13 | 4821,4822,48231,48232,48239,48240, 48241,48242,48249,48281 |
| | J14,J150,J151,J1520,J15211,J15212,J1529,J153,J154,J155,J156 | 48282,48283,48284,48289,4829,4830,4831, 4838,4841,4843,4845 |
| | J157,J158,J159,J160,J168,J17,J180,J181,J188,J189,J851,J95851 | 4847,4848,485,486,4870,4871,48801, 48811,48881,5130,5171,99731 |
| Pneumothorax | J930,J9311,J9312,J9381,J9382,J9383,J939,J95811 | 5120,5121,51281,51282,51283,51284, 51289 |
| Poisoning by drugs, | T360X1A,T360X2A,T360X3A,T360X4A,T361X1A,T361X2A,T361X3A | 9600,9601,9602,9603,9604,9605,9606, 9608,9609,9610,9614,9615 |
| initial encounter | T361X4A,T362X1A,T362X2A,T362X3A,T362X4A,T363X1A,T363X2A | 9616,9617,9618,9619,9620,9621,9622, 9623,9624,9627,9628,9629 |
| | T363X3A,T363X4A,T364X1A,T364X2A,T364X3A,T364X4A,T365X1A | 9630,9631,9634,9635,9638,9639,9640, 9642,9643,9644,9645,9646 |
| | T365X2A,T365X3A,T365X4A,T366X1A,T366X2A,T366X3A,T366X4A | 9648,9649,96500,96501,96502,96509,9651, 9654,9655,96561,96569 |
| | T367X1A,T367X2A,T367X3A,T367X4A,T368X1A,T368X2A,T368X3A | 9657,9659,9660,9661,9662,9663,9664, 9670,9678,9679,9680,9682 |
| | T368X4A,T3691XA,T3692XA,T3693XA,T3694XA,T370X1A,T370X2A | 9683,9684,9685,9689,96900,96901,96902, 96903,96904,96905,96909 |
| | T370X3A,T370X4A,T371X1A,T371X2A,T371X3A,T371X4A,T372X1A | 9691,9692,9693,9694,9696,96970,96971, 96972,96973,96979,9698 |
| | T372X2A,T372X3A,T372X4A,T373X1A,T373X2A,T373X3A,T373X4A | 9699,9700,9701,97081,9710,9711,9712, 9713,9719,9720,9721,9722 |
| | T374X1A,T374X2A,T374X3A,T374X4A,T375X1A,T375X2A,T375X3A | 9723,9724,9725,9726,9727,9729,9730, 9731,9732,9733,9734,9735 |
| | T375X4A,T378X1A,T378X2A,T378X3A,T378X4A,T3791XA,T3792XA | 9736,9738,9739,9742,9744,9745,9747, 9750,9751,9752,9753,9754 |
| | T3793XA,T3794XA,T380X1A,T380X2A,T380X3A,T380X4A,T381X1A | 9755,9756,9757,9758,9760,9761,9762, 9763,9764,9765,9766,9767 |
| | T381X2A,T381X3A,T381X4A,T382X1A,T382X2A,T382X3A,T382X4A | 9768,9769,9770,9772,9778,9779,9786, 9788,9789,9790,9796,9799 |
| | T383X1A,T383X2A,T383X3A,T383X4A,T384X1A,T384X2A,T384X3A | E8500,E8501,E8502,E8503,E8504,E8505, E8506,E8507,E851,E8528 |
| | T384X4A,T385X1A,T385X2A,T385X3A,T385X4A,T386X1A,T386X2A | E8530,E8531,E8532,E8540,E8541,E8542, E8543,E8548,E8550,E8551 |
| | T386X3A,T386X4A,T387X1A,T387X2A,T387X3A,T387X4A,T38801A | E8552,E8553,E8554,E8555,E8556,E8558, E856,E857,E8580,E8581 |

Continued on next page

Table S9 (continued).

| Diagnosis | ICD-10 | ICD-9 |
|---|---|---|
| | T38802A,T38803A,T38804A,T38811A,T38812A,T38813A,T38814A<br>T38891A,T38892A,T38893A,T38894A,T38901A,T38902A,T38903A<br>T38904A,T38991A,T38992A,T38993A,T38994A,T39011A,T39012A<br>T39013A,T39014A,T39091A,T39092A,T39093A,T39094A,T391X1A<br>T391X2A,T391X3A,T391X4A,T392X1A,T392X2A,T392X3A,T392X4A<br>T39311A,T39312A,T39313A,T39314A,T39391A,T39392A,T39393A<br>T39394A,T394X1A,T394X2A,T394X3A,T394X4A,T398X1A,T398X2A<br>T398X3A,T398X4A,T3991XA,T3992XA,T3993XA,T3994XA,T400X1A<br>T400X2A,T400X3A,T400X4A,T401X1A,T401X2A,T401X3A,T401X4A<br>T402X1A,T402X2A,T402X3A,T402X4A,T403X1A,T403X2A,T403X3A<br>T403X4A,T40411A,T40412A,T40413A,T40414A,T40421A,T40422A<br>T40423A,T40424A,T40491A,T40492A,T40493A,T40494A,T404X1A<br>T404X2A,T404X3A,T404X4A,T405X1A,T405X2A,T405X3A,T405X4A<br>T40601A,T40602A,T40603A,T40604A,T40691A,T40692A,T40693A<br>T40694A,T40711A,T40712A,T40713A,T40714A,T40721A,T40722A<br>T40723A,T40724A,T407X1A,T407X2A,T407X3A,T407X4A,T408X1A<br>T408X2A,T408X3A,T408X4A,T40901A,T40902A,T40903A,T40904A<br>T40991A,T40992A,T40993A,T40994A,T410X1A,T410X2A,T410X3A<br>T410X4A,T411X1A,T411X2A,T411X3A,T411X4A,T41201A,T41202A<br>T41203A,T41204A,T41291A,T41292A,T41293A,T41294A,T413X1A<br>T413X2A,T413X3A,T413X4A,T4141XA,T4142XA,T4143XA,T4144XA<br>T415X1A,T415X2A,T415X3A,T415X4A,T420X1A,T420X2A,T420X3A<br>T420X4A,T421X1A,T421X2A,T421X3A,T421X4A,T422X1A,T422X2A<br>T422X3A,T422X4A,T423X1A,T423X2A,T423X3A,T423X4A,T424X1A<br>T424X2A,T424X3A,T424X4A,T425X1A,T425X2A,T425X3A,T425X4A<br>T426X1A,T426X2A,T426X3A,T426X4A,T4271XA,T4272XA,T4273XA<br>T4274XA,T428X1A,T428X2A,T428X3A,T428X4A,T43011A,T43012A<br>T43013A,T43014A,T43021A,T43022A,T43023A,T43024A,T431X1A<br>T431X2A,T431X3A,T431X4A,T43201A,T43202A,T43203A,T43204A<br>T43211A,T43212A,T43213A,T43214A,T43221A,T43222A,T43223A<br>T43224A,T43291A,T43292A,T43293A,T43294A,T433X1A,T433X2A<br>T433X3A,T433X4A,T434X1A,T434X2A,T434X3A,T434X4A,T43501A<br>T43502A,T43503A,T43504A,T43591A,T43592A,T43593A,T43594A<br>T43601A,T43602A,T43603A,T43604A,T43611A,T43612A,T43613A<br>T43614A,T43621A,T43622A,T43623A,T43624A,T43631A,T43632A<br>T43633A,T43634A,T43641A,T43642A,T43643A,T43644A,T43651A<br>T43652A,T43653A,T43654A,T43691A,T43692A,T43693A,T43694A<br>T438X1A,T438X2A,T438X3A,T438X4A,T4391XA,T4392XA,T4393XA<br>T4394XA,T440X1A,T440X2A,T440X3A,T440X4A,T441X1A,T441X2A<br>T441X3A,T441X4A,T442X1A,T442X2A,T442X3A,T442X4A,T443X1A<br>T443X2A,T443X3A,T443X4A,T444X1A,T444X2A,T444X3A,T444X4A<br>T445X1A,T445X2A,T445X3A,T445X4A,T446X1A,T446X2A,T446X3A<br>T446X4A,T447X1A,T447X2A,T447X3A,T447X4A,T448X1A,T448X2A<br>T448X3A,T448X4A,T44901A,T44902A,T44903A,T44904A,T44991A<br>T44992A,T44993A,T44994A,T450X1A,T450X2A,T450X3A,T450X4A<br>T451X1A,T451X2A,T451X3A,T451X4A,T452X1A,T452X2A,T452X3A<br>T452X4A,T453X1A,T453X2A,T453X3A,T453X4A,T454X1A,T454X2A<br>T454X3A,T454X4A,T45511A,T45512A,T45513A,T45514A,T45521A<br>T45522A,T45523A,T45524A,T45601A,T45602A,T45603A,T45604A<br>T45611A,T45612A,T45613A,T45614A,T45621A,T45622A,T45623A<br>T45624A,T45691A,T45692A,T45693A,T45694A,T457X1A,T457X2A<br>T457X3A,T457X4A,T458X1A,T458X2A,T458X3A,T458X4A,T4591XA<br>T4592XA,T4593XA,T4594XA,T460X1A,T460X2A,T460X3A,T460X4A<br>T461X1A,T461X2A,T461X3A,T461X4A,T462X1A,T462X2A,T462X3A<br>T462X4A,T463X1A,T463X2A,T463X3A,T463X4A,T464X1A,T464X2A<br>T464X3A,T464X4A,T465X1A,T465X2A,T465X3A,T465X4A,T466X1A<br>T466X2A,T466X3A,T466X4A,T467X1A,T467X2A,T467X3A,T467X4A<br>T468X1A,T468X2A,T468X3A,T468X4A,T46901A,T46902A,T46903A<br>T46904A,T46991A,T46992A,T46993A,T46994A,T470X1A,T470X2A<br>T470X3A,T470X4A,T471X1A,T471X2A,T471X3A,T471X4A,T472X1A<br>T472X2A,T472X3A,T472X4A,T473X1A,T473X2A,T473X3A,T473X4A<br>T474X1A,T474X2A,T474X3A,T474X4A,T475X1A,T475X2A,T475X3A<br>T475X4A,T476X1A,T476X2A,T476X3A,T476X4A,T477X1A,T477X2A<br>T477X3A,T477X4A,T478X1A,T478X2A,T478X3A,T478X4A,T4791XA<br>T4792XA,T4793XA,T4794XA,T480X1A,T480X2A,T480X3A,T480X4A<br>T481X1A,T481X2A,T481X3A,T481X4A,T48201A,T48202A,T48203A<br>T48204A,T48291A,T48292A,T48293A,T48294A,T483X1A,T483X2A<br>T483X3A,T483X4A,T484X1A,T484X2A,T484X3A,T484X4A,T485X1A<br>T485X2A,T485X3A,T485X4A,T486X1A,T486X2A,T486X3A,T486X4A<br>T48901A,T48902A,T48903A,T48904A,T48991A,T48992A,T48993A<br>T48994A,T490X1A,T490X2A,T490X3A,T490X4A,T491X1A,T491X2A<br>T491X3A,T491X4A,T492X1A,T492X2A,T492X3A,T492X4A,T493X1A<br>T493X2A,T493X3A,T493X4A,T494X1A,T494X2A,T494X3A,T494X4A<br>T495X1A,T495X2A,T495X3A,T495X4A,T496X1A,T496X2A,T496X3A<br>T496X4A,T497X1A,T497X2A,T497X3A,T497X4A,T498X1A,T498X2A<br>T498X3A,T498X4A,T4991XA,T4992XA,T4993XA,T4994XA,T500X1A<br>T500X2A,T500X3A,T500X4A,T501X1A,T501X2A,T501X3A,T501X4A<br>T502X1A,T502X2A,T502X3A,T502X4A,T503X1A,T503X2A,T503X3A<br>T503X4A,T504X1A,T504X2A,T504X3A,T504X4A,T505X1A,T505X2A<br>T505X3A,T505X4A,T506X1A,T506X2A,T506X3A,T506X4A,T507X1A<br>T507X2A,T507X3A,T507X4A,T508X1A,T508X2A,T508X3A,T508X4A<br>T50901A,T50902A,T50903A,T50904A,T50911A,T50912A,T50913A<br>T50914A,T50991A,T50992A,T50993A,T50994A,T50A11A,T50A12A | E8582,E8583,E8584,E8585,E8586,E8587,<br>E8588,E9500,E9501,E9502<br>E9503,E9504,E9528,E9620,E9800,E9801,<br>E9802,E9803,E9804,E9805 |

Continued on next page

Table S9 (continued).

| Diagnosis | ICD-10 | ICD-9 |
|---|---|---|
| | T50A13A,T50A14A,T50A21A,T50A22A,T50A23A,T50A24A,T50A91A<br>T50A92A,T50A93A,T50A94A,T50B11A,T50B12A,T50B13A,T50B14A<br>T50B91A,T50B92A,T50B93A,T50B94A,T50Z11A,T50Z12A,T50Z13A<br>T50Z14A,T50Z91A,T50Z92A,T50Z93A,T50Z94A | |
| Poisoning by drugs, subsequent encounter | T360X1D,T360X2D,T360X3D,T360X4D,T361X1D,T361X2D,T361X3D<br>T361X4D,T362X1D,T362X2D,T362X3D,T362X4D,T363X1D,T363X2D<br>T363X3D,T363X4D,T364X1D,T364X2D,T364X3D,T364X4D,T365X1D<br>T365X2D,T365X3D,T365X4D,T366X1D,T366X2D,T366X3D,T366X4D<br>T367X1D,T367X2D,T367X3D,T367X4D,T368X1D,T368X2D,T368X3D<br>T368X4D,T3691XD,T3692XD,T3693XD,T3694XD,T370X1D,T370X2D<br>T370X3D,T370X4D,T371X1D,T371X2D,T371X3D,T371X4D,T372X1D<br>T372X2D,T372X3D,T372X4D,T373X1D,T373X2D,T373X3D,T373X4D<br>T374X1D,T374X2D,T374X3D,T374X4D,T375X1D,T375X2D,T375X3D<br>T375X4D,T378X1D,T378X2D,T378X3D,T378X4D,T3791XD,T3792XD<br>T3793XD,T3794XD,T380X1D,T380X2D,T380X3D,T380X4D,T381X1D<br>T381X2D,T381X3D,T381X4D,T382X1D,T382X2D,T382X3D,T382X4D<br>T383X1D,T383X2D,T383X3D,T383X4D,T384X1D,T384X2D,T384X3D<br>T384X4D,T385X1D,T385X2D,T385X3D,T385X4D,T386X1D,T386X2D<br>T386X3D,T386X4D,T387X1D,T387X2D,T387X3D,T387X4D,T38801D<br>T38802D,T38803D,T38804D,T38811D,T38812D,T38813D,T38814D<br>T38891D,T38892D,T38893D,T38894D,T38901D,T38902D,T38903D<br>T38904D,T38991D,T38992D,T38993D,T38994D,T39011D,T39012D<br>T39013D,T39014D,T39091D,T39092D,T39093D,T39094D,T391X1D<br>T391X2D,T391X3D,T391X4D,T392X1D,T392X2D,T392X3D,T392X4D<br>T39311D,T39312D,T39313D,T39314D,T39391D,T39392D,T39393D<br>T39394D,T394X1D,T394X2D,T394X3D,T394X4D,T398X1D,T398X2D<br>T398X3D,T398X4D,T3991XD,T3992XD,T3993XD,T3994XD,T400X1D<br>T400X2D,T400X3D,T400X4D,T401X1D,T401X2D,T401X3D,T401X4D<br>T402X1D,T402X2D,T402X3D,T402X4D,T403X1D,T403X2D,T403X3D<br>T403X4D,T40411D,T40412D,T40413D,T40414D,T40421D,T40422D<br>T40423D,T40424D,T40491D,T40492D,T40493D,T40494D,T404X1D<br>T404X2D,T404X3D,T404X4D,T405X1D,T405X2D,T405X3D,T405X4D<br>T40601D,T40602D,T40603D,T40604D,T40691D,T40692D,T40693D<br>T40694D,T40711D,T40712D,T40713D,T40714D,T40721D,T40722D<br>T40723D,T40724D,T407X1D,T407X2D,T407X3D,T407X4D,T408X1D<br>T408X2D,T408X3D,T408X4D,T40901D,T40902D,T40903D,T40904D<br>T40991D,T40992D,T40993D,T40994D,T410X1D,T410X2D,T410X3D<br>T410X4D,T411X1D,T411X2D,T411X3D,T411X4D,T41201D,T41202D<br>T41203D,T41204D,T41291D,T41292D,T41293D,T41294D,T413X1D<br>T413X2D,T413X3D,T413X4D,T4141XD,T4142XD,T4143XD,T4144XD<br>T415X1D,T415X2D,T415X3D,T415X4D,T420X1D,T420X2D,T420X3D<br>T420X4D,T421X1D,T421X2D,T421X3D,T421X4D,T422X1D,T422X2D<br>T422X3D,T422X4D,T423X1D,T423X2D,T423X3D,T423X4D,T424X1D<br>T424X2D,T424X3D,T424X4D,T425X1D,T425X2D,T425X3D,T425X4D<br>T426X1D,T426X2D,T426X3D,T426X4D,T4271XD,T4272XD,T4273XD<br>T4274XD,T428X1D,T428X2D,T428X3D,T428X4D,T43011D,T43012D<br>T43013D,T43014D,T43021D,T43022D,T43023D,T43024D,T431X1D<br>T431X2D,T431X3D,T431X4D,T43201D,T43202D,T43203D,T43204D<br>T43211D,T43212D,T43213D,T43214D,T43221D,T43222D,T43223D<br>T43224D,T43291D,T43292D,T43293D,T43294D,T433X1D,T433X2D<br>T433X3D,T433X4D,T434X1D,T434X2D,T434X3D,T434X4D,T43501D<br>T43502D,T43503D,T43504D,T43591D,T43592D,T43593D,T43594D<br>T43601D,T43602D,T43603D,T43604D,T43611D,T43612D,T43613D<br>T43614D,T43621D,T43622D,T43623D,T43624D,T43631D,T43632D<br>T43633D,T43634D,T43641D,T43642D,T43643D,T43644D,T43651D<br>T43652D,T43653D,T43654D,T43691D,T43692D,T43693D,T43694D<br>T438X1D,T438X2D,T438X3D,T438X4D,T4391XD,T4392XD,T4393XD<br>T4394XD,T440X1D,T440X2D,T440X3D,T440X4D,T441X1D,T441X2D<br>T441X3D,T441X4D,T442X1D,T442X2D,T442X3D,T442X4D,T443X1D<br>T443X2D,T443X3D,T443X4D,T444X1D,T444X2D,T444X3D,T444X4D<br>T445X1D,T445X2D,T445X3D,T445X4D,T446X1D,T446X2D,T446X3D<br>T446X4D,T447X1D,T447X2D,T447X3D,T447X4D,T448X1D,T448X2D<br>T448X3D,T448X4D,T44901D,T44902D,T44903D,T44904D,T44991D<br>T44992D,T44993D,T44994D,T450X1D,T450X2D,T450X3D,T450X4D<br>T451X1D,T451X2D,T451X3D,T451X4D,T452X1D,T452X2D,T452X3D<br>T452X4D,T453X1D,T453X2D,T453X3D,T453X4D,T454X1D,T454X2D<br>T454X3D,T454X4D,T45511D,T45512D,T45513D,T45514D,T45521D<br>T45522D,T45523D,T45524D,T45601D,T45602D,T45603D,T45604D<br>T45611D,T45612D,T45613D,T45614D,T45621D,T45622D,T45623D<br>T45624D,T45691D,T45692D,T45693D,T45694D,T457X1D,T457X2D<br>T457X3D,T457X4D,T458X1D,T458X2D,T458X3D,T458X4D,T4591XD<br>T4592XD,T4593XD,T4594XD,T460X1D,T460X2D,T460X3D,T460X4D<br>T461X1D,T461X2D,T461X3D,T461X4D,T462X1D,T462X2D,T462X3D<br>T462X4D,T463X1D,T463X2D,T463X3D,T463X4D,T464X1D,T464X2D<br>T464X3D,T464X4D,T465X1D,T465X2D,T465X3D,T465X4D,T466X1D<br>T466X2D,T466X3D,T466X4D,T467X1D,T467X2D,T467X3D,T467X4D<br>T468X1D,T468X2D,T468X3D,T468X4D,T46901D,T46902D,T46903D<br>T46904D,T46991D,T46992D,T46993D,T46994D,T470X1D,T470X2D<br>T470X3D,T470X4D,T471X1D,T471X2D,T471X3D,T471X4D,T472X1D<br>T472X2D,T472X3D,T472X4D,T473X1D,T473X2D,T473X3D,T473X4D<br>T474X1D,T474X2D,T474X3D,T474X4D,T475X1D,T475X2D,T475X3D<br>T475X4D,T476X1D,T476X2D,T476X3D,T476X4D,T477X1D,T477X2D<br>T477X3D,T477X4D,T478X1D,T478X2D,T478X3D,T478X4D,T4791XD<br>T4792XD,T4793XD,T4794XD,T480X1D,T480X2D,T480X3D,T480X4D | V5889 |

Continued on next page

Table S9 (continued).

| Diagnosis | ICD-10 | ICD-9 |
|---|---|---|
| | T481X1D,T481X2D,T481X3D,T481X4D,T48201D,T48202D,T48203D | |
| | T48204D,T48291D,T48292D,T48293D,T48294D,T483X1D,T483X2D | |
| | T483X3D,T483X4D,T484X1D,T484X2D,T484X3D,T484X4D,T485X1D | |
| | T485X2D,T485X3D,T485X4D,T486X1D,T486X2D,T486X3D,T486X4D | |
| | T48901D,T48902D,T48903D,T48904D,T48991D,T48992D,T48993D | |
| | T48994D,T490X1D,T490X2D,T490X3D,T490X4D,T491X1D,T491X2D | |
| | T491X3D,T491X4D,T492X1D,T492X2D,T492X3D,T492X4D,T493X1D | |
| | T493X2D,T493X3D,T493X4D,T494X1D,T494X2D,T494X3D,T494X4D | |
| | T495X1D,T495X2D,T495X3D,T495X4D,T496X1D,T496X2D,T496X3D | |
| | T496X4D,T497X1D,T497X2D,T497X3D,T497X4D,T498X1D,T498X2D | |
| | T498X3D,T498X4D,T4991XD,T4992XD,T4993XD,T4994XD,T500X1D | |
| | T500X2D,T500X3D,T500X4D,T501X1D,T501X2D,T501X3D,T501X4D | |
| | T502X1D,T502X2D,T502X3D,T502X4D,T503X1D,T503X2D,T503X3D | |
| | T503X4D,T504X1D,T504X2D,T504X3D,T504X4D,T505X1D,T505X2D | |
| | T505X3D,T505X4D,T506X1D,T506X2D,T506X3D,T506X4D,T507X1D | |
| | T507X2D,T507X3D,T507X4D,T508X1D,T508X2D,T508X3D,T508X4D | |
| | T50901D,T50902D,T50903D,T50904D,T50911D,T50912D,T50913D | |
| | T50914D,T50991D,T50992D,T50993D,T50994D,T50A11D,T50A12D | |
| | T50A13D,T50A14D,T50A21D,T50A22D,T50A23D,T50A24D,T50A91D | |
| | T50A92D,T50A93D,T50A94D,T50B11D,T50B12D,T50B13D,T50B14D | |
| | T50B91D,T50B92D,T50B93D,T50B94D,T50Z11D,T50Z12D,T50Z13D | |
| | T50Z14D,T50Z91D,T50Z92D,T50Z93D,T50Z94D | |
| Poisoning toxic | T360X1S,T360X2S,T360X3S,T360X4S,T360X5S,T360X6S,T361X1S | 9090,9091,9095,9099,E9292,E9300,E9301, E9302,E9303,E9304,E9305 |
| effect adverse | T361X2S,T361X3S,T361X4S,T361X5S,T361X6S,T362X1S,T362X2S | E9306,E9308,E9310,E9314,E9315,E9316, E9317,E9319,E9320,E9321 |
| effects underdosing, | T362X3S,T362X4S,T362X5S,T362X6S,T363X1S,T363X2S,T363X3S | E9322,E9323,E9324,E9327,E9328,E9329, E9330,E9331,E9334,E9335 |
| sequela | T363X4S,T363X5S,T363X6S,T364X1S,T364X2S,T364X3S,T364X4S | E9336,E9337,E9338,E9340,E9342,E9344, E9345,E9346,E9348,E9349 |
| | T364X5S,T364X6S,T365X1S,T365X2S,T365X3S,T365X4S,T365X5S | E9350,E9351,E9352,E9353,E9355,E9356, E9357,E9360,E9361,E9362 |
| | T365X6S,T366X1S,T366X2S,T366X3S,T366X4S,T366X5S,T366X6S | E9363,E9364,E9370,E9378,E9379,E9382, E9383,E9384,E9389,E9390 |
| | T367X1S,T367X2S,T367X3S,T367X4S,T367X5S,T367X6S,T368X1S | E9391,E9392,E9393,E9394,E9396,E9397, E9398,E9399,E9400,E9401 |
| | T368X2S,T368X3S,T368X4S,T368X5S,T368X6S,T3691XS,T3692XS | E9410,E9411,E9412,E9413,E9419,E9420, E9421,E9422,E9424,E9425 |
| | T3693XS,T3694XS,T3695XS,T3696XS,T370X1S,T370X2S,T370X3S | E9426,E9427,E9429,E9430,E9431,E9432, E9433,E9434,E9435,E9436 |
| | T370X4S,T370X5S,T370X6S,T371X1S,T371X2S,T371X3S,T371X4S | E9438,E9439,E9442,E9444,E9445,E9447, E9450,E9452,E9453,E9454 |
| | T371X5S,T371X6S,T372X1S,T372X2S,T372X3S,T372X4S,T372X5S | E9455,E9456,E9457,E9458,E9460,E9461, E9462,E9463,E9464,E9465 |
| | T372X6S,T373X1S,T373X2S,T373X3S,T373X4S,T373X5S,T373X6S | E9466,E9467,E9468,E9469,E9470,E9472, E9478,E9479,E9486,E9488 |
| | T374X1S,T374X2S,T374X3S,T374X4S,T374X5S,T374X6S,T375X1S | E9489,E9490,E9496,E9499,E959,E969, E989,NoDx |
| | T375X2S,T375X3S,T375X4S,T375X5S,T375X6S,T378X1S,T378X2S | |
| | T378X3S,T378X4S,T378X5S,T378X6S,T3791XS,T3792XS,T3793XS | |
| | T3794XS,T3795XS,T3796XS,T380X1S,T380X2S,T380X3S,T380X4S | |
| | T380X5S,T380X6S,T381X1S,T381X2S,T381X3S,T381X4S,T381X5S | |
| | T381X6S,T382X1S,T382X2S,T382X3S,T382X4S,T382X5S,T382X6S | |
| | T383X1S,T383X2S,T383X3S,T383X4S,T383X5S,T383X6S,T384X1S | |
| | T384X2S,T384X3S,T384X4S,T384X5S,T384X6S,T385X1S,T385X2S | |
| | T385X3S,T385X4S,T385X5S,T385X6S,T386X1S,T386X2S,T386X3S | |
| | T386X4S,T386X5S,T386X6S,T387X1S,T387X2S,T387X3S,T387X4S | |
| | T387X5S,T387X6S,T38801S,T38802S,T38803S,T38804S,T38805S | |
| | T38806S,T38811S,T38812S,T38813S,T38814S,T38815S,T38816S | |
| | T38891S,T38892S,T38893S,T38894S,T38895S,T38896S,T38901S | |
| | T38902S,T38903S,T38904S,T38905S,T38906S,T38991S,T38992S | |
| | T38993S,T38994S,T38995S,T38996S,T39011S,T39012S,T39013S | |
| | T39014S,T39015S,T39016S,T39091S,T39092S,T39093S,T39094S | |
| | T39095S,T39096S,T391X1S,T391X2S,T391X3S,T391X4S,T391X5S | |
| | T391X6S,T392X1S,T392X2S,T392X3S,T392X4S,T392X5S,T392X6S | |
| | T39311S,T39312S,T39313S,T39314S,T39315S,T39316S,T39391S | |
| | T39392S,T39393S,T39394S,T39395S,T39396S,T394X1S,T394X2S | |
| | T394X3S,T394X4S,T394X5S,T394X6S,T398X1S,T398X2S,T398X3S | |
| | T398X4S,T398X5S,T398X6S,T3991XS,T3992XS,T3993XS,T3994XS | |
| | T3995XS,T3996XS,T400X1S,T400X2S,T400X3S,T400X4S,T400X5S | |
| | T400X6S,T401X1S,T401X2S,T401X3S,T401X4S,T401X5S,T402X1S | |
| | T402X2S,T402X3S,T402X4S,T402X5S,T402X6S,T403X1S,T403X2S | |
| | T403X3S,T403X4S,T403X5S,T403X6S,T40411S,T40412S,T40413S | |
| | T40414S,T40415S,T40416S,T40421S,T40422S,T40423S,T40424S | |
| | T40425S,T40426S,T40491S,T40492S,T40493S,T40494S,T40495S | |
| | T40496S,T404X1S,T404X2S,T404X3S,T404X4S,T404X5S,T404X6S | |
| | T405X1S,T405X2S,T405X3S,T405X4S,T405X5S,T405X6S,T40601S | |
| | T40602S,T40603S,T40604S,T40605S,T40606S,T40691S,T40692S | |
| | T40693S,T40694S,T40695S,T40696S,T40711S,T40712S,T40713S | |
| | T40714S,T40715S,T40716S,T40721S,T40722S,T40723S,T40724S | |
| | T40725S,T40726S,T407X1S,T407X2S,T407X3S,T407X4S,T407X5S | |
| | T407X6S,T408X1S,T408X2S,T408X3S,T408X4S,T408X5S,T40901S | |
| | T40902S,T40903S,T40904S,T40905S,T40906S,T40991S,T40992S | |
| | T40993S,T40994S,T40995S,T40996S,T410X1S,T410X2S,T410X3S | |
| | T410X4S,T410X5S,T410X6S,T411X1S,T411X2S,T411X3S,T411X4S | |

Continued on next page

Table S9 (continued).

| Diagnosis | ICD-10 | ICD-9 |
|---|---|---|
| | T411X5S,T411X6S,T41201S,T41202S,T41203S,T41204S,T41205S | |
| | T41206S,T41291S,T41292S,T41293S,T41294S,T41295S,T41296S | |
| | T413X1S,T413X2S,T413X3S,T413X4S,T413X5S,T413X6S,T4141XS | |
| | T4142XS,T4143XS,T4144XS,T4145XS,T4146XS,T415X1S,T415X2S | |
| | T415X3S,T415X4S,T415X5S,T415X6S,T420X1S,T420X2S,T420X3S | |
| | T420X4S,T420X5S,T420X6S,T421X1S,T421X2S,T421X3S,T421X4S | |
| | T421X5S,T421X6S,T422X1S,T422X2S,T422X3S,T422X4S,T422X5S | |
| | T422X6S,T423X1S,T423X2S,T423X3S,T423X4S,T423X5S,T423X6S | |
| | T424X1S,T424X2S,T424X3S,T424X4S,T424X5S,T424X6S,T425X1S | |
| | T425X2S,T425X3S,T425X4S,T425X5S,T425X6S,T426X1S,T426X2S | |
| | T426X3S,T426X4S,T426X5S,T426X6S,T4271XS,T4272XS,T4273XS | |
| | T4274XS,T4275XS,T4276XS,T428X1S,T428X2S,T428X3S,T428X4S | |
| | T428X5S,T428X6S,T43011S,T43012S,T43013S,T43014S,T43015S | |
| | T43016S,T43021S,T43022S,T43023S,T43024S,T43025S,T43026S | |
| | T431X1S,T431X2S,T431X3S,T431X4S,T431X5S,T431X6S,T43201S | |
| | T43202S,T43203S,T43204S,T43205S,T43206S,T43211S,T43212S | |
| | T43213S,T43214S,T43215S,T43216S,T43221S,T43222S,T43223S | |
| | T43224S,T43225S,T43226S,T43291S,T43292S,T43293S,T43294S | |
| | T43295S,T43296S,T433X1S,T433X2S,T433X3S,T433X4S,T433X5S | |
| | T433X6S,T434X1S,T434X2S,T434X3S,T434X4S,T434X5S,T434X6S | |
| | T43501S,T43502S,T43503S,T43504S,T43505S,T43506S,T43591S | |
| | T43592S,T43593S,T43594S,T43595S,T43596S,T43601S,T43602S | |
| | T43603S,T43604S,T43605S,T43606S,T43611S,T43612S,T43613S | |
| | T43614S,T43615S,T43616S,T43621S,T43622S,T43623S,T43624S | |
| | T43625S,T43626S,T43631S,T43632S,T43633S,T43634S,T43635S | |
| | T43636S,T43641S,T43642S,T43643S,T43644S,T43651S,T43652S | |
| | T43653S,T43654S,T43655S,T43656S,T43691S,T43692S,T43693S | |
| | T43694S,T43695S,T43696S,T438X1S,T438X2S,T438X3S,T438X4S | |
| | T438X5S,T438X6S,T4391XS,T4392XS,T4393XS,T4394XS,T4395XS | |
| | T4396XS,T440X1S,T440X2S,T440X3S,T440X4S,T440X5S,T440X6S | |
| | T441X1S,T441X2S,T441X3S,T441X4S,T441X5S,T441X6S,T442X1S | |
| | T442X2S,T442X3S,T442X4S,T442X5S,T442X6S,T443X1S,T443X2S | |
| | T443X3S,T443X4S,T443X5S,T443X6S,T444X1S,T444X2S,T444X3S | |
| | T444X4S,T444X5S,T444X6S,T445X1S,T445X2S,T445X3S,T445X4S | |
| | T445X5S,T445X6S,T446X1S,T446X2S,T446X3S,T446X4S,T446X5S | |
| | T446X6S,T447X1S,T447X2S,T447X3S,T447X4S,T447X5S,T447X6S | |
| | T448X1S,T448X2S,T448X3S,T448X4S,T448X5S,T448X6S,T44901S | |
| | T44902S,T44903S,T44904S,T44905S,T44906S,T44991S,T44992S | |
| | T44993S,T44994S,T44995S,T44996S,T450X1S,T450X2S,T450X3S | |
| | T450X4S,T450X5S,T450X6S,T451X1S,T451X2S,T451X3S,T451X4S | |
| | T451X5S,T451X6S,T452X1S,T452X2S,T452X3S,T452X4S,T452X5S | |
| | T452X6S,T453X1S,T453X2S,T453X3S,T453X4S,T453X5S,T453X6S | |
| | T454X1S,T454X2S,T454X3S,T454X4S,T454X5S,T454X6S,T45511S | |
| | T45512S,T45513S,T45514S,T45515S,T45516S,T45521S,T45522S | |
| | T45523S,T45524S,T45525S,T45526S,T45601S,T45602S,T45603S | |
| | T45604S,T45605S,T45606S,T45611S,T45612S,T45613S,T45614S | |
| | T45615S,T45616S,T45621S,T45622S,T45623S,T45624S,T45625S | |
| | T45626S,T45691S,T45692S,T45693S,T45694S,T45695S,T45696S | |
| | T457X1S,T457X2S,T457X3S,T457X4S,T457X5S,T457X6S,T458X1S | |
| | T458X2S,T458X3S,T458X4S,T458X5S,T458X6S,T4591XS,T4592XS | |
| | T4593XS,T4594XS,T4595XS,T4596XS,T460X1S,T460X2S,T460X3S | |
| | T460X4S,T460X5S,T460X6S,T461X1S,T461X2S,T461X3S,T461X4S | |
| | T461X5S,T461X6S,T462X1S,T462X2S,T462X3S,T462X4S,T462X5S | |
| | T462X6S,T463X1S,T463X2S,T463X3S,T463X4S,T463X5S,T463X6S | |
| | T464X1S,T464X2S,T464X3S,T464X4S,T464X5S,T464X6S,T465X1S | |
| | T465X2S,T465X3S,T465X4S,T465X5S,T465X6S,T466X1S,T466X2S | |
| | T466X3S,T466X4S,T466X5S,T466X6S,T467X1S,T467X2S,T467X3S | |
| | T467X4S,T467X5S,T467X6S,T468X1S,T468X2S,T468X3S,T468X4S | |
| | T468X5S,T468X6S,T46901S,T46902S,T46903S,T46904S,T46905S | |
| | T46906S,T46991S,T46992S,T46993S,T46994S,T46995S,T46996S | |
| | T470X1S,T470X2S,T470X3S,T470X4S,T470X5S,T470X6S,T471X1S | |
| | T471X2S,T471X3S,T471X4S,T471X5S,T471X6S,T472X1S,T472X2S | |
| | T472X3S,T472X4S,T472X5S,T472X6S,T473X1S,T473X2S,T473X3S | |
| | T473X4S,T473X5S,T473X6S,T474X1S,T474X2S,T474X3S,T474X4S | |
| | T474X5S,T474X6S,T475X1S,T475X2S,T475X3S,T475X4S,T475X5S | |
| | T475X6S,T476X1S,T476X2S,T476X3S,T476X4S,T476X5S,T476X6S | |
| | T477X1S,T477X2S,T477X3S,T477X4S,T477X5S,T477X6S,T478X1S | |
| | T478X2S,T478X3S,T478X4S,T478X5S,T478X6S,T4791XS,T4792XS | |
| | T4793XS,T4794XS,T4795XS,T4796XS,T480X1S,T480X2S,T480X3S | |
| | T480X4S,T480X5S,T480X6S,T481X1S,T481X2S,T481X3S,T481X4S | |
| | T481X5S,T481X6S,T48201S,T48202S,T48203S,T48204S,T48205S | |
| | T48206S,T48291S,T48292S,T48293S,T48294S,T48295S,T48296S | |
| | T483X1S,T483X2S,T483X3S,T483X4S,T483X5S,T483X6S,T484X1S | |
| | T484X2S,T484X3S,T484X4S,T484X5S,T484X6S,T485X1S,T485X2S | |
| | T485X3S,T485X4S,T485X5S,T485X6S,T486X1S,T486X2S,T486X3S | |
| | T486X4S,T486X5S,T486X6S,T48901S,T48902S,T48903S,T48904S | |
| | T48905S,T48906S,T48991S,T48992S,T48993S,T48994S,T48995S | |
| | T48996S,T490X1S,T490X2S,T490X3S,T490X4S,T490X5S,T490X6S | |
| | T491X1S,T491X2S,T491X3S,T491X4S,T491X5S,T491X6S,T492X1S | |
| | T492X2S,T492X3S,T492X4S,T492X5S,T492X6S,T493X1S,T493X2S | |
| | T493X3S,T493X4S,T493X5S,T493X6S,T494X1S,T494X2S,T494X3S | |
| | T494X4S,T494X5S,T494X6S,T495X1S,T495X2S,T495X3S,T495X4S | |
| | T495X5S,T495X6S,T496X1S,T496X2S,T496X3S,T496X4S,T496X5S | |
| | T496X6S,T497X1S,T497X2S,T497X3S,T497X4S,T497X5S,T497X6S | |
| | T498X1S,T498X2S,T498X3S,T498X4S,T498X5S,T498X6S,T4991XS | |

Continued on next page

Table S9 (continued).

| Diagnosis | ICD-10 | ICD-9 |
|---|---|---|
| | T4992XS,T4993XS,T4994XS,T4995XS,T4996XS,T500X1S,T500X2S | |
| | T500X3S,T500X4S,T500X5S,T500X6S,T501X1S,T501X2S,T501X3S | |
| | T501X4S,T501X5S,T501X6S,T502X1S,T502X2S,T502X3S,T502X4S | |
| | T502X5S,T502X6S,T503X1S,T503X2S,T503X3S,T503X4S,T503X5S | |
| | T503X6S,T504X1S,T504X2S,T504X3S,T504X4S,T504X5S,T504X6S | |
| | T505X1S,T505X2S,T505X3S,T505X4S,T505X5S,T505X6S,T506X1S | |
| | T506X2S,T506X3S,T506X4S,T506X5S,T506X6S,T507X1S,T507X2S | |
| | T507X3S,T507X4S,T507X5S,T507X6S,T508X1S,T508X2S,T508X3S | |
| | T508X4S,T508X5S,T508X6S,T50901S,T50902S,T50903S,T50904S | |
| | T50905S,T50906S,T50911S,T50912S,T50913S,T50914S,T50915S | |
| | T50916S,T50991S,T50992S,T50993S,T50994S,T50995S,T50996S | |
| | T50A11S,T50A12S,T50A13S,T50A14S,T50A15S,T50A16S,T50A21S | |
| | T50A22S,T50A23S,T50A24S,T50A25S,T50A26S,T50A91S,T50A92S | |
| | T50A93S,T50A94S,T50A95S,T50A96S,T50B11S,T50B12S,T50B13S | |
| | T50B14S,T50B15S,T50B16S,T50B91S,T50B92S,T50B93S,T50B94S | |
| | T50B95S,T50B96S,T50Z11S,T50Z12S,T50Z13S,T50Z14S,T50Z15S | |
| | T50Z16S,T50Z91S,T50Z92S,T50Z93S,T50Z94S,T50Z95S,T50Z96S | |
| | T510X1S,T510X2S,T510X3S,T510X4S,T511X1S,T511X2S,T511X3S | |
| | T511X4S,T512X1S,T512X2S,T512X3S,T512X4S,T513X1S,T513X2S | |
| | T513X3S,T513X4S,T518X1S,T518X2S,T518X3S,T518X4S,T5191XS | |
| | T5192XS,T5193XS,T5194XS,T520X1S,T520X2S,T520X3S,T520X4S | |
| | T521X1S,T521X2S,T521X3S,T521X4S,T522X1S,T522X2S,T522X3S | |
| | T522X4S,T523X1S,T523X2S,T523X3S,T523X4S,T524X1S,T524X2S | |
| | T524X3S,T524X4S,T528X1S,T528X2S,T528X3S,T528X4S,T5291XS | |
| | T5292XS,T5293XS,T5294XS,T530X1S,T530X2S,T530X3S,T530X4S | |
| | T531X1S,T531X2S,T531X3S,T531X4S,T532X1S,T532X2S,T532X3S | |
| | T532X4S,T533X1S,T533X2S,T533X3S,T533X4S,T534X1S,T534X2S | |
| | T534X3S,T534X4S,T535X1S,T535X2S,T535X3S,T535X4S,T536X1S | |
| | T536X2S,T536X3S,T536X4S,T537X1S,T537X2S,T537X3S,T537X4S | |
| | T5391XS,T5392XS,T5393XS,T5394XS,T540X1S,T540X2S,T540X3S | |
| | T540X4S,T541X1S,T541X2S,T541X3S,T541X4S,T542X1S,T542X2S | |
| | T542X3S,T542X4S,T543X1S,T543X2S,T543X3S,T543X4S,T5491XS | |
| | T5492XS,T5493XS,T5494XS,T550X1S,T550X2S,T550X3S,T550X4S | |
| | T551X1S,T551X2S,T551X3S,T551X4S,T560X1S,T560X2S,T560X3S | |
| | T560X4S,T561X1S,T561X2S,T561X3S,T561X4S,T562X1S,T562X2S | |
| | T562X3S,T562X4S,T563X1S,T563X2S,T563X3S,T563X4S,T564X1S | |
| | T564X2S,T564X3S,T564X4S,T565X1S,T565X2S,T565X3S,T565X4S | |
| | T566X1S,T566X2S,T566X3S,T566X4S,T567X1S,T567X2S,T567X3S | |
| | T567X4S,T56811S,T56812S,T56813S,T56814S,T56891S,T56892S | |
| | T56893S,T56894S,T5691XS,T5692XS,T5693XS,T5694XS,T570X1S | |
| | T570X2S,T570X3S,T570X4S,T571X1S,T571X2S,T571X3S,T571X4S | |
| | T572X1S,T572X2S,T572X3S,T572X4S,T573X1S,T573X2S,T573X3S | |
| | T573X4S,T578X1S,T578X2S,T578X3S,T578X4S,T5791XS,T5792XS | |
| | T5793XS,T5794XS,T5801XS,T5802XS,T5803XS,T5804XS,T5811XS | |
| | T5812XS,T5813XS,T5814XS,T582X1S,T582X2S,T582X3S,T582X4S | |
| | T588X1S,T588X2S,T588X3S,T588X4S,T5891XS,T5892XS,T5893XS | |
| | T5894XS,T590X1S,T590X2S,T590X3S,T590X4S,T591X1S,T591X2S | |
| | T591X3S,T591X4S,T592X1S,T592X2S,T592X3S,T592X4S,T593X1S | |
| | T593X2S,T593X3S,T593X4S,T594X1S,T594X2S,T594X3S,T594X4S | |
| | T595X1S,T595X2S,T595X3S,T595X4S,T596X1S,T596X2S,T596X3S | |
| | T596X4S,T597X1S,T597X2S,T597X3S,T597X4S,T59811S,T59812S | |
| | T59813S,T59814S,T59891S,T59892S,T59893S,T59894S,T5991XS | |
| | T5992XS,T5993XS,T5994XS,T600X1S,T600X2S,T600X3S,T600X4S | |
| | T601X1S,T601X2S,T601X3S,T601X4S,T602X1S,T602X2S,T602X3S | |
| | T602X4S,T603X1S,T603X2S,T603X3S,T603X4S,T604X1S,T604X2S | |
| | T604X3S,T604X4S,T608X1S,T608X2S,T608X3S,T608X4S,T6091XS | |
| | T6092XS,T6093XS,T6094XS,T6101XS,T6102XS,T6103XS,T6104XS | |
| | T6111XS,T6112XS,T6113XS,T6114XS,T61771S,T61772S,T61773S | |
| | T61774S,T61781S,T61782S,T61783S,T61784S,T618X1S,T618X2S | |
| | T618X3S,T618X4S,T6191XS,T6192XS,T6193XS,T6194XS,T620X1S | |
| | T620X2S,T620X3S,T620X4S,T621X1S,T621X2S,T621X3S,T621X4S | |
| | T622X1S,T622X2S,T622X3S,T622X4S,T628X1S,T628X2S,T628X3S | |
| | T628X4S,T6291XS,T6292XS,T6293XS,T6294XS,T63001S,T63002S | |
| | T63003S,T63004S,T63011S,T63012S,T63013S,T63014S,T63021S | |
| | T63022S,T63023S,T63024S,T63031S,T63032S,T63033S,T63034S | |
| | T63041S,T63042S,T63043S,T63044S,T63061S,T63062S,T63063S | |
| | T63064S,T63071S,T63072S,T63073S,T63074S,T63081S,T63082S | |
| | T63083S,T63084S,T63091S,T63092S,T63093S,T63094S,T63111S | |
| | T63112S,T63113S,T63114S,T63121S,T63122S,T63123S,T63124S | |
| | T63191S,T63192S,T63193S,T63194S,T632X1S,T632X2S,T632X3S | |
| | T632X4S,T63301S,T63302S,T63303S,T63304S,T63311S,T63312S | |
| | T63313S,T63314S,T63321S,T63322S,T63323S,T63324S,T63331S | |
| | T63332S,T63333S,T63334S,T63391S,T63392S,T63393S,T63394S | |
| | T63411S,T63412S,T63413S,T63414S,T63421S,T63422S,T63423S | |
| | T63424S,T63431S,T63432S,T63433S,T63434S,T63441S,T63442S | |
| | T63443S,T63444S,T63451S,T63452S,T63453S,T63454S,T63461S | |
| | T63462S,T63463S,T63464S,T63481S,T63482S,T63483S,T63484S | |
| | T63511S,T63512S,T63513S,T63514S,T63591S,T63592S,T63593S | |
| | T63594S,T63611S,T63612S,T63613S,T63614S,T63621S,T63622S | |
| | T63623S,T63624S,T63631S,T63632S,T63633S,T63634S,T63691S | |
| | T63692S,T63693S,T63694S,T63711S,T63712S,T63713S,T63714S | |
| | T63791S,T63792S,T63793S,T63794S,T63811S,T63812S,T63813S | |
| | T63814S,T63821S,T63822S,T63823S,T63824S,T63831S,T63832S | |
| | T63833S,T63834S,T63891S,T63892S,T63893S,T63894S,T6391XS | |
| | T6392XS,T6393XS,T6394XS,T6401XS,T6402XS,T6403XS,T6404XS | |

Continued on next page

Table S9 (continued).

| Diagnosis | ICD-10 | ICD-9 |
|---|---|---|
| | T6481XS,T6482XS,T6483XS,T6484XS,T650X1S,T650X2S,T650X3S T650X4S,T651X1S,T651X2S,T651X3S,T651X4S,T65211S,T65212S T65213S,T65214S,T65221S,T65222S,T65223S,T65224S,T65291S T65292S,T65293S,T65294S,T653X1S,T653X2S,T653X3S,T653X4S T654X1S,T654X2S,T654X3S,T654X4S,T655X1S,T655X2S,T655X3S T655X4S,T656X1S,T656X2S,T656X3S,T656X4S,T65811S,T65812S T65813S,T65814S,T65821S,T65822S,T65823S,T65824S,T65831S T65832S,T65833S,T65834S,T65891S,T65892S,T65893S,T65894S T6591XS,T6592XS,T6593XS,T6594XS,T788XXS | |
| Polyhydramnios and other problems of amniotic cavity | O401XX0,O401XX1,O401XX2,O401XX3,O401XX4,O401XX5,O401XX9 O402XX0,O402XX1,O402XX2,O402XX3,O402XX4,O402XX5,O402XX9 O403XX0,O403XX1,O403XX2,O403XX3,O403XX4,O403XX5,O403XX9 O409XX0,O409XX1,O409XX2,O409XX3,O409XX4,O409XX5,O409XX9 O4100X0,O4100X1,O4100X2,O4100X3,O4100X4,O4100X5,O4100X9 O4101X0,O4101X1,O4101X2,O4101X3,O4101X4,O4101X5,O4101X9 O4102X0,O4102X1,O4102X2,O4102X3,O4102X4,O4102X5,O4102X9 O4103X0,O4103X1,O4103X2,O4103X3,O4103X4,O4103X5,O4103X9 O418X10,O418X11,O418X12,O418X13,O418X14,O418X15,O418X19 O418X20,O418X21,O418X22,O418X23,O418X24,O418X25,O418X29 O418X30,O418X31,O418X32,O418X33,O418X34,O418X35,O418X39 O418X90,O418X91,O418X92,O418X93,O418X94,O418X95,O418X99 O4190X0,O4190X1,O4190X2,O4190X3,O4190X4,O4190X5,O4190X9 O4191X0,O4191X1,O4191X2,O4191X3,O4191X4,O4191X5,O4191X9 O4192X0,O4192X1,O4192X2,O4192X3,O4192X4,O4192X5,O4192X9 O4193X0,O4193X1,O4193X2,O4193X3,O4193X4,O4193X5,O4193X9 | 65700,65701,65703,65800,65801,65803, 65880,65881,65883,65890 65891,65893 |
| Polyneuropathies | A3683,A5043,A5215,B0223,B2684,B2701,B2711,B2781,B2791,E0842 E0843,E0942,E0943,E1042,E1043,E1142,E1143,E1342,E1343,G600 G601,G602,G603,G608,G609,G610,G611,G6181,G6182,G6189,G619 G620,G621,G622,G6281,G6282,G6289,G629,G63,M0550,M05511,M05512 M05519,M05521,M05522,M05529,M05531,M05532,M05539,M05541 M05542,M05549,M05551,M05552,M05559,M05561,M05562,M05569 M05571,M05572,M05579,M0559,M3483 | 03289,05313,07272,075,09040,09484, 09485,09486,09489,24960 25060,25061,3560,3561,3562,3563,3564, 3568,3569,3570,3571,3572 3574,3575,3576,3577,35781,35782,35789, 3579,5363,7101,7140 |
| Postprocedural or postoperative circulatory system complication | I970,I97110,I97111,I97120,I97121,I97130,I97131,I97190,I97191 I972,I973,I97410,I97411,I97418,I9742,I9751,I9752,I97610 I97611,I97618,I9762,I97620,I97621,I97622,I97630,I97631,I97638 I97640,I97641,I97648,I97710,I97711,I97790,I97791,I97810 I97811,I97820,I97821,I9788,I9789 | 4294,4570,99702,9971,99791,99811,99812, 9982 |
| Postprocedural or postoperative complications of the spleen | D7801,D7802,D7811,D7812,D7821,D7822,D7831,D7832,D7833,D7834 D7881,D7889 | 99799,99811,99812,9982 |
| Postprocedural or postoperative digestive system complication | K910,K911,K912,K913,K9130,K9131,K9132,K915,K9161,K9162,K9171 K9172,K9181,K9182,K9183,K91840,K91841,K91850,K91858,K9186 K91870,K91871,K91872,K91873,K9189,K9400,K9401,K9402,K9403 K9409,K9410,K9411,K9412,K9413,K9419,K9420,K9421,K9422,K9423 K9429,K9430,K9431,K9432,K9433,K9439,K9501,K9509,K9581,K9589 | 53086,53087,53640,53641,53642,53649, 53901,53909,53981,53989 5642,5643,5644,56960,56961,56962,56969, 56971,56979,5760,5793 99741,99749,99811,99812,9982 |
| Postprocedural or postoperative ear and or mastoid process complication | H9500,H9501,H9502,H9503,H95111,H95112,H95113,H95119,H95121 H95122,H95123,H95129,H95131,H95132,H95133,H95139,H95191 H95192,H95193,H95199,H9521,H9522,H9531,H9532,H9541,H9542 H9551,H9552,H9553,H9554,H95811,H95812,H95813,H95819,H9588 H9589 | 38330,38331,38332,38333,99799,99811, 99812,9982 |
| Postprocedural or postoperative endocrine or metabolic complication | E3601,E3602,E3611,E3612,E368,E890,E891,E892,E893,E8940,E8941 E895,E896,E89810,E89811,E89820,E89821,E89822,E89823,E8989 | 2440,2441,2513,2521,2537,25541,2562, 2571,99799,99811,99812 9982,99889 |
| Postprocedural or postoperative eye complication | H59011,H59012,H59013,H59019,H59021,H59022,H59023,H59029 H59031,H59032,H59033,H59039,H59091,H59092,H59093,H59099 H59111,H59112,H59113,H59119,H59121,H59122,H59123,H59129 H59211,H59212,H59213,H59219,H59221,H59222,H59223,H59229 H59311,H59312,H59313,H59319,H59321,H59322,H59323,H59329 H59331,H59332,H59333,H59339,H59341,H59342,H59343,H59349 H59351,H59352,H59353,H59359,H59361,H59362,H59363,H59369,H5940 H5941,H5942,H5943,H59811,H59812,H59813,H59819,H5988,H5989 | 37960,37961,37962,37963,99799,99811, 99812,9982,99882 |
| Postprocedural or postoperative | N980,N981,N982,N983,N988,N989,N990,N99110,N99111,N99112 N99113,N99114,N99115,N99116,N9912,N992,N993,N994,N99510 | 2561,5680,59681,59682,59683,5982,6185, 6208,6232,9975,99799 99811,99812,9982,99939 |

Continued on next page

Table S9 (continued).

| Diagnosis | ICD-10 | ICD-9 |
|---|---|---|
| genitourinary system complication | N99511,N99512,N99518,N99520,N99521,N99522,N99523,N99524 N99528,N99530,N99531,N99532,N99533,N99534,N99538,N9961,N9962 N9971,N9972,N9981,N99820,N99821,N9983,N99840,N99841,N99842 N99843,N9985,N9989 | |
| Postprocedural or postoperative musculoskeletal system complication | M960,M961,M962,M963,M964,M965,M96621,M96622,M96629,M96631 M96632,M96639,M9665,M96661,M96662,M96669,M96671,M96672,M96679 M9669,M96810,M96811,M96820,M96821,M96830,M96831,M96840,M96841 M96842,M96843,M9689 | 72280,72281,72282,72283,73711,73712, 73721,73722,73733,99649 99799,99811,99812,9982,V454 |
| Postprocedural or postoperative nervous system complication | G970,G971,G972,G9731,G9732,G9741,G9748,G9749,G9751,G9752 G9761,G9762,G9763,G9764,G9781,G9782,G9783,G9784 | 3490,3491,34931,99701,99702,99709, 99811,99812,9982 |
| Postprocedural or postoperative respiratory system complication | J9500,J9501,J9502,J9503,J9504,J9509,J951,J952,J953,J954,J955 J9561,J9562,J9571,J9572,J95811,J95812,J95821,J95822,J95830 J95831,J9584,J95850,J95851,J95859,J95860,J95861,J95862,J95863 J9587,J9588,J9589 | 5121,5122,51851,51852,51853,5187,51900, 51901,51902,51909 99731,99732,99739,99811,99812,9982, V4614 |
| Postprocedural or postoperative skin complication | L7601,L7602,L7611,L7612,L7621,L7622,L7631,L7632,L7633,L7634 L7681,L7682 | 99811,99812,9982,99889 |
| Postthrombotic syndrome and venous insufficiency hypertension | I868,I87001,I87002,I87003,I87009,I87011,I87012,I87013,I87019 I87021,I87022,I87023,I87029,I87031,I87032,I87033,I87039 I87091,I87092,I87093,I87099,I871,I872,I87301,I87302,I87303 I87309,I87311,I87312,I87313,I87319,I87321,I87322,I87323 I87329,I87331,I87332,I87333,I87339,I87391,I87392,I87393 I87399 | 4568,45910,45911,45912,45913,45919, 4592,45930,45931,45932 45933,45939,45981 |
| Pressure ulcer of skin | E08621,E08622,E09621,E09622,E10621,E10622,E11621,E11622 E13621,E13622,L89000,L89001,L89002,L89003,L89004,L89006 L89009,L89010,L89011,L89012,L89013,L89014,L89016,L89019 L89020,L89021,L89022,L89023,L89024,L89026,L89029,L89100 L89101,L89102,L89103,L89104,L89106,L89109,L89110,L89111 L89112,L89113,L89114,L89116,L89119,L89120,L89121,L89122 L89123,L89124,L89126,L89129,L89130,L89131,L89132,L89133 L89134,L89136,L89139,L89140,L89141,L89142,L89143,L89144 L89146,L89149,L89150,L89151,L89152,L89153,L89154,L89156 L89159,L89200,L89201,L89202,L89203,L89204,L89206,L89209 L89210,L89211,L89212,L89213,L89214,L89216,L89219,L89220 L89221,L89222,L89223,L89224,L89226,L89229,L89300,L89301 L89302,L89303,L89304,L89306,L89309,L89310,L89311,L89312 L89313,L89314,L89316,L89319,L89320,L89321,L89322,L89323 L89324,L89326,L89329,L8940,L8941,L8942,L8943,L8944,L8945 L8946,L89500,L89501,L89502,L89503,L89504,L89506,L89509,L89510 L89511,L89512,L89513,L89514,L89516,L89519,L89520,L89521 L89522,L89523,L89524,L89526,L89529,L89600,L89601,L89602 L89603,L89604,L89606,L89609,L89610,L89611,L89612,L89613 L89614,L89616,L89619,L89620,L89621,L89622,L89623,L89624 L89626,L89629,L89810,L89811,L89812,L89813,L89814,L89816 L89819,L89890,L89891,L89892,L89893,L89894,L89896,L89899,L8990 L8991,L8992,L8993,L8994,L8995,L8996 | 24980,25080,25081,70700,70701,70702, 70703,70704,70705,70706 70707,70709,70720,70721,70722,70723, 70724,70725 |
| Previous C-section | O3421,O34211,O34212,O34218,O34219,O3422 | 65421,65423 |
| Prolapse of female genital organs | N810,N8110,N8111,N8112,N812,N813,N814,N815,N816,N8181,N8182 N8183,N8184,N8185,N8189,N819 | 61800,61801,61802,61803,61804,61805, 6182,6183,6184,6186,6187 61881,61882,61883,61884,61889,6189 |
| Prolonged pregnancy | O480,O481 | 64511,64513,64523 |
| Proteinuria | N060,N061,N062,N063,N064,N065,N066,N067,N068,N069,N06A,O1210 O1211,O1212,O1213,O1214,O1215,O1220,O1221,O1222,O1223,O1224 O1225,R800,R801,R802,R803,R808,R809 | 5831,5832,58389,5839,5936,64610,64611, 64613,64620,64621,64623 7910 |
| Pulmonary heart disease | I270,I271,I272,I2720,I2721,I2722,I2723,I2724,I2729,I2781 I2782,I2783,I2789,I279,I280,I281,I288,I289 | 4160,4161,4162,4168,4169,4170,4171, 4178,4179 |
| Refractive error | H5200,H5201,H5202,H5203,H5210,H5211,H5212,H5213,H52201,H52202 H52203,H52209,H52211,H52212,H52213,H52219,H52221,H52222 H52223,H52229,H5231,H5232,H524,H52511,H52512,H52513,H52519 H52521,H52522,H52523,H52529,H52531,H52532,H52533,H52539,H526 H527 | 3670,3671,36720,36721,36722,36731, 36732,3674,36751,36752 36753,36789,3679 |

Continued on next page

Table S9 (continued).

| Diagnosis | ICD-10 | ICD-9 |
|---|---|---|
| Regional enteritis and ulcerative colitis | K5000,K50011,K50012,K50013,K50014,K50018,K50019,K5010,K50111 K50112,K50113,K50114,K50118,K50119,K5080,K50811,K50812,K50813 K50814,K50818,K50819,K5090,K50911,K50912,K50913,K50914,K50918 K50919,K5100,K51011,K51012,K51013,K51014,K51018,K51019,K5120 K51211,K51212,K51213,K51214,K51218,K51219,K5130,K51311,K51312 K51313,K51314,K51318,K51319,K5140,K51411,K51412,K51413,K51414 K51418,K51419,K5150,K51511,K51512,K51513,K51514,K51518,K51519 K5180,K51811,K51812,K51813,K51814,K51818,K51819,K5190,K51911 K51912,K51913,K51914,K51918,K51919 | 5550,5551,5552,5559,5562,5563,5564, 5565,5566,5568,5569,56089 5609,566,5695,56981 |
| Respiratory cancers | C33,C3400,C3401,C3402,C3410,C3411,C3412,C342,C3430,C3431 C3432,C3480,C3481,C3482,C3490,C3491,C3492,C384,C390,C399,D021 D0220,D0221,D0222,D023,D024 | 1620,1622,1623,1624,1625,1628,1629, 1639,1650,1658,1659,2311 2312,2318,2319 |
| Respiratory congenital malformations | Q300,Q301,Q302,Q303,Q308,Q309,Q310,Q311,Q312,Q313,Q315,Q318 Q319,Q320,Q321,Q322,Q323,Q324,Q330,Q331,Q332,Q333,Q334,Q335 Q336,Q338,Q339,Q340,Q341,Q348,Q349 | 7480,7481,7482,7483,7484,7485,74860, 74861,74869,7488,7489 |
| Respiratory distress syndrome | P220,P228,P229 | 769,77089 |
| Respiratory failure; insufficiency; arrest | J80,J95821,J95822,J9600,J9601,J9602,J9610,J9611,J9612,J9620 J9621,J9622,J9690,J9691,J9692,R092 | 51851,51853,51881,51882,51883,51884, 7991 |
| Respiratory perinatal condition | P230,P231,P232,P233,P234,P235,P236,P238,P239,P2400,P2401 P2410,P2411,P2420,P2421,P2430,P2431,P2480,P2481,P249,P250 P251,P252,P253,P258,P260,P261,P268,P269,P270,P271,P278,P279 P280,P2810,P2811,P2819,P282,P283,P2830,P2831,P2832,P2833 P2839,P284,P2840,P2841,P2842,P2843,P2849,P285,P2881,P2889 P289 | 7700,77010,77011,77012,77013,77014, 77015,77016,77017,77018 7702,7703,7704,7705,7707,77081,77082, 77083,77084,77085,77086 77087,77089,7709 |
| Respiratory signs and symptoms | R040,R041,R042,R0481,R0489,R049,R05,R051,R052,R053,R054,R058 R059,R0600,R0601,R0602,R0603,R0609,R061,R062,R063,R064,R065 R066,R067,R0681,R0682,R0683,R0689,R069,R070,R071,R0781,R0782 R0901,R0902,R093,R0981,R0982 | 47819,7841,7847,7848,78491,78499,78600, 78601,78602,78603 78604,78605,78606,78607,78609,7861, 7862,78630,78631,78639 7864,78652,78659,7868,7869,79901,79902 |
| Retinal and vitreous conditions | A1853,A5032,B5801,E08311,E08319,E08321,E083211,E083212 E083213,E083219,E08329,E083291,E083292,E083293,E083299,E08331 E083311,E083312,E083313,E083319,E08339,E083391,E083392 E083393,E083399,E08341,E083411,E083412,E083413,E083419,E08349 E083491,E083492,E083493,E083499,E08351,E083511,E083512 E083513,E083519,E083521,E083522,E083523,E083529,E083531 E083532,E083533,E083539,E083541,E083542,E083543,E083549 E083551,E083552,E083553,E083559,E08359,E083591,E083592 E083593,E083599,E09311,E09319,E09321,E093211,E093212,E093213 E093219,E09329,E093291,E093292,E093293,E093299,E09331,E093311 E093312,E093313,E093319,E09339,E093391,E093392,E093393 E093399,E09341,E093411,E093412,E093413,E093419,E09349,E093491 E093492,E093493,E093499,E09351,E093511,E093512,E093513 E093519,E093521,E093522,E093523,E093529,E093531,E093532 E093533,E093539,E093541,E093542,E093543,E093549,E093551 E093552,E093553,E093559,E09359,E093591,E093592,E093593 E093599,E10311,E10319,E10321,E103211,E103212,E103213,E103219 E10329,E103291,E103292,E103293,E103299,E10331,E103311,E103312 E103313,E103319,E10339,E103391,E103392,E103393,E103399,E10341 E103411,E103412,E103413,E103419,E10349,E103491,E103492 E103493,E103499,E10351,E103511,E103512,E103513,E103519 E103521,E103522,E103523,E103529,E103531,E103532,E103533 E103539,E103541,E103542,E103543,E103549,E103551,E103552 E103553,E103559,E10359,E103591,E103592,E103593,E103599,E11311 E11319,E11321,E113211,E113212,E113213,E113219,E11329,E113291 E113292,E113293,E113299,E11331,E113311,E113312,E113313 E113319,E11339,E113391,E113392,E113393,E113399,E11341,E113411 E113412,E113413,E113419,E11349,E113491,E113492,E113493 E113499,E11351,E113511,E113512,E113513,E113519,E113521 | 01730,0905,1302,24950,25050,25051, 36013,36100,36101,36102 36103,36104,36105,36107,36110,36111, 36112,36113,36114,36119 3612,36130,36131,36132,36133,36181, 36189,36201,36202,36204 36205,36206,36207,36210,36211,36212, 36213,36214,36215,36216 36217,36218,36220,36221,36222,36223, 36224,36225,36226,36227 36229,36230,36231,36232,36233,36234, 36235,36236,36237,36240 36241,36242,36243,36250,36251,36252, 36253,36254,36255,36256 36257,36260,36261,36262,36263,36264, 36265,36266,36270,36271 36272,36273,36274,36275,36276,36277, 36281,36282,36283,36284 36285,36289,3629,36300,36301,36303, 36304,36305,36306,36307 36308,36310,36311,36312,36313,36314, 36315,36320,36321,36322 36330,36331,36332,36333,36334,36335, 36340,36341,36342,36343 36350,36353,36354,36355,36356,36357, 36361,36362,36363,36370 36371,36372,3638,3639,37913,37921, 37922,37923,37924,37925 37926,37927,37929 |

Continued on next page

Table S9 (continued).

| Diagnosis | ICD-10 | ICD-9 |
|---|---|---|
| | E113522,E113523,E113529,E113531,E113532,E113533,E113539 | |
| | E113541,E113542,E113543,E113549,E113551,E113552,E113553 | |
| | E113559,E11359,E113591,E113592,E113593,E113599,E13311,E13319 | |
| | E13321,E133211,E133212,E133213,E133219,E13329,E133291,E133292 | |
| | E133293,E133299,E13331,E133311,E133312,E133313,E133319,E13339 | |
| | E133391,E133392,E133393,E133399,E13341,E133411,E133412 | |
| | E133413,E133419,E13349,E133491,E133492,E133493,E133499,E13351 | |
| | E133511,E133512,E133513,E133519,E133521,E133522,E133523 | |
| | E133529,E133531,E133532,E133533,E133539,E133541,E133542 | |
| | E133543,E133549,E133551,E133552,E133553,E133559,E13359 | |
| | E133591,E133592,E133593,E133599,H15811,H15812,H15813,H15819 | |
| | H30001,H30002,H30003,H30009,H30011,H30012,H30013,H30019 | |
| | H30021,H30022,H30023,H30029,H30031,H30032,H30033,H30039 | |
| | H30041,H30042,H30043,H30049,H30101,H30102,H30103,H30109 | |
| | H30111,H30112,H30113,H30119,H30121,H30122,H30123,H30129 | |
| | H30131,H30132,H30133,H30139,H30141,H30142,H30143,H30149,H3020 | |
| | H3021,H3022,H3023,H30811,H30812,H30813,H30819,H30891,H30892 | |
| | H30893,H30899,H3090,H3091,H3092,H3093,H31001,H31002,H31003 | |
| | H31009,H31011,H31012,H31013,H31019,H31021,H31022,H31023 | |
| | H31029,H31091,H31092,H31093,H31099,H31101,H31102,H31103 | |
| | H31109,H31111,H31112,H31113,H31119,H31121,H31122,H31123 | |
| | H31129,H3120,H3121,H3122,H3123,H3129,H31301,H31302,H31303 | |
| | H31309,H31311,H31312,H31313,H31319,H31321,H31322,H31323 | |
| | H31329,H31401,H31402,H31403,H31409,H31411,H31412,H31413 | |
| | H31419,H31421,H31422,H31423,H31429,H318,H319,H32,H33001 | |
| | H33002,H33003,H33009,H33011,H33012,H33013,H33019,H33021 | |
| | H33022,H33023,H33029,H33031,H33032,H33033,H33039,H33041 | |
| | H33042,H33043,H33049,H33051,H33052,H33053,H33059,H33101 | |
| | H33102,H33103,H33109,H33111,H33112,H33113,H33119,H33121 | |
| | H33122,H33123,H33129,H33191,H33192,H33193,H33199,H3320,H3321 | |
| | H3322,H3323,H33301,H33302,H33303,H33309,H33311,H33312,H33313 | |
| | H33319,H33321,H33322,H33323,H33329,H33331,H33332,H33333 | |
| | H33339,H3340,H3341,H3342,H3343,H338,H3400,H3401,H3402,H3403 | |
| | H3410,H3411,H3412,H3413,H34211,H34212,H34213,H34219,H34231 | |
| | H34232,H34233,H34239,H34811,H348110,H348111,H348112,H34812 | |
| | H348120,H348121,H348122,H34813,H348130,H348131,H348132,H34819 | |
| | H348190,H348191,H348192,H34821,H34822,H34823,H34829,H34831 | |
| | H348310,H348311,H348312,H34832,H348320,H348321,H348322,H34833 | |
| | H348330,H348331,H348332,H34839,H348390,H348391,H348392,H349 | |
| | H3500,H35011,H35012,H35013,H35019,H35021,H35022,H35023,H35029 | |
| | H35031,H35032,H35033,H35039,H35041,H35042,H35043,H35049 | |
| | H35051,H35052,H35053,H35059,H35061,H35062,H35063,H35069 | |
| | H35071,H35072,H35073,H35079,H3509,H35101,H35102,H35103,H35109 | |
| | H35111,H35112,H35113,H35119,H35121,H35122,H35123,H35129 | |
| | H35131,H35132,H35133,H35139,H35141,H35142,H35143,H35149 | |
| | H35151,H35152,H35153,H35159,H35161,H35162,H35163,H35169 | |
| | H35171,H35172,H35173,H35179,H3520,H3521,H3522,H3523,H3530 | |
| | H3531,H353110,H353111,H353112,H353113,H353114,H353120,H353121 | |
| | H353122,H353123,H353124,H353130,H353131,H353132,H353133 | |
| | H353134,H353190,H353191,H353192,H353193,H353194,H3532,H353210 | |
| | H353211,H353212,H353213,H353220,H353221,H353222,H353223 | |
| | H353230,H353231,H353232,H353233,H353290,H353291,H353292 | |
| | H353293,H3533,H35341,H35342,H35343,H35349,H35351,H35352 | |
| | H35353,H35359,H35361,H35362,H35363,H35369,H35371,H35372 | |
| | H35373,H35379,H35381,H35382,H35383,H35389,H3540,H35411,H35412 | |
| | H35413,H35419,H35421,H35422,H35423,H35429,H35431,H35432 | |
| | H35433,H35439,H35441,H35442,H35443,H35449,H35451,H35452 | |
| | H35453,H35459,H35461,H35462,H35463,H35469,H3550,H3551,H3552 | |
| | H3553,H3554,H3560,H3561,H3562,H3563,H3570,H35711,H35712 | |
| | H35713,H35719,H35721,H35722,H35723,H35729,H35731,H35732 | |
| | H35733,H35739,H3581,H3582,H3589,H359,H36,H4300,H4301,H4302 | |
| | H4303,H4310,H4311,H4312,H4313,H4320,H4321,H4322,H4323,H43311 | |
| | H43312,H43313,H43319,H43391,H43392,H43393,H43399,H43811 | |
| | H43812,H43813,H43819,H43821,H43822,H43823,H43829,H4389,H439 | |
| Rheumatoid arthritis | M0500,M05011,M05012,M05019,M05021,M05022,M05029,M05031,M05032 | 3571,3596,7140,7141,7142,71481,71489, 7149,7200 |
| and related disease | M05039,M05041,M05042,M05049,M05051,M05052,M05059,M05061 | |
| | M05062,M05069,M05071,M05072,M05079,M0509,M0510,M05111,M05112 | |
| | M05119,M05121,M05122,M05129,M05131,M05132,M05139,M05141 | |
| | M05142,M05149,M05151,M05152,M05159,M05161,M05162,M05169 | |
| | M05171,M05172,M05179,M0519,M0520,M05211,M05212,M05219,M05221 | |
| | M05222,M05229,M05231,M05232,M05239,M05241,M05242,M05249 | |
| | M05251,M05252,M05259,M05261,M05262,M05269,M05271,M05272 | |
| | M05279,M0529,M0530,M05311,M05312,M05319,M05321,M05322,M05329 | |
| | M05331,M05332,M05339,M05341,M05342,M05349,M05351,M05352 | |
| | M05359,M05361,M05362,M05369,M05371,M05372,M05379,M0539,M0540 | |
| | M05411,M05412,M05419,M05421,M05422,M05429,M05431,M05432 | |
| | M05439,M05441,M05442,M05449,M05451,M05452,M05459,M05461 | |
| | M05462,M05469,M05471,M05472,M05479,M0549,M0550,M05511,M05512 | |
| | M05519,M05521,M05522,M05529,M05531,M05532,M05539,M05541 | |
| | M05542,M05549,M05551,M05552,M05559,M05561,M05562,M05569 | |
| | M05571,M05572,M05579,M0559,M0560,M05611,M05612,M05619,M05621 | |
| | M05622,M05629,M05631,M05632,M05639,M05641,M05642,M05649 | |
| | M05651,M05652,M05659,M05661,M05662,M05669,M05671,M05672 | |

Continued on next page

**Table S9 (continued).**

| Diagnosis | ICD-10 | ICD-9 |
|---|---|---|
| | M05679,M0569,M0570,M05711,M05712,M05719,M05721,M05722,M05729<br>M05731,M05732,M05739,M05741,M05742,M05749,M05751,M05752<br>M05759,M05761,M05762,M05769,M05771,M05772,M05779,M0579,M057A<br>M0580,M05811,M05812,M05819,M05821,M05822,M05829,M05831,M05832<br>M05839,M05841,M05842,M05849,M05851,M05852,M05859,M05861<br>M05862,M05869,M05871,M05872,M05879,M0589,M058A,M059,M0600<br>M06011,M06012,M06019,M06021,M06022,M06029,M06031,M06032<br>M06039,M06041,M06042,M06049,M06051,M06052,M06059,M06061<br>M06062,M06069,M06071,M06072,M06079,M0608,M0609,M060A,M061<br>M0620,M06211,M06212,M06219,M06221,M06222,M06229,M06231,M06232<br>M06239,M06241,M06242,M06249,M06251,M06252,M06259,M06261<br>M06262,M06269,M06271,M06272,M06279,M0628,M0629,M0630,M06311<br>M06312,M06319,M06321,M06322,M06329,M06331,M06332,M06339<br>M06341,M06342,M06349,M06351,M06352,M06359,M06361,M06362<br>M06369,M06371,M06372,M06379,M0638,M0639,M064,M0680,M06811<br>M06812,M06819,M06821,M06822,M06829,M06831,M06832,M06839<br>M06841,M06842,M06849,M06851,M06852,M06859,M06861,M06862<br>M06869,M06871,M06872,M06879,M0688,M0689,M068A,M069,M450,M451<br>M452,M453,M454,M455,M456,M457,M458,M459,M45A0,M45A1,M45A2<br>M45A3,M45A4,M45A5,M45A6,M45A7,M45A8,M45AB,M488X1,M488X2<br>M488X3,M488X4,M488X5,M488X6,M488X7,M488X8,M488X9 | |
| Sarcoma | C460,C461,C462,C463,C464,C4650,C4651,C4652,C467,C469,C490<br>C4910,C4911,C4912,C4920,C4921,C4922,C493,C494,C495,C496,C498<br>C499,C9230,C9231,C9232,C9622,C964,C96A | 1710,1712,1713,1714,1715,1716,1717,<br>1718,1719,1760,1761,1762<br>1763,1764,1765,1768,1769,20230,20290,<br>20530,20531,20532 |
| Schizophrenia spectrum and other psychotic disorders | F060,F061,F062,F10150,F10151,F10159,F10250,F10251,F10259<br>F10950,F10951,F10959,F11150,F11151,F11159,F11250,F11251<br>F11259,F11950,F11951,F11959,F12150,F12151,F12159,F12250<br>F12251,F12259,F12950,F12951,F12959,F13150,F13151,F13159<br>F13250,F13251,F13259,F13950,F13951,F13959,F14150,F14151<br>F14159,F14250,F14251,F14259,F14950,F14951,F14959,F15150<br>F15151,F15159,F15250,F15251,F15259,F15950,F15951,F15959<br>F16150,F16151,F16159,F16250,F16251,F16259,F16950,F16951<br>F16959,F18150,F18151,F18159,F18250,F18251,F18259,F18950<br>F18951,F18959,F19150,F19151,F19159,F19250,F19251,F19259<br>F19950,F19951,F19959,F200,F201,F202,F203,F205,F2081,F2089<br>F209,F21,F22,F23,F24,F250,F251,F258,F259,F28,F29,F53,F531 | 2913,2915,29189,29211,29212,29289,<br>29381,29382,29389,2948<br>29510,29520,29530,29540,29560,29570,<br>29580,29590,2970,2971<br>2972,2973,2983,2984,2988,2989,30122,<br>30400,30410,30420,30430<br>30440,30450,30460 |
| Scoliosis and other postural dorsopathic deformities | M4000,M4003,M4004,M4005,M4010,M4012,M4013,M4014,M4015,M40202<br>M40203,M40204,M40205,M40209,M40292,M40293,M40294,M40295<br>M40299,M4030,M4035,M4036,M4037,M4040,M4045,M4046,M4047,M4050<br>M4055,M4056,M4057,M4100,M4102,M4103,M4104,M4105,M4106,M4107<br>M4108,M41112,M41113,M41114,M41115,M41116,M41117,M41119,M41122<br>M41123,M41124,M41125,M41126,M41127,M41129,M4120,M4122,M4123<br>M4124,M4125,M4126,M4127,M4130,M4134,M4135,M4140,M4141,M4142<br>M4143,M4144,M4145,M4146,M4147,M4150,M4152,M4153,M4154,M4155<br>M4156,M4157,M4180,M4182,M4183,M4184,M4185,M4186,M4187,M419<br>M4200,M4201,M4202,M4203,M4204,M4205,M4206,M4207,M4208,M4209<br>M4210,M4211,M4212,M4213,M4214,M4215,M4216,M4217,M4218,M4219<br>M429,M962,M963,M964,M965 | 7320,7328,7329,7370,73710,73711,73712,<br>73719,73720,73721,73722<br>73729,73730,73731,73732,73733,73734,<br>73739,73741,73742,73743 |
| Screening for neurocognitive or neurodevelopmental condition | Z134,Z1340,Z1341,Z1342,Z1349 | V792,V793,V798 |
| Secondary malignancies | C770,C771,C772,C773,C774,C775,C778,C779,C7800,C7801,C7802<br>C781,C782,C7830,C7839,C784,C785,C786,C787,C7880,C7889,C7900<br>C7901,C7902,C7910,C7911,C7919,C792,C7931,C7932,C7940,C7949<br>C7951,C7952,C7960,C7961,C7962,C7963,C7970,C7971,C7972,C7981<br>C7982,C7989,C799 | 1960,1961,1962,1963,1965,1966,1968,<br>1969,1970,1971,1972,1973<br>1974,1975,1976,1977,1978,1980,1981,<br>1982,1983,1984,1985,1986<br>1987,19881,19882,19889 |
| Sedative-related disorders | F1310,F13120,F13121,F13129,F13130,F13131,F13132,F13139,F1314<br>F13150,F13151,F13159,F13180,F13181,F13182,F13188,F1319,F1320<br>F13220,F13221,F13229,F13230,F13231,F13232,F13239,F1324,F13250<br>F13251,F13259,F1326,F1327,F13280,F13281,F13282,F13288,F1329<br>F1390,F1391,F13920,F13921,F13929,F13930,F13931,F13932,F13939<br>F1394,F13950,F13951,F13959,F1396,F1397,F13980,F13981,F13982<br>F13988,F1399,T426X1A,T426X2A,T426X3A,T426X4A,T426X5A,T4271XA<br>T4272XA,T4273XA,T4274XA,T4275XA | 2920,29211,29212,2922,29281,29282,<br>29283,29284,29285,29289<br>2929,30410,30411,30412,30540,30541,<br>30542,30543,9663,9678,9679<br>99529,E8528,E8588,E9363,E9378,E9379,<br>E9502,E9503,E9504,E9620<br>E9804 |
| Septicemia | A021,A207,A227,A267,A327,A392,A393,A394,A400,A401,A403,A408 | 0031,0202,0223,0270,0271,0362,0380,<br>03810,03811,03812,03819 |

Continued on next page

Table S9 (continued).

| Diagnosis | ICD-10 | ICD-9 |
|---|---|---|
| | A409,A4101,A4102,A411,A412,A413,A414,A4150,A4151,A4152,A4153 | 0382,0383,03840,03841,03842,03843, 03844,03849,0388,0389,0545 |
| | A4159,A4181,A4189,A419,A427,A5486,B007,B377,I76,O0337,O0387 | 09889,1125,449,63400,63401,63402,63502, 6380,6390,67022,67024 |
| | O0487,O0737,O0882,O85,O8604,P360,P3610,P3619,P362,P3630,P3639 P364,P365,P368,P369,R6520,R6521,T8112XA,T8144XA | 77181,78552,99591,99592,99802 |
| Sequela of cerebral | I6930,I6931,I69310,I69311,I69312,I69313,I69314,I69315,I69318 | 4380,43810,43811,43812,43813,43814, 43819,43820,43821,43822 |
| infarction and other | I69319,I69320,I69321,I69322,I69323,I69328,I69331,I69332 | 43830,43831,43832,43840,43841,43842, 43850,43851,43852,43853 |
| cerebrovascular | I69333,I69334,I69339,I69341,I69342,I69343,I69344,I69349 | 4386,4387,43881,43882,43883,43884, 43889,4389 |
| disease | I69351,I69352,I69353,I69354,I69359,I69361,I69362,I69363 I69364,I69365,I69369,I69390,I69391,I69392,I69393,I69398,I6980 I6981,I69810,I69811,I69812,I69813,I69814,I69815,I69818,I69819 I69820,I69821,I69822,I69823,I69828,I69831,I69832,I69833 I69834,I69839,I69841,I69842,I69843,I69844,I69849,I69851 I69852,I69853,I69854,I69859,I69861,I69862,I69863,I69864 I69865,I69869,I69890,I69891,I69892,I69893,I69898,I6990,I6991 I69910,I69911,I69912,I69913,I69914,I69915,I69918,I69919 I69920,I69921,I69922,I69923,I69928,I69931,I69932,I69933 I69934,I69939,I69941,I69942,I69943,I69944,I69949,I69951 I69952,I69953,I69954,I69959,I69961,I69962,I69963,I69964 I69965,I69969,I69990,I69991,I69992,I69993,I69998 | |
| Sequela of | I6900,I6901,I69010,I69011,I69012,I69013,I69014,I69015,I69018 | 4380,43811,43812,43813,43814,43819, 43820,43821,43822,43830 |
| hemorrhagic | I69019,I69020,I69021,I69022,I69023,I69028,I69031,I69032 | 43831,43832,43840,43841,43842,43850, 43851,43852,43853,43881 |
| cerebrovascular disease | I69033,I69034,I69039,I69041,I69042,I69043,I69044,I69049 I69051,I69052,I69053,I69054,I69059,I69061,I69062,I69063 I69064,I69065,I69069,I69090,I69091,I69092,I69093,I69098,I6910 I6911,I69110,I69111,I69112,I69113,I69114,I69115,I69118,I69119 I69120,I69121,I69122,I69123,I69128,I69131,I69132,I69133 I69134,I69139,I69141,I69142,I69143,I69144,I69149,I69151 I69152,I69153,I69154,I69159,I69161,I69162,I69163,I69164 I69165,I69169,I69190,I69191,I69192,I69193,I69198,I6920,I6921 I69210,I69211,I69212,I69213,I69214,I69215,I69218,I69219 I69220,I69221,I69222,I69223,I69228,I69231,I69232,I69233 I69234,I69239,I69241,I69242,I69243,I69244,I69249,I69251 I69252,I69253,I69254,I69259,I69261,I69262,I69263,I69264 I69265,I69269,I69290,I69291,I69292,I69293,I69298 | 43882,43883,43884,43889,4389 |
| Sequela of malnutrition and other nutritional deficiencies | E640,E641,E642,E643,E648,E649,E68 | 2639,2649,267,2681,2698,2699,2788 |
| Sequela of specified | B900,B901,B902,B908,B909,B91,B92,B940,B941,B942,B948,B949 | 1370,1371,1372,1373,1374,138,1390,1391, 1398 |
| infectious disease conditions | | |
| Sequela of specified nervous system conditions | B91,B941,G09,G650,G651,G652 | 138,1390,326,3574 |
| Sexually transmitted | A5001,A5002,A5003,A5004,A5005,A5006,A5007,A5008,A5009,A501 | 05410,05411,05412,05413,05419,07811, 0900,0901,0902,0903,09040 |
| infections (excluding | A502,A5030,A5031,A5032,A5039,A5040,A5041,A5042,A5043,A5044 | 09041,09042,09049,0905,0906,0907,0909, 0910,0911,0912,0913 |
| HIV and hepatitis) | A5045,A5049,A5051,A5052,A5053,A5054,A5055,A5056,A5057,A5059 | 0914,09150,09151,09152,09161,09162, 09169,0917,09181,09182 |
| | A506,A507,A509,A510,A511,A512,A5131,A5132,A5139,A5141,A5142 | 09189,0920,0929,0930,0931,09320,09321, 09322,09323,09324,09381 |
| | A5143,A5144,A5145,A5146,A5149,A515,A519,A5200,A5201,A5202 | 09382,09389,0939,0940,0941,0942,0943, 09481,09482,09483,09484 |
| | A5203,A5204,A5205,A5206,A5209,A5210,A5211,A5212,A5213,A5214 | 09485,09486,09489,0949,0950,0951,0952, 0953,0954,0955,0956 |
| | A5215,A5216,A5217,A5219,A522,A523,A5271,A5272,A5273,A5274 | 0957,0958,0959,096,0970,0971,0979,0980, 09811,09812,09813 |
| | A5275,A5276,A5277,A5278,A5279,A528,A529,A530,A539,A5400,A5401 | 09814,09815,09816,09819,0982,09831, 09832,09833,09834,09835 |
| | A5402,A5403,A5409,A541,A5421,A5422,A5423,A5424,A5429,A5430 | 09836,09840,09841,09842,09843,09849, 09850,09851,09852,09853 |
| | A5431,A5432,A5433,A5439,A5440,A5441,A5442,A5443,A5449,A545 | 09859,0986,0987,09881,09882,09883, 09884,09885,09886,09889 |
| | A546,A5481,A5482,A5483,A5484,A5485,A5486,A5489,A549,A55,A5600 | 0990,0991,0992,09951,09952,09953,09954, 09955,09959,0998,0999 |
| | A5601,A5602,A5609,A5611,A5619,A562,A563,A564,A568,A57,A58 | 13100,13101,13102,13103,13109,1318, 1319,3574,5954,6149,61611 |
| | A5900,A5901,A5902,A5903,A5909,A598,A599,A6000,A6001,A6002 | 64700,64701,64702,64703,64704,64710, 64711,64712,64713,64714 |
| | A6003,A6004,A6009,A601,A609,A630,A638,A64,O98111,O98112 | 64720,64721,64722,64723,64724,7135, 99591 |
| | O98113,O98119,O9812,O9813,O98211,O98212,O98213,O98219,O9822 | |

Continued on next page

Table S9 (continued).

| Diagnosis | ICD-10 | ICD-9 |
|---|---|---|
| | O9823,O98311,O98312,O98313,O98319,O9832,O9833 | |
| Shock | O0331,O0381,O0481,O0731,O083,O751,R570,R571,R578,R579,R6521 | 63450,63451,63452,63552,6385,6395, 66911,66912,66914,78550 |
| | T8110XA,T8111XA,T8112XA,T8119XA,T882XXA | 78551,78552,78559,9954,99592,99800, 99801,99802,99809 |
| Short gestation; low | P0500,P0501,P0502,P0503,P0504,P0505,P0506,P0507,P0508,P0509 | 76400,76401,76402,76403,76404,76405, 76406,76407,76408,76410 |
| birth weight; and | P0510,P0511,P0512,P0513,P0514,P0515,P0516,P0517,P0518,P0519 | 76411,76412,76413,76414,76415,76416, 76417,76418,76420,76490 |
| fetal growth | P052,P059,P0700,P0701,P0702,P0703,P0710,P0714,P0715,P0716 | 76500,76501,76502,76503,76504,76505, 76506,76507,76508,76509 |
| retardation | P0717,P0718,P0720,P0721,P0722,P0723,P0724,P0725,P0726,P0730 | 76510,76511,76512,76513,76514,76515, 76516,76517,76518,76519 |
| | P0731,P0732,P0733,P0734,P0735,P0736,P0737,P0738,P0739 | 76520,76521,76522,76523,76524,76525, 76526,76527,76528 |
| Sickle cell trait | D5700,D5701,D5702,D5703,D5709,D571,D5720,D57211,D57212,D57213 | 28241,28242,2825,28260,28261,28262, 28263,28264,28268,28269 |
| anemia | D57218,D57219,D573,D5740,D57411,D57412,D57413,D57418,D57419 D5742,D57431,D57432,D57433,D57438,D57439,D5744,D57451,D57452 D57453,D57458,D57459,D5780,D57811,D57812,D57813,D57818,D57819 | 28952,5173 |
| Sinusitis | J0100,J0101,J0110,J0111,J0120,J0121,J0130,J0131,J0140,J0141 | 4610,4611,4612,4613,4618,4619,4730, 4731,4732,4733,4738,4739 |
| | J0180,J0181,J0190,J0191,J320,J321,J322,J323,J324,J328,J329 | |
| Skin and subcutaneous | A201,A210,A220,A311,A363,A431,A46,A5139,H05011,H05012,H05013 | 0201,0210,0220,0311,03285,035,0390, 0913,37601,6800,6801,6802 |
| tissue infections | H05019,L00,L0100,L0101,L0102,L0103,L0109,L011,L0201,L0202 | 6803,6804,6805,6806,6807,6808,6809, 68100,68101,68102,68110 |
| | L0203,L0211,L0212,L0213,L02211,L02212,L02213,L02214,L02215 | 68111,6819,6820,6821,6822,6823,6824, 6825,6826,6827,6828,6829 |
| | L02216,L02219,L02221,L02222,L02223,L02224,L02225,L02226 | 683,684,6850,6851,68600,68609,6868, 6869,6908,69581 |
| | L02229,L02231,L02232,L02233,L02234,L02235,L02236,L02239,L0231 L0232,L0233,L02411,L02412,L02413,L02414,L02415,L02416,L02419 L02421,L02422,L02423,L02424,L02425,L02426,L02429,L02431 L02432,L02433,L02434,L02435,L02436,L02439,L02511,L02512 L02519,L02521,L02522,L02529,L02531,L02532,L02539,L02611 L02612,L02619,L02621,L02622,L02629,L02631,L02632,L02639 L02811,L02818,L02821,L02828,L02831,L02838,L0291,L0292,L0293 L03011,L03012,L03019,L03021,L03022,L03029,L03031,L03032 L03039,L03041,L03042,L03049,L03111,L03112,L03113,L03114 L03115,L03116,L03119,L03121,L03122,L03123,L03124,L03125 L03126,L03129,L03211,L03212,L03213,L03221,L03222,L03311 L03312,L03313,L03314,L03315,L03316,L03317,L03319,L03321 L03322,L03323,L03324,L03325,L03326,L03327,L03329,L03811 L03818,L03891,L03898,L0390,L0391,L040,L041,L042,L043,L048 L049,L0501,L0502,L0591,L0592,L080,L081,L0881,L0882,L0889,L089 L303 | |
| Skin cancers - all | C4400,C4409,C44101,C44102,C441021,C441022,C44109,C441091 | 17300,17309,17310,17319,17320,17329, 17330,17339,17340,17349 |
| other types | C441092,C44131,C441321,C441322,C441391,C441392,C44191,C44192 | 17350,17359,17360,17369,17370,17379, 17380,17389,17390,17399 |
| | C441921,C441922,C44199,C441991,C441992,C44201,C44202,C44209 | 20931,20932,20933,20934,20935,20936, 2320,2321,2322,2323,2324 |
| | C44291,C44292,C44299,C44300,C44301,C44309,C44390,C44391 C44399,C4440,C4449,C44500,C44501,C44509,C44590,C44591,C44599 C44601,C44602,C44609,C44691,C44692,C44699,C44701,C44702 C44709,C44791,C44792,C44799,C4480,C4489,C4490,C4499,C4A0 C4A10,C4A11,C4A111,C4A112,C4A12,C4A121,C4A122,C4A20,C4A21 C4A22,C4A30,C4A31,C4A39,C4A4,C4A51,C4A52,C4A59,C4A60,C4A61 C4A62,C4A70,C4A71,C4A72,C4A8,C4A9,D040,D0410,D0411,D04111 D04112,D0412,D04121,D04122,D0420,D0421,D0422,D0430,D0439,D044 D045,D0460,D0461,D0462,D0470,D0471,D0472,D048,D049 | 2325,2326,2327,2328,2329 |
| Skin cancers - basal | C4401,C44111,C44112,C441121,C441122,C44119,C441191,C441192 | 17301,17311,17321,17331,17341,17351, 17361,17371,17381,17391 |
| cell carcinoma | C44211,C44212,C44219,C44310,C44311,C44319,C4441,C44510,C44511 C44519,C44611,C44612,C44619,C44711,C44712,C44719,C4481,C4491 | |
| Skin cancers - | C430,C4310,C4311,C43111,C43112,C4312,C43121,C43122,C4320 | 1720,1721,1722,1723,1724,1725,1726, 1727,1728,1729 |
| melanoma | C4321,C4322,C4330,C4331,C4339,C434,C4351,C4352,C4359,C4360 C4361,C4362,C4370,C4371,C4372,C438,C439,D030,D0310,D0311 D03111,D03112,D0312,D03121,D03122,D0320,D0321,D0322,D0330 D0339,D034,D0351,D0352,D0359,D0360,D0361,D0362,D0370,D0371 D0372,D038,D039 | |
| Skin cancers - | C4402,C44121,C44122,C441221,C441222,C44129,C441291,C441292 | 17302,17312,17322,17332,17342,17352, 17362,17372,17382,17392 |
| squamous cell carcinoma | C44221,C44222,C44229,C44320,C44321,C44329,C4442,C44520,C44521 C44529,C44621,C44622,C44629,C44721,C44722,C44729,C4482,C4492 | |

Continued on next page

**Table S9 (continued).**

| Diagnosis | ICD-10 | ICD-9 |
|---|---|---|
| Skin Subcutaneous | R200,R201,R202,R203,R208,R209,R21,R220,R221,R222,R2230,R2231 | 7820,7821,7822,7825,78261,78262,7827,<br>7828,7829,7842,7866 |
| signs and symptoms | R2232,R2233,R2240,R2241,R2242,R2243,R229,R230,R231,R232,R233<br>R234,R238,R239 | |
| Sleep wake disorders | F19182,F19282,F19982,F5101,F5102,F5103,F5104,F5105,F5109 | 29285,30460,30740,30741,30742,30744,<br>30746,30747,30749,32700 |
| | F5111,F5112,F5113,F5119,F513,F514,F515,F518,F519,G2581,G4700 | 32701,32702,32709,32710,32711,32712,<br>32713,32714,32715,32719 |
| | G4701,G4709,G4710,G4711,G4712,G4713,G4714,G4719,G4720,G4721 | 32720,32721,32722,32723,32724,32725,<br>32726,32727,32729,32730 |
| | G4722,G4723,G4724,G4725,G4726,G4727,G4729,G4730,G4731,G4732 | 32731,32732,32733,32734,32735,32736,<br>32737,32739,32740,32741 |
| | G4733,G4734,G4735,G4736,G4737,G4739,G47411,G47419,G47421 | 32742,32743,32744,32749,32751,32752,<br>32753,32759,3278,33394 |
| | G47429,G4750,G4751,G4752,G4753,G4754,G4759,G4761,G4762,G4763 | 34700,34701,34710,34711,78050,78051,<br>78052,78053,78054,78057 |
| | G4769,G478,G479,R063 | 78604 |
| Somatic disorders | F450,F451,F4520,F4521,F4529,F4541,F4542,F458,F459,F6810,F6811 | 30016,3007,30081,30082,30089,30151,<br>3060,3061,3062,3063,3064 |
| | F6812,F6813,F68A | 30650,30652,30653,30659,3067,3068,3069,<br>30780,30789 |
| Spinal cord injury | S140XXA,S14101A,S14102A,S14103A,S14104A,S14105A,S14106A | 95200,95201,95202,95203,95204,95205,<br>95206,95207,95208,95209 |
| (SCI), initial | S14107A,S14108A,S14109A,S14111A,S14112A,S14113A,S14114A | 95210,95211,95212,95213,95214,95215,<br>95216,95217,95218,95219 |
| encounter | S14115A,S14116A,S14117A,S14118A,S14119A,S14121A,S14122A<br>S14123A,S14124A,S14125A,S14126A,S14127A,S14128A,S14129A<br>S14131A,S14132A,S14133A,S14134A,S14135A,S14136A,S14137A<br>S14138A,S14139A,S14141A,S14142A,S14143A,S14144A,S14145A<br>S14146A,S14147A,S14148A,S14149A,S14151A,S14152A,S14153A<br>S14154A,S14155A,S14156A,S14157A,S14158A,S14159A,S240XXA<br>S24101A,S24102A,S24103A,S24104A,S24109A,S24111A,S24112A<br>S24113A,S24114A,S24119A,S24131A,S24132A,S24133A,S24134A<br>S24139A,S24141A,S24142A,S24143A,S24144A,S24149A,S24151A<br>S24152A,S24153A,S24154A,S24159A,S3401XA,S3402XA,S34101A<br>S34102A,S34103A,S34104A,S34105A,S34109A,S34111A,S34112A<br>S34113A,S34114A,S34115A,S34119A,S34121A,S34122A,S34123A<br>S34124A,S34125A,S34129A,S34131A,S34132A,S34139A,S343XXA | 9522,9523,9524,9529 |
| Spinal cord injury<br>(SCI), subsequent<br>encounter | S140XXD,S14101D,S14102D,S14103D,S14104D,S14105D,S14106D<br>S14107D,S14108D,S14109D,S14111D,S14112D,S14113D,S14114D<br>S14115D,S14116D,S14117D,S14118D,S14119D,S14121D,S14122D<br>S14123D,S14124D,S14125D,S14126D,S14127D,S14128D,S14129D<br>S14131D,S14132D,S14133D,S14134D,S14135D,S14136D,S14137D<br>S14138D,S14139D,S14141D,S14142D,S14143D,S14144D,S14145D<br>S14146D,S14147D,S14148D,S14149D,S14151D,S14152D,S14153D<br>S14154D,S14155D,S14156D,S14157D,S14158D,S14159D,S240XXD<br>S24101D,S24102D,S24103D,S24104D,S24109D,S24111D,S24112D<br>S24113D,S24114D,S24119D,S24131D,S24132D,S24133D,S24134D<br>S24139D,S24141D,S24142D,S24143D,S24144D,S24149D,S24151D<br>S24152D,S24153D,S24154D,S24159D,S3401XD,S3402XD,S34101D<br>S34102D,S34103D,S34104D,S34105D,S34109D,S34111D,S34112D<br>S34113D,S34114D,S34115D,S34119D,S34121D,S34122D,S34123D<br>S34124D,S34125D,S34129D,S34131D,S34132D,S34139D,S343XXD | V5889 |
| Spondylopathies | A5441,L4053,M081,M4300,M4301,M4302,M4303,M4304,M4305,M4306 | 09853,6960,71838,71880,71888,7200,7201,<br>7202,72081,72089,7209 |
| spondyloarthropathy | M4307,M4308,M4309,M4310,M4311,M4312,M4313,M4314,M4315,M4316 | 7210,7211,7212,7213,72141,72142,7215,<br>7216,7217,7218,72190 |
| (including infective) | M4317,M4318,M4319,M4320,M4321,M4322,M4323,M4324,M4325,M4326 | 72191,7220,72210,72211,72231,72232,<br>7224,72251,72252,72271 |
| | M4327,M4328,M433,M434,M435X2,M435X3,M435X4,M435X5,M435X6 | 72272,72273,72280,72281,72282,72283,<br>72290,72291,72292,72293 |
| | M435X7,M435X8,M435X9,M436,M438X1,M438X2,M438X3,M438X4,M438X5 | 7230,7231,7232,7233,7234,7235,7238,<br>72400,72401,72402,72409 |
| | M438X6,M438X7,M438X8,M438X9,M439,M450,M451,M452,M453,M454 | 7243,7244,7246,72470,72471,72479,7249,<br>7292,73098,73740,7378 |
| | M455,M456,M457,M458,M459,M45A0,M45A1,M45A2,M45A3,M45A4,M45A5<br>M45A6,M45A7,M45A8,M45AB,M4600,M4601,M4602,M4603,M4604,M4605<br>M4606,M4607,M4608,M4609,M461,M4630,M4631,M4632,M4633,M4634<br>M4635,M4636,M4637,M4638,M4639,M4640,M4641,M4642,M4643,M4644<br>M4645,M4646,M4647,M4648,M4649,M4650,M4651,M4652,M4653,M4654<br>M4655,M4656,M4657,M4658,M4659,M4680,M4681,M4682,M4683,M4684<br>M4685,M4686,M4687,M4688,M4689,M4690,M4691,M4692,M4693,M4694<br>M4695,M4696,M4697,M4698,M4699,M47011,M47012,M47013,M47014<br>M47015,M47016,M47019,M47021,M47022,M47029,M4710,M4711,M4712<br>M4713,M4714,M4715,M4716,M4720,M4721,M4722,M4723,M4724,M4725<br>M4726,M4727,M4728,M47811,M47812,M47813,M47814,M47815,M47816<br>M47817,M47818,M47819,M47891,M47892,M47893,M47894,M47895<br>M47896,M47897,M47898,M47899,M479,M4800,M4801,M4802,M4803<br>M4804,M4805,M4806,M48061,M48062,M4807,M4808,M4810,M4811,M4812<br>M4813,M4814,M4815,M4816,M4817,M4818,M4819,M4820,M4821,M4822<br>M4823,M4824,M4825,M4826,M4827,M4830,M4831,M4832,M4833,M4834 | 7379,7384 |

Continued on next page

Table S9 (continued).

| Diagnosis | ICD-10 | ICD-9 |
|---|---|---|
| | M4835,M4836,M4837,M4838,M488X1,M488X2,M488X3,M488X4,M488X5<br>M488X6,M488X7,M488X8,M488X9,M489,M4980,M4981,M4982,M4983<br>M4984,M4985,M4986,M4987,M4988,M4989,M5000,M5001,M5002,M50020<br>M50021,M50022,M50023,M5003,M5010,M5011,M5012,M50120,M50121<br>M50122,M50123,M5013,M5020,M5021,M5022,M50220,M50221,M50222<br>M50223,M5023,M5030,M5031,M5032,M50320,M50321,M50322,M50323<br>M5033,M5080,M5081,M5082,M50820,M50821,M50822,M50823,M5083<br>M5090,M5091,M5092,M50920,M50921,M50922,M50923,M5093,M5104<br>M5105,M5106,M5114,M5115,M5116,M5117,M5124,M5125,M5126,M5127<br>M5134,M5135,M5136,M5137,M5144,M5145,M5146,M5147,M5184,M5185<br>M5186,M5187,M519,M51A0,M51A1,M51A2,M51A3,M51A4,M51A5,M530<br>M531,M532X1,M532X2,M532X3,M532X4,M532X5,M532X6,M532X7,M532X8<br>M532X9,M533,M5380,M5381,M5382,M5383,M5384,M5385,M5386,M5387<br>M5388,M539,M5410,M5411,M5412,M5413,M5414,M5415,M5416,M5417<br>M5418,M542,M5430,M5431,M5432,M5440,M5441,M5442,M961 | |
| Spontaneous abortion | O030,O031,O032,O0330,O0331,O0332,O0333,O0334,O0335,O0336 | 63400,63401,63402,63410,63411,63412,<br>63420,63421,63422,63430 |
| and complications of | O0337,O0338,O0339,O034,O035,O036,O037,O0380,O0381,O0382,O0383 | 63431,63432,63440,63441,63442,63450,<br>63451,63452,63460,63461 |
| spontaneous abortion | O0384,O0385,O0386,O0387,O0388,O0389,O039 | 63462,63470,63471,63472,63480,63481,<br>63482,63490,63491,63492 |
| Sprains and strains, | S0340XA,S0341XA,S0342XA,S0343XA,S034XXA,S038XXA,S039XXA | 8360,8361,8362,8400,8401,8402,8403,<br>8404,8405,8406,8407,8408 |
| initial encounter | S0911XA,S134XXA,S135XXA,S138XXA,S139XXA,S161XXA,S233XXA | 8409,8410,8411,8412,8413,8418,8419,<br>84200,84201,84202,84209 |
| | S2341XA,S23420A,S23421A,S23428A,S23429A,S238XXA,S239XXA | 84210,84212,84213,84219,8430,8431,8438,<br>8439,8440,8441,8442 |
| | S29011A,S29012A,S29019A,S335XXA,S336XXA,S338XXA,S339XXA | 8443,8448,8449,84500,84501,84502,84503,<br>84509,84510,84511 |
| | S39011A,S39012A,S39013A,S43401A,S43402A,S43409A,S43411A | 84512,84513,84519,8460,8461,8469,8470,<br>8471,8472,8479,8481 |
| | S43412A,S43419A,S43421A,S43422A,S43429A,S43431A,S43432A | 8482,8483,84840,84841,84842,84849,8485,<br>8488,95901 |
| | S43439A,S43491A,S43492A,S43499A,S4350XA,S4351XA,S4352XA<br>S4360XA,S4361XA,S4362XA,S4380XA,S4381XA,S4382XA,S4390XA<br>S4391XA,S4392XA,S46011A,S46012A,S46019A,S46111A,S46112A<br>S46119A,S46211A,S46212A,S46219A,S46311A,S46312A,S46319A<br>S46811A,S46812A,S46819A,S46911A,S46912A,S46919A,S5320XA<br>S5321XA,S5322XA,S5330XA,S5331XA,S5332XA,S53401A,S53402A<br>S53409A,S53411A,S53412A,S53419A,S53421A,S53422A,S53429A<br>S53431A,S53432A,S53439A,S53441A,S53442A,S53449A,S53491A<br>S53492A,S53499A,S56011A,S56012A,S56019A,S56111A,S56112A<br>S56113A,S56114A,S56115A,S56116A,S56117A,S56118A,S56119A<br>S56211A,S56212A,S56219A,S56311A,S56312A,S56319A,S56411A<br>S56412A,S56413A,S56414A,S56415A,S56416A,S56417A,S56418A<br>S56419A,S56511A,S56512A,S56519A,S56811A,S56812A,S56819A<br>S56911A,S56912A,S56919A,S63301A,S63302A,S63309A,S63311A<br>S63312A,S63319A,S63321A,S63322A,S63329A,S63331A,S63332A<br>S63339A,S63391A,S63392A,S63399A,S63400A,S63401A,S63402A<br>S63403A,S63404A,S63405A,S63406A,S63407A,S63408A,S63409A<br>S63410A,S63411A,S63412A,S63413A,S63414A,S63415A,S63416A<br>S63417A,S63418A,S63419A,S63420A,S63421A,S63422A,S63423A<br>S63424A,S63425A,S63426A,S63427A,S63428A,S63429A,S63430A<br>S63431A,S63432A,S63433A,S63434A,S63435A,S63436A,S63437A<br>S63438A,S63439A,S63490A,S63491A,S63492A,S63493A,S63494A<br>S63495A,S63496A,S63497A,S63498A,S63499A,S63501A,S63502A<br>S63509A,S63511A,S63512A,S63519A,S63521A,S63522A,S63529A<br>S63591A,S63592A,S63599A,S63601A,S63602A,S63609A,S63610A<br>S63611A,S63612A,S63613A,S63614A,S63615A,S63616A,S63617A<br>S63618A,S63619A,S63621A,S63622A,S63629A,S63630A,S63631A<br>S63632A,S63633A,S63634A,S63635A,S63636A,S63637A,S63638A<br>S63639A,S63641A,S63642A,S63649A,S63650A,S63651A,S63652A<br>S63653A,S63654A,S63655A,S63656A,S63657A,S63658A,S63659A<br>S63681A,S63682A,S63689A,S63690A,S63691A,S63692A,S63693A<br>S63694A,S63695A,S63696A,S63697A,S63698A,S63699A,S638X1A<br>S638X2A,S638X9A,S6390XA,S6391XA,S6392XA,S66011A,S66012A<br>S66019A,S66110A,S66111A,S66112A,S66113A,S66114A,S66115A<br>S66116A,S66117A,S66118A,S66119A,S66211A,S66212A,S66219A<br>S66310A,S66311A,S66312A,S66313A,S66314A,S66315A,S66316A<br>S66317A,S66318A,S66319A,S66411A,S66412A,S66419A,S66510A<br>S66511A,S66512A,S66513A,S66514A,S66515A,S66516A,S66517A<br>S66518A,S66519A,S66811A,S66812A,S66819A,S66911A,S66912A<br>S66919A,S73101A,S73102A,S73109A,S73111A,S73112A,S73119A<br>S73121A,S73122A,S73129A,S73191A,S73192A,S73199A,S76011A<br>S76012A,S76019A,S76111A,S76112A,S76119A,S76211A,S76212A<br>S76219A,S76311A,S76312A,S76319A,S76811A,S76812A,S76819A<br>S76911A,S76912A,S76919A,S83200A,S83201A,S83202A,S83203A<br>S83204A,S83205A,S83206A,S83207A,S83209A,S83211A,S83212A<br>S83219A,S83221A,S83222A,S83229A,S83231A,S83232A,S83239A<br>S83241A,S83242A,S83249A,S83251A,S83252A,S83259A,S83261A<br>S83262A,S83269A,S83271A,S83272A,S83279A,S83281A,S83282A<br>S83289A,S8330XA,S8331XA,S8332XA,S83401A,S83402A,S83409A<br>S83411A,S83412A,S83419A,S83421A,S83422A,S83429A,S83501A<br>S83502A,S83509A,S83511A,S83512A,S83519A,S83521A,S83522A | |

Continued on next page

Table S9 (continued).

| Diagnosis | ICD-10 | ICD-9 |
|---|---|---|
| | S83529A,S8360XA,S8361XA,S8362XA,S838X1A,S838X2A,S838X9A | |
| | S8390XA,S8391XA,S8392XA,S86011A,S86012A,S86019A,S86111A | |
| | S86112A,S86119A,S86211A,S86212A,S86219A,S86311A,S86312A | |
| | S86319A,S86811A,S86812A,S86819A,S86911A,S86912A,S86919A | |
| | S93401A,S93402A,S93409A,S93411A,S93412A,S93419A,S93421A | |
| | S93422A,S93429A,S93431A,S93432A,S93439A,S93491A,S93492A | |
| | S93499A,S93501A,S93502A,S93503A,S93504A,S93505A,S93506A | |
| | S93509A,S93511A,S93512A,S93513A,S93514A,S93515A,S93516A | |
| | S93519A,S93521A,S93522A,S93523A,S93524A,S93525A,S93526A | |
| | S93529A,S93601A,S93602A,S93609A,S93611A,S93612A,S93619A | |
| | S93621A,S93622A,S93629A,S93691A,S93692A,S93699A,S96011A | |
| | S96012A,S96019A,S96111A,S96112A,S96119A,S96211A,S96212A | |
| | S96219A,S96811A,S96812A,S96819A,S96911A,S96912A,S96919A | |
| Sprains and strains, | S0340XD,S0341XD,S0342XD,S0343XD,S034XXD,S038XXD,S039XXD | V5889 |
| subsequent encounter | S0911XD,S134XXD,S135XXD,S138XXD,S139XXD,S161XXD,S233XXD | |
| | S2341XD,S23420D,S23421D,S23428D,S23429D,S238XXD,S239XXD | |
| | S29011D,S29012D,S29019D,S335XXD,S336XXD,S338XXD,S339XXD | |
| | S39011D,S39012D,S39013D,S43401D,S43402D,S43409D,S43411D | |
| | S43412D,S43419D,S43421D,S43422D,S43429D,S43431D,S43432D | |
| | S43439D,S43491D,S43492D,S43499D,S4350XD,S4351XD,S4352XD | |
| | S4360XD,S4361XD,S4362XD,S4380XD,S4381XD,S4382XD,S4390XD | |
| | S4391XD,S4392XD,S46011D,S46012D,S46019D,S46111D,S46112D | |
| | S46119D,S46211D,S46212D,S46219D,S46311D,S46312D,S46319D | |
| | S46811D,S46812D,S46819D,S46911D,S46912D,S46919D,S5320XD | |
| | S5321XD,S5322XD,S5330XD,S5331XD,S5332XD,S53401D,S53402D | |
| | S53409D,S53411D,S53412D,S53419D,S53421D,S53422D,S53429D | |
| | S53431D,S53432D,S53439D,S53441D,S53442D,S53449D,S53491D | |
| | S53492D,S53499D,S56011D,S56012D,S56019D,S56111D,S56112D | |
| | S56113D,S56114D,S56115D,S56116D,S56117D,S56118D,S56119D | |
| | S56211D,S56212D,S56219D,S56311D,S56312D,S56319D,S56411D | |
| | S56412D,S56413D,S56414D,S56415D,S56416D,S56417D,S56418D | |
| | S56419D,S56511D,S56512D,S56519D,S56811D,S56812D,S56819D | |
| | S56911D,S56912D,S56919D,S63301D,S63302D,S63309D,S63311D | |
| | S63312D,S63319D,S63321D,S63322D,S63329D,S63331D,S63332D | |
| | S63339D,S63391D,S63392D,S63399D,S63400D,S63401D,S63402D | |
| | S63403D,S63404D,S63405D,S63406D,S63407D,S63408D,S63409D | |
| | S63410D,S63411D,S63412D,S63413D,S63414D,S63415D,S63416D | |
| | S63417D,S63418D,S63419D,S63420D,S63421D,S63422D,S63423D | |
| | S63424D,S63425D,S63426D,S63427D,S63428D,S63429D,S63430D | |
| | S63431D,S63432D,S63433D,S63434D,S63435D,S63436D,S63437D | |
| | S63438D,S63439D,S63490D,S63491D,S63492D,S63493D,S63494D | |
| | S63495D,S63496D,S63497D,S63498D,S63499D,S63501D,S63502D | |
| | S63509D,S63511D,S63512D,S63519D,S63521D,S63522D,S63529D | |
| | S63591D,S63592D,S63599D,S63601D,S63602D,S63609D,S63610D | |
| | S63611D,S63612D,S63613D,S63614D,S63615D,S63616D,S63617D | |
| | S63618D,S63619D,S63621D,S63622D,S63629D,S63630D,S63631D | |
| | S63632D,S63633D,S63634D,S63635D,S63636D,S63637D,S63638D | |
| | S63639D,S63641D,S63642D,S63649D,S63650D,S63651D,S63652D | |
| | S63653D,S63654D,S63655D,S63656D,S63657D,S63658D,S63659D | |
| | S63681D,S63682D,S63689D,S63690D,S63691D,S63692D,S63693D | |
| | S63694D,S63695D,S63696D,S63697D,S63698D,S63699D,S638X1D | |
| | S638X2D,S638X9D,S6390XD,S6391XD,S6392XD,S66011D,S66012D | |
| | S66019D,S66110D,S66111D,S66112D,S66113D,S66114D,S66115D | |
| | S66116D,S66117D,S66118D,S66119D,S66211D,S66212D,S66219D | |
| | S66310D,S66311D,S66312D,S66313D,S66314D,S66315D,S66316D | |
| | S66317D,S66318D,S66319D,S66411D,S66412D,S66419D,S66510D | |
| | S66511D,S66512D,S66513D,S66514D,S66515D,S66516D,S66517D | |
| | S66518D,S66519D,S66811D,S66812D,S66819D,S66911D,S66912D | |
| | S66919D,S73101D,S73102D,S73109D,S73111D,S73112D,S73119D | |
| | S73121D,S73122D,S73129D,S73191D,S73192D,S73199D,S76011D | |
| | S76012D,S76019D,S76111D,S76112D,S76119D,S76211D,S76212D | |
| | S76219D,S76311D,S76312D,S76319D,S76811D,S76812D,S76819D | |
| | S76911D,S76912D,S76919D,S83200D,S83201D,S83202D,S83203D | |
| | S83204D,S83205D,S83206D,S83207D,S83209D,S83211D,S83212D | |
| | S83219D,S83221D,S83222D,S83229D,S83231D,S83232D,S83239D | |
| | S83241D,S83242D,S83249D,S83251D,S83252D,S83259D,S83261D | |
| | S83262D,S83269D,S83271D,S83272D,S83279D,S83281D,S83282D | |
| | S83289D,S8330XD,S8331XD,S8332XD,S83401D,S83402D,S83409D | |
| | S83411D,S83412D,S83419D,S83421D,S83422D,S83429D,S83501D | |
| | S83502D,S83509D,S83511D,S83512D,S83519D,S83521D,S83522D | |
| | S83529D,S8360XD,S8361XD,S8362XD,S838X1D,S838X2D,S838X9D | |
| | S8390XD,S8391XD,S8392XD,S86011D,S86012D,S86019D,S86111D | |
| | S86112D,S86119D,S86211D,S86212D,S86219D,S86311D,S86312D | |
| | S86319D,S86811D,S86812D,S86819D,S86911D,S86912D,S86919D | |
| | S93401D,S93402D,S93409D,S93411D,S93412D,S93419D,S93421D | |
| | S93422D,S93429D,S93431D,S93432D,S93439D,S93491D,S93492D | |
| | S93499D,S93501D,S93502D,S93503D,S93504D,S93505D,S93506D | |
| | S93509D,S93511D,S93512D,S93513D,S93514D,S93515D,S93516D | |
| | S93519D,S93521D,S93522D,S93523D,S93524D,S93525D,S93526D | |
| | S93529D,S93601D,S93602D,S93609D,S93611D,S93612D,S93619D | |
| | S93621D,S93622D,S93629D,S93691D,S93692D,S93699D,S96011D | |
| | S96012D,S96019D,S96111D,S96112D,S96119D,S96211D,S96212D | |
| | S96219D,S96811D,S96812D,S96819D,S96911D,S96912D,S96919D | |

Continued on next page

Table S9 (continued).

| Diagnosis | ICD-10 | ICD-9 |
|---|---|---|
| Stimulant-related disorders | F1410,F14120,F14121,F14122,F14129,F1413,F1414,F14150,F14151<br>F14159,F14180,F14181,F14182,F14188,F1419,F1420,F14220,F14221<br>F14222,F14229,F1423,F1424,F14250,F14251,F14259,F14280,F14281<br>F14282,F14288,F1429,F1490,F1491,F14920,F14921,F14922,F14929<br>F1493,F1494,F14950,F14951,F14959,F14980,F14981,F14982,F14988<br>F1499,F1510,F15120,F15121,F15122,F15129,F1513,F1514,F15150<br>F15151,F15159,F15180,F15181,F15182,F15188,F1519,F1520,F15220<br>F15221,F15222,F15229,F1523,F1524,F15250,F15251,F15259,F15280<br>F15281,F15282,F15288,F1529,F1590,F1591,F15920,F15921,F15922<br>F15929,F1593,F1594,F15950,F15951,F15959,F15980,F15981,F15982<br>F15988,F1599,T405X1A,T405X2A,T405X3A,T405X4A,T405X5A,T43601A<br>T43602A,T43603A,T43604A,T43605A,T43611A,T43612A,T43613A<br>T43614A,T43615A,T43621A,T43622A,T43623A,T43624A,T43625A<br>T43631A,T43632A,T43633A,T43634A,T43635A,T43641A,T43642A<br>T43643A,T43644A,T43651A,T43652A,T43653A,T43654A,T43655A<br>T43691A,T43692A,T43693A,T43694A,T43695A | 2920,29211,29212,2922,29281,29284,<br>29285,29289,2929,30420<br>30421,30422,30440,30441,30442,30560,<br>30561,30562,30563,30570<br>30571,30572,30573,96970,96971,96972,<br>96973,96979,97081,99529<br>E8542,E9397,E9503,E9504,E9620,E9803,<br>E9804 |
| Strabismus | H4900,H4901,H4902,H4903,H4910,H4911,H4912,H4913,H4920,H4921<br>H4922,H4923,H4930,H4931,H4932,H4933,H4940,H4941,H4942,H4943<br>H49811,H49812,H49813,H49819,H49881,H49882,H49883,H49889,H499<br>H5000,H50011,H50012,H50021,H50022,H50031,H50032,H50041,H50042<br>H5005,H5006,H5007,H5008,H5010,H50111,H50112,H50121,H50122<br>H50131,H50132,H50141,H50142,H5015,H5016,H5017,H5018,H5021<br>H5022,H5030,H50311,H50312,H5032,H50331,H50332,H5034,H5040<br>H50411,H50412,H5042,H5043,H5050,H5051,H5052,H5053,H5054,H5055<br>H5060,H50611,H50612,H5069,H50811,H50812,H5089,H509 | 27787,37800,37801,37802,37803,37804,<br>37805,37806,37807,37808<br>37810,37811,37812,37813,37814,37815,<br>37816,37817,37818,37820<br>37821,37822,37823,37824,37830,37831,<br>37833,37834,37835,37840<br>37841,37842,37843,37844,37845,37850,<br>37851,37852,37853,37854<br>37855,37856,37860,37861,37862,37863,<br>37871,37873,3789 |
| Stress fracture, initial encounter | M4840XA,M4841XA,M4842XA,M4843XA,M4844XA,M4845XA,M4846XA<br>M4847XA,M4848XA,M4850XA,M4851XA,M4852XA,M4853XA,M4854XA<br>M4855XA,M4856XA,M4857XA,M4858XA,M8430XA,M84311A,M84312A<br>M84319A,M84321A,M84322A,M84329A,M84331A,M84332A,M84333A<br>M84334A,M84339A,M84341A,M84342A,M84343A,M84344A,M84345A<br>M84346A,M84350A,M84351A,M84352A,M84353A,M84359A,M84361A<br>M84362A,M84363A,M84364A,M84369A,M84371A,M84372A,M84373A<br>M84374A,M84375A,M84376A,M84377A,M84378A,M84379A,M8438XA | 73313,73393,73394,73395,73396,73397,<br>73398 |
| Stress fracture, subsequent encounter | M4840XD,M4840XG,M4841XD,M4841XG,M4842XD,M4842XG,M4843XD<br>M4843XG,M4844XD,M4844XG,M4845XD,M4845XG,M4846XD,M4846XG<br>M4847XD,M4847XG,M4848XD,M4848XG,M4850XD,M4850XG,M4851XD<br>M4851XG,M4852XD,M4852XG,M4853XD,M4853XG,M4854XD,M4854XG<br>M4855XD,M4855XG,M4856XD,M4856XG,M4857XD,M4857XG,M4858XD<br>M4858XG,M8430XD,M8430XG,M8430XK,M8430XP,M84311D,M84311G<br>M84311K,M84311P,M84312D,M84312G,M84312K,M84312P,M84319D<br>M84319G,M84319K,M84319P,M84321D,M84321G,M84321K,M84321P<br>M84322D,M84322G,M84322K,M84322P,M84329D,M84329G,M84329K<br>M84329P,M84331D,M84331G,M84331K,M84331P,M84332D,M84332G<br>M84332K,M84332P,M84333D,M84333G,M84333K,M84333P,M84334D<br>M84334G,M84334K,M84334P,M84339D,M84339G,M84339K,M84339P<br>M84341D,M84341G,M84341K,M84341P,M84342D,M84342G,M84342K<br>M84342P,M84343D,M84343G,M84343K,M84343P,M84344D,M84344G<br>M84344K,M84344P,M84345D,M84345G,M84345K,M84345P,M84346D<br>M84346G,M84346K,M84346P,M84350D,M84350G,M84350K,M84350P<br>M84351D,M84351G,M84351K,M84351P,M84352D,M84352G,M84352K<br>M84352P,M84353D,M84353G,M84353K,M84353P,M84359D,M84359G<br>M84359K,M84359P,M84361D,M84361G,M84361K,M84361P,M84362D<br>M84362G,M84362K,M84362P,M84363D,M84363G,M84363K,M84363P<br>M84364D,M84364G,M84364K,M84364P,M84369D,M84369G,M84369K<br>M84369P,M84371D,M84371G,M84371K,M84371P,M84372D,M84372G<br>M84372K,M84372P,M84373D,M84373G,M84373K,M84373P,M84374D<br>M84374G,M84374K,M84374P,M84375D,M84375G,M84375K,M84375P<br>M84376D,M84376G,M84376K,M84376P,M84377D,M84377G,M84377K<br>M84377P,M84378D,M84378G,M84378K,M84378P,M84379D,M84379G<br>M84379K,M84379P,M8438XD,M8438XG,M8438XK,M8438XP | 73381,73382,V5421,V5422,V5423,V5425,<br>V5426,V5427,V5429 |
| Suicidal ideation attempt intentional self-harm | R45851,R4588,T1491,T1491XA,T360X2A,T361X2A,T362X2A,T363X2A<br>T364X2A,T365X2A,T366X2A,T367X2A,T368X2A,T3692XA,T370X2A<br>T371X2A,T372X2A,T373X2A,T374X2A,T375X2A,T378X2A,T3792XA<br>T380X2A,T381X2A,T382X2A,T383X2A,T384X2A,T385X2A,T386X2A<br>T387X2A,T38802A,T38812A,T38892A,T38902A,T38992A,T39012A<br>T39092A,T391X2A,T392X2A,T39312A,T39392A,T394X2A,T398X2A | 9599,9600,9601,9602,9603,9604,9605,<br>9606,9608,9609,9610,9614<br>9615,9616,9617,9618,9619,9620,9621,<br>9622,9623,9624,9627,9628<br>9629,9630,9631,9634,9635,9638,9639,<br>9640,9642,9643,9644,9645<br>9646,9648,9649,96500,96501,96502,96509,<br>9651,9654,9655,96561<br>96569,9657,9659,9660,9661,9662,9663,<br>9664,9670,9678,9679,9680<br>9682,9683,9684,9685,9689,96900,96901,<br>96902,96903,96904,96905 |

Continued on next page

Table S9 (continued).

| Diagnosis | ICD-10 | ICD-9 |
|---|---|---|
| | T3992XA,T400X2A,T401X2A,T402X2A,T403X2A,T40412A,T40422A | 96909,9691,9692,9693,9694,9696,96970,96971,96972,96973,96979 |
| | T40492A,T404X2A,T405X2A,T40602A,T40692A,T40712A,T40722A | 9698,9699,9700,9701,97081,9710,9711,9712,9713,9719,9720,9721 |
| | T407X2A,T408X2A,T40902A,T40992A,T410X2A,T411X2A,T41202A | 9722,9723,9724,9725,9726,9727,9729,9730,9731,9732,9733,9734 |
| | T41292A,T413X2A,T4142XA,T415X2A,T420X2A,T421X2A,T422X2A | 9735,9736,9738,9739,9742,9744,9745,9747,9750,9752,9753,9754 |
| | T423X2A,T424X2A,T425X2A,T426X2A,T4272XA,T428X2A,T43012A | 9755,9756,9757,9758,9760,9761,9762,9763,9764,9765,9766,9767 |
| | T43022A,T431X2A,T43202A,T43212A,T43222A,T43292A,T433X2A | 9768,9769,9770,9772,9778,9779,9786,9788,9789,9790,9796,9799 |
| | T434X2A,T43502A,T43592A,T43602A,T43612A,T43622A,T43632A | 9800,9801,9802,9803,9808,9809,981,9820,9821,9822,9823,9824 |
| | T43642A,T43652A,T43692A,T438X2A,T4392XA,T440X2A,T441X2A | 9828,9830,9831,9832,9839,9849,9850,9851,9852,9853,9855,9856 |
| | T442X2A,T443X2A,T444X2A,T445X2A,T446X2A,T447X2A,T448X2A | 9858,9859,986,9871,9872,9873,9874,9875,9876,9877,9878,9879 |
| | T44902A,T44992A,T450X2A,T451X2A,T452X2A,T453X2A,T454X2A | 9880,9881,9882,9888,9889,9890,9891,9893,9894,9895,9896,9897 |
| | T45512A,T45522A,T45602A,T45612A,T45622A,T45692A,T457X2A | 98982,98984,98989,9899,9947,E9500,E9501,E9502,E9503,E9504 |
| | T458X2A,T4592XA,T460X2A,T461X2A,T462X2A,T463X2A,T464X2A | E9506,E9507,E9508,E9509,E9520,E9521,E9528,E9529,E9530,E9531 |
| | T465X2A,T466X2A,T467X2A,T468X2A,T46902A,T46992A,T470X2A | E9538,E954,E9550,E9551,E9552,E9553,E9554,E9555,E9556,E9557 |
| | T471X2A,T472X2A,T473X2A,T474X2A,T475X2A,T476X2A,T477X2A | E9559,E956,E9579,E9580,E9581,E9582,E9583,E9584,E9585,E9586 |
| | T478X2A,T4792XA,T480X2A,T481X2A,T48202A,T48292A,T483X2A | E9587,E9588,E9589,V6284 |
| | T484X2A,T485X2A,T486X2A,T48902A,T48992A,T490X2A,T491X2A | |
| | T492X2A,T493X2A,T494X2A,T495X2A,T496X2A,T497X2A,T498X2A | |
| | T4992XA,T500X2A,T501X2A,T502X2A,T503X2A,T504X2A,T505X2A | |
| | T506X2A,T507X2A,T508X2A,T50902A,T50912A,T50992A,T50A12A | |
| | T50A22A,T50A92A,T50B12A,T50B92A,T50Z12A,T50Z92A,T510X2A | |
| | T511X2A,T512X2A,T513X2A,T518X2A,T5192XA,T520X2A,T521X2A | |
| | T522X2A,T523X2A,T524X2A,T528X2A,T5292XA,T530X2A,T531X2A | |
| | T532X2A,T533X2A,T534X2A,T535X2A,T536X2A,T537X2A,T5392XA | |
| | T540X2A,T541X2A,T542X2A,T543X2A,T5492XA,T550X2A,T551X2A | |
| | T560X2A,T561X2A,T562X2A,T563X2A,T564X2A,T565X2A,T566X2A | |
| | T567X2A,T56812A,T56892A,T5692XA,T570X2A,T571X2A,T572X2A | |
| | T573X2A,T578X2A,T5792XA,T5802XA,T5812XA,T582X2A,T588X2A | |
| | T5892XA,T590X2A,T591X2A,T592X2A,T593X2A,T594X2A,T595X2A | |
| | T596X2A,T597X2A,T59812A,T59892A,T5992XA,T600X2A,T601X2A | |
| | T602X2A,T603X2A,T604X2A,T608X2A,T6092XA,T6102XA,T6112XA | |
| | T61772A,T61782A,T618X2A,T6192XA,T620X2A,T621X2A,T622X2A | |
| | T628X2A,T6292XA,T63002A,T63012A,T63022A,T63032A,T63042A | |
| | T63062A,T63072A,T63082A,T63092A,T63112A,T63122A,T63192A | |
| | T632X2A,T63302A,T63312A,T63322A,T63332A,T63392A,T63412A | |
| | T63422A,T63432A,T63442A,T63452A,T63462A,T63482A,T63512A | |
| | T63592A,T63612A,T63622A,T63632A,T63692A,T63712A,T63792A | |
| | T63812A,T63822A,T63832A,T63892A,T6392XA,T6402XA,T6482XA | |
| | T650X2A,T651X2A,T65212A,T65222A,T65292A,T653X2A,T654X2A | |
| | T655X2A,T656X2A,T65812A,T65822A,T65832A,T65892A,T6592XA | |
| | T71112A,T71122A,T71132A,T71152A,T71162A,T71192A,T71222A | |
| | T71232A,X710XXA,X711XXA,X712XXA,X713XXA,X718XXA,X719XXA | |
| | X72XXXA,X730XXA,X731XXA,X732XXA,X738XXA,X739XXA,X7401XA | |
| | X7402XA,X7409XA,X748XXA,X749XXA,X75XXXA,X76XXXA,X770XXA | |
| | X771XXA,X772XXA,X773XXA,X778XXA,X779XXA,X780XXA,X781XXA | |
| | X782XXA,X788XXA,X789XXA,X79XXXA,X80XXXA,X810XXA,X811XXA | |
| | X818XXA,X820XXA,X821XXA,X822XXA,X828XXA,X830XXA,X831XXA | |
| | X832XXA,X838XXA | |
| Suicide attempt | T1491XD,T360X2D,T361X2D,T362X2D,T363X2D,T364X2D,T365X2D | E954,E9550,E9551,E9552,E9553,E9554,E9555,E9556,E9557,E9559 |
| intentional | T366X2D,T367X2D,T368X2D,T3692XD,T370X2D,T371X2D,T372X2D | E956,E9579,E9580,E9581,E9582,E9583,E9584,E9585,E9586,E9587 |
| self-harm; subsequent encounter | T373X2D,T374X2D,T375X2D,T378X2D,T3792XD,T380X2D,T381X2D | E9588,V5889 |
| | T382X2D,T383X2D,T384X2D,T385X2D,T386X2D,T387X2D,T38802D | |
| | T38812D,T38892D,T38902D,T38992D,T39012D,T39092D,T391X2D | |
| | T392X2D,T39312D,T39392D,T394X2D,T398X2D,T3992XD,T400X2D | |
| | T401X2D,T402X2D,T403X2D,T40412D,T40422D,T40492D,T404X2D | |
| | T405X2D,T40602D,T40692D,T40712D,T40722D,T407X2D,T408X2D | |
| | T40902D,T40992D,T410X2D,T411X2D,T41202D,T41292D,T413X2D | |
| | T4142XD,T415X2D,T420X2D,T421X2D,T422X2D,T423X2D,T424X2D | |
| | T425X2D,T426X2D,T4272XD,T428X2D,T43012D,T43022D,T431X2D | |
| | T43202D,T43212D,T43222D,T43292D,T433X2D,T434X2D,T43502D | |
| | T43592D,T43602D,T43612D,T43622D,T43632D,T43642D,T43652D | |
| | T43692D,T438X2D,T4392XD,T440X2D,T441X2D,T442X2D,T443X2D | |
| | T444X2D,T445X2D,T446X2D,T447X2D,T448X2D,T44902D,T44992D | |
| | T450X2D,T451X2D,T452X2D,T453X2D,T454X2D,T45512D,T45522D | |
| | T45602D,T45612D,T45622D,T45692D,T457X2D,T458X2D,T4592XD | |
| | T460X2D,T461X2D,T462X2D,T463X2D,T464X2D,T465X2D,T466X2D | |
| | T467X2D,T468X2D,T46902D,T46992D,T470X2D,T471X2D,T472X2D | |
| | T473X2D,T474X2D,T475X2D,T476X2D,T477X2D,T478X2D,T4792XD | |
| | T480X2D,T481X2D,T48202D,T48292D,T483X2D,T484X2D,T485X2D | |
| | T486X2D,T48902D,T48992D,T490X2D,T491X2D,T492X2D,T493X2D | |

Continued on next page

Table S9 (continued).

| Diagnosis | ICD-10 | ICD-9 |
|---|---|---|
| | T494X2D,T495X2D,T496X2D,T497X2D,T498X2D,T4992XD,T500X2D | |
| | T501X2D,T502X2D,T503X2D,T504X2D,T505X2D,T506X2D,T507X2D | |
| | T508X2D,T50902D,T50912D,T50992D,T50A12D,T50A22D,T50A92D | |
| | T50B12D,T50B92D,T50Z12D,T50Z92D,T510X2D,T511X2D,T512X2D | |
| | T513X2D,T518X2D,T5192XD,T520X2D,T521X2D,T522X2D,T523X2D | |
| | T524X2D,T528X2D,T5292XD,T530X2D,T531X2D,T532X2D,T533X2D | |
| | T534X2D,T535X2D,T536X2D,T537X2D,T5392XD,T540X2D,T541X2D | |
| | T542X2D,T543X2D,T5492XD,T550X2D,T551X2D,T560X2D,T561X2D | |
| | T562X2D,T563X2D,T564X2D,T565X2D,T566X2D,T567X2D,T56812D | |
| | T56892D,T5692XD,T570X2D,T571X2D,T572X2D,T573X2D,T578X2D | |
| | T5792XD,T5802XD,T5812XD,T582X2D,T588X2D,T5892XD,T590X2D | |
| | T591X2D,T592X2D,T593X2D,T594X2D,T595X2D,T596X2D,T597X2D | |
| | T59812D,T59892D,T5992XD,T600X2D,T601X2D,T602X2D,T603X2D | |
| | T604X2D,T608X2D,T6092XD,T6102XD,T6112XD,T61772D,T61782D | |
| | T618X2D,T6192XD,T620X2D,T621X2D,T622X2D,T628X2D,T6292XD | |
| | T63002D,T63012D,T63022D,T63032D,T63042D,T63062D,T63072D | |
| | T63082D,T63092D,T63112D,T63122D,T63192D,T632X2D,T63302D | |
| | T63312D,T63322D,T63332D,T63392D,T63412D,T63422D,T63432D | |
| | T63442D,T63452D,T63462D,T63482D,T63512D,T63592D,T63612D | |
| | T63622D,T63632D,T63692D,T63712D,T63792D,T63812D,T63822D | |
| | T63832D,T63892D,T6392XD,T6402XD,T6482XD,T650X2D,T651X2D | |
| | T65212D,T65222D,T65292D,T653X2D,T654X2D,T655X2D,T656X2D | |
| | T65812D,T65822D,T65832D,T65892D,T6592XD,T71112D,T71122D | |
| | T71132D,T71152D,T71162D,T71192D,T71222D,T71232D,X710XXD | |
| | X711XXD,X712XXD,X713XXD,X718XXD,X719XXD,X72XXXD,X730XXD | |
| | X731XXD,X732XXD,X738XXD,X739XXD,X7401XD,X7402XD,X7409XD | |
| | X748XXD,X749XXD,X75XXXD,X76XXXD,X770XXD,X771XXD,X772XXD | |
| | X773XXD,X778XXD,X779XXD,X780XXD,X781XXD,X782XXD,X788XXD | |
| | X789XXD,X79XXXD,X80XXXD,X810XXD,X811XXD,X818XXD,X828XXD | |
| | X830XXD,X831XXD,X832XXD,X838XXD | |
| Superficial injury; | S0000XA,S0001XA,S0002XA,S0003XA,S0004XA,S0005XA,S0006XA | 9100,9102,9104,9106,9108,9110,9112,<br>9114,9116,9118,9120,9122 |
| contusion, initial | S0007XA,S0010XA,S0011XA,S0012XA,S00201A,S00202A,S00209A | 9124,9126,9128,9130,9132,9134,9136,<br>9138,9140,9142,9144,9146 |
| encounter | S00211A,S00212A,S00219A,S00221A,S00222A,S00229A,S00241A | 9148,9150,9152,9154,9156,9158,9160,<br>9162,9164,9166,9168,9170 |
| | S00242A,S00249A,S00251A,S00252A,S00259A,S00261A,S00262A | 9172,9174,9176,9178,9180,9181,9182,920,<br>9210,9211,9212,9213 |
| | S00269A,S00271A,S00272A,S00279A,S0030XA,S0031XA,S0032XA | 9220,9221,9222,92231,92232,92233,9224,<br>9228,9229,92300,92301 |
| | S0033XA,S0034XA,S0035XA,S0036XA,S0037XA,S00401A,S00402A | 92302,92303,92310,92311,92320,92321,<br>9233,92400,92401,92410 |
| | S00409A,S00411A,S00412A,S00419A,S00421A,S00422A,S00429A | 92411,92420,92421,9243,9244,9245,9248 |
| | S00431A,S00432A,S00439A,S00441A,S00442A,S00449A,S00451A | |
| | S00452A,S00459A,S00461A,S00462A,S00469A,S00471A,S00472A | |
| | S00479A,S00501A,S00502A,S00511A,S00512A,S00521A,S00522A | |
| | S00531A,S00532A,S00541A,S00542A,S00551A,S00552A,S00561A | |
| | S00562A,S00571A,S00572A,S0080XA,S0081XA,S0082XA,S0083XA | |
| | S0084XA,S0085XA,S0086XA,S0087XA,S0090XA,S0091XA,S0092XA | |
| | S0093XA,S0094XA,S0095XA,S0096XA,S0097XA,S0500XA,S0501XA | |
| | S0502XA,S0510XA,S0511XA,S0512XA,S100XXA,S1010XA,S1011XA | |
| | S1012XA,S1014XA,S1015XA,S1016XA,S1017XA,S1080XA,S1081XA | |
| | S1082XA,S1083XA,S1084XA,S1085XA,S1086XA,S1087XA,S1090XA | |
| | S1091XA,S1092XA,S1093XA,S1094XA,S1095XA,S1096XA,S1097XA | |
| | S2000XA,S2001XA,S2002XA,S20101A,S20102A,S20109A,S20111A | |
| | S20112A,S20119A,S20121A,S20122A,S20129A,S20141A,S20142A | |
| | S20149A,S20151A,S20152A,S20159A,S20161A,S20162A,S20169A | |
| | S20171A,S20172A,S20179A,S2020XA,S20211A,S20212A,S20213A | |
| | S20214A,S20219A,S20221A,S20222A,S20223A,S20224A,S20229A | |
| | S20301A,S20302A,S20303A,S20304A,S20309A,S20311A,S20312A | |
| | S20313A,S20314A,S20319A,S20321A,S20322A,S20323A,S20324A | |
| | S20329A,S20341A,S20342A,S20343A,S20344A,S20349A,S20351A | |
| | S20352A,S20353A,S20354A,S20359A,S20361A,S20362A,S20363A | |
| | S20364A,S20369A,S20371A,S20372A,S20373A,S20374A,S20379A | |
| | S20401A,S20402A,S20409A,S20411A,S20412A,S20419A,S20421A | |
| | S20422A,S20429A,S20441A,S20442A,S20449A,S20451A,S20452A | |
| | S20459A,S20461A,S20462A,S20469A,S20471A,S20472A,S20479A | |
| | S2090XA,S2091XA,S2092XA,S2094XA,S2095XA,S2096XA,S2097XA | |
| | S300XXA,S301XXA,S30201A,S30202A,S3021XA,S3022XA,S3023XA | |
| | S303XXA,S30810A,S30811A,S30812A,S30813A,S30814A,S30815A | |
| | S30816A,S30817A,S30820A,S30821A,S30822A,S30823A,S30824A | |
| | S30825A,S30826A,S30827A,S30840A,S30841A,S30842A,S30843A | |
| | S30844A,S30845A,S30846A,S30850A,S30851A,S30852A,S30853A | |
| | S30854A,S30855A,S30856A,S30857A,S30860A,S30861A,S30862A | |
| | S30863A,S30864A,S30865A,S30866A,S30867A,S30870A,S30871A | |
| | S30872A,S30873A,S30874A,S30875A,S30876A,S30877A,S3091XA | |
| | S3092XA,S3093XA,S3094XA,S3095XA,S3096XA,S3097XA,S3098XA | |
| | S40011A,S40012A,S40019A,S40021A,S40022A,S40029A,S40211A | |
| | S40212A,S40219A,S40221A,S40222A,S40229A,S40241A,S40242A | |
| | S40249A,S40251A,S40252A,S40259A,S40261A,S40262A,S40269A | |
| | S40271A,S40272A,S40279A,S40811A,S40812A,S40819A,S40821A | |
| | S40822A,S40829A,S40841A,S40842A,S40849A,S40851A,S40852A | |
| | S40859A,S40861A,S40862A,S40869A,S40871A,S40872A,S40879A | |
| | S40911A,S40912A,S40919A,S40921A,S40922A,S40929A,S5000XA | |

Continued on next page

Table S9 (continued).

| Diagnosis | ICD-10 | ICD-9 |
|---|---|---|
| | S5001XA,S5002XA,S5010XA,S5011XA,S5012XA,S50311A,S50312A<br>S50319A,S50321A,S50322A,S50329A,S50341A,S50342A,S50349A<br>S50351A,S50352A,S50359A,S50361A,S50362A,S50369A,S50371A<br>S50372A,S50379A,S50811A,S50812A,S50819A,S50821A,S50822A<br>S50829A,S50841A,S50842A,S50849A,S50851A,S50852A,S50859A<br>S50861A,S50862A,S50869A,S50871A,S50872A,S50879A,S50901A<br>S50902A,S50909A,S50911A,S50912A,S50919A,S6000XA,S60011A<br>S60012A,S60019A,S60021A,S60022A,S60029A,S60031A,S60032A<br>S60039A,S60041A,S60042A,S60049A,S60051A,S60052A,S60059A<br>S6010XA,S60111A,S60112A,S60119A,S60121A,S60122A,S60129A<br>S60131A,S60132A,S60139A,S60141A,S60142A,S60149A,S60151A<br>S60152A,S60159A,S60211A,S60212A,S60219A,S60221A,S60222A<br>S60229A,S60311A,S60312A,S60319A,S60321A,S60322A,S60329A<br>S60341A,S60342A,S60349A,S60351A,S60352A,S60359A,S60361A<br>S60362A,S60369A,S60371A,S60372A,S60379A,S60391A,S60392A<br>S60399A,S60410A,S60411A,S60412A,S60413A,S60414A,S60415A<br>S60416A,S60417A,S60418A,S60419A,S60420A,S60421A,S60422A<br>S60423A,S60424A,S60425A,S60426A,S60427A,S60428A,S60429A<br>S60440A,S60441A,S60442A,S60443A,S60444A,S60445A,S60446A<br>S60447A,S60448A,S60449A,S60450A,S60451A,S60452A,S60453A<br>S60454A,S60455A,S60456A,S60457A,S60458A,S60459A,S60460A<br>S60461A,S60462A,S60463A,S60464A,S60465A,S60466A,S60467A<br>S60468A,S60469A,S60470A,S60471A,S60472A,S60473A,S60474A<br>S60475A,S60476A,S60477A,S60478A,S60479A,S60511A,S60512A<br>S60519A,S60521A,S60522A,S60529A,S60541A,S60542A,S60549A<br>S60551A,S60552A,S60559A,S60561A,S60562A,S60569A,S60571A<br>S60572A,S60579A,S60811A,S60812A,S60819A,S60821A,S60822A<br>S60829A,S60841A,S60842A,S60849A,S60851A,S60852A,S60859A<br>S60861A,S60862A,S60869A,S60871A,S60872A,S60879A,S60911A<br>S60912A,S60919A,S60921A,S60922A,S60929A,S60931A,S60932A<br>S60939A,S60940A,S60941A,S60942A,S60943A,S60944A,S60945A<br>S60946A,S60947A,S60948A,S60949A,S7000XA,S7001XA,S7002XA<br>S7010XA,S7011XA,S7012XA,S70211A,S70212A,S70219A,S70221A<br>S70222A,S70229A,S70241A,S70242A,S70249A,S70251A,S70252A<br>S70259A,S70261A,S70262A,S70269A,S70271A,S70272A,S70279A<br>S70311A,S70312A,S70319A,S70321A,S70322A,S70329A,S70341A<br>S70342A,S70349A,S70351A,S70352A,S70359A,S70361A,S70362A<br>S70369A,S70371A,S70372A,S70379A,S70911A,S70912A,S70919A<br>S70921A,S70922A,S70929A,S8000XA,S8001XA,S8002XA,S8010XA<br>S8011XA,S8012XA,S80211A,S80212A,S80219A,S80221A,S80222A<br>S80229A,S80241A,S80242A,S80249A,S80251A,S80252A,S80259A<br>S80261A,S80262A,S80269A,S80271A,S80272A,S80279A,S80811A<br>S80812A,S80819A,S80821A,S80822A,S80829A,S80841A,S80842A<br>S80849A,S80851A,S80852A,S80859A,S80861A,S80862A,S80869A<br>S80871A,S80872A,S80879A,S80911A,S80912A,S80919A,S80921A<br>S80922A,S80929A,S9000XA,S9001XA,S9002XA,S90111A,S90112A<br>S90119A,S90121A,S90122A,S90129A,S90211A,S90212A,S90219A<br>S90221A,S90222A,S90229A,S9030XA,S9031XA,S9032XA,S90411A<br>S90412A,S90413A,S90414A,S90415A,S90416A,S90421A,S90422A<br>S90423A,S90424A,S90425A,S90426A,S90441A,S90442A,S90443A<br>S90444A,S90445A,S90446A,S90451A,S90452A,S90453A,S90454A<br>S90455A,S90456A,S90461A,S90462A,S90463A,S90464A,S90465A<br>S90466A,S90471A,S90472A,S90473A,S90474A,S90475A,S90476A<br>S90511A,S90512A,S90519A,S90521A,S90522A,S90529A,S90541A<br>S90542A,S90549A,S90551A,S90552A,S90559A,S90561A,S90562A<br>S90569A,S90571A,S90572A,S90579A,S90811A,S90812A,S90819A<br>S90821A,S90822A,S90829A,S90841A,S90842A,S90849A,S90851A<br>S90852A,S90859A,S90861A,S90862A,S90869A,S90871A,S90872A<br>S90879A,S90911A,S90912A,S90919A,S90921A,S90922A,S90929A<br>S90931A,S90932A,S90933A,S90934A,S90935A,S90936A | |
| Superficial injury; contusion, subsequent encounter | S0000XD,S0001XD,S0002XD,S0003XD,S0004XD,S0005XD,S0006XD<br>S0007XD,S0010XD,S0011XD,S0012XD,S00201D,S00202D,S00209D<br>S00211D,S00212D,S00219D,S00221D,S00222D,S00229D,S00241D<br>S00242D,S00249D,S00251D,S00252D,S00259D,S00261D,S00262D<br>S00269D,S00271D,S00272D,S00279D,S0030XD,S0031XD,S0032XD<br>S0033XD,S0034XD,S0035XD,S0036XD,S0037XD,S00401D,S00402D<br>S00409D,S00411D,S00412D,S00419D,S00421D,S00422D,S00429D<br>S00431D,S00432D,S00439D,S00441D,S00442D,S00449D,S00451D<br>S00452D,S00459D,S00461D,S00462D,S00469D,S00471D,S00472D<br>S00479D,S00501D,S00502D,S00511D,S00512D,S00521D,S00522D<br>S00531D,S00532D,S00541D,S00542D,S00551D,S00552D,S00561D<br>S00562D,S00571D,S00572D,S0080XD,S0081XD,S0082XD,S0083XD<br>S0084XD,S0085XD,S0086XD,S0087XD,S0090XD,S0091XD,S0092XD<br>S0093XD,S0094XD,S0095XD,S0096XD,S0097XD,S0500XD,S0501XD<br>S0502XD,S0510XD,S0511XD,S0512XD,S100XXD,S1010XD,S1011XD<br>S1012XD,S1014XD,S1015XD,S1016XD,S1017XD,S1080XD,S1081XD<br>S1082XD,S1083XD,S1084XD,S1085XD,S1086XD,S1087XD,S1090XD<br>S1091XD,S1092XD,S1093XD,S1094XD,S1095XD,S1096XD,S1097XD<br>S2000XD,S2001XD,S2002XD,S20101D,S20102D,S20109D,S20111D<br>S20112D,S20119D,S20121D,S20122D,S20129D,S20141D,S20142D<br>S20149D,S20151D,S20152D,S20159D,S20161D,S20162D,S20169D<br>S20171D,S20172D,S20179D,S2020XD,S20211D,S20212D,S20213D<br>S20214D,S20219D,S20221D,S20222D,S20223D,S20224D,S20229D<br>S20301D,S20302D,S20303D,S20304D,S20309D,S20311D,S20312D | V5889 |

Continued on next page

Table S9 (continued).

| Diagnosis | ICD-10 | ICD-9 |
|---|---|---|
| | S20313D,S20314D,S20319D,S20321D,S20322D,S20323D,S20324D | |
| | S20329D,S20341D,S20342D,S20343D,S20344D,S20349D,S20351D | |
| | S20352D,S20353D,S20354D,S20359D,S20361D,S20362D,S20363D | |
| | S20364D,S20369D,S20371D,S20372D,S20373D,S20374D,S20379D | |
| | S20401D,S20402D,S20409D,S20411D,S20412D,S20419D,S20421D | |
| | S20422D,S20429D,S20441D,S20442D,S20449D,S20451D,S20452D | |
| | S20459D,S20461D,S20462D,S20469D,S20471D,S20472D,S20479D | |
| | S2090XD,S2091XD,S2092XD,S2094XD,S2095XD,S2096XD,S2097XD | |
| | S300XXD,S301XXD,S30201D,S30202D,S3021XD,S3022XD,S3023XD | |
| | S303XXD,S30810D,S30811D,S30812D,S30813D,S30814D,S30815D | |
| | S30816D,S30817D,S30820D,S30821D,S30822D,S30823D,S30824D | |
| | S30825D,S30826D,S30827D,S30840D,S30841D,S30842D,S30843D | |
| | S30844D,S30845D,S30846D,S30850D,S30851D,S30852D,S30853D | |
| | S30854D,S30855D,S30856D,S30857D,S30860D,S30861D,S30862D | |
| | S30863D,S30864D,S30865D,S30866D,S30867D,S30870D,S30871D | |
| | S30872D,S30873D,S30874D,S30875D,S30876D,S30877D,S3091XD | |
| | S3092XD,S3093XD,S3094XD,S3095XD,S3096XD,S3097XD,S3098XD | |
| | S40011D,S40012D,S40019D,S40021D,S40022D,S40029D,S40211D | |
| | S40212D,S40219D,S40221D,S40222D,S40229D,S40241D,S40242D | |
| | S40249D,S40251D,S40252D,S40259D,S40261D,S40262D,S40269D | |
| | S40271D,S40272D,S40279D,S40811D,S40812D,S40819D,S40821D | |
| | S40822D,S40829D,S40841D,S40842D,S40849D,S40851D,S40852D | |
| | S40859D,S40861D,S40862D,S40869D,S40871D,S40872D,S40879D | |
| | S40911D,S40912D,S40919D,S40921D,S40922D,S40929D,S5000XD | |
| | S5001XD,S5002XD,S5010XD,S5011XD,S5012XD,S50311D,S50312D | |
| | S50319D,S50321D,S50322D,S50329D,S50341D,S50342D,S50349D | |
| | S50351D,S50352D,S50359D,S50361D,S50362D,S50369D,S50371D | |
| | S50372D,S50379D,S50811D,S50812D,S50819D,S50821D,S50822D | |
| | S50829D,S50841D,S50842D,S50849D,S50851D,S50852D,S50859D | |
| | S50861D,S50862D,S50869D,S50871D,S50872D,S50879D,S50901D | |
| | S50902D,S50909D,S50911D,S50912D,S50919D,S6000XD,S60011D | |
| | S60012D,S60019D,S60021D,S60022D,S60029D,S60031D,S60032D | |
| | S60039D,S60041D,S60042D,S60049D,S60051D,S60052D,S60059D | |
| | S6010XD,S60111D,S60112D,S60119D,S60121D,S60122D,S60129D | |
| | S60131D,S60132D,S60139D,S60141D,S60142D,S60149D,S60151D | |
| | S60152D,S60159D,S60211D,S60212D,S60219D,S60221D,S60222D | |
| | S60229D,S60311D,S60312D,S60319D,S60321D,S60322D,S60329D | |
| | S60341D,S60342D,S60349D,S60351D,S60352D,S60359D,S60361D | |
| | S60362D,S60369D,S60371D,S60372D,S60379D,S60391D,S60392D | |
| | S60399D,S60410D,S60411D,S60412D,S60413D,S60414D,S60415D | |
| | S60416D,S60417D,S60418D,S60419D,S60420D,S60421D,S60422D | |
| | S60423D,S60424D,S60425D,S60426D,S60427D,S60428D,S60429D | |
| | S60440D,S60441D,S60442D,S60443D,S60444D,S60445D,S60446D | |
| | S60447D,S60448D,S60449D,S60450D,S60451D,S60452D,S60453D | |
| | S60454D,S60455D,S60456D,S60457D,S60458D,S60459D,S60460D | |
| | S60461D,S60462D,S60463D,S60464D,S60465D,S60466D,S60467D | |
| | S60468D,S60469D,S60470D,S60471D,S60472D,S60473D,S60474D | |
| | S60475D,S60476D,S60477D,S60478D,S60479D,S60511D,S60512D | |
| | S60519D,S60521D,S60522D,S60529D,S60541D,S60542D,S60549D | |
| | S60551D,S60552D,S60559D,S60561D,S60562D,S60569D,S60571D | |
| | S60572D,S60579D,S60811D,S60812D,S60819D,S60821D,S60822D | |
| | S60829D,S60841D,S60842D,S60849D,S60851D,S60852D,S60859D | |
| | S60861D,S60862D,S60869D,S60871D,S60872D,S60879D,S60911D | |
| | S60912D,S60919D,S60921D,S60922D,S60929D,S60931D,S60932D | |
| | S60939D,S60940D,S60941D,S60942D,S60943D,S60944D,S60945D | |
| | S60946D,S60947D,S60948D,S60949D,S7000XD,S7001XD,S7002XD | |
| | S7010XD,S7011XD,S7012XD,S70211D,S70212D,S70219D,S70221D | |
| | S70222D,S70229D,S70241D,S70242D,S70249D,S70251D,S70252D | |
| | S70259D,S70261D,S70262D,S70269D,S70271D,S70272D,S70279D | |
| | S70311D,S70312D,S70319D,S70321D,S70322D,S70329D,S70341D | |
| | S70342D,S70349D,S70351D,S70352D,S70359D,S70361D,S70362D | |
| | S70369D,S70371D,S70372D,S70379D,S70911D,S70912D,S70919D | |
| | S70921D,S70922D,S70929D,S8000XD,S8001XD,S8002XD,S8010XD | |
| | S8011XD,S8012XD,S80211D,S80212D,S80219D,S80221D,S80222D | |
| | S80229D,S80241D,S80242D,S80249D,S80251D,S80252D,S80259D | |
| | S80261D,S80262D,S80269D,S80271D,S80272D,S80279D,S80811D | |
| | S80812D,S80819D,S80821D,S80822D,S80829D,S80841D,S80842D | |
| | S80849D,S80851D,S80852D,S80859D,S80861D,S80862D,S80869D | |
| | S80871D,S80872D,S80879D,S80911D,S80912D,S80919D,S80921D | |
| | S80922D,S80929D,S9000XD,S9001XD,S9002XD,S90111D,S90112D | |
| | S90119D,S90121D,S90122D,S90129D,S90211D,S90212D,S90219D | |
| | S90221D,S90222D,S90229D,S9030XD,S9031XD,S9032XD,S90411D | |
| | S90412D,S90413D,S90414D,S90415D,S90416D,S90421D,S90422D | |
| | S90423D,S90424D,S90425D,S90426D,S90441D,S90442D,S90443D | |
| | S90444D,S90445D,S90446D,S90451D,S90452D,S90453D,S90454D | |
| | S90455D,S90456D,S90461D,S90462D,S90463D,S90464D,S90465D | |
| | S90466D,S90471D,S90472D,S90473D,S90474D,S90475D,S90476D | |
| | S90511D,S90512D,S90519D,S90521D,S90522D,S90529D,S90541D | |
| | S90542D,S90549D,S90551D,S90552D,S90559D,S90561D,S90562D | |
| | S90569D,S90571D,S90572D,S90579D,S90811D,S90812D,S90819D | |
| | S90821D,S90822D,S90829D,S90841D,S90842D,S90849D,S90851D | |
| | S90852D,S90859D,S90861D,S90862D,S90869D,S90871D,S90872D | |
| | S90879D,S90911D,S90912D,S90919D,S90921D,S90922D,S90929D | |
| | S90931D,S90932D,S90933D,S90934D,S90935D,S90936D | |

Continued on next page

Table S9 (continued).

| Diagnosis | ICD-10 | ICD-9 |
|---|---|---|
| Symptoms of mental and substance use conditions | R450,R451,R452,R453,R454,R455,R456,R457,R4581,R4582,R4583 R4584,R45850,R4586,R4587,R4589,R460,R461,R462,R463,R464,R465 R466,R467,R4681,R4689 | 3009,3079,3089,78095,78099,79921,79922, 79923,79924,79925 79929,79989,V4039,V6285 |
| Syncope | G9001,R55 | 33701,7802 |
| Systemic lupus erythematosus and connective tissue disorders | H01121,H01122,H01123,H01124,H01125,H01126,H01129,L930,L931 L932,M300,M301,M302,M303,M308,M310,M311,M3110,M3111,M3119 M312,M3130,M3131,M314,M315,M316,M317,M318,M319,M320,M3210 M3211,M3212,M3213,M3214,M3215,M3219,M328,M329,M3300,M3301 M3302,M3303,M3309,M3310,M3311,M3312,M3313,M3319,M3320,M3321 M3322,M3329,M3390,M3391,M3392,M3393,M3399,M340,M341,M342 M3481,M3482,M3483,M3489,M349,M3500,M3501,M3502,M3503,M3504 M3505,M3506,M3507,M3508,M3509,M350A,M350B,M350C,M351,M352 M353,M354,M355,M356,M357,M358,M3581,M3589,M359,M360,M368 | 1361,3574,3596,37334,4200,42491,4460, 4461,44620,44621,44629 4463,4464,4465,4466,4467,4475,5172, 5178,58381,6954,7100,7101 7102,7103,7104,7105,7108,7109,71120, 725,7285,72889,72930 |
| Tendon and synovial disorders | M1220,M12211,M12212,M12219,M12221,M12222,M12229,M12231,M12232 M12239,M12241,M12242,M12249,M12251,M12252,M12259,M12261 M12262,M12269,M12271,M12272,M12279,M1228,M1229,M6500,M65011 M65012,M65019,M65021,M65022,M65029,M65031,M65032,M65039 M65041,M65042,M65049,M65051,M65052,M65059,M65061,M65062 M65069,M65071,M65072,M65079,M6508,M6510,M65111,M65112,M65119 M65121,M65122,M65129,M65131,M65132,M65139,M65141,M65142 M65149,M65151,M65152,M65159,M65161,M65162,M65169,M65171 M65172,M65179,M6518,M6519,M6520,M65221,M65222,M65229,M65231 M65232,M65239,M65241,M65242,M65249,M65251,M65252,M65259 M65261,M65262,M65269,M65271,M65272,M65279,M6528,M6529,M6530 M65311,M65312,M65319,M65321,M65322,M65329,M65331,M65332 M65339,M65341,M65342,M65349,M65351,M65352,M65359,M654,M6580 M65811,M65812,M65819,M65821,M65822,M65829,M65831,M65832 M65839,M65841,M65842,M65849,M65851,M65852,M65859,M65861 M65862,M65869,M65871,M65872,M65879,M6588,M6589,M659,M660 M6610,M66111,M66112,M66119,M66121,M66122,M66129,M66131,M66132 M66139,M66141,M66142,M66143,M66144,M66145,M66146,M66151 M66152,M66159,M66171,M66172,M66173,M66174,M66175,M66176 M66177,M66178,M66179,M6618,M6620,M66211,M66212,M66219,M66221 M66222,M66229,M66231,M66232,M66239,M66241,M66242,M66249 M66251,M66252,M66259,M66261,M66262,M66269,M66271,M66272 M66279,M6628,M6629,M6630,M66311,M66312,M66319,M66321,M66322 M66329,M66331,M66332,M66339,M66341,M66342,M66349,M66351 M66352,M66359,M66361,M66362,M66369,M66371,M66372,M66379,M6638 M6639,M6680,M66811,M66812,M66819,M66821,M66822,M66829,M66831 M66832,M66839,M66841,M66842,M66849,M66851,M66852,M66859 M66861,M66862,M66869,M66871,M66872,M66879,M6688,M6689,M669 M6700,M6701,M6702,M6720,M67211,M67212,M67219,M67221,M67222 M67229,M67231,M67232,M67239,M67241,M67242,M67249,M67251 M67252,M67259,M67261,M67262,M67269,M67271,M67272,M67279,M6728 M6729,M6730,M67311,M67312,M67319,M67321,M67322,M67329,M67331 M67332,M67339,M67341,M67342,M67349,M67351,M67352,M67359 M67361,M67362,M67369,M67371,M67372,M67379,M6738,M6739,M6740 M67411,M67412,M67419,M67421,M67422,M67429,M67431,M67432 M67439,M67441,M67442,M67449,M67451,M67452,M67459,M67461 M67462,M67469,M67471,M67472,M67479,M6748,M6749,M6750,M6751 M6752,M6780,M67811,M67812,M67813,M67814,M67819,M67821,M67822 M67823,M67824,M67829,M67831,M67832,M67833,M67834,M67839 M67841,M67842,M67843,M67844,M67849,M67851,M67852,M67853 M67854,M67859,M67861,M67862,M67863,M67864,M67869,M67871 M67872,M67873,M67874,M67879,M6788,M6789,M6790,M67911,M67912 M67919,M67921,M67922,M67929,M67931,M67932,M67939,M67941 M67942,M67949,M67951,M67952,M67959,M67961,M67962,M67969 M67971,M67972,M67979,M6798,M6799,M70031,M70032,M70039,M70041 M70042,M70049,M7120,M7121,M7122,M75100,M75101,M75102,M75110 M75111,M75112,M75120,M75121,M75122,M7520,M7521,M7522,M7530 M7531,M7532,M7600,M7601,M7602,M7610,M7611,M7612,M7650,M7651 M7652,M7660,M7661,M7662,M7670,M7671,M7672,M76821,M76822 M76829,M76891,M76892,M76899,M769,M7750,M7751,M7752,M778,M779 | 71920,71921,71922,71923,71924,71925, 71926,71927,71928,71929 72610,72611,72612,72613,7262,7265, 72664,72669,72670,72671 72672,72679,7268,72690,72700,72701, 72703,72704,72705,72706 72709,7272,72741,72742,72743,72750, 72751,72759,72760,72761 72762,72763,72764,72765,72766,72767, 72768,72769,72781,72782 72783,72789,7279 |
| Thyroid disorders | A1881,B6731,E000,E001,E002,E009,E010,E011,E012,E018,E02,E030 E031,E032,E033,E034,E035,E038,E039,E040,E041,E042,E048,E049 E0500,E0501,E0510,E0511,E0520,E0521,E0530,E0531,E0540,E0541 E0580,E0581,E0590,E0591,E060,E061,E062,E063,E064,E065,E069 E070,E071,E0781,E0789,E079,E890,O905 | 01750,1222,2400,2409,2410,2411,2419, 24200,24201,24210,24211 24220,24221,24230,24231,24240,24241, 24280,24281,24290,24291 243,2440,2441,2442,2443,2448,2449,2450, 2451,2452,2453,2454 2458,2459,2460,2461,2462,2463,2468, 2469,64812,64814,78001 79094 |

Continued on next page

**Table S9 (continued).**

| Diagnosis | ICD-10 | ICD-9 |
|---|---|---|
| Tobacco-related | F17200,F17203,F17208,F17209,F17210,F17213,F17218,F17219 | 2920,29289,2929,3051,64900,64901,64902,<br>64903,64904 |
| disorders | F17220,F17223,F17228,F17229,F17290,F17293,F17298,F17299<br>O99330,O99331,O99332,O99333,O99334,O99335 | |
| Toxic effects, | T510X1A,T510X2A,T510X3A,T510X4A,T511X1A,T511X2A,T511X3A | 9800,9801,9802,9803,9808,9809,981,9820,<br>9821,9822,9823,9824 |
| initial encounter | T511X4A,T512X1A,T512X2A,T512X3A,T512X4A,T513X1A,T513X2A | 9828,9830,9831,9832,9839,9849,9850,<br>9851,9852,9853,9855,9856 |
| | T513X3A,T513X4A,T518X1A,T518X2A,T518X3A,T518X4A,T5191XA | 9858,9859,986,9871,9872,9873,9874,9875,<br>9876,9877,9878,9879 |
| | T5192XA,T5193XA,T5194XA,T520X1A,T520X2A,T520X3A,T520X4A | 9880,9881,9882,9888,9889,9890,9891,<br>9893,9894,9895,9896,9897 |
| | T521X1A,T521X2A,T521X3A,T521X4A,T522X1A,T522X2A,T522X3A | 98982,98984,98989,9899,E8601,E8602,<br>E8603,E8604,E8608,E8609 |
| | T522X4A,T523X1A,T523X2A,T523X3A,T523X4A,T524X1A,T524X2A | E8610,E8611,E8616,E8621,E8624,E8631,<br>E8634,E8635,E8637,E8640 |
| | T524X3A,T524X4A,T528X1A,T528X2A,T528X3A,T528X4A,T5291XA | E8641,E8643,E8644,E8651,E8652,E8653,<br>E8654,E8655,E8658,E8659 |
| | T5292XA,T5293XA,T5294XA,T530X1A,T530X2A,T530X3A,T530X4A | E8660,E8661,E8663,E8664,E8668,E8681,<br>E8682,E8683,E8688,E8689 |
| | T531X1A,T531X2A,T531X3A,T531X4A,T532X1A,T532X2A,T532X3A | E8690,E8691,E8692,E8693,E8698,E9050,<br>E9051,E9052,E9053,E9054 |
| | T532X4A,T533X1A,T533X2A,T533X3A,T533X4A,T534X1A,T534X2A | E9055,E9056,E9241,E9286,E9506,E9507,<br>E9508,E9509,E9520,E9521 |
| | T534X3A,T534X4A,T535X1A,T535X2A,T535X3A,T535X4A,T536X1A | E9528,E9529,E961,E9621,E9622,E9629,<br>E9806,E9807,E9808,E9809 |
| | T536X2A,T536X3A,T536X4A,T537X1A,T537X2A,T537X3A,T537X4A<br>T5391XA,T5392XA,T5393XA,T5394XA,T540X1A,T540X2A,T540X3A<br>T540X4A,T541X1A,T541X2A,T541X3A,T541X4A,T542X1A,T542X2A<br>T542X3A,T542X4A,T543X1A,T543X2A,T543X3A,T543X4A,T5491XA<br>T5492XA,T5493XA,T5494XA,T550X1A,T550X2A,T550X3A,T550X4A<br>T551X1A,T551X2A,T551X3A,T551X4A,T560X1A,T560X2A,T560X3A<br>T560X4A,T561X1A,T561X2A,T561X3A,T561X4A,T562X1A,T562X2A<br>T562X3A,T562X4A,T563X1A,T563X2A,T563X3A,T563X4A,T564X1A<br>T564X2A,T564X3A,T564X4A,T565X1A,T565X2A,T565X3A,T565X4A<br>T566X1A,T566X2A,T566X3A,T566X4A,T567X1A,T567X2A,T567X3A<br>T567X4A,T56811A,T56812A,T56813A,T56814A,T56891A,T56892A<br>T56893A,T56894A,T5691XA,T5692XA,T5693XA,T5694XA,T570X1A<br>T570X2A,T570X3A,T570X4A,T571X1A,T571X2A,T571X3A,T571X4A<br>T572X1A,T572X2A,T572X3A,T572X4A,T573X1A,T573X2A,T573X3A<br>T573X4A,T578X1A,T578X2A,T578X3A,T578X4A,T5791XA,T5792XA<br>T5793XA,T5794XA,T5801XA,T5802XA,T5803XA,T5804XA,T5811XA<br>T5812XA,T5813XA,T5814XA,T582X1A,T582X2A,T582X3A,T582X4A<br>T588X1A,T588X2A,T588X3A,T588X4A,T5891XA,T5892XA,T5893XA<br>T5894XA,T590X1A,T590X2A,T590X3A,T590X4A,T591X1A,T591X2A<br>T591X3A,T591X4A,T592X1A,T592X2A,T592X3A,T592X4A,T593X1A<br>T593X2A,T593X3A,T593X4A,T594X1A,T594X2A,T594X3A,T594X4A<br>T595X1A,T595X2A,T595X3A,T595X4A,T596X1A,T596X2A,T596X3A<br>T596X4A,T597X1A,T597X2A,T597X3A,T597X4A,T59811A,T59812A<br>T59813A,T59814A,T59891A,T59892A,T59893A,T59894A,T5991XA<br>T5992XA,T5993XA,T5994XA,T600X1A,T600X2A,T600X3A,T600X4A<br>T601X1A,T601X2A,T601X3A,T601X4A,T602X1A,T602X2A,T602X3A<br>T602X4A,T603X1A,T603X2A,T603X3A,T603X4A,T604X1A,T604X2A<br>T604X3A,T604X4A,T608X1A,T608X2A,T608X3A,T608X4A,T6091XA<br>T6092XA,T6093XA,T6094XA,T6101XA,T6102XA,T6103XA,T6104XA<br>T6111XA,T6112XA,T6113XA,T6114XA,T61771A,T61772A,T61773A<br>T61774A,T61781A,T61782A,T61783A,T61784A,T618X1A,T618X2A<br>T618X3A,T618X4A,T6191XA,T6192XA,T6193XA,T6194XA,T620X1A<br>T620X2A,T620X3A,T620X4A,T621X1A,T621X2A,T621X3A,T621X4A<br>T622X1A,T622X2A,T622X3A,T622X4A,T628X1A,T628X2A,T628X3A<br>T628X4A,T6291XA,T6292XA,T6293XA,T6294XA,T63001A,T63002A<br>T63003A,T63004A,T63011A,T63012A,T63013A,T63014A,T63021A<br>T63022A,T63023A,T63024A,T63031A,T63032A,T63033A,T63034A<br>T63041A,T63042A,T63043A,T63044A,T63061A,T63062A,T63063A<br>T63064A,T63071A,T63072A,T63073A,T63074A,T63081A,T63082A<br>T63083A,T63084A,T63091A,T63092A,T63093A,T63094A,T63111A<br>T63112A,T63113A,T63114A,T63121A,T63122A,T63123A,T63124A<br>T63191A,T63192A,T63193A,T63194A,T632X1A,T632X2A,T632X3A<br>T632X4A,T63301A,T63302A,T63303A,T63304A,T63311A,T63312A<br>T63313A,T63314A,T63321A,T63322A,T63323A,T63324A,T63331A<br>T63332A,T63333A,T63334A,T63391A,T63392A,T63393A,T63394A<br>T63411A,T63412A,T63413A,T63414A,T63421A,T63422A,T63423A<br>T63424A,T63431A,T63432A,T63433A,T63434A,T63441A,T63442A<br>T63443A,T63444A,T63451A,T63452A,T63453A,T63454A,T63461A<br>T63462A,T63463A,T63464A,T63481A,T63482A,T63483A,T63484A<br>T63511A,T63512A,T63513A,T63514A,T63591A,T63592A,T63593A<br>T63594A,T63611A,T63612A,T63613A,T63614A,T63621A,T63622A<br>T63623A,T63624A,T63631A,T63632A,T63633A,T63634A,T63691A<br>T63692A,T63693A,T63694A,T63711A,T63712A,T63713A,T63714A<br>T63791A,T63792A,T63793A,T63794A,T63811A,T63812A,T63813A<br>T63814A,T63821A,T63822A,T63823A,T63824A,T63831A,T63832A<br>T63833A,T63834A,T63891A,T63892A,T63893A,T63894A,T6391XA<br>T6392XA,T6393XA,T6394XA,T6401XA,T6402XA,T6403XA,T6404XA<br>T6481XA,T6482XA,T6483XA,T6484XA,T650X1A,T650X2A,T650X3A<br>T650X4A,T651X1A,T651X2A,T651X3A,T651X4A,T65211A,T65212A | E9820,E9821,E9828 |

Continued on next page

Table S9 (continued).

| Diagnosis | ICD-10 | ICD-9 |
|---|---|---|
| | T65213A,T65214A,T65221A,T65222A,T65223A,T65224A,T65291A T65292A,T65293A,T65294A,T653X1A,T653X2A,T653X3A,T653X4A T654X1A,T654X2A,T654X3A,T654X4A,T655X1A,T655X2A,T655X3A T655X4A,T656X1A,T656X2A,T656X3A,T656X4A,T65811A,T65812A T65813A,T65814A,T65821A,T65822A,T65823A,T65824A,T65831A T65832A,T65833A,T65834A,T65891A,T65892A,T65893A,T65894A T6591XA,T6592XA,T6593XA,T6594XA | |
| Toxic effects, subsequent encounter | T510X1D,T510X2D,T510X3D,T510X4D,T511X1D,T511X2D,T511X3D T511X4D,T512X1D,T512X2D,T512X3D,T512X4D,T513X1D,T513X2D T513X3D,T513X4D,T518X1D,T518X2D,T518X3D,T518X4D,T5191XD T5192XD,T5193XD,T5194XD,T520X1D,T520X2D,T520X3D,T520X4D T521X1D,T521X2D,T521X3D,T521X4D,T522X1D,T522X2D,T522X3D T522X4D,T523X1D,T523X2D,T523X3D,T523X4D,T524X1D,T524X2D T524X3D,T524X4D,T528X1D,T528X2D,T528X3D,T528X4D,T5291XD T5292XD,T5293XD,T5294XD,T530X1D,T530X2D,T530X3D,T530X4D T531X1D,T531X2D,T531X3D,T531X4D,T532X1D,T532X2D,T532X3D T532X4D,T533X1D,T533X2D,T533X3D,T533X4D,T534X1D,T534X2D T534X3D,T534X4D,T535X1D,T535X2D,T535X3D,T535X4D,T536X1D T536X2D,T536X3D,T536X4D,T537X1D,T537X2D,T537X3D,T537X4D T5391XD,T5392XD,T5393XD,T5394XD,T540X1D,T540X2D,T540X3D T540X4D,T541X1D,T541X2D,T541X3D,T541X4D,T542X1D,T542X2D T542X3D,T542X4D,T543X1D,T543X2D,T543X3D,T543X4D,T5491XD T5492XD,T5493XD,T5494XD,T550X1D,T550X2D,T550X3D,T550X4D T551X1D,T551X2D,T551X3D,T551X4D,T560X1D,T560X2D,T560X3D T560X4D,T561X1D,T561X2D,T561X3D,T561X4D,T562X1D,T562X2D T562X3D,T562X4D,T563X1D,T563X2D,T563X3D,T563X4D,T564X1D T564X2D,T564X3D,T564X4D,T565X1D,T565X2D,T565X3D,T565X4D T566X1D,T566X2D,T566X3D,T566X4D,T567X1D,T567X2D,T567X3D T567X4D,T56811D,T56812D,T56813D,T56814D,T56891D,T56892D T56893D,T56894D,T5691XD,T5692XD,T5693XD,T5694XD,T570X1D T570X2D,T570X3D,T570X4D,T571X1D,T571X2D,T571X3D,T571X4D T572X1D,T572X2D,T572X3D,T572X4D,T573X1D,T573X2D,T573X3D T573X4D,T578X1D,T578X2D,T578X3D,T578X4D,T5791XD,T5792XD T5793XD,T5794XD,T5801XD,T5802XD,T5803XD,T5804XD,T5811XD T5812XD,T5813XD,T5814XD,T582X1D,T582X2D,T582X3D,T582X4D T588X1D,T588X2D,T588X3D,T588X4D,T5891XD,T5892XD,T5893XD T5894XD,T590X1D,T590X2D,T590X3D,T590X4D,T591X1D,T591X2D T591X3D,T591X4D,T592X1D,T592X2D,T592X3D,T592X4D,T593X1D T593X2D,T593X3D,T593X4D,T594X1D,T594X2D,T594X3D,T594X4D T595X1D,T595X2D,T595X3D,T595X4D,T596X1D,T596X2D,T596X3D T596X4D,T597X1D,T597X2D,T597X3D,T597X4D,T59811D,T59812D T59813D,T59814D,T59891D,T59892D,T59893D,T59894D,T5991XD T5992XD,T5993XD,T5994XD,T600X1D,T600X2D,T600X3D,T600X4D T601X1D,T601X2D,T601X3D,T601X4D,T602X1D,T602X2D,T602X3D T602X4D,T603X1D,T603X2D,T603X3D,T603X4D,T604X1D,T604X2D T604X3D,T604X4D,T608X1D,T608X2D,T608X3D,T608X4D,T6091XD T6092XD,T6093XD,T6094XD,T6101XD,T6102XD,T6103XD,T6104XD T6111XD,T6112XD,T6113XD,T6114XD,T61771D,T61772D,T61773D T61774D,T61781D,T61782D,T61783D,T61784D,T618X1D,T618X2D T618X3D,T618X4D,T6191XD,T6192XD,T6193XD,T6194XD,T620X1D T620X2D,T620X3D,T620X4D,T621X1D,T621X2D,T621X3D,T621X4D T622X1D,T622X2D,T622X3D,T622X4D,T628X1D,T628X2D,T628X3D T628X4D,T6291XD,T6292XD,T6293XD,T6294XD,T63001D,T63002D T63003D,T63004D,T63011D,T63012D,T63013D,T63014D,T63021D T63022D,T63023D,T63024D,T63031D,T63032D,T63033D,T63034D T63041D,T63042D,T63043D,T63044D,T63061D,T63062D,T63063D T63064D,T63071D,T63072D,T63073D,T63074D,T63081D,T63082D T63083D,T63084D,T63091D,T63092D,T63093D,T63094D,T63111D T63112D,T63113D,T63114D,T63121D,T63122D,T63123D,T63124D T63191D,T63192D,T63193D,T63194D,T632X1D,T632X2D,T632X3D T632X4D,T63301D,T63302D,T63303D,T63304D,T63311D,T63312D T63313D,T63314D,T63321D,T63322D,T63323D,T63324D,T63331D T63332D,T63333D,T63334D,T63391D,T63392D,T63393D,T63394D T63411D,T63412D,T63413D,T63414D,T63421D,T63422D,T63423D T63424D,T63431D,T63432D,T63433D,T63434D,T63441D,T63442D T63443D,T63444D,T63451D,T63452D,T63453D,T63454D,T63461D T63462D,T63463D,T63464D,T63481D,T63482D,T63483D,T63484D T63511D,T63512D,T63513D,T63514D,T63591D,T63592D,T63593D T63594D,T63611D,T63612D,T63613D,T63614D,T63621D,T63622D T63623D,T63624D,T63631D,T63632D,T63633D,T63634D,T63691D T63692D,T63693D,T63694D,T63711D,T63712D,T63713D,T63714D T63791D,T63792D,T63793D,T63794D,T63811D,T63812D,T63813D T63814D,T63821D,T63822D,T63823D,T63824D,T63831D,T63832D T63833D,T63834D,T63891D,T63892D,T63893D,T63894D,T6391XD T6392XD,T6393XD,T6394XD,T6401XD,T6402XD,T6403XD,T6404XD T6481XD,T6482XD,T6483XD,T6484XD,T650X1D,T650X2D,T650X3D T650X4D,T651X1D,T651X2D,T651X3D,T651X4D,T65211D,T65212D T65213D,T65214D,T65221D,T65222D,T65223D,T65224D,T65291D T65292D,T65293D,T65294D,T653X1D,T653X2D,T653X3D,T653X4D T654X1D,T654X2D,T654X3D,T654X4D,T655X1D,T655X2D,T655X3D T655X4D,T656X1D,T656X2D,T656X3D,T656X4D,T65811D,T65812D T65813D,T65814D,T65821D,T65822D,T65823D,T65824D,T65831D T65832D,T65833D,T65834D,T65891D,T65892D,T65893D,T65894D T6591XD,T6592XD,T6593XD,T6594XD | V5889 |

Continued on next page

**Table S9 (continued).**

| Diagnosis | ICD-10 | ICD-9 |
|---|---|---|
| Transient cerebral ischemia | G450,G451,G452,G453,G454,G458,G459 | 36234,4350,4351,4352,4353,4358,4359,<br>4377 |
| Trauma- and stressor-related disorders | F430,F4310,F4311,F4312,F4320,F4321,F4322,F4323,F4324,F4325<br>F4329,F438,F4381,F4389,F439,F440,F441,F442,F444,F445,F446<br>F447,F4481,F4489,F449,F481,F941,F942 | 2982,30011,30012,30013,30014,30015,<br>30016,30019,3006,3089,3090<br>3091,30924,30928,30929,3093,3094,30981,<br>30989,3099,31389 |
| Traumatic arthropathy | M1250,M12511,M12512,M12519,M12521,M12522,M12529,M12531,M12532<br>M12539,M12541,M12542,M12549,M12551,M12552,M12559,M12561<br>M12562,M12569,M12571,M12572,M12579,M1258,M1259 | 71610,71611,71612,71613,71614,71615,<br>71616,71617,71618,71619 |
| Traumatic brain injury (TBI); concussion, initial encounter | S060X0A,S060X1A,S060X2A,S060X3A,S060X4A,S060X5A,S060X6A<br>S060X7A,S060X8A,S060X9A,S060XAA,S061X0A,S061X1A,S061X2A<br>S061X3A,S061X4A,S061X5A,S061X6A,S061X7A,S061X8A,S061X9A<br>S061XAA,S062X0A,S062X1A,S062X2A,S062X3A,S062X4A,S062X5A<br>S062X6A,S062X7A,S062X8A,S062X9A,S062XAA,S06300A,S06301A<br>S06302A,S06303A,S06304A,S06305A,S06306A,S06307A,S06308A<br>S06309A,S0630AA,S06310A,S06311A,S06312A,S06313A,S06314A<br>S06315A,S06316A,S06317A,S06318A,S06319A,S0631AA,S06320A<br>S06321A,S06322A,S06323A,S06324A,S06325A,S06326A,S06327A<br>S06328A,S06329A,S0632AA,S06330A,S06331A,S06332A,S06333A<br>S06334A,S06335A,S06336A,S06337A,S06338A,S06339A,S0633AA<br>S06340A,S06341A,S06342A,S06343A,S06344A,S06345A,S06346A<br>S06347A,S06348A,S06349A,S0634AA,S06350A,S06351A,S06352A<br>S06353A,S06354A,S06355A,S06356A,S06357A,S06358A,S06359A<br>S0635AA,S06360A,S06361A,S06362A,S06363A,S06364A,S06365A<br>S06366A,S06367A,S06368A,S06369A,S0636AA,S06370A,S06371A<br>S06372A,S06373A,S06374A,S06375A,S06376A,S06377A,S06378A<br>S06379A,S0637AA,S06380A,S06381A,S06382A,S06383A,S06384A<br>S06385A,S06386A,S06387A,S06388A,S06389A,S0638AA,S064X0A<br>S064X1A,S064X2A,S064X3A,S064X4A,S064X5A,S064X6A,S064X7A<br>S064X8A,S064X9A,S064XAA,S065X0A,S065X1A,S065X2A,S065X3A<br>S065X4A,S065X5A,S065X6A,S065X7A,S065X8A,S065X9A,S065XAA<br>S066X0A,S066X1A,S066X2A,S066X3A,S066X4A,S066X5A,S066X6A<br>S066X7A,S066X8A,S066X9A,S066XAA,S06810A,S06811A,S06812A<br>S06813A,S06814A,S06815A,S06816A,S06817A,S06818A,S06819A<br>S0681AA,S06820A,S06821A,S06822A,S06823A,S06824A,S06825A<br>S06826A,S06827A,S06828A,S06829A,S0682AA,S06890A,S06891A<br>S06892A,S06893A,S06894A,S06895A,S06896A,S06897A,S06898A<br>S06899A,S0689AA,S068A0A,S068A1A,S068A2A,S068A3A,S068A4A<br>S068A5A,S068A6A,S068A7A,S068A8A,S068A9A,S068AAA,S069X0A<br>S069X1A,S069X2A,S069X3A,S069X4A,S069X5A,S069X6A,S069X7A<br>S069X8A,S069X9A,S069XAA,S06A0XA,S06A1XA | 3485,8500,85011,85012,8502,8503,8504,<br>8505,85141,85142,85143<br>85144,85145,85146,85161,85162,85163,<br>85164,85165,85166,85181<br>85182,85183,85184,85185,85186,85201,<br>85202,85203,85204,85205<br>85206,85221,85222,85223,85224,85225,<br>85226,85241,85242,85243<br>85244,85245,85246,85301,85302,85303,<br>85304,85305,85306,85401<br>85402,85403,85404,85405,85406 |
| Traumatic brain injury (TBI); concussion, subsequent encounter | S060X0D,S060X1D,S060X2D,S060X3D,S060X4D,S060X5D,S060X6D<br>S060X7D,S060X8D,S060X9D,S060XAD,S061X0D,S061X1D,S061X2D<br>S061X3D,S061X4D,S061X5D,S061X6D,S061X7D,S061X8D,S061X9D<br>S061XAD,S062X0D,S062X1D,S062X2D,S062X3D,S062X4D,S062X5D<br>S062X6D,S062X7D,S062X8D,S062X9D,S062XAD,S06300D,S06301D<br>S06302D,S06303D,S06304D,S06305D,S06306D,S06307D,S06308D<br>S06309D,S0630AD,S06310D,S06311D,S06312D,S06313D,S06314D<br>S06315D,S06316D,S06317D,S06318D,S06319D,S0631AD,S06320D<br>S06321D,S06322D,S06323D,S06324D,S06325D,S06326D,S06327D<br>S06328D,S06329D,S0632AD,S06330D,S06331D,S06332D,S06333D<br>S06334D,S06335D,S06336D,S06337D,S06338D,S06339D,S0633AD<br>S06340D,S06341D,S06342D,S06343D,S06344D,S06345D,S06346D<br>S06347D,S06348D,S06349D,S0634AD,S06350D,S06351D,S06352D<br>S06353D,S06354D,S06355D,S06356D,S06357D,S06358D,S06359D<br>S0635AD,S06360D,S06361D,S06362D,S06363D,S06364D,S06365D<br>S06366D,S06367D,S06368D,S06369D,S0636AD,S06370D,S06371D<br>S06372D,S06373D,S06374D,S06375D,S06376D,S06377D,S06378D<br>S06379D,S0637AD,S06380D,S06381D,S06382D,S06383D,S06384D<br>S06385D,S06386D,S06387D,S06388D,S06389D,S0638AD,S064X0D<br>S064X1D,S064X2D,S064X3D,S064X4D,S064X5D,S064X6D,S064X7D<br>S064X8D,S064X9D,S064XAD,S065X0D,S065X1D,S065X2D,S065X3D<br>S065X4D,S065X5D,S065X6D,S065X7D,S065X8D,S065X9D,S065XAD<br>S066X0D,S066X1D,S066X2D,S066X3D,S066X4D,S066X5D,S066X6D<br>S066X7D,S066X8D,S066X9D,S066XAD,S06810D,S06811D,S06812D<br>S06813D,S06814D,S06815D,S06816D,S06817D,S06818D,S06819D<br>S0681AD,S06820D,S06821D,S06822D,S06823D,S06824D,S06825D<br>S06826D,S06827D,S06828D,S06829D,S0682AD,S06890D,S06891D<br>S06892D,S06893D,S06894D,S06895D,S06896D,S06897D,S06898D<br>S06899D,S0689AD,S068A0D,S068A1D,S068A2D,S068A3D,S068A4D<br>S068A5D,S068A6D,S068A9D,S068AAD,S069X0D,S069X1D,S069X2D<br>S069X3D,S069X4D,S069X5D,S069X6D,S069X7D,S069X8D,S069X9D<br>S069XAD,S06A0XD,S06A1XD | V5889 |

Continued on next page

Table S9 (continued).

| Diagnosis | ICD-10 | ICD-9 |
|---|---|---|
| Tuberculosis | A150,A154,A155,A156,A157,A158,A159,A170,A171,A1781,A1782 A1783,A1789,A179,A1801,A1802,A1803,A1809,A1810,A1811,A1812 A1813,A1814,A1815,A1816,A1817,A1818,A182,A1831,A1832,A1839 A184,A1850,A1851,A1852,A1853,A1854,A1859,A186,A187,A1881 A1882,A1883,A1884,A1885,A1889,A190,A191,A192,A198,A199,J65 O98011,O98012,O98013,O98019,O9802,O9803,P370 | 01090,01130,01190,01200,01210,01220, 01230,01280,01300,01310 01320,01330,01340,01350,01360,01362, 01380,01400,01480,01500 01510,01520,01560,01570,01580,01590, 01600,01610,01620,01630 01650,01660,01670,01690,01700,01710, 01720,01730,01740,01750 01760,01770,01780,01790,01800,01880, 01890,36313,36411,37031 37059,37909,4200,4220,42491,4258,505, 6014,64730,64731,64732 64733,64734,71145,71146,71148,72081, 73088,73740,7712 |
| Uncomplicated pregnancy, delivery or puerperium | O4202,O4212,O4292,O6020X0,O6020X1,O6020X2,O6020X3,O6020X4 O6020X5,O6020X9,O6022X0,O6022X1,O6022X2,O6022X3,O6022X4 O6022X5,O6022X9,O6023X0,O6023X1,O6023X2,O6023X3,O6023X4 O6023X5,O6023X9,O80,Z3201,Z331,Z333,Z3400,Z3401,Z3402,Z3403 Z3480,Z3481,Z3482,Z3483,Z3490,Z3491,Z3492,Z3493,Z390,Z391 Z392 | 64420,64421,650,65811,65821,V220,V221, V222,V240,V241,V242 V7242 |
| Urinary incontinence | F980,F981,N393,N3941,N3942,N3943,N3944,N3945,N3946,N39490 N39491,N39492,N39498,R32,R3981 | 3076,3077,6256,78830,78831,78832,78833, 78834,78835,78836 78837,78838,78839,78891 |
| Urinary system cancers - all other types | C688,C689,D0910,D0919 | 1898,1899,2339 |
| Urinary system cancers - bladder | C670,C671,C672,C673,C674,C675,C676,C677,C678,C679,D090 | 1880,1881,1882,1883,1884,1885,1886, 1887,1888,1889,2337 |
| Urinary system cancers - kidney | C641,C642,C649 | 1890 |
| Urinary system cancers - ureter and renal pelvis | C651,C652,C659,C661,C662,C669 | 1891,1892 |
| Urinary system cancers - urethra | C680,C681 | 1893,1894 |
| Urinary tract infections | A0225,A1810,A1813,A3685,A5275,A5401,A5601,A5903,B3741,B3749 N10,N110,N111,N118,N119,N12,N136,N151,N3000,N3001,N3010,N3011 N3020,N3021,N3030,N3031,N3040,N3041,N3080,N3081,N3090,N3091 N340,N341,N342,N343,N390,O2300,O2301,O2302,O2303,O2310,O2311 O2312,O2313,O2320,O2321,O2322,O2323,O2330,O2331,O2332,O2333 O2340,O2341,O2342,O2343,O8620,O8621,O8622,O8629 | 00329,01630,01690,03284,0954,09811, 09831,09940,09953,1122 13102,59000,59001,59010,59011,5902, 59080,5933,5934,5950,5951 5952,5953,5954,59581,59582,59589,5959, 5970,59780,59781,59789 5990,64660,64661,64663,64664 |
| Uveitis and ocular inflammation | H15001,H15002,H15003,H15009,H15011,H15012,H15013,H15019 H15021,H15022,H15023,H15029,H15031,H15032,H15033,H15039 H15041,H15042,H15043,H15049,H15051,H15052,H15053,H15059 H15091,H15092,H15093,H15099,H15101,H15102,H15103,H15109 H15111,H15112,H15113,H15119,H15121,H15122,H15123,H15129,H2000 H20011,H20012,H20013,H20019,H20021,H20022,H20023,H20029 H20031,H20032,H20033,H20039,H20041,H20042,H20043,H20049 H20051,H20052,H20053,H20059,H2010,H2011,H2012,H2013,H2020 H2021,H2022,H2023,H20811,H20812,H20813,H20819,H20821,H20822 H20823,H20829,H209,H30001,H30002,H30003,H30009,H30011,H30012 H30013,H30019,H30021,H30022,H30023,H30029,H30031,H30032 H30033,H30039,H30041,H30042,H30043,H30049,H30101,H30102 H30103,H30109,H30111,H30112,H30113,H30119,H30121,H30122 H30123,H30129,H30131,H30132,H30133,H30139,H30141,H30142 H30143,H30149,H3020,H3021,H3022,H3023,H30811,H30812,H30813 H30819,H30891,H30892,H30893,H30899,H3090,H3091,H3092,H3093 H44001,H44002,H44003,H44009,H44011,H44012,H44013,H44019 H44021,H44022,H44023,H44029,H44111,H44112,H44113,H44119 H44121,H44122,H44123,H44129,H44131,H44132,H44133,H44139,H4419 | 36000,36001,36002,36003,36004,36011, 36012,36013,36019,36300 36301,36303,36304,36305,36306,36307, 36308,36310,36311,36312 36313,36314,36315,36320,36321,36322, 36400,36401,36402,36403 36404,36405,36410,36421,36423,36424, 3643,37900,37901,37902 37903,37904,37905,37906,37907,37909 |
| Varicose veins of lower extremity | I83001,I83002,I83003,I83004,I83005,I83008,I83009,I83011 I83012,I83013,I83014,I83015,I83018,I83019,I83021,I83022 I83023,I83024,I83025,I83028,I83029,I8310,I8311,I8312,I83201 I83202,I83203,I83204,I83205,I83208,I83209,I83211,I83212 I83213,I83214,I83215,I83218,I83219,I83221,I83222,I83223 | 4540,4541,4542,4548,4549,67100,67101, 67102,67103,67104 |

Continued on next page

Table S9 (continued).

| Diagnosis | ICD-10 | ICD-9 |
|---|---|---|
| | I83224,I83225,I83228,I83229,I83811,I83812,I83813,I83819 I83891,I83892,I83893,I83899,I8390,I8391,I8392,I8393,O2200 O2201,O2202,O2203,O874 | |
| Vesicoureteral reflux | N1370,N1371,N13721,N13722,N13729,N13731,N13732,N13739 | 59370,59371,59372,59373 |
| Viral infection | A080,A0811,A0819,A082,A0831,A0832,A0839,A084,A088,A6000,A6001 | 00861,00862,00863,00864,00865,00866, 00867,00869,0088,04510 |
| | A6002,A6003,A6004,A6009,A601,A609,A630,A800,A801,A802,A8030 | 04520,04590,0460,04611,04619,0462,0463, 04671,04672,04679,0468 |
| | A8039,A804,A809,A8100,A8101,A8109,A811,A812,A8181,A8182,A8183 | 0469,0470,0471,0478,0479,048,0490,0491, 0498,0499,0500,0501 |
| | A8189,A819,A820,A821,A829,A830,A831,A832,A833,A834,A835,A836 | 0509,05101,05102,0511,0512,0519,0520, 0521,0522,0527,0528,0529 |
| | A838,A839,A840,A841,A848,A8481,A8489,A849,A850,A851,A852,A858 | 0530,05310,05311,05312,05313,05314, 05319,05320,05321,05322 |
| | A86,A870,A871,A872,A878,A879,A880,A881,A888,A89,A90,A91,A920 | 05329,05371,05379,0538,0539,0540,05410, 05411,05412,05413 |
| | A921,A922,A9230,A9231,A9232,A9239,A924,A925,A928,A929,A930 | 05419,0542,0543,05440,05441,05442, 05443,05444,05449,0545,0546 |
| | A931,A932,A938,A94,A950,A951,A959,A960,A961,A962,A968,A969 | 05471,05472,05473,05474,05479,0548, 0549,0550,0551,0552,05571 |
| | A980,A981,A982,A983,A984,A985,A988,A99,B000,B001,B002,B003 | 05579,0559,05600,05601,05609,05671, 05679,0569,0570,0578,0579 |
| | B004,B0050,B0051,B0052,B0053,B0059,B007,B0081,B0082,B0089 | 05810,05811,05812,05821,05829,05881, 05882,05889,05900,05901 |
| | B009,B010,B0111,B0112,B012,B0181,B0189,B019,B020,B021,B0221 | 05909,05910,05911,05912,05919,05920, 05921,05922,0598,0600 |
| | B0222,B0223,B0224,B0229,B0230,B0231,B0232,B0233,B0234,B0239 | 0601,0609,061,0620,0621,0622,0623,0624, 0625,0628,0629,0630 |
| | B027,B028,B029,B03,B04,B050,B051,B052,B053,B054,B0581,B0589 | 0631,0632,0638,0639,064,0650,0651,0652, 0653,0654,0659,0660 |
| | B059,B0600,B0601,B0602,B0609,B0681,B0682,B0689,B069,B070,B078 | 0661,0662,0663,06640,06641,06642,06649, 0668,0669,0700,0701 |
| | B079,B08010,B08011,B0802,B0803,B0804,B0809,B081,B0820,B0821 | 07020,07021,07030,07031,07032,07033, 07041,07049,07051,07052 |
| | B0822,B083,B084,B085,B0860,B0861,B0862,B0869,B0870,B0871 | 07053,07054,07059,0706,07070,07071, 0709,071,0720,0721,0722 |
| | B0872,B0879,B088,B09,B1001,B1009,B1081,B1082,B1089,B150,B159 | 0723,07271,07272,07279,0729,0740,0741, 07420,07421,07422,07423 |
| | B160,B161,B162,B169,B170,B1710,B1711,B172,B178,B179,B180,B181 | 0743,0748,075,0771,0772,0773,0774,0778, 07799,0780,07810,07811 |
| | B182,B188,B189,B190,B1910,B1911,B1920,B1921,B199,B250,B251 | 07812,07819,0782,0784,0785,0786,0787, 07881,07889,0790,0791 |
| | B252,B258,B259,B260,B261,B262,B263,B2681,B2682,B2683,B2684 | 0792,0793,0794,07950,07951,07952,07959, 0796,07981,07982,07983 |
| | B2685,B2689,B269,B2700,B2701,B2702,B2709,B2710,B2711,B2712 | 07989,07999,4660,46611,46619,4800,4801, 4802,4803,4808,4809 |
| | B2719,B2780,B2781,B2782,B2789,B2790,B2791,B2792,B2799,B300 | 4841,4870,5731,5770,64680,64681,64683, 7700,7710,7711,7712 |
| | B301,B302,B303,B308,B309,B330,B331,B3320,B3321,B3322,B3323 B3324,B333,B334,B338,B340,B341,B342,B343,B344,B348,B349,B970 B9710,B9711,B9712,B9719,B9721,B9729,B9730,B9731,B9732,B9733 B9734,B9739,B974,B975,B976,B977,B9781,B9789,J120,J121,J122 J123,J1281,J1289,J129,J203,J204,J205,J206,J207,J210,J211 O2640,O2641,O2642,O2643,P230,P350,P351,P352,P354,P358,P359 | 7908 |
| Counseling related to sexual behavior or orientation | Z700,Z701,Z702,Z703,Z708,Z709 | V6549 |
| Encounter for prophylactic measures (excludes immunization) | Z2911,Z2912,Z2913,Z2914,Z293,Z298,Z299 | |
| Gestational weeks | Z3A00,Z3A01,Z3A08,Z3A09,Z3A10,Z3A11,Z3A12,Z3A13,Z3A14,Z3A15 Z3A16,Z3A17,Z3A18,Z3A19,Z3A20,Z3A21,Z3A22,Z3A23,Z3A24,Z3A25 Z3A26,Z3A27,Z3A28,Z3A29,Z3A30,Z3A31,Z3A32,Z3A33,Z3A34,Z3A35 Z3A36,Z3A37,Z3A38,Z3A39,Z3A40,Z3A41,Z3A42,Z3A49 | NoDx |
| Maternal outcome of | Z370,Z371,Z372,Z373,Z374,Z3750,Z3751,Z3752,Z3753,Z3754,Z3759 | V270,V271,V272,V273,V274,V275,V276, V277,V279 |
| delivery | Z3760,Z3761,Z3762,Z3763,Z3764,Z3769,Z377,Z379 | |
| No immunization or | Z2801,Z2802,Z2803,Z2804,Z2809,Z281,Z2820,Z2821,Z2829,Z283 | V1583,V6400,V6401,V6402,V6403, V6404,V6405,V6406,V6407,V6408 |
| underimmunization | Z28310,Z28311,Z2839,Z2881,Z2882,Z2883,Z2889,Z289 | V6409 |
| Organ transplant | Z940,Z941,Z942,Z943,Z944,Z945,Z946,Z947,Z9481,Z9482,Z9483 | V420,V421,V423,V424,V425,V426,V427, V4281,V4282,V4283,V4284 |
| status | Z9484,Z9489,Z949,Z9885 | V4289,V429,V4587 |
| Resistance to | Z1610,Z1611,Z1612,Z1619,Z1620,Z1621,Z1622,Z1623,Z1624,Z1629 | V090,V091,V092,V093,V094,V0950, V0951,V096,V0970,V0971,V0980 |
| antimicrobial drugs | Z1630,Z1631,Z1632,Z1633,Z16341,Z16342,Z1635,Z1639 | V0981,V0990,V0991 |

Continued on next page

Table S9 (continued).

| Diagnosis | ICD-10 | ICD-9 |
|---|---|---|
| Supervision of | O0900,O0901,O0902,O0903,O0910,O0911,O0912,O0913,O09211,O09212 | 65941,65943,65950,65951,65953,65960, 65961,65963,V230,V231 |
| high-risk pregnancy | O09213,O09219,O09291,O09292,O09293,O09299,O0930,O0931,O0932 | V233,V2341,V2342,V2349,V235,V237, V2381,V2382,V2383,V2384 |
| | O0933,O0940,O0941,O0942,O0943,O09511,O09512,O09513,O09519 O09521,O09522,O09523,O09529,O09611,O09612,O09613,O09619 O09621,O09622,O09623,O09629,O0970,O0971,O0972,O0973,O09811 O09812,O09813,O09819,O09821,O09822,O09823,O09829,O09891 O09892,O09893,O09899,O0990,O0991,O0992,O0993,O09A0,O09A1 O09A2,O09A3 | V2385,V2386,V2389,V239 |
| Any dental condition | A690,A691,B002,B084,B370,B3783,K000,K001,K002,K003,K004,K005 | 0542,0743,101,1120,5200,5201,5202,5203, 5204,5205,5206,5207 |
| including traumatic | K006,K007,K008,K009,K010,K011,K023,K0251,K0252,K0253,K0261 | 5208,5209,52100,52101,52102,52103, 52104,52105,52106,52107 |
| injury | K0262,K0263,K027,K029,K030,K031,K032,K033,K034,K035,K036,K037 | 52108,52110,52120,52130,52140,52141, 52142,5215,5216,5217 |
| | K0381,K0389,K039,K040,K0401,K0402,K041,K042,K043,K044,K045 | 52181,52189,5219,5220,5221,5222,5223, 5224,5225,5226,5227,5228 |
| | K046,K047,K048,K0490,K0499,K0500,K0501,K0510,K0511,K0520 | 5229,52300,52301,52310,52311,52320, 52330,52331,52332,52333 |
| | K0521,K05211,K05212,K05213,K05219,K0522,K05221,K05222,K05223 | 52340,52341,52342,5235,5236,5238,5239, 52400,52401,52402,52403 |
| | K05229,K0530,K0531,K05311,K05312,K05313,K05319,K0532,K05321 | 52404,52405,52406,52407,52409,52410, 52411,52412,52419,52420 |
| | K05322,K05323,K05329,K054,K055,K056,K060,K06010,K06011,K06012 | 52421,52422,52423,52424,52425,52426, 52427,52428,52429,52430 |
| | K06013,K06020,K06021,K06022,K06023,K061,K062,K063,K068,K069 | 52431,52432,52433,52434,52435,52436, 52437,52439,5244,52450 |
| | K080,K08101,K08102,K08103,K08104,K08109,K08111,K08112,K08113 | 52451,52452,52453,52454,52455,52456, 52457,52459,52460,52461 |
| | K08114,K08119,K08121,K08122,K08123,K08124,K08129,K08131 | 52462,52463,52469,52470,52471,52472, 52473,52474,52479,52481 |
| | K08132,K08133,K08134,K08139,K08191,K08192,K08193,K08194 | 52482,52489,5249,5250,52510,52511, 52512,52513,52519,52520 |
| | K08199,K0820,K0821,K0822,K0823,K0824,K0825,K0826,K083,K08401 | 52521,52522,52523,52524,52525,52526, 5253,52540,52541,52542 |
| | K08402,K08403,K08404,K08409,K08411,K08412,K08413,K08414 | 52543,52544,52550,52551,52552,52553, 52554,52560,52561,52562 |
| | K08419,K08421,K08422,K08423,K08424,K08429,K08431,K08432 | 52563,52564,52565,52566,52567,52569, 52571,52572,52573,52579 |
| | K08433,K08434,K08439,K08491,K08492,K08493,K08494,K08499,K0850 | 5258,5259,5260,5261,5262,5263,5264, 5265,52661,52662,52663 |
| | K0851,K0852,K08530,K08531,K08539,K0854,K0855,K0856,K0859,K088 | 52669,52681,52689,5269,5270,5271,5272, 5273,5274,5275,5276 |
| | K0881,K0882,K0889,K089,K090,K091,K098,K099,K110,K111,K1120 | 5277,5278,5279,52800,52801,52802,52809, 5281,5282,5283,5284 |
| | K1121,K1122,K1123,K113,K114,K115,K116,K117,K118,K119,K120 | 5285,5286,52871,52872,52879,5288,5289, 5290,5291,5292,5293 |
| | K121,K122,K1230,K1231,K1232,K1233,K1239,K130,K131,K1321,K1322 | 5294,5295,5296,5298,5299,6820,73382, 78492,80220,80221,80222 |
| | K1323,K1324,K1329,K133,K134,K135,K136,K1370,K1379,K140,K141 | 80223,80224,80225,80226,80227,80228, 80230,80231,80232,80233 |
| | K142,K143,K144,K145,K146,K148,K149,M2600,M2601,M2602,M2603 | 80234,80235,80236,80237,80238,8024, 8025,8028,8029,8300,8481 |
| | M2604,M2605,M2606,M2607,M2609,M2610,M2611,M2612,M2619,M2620 | 87341,87342,87343,87344,87349,87351, 87352,87353,87354,87359 |
| | M26211,M26212,M26213,M26219,M26220,M26221,M2623,M2624,M2625 | 87360,87361,87362,87363,87364,87365, 87370,87371,87372,87374 |
| | M2629,M2630,M2631,M2632,M2633,M2634,M2635,M2636,M2637,M2639 | 87375,8738,8739,9050,9056,9057,9060, 9089,95909,V523,V534 |
| | M264,M2650,M2651,M2652,M2653,M2654,M2655,M2656,M2657,M2659 M2660,M26601,M26602,M26603,M26609,M2661,M26611,M26612,M26613 M26619,M2662,M26621,M26622,M26623,M26629,M2663,M26631,M26632 M26633,M26639,M26641,M26642,M26643,M26649,M26651,M26652 M26653,M26659,M2669,M2670,M2671,M2672,M2673,M2674,M2679,M2681 M2682,M2689,M269,M270,M271,M272,M273,M2740,M2749,M2751,M2752 M2753,M2759,M2761,M2762,M2763,M2769,M278,M279,M350C,M7911 R682,R6884,S01401A,S01401D,S01401S,S01402A,S01402D,S01402S S01409A,S01409D,S01409S,S01411A,S01411D,S01411S,S01412A S01412D,S01412S,S01419A,S01419D,S01419S,S01421A,S01421D S01421S,S01422A,S01422D,S01422S,S01429A,S01429D,S01429S S01431A,S01431D,S01431S,S01432A,S01432D,S01432S,S01439A S01439D,S01439S,S01441A,S01441D,S01441S,S01442A,S01442D S01442S,S01449A,S01449D,S01449S,S01451A,S01451D,S01451S S01452A,S01452D,S01452S,S01459A,S01459D,S01459S,S01501A S01501D,S01501S,S01502A,S01502D,S01502S,S01511A,S01511D S01511S,S01512A,S01512D,S01512S,S01521A,S01521D,S01521S S01522A,S01522D,S01522S,S01531A,S01531D,S01531S,S01532A S01532D,S01532S,S01541A,S01541D,S01541S,S01542A,S01542D S01542S,S01551A,S01551D,S01551S,S01552A,S01552D,S01552S S0180XA,S0180XD,S0180XS,S0181XA,S0181XD,S0181XS,S0182XA S0182XD,S0182XS,S0190XA,S0190XD,S0190XS,S0191XA,S0191XD S0191XS,S0192XA,S0192XD,S0192XS,S02401A,S02401B,S02401D S02401G,S02401K,S02401S,S0240CA,S0240CB,S0240CD,S0240CG S0240CK,S0240CS,S0240DA,S0240DB,S0240DD,S0240DG,S0240DK S0240DS,S0242XA,S0242XB,S0242XD,S0242XG,S0242XK,S0242XS | V5419,V585,V5889,V722 |

Continued on next page

Table S9 (continued).

| Diagnosis | ICD-10 | ICD-9 |
|---|---|---|
| | S025XXA,S025XXB,S025XXD,S025XXG,S025XXK,S025XXS,S02600A S02600B,S02600D,S02600G,S02600K,S02600S,S02601A,S02601B S02601D,S02601G,S02601K,S02601S,S02602A,S02602B,S02602D S02602G,S02602K,S02602S,S02609A,S02609B,S02609D,S02609G S02609K,S02609S,S02610A,S02610B,S02610D,S02610G,S02610K S02610S,S02611A,S02611B,S02611D,S02611G,S02611K,S02611S S02612A,S02612B,S02612D,S02612G,S02612K,S02612S,S0261XA S0261XB,S0261XD,S0261XG,S0261XK,S0261XS,S02620A,S02620B S02620D,S02620G,S02620K,S02620S,S02621A,S02621B,S02621D S02621G,S02621K,S02621S,S02622A,S02622B,S02622D,S02622G S02622K,S02622S,S0262XA,S0262XB,S0262XD,S0262XG,S0262XK S0262XS,S02630A,S02630B,S02630D,S02630G,S02630K,S02630S S02631A,S02631B,S02631D,S02631G,S02631K,S02631S,S02632A S02632B,S02632D,S02632G,S02632K,S02632S,S0263XA,S0263XB S0263XD,S0263XG,S0263XK,S0263XS,S02640A,S02640B,S02640D S02640G,S02640K,S02640S,S02641A,S02641B,S02641D,S02641G S02641K,S02641S,S02642A,S02642B,S02642D,S02642G,S02642K S02642S,S0264XA,S0264XB,S0264XD,S0264XG,S0264XK,S0264XS S02650A,S02650B,S02650D,S02650G,S02650K,S02650S,S02651A S02651B,S02651D,S02651G,S02651K,S02651S,S02652A,S02652B S02652D,S02652G,S02652K,S02652S,S0265XA,S0265XB,S0265XD S0265XG,S0265XK,S0265XS,S0266XA,S0266XB,S0266XD,S0266XG S0266XK,S0266XS,S02670A,S02670B,S02670D,S02670G,S02670K S02670S,S02671A,S02671B,S02671D,S02671G,S02671K,S02671S S02672A,S02672B,S02672D,S02672G,S02672K,S02672S,S0267XA S0267XB,S0267XD,S0267XG,S0267XK,S0267XS,S0269XA,S0269XB S0269XD,S0269XG,S0269XK,S0269XS,S0300XA,S0300XD,S0300XS S0301XA,S0301XD,S0301XS,S0302XA,S0302XD,S0302XS,S0303XA S0303XD,S0303XS,S030XXA,S030XXD,S030XXS,S032XXA,S032XXD S032XXS,S0340XA,S0340XD,S0340XS,S0341XA,S0341XD,S0341XS S0342XA,S0342XD,S0342XS,S0343XA,S0343XD,S0343XS,S034XXA S034XXD,S034XXS,S0993XA,S0993XD,S0993XS,Z0120,Z0121,Z463,Z464 | |
| External cause codes: | Y9301,Y9302,Y9311,Y9312,Y9313,Y9314,Y9315,Y9316,Y9317,Y9318 | E0000,E0001,E0002,E0008,E0009,E0010, E0011,E0020,E0021,E0022 |
| activity codes | Y9319,Y9321,Y9322,Y9323,Y9324,Y9329,Y9331,Y9332,Y9333,Y9334 | E0023,E0024,E0025,E0026,E0027,E0028, E0029,E0030,E0031,E0032 |
| | Y9335,Y9339,Y9341,Y9342,Y9343,Y9344,Y9345,Y9349,Y9351,Y9352 | E0033,E0039,E0040,E0041,E0042,E0043, E0044,E0049,E0050,E0051 |
| | Y9353,Y9354,Y9355,Y9356,Y9357,Y9359,Y9361,Y9362,Y9363,Y9364 | E0052,E0053,E0054,E0059,E0060,E0061, E0062,E0063,E0064,E0065 |
| | Y9365,Y9366,Y9367,Y9368,Y9369,Y936A,Y9371,Y9372,Y9373,Y9374 | E0066,E0069,E0070,E0071,E0072,E0073, E0074,E0075,E0076,E0077 |
| | Y9375,Y9379,Y9381,Y9382,Y9383,Y9384,Y9385,Y9389,Y939,Y93A1 | E0078,E0079,E0080,E0081,E0082,E0083, E0084,E0089,E0090,E0091 |
| | Y93A2,Y93A3,Y93A4,Y93A5,Y93A6,Y93A9,Y93B1,Y93B2,Y93B3,Y93B4 | E0092,E0093,E0094,E0095,E0099,E0100, E0101,E0102,E0103,E0109 |
| | Y93B9,Y93C1,Y93C2,Y93C9,Y93D1,Y93D2,Y93D3,Y93D9,Y93E1,Y93E2 | E0110,E0111,E0119,E0120,E0121,E0122, E0129,E0130,E0131,E0132 |
| | Y93E3,Y93E4,Y93E5,Y93E6,Y93E8,Y93E9,Y93F1,Y93F2,Y93F9,Y93G1 | E0133,E0134,E0135,E0138,E0139,E0140, E0141,E0149,E0150,E0151 |
| | Y93G2,Y93G3,Y93G9,Y93H1,Y93H2,Y93H3,Y93H9,Y93I1,Y93I9,Y93J1 | E0152,E0159,E0160,E0161,E0162,E0169, E0170,E0179,E0180,E0181 |
| | Y93J2,Y93J3,Y93J4,Y93K1,Y93K2,Y93K3,Y93K9,Y990,Y991,Y992,Y998 | E0182,E0183,E0190,E0191,E0192,E0199, E0290,E0291,E0292,E0299 |
| | Y999 | E030 |
| External cause codes: | T63001A,T63002A,T63003A,T63004A,T63011A,T63012A,T63013A | 9895,E9050,E9051,E9052,E9053,E9054, E9055,E9056,E9060,E9061 |
| bites; initial encounter | T63014A,T63021A,T63022A,T63023A,T63024A,T63031A,T63032A T63033A,T63034A,T63041A,T63042A,T63043A,T63044A,T63061A T63062A,T63063A,T63064A,T63071A,T63072A,T63073A,T63074A T63081A,T63082A,T63083A,T63084A,T63091A,T63092A,T63093A T63094A,T63111A,T63112A,T63113A,T63114A,T63121A,T63122A T63123A,T63124A,T63191A,T63192A,T63193A,T63194A,T632X1A T632X2A,T632X3A,T632X4A,T63301A,T63302A,T63303A,T63304A T63311A,T63312A,T63313A,T63314A,T63321A,T63322A,T63323A T63324A,T63331A,T63332A,T63333A,T63334A,T63391A,T63392A T63393A,T63394A,T63411A,T63412A,T63413A,T63414A,T63421A T63422A,T63423A,T63424A,T63431A,T63432A,T63433A,T63434A T63441A,T63442A,T63443A,T63444A,T63451A,T63452A,T63453A T63454A,T63461A,T63462A,T63463A,T63464A,T63481A,T63482A T63483A,T63484A,T63511A,T63512A,T63513A,T63514A,T63591A T63592A,T63593A,T63594A,T63611A,T63612A,T63613A,T63614A T63621A,T63622A,T63623A,T63624A,T63631A,T63632A,T63633A T63634A,T63691A,T63692A,T63693A,T63694A,T63811A,T63812A T63813A,T63814A,T63821A,T63822A,T63823A,T63824A,T63831A T63832A,T63833A,T63834A,T63891A,T63892A,T63893A,T63894A T6391XA,T6392XA,T6393XA,T6394XA,W5301XA,W5311XA,W5321XA W5381XA,W540XXA,W5501XA,W5511XA,W5521XA,W5531XA,W5541XA W5551XA,W5581XA,W5601XA,W5611XA,W5621XA,W5631XA,W5641XA W5651XA,W5681XA,W57XXXA,W5801XA,W5811XA,W5901XA,W5911XA W5921XA,W5981XA,W6101XA,W6111XA,W6121XA,W6151XA,W6161XA W6191XA | E9062,E9063,E9064,E9509,E9621,E9809 |

Continued on next page

Table S9 (continued).

| Diagnosis | ICD-10 | ICD-9 |
|---|---|---|
| External cause codes: complications of medical and surgical care, initial encounter | Y620,Y621,Y622,Y623,Y624,Y625,Y626,Y628,Y629,Y630,Y631,Y632<br>Y633,Y634,Y635,Y636,Y638,Y639,Y640,Y641,Y648,Y649,Y650,Y651<br>Y652,Y653,Y654,Y6551,Y6552,Y6553,Y658,Y66,Y69,Y700,Y701,Y702<br>Y703,Y708,Y710,Y711,Y712,Y713,Y718,Y720,Y721,Y722,Y723,Y728<br>Y730,Y731,Y732,Y733,Y738,Y740,Y741,Y742,Y743,Y748,Y750,Y751<br>Y752,Y753,Y758,Y760,Y761,Y762,Y763,Y768,Y770,Y771,Y7711,Y7719<br>Y772,Y773,Y778,Y780,Y781,Y782,Y783,Y788,Y790,Y791,Y792,Y793<br>Y798,Y800,Y801,Y802,Y803,Y808,Y810,Y811,Y812,Y813,Y818,Y828<br>Y829,Y830,Y831,Y832,Y833,Y834,Y835,Y836,Y838,Y839,Y840,Y841<br>Y842,Y843,Y844,Y845,Y846,Y847,Y848,Y849,Y95 | E8720,E8721,E8722,E8723,E8724,E8725,<br>E8726,E8728,E8729,E8730<br>E8731,E8732,E8733,E8734,E8735,E8736,<br>E8738,E8739,E8750,E8751<br>E8752,E8758,E8759,E8760,E8761,E8762,<br>E8763,E8764,E8765,E8766<br>E8767,E8768,E8769,E8780,E8781,E8782,<br>E8783,E8784,E8785,E8786<br>E8788,E8789,E8790,E8791,E8792,E8793,<br>E8794,E8795,E8796,E8797<br>E8798,NoDx |
| External cause codes: cut pierce; initial encounter | W25XXXA,W260XXA,W261XXA,W262XXA,W268XXA,W269XXA,W270XXA<br>W271XXA,W272XXA,W273XXA,W274XXA,W275XXA,W278XXA,W28XXXA<br>W290XXA,W291XXA,W292XXA,W293XXA,W294XXA,W298XXA,W450XXA<br>W451XXA,W452XXA,W458XXA,W460XXA,W461XXA,X780XXA,X781XXA<br>X782XXA,X788XXA,X789XXA,X990XXA,X991XXA,X992XXA,X998XXA<br>X999XXA,Y280XXA,Y281XXA,Y282XXA,Y288XXA,Y289XXA,Y35401A<br>Y35402A,Y35403A,Y35409A,Y35411A,Y35412A,Y35413A,Y35419A<br>Y35491A,Y35492A,Y35493A,Y35499A | E9200,E9201,E9202,E9203,E9204,E9205,<br>E9208,E956,E966,E974,E986 |
| External cause codes: drowning submersion; initial encounter | T751XXA,V9000XA,V9001XA,V9002XA,V9003XA,V9004XA,V9005XA<br>V9006XA,V9008XA,V9009XA,V9010XA,V9011XA,V9012XA,V9013XA<br>V9014XA,V9015XA,V9016XA,V9018XA,V9019XA,V9020XA,V9021XA<br>V9022XA,V9023XA,V9024XA,V9025XA,V9026XA,V9027XA,V9028XA<br>V9029XA,V9030XA,V9031XA,V9032XA,V9033XA,V9034XA,V9035XA<br>V9036XA,V9037XA,V9038XA,V9039XA,V9080XA,V9081XA,V9082XA<br>V9083XA,V9084XA,V9085XA,V9086XA,V9087XA,V9088XA,V9089XA<br>V9200XA,V9201XA,V9202XA,V9203XA,V9204XA,V9205XA,V9206XA<br>V9207XA,V9208XA,V9209XA,V9210XA,V9211XA,V9212XA,V9213XA<br>V9214XA,V9215XA,V9216XA,V9219XA,V9220XA,V9221XA,V9222XA<br>V9223XA,V9224XA,V9225XA,V9226XA,V9227XA,V9228XA,V9229XA<br>W16011A,W16021A,W16031A,W16111A,W16121A,W16131A,W16211A<br>W16221A,W16311A,W16321A,W16331A,W1641XA,W16511A,W16521A<br>W16531A,W16611A,W16621A,W16711A,W16721A,W16811A,W16821A<br>W16831A,W1691XA,W22041A,W65XXXA,W67XXXA,W69XXXA,W73XXXA<br>W74XXXA,X710XXA,X711XXA,X712XXA,X713XXA,X718XXA,X719XXA<br>X920XXA,X921XXA,X922XXA,X923XXA,X928XXA,X929XXA,Y210XXA<br>Y211XXA,Y212XXA,Y213XXA,Y214XXA,Y218XXA,Y219XXA | 9941,E8309,E8329,E8830,E9102,E9104,<br>E9108,E9109,E9174,E954<br>E964,E984 |
| External cause codes: evidence of alcohol involvement | Y900,Y901,Y902,Y903,Y904,Y905,Y906,Y907,Y908,Y909 | NoDx |
| External cause codes: fall; initial encounter | V00111A,V00121A,V00131A,V00141A,V00151A,V00181A,V00211A<br>V00221A,V00281A,V00311A,V00321A,V00381A,V00811A,V00821A<br>V00831A,V00841A,V00891A,W000XXA,W001XXA,W002XXA,W009XXA<br>W010XXA,W0110XA,W01110A,W01111A,W01118A,W01119A,W01190A<br>W01198A,W03XXXA,W04XXXA,W050XXA,W051XXA,W052XXA,W06XXXA<br>W07XXXA,W08XXXA,W090XXA,W091XXA,W092XXA,W098XXA,W100XXA<br>W101XXA,W102XXA,W108XXA,W109XXA,W11XXXA,W12XXXA,W130XXA<br>W131XXA,W132XXA,W133XXA,W134XXA,W138XXA,W139XXA,W14XXXA<br>W15XXXA,W16012A,W16022A,W16032A,W16112A,W16122A,W16132A<br>W16212A,W16222A,W16312A,W16322A,W16332A,W1642XA,W16512A<br>W16522A,W16532A,W16612A,W16622A,W16712A,W16722A,W16812A<br>W16822A,W16832A,W1692XA,W170XXA,W171XXA,W172XXA,W173XXA<br>W174XXA,W1781XA,W1782XA,W1789XA,W1811XA,W1812XA,W182XXA<br>W1830XA,W1831XA,W1839XA,W19XXXA,X80XXXA,Y01XXXA,Y30XXXA | E8800,E8801,E8809,E8810,E8811,E882,<br>E8830,E8831,E8832,E8839<br>E8840,E8841,E8842,E8843,E8844,E8845,<br>E8846,E8849,E8850,E8851<br>E8852,E8853,E8854,E8859,E8860,E8880,<br>E8881,E8888,E8889,E9579<br>E9681,E9879 |
| External cause codes: fire burn; initial encounter | T541X1A,T541X2A,T541X3A,T541X4A,T542X1A,T542X2A,T542X3A<br>T542X4A,T543X1A,T543X2A,T543X3A,T543X4A,T5491XA,T5492XA<br>T5493XA,T5494XA,X000XXA,X001XXA,X002XXA,X003XXA,X004XXA<br>X005XXA,X008XXA,X010XXA,X011XXA,X013XXA,X014XXA,X018XXA<br>X020XXA,X021XXA,X022XXA,X023XXA,X024XXA,X025XXA,X028XXA<br>X030XXA,X031XXA,X033XXA,X034XXA,X038XXA,X04XXXA,X05XXXA<br>X060XXA,X061XXA,X062XXA,X063XXA,X0800XA,X0801XA,X0809XA<br>X0810XA,X0811XA,X0819XA,X0820XA,X0821XA,X0829XA,X088XXA<br>X100XXA,X101XXA,X102XXA,X110XXA,X111XXA,X118XXA,X12XXXA<br>X130XXA,X131XXA,X140XXA,X141XXA,X150XXA,X151XXA,X152XXA<br>X153XXA,X158XXA,X16XXXA,X17XXXA,X18XXXA,X19XXXA,X76XXXA<br>X770XXA,X771XXA,X772XXA,X773XXA,X778XXA,X779XXA,X97XXXA<br>X980XXA,X981XXA,X982XXA,X983XXA,X988XXA,X989XXA,Y26XXXA<br>Y270XXA,Y271XXA,Y272XXA,Y273XXA,Y278XXA,Y279XXA,Y36300A<br>Y36301A,Y36330A,Y36331A,Y36390A,Y36391A,Y37300A,Y37301A | 9831,9832,9839,E8902,E8903,E8908,<br>E892,E8930,E8938,E894,E896<br>E897,E8980,E8981,E9240,E9241,E9242,<br>E9248,E9507,E9581,E9582<br>E961,E9680,E9683,E9688,E9793,E9806,<br>E9881,E9882,E9901,E9903<br>E9909 |

Continued on next page

**Table S9 (continued).**

| Diagnosis | ICD-10 | ICD-9 |
|---|---|---|
| | Y37330A,Y37331A,Y37390A,Y37391A,Y383X1A,Y383X2A,Y383X3A | |
| External cause codes: firearm; initial encounter | W320XXA,W321XXA,W3300XA,W3301XA,W3302XA,W3303XA,W3309XA<br>W3310XA,W3311XA,W3312XA,W3313XA,W3319XA,W3400XA,W3409XA<br>W3410XA,W3419XA,X72XXXA,X730XXA,X731XXA,X732XXA,X738XXA<br>X739XXA,X748XXA,X749XXA,X93XXXA,X940XXA,X941XXA,X942XXA<br>X948XXA,X949XXA,X958XXA,X959XXA,Y22XXXA,Y230XXA,Y231XXA<br>Y232XXA,Y233XXA,Y238XXA,Y239XXA,Y248XXA,Y249XXA,Y35001A<br>Y35002A,Y35003A,Y35009A,Y35011A,Y35012A,Y35013A,Y35019A<br>Y35021A,Y35022A,Y35023A,Y35029A,Y35031A,Y35032A,Y35033A<br>Y35039A,Y35091A,Y35092A,Y35093A,Y35099A,Y36420A,Y36421A<br>Y36430A,Y36431A,Y3692XA,Y37420A,Y37421A,Y37430A,Y37431A<br>Y3792XA,Y384X1A,Y384X2A,Y384X3A | E9220,E9221,E9222,E9223,E9228,E9229,<br>E9550,E9551,E9552,E9553<br>E9554,E9559,E9650,E9651,E9652,E9653,<br>E9654,E970,E9794,E9850<br>E9851,E9852,E9853,E9854,E9857,E9909,<br>E9911,E9912 |
| External cause codes: machinery; initial encounter | W240XXA,W241XXA,W300XXA,W301XXA,W302XXA,W303XXA,W3081XA<br>W3089XA,W309XXA,W310XXA,W311XXA,W312XXA,W313XXA,W3181XA<br>W3182XA,W3183XA,W3189XA,W319XXA | E9190,E9191,E9192,E9193,E9194,E9195,<br>E9196,E9197,E9198,E9199 |
| External cause codes: motor vehicle traffic (MVT); initial encounter | V0210XA,V0211XA,V0212XA,V02131A,V02138A,V0219XA,V0290XA<br>V0291XA,V0292XA,V02931A,V02938A,V0299XA,V0310XA,V0311XA<br>V0312XA,V03131A,V03138A,V0319XA,V0390XA,V0391XA,V0392XA<br>V03931A,V03938A,V0399XA,V0410XA,V0411XA,V0412XA,V04131A<br>V04138A,V0419XA,V0490XA,V0491XA,V0492XA,V04931A,V04938A<br>V0499XA,V0920XA,V0921XA,V0929XA,V093XXA,V123XXA,V124XXA<br>V125XXA,V129XXA,V133XXA,V134XXA,V135XXA,V139XXA,V143XXA<br>V144XXA,V145XXA,V149XXA,V1940XA,V1949XA,V1950XA,V1959XA<br>V1960XA,V1969XA,V199XXA,V2039XA,V203XXA,V2049XA,V204XXA<br>V2059XA,V205XXA,V2099XA,V209XXA,V2139XA,V213XXA,V2149XA<br>V214XXA,V2159XA,V215XXA,V2199XA,V219XXA,V2231XA,V2239XA<br>V223XXA,V2241XA,V2249XA,V224XXA,V2251XA,V2259XA,V225XXA<br>V2291XA,V2299XA,V229XXA,V2331XA,V2339XA,V233XXA,V2341XA<br>V2349XA,V234XXA,V2351XA,V2359XA,V235XXA,V2391XA,V2399XA<br>V239XXA,V2431XA,V2439XA,V243XXA,V2441XA,V2449XA,V244XXA<br>V2451XA,V2459XA,V245XXA,V2491XA,V2499XA,V249XXA,V2539XA<br>V253XXA,V2549XA,V254XXA,V2559XA,V255XXA,V2599XA,V259XXA<br>V2639XA,V263XXA,V2649XA,V264XXA,V2659XA,V265XXA,V2699XA<br>V269XXA,V2739XA,V273XXA,V2749XA,V274XXA,V2759XA,V275XXA<br>V2799XA,V279XXA,V2839XA,V283XXA,V2849XA,V284XXA,V2859XA<br>V285XXA,V2899XA,V289XXA,V29401A,V29408A,V2940XA,V29491A<br>V29498A,V2949XA,V29501A,V29508A,V2950XA,V29591A,V29598A<br>V2959XA,V29601A,V29608A,V2960XA,V29691A,V29698A,V2969XA<br>V29818A,V2981XA,V29888A,V2988XA,V2991XA,V2999XA,V299XXA<br>V304XXA,V305XXA,V306XXA,V307XXA,V309XXA,V314XXA,V315XXA<br>V316XXA,V317XXA,V319XXA,V324XXA,V325XXA,V326XXA,V327XXA<br>V329XXA,V334XXA,V335XXA,V336XXA,V337XXA,V339XXA,V344XXA<br>V345XXA,V346XXA,V347XXA,V349XXA,V354XXA,V355XXA,V356XXA<br>V357XXA,V359XXA,V364XXA,V365XXA,V366XXA,V367XXA,V369XXA<br>V374XXA,V375XXA,V376XXA,V377XXA,V379XXA,V384XXA,V385XXA<br>V386XXA,V387XXA,V389XXA,V3940XA,V3949XA,V3950XA,V3959XA<br>V3960XA,V3969XA,V3981XA,V3989XA,V399XXA,V404XXA,V405XXA<br>V406XXA,V407XXA,V409XXA,V414XXA,V415XXA,V416XXA,V417XXA<br>V419XXA,V424XXA,V425XXA,V426XXA,V427XXA,V429XXA,V4341XA<br>V4342XA,V4343XA,V4344XA,V4351XA,V4352XA,V4353XA,V4354XA<br>V4361XA,V4362XA,V4363XA,V4364XA,V4371XA,V4372XA,V4373XA<br>V4374XA,V4391XA,V4392XA,V4393XA,V4394XA,V444XXA,V445XXA<br>V446XXA,V447XXA,V449XXA,V454XXA,V455XXA,V456XXA,V457XXA<br>V459XXA,V464XXA,V465XXA,V466XXA,V467XXA,V469XXA,V474XXA<br>V4751XA,V4752XA,V475XXA,V4761XA,V4762XA,V476XXA,V477XXA<br>V4791XA,V4792XA,V479XXA,V484XXA,V485XXA,V486XXA,V487XXA<br>V489XXA,V4940XA,V4949XA,V4950XA,V4959XA,V4960XA,V4969XA<br>V4981XA,V4988XA,V499XXA,V504XXA,V505XXA,V506XXA,V507XXA<br>V509XXA,V514XXA,V515XXA,V516XXA,V517XXA,V519XXA,V524XXA<br>V525XXA,V526XXA,V527XXA,V529XXA,V534XXA,V535XXA,V536XXA<br>V537XXA,V539XXA,V544XXA,V545XXA,V546XXA,V547XXA,V549XXA<br>V554XXA,V555XXA,V556XXA,V557XXA,V559XXA,V564XXA,V565XXA<br>V566XXA,V567XXA,V569XXA,V574XXA,V575XXA,V576XXA,V577XXA<br>V579XXA,V584XXA,V585XXA,V586XXA,V587XXA,V589XXA,V5940XA<br>V5949XA,V5950XA,V5959XA,V5960XA,V5969XA,V5981XA,V5988XA<br>V599XXA,V604XXA,V605XXA,V606XXA,V607XXA,V609XXA,V614XXA<br>V615XXA,V616XXA,V617XXA,V619XXA,V624XXA,V625XXA,V626XXA<br>V627XXA,V629XXA,V634XXA,V635XXA,V636XXA,V637XXA,V639XXA<br>V644XXA,V645XXA,V646XXA,V647XXA,V649XXA,V654XXA,V655XXA<br>V656XXA,V657XXA,V659XXA,V664XXA,V665XXA,V666XXA,V667XXA<br>V669XXA,V674XXA,V675XXA,V676XXA,V677XXA,V679XXA,V684XXA<br>V685XXA,V686XXA,V687XXA,V689XXA,V6940XA,V6949XA,V6950XA<br>V6959XA,V6960XA,V6969XA,V6981XA,V6988XA,V699XXA,V704XXA | E8100,E8101,E8102,E8103,E8108,E8109,<br>E8120,E8121,E8122,E8123<br>E8128,E8129,E8130,E8131,E8132,E8133,<br>E8134,E8135,E8136,E8138<br>E8139,E8140,E8141,E8142,E8143,E8147,<br>E8148,E8149,E8150,E8151<br>E8152,E8153,E8158,E8159,E8160,E8161,<br>E8162,E8163,E8168,E8169<br>E8178,E8188,E8189,E8190,E8192,E8199,<br>E8222,E8223,E8239,E8242<br>E8246,E8248,E8261,E8290,E846,E9580,<br>E9585,E9685,E9885 |

Continued on next page

Table S9 (continued).

| Diagnosis | ICD-10 | ICD-9 |
|---|---|---|
| | V705XXA,V706XXA,V707XXA,V709XXA,V714XXA,V715XXA,V716XXA<br>V717XXA,V719XXA,V724XXA,V725XXA,V726XXA,V727XXA,V729XXA<br>V734XXA,V735XXA,V736XXA,V737XXA,V739XXA,V744XXA,V745XXA<br>V746XXA,V747XXA,V749XXA,V754XXA,V755XXA,V756XXA,V757XXA<br>V759XXA,V764XXA,V765XXA,V766XXA,V767XXA,V769XXA,V774XXA<br>V775XXA,V776XXA,V777XXA,V779XXA,V784XXA,V785XXA,V786XXA<br>V787XXA,V789XXA,V7940XA,V7949XA,V7950XA,V7959XA,V7960XA<br>V7969XA,V7981XA,V7988XA,V799XXA,V8031XA,V8032XA,V8041XA<br>V8042XA,V8051XA,V8052XA,V811XXA,V821XXA,V830XXA,V831XXA<br>V832XXA,V833XXA,V840XXA,V841XXA,V842XXA,V843XXA,V850XXA<br>V851XXA,V852XXA,V853XXA,V8601XA,V8602XA,V8603XA,V8604XA<br>V8605XA,V8606XA,V8609XA,V8611XA,V8612XA,V8613XA,V8614XA<br>V8615XA,V8616XA,V8619XA,V8621XA,V8622XA,V8623XA,V8624XA<br>V8625XA,V8626XA,V8629XA,V8631XA,V8632XA,V8633XA,V8634XA<br>V8635XA,V8636XA,V8639XA,V870XXA,V871XXA,V872XXA,V873XXA<br>V874XXA,V875XXA,V876XXA,V877XXA,V878XXA,V892XXA,X810XXA<br>X820XXA,X821XXA,X822XXA,X828XXA,Y020XXA,Y030XXA,Y038XXA<br>Y32XXXA | |
| External cause codes:<br>natural environment;<br>initial encounter | T63711A,T63712A,T63713A,T63714A,T63791A,T63792A,T63793A<br>T63794A,T65821A,T65822A,T65823A,T65824A,T730XXA,T731XXA<br>T732XXA,T738XXA,T739XXA,T7500XA,T7501XA,T7509XA,T7520XA<br>T7521XA,T7522XA,T7523XA,T7529XA,T753XXA,W420XXA,W429XXA<br>W5309XA,W5319XA,W5329XA,W5389XA,W541XXA,W548XXA,W5503XA<br>W5509XA,W5512XA,W5519XA,W5522XA,W5529XA,W5532XA,W5539XA<br>W5542XA,W5549XA,W5552XA,W5559XA,W5582XA,W5589XA,W5602XA<br>W5609XA,W5612XA,W5619XA,W5622XA,W5629XA,W5632XA,W5639XA<br>W5642XA,W5649XA,W5652XA,W5659XA,W5682XA,W5689XA,W5802XA<br>W5803XA,W5809XA,W5812XA,W5813XA,W5819XA,W5902XA,W5909XA<br>W5912XA,W5913XA,W5919XA,W5922XA,W5929XA,W5982XA,W5983XA<br>W5989XA,W60XXXA,W6102XA,W6109XA,W6112XA,W6119XA,W6122XA<br>W6129XA,W6132XA,W6133XA,W6139XA,W6142XA,W6143XA,W6149XA<br>W6152XA,W6159XA,W6162XA,W6169XA,W6192XA,W6199XA,W620XXA<br>W621XXA,W629XXA,W64XXXA,W92XXXA,W9301XA,W9302XA,W9311XA<br>W9312XA,W932XXA,W938XXA,W940XXA,W9411XA,W9412XA,W9421XA<br>W9422XA,W9423XA,W9429XA,W9431XA,W9432XA,W9439XA,W99XXXA<br>X30XXXA,X31XXXA,X32XXXA,X34XXXA,X35XXXA,X360XXA,X361XXA<br>X370XXA,X371XXA,X372XXA,X373XXA,X3741XA,X3742XA,X3743XA<br>X378XXA,X379XXA,X38XXXA,X3901XA,X3908XA,X398XXA,X52XXXA<br>X832XXA | 9895,98989,9940,9942,9943,9944,9946,<br>9949,E9000,E9001,E9010<br>E9011,E9018,E9020,E9021,E9022,E9028,<br>E903,E9041,E9042,E9043<br>E9049,E9056,E9061,E9063,E9068,E9069,<br>E9080,E9081,E9082,E9083<br>E9084,E9088,E9089,E9090,E9091,E9092,<br>E9093,E9094,E9098,E9208<br>E9268,E9280,E9281,E9286,E9288,E9509,<br>E9583,E9621,E9809 |
| External cause codes:<br>other specified,<br>classifiable and NEC;<br>initial encounter | T1500XA,T1501XA,T1502XA,T1510XA,T1511XA,T1512XA,T1580XA<br>T1581XA,T1582XA,T1590XA,T1591XA,T1592XA,T161XXA,T162XXA<br>T169XXA,T170XXA,T171XXA,T17208A,T17218A,T17228A,T17298A<br>T17308A,T17318A,T17328A,T17398A,T17408A,T17418A,T17428A<br>T17498A,T17508A,T17518A,T17528A,T17598A,T17808A,T17818A<br>T17828A,T17898A,T17908A,T17918A,T17928A,T17998A,T180XXA<br>T18100A,T18108A,T18110A,T18118A,T18120A,T18128A,T18190A<br>T18198A,T182XXA,T183XXA,T184XXA,T185XXA,T188XXA,T189XXA<br>T190XXA,T191XXA,T192XXA,T193XXA,T194XXA,T198XXA,T199XXA<br>T7401XA,T7402XA,T7411XA,T7412XA,T7421XA,T7422XA,T7431XA<br>T7432XA,T744XXA,T7451XA,T7452XA,T7461XA,T7462XA,T7491XA<br>T7492XA,T754XXA,T7601XA,T7602XA,T7611XA,T7612XA,T7621XA<br>T7622XA,T7631XA,T7632XA,T7651XA,T7652XA,T7661XA,T7662XA<br>T7691XA,T7692XA,V00118A,V00128A,V00138A,V00148A,V00158A<br>V00188A,V00218A,V00228A,V00288A,V00318A,V00328A,V00388A<br>V00818A,V00828A,V00838A,V00848A,V00898A,W230XXA,W231XXA<br>W232XXA,W34010A,W34011A,W34018A,W34110A,W34111A,W34118A<br>W35XXXA,W361XXA,W362XXA,W363XXA,W368XXA,W369XXA,W370XXA<br>W378XXA,W38XXXA,W39XXXA,W400XXA,W401XXA,W408XXA,W409XXA<br>W4901XA,W4902XA,W4903XA,W4904XA,W4909XA,W499XXA,W85XXXA<br>W860XXA,W861XXA,W868XXA,W880XXA,W881XXA,W888XXA,W890XXA<br>W891XXA,W898XXA,W899XXA,W900XXA,W901XXA,W902XXA,W908XXA<br>X7401XA,X7402XA,X7409XA,X75XXXA,X818XXA,X831XXA,X838XXA<br>X9501XA,X9502XA,X9509XA,X960XXA,X961XXA,X962XXA,X963XXA<br>X964XXA,X968XXA,X969XXA,Y028XXA,Y0701,Y0702,Y0703,Y0704,Y0711<br>Y0712,Y0713,Y0714,Y07410,Y07411,Y07420,Y07421,Y07430,Y07432 | 9300,9301,9302,9308,9309,931,932,9330,<br>9331,9340,9341,9348<br>9349,9350,9351,9352,936,937,938,9390,<br>9391,9392,9393,9399,9948<br>99550,99551,99552,99553,99554,99555,<br>99580,99581,99582,99583<br>99584,E8843,E8845,E8849,E8850,E8851,<br>E8852,E8853,E8854,E8869<br>E9040,E911,E912,E914,E915,E918,<br>E9210,E9211,E9218,E9224,E9225<br>E9228,E9230,E9231,E9232,E9238,E9239,<br>E9250,E9251,E9252,E9258<br>E9260,E9261,E9262,E9263,E9264,E9265,<br>E9268,E9284,E9285,E9288<br>E9555,E9556,E9557,E9580,E9584,E9587,<br>E9588,E9654,E9655,E9656<br>E9657,E9658,E9659,E9670,E9672,E9673,<br>E9675,E9677,E9678,E9679<br>E9684,E9685,E9686,E9688,E970,E971,<br>E975,E9790,E9792,E9795<br>E9796,E9798,E9799,E9855,E9856,E9880,<br>E9888,E9900,E9902,E9910<br>E9913,E9914,E9915,E9916,E9917,E9918,<br>E9919,E9920,E9921,E9922<br>E9923,E9928,E9929,E9930,E9931,E9932,<br>E9933,E9934,E9935,E9936<br>E9937,E9938,E9939,E9951,E9952,E9958,<br>E9959,E9960,E9961,E9962<br>E9963,E9968,E9969,E9971,E9973,E9980,<br>E9981,E9988 |

Continued on next page

**Table S9 (continued).**

| Diagnosis | ICD-10 | ICD-9 |
|---|---|---|
| | Y07433,Y07434,Y07435,Y07436,Y07490,Y07491,Y07499,Y0750,Y07510 Y07511,Y07512,Y07513,Y07519,Y07521,Y07528,Y07529,Y0753,Y0759 Y076,Y079,Y0889XA,Y240XXA,Y25XXXA,Y31XXXA,Y33XXXA,Y35041A Y35042A,Y35043A,Y35049A,Y35101A,Y35102A,Y35103A,Y35109A Y35111A,Y35112A,Y35113A,Y35119A,Y35121A,Y35122A,Y35123A Y35129A,Y35191A,Y35192A,Y35193A,Y35199A,Y35831A,Y35832A Y35833A,Y35839A,Y35891A,Y35892A,Y35893A,Y36000A,Y36001A Y36010A,Y36011A,Y36020A,Y36021A,Y36030A,Y36031A,Y36040A Y36041A,Y36050A,Y36051A,Y36090A,Y36091A,Y36200A,Y36201A Y36210A,Y36211A,Y36220A,Y36221A,Y36230A,Y36231A,Y36240A Y36241A,Y36250A,Y36251A,Y36260A,Y36261A,Y36270A,Y36271A Y36290A,Y36291A,Y36310A,Y36311A,Y36320A,Y36321A,Y36410A Y36411A,Y36450A,Y36451A,Y36490A,Y36491A,Y36500A,Y36501A Y36510A,Y36511A,Y36520A,Y36521A,Y36530A,Y36531A,Y36540A Y36541A,Y36590A,Y36591A,Y366X0A,Y366X1A,Y36810A,Y36811A Y36820A,Y36821A,Y36880A,Y36881A,Y3691XA,Y37000A,Y37001A Y37010A,Y37011A,Y37020A,Y37021A,Y37030A,Y37031A,Y37040A Y37041A,Y37050A,Y37051A,Y37090A,Y37091A,Y37200A,Y37201A Y37210A,Y37211A,Y37220A,Y37221A,Y37230A,Y37231A,Y37240A Y37241A,Y37250A,Y37251A,Y37260A,Y37261A,Y37270A,Y37271A Y37290A,Y37291A,Y37310A,Y37311A,Y37320A,Y37321A,Y37410A Y37411A,Y37450A,Y37451A,Y37490A,Y37491A,Y37500A,Y37501A Y37510A,Y37511A,Y37520A,Y37521A,Y37530A,Y37531A,Y37540A Y37541A,Y37590A,Y37591A,Y376X0A,Y376X1A,Y3791XA,Y380X1A Y380X2A,Y380X3A,Y382X1A,Y382X2A,Y382X3A,Y385X1A,Y385X2A Y385X3A,Y386X1A,Y386X2A,Y386X3A,Y38811A,Y38812A,Y38891A Y38892A,Y38893A,Y389X1A,Y389X2A | |
| External cause codes: overexertion; initial encounter | T733XXA,W1840XA,W1841XA,W1842XA,W1843XA,W1849XA,X500XXA X501XXA,X503XXA,X509XXA | 9945,E8859,E9272 |
| External cause codes: pedal cyclist; not MVT; initial encounter | V100XXA,V101XXA,V102XXA,V103XXA,V104XXA,V105XXA,V109XXA V110XXA,V111XXA,V112XXA,V113XXA,V114XXA,V115XXA,V119XXA V120XXA,V121XXA,V122XXA,V130XXA,V131XXA,V132XXA,V140XXA V141XXA,V142XXA,V150XXA,V151XXA,V152XXA,V153XXA,V154XXA V155XXA,V159XXA,V160XXA,V161XXA,V162XXA,V163XXA,V164XXA V165XXA,V169XXA,V170XXA,V171XXA,V172XXA,V173XXA,V174XXA V175XXA,V179XXA,V180XXA,V181XXA,V182XXA,V183XXA,V184XXA V185XXA,V189XXA,V1900XA,V1909XA,V1910XA,V1919XA,V1920XA V1929XA,V193XXA,V1981XA,V1988XA,V2001XA,V2011XA,V2021XA V2031XA,V2041XA,V2051XA,V2091XA,V2101XA,V2111XA,V2121XA V2131XA,V2141XA,V2151XA,V2191XA,V2201XA,V2211XA,V2221XA V2301XA,V2311XA,V2321XA,V2401XA,V2411XA,V2421XA,V2501XA V2511XA,V2521XA,V2531XA,V2541XA,V2551XA,V2591XA,V2601XA V2611XA,V2621XA,V2631XA,V2641XA,V2651XA,V2691XA,V2701XA V2711XA,V2721XA,V2731XA,V2741XA,V2751XA,V2791XA,V2801XA V2811XA,V2821XA,V2831XA,V2841XA,V2851XA,V2891XA,V29001A V29091A,V29101A,V29191A,V29201A,V29291A,V2931XA,V29811A V29881A | E8013,E8226,E8261,E8269 |
| External cause codes: pedestrian; not MVT; initial encounter | V0100XA,V0101XA,V0102XA,V01031A,V01038A,V0109XA,V0110XA V0111XA,V0112XA,V01131A,V01138A,V0119XA,V0190XA,V0191XA V0192XA,V01931A,V01938A,V0199XA,V0200XA,V0201XA,V0202XA V02031A,V02038A,V0209XA,V0300XA,V0301XA,V0302XA,V03031A V03038A,V0309XA,V0400XA,V0401XA,V0402XA,V04031A,V04038A V0409XA,V0500XA,V0501XA,V0502XA,V05031A,V05038A,V0509XA V0510XA,V0511XA,V0512XA,V05131A,V05138A,V0519XA,V0590XA V0591XA,V0592XA,V05931A,V05938A,V0599XA,V0600XA,V0601XA V0602XA,V06031A,V06038A,V0609XA,V0610XA,V0611XA,V0612XA V06131A,V06138A,V0619XA,V0690XA,V0691XA,V0692XA,V06931A V06938A,V0699XA,V0900XA,V0901XA,V0909XA,V091XXA,V099XXA | E8052,E8072,E8107,E8227,E8260,E8290 |
| External cause codes: place of occurrence of the external cause | Y92000,Y92001,Y92002,Y92003,Y92007,Y92008,Y92009,Y92010 Y92011,Y92012,Y92013,Y92014,Y92015,Y92016,Y92017,Y92018 Y92019,Y92020,Y92021,Y92022,Y92023,Y92024,Y92025,Y92026 Y92027,Y92028,Y92029,Y92030,Y92031,Y92032,Y92038,Y92039 Y92040,Y92041,Y92042,Y92043,Y92044,Y92045,Y92046,Y92048 Y92049,Y92090,Y92091,Y92092,Y92093,Y92094,Y92095,Y92096 Y92098,Y92099,Y9210,Y92110,Y92111,Y92112,Y92113,Y92114,Y92115 Y92116,Y92118,Y92119,Y92120,Y92121,Y92122,Y92123,Y92124 Y92125,Y92126,Y92128,Y92129,Y92130,Y92131,Y92133,Y92135 Y92136,Y92137,Y92138,Y92139,Y92140,Y92141,Y92142,Y92143 Y92146,Y92147,Y92148,Y92149,Y92150,Y92151,Y92152,Y92153 Y92154,Y92155,Y92156,Y92157,Y92158,Y92159,Y92160,Y92161 Y92162,Y92163,Y92168,Y92169,Y92190,Y92191,Y92192,Y92193 Y92194,Y92195,Y92196,Y92197,Y92198,Y92199,Y92210,Y92211 Y92212,Y92213,Y92214,Y92215,Y92218,Y92219,Y9222,Y92230,Y92231 Y92232,Y92233,Y92234,Y92238,Y92239,Y92240,Y92241,Y92242 Y92243,Y92248,Y92250,Y92251,Y92252,Y92253,Y92254,Y92258,Y9226 Y9229,Y92310,Y92311,Y92312,Y92318,Y92320,Y92321,Y92322,Y92328 Y92330,Y92331,Y9234,Y9239,Y92410,Y92411,Y92412,Y92413,Y92414 Y92415,Y92480,Y92481,Y92482,Y92488,Y92510,Y92511,Y92512 Y92513,Y92520,Y92521,Y92522,Y92523,Y92524,Y92530,Y92531 | E8490,E8491,E8492,E8493,E8494,E8495, E8496,E8497,E8498,E8499 |

Continued on next page

Table S9 (continued).

| Diagnosis | ICD-10 | ICD-9 |
|---|---|---|
| | Y92532,Y92538,Y9259,Y9261,Y9262,Y9263,Y9264,Y9265,Y9269,Y9271 | |
| | Y9272,Y9273,Y9274,Y9279,Y92810,Y92811,Y92812,Y92813,Y92814 | |
| | Y92815,Y92816,Y92818,Y92820,Y92821,Y92828,Y92830,Y92831 | |
| | Y92832,Y92833,Y92834,Y92838,Y9284,Y9285,Y9286,Y9289,Y929 | |
| External cause codes: | T360X1A,T360X2A,T360X3A,T360X4A,T361X1A,T361X2A,T361X3A | 9600,9601,9602,9603,9604,9605,9606, 9608,9609,9610,9614,9615 |
| poisoning by drug | T361X4A,T362X1A,T362X2A,T362X3A,T362X4A,T363X1A,T363X2A | 9616,9617,9618,9619,9620,9621,9622, 9623,9624,9627,9628,9629 |
| | T363X3A,T363X4A,T364X1A,T364X2A,T364X3A,T364X4A,T365X1A | 9630,9631,9634,9635,9638,9639,9640, 9642,9643,9644,9645,9646 |
| | T365X2A,T365X3A,T365X4A,T366X1A,T366X2A,T366X3A,T366X4A | 9648,9649,96500,96501,96502,96509,9651, 9654,9655,96561,96569 |
| | T367X1A,T367X2A,T367X3A,T367X4A,T368X1A,T368X2A,T368X3A | 9657,9659,9660,9661,9662,9663,9664, 9670,9678,9679,9680,9682 |
| | T368X4A,T3691XA,T3692XA,T3693XA,T3694XA,T370X1A,T370X2A | 9683,9684,9685,9689,96900,96901,96902, 96903,96904,96905,96909 |
| | T370X3A,T370X4A,T371X1A,T371X2A,T371X3A,T371X4A,T372X1A | 9691,9692,9693,9694,9696,96970,96971, 96972,96973,96979,9698 |
| | T372X2A,T372X3A,T372X4A,T373X1A,T373X2A,T373X3A,T373X4A | 9699,9700,9701,97081,9710,9711,9712, 9713,9719,9720,9721,9722 |
| | T374X1A,T374X2A,T374X3A,T374X4A,T375X1A,T375X2A,T375X3A | 9723,9724,9725,9726,9727,9729,9730, 9731,9732,9733,9734,9735 |
| | T375X4A,T378X1A,T378X2A,T378X3A,T378X4A,T3791XA,T3792XA | 9736,9738,9739,9742,9744,9745,9747, 9750,9751,9752,9753,9754 |
| | T3793XA,T3794XA,T380X1A,T380X2A,T380X3A,T380X4A,T381X1A | 9755,9756,9757,9758,9760,9761,9762, 9763,9764,9765,9766,9767 |
| | T381X2A,T381X3A,T381X4A,T382X1A,T382X2A,T382X3A,T382X4A | 9768,9769,9770,9772,9778,9779,9786, 9788,9789,9790,9796,9799 |
| | T383X1A,T383X2A,T383X3A,T383X4A,T384X1A,T384X2A,T384X3A | E8500,E8501,E8502,E8503,E8504,E8505, E8506,E8507,E851,E8528 |
| | T384X4A,T385X1A,T385X2A,T385X3A,T385X4A,T386X1A,T386X2A | E8530,E8531,E8532,E8540,E8541,E8542, E8543,E8548,E8550,E8551 |
| | T386X3A,T386X4A,T387X1A,T387X2A,T387X3A,T387X4A,T38801A | E8552,E8553,E8554,E8555,E8556,E8558, E856,E857,E8580,E8581 |
| | T38802A,T38803A,T38804A,T38811A,T38812A,T38813A,T38814A | E8582,E8583,E8584,E8585,E8586,E8587, E8588,E9500,E9501,E9502 |
| | T38891A,T38892A,T38893A,T38894A,T38901A,T38902A,T38903A | E9503,E9504,E9528,E9620,E9800,E9801, E9802,E9803,E9804,E9805 |
| | T38904A,T38991A,T38992A,T38993A,T38994A,T39011A,T39012A | |
| | T39013A,T39014A,T39091A,T39092A,T39093A,T39094A,T391X1A | |
| | T391X2A,T391X3A,T391X4A,T392X1A,T392X2A,T392X3A,T392X4A | |
| | T39311A,T39312A,T39313A,T39314A,T39391A,T39392A,T39393A | |
| | T39394A,T394X1A,T394X2A,T394X3A,T394X4A,T398X1A,T398X2A | |
| | T398X3A,T398X4A,T3991XA,T3992XA,T3993XA,T3994XA,T400X1A | |
| | T400X2A,T400X3A,T400X4A,T401X1A,T401X2A,T401X3A,T401X4A | |
| | T402X1A,T402X2A,T402X3A,T402X4A,T403X1A,T403X2A,T403X3A | |
| | T403X4A,T40411A,T40412A,T40413A,T40414A,T40421A,T40422A | |
| | T40423A,T40424A,T40491A,T40492A,T40493A,T40494A,T404X1A | |
| | T404X2A,T404X3A,T404X4A,T405X1A,T405X2A,T405X3A,T405X4A | |
| | T40601A,T40602A,T40603A,T40604A,T40691A,T40692A,T40693A | |
| | T40694A,T40711A,T40712A,T40713A,T40714A,T40721A,T40722A | |
| | T40723A,T40724A,T407X1A,T407X2A,T407X3A,T407X4A,T408X1A | |
| | T408X2A,T408X3A,T408X4A,T40901A,T40902A,T40903A,T40904A | |
| | T40991A,T40992A,T40993A,T40994A,T410X1A,T410X2A,T410X3A | |
| | T410X4A,T411X1A,T411X2A,T411X3A,T411X4A,T41201A,T41202A | |
| | T41203A,T41204A,T41291A,T41292A,T41293A,T41294A,T413X1A | |
| | T413X2A,T413X3A,T413X4A,T4141XA,T4142XA,T4143XA,T4144XA | |
| | T415X1A,T415X2A,T415X3A,T415X4A,T420X1A,T420X2A,T420X3A | |
| | T420X4A,T421X1A,T421X2A,T421X3A,T421X4A,T422X1A,T422X2A | |
| | T422X3A,T422X4A,T423X1A,T423X2A,T423X3A,T423X4A,T424X1A | |
| | T424X2A,T424X3A,T424X4A,T425X1A,T425X2A,T425X3A,T425X4A | |
| | T426X1A,T426X2A,T426X3A,T426X4A,T4271XA,T4272XA,T4273XA | |
| | T4274XA,T428X1A,T428X2A,T428X3A,T428X4A,T43011A,T43012A | |
| | T43013A,T43014A,T43021A,T43022A,T43023A,T43024A,T431X1A | |
| | T431X2A,T431X3A,T431X4A,T43201A,T43202A,T43203A,T43204A | |
| | T43211A,T43212A,T43213A,T43214A,T43221A,T43222A,T43223A | |
| | T43224A,T43291A,T43292A,T43293A,T43294A,T433X1A,T433X2A | |
| | T433X3A,T433X4A,T434X1A,T434X2A,T434X3A,T434X4A,T43501A | |
| | T43502A,T43503A,T43504A,T43591A,T43592A,T43593A,T43594A | |
| | T43601A,T43602A,T43603A,T43604A,T43611A,T43612A,T43613A | |
| | T43614A,T43621A,T43622A,T43623A,T43624A,T43631A,T43632A | |
| | T43633A,T43634A,T43641A,T43642A,T43643A,T43644A,T43651A | |
| | T43652A,T43653A,T43654A,T43691A,T43692A,T43693A,T43694A | |
| | T438X1A,T438X2A,T438X3A,T438X4A,T4391XA,T4392XA,T4393XA | |
| | T4394XA,T440X1A,T440X2A,T440X3A,T440X4A,T441X1A,T441X2A | |
| | T441X3A,T441X4A,T442X1A,T442X2A,T442X3A,T442X4A,T443X1A | |
| | T443X2A,T443X3A,T443X4A,T444X1A,T444X2A,T444X3A,T444X4A | |
| | T445X1A,T445X2A,T445X3A,T445X4A,T446X1A,T446X2A,T446X3A | |
| | T446X4A,T447X1A,T447X2A,T447X3A,T447X4A,T448X1A,T448X2A | |
| | T448X3A,T448X4A,T44901A,T44902A,T44903A,T44904A,T44991A | |
| | T44992A,T44993A,T44994A,T450X1A,T450X2A,T450X3A,T450X4A | |
| | T451X1A,T451X2A,T451X3A,T451X4A,T452X1A,T452X2A,T452X3A | |
| | T452X4A,T453X1A,T453X2A,T453X3A,T453X4A,T454X1A,T454X2A | |
| | T454X3A,T454X4A,T45511A,T45512A,T45513A,T45514A,T45521A | |
| | T45522A,T45523A,T45524A,T45601A,T45602A,T45603A,T45604A | |

Continued on next page

Table S9 (continued).

| Diagnosis | ICD-10 | ICD-9 |
|---|---|---|
| | T45611A,T45612A,T45613A,T45614A,T45621A,T45622A,T45623A | |
| | T45624A,T45691A,T45692A,T45693A,T45694A,T457X1A,T457X2A | |
| | T457X3A,T457X4A,T458X1A,T458X2A,T458X3A,T458X4A,T4591XA | |
| | T4592XA,T4593XA,T4594XA,T460X1A,T460X2A,T460X3A,T460X4A | |
| | T461X1A,T461X2A,T461X3A,T461X4A,T462X1A,T462X2A,T462X3A | |
| | T462X4A,T463X1A,T463X2A,T463X3A,T463X4A,T464X1A,T464X2A | |
| | T464X3A,T464X4A,T465X1A,T465X2A,T465X3A,T465X4A,T466X1A | |
| | T466X2A,T466X3A,T466X4A,T467X1A,T467X2A,T467X3A,T467X4A | |
| | T468X1A,T468X2A,T468X3A,T468X4A,T46901A,T46902A,T46903A | |
| | T46904A,T46991A,T46992A,T46993A,T46994A,T470X1A,T470X2A | |
| | T470X3A,T470X4A,T471X1A,T471X2A,T471X3A,T471X4A,T472X1A | |
| | T472X2A,T472X3A,T472X4A,T473X1A,T473X2A,T473X3A,T473X4A | |
| | T474X1A,T474X2A,T474X3A,T474X4A,T475X1A,T475X2A,T475X3A | |
| | T475X4A,T476X1A,T476X2A,T476X3A,T476X4A,T477X1A,T477X2A | |
| | T477X3A,T477X4A,T478X1A,T478X2A,T478X3A,T478X4A,T4791XA | |
| | T4792XA,T4793XA,T4794XA,T480X1A,T480X2A,T480X3A,T480X4A | |
| | T481X1A,T481X2A,T481X3A,T481X4A,T48201A,T48202A,T48203A | |
| | T48204A,T48291A,T48292A,T48293A,T48294A,T483X1A,T483X2A | |
| | T483X3A,T483X4A,T484X1A,T484X2A,T484X3A,T484X4A,T485X1A | |
| | T485X2A,T485X3A,T485X4A,T486X1A,T486X2A,T486X3A,T486X4A | |
| | T48901A,T48902A,T48903A,T48904A,T48991A,T48992A,T48993A | |
| | T48994A,T490X1A,T490X2A,T490X3A,T490X4A,T491X1A,T491X2A | |
| | T491X3A,T491X4A,T492X1A,T492X2A,T492X3A,T492X4A,T493X1A | |
| | T493X2A,T493X3A,T493X4A,T494X1A,T494X2A,T494X3A,T494X4A | |
| | T495X1A,T495X2A,T495X3A,T495X4A,T496X1A,T496X2A,T496X3A | |
| | T496X4A,T497X1A,T497X2A,T497X3A,T497X4A,T498X1A,T498X2A | |
| | T498X3A,T498X4A,T4991XA,T4992XA,T4993XA,T4994XA,T500X1A | |
| | T500X2A,T500X3A,T500X4A,T501X1A,T501X2A,T501X3A,T501X4A | |
| | T502X1A,T502X2A,T502X3A,T502X4A,T503X1A,T503X2A,T503X3A | |
| | T503X4A,T504X1A,T504X2A,T504X3A,T504X4A,T505X1A,T505X2A | |
| | T505X3A,T505X4A,T506X1A,T506X2A,T506X3A,T506X4A,T507X1A | |
| | T507X2A,T507X3A,T507X4A,T508X1A,T508X2A,T508X3A,T508X4A | |
| | T50901A,T50902A,T50903A,T50904A,T50911A,T50912A,T50913A | |
| | T50914A,T50991A,T50992A,T50993A,T50994A,T50A11A,T50A12A | |
| | T50A13A,T50A14A,T50A21A,T50A22A,T50A23A,T50A24A,T50A91A | |
| | T50A92A,T50A93A,T50A94A,T50B11A,T50B12A,T50B13A,T50B14A | |
| | T50B91A,T50B92A,T50B93A,T50B94A,T50Z11A,T50Z12A,T50Z13A | |
| | T50Z14A,T50Z91A,T50Z92A,T50Z93A,T50Z94A | |
| External cause codes: | T510X1A,T510X2A,T510X3A,T510X4A,T511X1A,T511X2A,T511X3A | 9800,9801,9802,9803,9808,9809,981,9820, 9821,9822,9823,9824 |
| poisoning by non-drug | T511X4A,T512X1A,T512X2A,T512X3A,T512X4A,T513X1A,T513X2A | 9828,9830,9839,9849,9850,9851,9852, 9853,9855,9856,9858,9859 |
| | T513X3A,T513X4A,T518X1A,T518X2A,T518X3A,T518X4A,T5191XA | 986,9871,9872,9873,9874,9875,9876,9877, 9878,9879,9880,9881 |
| | T5192XA,T5193XA,T5194XA,T520X1A,T520X2A,T520X3A,T520X4A | 9882,9888,9889,9890,9891,9893,9894, 9896,9897,98982,98984 |
| | T521X1A,T521X2A,T521X3A,T521X4A,T522X1A,T522X2A,T522X3A | 98989,9899,E8601,E8602,E8603,E8604, E8608,E8609,E8610,E8611 |
| | T522X4A,T523X1A,T523X2A,T523X3A,T523X4A,T524X1A,T524X2A | E8616,E8621,E8624,E8631,E8634,E8635, E8637,E8640,E8641,E8643 |
| | T524X3A,T524X4A,T528X1A,T528X2A,T528X3A,T528X4A,T5291XA | E8644,E8651,E8652,E8653,E8654,E8655, E8658,E8659,E8660,E8661 |
| | T5292XA,T5293XA,T5294XA,T530X1A,T530X2A,T530X3A,T530X4A | E8663,E8664,E8668,E8681,E8682,E8683, E8688,E8689,E8690,E8691 |
| | T531X1A,T531X2A,T531X3A,T531X4A,T532X1A,T532X2A,T532X3A | E8692,E8693,E8698,E9506,E9507,E9508, E9509,E9520,E9521,E9528 |
| | T532X4A,T533X1A,T533X2A,T533X3A,T533X4A,T534X1A,T534X2A | E9529,E961,E9621,E9622,E9629,E972, E9797,E9806,E9807,E9808 |
| | T534X3A,T534X4A,T535X1A,T535X2A,T535X3A,T535X4A,T536X1A | E9809,E9820,E9821,E9828,E9970,E9972, E9978,E9979 |
| | T536X2A,T536X3A,T536X4A,T537X1A,T537X2A,T537X3A,T537X4A | |
| | T5391XA,T5392XA,T5393XA,T5394XA,T540X1A,T540X2A,T540X3A | |
| | T540X4A,T550X1A,T550X2A,T550X3A,T550X4A,T551X1A,T551X2A | |
| | T551X3A,T551X4A,T560X1A,T560X2A,T560X3A,T560X4A,T561X1A | |
| | T561X2A,T561X3A,T561X4A,T562X1A,T562X2A,T562X3A,T562X4A | |
| | T563X1A,T563X2A,T563X3A,T563X4A,T564X1A,T564X2A,T564X3A | |
| | T564X4A,T565X1A,T565X2A,T565X3A,T565X4A,T566X1A,T566X2A | |
| | T566X3A,T566X4A,T567X1A,T567X2A,T567X3A,T567X4A,T56811A | |
| | T56812A,T56813A,T56814A,T56891A,T56892A,T56893A,T56894A | |
| | T5691XA,T5692XA,T5693XA,T5694XA,T570X1A,T570X2A,T570X3A | |
| | T570X4A,T571X1A,T571X2A,T571X3A,T571X4A,T572X1A,T572X2A | |
| | T572X3A,T572X4A,T573X1A,T573X2A,T573X3A,T573X4A,T578X1A | |
| | T578X2A,T578X3A,T578X4A,T5791XA,T5792XA,T5793XA,T5794XA | |
| | T5801XA,T5802XA,T5803XA,T5804XA,T5811XA,T5812XA,T5813XA | |
| | T5814XA,T582X1A,T582X2A,T582X3A,T582X4A,T588X1A,T588X2A | |
| | T588X3A,T588X4A,T5891XA,T5892XA,T5893XA,T5894XA,T590X1A | |
| | T590X2A,T590X3A,T590X4A,T591X1A,T591X2A,T591X3A,T591X4A | |
| | T592X1A,T592X2A,T592X3A,T592X4A,T593X1A,T593X2A,T593X3A | |
| | T593X4A,T594X1A,T594X2A,T594X3A,T594X4A,T595X1A,T595X2A | |
| | T595X3A,T595X4A,T596X1A,T596X2A,T596X3A,T596X4A,T597X1A | |
| | T597X2A,T597X3A,T597X4A,T59811A,T59812A,T59813A,T59814A | |
| | T59891A,T59892A,T59893A,T59894A,T5991XA,T5992XA,T5993XA | |
| | T5994XA,T600X1A,T600X2A,T600X3A,T600X4A,T601X1A,T601X2A | |
| | T601X3A,T601X4A,T602X1A,T602X2A,T602X3A,T602X4A,T603X1A | |
| | T603X2A,T603X3A,T603X4A,T604X1A,T604X2A,T604X3A,T604X4A | |

Continued on next page

Table S9 (continued).

| Diagnosis | ICD-10 | ICD-9 |
|---|---|---|
| | T608X1A,T608X2A,T608X3A,T608X4A,T6091XA,T6092XA,T6093XA | |
| | T6094XA,T6101XA,T6102XA,T6103XA,T6104XA,T6111XA,T6112XA | |
| | T6113XA,T6114XA,T61771A,T61772A,T61773A,T61774A,T61781A | |
| | T61782A,T61783A,T61784A,T618X1A,T618X2A,T618X3A,T618X4A | |
| | T6191XA,T6192XA,T6193XA,T6194XA,T620X1A,T620X2A,T620X3A | |
| | T620X4A,T621X1A,T621X2A,T621X3A,T621X4A,T622X1A,T622X2A | |
| | T622X3A,T622X4A,T628X1A,T628X2A,T628X3A,T628X4A,T6291XA | |
| | T6292XA,T6293XA,T6294XA,T6401XA,T6402XA,T6403XA,T6404XA | |
| | T6481XA,T6482XA,T6483XA,T6484XA,T650X1A,T650X2A,T650X3A | |
| | T650X4A,T651X1A,T651X2A,T651X3A,T651X4A,T65211A,T65212A | |
| | T65213A,T65214A,T65221A,T65222A,T65223A,T65224A,T65291A | |
| | T65292A,T65293A,T65294A,T653X1A,T653X2A,T653X3A,T653X4A | |
| | T654X1A,T654X2A,T654X3A,T654X4A,T655X1A,T655X2A,T655X3A | |
| | T655X4A,T656X1A,T656X2A,T656X3A,T656X4A,T65811A,T65812A | |
| | T65813A,T65814A,T65831A,T65832A,T65833A,T65834A,T65891A | |
| | T65892A,T65893A,T65894A,T6591XA,T6592XA,T6593XA,T6594XA | |
| | Y35201A,Y35202A,Y35203A,Y35209A,Y35211A,Y35212A,Y35213A | |
| | Y35219A,Y35291A,Y35292A,Y35293A,Y35299A,Y367X0A,Y367X1A | |
| | Y377X0A,Y377X1A,Y387X1A,Y387X2A,Y387X3A | |
| External cause codes: | V0001XS,V0002XS,V00031S,V00038S,V0009XS,V00111S,V00112S | E9290,E9291,E9293,E9294,E9295,E9298,<br>E959,E969,E977,E9990 |
| sequela | V00118S,V00121S,V00122S,V00128S,V00131S,V00132S,V00138S | E9991 |
| | V00141S,V00142S,V00148S,V00151S,V00152S,V00158S,V00181S | |
| | V00182S,V00188S,V00211S,V00212S,V00218S,V00221S,V00222S | |
| | V00228S,V00281S,V00282S,V00288S,V00311S,V00312S,V00318S | |
| | V00321S,V00322S,V00328S,V00381S,V00382S,V00388S,V00811S | |
| | V00812S,V00818S,V00821S,V00822S,V00828S,V00831S,V00832S | |
| | V00838S,V00841S,V00842S,V00848S,V00891S,V00892S,V00898S | |
| | V0100XS,V0101XS,V0102XS,V01031S,V01038S,V0109XS,V0110XS | |
| | V0111XS,V0112XS,V01131S,V01138S,V0119XS,V0190XS,V0191XS | |
| | V0192XS,V01931S,V01938S,V0199XS,V0200XS,V0201XS,V0202XS | |
| | V02031S,V02038S,V0209XS,V0210XS,V0211XS,V0212XS,V02131S | |
| | V02138S,V0219XS,V0290XS,V0291XS,V0292XS,V02931S,V02938S | |
| | V0299XS,V0300XS,V0301XS,V0302XS,V03031S,V03038S,V0309XS | |
| | V0310XS,V0311XS,V0312XS,V03131S,V03138S,V0319XS,V0390XS | |
| | V0391XS,V0392XS,V03931S,V03938S,V0399XS,V0400XS,V0401XS | |
| | V0402XS,V04031S,V04038S,V0409XS,V0410XS,V0411XS,V0412XS | |
| | V04131S,V04138S,V0419XS,V0490XS,V0491XS,V0492XS,V04931S | |
| | V04938S,V0499XS,V0500XS,V0501XS,V0502XS,V05031S,V05038S | |
| | V0509XS,V0510XS,V0511XS,V0512XS,V05131S,V05138S,V0519XS | |
| | V0590XS,V0591XS,V0592XS,V05931S,V05938S,V0599XS,V0600XS | |
| | V0601XS,V0602XS,V06031S,V06038S,V0609XS,V0610XS,V0611XS | |
| | V0612XS,V06131S,V06138S,V0619XS,V0690XS,V0691XS,V0692XS | |
| | V06931S,V06938S,V0699XS,V0900XS,V0901XS,V0909XS,V091XXS | |
| | V0920XS,V0921XS,V0929XS,V093XXS,V099XXS,V100XXS,V101XXS | |
| | V102XXS,V103XXS,V104XXS,V105XXS,V109XXS,V110XXS,V111XXS | |
| | V112XXS,V113XXS,V114XXS,V115XXS,V119XXS,V120XXS,V121XXS | |
| | V122XXS,V123XXS,V124XXS,V125XXS,V129XXS,V130XXS,V131XXS | |
| | V132XXS,V133XXS,V134XXS,V135XXS,V139XXS,V140XXS,V141XXS | |
| | V142XXS,V143XXS,V144XXS,V145XXS,V149XXS,V150XXS,V151XXS | |
| | V152XXS,V153XXS,V154XXS,V155XXS,V159XXS,V160XXS,V161XXS | |
| | V162XXS,V163XXS,V164XXS,V165XXS,V169XXS,V170XXS,V171XXS | |
| | V172XXS,V173XXS,V174XXS,V175XXS,V179XXS,V180XXS,V181XXS | |
| | V182XXS,V183XXS,V184XXS,V185XXS,V189XXS,V1900XS,V1909XS | |
| | V1910XS,V1919XS,V1920XS,V1929XS,V193XXS,V1940XS,V1949XS | |
| | V1950XS,V1959XS,V1960XS,V1969XS,V1981XS,V1988XS,V199XXS | |
| | V2001XS,V2009XS,V200XXS,V2011XS,V2019XS,V201XXS,V2021XS | |
| | V2029XS,V202XXS,V2031XS,V2039XS,V203XXS,V2041XS,V2049XS | |
| | V204XXS,V2051XS,V2059XS,V205XXS,V2091XS,V2099XS,V209XXS | |
| | V2101XS,V2109XS,V210XXS,V2111XS,V2119XS,V211XXS,V2121XS | |
| | V2129XS,V212XXS,V2131XS,V2139XS,V213XXS,V2141XS,V2149XS | |
| | V214XXS,V2151XS,V2159XS,V215XXS,V2191XS,V2199XS,V219XXS | |
| | V2201XS,V2209XS,V220XXS,V2211XS,V2219XS,V221XXS,V2221XS | |
| | V2229XS,V222XXS,V2231XS,V2239XS,V223XXS,V2241XS,V2249XS | |
| | V224XXS,V2251XS,V2259XS,V225XXS,V2291XS,V2299XS,V229XXS | |
| | V2301XS,V2309XS,V230XXS,V2311XS,V2319XS,V231XXS,V2321XS | |
| | V2329XS,V232XXS,V2331XS,V2339XS,V233XXS,V2341XS,V2349XS | |
| | V234XXS,V2351XS,V2359XS,V235XXS,V2391XS,V2399XS,V239XXS | |
| | V2401XS,V2409XS,V240XXS,V2411XS,V2419XS,V241XXS,V2421XS | |
| | V2429XS,V242XXS,V2431XS,V2439XS,V243XXS,V2441XS,V2449XS | |
| | V244XXS,V2451XS,V2459XS,V245XXS,V2491XS,V2499XS,V249XXS | |
| | V2501XS,V2509XS,V250XXS,V2511XS,V2519XS,V251XXS,V2521XS | |
| | V2529XS,V252XXS,V2531XS,V2539XS,V253XXS,V2541XS,V2549XS | |
| | V254XXS,V2551XS,V2559XS,V255XXS,V2591XS,V2599XS,V259XXS | |
| | V2601XS,V2609XS,V260XXS,V2611XS,V2619XS,V261XXS,V2621XS | |
| | V2629XS,V262XXS,V2631XS,V2639XS,V263XXS,V2641XS,V2649XS | |
| | V264XXS,V2651XS,V2659XS,V265XXS,V2691XS,V2699XS,V269XXS | |
| | V2701XS,V2709XS,V270XXS,V2711XS,V2719XS,V271XXS,V2721XS | |
| | V2729XS,V272XXS,V2731XS,V2739XS,V273XXS,V2741XS,V2749XS | |
| | V274XXS,V2751XS,V2759XS,V275XXS,V2791XS,V2799XS,V279XXS | |
| | V2801XS,V2809XS,V280XXS,V2811XS,V2819XS,V281XXS,V2821XS | |
| | V2829XS,V282XXS,V2831XS,V2839XS,V283XXS,V2841XS,V2849XS | |
| | V284XXS,V2851XS,V2859XS,V285XXS,V2891XS,V2899XS,V289XXS | |
| | V29001S,V29008S,V2900XS,V29091S,V29098S,V2909XS,V29101S | |

Continued on next page

Table S9 (continued).

| Diagnosis | ICD-10 | ICD-9 |
|---|---|---|
| | V29108S,V2910XS,V29191S,V29198S,V2919XS,V29201S,V29208S V2920XS,V29291S,V29298S,V2929XS,V2931XS,V2939XS,V293XXS V29401S,V29408S,V2940XS,V29491S,V29498S,V2949XS,V29501S V29508S,V2950XS,V29591S,V29598S,V2959XS,V29601S,V29608S V2960XS,V29691S,V29698S,V2969XS,V29811S,V29818S,V2981XS V29881S,V29888S,V2988XS,V2991XS,V2999XS,V299XXS,V300XXS V301XXS,V302XXS,V303XXS,V304XXS,V305XXS,V306XXS,V307XXS V309XXS,V310XXS,V311XXS,V312XXS,V313XXS,V314XXS,V315XXS V316XXS,V317XXS,V319XXS,V320XXS,V321XXS,V322XXS,V323XXS V324XXS,V325XXS,V326XXS,V327XXS,V329XXS,V330XXS,V331XXS V332XXS,V333XXS,V334XXS,V335XXS,V336XXS,V337XXS,V339XXS V340XXS,V341XXS,V342XXS,V343XXS,V344XXS,V345XXS,V346XXS V347XXS,V349XXS,V350XXS,V351XXS,V352XXS,V353XXS,V354XXS V355XXS,V356XXS,V357XXS,V359XXS,V360XXS,V361XXS,V362XXS V363XXS,V364XXS,V365XXS,V366XXS,V367XXS,V369XXS,V370XXS V371XXS,V372XXS,V373XXS,V374XXS,V375XXS,V376XXS,V377XXS V379XXS,V380XXS,V381XXS,V382XXS,V383XXS,V384XXS,V385XXS V386XXS,V387XXS,V389XXS,V3900XS,V3909XS,V3910XS,V3919XS V3920XS,V3929XS,V393XXS,V3940XS,V3949XS,V3950XS,V3959XS V3960XS,V3969XS,V3981XS,V3989XS,V399XXS,V400XXS,V401XXS V402XXS,V403XXS,V404XXS,V405XXS,V406XXS,V407XXS,V409XXS V410XXS,V411XXS,V412XXS,V413XXS,V414XXS,V415XXS,V416XXS V417XXS,V419XXS,V420XXS,V421XXS,V422XXS,V423XXS,V424XXS V425XXS,V426XXS,V427XXS,V429XXS,V4301XS,V4302XS,V4303XS V4304XS,V4311XS,V4312XS,V4313XS,V4314XS,V4321XS,V4322XS V4323XS,V4324XS,V4331XS,V4332XS,V4333XS,V4334XS,V4341XS V4342XS,V4343XS,V4344XS,V4351XS,V4352XS,V4353XS,V4354XS V4361XS,V4362XS,V4363XS,V4364XS,V4371XS,V4372XS,V4373XS V4374XS,V4391XS,V4392XS,V4393XS,V4394XS,V440XXS,V441XXS V442XXS,V443XXS,V444XXS,V445XXS,V446XXS,V447XXS,V449XXS V450XXS,V451XXS,V452XXS,V453XXS,V454XXS,V455XXS,V456XXS V457XXS,V459XXS,V460XXS,V461XXS,V462XXS,V463XXS,V464XXS V465XXS,V466XXS,V467XXS,V469XXS,V4701XS,V4702XS,V470XXS V4711XS,V4712XS,V471XXS,V472XXS,V4731XS,V4732XS,V473XXS V474XXS,V4751XS,V4752XS,V475XXS,V4761XS,V4762XS,V476XXS V477XXS,V4791XS,V4792XS,V479XXS,V480XXS,V481XXS,V482XXS V483XXS,V484XXS,V485XXS,V486XXS,V487XXS,V489XXS,V4900XS V4909XS,V4910XS,V4919XS,V4920XS,V4929XS,V493XXS,V4940XS V4949XS,V4950XS,V4959XS,V4960XS,V4969XS,V4981XS,V4988XS V499XXS,V500XXS,V501XXS,V502XXS,V503XXS,V504XXS,V505XXS V506XXS,V507XXS,V509XXS,V510XXS,V511XXS,V512XXS,V513XXS V514XXS,V515XXS,V516XXS,V517XXS,V519XXS,V520XXS,V521XXS V522XXS,V523XXS,V524XXS,V525XXS,V526XXS,V527XXS,V529XXS V530XXS,V531XXS,V532XXS,V533XXS,V534XXS,V535XXS,V536XXS V537XXS,V539XXS,V540XXS,V541XXS,V542XXS,V543XXS,V544XXS V545XXS,V546XXS,V547XXS,V549XXS,V550XXS,V551XXS,V552XXS V553XXS,V554XXS,V555XXS,V556XXS,V557XXS,V559XXS,V560XXS V561XXS,V562XXS,V563XXS,V564XXS,V565XXS,V566XXS,V567XXS V569XXS,V570XXS,V571XXS,V572XXS,V573XXS,V574XXS,V575XXS V576XXS,V577XXS,V579XXS,V580XXS,V581XXS,V582XXS,V583XXS V584XXS,V585XXS,V586XXS,V587XXS,V589XXS,V5900XS,V5909XS V5910XS,V5919XS,V5920XS,V5929XS,V593XXS,V5940XS,V5949XS V5950XS,V5959XS,V5960XS,V5969XS,V5981XS,V5988XS,V599XXS V600XXS,V601XXS,V602XXS,V603XXS,V604XXS,V605XXS,V606XXS V607XXS,V609XXS,V610XXS,V611XXS,V612XXS,V613XXS,V614XXS V615XXS,V616XXS,V617XXS,V619XXS,V620XXS,V621XXS,V622XXS V623XXS,V624XXS,V625XXS,V626XXS,V627XXS,V629XXS,V630XXS V631XXS,V632XXS,V633XXS,V634XXS,V635XXS,V636XXS,V637XXS V639XXS,V640XXS,V641XXS,V642XXS,V643XXS,V644XXS,V645XXS V646XXS,V647XXS,V649XXS,V650XXS,V651XXS,V652XXS,V653XXS V654XXS,V655XXS,V656XXS,V657XXS,V659XXS,V660XXS,V661XXS V662XXS,V663XXS,V664XXS,V665XXS,V666XXS,V667XXS,V669XXS V670XXS,V671XXS,V672XXS,V673XXS,V674XXS,V675XXS,V676XXS V677XXS,V679XXS,V680XXS,V681XXS,V682XXS,V683XXS,V684XXS V685XXS,V686XXS,V687XXS,V689XXS,V6900XS,V6909XS,V6910XS V6919XS,V6920XS,V6929XS,V693XXS,V6940XS,V6949XS,V6950XS V6959XS,V6960XS,V6969XS,V6981XS,V6988XS,V699XXS,V700XXS V701XXS,V702XXS,V703XXS,V704XXS,V705XXS,V706XXS,V707XXS V709XXS,V710XXS,V711XXS,V712XXS,V713XXS,V714XXS,V715XXS V716XXS,V717XXS,V719XXS,V720XXS,V721XXS,V722XXS,V723XXS V724XXS,V725XXS,V726XXS,V727XXS,V729XXS,V730XXS,V731XXS V732XXS,V733XXS,V734XXS,V735XXS,V736XXS,V737XXS,V739XXS V740XXS,V741XXS,V742XXS,V743XXS,V744XXS,V745XXS,V746XXS V747XXS,V749XXS,V750XXS,V751XXS,V752XXS,V753XXS,V754XXS V755XXS,V756XXS,V757XXS,V759XXS,V760XXS,V761XXS,V762XXS V763XXS,V764XXS,V765XXS,V766XXS,V767XXS,V769XXS,V770XXS V771XXS,V772XXS,V773XXS,V774XXS,V775XXS,V776XXS,V777XXS V779XXS,V780XXS,V781XXS,V782XXS,V783XXS,V784XXS,V785XXS V786XXS,V787XXS,V789XXS,V7900XS,V7909XS,V7910XS,V7919XS V7920XS,V7929XS,V793XXS,V7940XS,V7949XS,V7950XS,V7959XS V7960XS,V7969XS,V7981XS,V7988XS,V799XXS,V80010S,V80018S V8002XS,V8011XS,V8012XS,V8021XS,V8022XS,V8031XS,V8032XS V8041XS,V8042XS,V8051XS,V8052XS,V8061XS,V8062XS,V80710S V80711S,V80720S,V80721S,V80730S,V80731S,V80790S,V80791S V8081XS,V8082XS,V80910S,V80918S,V80919S,V80920S,V80928S | |

Continued on next page

Table S9 (continued).

| Diagnosis | ICD-10 | ICD-9 |
|---|---|---|
| | V80929S,V810XXS,V811XXS,V812XXS,V813XXS,V814XXS,V815XXS<br>V816XXS,V817XXS,V8181XS,V8182XS,V8183XS,V8189XS,V819XXS<br>V820XXS,V821XXS,V822XXS,V823XXS,V824XXS,V825XXS,V826XXS<br>V827XXS,V828XXS,V829XXS,V830XXS,V831XXS,V832XXS,V833XXS<br>V834XXS,V835XXS,V836XXS,V837XXS,V839XXS,V840XXS,V841XXS<br>V842XXS,V843XXS,V844XXS,V845XXS,V846XXS,V847XXS,V849XXS<br>V850XXS,V851XXS,V852XXS,V853XXS,V854XXS,V855XXS,V856XXS<br>V857XXS,V859XXS,V8601XS,V8602XS,V8603XS,V8604XS,V8605XS<br>V8606XS,V8609XS,V8611XS,V8612XS,V8613XS,V8614XS,V8615XS<br>V8616XS,V8619XS,V8621XS,V8622XS,V8623XS,V8624XS,V8625XS<br>V8626XS,V8629XS,V8631XS,V8632XS,V8633XS,V8634XS,V8635XS<br>V8636XS,V8639XS,V8641XS,V8642XS,V8643XS,V8644XS,V8645XS<br>V8646XS,V8649XS,V8651XS,V8652XS,V8653XS,V8654XS,V8655XS<br>V8656XS,V8659XS,V8661XS,V8662XS,V8663XS,V8664XS,V8665XS<br>V8666XS,V8669XS,V8671XS,V8672XS,V8673XS,V8674XS,V8675XS<br>V8676XS,V8679XS,V8691XS,V8692XS,V8693XS,V8694XS,V8695XS<br>V8696XS,V8699XS,V870XXS,V871XXS,V872XXS,V873XXS,V874XXS<br>V875XXS,V876XXS,V877XXS,V878XXS,V879XXS,V880XXS,V881XXS<br>V882XXS,V883XXS,V884XXS,V885XXS,V886XXS,V887XXS,V888XXS<br>V889XXS,V890XXS,V891XXS,V892XXS,V893XXS,V899XXS,V9000XS<br>V9001XS,V9002XS,V9003XS,V9004XS,V9005XS,V9006XS,V9008XS<br>V9009XS,V9010XS,V9011XS,V9012XS,V9013XS,V9014XS,V9015XS<br>V9016XS,V9018XS,V9019XS,V9020XS,V9021XS,V9022XS,V9023XS<br>V9024XS,V9025XS,V9026XS,V9027XS,V9028XS,V9029XS,V9030XS<br>V9031XS,V9032XS,V9033XS,V9034XS,V9035XS,V9036XS,V9037XS<br>V9038XS,V9039XS,V9080XS,V9081XS,V9082XS,V9083XS,V9084XS<br>V9085XS,V9086XS,V9087XS,V9088XS,V9089XS,V9100XS,V9101XS<br>V9102XS,V9103XS,V9104XS,V9105XS,V9106XS,V9107XS,V9108XS<br>V9109XS,V9110XS,V9111XS,V9112XS,V9113XS,V9114XS,V9115XS<br>V9116XS,V9118XS,V9119XS,V9120XS,V9121XS,V9122XS,V9123XS<br>V9124XS,V9125XS,V9126XS,V9129XS,V9130XS,V9131XS,V9132XS<br>V9133XS,V9134XS,V9135XS,V9136XS,V9137XS,V9138XS,V9139XS<br>V9180XS,V9181XS,V9182XS,V9183XS,V9184XS,V9185XS,V9186XS<br>V9187XS,V9188XS,V9189XS,V9200XS,V9201XS,V9202XS,V9203XS<br>V9204XS,V9205XS,V9206XS,V9207XS,V9208XS,V9209XS,V9210XS<br>V9211XS,V9212XS,V9213XS,V9214XS,V9215XS,V9216XS,V9219XS<br>V9220XS,V9221XS,V9222XS,V9223XS,V9224XS,V9225XS,V9226XS<br>V9227XS,V9228XS,V9229XS,V9300XS,V9301XS,V9302XS,V9303XS<br>V9304XS,V9309XS,V9310XS,V9311XS,V9312XS,V9313XS,V9314XS<br>V9319XS,V9320XS,V9321XS,V9322XS,V9323XS,V9324XS,V9329XS<br>V9330XS,V9331XS,V9332XS,V9333XS,V9334XS,V9335XS,V9336XS<br>V9338XS,V9339XS,V9340XS,V9341XS,V9342XS,V9343XS,V9344XS<br>V9348XS,V9349XS,V9350XS,V9351XS,V9352XS,V9353XS,V9354XS<br>V9359XS,V9360XS,V9361XS,V9362XS,V9363XS,V9364XS,V9369XS<br>V9380XS,V9381XS,V9382XS,V9383XS,V9384XS,V9385XS,V9386XS<br>V9387XS,V9388XS,V9389XS,V940XXS,V9411XS,V9412XS,V9421XS<br>V9422XS,V9431XS,V9432XS,V944XXS,V94810S,V94811S,V94818S<br>V9489XS,V949XXS,V9500XS,V9501XS,V9502XS,V9503XS,V9504XS<br>V9505XS,V9509XS,V9510XS,V9511XS,V9512XS,V9513XS,V9514XS<br>V9515XS,V9519XS,V9520XS,V9521XS,V9522XS,V9523XS,V9524XS<br>V9525XS,V9529XS,V9530XS,V9531XS,V9532XS,V9533XS,V9534XS<br>V9535XS,V9539XS,V9540XS,V9541XS,V9542XS,V9543XS,V9544XS<br>V9545XS,V9549XS,V958XXS,V959XXS,V9600XS,V9601XS,V9602XS<br>V9603XS,V9604XS,V9605XS,V9609XS,V9610XS,V9611XS,V9612XS<br>V9613XS,V9614XS,V9615XS,V9619XS,V9620XS,V9621XS,V9622XS<br>V9623XS,V9624XS,V9625XS,V9629XS,V968XXS,V969XXS,V970XXS<br>V971XXS,V9721XS,V9722XS,V9729XS,V9731XS,V9732XS,V9733XS<br>V9739XS,V97810S,V97811S,V97818S,V9789XS,V980XXS,V981XXS<br>V982XXS,V983XXS,V988XXS,V99XXXS,W000XXS,W001XXS,W002XXS<br>W009XXS,W010XXS,W0110XS,W01110S,W01111S,W01118S,W01119S<br>W01190S,W01198S,W03XXXS,W04XXXS,W050XXS,W051XXS,W052XXS<br>W06XXXS,W07XXXS,W08XXXS,W090XXS,W091XXS,W092XXS,W098XXS<br>W100XXS,W101XXS,W102XXS,W108XXS,W109XXS,W11XXXS,W12XXXS<br>W130XXS,W131XXS,W132XXS,W133XXS,W134XXS,W138XXS,W139XXS<br>W14XXXS,W15XXXS,W16011S,W16012S,W16021S,W16022S,W16031S<br>W16032S,W16111S,W16112S,W16121S,W16122S,W16131S,W16132S<br>W16211S,W16212S,W16221S,W16222S,W16311S,W16312S,W16321S<br>W16322S,W16331S,W16332S,W1641XS,W1642XS,W16511S,W16512S<br>W16521S,W16522S,W16531S,W16532S,W16611S,W16612S,W16621S<br>W16622S,W16711S,W16712S,W16721S,W16722S,W16811S,W16812S<br>W16821S,W16822S,W16831S,W16832S,W1691XS,W1692XS,W170XXS<br>W171XXS,W172XXS,W173XXS,W174XXS,W1781XS,W1782XS,W1789XS<br>W1800XS,W1801XS,W1802XS,W1809XS,W1811XS,W1812XS,W182XXS<br>W1830XS,W1831XS,W1839XS,W1840XS,W1841XS,W1842XS,W1843XS<br>W1849XS,W19XXXS,W200XXS,W201XXS,W208XXS,W2100XS,W2101XS<br>W2102XS,W2103XS,W2104XS,W2105XS,W2106XS,W2107XS,W2109XS<br>W2111XS,W2112XS,W2113XS,W2119XS,W21210S,W21211S,W21220S<br>W21221S,W2131XS,W2132XS,W2139XS,W214XXS,W2181XS,W2189XS<br>W219XXS,W2201XS,W2202XS,W2203XS,W22041S,W22042S,W2209XS<br>W2210XS,W2211XS,W2212XS,W2219XS,W228XXS,W230XXS,W231XXS<br>W232XXS,W240XXS,W241XXS,W25XXXS,W260XXS,W261XXS,W262XXS<br>W268XXS,W269XXS,W270XXS,W271XXS,W272XXS,W273XXS,W274XXS<br>W275XXS,W278XXS,W28XXXS,W290XXS,W291XXS,W292XXS,W293XXS<br>W294XXS,W298XXS,W300XXS,W301XXS,W302XXS,W303XXS,W3081XS<br>W3089XS,W309XXS,W310XXS,W311XXS,W312XXS,W313XXS,W3181XS | |

Continued on next page

Table S9 (continued).

| Diagnosis | ICD-10 | ICD-9 |
|---|---|---|
| | W3182XS,W3183XS,W3189XS,W319XXS,W320XXS,W321XXS,W3300XS | |
| | W3301XS,W3302XS,W3303XS,W3309XS,W3310XS,W3311XS,W3312XS | |
| | W3313XS,W3319XS,W3400XS,W34010S,W34011S,W34018S,W3409XS | |
| | W3410XS,W34110S,W34111S,W34118S,W3419XS,W35XXXS,W361XXS | |
| | W362XXS,W363XXS,W368XXS,W369XXS,W370XXS,W378XXS,W38XXXS | |
| | W39XXXS,W400XXS,W401XXS,W408XXS,W409XXS,W420XXS,W429XXS | |
| | W450XXS,W451XXS,W452XXS,W458XXS,W460XXS,W461XXS,W4901XS | |
| | W4902XS,W4903XS,W4904XS,W4909XS,W499XXS,W500XXS,W501XXS | |
| | W502XXS,W503XXS,W504XXS,W51XXXS,W52XXXS,W5301XS,W5309XS | |
| | W5311XS,W5319XS,W5321XS,W5329XS,W5381XS,W5389XS,W540XXS | |
| | W541XXS,W548XXS,W5501XS,W5503XS,W5509XS,W5511XS,W5512XS | |
| | W5519XS,W5521XS,W5522XS,W5529XS,W5531XS,W5532XS,W5539XS | |
| | W5541XS,W5542XS,W5549XS,W5551XS,W5552XS,W5559XS,W5581XS | |
| | W5582XS,W5589XS,W5601XS,W5602XS,W5609XS,W5611XS,W5612XS | |
| | W5619XS,W5621XS,W5622XS,W5629XS,W5631XS,W5632XS,W5639XS | |
| | W5641XS,W5642XS,W5649XS,W5651XS,W5652XS,W5659XS,W5681XS | |
| | W5682XS,W5689XS,W57XXXS,W5801XS,W5802XS,W5803XS,W5809XS | |
| | W5811XS,W5812XS,W5813XS,W5819XS,W5901XS,W5902XS,W5909XS | |
| | W5911XS,W5912XS,W5913XS,W5919XS,W5921XS,W5922XS,W5929XS | |
| | W5981XS,W5982XS,W5983XS,W5989XS,W60XXXS,W6101XS,W6102XS | |
| | W6109XS,W6111XS,W6112XS,W6119XS,W6121XS,W6122XS,W6129XS | |
| | W6132XS,W6133XS,W6139XS,W6142XS,W6143XS,W6149XS,W6151XS | |
| | W6152XS,W6159XS,W6161XS,W6162XS,W6169XS,W6191XS,W6192XS | |
| | W6199XS,W620XXS,W621XXS,W629XXS,W64XXXS,W65XXXS,W67XXXS | |
| | W69XXXS,W73XXXS,W74XXXS,W85XXXS,W860XXS,W861XXS,W868XXS | |
| | W880XXS,W881XXS,W888XXS,W890XXS,W891XXS,W898XXS,W899XXS | |
| | W900XXS,W901XXS,W902XXS,W908XXS,W92XXXS,W9301XS,W9302XS | |
| | W9311XS,W9312XS,W932XXS,W938XXS,W940XXS,W9411XS,W9412XS | |
| | W9421XS,W9422XS,W9423XS,W9429XS,W9431XS,W9432XS,W9439XS | |
| | W99XXXS,X000XXS,X001XXS,X002XXS,X003XXS,X004XXS,X005XXS | |
| | X008XXS,X010XXS,X011XXS,X013XXS,X014XXS,X018XXS,X020XXS | |
| | X021XXS,X022XXS,X023XXS,X024XXS,X025XXS,X028XXS,X030XXS | |
| | X031XXS,X033XXS,X034XXS,X038XXS,X04XXXS,X05XXXS,X060XXS | |
| | X061XXS,X062XXS,X063XXS,X0800XS,X0801XS,X0809XS,X0810XS | |
| | X0811XS,X0819XS,X0820XS,X0821XS,X0829XS,X088XXS,X100XXS | |
| | X101XXS,X102XXS,X110XXS,X111XXS,X118XXS,X12XXXS,X130XXS | |
| | X131XXS,X140XXS,X141XXS,X150XXS,X151XXS,X152XXS,X153XXS | |
| | X158XXS,X16XXXS,X17XXXS,X18XXXS,X19XXXS,X30XXXS,X31XXXS | |
| | X32XXXS,X34XXXS,X35XXXS,X360XXS,X361XXS,X370XXS,X371XXS | |
| | X372XXS,X373XXS,X3741XS,X3742XS,X3743XS,X378XXS,X379XXS | |
| | X38XXXS,X3901XS,X3908XS,X398XXS,X500XXS,X501XXS,X503XXS | |
| | X509XXS,X52XXXS,X58XXXS,X710XXS,X711XXS,X712XXS,X713XXS | |
| | X718XXS,X719XXS,X72XXXS,X730XXS,X731XXS,X732XXS,X738XXS | |
| | X739XXS,X7401XS,X7402XS,X7409XS,X748XXS,X749XXS,X75XXXS | |
| | X76XXXS,X770XXS,X771XXS,X772XXS,X773XXS,X778XXS,X779XXS | |
| | X780XXS,X781XXS,X782XXS,X788XXS,X789XXS,X79XXXS,X80XXXS | |
| | X810XXS,X811XXS,X818XXS,X820XXS,X821XXS,X822XXS,X828XXS | |
| | X830XXS,X831XXS,X832XXS,X838XXS,X920XXS,X921XXS,X922XXS | |
| | X923XXS,X928XXS,X929XXS,X93XXXS,X940XXS,X941XXS,X942XXS | |
| | X948XXS,X949XXS,X9501XS,X9502XS,X9509XS,X958XXS,X959XXS | |
| | X960XXS,X961XXS,X962XXS,X963XXS,X964XXS,X968XXS,X969XXS | |
| | X97XXXS,X980XXS,X981XXS,X982XXS,X983XXS,X988XXS,X989XXS | |
| | X990XXS,X991XXS,X992XXS,X998XXS,X999XXS,Y00XXXS,Y01XXXS | |
| | Y020XXS,Y021XXS,Y028XXS,Y030XXS,Y038XXS,Y040XXS,Y041XXS | |
| | Y042XXS,Y048XXS,Y0801XS,Y0802XS,Y0809XS,Y0881XS,Y0889XS | |
| | Y210XXS,Y211XXS,Y212XXS,Y213XXS,Y214XXS,Y218XXS,Y219XXS | |
| | Y22XXXS,Y230XXS,Y231XXS,Y232XXS,Y233XXS,Y238XXS,Y239XXS | |
| | Y240XXS,Y248XXS,Y249XXS,Y25XXXS,Y26XXXS,Y270XXS,Y271XXS | |
| | Y272XXS,Y273XXS,Y278XXS,Y279XXS,Y280XXS,Y281XXS,Y282XXS | |
| | Y288XXS,Y289XXS,Y29XXXS,Y30XXXS,Y31XXXS,Y32XXXS,Y33XXXS | |
| | Y35001S,Y35002S,Y35003S,Y35009S,Y35011S,Y35012S,Y35013S | |
| | Y35019S,Y35021S,Y35022S,Y35023S,Y35029S,Y35031S,Y35032S | |
| | Y35033S,Y35039S,Y35041S,Y35042S,Y35043S,Y35049S,Y35091S | |
| | Y35092S,Y35093S,Y35099S,Y35101S,Y35102S,Y35103S,Y35109S | |
| | Y35111S,Y35112S,Y35113S,Y35119S,Y35121S,Y35122S,Y35123S | |
| | Y35129S,Y35191S,Y35192S,Y35193S,Y35199S,Y35201S,Y35202S | |
| | Y35203S,Y35209S,Y35211S,Y35212S,Y35213S,Y35219S,Y35291S | |
| | Y35292S,Y35293S,Y35299S,Y35301S,Y35302S,Y35303S,Y35309S | |
| | Y35311S,Y35312S,Y35313S,Y35319S,Y35391S,Y35392S,Y35393S | |
| | Y35399S,Y35401S,Y35402S,Y35403S,Y35409S,Y35411S,Y35412S | |
| | Y35413S,Y35419S,Y35491S,Y35492S,Y35493S,Y35499S,Y35811S | |
| | Y35812S,Y35813S,Y35819S,Y35831S,Y35832S,Y35833S,Y35839S | |
| | Y35891S,Y35892S,Y35893S,Y35899S,Y3591XS,Y3592XS,Y3593XS | |
| | Y3599XS,Y36000S,Y36001S,Y36010S,Y36011S,Y36020S,Y36021S | |
| | Y36030S,Y36031S,Y36040S,Y36041S,Y36050S,Y36051S,Y36090S | |
| | Y36091S,Y36100S,Y36101S,Y36110S,Y36111S,Y36120S,Y36121S | |
| | Y36130S,Y36131S,Y36140S,Y36141S,Y36190S,Y36191S,Y36200S | |
| | Y36201S,Y36210S,Y36211S,Y36220S,Y36221S,Y36230S,Y36231S | |
| | Y36240S,Y36241S,Y36250S,Y36251S,Y36260S,Y36261S,Y36270S | |
| | Y36271S,Y36290S,Y36291S,Y36300S,Y36301S,Y36310S,Y36311S | |
| | Y36320S,Y36321S,Y36330S,Y36331S,Y36390S,Y36391S,Y36410S | |
| | Y36411S,Y36420S,Y36421S,Y36430S,Y36431S,Y36440S,Y36441S | |
| | Y36450S,Y36451S,Y36460S,Y36461S,Y36470S,Y36471S,Y36490S | |
| | Y36491S,Y36500S,Y36501S,Y36510S,Y36511S,Y36520S,Y36521S | |
| | Y36530S,Y36531S,Y36540S,Y36541S,Y36590S,Y36591S,Y366X0S | |

Continued on next page

Table S9 (continued).

| Diagnosis | ICD-10 | ICD-9 |
|---|---|---|
| | Y366X1S,Y367X0S,Y367X1S,Y36810S,Y36811S,Y36820S,Y36821S | |
| | Y36880S,Y36881S,Y36890S,Y36891S,Y3690XS,Y3691XS,Y3692XS | |
| | Y37000S,Y37001S,Y37010S,Y37011S,Y37020S,Y37021S,Y37030S | |
| | Y37031S,Y37040S,Y37041S,Y37050S,Y37051S,Y37090S,Y37091S | |
| | Y37100S,Y37101S,Y37110S,Y37111S,Y37120S,Y37121S,Y37130S | |
| | Y37131S,Y37140S,Y37141S,Y37190S,Y37191S,Y37200S,Y37201S | |
| | Y37210S,Y37211S,Y37220S,Y37221S,Y37230S,Y37231S,Y37240S | |
| | Y37241S,Y37250S,Y37251S,Y37260S,Y37261S,Y37270S,Y37271S | |
| | Y37290S,Y37291S,Y37300S,Y37301S,Y37310S,Y37311S,Y37320S | |
| | Y37321S,Y37330S,Y37331S,Y37390S,Y37391S,Y37410S,Y37411S | |
| | Y37420S,Y37421S,Y37430S,Y37431S,Y37440S,Y37441S,Y37450S | |
| | Y37451S,Y37460S,Y37461S,Y37470S,Y37471S,Y37490S,Y37491S | |
| | Y37500S,Y37501S,Y37510S,Y37511S,Y37520S,Y37521S,Y37530S | |
| | Y37531S,Y37540S,Y37541S,Y37590S,Y37591S,Y376X0S,Y376X1S | |
| | Y377X0S,Y377X1S,Y3790XS,Y3791XS,Y3792XS,Y380X1S,Y380X2S | |
| | Y380X3S,Y381X1S,Y381X2S,Y381X3S,Y382X1S,Y382X2S,Y382X3S | |
| | Y383X1S,Y383X2S,Y383X3S,Y384X1S,Y384X2S,Y384X3S,Y385X1S | |
| | Y385X2S,Y385X3S,Y386X1S,Y386X2S,Y386X3S,Y387X1S,Y387X2S | |
| | Y387X3S,Y3880XS,Y38811S,Y38812S,Y38891S,Y38892S,Y38893S | |
| | Y389X1S,Y389X2S | |
| External cause codes: | V0001XA,V0002XA,V00031A,V00038A,V0009XA,V00112A,V00122A | E8843,E8845,E8849,E8850,E8851,E8852, E8853,E8854,E8859,E8880 |
| struck by; against; | V00132A,V00142A,V00152A,V00182A,V00212A,V00222A,V00282A | E8881,E916,E9170,E9171,E9173,E9174, E9178,E9179,E9283,E9588 |
| initial encounter | V00312A,V00322A,V00382A,V00812A,V00822A,V00832A,V00842A | E9600,E9682,E9687,E973,E975,E9888, E9950 |
| | V00892A,W1800XA,W1801XA,W1802XA,W1809XA,W200XXA,W201XXA | |
| | W208XXA,W2100XA,W2101XA,W2102XA,W2103XA,W2104XA,W2105XA | |
| | W2106XA,W2107XA,W2109XA,W2111XA,W2112XA,W2113XA,W2119XA | |
| | W21210A,W21211A,W21220A,W21221A,W2131XA,W2132XA,W2139XA | |
| | W214XXA,W2181XA,W2189XA,W219XXA,W2201XA,W2202XA,W2203XA | |
| | W22042A,W2209XA,W2210XA,W2211XA,W2212XA,W2219XA,W228XXA | |
| | W500XXA,W501XXA,W502XXA,W503XXA,W504XXA,W51XXXA,W52XXXA | |
| | X79XXXA,Y00XXXA,Y040XXA,Y041XXA,Y042XXA,Y048XXA,Y0801XA | |
| | Y0802XA,Y0809XA,Y29XXXA,Y35301A,Y35302A,Y35303A,Y35309A | |
| | Y35311A,Y35312A,Y35313A,Y35319A,Y35391A,Y35392A,Y35393A | |
| | Y35399A,Y35811A,Y35812A,Y35813A,Y35819A,Y36440A,Y36441A | |
| | Y37440A,Y37441A | |
| External cause codes: | V0001XD,V0002XD,V00031D,V00038D,V0009XD,V00111D,V00112D | E8001,E8011,E8013,E8018,E8019,E8021, E8031,E8041,E8052,E8061 |
| subsequent encounter | V00118D,V00121D,V00122D,V00128D,V00131D,V00132D,V00138D | E8071,E8072,E8100,E8101,E8102,E8103, E8107,E8108,E8109,E8120 |
| | V00141D,V00142D,V00148D,V00151D,V00152D,V00158D,V00181D | E8121,E8122,E8123,E8128,E8129,E8130, E8131,E8132,E8133,E8134 |
| | V00182D,V00188D,V00211D,V00212D,V00218D,V00221D,V00222D | E8135,E8136,E8138,E8139,E8140,E8141, E8142,E8143,E8147,E8148 |
| | V00228D,V00281D,V00282D,V00288D,V00311D,V00312D,V00318D | E8149,E8150,E8151,E8152,E8153,E8158, E8159,E8160,E8161,E8162 |
| | V00321D,V00322D,V00328D,V00381D,V00382D,V00388D,V00811D | E8163,E8168,E8169,E8178,E8188,E8189, E8190,E8192,E8199,E8200 |
| | V00812D,V00818D,V00821D,V00822D,V00828D,V00831D,V00832D | E8201,E8208,E8209,E8210,E8211,E8218, E8219,E8220,E8221,E8222 |
| | V00838D,V00841D,V00842D,V00848D,V00891D,V00892D,V00898D | E8223,E8224,E8226,E8227,E8228,E8229, E8230,E8231,E8232,E8233 |
| | V0100XD,V0101XD,V0102XD,V01031D,V01038D,V0109XD,V0110XD | E8238,E8239,E8242,E8246,E8248,E8250, E8251,E8252,E8253,E8258 |
| | V0111XD,V0112XD,V01131D,V01138D,V0119XD,V0190XD,V0191XD | E8259,E8260,E8261,E8262,E8263,E8269, E8272,E8273,E8282,E8290 |
| | V0192XD,V01931D,V01938D,V0199XD,V0200XD,V0201XD,V0202XD | E8294,E8299,E8309,E8319,E8329,E8359, E8369,E8379,E8380,E8381 |
| | V02031D,V02038D,V0209XD,V0210XD,V0211XD,V0212XD,V02131D | E8384,E8385,E8389,E8403,E8406,E8411, E8413,E8426,E8433,E8437 |
| | V02138D,V0219XD,V0290XD,V0291XD,V0292XD,V02931D,V02938D | E8439,E8441,E8443,E8447,E8449,E8450, E846,E847,E848,E8800 |
| | V0299XD,V0300XD,V0301XD,V0302XD,V03031D,V03038D,V0309XD | E8801,E8809,E8810,E8811,E882,E8830, E8831,E8832,E8839,E8840 |
| | V0310XD,V0311XD,V0312XD,V03131D,V03138D,V0319XD,V0390XD | E8841,E8842,E8843,E8844,E8845,E8846, E8849,E8850,E8851,E8852 |
| | V0391XD,V0392XD,V03931D,V03938D,V0399XD,V0400XD,V0401XD | E8853,E8854,E8859,E8860,E8869,E8880, E8881,E8888,E8889,E8902 |
| | V0402XD,V04031D,V04038D,V0409XD,V0410XD,V0411XD,V0412XD | E8903,E8908,E892,E8930,E8938,E894, E897,E8980,E8981,E9000 |
| | V04131D,V04138D,V0419XD,V0490XD,V0491XD,V0492XD,V04931D | E9001,E9010,E9011,E9018,E9020,E9021, E9022,E9028,E9060,E9061 |
| | V04938D,V0499XD,V0500XD,V0501XD,V0502XD,V05031D,V05038D | E9062,E9063,E9064,E9068,E9069,E9080, E9081,E9082,E9083,E9084 |
| | V0509XD,V0510XD,V0511XD,V0512XD,V05131D,V05138D,V0519XD | E9088,E9089,E9090,E9091,E9092,E9093, E9094,E9098,E9102,E9104 |
| | V0590XD,V0591XD,V0592XD,V05931D,V05938D,V0599XD,V0600XD | E9108,E9109,E916,E9170,E9171,E9173, E9174,E9179,E918,E9190 |
| | V0601XD,V0602XD,V06031D,V06038D,V0609XD,V0610XD,V0611XD | E9191,E9192,E9193,E9194,E9195,E9196, E9197,E9198,E9199,E9200 |
| | V0612XD,V06131D,V06138D,V0619XD,V0690XD,V0691XD,V0692XD | E9201,E9202,E9203,E9204,E9205,E9208, E9210,E9211,E9218,E9220 |
| | V06931D,V06938D,V0699XD,V0900XD,V0901XD,V0909XD,V091XXD | E9221,E9222,E9223,E9224,E9225,E9228, E9229,E9230,E9231,E9232 |

Continued on next page

Table S9 (continued).

| Diagnosis | ICD-10 | ICD-9 |
|---|---|---|
| | V0920XD,V0921XD,V0929XD,V093XXD,V099XXD,V100XXD,V101XXD | E9238,E9239,E9240,E9242,E9248,E9250, E9251,E9252,E9258,E9260 |
| | V102XXD,V103XXD,V104XXD,V105XXD,V109XXD,V110XXD,V111XXD | E9261,E9262,E9263,E9264,E9265,E9268, E9280,E9281,E9283,E9284 |
| | V112XXD,V113XXD,V114XXD,V115XXD,V119XXD,V120XXD,V121XXD | E9285,E9288,E954,E9550,E9551,E9552, E9553,E9554,E9555,E9556 |
| | V122XXD,V123XXD,V124XXD,V125XXD,V129XXD,V130XXD,V131XXD | E9557,E9559,E956,E9579,E9580,E9581, E9582,E9583,E9584,E9585 |
| | V132XXD,V133XXD,V134XXD,V135XXD,V139XXD,V140XXD,V141XXD | E9586,E9587,E9588,E9600,E964,E9650, E9651,E9652,E9653,E9654 |
| | V142XXD,V143XXD,V144XXD,V145XXD,V149XXD,V150XXD,V151XXD | E9655,E9656,E9657,E9658,E9659,E966, E9680,E9681,E9682,E9683 |
| | V152XXD,V153XXD,V154XXD,V155XXD,V159XXD,V160XXD,V161XXD | E9685,E9686,E9687,E9688,E970,E971, E972,E973,E974,E975,E976 |
| | V162XXD,V163XXD,V164XXD,V165XXD,V169XXD,V170XXD,V171XXD | E9790,E9791,E9792,E9793,E9794,E9795, E9796,E9797,E9798,E9799 |
| | V172XXD,V173XXD,V174XXD,V175XXD,V179XXD,V180XXD,V181XXD | E984,E9850,E9851,E9852,E9853,E9854, E9855,E9856,E9857,E986 |
| | V182XXD,V183XXD,V184XXD,V185XXD,V189XXD,V1900XD,V1909XD | E9879,E9880,E9881,E9882,E9885,E9888, E9900,E9901,E9902,E9903 |
| | V1910XD,V1919XD,V1920XD,V1929XD,V193XXD,V1940XD,V1949XD | E9909,E9910,E9911,E9912,E9913,E9914, E9915,E9916,E9917,E9918 |
| | V1950XD,V1959XD,V1960XD,V1969XD,V1981XD,V1988XD,V199XXD | E9919,E9920,E9921,E9922,E9923,E9928, E9929,E9930,E9931,E9932 |
| | V2001XD,V2009XD,V200XXD,V2011XD,V2019XD,V201XXD,V2021XD | E9933,E9934,E9935,E9936,E9937,E9938, E9939,E9940,E9941,E9942 |
| | V2029XD,V202XXD,V2031XD,V2039XD,V203XXD,V2041XD,V2049XD | E9943,E9948,E9949,E9950,E9951,E9952, E9953,E9954,E9958,E9959 |
| | V204XXD,V2051XD,V2059XD,V205XXD,V2091XD,V2099XD,V209XXD | E9960,E9961,E9962,E9963,E9968,E9969, E9970,E9971,E9972,E9973 |
| | V2101XD,V2109XD,V210XXD,V2111XD,V2119XD,V211XXD,V2121XD | E9978,E9979,E9980,E9981,E9988,E9989 |
| | V2129XD,V212XXD,V2131XD,V2139XD,V213XXD,V2141XD,V2149XD | |
| | V214XXD,V2151XD,V2159XD,V215XXD,V2191XD,V2199XD,V219XXD | |
| | V2201XD,V2209XD,V220XXD,V2211XD,V2219XD,V221XXD,V2221XD | |
| | V2229XD,V222XXD,V2231XD,V2239XD,V223XXD,V2241XD,V2249XD | |
| | V224XXD,V2251XD,V2259XD,V225XXD,V2291XD,V2299XD,V229XXD | |
| | V2301XD,V2309XD,V230XXD,V2311XD,V2319XD,V231XXD,V2321XD | |
| | V2329XD,V232XXD,V2331XD,V2339XD,V233XXD,V2341XD,V2349XD | |
| | V234XXD,V2351XD,V2359XD,V235XXD,V2391XD,V2399XD,V239XXD | |
| | V2401XD,V2409XD,V240XXD,V2411XD,V2419XD,V241XXD,V2421XD | |
| | V2429XD,V242XXD,V2431XD,V2439XD,V243XXD,V2441XD,V2449XD | |
| | V244XXD,V2451XD,V2459XD,V245XXD,V2491XD,V2499XD,V249XXD | |
| | V2501XD,V2509XD,V250XXD,V2511XD,V2519XD,V251XXD,V2521XD | |
| | V2529XD,V252XXD,V2531XD,V2539XD,V253XXD,V2541XD,V2549XD | |
| | V254XXD,V2551XD,V2559XD,V255XXD,V2591XD,V2599XD,V259XXD | |
| | V2601XD,V2609XD,V260XXD,V2611XD,V2619XD,V261XXD,V2621XD | |
| | V2629XD,V262XXD,V2631XD,V2639XD,V263XXD,V2641XD,V2649XD | |
| | V264XXD,V2651XD,V2659XD,V265XXD,V2691XD,V2699XD,V269XXD | |
| | V2701XD,V2709XD,V270XXD,V2711XD,V2719XD,V271XXD,V2721XD | |
| | V2729XD,V272XXD,V2731XD,V2739XD,V273XXD,V2741XD,V2749XD | |
| | V274XXD,V2751XD,V2759XD,V275XXD,V2791XD,V2799XD,V279XXD | |
| | V2801XD,V2809XD,V280XXD,V2811XD,V2819XD,V281XXD,V2821XD | |
| | V2829XD,V282XXD,V2831XD,V2839XD,V283XXD,V2841XD,V2849XD | |
| | V284XXD,V2851XD,V2859XD,V285XXD,V2891XD,V2899XD,V289XXD | |
| | V29001D,V29008D,V2900XD,V29091D,V29098D,V2909XD,V29101D | |
| | V29108D,V2910XD,V29191D,V29198D,V2919XD,V29201D,V29208D | |
| | V2920XD,V29291D,V29298D,V2929XD,V2931XD,V2939XD,V293XXD | |
| | V29401D,V29408D,V2940XD,V29491D,V29498D,V2949XD,V29501D | |
| | V29508D,V2950XD,V29591D,V29598D,V2959XD,V29601D,V29608D | |
| | V2960XD,V29691D,V29698D,V2969XD,V29811D,V29818D,V2981XD | |
| | V29881D,V29888D,V2988XD,V2991XD,V2999XD,V299XXD,V300XXD | |
| | V301XXD,V302XXD,V303XXD,V304XXD,V305XXD,V306XXD,V307XXD | |
| | V309XXD,V310XXD,V311XXD,V312XXD,V313XXD,V314XXD,V315XXD | |
| | V316XXD,V317XXD,V319XXD,V320XXD,V321XXD,V322XXD,V323XXD | |
| | V324XXD,V325XXD,V326XXD,V327XXD,V329XXD,V330XXD,V331XXD | |
| | V332XXD,V333XXD,V334XXD,V335XXD,V336XXD,V337XXD,V339XXD | |
| | V340XXD,V341XXD,V342XXD,V343XXD,V344XXD,V345XXD,V346XXD | |
| | V347XXD,V349XXD,V350XXD,V351XXD,V352XXD,V353XXD,V354XXD | |
| | V355XXD,V356XXD,V357XXD,V359XXD,V360XXD,V361XXD,V362XXD | |
| | V363XXD,V364XXD,V365XXD,V366XXD,V367XXD,V369XXD,V370XXD | |
| | V371XXD,V372XXD,V373XXD,V374XXD,V375XXD,V376XXD,V377XXD | |
| | V379XXD,V380XXD,V381XXD,V382XXD,V383XXD,V384XXD,V385XXD | |
| | V386XXD,V387XXD,V389XXD,V3900XD,V3909XD,V3910XD,V3919XD | |
| | V3920XD,V3929XD,V393XXD,V3940XD,V3949XD,V3950XD,V3959XD | |
| | V3960XD,V3969XD,V3981XD,V3989XD,V399XXD,V400XXD,V401XXD | |
| | V402XXD,V403XXD,V404XXD,V405XXD,V406XXD,V407XXD,V409XXD | |
| | V410XXD,V411XXD,V412XXD,V413XXD,V414XXD,V415XXD,V416XXD | |
| | V417XXD,V419XXD,V420XXD,V421XXD,V422XXD,V423XXD,V424XXD | |
| | V425XXD,V426XXD,V427XXD,V429XXD,V4301XD,V4302XD,V4303XD | |
| | V4304XD,V4311XD,V4312XD,V4313XD,V4314XD,V4321XD,V4322XD | |
| | V4323XD,V4324XD,V4331XD,V4332XD,V4333XD,V4334XD,V4341XD | |
| | V4342XD,V4343XD,V4344XD,V4351XD,V4352XD,V4353XD,V4354XD | |
| | V4361XD,V4362XD,V4363XD,V4364XD,V4371XD,V4372XD,V4373XD | |
| | V4374XD,V4391XD,V4392XD,V4393XD,V4394XD,V440XXD,V441XXD | |
| | V442XXD,V443XXD,V444XXD,V445XXD,V446XXD,V447XXD,V449XXD | |
| | V450XXD,V451XXD,V452XXD,V453XXD,V454XXD,V455XXD,V456XXD | |

Continued on next page

Table S9 (continued).

| Diagnosis | ICD-10 | ICD-9 |
|---|---|---|
| | V457XXD,V459XXD,V460XXD,V461XXD,V462XXD,V463XXD,V464XXD V465XXD,V466XXD,V467XXD,V469XXD,V4701XD,V4702XD,V470XXD V4711XD,V4712XD,V471XXD,V472XXD,V4731XD,V4732XD,V473XXD V474XXD,V4751XD,V4752XD,V475XXD,V4761XD,V4762XD,V476XXD V477XXD,V4791XD,V4792XD,V479XXD,V480XXD,V481XXD,V482XXD V483XXD,V484XXD,V485XXD,V486XXD,V487XXD,V489XXD,V4900XD V4909XD,V4910XD,V4919XD,V4920XD,V4929XD,V493XXD,V4940XD V4949XD,V4950XD,V4959XD,V4960XD,V4969XD,V4981XD,V4988XD V499XXD,V500XXD,V501XXD,V502XXD,V503XXD,V504XXD,V505XXD V506XXD,V507XXD,V509XXD,V510XXD,V511XXD,V512XXD,V513XXD V514XXD,V515XXD,V516XXD,V517XXD,V519XXD,V520XXD,V521XXD V522XXD,V523XXD,V524XXD,V525XXD,V526XXD,V527XXD,V529XXD V530XXD,V531XXD,V532XXD,V533XXD,V534XXD,V535XXD,V536XXD V537XXD,V539XXD,V540XXD,V541XXD,V542XXD,V543XXD,V544XXD V545XXD,V546XXD,V547XXD,V549XXD,V550XXD,V551XXD,V552XXD V553XXD,V554XXD,V555XXD,V556XXD,V557XXD,V559XXD,V560XXD V561XXD,V562XXD,V563XXD,V564XXD,V565XXD,V566XXD,V567XXD V569XXD,V570XXD,V571XXD,V572XXD,V573XXD,V574XXD,V575XXD V576XXD,V577XXD,V579XXD,V580XXD,V581XXD,V582XXD,V583XXD V584XXD,V585XXD,V586XXD,V587XXD,V589XXD,V5900XD,V5909XD V5910XD,V5919XD,V5920XD,V5929XD,V593XXD,V5940XD,V5949XD V5950XD,V5959XD,V5960XD,V5969XD,V5981XD,V5988XD,V599XXD V600XXD,V601XXD,V602XXD,V603XXD,V604XXD,V605XXD,V606XXD V607XXD,V609XXD,V610XXD,V611XXD,V612XXD,V613XXD,V614XXD V615XXD,V616XXD,V617XXD,V619XXD,V620XXD,V621XXD,V622XXD V623XXD,V624XXD,V625XXD,V626XXD,V627XXD,V629XXD,V630XXD V631XXD,V632XXD,V633XXD,V634XXD,V635XXD,V636XXD,V637XXD V639XXD,V640XXD,V641XXD,V642XXD,V643XXD,V644XXD,V645XXD V646XXD,V647XXD,V649XXD,V650XXD,V651XXD,V652XXD,V653XXD V654XXD,V655XXD,V656XXD,V657XXD,V659XXD,V660XXD,V661XXD V662XXD,V663XXD,V664XXD,V665XXD,V666XXD,V667XXD,V669XXD V670XXD,V671XXD,V672XXD,V673XXD,V674XXD,V675XXD,V676XXD V677XXD,V679XXD,V680XXD,V681XXD,V682XXD,V683XXD,V684XXD V685XXD,V686XXD,V687XXD,V689XXD,V6900XD,V6909XD,V6910XD V6919XD,V6920XD,V6929XD,V693XXD,V6940XD,V6949XD,V6950XD V6959XD,V6960XD,V6969XD,V6981XD,V6988XD,V699XXD,V700XXD V701XXD,V702XXD,V703XXD,V704XXD,V705XXD,V706XXD,V707XXD V709XXD,V710XXD,V711XXD,V712XXD,V713XXD,V714XXD,V715XXD V716XXD,V717XXD,V719XXD,V720XXD,V721XXD,V722XXD,V723XXD V724XXD,V725XXD,V726XXD,V727XXD,V729XXD,V730XXD,V731XXD V732XXD,V733XXD,V734XXD,V735XXD,V736XXD,V737XXD,V739XXD V740XXD,V741XXD,V742XXD,V743XXD,V744XXD,V745XXD,V746XXD V747XXD,V749XXD,V750XXD,V751XXD,V752XXD,V753XXD,V754XXD V755XXD,V756XXD,V757XXD,V759XXD,V760XXD,V761XXD,V762XXD V763XXD,V764XXD,V765XXD,V766XXD,V767XXD,V769XXD,V770XXD V771XXD,V772XXD,V773XXD,V774XXD,V775XXD,V776XXD,V777XXD V779XXD,V780XXD,V781XXD,V782XXD,V783XXD,V784XXD,V785XXD V786XXD,V787XXD,V789XXD,V7900XD,V7909XD,V7910XD,V7919XD V7920XD,V7929XD,V793XXD,V7940XD,V7949XD,V7950XD,V7959XD V7960XD,V7969XD,V7981XD,V7988XD,V799XXD,V80010D,V80018D V8002XD,V8011XD,V8012XD,V8021XD,V8022XD,V8031XD,V8032XD V8041XD,V8042XD,V8051XD,V8052XD,V8061XD,V8062XD,V80710D V80711D,V80720D,V80721D,V80730D,V80731D,V80790D,V80791D V8081XD,V8082XD,V80910D,V80918D,V80919D,V80920D,V80928D V80929D,V810XXD,V811XXD,V812XXD,V813XXD,V814XXD,V815XXD V816XXD,V817XXD,V8181XD,V8182XD,V8183XD,V8189XD,V819XXD V820XXD,V821XXD,V822XXD,V823XXD,V824XXD,V825XXD,V826XXD V827XXD,V828XXD,V829XXD,V830XXD,V831XXD,V832XXD,V833XXD V834XXD,V835XXD,V836XXD,V837XXD,V839XXD,V840XXD,V841XXD V842XXD,V843XXD,V844XXD,V845XXD,V846XXD,V847XXD,V849XXD V850XXD,V851XXD,V852XXD,V853XXD,V854XXD,V855XXD,V856XXD V857XXD,V859XXD,V8601XD,V8602XD,V8603XD,V8604XD,V8605XD V8606XD,V8609XD,V8611XD,V8612XD,V8613XD,V8614XD,V8615XD V8616XD,V8619XD,V8621XD,V8622XD,V8623XD,V8624XD,V8625XD V8626XD,V8629XD,V8631XD,V8632XD,V8633XD,V8634XD,V8635XD V8636XD,V8639XD,V8641XD,V8642XD,V8643XD,V8644XD,V8645XD V8646XD,V8649XD,V8651XD,V8652XD,V8653XD,V8654XD,V8655XD V8656XD,V8659XD,V8661XD,V8662XD,V8663XD,V8664XD,V8665XD V8666XD,V8669XD,V8671XD,V8672XD,V8673XD,V8674XD,V8675XD V8676XD,V8679XD,V8691XD,V8692XD,V8693XD,V8694XD,V8695XD V8696XD,V8699XD,V870XXD,V871XXD,V872XXD,V873XXD,V874XXD V875XXD,V876XXD,V877XXD,V878XXD,V879XXD,V880XXD,V881XXD V882XXD,V883XXD,V884XXD,V885XXD,V886XXD,V887XXD,V888XXD V889XXD,V890XXD,V891XXD,V892XXD,V893XXD,V899XXD,V9000XD V9001XD,V9002XD,V9003XD,V9004XD,V9005XD,V9006XD,V9008XD V9009XD,V9010XD,V9011XD,V9012XD,V9013XD,V9014XD,V9015XD V9016XD,V9018XD,V9019XD,V9020XD,V9021XD,V9022XD,V9023XD V9024XD,V9025XD,V9026XD,V9027XD,V9028XD,V9029XD,V9030XD V9031XD,V9032XD,V9033XD,V9034XD,V9035XD,V9036XD,V9037XD V9038XD,V9039XD,V9080XD,V9081XD,V9082XD,V9083XD,V9084XD V9085XD,V9086XD,V9087XD,V9088XD,V9089XD,V9100XD,V9101XD V9102XD,V9103XD,V9104XD,V9105XD,V9106XD,V9107XD,V9108XD V9109XD,V9110XD,V9111XD,V9112XD,V9113XD,V9114XD,V9115XD V9116XD,V9118XD,V9119XD,V9120XD,V9121XD,V9122XD,V9123XD V9124XD,V9125XD,V9126XD,V9129XD,V9130XD,V9131XD,V9132XD | |

Continued on next page

Table S9 (continued).

| Diagnosis | ICD-10 | ICD-9 |
|---|---|---|
| | V9133XD,V9134XD,V9135XD,V9136XD,V9137XD,V9138XD,V9139XD | |
| | V9180XD,V9181XD,V9182XD,V9183XD,V9184XD,V9185XD,V9186XD | |
| | V9187XD,V9188XD,V9189XD,V9200XD,V9201XD,V9202XD,V9203XD | |
| | V9204XD,V9205XD,V9206XD,V9207XD,V9208XD,V9209XD,V9210XD | |
| | V9211XD,V9212XD,V9213XD,V9214XD,V9215XD,V9216XD,V9219XD | |
| | V9220XD,V9221XD,V9222XD,V9223XD,V9224XD,V9225XD,V9226XD | |
| | V9227XD,V9228XD,V9229XD,V9300XD,V9301XD,V9302XD,V9303XD | |
| | V9304XD,V9309XD,V9310XD,V9311XD,V9312XD,V9313XD,V9314XD | |
| | V9319XD,V9320XD,V9321XD,V9322XD,V9323XD,V9324XD,V9329XD | |
| | V9330XD,V9331XD,V9332XD,V9333XD,V9334XD,V9335XD,V9336XD | |
| | V9338XD,V9339XD,V9340XD,V9341XD,V9342XD,V9343XD,V9344XD | |
| | V9348XD,V9349XD,V9350XD,V9351XD,V9352XD,V9353XD,V9354XD | |
| | V9359XD,V9360XD,V9361XD,V9362XD,V9363XD,V9364XD,V9369XD | |
| | V9380XD,V9381XD,V9382XD,V9383XD,V9384XD,V9385XD,V9386XD | |
| | V9387XD,V9388XD,V9389XD,V940XXD,V9411XD,V9412XD,V9421XD | |
| | V9422XD,V9431XD,V9432XD,V944XXD,V94810D,V94811D,V94818D | |
| | V9489XD,V949XXD,V9500XD,V9501XD,V9502XD,V9503XD,V9504XD | |
| | V9505XD,V9509XD,V9510XD,V9511XD,V9512XD,V9513XD,V9514XD | |
| | V9515XD,V9519XD,V9520XD,V9521XD,V9522XD,V9523XD,V9524XD | |
| | V9525XD,V9529XD,V9530XD,V9531XD,V9532XD,V9533XD,V9534XD | |
| | V9535XD,V9539XD,V9540XD,V9541XD,V9542XD,V9543XD,V9544XD | |
| | V9545XD,V9549XD,V958XXD,V959XXD,V9600XD,V9601XD,V9602XD | |
| | V9603XD,V9604XD,V9605XD,V9609XD,V9610XD,V9611XD,V9612XD | |
| | V9613XD,V9614XD,V9615XD,V9619XD,V9620XD,V9621XD,V9622XD | |
| | V9623XD,V9624XD,V9625XD,V9629XD,V968XXD,V969XXD,V970XXD | |
| | V971XXD,V9721XD,V9722XD,V9729XD,V9731XD,V9732XD,V9733XD | |
| | V9739XD,V97810D,V97811D,V97818D,V9789XD,V980XXD,V981XXD | |
| | V982XXD,V983XXD,V988XXD,V99XXXD,W000XXD,W001XXD,W002XXD | |
| | W009XXD,W010XXD,W0110XD,W01110D,W01111D,W01118D,W01119D | |
| | W01190D,W01198D,W03XXXD,W04XXXD,W050XXD,W051XXD,W052XXD | |
| | W06XXXD,W07XXXD,W08XXXD,W090XXD,W091XXD,W092XXD,W098XXD | |
| | W100XXD,W101XXD,W102XXD,W108XXD,W109XXD,W11XXXD,W12XXXD | |
| | W130XXD,W131XXD,W132XXD,W133XXD,W134XXD,W138XXD,W139XXD | |
| | W14XXXD,W15XXXD,W16011D,W16012D,W16021D,W16022D,W16031D | |
| | W16032D,W16111D,W16112D,W16121D,W16122D,W16131D,W16132D | |
| | W16211D,W16212D,W16221D,W16222D,W16311D,W16312D,W16321D | |
| | W16322D,W16331D,W16332D,W1641XD,W1642XD,W16511D,W16512D | |
| | W16521D,W16522D,W16531D,W16532D,W16611D,W16612D,W16621D | |
| | W16622D,W16711D,W16712D,W16721D,W16722D,W16811D,W16812D | |
| | W16821D,W16822D,W16831D,W16832D,W1691XD,W1692XD,W170XXD | |
| | W171XXD,W172XXD,W173XXD,W174XXD,W1781XD,W1782XD,W1789XD | |
| | W1800XD,W1801XD,W1802XD,W1809XD,W1811XD,W1812XD,W182XXD | |
| | W1830XD,W1831XD,W1839XD,W1840XD,W1841XD,W1842XD,W1843XD | |
| | W1849XD,W19XXXD,W200XXD,W201XXD,W208XXD,W2100XD,W2101XD | |
| | W2102XD,W2103XD,W2104XD,W2105XD,W2106XD,W2107XD,W2109XD | |
| | W2111XD,W2112XD,W2113XD,W2119XD,W21210D,W21211D,W21220D | |
| | W21221D,W2131XD,W2132XD,W2139XD,W214XXD,W2181XD,W2189XD | |
| | W219XXD,W2201XD,W2202XD,W2203XD,W22041D,W22042D,W2209XD | |
| | W2210XD,W2211XD,W2212XD,W2219XD,W228XXD,W230XXD,W231XXD | |
| | W232XXD,W240XXD,W241XXD,W25XXXD,W260XXD,W261XXD,W262XXD | |
| | W268XXD,W269XXD,W270XXD,W271XXD,W272XXD,W273XXD,W274XXD | |
| | W275XXD,W278XXD,W28XXXD,W290XXD,W291XXD,W292XXD,W293XXD | |
| | W294XXD,W298XXD,W300XXD,W301XXD,W302XXD,W303XXD,W3081XD | |
| | W3089XD,W309XXD,W310XXD,W311XXD,W312XXD,W313XXD,W3181XD | |
| | W3182XD,W3183XD,W3189XD,W319XXD,W320XXD,W321XXD,W3300XD | |
| | W3301XD,W3302XD,W3303XD,W3309XD,W3310XD,W3311XD,W3312XD | |
| | W3313XD,W3319XD,W3400XD,W34010D,W34011D,W34018D,W3409XD | |
| | W3410XD,W34110D,W34111D,W34118D,W3419XD,W35XXXD,W361XXD | |
| | W362XXD,W363XXD,W368XXD,W369XXD,W370XXD,W378XXD,W38XXXD | |
| | W39XXXD,W400XXD,W401XXD,W408XXD,W409XXD,W420XXD,W429XXD | |
| | W450XXD,W451XXD,W452XXD,W458XXD,W460XXD,W461XXD,W4901XD | |
| | W4902XD,W4903XD,W4904XD,W4909XD,W499XXD,W500XXD,W501XXD | |
| | W502XXD,W503XXD,W504XXD,W51XXXD,W52XXXD,W5301XD,W5309XD | |
| | W5311XD,W5319XD,W5321XD,W5329XD,W5381XD,W5389XD,W540XXD | |
| | W541XXD,W548XXD,W5501XD,W5503XD,W5509XD,W5511XD,W5512XD | |
| | W5519XD,W5521XD,W5522XD,W5529XD,W5531XD,W5532XD,W5539XD | |
| | W5541XD,W5542XD,W5549XD,W5551XD,W5552XD,W5559XD,W5581XD | |
| | W5582XD,W5589XD,W5601XD,W5602XD,W5609XD,W5611XD,W5612XD | |
| | W5619XD,W5621XD,W5622XD,W5629XD,W5631XD,W5632XD,W5639XD | |
| | W5641XD,W5642XD,W5649XD,W5651XD,W5652XD,W5659XD,W5681XD | |
| | W5682XD,W5689XD,W57XXXD,W5801XD,W5802XD,W5803XD,W5809XD | |
| | W5811XD,W5812XD,W5813XD,W5819XD,W5901XD,W5902XD,W5909XD | |
| | W5911XD,W5912XD,W5913XD,W5919XD,W5921XD,W5922XD,W5929XD | |
| | W5981XD,W5982XD,W5983XD,W5989XD,W60XXXD,W6101XD,W6102XD | |
| | W6109XD,W6111XD,W6112XD,W6119XD,W6121XD,W6122XD,W6129XD | |
| | W6132XD,W6133XD,W6139XD,W6142XD,W6143XD,W6149XD,W6151XD | |
| | W6152XD,W6159XD,W6161XD,W6162XD,W6169XD,W6191XD,W6192XD | |
| | W6199XD,W620XXD,W621XXD,W629XXD,W64XXXD,W65XXXD,W67XXXD | |
| | W69XXXD,W73XXXD,W74XXXD,W85XXXD,W860XXD,W861XXD,W868XXD | |
| | W880XXD,W881XXD,W888XXD,W890XXD,W891XXD,W898XXD,W899XXD | |
| | W900XXD,W901XXD,W902XXD,W908XXD,W92XXXD,W9301XD,W9302XD | |
| | W9311XD,W9312XD,W932XXD,W938XXD,W940XXD,W9411XD,W9412XD | |
| | W9421XD,W9422XD,W9423XD,W9429XD,W9431XD,W9432XD,W9439XD | |
| | W99XXXD,X000XXD,X001XXD,X002XXD,X003XXD,X004XXD,X005XXD | |
| | X008XXD,X010XXD,X011XXD,X013XXD,X014XXD,X018XXD,X020XXD | |

Continued on next page

Table S9 (continued).

| Diagnosis | ICD-10 | ICD-9 |
|---|---|---|
| | X021XXD,X022XXD,X023XXD,X024XXD,X025XXD,X028XXD,X030XXD<br>X031XXD,X033XXD,X034XXD,X038XXD,X04XXXD,X05XXXD,X060XXD<br>X061XXD,X062XXD,X063XXD,X0800XD,X0801XD,X0809XD,X0810XD<br>X0811XD,X0819XD,X0820XD,X0821XD,X0829XD,X088XXD,X100XXD<br>X101XXD,X102XXD,X110XXD,X111XXD,X118XXD,X12XXXD,X130XXD<br>X131XXD,X140XXD,X141XXD,X150XXD,X151XXD,X152XXD,X153XXD<br>X158XXD,X16XXXD,X17XXXD,X18XXXD,X19XXXD,X30XXXD,X31XXXD<br>X32XXXD,X34XXXD,X35XXXD,X360XXD,X361XXD,X370XXD,X371XXD<br>X372XXD,X373XXD,X3741XD,X3742XD,X3743XD,X378XXD,X379XXD<br>X38XXXD,X3901XD,X3908XD,X398XXD,X500XXD,X501XXD,X503XXD<br>X509XXD,X52XXXD,X58XXXD,X710XXD,X711XXD,X712XXD,X713XXD<br>X718XXD,X719XXD,X72XXXD,X730XXD,X731XXD,X732XXD,X738XXD<br>X739XXD,X7401XD,X7402XD,X7409XD,X748XXD,X749XXD,X75XXXD<br>X76XXXD,X770XXD,X771XXD,X772XXD,X773XXD,X778XXD,X779XXD<br>X780XXD,X781XXD,X782XXD,X788XXD,X789XXD,X79XXXD,X80XXXD<br>X810XXD,X811XXD,X818XXD,X820XXD,X821XXD,X822XXD,X828XXD<br>X830XXD,X831XXD,X832XXD,X838XXD,X920XXD,X921XXD,X922XXD<br>X923XXD,X928XXD,X929XXD,X93XXXD,X940XXD,X941XXD,X942XXD<br>X948XXD,X949XXD,X9501XD,X9502XD,X9509XD,X958XXD,X959XXD<br>X960XXD,X961XXD,X962XXD,X963XXD,X964XXD,X968XXD,X969XXD<br>X97XXXD,X980XXD,X981XXD,X982XXD,X983XXD,X988XXD,X989XXD<br>X990XXD,X991XXD,X992XXD,X998XXD,X999XXD,Y00XXXD,Y01XXXD<br>Y020XXD,Y021XXD,Y028XXD,Y030XXD,Y038XXD,Y040XXD,Y041XXD<br>Y042XXD,Y048XXD,Y0801XD,Y0802XD,Y0809XD,Y0881XD,Y0889XD<br>Y210XXD,Y211XXD,Y212XXD,Y213XXD,Y214XXD,Y218XXD,Y219XXD<br>Y22XXXD,Y230XXD,Y231XXD,Y232XXD,Y233XXD,Y238XXD,Y239XXD<br>Y240XXD,Y248XXD,Y249XXD,Y25XXXD,Y26XXXD,Y270XXD,Y271XXD<br>Y272XXD,Y273XXD,Y278XXD,Y279XXD,Y280XXD,Y281XXD,Y282XXD<br>Y288XXD,Y289XXD,Y29XXXD,Y30XXXD,Y31XXXD,Y32XXXD,Y33XXXD<br>Y35001D,Y35002D,Y35003D,Y35009D,Y35011D,Y35012D,Y35013D<br>Y35019D,Y35021D,Y35022D,Y35023D,Y35029D,Y35031D,Y35032D<br>Y35033D,Y35039D,Y35041D,Y35042D,Y35043D,Y35049D,Y35091D<br>Y35092D,Y35093D,Y35099D,Y35101D,Y35102D,Y35103D,Y35109D<br>Y35111D,Y35112D,Y35113D,Y35119D,Y35121D,Y35122D,Y35123D<br>Y35129D,Y35191D,Y35192D,Y35193D,Y35199D,Y35201D,Y35202D<br>Y35203D,Y35209D,Y35211D,Y35212D,Y35213D,Y35219D,Y35291D<br>Y35292D,Y35293D,Y35299D,Y35301D,Y35302D,Y35303D,Y35309D<br>Y35311D,Y35312D,Y35313D,Y35319D,Y35391D,Y35392D,Y35393D<br>Y35399D,Y35401D,Y35402D,Y35403D,Y35409D,Y35411D,Y35412D<br>Y35413D,Y35419D,Y35491D,Y35492D,Y35493D,Y35499D,Y35811D<br>Y35812D,Y35813D,Y35819D,Y35831D,Y35832D,Y35833D,Y35839D<br>Y35891D,Y35892D,Y35893D,Y35899D,Y3591XD,Y3592XD,Y3593XD<br>Y3599XD,Y36000D,Y36001D,Y36010D,Y36011D,Y36020D,Y36021D<br>Y36030D,Y36031D,Y36040D,Y36041D,Y36050D,Y36051D,Y36090D<br>Y36091D,Y36100D,Y36101D,Y36110D,Y36111D,Y36120D,Y36121D<br>Y36130D,Y36131D,Y36140D,Y36141D,Y36190D,Y36191D,Y36200D<br>Y36201D,Y36210D,Y36211D,Y36220D,Y36221D,Y36230D,Y36231D<br>Y36240D,Y36241D,Y36250D,Y36251D,Y36260D,Y36261D,Y36270D<br>Y36271D,Y36290D,Y36291D,Y36300D,Y36301D,Y36310D,Y36311D<br>Y36320D,Y36321D,Y36330D,Y36331D,Y36390D,Y36391D,Y36410D<br>Y36411D,Y36420D,Y36421D,Y36430D,Y36431D,Y36440D,Y36441D<br>Y36450D,Y36451D,Y36460D,Y36461D,Y36470D,Y36471D,Y36490D<br>Y36491D,Y36500D,Y36501D,Y36510D,Y36511D,Y36520D,Y36521D<br>Y36530D,Y36531D,Y36540D,Y36541D,Y36590D,Y36591D,Y366X0D<br>Y366X1D,Y367X0D,Y367X1D,Y36810D,Y36811D,Y36820D,Y36821D<br>Y36880D,Y36881D,Y36890D,Y36891D,Y3690XD,Y3691XD,Y3692XD<br>Y37000D,Y37001D,Y37010D,Y37011D,Y37020D,Y37021D,Y37030D<br>Y37031D,Y37040D,Y37041D,Y37050D,Y37051D,Y37090D,Y37091D<br>Y37100D,Y37101D,Y37110D,Y37111D,Y37120D,Y37121D,Y37130D<br>Y37131D,Y37140D,Y37141D,Y37190D,Y37191D,Y37200D,Y37201D<br>Y37210D,Y37211D,Y37220D,Y37221D,Y37230D,Y37231D,Y37240D<br>Y37241D,Y37250D,Y37251D,Y37260D,Y37261D,Y37270D,Y37271D<br>Y37290D,Y37291D,Y37300D,Y37301D,Y37310D,Y37311D,Y37320D<br>Y37321D,Y37330D,Y37331D,Y37390D,Y37391D,Y37410D,Y37411D<br>Y37420D,Y37421D,Y37430D,Y37431D,Y37440D,Y37441D,Y37450D<br>Y37451D,Y37460D,Y37461D,Y37470D,Y37471D,Y37490D,Y37491D<br>Y37500D,Y37501D,Y37510D,Y37511D,Y37520D,Y37521D,Y37530D<br>Y37531D,Y37540D,Y37541D,Y37590D,Y37591D,Y376X0D,Y376X1D<br>Y377X0D,Y377X1D,Y3790XD,Y3791XD,Y3792XD,Y380X1D,Y380X2D<br>Y380X3D,Y381X1D,Y381X2D,Y381X3D,Y382X1D,Y382X2D,Y382X3D<br>Y383X1D,Y383X2D,Y383X3D,Y384X1D,Y384X2D,Y384X3D,Y385X1D<br>Y385X2D,Y385X3D,Y386X1D,Y386X2D,Y386X3D,Y387X1D,Y387X2D<br>Y387X3D,Y3880XD,Y38811D,Y38812D,Y38891D,Y38892D,Y38893D<br>Y389X1D,Y389X2D | |
| External cause codes:<br>suffocation<br>inhalation; initial encounter | T17200A,T17210A,T17220A,T17290A,T17300A,T17310A,T17320A<br>T17390A,T17400A,T17410A,T17420A,T17490A,T17500A,T17510A<br>T17520A,T17590A,T17800A,T17810A,T17820A,T17890A,T17900A<br>T17910A,T17920A,T17990A,T71111A,T71112A,T71113A,T71114A<br>T71121A,T71122A,T71123A,T71124A,T71131A,T71132A,T71133A<br>T71134A,T71141A,T71143A,T71144A,T71151A,T71152A,T71153A<br>T71154A,T71161A,T71162A,T71163A,T71164A,T71191A,T71192A<br>T71193A,T71194A,T7120XA,T7121XA,T71221A,T71222A,T71223A | 9330,9331,9340,9341,9348,9349,9947,<br>E911,E912,E9130,E9131<br>E9132,E9133,E9138,E9139,E9530,E9531,<br>E9538,E963,E9830,E9831<br>E9838,E9953,E9954 |

Continued on next page

**Table S9 (continued).**

| Diagnosis | ICD-10 | ICD-9 |
|---|---|---|
| | T71224A,T71231A,T71232A,T71233A,T71234A,T7129XA,T719XXA<br>Y36460A,Y36461A,Y36470A,Y36471A,Y37460A,Y37461A,Y37470A<br>Y37471A | |
| External cause codes: | V2009XA,V200XXA,V2019XA,V201XXA,V2029XA,V202XXA,V2109XA | E8001,E8011,E8018,E8019,E8021,E8031,<br>E8041,E8061,E8071,E8129 |
| transport; not MVT; | V210XXA,V2119XA,V211XXA,V2129XA,V212XXA,V2209XA,V220XXA | E8139,E8199,E8200,E8201,E8208,E8209,<br>E8210,E8211,E8218,E8219 |
| initial encounter | V2219XA,V221XXA,V2229XA,V222XXA,V2309XA,V230XXA,V2319XA | E8220,E8221,E8222,E8223,E8224,E8228,<br>E8229,E8230,E8231,E8232 |
| | V231XXA,V2329XA,V232XXA,V2409XA,V240XXA,V2419XA,V241XXA | E8233,E8238,E8239,E8248,E8250,E8251,<br>E8252,E8253,E8258,E8259 |
| | V2429XA,V242XXA,V2509XA,V250XXA,V2519XA,V251XXA,V2529XA | E8262,E8263,E8272,E8273,E8282,E8294,<br>E8299,E8319,E8359,E8369 |
| | V252XXA,V2609XA,V260XXA,V2619XA,V261XXA,V2629XA,V262XXA | E8379,E8380,E8381,E8384,E8385,E8389,<br>E8401,E8403,E8406,E8411 |
| | V2709XA,V270XXA,V2719XA,V271XXA,V2729XA,V272XXA,V2809XA | E8413,E8426,E8433,E8437,E8439,E8441,<br>E8443,E8447,E8449,E8450 |
| | V280XXA,V2819XA,V281XXA,V2829XA,V282XXA,V29008A,V2900XA | E846,E847,E848,E9580,E9586,E9685,<br>E9791,E9940,E9941,E9942 |
| | V29098A,V2909XA,V29108A,V2910XA,V29198A,V2919XA,V29208A<br>V2920XA,V29298A,V2929XA,V2939XA,V293XXA,V300XXA,V301XXA<br>V302XXA,V303XXA,V310XXA,V311XXA,V312XXA,V313XXA,V320XXA<br>V321XXA,V322XXA,V323XXA,V330XXA,V331XXA,V332XXA,V333XXA<br>V340XXA,V341XXA,V342XXA,V343XXA,V350XXA,V351XXA,V352XXA<br>V353XXA,V360XXA,V361XXA,V362XXA,V363XXA,V370XXA,V371XXA<br>V372XXA,V373XXA,V380XXA,V381XXA,V382XXA,V383XXA,V3900XA<br>V3909XA,V3910XA,V3919XA,V3920XA,V3929XA,V393XXA,V400XXA<br>V401XXA,V402XXA,V403XXA,V410XXA,V411XXA,V412XXA,V413XXA<br>V420XXA,V421XXA,V422XXA,V423XXA,V4301XA,V4302XA,V4303XA<br>V4304XA,V4311XA,V4312XA,V4313XA,V4314XA,V4321XA,V4322XA<br>V4323XA,V4324XA,V4331XA,V4332XA,V4333XA,V4334XA,V440XXA<br>V441XXA,V442XXA,V443XXA,V450XXA,V451XXA,V452XXA,V453XXA<br>V460XXA,V461XXA,V462XXA,V463XXA,V4701XA,V4702XA,V470XXA<br>V4711XA,V4712XA,V471XXA,V472XXA,V4731XA,V4732XA,V473XXA<br>V480XXA,V481XXA,V482XXA,V483XXA,V4900XA,V4909XA,V4910XA<br>V4919XA,V4920XA,V4929XA,V493XXA,V500XXA,V501XXA,V502XXA<br>V503XXA,V510XXA,V511XXA,V512XXA,V513XXA,V520XXA,V521XXA<br>V522XXA,V523XXA,V530XXA,V531XXA,V532XXA,V533XXA,V540XXA<br>V541XXA,V542XXA,V543XXA,V550XXA,V551XXA,V552XXA,V553XXA<br>V560XXA,V561XXA,V562XXA,V563XXA,V570XXA,V571XXA,V572XXA<br>V573XXA,V580XXA,V581XXA,V582XXA,V583XXA,V5900XA,V5909XA<br>V5910XA,V5919XA,V5920XA,V5929XA,V593XXA,V600XXA,V601XXA<br>V602XXA,V603XXA,V610XXA,V611XXA,V612XXA,V613XXA,V620XXA<br>V621XXA,V622XXA,V623XXA,V630XXA,V631XXA,V632XXA,V633XXA<br>V640XXA,V641XXA,V642XXA,V643XXA,V650XXA,V651XXA,V652XXA<br>V653XXA,V660XXA,V661XXA,V662XXA,V663XXA,V670XXA,V671XXA<br>V672XXA,V673XXA,V680XXA,V681XXA,V682XXA,V683XXA,V6900XA<br>V6909XA,V6910XA,V6919XA,V6920XA,V6929XA,V693XXA,V700XXA<br>V701XXA,V702XXA,V703XXA,V710XXA,V711XXA,V712XXA,V713XXA<br>V720XXA,V721XXA,V722XXA,V723XXA,V730XXA,V731XXA,V732XXA<br>V733XXA,V740XXA,V741XXA,V742XXA,V743XXA,V750XXA,V751XXA<br>V752XXA,V753XXA,V760XXA,V761XXA,V762XXA,V763XXA,V770XXA<br>V771XXA,V772XXA,V773XXA,V780XXA,V781XXA,V782XXA,V783XXA<br>V7900XA,V7909XA,V7910XA,V7919XA,V7920XA,V7929XA,V793XXA<br>V80010A,V80018A,V8002XA,V8011XA,V8012XA,V8021XA,V8022XA<br>V8061XA,V8062XA,V80710A,V80711A,V80720A,V80721A,V80730A<br>V80731A,V80790A,V80791A,V8081XA,V8082XA,V80910A,V80918A<br>V80919A,V80920A,V80928A,V80929A,V810XXA,V812XXA,V813XXA<br>V814XXA,V815XXA,V816XXA,V817XXA,V8181XA,V8182XA,V8183XA<br>V8189XA,V819XXA,V820XXA,V822XXA,V823XXA,V824XXA,V825XXA<br>V826XXA,V827XXA,V828XXA,V829XXA,V834XXA,V835XXA,V836XXA<br>V837XXA,V839XXA,V844XXA,V845XXA,V846XXA,V847XXA,V849XXA<br>V854XXA,V855XXA,V856XXA,V857XXA,V859XXA,V8641XA,V8642XA<br>V8643XA,V8644XA,V8645XA,V8646XA,V8649XA,V8651XA,V8652XA<br>V8653XA,V8654XA,V8655XA,V8656XA,V8659XA,V8661XA,V8662XA<br>V8663XA,V8664XA,V8665XA,V8666XA,V8669XA,V8671XA,V8672XA<br>V8673XA,V8674XA,V8675XA,V8676XA,V8679XA,V8691XA,V8692XA<br>V8693XA,V8694XA,V8695XA,V8696XA,V8699XA,V879XXA,V880XXA<br>V881XXA,V882XXA,V883XXA,V884XXA,V885XXA,V886XXA,V887XXA<br>V888XXA,V889XXA,V890XXA,V891XXA,V893XXA,V899XXA,V9100XA<br>V9101XA,V9102XA,V9103XA,V9104XA,V9105XA,V9106XA,V9107XA<br>V9108XA,V9109XA,V9110XA,V9111XA,V9112XA,V9113XA,V9114XA<br>V9115XA,V9116XA,V9118XA,V9119XA,V9120XA,V9121XA,V9122XA<br>V9123XA,V9124XA,V9125XA,V9126XA,V9129XA,V9130XA,V9131XA<br>V9132XA,V9133XA,V9134XA,V9135XA,V9136XA,V9137XA,V9138XA<br>V9139XA,V9180XA,V9181XA,V9182XA,V9183XA,V9184XA,V9185XA<br>V9186XA,V9187XA,V9188XA,V9189XA,V9300XA,V9301XA,V9302XA<br>V9303XA,V9304XA,V9309XA,V9310XA,V9311XA,V9312XA,V9313XA<br>V9314XA,V9319XA,V9320XA,V9321XA,V9322XA,V9323XA,V9324XA<br>V9329XA,V9330XA,V9331XA,V9332XA,V9333XA,V9334XA,V9335XA<br>V9336XA,V9338XA,V9339XA,V9340XA,V9341XA,V9342XA,V9343XA<br>V9344XA,V9348XA,V9349XA,V9350XA,V9351XA,V9352XA,V9353XA<br>V9354XA,V9359XA,V9360XA,V9361XA,V9362XA,V9363XA,V9364XA<br>V9369XA,V9380XA,V9381XA,V9382XA,V9383XA,V9384XA,V9385XA<br>V9386XA,V9387XA,V9388XA,V9389XA,V940XXA,V9411XA,V9412XA | E9943,E9948,E9949 |

Continued on next page

Table S9 (continued).

| Diagnosis | ICD-10 | ICD-9 |
|---|---|---|
| | V9421XA,V9422XA,V9431XA,V9432XA,V944XXA,V94810A,V94811A<br>V94818A,V9489XA,V949XXA,V9500XA,V9501XA,V9502XA,V9503XA<br>V9504XA,V9505XA,V9509XA,V9510XA,V9511XA,V9512XA,V9513XA<br>V9514XA,V9515XA,V9519XA,V9520XA,V9521XA,V9522XA,V9523XA<br>V9524XA,V9525XA,V9529XA,V9530XA,V9531XA,V9532XA,V9533XA<br>V9534XA,V9535XA,V9539XA,V9540XA,V9541XA,V9542XA,V9543XA<br>V9544XA,V9545XA,V9549XA,V958XXA,V959XXA,V9600XA,V9601XA<br>V9602XA,V9603XA,V9604XA,V9605XA,V9609XA,V9610XA,V9611XA<br>V9612XA,V9613XA,V9614XA,V9615XA,V9619XA,V9620XA,V9621XA<br>V9622XA,V9623XA,V9624XA,V9625XA,V9629XA,V968XXA,V969XXA<br>V970XXA,V971XXA,V9721XA,V9722XA,V9729XA,V9731XA,V9732XA<br>V9733XA,V9739XA,V97810A,V97811A,V97818A,V9789XA,V980XXA<br>V981XXA,V982XXA,V983XXA,V988XXA,V99XXXA,X811XXA,X830XXA<br>Y021XXA,Y0881XA,Y36100A,Y36101A,Y36110A,Y36111A,Y36120A<br>Y36121A,Y36130A,Y36131A,Y36140A,Y36141A,Y36190A,Y36191A<br>Y37100A,Y37101A,Y37110A,Y37111A,Y37120A,Y37121A,Y37130A<br>Y37131A,Y37140A,Y37141A,Y37190A,Y37191A,Y381X1A,Y381X2A<br>Y381X3A | |
| External cause codes:<br>unspecified mechanism | R4588,T1491,T1491XA,X58XXXA,Y09,Y35899A,Y3591XA,Y3592XA<br>Y3593XA,Y3599XA,Y36890A,Y36891A,Y3690XA,Y3790XA,Y3880XA | 9599,E9288,E9589,E9689,E976,E9798,<br>E9909,E9989 |
| Socioeconomic<br>psychosocial factors | Z550,Z551,Z552,Z553,Z554,Z555,Z558,Z559,Z560,Z561,Z562,Z563<br>Z564,Z565,Z566,Z5681,Z5682,Z5689,Z569,Z570,Z571,Z572,Z5731<br>Z5739,Z574,Z575,Z576,Z577,Z578,Z579,Z586,Z590,Z5900,Z5901<br>Z5902,Z591,Z592,Z593,Z594,Z5941,Z5948,Z595,Z596,Z597,Z598<br>Z59811,Z59812,Z59819,Z5982,Z5986,Z5987,Z5989,Z599,Z600,Z602<br>Z603,Z604,Z605,Z608,Z609,Z620,Z621,Z6221,Z6222,Z6229,Z623<br>Z626,Z62810,Z62811,Z62812,Z62813,Z62819,Z62820,Z62821,Z62822<br>Z62890,Z62891,Z62898,Z629,Z630,Z631,Z6331,Z6332,Z634,Z635<br>Z636,Z6371,Z6372,Z6379,Z638,Z639,Z640,Z641,Z644,Z650,Z651<br>Z652,Z653,Z654,Z655,Z658,Z659 | V1585,V600,V601,V602,V603,V606,<br>V6081,V6089,V609,V6103,V6106<br>V6107,V6108,V6109,V6129,V613,V6141,<br>V6149,V615,V616,V617,V618<br>V619,V620,V621,V6222,V6229,V623,<br>V624,V625,V6281,V6282,V6289<br>V629 |
| Underdosing of drugs<br>and medicaments,<br>initial encounter | T360X6A,T361X6A,T362X6A,T363X6A,T364X6A,T365X6A,T366X6A<br>T367X6A,T368X6A,T3696XA,T370X6A,T371X6A,T372X6A,T373X6A<br>T374X6A,T375X6A,T378X6A,T3796XA,T380X6A,T381X6A,T382X6A<br>T383X6A,T384X6A,T385X6A,T386X6A,T387X6A,T38806A,T38816A<br>T38896A,T38906A,T38996A,T39016A,T39096A,T391X6A,T392X6A<br>T39316A,T39396A,T394X6A,T398X6A,T3996XA,T400X6A,T402X6A<br>T403X6A,T40416A,T40426A,T40496A,T404X6A,T405X6A,T40606A<br>T40696A,T40716A,T40726A,T407X6A,T40906A,T40996A,T410X6A<br>T411X6A,T41206A,T41296A,T413X6A,T4146XA,T415X6A,T420X6A<br>T421X6A,T422X6A,T423X6A,T424X6A,T425X6A,T426X6A,T4276XA<br>T428X6A,T43016A,T43026A,T431X6A,T43206A,T43216A,T43226A<br>T43296A,T433X6A,T434X6A,T43506A,T43596A,T43606A,T43616A<br>T43626A,T43636A,T43656A,T43696A,T438X6A,T4396XA,T440X6A<br>T441X6A,T442X6A,T443X6A,T444X6A,T445X6A,T446X6A,T447X6A<br>T448X6A,T44906A,T44996A,T450X6A,T451X6A,T452X6A,T453X6A<br>T454X6A,T45516A,T45526A,T45606A,T45616A,T45626A,T45696A<br>T457X6A,T458X6A,T4596XA,T460X6A,T461X6A,T462X6A,T463X6A<br>T464X6A,T465X6A,T466X6A,T467X6A,T468X6A,T46906A,T46996A<br>T470X6A,T471X6A,T472X6A,T473X6A,T474X6A,T475X6A,T476X6A<br>T477X6A,T478X6A,T4796XA,T480X6A,T481X6A,T48206A,T48296A<br>T483X6A,T484X6A,T485X6A,T486X6A,T48906A,T48996A,T490X6A<br>T491X6A,T492X6A,T493X6A,T494X6A,T495X6A,T496X6A,T497X6A<br>T498X6A,T4996XA,T500X6A,T501X6A,T502X6A,T503X6A,T504X6A<br>T505X6A,T506X6A,T507X6A,T508X6A,T50906A,T50916A,T50996A<br>T50A16A,T50A26A,T50A96A,T50B16A,T50B96A,T50Z16A,T50Z96A | NoDx |
| Underdosing of drugs<br>and medicaments,<br>subsequent encounter | T360X6D,T361X6D,T362X6D,T363X6D,T364X6D,T365X6D,T366X6D<br>T367X6D,T368X6D,T3696XD,T370X6D,T371X6D,T372X6D,T373X6D<br>T374X6D,T375X6D,T378X6D,T3796XD,T380X6D,T381X6D,T382X6D<br>T383X6D,T384X6D,T385X6D,T386X6D,T387X6D,T38806D,T38816D<br>T38896D,T38906D,T38996D,T39016D,T39096D,T391X6D,T392X6D<br>T39316D,T39396D,T394X6D,T398X6D,T3996XD,T400X6D,T402X6D<br>T403X6D,T40416D,T40426D,T40496D,T404X6D,T405X6D,T40606D<br>T40696D,T40716D,T40726D,T407X6D,T40906D,T40996D,T410X6D<br>T411X6D,T41206D,T41296D,T413X6D,T4146XD,T415X6D,T420X6D<br>T421X6D,T422X6D,T423X6D,T424X6D,T425X6D,T426X6D,T4276XD<br>T428X6D,T43016D,T43026D,T431X6D,T43206D,T43216D,T43226D<br>T43296D,T433X6D,T434X6D,T43506D,T43596D,T43606D,T43616D<br>T43626D,T43636D,T43656D,T43696D,T438X6D,T4396XD,T440X6D<br>T441X6D,T442X6D,T443X6D,T444X6D,T445X6D,T446X6D,T447X6D<br>T448X6D,T44906D,T44996D,T450X6D,T451X6D,T452X6D,T453X6D<br>T454X6D,T45516D,T45526D,T45606D,T45616D,T45626D,T45696D<br>T457X6D,T458X6D,T4596XD,T460X6D,T461X6D,T462X6D,T463X6D<br>T464X6D,T465X6D,T466X6D,T467X6D,T468X6D,T46906D,T46996D<br>T470X6D,T471X6D,T472X6D,T473X6D,T474X6D,T475X6D,T476X6D<br>T477X6D,T478X6D,T4796XD,T480X6D,T481X6D,T48206D,T48296D<br>T483X6D,T484X6D,T485X6D,T486X6D,T48906D,T48996D,T490X6D<br>T491X6D,T492X6D,T493X6D,T494X6D,T495X6D,T496X6D,T497X6D | NoDx |

Continued on next page

Table S9 (continued).

| Diagnosis | ICD-10 | ICD-9 |
|---|---|---|
| | T498X6D,T4996XD,T500X6D,T501X6D,T502X6D,T503X6D,T504X6D<br>T505X6D,T506X6D,T507X6D,T508X6D,T50906D,T50916D,T50996D<br>T50A16D,T50A26D,T50A96D,T50B16D,T50B96D,T50Z16D,T50Z96D | |
| Vasculitis | D690,I776,L950,L951,L958,L959,M0520,M05211,M05212,M05219<br>M05221,M05222,M05229,M05231,M05232,M05239,M05241,M05242<br>M05249,M05251,M05252,M05259,M05261,M05262,M05269,M05271<br>M05272,M05279,M0529,M300,M301,M302,M303,M308,M310,M311,M3110<br>M3111,M3119,M312,M3130,M3131,M314,M315,M316,M317,M318,M319<br>M350B | 2870,4460,4461,44620,44621,44629,4463,<br>4464,4465,4466,4467<br>4475,4476,69589,7091,7142 |
| Cannabis-related disorders; subsequent encounter | T40711D,T40712D,T40713D,T40714D,T40715D,T40721D,T40722D<br>T40723D,T40724D,T40725D,T407X1D,T407X2D,T407X3D,T407X4D<br>T407X5D | V5889 |
| Diabetes mellitus,<br>Type 1 | E1010,E1011,E1021,E1022,E1029,E10311,E10319,E10321,E103211<br>E103212,E103213,E103219,E10329,E103291,E103292,E103293<br>E103299,E10331,E103311,E103312,E103313,E103319,E10339,E103391<br>E103392,E103393,E103399,E10341,E103411,E103412,E103413<br>E103419,E10349,E103491,E103492,E103493,E103499,E10351,E103511<br>E103512,E103513,E103519,E103521,E103522,E103523,E103529<br>E103531,E103532,E103533,E103539,E103541,E103542,E103543<br>E103549,E103551,E103552,E103553,E103559,E10359,E103591<br>E103592,E103593,E103599,E1036,E1037X1,E1037X2,E1037X3,E1037X9<br>E1039,E1040,E1041,E1042,E1043,E1044,E1049,E1051,E1052,E1059<br>E10610,E10618,E10620,E10621,E10622,E10628,E10630,E10638<br>E10641,E10649,E1065,E1069,E108,E109,O24011,O24012,O24013<br>O24019,O2402,O2403 | 25001,25011,25031,25041,25051,25061,<br>25071,25081,25091,34989<br>3535,3559,3572,36201,36202,36204,36205,<br>36206,36207,36641<br>44381,5238,5363,64800,64801,64803,<br>64804,7135,71680,7854 |
| Diabetes mellitus,<br>Type 2 | E1100,E1101,E1110,E1111,E1121,E1122,E1129,E11311,E11319<br>E11321,E113211,E113212,E113213,E113219,E11329,E113291,E113292<br>E113293,E113299,E11331,E113311,E113312,E113313,E113319,E11339<br>E113391,E113392,E113393,E113399,E11341,E113411,E113412<br>E113413,E113419,E11349,E113491,E113492,E113493,E113499,E11351<br>E113511,E113512,E113513,E113519,E113521,E113522,E113523<br>E113529,E113531,E113532,E113533,E113539,E113541,E113542<br>E113543,E113549,E113551,E113552,E113553,E113559,E11359<br>E113591,E113592,E113593,E113599,E1136,E1137X1,E1137X2,E1137X3<br>E1137X9,E1139,E1140,E1141,E1142,E1143,E1144,E1149,E1151,E1152<br>E1159,E11610,E11618,E11620,E11621,E11622,E11628,E11630,E11638<br>E11641,E11649,E1165,E1169,E118,E119,O24111,O24112,O24113<br>O24119,O2412,O2413 | 25000,25020,25030,25040,25050,25060,<br>25070,25080,25090,34989<br>3535,3559,3572,36201,36202,36204,36205,<br>36206,36207,36641<br>44381,5238,5363,64800,64801,64803,<br>64804,7135,71680,7854 |
| Diabetes mellitus,<br>due to underlying<br>condition, drug or<br>chemical induced, or<br>other specified type | E0800,E0801,E0810,E0811,E0821,E0822,E0829,E08311,E08319<br>E08321,E083211,E083212,E083213,E083219,E08329,E083291,E083292<br>E083293,E083299,E08331,E083311,E083312,E083313,E083319,E08339<br>E083391,E083392,E083393,E083399,E08341,E083411,E083412<br>E083413,E083419,E08349,E083491,E083492,E083493,E083499,E08351<br>E083511,E083512,E083513,E083519,E083521,E083522,E083523<br>E083529,E083531,E083532,E083533,E083539,E083541,E083542<br>E083543,E083549,E083551,E083552,E083553,E083559,E08359<br>E083591,E083592,E083593,E083599,E0836,E0837X1,E0837X2,E0837X3<br>E0837X9,E0839,E0840,E0841,E0842,E0843,E0844,E0849,E0851,E0852<br>E0859,E08610,E08618,E08620,E08621,E08622,E08628,E08630,E08638<br>E08641,E08649,E0865,E0869,E088,E089,E0900,E0901,E0910,E0911<br>E0921,E0922,E0929,E09311,E09319,E09321,E093211,E093212<br>E093213,E093219,E09329,E093291,E093292,E093293,E093299,E09331<br>E093311,E093312,E093313,E093319,E09339,E093391,E093392<br>E093393,E093399,E09341,E093411,E093412,E093413,E093419,E09349<br>E093491,E093492,E093493,E093499,E09351,E093511,E093512<br>E093513,E093519,E093521,E093522,E093523,E093529,E093531<br>E093532,E093533,E093539,E093541,E093542,E093543,E093549<br>E093551,E093552,E093553,E093559,E09359,E093591,E093592<br>E093593,E093599,E0936,E0937X1,E0937X2,E0937X3,E0937X9,E0939<br>E0940,E0941,E0942,E0943,E0944,E0949,E0951,E0952,E0959,E09610<br>E09618,E09620,E09621,E09622,E09628,E09630,E09638,E09641<br>E09649,E0965,E0969,E098,E099,E1300,E1301,E1310,E1311,E1321<br>E1322,E1329,E13311,E13319,E13321,E133211,E133212,E133213<br>E133219,E13329,E133291,E133292,E133293,E133299,E13331,E133311<br>E133312,E133313,E133319,E13339,E133391,E133392,E133393<br>E133399,E13341,E133411,E133412,E133413,E133419,E13349,E133491<br>E133492,E133493,E133499,E13351,E133511,E133512,E133513<br>E133519,E133521,E133522,E133523,E133529,E133531,E133532<br>E133533,E133539,E133541,E133542,E133543,E133549,E133551<br>E133552,E133553,E133559,E13359,E133591,E133592,E133593 | 24900,24910,24920,24930,24940,24950,<br>24960,24970,24980,24990<br>25000,25010,25020,25030,25040,25050,<br>25060,25070,25080,25090<br>2511,34989,3535,3559,3572,36201,36202,<br>36204,36205,36206,36207<br>36641,44381,5238,5289,5363,58181,58381,<br>7098,7135,71680,7854 |

Continued on next page

Table S9 (continued).

| Diagnosis | ICD-10 | ICD-9 |
|---|---|---|
| | E133599,E1336,E1339,E1340,E1341,E1342,E1343,E1344,E1349,E1351 E1352,E1359,E13610,E13618,E13620,E13621,E13622,E13628,E13630 E13638,E13641,E13649,E1365,E1369,E138,E139 | |
| External cause codes: | T1500XA,T1501XA,T1502XA,T1510XA,T1511XA,T1512XA,T1580XA | 9300,9301,9302,9308,9309,931,932,9330, 9331,9340,9341,9348 |
| intent of injury, | T1581XA,T1582XA,T1590XA,T1591XA,T1592XA,T161XXA,T162XXA | 9349,9350,9351,9352,936,937,938,9390, 9391,9392,9393,9399,9600 |
| accidental | T169XXA,T170XXA,T171XXA,T17200A,T17208A,T17210A,T17218A | 9601,9602,9603,9604,9605,9606,9608, 9609,9610,9614,9615,9616 |
| unintentional | T17220A,T17228A,T17290A,T17298A,T17300A,T17308A,T17310A | 9617,9618,9619,9620,9621,9622,9623, 9624,9627,9628,9629,9630 |
| | T17318A,T17320A,T17328A,T17390A,T17398A,T17400A,T17408A | 9631,9634,9635,9638,9639,9640,9642, 9644,9645,9646,9648,9649 |
| | T17410A,T17418A,T17420A,T17428A,T17490A,T17498A,T17500A | 96500,96501,96502,96509,9651,9654,9655, 96561,96569,9657,9659 |
| | T17508A,T17510A,T17518A,T17520A,T17528A,T17590A,T17598A | 9660,9661,9662,9663,9664,9670,9678, 9679,9680,9682,9683,9684 |
| | T17800A,T17808A,T17810A,T17818A,T17820A,T17828A,T17890A | 9685,9689,96900,96901,96902,96903, 96904,96905,96909,9691,9692 |
| | T17898A,T17900A,T17908A,T17910A,T17918A,T17920A,T17928A | 9693,9694,9696,96970,96971,96972,96973, 96979,9698,9699,9700 |
| | T17990A,T17998A,T180XXA,T18100A,T18108A,T18110A,T18118A | 9701,97081,9710,9711,9712,9713,9719, 9720,9721,9722,9723,9724 |
| | T18120A,T18128A,T18190A,T18198A,T182XXA,T183XXA,T184XXA | 9725,9726,9727,9729,9730,9731,9732, 9733,9734,9735,9736,9738 |
| | T185XXA,T188XXA,T189XXA,T190XXA,T191XXA,T192XXA,T193XXA | 9739,9742,9744,9745,9747,9750,9751, 9752,9753,9754,9755,9756 |
| | T194XXA,T198XXA,T199XXA,T360X1A,T361X1A,T362X1A,T363X1A | 9757,9758,9760,9761,9762,9763,9764, 9765,9766,9767,9768,9769 |
| | T364X1A,T365X1A,T366X1A,T367X1A,T368X1A,T3691XA,T370X1A | 9770,9772,9778,9779,9786,9788,9789, 9790,9796,9799,9800,9801 |
| | T371X1A,T372X1A,T373X1A,T374X1A,T375X1A,T378X1A,T3791XA | 9802,9803,9808,9809,981,9820,9821,9822, 9823,9824,9828,9830 |
| | T380X1A,T381X1A,T382X1A,T383X1A,T384X1A,T385X1A,T386X1A | 9831,9832,9839,9849,9850,9851,9852, 9853,9855,9856,9858,9859 |
| | T387X1A,T38801A,T38811A,T38891A,T38901A,T38991A,T39011A | 986,9871,9872,9873,9874,9875,9876,9877, 9878,9879,9880,9881 |
| | T39091A,T391X1A,T392X1A,T39311A,T39391A,T394X1A,T398X1A | 9882,9888,9889,9890,9891,9893,9894, 9895,9896,9897,98982,98984 |
| | T3991XA,T400X1A,T401X1A,T402X1A,T403X1A,T40411A,T40421A | 98989,9899,9940,9942,9943,9944,9945, 9946,9947,9948,9949,E8001 |
| | T40491A,T404X1A,T405X1A,T40601A,T40691A,T40711A,T40721A | E8011,E8013,E8018,E8019,E8021,E8031, E8041,E8052,E8061,E8071 |
| | T407X1A,T408X1A,T40901A,T40991A,T410X1A,T411X1A,T41201A | E8072,E8100,E8101,E8102,E8103,E8107, E8108,E8109,E8120,E8121 |
| | T41291A,T413X1A,T4141XA,T415X1A,T420X1A,T421X1A,T422X1A | E8122,E8123,E8128,E8129,E8130,E8131, E8132,E8133,E8134,E8135 |
| | T423X1A,T424X1A,T425X1A,T426X1A,T4271XA,T428X1A,T43011A | E8136,E8138,E8139,E8140,E8141,E8142, E8143,E8147,E8148,E8149 |
| | T43021A,T431X1A,T43201A,T43211A,T43221A,T43291A,T433X1A | E8150,E8151,E8152,E8153,E8158,E8159, E8160,E8161,E8162,E8163 |
| | T434X1A,T43501A,T43591A,T43601A,T43611A,T43621A,T43631A | E8168,E8169,E8178,E8188,E8189,E8190, E8192,E8199,E8200,E8201 |
| | T43641A,T43651A,T43691A,T438X1A,T4391XA,T440X1A,T441X1A | E8208,E8209,E8210,E8211,E8218,E8219, E8220,E8221,E8222,E8223 |
| | T442X1A,T443X1A,T444X1A,T445X1A,T446X1A,T447X1A,T448X1A | E8224,E8226,E8227,E8228,E8229,E8230, E8231,E8232,E8233,E8238 |
| | T44901A,T44991A,T450X1A,T451X1A,T452X1A,T453X1A,T454X1A | E8239,E8242,E8246,E8248,E8250,E8251, E8252,E8253,E8258,E8259 |
| | T45511A,T45521A,T45601A,T45611A,T45621A,T45691A,T457X1A | E8260,E8261,E8262,E8263,E8269,E8272, E8273,E8282,E8290,E8294 |
| | T458X1A,T4591XA,T460X1A,T461X1A,T462X1A,T463X1A,T464X1A | E8299,E8309,E8319,E8329,E8359,E8369, E8379,E8380,E8381,E8384 |
| | T465X1A,T466X1A,T467X1A,T468X1A,T46901A,T46991A,T470X1A | E8385,E8389,E8401,E8403,E8406,E8411, E8413,E8426,E8433,E8437 |
| | T471X1A,T472X1A,T473X1A,T474X1A,T475X1A,T476X1A,T477X1A | E8439,E8441,E8443,E8447,E8449,E8450, E846,E847,E848,E8500 |
| | T478X1A,T4791XA,T480X1A,T481X1A,T48201A,T48291A,T483X1A | E8501,E8502,E8503,E8504,E8505,E8506, E8507,E851,E8528,E8530 |
| | T484X1A,T485X1A,T486X1A,T48901A,T48991A,T490X1A,T491X1A | E8531,E8532,E8540,E8541,E8542,E8543, E8548,E8550,E8551,E8552 |
| | T492X1A,T493X1A,T494X1A,T495X1A,T496X1A,T497X1A,T498X1A | E8553,E8554,E8555,E8556,E8558,E856, E857,E8580,E8581,E8582 |
| | T4991XA,T500X1A,T501X1A,T502X1A,T503X1A,T504X1A,T505X1A | E8583,E8584,E8585,E8586,E8587,E8588, E8601,E8602,E8603,E8604 |
| | T506X1A,T507X1A,T508X1A,T50901A,T50911A,T50991A,T50A11A | E8608,E8609,E8610,E8611,E8616,E8621, E8624,E8631,E8634,E8635 |
| | T50A21A,T50A91A,T50B11A,T50B91A,T50Z11A,T50Z91A,T510X1A | E8637,E8640,E8641,E8643,E8644,E8651, E8652,E8653,E8654,E8655 |
| | T511X1A,T512X1A,T513X1A,T518X1A,T5191XA,T520X1A,T521X1A | E8658,E8659,E8660,E8661,E8663,E8664, E8668,E8681,E8682,E8683 |
| | T522X1A,T523X1A,T524X1A,T528X1A,T5291XA,T530X1A,T531X1A | E8688,E8689,E8690,E8691,E8692,E8693, E8698,E8800,E8801,E8809 |
| | T532X1A,T533X1A,T534X1A,T535X1A,T536X1A,T537X1A,T5391XA | E8810,E8811,E882,E8830,E8831,E8832, E8839,E8840,E8841,E8842 |
| | T540X1A,T541X1A,T542X1A,T543X1A,T5491XA,T550X1A,T551X1A | E8843,E8844,E8845,E8846,E8849,E8850, E8851,E8852,E8853,E8854 |

Continued on next page

Table S9 (continued).

| Diagnosis | ICD-10 | ICD-9 |
|---|---|---|
| | T560X1A,T561X1A,T562X1A,T563X1A,T564X1A,T565X1A,T566X1A | E8859,E8860,E8869,E8880,E8881,E8888, E8889,E8902,E8903,E8908 |
| | T567X1A,T56811A,T56891A,T5691XA,T570X1A,T571X1A,T572X1A | E892,E8930,E8938,E894,E896,E897, E8980,E8981,E9000,E9001,E9010 |
| | T573X1A,T578X1A,T5791XA,T5801XA,T5811XA,T582X1A,T588X1A | E9011,E9018,E9020,E9021,E9022,E9028, E903,E9041,E9042,E9043 |
| | T5891XA,T590X1A,T591X1A,T592X1A,T593X1A,T594X1A,T595X1A | E9049,E9050,E9051,E9052,E9053,E9054, E9055,E9056,E9060,E9061 |
| | T596X1A,T597X1A,T59811A,T59891A,T5991XA,T600X1A,T601X1A | E9062,E9063,E9064,E9068,E9069,E9080, E9081,E9082,E9083,E9084 |
| | T602X1A,T603X1A,T604X1A,T608X1A,T6091XA,T6101XA,T6111XA | E9088,E9089,E9090,E9091,E9092,E9093, E9094,E9098,E9102,E9104 |
| | T61771A,T61781A,T618X1A,T6191XA,T620X1A,T621X1A,T622X1A | E9108,E9109,E911,E912,E9130,E9131, E9132,E9133,E9138,E9139 |
| | T628X1A,T6291XA,T63001A,T63011A,T63021A,T63031A,T63041A | E914,E915,E916,E9170,E9171,E9173, E9174,E9178,E9179,E918,E9190 |
| | T63061A,T63071A,T63081A,T63091A,T63111A,T63121A,T63191A | E9191,E9192,E9193,E9194,E9195,E9196, E9197,E9198,E9199,E9200 |
| | T632X1A,T63301A,T63311A,T63321A,T63331A,T63391A,T63411A | E9201,E9202,E9203,E9204,E9205,E9208, E9210,E9211,E9218,E9220 |
| | T63421A,T63431A,T63441A,T63451A,T63461A,T63481A,T63511A | E9221,E9222,E9223,E9224,E9225,E9228, E9229,E9230,E9231,E9232 |
| | T63591A,T63611A,T63621A,T63631A,T63691A,T63711A,T63791A | E9238,E9239,E9240,E9241,E9242,E9248, E9250,E9251,E9252,E9258 |
| | T63811A,T63821A,T63831A,T63891A,T6391XA,T6401XA,T6481XA | E9260,E9261,E9262,E9263,E9264,E9265, E9268,E9272,E9280,E9281 |
| | T650X1A,T651X1A,T65211A,T65221A,T65291A,T653X1A,T654X1A | E9283,E9284,E9285,E9286,E9288 |
| | T655X1A,T656X1A,T65811A,T65821A,T65831A,T65891A,T6591XA | |
| | T71111A,T71121A,T71131A,T71141A,T71151A,T71161A,T71191A | |
| | T7120XA,T7121XA,T71221A,T71231A,T7129XA,T719XXA,T730XXA | |
| | T731XXA,T732XXA,T733XXA,T738XXA,T739XXA,T7500XA,T7501XA | |
| | T7509XA,T7520XA,T7521XA,T7522XA,T7523XA,T7529XA,T753XXA | |
| | T754XXA,V0001XA,V0002XA,V00031A,V00038A,V0009XA,V00111A | |
| | V00112A,V00118A,V00121A,V00122A,V00128A,V00131A,V00132A | |
| | V00138A,V00141A,V00142A,V00148A,V00151A,V00152A,V00158A | |
| | V00181A,V00182A,V00188A,V00211A,V00212A,V00218A,V00221A | |
| | V00222A,V00228A,V00281A,V00282A,V00288A,V00311A,V00312A | |
| | V00318A,V00321A,V00322A,V00328A,V00381A,V00382A,V00388A | |
| | V00811A,V00812A,V00818A,V00821A,V00822A,V00828A,V00831A | |
| | V00832A,V00838A,V00841A,V00842A,V00848A,V00891A,V00892A | |
| | V00898A,V0100XA,V0101XA,V0102XA,V01031A,V01038A,V0109XA | |
| | V0110XA,V0111XA,V0112XA,V01131A,V01138A,V0119XA,V0190XA | |
| | V0191XA,V0192XA,V01931A,V01938A,V0199XA,V0200XA,V0201XA | |
| | V0202XA,V02031A,V02038A,V0209XA,V0210XA,V0211XA,V0212XA | |
| | V02131A,V02138A,V0219XA,V0290XA,V0291XA,V0292XA,V02931A | |
| | V02938A,V0299XA,V0300XA,V0301XA,V0302XA,V03031A,V03038A | |
| | V0309XA,V0310XA,V0311XA,V0312XA,V03131A,V03138A,V0319XA | |
| | V0390XA,V0391XA,V0392XA,V03931A,V03938A,V0399XA,V0400XA | |
| | V0401XA,V0402XA,V04031A,V04038A,V0409XA,V0410XA,V0411XA | |
| | V0412XA,V04131A,V04138A,V0419XA,V0490XA,V0491XA,V0492XA | |
| | V04931A,V04938A,V0499XA,V0500XA,V0501XA,V0502XA,V05031A | |
| | V05038A,V0509XA,V0510XA,V0511XA,V0512XA,V05131A,V05138A | |
| | V0519XA,V0590XA,V0591XA,V0592XA,V05931A,V05938A,V0599XA | |
| | V0600XA,V0601XA,V0602XA,V06031A,V06038A,V0609XA,V0610XA | |
| | V0611XA,V0612XA,V06131A,V06138A,V0619XA,V0690XA,V0691XA | |
| | V0692XA,V06931A,V06938A,V0699XA,V0900XA,V0901XA,V0909XA | |
| | V091XXA,V0920XA,V0921XA,V0929XA,V093XXA,V099XXA,V100XXA | |
| | V101XXA,V102XXA,V103XXA,V104XXA,V105XXA,V109XXA,V110XXA | |
| | V111XXA,V112XXA,V113XXA,V114XXA,V115XXA,V119XXA,V120XXA | |
| | V121XXA,V122XXA,V123XXA,V124XXA,V125XXA,V129XXA,V130XXA | |
| | V131XXA,V132XXA,V133XXA,V134XXA,V135XXA,V139XXA,V140XXA | |
| | V141XXA,V142XXA,V143XXA,V144XXA,V145XXA,V149XXA,V150XXA | |
| | V151XXA,V152XXA,V153XXA,V154XXA,V155XXA,V159XXA,V160XXA | |
| | V161XXA,V162XXA,V163XXA,V164XXA,V165XXA,V169XXA,V170XXA | |
| | V171XXA,V172XXA,V173XXA,V174XXA,V175XXA,V179XXA,V180XXA | |
| | V181XXA,V182XXA,V183XXA,V184XXA,V185XXA,V189XXA,V1900XA | |
| | V1909XA,V1910XA,V1919XA,V1920XA,V1929XA,V193XXA,V1940XA | |
| | V1949XA,V1950XA,V1959XA,V1960XA,V1969XA,V1981XA,V1988XA | |
| | V199XXA,V2001XA,V2009XA,V200XXA,V2011XA,V2019XA,V201XXA | |
| | V2021XA,V2029XA,V202XXA,V2031XA,V2039XA,V203XXA,V2041XA | |
| | V2049XA,V204XXA,V2051XA,V2059XA,V205XXA,V2091XA,V2099XA | |
| | V209XXA,V2101XA,V2109XA,V210XXA,V2111XA,V2119XA,V211XXA | |
| | V2121XA,V2129XA,V212XXA,V2131XA,V2139XA,V213XXA,V2141XA | |
| | V2149XA,V214XXA,V2151XA,V2159XA,V215XXA,V2191XA,V2199XA | |
| | V219XXA,V2201XA,V2209XA,V220XXA,V2211XA,V2219XA,V221XXA | |
| | V2221XA,V2229XA,V222XXA,V2231XA,V2239XA,V223XXA,V2241XA | |
| | V2249XA,V224XXA,V2251XA,V2259XA,V225XXA,V2291XA,V2299XA | |
| | V229XXA,V2301XA,V2309XA,V230XXA,V2311XA,V2319XA,V231XXA | |
| | V2321XA,V2329XA,V232XXA,V2331XA,V2339XA,V233XXA,V2341XA | |
| | V2349XA,V234XXA,V2351XA,V2359XA,V235XXA,V2391XA,V2399XA | |
| | V239XXA,V2401XA,V2409XA,V240XXA,V2411XA,V2419XA,V241XXA | |
| | V2421XA,V2429XA,V242XXA,V2431XA,V2439XA,V243XXA,V2441XA | |
| | V2449XA,V244XXA,V2451XA,V2459XA,V245XXA,V2491XA,V2499XA | |
| | V249XXA,V2501XA,V2509XA,V250XXA,V2511XA,V2519XA,V251XXA | |
| | V2521XA,V2529XA,V252XXA,V2531XA,V2539XA,V253XXA,V2541XA | |
| | V2549XA,V254XXA,V2551XA,V2559XA,V255XXA,V2591XA,V2599XA | |

Continued on next page

Table S9 (continued).

| Diagnosis | ICD-10 | ICD-9 |
| --- | --- | --- |
| | V259XXA,V2601XA,V2609XA,V260XXA,V2611XA,V2619XA,V261XXA V2621XA,V2629XA,V262XXA,V2631XA,V2639XA,V263XXA,V2641XA V2649XA,V264XXA,V2651XA,V2659XA,V265XXA,V2691XA,V2699XA V269XXA,V2701XA,V2709XA,V270XXA,V2711XA,V2719XA,V271XXA V2721XA,V2729XA,V272XXA,V2731XA,V2739XA,V273XXA,V2741XA V2749XA,V274XXA,V2751XA,V2759XA,V275XXA,V2791XA,V2799XA V279XXA,V2801XA,V2809XA,V280XXA,V2811XA,V2819XA,V281XXA V2821XA,V2829XA,V282XXA,V2831XA,V2839XA,V283XXA,V2841XA V2849XA,V284XXA,V2851XA,V2859XA,V285XXA,V2891XA,V2899XA V289XXA,V29001A,V29008A,V2900XA,V29091A,V29098A,V2909XA V29101A,V29108A,V2910XA,V29191A,V29198A,V2919XA,V29201A V29208A,V2920XA,V29291A,V29298A,V2929XA,V2931XA,V2939XA V293XXA,V29401A,V29408A,V2940XA,V29491A,V29498A,V2949XA V29501A,V29508A,V2950XA,V29591A,V29598A,V2959XA,V29601A V29608A,V2960XA,V29691A,V29698A,V2969XA,V29811A,V29818A V2981XA,V29881A,V29888A,V2988XA,V2991XA,V2999XA,V299XXA V300XXA,V301XXA,V302XXA,V303XXA,V304XXA,V305XXA,V306XXA V307XXA,V309XXA,V310XXA,V311XXA,V312XXA,V313XXA,V314XXA V315XXA,V316XXA,V317XXA,V319XXA,V320XXA,V321XXA,V322XXA V323XXA,V324XXA,V325XXA,V326XXA,V327XXA,V329XXA,V330XXA V331XXA,V332XXA,V333XXA,V334XXA,V335XXA,V336XXA,V337XXA V339XXA,V340XXA,V341XXA,V342XXA,V343XXA,V344XXA,V345XXA V346XXA,V347XXA,V349XXA,V350XXA,V351XXA,V352XXA,V353XXA V354XXA,V355XXA,V356XXA,V357XXA,V359XXA,V360XXA,V361XXA V362XXA,V363XXA,V364XXA,V365XXA,V366XXA,V367XXA,V369XXA V370XXA,V371XXA,V372XXA,V373XXA,V374XXA,V375XXA,V376XXA V377XXA,V379XXA,V380XXA,V381XXA,V382XXA,V383XXA,V384XXA V385XXA,V386XXA,V387XXA,V389XXA,V3900XA,V3909XA,V3910XA V3919XA,V3920XA,V3929XA,V393XXA,V3940XA,V3949XA,V3950XA V3959XA,V3960XA,V3969XA,V3981XA,V3989XA,V399XXA,V400XXA V401XXA,V402XXA,V403XXA,V404XXA,V405XXA,V406XXA,V407XXA V409XXA,V410XXA,V411XXA,V412XXA,V413XXA,V414XXA,V415XXA V416XXA,V417XXA,V419XXA,V420XXA,V421XXA,V422XXA,V423XXA V424XXA,V425XXA,V426XXA,V427XXA,V429XXA,V4301XA,V4302XA V4303XA,V4304XA,V4311XA,V4312XA,V4313XA,V4314XA,V4321XA V4322XA,V4323XA,V4324XA,V4331XA,V4332XA,V4333XA,V4334XA V4341XA,V4342XA,V4343XA,V4344XA,V4351XA,V4352XA,V4353XA V4354XA,V4361XA,V4362XA,V4363XA,V4364XA,V4371XA,V4372XA V4373XA,V4374XA,V4391XA,V4392XA,V4393XA,V4394XA,V440XXA V441XXA,V442XXA,V443XXA,V444XXA,V445XXA,V446XXA,V447XXA V449XXA,V450XXA,V451XXA,V452XXA,V453XXA,V454XXA,V455XXA V456XXA,V457XXA,V459XXA,V460XXA,V461XXA,V462XXA,V463XXA V464XXA,V465XXA,V466XXA,V467XXA,V469XXA,V4701XA,V4702XA V470XXA,V4711XA,V4712XA,V471XXA,V472XXA,V4731XA,V4732XA V473XXA,V474XXA,V4751XA,V4752XA,V475XXA,V4761XA,V4762XA V476XXA,V477XXA,V4791XA,V4792XA,V479XXA,V480XXA,V481XXA V482XXA,V483XXA,V484XXA,V485XXA,V486XXA,V487XXA,V489XXA V4900XA,V4909XA,V4910XA,V4919XA,V4920XA,V4929XA,V493XXA V4940XA,V4949XA,V4950XA,V4959XA,V4960XA,V4969XA,V4981XA V4988XA,V499XXA,V500XXA,V501XXA,V502XXA,V503XXA,V504XXA V505XXA,V506XXA,V507XXA,V509XXA,V510XXA,V511XXA,V512XXA V513XXA,V514XXA,V515XXA,V516XXA,V517XXA,V519XXA,V520XXA V521XXA,V522XXA,V523XXA,V524XXA,V525XXA,V526XXA,V527XXA V529XXA,V530XXA,V531XXA,V532XXA,V533XXA,V534XXA,V535XXA V536XXA,V537XXA,V539XXA,V540XXA,V541XXA,V542XXA,V543XXA V544XXA,V545XXA,V546XXA,V547XXA,V549XXA,V550XXA,V551XXA V552XXA,V553XXA,V554XXA,V555XXA,V556XXA,V557XXA,V559XXA V560XXA,V561XXA,V562XXA,V563XXA,V564XXA,V565XXA,V566XXA V567XXA,V569XXA,V570XXA,V571XXA,V572XXA,V573XXA,V574XXA V575XXA,V576XXA,V577XXA,V579XXA,V580XXA,V581XXA,V582XXA V583XXA,V584XXA,V585XXA,V586XXA,V587XXA,V589XXA,V5900XA V5909XA,V5910XA,V5919XA,V5920XA,V5929XA,V593XXA,V5940XA V5949XA,V5950XA,V5959XA,V5960XA,V5969XA,V5981XA,V5988XA V599XXA,V600XXA,V601XXA,V602XXA,V603XXA,V604XXA,V605XXA V606XXA,V607XXA,V609XXA,V610XXA,V611XXA,V612XXA,V613XXA V614XXA,V615XXA,V616XXA,V617XXA,V619XXA,V620XXA,V621XXA V622XXA,V623XXA,V624XXA,V625XXA,V626XXA,V627XXA,V629XXA V630XXA,V631XXA,V632XXA,V633XXA,V634XXA,V635XXA,V636XXA V637XXA,V639XXA,V640XXA,V641XXA,V642XXA,V643XXA,V644XXA V645XXA,V646XXA,V647XXA,V649XXA,V650XXA,V651XXA,V652XXA V653XXA,V654XXA,V655XXA,V656XXA,V657XXA,V659XXA,V660XXA V661XXA,V662XXA,V663XXA,V664XXA,V665XXA,V666XXA,V667XXA V669XXA,V670XXA,V671XXA,V672XXA,V673XXA,V674XXA,V675XXA V676XXA,V677XXA,V679XXA,V680XXA,V681XXA,V682XXA,V683XXA V684XXA,V685XXA,V686XXA,V687XXA,V689XXA,V6900XA,V6909XA V6910XA,V6919XA,V6920XA,V6929XA,V693XXA,V6940XA,V6949XA V6950XA,V6959XA,V6960XA,V6969XA,V6981XA,V6988XA,V699XXA V700XXA,V701XXA,V702XXA,V703XXA,V704XXA,V705XXA,V706XXA V707XXA,V709XXA,V710XXA,V711XXA,V712XXA,V713XXA,V714XXA V715XXA,V716XXA,V717XXA,V719XXA,V720XXA,V721XXA,V722XXA V723XXA,V724XXA,V725XXA,V726XXA,V727XXA,V729XXA,V730XXA V731XXA,V732XXA,V733XXA,V734XXA,V735XXA,V736XXA,V737XXA V739XXA,V740XXA,V741XXA,V742XXA,V743XXA,V744XXA,V745XXA V746XXA,V747XXA,V749XXA,V750XXA,V751XXA,V752XXA,V753XXA V754XXA,V755XXA,V756XXA,V757XXA,V759XXA,V760XXA,V761XXA | |

Continued on next page

Table S9 (continued).

| Diagnosis | ICD-10 | ICD-9 |
|---|---|---|
| | V762XXA,V763XXA,V764XXA,V765XXA,V766XXA,V767XXA,V769XXA | |
| | V770XXA,V771XXA,V772XXA,V773XXA,V774XXA,V775XXA,V776XXA | |
| | V777XXA,V779XXA,V780XXA,V781XXA,V782XXA,V783XXA,V784XXA | |
| | V785XXA,V786XXA,V787XXA,V789XXA,V7900XA,V7909XA,V7910XA | |
| | V7919XA,V7920XA,V7929XA,V793XXA,V7940XA,V7949XA,V7950XA | |
| | V7959XA,V7960XA,V7969XA,V7981XA,V7988XA,V799XXA,V80010A | |
| | V80018A,V8002XA,V8011XA,V8012XA,V8021XA,V8022XA,V8031XA | |
| | V8032XA,V8041XA,V8042XA,V8051XA,V8052XA,V8061XA,V8062XA | |
| | V80710A,V80711A,V80720A,V80721A,V80730A,V80731A,V80790A | |
| | V80791A,V8081XA,V8082XA,V80910A,V80918A,V80919A,V80920A | |
| | V80928A,V80929A,V810XXA,V811XXA,V812XXA,V813XXA,V814XXA | |
| | V815XXA,V816XXA,V817XXA,V8181XA,V8182XA,V8183XA,V8189XA | |
| | V819XXA,V820XXA,V821XXA,V822XXA,V823XXA,V824XXA,V825XXA | |
| | V826XXA,V827XXA,V828XXA,V829XXA,V830XXA,V831XXA,V832XXA | |
| | V833XXA,V834XXA,V835XXA,V836XXA,V837XXA,V839XXA,V840XXA | |
| | V841XXA,V842XXA,V843XXA,V844XXA,V845XXA,V846XXA,V847XXA | |
| | V849XXA,V850XXA,V851XXA,V852XXA,V853XXA,V854XXA,V855XXA | |
| | V856XXA,V857XXA,V859XXA,V8601XA,V8602XA,V8603XA,V8604XA | |
| | V8605XA,V8606XA,V8609XA,V8611XA,V8612XA,V8613XA,V8614XA | |
| | V8615XA,V8616XA,V8619XA,V8621XA,V8622XA,V8623XA,V8624XA | |
| | V8625XA,V8626XA,V8629XA,V8631XA,V8632XA,V8633XA,V8634XA | |
| | V8635XA,V8636XA,V8639XA,V8641XA,V8642XA,V8643XA,V8644XA | |
| | V8645XA,V8646XA,V8649XA,V8651XA,V8652XA,V8653XA,V8654XA | |
| | V8655XA,V8656XA,V8659XA,V8661XA,V8662XA,V8663XA,V8664XA | |
| | V8665XA,V8666XA,V8669XA,V8671XA,V8672XA,V8673XA,V8674XA | |
| | V8675XA,V8676XA,V8679XA,V8691XA,V8692XA,V8693XA,V8694XA | |
| | V8695XA,V8696XA,V8699XA,V870XXA,V871XXA,V872XXA,V873XXA | |
| | V874XXA,V875XXA,V876XXA,V877XXA,V878XXA,V879XXA,V880XXA | |
| | V881XXA,V882XXA,V883XXA,V884XXA,V885XXA,V886XXA,V887XXA | |
| | V888XXA,V889XXA,V890XXA,V891XXA,V892XXA,V893XXA,V899XXA | |
| | V9000XA,V9001XA,V9002XA,V9003XA,V9004XA,V9005XA,V9006XA | |
| | V9008XA,V9009XA,V9010XA,V9011XA,V9012XA,V9013XA,V9014XA | |
| | V9015XA,V9016XA,V9018XA,V9019XA,V9020XA,V9021XA,V9022XA | |
| | V9023XA,V9024XA,V9025XA,V9026XA,V9027XA,V9028XA,V9029XA | |
| | V9030XA,V9031XA,V9032XA,V9033XA,V9034XA,V9035XA,V9036XA | |
| | V9037XA,V9038XA,V9039XA,V9080XA,V9081XA,V9082XA,V9083XA | |
| | V9084XA,V9085XA,V9086XA,V9087XA,V9088XA,V9089XA,V9100XA | |
| | V9101XA,V9102XA,V9103XA,V9104XA,V9105XA,V9106XA,V9107XA | |
| | V9108XA,V9109XA,V9110XA,V9111XA,V9112XA,V9113XA,V9114XA | |
| | V9115XA,V9116XA,V9118XA,V9119XA,V9120XA,V9121XA,V9122XA | |
| | V9123XA,V9124XA,V9125XA,V9126XA,V9129XA,V9130XA,V9131XA | |
| | V9132XA,V9133XA,V9134XA,V9135XA,V9136XA,V9137XA,V9138XA | |
| | V9139XA,V9180XA,V9181XA,V9182XA,V9183XA,V9184XA,V9185XA | |
| | V9186XA,V9187XA,V9188XA,V9189XA,V9200XA,V9201XA,V9202XA | |
| | V9203XA,V9204XA,V9205XA,V9206XA,V9207XA,V9208XA,V9209XA | |
| | V9210XA,V9211XA,V9212XA,V9213XA,V9214XA,V9215XA,V9216XA | |
| | V9219XA,V9220XA,V9221XA,V9222XA,V9223XA,V9224XA,V9225XA | |
| | V9226XA,V9227XA,V9228XA,V9229XA,V9300XA,V9301XA,V9302XA | |
| | V9303XA,V9304XA,V9309XA,V9310XA,V9311XA,V9312XA,V9313XA | |
| | V9314XA,V9319XA,V9320XA,V9321XA,V9322XA,V9323XA,V9324XA | |
| | V9329XA,V9330XA,V9331XA,V9332XA,V9333XA,V9334XA,V9335XA | |
| | V9336XA,V9338XA,V9339XA,V9340XA,V9341XA,V9342XA,V9343XA | |
| | V9344XA,V9348XA,V9349XA,V9350XA,V9351XA,V9352XA,V9353XA | |
| | V9354XA,V9359XA,V9360XA,V9361XA,V9362XA,V9363XA,V9364XA | |
| | V9369XA,V9380XA,V9381XA,V9382XA,V9383XA,V9384XA,V9385XA | |
| | V9386XA,V9387XA,V9388XA,V9389XA,V940XXA,V9411XA,V9412XA | |
| | V9421XA,V9422XA,V9431XA,V9432XA,V944XXA,V94810A,V94811A | |
| | V94818A,V9489XA,V949XXA,V9500XA,V9501XA,V9502XA,V9503XA | |
| | V9504XA,V9505XA,V9509XA,V9510XA,V9511XA,V9512XA,V9513XA | |
| | V9514XA,V9515XA,V9519XA,V9520XA,V9521XA,V9522XA,V9523XA | |
| | V9524XA,V9525XA,V9529XA,V9530XA,V9531XA,V9532XA,V9533XA | |
| | V9534XA,V9535XA,V9539XA,V9540XA,V9541XA,V9542XA,V9543XA | |
| | V9544XA,V9545XA,V9549XA,V958XXA,V959XXA,V9600XA,V9601XA | |
| | V9602XA,V9603XA,V9604XA,V9605XA,V9609XA,V9610XA,V9611XA | |
| | V9612XA,V9613XA,V9614XA,V9615XA,V9619XA,V9620XA,V9621XA | |
| | V9622XA,V9623XA,V9624XA,V9625XA,V9629XA,V968XXA,V969XXA | |
| | V970XXA,V971XXA,V9721XA,V9722XA,V9729XA,V9731XA,V9732XA | |
| | V9733XA,V9739XA,V97810A,V97811A,V97818A,V9789XA,V980XXA | |
| | V981XXA,V982XXA,V983XXA,V988XXA,V99XXXA,W000XXA,W001XXA | |
| | W002XXA,W009XXA,W010XXA,W0110XA,W01110A,W01111A,W01118A | |
| | W01119A,W01190A,W01198A,W03XXXA,W04XXXA,W050XXA,W051XXA | |
| | W052XXA,W06XXXA,W07XXXA,W08XXXA,W090XXA,W091XXA,W092XXA | |
| | W098XXA,W100XXA,W101XXA,W102XXA,W108XXA,W109XXA,W11XXXA | |
| | W12XXXA,W130XXA,W131XXA,W132XXA,W133XXA,W134XXA,W138XXA | |
| | W139XXA,W14XXXA,W15XXXA,W16011A,W16012A,W16021A,W16022A | |
| | W16031A,W16032A,W16111A,W16112A,W16121A,W16122A,W16131A | |
| | W16132A,W16211A,W16212A,W16221A,W16222A,W16311A,W16312A | |
| | W16321A,W16322A,W16331A,W16332A,W1641XA,W1642XA,W16511A | |
| | W16512A,W16521A,W16522A,W16531A,W16532A,W16611A,W16612A | |
| | W16621A,W16622A,W16711A,W16712A,W16721A,W16722A,W16811A | |
| | W16812A,W16821A,W16822A,W16831A,W16832A,W1691XA,W1692XA | |
| | W170XXA,W171XXA,W172XXA,W173XXA,W174XXA,W1781XA,W1782XA | |
| | W1789XA,W1800XA,W1801XA,W1802XA,W1809XA,W1811XA,W1812XA | |
| | W182XXA,W1830XA,W1831XA,W1839XA,W1840XA,W1841XA,W1842XA | |
| | W1843XA,W1849XA,W19XXXA,W200XXA,W201XXA,W208XXA,W2100XA | |

Continued on next page

Table S9 (continued).

| Diagnosis | ICD-10 | ICD-9 |
|---|---|---|
| | W2101XA,W2102XA,W2103XA,W2104XA,W2105XA,W2106XA,W2107XA<br>W2109XA,W2111XA,W2112XA,W2113XA,W2119XA,W21210A,W21211A<br>W21220A,W21221A,W2131XA,W2132XA,W2139XA,W214XXA,W2181XA<br>W2189XA,W219XXA,W2201XA,W2202XA,W2203XA,W22041A,W22042A<br>W2209XA,W2210XA,W2211XA,W2212XA,W2219XA,W228XXA,W230XXA<br>W231XXA,W232XXA,W240XXA,W241XXA,W25XXXA,W260XXA,W261XXA<br>W262XXA,W268XXA,W269XXA,W270XXA,W271XXA,W272XXA,W273XXA<br>W274XXA,W275XXA,W278XXA,W28XXXA,W290XXA,W291XXA,W292XXA<br>W293XXA,W294XXA,W298XXA,W300XXA,W301XXA,W302XXA,W303XXA<br>W3081XA,W3089XA,W309XXA,W310XXA,W311XXA,W312XXA,W313XXA<br>W3181XA,W3182XA,W3183XA,W3189XA,W319XXA,W320XXA,W321XXA<br>W3300XA,W3301XA,W3302XA,W3303XA,W3309XA,W3310XA,W3311XA<br>W3312XA,W3313XA,W3319XA,W3400XA,W34010A,W34011A,W34018A<br>W3409XA,W3410XA,W34110A,W34111A,W34118A,W3419XA,W35XXXA<br>W361XXA,W362XXA,W363XXA,W368XXA,W369XXA,W370XXA,W378XXA<br>W38XXXA,W39XXXA,W400XXA,W401XXA,W408XXA,W409XXA,W420XXA<br>W429XXA,W450XXA,W451XXA,W452XXA,W458XXA,W460XXA,W461XXA<br>W4901XA,W4902XA,W4903XA,W4904XA,W4909XA,W499XXA,W500XXA<br>W501XXA,W502XXA,W503XXA,W504XXA,W51XXXA,W52XXXA,W5301XA<br>W5309XA,W5311XA,W5319XA,W5321XA,W5329XA,W5381XA,W5389XA<br>W540XXA,W541XXA,W548XXA,W5501XA,W5503XA,W5509XA,W5511XA<br>W5512XA,W5519XA,W5521XA,W5522XA,W5529XA,W5531XA,W5532XA<br>W5539XA,W5541XA,W5542XA,W5549XA,W5551XA,W5552XA,W5559XA<br>W5581XA,W5582XA,W5589XA,W5601XA,W5602XA,W5609XA,W5611XA<br>W5612XA,W5619XA,W5621XA,W5622XA,W5629XA,W5631XA,W5632XA<br>W5639XA,W5641XA,W5642XA,W5649XA,W5651XA,W5652XA,W5659XA<br>W5681XA,W5682XA,W5689XA,W57XXXA,W5801XA,W5802XA,W5803XA<br>W5809XA,W5811XA,W5812XA,W5813XA,W5819XA,W5901XA,W5902XA<br>W5909XA,W5911XA,W5912XA,W5913XA,W5919XA,W5921XA,W5922XA<br>W5929XA,W5981XA,W5982XA,W5983XA,W5989XA,W60XXXA,W6101XA<br>W6102XA,W6109XA,W6111XA,W6112XA,W6119XA,W6121XA,W6122XA<br>W6129XA,W6132XA,W6133XA,W6139XA,W6142XA,W6143XA,W6149XA<br>W6151XA,W6152XA,W6159XA,W6161XA,W6162XA,W6169XA,W6191XA<br>W6192XA,W6199XA,W620XXA,W621XXA,W629XXA,W64XXXA,W65XXXA<br>W67XXXA,W69XXXA,W73XXXA,W74XXXA,W85XXXA,W860XXA,W861XXA<br>W868XXA,W880XXA,W881XXA,W888XXA,W890XXA,W891XXA,W898XXA<br>W899XXA,W900XXA,W901XXA,W902XXA,W908XXA,W92XXXA,W9301XA<br>W9302XA,W9311XA,W9312XA,W932XXA,W938XXA,W940XXA,W9411XA<br>W9412XA,W9421XA,W9422XA,W9423XA,W9429XA,W9431XA,W9432XA<br>W9439XA,W99XXXA,X000XXA,X001XXA,X002XXA,X003XXA,X004XXA<br>X005XXA,X008XXA,X010XXA,X011XXA,X013XXA,X014XXA,X018XXA<br>X020XXA,X021XXA,X022XXA,X023XXA,X024XXA,X025XXA,X028XXA<br>X030XXA,X031XXA,X033XXA,X034XXA,X038XXA,X04XXXA,X05XXXA<br>X060XXA,X061XXA,X062XXA,X063XXA,X0800XA,X0801XA,X0809XA<br>X0810XA,X0811XA,X0819XA,X0820XA,X0821XA,X0829XA,X088XXA<br>X100XXA,X101XXA,X102XXA,X110XXA,X111XXA,X118XXA,X12XXXA<br>X130XXA,X131XXA,X140XXA,X141XXA,X150XXA,X151XXA,X152XXA<br>X153XXA,X158XXA,X16XXXA,X17XXXA,X18XXXA,X19XXXA,X30XXXA<br>X31XXXA,X32XXXA,X34XXXA,X35XXXA,X360XXA,X361XXA,X370XXA<br>X371XXA,X372XXA,X373XXA,X3741XA,X3742XA,X3743XA,X378XXA<br>X379XXA,X38XXXA,X3901XA,X3908XA,X398XXA,X500XXA,X501XXA<br>X503XXA,X509XXA,X52XXXA,X58XXXA | |
| External cause codes: | T360X3A,T361X3A,T362X3A,T363X3A,T364X3A,T365X3A,T366X3A | 9600,9601,9602,9603,9604,9605,9606,<br>9608,9609,9610,9614,9615 |
| intent of injury, | T367X3A,T368X3A,T3693XA,T370X3A,T371X3A,T372X3A,T373X3A | 9616,9617,9618,9619,9620,9621,9622,<br>9623,9624,9627,9628,9629 |
| assault | T374X3A,T375X3A,T378X3A,T3793XA,T380X3A,T381X3A,T382X3A | 9630,9631,9634,9635,9638,9639,9640,<br>9642,9643,9644,9645,9646 |
| | T383X3A,T384X3A,T385X3A,T386X3A,T387X3A,T38803A,T38813A | 9648,9649,96500,96501,96502,96509,9651,<br>9654,9655,96561,96569 |
| | T38893A,T38903A,T38993A,T39013A,T39093A,T391X3A,T392X3A | 9657,9659,9660,9661,9662,9663,9664,<br>9670,9678,9679,9680,9682 |
| | T39313A,T39393A,T394X3A,T398X3A,T3993XA,T400X3A,T401X3A | 9683,9684,9685,9689,96900,96901,96902,<br>96903,96904,96905,96909 |
| | T402X3A,T403X3A,T40413A,T40423A,T40493A,T404X3A,T405X3A | 9691,9692,9693,9694,9696,96970,96971,<br>96972,96973,96979,9698 |
| | T40603A,T40693A,T40713A,T40723A,T407X3A,T408X3A,T40903A | 9699,9700,9701,97081,9710,9711,9712,<br>9713,9719,9720,9721,9722 |
| | T40993A,T410X3A,T411X3A,T41203A,T41293A,T413X3A,T4143XA | 9723,9724,9725,9726,9727,9729,9730,<br>9731,9732,9733,9734,9735 |
| | T415X3A,T420X3A,T421X3A,T422X3A,T423X3A,T424X3A,T425X3A | 9736,9738,9739,9742,9744,9745,9747,<br>9750,9752,9753,9754,9755 |
| | T426X3A,T4273XA,T428X3A,T43013A,T43023A,T431X3A,T43203A | 9756,9757,9758,9760,9761,9762,9763,<br>9764,9765,9766,9767,9768 |
| | T43213A,T43223A,T43293A,T433X3A,T434X3A,T43503A,T43593A | 9769,9770,9772,9778,9779,9786,9788,<br>9789,9790,9796,9799,9800 |
| | T43603A,T43613A,T43623A,T43633A,T43643A,T43653A,T43693A | 9801,9802,9803,9808,9809,981,9820,9821,<br>9822,9823,9824,9828 |
| | T438X3A,T4393XA,T440X3A,T441X3A,T442X3A,T443X3A,T444X3A | 9830,9831,9832,9839,9849,9850,9851,<br>9852,9853,9855,9856,9858 |
| | T445X3A,T446X3A,T447X3A,T448X3A,T44903A,T44993A,T450X3A | 9859,986,9871,9872,9873,9874,9875,9876,<br>9877,9878,9879,9880 |
| | T451X3A,T452X3A,T453X3A,T454X3A,T45513A,T45523A,T45603A | 9881,9882,9888,9889,9890,9891,9893,<br>9894,9895,9896,9897,98982 |
| | T45613A,T45623A,T45693A,T457X3A,T458X3A,T4593XA,T460X3A | 98984,98989,9899,9947,99550,99551,<br>99552,99553,99554,99555 |

Continued on next page

Table S9 (continued).

| Diagnosis | ICD-10 | ICD-9 |
|---|---|---|
| | T461X3A,T462X3A,T463X3A,T464X3A,T465X3A,T466X3A,T467X3A | 99580,99581,99582,99583,99584,E9040, E9600,E961,E9620,E9621 |
| | T468X3A,T46903A,T46993A,T470X3A,T471X3A,T472X3A,T473X3A | E9622,E9629,E963,E964,E9650,E9651, E9652,E9653,E9654,E9655 |
| | T474X3A,T475X3A,T476X3A,T477X3A,T478X3A,T4793XA,T480X3A | E9656,E9657,E9658,E9659,E966,E9670, E9672,E9673,E9675,E9677 |
| | T481X3A,T48203A,T48293A,T483X3A,T484X3A,T485X3A,T486X3A | E9678,E9679,E9680,E9681,E9682,E9683, E9684,E9685,E9686,E9687 |
| | T48903A,T48993A,T490X3A,T491X3A,T492X3A,T493X3A,T494X3A | E9688,E9689,E9790,E9791,E9792,E9793, E9794,E9795,E9796,E9797 |
| | T495X3A,T496X3A,T497X3A,T498X3A,T4993XA,T500X3A,T501X3A | E9798,E9799 |
| | T502X3A,T503X3A,T504X3A,T505X3A,T506X3A,T507X3A,T508X3A | |
| | T50903A,T50913A,T50993A,T50A13A,T50A23A,T50A93A,T50B13A | |
| | T50B93A,T50Z13A,T50Z93A,T510X3A,T511X3A,T512X3A,T513X3A | |
| | T518X3A,T5193XA,T520X3A,T521X3A,T522X3A,T523X3A,T524X3A | |
| | T528X3A,T5293XA,T530X3A,T531X3A,T532X3A,T533X3A,T534X3A | |
| | T535X3A,T536X3A,T537X3A,T5393XA,T540X3A,T541X3A,T542X3A | |
| | T543X3A,T5493XA,T550X3A,T551X3A,T560X3A,T561X3A,T562X3A | |
| | T563X3A,T564X3A,T565X3A,T566X3A,T567X3A,T56813A,T56893A | |
| | T5693XA,T570X3A,T571X3A,T572X3A,T573X3A,T578X3A,T5793XA | |
| | T5803XA,T5813XA,T582X3A,T588X3A,T5893XA,T590X3A,T591X3A | |
| | T592X3A,T593X3A,T594X3A,T595X3A,T596X3A,T597X3A,T59813A | |
| | T59893A,T5993XA,T600X3A,T601X3A,T602X3A,T603X3A,T604X3A | |
| | T608X3A,T6093XA,T6103XA,T6113XA,T61773A,T61783A,T618X3A | |
| | T6193XA,T620X3A,T621X3A,T622X3A,T628X3A,T6293XA,T63003A | |
| | T63013A,T63023A,T63033A,T63043A,T63063A,T63073A,T63083A | |
| | T63093A,T63113A,T63123A,T63193A,T632X3A,T63303A,T63313A | |
| | T63323A,T63333A,T63393A,T63413A,T63423A,T63433A,T63443A | |
| | T63453A,T63463A,T63483A,T63513A,T63593A,T63613A,T63623A | |
| | T63633A,T63693A,T63713A,T63793A,T63813A,T63823A,T63833A | |
| | T63893A,T6393XA,T6403XA,T6483XA,T650X3A,T651X3A,T65213A | |
| | T65223A,T65293A,T653X3A,T654X3A,T655X3A,T656X3A,T65813A | |
| | T65823A,T65833A,T65893A,T6593XA,T71113A,T71123A,T71133A | |
| | T71143A,T71153A,T71163A,T71193A,T71223A,T71233A,T7401XA | |
| | T7402XA,T7411XA,T7412XA,T7421XA,T7422XA,T7431XA,T7432XA | |
| | T744XXA,T7451XA,T7452XA,T7461XA,T7462XA,T7491XA,T7492XA | |
| | T7601XA,T7602XA,T7611XA,T7612XA,T7621XA,T7622XA,T7631XA | |
| | T7632XA,T7651XA,T7652XA,T7661XA,T7662XA,T7691XA,T7692XA | |
| | X920XXA,X921XXA,X922XXA,X923XXA,X928XXA,X929XXA,X93XXXA | |
| | X940XXA,X941XXA,X942XXA,X948XXA,X949XXA,X9501XA,X9502XA | |
| | X9509XA,X958XXA,X959XXA,X960XXA,X961XXA,X962XXA,X963XXA | |
| | X964XXA,X968XXA,X969XXA,X97XXXA,X980XXA,X981XXA,X982XXA | |
| | X983XXA,X988XXA,X989XXA,X990XXA,X991XXA,X992XXA,X998XXA | |
| | X999XXA,Y00XXXA,Y01XXXA,Y020XXA,Y021XXA,Y028XXA,Y030XXA | |
| | Y038XXA,Y040XXA,Y041XXA,Y042XXA,Y048XXA,Y0701,Y0702,Y0703 | |
| | Y0704,Y0711,Y0712,Y0713,Y0714,Y07410,Y07411,Y07420,Y07421 | |
| | Y07430,Y07432,Y07433,Y07434,Y07435,Y07436,Y07490,Y07491 | |
| | Y07499,Y0750,Y07510,Y07511,Y07512,Y07513,Y07519,Y07521,Y07528 | |
| | Y07529,Y0753,Y0759,Y076,Y079,Y0801XA,Y0802XA,Y0809XA,Y0881XA | |
| | Y0889XA,Y09,Y380X1A,Y380X2A,Y380X3A,Y381X1A,Y381X2A,Y381X3A | |
| | Y382X1A,Y382X2A,Y382X3A,Y383X1A,Y383X2A,Y383X3A,Y384X1A | |
| | Y384X2A,Y384X3A,Y385X1A,Y385X2A,Y385X3A,Y386X1A,Y386X2A | |
| | Y386X3A,Y387X1A,Y387X2A,Y387X3A,Y3880XA,Y38811A,Y38812A | |
| | Y38891A,Y38892A,Y38893A,Y389X1A,Y389X2A | |
| External cause codes: | Y35001A,Y35002A,Y35003A,Y35009A,Y35011A,Y35012A,Y35013A | E970,E971,E972,E973,E974,E975,E976, E9900,E9901,E9902,E9903 |
| intent of injury, | Y35019A,Y35021A,Y35022A,Y35023A,Y35029A,Y35031A,Y35032A | E9909,E9910,E9911,E9912,E9913,E9914, E9915,E9916,E9917,E9918 |
| legal intervention | Y35033A,Y35039A,Y35041A,Y35042A,Y35043A,Y35049A,Y35091A | E9919,E9920,E9921,E9922,E9923,E9928, E9929,E9930,E9931,E9932 |
| war | Y35092A,Y35093A,Y35099A,Y35101A,Y35102A,Y35103A,Y35109A | E9933,E9934,E9935,E9936,E9937,E9938, E9939,E9940,E9941,E9942 |
| | Y35111A,Y35112A,Y35113A,Y35119A,Y35121A,Y35122A,Y35123A | E9943,E9948,E9949,E9950,E9951,E9952, E9953,E9954,E9958,E9959 |
| | Y35129A,Y35191A,Y35192A,Y35193A,Y35199A,Y35201A,Y35202A | E9960,E9961,E9962,E9963,E9968,E9969, E9970,E9971,E9972,E9973 |
| | Y35203A,Y35209A,Y35211A,Y35212A,Y35213A,Y35219A,Y35291A | E9978,E9979,E9980,E9981,E9988,E9989 |
| | Y35292A,Y35293A,Y35299A,Y35301A,Y35302A,Y35303A,Y35309A | |
| | Y35311A,Y35312A,Y35313A,Y35319A,Y35391A,Y35392A,Y35393A | |
| | Y35399A,Y35401A,Y35402A,Y35403A,Y35409A,Y35411A,Y35412A | |
| | Y35413A,Y35419A,Y35491A,Y35492A,Y35493A,Y35499A,Y35811A | |
| | Y35812A,Y35813A,Y35819A,Y35831A,Y35832A,Y35833A,Y35839A | |
| | Y35891A,Y35892A,Y35893A,Y35899A,Y3591XA,Y3592XA,Y3593XA | |
| | Y3599XA,Y36000A,Y36001A,Y36010A,Y36011A,Y36020A,Y36021A | |
| | Y36030A,Y36031A,Y36040A,Y36041A,Y36050A,Y36051A,Y36090A | |
| | Y36091A,Y36100A,Y36101A,Y36110A,Y36111A,Y36120A,Y36121A | |
| | Y36130A,Y36131A,Y36140A,Y36141A,Y36190A,Y36191A,Y36200A | |
| | Y36201A,Y36210A,Y36211A,Y36220A,Y36221A,Y36230A,Y36231A | |
| | Y36240A,Y36241A,Y36250A,Y36251A,Y36260A,Y36261A,Y36270A | |
| | Y36271A,Y36290A,Y36291A,Y36300A,Y36301A,Y36310A,Y36311A | |
| | Y36320A,Y36321A,Y36330A,Y36331A,Y36390A,Y36391A,Y36410A | |
| | Y36411A,Y36420A,Y36421A,Y36430A,Y36431A,Y36440A,Y36441A | |
| | Y36450A,Y36451A,Y36460A,Y36461A,Y36470A,Y36471A,Y36490A | |
| | Y36491A,Y36500A,Y36501A,Y36510A,Y36511A,Y36520A,Y36521A | |
| | Y36530A,Y36531A,Y36540A,Y36541A,Y36590A,Y36591A,Y366X0A | |

Continued on next page

Table S9 (continued).

| Diagnosis | ICD-10 | ICD-9 |
|---|---|---|
| | Y366X1A,Y367X0A,Y367X1A,Y36810A,Y36811A,Y36820A,Y36821A | |
| | Y36880A,Y36881A,Y36890A,Y36891A,Y3690XA,Y3691XA,Y3692XA | |
| | Y37000A,Y37001A,Y37010A,Y37011A,Y37020A,Y37021A,Y37030A | |
| | Y37031A,Y37040A,Y37041A,Y37050A,Y37051A,Y37090A,Y37091A | |
| | Y37100A,Y37101A,Y37110A,Y37111A,Y37120A,Y37121A,Y37130A | |
| | Y37131A,Y37140A,Y37141A,Y37190A,Y37191A,Y37200A,Y37201A | |
| | Y37210A,Y37211A,Y37220A,Y37221A,Y37230A,Y37231A,Y37240A | |
| | Y37241A,Y37250A,Y37251A,Y37260A,Y37261A,Y37270A,Y37271A | |
| | Y37290A,Y37291A,Y37300A,Y37301A,Y37310A,Y37311A,Y37320A | |
| | Y37321A,Y37330A,Y37331A,Y37390A,Y37391A,Y37410A,Y37411A | |
| | Y37420A,Y37421A,Y37430A,Y37431A,Y37440A,Y37441A,Y37450A | |
| | Y37451A,Y37460A,Y37461A,Y37470A,Y37471A,Y37490A,Y37491A | |
| | Y37500A,Y37501A,Y37510A,Y37511A,Y37520A,Y37521A,Y37530A | |
| | Y37531A,Y37540A,Y37541A,Y37590A,Y37591A,Y376X0A,Y376X1A | |
| | Y377X0A,Y377X1A,Y3790XA,Y3791XA,Y3792XA | |
| External cause codes: | R4588,T1491,T1491XA,T360X2A,T361X2A,T362X2A,T363X2A,T364X2A | 9599,9600,9601,9602,9603,9604,9605, 9606,9608,9609,9610,9614 |
| intent of injury, | T365X2A,T366X2A,T367X2A,T368X2A,T3692XA,T370X2A,T371X2A | 9615,9616,9617,9618,9619,9620,9621, 9622,9623,9624,9627,9628 |
| self-harm | T372X2A,T373X2A,T374X2A,T375X2A,T378X2A,T3792XA,T380X2A | 9629,9630,9631,9634,9635,9638,9639, 9640,9642,9643,9644,9645 |
| | T381X2A,T382X2A,T383X2A,T384X2A,T385X2A,T386X2A,T387X2A | 9646,9648,9649,96500,96501,96502,96509, 9651,9654,9655,96561 |
| | T38802A,T38812A,T38892A,T38902A,T38992A,T39012A,T39092A | 96569,9657,9659,9660,9661,9662,9663, 9664,9670,9678,9679,9680 |
| | T391X2A,T392X2A,T39312A,T39392A,T394X2A,T398X2A,T3992XA | 9682,9683,9684,9685,9689,96900,96901, 96902,96903,96904,96905 |
| | T400X2A,T401X2A,T402X2A,T403X2A,T40412A,T40422A,T40492A | 96909,9691,9692,9693,9694,9696,96970, 96971,96972,96973,96979 |
| | T404X2A,T405X2A,T40602A,T40692A,T40712A,T40722A,T407X2A | 9698,9699,9700,9701,97081,9710,9711, 9712,9713,9719,9720,9721 |
| | T408X2A,T40902A,T40992A,T410X2A,T411X2A,T41202A,T41292A | 9722,9723,9724,9725,9726,9727,9729, 9730,9731,9732,9733,9734 |
| | T413X2A,T4142XA,T415X2A,T420X2A,T421X2A,T422X2A,T423X2A | 9735,9736,9738,9739,9742,9744,9745, 9747,9750,9752,9753,9754 |
| | T424X2A,T425X2A,T426X2A,T4272XA,T428X2A,T43012A,T43022A | 9755,9756,9757,9758,9760,9761,9762, 9763,9764,9765,9766,9767 |
| | T431X2A,T43202A,T43212A,T43222A,T43292A,T433X2A,T434X2A | 9768,9769,9770,9772,9778,9779,9786, 9788,9789,9790,9796,9799 |
| | T43502A,T43592A,T43602A,T43612A,T43622A,T43632A,T43642A | 9800,9801,9802,9803,9808,9809,981,9820, 9821,9822,9823,9824 |
| | T43652A,T43692A,T438X2A,T4392XA,T440X2A,T441X2A,T442X2A | 9828,9830,9831,9832,9839,9849,9850, 9851,9852,9853,9855,9856 |
| | T443X2A,T444X2A,T445X2A,T446X2A,T447X2A,T448X2A,T44902A | 9858,9859,986,9871,9872,9873,9874,9875, 9876,9877,9878,9879 |
| | T44992A,T450X2A,T451X2A,T452X2A,T453X2A,T454X2A,T45512A | 9880,9881,9882,9888,9889,9890,9891, 9893,9894,9895,9896,9897 |
| | T45522A,T45602A,T45612A,T45622A,T45692A,T457X2A,T458X2A | 98982,98984,98989,9899,9947,E9500, E9501,E9502,E9503,E9504 |
| | T4592XA,T460X2A,T461X2A,T462X2A,T463X2A,T464X2A,T465X2A | E9506,E9507,E9508,E9509,E9520,E9521, E9528,E9529,E9530,E9531 |
| | T466X2A,T467X2A,T468X2A,T46902A,T46992A,T470X2A,T471X2A | E9538,E954,E9550,E9551,E9552,E9553, E9554,E9555,E9556,E9557 |
| | T472X2A,T473X2A,T474X2A,T475X2A,T476X2A,T477X2A,T478X2A | E9559,E956,E9579,E9580,E9581,E9582, E9583,E9584,E9585,E9586 |
| | T4792XA,T480X2A,T481X2A,T48202A,T48292A,T483X2A,T484X2A | E9587,E9588,E9589 |
| | T485X2A,T486X2A,T48902A,T48992A,T490X2A,T491X2A,T492X2A | |
| | T493X2A,T494X2A,T495X2A,T496X2A,T497X2A,T498X2A,T4992XA | |
| | T500X2A,T501X2A,T502X2A,T503X2A,T504X2A,T505X2A,T506X2A | |
| | T507X2A,T508X2A,T50902A,T50912A,T50992A,T50A12A,T50A22A | |
| | T50A92A,T50B12A,T50B92A,T50Z12A,T50Z92A,T510X2A,T511X2A | |
| | T512X2A,T513X2A,T518X2A,T5192XA,T520X2A,T521X2A,T522X2A | |
| | T523X2A,T524X2A,T528X2A,T5292XA,T530X2A,T531X2A,T532X2A | |
| | T533X2A,T534X2A,T535X2A,T536X2A,T537X2A,T5392XA,T540X2A | |
| | T541X2A,T542X2A,T543X2A,T5492XA,T550X2A,T551X2A,T560X2A | |
| | T561X2A,T562X2A,T563X2A,T564X2A,T565X2A,T566X2A,T567X2A | |
| | T56812A,T56892A,T5692XA,T570X2A,T571X2A,T572X2A,T573X2A | |
| | T578X2A,T5792XA,T5802XA,T5812XA,T582X2A,T588X2A,T5892XA | |
| | T590X2A,T591X2A,T592X2A,T593X2A,T594X2A,T595X2A,T596X2A | |
| | T597X2A,T59812A,T59892A,T5992XA,T600X2A,T601X2A,T602X2A | |
| | T603X2A,T604X2A,T608X2A,T6092XA,T6102XA,T6112XA,T61772A | |
| | T61782A,T618X2A,T6192XA,T620X2A,T621X2A,T622X2A,T628X2A | |
| | T6292XA,T63002A,T63012A,T63022A,T63032A,T63042A,T63062A | |
| | T63072A,T63082A,T63092A,T63112A,T63122A,T63192A,T632X2A | |
| | T63302A,T63312A,T63322A,T63332A,T63392A,T63412A,T63422A | |
| | T63432A,T63442A,T63452A,T63462A,T63482A,T63512A,T63592A | |
| | T63612A,T63622A,T63632A,T63692A,T63712A,T63792A,T63812A | |
| | T63822A,T63832A,T63892A,T6392XA,T6402XA,T6482XA,T650X2A | |
| | T651X2A,T65212A,T65222A,T65292A,T653X2A,T654X2A,T655X2A | |
| | T656X2A,T65812A,T65822A,T65832A,T65892A,T6592XA,T71112A | |
| | T71122A,T71132A,T71152A,T71162A,T71192A,T71222A,T71232A | |
| | X710XXA,X711XXA,X712XXA,X713XXA,X718XXA,X719XXA,X72XXXA | |
| | X730XXA,X731XXA,X732XXA,X738XXA,X739XXA,X7401XA,X7402XA | |
| | X7409XA,X748XXA,X749XXA,X75XXXA,X76XXXA,X770XXA,X771XXA | |
| | X772XXA,X773XXA,X778XXA,X779XXA,X780XXA,X781XXA,X782XXA | |
| | X788XXA,X789XXA,X79XXXA,X80XXXA,X810XXA,X811XXA,X818XXA | |

Continued on next page

**Table S9 (continued).**

| Diagnosis | ICD-10 | ICD-9 |
|---|---|---|
| | X820XXA,X821XXA,X822XXA,X828XXA,X830XXA,X831XXA,X832XXA<br>X838XXA | |
| External cause codes: | T360X4A,T361X4A,T362X4A,T363X4A,T364X4A,T365X4A,T366X4A | 9600,9601,9602,9603,9604,9605,9606,<br>9608,9609,9610,9614,9615 |
| intent of injury, | T367X4A,T368X4A,T3694XA,T370X4A,T371X4A,T372X4A,T373X4A | 9616,9617,9618,9619,9620,9621,9622,<br>9623,9624,9627,9628,9629 |
| undetermined | T374X4A,T375X4A,T378X4A,T3794XA,T380X4A,T381X4A,T382X4A | 9630,9631,9634,9635,9638,9639,9640,<br>9642,9644,9645,9646,9648 |
| | T383X4A,T384X4A,T385X4A,T386X4A,T387X4A,T38804A,T38814A | 9649,96500,96501,96502,96509,9651,9654,<br>9655,96561,96569,9657 |
| | T38894A,T38904A,T38994A,T39014A,T39094A,T391X4A,T392X4A | 9659,9660,9661,9662,9663,9664,9670,<br>9678,9679,9680,9682,9683 |
| | T39314A,T39394A,T394X4A,T398X4A,T3994XA,T400X4A,T401X4A | 9684,9685,9689,96900,96901,96902,96903,<br>96904,96905,96909,9691 |
| | T402X4A,T403X4A,T40414A,T40424A,T40494A,T404X4A,T405X4A | 9692,9693,9694,9696,96970,96971,96972,<br>96973,96979,9698,9699 |
| | T40604A,T40694A,T40714A,T40724A,T407X4A,T408X4A,T40904A | 9700,9701,97081,9710,9711,9712,9713,<br>9719,9720,9721,9722,9723 |
| | T40994A,T410X4A,T411X4A,T41204A,T41294A,T413X4A,T4144XA | 9724,9725,9726,9727,9729,9730,9731,<br>9732,9733,9734,9735,9736 |
| | T415X4A,T420X4A,T421X4A,T422X4A,T423X4A,T424X4A,T425X4A | 9738,9739,9742,9744,9745,9747,9750,<br>9752,9753,9754,9755,9756 |
| | T426X4A,T4274XA,T428X4A,T43014A,T43024A,T431X4A,T43204A | 9757,9758,9760,9761,9762,9763,9764,<br>9765,9766,9767,9768,9769 |
| | T43214A,T43224A,T43294A,T433X4A,T434X4A,T43504A,T43594A | 9770,9772,9778,9779,9786,9788,9789,<br>9790,9796,9799,9800,9801 |
| | T43604A,T43614A,T43624A,T43634A,T43644A,T43654A,T43694A | 9802,9803,9808,9809,981,9820,9821,9822,<br>9823,9824,9828,9830 |
| | T438X4A,T4394XA,T440X4A,T441X4A,T442X4A,T443X4A,T444X4A | 9831,9832,9839,9849,9850,9851,9852,<br>9853,9855,9856,9858,9859 |
| | T445X4A,T446X4A,T447X4A,T448X4A,T44904A,T44994A,T450X4A | 986,9871,9872,9873,9874,9875,9876,9877,<br>9878,9879,9880,9881 |
| | T451X4A,T452X4A,T453X4A,T454X4A,T45514A,T45524A,T45604A | 9882,9888,9889,9890,9891,9893,9894,<br>9895,9896,9897,98982,98984 |
| | T45614A,T45624A,T45694A,T457X4A,T458X4A,T4594XA,T460X4A | 98989,9899,9941,9947,E8585,E8698,<br>E9104,E9108,E9241,E9286 |
| | T461X4A,T462X4A,T463X4A,T464X4A,T465X4A,T466X4A,T467X4A | E9502,E9504,E9800,E9801,E9802,E9803,<br>E9804,E9805,E9806,E9807 |
| | T468X4A,T46904A,T46994A,T470X4A,T471X4A,T472X4A,T473X4A | E9808,E9809,E9820,E9821,E9828,E9830,<br>E9831,E9838,E984,E9850 |
| | T474X4A,T475X4A,T476X4A,T477X4A,T478X4A,T4794XA,T480X4A | E9851,E9852,E9853,E9854,E9855,E9856,<br>E9857,E986,E9879,E9880 |
| | T481X4A,T48204A,T48294A,T483X4A,T484X4A,T485X4A,T486X4A<br>T48904A,T48994A,T490X4A,T491X4A,T492X4A,T493X4A,T494X4A<br>T495X4A,T496X4A,T497X4A,T498X4A,T4994XA,T500X4A,T501X4A<br>T502X4A,T503X4A,T504X4A,T505X4A,T506X4A,T507X4A,T508X4A<br>T50904A,T50914A,T50994A,T50A14A,T50A24A,T50A94A,T50B14A<br>T50B94A,T50Z14A,T50Z94A,T510X4A,T511X4A,T512X4A,T513X4A<br>T518X4A,T5194XA,T520X4A,T521X4A,T522X4A,T523X4A,T524X4A<br>T528X4A,T5294XA,T530X4A,T531X4A,T532X4A,T533X4A,T534X4A<br>T535X4A,T536X4A,T537X4A,T5394XA,T540X4A,T541X4A,T542X4A<br>T543X4A,T5494XA,T550X4A,T551X4A,T560X4A,T561X4A,T562X4A<br>T563X4A,T564X4A,T565X4A,T566X4A,T567X4A,T56814A,T56894A<br>T5694XA,T570X4A,T571X4A,T572X4A,T573X4A,T578X4A,T5794XA<br>T5804XA,T5814XA,T582X4A,T588X4A,T5894XA,T590X4A,T591X4A<br>T592X4A,T593X4A,T594X4A,T595X4A,T596X4A,T597X4A,T59814A<br>T59894A,T5994XA,T600X4A,T601X4A,T602X4A,T603X4A,T604X4A<br>T608X4A,T6094XA,T6104XA,T6114XA,T61774A,T61784A,T618X4A<br>T6194XA,T620X4A,T621X4A,T622X4A,T628X4A,T6294XA,T63004A<br>T63014A,T63024A,T63034A,T63044A,T63064A,T63074A,T63084A<br>T63094A,T63114A,T63124A,T63194A,T632X4A,T63304A,T63314A<br>T63324A,T63334A,T63394A,T63414A,T63424A,T63434A,T63444A<br>T63454A,T63464A,T63484A,T63514A,T63594A,T63614A,T63624A<br>T63634A,T63694A,T63714A,T63794A,T63814A,T63824A,T63834A<br>T63894A,T6394XA,T6404XA,T6484XA,T650X4A,T651X4A,T65214A<br>T65224A,T65294A,T653X4A,T654X4A,T655X4A,T656X4A,T65814A<br>T65824A,T65834A,T65894A,T6594XA,T71114A,T71124A,T71134A<br>T71144A,T71154A,T71164A,T71194A,T71224A,T71234A,T751XXA<br>Y210XXA,Y211XXA,Y212XXA,Y213XXA,Y214XXA,Y218XXA,Y219XXA<br>Y22XXXA,Y230XXA,Y231XXA,Y232XXA,Y233XXA,Y238XXA,Y239XXA<br>Y240XXA,Y248XXA,Y249XXA,Y25XXXA,Y26XXXA,Y270XXA,Y271XXA<br>Y272XXA,Y273XXA,Y278XXA,Y279XXA,Y280XXA,Y281XXA,Y282XXA<br>Y288XXA,Y289XXA,Y29XXXA,Y30XXXA,Y31XXXA,Y32XXXA,Y33XXXA | E9881,E9882,E9885,E9888 |
| Hallucinogen-related disorders; subsequent encounter | T408X1D,T408X2D,T408X3D,T408X4D,T40901D,T40902D,T40903D<br>T40904D,T40905D,T40991D,T40992D,T40993D,T40994D,T40995D<br>T43641D,T43642D,T43643D,T43644D | V5889 |
| Inhalant-related disorders; subsequent encounter | T410X1D,T410X2D,T410X3D,T410X4D,T410X5D | V5889 |
| Nontraumatic dental | A690,A691,B002,B084,B370,B3783,K000,K001,K002,K003,K004,K005 | 0542,0743,101,1120,5200,5201,5202,5203,<br>5204,5205,5206,5207 |
| conditions | K006,K007,K008,K009,K010,K011,K023,K0251,K0252,K0253,K0261 | 5208,5209,52100,52101,52102,52103,<br>52104,52105,52106,52107 |

Continued on next page

Table S9 (continued).

| Diagnosis | ICD-10 | ICD-9 |
|---|---|---|
| | K0262,K0263,K027,K029,K030,K031,K032,K033,K034,K035,K036,K037 | 52108,52110,52120,52130,52140,52141,52142,5215,5216,5217 |
| | K0381,K0389,K039,K040,K0401,K0402,K041,K042,K043,K044,K045 | 52181,52189,5219,5220,5221,5222,5223,5224,5225,5226,5227,5228 |
| | K046,K047,K048,K0490,K0499,K0500,K0501,K0510,K0511,K0520 | 5229,52300,52301,52310,52311,52320,52330,52331,52332,52333 |
| | K0521,K05211,K05212,K05213,K05219,K0522,K05221,K05222,K05223 | 52340,52341,52342,5235,5236,5238,5239,52400,52401,52402,52403 |
| | K05229,K0530,K0531,K05311,K05312,K05313,K05319,K0532,K05321 | 52404,52405,52406,52407,52409,52410,52411,52412,52419,52420 |
| | K05322,K05323,K05329,K054,K055,K056,K060,K06010,K06011,K06012 | 52421,52422,52423,52424,52425,52426,52427,52428,52429,52430 |
| | K06013,K06020,K06021,K06022,K06023,K061,K063,K068,K069,K080 | 52431,52432,52433,52434,52435,52436,52437,52439,5244,52450 |
| | K08101,K08102,K08103,K08104,K08109,K08121,K08122,K08123 | 52451,52452,52453,52454,52455,52456,52457,52459,52460,52461 |
| | K08124,K08129,K08131,K08132,K08133,K08134,K08139,K08191 | 52462,52463,52469,52470,52471,52472,52473,52474,52479,52481 |
| | K08192,K08193,K08194,K08199,K0820,K0821,K0822,K0823,K0824 | 52482,52489,5249,5250,52510,52512,52513,52519,52520,52521 |
| | K0825,K0826,K083,K08401,K08402,K08403,K08404,K08409,K08421 | 52522,52523,52524,52525,52526,5253,52540,52541,52542,52543 |
| | K08422,K08423,K08424,K08429,K08431,K08432,K08433,K08434 | 52544,52550,52551,52552,52553,52554,52560,52561,52562,52563 |
| | K08439,K08491,K08492,K08493,K08494,K08499,K0850,K0851,K0852 | 52564,52565,52566,52567,52569,52571,52572,52573,52579,5258 |
| | K08530,K08531,K08539,K0854,K0855,K0856,K0859,K088,K0889,K089 | 5259,5260,5261,5262,5263,5264,5265,52661,52662,52663,52669 |
| | K090,K091,K098,K099,K110,K111,K1120,K1121,K1122,K1123,K113 | 52681,52689,5269,5270,5271,5272,5273,5274,5275,5276,5277,5278 |
| | K114,K115,K116,K117,K118,K119,K120,K121,K122,K1230,K1231 | 5279,52800,52801,52802,52809,5281,5282,5283,5284,5285,5286 |
| | K1232,K1233,K1239,K130,K131,K1321,K1322,K1323,K1324,K1329 | 52871,52872,52879,5288,5289,5290,5291,5292,5293,5294,5295 |
| | K133,K134,K135,K136,K1370,K1379,K140,K141,K142,K143,K144,K145 | 5296,5298,5299,6820,78492,V523,V534,V585,V722 |
| | K146,K148,K149,M2600,M2601,M2602,M2603,M2604,M2605,M2606 M2607,M2609,M2610,M2611,M2612,M2619,M2620,M26211,M26212 M26213,M26219,M26220,M26221,M2623,M2624,M2625,M2629,M2630 M2631,M2632,M2633,M2634,M2635,M2636,M2637,M2639,M264,M2650 M2651,M2652,M2653,M2654,M2655,M2656,M2657,M2659,M2660,M26601 M26602,M26603,M26609,M2661,M26611,M26612,M26613,M26619,M2662 M26621,M26622,M26623,M26629,M2663,M26631,M26632,M26633,M26639 M26641,M26642,M26643,M26649,M26651,M26652,M26653,M26659,M2669 M2670,M2671,M2672,M2673,M2674,M2679,M2681,M2682,M2689,M269 M270,M271,M272,M273,M2740,M2749,M2751,M2752,M2753,M2759,M2761 M2762,M2763,M2769,M278,M279,M350C,M7911,R682,R6884,Z0120 Z0121,Z463,Z464 | |
| Opioid-related disorders; subsequent encounter | T400X1D,T400X2D,T400X3D,T400X4D,T400X5D,T401X1D,T401X2D T401X3D,T401X4D,T402X1D,T402X2D,T402X3D,T402X4D,T402X5D T403X1D,T403X2D,T403X3D,T403X4D,T403X5D,T40411D,T40412D T40413D,T40414D,T40415D,T40421D,T40422D,T40423D,T40424D T40425D,T40491D,T40492D,T40493D,T40494D,T40495D,T404X1D T404X2D,T404X3D,T404X4D,T404X5D,T40601D,T40602D,T40603D T40604D,T40605D,T40691D,T40692D,T40693D,T40694D,T40695D | V5889 |
| Sedative-related disorders; subsequent encounter | T426X1D,T426X2D,T426X3D,T426X4D,T426X5D,T4271XD,T4272XD T4273XD,T4274XD,T4275XD | V5889 |
| Stimulant-related disorders; subsequent encounter | T405X1D,T405X2D,T405X3D,T405X4D,T405X5D,T43601D,T43602D T43603D,T43604D,T43605D,T43611D,T43612D,T43613D,T43614D T43615D,T43621D,T43622D,T43623D,T43624D,T43625D,T43631D T43632D,T43633D,T43634D,T43635D,T43641D,T43642D,T43643D T43644D,T43651D,T43652D,T43653D,T43654D,T43655D,T43691D T43692D,T43693D,T43694D,T43695D | V5889 |
| Caries, | B002,B084,B370,B3783,K003,K007,K023,K0251,K0252,K0253,K0261 | 0542,0743,1120,5203,5207,52100,52101,52102,52103,52104,52105 |
| periodontitis, and | K0262,K0263,K027,K029,K030,K031,K032,K036,K037,K0381,K0389 | 52106,52107,52108,52110,52120,52130,5217,52181,52189,5219 |
| other preventable | K039,K040,K0401,K042,K044,K045,K0500,K0501,K0510,K0511,K0520 | 5220,5222,5224,5226,52300,52301,52310,52311,52320,52330,52331 |
| dental conditions | K0521,K05211,K05212,K05213,K05219,K0522,K05221,K05222,K05223 | 52332,52333,52340,52341,52342,5235,5236,5238,5239,52510,52512 |
| | K05229,K0530,K0531,K05311,K05312,K05313,K05319,K0532,K05321 | 52513,52519,52540,52541,52542,52543,52544,52550,52551,52552 |
| | K05322,K05323,K05329,K054,K055,K056,K060,K06010,K06011,K06012 | 52553,52554,52560,52561,52562,52563,52564,52565,52566,52567 |
| | K06013,K06020,K06021,K06022,K06023,K061,K08101,K08102,K08103 | 52569,52571,52572,52573,52579,5258,5259,52661,52662,52663 |
| | K08104,K08109,K08121,K08122,K08123,K08124,K08129,K08131 K08132,K08133,K08134,K08139,K08191,K08192,K08193,K08194 K08199,K08401,K08402,K08403,K08404,K08409,K08421,K08422 K08423,K08424,K08429,K08431,K08432,K08433,K08434,K08439 K08491,K08492,K08493,K08494,K08499,K0850,K0851,K0852,K08530 | 52669,78492,V523,V534,V585,V722 |

Continued on next page

Table S9 (continued).

| Diagnosis | ICD-10 | ICD-9 |
|---|---|---|
| | K08531,K08539,K0854,K0855,K0856,K0859,K088,K0889,K089,M2751 M2752,M2753,M2759,M2761,M2762,M2763,M2769,M350C,R6884,Z0120 Z0121,Z463,Z464 | |
| Chest pain | R070,R071,R0781,R0782,R0789,R079 | 7841,78650,78652,78659 |
| Neuropathic pain | M792 | 7292 |
| Pain | R52 | 33819,78096 |
| Pleuritic chest pain | R071 | 78652 |
| Unusual muscle pain | M791,M7910,M7911,M7912,M7918 | 7291 |
| Back pain | M5450,M5451,M549 | 7245 |
| Fatigue | G933,R5382,R5383 | 78071,78079 |
| Post exertional fatigue | T733XXA,T733XXD,T733XXS | 9094,9945,E9272,V5889 |
| Anosmia (Loss of Smell) | R430 | 7811 |
| Ageusia (Loss of Taste) | R432,R439 | 7811,V415 |
| Cough | R05,R051 ,R052,R053,R054,R058,R059 | 7862 |
| Dysguesia | R438 | 7811 |
| Ear pain | H9201,H9202,H9203,H9209 | 38870 |
| Hoarse voice | R490 | 78442 |
| Hyperacusis | H9323 | |
| Nasal congestion | R0981 | 47819 |
| Phlegm | R848 | 7929 |
| Sneezing | R067 | 78499 |
| Sore throat | J029 | 462 |
| Tinnitus | H931 | |
| Runny Nose | J3489 | 47819 |
| Lump in throat (Globus) | R0989 | 7859,7867 |
| Diseases of middle | H6500,H6501,H6502,H6503,H6504,H6505,H6506,H6507,H65111,H65112 | 38100,38101,38102,38103,38104,38105, 38106,38110,38119,38120 |
| ear and mastoid | H65113,H65114,H65115,H65116,H65117,H65119,H65191,H65192 | 38129,3813,3814,38150,38151,38152, 38160,38161,38162,38163 |
| (except otitis media) | H65193,H65194,H65195,H65196,H65197,H65199,H6520,H6521,H6522 | 3817,38300,38301,38302,3831,38320, 38321,38322,38381,38389 |
| | H6523,H6530,H6531,H6532,H6533,H65411,H65412,H65413,H65419 | 3839,38400,38401,38409,38420,38421, 38422,38423,38424,38425 |
| | H65491,H65492,H65493,H65499,H6590,H6591,H6592,H6593,H68001 | 38500,38501,38502,38503,38509,38530, 38531,38532,38533,38535 |
| | H68002,H68003,H68009,H68011,H68012,H68013,H68019,H68021 H68022,H68023,H68029,H68101,H68102,H68103,H68109,H68111 H68112,H68113,H68119,H68121,H68122,H68123,H68129,H68131 H68132,H68133,H68139,H6900,H6901,H6902,H6903,H70001,H70002 H70003,H70009,H70011,H70012,H70013,H70019,H70091,H70092 H70093,H70099,H7010,H7011,H7012,H7013,H70201,H70202,H70203 H70209,H70211,H70212,H70213,H70219,H70221,H70222,H70223 H70229,H70811,H70812,H70813,H70819,H70891,H70892,H70893 H70899,H7090,H7091,H7092,H7093,H7100,H7101,H7102,H7103,H7110 H7111,H7112,H7113,H7120,H7121,H7122,H7123,H7130,H7131,H7132 H7133,H7190,H7191,H7192,H7193,H7200,H7201,H7202,H7203,H7210 H7211,H7212,H7213,H722X1,H722X2,H722X3,H722X9,H72811,H72812 H72813,H72819,H72821,H72822,H72823,H72829,H7290,H7291,H7292 H7293,H73001,H73002,H73003,H73009,H73011,H73012,H73013,H73019 H73091,H73092,H73093,H73099,H7401,H7402,H7403,H7409 | |
| Stomache pain | K219,K259,K299,K30,K319,K5090,K5190,K589,R100,R1010,R1011 | 53081,53190,5368,5369,5379,5559,5569, 5641,6089,6259,78900 |
| | R1012,R1013,R102,R1030,R1031,R1032,R1033,R10811,R10812,R10813 | 78901,78902,78903,78904,78905,78906, 78907,78909,78960,78961 |
| | R10814,R10815,R10816,R10817,R10819,R10821,R10822,R10823 | 78962,78963,78964,78965,78966,78967, 78969,7897 |
| | R10824,R10825,R10826,R10827,R10829,R1083,R1084,R109 | |

Continued on next page

**Table S9 (continued).**

| Diagnosis | ICD-10 | ICD-9 |
|---|---|---|
| Bloating | R140 | 7873 |
| Bowel incontinence | R150,R151,R152,R159 | 78760,78761,78762,78763 |
| Constipation | K5900,K5901,K5902,K5903,K5904,K5909,K591,K592,K593,K5931<br>K5939,K594,K598,K5981 ,K5989,K599 | 56400,56401,56402,56409,5645,5646,5647,<br>56481,56489,5649 |
| Diarrhoea | R197 | 78791 |
| Gastric reflux | K210,K2100,K2101,K219 | 53011,53081 |
| Vomiting | R1110,R1111,R1112,R1113,R1114,R1115 | 07882,5362,56987,78703,78704 |
| Weight gain | R635 | 7831 |
| Pancreatic disorders (excluding diabetes) | K860,K861,K862,K863,K868,K8681,K8689,K869 | 5771,5772,5778,5779 |
| Weight loss | R634 | 78321 |
| Anhedonia | R4584 | 78099 |
| Anorexia | R630 | 7830 |
| Depression | F320,F321,F322,F323,F324,F325,F328,F3281,F3289,F329,F32A,F330<br>F331,F332,F333,F3340,F3341,F3342,F338,F339 | 29620,29621,29622,29623,29624,29625,<br>29626,29630,29631,29632<br>29633,29634,29635,29636,29682,29699,<br>2980,311 |
| Increased appetite | R632 | 7836 |
| Loneliness | Z602,Z604 | V603,V624 |
| Mood swings bipolar | F3010,F3011,F3012,F3013,F302,F303,F304,F308,F309,F310,F3110<br>F3111,F3112,F3113,F312,F3130,F3131,F3132,F314,F315,F3160<br>F3161,F3162,F3163,F3164,F3170,F3171,F3172,F3173,F3174,F3175<br>F3176,F3177,F3178,F3181,F3189,F319,F340,F341,F348,F3481,F3489<br>F349,F39 | 29600,29601,29602,29603,29604,29605,<br>29606,29640,29641,29642<br>29643,29644,29645,29646,29650,29651,<br>29652,29653,29654,29655<br>29656,29660,29661,29662,29663,29664,<br>29665,29666,2967,29680<br>29681,29689,29690,29699,3004,30110,<br>30112,30113 |
| Post-traumatic stress disorder | F4310,F4311,F4312 | 30981 |
| Skipped Meals | Z594,Z724 | V602,V691 |
| Amnesia memory problems | R411,R412,R413 | 78093 |
| Brain fog cognitive deficit | F0100EZ,F0100FZ,F0100UZ,F0100YZ,F0100ZZ,F0101ZZ,F0103ZZ<br>F0104ZZ,F0105YZ,F0105ZZ,F0106YZ,F0106ZZ,F010GZZ,F0110EZ<br>F0110FZ,F0110UZ,F0110YZ,F0110ZZ,F0111ZZ,F0113ZZ,F0114ZZ<br>F0115YZ,F0115ZZ,F0116YZ,F0116ZZ,F011GZZ,F0120EZ,F0120FZ<br>F0120UZ,F0120YZ,F0120ZZ,F0121ZZ,F0123ZZ,F0124ZZ,F0125YZ<br>F0125ZZ,F0126YZ,F0126ZZ,F012GZZ,F0130EZ,F0130FZ,F0130UZ<br>F0130YZ,F0130ZZ,F0131ZZ,F0133ZZ,F0134ZZ,F0135YZ,F0135ZZ<br>F0136YZ,F0136ZZ,F013GZZ,F0150,F0151,F01511,F01518,F0152,F0153<br>F0154,F01A0,F01A11,F01A18,F01A2,F01A3,F01A4,F01B0,F01B11<br>F01B18,F01B2,F01B3,F01B4,F01C0,F01C11,F01C18,F01C2,F01C3<br>F01C4,F01D0EZ,F01D0FZ,F01D0UZ,F01D0YZ,F01D0ZZ,F01D1ZZ,F01D5YZ<br>F01D5ZZ,F01D6YZ,F01D6ZZ,F01F0EZ,F01F0FZ,F01F0UZ,F01F0YZ<br>F01F0ZZ,F01F1ZZ,F01F5YZ,F01F5ZZ,F01F6YZ,F01F6ZZ,F01G0EZ<br>F01G0FZ,F01G0UZ,F01G0YZ,F01G0ZZ,F01G1ZZ,F01G5YZ,F01G5ZZ<br>F01G6YZ,F01G6ZZ,F01H0EZ,F01H0FZ,F01H0UZ,F01H0YZ,F01H0ZZ<br>F01H1ZZ,F01H5YZ,F01H5ZZ,F01H6YZ,F01H6ZZ,F01J0EZ,F01J0FZ<br>F01J0UZ,F01J0YZ,F01J0ZZ,F01J1ZZ,F01J5YZ,F01J5ZZ,F01J6YZ<br>F01J6ZZ,F01K0EZ,F01K0FZ,F01K0UZ,F01K0YZ,F01K0ZZ,F01K1ZZ<br>F01K5YZ,F01K5ZZ,F01K6YZ,F01K6ZZ,F01L0EZ,F01L0FZ,F01L0UZ<br>F01L0YZ,F01L0ZZ,F01L1ZZ,F01L5YZ,F01L5ZZ,F01L6YZ,F01L6ZZ<br>F01M0EZ,F01M0FZ,F01M0UZ,F01M0YZ,F01M0ZZ,F01M1ZZ,F01M5YZ<br>F01M5ZZ,F01M6YZ,F01M6ZZ,F01N0EZ,F01N0FZ,F01N0UZ,F01N0YZ<br>F01N0ZZ,F01Z2KZ,F01Z2MZ,F01Z2NZ,F01Z2PZ,F01Z2QZ,F01Z2SZ<br>F01Z2YZ,F01Z2ZZ,F01Z77Z,F01Z9JZ,F01ZBEZ,F01ZBFZ,F01ZBUZ<br>F01ZBZZ,F01ZCEZ,F01ZCFZ,F01ZCUZ,F01ZCZZ,F01ZDEZ,F01ZDFZ<br>F01ZDUZ,F01ZDYZ,F01ZDZZ,F01ZFEZ,F01ZFFZ,F01ZFUZ,F01ZFZZ<br>F0209YZ,F0209ZZ,F020DYZ,F020DZZ,F021DYZ,F021DZZ,F022DYZ<br>F022DZZ,F023DYZ,F023DZZ,F024GCZ,F024GGZ,F024GYZ,F024GZZ<br>F025GCZ,F025GGZ,F025GYZ,F025GZZ,F026GCZ,F026GGZ,F026GYZ<br>F026GZZ,F0277EZ,F0277GZ,F0277UZ,F0277YZ,F0277ZZ,F027GCZ | 2900,29010,29011,29013,29021,29040,<br>29041,2908,2909,2930,2931<br>29410,29411,29420,29421,2948,3079,<br>31501,3310,33111,33119<br>33182,33183,34830,36816,78001,78002,<br>78003,78009,78460,78461<br>78469,8915,9301,9302,9304,9305,9306,<br>9307,9309,NoDx |

Continued on next page

**Table S9 (continued).**

| Diagnosis | ICD-10 | ICD-9 |
|---|---|---|
| | F027GGZ,F027GYZ,F027GZZ,F0280,F0281,F02811,F02818,F0282,F0283<br>F0284,F028GCZ,F028GGZ,F028GYZ,F028GZZ,F029GCZ,F029GGZ,F029GYZ<br>F029GZZ,F02A0,F02A11,F02A18,F02A2,F02A3,F02A4,F02B0,F02B11<br>F02B18,F02B2,F02B3,F02B4,F02BGCZ,F02BGGZ,F02BGYZ,F02BGZZ<br>F02C0,F02C11,F02C18,F02C2,F02C3,F02C4,F02C7EZ,F02C7GZ,F02C7UZ<br>F02C7YZ,F02C7ZZ,F02CGCZ,F02CGGZ,F02CGYZ,F02CGZZ,F02Z0EZ<br>F02Z0FZ,F02Z0UZ,F02Z0ZZ,F02Z1EZ,F02Z1FZ,F02Z1UZ,F02Z1ZZ<br>F02Z2YZ,F02Z2ZZ,F02Z3EZ,F02Z3FZ,F02Z3UZ,F02Z3ZZ,F02Z4EZ<br>F02Z4FZ,F02Z4UZ,F02Z4ZZ,F02Z5KZ,F02Z5MZ,F02Z5NZ,F02Z5PZ<br>F02Z5QZ,F02Z5SZ,F02Z5YZ,F02Z5ZZ,F02Z6ZZ,F02Z8YZ,F02Z8ZZ<br>F02ZBEZ,F02ZBFZ,F02ZBUZ,F02ZBYZ,F02ZBZZ,F02ZCEZ,F02ZCFZ<br>F02ZCUZ,F02ZCYZ,F02ZCZZ,F02ZFYZ,F02ZFZZ,F02ZHEZ,F02ZHFZ<br>F02ZHGZ,F02ZHUZ,F02ZHYZ,F02ZHZZ,F0390,F0391,F03911,F03918<br>F0392,F0393,F0394,F03A0,F03A11,F03A18,F03A2,F03A3,F03A4,F03B0<br>F03B11,F03B18,F03B2,F03B3,F03B4,F03C0,F03C11,F03C18,F03C2<br>F03C3,F03C4,F05,F068,G300,G301,G308,G309,G3101,G3109,G3183<br>G3184,G9340,R400,R401,R4020,R402110,R402111,R402112,R402113<br>R402114,R402120,R402121,R402122,R402123,R402124,R402130<br>R402131,R402132,R402133,R402134,R402140,R402141,R402142<br>R402143,R402144,R402210,R402211,R402212,R402213,R402214<br>R402220,R402221,R402222,R402223,R402224,R402230,R402231<br>R402232,R402233,R402234,R402240,R402241,R402242,R402243<br>R402244,R402250,R402251,R402252,R402253,R402254,R402310<br>R402311,R402312,R402313,R402314,R402320,R402321,R402322<br>R402323,R402324,R402330,R402331,R402332,R402333,R402334<br>R402340,R402341,R402342,R402343,R402344,R402350,R402351<br>R402352,R402353,R402354,R402360,R402361,R402362,R402363<br>R402364,R40241,R402410,R402411,R402412,R402413,R402414,R40242<br>R402420,R402421,R402422,R402423,R402424,R40243,R402430<br>R402431,R402432,R402433,R402434,R40244,R402440,R402441<br>R402442,R402443,R402444,R402A,R403,R404,R451,R480,R481,R482<br>R483,R488,R489 | |
| Dementia | F003GKZ,F003GMZ,F003GPZ,F003GYZ,F003GZZ,F00Z01Z,F00Z02Z<br>F00Z0KZ,F00Z0ZZ,F00Z11Z,F00Z12Z,F00Z19Z,F00Z1KZ,F00Z1ZZ<br>F00Z21Z,F00Z22Z,F00Z29Z,F00Z2KZ,F00Z2ZZ,F00Z31Z,F00Z32Z<br>F00Z3KZ,F00Z3ZZ,F00Z41Z,F00Z42Z,F00Z4ZZ,F00Z51Z,F00Z52Z<br>F00Z59Z,F00Z5KZ,F00Z6KZ,F00Z6MZ,F00Z6PZ,F00Z6YZ,F00Z6ZZ<br>F00Z7KZ,F00Z7MZ,F00Z7PZ,F00Z7YZ,F00Z7ZZ,F00Z8KZ,F00Z8MZ<br>F00Z8PZ,F00Z8YZ,F00Z8ZZ,F00Z9KZ,F00Z9PZ,F00Z9QZ,F00Z9YZ<br>F00Z9ZZ,F00ZBKZ,F00ZBNZ,F00ZBPZ,F00ZBQZ,F00ZBTZ,F00ZBYZ<br>F00ZBZZ,F00ZCKZ,F00ZCMZ,F00ZCPZ,F00ZCYZ,F00ZCZZ,F00ZDKZ<br>F00ZDNZ,F00ZDPZ,F00ZDQZ,F00ZDSZ,F00ZDTZ,F00ZDYZ,F00ZDZZ<br>F00ZFKZ,F00ZFNZ,F00ZFPZ,F00ZFSZ,F00ZFTZ,F00ZFYZ,F00ZFZZ<br>F00ZGKZ,F00ZGMZ,F00ZGPZ,F00ZGYZ,F00ZGZZ,F00ZHYZ,F00ZHZZ<br>F00ZJTZ,F00ZJWZ,F00ZJYZ,F00ZKKZ,F00ZKPZ,F00ZKYZ,F00ZKZZ<br>F00ZLKZ,F00ZLMZ,F00ZLPZ,F00ZLYZ,F00ZLZZ,F00ZMKZ,F00ZMPZ<br>F00ZMSZ,F00ZMVZ,F00ZMYZ,F00ZMZZ,F00ZNNZ,F00ZNPZ,F00ZNQZ<br>F00ZNSZ,F00ZNTZ,F00ZNYZ,F00ZPYZ,F00ZPZZ,F00ZQ1Z,F00ZQ2Z<br>F00ZQKZ,F00ZQZZ,F00ZR1Z,F00ZR2Z,F00ZRKZ,F00ZRZZ,F00ZS1Z<br>F00ZS2Z,F00ZSKZ,F00ZSZZ,F00ZT1Z,F00ZT2Z,F00ZTKZ,F00ZTZZ<br>F00ZV1Z,F00ZV2Z,F00ZVKZ,F00ZVZZ,F00ZW1Z,F00ZW2Z,F00ZWKZ<br>F00ZWZZ,F00ZXZZ,F0100EZ,F0100FZ,F0100UZ,F0100YZ,F0100ZZ<br>F0101ZZ,F0103ZZ,F0104ZZ,F0105YZ,F0105ZZ,F0106YZ,F0106ZZ<br>F010GZZ,F0110EZ,F0110FZ,F0110UZ,F0110YZ,F0110ZZ,F0111ZZ<br>F0113ZZ,F0114ZZ,F0115YZ,F0115ZZ,F0116YZ,F0116ZZ,F011GZZ<br>F0120EZ,F0120FZ,F0120UZ,F0120YZ,F0120ZZ,F0121ZZ,F0123ZZ<br>F0124ZZ,F0125YZ,F0125ZZ,F0126YZ,F0126ZZ,F012GZZ,F0130EZ<br>F0130FZ,F0130UZ,F0130YZ,F0130ZZ,F0131ZZ,F0133ZZ,F0134ZZ<br>F0135YZ,F0135ZZ,F0136YZ,F0136ZZ,F013GZZ,F0150,F0151,F01511<br>F01518,F0152,F0153,F0154,F01A0,F01A11,F01A18,F01A2,F01A3<br>F01A4,F01B0,F01B11,F01B18,F01B2,F01B3,F01B4,F01C0,F01C11<br>F01C18,F01C2,F01C3,F01C4,F01D0EZ,F01D0FZ,F01D0UZ,F01D0YZ<br>F01D0ZZ,F01D1ZZ,F01D5YZ,F01D5ZZ,F01D6YZ,F01D6ZZ,F01F0EZ<br>F01F0FZ,F01F0UZ,F01F0YZ,F01F0ZZ,F01F1ZZ,F01F5YZ,F01F5ZZ<br>F01F6YZ,F01F6ZZ,F01G0EZ,F01G0FZ,F01G0UZ,F01G0YZ,F01G0ZZ<br>F01G1ZZ,F01G5YZ,F01G5ZZ,F01G6YZ,F01G6ZZ,F01H0EZ,F01H0FZ<br>F01H0UZ,F01H0YZ,F01H0ZZ,F01H1ZZ,F01H5YZ,F01H5ZZ,F01H6YZ<br>F01H6ZZ,F01J0EZ,F01J0FZ,F01J0UZ,F01J0YZ,F01J0ZZ,F01J1ZZ<br>F01J5YZ,F01J5ZZ,F01J6YZ,F01J6ZZ,F01K0EZ,F01K0FZ,F01K0UZ<br>F01K0YZ,F01K0ZZ,F01K1ZZ,F01K5YZ,F01K5ZZ,F01K6YZ,F01K6ZZ<br>F01L0EZ,F01L0FZ,F01L0UZ,F01L0YZ,F01L0ZZ,F01L1ZZ,F01L5YZ<br>F01L5ZZ,F01L6YZ,F01L6ZZ,F01M0EZ,F01M0FZ,F01M0UZ,F01M0YZ<br>F01M0ZZ,F01M1ZZ,F01M5YZ,F01M5ZZ,F01M6YZ,F01M6ZZ,F01N0EZ<br>F01N0FZ,F01N0UZ,F01N0YZ,F01N0ZZ,F01Z2KZ,F01Z2MZ,F01Z2NZ<br>F01Z2PZ,F01Z2QZ,F01Z2SZ,F01Z2YZ,F01Z2ZZ,F01Z77Z,F01Z9JZ<br>F01ZBEZ,F01ZBFZ,F01ZBUZ,F01ZBZZ,F01ZCEZ,F01ZCFZ,F01ZCUZ<br>F01ZCZZ,F01ZDEZ,F01ZDFZ,F01ZDUZ,F01ZDYZ,F01ZDZZ,F01ZFEZ<br>F01ZFFZ,F01ZFUZ,F01ZFZZ,F0209YZ,F0209ZZ,F020DYZ,F020DZZ<br>F021DYZ,F021DZZ,F022DYZ,F022DZZ,F023DYZ,F023DZZ,F024GCZ<br>F024GGZ,F024GYZ,F024GZZ,F025GCZ,F025GGZ,F025GYZ,F025GZZ<br>F026GCZ,F026GGZ,F026GYZ,F026GZZ,F0277EZ,F0277GZ,F0277UZ<br>F0277YZ,F0277ZZ,F027GCZ,F027GGZ,F027GYZ,F027GZZ,F0280,F0281 | 2900,29010,29011,29013,29021,29040,<br>29041,2908,2909,29410<br>29411,29420,29421,8915,9301,9302,9303,<br>9304,9305,9306,9307<br>9309,9375 |

Continued on next page

Table S9 (continued).

| Diagnosis | ICD-10 | ICD-9 |
|---|---|---|
| | F02811,F02818,F0282,F0283,F0284,F028GCZ,F028GGZ,F028GYZ<br>F028GZZ,F029GCZ,F029GGZ,F029GYZ,F029GZZ,F02A0,F02A11,F02A18<br>F02A2,F02A3,F02A4,F02B0,F02B11,F02B18,F02B2,F02B3,F02B4<br>F02BGCZ,F02BGGZ,F02BGYZ,F02BGZZ,F02C0,F02C11,F02C18,F02C2<br>F02C3,F02C4,F02C7EZ,F02C7GZ,F02C7UZ,F02C7YZ,F02C7ZZ,F02CGCZ<br>F02CGGZ,F02CGYZ,F02CGZZ,F02Z0EZ,F02Z0FZ,F02Z0UZ,F02Z0ZZ<br>F02Z1EZ,F02Z1FZ,F02Z1UZ,F02Z1ZZ,F02Z2YZ,F02Z2ZZ,F02Z3EZ<br>F02Z3FZ,F02Z3UZ,F02Z3ZZ,F02Z4EZ,F02Z4FZ,F02Z4UZ,F02Z4ZZ<br>F02Z5KZ,F02Z5MZ,F02Z5NZ,F02Z5PZ,F02Z5QZ,F02Z5SZ,F02Z5YZ<br>F02Z5ZZ,F02Z6ZZ,F02Z8YZ,F02Z8ZZ,F02ZBEZ,F02ZBFZ,F02ZBUZ<br>F02ZBYZ,F02ZBZZ,F02ZCEZ,F02ZCFZ,F02ZCUZ,F02ZCYZ,F02ZCZZ<br>F02ZFYZ,F02ZFZZ,F02ZHEZ,F02ZHFZ,F02ZHGZ,F02ZHUZ,F02ZHYZ<br>F02ZHZZ,F0390,F0391,F03911,F03918,F0392,F0393,F0394,F03A0<br>F03A11,F03A18,F03A2,F03A3,F03A4,F03B0,F03B11,F03B18,F03B2<br>F03B3,F03B4,F03C0,F03C11,F03C18,F03C2,F03C3,F03C4 | |
| Difficulty understanding | R41841 | 79952 |
| Dysarthria | R471 | 78451 |
| Dysphasia | R4702 | 78459 |
| Reading difficulty | F810 | 31500,31502 |
| Intracranial haemorrhage | I6000,I6001,I6002,I6010,I6011,I6012,I602,I6020,I6021,I6022<br>I6030,I6031,I6032,I604,I6050,I6051,I6052,I606,I607,I608,I609<br>I610,I611,I612,I613,I614,I615,I616,I618,I619,I6200,I6201<br>I6202,I6203,I621,I629 | 430,431,4320,4321,4329 |
| Delirium | F05 | 2930,2931 |
| Alzheimer's | G300,G301,G308,G309 | 3310 |
| Apraxia | R482 | 78469 |
| Balance difficulty | R2681 | 7812 |
| Tremors | R250,R251,R252,R253,R258,R259 | 72982,7810 |
| Heavy arms or legs | R29898 | 72989,78199 |
| Dystonia | G2401,G2402,G2409,G241,G242,G243,G244,G245,G248,G249 | 3336,33372,33379,33381,33382,33383,<br>33385,33389 |
| Myoclonus | G253 | 3332 |
| Parkinson's | G20,G20A1,G20A2,G20B1,G20B2,G20C | 3320 |
| Excessive sleep | G4710,G4711,G4712,G4713,G4714,G4719 | 32710,32711,32712,32713,32714,32719,<br>78054 |
| Insomnia | F510,G470 | |
| Somnolence | R400 | 78009 |
| Diplopia | H532 | 3682 |
| Dry eye | H0412 | |
| Eye pain | H5710,H5711,H5712,H5713 | 37991 |
| Flashing lights and floaters | H53001,H53002,H53003,H53009,H53011,H53012,H53013,H53019<br>H53021,H53022,H53023,H53029,H53031,H53032,H53033,H53039<br>H53041,H53042,H53043,H53049,H5310,H5311,H53121,H53122,H53123<br>H53129,H53131,H53132,H53133,H53139,H53141,H53142,H53143<br>H53149,H5315,H5316,H5319,H532,H5330,H5331,H5332,H5333,H5334<br>H5340,H53411,H53412,H53413,H53419,H53421,H53422,H53423,H53429<br>H53431,H53432,H53433,H53439,H53451,H53452,H53453,H53459<br>H53461,H53462,H53469,H5347,H53481,H53482,H53483,H53489,H5350<br>H5351,H5352,H5353,H5354,H5355,H5359,H5360,H5361,H5362,H5363<br>H5369,H5371,H5372,H538,H539 | 36800,36801,36802,36803,36810,36811,<br>36812,36813,36814,36815<br>36816,3682,36830,36831,36832,36833,<br>36834,36840,36841,36842<br>36843,36844,36845,36846,36847,36851,<br>36852,36853,36854,36855<br>36859,36860,36861,36862,36863,36869,<br>3688,3689 |
| Itchy eyes | H01111,H01112,H01113,H01114,H01115,H01116,H01119,H01121<br>H01122,H01123,H01124,H01125,H01126,H01129,H01131,H01132<br>H01133,H01134,H01135,H01136,H01139,H01141,H01142,H01143<br>H01144,H01145,H01146,H01149 | 37331,37332,37333,37334 |
| Photophobia | H5319 | 36815 |
| Red eye | H10011,H10012,H10013,H10019,H10021,H10022,H10023,H10029,H1010 | 37200,37201,37202,37203,37204,37205,<br>37206,37210,37211,37212 |

Continued on next page

Table S9 (continued).

| Diagnosis | ICD-10 | ICD-9 |
|---|---|---|
| | H1011,H1012,H1013,H10211,H10212,H10213,H10219,H10221,H10222 H10223,H10229,H10231,H10232,H10233,H10239,H1030,H1031,H1032 H1033,H10401,H10402,H10403,H10409,H10411,H10412,H10413,H10419 H10421,H10422,H10423,H10429,H10431,H10432,H10433,H10439,H1044 H1045,H10501,H10502,H10503,H10509,H10511,H10512,H10513,H10519 H10521,H10522,H10523,H10529,H10531,H10532,H10533,H10539 H10811,H10812,H10813,H10819,H10821,H10822,H10823,H10829,H1089 H109 | 37213,37214,37220,37221,37222,37230, 37234,37239 |
| Watery eyes | H04201,H04202,H04203,H04209,H04211,H04212,H04213,H04219 H04221,H04222,H04223,H04229 | 37520,37521,37522 |
| Neuro-ophthalmologic disorders | H4600,H4601,H4602,H4603,H4610,H4611,H4612,H4613,H462,H463 H468,H469,H47011,H47012,H47013,H47019,H47021,H47022,H47023 H47029,H47031,H47032,H47033,H47039,H47091,H47092,H47093 H47099,H4710,H4711,H4712,H4713,H47141,H47142,H47143,H47149 H4720,H47211,H47212,H47213,H47219,H4722,H47231,H47232,H47233 H47239,H47291,H47292,H47293,H47299,H47311,H47312,H47313 H47319,H47321,H47322,H47323,H47329,H47331,H47332,H47333 H47339,H47391,H47392,H47393,H47399,H4741,H4742,H4743,H4749 H47511,H47512,H47519,H47521,H47522,H47529,H47531,H47532 H47539,H47611,H47612,H47619,H47621,H47622,H47629,H47631 H47632,H47639,H47641,H47642,H47649,H479 | 37700,37701,37702,37703,37704,37710, 37711,37712,37713,37714 37715,37716,37721,37722,37723,37724, 37730,37731,37732,37733 37734,37739,37741,37742,37743,37749, 37751,37752,37753,37754 37761,37762,37763,37771,37772,37773, 37775,3779 |
| Ejaculation difficulty | N5311,N5312,N5313,N5314,N5319 | 60887,60889 |
| Menorrhagia | N920,N922,N924 | 6262,6263,6270 |
| Menstrual changes | N920,N921,N922,N923,N924,N925,N926 | 6262,6263,6264,6265,6266,6268,6269, 6270 |
| Premenstrual syndrome | N943 | 6254 |
| Vaginal discharge | N898,N930,N931,N938,N939 | 6234,6235,6236,6238,6267,6268,6269 |
| Vaginal dryness | N952 | 6273 |
| Anorgasm | F5231,F5232 | 30273,30274 |
| Reduced libido | R6882 | 79981 |
| Cardiogenic shock | R570,R571,R578,R579 | 78550,78551,78559 |
| Atrial Fibrillation | I48 | |
| Deep vein thrombosis | I82401,I82402,I82403,I82409,I82411,I82412,I82413,I82419 I82421,I82422,I82423,I82429,I82431,I82432,I82433,I82439 I82441,I82442,I82443,I82449,I82451,I82452,I82453,I82459 I82461,I82462,I82463,I82469,I82491,I82492,I82493,I82499 I824Y1,I824Y2,I824Y3,I824Y9,I824Z1,I824Z2,I824Z3,I824Z9 I82629 | 45340,45341,45342,45382 |
| Superficial vein thrombosis | I82619,I82811,I82812,I82813,I82819 | 4536,45381 |
| Pulmonary embolism | I2601,I2602,I2609,I2690,I2692,I2693,I2694,I2699 | 4150,41512,41513,41519 |
| Phlebitis; thrombophlebitis and thromboembolism | I8000,I8001,I8002,I8003,I8010,I8011,I8012,I8013,I80201,I80202 I80203,I80209,I80211,I80212,I80213,I80219,I80221,I80222 I80223,I80229,I80231,I80232,I80233,I80239,I80241,I80242 I80243,I80249,I80251,I80252,I80253,I80259,I80291,I80292 I80293,I80299,I803,I808,I809 | 4510,45111,45119,4512,45181,45189,4519 |
| TIA | G450,G451,G452,G453,G454,G458,G459 | 36234,4350,4351,4352,4353,4358,4359, 4377 |
| Ischemic stroke | I6300,I63011,I63012,I63013,I63019,I6302,I63031,I63032,I63033 I63039,I6309,I6310,I63111,I63112,I63113,I63119,I6312,I63131 I63132,I63133,I63139,I6319,I6320,I63211,I63212,I63213,I63219 I6322,I63231,I63232,I63233,I63239,I6329,I6330,I63311,I63312 I63313,I63319,I63321,I63322,I63323,I63329,I63331,I63332 I63333,I63339,I63341,I63342,I63343,I63349,I6339,I6340,I63411 I63412,I63413,I63419,I63421,I63422,I63423,I63429,I63431 I63432,I63433,I63439,I63441,I63442,I63443,I63449,I6349,I6350 I63511,I63512,I63513,I63519,I63521,I63522,I63523,I63529 I63531,I63532,I63533,I63539,I63541,I63542,I63543,I63549,I6359 I636,I638,I6381,I6389,I639 | 43301,43311,43321,43331,43381,43391, 43401,43411,43491 |
| Ischemic | I255 | 4148 |

Continued on next page

Table S9 (continued).

| Diagnosis | ICD-10 | ICD-9 |
|---|---|---|
| cardiomyopathy | | |
| Hemorrhagic stroke | I610,I611,I612,I613,I614,I615,I616,I618,I619 | 431 |
| Cold extremities | R209 | 7820 |
| Limb swelling | R220,R221,R222,R2230,R2231,R2232,R2233,R2240,R2241,R2242 R2243,R229 | 7822,7842,7866 |
| Orthostatic hypotension | I951 | 4580 |
| Palpitations | R002 | 7851 |
| Presyncope | O14 | |
| Tachycardia | ROOO | |
| Bradycardia | R001 | 42781,42789 |
| Paresthesia (tingling limbs) | R202 | 7820 |
| Flushing | R232 | 78262 |
| Ventricular arrhythmia | I4901,I4902,I491,I492,I493,I4940,I4949,I495,I498,I499 | 4270,42741,42742,42760,42761,42769, 42781,42789,4279 |
| Atrial flutter | I483,I484,I4892 | 42732 |
| Encounter for observation and examination for conditions ruled out (excludes infectious disease, neoplasm, mental disorders) | Z036,Z0371,Z0372,Z0373,Z0374,Z0375,Z0379,Z03810,Z03818,Z0389 | V7182,V7183,V7189,V8901,V8902, V8903,V8904,V8905,V8909 |
| Sexually transmitted infections (excluding HIV and hepatitis) | A64 | 0999 |
| Asthenia | R531 | 78079 |
| Joint pain | M2550,M2551,M25511,M25512,M25519,M25521,M25522,M25529,M25531 M25532,M25539,M25541,M25542,M25549,M25551,M25552,M25559 M25561,M25562,M25569,M25571,M25572,M25579,M2559 | 71940,71941,71942,71943,71945,71946, 71947 |
| Joint stiffness | M2560,M25611,M25612,M25619,M25621,M25622,M25629,M25631,M25632 M25639,M25641,M25642,M25649,M25651,M25652,M25659,M25661 M25662,M25669,M25671,M25672,M25673,M25674,M25675,M25676,M2569 | 71950,71951,71952,71953,71954,71955, 71956,71957 |
| Myalgia | M791 | 7291 |
| Myopathy | G720,G721,G722,G723,G7241,G7249,G7281,G7289,G729 | 3593,3594,35971,35979,35981,35989,3599 |
| Muscle cramping | R252 | 72982,7810 |
| Muscle twitch | M6283 | |
| Paraesthesia | R202 | 7820 |
| Myoneural junction muscle disease | G7000,G7001,G701,G702,G7080,G7081,G7089,G709,G710,G7100,G7101 G7102,G7109,G7111,G7112,G7113,G7114,G7119,G712,G7120,G7121 G71220,G71228,G7129,G713,G718,G719,G720,G721,G722,G723,G7241 G7249,G7281,G7289,G729,G731,G733,G737 | 35800,35801,3581,3582,35830,35831, 35839,3588,3589,3590,3591 35921,35922,35923,35924,35929,3593, 3594,3595,35971,35979 35981,35989,3599 |
| Other and unspecified arthropathy | M1200,M12011,M12012,M12019,M12021,M12022,M12029,M12031,M12032 M12039,M12041,M12042,M12049,M12051,M12052,M12059,M12061 M12062,M12069,M12071,M12072,M12079,M1208,M1209,M1210,M12111 M12112,M12119,M12121,M12122,M12129,M12131,M12132,M12139 M12141,M12142,M12149,M12151,M12152,M12159,M12161,M12162 M12169,M12171,M12172,M12179,M1218,M1219,M1220,M12211,M12212 M12219,M12221,M12222,M12229,M12231,M12232,M12239,M12241 | 7144,71600,71601,71602,71603,71604, 71605,71606,71607,71608 71609,71610,71611,71612,71613,71614, 71615,71616,71617,71618 71619,71640,71641,71642,71643,71644, 71645,71646,71647,71648 71649,71680,71681,71682,71683,71684, 71685,71686,71687,71688 71689,71690,71920,71921,71922,71923, 71924,71925,71926,71927 71928,71929,71930,71931,71932,71933, 71934,71935,71936,71937 71938,71939 |

Continued on next page

Table S9 (continued).

| Diagnosis | ICD-10 | ICD-9 |
|---|---|---|
| | M12242,M12249,M12251,M12252,M12259,M12261,M12262,M12269<br>M12271,M12272,M12279,M1228,M1229,M1230,M12311,M12312,M12319<br>M12321,M12322,M12329,M12331,M12332,M12339,M12341,M12342<br>M12349,M12351,M12352,M12359,M12361,M12362,M12369,M12371<br>M12372,M12379,M1238,M1239,M1240,M12411,M12412,M12419,M12421<br>M12422,M12429,M12431,M12432,M12439,M12441,M12442,M12449<br>M12451,M12452,M12459,M12461,M12462,M12469,M12471,M12472<br>M12479,M1248,M1249,M1250,M12511,M12512,M12519,M12521,M12522<br>M12529,M12531,M12532,M12539,M12541,M12542,M12549,M12551<br>M12552,M12559,M12561,M12562,M12569,M12571,M12572,M12579,M1258<br>M1259,M1280,M12811,M12812,M12819,M12821,M12822,M12829,M12831<br>M12832,M12839,M12841,M12842,M12849,M12851,M12852,M12859<br>M12861,M12862,M12869,M12871,M12872,M12879,M1288,M1289,M129 | |
| Spondylopathies | M4600,M4601,M4602,M4603,M4604,M4605,M4606,M4607,M4608,M4609 | 7201,7202,72089,7209,72290,72291,72292,<br>72293,73028,73098 |
| spondyloarthropathy (including infective) | M461,M4620,M4621,M4622,M4623,M4624,M4625,M4626,M4627,M4628<br>M4630,M4631,M4632,M4633,M4634,M4635,M4636,M4637,M4638,M4639<br>M4640,M4641,M4642,M4643,M4644,M4645,M4646,M4647,M4648,M4649<br>M4650,M4651,M4652,M4653,M4654,M4655,M4656,M4657,M4658,M4659<br>M4680,M4681,M4682,M4683,M4684,M4685,M4686,M4687,M4688,M4689<br>M4690,M4691,M4692,M4693,M4694,M4695,M4696,M4697,M4698,M4699 | |
| Acquired deformities (excluding foot) | M2100,M21021,M21022,M21029,M21051,M21052,M21059,M21061,M21062<br>M21069,M21071,M21072,M21079,M2110,M21121,M21122,M21129,M21151<br>M21152,M21159,M21161,M21162,M21169,M21171,M21172,M21179,M2120<br>M21211,M21212,M21219,M21221,M21222,M21229,M21231,M21232<br>M21239,M21241,M21242,M21249,M21251,M21252,M21259,M21261<br>M21262,M21269,M21271,M21272,M21279,M21331,M21332,M21339<br>M21511,M21512,M21519,M21521,M21522,M21529,M2170,M21721,M21722<br>M21729,M21731,M21732,M21733,M21734,M21739,M21751,M21752<br>M21759,M21761,M21762,M21763,M21764,M21769,M2180,M21821,M21822<br>M21829,M21831,M21832,M21839,M21851,M21852,M21859,M21861<br>M21862,M21869,M2190,M21921,M21922,M21929,M21931,M21932,M21939<br>M21941,M21942,M21949,M21951,M21952,M21959,M21961,M21962<br>M21969 | 73600,73601,73602,73605,73606,73607,<br>73609,73630,73631,73632<br>73639,73641,73642,7366,73670,73671,<br>73679,73681,73689,7369 |
| Crystal arthropathies (excluding gout) | M119 | 71290 |
| Breast cancer - ductal carcinoma in situ (DCIS) | D0500,D0501,D0502,D0510,D0511,D0512,D0580,D0581,D0582,D0590<br>D0591,D0592 | 2330 |
| Leukemia - acute lymphoblastic leukemia (ALL) | C9100,C9101,C9102 | 20400,20401,20402 |
| Leukemia - acute myeloid leukemia (AML) | C9200 | 20500 |
| Leukemia - chronic lymphocytic leukemia (CLL) | C9110,C9111,C9112 | 20410,20411,20412 |
| Leukemia - chronic myeloid leukemia (CML) | C9210,C9211,C9212 | 20510,20511,20512 |
| Myelodysplastic syndrome (MDS) | D469 | 23875 |
| Transverse myelitis | G373 | 34120,34121 |
| Other specified hereditary and degenerative nervous system conditions | G3189 | 33189 |
| Paralysis (other than cerebral palsy) | G8100,G8101,G8102,G8103,G8104,G8110,G8111,G8112,G8113,G8114<br>G8190,G8191,G8192,G8193,G8194,G8220,G8221,G8222,G8250,G8251<br>G8252,G8253,G8254,G830,G8310,G8311,G8312,G8313,G8314,G8320<br>G8321,G8322,G8323,G8324,G8330,G8331,G8332,G8333,G8334,G834<br>G835,G8381,G8382,G8383,G8384,G8389,G839 | 34200,34201,34202,34210,34211,34212,<br>34280,34281,34282,34290<br>34291,34292,34400,34401,34402,34403,<br>34404,34409,3441,3442<br>34430,34431,34432,34440,34441,34442,<br>3445,34460,34461,34481<br>34489,3449 |
| Other specified nervous system | G890,G8911,G8912,G8918,G8921,G8922,G8928,G8929,G893,G894<br>G9001,G9009,G901,G902,G903,G904,G9050,G90511,G90512,G90513 | 32371,32372,3313,3314,3315,3317,33181,<br>3330,3360,3361,3363<br>3368,3369,33700,33701,33709,3371,33720,<br>33721,33722,33729,3373 |

Continued on next page

Table S9 (continued).

| Diagnosis | ICD-10 | ICD-9 |
|---|---|---|
| disorders | G90519,G90521,G90522,G90523,G90529,G9059,G908,G909,G90A,G90B | 3379,3380,33811,33812,33818,33821, 33822,33828,33829,3383,3384 |
| | G910,G911,G912,G913,G914,G918,G919,G92,G9200,G9201,G9202 | 3480,3481,3482,34830,34831,34839,3484, 3485,34881,34882,34889 |
| | G9203,G9204,G9205,G928,G929,G930,G931,G932,G933,G9331,G9332 | 3489,3490,3491,3492,34931,34939,34981, 34982,34989,3499,38861 |
| | G9339,G9340,G9341,G9342,G9343,G9344,G9349,G935,G936,G937 | 7428,78079,99701,99702,99709,99811, 99812,9982 |
| | G9381,G9382,G9389,G939,G94,G950,G9511,G9519,G9520,G9529,G9581 G9589,G959,G960,G9600,G9601,G9602,G9608 ,G9609,G9611,G9612 G9619,G96191,G96198,G968,G96810,G96811,G96819,G9689,G969,G970 G971,G972,G9731,G9732,G9741,G9748,G9749,G9751,G9752,G9761 G9762,G9763,G9764,G9781,G9782,G9783,G9784,G980,G988,G990,G992 G998 | |
| Bells palsy | G510 | 3510 |
| Dysautonomia | G9001,G9009,G901,G902,G903,G904,G9050,G90511,G90512,G90513 | 3330,33700,33701,33709,3371,33720, 33721,33722,33729,3373,3379 |
| | G90519,G90521,G90522,G90523,G90529,G9059,G908,G909,G90A,G90B G990,G998 | 34989,7428 |
| Peripheral neuropathy | G629 | 3579 |
| Shortness of Breath | R0602 | 78605 |
| Orthopnoea | R0601 | 78602 |
| Paroxysmal nocturnal | D595 | 2832 |
| Dyspnoea | R0600 | 78609 |
| Shortness of breath | R0602 | 78605 |
| Tachypnoea | R0682 | 78606 |
| Wheezing | R062 | 78607 |
| Pneumonia (except that caused by tuberculosis) | J180,J181,J182,J188,J189 | 481,485,486,514 |
| Hypoxemia | R0902 | 79902 |
| Dry and scaly skin | L853 | 7068 |
| Hair loss | L659 | 70400 |
| Hives | L509 | 7089 |
| Itchy skin | L299 | 6989 |
| Nail changes | L609 | 7039 |
| Purpura | D690,D691,D692,D693,D6941,D6942,D6949,D6951,D6959,D696,D698 | 2870,2871,2872,28731,28732,28733,28739, 28741,28749,2875,2878 |
| | D699 | 2879 |
| Rash | R21 | 7821 |
| Allergies | T7840XA | 9953 |
| Angioedema | T783 | |
| Body ache | R52 | 33819,78096 |
| Chills and shivering | R6883 | 78064 |
| Dizziness | R42 | 7804 |
| Dry mouth | R682 | 5277 |
| Haemoptysis | R042 | 78630 |
| Hallucinations | R443 | 7801 |
| Hot flashes | R232 | 78262 |
| Mouth ulcer | K1230 | 52800 |
| Neurasthenia | F480 | |
| Polydipsia | R631 | 7835 |
| Polyuria | R358,R3581,R3589 | 78842 |

Continued on next page

Table S9 (continued).

| Diagnosis | ICD-10 | ICD-9 |
|---|---|---|
| Epilepsy Seizures | G40001,G40009,G40011,G40019,G40101,G40109,G40111,G40119 G40201,G40209,G40211,G40219,G40301,G40309,G40311,G40319 G40401,G40409,G40411,G40419,G4042 ,G40501,G40509,G40801 G40802,G40803,G40804,G40811,G40812,G40813,G40814,G40821 G40822,G40823,G40824,G40833,G40834,G4089,G40901,G40909,G40911 G40919,G40A01,G40A09,G40A11,G40A19,G40B01,G40B09,G40B11 G40B19,G40C01,G40C09,G40C11,G40C19 | 34500,34501,34510,34511,3452,3453, 34540,34541,34550,34551 34560,34561,34570,34571,34580,34581, 34590,34591 |
| Sweating | R61 | 7808 |
| Swelling of lymph nodes | R599 | 7856 |
| Urinary retention | R339 | 78820 |
| Vertigo | H814 | |
| Guillain-Barre syndrome | G610 | 3570 |
| Nerve nerve root plexus disorders | G50,G51,G52,G53,G54,G55,G56,G57,G58,G59 | 3529,3538,3559 |
| Parkinsonism | G20,G21 | 3320 |
| Psychotic disorder | F20,F21,F22,F23,F24,F25,F26,F27,F28,F29 | 2970,2971,2972,2973,2983,2984,2988, 2989,30122 |
| ADULT AND CHILD ABUSE NEGLECT AND OTHER MALTREATMENT CONFIRMED | T7401XA,T7401XD,T7401XS,T7402XA,T7402XD,T7402XS,T7411XA T7411XD,T7411XS,T7412XA,T7412XD,T7412XS,T7421XA,T7421XD T7421XS,T7422XA,T7422XD,T7422XS,T7431XA,T7431XD,T7431XS T7432XA,T7432XD,T7432XS,T744XXA,T744XXD,T744XXS,T7451XA T7451XD,T7451XS,T7452XA,T7452XD,T7452XS,T7461XA,T7461XD T7461XS,T7462XA,T7462XD,T7462XS,T7491XA,T7491XD,T7491XS T7492XA,T7492XD,T7492XS | 9099,99550,99551,99552,99553,99554, 99555,99580,99581,99582 99583,99584,E9040,V5889 |
| LIGHTNING DROWNING SUBMERSION ELECTROCUTION VIBRATION | T7500XA,T7500XD,T7500XS,T7501XA,T7501XD,T7501XS,T7509XA T7509XD,T7509XS,T751XXA,T751XXD,T751XXS,T7520XA,T7520XD T7520XS,T7521XA,T7521XD,T7521XS,T7522XA,T7522XD,T7522XS T7523XA,T7523XD,T7523XS,T7529XA,T7529XD,T7529XS,T754XXA T754XXD,T754XXS | 9094,9940,9941,9948,9949,V5889 |
| OTHER AND UNSPECIFIED EFFECTS OF OTHER EXTERNAL CAUSES | T7500XA,T7500XD,T7500XS,T7501XA,T7501XD,T7501XS,T7509XA T7509XD,T7509XS,T751XXA,T751XXD,T751XXS,T7520XA,T7520XD T7520XS,T7521XA,T7521XD,T7521XS,T7522XA,T7522XD,T7522XS T7523XA,T7523XD,T7523XS,T7529XA,T7529XD,T7529XS,T753XXA T753XXD,T753XXS,T754XXA,T754XXD,T754XXS,T7581XA,T7581XD T7581XS,T7582XA,T7582XD,T7582XS,T7589XA,T7589XD,T7589XS | 9094,9940,9941,9946,9948,9949,E903, V5889 |
| ADULT AND CHILD ABUSE NEGLECT AND OTHER MALTREATMENT SUSPECTED | T7601XA,T7601XD,T7601XS,T7602XA,T7602XD,T7602XS,T7611XA T7611XD,T7611XS,T7612XA,T7612XD,T7612XS,T7621XA,T7621XD T7621XS,T7622XA,T7622XD,T7622XS,T7631XA,T7631XD,T7631XS T7632XA,T7632XD,T7632XS,T7651XA,T7651XD,T7651XS,T7652XA T7652XD,T7652XS,T7661XA,T7661XD,T7661XS,T7662XA,T7662XD T7662XS,T7691XA,T7691XD,T7691XS,T7692XA,T7692XD,T7692XS | 9099,99550,99551,99552,99553,99554, 99580,99581,99582,99583 99584,V5889 |
| TOXIC EFFECT OF CONTACT WITH VENOMOUS ANIMALS AND PLANTS | T63001A,T63001D,T63001S,T63002A,T63002D,T63002S,T63003A T63003D,T63003S,T63004A,T63004D,T63004S,T63011A,T63011D T63011S,T63012A,T63012D,T63012S,T63013A,T63013D,T63013S T63014A,T63014D,T63014S,T63021A,T63021D,T63021S,T63022A T63022D,T63022S,T63023A,T63023D,T63023S,T63024A,T63024D T63024S,T63031A,T63031D,T63031S,T63032A,T63032D,T63032S T63033A,T63033D,T63033S,T63034A,T63034D,T63034S,T63041A T63041D,T63041S,T63042A,T63042D,T63042S,T63043A,T63043D T63043S,T63044A,T63044D,T63044S,T63061A,T63061D,T63061S T63062A,T63062D,T63062S,T63063A,T63063D,T63063S,T63064A T63064D,T63064S,T63071A,T63071D,T63071S,T63072A,T63072D T63072S,T63073A,T63073D,T63073S,T63074A,T63074D,T63074S T63081A,T63081D,T63081S,T63082A,T63082D,T63082S,T63083A T63083D,T63083S,T63084A,T63084D,T63084S,T63091A,T63091D T63091S,T63092A,T63092D,T63092S,T63093A,T63093D,T63093S T63094A,T63094D,T63094S,T63111A,T63111D,T63111S,T63112A T63112D,T63112S,T63113A,T63113D,T63113S,T63114A,T63114D T63114S,T63121A,T63121D,T63121S,T63122A,T63122D,T63122S T63123A,T63123D,T63123S,T63124A,T63124D,T63124S,T63191A T63191D,T63191S,T63192A,T63192D,T63192S,T63193A,T63193D T63193S,T63194A,T63194D,T63194S,T632X1A,T632X1D,T632X1S T632X2A,T632X2D,T632X2S,T632X3A,T632X3D,T632X3S,T632X4A | 9091,9895,E9050,E9051,E9052,E9053, E9054,E9055,E9056,E9292 E9509,E959,E9621,E969,E9809,E989, V5889 |

Continued on next page

Table S9 (continued).

| Diagnosis | ICD-10 | ICD-9 |
|---|---|---|
| | T632X4D,T632X4S,T63301A,T63301D,T63301S,T63302A,T63302D T63302S,T63303A,T63303D,T63303S,T63304A,T63304D,T63304S T63311A,T63311D,T63311S,T63312A,T63312D,T63312S,T63313A T63313D,T63313S,T63314A,T63314D,T63314S,T63321A,T63321D T63321S,T63322A,T63322D,T63322S,T63323A,T63323D,T63323S T63324A,T63324D,T63324S,T63331A,T63331D,T63331S,T63332A T63332D,T63332S,T63333A,T63333D,T63333S,T63334A,T63334D T63334S,T63391A,T63391D,T63391S,T63392A,T63392D,T63392S T63393A,T63393D,T63393S,T63394A,T63394D,T63394S,T63411A T63411D,T63411S,T63412A,T63412D,T63412S,T63413A,T63413D T63413S,T63414A,T63414D,T63414S,T63421A,T63421D,T63421S T63422A,T63422D,T63422S,T63423A,T63423D,T63423S,T63424A T63424D,T63424S,T63431A,T63431D,T63431S,T63432A,T63432D T63432S,T63433A,T63433D,T63433S,T63434A,T63434D,T63434S T63441A,T63441D,T63441S,T63442A,T63442D,T63442S,T63443A T63443D,T63443S,T63444A,T63444D,T63444S,T63451A,T63451D T63451S,T63452A,T63452D,T63452S,T63453A,T63453D,T63453S T63454A,T63454D,T63454S,T63461A,T63461D,T63461S,T63462A T63462D,T63462S,T63463A,T63463D,T63463S,T63464A,T63464D T63464S,T63481A,T63481D,T63481S,T63482A,T63482D,T63482S T63483A,T63483D,T63483S,T63484A,T63484D,T63484S,T63511A T63511D,T63511S,T63512A,T63512D,T63512S,T63513A,T63513D T63513S,T63514A,T63514D,T63514S,T63591A,T63591D,T63591S T63592A,T63592D,T63592S,T63593A,T63593D,T63593S,T63594A T63594D,T63594S,T63611A,T63611D,T63611S,T63612A,T63612D T63612S,T63613A,T63613D,T63613S,T63614A,T63614D,T63614S T63621A,T63621D,T63621S,T63622A,T63622D,T63622S,T63623A T63623D,T63623S,T63624A,T63624D,T63624S,T63631A,T63631D T63631S,T63632A,T63632D,T63632S,T63633A,T63633D,T63633S T63634A,T63634D,T63634S,T63691A,T63691D,T63691S,T63692A T63692D,T63692S,T63693A,T63693D,T63693S,T63694A,T63694D T63694S,T63711A,T63711D,T63711S,T63712A,T63712D,T63712S T63713A,T63713D,T63713S,T63714A,T63714D,T63714S,T63791A T63791D,T63791S,T63792A,T63792D,T63792S,T63793A,T63793D T63793S,T63794A,T63794D,T63794S,T63811A,T63811D,T63811S T63812A,T63812D,T63812S,T63813A,T63813D,T63813S,T63814A T63814D,T63814S,T63821A,T63821D,T63821S,T63822A,T63822D T63822S,T63823A,T63823D,T63823S,T63824A,T63824D,T63824S T63831A,T63831D,T63831S,T63832A,T63832D,T63832S,T63833A T63833D,T63833S,T63834A,T63834D,T63834S,T63891A,T63891D T63891S,T63892A,T63892D,T63892S,T63893A,T63893D,T63893S T63894A,T63894D,T63894S,T6391XA,T6391XD,T6391XS,T6392XA T6392XD,T6392XS,T6393XA,T6393XD,T6393XS,T6394XA,T6394XD T6394XS | |
| CONTACT STRIKE OR BITTEN BY DOG INITIAL ENCOUNTER | W540XXA,W540XXD,W540XXS,W541XXA,W541XXD,W541XXS,W548XXA W548XXD,W548XXS | E9060,E9068,E9295 |
| CONTACT STRIKE OR BITTEN BY RODENT | W5301XA,W5301XD,W5301XS,W5309XA,W5309XD,W5309XS,W5311XA W5311XD,W5311XS,W5319XA,W5319XD,W5319XS,W5321XA,W5321XD W5321XS,W5329XA,W5329XD,W5329XS,W5381XA,W5381XD,W5381XS W5389XA,W5389XD,W5389XS | E9061,E9063,E9068,E9295 |
| CONTACT STRIKE OR BITTEN BY MARINE MAMMALS | W5601XA,W5601XD,W5601XS,W5602XA,W5602XD,W5602XS,W5609XA W5609XD,W5609XS,W5611XA,W5611XD,W5611XS,W5612XA,W5612XD W5612XS,W5619XA,W5619XD,W5619XS,W5621XA,W5621XD,W5621XS W5622XA,W5622XD,W5622XS,W5629XA,W5629XD,W5629XS,W5631XA W5631XD,W5631XS,W5632XA,W5632XD,W5632XS,W5639XA,W5639XD W5639XS,W5641XA,W5641XD,W5641XS,W5642XA,W5642XD,W5642XS W5649XA,W5649XD,W5649XS,W5651XA,W5651XD,W5651XS,W5652XA W5652XD,W5652XS,W5659XA,W5659XD,W5659XS,W5681XA,W5681XD W5681XS,W5682XA,W5682XD,W5682XS,W5689XA,W5689XD,W5689XS | E9063,E9068,E9295 |
| CONTACT STRIKE OR BITTEN BY OTHER MAMMALS EG CATS | W5501XA,W5501XD,W5501XS,W5503XA,W5503XD,W5503XS,W5509XA W5509XD,W5509XS,W5511XA,W5511XD,W5511XS,W5512XA,W5512XD W5512XS,W5519XA,W5519XD,W5519XS,W5521XA,W5521XD,W5521XS W5522XA,W5522XD,W5522XS,W5529XA,W5529XD,W5529XS,W5531XA W5531XD,W5531XS,W5532XA,W5532XD,W5532XS,W5539XA,W5539XD W5539XS,W5541XA,W5541XD,W5541XS,W5542XA,W5542XD,W5542XS W5549XA,W5549XD,W5549XS,W5551XA,W5551XD,W5551XS,W5552XA W5552XD,W5552XS,W5559XA,W5559XD,W5559XS,W5581XA,W5581XD W5581XS,W5582XA,W5582XD,W5582XS,W5589XA,W5589XD,W5589XS | E9063,E9068,E9295 |
| CONTACT STRIKE OR BITTEN BY WILD BIRD | W6101XA,W6101XD,W6101XS,W6102XA,W6102XD,W6102XS,W6109XA W6109XD,W6109XS,W6111XA,W6111XD,W6111XS,W6112XA,W6112XD W6112XS,W6119XA,W6119XD,W6119XS,W6121XA,W6121XD,W6121XS W6122XA,W6122XD,W6122XS,W6129XA,W6129XD,W6129XS,W6132XA W6132XD,W6132XS,W6133XA,W6133XD,W6133XS,W6139XA,W6139XD W6139XS,W6142XA,W6142XD,W6142XS,W6143XA,W6143XD,W6143XS W6149XA,W6149XD,W6149XS,W6151XA,W6151XD,W6151XS,W6152XA W6152XD,W6152XS,W6159XA,W6159XD,W6159XS,W6161XA,W6161XD W6161XS,W6162XA,W6162XD,W6162XS,W6169XA,W6169XD,W6169XS W6191XA,W6191XD,W6191XS,W6192XA,W6192XD,W6192XS,W6199XA W6199XD,W6199XS | E9063,E9068,E9295 |

Continued on next page

Table S9 (continued).

| Diagnosis | ICD-10 | ICD-9 |
|---|---|---|
| BITTEN OR STUNG BY NONVENOMOUS INSECT | W57XXXA,W57XXXD,W57XXXS | E9064,E9295 |
| CATACLYSM SUCH AS EXCESSIVE HEAT OR COLD SUNLIGHT EARTHQUAKE VOLCANO FLOOD | X30XXXA,X30XXXD,X30XXXS,X31XXXA,X31XXXD,X31XXXS,X32XXXA X32XXXD,X32XXXS,X34XXXA,X34XXXD,X34XXXS,X35XXXA,X35XXXD X35XXXS,X360XXA,X360XXD,X360XXS,X361XXA,X361XXD,X361XXS X370XXA,X370XXD,X370XXS,X371XXA,X371XXD,X371XXS,X372XXA X372XXD,X372XXS,X373XXA,X373XXD,X373XXS,X3741XA,X3741XD X3741XS,X3742XA,X3742XD,X3742XS,X3743XA,X3743XD,X3743XS X378XXA,X378XXD,X378XXS,X379XXA,X379XXD,X379XXS,X38XXXA X38XXXD,X38XXXS,X3901XA,X3901XD,X3901XS,X3908XA,X3908XD X3908XS,X398XXA,X398XXD,X398XXS | E9000,E9010,E9080,E9081,E9082,E9083, E9084,E9088,E9089,E9090 E9091,E9092,E9093,E9094,E9098,E9268, E9288,E9295,E9298 |
| STRUCK BY THROWN PROJECTED OR FALLING OBJECT OR OTHER OBJECT | W200XXA,W200XXD,W200XXS,W201XXA,W201XXD,W201XXS,W208XXA W208XXD,W208XXS,W2100XA,W2100XD,W2100XS,W2101XA,W2101XD W2101XS,W2102XA,W2102XD,W2102XS,W2103XA,W2103XD,W2103XS W2104XA,W2104XD,W2104XS,W2105XA,W2105XD,W2105XS,W2106XA W2106XD,W2106XS,W2107XA,W2107XD,W2107XS,W2109XA,W2109XD W2109XS,W2111XA,W2111XD,W2111XS,W2112XA,W2112XD,W2112XS W2113XA,W2113XD,W2113XS,W2119XA,W2119XD,W2119XS,W21210A W21210D,W21210S,W21211A,W21211D,W21211S,W21220A,W21220D W21220S,W21221A,W21221D,W21221S,W2131XA,W2131XD,W2131XS W2132XA,W2132XD,W2132XS,W2139XA,W2139XD,W2139XS,W214XXA W214XXD,W214XXS,W2181XA,W2181XD,W2181XS,W2189XA,W2189XD W2189XS,W219XXA,W219XXD,W219XXS,W2201XA,W2201XD,W2201XS W2202XA,W2202XD,W2202XS,W2203XA,W2203XD,W2203XS,W22041A W22041D,W22041S,W22042A,W22042D,W22042S,W2209XA,W2209XD W2209XS,W2210XA,W2210XD,W2210XS,W2211XA,W2211XD,W2211XS W2212XA,W2212XD,W2212XS,W2219XA,W2219XD,W2219XS,W228XXA W228XXD,W228XXS,W230XXA,W230XXD,W230XXS,W231XXA,W231XXD W231XXS,W232XXA,W232XXD,W232XXS,W240XXA,W240XXD,W240XXS W241XXA,W241XXD,W241XXS | E916,E9170,E9173,E9174,E918,E9192, E9196,E9298 |
| CONTACT WITH OTHER AND MACHINERY HAND TOOL OR GLASS | W25XXXA,W25XXXD,W25XXXS,W260XXA,W260XXD,W260XXS,W261XXA W261XXD,W261XXS,W262XXA,W262XXD,W262XXS,W268XXA,W268XXD W268XXS,W269XXA,W269XXD,W269XXS,W270XXA,W270XXD,W270XXS W271XXA,W271XXD,W271XXS,W272XXA,W272XXD,W272XXS,W273XXA W273XXD,W273XXS,W274XXA,W274XXD,W274XXS,W275XXA,W275XXD W275XXS,W278XXA,W278XXD,W278XXS,W28XXXA,W28XXXD,W28XXXS W290XXA,W290XXD,W290XXS,W291XXA,W291XXD,W291XXS,W292XXA W292XXD,W292XXS,W293XXA,W293XXD,W293XXS,W294XXA,W294XXD W294XXS,W298XXA,W298XXD,W298XXS,W300XXA,W300XXD,W300XXS W301XXA,W301XXD,W301XXS,W302XXA,W302XXD,W302XXS,W303XXA W303XXD,W303XXS,W3081XA,W3081XD,W3081XS,W3089XA,W3089XD W3089XS,W309XXA,W309XXD,W309XXS,W310XXA,W310XXD,W310XXS W311XXA,W311XXD,W311XXS,W312XXA,W312XXD,W312XXS,W313XXA W313XXD,W313XXS,W3181XA,W3181XD,W3181XS,W3182XA,W3182XD W3182XS,W3183XA,W3183XD,W3183XS,W3189XA,W3189XD,W3189XS W319XXA,W319XXD,W319XXS | E9190,E9191,E9193,E9194,E9195,E9197, E9198,E9199,E9200,E9201 E9202,E9203,E9204,E9208,E9298 |
| EXPLOSION OF PRESSURIZED DEVICE GAS CYLINDER BOILER | W35XXXA,W35XXXD,W35XXXS,W361XXA,W361XXD,W361XXS,W362XXA W362XXD,W362XXS,W363XXA,W363XXD,W363XXS,W368XXA,W368XXD W368XXS,W369XXA,W369XXD,W369XXS,W370XXA,W370XXD,W370XXS W378XXA,W378XXD,W378XXS,W38XXXA,W38XXXD,W38XXXS | E9210,E9211,E9218,E9298 |
| EXPLOSION OF OTHER MATERIALS | W400XXA,W400XXD,W400XXS,W401XXA,W401XXD,W401XXS,W408XXA W408XXD,W408XXS,W409XXA,W409XXD,W409XXS | E9231,E9232,E9238,E9239,E9298 |
| DISCHARGE OF FIRE-WORK | W39XXXA,W39XXXD,W39XXXS | E9230,E9298 |
| HIT STRUCK KICKED TWISTED BITTEN OR SCRATCHED BY AN-OTHER PERSON | W500XXA,W500XXD,W500XXS,W501XXA,W501XXD,W501XXS,W502XXA W502XXD,W502XXS,W503XXA,W503XXD,W503XXS,W504XXA,W504XXD W504XXS | E9179,E9283,E9298 |
| ACCIDENTAL BUMPING INTO ANOTHE PERSON | W51XXXA,W51XXXD,W51XXXS | E9179,E9298 |
| CRUSHED PUSHED OR STEPPED ON BY CROWD | W52XXXA,W52XXXD,W52XXXS | E9171,E9298 |
| DISCHARGE FROM OTHER AND UNSPECIFIED FIREARMS | W320XXA,W320XXD,W320XXS,W321XXA,W321XXD,W321XXS,W3300XA W3300XD,W3300XS,W3301XA,W3301XD,W3301XS,W3302XA,W3302XD W3302XS,W3303XA,W3303XD,W3303XS,W3309XA,W3309XD,W3309XS W3310XA,W3310XD,W3310XS,W3311XA,W3311XD,W3311XS,W3312XA W3312XD,W3312XS,W3313XA,W3313XD,W3313XS,W3319XA,W3319XD W3319XS,W3400XA,W3400XD,W3400XS,W34010A,W34010D,W34010S | E9220,E9221,E9222,E9223,E9224,E9225, E9228,E9229,E9298 |

Continued on next page

Table S9 (continued).

| Diagnosis | ICD-10 | ICD-9 |
|---|---|---|
| | W34011A,W34011D,W34011S,W34018A,W34018D,W34018S,W3409XA W3409XD,W3409XS,W3410XA,W3410XD,W3410XS,W34110A,W34110D W34110S,W34111A,W34111D,W34111S,W34118A,W34118D,W34118S W3419XA,W3419XD,W3419XS | |
| FOOD POSIONING | A050,A051,A052,A053,A054,A055,A058,A059 | 0050,0051,0052,0054,00581,00589,0059 |
| BENIGN NEOPLASMS | D100,D101,D102,D1030,D1039,D104,D105,D106,D107,D109,D110,D117 | 2100,2101,2102,2103,2104,2105,2106, 2107,2108,2109,2110,2111 |
| | D119,D120,D121,D122,D123,D124,D125,D126,D127,D128,D129,D130 | 2112,2113,2114,2115,2116,2117,2118, 2119,2120,2121,2122,2123 |
| | D131,D132,D1330,D1339,D134,D135,D136,D137,D139,D1391,D1399 | 2124,2125,2126,2127,2128,2129,2130, 2131,2132,2133,2134,2135 |
| | D140,D141,D142,D1430,D1431,D1432,D144,D150,D151,D152,D157 | 2136,2137,2138,2139,2140,2141,2142, 2143,2144,2148,2149,2150 |
| | D159,D1600,D1601,D1602,D1610,D1611,D1612,D1620,D1621,D1622 | 2152,2153,2154,2155,2156,2157,2159, 2160,2161,2162,2163,2164 |
| | D1630,D1631,D1632,D164,D165,D166,D167,D168,D169,D170,D171 | 2165,2166,2167,2168,2169,217,2180,2181, 2182,2189,2190,2191 |
| | D1720,D1721,D1722,D1723,D1724,D1730,D1739,D174,D175,D176 | 2198,2199,220,2210,2211,2212,2218,2219, 2220,2221,2222,2223 |
| | D1771,D1772,D1779,D179,D1800,D1801,D1802,D1803,D1809,D181 | 2224,2228,2229,2230,2231,2232,2233, 22381,22389,2239,2240,2241 |
| | D190,D191,D197,D199,D200,D201,D210,D2110,D2111,D2112,D2120 | 2242,2243,2244,2245,2246,2247,2248, 2249,2250,2251,2252,2253 |
| | D2121,D2122,D213,D214,D215,D216,D219,D220,D2210,D2211,D22111 | 2254,2258,2259,226,2270,2271,2273,2274, 2275,2276,2278,2279 |
| | D22112,D2212,D22121,D22122,D2220,D2221,D2222,D2230,D2239,D224 | 22800,22801,22802,22804,22809,2281, 2290,2298,2299 |
| | D225,D2260,D2261,D2262,D2270,D2271,D2272,D229,D230,D2310 D2311,D23111,D23112,D2312,D23121,D23122,D2320,D2321,D2322 D2330,D2339,D234,D235,D2360,D2361,D2362,D2370,D2371,D2372 D239,D241,D242,D249,D250,D251,D252,D259,D260,D261,D267,D269 D270,D271,D279,D280,D281,D282,D287,D289,D290,D291,D2920,D2921 D2922,D2930,D2931,D2932,D294,D298,D299,D3000,D3001,D3002 D3010,D3011,D3012,D3020,D3021,D3022,D303,D304,D308,D309,D3100 D3101,D3102,D3110,D3111,D3112,D3120,D3121,D3122,D3130,D3131 D3132,D3140,D3141,D3142,D3150,D3151,D3152,D3160,D3161,D3162 D3190,D3191,D3192,D320,D321,D329,D330,D331,D332,D333,D334 D337,D339,D34,D3500,D3501,D3502,D351,D352,D353,D354,D355,D356 D357,D359,D360,D3610,D3611,D3612,D3613,D3614,D3615,D3616 D3617,D367,D369 | |
| SCARRING | L905 | 7092 |
| FITTING OR ADJUST- MENT OF ORTHODONTIC OR DENTAL PROSTHETIC | Z463,Z464 | V523,V534,V585 |
| FITTING OR ADJUST- MENT OF HEARING AID | Z461 | V532 |
| FITTING OR ADJUST- MENT OF CASTS OR BANDAGES | Z4689,Z4801 | V537,V538,V5399,V5831 |
| Encounter for supervision of normal pregnancy | Z3400,Z3401,Z3402,Z3403,Z3480,Z3481,Z3482,Z3483,Z3490,Z3491 Z3492,Z3493 | V220,V221 |
| Encounter for supervision of normal first pregnancy TRIMESTER unspecified | Z3400 | V220 |
| Encounter for supervision of normal first pregnancy TRIMESTER 1 | Z3401 | V220 |
| Encounter for supervision of normal first pregnancy TRIMESTER 2 | Z3402 | V220 |
| Encounter for supervision of normal first pregnancy TRIMESTER 3 | Z3403 | V220 |
| Encounter for supervision of other normal pregnancy TRIMESTER unspecified | Z3480 | V221 |

Continued on next page

**Table S9 (continued).**

| **Diagnosis** | **ICD-10** | **ICD-9** |
|---|---|---|
| Encounter for supervision of other normal pregnancy TRIMESTER 1 | Z3481 | V221 |
| Encounter for supervision of other normal pregnancy TRIMESTER 2 | Z3482 | V221 |
| Encounter for supervision of other normal pregnancy TRIMESTER 3 | Z3483 | V221 |
| Encounter for supervision of normal pregnancy unspecified TRIMESTER unspecified | Z3490 | V221 |
| Encounter for supervision of normal pregnancy unspecified TRIMESTER 1 | Z3491 | V221 |
| Encounter for supervision of normal pregnancy unspecified TRIMESTER 2 | Z3492 | V221 |
| Encounter for supervision of normal pregnancy unspecified TRIMESTER 3 | Z3493 | V221 |

## References


1. B McCall, 15 ways silicon valley is harnessing big data for health. *Nat. Medicine* **26**, 7–10 (2020).
2. DE Knuth, *The art of computer programming.* (Pearson Education) Vol. 3, (1997).
3. DH Jaffe, L Lee, S Huynh, TP Haskell, Health inequalities in the use of telehealth in the united states in the lens of covid-19. *Popul. Heal. Manag.* **23**, 368–377 (2020).
4. JJ Jalbert, et al., Effectiveness of subcutaneous casirivimab and imdevimab in ambulatory patients with covid-19. *Infect. Dis. Ther.* **11**, 2125–2139 (2022).
5. JK DeMartino, et al., Global implications for covid-19 vaccine series completion: Insights from real-world data from the united states. *Vaccines* **10**, 1561 (2022).
6. M Hussein, et al., Real-world effectiveness of casirivimab and imdevimab among patients diagnosed with covid-19 in the ambulatory setting: a retrospective cohort study using a large claims database. *BMJ open* **12**, e064953 (2022).
7. A Silber, et al., Sa34 impact of covid-19 on telehealth utilization: Analysis of komodo healthcare map™ claims data. *Value Heal.* **25**, S611 (2022).
8. AM Khanani, et al., Safety outcomes of brolucizumab in neovascular age-related macular degeneration: results from the iris registry and komodo healthcare map. *JAMA ophthalmology* **140**, 20–28 (2022).
9. A Mohandas, C Tak, R Kavelaars, Eph156 treatment patterns and characteristics of people with newly diagnosed major depressive disorder across the komodo claims database 2018-2020. *Value Heal.* **25**, S220–S221 (2022).
10. J Catini, et al., Evaluation of the path to diagnosis and time to treatment in patients with light-chain amyloidosis using the komodo claims database. *Blood* **140**, 4330–4331 (2022).
11. T Zack, et al., Defining incidence and complications of fibrolamellar liver cancer through tiered computational analysis of clinical data. *NPJ Precis. Oncol.* **7**, 29 (2023).
12. K Sulham, J Reese, 1682. health resource utilization in patients with complicated urinary tract infections (cuti) and antibiotic resistance or treatment failure: A retrospective database analysis in *Open Forum Infectious Diseases.* (Oxford University Press), Vol. 7, p. S825 (2020).
13. Z Al-Aly, Y Xie, B Bowe, High-dimensional characterization of post-acute sequelae of covid-19. *Nature* **594**, 259–264 (2021).
14. AJ Kind, WR Buckingham, Making neighborhood-disadvantage metrics accessible—the neighborhood atlas. *The New Engl. journal medicine* **378**, 2456 (2018).
15. UC Bureau, American community survey 5-year estimates (2022) Accessed August 30, 2023.
16. UC Bureau, P9 hispanic or latino, and not hispanic or latino by race (2020) Accessed April 17, 2024.
17. E Buajitti, S Chiodo, LC Rosella, Agreement between area-and individual-level income measures in a population-based cohort: implications for population health research. *SSM-population health* **10**, 100553 (2020).
18. T Althoff, H Nilforoshan, J Hua, J Leskovec, Large-scale diet tracking data reveal disparate associations between food environment and diet. *Nat. communications* **13**, 267 (2022).
19. H CCSR, Healthcare cost and utilization project (hcup) (agency for healthcare research and quality. *Agency for Healthc. Res. Qual.* (2020).
20. A Subramanian, et al., Symptoms and risk factors for long covid in non-hospitalized adults. *Nat. medicine* **28**, 1706–1714 (2022).
21. AV Ballering, SK van Zon, TC Olde Hartman, JG Rosmalen, Persistence of somatic symptoms after covid-19 in the netherlands: an observational cohort study. *The Lancet* **400**, 452–461 (2022).
22. Y Xie, E Xu, B Bowe, Z Al-Aly, Long-term cardiovascular outcomes of covid-19. *Nat. medicine* **28**, 583–590 (2022).
23. ETT Xu Shi, Wang Miao, A selective review of negative control methods in epidemiology. *Curr Epidemiol* (2020).
24. M Lipsitch, ET Tchetgen, T Cohen, Negative controls: A tool for detecting confounding and bias in observational studies. *Epidemiology* **21**, 383–388 (2010).
25. E Sanderson, C Macdonald-Wallis, G Davey Smith, Negative control exposure studies in the presence of measurement error: implications for attempted effect estimate calibration. *Int. journal epidemiology* **47**, 587–596 (2018).
26. A Abadie, Using synthetic controls: Feasibility, data requirements, and methodological aspects. *J. Econ. Lit.* **59**, 391–425 (2021).
27. A Abadie, A Diamond, J Hainmueller, Synthetic control methods for comparative case studies: Estimating the effect of california's tobacco control program. *J. Am. Stat. Assoc.* **105**, 493–505 (2010).
28. D Arkhangelsky, S Athey, DA Hirshberg, GW Imbens, S Wager, Synthetic difference-in-differences. *Am. Econ. Rev.* **111**, 4088–4118 (2021).
29. E Ben-Michael, A Feller, J Rothstein, The augmented synthetic control method. *J. Am. Stat. Assoc.* **116**, 1789–1803 (2021).
30. GJ Griffith, et al., Collider bias undermines our understanding of covid-19 disease risk and severity. *Nat. communications* **11**, 5749 (2020).
31. MA Hernán, JM Robins, *Causal Inference.* (Chapman and Hall/CRC, Boca Raton), (2019).
32. EA Stuart, Matching methods for causal inference: A review and a look forward. *Stat. Sci.* **25**, 1 (2010).
33. B Efron, RJ Tibshirani, *An introduction to the bootstrap.* (Chapman and Hall/CRC), (1994).
34. L Myers, MJ Sirois, Spearman correlation coefficients, differences between. *Encycl. statistical sciences* **12** (2004).
35. JH Steiger, Tests for comparing elements of a correlation matrix. *Psychol. bulletin* **87**, 245 (1980).

36. GW Corder, DI Foreman, *Nonparametric statistics: A step-by-step approach.* (John Wiley & Sons), (2014).
37. The Ohio State University Wexner Medical Center, Covid-19: Symptoms, prevention and what you can do (https://wexnermedical.osu.edu/infectious-diseases/covid-19/symptoms-and-prevention) (2024) Accessed: 2024-06-12.
38. Center for Systems Science and Engineering, Johns Hopkins University, Time series summary (csse_covid_19_time_series) (https://github.com/CSSEGISandData/COVID-19/tree/master/csse_covid_19_data/csse_covid_19_time_series) (year?) GitHub repository. Accessed 24 July 2026.
